# Genome diversity in Ukraine

**DOI:** 10.1093/gigascience/giaa159

**Published:** 2021-01-13

**Authors:** Taras K Oleksyk, Walter W Wolfsberger, Alexandra M Weber, Khrystyna Shchubelka, Olga T Oleksyk, Olga Levchuk, Alla Patrus, Nelya Lazar, Stephanie O Castro-Marquez, Yaroslava Hasynets, Patricia Boldyzhar, Mikhailo Neymet, Alina Urbanovych, Viktoriya Stakhovska, Kateryna Malyar, Svitlana Chervyakova, Olena Podoroha, Natalia Kovalchuk, Juan L Rodriguez-Flores, Weichen Zhou, Sarah Medley, Fabia Battistuzzi, Ryan Liu, Yong Hou, Siru Chen, Huanming Yang, Meredith Yeager, Michael Dean, Ryan E Mills, Volodymyr Smolanka

**Affiliations:** Department of Biological Sciences, Uzhhorod National University, 32 Voloshyna Str., Uzhhorod 88000, Ukraine; Department of Biological Sciences,Oakland University, Dodge Hall, 118 Library Dr., Rochester, MI 48309, USA; Departamento de Biología, Universidad de Puerto Rico, Mayagüez, PR 00682, USA; Department of Biological Sciences, Uzhhorod National University, 32 Voloshyna Str., Uzhhorod 88000, Ukraine; Department of Biological Sciences,Oakland University, Dodge Hall, 118 Library Dr., Rochester, MI 48309, USA; Departamento de Biología, Universidad de Puerto Rico, Mayagüez, PR 00682, USA; Department of Computational Medicine and Bioinformatics, University of Michigan, Ann Arbor, MI 48109, USA; Department of Biological Sciences,Oakland University, Dodge Hall, 118 Library Dr., Rochester, MI 48309, USA; Departamento de Biología, Universidad de Puerto Rico, Mayagüez, PR 00682, USA; Department of Medicine, Uzhhorod National University, Uzhhorod 88000, Ukraine; A. Novak Transcarpathian Regional Clinical Hospital, Uzhhorod 88000, Ukraine; Astra Dia Inc., Uzhhorod 88000, Ukraine; Astra Dia Inc., Uzhhorod 88000, Ukraine; Astra Dia Inc., Uzhhorod 88000, Ukraine; Department of Biological Sciences,Oakland University, Dodge Hall, 118 Library Dr., Rochester, MI 48309, USA; Departamento de Biología, Universidad de Puerto Rico, Mayagüez, PR 00682, USA; Department of Biological Sciences, Uzhhorod National University, 32 Voloshyna Str., Uzhhorod 88000, Ukraine; Department of Medicine, Uzhhorod National University, Uzhhorod 88000, Ukraine; Velyka Kopanya Family Hospital, Transcarpatia 90330, Ukraine; Lviv National Medical University, Lviv 79010, Ukraine; Zhytomyr Regional Hospital, Zhytomyr 10002, Ukraine; I.I.Mechnikov Dnipro Regional Clinical Hospital, Dnipro 49000, Ukraine; Chernihiv Regional Hospital, Chernihiv 14039, Ukraine; Sumy Diagnostic Center, Sumy 40000, Ukraine; Rivne Regional Specialized Hospital of Radiation Protection, Rivne 33028, Ukraine; Department of Genetic Medicine, Weill Cornell Medical College, New York, NY 10065, USA; Department of Computational Medicine and Bioinformatics, University of Michigan, Ann Arbor, MI 48109, USA; Department of Biological Sciences,Oakland University, Dodge Hall, 118 Library Dr., Rochester, MI 48309, USA; Department of Biological Sciences,Oakland University, Dodge Hall, 118 Library Dr., Rochester, MI 48309, USA; BGI Shenzhen, Shenzhen, 518083, China; BGI Shenzhen, Shenzhen, 518083, China; BGI Shenzhen, Shenzhen, 518083, China; BGI Shenzhen, Shenzhen, 518083, China; Division of Cancer Epidemiology and Genetics, National Cancer Institute, Bethesda, MD 20892, USA; Division of Cancer Epidemiology and Genetics, National Cancer Institute, Bethesda, MD 20892, USA; Department of Computational Medicine and Bioinformatics, University of Michigan, Ann Arbor, MI 48109, USA; Department of Human Genetics, University of Michigan, Ann Arbor, MI, 48109, USA; Department of Medicine, Uzhhorod National University, Uzhhorod 88000, Ukraine

**Keywords:** genomes, NGS, genotyping, variant calling, SNP, CNV, indels, BGISEQ-500, DNBSEQ, Illumina

## Abstract

**Background:**

The main goal of this collaborative effort is to provide genome-wide data for the previously underrepresented population in Eastern Europe, and to provide cross-validation of the data from genome sequences and genotypes of the same individuals acquired by different technologies. We collected 97 genome-grade DNA samples from consented individuals representing major regions of Ukraine that were consented for public data release. BGISEQ-500 sequence data and genotypes by an Illumina GWAS chip were cross-validated on multiple samples and additionally referenced to 1 sample that has been resequenced by Illumina NovaSeq6000 S4 at high coverage.

**Results:**

The genome data have been searched for genomic variation represented in this population, and a number of variants have been reported: large structural variants, indels, copy number variations, single-nucletide polymorphisms, and microsatellites. To our knowledge, this study provides the largest to-date survey of genetic variation in Ukraine, creating a public reference resource aiming to provide data for medical research in a large understudied population.

**Conclusions:**

Our results indicate that the genetic diversity of the Ukrainian population is uniquely shaped by evolutionary and demographic forces and cannot be ignored in future genetic and biomedical studies. These data will contribute a wealth of new information bringing forth a wealth of novel, endemic and medically related alleles.

## Data Description

### Context

Ukraine is the largest country located fully in Europe, with a population that was formed as a result of several millennia of migration and admixture. It occupies the intersection between the westernmost reach of the great steppe and the easternmost extent of the great forests that spread across Europe, at the crossroads of the great trade routes from “Variangians to the Greeks” along the river Dnipro, which the ancient Greeks referred to as Borysthenes, and the Silk Road linking civilizations of Europe and Asia [1]. This land has seen the great human migrations of the Middle Ages sweeping from across the great plains, and even before that in the more distant past, of the early farmers [2] and the nomads who first domesticated the horse [3–6]. Here, at the dawn of the modern human expansion, our ancestors met the Neanderthals who used to hunt the great game along the glacier of the Ice Age [7, 8].

The rich history shaped genetic diversity in the population living in the country of Ukraine today. As people have moved and settled across this land, they have contributed unique genetic variation that varies across the country. While the ethnic Ukrainians constitute approximately more than three-quarters of the total population, this majority is not uniform. A large Russian minority compose approximately one-fifth of the total population, with higher concentration in the southeast of the country. Smaller minority groups are historically present in different parts of the country: Belarusians, Bulgarians, Crimean Tatars, Greeks, Gagauz, Hungarians, Jews, Moldovans, Poles, Romanians, Roma (Gypsies), and others [9].

This study offers genome data from 97 individuals from Ukraine (Ukrainians from Ukraine [UAU]) to the scientific community to help fill the gaps in the current knowledge about genomic variation in Eastern Europe, a part of the world that has been largely and consistently overlooked in global genomic surveys [10]. To our knowledge, this was the first effort to describe and evaluate the genome-wide diversity in Ukraine. Samples were successfully sequenced using BGI's DNA Nanoball (DNBSEQ™) sequencing technology and cross-validated by Illumina sequencing and genotyping. The major objectives of this study were to demonstrate the importance of studying local variation in the region and to demonstrate the distinct and unique genetic components of this population. Of particular interest were the medically related variants, especially those with allele frequencies that differed with the neighboring populations. As a result, we present and describe an annotated dataset of genome-wide variation in genomes of healthy adults sampled across the country.

### Dataset

The new dataset includes 97 whole genomes of self-reported UAU at 30× coverage sequenced using BGISEQ-500 (one of the range of DNBSEQ™ sequencers; BGI Inc., Shenzhen, China) and annotated for genomic variants: single-nucleotide polymorphisms (SNPs), indels, structural variants, and mobile elements. The samples were collected across the entire territory of Ukraine, after obtaining institutional review board (IRB) approval (Protocol 1 from 09/18/2018, Supplementary File S1) for the entire study design and informed consent from each participating volunteer (Supplementary File S2). Each participant in this study had an opportunity to review the informed consent, received an explanation of the nature of the genome data, and made a personal decision about making it public.

The majority of samples in this study (86 of 97) were additionally genotyped using Illumina Global Screening Array (Illumina Inc., San Diego, CA, USA) to confirm the accuracy of base calling between the 2 platforms. In addition, 1 sample (EG600036) was also sequenced on the Illumina NovaSeq 6000 S4 (2 × 150 bp; ∼60× coverage) and used for validation of the variant calls (see summary in Supplementary Table S1 and full sequencing statistics for individual samples in Supplementary Table S1.2). The list of the cross-validated samples and the source technology of the data is presented in Supplementary File S3.

The present dataset contains locations and frequencies of >13 million unique variants in UAU that are further interrogated for functional impact and relevance to medically related phenotypes (Table 1 and data in GigaDB [11]). As much as 3.7% of these alleles, or 478,000, are novel genomic SNPs that have never been previously registered in the Genome Aggregation Database (gnomAD) [12] (Table 1). This number is similar in magnitude to what was reported earlier in 2 populations from European Russia (3–4% [13]). Many of the discovered variants (12.6%) are also currently missing from the global survey of genomic diversity in the 1000 Genomes Project (1KG) [14]. The majority of these described variants are rare or very rare (<5%; Supplementary Fig. S2).

**Table 1:** Summary of variation in the 97 whole-genome sequences from Ukraine

	All samples			Mean per sample	
Sequencing results			Novel % gnomAD (1000 Genomes)^a^	All	Novel %
Total sequence reads	99.8 Bn			1.03 Bn	
Mean coverage	97 Samples at 30×			30×	
**Variation**					
	No. of total unique variants	Novel gnomAD count			
SNPs	13,010,979	477,564	3.7 (12.6)	3,488,083	0.1 (0.7)
Bi-allelic	12,667,283	470,667	3.7 (12.7)	3,340,557	0.3 (0.6)
Multi-allelic	343,696	6,897	2.0 (7.4)	146,340	0.8 (4.7)
Small indels^b^	2,727,604	76,484	2.8 (7.4)	917,731	0.3 (1.0)
Deletions	1,805,739	55,599	3.1 (9.0)	624,919	0.3 (2.4)
Insertions	14,459,87	30,453	2.1 (6.7)	571,461	0.2 (2.1)
**Structural variants** ^c^					
Large deletions	16,078	10,914	67.9 (48.3)	3,524	52.6 (19.1)
Large duplications	1,845	1,356	73.5 (42.3)	562	89.4 (35.2)
Inversions	337	314	93.2 (47.8)	185	94.1 (48.6)
**Mobile element insertions**					
Alu	2,316	1,805	77.9 (38.1)	473	68.1 (18.0)
L1	451	289	64 (50.1)	79	60.8 (27.8)
SVA	100	75	75 (52.0)	20	70 (50)
NUMT	714			16	

a Defined as percent not reported in gnomAD (1000 Genomes).

b Small indels are insertions and deletions <50 bp called by GATK [16].

c Large deletions and duplications are those called by lumpy [17], which are >50 bp.

Because other indigenous ethnic groups from Ukraine (such as the Crimean Tatars or the Gagauz) are not included in the study, increasing the aforementioned sample size from 100 to 1,000 individuals is not likely to greatly contribute to discovery of novel mutations [15]. The proportion of the novel structural variants and mobile elements compared to the earlier databases is even higher: almost 1M (909,991) complex indels, regions of simultaneous deletions and insertions of DNA fragments of different sizes that lead to net a change in length, the majority of which are novel (Table 1). Many of the newly discovered variants are functional and potentially contribute to the phenotype (classified in Table 2). We report many important variants that are overlooked or require special modifications in the commonly used resources and tools in genomic research and diagnostics. This wealth of novel variation underscores the importance of variant discovery in local populations that cannot be ignored in biomedical studies.

**Table 2:** Summary annotation of different genomic elements in the Ukrainian genomes annotated in BGISEQ-500 data^a^ from 97 Ukrainian samples

Variant	No. of alles		Mean per sample
	Unique^b^	Total	
**Variants by location**			
Upstream	2,023,920	6,716,794	69,246
UTR 5′	31,026	122,417	1,263
Exon	320,979	839,045	8,650
UTR 3′	150,302	389,528	4,016
Downstream	2,036,111	6,591,978	67,959
Intergenic	9,844,120	9,844,120	101,486
Intron	9,297,384	42,268,211	435,755
Motif	58,164	58,164	600
**Functional variants by type** ^c^			
Splice site acceptor	1,105	3,844	40
Splice site donor	969	3,609	38
Splice site region	19,436	79,853	824
TFBS ablation	2,229	2,229	23
Conservative in-frame indels	1,544	2,475	26
Gene fusion	98	1,482	16
Disruptive in-frame indels	978	4,093	43
Missense	61,181	169,454	1,747
Start lost	116	413	5
Stop gained	885	2,442	26
Stop loss	95	324	4
Synonymous	49,731	146,066	1,506
Protein folding	105,436	258,767	2,668

a BGISEQ-500 DNBSEQ™ sequencing (BGI Inc., Shenzhen, China).

b Unique alleles represent mutations that were counted only once using the largest transcript, disregarding their frequency in the population.

c Some of the mutations listed can be classified in >1 category.

### Variant calling and confirmation

For each sample in the database, we estimated the number of passing bi-allelic SNP calls (i.e., loci with the non-reference genotypes relative to the most current major human genome assembly, GRCh38 [18]) (Table 1). Then ∼12% of these were filtered out on the basis of excess heterozygosity and low variant quality scores (Supplementary Table S2). For the indels, we also estimated the number of passing calls compared to GRCh38 and excluded 4% of those that did not pass filtering. The total number of the unique SNPs, small and large indels (Table 1) was calculated from the raw read alignments of all 97 sequenced genomes (Total Unique SNPs, Supplementary Table S2) with the exception of those filtered out for low variant quality scores and containing excess heterozygosity (Filtered Count; Supplementary Table S2). In addition, we filtered out 4,135,903 variants that only appeared once in a single sample (for both indels and SNPs) and designated them as “singletons.”

**Figure 1:**
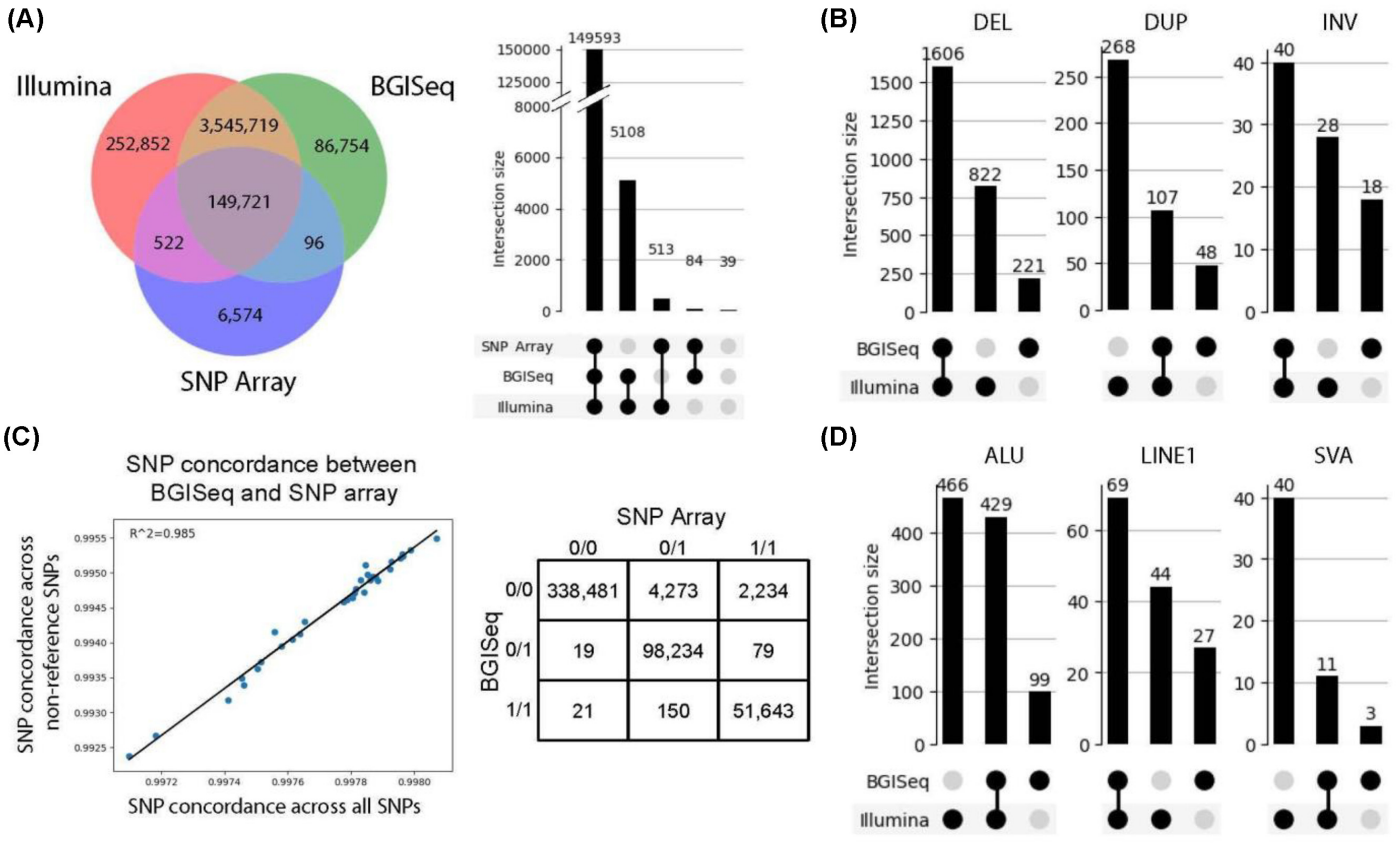
Variant concordance across the 3 sequencing/genotype methods: (**A**) Left: Overlap of SNP positions identified in 1 sample (EG600036) using each of the 3 platforms. Right: Concordance of SNP genotypes in 1 sample derived from each of the 3 platforms. This only includes the subset of SNPs with alternate alleles included in the Illumina genotyping array (the smallest of the 3 variant sets). The variants indicated as belonging to none of the categories are variants whose genotypes differ between all 3 platforms. (**B**) Left: The percentage of concordance between the Illumina SNP array and BGISEQ-500 for all SNPs compared to the percentage concordance of only SNPs with non-reference alleles in the Illumina SNP array for the 86 samples genotyped on both platforms. Right: Concordance of SNP genotypes between BGISEQ-500 and Illumina SNP Array for 1 sample (EG600036). (**C**) Overlap within the numbers of the 3 major structural variants detected in 1 sample using the 2 whole-genome sequencing datasets. (**D**) Overlap within the numbers of the 3 major mobile element insertions detected in 1 sample using the 2 whole-genome sequencing datasets.

We report a good correspondence between the SNP calls made using BGISEQ-500 and NovaSeq 6000 S4 data. A comparison of the variants detected using these 3 platforms for sample EG600036 is summarized in Fig. 1A. The SNP concordance for samples with both BGISEQ-500 and SNP array data is summarized in Fig. 1C. The cross-platform comparison shows a very good overlap across all 3 technologies: >3.5 M (97.7%) of the SNPs identified in the BGISEQ-500 were also verified in the whole-genome sequence of EG600036 sequenced by the Illumina NovaSeq 6000 S4. The correspondence with the Illumina SNP Array for sample EG600036 was also very good: 95.8% of all the SNP genotypes called by the Illumina method were also detected by the BGISEQ-500 (Fig. 1A, right, and C, right). The concordance between the non-reference alleles between the 2 platforms in all 86 samples was nearly linear (*r^2^*= 0.985, Fig. 1C, left).

The transition/transversion (TITV) ratio for the novel SNPs (estimated with TiTvtools [19] and visualized by plotTiTv in Supplementary Fig. S1) was lower than the TITV ratio for SNPs in the dbSNPs database (1.9 vs 2.2; [20]). Similarly, the insertions to deletions (ins/del) ratio for novel indels is lower than for the indels already reported in the dbSNP database (0.63 vs 0.75). This observation likely reflects our improved ability to detect small insertions in newer sequencing technologies compared to many platforms that historically submitted variation to dbSNP.

We have defined the multi-allelic SNPs as observations of genomic positions having 2 or more alternative alleles [21]. These are important variants that are overlooked or require special modifications in the commonly used resources and tools in genomic research and diagnostics. We report a total of 343,696 multiallelic sites in the sequences from our sample, of which 2.0% are at locations unreported in the gnomAD database [12] (Table 1).

In addition to the SNPs, we have identified and quantified major classes of structural variations in the Ukrainian population: small indels (insertions and deletions <50 bp),  large structural variants (deletions, duplications, and inversions > 50 bp), and mobile element insertions (MEI) (Alu elements [ALU], L1 elements, non-autonomous retroelements [SVA], and nuclear mitochondrial DNA [NUMT] copies). A number of structural elements were reported, including common and novel ones. While among the small variants most were common (6–9%), a large proportion of large variants and MEIs (38–52%) have not been reported previously in the 1GP Database (Table 1).

Once more, there is a significant correspondence between the calls made using BGISEQ-500 and Illumina NovaSeq 6000 S4 data. The 2 sequencing platforms show a significant overlap in calling indels (DEL): 87.9% of the variants called by the BGISEQ-500 were also detected by the Illumina platform. At the same time, there were 822 deletions, or 33.8% of all the indels called by the Illumina that were not detected by the BGISEQ-500 (Fig. 1B). A similar picture, where BGISEQ-500 performs competitively well, is also observed for inversions (INV) (Fig. 1B) and LINE1 transposable elements (Fig. 1D). At the same time, there were more duplications (DUP) (Fig. 1B) and the 2 classes of transposable elements evaluated: ALU and SVA (Fig. 1D). Evaluation tests show that current algorithms are platform dependent, in the sense that they exhibit their best performance for specific types of structural variation, as well as for specific size ranges [22], and the algorithms designed for detection and archived datasets are predominantly for Illumina pair-end sequencing [23, 24]. While it is possible that these results indicate Illumina's superiority at detecting structural variation, it can also be the consequence of the bioinformatics tools for calling structural variants developed using mainly the Illumina data, as suggested by previous comparative evaluations of the 2 technologies [25, 26]. Additionally, higher coverage of the Illumina data (60×) could have contributed to the differences observed between the platforms.

The database was compared to the existing global resources of population variation such as gnomAD [12] and the 1KG [14]. Specifically, under our search criteria, the small variants (SNPs and small indels) were considered “novel” if they were absent from all the samples in the 2 global datasets (gnomAD and 1KG; Table 1). The large structural variants and MEIs were considered novel if the variant was not present in the gnomAD and 1KG databases. To determine whether a given variant was present in 1 of the databases, a variant of the same type in the database had to overlap the Ukrainian variant with a minimum fraction of 0.95. We observed no significant deviation of the rate at which reference bases were observed at REF/alt heterozygous SNP sites (reference bias was near 50%).

### Collection of functional variants

A particular interest in this study is the distribution of functional variation, not in the least due to the potential impact on phenotypes, especially to those with medical relevance [27]. As much as 97.5% of all annotated variation was discovered outside of the known functional elements (upstream, downstream, intron, and intergenic). These results are similar to the expected distributions of mutations shown with the simulated data [28]. Nevertheless, there were >8,000 mutations discovered within exons of each individual on average (top half of Table 2). We annotated several classes of functional mutations within the coding regions (bottom half of Table 2). As expected, the nonsense mutations classified in the annotation file as “disruptive in-frame indel,” “start lost,” “stop gained,” and “stop loss” were rare, while categories with minimal effect on the function, such as “synonymous,” “motiff,” “protein folding,” and “missense,” were more common. Some of the mutations listed in the annotation filecan be classified in >1 category (e.g., “synonymous variants” can also be counted in “exonic variants”).

In addition to the gene-coding mutations, we report a number of regulatory variants. For example, the database contains a total of 2,229 transcription factor binding site ablation (TFBS) mutations (bottom half of Table 2). A summary of functional variation discovered in this study is presented in Table 2. The full list of high-impact functional variants (including frameshift, start lost/stop lost or gained, transcript ablations, and splice alterations) that had an allele count of 2 or more with their predicted function, number of gene transcripts of the gene affected, and frequencies is presented in Supplementary Table S3. The full annotation database with classifications is available alongside the associated data deposited in GigaDB [11].

### Collection of the medically relevant variants

Many of the reported variants are already known to be medically related and are listed either in genome-wide association studies (GWAS) [29] or ClinVar (an NCBI archive of reports of the relationships among human variations and phenotypes with supporting evidence) [30] catalogues (Table 3). Our database contains a total of 43,892 benign mutations in medically related genes but also 189 unique pathogenic or likely pathogenic variants, as well as 20 protective or likely protective alleles as defined in ClinVar [30, 31]. Each individual in this study carries 19 pathogenic and 12 protective mutations on average. While at least some individuals were homozygous for the pathogenic allele, none of the associated disease phenotypes have been reported, which could be largely attributed to heterozygosity, age-dependent penetrance, expressivity, and gene-by-environment interactions [32, 33].

**Table 3:** Medically relevant variants in the Ukrainian population included in GWAS [29] and ClinVar [30] databases

Source of annotation	No. unique substitutions^a^	Total allele No.	Mean per sample
GWAS catalog	102,551	6,479,953	66,804
ClinVar: pathogenic (or likely pathogenic)	189	1,830	19
ClinVar: benign (or likely benign)	43,892	1,842,668	18,997
ClinVar: protective (or likely protective)	20	1,209	12

a Unique variants represent substitutions that were counted only once, disregarding their frequency in the population.

As expected, our study shared a lot more variants with the GWAS [29] than with the ClinVar [30] catalogue. While GWAS has recently become the tool of choice to identify genetic variants associated with complex disease and other phenotypes of interest [34], because the amount of genetic variance explained by these variants is low, they are generally not very useful for predicting pathogenic phenotypes [35]. It is also important to note that not all ClinVar variants carry the same weight of supporting evidence; attributing disease causation to prioritized variants remains an inexact process and some of the reported associations eventually are proven to be spurious [36]. Nevertheless, the importance of the unique set of mutations published here is difficult to overemphasize because it constitutes the first published set of pathological variants in an understudied population, an important step towards a local catalogue of medically relevant mutations. In addition, as the attention in the genomic community is shifting from monogenic to polygenic traits, many of these may become relevant in future research and exploration [37]. A full list of the medically relevant functional markers found in the Ukrainian population and reported in GWAS [29] and ClinVar [30] databases is presented in Supplementary Table S4 with alternative allele frequencies and annotations.

Disease variants with frequencies that differed between the Ukrainians and the neighboring populations are of particular interest to the medical community. It is well established that differences in allele frequencies are a consequence of evolutionary forces acting in populations (such as drift, mutation, migration, nonrandom mating, and natural selection) and that certain diseases and heritable traits display marked differences in frequency between populations [38]. With this in mind, we created a list of the known disease variants whose frequencies differ between Ukrainians and other European populations (the combined European sample [EUR] from the 1KG, comprising Utah residents [CEU] with Northern and Western European ancestry, Toscani in Italy [TSI], Finnish in Finland [FIN], British in England and Scotland [GBR], Iberian population in Spain [IBS] [14, 39], and French population from Human Genome Diversity Project [HGDP] [FRA] [40]) and Russians from HGDP (RUS) [40]. Several examples of these variants are presented in Table 4. Among these are variants involved in a number of medical conditions such as hyperglycinuria/iminoglycinuria (rs35329108,*SLC6A19*), efficacy of bisphosphonate response (rs2297480,*FDPS*), autism (rs7794745, *CNTNAP2*), Leber congenital amaurosis (rs10151259,*RPGRIP1*), and breast cancer susceptibility in *BRCA1* and *BRCA2* carriers (rs1801320,*RAD51*) (Table 4).

**Table 4:** Examples of the functional SNPs with highly differentiating functional markers reported in ClinVar [29] with high differences in the Ukrainian population compared to other neighboring European populations

SNP	Chr	Gene	REF/alt^a^	Associated medical condition	Non-reference allele frequency			Fisher exact test *P*-value	
					UKR	EUR	RUS	vs EUR	vs RUS
rs2297480	1	*FDPS*	T/G	Efficacy of the bisphosphonate response	0.13	0.26	0.27	0.038	>0.001
rs35329108	5	*SLC6A19*	G/A	Hyperglycinuria, iminoglycinuria	0.32	0.23	0.17	0.049	0.004
rs7794745	7	*CNTNAP2*	A/T	Autism	0.48	0.38	0.30	0.032	0.010
rs10151259	14	*RPGRIP1*	G/T	Leber congenital amaurosis, cone-rod dystrophy	0.32	0.24	0.11	0.003	0.014
rs1801320	15	*RAD51*	G/C	Breast cancer susceptibility in*BRCA1*and*BRCA2*carriers	0.19	0.08	0.07	0.047	0

a The reference allele (REF) is set according to the reference allele in GrCH38.p13 [18]. Other European populations consist of the combined sample from Western and Central Europe from 1KG (EUR) [14, 39] and French population from HGDP (FRA) [40], as well as Russians (RUS) from HGDP [40]. Non-reference allele frequency is reported compared to the reference allele in GRCh38. Differences are evaluated by the Fisher exact test. All the functional SNPs with significant population frequency differences are listed in Supplementary Table S5.

Of course, not all the medically related variants are currently known, and many remain to be discovered and verified in local populations. This is, to some extent, a consequence of underreporting of allelic endemism within understudied populations, particularly in Eastern Europe [10] but also elsewhere [41, 42]. By offering public annotations of functional mutations in a population sampled across the territory of Ukraine, our database contributes a number of candidates to direct future research in medical genomics. We chose only the markers with the highest non-reference allele frequency differences compared to the neighboring populations EUR [14] and RUS [40], evaluated by the Fisher exact test, and listed them in Table 5.

**Table 5:** Examples of the functional markers with the highest non-reference allele frequency differences in the Ukrainian population evaluated by the Fisher exact test compared to the frequencies in the neighboring populations: the combined population from Europe (EUR) [14] and Russians from HGDP (RUS) [40]

SNP	Chr	Gene	Ref/Alt	Function	Non-reference allele frequency			Fisher exact test *P*-value	
					UKR	EUR	RUS	vs CEU	vs RUS
rs72625995	17	*POM121L8P*	C/T	Exonic, nonsynonymous SNV	0.03	0.62	0.75	2.50E−07	1.86E−06
rs9930886	16	*PTPRN2*	A/G	Exonic, synonymous SNV	0.01	0.33	0.35	2.56E−07	2.19E−06
rs4779816	15	*ZBTB9; BAK1*	A/G	Exonic, nonsynonymous SNV	0.41	0.80	0.83	3.29E−06	7.82E−07
rs58580222	12	*ABCC1*	G/A	Exonic, synonymous SNV	0.03	0.13	0.26	3.06E−04	1.17E−02
rs80150964	11	*SMIM40; KIFC1*	T/C	Exonic, non-synonymous SNV	0.03	0.23	0.19	4.95E−04	1.96E−06

### Population structure and ancestry informative markers

We performed several population analyses, but only to demonstrate the uniqueness and usefulness of this new dataset. Our results indicate that genetic diversity of the Ukrainian population is uniquely shaped by evolutionary and demographic forces and cannot be ignored in future genetic studies. However, we do not evaluate any historical hypotheses on the timing of origins, founding, migration, and admixture of this population and use only the naive approaches, based on the statistical models.

**Figure 2:**
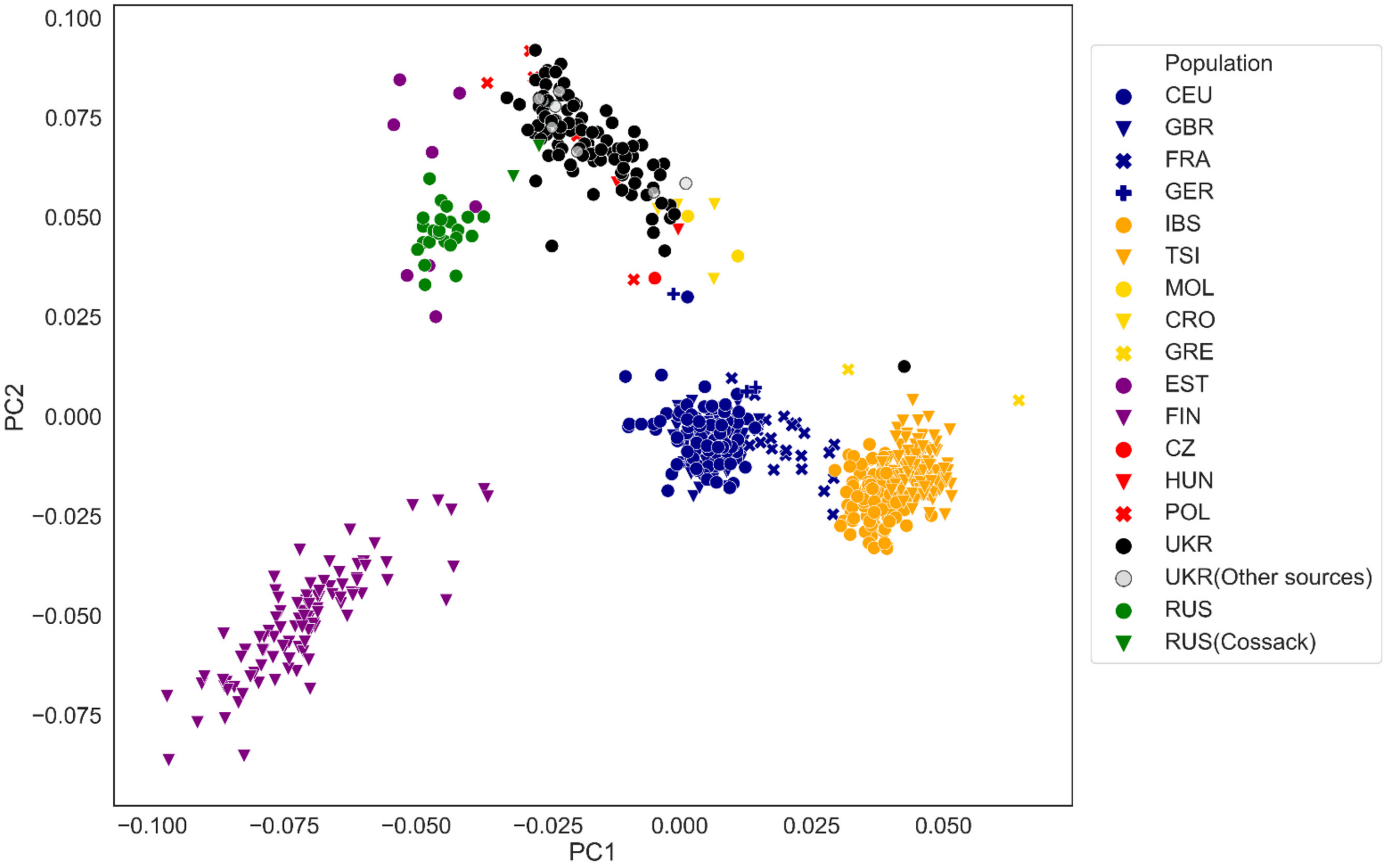
Principal component (PC) analysis of genetic merged dataset, containing European populations. Colors reflect prior population assignments from the European samples from the 1KG (Utah residents [CEU] with Northern and Western European ancestry, Toscani in Italy [TSI], Finnish in Finland [FIN], British in England and Scotland [GBR], Iberian population in Spain [IBS]) [14, 39] and French (FRA) and Russians (RUS) from HGDP (RUS) [40], as well as the relevant high-coverage human genomes Croatian (CRO), Czech (CZ), Estonian (EST), German (GER), Greek (GRE), Hungarian (HUN), Moldovan (MOL), Polish (POL), Russian Cossack (RUS), and Ukrainian (UKR) from the Estonian Biocentre Human Genome Diversity Panel (EGDP) [43] as well as Simons Genome Diversity Project [44]. The analysis was performed with Eigensoft [48].

To demonstrate the extent to which our dataset contributes to the genetic map of Europe, we explored genetic relationships between Ukrainian individuals within our sample and evaluated genetic differences between this population and its immediate neighbors on the European continent for which population data of full genome sequences were publicly available. A principal component analysis (PCA) of the merged dataset of 654 samples included European populations from the 1KG (Utah residents [CEU] with Northern and Western European ancestry, Toscani in Italy [TSI], Finnish in Finland [FIN], British in England and Scotland [GBR], Iberian population in Spain [IBS] [14, 39]) and French (FRA) and Russian (RUS) populations from the HGDP [40], as well as the relevant high-coverage human genomes from the Estonian Biocentre Human Genome Diversity Panel (EGDP: Croatians [CRO], Estonians [EST], Germans [GER], Moldovans [MOL], Polish [POL], and Ukrainians [UKR]) [43] and Simons Genome Diversity Project (Czechs [CZ], Estonians [EST], French [FRA], Greeks [GRE], and Polish [POL]) [43] (Fig. 2). The latter article also identifies “Cossacks” as a separate self-identified ethnic group within Russians (Cossacks [RUS]) or Ukrainians (Cossacks [UKR]) [44] (Supplementary File S3).

**Figure 3:**
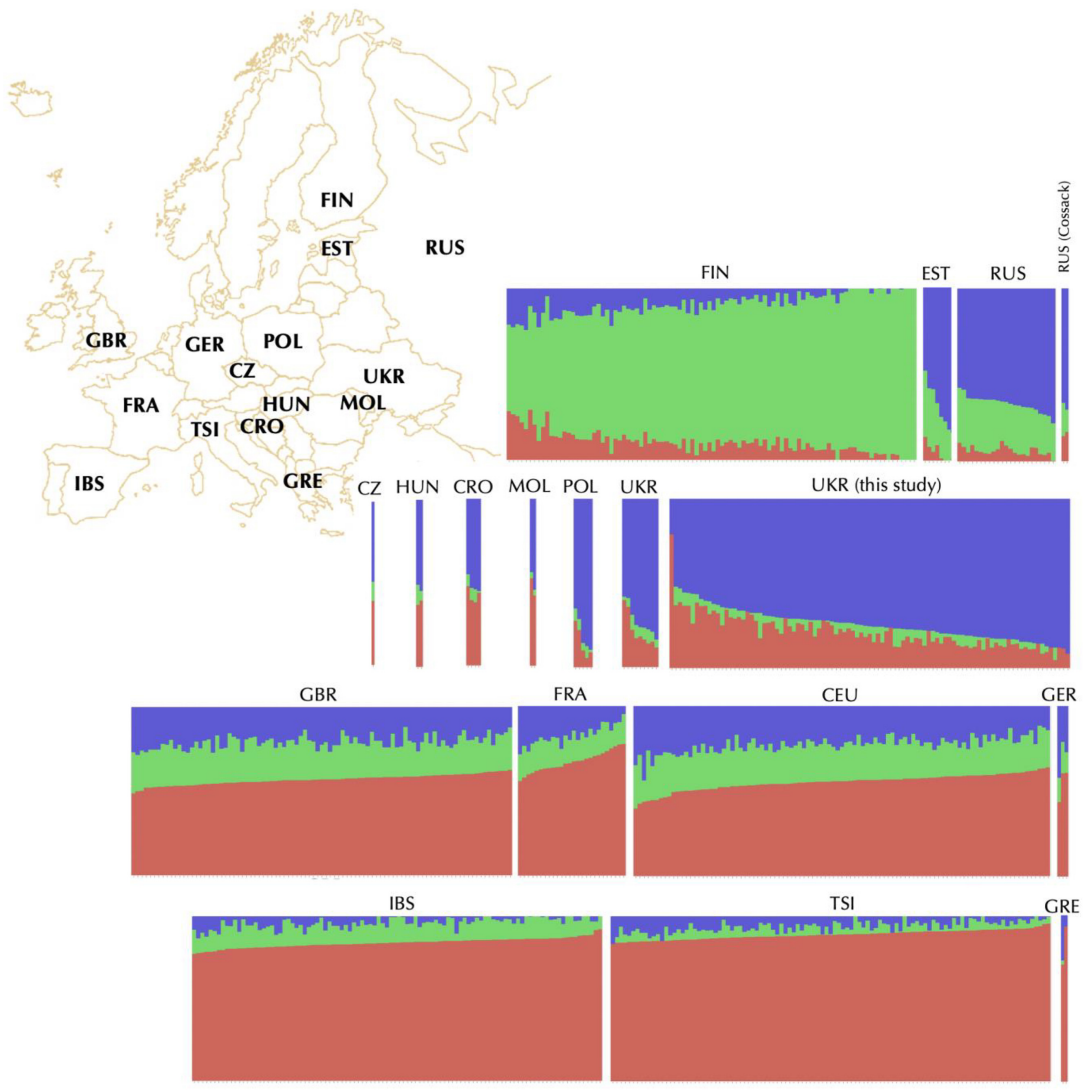
Genetic structure of Ukrainian population in comparison to other European populations. Structure plot constructed using ADMIXTURE package [49] at K = 3 illustrates similarity and differences between genomes from this study as well as samples from the 1KG (Utah residents [CEU] with Northern and Western European ancestry, Toscani in Italy [TSI], Finnish in Finland [FIN], British in England and Scotland [GBR], and Iberian population in Spain [IBS]) [14, 39] and French (FRA) and Russians (RUS) from HGDP [40], as well as the relevant high-coverage human genomes Croatian (CRO), Czech (CZ), Estonian (EST), German (GER), Greek (GRE), Hungarian (HUN), Moldovan (MOL), Polish (POL), Russian Cossack (RUS), and Ukrainian (UKR) from the Estonian Biocentre Human Genome Diversity Panel (EGDP) [43] as well as Simons Genome Diversity Project [44]. For identification of the optimal K parameter, we evaluated a range from 2 to 8, with K = 3 resulting in the lowest error. Plots with K = 3 to K = 6 are presented in Supplementary Fig. S3.

Ukrainian genomes from this as well as other studies [43, 44] form a single cluster positioned between the Northern (Russians, Estonians) on 1 side, and Western European populations on the other (CEU, French, British, and Germans, Fig. 2). There was a significant overlap with the other Central and Eastern European populations, such as Czechs, Polish, and the people from the Balkans (Croats, Greeks, and Moldovans). This is not surprising; in addition to the close geographic distance between these populations, this may also reflect the insufficient representation of samples from the surrounding populations (see data in GigaDB [11]). Similarly, the admixture analysis demonstrates distinctiveness of our dataset but also demonstrates unique combinations of genetic components that may have shaped this population (Fig. 3 and Supplementary Fig. S3).

Addition of the new genomic data will most likely add to the resolution of the genetic map of this region and further reveal differences between the populations of Eastern and Central Europe. Our dataset showed a limited amount of inbreeding (Supplementary Fig. S4) and contains information for future population studies. All the variants with significant difference in frequencies between Ukrainians and other European populations are listed in Supplementary Table S6. This database can be a starting point for association studies, as the candidate ancestry informative markers (AIMs) [45] can be used for mapping disease alleles by admixture disequilibrium [46, 47].

To provide a more extended view of the genetic components contributing to the Ukrainian population, we used the population structure plots using the ADMIXTURE package [49]. This allowed us to construct a preliminary picture of putative ancestry contributions and population admixture. To identify the optimal K, we implied the 10-fold cross-validation function in range of K = 2−6. The results with the optimal K = 3 shown in Fig. 3 illustrate similarity and the difference of Ukrainian population compared to the other populations in Central and Eastern Europe (Fig. 3, second row). While the higher values of K (K = 3−8; Supplementary Fig. S3) show an increasing number of clusters, they also show an increasing amount of error in the cross-validation function. This analysis already shows the potential of the present database in helping to resolve population structure in Eastern Europe, but additional genome-wide data from neighboring populations would be helpful to refine the picture in this geographical region. Unfortunately, valuable genome-wide data collected from 3 populations in Russia have been retracted from public databases after publication [13].

Despite the fact that all of the samples were collected from self-identified ethnic Ukrainians, there were 2 notable outliers: sample EG600048 clustered with the Southern Europeans (Iberia and Italian populations) Fig. 2. This illustrates an important point that ignoring the unique composition of the population will result in ascertainment bias in biomedical studies. Genetics is not a reliable determinant of ethnicity but can be used to evaluate individual contributions of ancestry. In anticipating future ancestry studies, we contribute the full list of candidates for AIMs differentiating Ukrainians from neighboring populations in Europe (Supplementary Table S6).

People of Ukraine carry many previously known and several novel genetic variants with clinical and functional importance that in many cases show allele frequencies different from neighboring populations in the rest of Europe, including Poland to the west, Romania to the south, the Baltics to the north, and Russia to the northeast. While several large genome projects already exist contributing to the understanding of global genetic variation, many rare and endemic alleles have not yet been identified by international databases such as 1KG and are currently not available in standard genotyping panels for association testing for human diseases, and glaring white spots still exist on the genetic maps in local populations of Eastern Europe [10]. We fully expect future sampling and sequencing to continue to improve and complete the detailed picture of genomic diversity in people across the country and contribute to the further development of genetic approaches in biomedical research and applications.

## Methods

### Sampling strategy

The collection and consent procedure was approved as part of the “Genome Diversity in Ukraine” project by the IRB of Uzhhorod National University in Uzhhorod, Ukraine (Protocol 1 from 09/18/2018, Supplementary File S1). We employed doctors and medical professionals from different regions of Ukraine to oversee collection of blood samples at hospitals. Healthy (non-hospitalized) volunteers were contacted through advertisements and invited for personal interviews at outpatient offices. During the visit the volunteers were familiarized with the study and the collection procedure and gave full consent to participate and let their genotypic and phenotypic data be freely and publicly available. During each interview, the volunteer participants also completed a questionnaire indicating self-reported region of origin, place of birth of all 4grandparents (if remembered), sex, and several phenotypical features, such as daily history of disease (Supplementary File S3). The hard copies of the consents and personal interviews remain sealed and stored at the Biology Department of Uzhhorod National University. After the conclusion of the interview and sample collection, all personal identifiers were removed from the vials containing blood samples, except for an alphanumeric identifier and a barcode. All the subsequent analysis and publication was done in a blind design where neither the participants nor the researchers could identify the person who donated the sample.

At the conclusion of the interview a whole-blood sample was collected from a vein into two 5-mL EDTA tubes by a certified nurse or a phlebotomist, assigned a barcode number, and shipped by courier on dry ice to a biomedical laboratory certified to handle blood samples in Uzhhorod, Ukraine (Astra Dia Inc.), for DNA extraction immediately on arrival. The excess of the blood and DNA from samples remaining after the genetic analysis is stored frozen at the biobank of the Biology Department, Uzhhorod National University, Ukraine. As a result, blood samples were collected from a total 113 individuals.

### DNA extraction

Immediately upon arrival to the laboratory, DNA isolation from 200 μL of blood was carried out with the innuPREP DNA Blood Minikit (AAnalytik Jena GmbH, Jena, 07745, Germany). High molecular weight genomic DNA was lightly fragmented by vortexing. The initial DNA concentration was measured with the Implen C40 Nanophotometer (München, Germany), and quality was verified visually on a 2% agarose gel. The 97 successfully extracted DNA samples were normalized to 20–30 ng/μL concentration for downstream application. After extraction the samples were recoded and sent to NIH for the genotyping procedure, whence the aliquots were further shipped to a BGI facility (BGI Shenzhen, China) or to Psomagen Inc. (Gaithersburg, MD, USA) for the whole-genome sequencing (WGS). The remaining ∼2 mL was frozen for future use.

### Sequencing and genotyping

All 97 individuals in this study were sequenced with BGISEQ-500 and 88 individuals were cross-validated by genotyping using Illumina Global Screening Array. The record of which individual samples have been cross-validated by both technologies is presented in Supplementary Table S2. In addition, a single sample (EG600036) was also sequenced on Illumina NovaSeq 6000 S4 (∼60× coverage).

#### Sequencing with BGISEQ-500

All 97 DNA samples were sequenced on BGISEQ-500 (BGI Shenzhen, China). Upon receipt at the BGI facility, and prior to sequencing, samples were checked again for quality. Concentration was once more detected by fluorometer or Microplate Reader (e.g., Qubit Fluorometer, Invitrogen). Sample integrity and purity were detected by agarose gel electrophoresis (concentration of agarose gel: 1%; voltage: 150 V; electrophoresis time: 40 min). Aliquots of 1 μg genomic DNA were fragmented by Covaris. The fragmented genomic DNA was selected by Agencourt AMPure XP-Medium kit to a mean size of 200–400 bp. Fragments were end-repaired and then 3′-adenylated. Adaptors were ligated to the ends of these 3′-adenylated fragments. PCR products were purified by the Agencourt AMPure XP-Medium kit. The double-stranded PCR products were heat denatured and circularized by the splint oligo sequence. The single-strand circle DNA was formatted as the final library. The qualified libraries were sequenced by BGISEQ-500: the single-strand circle DNA molecule formed a DNA nanoball (DNB) containing >300 copies through a rolling-cycle replication. The DNBs were loaded into the patterned nanoarray by using high-density DNA nanochip technology. Finally, pair-end 100-bp reads were obtained by combinatorial probe-anchor synthesis. Raw reads were filtered to remove adaptor sequences, contamination, and low-quality reads. Sequencing of all 97 full genome samples submitted for sequencing at BGI was successful.

#### Short-read sequencing with Illumina NovaSeek6000

One individual was resequenced by Illumina NovaSeq6000 S4 at Psomagen Inc. (Gaithersburg, MD, USA). The library was prepared using TruSeq DNA PCR Free 350 bp protocol by Illumina. The library was sequenced at ∼64× depth, producing 150-bp–long reads, resulting in 241.7 Gb of data.

#### Genotyping with the Illumina Infinium Global Screening Array

We attempted to genotype all 97 of the collected samples using the Illumina Infinium Global Screening BeadChip Array-24 v1.0 (GSAMD-24v1–0) for 700,078 loci at the National Cancer Institute's Division of Cancer Epidemiology and Genetics (Bethesda, MD, USA) [50]. Data were analyzed by using the standard Illumina microarray data analysis workflow. During quality control (QC), samples were filtered for contamination, completion rate, and relatedness. As part of QC, we performed ancestry assessment using SNPweights software [45] with a reference panel consisting of 3 populations (European, West African, and East Asian). All samples were attributed to the European ancestry group. After QC and sample exclusion, 87 (86 samples and 1 QC) samples with 689,918 loci and completion rate of 99.9% were retained for further analysis.

### Variant calling

#### Variant calling of the BGISEQ-500 data

The sequencing data produced using the BGISEQ-500 platform for 97 samples were analyzed using the Sentieon tools (Sentieon Inc., San Jose, CA, USA) high-performance implementation of the BWA/GATK best practices pipeline on servers hosted by the Cornell University Biotechnology Resource Center. Reads were aligned to the GRCh38 human reference genome using BWA-MEM (Version: 0.7.16a-r1181), and mapped reads were prepared for variant calling using GATK (v3.8-1-0-gf15c1c3ef by Broad), including marking duplicates (picard MarkDuplicates, Version 2.12.1), indel realignment (GATK RealignerTargetCreator,IndelRealigner,Version 3.7-0), and base quality score recalibration (GATK BaseRecalibrator,PrintReads,Version 3.7-0). SNP and indel discovery were performed for each individual using GATK HaplotypeCaller and merged into a single pVCF using bcftools. Sample EG600036 was also run without joint calling, which was used when calculating concordance between the Illumina and BGISeq variant callsets, estimated with TiTvtools and visualized by plotTiTv [19].

#### Repetitive variant calling

Mobile element discovery was performed using MELT (Version 2.2.0) [51] and structural variant discovery using lumpy-sv with Smoove (Version 0.2.5) [16]. Short tandem repeats were called using GangSTR (Version 2.4.2) [52] and nuclear mitochondrial DNA using dinumt [53].

### Data validation and quality control

Variant files were compared for consistency across the 3 different platforms: BGISEQ-500 sequencing, Illumina genotyping, and Illumina NovaSeq6000 S4 sequencing. Illumina genotyping was performed on 86 of the 97 samples previously sequenced with BGISEQ-500. Additionally, 1 sample (EG600036) was also sequenced with Illumina NovaSeq6000 S4. The variant detection programs were rerun without joint calling for the BGISEQ-500 sequencing for sample EG600036 for comparison with the single Illumina-sequenced sample. In this sample, the SNPs derived from the WGS platforms were compared to those identified using the Illumina SNP array for both matching position and matching genotype. Structural variants and MEIs were compared between the WGS platforms in EG600036. Variants were considered the same if they had 95% reciprocal overlap. Overall, we found that Illumina identified a higher number of larger variants than BGISEQ-500. This could potentially be due to its higher coverage (∼60×) compared to BGISEQ-500 (∼30×). However, because both have high coverage, we may see diminishing returns for coverage >30×. An alternative explanation is that the variant identification tools have been built to detect variation from Illumina sequencing data and therefore may not be able detect variants in BGISEQ-500 data as accurately.

### Annotation

Sequence variant files were annotated using ANNOVAR (ANNOVAR, RRID:SCR_012821) [54] and SNPEff (SNPEff, RRID:SCR_005191) [55] software using GRCh38 reference databases. The following databases were used for the For ANNOVAR annotations: RefSeq Gene, 1GP superpopulation, dbSNP150 with allelic splitting and left-normalization. For annotation of the medically related and functional variants we used ClinVar Version 20200316 [30], InterVar gnomeAd Version 3.0 [12], and dbnsfp Version 35c [56]. For SNPEff, the default GRCh38 annotation database [57] was complemented with ClinVar (ClinVar, RRID:SCR_006169) [30] and GWAS catalog [29] database annotation using the snpSift tool (snpSift, RRID:SCR_015624) [58].

### Population analysis

#### Principal component analysis

For PCA, we used WGS variants of our samples and merged them with samples from neighboring countries available from the European samples from 1KG (Utah residents [CEU] with Northern and Western European ancestry, Toscani in Italy [TSI], Finnish in Finland [FIN], British in England and Scotland [GBR], Iberian population in Spain [IBS] [14, 39]) and French (FRA) and Russians (RUS) from HGDP [40], as well as the relevant high-coverage human genomes Croatian (CRO), Czech (CZ), Estonian (EST), German (GER), Greek (GRE), Hungarian (HUN), Moldovan (MOL), Polish (POL), Russian Cossack (RUS), and Ukrainian (UKR) from the EGDP [43], and the Simons Genome Diversity Project [44]. The analysis was performed with Eigensoft (Eigensoft, RRID:SCR_004965) [48].

To produce a meaningful number of alleles to analyze, the resulting dataset was filtered by genotyping rate (1) and pruned for variants in linkage disequilibrium by excluding those with high pairwise correlation within a moving window (–indep-pairwise 50 10 0.5). This resulted in 677 samples with 208,945 variants. We used EIGENSOFT [48] to calculate the eigenvectors, of which PC1 and PC2 were visualized using Python programming language, with pandas, matplotlib, and seaborn libraries [59]. Two extreme outlier samples (EG600056, and EG600052) were excluded from the visible range of the PCA plot because they clustered with each other far away from any known European group.

#### Model-based population structure analysis

For the naive (model-based) structure analysis, we used the same dataset described in the PCA (above). The analysis was performed using ADMIXTURE software (ADMIXTURE, RRID:SCR_001263) [49]. For identification of the optimal K parameter, we used the 10-fold cross-validation function of ADMIXTURE in the range 2–6, with K = 3 resulting in the lowest error, deeming it optimal. The results were visualized using Python programming language, with pandas, matplotlib, and seaborn libraries [59, 60] to construct a population structure plot using samples from the 1KG (Utah residents [CEU] with Northern and Western European ancestry, Toscani in Italy [TSI], Finnish in Finland [FIN], British in England and Scotland [GBR], and Iberian population in Spain [IBS]) and French population (FRA) [14, 39] and Russians (RUS) from HGDP [40], as well as the relevant high-coverage human genomes from the EGDP [43], and Simons Genome Diversity Project [44]. The resulting plot with K = 3 is presented in Fig. 3, and plots with K = 4 to K = 8 are in Supplementary Fig. S3.

#### Inbreeding estimates

We estimated inbreeding coefficients for all the genotype samples in the same dataset. For this analysis the samples were pruned for genotyping rate (>0.9) and linkage disequilibrium by excluding those with high pairwise correlation within a moving window (PLINK parameter –indep-pairwise 50 10 0.1). Using the resulting dataset containing the remaining 117,641 loci from 84 samples, we performed several inbreeding estimates: (i) method-of-moments F-coefficient estimates, (ii) variance-standardized relationship minus 1 estimates, and (iii) F-estimates based on correlation between uniting gametes [61]. All the resulting values are presented in Supplementary Table S7, and the method-of-moments F-coefficient estimates are visualized in a histogram (Supplementary Fig. S4).

#### Reuse potential

Since the publication of the first human genome [62, 63] and the first surveys of worldwide variation such as 1KG [14, 39], efforts have been directed outward, to expanding the exploration of human diversity across the world and filling out more and more “white spots” of genome variation [13, 44], as well as inward, to fill the remaining white spots in the human genome itself: to map the remaining gaps in the chromosome assembly and identify new structural and functional variation [64] and to map the 3D structure of the human genome [65]. The new data present a valuable addition to the former and represent the first exploration of the genome landscape in the important component of European genomic diversity.

The genome diversity of Ukraine is an important clue to advance modern genome studies of the population history of Europe. The country is positioned in the crossroads of the early migration of modern humans and the westward expansion of the Indo-Europeans, and represents an aftermath of centuries of migration, admixture, and demographic and selective processes. As wave after wave of great human migrations moved across this land for millennia, they were followed by the exchange of cultural knowledge and technology along the great trade routes that continue to transect this territory until the present day.

The justifications for collecting, sequencing, and analyzing populations from this part of Europe have been outlined previously [10, 66], and the new database is a step in that direction. Given its unique history, the genome diversity data from Ukraine will contribute a wealth of new information, bringing forth different risk and/or protective alleles that neither exist nor associate with disease elsewhere in the world. This project identified 13M variants in Ukrainians of which 478,000 were novel genomic SNPs currently missing from global surveys of genomic diversity [12, 13]. We also report almost 1M (909,991) complex indels, regions of simultaneous deletions and insertions of DNA fragments of different sizes that lead to a net change in length, with only 713,858 previously reported in gnomAD [12] (Table 1). The newly discovered local variants can be used to augment the current genotyping arrays and used to screen individuals with genetic disorders in GWAS, in clinical trials, and in genome assessment of proliferating cancer cells.

The present project is built upon the open release/access philosophy. The data have been released and can be used to search for population ancestry markers, as well as medically related variants, in subsequent studies. The public nature of the data deposited on the specially created web resource located at Uzhhorod National University will ensure that the nation's biomedical researchers will receive access to a useful information resource for future projects in genomics, bioinformatics, and personalized medicine. Engaging local Ukrainian scientists in this collaborative international project lays the foundation for future studies and ensures their participation in the worldwide research community.

## Data Availability

The raw reads are available at the SRA (Project PRJNA661978, SUB7904361). All other datasets mentioned in this project are available in *GigaScience* GigaDB [11].

## Additional Files

**Supplementary Table S1:** Sequencing summaries of output from BGISEQ-500 and Illumina NovaSeq6000 S4. Full sequencing statistics for individual samples in Table S1.2.

**Supplementary Table S2:** Filtering summary of the data obtained from 97 whole genomes sequenced with BGISEQ-500.

**Supplementary Table S3:** The full list of high-impact functional variants (including frameshift, start lost/stop lost or gained, transcript ablations, and splice alterations) that had an allele count of ≥2 with their predicted function, number of gene transcripts of the gene affected, and frequencies.

**Supplementary Table S4:** List of the medically relevant functional markers found in the Ukrainian population and reported in (A) GWAS catalog [29] and (B) ClinVar [30] databases. Allele frequency is reported compared to the reference allele in GRCh38.

**Supplementary Table S5:** Complete list of the highly differentiating markers, reported in ClinVar [30], with high differences in the Ukrainian population compared with other neighboring European populations (the combined sample from Western and Central Europe from 1KG with French samples from HGDP [EUR]) [14, 39, 40] and Russians (RUS) from HGDP [40]. Non-reference allele frequency (NAF) is reported compared with the reference allele in GRCh38. Differences are evaluated by the Fisher exact test (FET).

**Supplementary Table S6:** A list of markers with the highest non-reference allele frequency (NAF) differences in the Ukrainian population evaluated by the Fisher exact test (FET) compared with the frequencies in neighboring populations: the combined population from Europe (EUR) [14] and Russians (RUS) from HGDP [40]. This database contains candidate ancestry informative markers (AIMs) [44] that can be used for mapping disease alleles by admixture disequilibrium [46, 47].

**Supplementary Table S7:** Inbreeding estimates in a dataset of 117,641 loci from 84 samples: (a) method-of-moments F-coefficient estimates, (b) variance-standardized relationship minus 1 estimates, and (c) F-estimates based on correlation between uniting gametes [61].

**Supplementary File S1:** IRB approval of the study “Genomic Diversity of Ukraine's Population” (*in Ukrainian*).

**Supplementary File S2:** Genomic Diversity of Ukraine's Population Project: Protocol description, questionnaire, and informed consent to participate and publish (*in Ukrainian with English translation*).

**Supplementary File S3:** The list of the samples in this study, their characteristics and geographical locations, and sources of genomic data for each (BGISEQ-500 sequencing [BGI Inc., Shenzhen, China], Illumina Global Screening Array genotyping, and Illumina NovaSeq sequencing array [Illumina Inc., San Diego, USA]).

**Supplementary File S4:** List of the samples from different studies used in the present population analysis.

**Supplementary File S5:** Sample sources.

**Supplementary Figure S1:** Transition/transversion (TITV) ratio for the novel SNPs (estimated with TiTvtools [18] and visualized by plotTiTv) (top) for the SNPs where Illumina SNP array identified more alternate haplotypes than BGI (top right triangle in Fig. 1C) and (bottom) for the SNPs where BGISeq identified more alternate haplotypes than Illumina SNP array (bottom left triangle in Fig. 1C table).

**Supplementary Figure S2:** A. Frequencies of various classes of SNPs in the Ukrainian genome variation database. Categories are defined as follows: singleton (passed the GATK QC once), doubleton, rare (3–10 counts roughly equivalent to 1%< x < 5%), and common (>5%) to more closely approximate the 1KGP definitions. B. Percent novel mutations in various classes of SNPs.

**Supplementary Figure S3:** Genetic structure of Ukrainian population in comparison with other European populations. For identification of the optimal K parameter, we used the 10-fold cross-validation function of ADMIXTURE in the range 2–8, with K = 3 resulting in the lowest error [49]. This analysis included genomes from this study as well as samples from the 1KG (Utah residents [CEU] with Northern and Western European ancestry, Toscani in Italy [TSI], Finnish in Finland [FIN], British in England and Scotland [GBR], and Iberian population in Spain [IBS]) [14, 39] and French (FRA) and Russians (RUS) from HGDP [39], as well as the relevant high-coverage human genomes Croatian (CRO), Czech (CZ), Estonian (EST), German (GER), Greek (GRE), Hungarian (HUN), Moldovan (MOL), Polish (POL), Russian Cossack (RUS), and Ukrainian (UKR) from the Estonian Biocentre Human Genome Diversity Panel (EGDP) [43], as well as Simons Genome Diversity Project [44].

**Supplementary Figure S4:** Distribution of inbreeding coefficients in the Ukrainian sample. The individual values corresponding to the samples are presented in Table S7.

## Abbreviations

1KG: 1000 Genomes Project; AIM: ancestry informative marker; ALU: Alu elements; bp: base pairs; BWA: Burrows-Wheeler Aligner; DNB: DNA nanoball; DUP: duplications; EGDP: Estonian Biocentre Human Genome Diversity Panel; GATK: Genome Analysis Toolkit; Gb: gigabase pairs; gnomAD: Genome Aggregation Database; GWAS: genome-wide association studies; HGDP: Human Genome Diversity Project; INV: inversions; IRB: institutional review board; MEI: mobile element insertions; NCBI: National Center for Biotechnolgy Information; NIH: National Institutes of Health; NUMT: nuclear mitochondrial DNA; PC: principal component; PCA: principal component analysis; QC: quality control; SRA: Sequence Read Archive; SVA: non-autonomous retroelements; TFBS: transcription factor binding site ablation; TITV: transition/transversion; UAU: Ukrainians from Ukraine; WGS: whole-genome sequencing.

## Consent for Publication

The collection procedure was approved as part of the “Genome Diversity in Ukraine” project by the IRB of Uzhhorod National University, Uzhhorod, Ukraine (Supplementary File S1). Each participant had an opportunity to review the informed consent materials (Supplementary File S2) and received an explanation of the issues around the sharing of genome data before making any decisions about making them public in this manner.

## Competing Interests

Y.L. and H.Y. are employed by BGI, which owns DNBSEQ™ technology; Olga Levchuk, Alla Patrus, and Nelya Lazar represent AstraDIA (Ukraine), which collected and extracted DNA samples. All other authors declare that they have no competing interests.

## Funding

This research was funded in part by internal funding from BGI (China), Uzhhorod National University (Ukraine), Division of Cancer Epidemiology and Genetics, National Cancer Institute (USA), and the startup fund of Oakland University, Rochester, Michigan.

## Authors' Contributions

**Conceptualization:** T.K.O., K.S., W.W.W., S.C., H.Y., Y.L., J.L.R.F., F.B, O.T.O., M.D., M.Y., R.E.M., V. Smolanka

**Data curation:** W.W.W., K.S., A.W., A.U., P.B., V. Stakhovska, K.M., Y.H., N.L., O.T.O., M.N., S.C., O.P., N.V.

**Formal analysis:** W.W.W., A.W.

**Funding acquisition:** T.K.O.

**Investigation:** K.S., W.W.W., A.W., S.O.C.M.

**Methodology:** T.K.O., M.D., R.E.M.

**Project administration:** T.K.O., M.D., V. Smolanka

**Resources:** T.K.O., H.Y., Y.L., R.E.M., M.Y., M.D., O.T.O., V. Smolanka

**Software:** W.W.W., A.W.

**Supervision:** T.K.O., V. Smolanka, M.D., R.E.M.

**Visualization:** W.W.W., K.S., A.W.

**Writing:** T.K.O., K.S., W.W.W., A.W. (original draft), and T.K.O. and R.E.M. (review and editing).

## Supplementary Material

giaa159_GIGA-D-20-00230_Original_Submission

giaa159_GIGA-D-20-00230_Revision_1

giaa159_GIGA-D-20-00230_Revision_2

giaa159_Response_to_Reviewer_Comments_Original_Submission

giaa159_Response_to_Reviewer_Comments_Revision_1

giaa159_Reviewer_1_Report_Revision_1 Jong Hwa Bhak, Ph.D. -- 8/25/2020 Reviewed

giaa159_Reviewer_2_Report_Original_Submission Chaochun Wei -- 9/14/2020 Reviewed

giaa159_Reviewer_2_Report_Revision_1 Chaochun Wei -- 11/10/2020 Reviewed

giaa159_Supplemental_Files
