# Supplementary material for: Genome diversity in Ukraine
Source: Gigascience. 2021 Jan 13;10(1):giaa159. doi: 10.1093/gigascience/giaa159 (PMC7804371; doi:10.1093/gigascience/giaa159)
Supplement: giaa159_GIGA-D-20-00230_Revision_2 [file giaa159_giga-d-20-00230_revision_2.pdf]

GigaScience

Genome Diversity in Ukraine

--Manuscript Draft--

**Manuscript Number:**	GIGA-D-20-00230R2
**Full Title:**	Genome Diversity in Ukraine
**Article Type:**	Data Note
**Funding Information:**	Oakland University — Dr Taras K Oleksyk<br>BGI — Dr Taras K Oleksyk
**Abstract:**	The main goal of this collaborative effort is to provide genome wide data for the previously underrepresented population in Eastern Europe, and to provide cross-validation of the data from genome sequences and genotypes of the same individuals acquired by different technologies. We collected 97 genome-grade DNA samples from consented individuals representing major regions of Ukraine that were consented for the public data release. BGISEQ-500 sequence data and genotypes by an Illumina GWAS chip were cross-validated on multiple samples, and additionally referenced to one sample that has been resequenced by Illumina NovaSeq6000 S4 at high coverage. The genome data has been searched for genomic variation represented in this population, and a number of variants have been reported: large structural variants, indels, CNVs, SNPs and microsatellites. This study provides the largest to-date survey of genetic variation in Ukraine, creating a public reference resource aiming to provide data for historic and medical research in a large understudied population. While most of the common variation is shared with other European populations, this survey of population variation contributes a number of novel SNPs and structural variants that have not been reported in the gnomAD/1KG databases representing global distribution of genomic variation. These endemic variants will become a valuable resource for designing future population and clinical studies, help address questions about ancestry and admixture, and will fill a missing place in the puzzle characterizing human population diversity in Eastern Europe. Our results indicate that genetic diversity of the Ukrainian population is uniquely shaped by the evolutionary and demographic forces and cannot be ignored in the future genetic and biomedical studies. This data will contribute a wealth of new information bringing forth different risk and/or protective alleles. The newly discovered low frequency and local variants can be added to the current genotyping arrays for genome wide association studies, clinical trials, and in genome assessment of proliferating cancer cells.
**Corresponding Author:**	Taras K Oleksyk, Ph.D.<br>Oakland University<br>Rochester, MI UNITED STATES
**Corresponding Author Secondary Information:**	
**Corresponding Author's Institution:**	Oakland University
**Corresponding Author's Secondary Institution:**	
**First Author:**	Taras K Oleksyk, Ph.D.
**First Author Secondary Information:**	
**Order of Authors:**	Taras K Oleksyk, Ph.D.
	Walter W. Wolfsberger, M.S.
	Alexandra Weber
	Khrystyna Shchubelka, M.D.
	Olga T. Oleksyk, M.D., Ph.D.
	Olga Levchuk, Ph.D.

Powered by Editorial Manager® and ProduXion Manager® from Aries Systems Corporation

	Alla Patrus
	Nelya Lazar, M.D.
	Stephanie O. Castro-Marquez
	Patricia Boldyzhar, M.D.
	Mikhailo Neymet, M.D.
	Alina Urbanovych, M.D.
	Viktoriya Stakhovska, M.D.
	Kateryna Malyar
	Svitlana Chervyakova, M.D.
	Olena Podoroha, M.D.
	Natalia Kovalchuk
	Yaroslava Hasynets, Ph.D.
	Juan L. Rodriguez-Flores
	Sarah Medley
	Fabia U. Battistuzzi
	Siru Chen
	Ryan Liu
	Yong Hou, Ph.D.
	Huanming Yang, Ph.D.
	Meredith Yeager, Ph.D.
	Michael Dean, Ph.D.
	Ryan E. Mills, Ph.D.
	Volodymyr Smolanka, M.D., Ph.D.
**Order of Authors Secondary Information:**	
**Response to Reviewers:**	Dear Dr. Edmunds:<br><br>Thank you for considering our communication presenting a population genomics dataset from a largely unexplored region of Eastern Europe, entitled "Genome Diversity in Ukraine". We have carefully considered your comments and comments from the reviewers, answered them to our best ability and made the appropriate changes in the revised manuscript.<br><br>After our last communication we have revised the additional data to exclude anything that has not been specifically published or publicly released. Specifically, we have replaced the recently retracted dataset from Russia with the newly released data from HGDP.<br><br>I would like to thank you again for taking time to review our manuscript, and to the reviewers for the helpful suggestions. I think that you will find our efforts satisfactory, and looking forward to publication of this important data<br><br>Sincerely yours<br><br>Taras Oleksyk<br><br><br>ANSWERS TO THE REVIEWERS:

Powered by Editorial Manager® and ProduXion Manager® from Aries Systems Corporation

Reviewer #1:

This manuscript describes 97 genome sequences from Ukraine.
All the sequencing, processing, and data depository are done professionally and the analyses are at standard quality.
This reviewer thinks this genomic resource should be publicized as soon as possible:
1) The data set are unique enough
2) The data set contains nearly 100 whole genome sequences.

ADMIXTURE and PCA show the expected characteristics of the population and its history.

Answer: Thank you very much for a kind review. We have undated the ADMIXTURE and PCA to reflect the currently publicly available data to which we hope to contribute with our research.

Reviewer #2:

This paper reported 97 whole sequencing data of representative Ukrainians and their variation annotations using DNBSEQ-G50 sequencing platform. Illumina NovaSeq6000 S4 sequencing for one individual was done as quality control of sequencing. Genomic variants including SVs, indels, CNVs, SNPs and microsatellites were annotated and compared to neighboring populations. The goal of this paper is to provide a genomic resource for Eastern Europe. A few issues are listed below.

Major issues

Comment 1. Accession numbers or IDs for all data are not provided yet.

Answer:
1)The list of the cross validated samples and the source technology of the data is presented in the Supplementary File 3. All the supplementary materials mentioned in the paper are uploaded to GigaScience ftp: and should be accessible to the editors

2)Additionally, all the reads are uploaded to NCBI SRA and are processed, and ready to release:
SRA submission information: SUB7904361
BioProject status: Processed
PRJNA661978: Ukrainian Genomes \ UA genomes
BioSample: Processed
SRA: Processed

We can add reviewers to our project team and provide them early access to the reads before the paper is released.

Comment 2. In Table S2, the number of SNPs, filtered counts and percentage filtered has no value.

Answer: We updated Table S2 with the most current data as it is submitted in the corrected article.

Comment 3. Will the sequencing depth impact the assembled genomes? For example, the illumine sequencing depth was 60 while DNBSEQ-G50 sequencing data have about 30X coverage.

Answer: We only sampled only one sample with Illumina technology and only for the comparison. Higher coverage of the Illumina data (60x) could have contributed to the differences observed between the platforms.is why there was more SNPs identified and higher concordance. However, since there Is only one sample sequenced with

Powered by Editorial Manager® and ProduXion Manager® from Aries Systems Corporation

	both platforms so we cannot make further conclusions from this comparison.<br><br>Action: We have modified paragraph 2 page 6: “Evaluation tests show that current algorithms are platform dependent, in the sense that they exhibit their best performance for specific types of structural variation as well as for specific size ranges [21], and the algorithms designed for detection and archived datasets are predominantly for Illumina pair-end sequencing [22,23]. While it is possible that these results indicate Illumina’s superiority at detecting structural variation, it also can also be the consequence of the bioinformatics tools for calling structural variants developed using mainly the Illumina data, as suggested by previous comparative evaluations of the two technologies [24,25]. Additionally, higher coverage of the Illumina data (60x) could have contributed to the differences observed between the platforms.”<br><br>Comment 4. In the first paragraph of page 14, the authors mentioned that "genetics is not a reliable determinant of ethnicity", this is conclusion is not well supported with evidences. Another explanation can be that the self-identified ethnic group is not reliable.<br><br>Answer: What reviewer has in mind is probably human population, not ethnicity.<br><br>We believe that the term “ethnicity” recognizes differences between people mostly on the basis of language and shared culture. Oxford Dictionary defines “ethnicity” as “as the fact or state of belonging to a social group that has a common national or cultural tradition”. Wikipedia defines it as “an ethnic group or ethnicity is a named social category of people who identify with each other on the basis of shared attributes that distinguish them from other groups such as a common set of traditions, ancestry, language, history, society, culture, nation, religion, or social treatment within their residing area”. Encyclopedia Britannica defines ethnicity as a characteristic that “relates to culturally contingent features, characterizes all human groups. It refers to a sense of identity and membership in a group that shares common language, cultural traits (values, beliefs, religion, food habits, customs, etc.), and a sense of a common history.”<br><br>Therefore, we still believe that individual ethnicity cannot be defined in genetic sense. A Chinese baby adopted in Ukraine and raised in a Ukrainian cultural environment will have a Ukrainian ethnicity despite of its genes, due to this person’s upbringing.<br><br>On the other hand, genetics clearly can still assign people to ancestral populations with high certainty, even in places like Eastern Europe, where differences between populations have been traditionally ignored by the medical research.<br><br>Action: We modified sentence in Paragraph 1, page 13. “Genetics is not a reliable determinant of ethnicity, but can be used to evaluate contributions of population ancestry”.<br><br>Minor issues<br><br>1. There are some language issues. For example, in the first paragraph of Page 4, "while the ethnic Ukrainians constitute approximately than three quarters of the total population of the modern Ukraine". "than" in this sentence should be removed.<br><br>Action: we corrected this typo: “while the ethnic Ukrainians constitute approximately three quarters of the total population of the modern Ukraine”. We additionally screened the manuscript for typos and grammatical errors and fixed what we could.
**Additional Information:**	
**Question**	**Response**
Are you submitting this manuscript to a special series or article collection?	No

**Experimental design and statistics**<br><br>Full details of the experimental design and statistical methods used should be given in the Methods section, as detailed in our Minimum Standards Reporting Checklist. Information essential to interpreting the data presented should be made available in the figure legends.<br><br>Have you included all the information requested in your manuscript?	Yes
**Resources**<br><br>A description of all resources used, including antibodies, cell lines, animals and software tools, with enough information to allow them to be uniquely identified, should be included in the Methods section. Authors are strongly encouraged to cite Research Resource Identifiers (RRIDs) for antibodies, model organisms and tools, where possible.<br><br>Have you included the information requested as detailed in our Minimum Standards Reporting Checklist?	Yes
**Availability of data and materials**<br><br>All datasets and code on which the conclusions of the paper rely must be either included in your submission or deposited in publicly available repositories (where available and ethically appropriate), referencing such data using a unique identifier in the references and in the "Availability of Data and Materials" section of your manuscript.<br><br>Have you have met the above requirement as detailed in our Minimum Standards Reporting Checklist?	Yes

Manuscript
Click here to access/download;Manuscript;Ukrainian Genomes (TKO 2020).SCEcomments.docx

DATA NOTE

Genome Diversity in Ukraine

Taras K. Oleksyk [1,2,3,·], Walter W. Wolfsberger [1,2,3#], Alexandra Weber [4#], Khrystyna Shchubelka [2,3,5#], Olga T. Oleksyk [6], Olga Levchuk [7], Alla Patrus [7], Nelya Lazar [7], Stephanie O. Castro-Marquez [2,3], Patricia Boldyzhar [5], Mikhailo Neymet [8]; Alina Urbanovych [9], Viktoriya Stakhovska [10], Kateryna Malyar [11], Svitlana Chervyakova [12], Olena Podoroha [13], Natalia Kovalchuk [14], Yaroslava Hasynets [1], Juan L. Rodriguez-Flores [15], Sarah Medley [2], Fabia Battistuzzi [2], Ryan Liu [15], Yong Hou [15], Siru Chen [15], Huanming Yang [15], Meredith Yeager [17], Michael Dean [17], Ryan E. Mills [18,·], and Volodymyr Smolanka [5]

[1] Department of Biological Sciences, Uzhhorod National University, Uzhhorod 88000, Ukraine;
[2] Department of Biological Sciences, Oakland University, Rochester, MI 48309, USA;
[3] Departamento de Biología, Universidad de Puerto Rico, Mayagüez, 00682, Puerto Rico;
[4] Department of Computational Medicine and Bioinformatics, University of Michigan, Ann Arbor, MI, 48109, USA;
[5] Department of Medicine, Uzhhorod National University, Uzhhorod 88000, Ukraine;
[6] A. Novak Transcarpathian Regional Clinical Hospital, Uzhhorod 88000, Ukraine;
[7] Astra Dia Inc., Uzhhorod 88000, Ukraine
8 Velyka Kopanya Family Hospital, Transcarpatia 90330, Ukraine;
[9] Lviv National Medical University, Lviv 79010, Ukraine
[10] Zhytomyr Regional Hospital, Zhytomyr 10002, Ukraine
[11] I.I.Mechnikov Dnipro Regional Clinical Hospital, Dnipro 49000, Ukraine
[12] Chernihiv Regional Hospital, Chernihiv 14039, Ukraine
[13] Sumy Diagnostic Center, Sumy 40000, Ukraine
[14] Rivne Regional Specialized Hospital of Radiation Protection, Rivne 33028, Ukraine
[15] Department of Genetic Medicine, Weill Cornell Medical College, New York, NY 10065, USA
[16] BGI, Shenzhen CHINA;
[17] Division of Cancer Epidemiology and Genetics, National Cancer Institute, Bethesda, MD 20892, USA;
[18] Department of Human Genetics, University of Michigan, Ann Arbor, MI, 48109, USA;

·*Corresponding address: Dr. Taras K. Oleksyk. E-mail: oleksyk@oakland.edu or Dr. Ryan Mills. E-mail: remills@umich.edu*
# *these authors contributed equally*

ORCIDs:
Taras K. Oleksyk, 0000-0002-8148-3918;
Juan L. Rodriguez-Flores, 0000-0002-0394-8062;
Yong Hou, 0000-0002-0420-0726;
Huanming Yang, 0000-0002-0858-3410;
Michael Dean, 0000-0003-2234-0631;
Ryan Mills, 0000-0003-3425-6998;
Volodymyr Smolanka, 0000-0001-7296-8297.

Abstract

The main goal of this collaborative effort is to provide genome wide data for the previously underrepresented population in Eastern Europe, and to provide cross-validation of the data from genome sequences and genotypes of the same individuals acquired by different technologies. We collected 97 genome-grade DNA samples from consented individuals representing major regions of Ukraine that were consented for the public data release. BGISEQ-500 sequence data and genotypes by an Illumina GWAS chip were cross-validated on multiple samples, and additionally referenced to one sample that has been resequenced by Illumina NovaSeq6000 S4 at high coverage. The genome data has been searched for genomic variation represented in this population, and a number of variants have been reported: large structural variants, indels, CNVs, SNPs and microsatellites. This study provides the largest to-date survey of genetic variation in Ukraine, creating a public reference resource aiming to provide data for historic and medical research in a large understudied population. While most of the common variation is shared with other European populations, this survey of population variation contributes a number of novel SNPs and structural variants that have not been reported in the gnomAD/1KG databases representing global distribution of genomic variation. These endemic variants will become a valuable resource for designing future population and clinical studies, help address questions about ancestry and admixture, and will fill a missing place in the puzzle characterizing human population diversity in Eastern Europe. Our results indicate that genetic diversity of the Ukrainian population is uniquely shaped by the evolutionary and demographic forces and cannot be ignored

in the future genetic and biomedical studies. This data will contribute a wealth of new information bringing forth different risk and/or protective alleles. The newly discovered low frequency and local variants can be added to the current genotyping arrays for genome wide association studies, clinical trials, and in genome assessment of proliferating cancer cells.

Keywords: genomes, NGS, genotyping, variant calling, copy number polymorphisms, SNP, CNV, indels, BGISEQ-500, DNBSEQ, Illumina

Data Description

The context

Ukraine is the largest country located fully in Europe with a population that was formed as a result of several millennia of migration and admixture. It occupies the intersection between the westernmost reach of the great steppe and the easternmost extent of the great forests that spread across Europe, at the crossroad of the great trade routes from "Variangians to the Greeks" along the river Dnipro, which the ancient Greeks referred to as Borysthenes, and the Silk Road linking civilizations of Europe and Asia [1]. This land has seen the great human migrations of the Middle Ages sweeping from across the great plains, and even before that in the more distant past, of the early farmers [2] and the nomads who first domesticated the horse [3–6]. Here, at the dawn of the modern human expansion, our ancestors met the Neanderthals who used to hunt the great game along the glacier of the Ice Age [7,8].

The rich history shaped genetic diversity in the population living in the country of Ukraine today. As people have moved and settled across this land, they have contributed unique genetic variation that varies across the country. While the ethnic Ukrainians constitute approximately more than three quarters of the total population, this majority is not uniform. A large Russian minority compose approximately one-fifth of the total population with higher concentration in the southeast of the country. Smaller minority groups are historically present in different parts of the country: Belarusians, Bulgarians, Crimean Tatars, Greeks, Gagauz, Hungarians, Jews, Moldovans, Poles, Romanians, Roma (Gypsies), and others [9].

This study offers genome data from 97 individuals from Ukraine (Ukrainians from Ukraine or UAU) to the scientific community in order to help fill the gaps in the current knowledge about the genomic variation in Eastern Europe, a part of the world that has been largely and consistently overlooked in the global genomic surveys [10]. This was the first effort to describe and evaluate the genome wide diversity in Ukraine. Samples were successfully sequenced using BGI's DNA Nanoball (DNBSEQ™) sequencing technology, and cross-validated by Illumina sequencing and genotyping. The major objectives of this study were to demonstrate the importance of studying local variation in the region and to demonstrate the distinct and unique genetic components of this population. Of particular interest were the medically related variants, especially those with allele frequencies that differed with the neighboring populations. As a result, we present and describe an annotated dataset of genome-wide variation in genomes of healthy adults sampled across the country.

The dataset

The new dataset includes 97 whole genomes of self-reported Ukrainians from Ukraine at 30x coverage sequenced using BGISEQ-500 (one of the range of DNBSEQ™ sequencers; BGI Inc., Shenzhen, China)

and annotated for genomic variants: SNPs, indels, structural variants and mobile elements. The samples have been collected across the entire territory of Ukraine, after obtaining the IRB approval (Protocol #1 from 09/18/2018, **Supplementary File 1**) for the entire study design, and informed consent from each participating volunteer (**Supplementary File 2**). Each participant in this study had an opportunity to review the informed consent, have been explained the nature of the genome data, and made a personal decision about making it public.

The majority of samples in this study (86 out of 97) were additionally genotyped using Illumina Global Screening Array (Illumina Inc., San Diego, USA) in order to confirm the accuracy of base calling between the two platforms. In addition, one sample (EG600036) was also sequenced on the Illumina NovaSeq 6000 S4 (2x150 bp; ~60x coverage) and used for validation of the variant calls (see summary in **Table S1,** and full sequencing statistics for individual samples in **Table S1.2**). The list of the cross validated samples and the source technology of the data is presented in the **Supplementary File 3**.

The current dataset contains locations and frequencies of more than 13M unique variants in Ukrainians from Ukraine (**UAU**) which are further interrogated for functional impact and relevance to the medically related phenotypes (**Table 1,** and data in GigaDB[11]). As much as 3.7% of these alleles, or 478 K, are novel genomic SNPs that have never been previously registered in the gnomAD database [12] (**Table 1**). This number is similar in magnitude to what was reported earlier in two populations from European Russia (3-4%; [13]). Many of the discovered variants (12.6%) are also currently missing from the global survey of genomic diversity in the 1,000 Genomes Project [14]. Majority of these described variants are rare or very rare (<5%; **Figure S2**).

As other indigenous ethnic groups from Ukraine (such as the Crimean Tatars or the Gagauz) are not included in the study, increasing the sample size above from 100 to 1,000 individuals is not likely to greatly contribute to discovery of novel mutations [15]. The proportion of the novel structural variants and mobile elements compared to the earlier databases is even higher: almost 1M (909,991) complex indels, regions of simultaneous deletions and insertions of DNA fragments of different sizes which lead to net a change in length, majority of which are novel (**Table 1**). Many of the newly discovered variants are functional and potentially contribute to the phenotype (classified in **Table 2).** We report many important variants that are overlooked or require special modifications in the commonly used resources and tools in genomic research and diagnostics. This wealth of novel variation underscores the importance of variant discovery in local populations that cannot be ignored in biomedical studies.

Table 1. Summary of variation in the 97 whole genome sequences from Ukraine.

Sequencing results	All samples			On average	
	Total Unique Variants #	Novel gnomAD Count	% Novel gnomAD (1000Genomes) [ƨ]	Average # /sample	Average # Novel /sample
**Total sequence reads**	99.8 Bn	--	--	1.03 Bn	--
**Mean coverage**	97 samples at 30X each	--	--	30X	--
**Variation**					
**SNPs**	13,010,979	477,564	3.7%(12.6%)	3,488,083	0.1% (0.7%)
**Bi-allelic**	12,667,283	470,667	3.7%(12.7%)	3,340,557	0.3%(0.6%)
**Multi-allelic**	343,696	6,897	2.0%(7.4%)	146,340	0.8%(4.7%)
**Small Indels [¥]**	2,727,604	76,484	2.8%(7.4%)	917,731	0.3% (1.0%)
**Deletions**	1,805,739	55,599	3.1% (9.0%)	624,919	0.3% (2.4%)
**Insertions**	1,4459,87	30,453	2.1%(6.7%)	571,461	0.2% (2.1%)
**Structural Variants [$]**					
**Large Deletions**	16,078	10,914	67.9(48.3%)	3,524	52.6%(19.1%)
**Large Duplications**	1,845	1,356	73.5%(42.3%)	562	89.4%(35.2%)
**Inversions**	337	314	93.2% (47.8%)	185	94.1%(48.6%)
**Mobile Element Insertions**					
**Alu**	2,316	1805	77.9%(38.1%)	473	68.1%(18.0%)
**L1**	451	289	64%(50.1%)	79	60.8%(27.8%)
**SVA**	100	75	75%(52.0%)	20	70%(50%)
**NUMT**	714	--	--	16	--

[ƨ] Defined as "percent not reported in gnomAD(1000Genomes)"

[¥] Small indels are insertions and deletions < 50bp called by GATK [16].

[$] Large deletions and duplications are those called by *lumpy* [17] which are > 50 bp.

Variant calling and confirmation

For each sample in the database, we estimated the number of passing bi-allelic SNPs calls (i.e. loci with the non-reference genotypes relative to the most current major human genome assembly, GRCh38 [18])(**Table 1**). Approximately 12% of these were filtered out based on excess heterozygosity and low variant quality scores (**Table S2**). For the indels, we also estimated the number of passing calls compared to GRCh38 and excluded 4% of those which did not pass filtering. The total number of the unique SNPs, small and large indels (**Table 1**) was calculated from the raw reads alignments of all the 97 sequenced genomes (**Total Unique SNPs, Table S2**) with the exception of those filtered out for low variant quality scores and containing excess heterozygosity (**Filtered Count**; **Table S2**). In addition, we filtered out 4,135,903 variants that only appeared once in a single sample (for both indels and SNPs) and designated them as "*singletons*".

We report a good correspondence between the SNP calls made using BGISEQ-500 and NovaSeq 6000 S4 data. A comparison of the variants detected using these three platforms for sample EG600036 are summarized in **Figure 1.A**. The SNP concordance for samples with both BGISEQ-500 and SNP array data is summarized in **Figure 1C**. The cross-platform comparison shows a very good overlap across all three technologies: with more than 3.5 M SNPs (or **97.7%**) of the SNPs identified in the BGISEQ-500 were also verified in the whole genome sequence of EG600036 sequenced by the Illumina NovaSeq 6000 S4. The correspondence with the Illumina SNP Array for sample EG600036 was also very good: **95.8%** of all the SNPs genotypes called by the Illumina method were also detected by the BGISEQ-500 (**Figure 1.A(Right), C(Right)**). The concordance between the non-reference alleles between the two platforms in all the 86 samples was nearly linear (r^2=0.985, **Figure 1.C(Left)**).

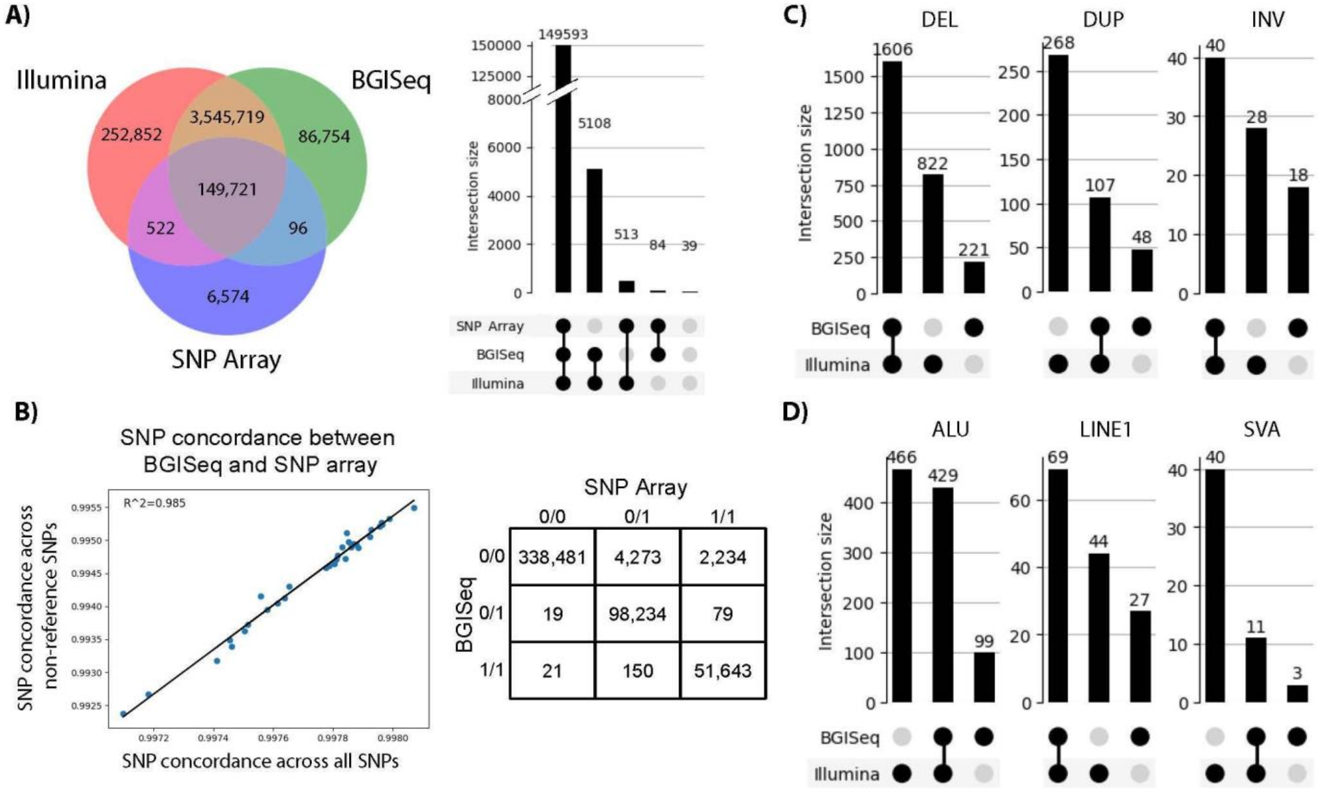

Figure 1. Variant concordance across the three sequencing/genotype methods: A) Left: Overlap of SNP positions identified in one sample (EG600036) using each of the three platforms. **Right:** Concordance of SNP genotypes in one sample derived from each of the three platforms. This only includes the subset of SNPs with alternate alleles included in the Illumina genotyping array (the smallest of the three variant sets). The variants indicated as belonging to none of the categories are variants whose genotypes differ between all three platforms. **B) Left:** The percentage (%) of concordance between the Illumina SNP array and BGISEQ-500 for all SNPs compared to the % concordance of only SNPs with non-reference alleles in the Illumina SNP array for the 86 samples genotyped on both platforms. **Right:** Concordance of SNP genotypes between BGISEQ-500 and Illumina SNP Array for one sample (EG600036). **C)** Overlap within the numbers of the three major structural variants detected in one sample using the two whole genome sequencing datasets. **D)** Overlap within the numbers of the three major mobile element insertions detected in one sample using the two whole genome sequencing datasets.

Transition/Transversion ratio (or TITV ratio) for the novel SNPs (estimated with *TiTvtools* [19] and visualized by *plotTiTv* in **Figure S1**) was lower than the TITV ratio for SNPs in the dbSNPs database (**1.9 vs 2.2**; [20]). Similarly, insertions to deletions (ins/del) ratio for novel indels is lower than for the indels already reported in the dbSNP database (**0.63 vs 0.75**). This observation likely reflects our improved ability to detect small insertions in newer sequencing technologies compared to many platforms which historically submitted variation to dbSNP.

We have defined the multi-allelic SNPs as observations of genomic positions having two or more alternative alleles [21]. These are important variants that are overlooked or require special modifications in the commonly used resources and tools in genomic research and diagnostics. We report a total of 343,696 multiallelic sites in the sequences from our sample of which 2.0% are at locations unreported in the gnomAD database [12] (**Table 1**).

In addition to the SNPs, we have identified and quantified major classes of structural variations in the Ukrainian population: small indels (insertions and deletions < 50bp), large structural variants (deletions, duplications and inversions > 50 bp) and Mobile Element Insertions (MEI)(Alu-s, L1 elements, non-autonomous retroelements (SVA), and nuclear mitochondrial DNA (NUMT) copies). A number of structural elements were reported, including common and novel ones. While among the small variants most were common (6-9%), a large proportion of large variants and MEIs (38-52%) have not been reported previously in the 1000Genomes Database (**Table 1**).

Once more, there is a significant correspondence between the calls made using BGISEQ-500 and Illumina NovaSeq 6000 S4 data. The two sequencing platforms show a significant overlap in calling indels (**DEL**): 87.9% of the variants called by the BGISEQ-500 were also detected by the Illumina platform. At the same time, there were 822 deletions, or 33.8% of all the indels called by the Illumina that were not detected by the BGISEQ-500 (**Figure 1.B**). A similar picture, where BGISEQ-500 performs competitively well, is also observed for inversions (**INV**)(**Figure 1.B**), and **LINE1** transposable elements (**Figure 1.D**). At the same time, more Duplications (**DUP**)(**Figure 1.B**), and the two classes of transposable elements evaluated: Alu elements (**ALU**) and the non-autonomous retroelements (**SVA**)(**Figure 1.D**). Evaluation tests show that current algorithms are platform dependent, in the sense that they exhibit their best performance for specific types of structural variation as well as for specific size ranges [22], and the algorithms designed for detection and archived datasets are predominantly for Illumina pair-end sequencing [23,24]. While it is possible that these results indicate Illumina's superiority at detecting structural variation, it also can also be the consequence of the bioinformatics tools for calling

structural variants developed using mainly the Illumina data, as suggested by previous comparative evaluations of the two technologies [25,26]. Additionally, higher coverage of the Illumina data (60x) could have contributed to the differences observed between the platforms.

The database was compared to the existing global resources of population variation such as Genome Aggregation Database (gnomAD)[12] and the 1000Genomes Project (1KG)[14]. Specifically, under our search criteria, the small variants (SNPs and Small Indels) were considered "*novel*" if they were absent from all the samples in the two global datasets (gnomAD and 1KG; **Table 1**). The large structural variants and Mobile Element Insertions were considered "novel" if the variant was not present in the gnomAD and 1KG databases. To determine if a given variant was present in one of the databases, a variant of the same type in the database had to overlap the Ukrainian variant with a minimum fraction of 0.95. We observed no significant deviation of the rate at which reference bases were observed at REF/alt heterozygous SNP sites (reference bias was near 50%).

Collection of functional variants

A particular interest in this study is the distribution of functional variation, not in the least due to the potential impact on phenotypes, especially to those with medical relevance [27]. As much as 97.5% of all annotated variation was discovered outside of the known functional elements (upstream, downstream, intron and intergenic). These results are similar to the expected distributions of mutations shown with the simulated data [28]. Nevertheless, there were more than 8,000 mutations discovered within exons of each individual on average (**Table 2.A**). We annotated several classes of functional mutations within the coding regions (**Table 2.B**). As expected, the nonsense mutations classified in the annotation file as "*Disruptive in-frame indel*", "*Start lost*", "*Stop gained*", and "*Stop loss*" were rare, while categories with minimal effect on the function, such as "*Synonymous*", "*Motiff*", "*Protein folding*", "*Missense*" were more common. Some of the mutations listed in the can be classified in more than one category (e.g. "*Synonymous variants*" can also be counted in "*Exonic variants*").

In addition to the gene coding mutations, we report a number of regulatory variants. For example, the database contains a total of 2,229 transcription factor binding site ablation (TFBS) mutations (**Table 2.B**). A summary of functional variation discovered in this study is presented in **Table 2.** The full list of high impact functional variants (including frameshift, start lost/stop lost or gained, transcript ablations and splice alterations) that had an allele count of two or more with their predicted function, number of gene transcripts of the gene affected, and frequencies is presented in **Table S3**. The full annotation database with classifications is available alongside the associated data deposited in GigaDB [11]**.**

Table 2. Summary annotation of different genomic elements in the Ukrainian genomes annotated in BGISEQ-500 data[&] from 97 Ukrainian samples

**A. Variants by Location**	**# of unique alleles [a]**	**Total allele #**	**Average /sample**
**Upstream**	2,023,920	6,716,794	69,246
**UTR 5 Prime**	31,026	122,417	1,263
**Exon**	320,979	839,045	8,650
**UTR 3 Prime**	150,302	389,528	4,016
**Downstream**	2,036,111	6,591,978	67,959
**Intergenic**	9,844,120	9,844,120	101,486

**Intron**	9,297,384	42,268,211	435,755
**Motif**	58,164	58,164	600
**B. Functional Variants by Type** £			
**Splice site acceptor**	1,105	3,844	40
**Splice site donor**	969	3,609	38
**Splice site region**	19,436	79,853	824
**Transcription factor binding site (TFBS) ablation**	2,229	2,229	23
**Conservative in-frame indels**	1544	2,475	26
**Gene Fusion**	98	1,482	16
**Disruptive in-frame indels**	978	4,093	43
**Missense**	61,181	169,454	1,747
**Start lost**	116	413	5
**Stop gained**	885	2,442	26
**Stop loss**	95	324	4
**Synonymous**	49,731	146,066	1,506
**Protein folding**	105,436	258,767	2,668

ᘯ Unique alleles represent mutations that were counted only once using the largest transcript, disregarding their frequency in the population
£ Some of the mutations listed in the can be classified in more than one category
& BGISEQ-500 DNBSEQ™ sequencing (BGI Inc., Shenzhen, China)

Collection of the medically relevant variants

Many of the reported variants are already known to be medically related and are listed either in Genome-wide association studies (GWAS) [29] or ClinVar (a NCBI archive of reports of the relationships among human variations and phenotypes with supporting evidence) [30] catalogues (**Table 3**). Our database contains a total of 43,892 benign mutations in medically related genes, but also 189 unique pathogenic or likely pathogenic variants, as well as 20 protective or likely protective alleles as defined in ClinVar [30,31]. Each individual in this study carries 19 pathogenic and 12 protective mutations on average. While least some individuals were homozygous for the pathogenic allele, none of the associated disease phenotypes have been reported, which could be largely attributed to heterozygosity, age-dependent penetrance, expressivity and gene-by-environment interactions [32,33].

As expected, our study shared a lot more variants with the GWAS [29] than with the ClinVar [30] catalogue. While GWAS has recently become the tool of choice to identify genetic variants associated with complex disease and other phenotypes of interest [34], since the amount of genetic variance explained by these variants is low, they are generally not very useful for prediction pathogenic phenotypes [35]. It is also important to note, that not all ClinVar variants carry the same weight of supporting evidence, attributing disease causation to prioritized variants remains an inexact process and some of the reported associations eventually are proven to be spurious [36]. Nevertheless, the importance of the unique set of mutations published here is difficult to overemphasize, as it constitutes the first published set of pathological variants in an understudied population, an important step towards a local catalogue of medically relevant mutations. In addition, as the attention in the genomic community is shifting from monogenic to polygenic traits, many of these may become relevant in the future research and exploration

[37]. A full list of the medically relevant functional markers found in the Ukrainian population and reported in GWAS [29] and ClinVar [30] databases are presented in **Table S4** with alternative allele frequencies and annotations.

Table 3. Medically-relevant variants in the Ukrainian population included in GWAS [29] and ClinVar [30] databases

Source of Annotation	# Unique substitutions [a]	Total allele #	Average /sample
**GWAS catalog**	102551	6,479,953	66804
**ClinVar:** pathogenic (or likely pathogenic)	189	1,830	19
**ClinVar:** benign (or likely benign)	43,892	1,842,668	18997
**ClinVar:** protective (or likely protective)	20	1,209	12

[a] Unique variants represent substitutions that were counted only once, disregarding their frequency in the population

Disease variants with frequencies that differed between the Ukrainians and the neighboring populations are of particular interest to the medical community. It is well established that differences in allele frequencies are a consequence of evolutionary forces acting in populations (such as drift, mutation, migration, nonrandom mating and natural selection), the certain diseases and heritable traits display marked differences in frequency between populations [38]. With this in mind, we created a list of the known disease variants that whose frequencies differ between Ukrainians and other European populations (the combined European sample (EUR) from the 1000Genomes Project (Utah Residents (CEPH) with Northern and Western European Ancestry, Toscani in Italy (TSI), Finnish in Finland (FIN) British in England and Scotland (GBR), Iberian Population in Spain (IBS)[14,39] and French population from HGDP (FRA)[40]) and Russians from HGDP (RUS)[40]. Several examples of these variants are presented in **Table 4**. Among these are variants involved in a number of medical conditions such as hyperglycinuria/iminoglycinuria (*rs35329108; SLC6A19*), efficacy of bisphosphonate response (*rs2297480; FDPS*), autism (rs7794745, *CNTNAP2*), Leber congenital amaurosis (*rs10151259, RPGRIP1*), and breast cancer susceptibility in *BRCA1* and *BRCA2* carriers n (*rs1801320, RAD51*)(**Table 4**).

Table 4. Examples of the functional SNPs with highly differentiating functional markers reported in ClinVar [29], with high differences in the Ukrainian population compared to the neighboring populations in other European populations (the combined sample from Western and Central Europe from 1000Genomes Project (EUR)[14,39] and French population from HGDP (FRA)[40], as well as Russians (RUS) from HGDP [40]. Non-reference allele frequency (NAF) is reported compared to the reference allele in GRCh38. Differences are evaluated by the Fisher Exact Test (FET). All the functional SNPs with significant population frequency differences are listed in **Table S5**.

SNP	Chr	Gene	REF/ alt [β]	Associated medical condition	NAF UKR	NAF EUR	NAF RUS	FET vs. EUR (p-value)	FET vs. RUS (p-value)
rs2297480	*1*	*FDPS*	*T/G*	*Efficacy of the Bisphosphonate response*	*0.13*	*0.23*	*0.27*	*0.038*	*>0.001*

rs35329108	*5*	*SLC6A19*	*G/A*	*Hyperglycinuria. Iminoglycinuria*	*0.32*	*0.26*	*0.17*	*0.049*	*0.004*
rs7794745	*7*	*CNTNAP2*	*A/T*	*Autism*	*0.48*	*0.22*	*0.30*	*0.032*	*0.010*
rs10151259	*14*	*RPGRIP1*	*G/T*	*Leber congenital amaurosis Cone-rod. Dystrophy*	*0.32*	*0.66*	*0.11*	*0.003*	*0.014*
rs1801320	*15*	*RAD51*	*G/C*	*Breast cancer susceptibility in BRCA1 and BRCA2 carriers*	*0.19*	*0.31*	*0.07*	*0.047*	*0.000*

[P] The reference allele is set according to the reference allele in GrCH38.p13 [18].

Of course, not all the medically related variants are currently known, and many remain to be discovered and verified in local populations. This is, to some extent, a consequence underreporting of allelic endemism within understudied populations, particularly in Eastern Europe [10] but also elsewhere [41,42]. By offering public annotations of functional mutations in a population sampled across the territory of Ukraine, our database contributes a number of candidates to direct future research in medical genomics. We chose only the **markers with the highest non-reference allele frequency (NAF) differences** compared to the neighboring populations: the combined population from Europe (EUR; [14]) and Russians from HGDP (RUS)[40] evaluated by the Fisher Exact Test (FET) and listed them **Table 5**.

Table 5. Examples of the functional markers with the highest **non-reference allele frequency (NAF) differences** in the Ukrainian population evaluated by the Fisher Exact Test (**FET**) compared to the frequencies in the neighboring populations: the combined population from Europe (**EUR**; [14]) and Russians from HGDP (RUS) [40].

**SNP**	**Chr**	**Gene**	**Ref/ Alt**	**Function**	**NAF UKR**	**NAF EUR**	**NAF RUS**	**FET vs. CEU (p-value)**	**FET vs. RUS (p-value)**
rs72625995	*17*	*POM121L8P*	*C/T*	*exonic , nonsynonymous SNV*	*0.03*	*0.62*	*0.75*	*2.50E-07*	*1.86E-06*
rs9930886	*16*	*PTPRN2*	*A/G*	*exonic, synonymous SNV*	*0.01*	*0.33*	*0.35*	*2.56E-07*	*2.19E-06*
rs4779816	*15*	*ZBTB9; BAK1*	*A/G*	*exonic , nonsynonymous SNV*	*0.41*	*0.80*	*0.83*	*3.29E-06*	*7.82E-07*
rs58580222	*12*	*ABCC1*	*G/A*	*exonic, synonymous SNV*	*0.03*	*0.13*	*0.26*	*3.06E-04*	*1.17E-02*
rs80150964	*11*	*SMIM40; KIFC1*	*T/C*	*exonic , non-synonymous SNV*	*0.03*	*0.23*	*0.19*	*4.95E-04*	*1.96E-06*

Population structure and ancestry informative markers

We performed several population analyses, but only to demonstrate the uniqueness and usefulness of this new dataset. Our results indicate that genetic diversity of the Ukrainian population is uniquely shaped by the evolutionary and demographic forces and cannot be ignored in the future genetic studies. However, we do not evaluate any historical hypotheses on the timing of origins, founding, migration, and admixture of this population and use only the naive approaches, choosing models based on the statistical models.

To demonstrate the extent to which our dataset contributes to the genetic map of Europe, we explored genetic relationships between Ukrainian individuals within our sample and evaluated genetic differences between this population and its immediate neighbors on the European continent for which population data of full genome sequences was publicly available. A Principal Component Analysis (PCA) of the merged dataset of 654 samples included European populations from the 1000Genomes Project (Utah Residents (CEU) with Northern and Western European Ancestry, Toscani in Italy (TSI), Finnish in Finland (FIN) British in England and Scotland (GBR), Iberian Population in Spain (IBS)) [14,39]), and French and Russians (RUS) populations from the HGDP [40] as well as the relevant high-coverage human genomes from the Estonian Biocentre Human Genome Diversity Panel (EGDP: Croatians (CRO), Estonians (EST), Germans (GER), Moldovans (MOL), Polish (POL), and Ukrainians (UKR)[43], and Simmons Genome Diversity project (Czechs (CZ), Estonians (EST), French (FRA), Greeks (GRE), and Polish (POL) [43] (**Figure 2**). The latter paper also identifies "Cossacks" as a separate self-identified ethnic group within Russians (Cossacks (RUS) or Ukrainians (Cossacks (UKR)) [44] (**Supplementary File 3**).

Ukrainian genomes from this (**black dots**) as well as other studies (**black circles**) [43,44] form a single cluster positioned between the Northern (Russians **(green circles),** Estonians (**purple circles**) on one side, and Western European populations on the other (**blue shapes are:** CEU, French, British and Germans, **Figure 2**). There was a significant overlap with the other Central and Eastern European populations, such as Czechs (**red dots**), Polish (**red crosses**), and the people from the Balkans (Croats, Greeks and Moldovans; **light orange shapes**). This is not surprising, in addition to the close geographic distance between these populations, this may also reflect the insufficient representation of samples from the surrounding populations (see data in GigaDB [11]). Similarly, the admixture analysis demonstrates distinctiveness of our dataset, but also demonstrates unique combinations of genetic components that may have shaped this population (**Figure 3** and **Figure S3**).

Addition of the new genomic data will most likely add to the resolution of the genetic map of this region and further reveal differences between the populations of Eastern and Central Europe. Meanwhile, our dataset showed a limited amount of inbreeding (**Figure S4**) and contains information for future population studies. A list of all the variants with significant difference in frequencies between Ukrainians and other European populations are listed in **Table S6**. This database can be a starting point for association studies, as ancestry informative markers (AIMs)[45], and to be used for mapping disease alleles by admixture disequilibrium [46,47].

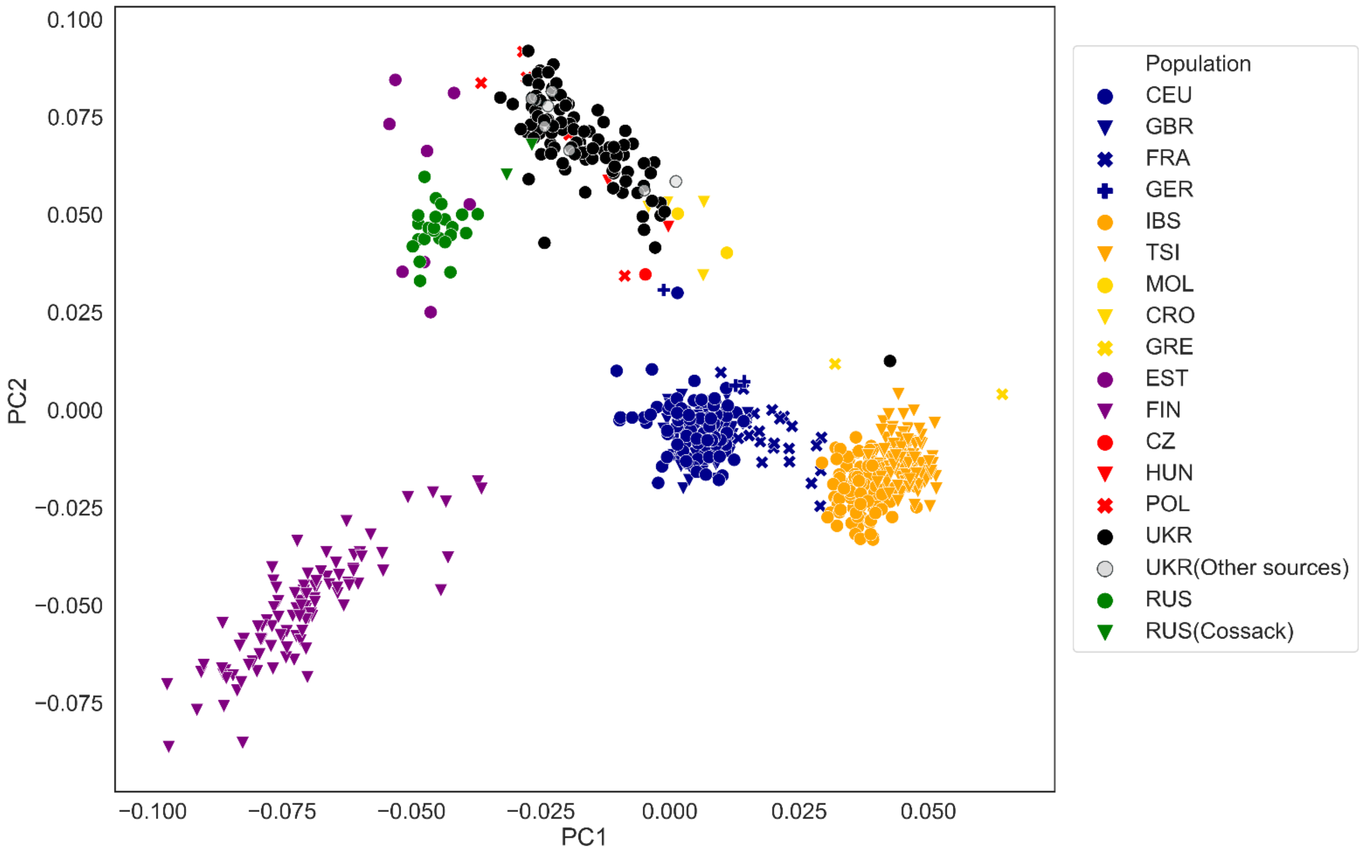

Figure 2. The Principal Component (PC) analysis of genetic merged dataset, containing European populations. Colors reflect prior population assignments from the European samples from the 1000Genomes Project (Utah Residents (CEPH) with Northern and Western European Ancestry, Toscani in Italy (TSI), Finnish in Finland (FIN), British in England and Scotland (GBR), Iberian Population in Spain (IBS)[14,39], French (FRA) and Russians (RUS) from HGDP (RUS) [40] as well as the relevant high-coverage human genomes Croatian (CRO), Czech (CZ), Estonian (EST), German (GER), Greek (GRE), Hungarian (HUN), Moldovan (MOL), Polish (POL), Russian Cossack (RUS) and Ukrainian (UKR) from the Estonian Biocentre Human Genome Diversity Panel (EGDP) [43] as well as Simmons Genome Diversity project [44]. The analysis was performed with *Eigensoft* [48].

To provide a more extended view of the genetic components contributing to the Ukrainian population, we used the population structure plots using the ADMIXTURE package [49]. This allowed us to construct a preliminary picture of putative ancestry contributions and population admixture. In order to identify the optimal K, we implied the 10-fold cross-validation function in range from K=2 to 6. The results with the optimal K=3 shown in **Figure 3** illustrate similarity and the difference of Ukrainian population compared to the other populations in Central and Eastern Europe (**Figure 3, second row**). While the higher values of K (K=3-8; **Figure S3**) show an increasing number of clusters, they also show an increasing amount of error in the cross-validation function. This analysis already shows the potential of the current database in helping to resolve population structure in Eastern Europe, but additional genome wide data from neighboring populations would be very helpful to refine the picture in this geographical region. Unfortunately, valuable genome wide data collected from three populations in Russia has been retracted from public databases after publication [13].

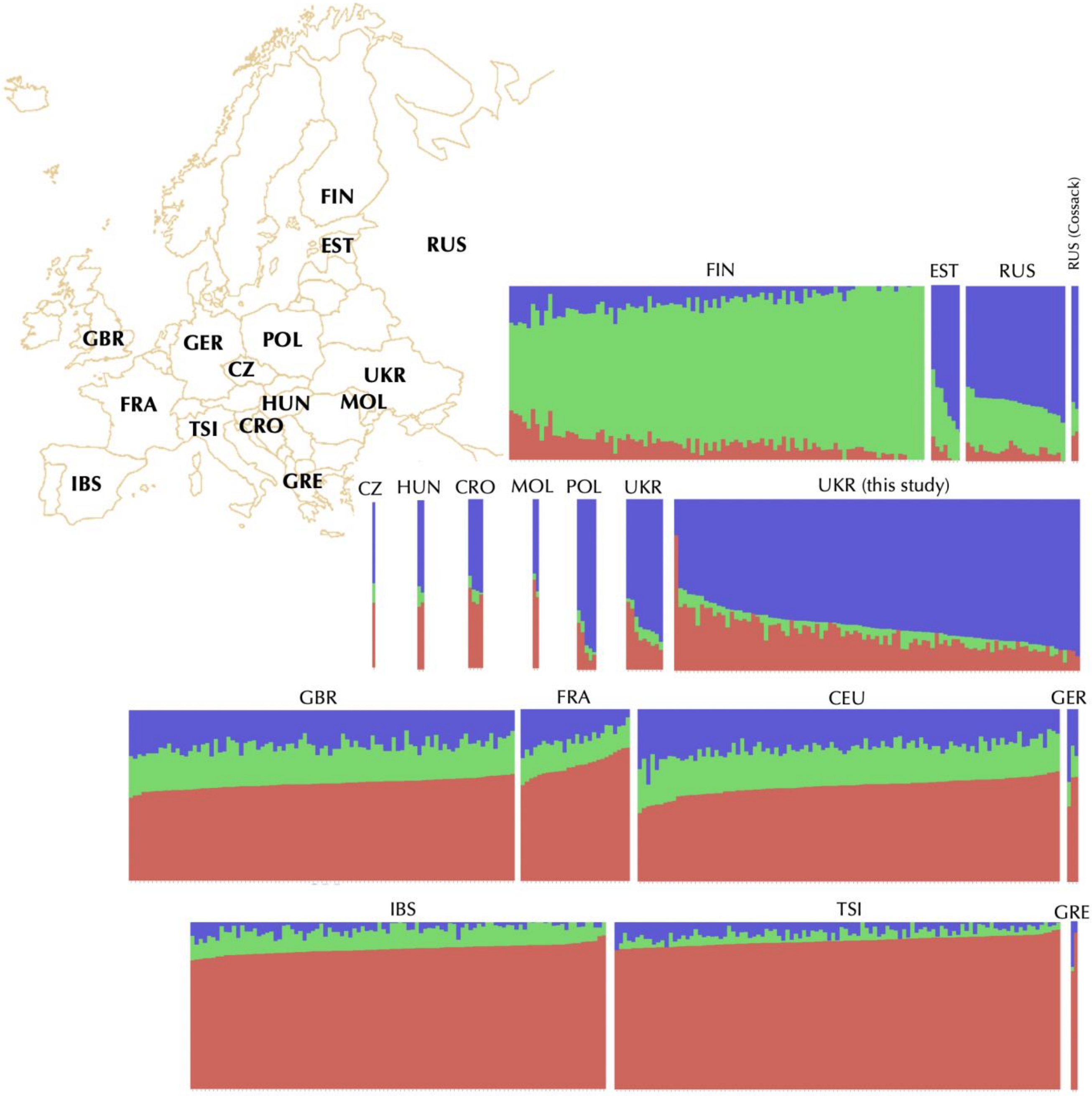

Figure 3. Genetic structure of Ukrainian population in comparison to other European populations. Structure plot constructed ADMIXTURE package [49] at K=3 illustrates similarity and differences between genomes from this study as well as samples from the 1000Genomes Project (Utah Residents (CEU) with Northern and Western European Ancestry, Toscani in Italy (TSI), Finnish in Finland (FIN), British in England and Scotland (GBR), and Iberian Population in Spain (IBS)[14,39], French(FRA) and Russians (RUS) from HGDP [40], as well as the relevant high-coverage human genomes Croatian (CRO), Czech (CZ), Estonian (EST), German (GER), Greek (GRE), Hungarian (HUN), Moldovan (MOL), Polish (POL), Russian Cossack (RUS) and Ukrainian (UKR) from the Estonian Biocentre Human Genome Diversity Panel (EGDP) [43] as well as Simmons Genome Diversity project [44]. For identification of the optimal K parameter, we evaluated a range from 2 to 8, with K=3 resulting in the lowest error. Plots with K=3 to K=6 are presented in **Figure S3**.

Despite of the fact that all of the samples were collected from self-identified ethnic Ukrainians, there were two notable outliers. Sample EG600048 that clustered with the Southern Europeans (Iberia and Italian populations), and EG6000xx clustered with the Western Europeans (CEU, French, British and Germans) (**Figure 2**). This illustrates an important point that while ignoring the unique composition of this population will result in ascertainment bias in biomedical studies. Genetics is not a reliable determinant of ethnicity but can be used to evaluate individual contributions of ancestry. In anticipating the future ancestry studies, we contribute the full list of candidates for Ancestry Informative Markers differentiating Ukrainians with their neighboring populations in Europe (**Table S6**).

People of Ukraine carry many previously known and several novel genetic variants with clinical and functional importance that in many cases show allele frequencies different from neighboring populations in the rest of Europe, including Poland to the West, Romania to the South, the Baltics to the north and Russia to the northeast. While several large genome projects already exists contributing to the understanding of the global genetic variation, many of the rare and endemic alleles that have not been yet identified by the international databases such as the 1,000 Genomes project, and currently not available in standard genotyping panels for association testing for human diseases, and glaring white spots still exists on the genetic maps in local populations of Eastern Europe [10]. We fully expect that the future sampling and sequencing will continue to improve and complete the detailed picture of genomic diversity in people across the country and contribute to the further development of genetic approaches in biomedical research and applications.

Methods

a) Sampling strategy

The collection and consent procedure was approved as part of the "*Genome Diversity in Ukraine* " project by the Institutional Review Board (IRB) of Uzhhorod National University in Uzhhorod, Ukraine (Protocol #1 from 09/18/2018, Supplementary File 1). We employed doctors and medical professionals from different regions of Ukraine to oversee collection of blood samples at hospitals. Healthy (non-hospitalized) volunteers were contacted through advertisements and invited for personal interviews at outpatient offices. During the visit the volunteers were familiarized with the study and the collection procedure and gave full consent to participate and have their genotypic and phenotypic data to be freely and publicly available. During each interview, the volunteer participants also completed a questionnaire indicating self-reported region of origin, place of birth of both grandparents (if remembered), sex and several phenotypical features, such as daily history of disease (**Supplementary File 3**). The hard copies of the consents and personal interviews remain sealed and stored at the Biology Department of Uzhhorod National University. After the conclusion of the interview and sample collection, all personal identifiers were removed from the vials containing blood samples, except for an alphanumeric identifier and a barcode. All the subsequent analysis and publication was done in a blind design where neither the participants nor the researchers could identify the person who donated the sample.

At the conclusion of the interview a whole blood sample was collected from a vein into two a 5 ml EDTA tubes by a certified nurse or a phlebotomist, assigned a barcode number, and shipped by courier on dry ice to a biomedical laboratory certified to handle blood samples in Uzhhorod, Ukraine (**Astra Dia Inc**.) for DNA extraction immediately on arrival. The excess of the blood and DNA from samples remaining after the genetic analysis is stored frozen at the biobank of the Biology Department, Uzhhorod National University, Ukraine. As a result, blood samples were collected from a total 113 individuals.

b) DNA extraction

Immediately upon arrival to the laboratory, DNA isolation from 200 uL of blood was carried out with the *innuPREP DNA Blood Minikit* (Analitik Gena, Germany). High molecular weight genomic DNA was lightly fragmented by vortexing. The initial DNA concentration was measured with the Implen C40 Nanophotometer (München, Germany), and quality was verified visually on a 2% agarose gel. The 97 successfully extracted DNA samples were normalized to 20-30 ng/µl concentration for downstream application. After the extraction the samples were re-coded and sent to NIH for genotyping procedure, from where the aliquots were further shipped to BGI facility (BGI Shenzhen, CHINA) or to Psomagen Inc. (Gaithersburg, MD, USA) for the whole genome sequencing (WGS). The remaining ~2 ml was frozen for future use.

c) Sequencing and Genotyping

All the 97 individuals in this study were sequenced with BGISEQ-500 and 88 individuals were cross validated by genotyping using Illumina Global Screening Array. The record of which individual samples have been cross validated by both technologies is presented in **Table S2**. In addition, a single sample (EG600036) was also sequenced on Illumina NovaSeq 6000 S4 (~60x coverage).

Sequencing with BGISEQ-500

All 97 DNA samples were sequenced on BGISEQ-500 (BGI Shenzhen, CHINA). Upon the receipt at the BGI facility, and prior to sequencing, samples were checked again for quality. Concentration was once more detected by fluorometer or Microplate Reader (e.g. Qubit Fluorometer, Invitrogen). Sample integrity and purity were detected by Agarose Gel Electrophoresis (Concentration of Agarose Gel: 1% Voltage:150 V, Electrophoresis Time: 40 min). 1μg genomic DNA was aliquoted and fragmented by Covaris. The fragmented genomic DNA was selected by Agencourt AMPure XP-Medium kit to an average size of 200-400bp. Fragments were end repaired and then 3' adenylated. Adaptors were ligated to the ends of these 3' adenylated fragments. PCR products were purified by the Agencourt AMPure XP-Medium kit. The double stranded PCR products were heat denatured and circularized by the splint oligo sequence. The single strand circle DNA (ssCir DNA) was formatted as the final library. The qualified libraries were sequenced by BGISEQ-500: ssCir DNA molecule formed a DNA nanoball (DNB) containing more than 300 copies through a rolling-cycle replication. The DNBs were loaded into the patterned nanoarray by using high density DNA nanochip technology. Finally, pair- end 100 bp reads were obtained by combinatorial Probe-Anchor Synthesis (cPAS). Raw reads were filtered removing adaptor sequences, contamination and low-quality reads. Sequencing of all the 97 full genome samples submitted for sequencing at BGI was successful.

Short Read Sequencing with Illumina NovaSeek6000

One individual was resequenced by Illumina NovaSeq6000 S4 at Psomagen Inc. (Gaithersburg, MD, USA). Library was prepared using TruSeq DNA PCR Free 350bp protocol by Illumina. The library was sequenced at approximately 64X depth, producing 150bp-long reads, resulting in 241.7G bp of data.

Genotyping with the Illumina Infinium Global Screening Array

We attempted to genotype all 97 of the collected samples using the Illumina Infinium Global Screening BeadChip Array-24 v1.0 (GSAMD-24v1-0) for 700,078 loci at the NCI's DCEG (Bethesda, MD; *https://grcf.jhmi.edu/wp-content/uploads/2017/12/infinium-commercial-gsa-data-sheet-370-2016-016.pdf*). Data was analyzed by using the standard Illumina microarray data analysis workflow. During QC, samples were filtered for contamination, completion rate, and relatedness. As part of QC, we performed ancestry assessment using SNPweights software [45] with a reference panel consisting of 3 populations (European, West African, and East Asian). All samples were attributed to the European ancestry group. After OC and sample exclusion, 87 (86 samples and 1 QC) samples with 689,918 loci and completion rate of 99.9 were retained for further analysis.

d). Variant Calling

Variant Calling of the BGISEQ-500 data

The sequencing data produced using the BGISEQ-500 platform for 97 samples were analyzed using the Sention tools (Sentieon Inc, San Jose, CA, USA) high-performance implementation of the BWA/GATK best practices pipeline on servers hosted by the Cornell University Biotechnology Resource Center. Reads were aligned to the GRCh38 human reference genome using BWA-MEM (Version: 0.7.16a-r1181), and mapped reads were prepared for variant calling using Genome Analysis Toolkit (GATK) v3.8-1-0-

gf15c1c3ef by Broad), including marking duplicates (*picard MarkDuplicates*, Version 2.12.1), indel realignment (*GATK RealignerTargetCreator, IndelRealigner, Version 3.7-0*), and base quality score recalibration (*GATK BaseRecalibrator, PrintReads, Version 3.7-0*). SNP and Indel discovery were performed for each individual using GATK HaplotypeCaller and merged into a single pVCF using *bcftools*. Sample EG600036 was also run without joint calling which was used when calculating concordance between the Illumina and BGISeq variant callsets. estimated with *TiTvtools* and visualized by *plotTiTv* [19].

Repetitive variant calling

Mobile element discovery was performed using MELT (Version 2.2.0) [50] and structural variant discovery using *lumpy-sv* with *Smoove* (Version: 0.2.5)[16]. Short tandem repeats were called using *GangSTR* (Version: 2.4.2) [51] and nuclear mitochondrial DNA using *dinumt* [52].

e) Data validation and quality control

Variant files were compared for consistency across the three different platforms: BGISEQ-500 sequencing, Illumina genotyping, and Illumina NovaSeq6000 S4 sequencing. Illumina genotyping was performed on 86 of the 97 samples previously sequenced with BGISEQ-500. Additionally, one sample (EG600036) was also sequenced with Illumina NovaSeq6000 S4. The variant detection programs were re-run without joint calling for the BGISEQ-500 sequencing for sample EG600036 for comparison with the single Illumina sequenced sample. In this sample, the SNPs derived from the WGS platforms were compared to those identified using the Illumina SNP array both for matching position and matching genotype. Structural variants and mobile element insertions were compared between the WGS platforms in EG600036. Variants were considered the same if they had 95% reciprocal overlap. Overall, we found Illumina identified a higher number of larger variants than BGISEQ-500. This could potentially be due to its higher coverage (~60X) compared to BGISEQ-500 (~30X). However, as both have high coverage, we may see diminishing returns for coverage over 30X. An alternative explanation is that the variant identification tools have been built to detect variation from Illumina sequencing data and therefore, may not be able detect variants BGISEQ-500 as accurately.

f) Annotation

Sequence variant files were annotated using *ANNOVAR* (RRID:SCR_012821) [53] and *SNPEff* (RRID:SCR_005191)[54] software using GRCh38 reference databases. The following databases were used for the For ANNOVAR annotations: RefSeq Gene, 1000 genomes superpopulation, dbSNP150 with allelic splitting and left-normalization. For annotation of the medically related and functional variants we used ClinVar version 20200316 [30], InterVar gnomeAd ver 3.0 [12], and *dbnsfp ver. 35c* [55]. For S*NPEff*, the default GRCh38 annotation database [56] was complemented with ClinVar (RRID:SCR_006169) [30] and GWAS catalog [29] database annotation using *snpSift* tool (RRID:SCR_015624) [57].

g) Population analysis

Principal Component analysis (PCA)

For principal component analysis, we used WGS variants of our samples and merged them with samples

from neighboring countries available from the European samples from the 1000Genomes Project (Utah Residents (CEPH) with Northern and Western European Ancestry, Toscani in Italy (TSI), Finnish in Finland (FIN), British in England and Scotland (GBR), Iberian Population in Spain (IBS)[14,39]) and French (FRA) and Russians (RUS) from HGDP [40] as well as the relevant high-coverage human genomes Croatian (CRO), Czech (CZ), Estonian (EST), German (GER), Greek (GRE), Hungarian (HUN), Moldovan (MOL), Polish (POL), Russian Cossack (RUS) and Ukrainian (UKR) from the Estonian Biocentre Human Genome Diversity Panel (EGDP) [43], and the Simmons Genome Diversity project [44]. The analysis was performed with *Eigensoft* (RRID:SCR_004965) [48].

To produce a meaningful number of alleles to analyze, the resulting dataset was filtered by genotyping rate (1) and pruned for variants in LD by excluding those with high pairwise correlation within a moving window(--*indep-pairwise* 50 10 0.5). This resulted in 677 samples with 208,945 variants. We used *EIGENSOFT* [48] to calculate the eigenvectors, of which, PC1 and PC2 were visualized using Python programming language, with *pandas, matplotlib and seaborn* libraries [58]. Two extreme outlier samples (EG600056, and EG600052) were left out from the visible range of the PCA plot as they clustered with each other far away from any known European group.

Model-based population structure analysis

For the naive (model-based) structure analysis, we used the same dataset described in the Principal Component Analysis (above). The analysis was performed using *ADMIXTURE* software (RRID:SCR_001263) [49].   For identification of the optimal K parameter, we used the 10-fold cross-validation function of *ADMIXTURE* in range from 2 to 6, with K=3 resulting in the lowest error, deeming it optimal. The results were visualized using Python programming language, with *pandas*, *matplotlib* and *seaborn* libraries [58,59] to construct a population structure plot using samples from the 1000Genomes Project (Utah Residents (CEU) with Northern and Western European Ancestry, Toscani in Italy (TSI), Finnish in Finland (FIN), British in England and Scotland (GBR), and Iberian Population in Spain (IBS), French population from HGDP(FRA)); [14,39]) and Russians (RUS) from HGDP [40] as well as the relevant high-coverage human genomes from the Estonian Biocentre Human Genome Diversity Panel (EGDP) [43], and Simmons Genome Diversity project [44]. The resulting plot with K=3 is presented in **Figure 3**, and plots with K=4 to K=8 are in the **Figure S3**.

Inbreeding estimates

We estimated inbreeding coefficients for all the genotype samples in the same dataset. For this analysis the samples were pruned for genotyping rate (>0.9) and linkage disequilibrium by excluding those with high pairwise correlation within a moving window (plink parameter--*indep-pairwise* 50 10 0.1). Using the resulting dataset containing the remaining 117,641 loci from 84 samples, we performed several inbreeding estimates: (a) method-of-moments F-coefficient estimates, (b) variance-standardized relationship minus 1 estimates, and (c) F-estimates based on correlation between uniting gametes [60]. All the resulting values are presented in **Table S7**, and the estimates for the of method method-of-moments F-coefficient estimates are visualized in a histogram (**Figure S4**).

Re-use potential

Since the publication of the first human genome [61,62] , and the first surveys of worldwide variation

such as the 1,000 Genomes project [14,39], the efforts have been directed to expand outwards by expanding the exploration of the human diversity across the world, and filling out more and more "white spots" of genome variation [13,44], as well as inward, to fill the remaining white spots in the human genome itself: to map the remaining gaps in the chromosome assembly and identify new structural and functional variation [63] and to map the three dimensional structure of the human genome [64]. The new data presents a valuable addition to the former and represents the first exploration of the genome landscape in the important component of European genomic diversity.

Genome diversity of Ukraine is an important puzzle to help modern genome studies of population history of Europe. The country is positioned in the crossroad of the early migration of modern humans and the westward expansion of the Indo-Europeans, and represents an aftermath of centuries of migration, admixture, demographic and selective processes. As wave after wave of great human migrations moved across this land for millennia, they were followed by exchange of cultural knowledge and technology along the great trade routes that transect this territory until this day.

The justifications for collecting, sequencing and analyzing populations from this part of Europe has been outlined earlier [10,65], and the new database is a step into that direction. Given its unique history, the genome diversity data from Ukraine will contribute a wealth of new information bringing forth different risk and/or protective alleles that do not exist nor associate with disease, elsewhere in the world. This project identified 13M variants in Ukrainians of which 478 K were novel genomic SNPs currently missing from the global surveys of genomic diversity [12,13]. We also report almost 1M (909,991) complex indels, regions of simultaneous deletions and insertions of DNA fragments of different sizes which lead to net a change in length, with only 713,858 previously reported in gnomAD [12] (**Table 1**). The newly discovered local variants can be used to augment the current genotyping arrays and used to screen individuals with genetic disorders in genome wide association studies (GWAS), in clinical trials, and in genome assessment of proliferating cancer cells.

The current project is built upon the open release/access philosophy. The data has been released and can be used to search from population ancestry markers and well as the medically related variants in the subsequent studies. The public nature of the data deposited on the specially created web resource located at Uzhhorod National University, will ensure that the biomedical researchers in the country will receive access to a useful information resource for future projects in genomics, bioinformatics and personalized medicine. Engaging local Ukrainian scientists in this collaborative international project like building the foundation for the future studies and ensuring their participation in the worldwide research community.

Availability of the Supporting Data

The raw reads are available at the SRA (Project PRJNA661978, SUB7904361). All other datasets mentioned in this project are available in *GigaScience* GigaDB [11].

List of Supplementary Tables (available in GigaDB)

Table S1. Sequencing summaries of output from **BGISEQ-500** and Illumina NovaSeq6000 S4. Full sequencing statistics for individual samples in **Table S1.2**

Table S2. Filtering summary of the data obtained from 97 whole genomes sequenced with BGISEQ-500.

Table S3. The full list of high impact functional variants (including frameshift, start lost/stop lost or gained, transcript ablations and splice alterations) that had an allele count of two or more with their predicted function, number of gene transcripts of the gene affected, and frequencies.

Table S4. List of the medically relevant functional markers found in the Ukrainian population and reported in **A. GWAS catalog** [29] and B. **ClinVar** [30] databases. Allele frequency is reported compared to the reference allele in GRCh38.

Table S5. Complete list of the highly differentiating markers, reported in ClinVar [30], with high differences in the Ukrainian population compared to the neighboring populations in other European populations (the combined sample from Western and Central Europe from 1000Genomes Project with French samples from HGDP (EUR)[14,39,40] and Russians (RUS) from HGDP [40]. Non-reference allele frequency (NAF) is reported compared to the reference allele in GRCh38. Differences are evaluated by the Fisher Exact Test (FET).

Table S6. A list of markers with the highest non-reference allele frequency (NAF) differences in the Ukrainian population evaluated by the Fisher Exact Test (FET) compared to the frequencies in the neighboring populations: the combined population from Europe (EUR) [14] and Russians (RUS) from HGDP [40]. This database contains candidate ancestry informative markers (or AIMs)[44], that can be used for mapping disease alleles by admixture disequilibrium [46,47].

Table S7. Inbreeding estimates in a dataset of 117,641 loci from 84 samples: (a) method-of-moments F-coefficient estimates, (b) variance-standardized relationship minus 1 estimates, and (c) F-estimates based on correlation between uniting gametes [60].

List of Supplementary Files (available in GigaDB)

Supplementary File 1. IRB approval of the study "Genomic Diversity of Ukraine's Population" (*in Ukrainian*). Supplementary File 1. The IRB Approval.jpg

Supplementary File 2. Genomic Diversity of Ukraine's Population Project: Protocol description, questionnaire, and informed consent to participate and publish (*in Ukrainian with English Translation*). Supplementary File 2. The Informed Consent

Supplementary File 3. The list of the samples in this study, their characteristics and geographical locations, and sources of genomic data for each (BGISEQ-500 sequencing (BGI Inc., Shenzhen, China), Illumina Global Screening Array genotyping, and Illumina NovaSeq sequencing array (Illumina Inc., San Diego, USA). Supplementary File 3. The List of Samples

Supplementary File 4. List of the samples from different studies used in the current population analysis. Supplementary File 5. Sample Sources

Supplementary Figures

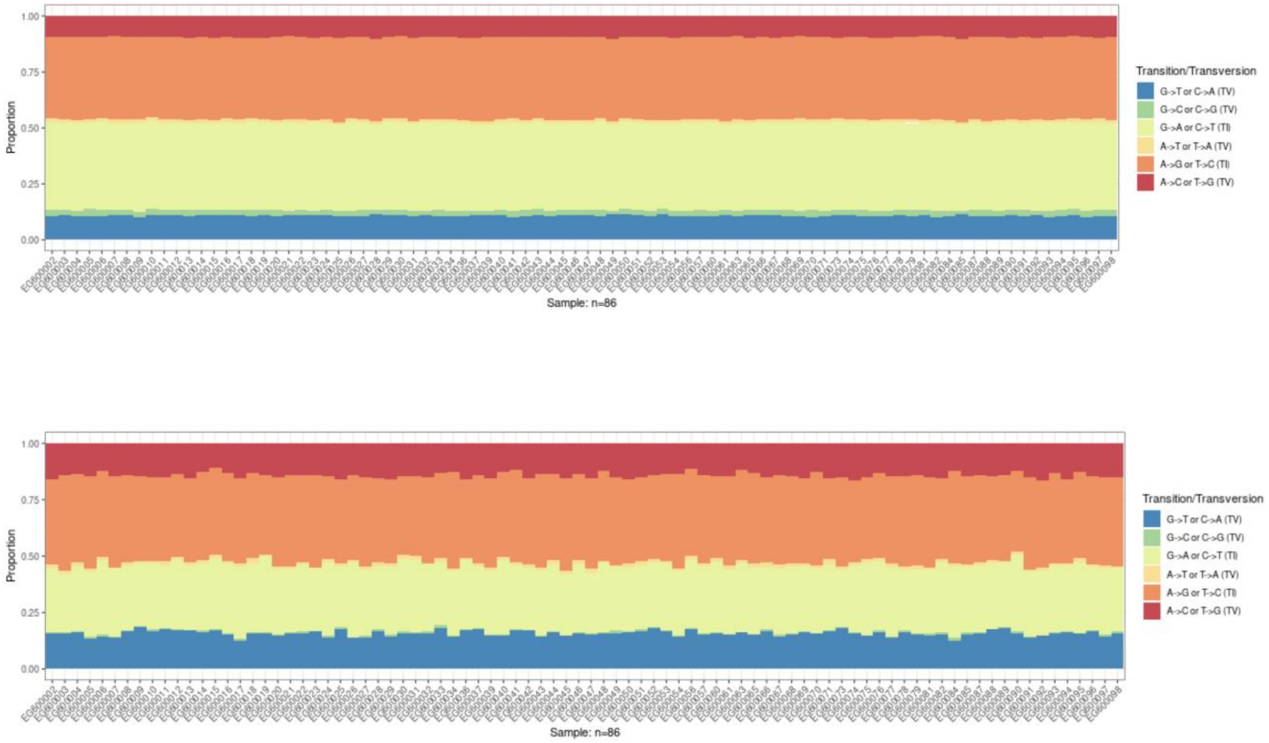

Figure S1. Transition/Transversion ratio (or TITV ratio) for the novel SNPs (estimated with *TiTvtools* [18] and visualized by *plotTiTv*) (top) for the SNPs where Illumina SNP array identified more alternate haplotypes than BGI (top right triangle in Figure 1C) and (bottom) for the SNPs where BGISeq identified more alternate haplotypes than Illumina SNP Array (bottom left triangle on Figure 1C table).

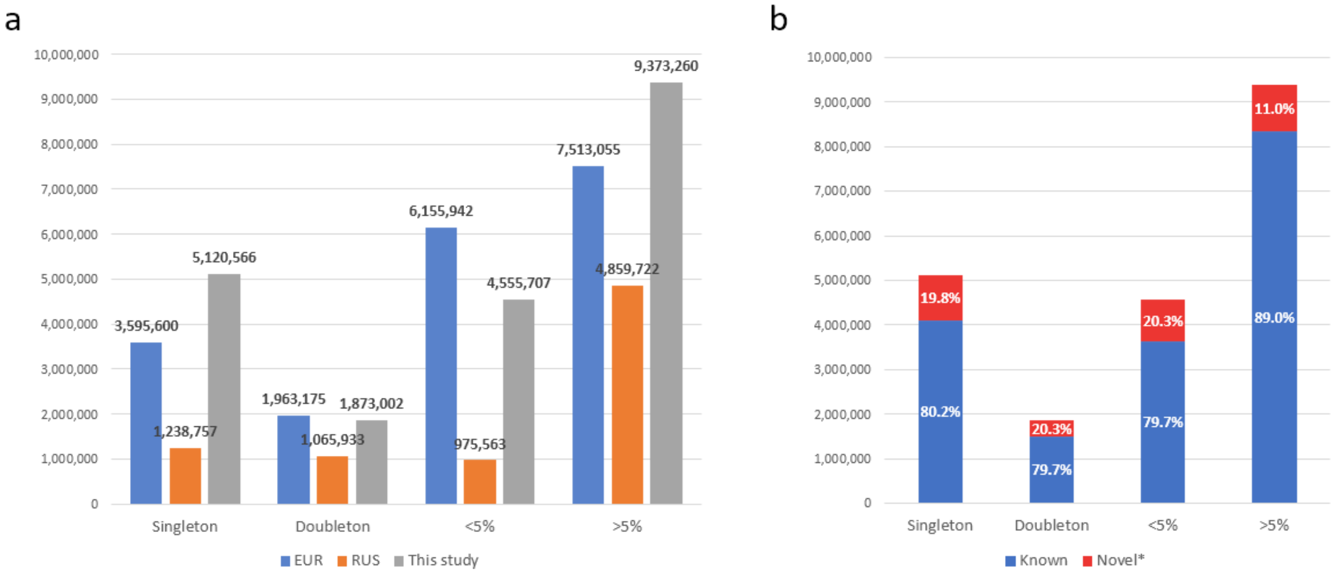

Figure S2. A. Frequencies of various classes of SNPs in the Ukrainian genome variation database. Definitions are as follows: Singleton (passed the GATK QC once), Doubleton, Rare (3-10 counts roughly equivalent to 1%< x < 5%) and Common (>5%) to make it closer to the 1KGP definitions. B. Percent novel mutations in various classes of SNPs.

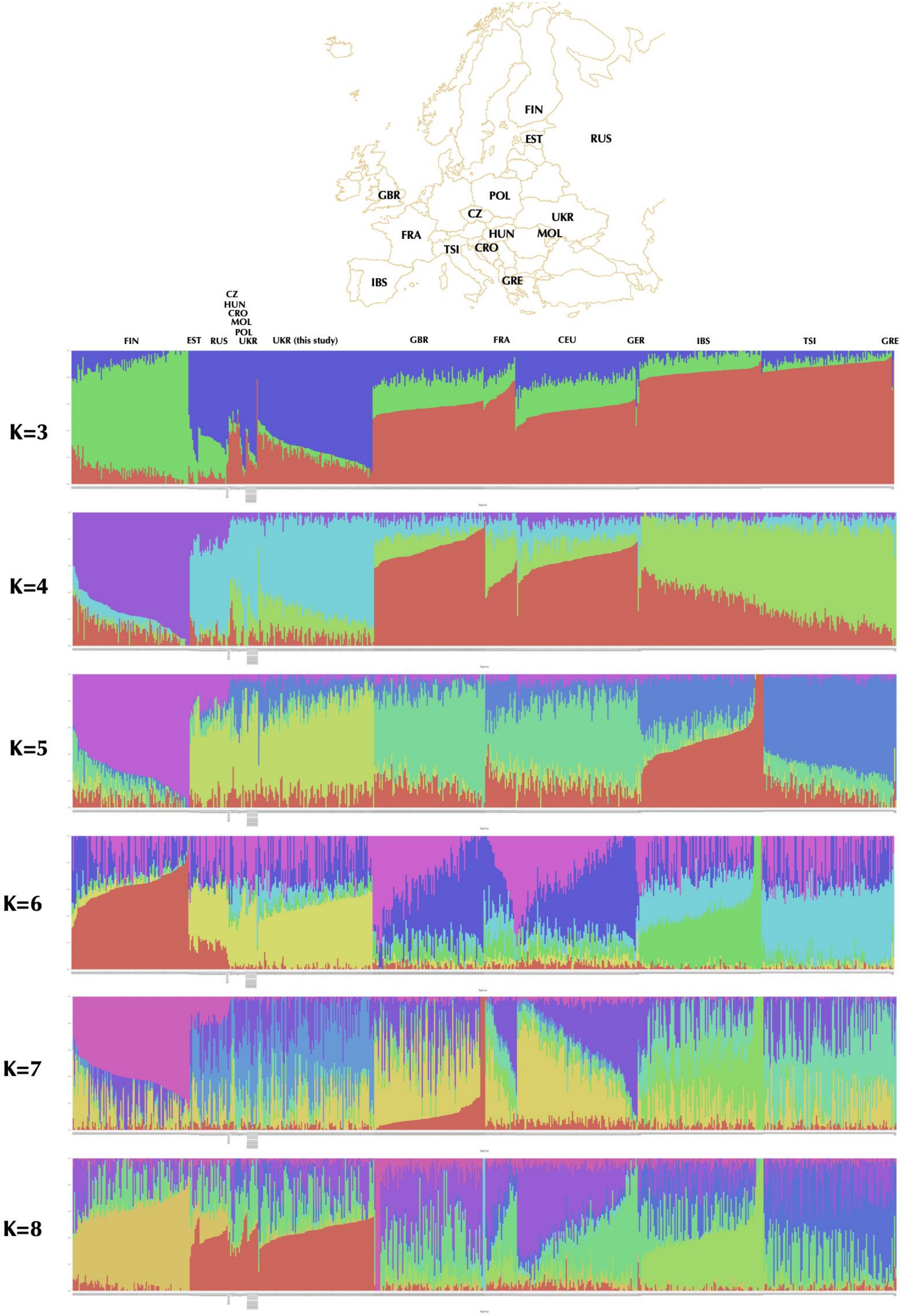
FIN
EST
RUS
GBR
POL
CZ
UKR
FRA
HUN
MOL
TSI
CRO
IBS
GRE
CZ
HUN
CRO
MOL
POL
FIN
EST
RUS
UKR
UKR (this study)
GBR
FRA
CEU
GER
IBS
TSI
GRE
K=3
K=4
K=5
K=6
K=7
K=8

Figure S3. Genetic structure of Ukrainian population in comparison to other European populations. For identification of the optimal K parameter, we used the 10-fold cross-validation function of *ADMIXTURE* in range from 2 to 8, with K=3 resulting in the lowest error [49]. This analysis included genomes from this study as well as samples from the 1000Genomes Project (Utah Residents (CEU) with Northern and Western European Ancestry, Toscani in Italy (TSI), Finnish in Finland (FIN), British in England and Scotland (GBR), and Iberian Population in Spain (IBS)[14,39], French(FRA) and Russians (RUS) from HGDP [39], as well as the relevant high-coverage human genomes Croatian (CRO), Czech (CZ), Estonian (EST), German (GER), Greek (GRE), Hungarian (HUN), Moldovan (MOL), Polish (POL), Russian Cossack (RUS) and Ukrainian (UKR) from the Estonian Biocentre Human Genome Diversity Panel (EGDP) [43] as well as Simmons Genome Diversity project [44].

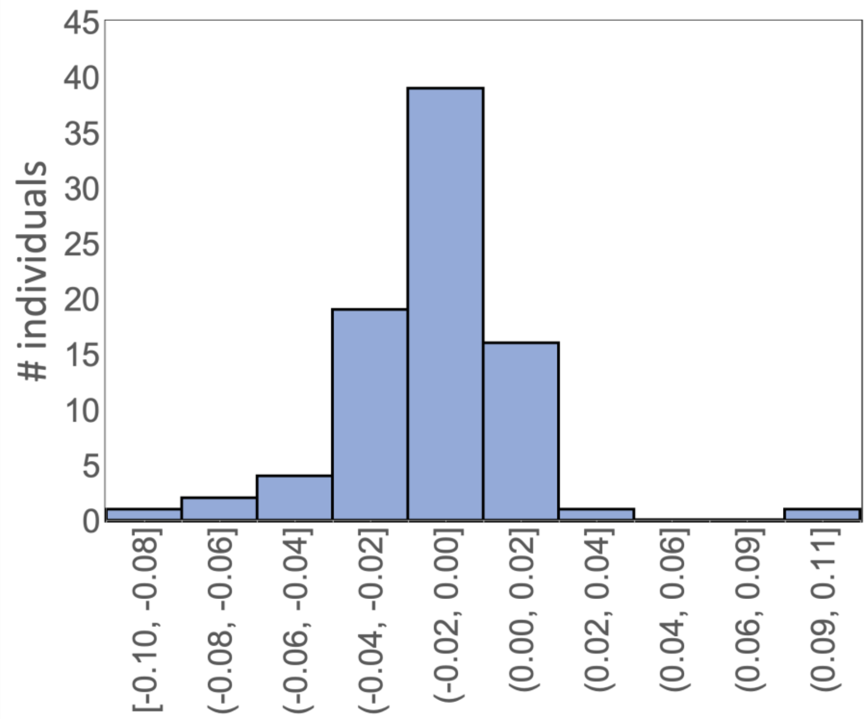

Figure S4. Distribution of inbreeding coefficients in the Ukrainian sample. The individual values corresponding to the samples are presented in **Table S7**

Supplementary Tables

Table S1. Sequencing summary of output from BGISEQ-500 and Illumina NovaSeq6000 S4.

	**BGISEQ-500** [ƨ]	**Illumina NovaSeq6000 S4** [¥]
**Samples sequenced**	97	1
**Read length (bp)**	100	150
**Reads above Q20 (>99% quality score)**	97.85%	96.91 %
**Total Reads**	99,638,538,182	1,600,898,738
**Average reads/sample**	1,027,201,425	1,600,898,738
**Average GC content**	42.05%	41.07

[ƨ] Sequencing of 97 samples were attempted on BGISEQ-500 sequencer at BGI sequencing facility (BGI Shenzhen, CHINA), and all 97 were successful.

[¥] One sample (EG600036) was sent for Illumina NovaSeq6000 S4 sequencing at Psomagen Inc. (Gaithersburg, MD, USA). In addition, 96 samples were genotyped using Illumina Global Screening Array (Illumina Inc., San Diego, USA), and 87 were successful (86 individual samples and 1 internal QC) remained after filtering.

Table S2. Filtering summary of the data obtained from 97 whole genomes sequenced with BGISEQ-500.

Sequencing results	All samples		
	Total Unique SNPs #	Filtered Count	% Filtered[₴]
**Variation**			
**SNPs**	14,738,063	1,727,084	11.7
**Bi-allelic**	14,254,070	1,586,787	11.1
**Multi-allelic**	483,993	140,297	29.0
**Small Indels** [¥]	2,808,384	80,780	2.9
**Deletions**	1,864,698	57,959	3.1
**Insertions**	1,488,408	42,421	2.9
**Structural Variants** [$]			
**Large Deletions**	685,56	52,478	76.5
**Large Duplications**	3,374	52,478	45.3
**Inversions**	430	93	21.6
**Mobile Element Insertions**			
**Alu**	7550	1790	23.7
**L1**	3123	2672	85.6
**SVA**	222	122	55.0
**NUMT**	1169	455	38.9

Declarations

List of abbreviations

UAU: Ukrainians from Ukraine; MEI: Mobile Element Insertions; NUMT: nuclear mitochondrial DNA; SVA: non-autonomous retroelements; INV: inversions; DUP: Duplications; ALU: Alu elements; gnomAD: Genome Aggregation Database; 1KG: 1000Genomes Project; TFBS: transcription factor binding site ablation; GWAS: Genome-wide association studies; PCA: Principal Component Analysis; AIMs: ancestry informative markers; PC: Principal Component; WGS: whole genome sequencing.

Consent for publication

The collection procedure was approved as part of the "*Genome Diversity in Ukraine*" project by the Institutional Review Board (IRB) of Uzhhorod National University, Uzhhorod Ukraine (**Supplementary File 1**). Each participant had an opportunity to review the informed consent materials (**Supplementary File 2**), and have had the issues around the sharing of genome data explained to them before making any decisions about making it public in this manner.

Competing interests

The following authors declare that they have no competing interests:

Taras K. Oleksyk, Walter W. Wolfsberger, Alexandra Weber, Khrystyna Shchubelka, Stephanie O. Castro-Marquez, Sarah Medley, Alina Urbanovych, Patricia Boldyzhar, Viktoriya Stakhovska, Kateryna Malyar, Mikhailo Neymet, Svitlana Chervyakova, Olena Podoroha, Natalia Kovalchuk, Yaroslava Hasynets, Juan L. Rodriguez-Flores, Fabia Battistuzzi, Siru Chen, Meredith Yeager, Michael Dean, Olga T. Oleksyk, Ryan E. Mills , and Volodymyr Smolanka

The following authors may have competing interests:
Yuan Liu, Huanming Yang are employed by BGI that owns DNBSEQ™ technology, Olga Levchuk, Alla Patrus, Nelya Lazar represent AstraDIA (Ukraine) that collected and extracted DNA samples.

Funding

This research was funded in part by the internal funding from BGI (China), Uzhhorod National University (Ukraine), Division of Cancer Epidemiology and Genetics, National Cancer Institute (USA), and the startup fund of Oakland University, Rochester, Michigan.

Authors' contributions

Conceptualization: Taras K. Oleksyk, Khrystyna Shchubelka, Walter Wolfsberger, Siru Chen, Huanming Yang, Yuan Liu, Volodymyr Smolanka, Juan L. Rodriguez-Flores, Fabia Battistuzzi, Olga T. Oleksyk, Michael Dean, Meredith Yeager, Ryan Mills, and Volodymyr Smolanka

Data curation: Walter Wolfsberger, Khrystyna Shchubelka, Alexandra Weber, Alina Urbanovych, Patricia Boldyzhar, Viktoriya Stakhovska, Kateryna Malyar, Yaroslava Hasynets, Nelya Lazar, Olga T. Oleksyk, Mikhailo Neymet, Svitlana Chervyakova, Olena Podoroha, Natalia Kovalchuk

Formal analysis: Walter Wolfsberger, Alexandra Weber;

Funding acquisition: Taras K. Oleksyk;

Investigation: Khrystyna Shchubelka, Walter Wolfsberger, Alexandra Weber, Stephanie Castro-Marquez;

Methodology: Taras K. Oleksyk, Michael Dean, and Ryan Mills

Project administration: Taras K. Oleksyk, Michael Dean, and Volodymyr Smolanka;

Resources: Taras K. Oleksyk, Huanming Yang, Yuan Liu, Ryan Mills, Meredith Yeager, Michel Dean, Olga T. Oleksyk, and Volodymyr Smolanka

Software: Walter Wolfsberger, Alexandra Weber;

Supervision: Taras K. Oleksyk, Volodymyr Smolanka, Michael Dean, and Ryan Mills;

Visualization: Walter Wolfsberger, Khrystyna Shchubelka, Alexandra Weber;

Writing, Taras K. Oleksyk, Khrystyna Shchubelka, Walter Wolfsberger, Alexandra Weber (original draft), and Taras K. Oleksyk and Ryan Mills (review & editing).

Acknowledgements

We thank all the Ukrainian volunteers who contributed their data for the project.

References

1. Subtelny O. Ukraine: A History, 4th Edition. University of Toronto Press; 2009. 784 p. ISBN 1442609915, 9781442609914. https://utorontopress.com/us/ukraine-10

2. Mathieson I, Alpaslan-Roodenberg S, Posth C, Szécsényi-Nagy A, Rohland N, Mallick S, et al. The genomic history of southeastern Europe. Nature. 2018 **555**: 197–203; doi: 10.1038/nature25778

3. Warmuth V, Eriksson A, Bower MA, Barker G, Barrett E, Hanks BK, et al. Reconstructing the origin and spread of horse domestication in the Eurasian steppe. Proc Natl Acad Sci USA. 2012 109(21):8202-8206; doi: 10.1073/pnas.1111122109.

4. Schubert M, Jónsson H, Chang D, Der Sarkissian C, Ermini L, Ginolhac A, et al. Prehistoric genomes reveal the genetic foundation and cost of horse domestication. Proc Natl Acad Sci USA. 2014, 111(52)E5661-E5669; doi: 10.1073/pnas.1416991111

5. Gaunitz C, Fages A, Hanghøj K, Albrechtsen A, Khan N, Schubert M, et al.. Ancient genomes revisit the ancestry of domestic and Przewalski's horses. Science. 2018 360(6384):111-114; doi: 10.1126/science.aao3297.

6. Librado P, Fages A, Gaunitz C, Leonardi M, Wagner S, Khan N, et al. The Evolutionary Origin and Genetic Makeup of Domestic Horses. Genetics. 2016, 204(2):423-434; doi: 10.1534/genetics.116.194860.

7. Demay L, Péan S, Patou-Mathis M. Mammoths used as food and building resources by Neanderthals: Zooarchaeological study applied to layer 4, Molodova I (Ukraine). Quat Int. 2012 (276-277):212-226; doi: 10.1016/j.quaint.2011.11.019

8. Seguin-Orlando A, Korneliussen TS, Sikora M, Malaspinas A-S, Manica A, Moltke I, et al.. Genomic structure in Europeans dating back at least 36,200 years. Science. 2014; 346:1113-1118, doi: 10.1126/science.aaa0114.

9. Eberhardt P, Owsinski J. Ethnic Groups and Population Changes in Twentieth Century Eastern Europe: History, Data and Analysis (J. Owsinski, Trans.; 1st ed.). Taylor and Francis (Routledge) New York; 1996. doi: 10.4324/9781315704470

10. Oleksyk TK, Brukhin V, O'Brien SJ. The Genome Russia project: closing the largest remaining omission on the world Genome map. GigaScience. 2015, 4(1): s13742–015–0095–0; 2015; doi: 10.1186/s13742-015-0095-0.

11. Oleksyk TK; Wolfsberger WW; Weber A; Shchubelka K; Oleksyk OT; Levchuk O, et. al.. Supporting data for "Genome Diversity in Ukraine". GigaScience Database 2020. http://dx.doi.org/10.5524/100835.

12. Karczewski KJ, Francioli LC, Tiao G, Cummings BB, Alföldi J, Wang Q, et al. The mutational constraint spectrum quantified from variation in 141,456 humans. Nature. 2020, **581:**434–443; doi: 10.1038/s41586-020-2308-7.

13. Zhernakova DV, Brukhin V, Malov S, Oleksyk TK, Koepfli KP, Zhuk A, et al.. Genome-wide sequence analyses of ethnic populations across Russia. Genomics 2019, 112(1):442-458. doi: 10.1016/j.ygeno.2019.03.007

14. Auton A, Abecasis GRGR, Altshuler DM, Durbin RM, Bentley DR, Chakravarti A, et al.. A global reference for human genetic variation. Nature. 2015 **526:** 68–74. ; doi: 10.1038/nature15393.

15. Kim J, Weber JA, Jho S, Jang J, Jun J, Cho YS, et al.. KoVariome: Korean National Standard Reference Variome database of whole genomes with comprehensive SNV, indel, CNV, and SV analyses. Sci Rep. 2018, 8:5677; doi: 10.1038/s41598-018-23837-x.

16. Van der Auwera GA, Carneiro MO, Hartl C, Poplin R, del Angel G, Levy-Moonshine A, et al. From fastQ data to high-confidence variant calls: The genome analysis toolkit best practices pipeline. Curr Protoc Bioinformatics. 2013, 43(1110):11.10.1-11.10.33; doi: 10.1002/0471250953.bi1110s43.

17. Layer RM, Chiang C, Quinlan AR, Hall IM. LUMPY: a probabilistic framework for structural variant discovery. Genome Biol. 2014 **15:**R84; doi: 10.1186/gb-2014-15-6-r84.

18. Schneider VA, Graves-Lindsay T, Howe K, Bouk N, Chen H-C, Kitts PA, et al. Evaluation of GRCh38 and de novo haploid genome assemblies demonstrates the enduring quality of the reference assembly. Genome Res. 2017, 27(5):849-864. doi: 10.1101/gr.213611.116.

19. Mayakonda A, Lin D-C, Assenov Y, Plass C, Koeffler HP. Maftools: efficient and comprehensive analysis of somatic variants in cancer. Genome Res. 2018, 28(11):1747-1756; doi: 10.1101/gr.239244.118.

20. Sherry ST, Ward M, Sirotkin K. dbSNP—Database for Single Nucleotide Polymorphisms and Other Classes of Minor Genetic Variation. Genome Res. 1999, 9(8):677-679; doi: 10.1101/gr.9.8.677.

21. Campbell IM, Gambin T, Jhangiani S, Grove ML, Veeraraghavan N, Muzny DM, et al. Multiallelic Positions in the Human Genome: Challenges for Genetic Analyses. Hum Mutat. 2016, 37(3):231-234; doi: 10.1002/humu.22944.

22. Kosugi S, Momozawa Y, Liu X, Terao C, Kubo M, Kamatani Y. Comprehensive evaluation of structural variation detection algorithms for whole genome sequencing. Genome Biol. 2019, 20:117. doi: 10.1186/s13059-019-1720-5.

23. Ye K, Hall G, Ning Z. Structural variation detection from next generation sequencing. Next Generat Sequenc & Applic. 2015, S1:007. doi: 10.4172/2469-9853.S1-007

24. MacDonald JR, Ziman R, Yuen RKC, Feuk L, Scherer SW. The Database of Genomic Variants: a curated collection of structural variation in the human genome. Nucleic Acids Res. 2014, 42(Database issue):D986-92; doi: 10.1093/nar/gkt958.

25. Mak SST, Gopalakrishnan S, Carøe C, Geng C, Liu S, Sinding M-HS, et al.. Comparative performance of the BGISEQ-500 vs Illumina HiSeq2500 sequencing platforms for palaeogenomic sequencing. GigaScience, 2017, 6(8): gix049. doi: 10.1093/gigascience/gix049

26. Zhou Y, Liu C, Zhou R, Lu A, Huang B, Liu L, et al.. SEQdata-BEACON: a comprehensive database of sequencing performance and statistical tools for performance evaluation and yield simulation in BGISEQ-500. BioData Min. 2019, 12:21; doi: 10.1186/s13040-019-0209-9.

27. Loewe L, Hill WG. The population genetics of mutations: good, bad and indifferent. Philos Trans R Soc Lond B Biol Sci. 2010, 365(1544):1153-67; doi: 10.1098/rstb.2009.0317.

28. Volfovsky N, Oleksyk TK, Cruz KC, Truelove AL, Stephens RM, Smith MW. Genome and gene alterations by insertions and deletions in the evolution of human and chimpanzee chromosome 22. BMC Genomics. 2009, 10:51; doi: 10.1186/1471-2164-10-51.

29. Buniello A, MacArthur JAL, Cerezo M, Harris LW, Hayhurst J, Malangone C, et al.. The NHGRI-EBI GWAS Catalog of published genome-wide association studies, targeted arrays and summary statistics 2019, Nucleic Acids Res. 2019, 47(D1):D1005-D1012; doi: 10.1093/nar/gky1120.

30. Landrum MJ, Lee JM, Benson M, Brown GR, Chao C, Chitipiralla S, et al. ClinVar: improving access to variant interpretations and supporting evidence. Nucleic Acids Res. 2018, 46(D1):D1062-D1067; doi: 10.1093/nar/gkx1153.

31. Landrum MJ, Lee JM, Benson M, Brown G, Chao C, Chitipiralla S, et al.. ClinVar: public archive of interpretations of clinically relevant variants. Nucleic Acids Res. 2016, 44(D1):D862-8; doi: 10.1093/nar/gkv1222.

32. Cooper DN, Krawczak M, Polychronakos C, Tyler-Smith C, Kehrer-Sawatzki H. Where genotype is not predictive of phenotype: towards an understanding of the molecular basis of reduced penetrance in human inherited disease. Hum Genet. 2013, 132(10): 1077–1130; doi: 10.1007/s00439-013-1331-2.

33. Lobo I. Same genetic mutation, different genetic disease phenotype. Scitable: Nature Education. 2008;1(1):64. https://www.nature.com/scitable/topicpage/same-genetic-mutation-different-genetic-disease-phenotype-938/

34. Visscher PM, Wray NR, Zhang Q, Sklar P, McCarthy MI, Brown MA, et al. 10 Years of GWAS Discovery: Biology, Function, and Translation. Am J Hum Genet. 2017, 101(1):5-22; doi: 10.1016/j.ajhg.2017.06.005.

35. Marigorta UM, Rodríguez JA, Gibson G, Navarro A. Replicability and Prediction: Lessons and Challenges from GWAS. Trends Genet. 2018, 34(7):504-517; doi: 10.1016/j.tig.2018.03.005.

36. Eilbeck K, Quinlan A, Yandell M. Settling the score: variant prioritization and Mendelian disease. Nat Rev Genet. 2017, **18:**599–612; doi: 10.1038/nrg.2017.52.

37. Boyle EA, Li YI, Pritchard JK. An Expanded View of Complex Traits: From Polygenic to Omnigenic. Cell. 2017, 169(7):1177-1186; doi: 10.1016/j.cell.2017.05.038.

38. Oleksyk TK, Smith MW, O'Brien SJ. Genome-wide scans for footprints of natural selection. Philos Trans R Soc Lond B Biol Sci. 2010, 365:185-205; doi: 10.1098/rstb.2009.0219.

39. Altshuler DM, Durbin RM, Abecasis GR, Bentley DR, Chakravarti A, Clark AG, et al.. An integrated map of genetic variation from 1,092 human genomes. Nature. 2012, **491:**56–65; doi: 10.1038/nature11632.

40. Bergström A, McCarthy SA, Hui R, Almarri MA, Ayub Q, Danecek P, et al.. Insights into human genetic variation and population history from 929 diverse genomes. Science. 2020 367(6484):eaay5012. doi: 10.1126/science.aay5012.

41. Nugent A, Conatser KR, Turner LL, Nugent JT, Sarino EMB, Ricks-Santi LJ. Reporting of race in genome and exome sequencing studies of cancer: a scoping review of the literature. Genet Med. 2019, **21:**2676–2680; doi: 10.1038/s41436-019-0558-2.

42. Spratt DE, Chan T, Waldron L, Speers C, Feng FY, Ogunwobi OO, et al. Racial/Ethnic Disparities in Genomic Sequencing. JAMA Oncol. 2016, 2(8): 1070–1074.; doi: 10.1001/jamaoncol.2016.1854.

43. Pagani L, Lawson DJ, Jagoda E, Mörseburg A, Eriksson A, Mitt M, et al. Genomic analyses inform on migration events during the peopling of Eurasia. Nature. 2016, 538(7624):238-24; doi: 10.1038/nature19792.

44. Mallick S, Li H, Lipson M, Mathieson I, Gymrek M, Racimo F, et al. The Simons Genome Diversity Project: 300 genomes from 142 diverse populations. Nature. 2016, **538:**201–206; doi: 10.1038/nature18964.

45. Chen C-Y, Pollack S, Hunter DJ, Hirschhorn JN, Kraft P, Price AL. Improved ancestry inference using weights from external reference panels. Bioinformatics. 2013, 29(11):1399-406; doi: 10.1093/bioinformatics/btt144.

46. Smith MW, O'Brien SJ. Mapping by admixture linkage disequilibrium: advances, limitations and guidelines. Nat Rev Genet. 2005, 6(8):623-32; doi: 10.1038/nrg1657.

47. Stephens JC, Briscoe D, O'Brien SJ. Mapping by admixture linkage disequilibrium in human populations: limits and guidelines. Am J Hum Genet. 55:809–824. PMID: 7942858; PMCID: PMC1918304.

48. Patterson N, Price AL, Reich D, Plenge RM, Weinblatt ME, Shadick NA, et al. EIGENSOFT version 5.01. Harvard University. 2013; Downloaded from: data.broadinstitute.org/alkesgroup/EIGENSOFT/

49. Alexander DH, Novembre J, Lange K. Fast model-based estimation of ancestry in unrelated individuals. Genome Res. 2009, 19(9): 1655–1664; doi: 10.1101/gr.094052.109.

50. Gardner EJ, Lam VK, Harris DN, Chuang NT, Scott EC, Pittard WS, et al. The Mobile Element Locator Tool (MELT): population-scale mobile element discovery and biology. Genome Res. 2017, 27(11):1916-1929; doi: 10.1101/gr.218032.116.

51. Mousavi N, Shleizer-Burko S, Yanicky R, Gymrek M. Profiling the genome-wide landscape of tandem repeat expansions. Nucleic Acids Res. 2019, 47(15): e90; doi: 10.1093/nar/gkz501.

52. Dayama G, Emery SB, Kidd JM, Mills RE. The genomic landscape of polymorphic human nuclear mitochondrial insertions. Nucleic Acids Res. 2014, 42(20):12640-12649; doi: 10.1093/nar/gku1038.

53. Wang K, Li M, Hakonarson H. ANNOVAR: functional annotation of genetic variants from high-throughput sequencing data. Nucleic Acids Res. 2010, 38(16):e164; doi: 10.1093/nar/gkq603.

54. Cingolani P, Platts A, Wang LL, Coon M, Nguyen T, Wang L, et al. A program for annotating and predicting the effects of single nucleotide polymorphisms, SnpEff: SNPs in the genome of Drosophila melanogaster strain w1118; iso-2; iso-3. Fly . 2012, 6(2):80-92; doi: 10.4161/fly.19695.

55. Liu X, Wu C, Li C, Boerwinkle E. dbNSFP v3.0: A One-Stop Database of Functional Predictions and Annotations for Human Nonsynonymous and Splice-Site SNVs. Hum Mutat. 2016, 37(3):235-41; doi: 10.1002/humu.22932.

56. Zheng-Bradley X, Streeter I, Fairley S, Richardson D, Clarke L, Flicek P, et al. Alignment of 1000 Genomes Project reads to reference assembly GRCh38. GigaScience. 2017, 6(7):1-8; doi: 10.1093/gigascience/gix038.

57. Cingolani P, Patel V, Coon M, Nguyen T, Land S, Ruden DM. Using Drosophila melanogaster as a Model for Genotoxic Chemical Mutational Studies with a New Program, SnpSift. Front Genet. 2012, 3:35; doi: 10.3389/fgene.2012.00035.

58. McKinney W, Others. Data structures for statistical computing in Python. Proceedings of the 9th Python in Science Conference. Austin, TX; p. 51–6. doi: 10.25080/MAJORA-92BF1922-00A

59. Virtanen P, Gommers R, Oliphant TE, Haberland M, Reddy T, Cournapeau D, et al. SciPy 1.0: fundamental algorithms for scientific computing in Python. Nat Methods. 2020, **17:**261–272; doi: 10.1038/s41592-019-0686-2.

60. Purcell S, Neale B, Todd-Brown K, Thomas L, Ferreira MAR, Bender D, et al. PLINK: a tool set for whole-genome association and population-based linkage analyses. Am J Hum Genet. 2007, 81(3):559-75; doi: 10.1086/519795.

61. Lander ES, Linton LM, Birren B, Nusbaum C, Zody MC, Baldwin J, et al. Initial sequencing and analysis of the human genome. Nature. 2001, **409:**860–921; doi: 10.1038/35057062.

62. Venter JC, Adams MD, Myers EW, Li PW, Mural RJ, Sutton GG, et al. The sequence of the human genome. Science. 2001, 291(5507):1304-1351; doi: 10.1126/science.1058040.

63. Sherman RM, Salzberg SL. Pan-genomics in the human genome era. Nat Rev Genet. 2020, 21(4):243-254. doi: 10.1038/s41576-020-0210-7.

64. Kempfer R, Pombo A. Methods for mapping 3D chromosome architecture. Nature Reviews Genetics. 2020 Apr;21(4):207-226. doi: 10.1038/s41576-019-0195-2.

65. Oleksyk TK, Brukhin V, O'Brien SJ, Sills J. Genome Russia. Science. 2015, 350(6262): 747; doi: 10.1126/science.350.6262.747-a.

Supplementary File 1

Click here to access/download
Supplementary Material
Supplementary File 1. The IRB Approval.pdf

Click here to access/download
Supplementary Material
Supplementary File 2. The Informed Consent.docx

Click here to access/download
Supplementary Material
Supplementary File 3. The List of Samples.xlsx

Supplementary File 4

Click here to access/download
Supplementary Material
Supplementary File 5. Sample Sources.xlsx

Table S1

Click here to access/download

Supplementary Material

Table S1_sequencing_stats.xlsx

Table S2

Click here to access/download
Supplementary Material
Table S2. Sequencing and Filtering Summary.xlsx

Table S3

Click here to access/download
Supplementary Material
Table S3_High_impact_full_2.xlsx

Table S4.1

Click here to access/download
Supplementary Material
Table S4_Clinvar_clinically_relevant.xlsx

Table S4.2

Click here to access/download
Supplementary Material
Table S4_differentiative_functional_R2.xlsx

Table S4.3

Click here to access/download
Supplementary Material
Table S4_Gwas_clinically_relevant.xlsx

Table S5

Click here to access/download
Supplementary Material
Table S5_all_differentiative_R2.xlsx

Table S6

Click here to access/download
Supplementary Material
Table S6_all_differentiative.xlsx

Table S7

Click here to access/download
Supplementary Material
Table S7_inbreeding.xlsx
