# Supplementary material for: Genome diversity in Ukraine
Source: Gigascience. 2021 Jan 13;10(1):giaa159. doi: 10.1093/gigascience/giaa159 (PMC7804371; doi:10.1093/gigascience/giaa159)
Supplement: giaa159_Supplemental_Files [file giaa159_supplemental_files.zip › Supplementary File 1. The IRB Approval.pdf]

Висновок етичного комітету № 1 від 19 вересня 2018
ДВНЗ «Ужгородський національний університет»
щодо дотримання морально-правових правил проведення біомедичних наукових досліджень

Етичний комітет ДВНЗ «Ужгородський національний університет» розглянув протокол дослідження, інформовану згоду та анкету учасника до проекту "100 українських геномів" (затверджений до виконання Вченою радою біологічного факультету, протокол №6 від 1 березня 2018 року) під керівництвом Тараса Олексика ***і встановив наступне:***

місце проведення досліджень: міжвідомча науково-дослідна лабораторія охорони природних екосистем УжНУ, Національний інститут раку (США), Пекінський Геномний Інститут (Китай);

мета проекту: пошук генетичних варіацій у населення України, яке ніколи масштабно не вивчалося з генетичної точки зору;

методи дослідження: наукове спостереження, анкетування. Дослідження не є клінічним.

Вивчивши сукупність наданих документів, етичний комітет ДВНЗ «Ужгородський національний університет» постановляє, що протокол дослідження відповідає основним положенням Ухвали Першого національного конгресу з біоетики «Загальні етичні принципи експериментів на тваринах» (2001 р.), ICH GCP (1996 р.), Конвенцій Ради Європи про права людини та біомедицину (від 04.04.1997р.) та про охорону хребетних тварин, що використовують в експериментах та інших наукових цілях (від 18.03.1986 р.), Гельсінської декларації Всесвітньої медичної асоціації про етичні принципи проведення наукових медичних досліджень за участю людини (1964-2013 рр.), Директив ЄС № 609 (від 24.11.1986 р.), наказів МОЗ України № 690 від 23.09.2009р., № 944 від 14.12.2009 р., № 616 від 03.08.2012 р. та надає висновок, що

запропоноване дослідження відповідає міжнародним та державним стандартам щодо біомедичних наукових досліджень.

Подальше внесення змін до протоколу дослідження, інформованої згоди чи анкети учасника дослідження можливе лише за погодженням етичного комітету.

Голова етичного комітету [signature] проф. Слабкий Г.О.

Підпис проф. Слабкий Г.О.
ЗАСВІДЧУЮ
Вчений секретар ДВНЗ «УжНУ»
[signature]
«19» вересня 2018

ДЕРЖАВНИЙ ВИЩИЙ НАВЧАЛЬНИЙ ЗАКЛАД "УЖГОРОДСЬКИЙ НАЦІОНАЛЬНИЙ УНІВЕРСИТЕТ" МІНІСТЕРСТВО ОСВІТИ І НАУКИ УКРАЇНИ
код 02070832
