# Supplementary material for: Genome diversity in Ukraine
Source: Gigascience. 2021 Jan 13;10(1):giaa159. doi: 10.1093/gigascience/giaa159 (PMC7804371; doi:10.1093/gigascience/giaa159)
Supplement: giaa159_Supplemental_Files [file giaa159_supplemental_files.zip › Supplementary File 2. The Informed Consent.docx]

### Проект «Геномне різноманіття населення України»:

### опис протоколу, анкета та інформована згода на участь

#### Основні визначення:

Результати дослідження - набір зразків у біорепозитаріях, дані геномної послідовності ДНК, отримані від кожного окремого учасника дослідження шляхом секвенування або генотипування, узагальнена статистична інформація, графіки, ілюстрації, статті та усні презентації, які є результатом даного проекту.

Професійні наукові джерела - друковані та електронні (онлайн) джерела, серед яких бази даних, сховища, журнали, матеріали конференцій, книги, публікації в пресі, що пов'язані з проектом.

#### Короткий опис протоколу для учасників

Проект «Геномне різноманіття населення України» проводиться з метою створення першої геномної бази даних та вивчення генетичної різноманітності і варіантів притаманних населенню України.

Результати цього дослідження доповнюють існуючі наукові каталоги генетичної різноманітності людей і включатимуть наступне:

**1. Зразки крові та/або слини та матеріали**, виділені з цих зразків, які зберігатимуться у біорепозитаріях та поширюватимуться серед дослідників для використання у майбутніх проектах на вимогу, за умови збереження конфіденційності кожного учасника;

**2. Первинні дані про послідовність ДНК з вказаних зразків**, які будуть завантажені у наукові бази даних, доступні у професійних наукових джерелах в Інтернеті для проведення майбутніх досліджень.

**3. Статистично проаналізовані узагальнені дані**, графіки, наукові статті, усні виступи, які будуть оприлюднені оголошення результатів цього дослідження.

У проекті братимуть участь щонайменше 100 добровольців кожної статі, предки яких походять з різних регіонів України. УжНУ організує набір учасників в Україні та сусідніх країнах.

Результати дослідження будуть використані у майбутніх дослідженнях, пов’язаних зі здоров’ям та хворобами людей. Міжнародна дослідна група з кількох країн спільно працює над цим проектом, до її складу входять вчені з Ужгородського національного університету (УжНУ, Україна), Оклендського університету (ОU, США), Пекінського геномного інституту (BGI, Китай), Національного інституту здоров’я США (NIH) та Мічиганського університету (UofM, США).

#### Щоб взяти участь у дослідженні, ваша кандидатура повинна задовольнити наступні критерії включення:

• ви повинні мати принаймні 18 років і мати змогу дати інформовану згоду за власним бажанням;

• ви повинні добровільно здати зразок крові та / або слини, щоб дослідники могли отримати вашу особисту послідовність нуклеотидів ДНК шляхом секвенування або генотипування;

• ви повинні дозволити використовувати ваш зразок у «клітинній лінії», тобто живій культурі клітин, виготовленій зі зразка крові, яка б дала можливість дослідникам тривалий час отримувати генетичний матеріал з того самого зразка у довготривалій перспективі (можливо, на невизначений час);

• ви повинні бути готовим до того, що генетична інформація, отримана в результаті цього дослідження, буде у вільному доступі в наукових базах даних в мережі Інтернет. Ми ніколи не розголошуватимемо вашу традиційну ідентифікаційну інформацію, яка пов'язує генетичні дані особисто з вами, такі як ваше ім’я, професія, адреса та дата народження;

• ви повинні бути готовим, що багато дослідників у всьому світі будуть вивчати генетичний матеріал та дані з вашого зразка у довготривалій перспективі, а також можуть зберігати інформацію, яку вони отримують з нього, щоб використовувати їх у наукових базах даних. Наголошуємо, що ім’я чи будь-які інші стандартні особисті ідентифікатори особи не будуть пов’язані з даними, оприлюдненими цим дослідженням. Дослідники, які вивчають матеріал та дані зразків, можуть називати лише стать, медичну інформацію, надану в анкеті, та власний звіт про походження (етнічну чи географічну групу) учасника дослідження та його предків.

Рішення про те, чи брати участь у цьому дослідженні, залежить від людини особисто і є абсолютно добровільним.

#### Мета цього дослідження

Гени - це основні інструктори для клітин, які складають наше тіло, вони утворюються з ДНК. Хоча ніхто, за виключенням ідентичних близнюків, не має абсолютно однакової послідовності ДНК, будь яка особа більш ніж на 99% схожа на будь-яку іншу неспоріднену людину на планеті. Таким чином вся різноманітність між людьми не виходить за межі 1%, проте кілька відмінностей, які пов’язані з конкретними ділянками хромосом, дозволяють зрозуміти деякі фізичні та поведінкові відмінності між людьми, а також можуть пояснити, чому деякі люди хворіють на рак, діабет, астму та депресію, а інші - ні. У той же час, послідовність ДНК не є на 100% визначальною для вашої зовнішності чи стану здоров’я: на захворювання можуть впливати також такі фактори, як харчування, фізичні навантаження, паління та забруднення навколишнього середовища, що ускладнює дослідження, які шукають гени, які мають вплив на ці захворювання.

Більшість генетичних варіантів (алелів) не відрізняються за частотою між різними людським популяціями у всьому світі. У той же час існують також алелі з яскраво вираженими географічними відмінностями у частоті між різними групами населення, континентальними та етнічними групами. Такі алелі можуть визначати регіональні відмінності в чутливості та стійкості до різних захворювань та впливати на відповідь до різних хімічних речовин, наприклад, таких як ліки, отрути, тощо.

Мета нашого проекту - краще розуміння унікальних генетичних варіацій в Україні, їх походження в контексті історії населення та хвороб. Ми робимо це, вивчаючи ДНК у зразках крові та/або слини, зібраних у багатьох людей, предки яких походять з різних куточків України, і порівнюємо її з уже вивченими сучасними та древніми популяціями. Ми завантажимо цю інформацію в наукові бази даних, щоб вона була доступною для вивчення іншими дослідниками. Ці наукові бази даних зберігатимуться протягом тривалого часу, і багато майбутніх вчених у всьому світі використовуватимуть їх для пошуку генів та генетичних варіантів, пов’язаних зі здоров’ям та хворобами. Наукові бази даних, які ми розробляємо для цього проекту, можуть містити медичну інформацію, яка буде корисною для того, щоб допомогти майбутнім дослідникам дізнатися про здоров'я, захворювання і персоналізувати методи лікування.

Науковці, які вивчають захворювання з генетичним компонентом (наприклад, цукровий діабет), порівнюють два різних набори зразків - деякі отримані від людей, у яких є захворювання, а інші - від людей, які на нього не хворіють. Проводиться пошук послідовності ДНК, яка відрізняються між двома групами, щоб пов’язати ці відмінності із хворобою. Наш проект надасть цим майбутнім дослідженням унікальний набір варіантів специфічних для нашої країни, який можна вивчити в Україні та сусідніх популяціях, щоб допомогти з’ясувати, які гени можуть мати вплив на захворювання. Незабаром вони можуть з’ясувати, як працюють гени, і врешті-решт знайти кращі способи запобігання, діагностики та лікування хвороб. Крім того, ця інформація допоможе зрозуміти, як різні люди реагують на різні лікарські засоби, та про те, як між людьми відрізняються такі фенотипові риси як довголіття або поведінка (в тому числі психологічні або наркологічні залежності). У кінцевому результаті, ця інформація може бути використана для розуміння історії походження, міграцій та генетичного змішування місцевого населення України. Для того, щоб допомогти зрозуміти ці закономірності, учасникам дослідження буде запропоновано заповнити анкету, додану нижче, особиста інформація буде вилучена, а анкети будуть закодовані незалежною стороною і не будуть включені до жодних оприлюднених даних, тож дослідники, які вивчатимуть і аналізуватимуть ваші дані в майбутньому, не матимуть можливості ідентифікувати ваші дані з вашою особою.

Важливо підкреслити, що наш проект є суто дослідницьким і не буде використовуватися з метою надання медичної допомоги. Якщо у вас є проблеми зі здоров’ям або запитання медичного характеру, ви повинні звернутися до лікаря.

#### Ризики щодо участі в дослідженні

Ми вважаємо, що ви маєте право знати свої дані та використовувати їх для особистої вигоди. Однак не виключено, що хтось може спробувати ідентифікувати вашу особисту послідовність в наукових базах даних ДНК послідовностей викладених в Інтернеті, використовуючи сторонні методи не пов’язані з нашим дослідженням. Це може призвести до того, що ваша персональна інформація може бути використана сторонніми особами, компаніями (наприклад, страхові компанії) та державними установами. Тому ми радимо проявляти обережність у наданні особистої інформації пов’язаної з вашими генетичними даними стороннім особам, щоб уникнути цього ризику.

Ми вважаємо, що біологія не дає причин для забобонів, а генетична історія не може трактуватися як правильна чи неправильна, але дискримінація за ознакою походження і досі існує. Наше дослідження допоможе розкрити історію місцевого населення, і ця інформація може допомогти приймати медичні рішення, які принесуть користь людському здоров'ю. Ось чому назви етнічних чи географічних груп будуть включені до зразків та до наукових баз даних. Завдяки цьому проекту науковці можуть виявити, що певні генетичні маркери з’являються частіше у людей з вашої групи, ніж у людей з інших груп, і що ці варіації частіше зустрічаються у людей з певним захворюванням. Ми будемо працювати над тим, щоб етнічна чи географічна ідентичність вашої громади була описана максимально ретельно - у колекції зразків, у наукових базах даних та у статтях, які вчені проекту пишуть на основі цього дослідження, але ми не можемо повністю контролювати, як ця інформація описується в публікаціях, які пишуть інші особи. Хтось може інтерпретувати інформацію з наукових баз даних або з майбутніх досліджень з використанням наукових баз даних, щоб перебільшити відмінності між групами з упереджених чи інших недобросовісних причин. Ця інформація може бути використана також для применшення відмінностей між групами людей, щоб вказати, що гени всіх людей практично однакові, з метою нівелювання особливих проблем та потреб певних груп населення. Такі інтерпретації можливі і хоча вони не є метою наших зусиль, ми не можемо гарантувати трактування доступної генетичної інформації особами, які не є причетними до наших досліджень.

### Добровільна інформована згода на участь у науковому дослідженні “Геномне різноманіття населення України”

**Інформація для учасника:** даний проект проводиться під керівництвом професора Тараса Олексика на базі Ужгородського національного університету у кооперації з іноземними партнерами. Проект має на меті встановити генетичний склад української популяції, яка ще ніколи не вивчалася з такого наукового ракурсу. Результатами дослідження буде створення базової колекції зразків ДНК, які можуть бути взяті за основу для подальших масштабних генетичних досліджень. Також отримані дані можуть мати практичне значення у разі їх впровадження у сфері персоніфікованої медицини і лікування хвороб згідно особливостей генетичного складу українців.

Дані учасників дослідження будуть знеособлені і оброблятимуться згідно Закону України “Про захист персональних даних”. Деякі результати дослідження можуть бути надані учасникам після закінчення проекту і аналізу отриманих даних.

Забір венозної крові проводиться стандартним способом з ліктьової вени, стерильним медичним інструментом в лабораторних умовах.

Я _______________________________________________________________,

надаю свою добровільну інформовану згоду на участь у даному дослідженні, забір, аналіз і обробку мого біологічного матеріалу.

**Я згоден(-на) на наступне:**

1. Публікацію анонімних первинних даних про послідовність ДНК з мого зразка у фахових виданнях доступних у мережі Інтернет. Я розумію, що ці дані будуть доступні для подальших досліджень іншими вченими.

2. Публікацію статистично проаналізованих узагальнених даних, графіків, наукових статей, усних виступів, які будуть результатами цього дослідження.

3. Збереження мого зразка крові у біорепозитарії та отримання клітинних ліній з нього.

Я не заперечую щодо публікації результатів дослідження у фахових наукових виданнях зі збереженням анонімності моїх персональних даних. Я мав змогу отримати відповіді на всі запитання у мого дослідника.

**Дата _________ Підпис ____________________**

### Genomic Diversity of Ukraine's Population Project: Protocol description, questionnaire, and informed consent to participate

#### Basic Definitions:

The results of the study are a set of samples in biorepositories, genomic DNA sequence data obtained from each individual study participant by sequencing or genotyping, summarized statistical information, graphs, illustrations, articles and oral presentations that result from this project.

Professional Scientific Sources - Printed and electronic (online) sources including databases, repositories, journals, conference materials, books, and press releases related to the project.

#### A brief description of the protocol for participants

The project "Genomic Diversity of the Population of Ukraine" is being carried out to create the first genomic database and to study genetic diversity and variants inherent in the human population of Ukraine.

The results of this study will complement existing scientific catalogs of human genetic diversity and will include the following:

1. Blood and / or saliva specimens and materials extracted from these specimens, which will be stored in biorepositories and distributed to researchers on-demand for use in future projects, provided that each participant is kept confidential;

2. Initial DNA sequence data from the specimens uploaded to scientific databases and available from professional scientific sources on the Internet for future research.

3. Generalized data, graphs, scientific articles, oral presentations will be statistically analyzed and the results of this study will be announced.

At least 100 volunteers of each gender, whose ancestors are from different regions of Ukraine will take part in this project. UzhNU (Uzhhorod National University) organizes recruitment of participants in Ukraine and neighboring countries.

The results of the study will be used in future studies related to human health and illness. An international research group from several countries is working together on this project, which includes scientists from Uzhgorod National University (UzhNU, Ukraine), Oakland University (OU, USA), Beijing Genomic Institute (BGI, China), National Institute of Health , USA (NIH) and the University of Michigan (UofM, USA).

#### In order to participate in the study, the participant must meet the following eligibility criteria:

• must be at least 18 years old and be able to give informed consent at will;

• must voluntarily donate a blood and / or saliva sample so that researchers can obtain personal DNA nucleotide sequence by sequencing or genotyping;

• must allow your sample to be used in a "cell line", that is, a live cell culture made from a blood sample that would allow researchers to obtain genetic material from the same sample for a long time (possibly forever);

• must be prepared that the genetic information obtained from this research will be freely available in scientific databases on the Internet. We will never disclose your traditional personally identifying information that connects personally-identifiable genetic information to you, such as your name, profession, address, and date of birth;

• must be prepared that many researchers around the world will study genetic material and data from your sample over the long term, and can store the information they receive from it to use in scientific databases. It is emphasized that the name or any other standard personal identity identifiers will not be associated with the data released by this study. Researchers who study material and sample data can only name the gender, the medical information provided in the questionnaire, and their own report on the origin (ethnic or geographical group) of the study participant and his or her ancestors.

The decision on whether to participate in this study depends on the individual personally and is completely voluntary.

#### The purpose of this study

Genes are the basic instructors for the cells that make up our body, they are composed of DNA. Although no one except the identical twins has exactly the same DNA sequence, any person is more than 99% similar to any other unrelated person on the planet. Thus, all human diversity does not go beyond 1%, but the few differences that are associated with specific chromosome areas allow us to understand some of the physical and behavioral differences between people, and can also explain why some people have cancer, diabetes, asthma and depression, and others do not. At the same time, the DNA sequence is not 100% determinative of your appearance or health: diseases can also be affected by factors such as nutrition, physical activity, smoking, and environmental pollution, which complicates gene-discovering studies of geneswhich have an impact on these diseases.

Most genetic variants (alleles) do not differ in frequency between different human populations around the world. Such alleles can detect regional differences in susceptibility and resistance to various diseases and affect the response to different chemicals, such as drugs, poisons, etc.

The purpose of our project is to better understand the unique genetic variations in Ukraine, their origins in the context of population history and disease. We do this by examining DNA in blood and / or saliva samples collected from many people, whose ancestors come from different parts of Ukraine, and compare it with current and ancient populations already studied. We will upload this information to a scientific database so that it can be shared with other researchers. These scientific databases will be stored for a long time, and many future scientists around the world will use them to search for genes and genetic variants related to health and disease. The scientific databases we are developing for this project may contain medical information that will be useful to help future researchers learn about health, disease and personalize treatments.

Scientists studying diseases with a genetic component (such as diabetes) compare two different sets of samples, some from people who have the disease and others from people who don't have the disease. DNA sequences that differ between the two groups are searched to relate these differences to the disease. Our project will provide these future studies with a unique set of country-specific options that can be explored in Ukraine and neighboring populations to help determine which genes may have an effect on the disease. Soon they can figure out how genes work, and eventually find better ways to prevent, diagnose, and treat diseases. In addition, this information will help you understand how different people respond to different medicines and how different phenotypic traits such as longevity or behavior (including psychological or drug addictions) differ between people. Ultimately, this information can be used to understand the history of origin, migration, and genetic admixture of the local population in Ukraine. In order to help us understand these patterns, research participants will be asked to complete the questionnaire attached below, personal information will be removed, and the questionnaires will be coded by an independent party and not included in any published data, so researchers who will study and analyze your data in the future , will not be able to identify your data with your person.

It is important to emphasize that our project is solely research-based and will not be used for medical assistance. If you have a health problem or a medical question, you should consult with your doctor.

#### Risks of participating in the study

We believe that you have the right to know your data and use it for your personal benefit. However, it is not excluded that someone may try to identify your personal sequence in the scientific DNA databases of the sequences posted on the Internet, using third-party methods not related to our study. This may result in your personal information being used by third parties, companies (such as insurance companies) and government agencies. Therefore, we advise caution in providing personal information related to your genetic data to third parties to avoid this risk.

We believe that biology provides no cause for prejudice, and genetic history cannot be interpreted as right or wrong, but discrimination on the basis of origin still exists. Our research will help uncover the history of the local population, and this information can help make medical decisions that will benefit human health. This is why ethnic or geographical group names will be included in samples and scientific databases. Through this project, scientists may find that certain genetic markers appear more often in people in your group than in people in other groups, and that these variations are more common in people with a specific disease. We will work to ensure that your community's ethnic or geographical identity is described as carefully as possible - in sample collections, scientific databases, and articles written by project scientists based on this research, but we cannot fully control how this information is described. in posts written by others. Someone may interpret information from scientific databases or from future research using scientific databases to magnify differences between groups for biased or other unfair reasons. This information can also be used to reduce differences between groups of people to indicate that the genes of all people are virtually identical, in order to counteract the particular problems and needs of certain populations. Such interpretations are possible and although they are not the goal of our efforts, we cannot guarantee the interpretation of available datasets.

This information can also be used to reduce differences between groups of people to indicate that the genes of all people are virtually identical, in order to counteract the particular problems and needs of certain populations. Such interpretations are possible, and although they are not the purpose of our efforts, we cannot guarantee the treatment of available genetic information by persons who are not involved in our research.

### Voluntary informed consent to participate in the research "Genomic Diversity of the Population of Ukraine"

Information for the participant: this project is conducted under the guidance of Professor Taras Oleksyk at Uzhgorod National University in cooperation with foreign partners. The project aims to establish the genetic makeup of a Ukrainian population that has never been studied from such scientific prospective. The results of the study will create a basic collection of DNA samples that can be taken as a basis for further large-scale genetic research. The data obtained may also be of practical importance in the case of their implementation in the field of personalized medicine and treatment of diseases according to the peculiarities of the genetic makeup of Ukrainians.

The data of the study participants will be depersonalized and will be processed in accordance with the Law of Ukraine “On Protection of Personal Data”. Some results of the study may be provided to participants after the end of the project and analysis of the data obtained.

Venous blood sampling is carried out in the standard way from the elbow vein, with a sterile medical instrument in the laboratory.

I_______________________________________________________________,

I give my voluntary informed consent to participate in the research, collection, analysis and to process my biological material.

I agree to the following:

1. Publication of anonymous primary DNA sequence data from my sample in professional publications available on the Internet. I understand that this data will be available for further research by other scientists.

2. Publication of statistically analyzed generalized data, graphs, scientific articles, oral presentations that will result from this study.

3. Keeping my blood sample in biorepositories and getting cell lines from it.

I do not dispute the publication of the results of the study in professional scientific publications, while maintaining the anonymity of my personal data. I was able to get all the answers from my researcher.

Date _________ Signature ____________________
