# Supplementary material for: Genome diversity in Ukraine
Source: Gigascience. 2021 Jan 13;10(1):giaa159. doi: 10.1093/gigascience/giaa159 (PMC7804371; doi:10.1093/gigascience/giaa159)
Supplement: giaa159_GIGA-D-20-00230_Original_Submission [file giaa159_giga-d-20-00230_original_submission.pdf]

GigaScience

Genome Diversity in Ukraine

--Manuscript Draft--

**Manuscript Number:**	GIGA-D-20-00230
**Full Title:**	Genome Diversity in Ukraine
**Article Type:**	Data Note
**Funding Information:**	
**Abstract:**	The main goal of this collaborative effort is to provide genome wide data for the previously underrepresented population in Eastern Europe, and to provide cross-validation of the data from genome sequences and genotypes of the same individuals acquired by different technologies. We collected 97 genome-grade DNA samples from consented individuals representing major regions of Ukraine that were consented for the public data release. DNBSEQ-G50 sequences, and genotypes by an Illumina GWAS chip were cross-validated on multiple samples, and additionally referenced to a sample that has been resequenced by Illumina NovaSeq6000 S4 at high coverage. The genome data has been searched for genomic variation represented in this population, and a number of variants have been reported: large structural variants, indels, CNVs, SNPs and microsatellites. This study is providing the largest to-date survey of genetic variation in Ukraine, creating a public reference resource aiming to provide data for historic and medical research in a large understudied population. While most of the common variation is shared with other European populations, this survey of population variation contributes a number of novel SNPs and structural variants that have not been reported in the gnomAD/1KG databases representing global distribution of genomic variation. These endemic variants will become a valuable resource for designing future population and clinical studies, help address questions about ancestry and admixture, and will fill a missing place in the puzzle characterizing human population diversity in Eastern Europe. Our results indicate that genetic diversity of the Ukrainian population is uniquely shaped by the evolutionary and demographic forces, and cannot be ignored in the future genetic and biomedical studies. This data will contribute a wealth of new information bringing forth different risk and/or protective alleles. The newly discovered low frequency and local variants can be added to the current genotyping arrays for genome wide association studies, clinical trials, and in genome assessment of proliferating cancer cells.
**Corresponding Author:**	Taras K Oleksyk, Ph.D.<br>Oakland University<br>Rochester, MI UNITED STATES
**Corresponding Author Secondary Information:**	
**Corresponding Author's Institution:**	Oakland University
**Corresponding Author's Secondary Institution:**	
**First Author:**	Taras K Oleksyk, Ph.D.
**First Author Secondary Information:**	
**Order of Authors:**	Taras K Oleksyk, Ph.D.
	Walter W. Wolfsberger, M.S.
	Alexandra Weber
	Khrystyna Shchubelka, M.D.
	Olga T. Oleksyk, M.D., Ph.D.
	Olga Levchuk, Ph.D.
	Alla Patrus
	Nelya Lazar, M.D.
	Stephanie O. Castro-Marquez

Powered by Editorial Manager® and ProduXion Manager® from Aries Systems Corporation

	Patricia Boldyzhar, M.D.
	Mikhailo Neymet, M.D.
	Alina Urbanovych, M.D.
	Viktoriya Stakhovska, M.D.
	Kateryna Malyar
	Svitlana Chervyakova, M.D.
	Olena Podoroha, M.D.
	Natalia Kovalchuk
	Yaroslava Hasynets, Ph.D.
	Juan L. Rodriguez-Flores
	Sarah Medley
	Fabia U. Battistuzzi
	Siru Chen
	Ryan Liu
	Yong Hou, Ph.D.
	Huanming Yang, Ph.D.
	Meredith Yeager, Ph.D.
	Michael Dean, Ph.D.
	Ryan E. Mills, Ph.D.
	Volodymyr Smolanka, M.D., Ph.D.
**Order of Authors Secondary Information:**	
**Additional Information:**	
**Question**	**Response**
Are you submitting this manuscript to a special series or article collection?	No
**Experimental design and statistics**<br><br>Full details of the experimental design and statistical methods used should be given in the Methods section, as detailed in our Minimum Standards Reporting Checklist. Information essential to interpreting the data presented should be made available in the figure legends.<br><br>Have you included all the information requested in your manuscript?	Yes
**Resources**<br><br>A description of all resources used,	Yes

including antibodies, cell lines, animals and software tools, with enough information to allow them to be uniquely identified, should be included in the Methods section. Authors are strongly encouraged to cite Research Resource Identifiers (RRIDs) for antibodies, model organisms and tools, where possible.<br><br>Have you included the information requested as detailed in our Minimum Standards Reporting Checklist?	
**Availability of data and materials**<br><br>All datasets and code on which the conclusions of the paper rely must be either included in your submission or deposited in publicly available repositories (where available and ethically appropriate), referencing such data using a unique identifier in the references and in the "Availability of Data and Materials" section of your manuscript.<br><br>Have you have met the above requirement as detailed in our Minimum Standards Reporting Checklist?	Yes

Manuscript
To be Submitted: August 4, 2020
Click here to access/download;Manuscript;Ukrainian Genomes (TKO 2020).docx

OXFORD UNIVERSITY PRESS

(GIGA)n SCIENCE

DATA NOTE

Genome Diversity in Ukraine

Taras K. Oleksyk [1,2,3,*], Walter W. Wolfsberger [1,2,3#], Alexandra Weber [4#], Khrystyna Shchubelka [2,3,5#], Olga T. Oleksyk [6], Olga Levchuk [7], Alla Patrus [7], Nelya Lazar [7], Stephanie O. Castro-Marquez [2,3], Patricia Boldyzhar [5], Alina Urbanovych [8], Viktoriya Stakhovska [9], Kateryna Malyar [10], Svitlana Chervyakova [11], Olena Podoroha [12], Natalia Kovalchuk [13], Yaroslava Hasynets [2], Juan L. Rodriguez-Flores [14], Sarah Medley [2], Fabia Battistuzzi [2], Yuan Liu [15], Yong Hou [15], Siru Chen [15], Huanming Yang [15], Meredith Yeager [16], Michael Dean [16], Ryan E. Mills [17,*], and Volodymyr Smolanka [5]

[1] Department of Biological Sciences, Uzhhorod National University, Uzhhorod 88000, Ukraine;
[2] Department of Biological Sciences, Oakland University, Rochester, MI 48309, USA;
[3] Departamento de Biología, Universidad de Puerto Rico, Mayagüez, 00682, Puerto Rico;
[4] Department of Computational Medicine and Bioinformatics, University of Michigan, Ann Arbor, MI, 48109, USA;
[5] Department of Medicine, Uzhhorod National University, Uzhhorod 88000, Ukraine;
[6] A. Novak Transcarpathian Regional Clinical Hospital, Uzhhorod 88000, Ukraine;
[7] Astra Dia Inc., Uzhhorod 88000, Ukraine
[8] Lviv National Medical University, Lviv 79010, Ukraine
[9] Zhytomyr Regional Hospital, Zhytomyr 10002, Ukraine
[9] I.I.Mechnikov Dnipro Regional Clinical Hospital, Dnipro 49000, Ukraine
[11] Chernihiv Regional Hospital , Chernihiv 14039, Ukraine
[12] Sumy Diagnostic Center, Sumy 40000, Ukraine
[13] Rivne Regional Specialized Hospital of Radiation Protection, Rivne 33028, Ukraine
[14] Department of Genetic Medicine, Weill Cornell Medical College, New York, NY 10065, USA
[15] BGI, Shenzhen CHINA;
[16] Division of Cancer Epidemiology and Genetics, National Cancer Institute, Bethesda, MD 20892, USA;
[17] Department of Human Genetics, University of Michigan, Ann Arbor, MI, 48109, USA;

Corresponding address: Dr. Taras K. Oleksyk. E-mail: oleksyk@oakland.edu or Dr. Ryan Mills. E-mail: remills@umich.edu
[#] *these authors contributed equally*

Abstract

The main goal of this collaborative effort is to provide genome wide data for the previously underrepresented population in Eastern Europe, and to provide cross-validation of the data from genome sequences and genotypes of the same individuals acquired by different technologies. We collected 97 genome-grade DNA samples from consented individuals representing major regions of Ukraine that were consented for the public data release. DNBSEQ-G50 sequences, and genotypes by an Illumina GWAS chip were cross-validated on multiple samples, and additionally referenced to a sample that has been resequenced by Illumina NovaSeq6000 S4 at high coverage. The genome data has been searched for genomic variation represented in this population, and a number of variants have been reported: large structural variants, indels, CNVs, SNPs and microsatellites. This study is providing the largest to-date survey of genetic variation in Ukraine, creating a public reference resource aiming to provide data for historic and medical research in a large understudied population. While most of the common variation is shared with other European populations, this survey of population variation contributes a number of novel SNPs and structural variants that have not been reported in the gnomAD/1KG databases representing global distribution of genomic variation. These endemic variants will become a valuable resource for designing future population and clinical studies, help address questions about ancestry and admixture, and will fill a missing place in the puzzle characterizing human population diversity in Eastern Europe. Our results indicate that genetic diversity of the Ukrainian population is uniquely shaped by the evolutionary and demographic forces, and cannot be ignored in the future genetic and biomedical studies. This data will contribute a wealth of new information bringing forth different risk and/or protective alleles. The newly discovered low frequency and local variants can be added to the current genotyping arrays for genome wide association studies, clinical trials, and in genome assessment of proliferating cancer cells.

Keywords: genomes, NGS, genotyping, variant calling, copy number polymorphisms, SNP, CNV, indels, BGISeq, Illumina

---

Data Description

The context

Ukraine is the largest country located fully in Europe with a population that has been formed as a result of millennia of migration, and admixture of people. It arose in the intersection of the westernmost reach of the great steppe and the easternmost extent of the great mixed forests that spread across Europe, on the interception of the great trade route from "Variangians to the Greeks" along the river Dnipro, which the ancient Greeks referred to as Borysthenes, and the Silk Road linking old civilizations of the East to the rising powers of the West [1]. This land has seen the great human migrations of the Middle Ages sweeping from across the great plains of Eurasia, and even before that in the more distant past the early farmers [2] and the nomads who first domesticated the horse [3–6], and finally, at the dawn of the modern human expansion, here our ancestors have met the Neanderthals who used to hunt the great game along the glacier in the last Ice Age [7,8]. While the ethnic Ukrainians constitute approximately than three quarters of the total population of the modern Ukraine, the country is not ethnically uniform: a large minority of ethnic Russians compose one-fifth of the total population mostly in the southeast, and smaller ethnic groups with sizeable minorities in different parts of the country: Belarusians, Moldovans, Bulgarians, Poles, Jews, Hungarians, Romanians, Roma (Gypsies), and others [9]. As people have moved and settled across this land, they have contributed unique genetic variation that hasn't been captured by next generation sequencing and reported on the genomic scale.

Here we present and describe an annotated dataset of genome-wide variation in newly sequenced genomes from healthy adults sampled across the country representing the first genome wide evaluation of genetic diversity in self-identified Ukrainians from Ukraine in its current borders. Samples were successfully sequenced using BGI's DNBSEQ™ technology, and cross-validated by Illumina sequencing and genotyping. This data is offered to the scientific community to help fill the gaps in the map of the local genomic variation in Eastern Europe that has been largely omitted in the global genomic surveys [10]. Each volunteer participant in this study had an opportunity to review the informed consent, have been explained the nature of the genome data, and made a personal decision about making it public. One of the major objectives of this study was to demonstrate the importance of studying local genetic variation, especially in identifying unique and endemic determinants of human disease. However, of particular interest was contributing the medically related variants with frequencies that differed between the Ukrainians and the neighboring populations.

The dataset

The new dataset includes 97 whole genomes of self-reported Ukrainians from Ukraine at 30x coverage sequenced using DNBSEQ-G50 (formerly known as BGISEQ-500; BGI Inc., Shenzhen, China) and annotated for genomic variants: SNPs, indels, structural variants and mobile elements. The samples have been collected across the entire territory of Ukraine, after obtaining the IRB approval (Protocol #1 from 09/18/2018, **Supplementary File 1**) for the entire study design, and informed consent from each participating volunteer (**Supplementary File 2**). The majority of samples in this study (86 out of 97) were additionally genotyped using Illumina Global Screening Array array (Illumina Inc., San Diego, USA) in order to confirm the accuracy of base calling between the two platforms (**Supplementary File 3**). In addition, one sample (EG600036) was also sequenced on the Illumina

HiSeq (~60x coverage) and also used for validation of the variant calls (see summary in **Table S1,** and full sequencing statistics for individual samples in **Table S1.2**).

The Ukrainian Genome Diversity database contains locations and frequencies of more than 13M unique variants in Ukrainians from Ukraine which are further interrogated for functional impact and relevance to the medically related phenotypes (**Table 1, Supplementary Data 4**). As much as 3.7% of these alleles, or 478 K, are novel genomic SNPs that have never been previously registered in the gnomAD database [11] (**Table 1**). This number is similar in magnitude to what was reported earlier in two populations from European Russia (3-4%; [12]). Many of the discovered variants (12.6%) are also currently missing from the global survey of genomic diversity in the 1,000 Genomes Project [13]. Majority of these described variants are rare or very rare (<5%; **Figure S2**). Unless other indigenous ethnic groups from Ukraine (such as Crimean Tatars), would be included in the study, increasing the sample size above from 100 to 1,000 individuals is not likely to greatly contribute to discovery of novel mutations [14]. The proportion of the novel structural variants and mobile elements compared to the earlier databases is even higher: almost 1M (909,991) complex indels, regions of simultaneous deletions and insertions of DNA fragments of different sizes which lead to net a change in length, majority of which are novel (**Table 1**). Many of the newly discovered variants are functional and potentially contribute to the phenotype (classified in **Table 2).** We report many important variants that are overlooked or require special modifications in the commonly used resources and tools in genomic research and diagnostics. This wealth of novel variation underscores the importance of variant discovery in local populations that cannot be ignored in biomedical studies.

Table 1. Summary of variation in the 97 whole genome sequences from Ukraine.

**Sequencing results**	**All samples**			**On average**	
	**Total Unique Variants #**	**Novel gnomAD Count**	**% Novel gnomAD (1000Genomes)** [a]	**Average # /sample**	**Average # Novel /sample**
**Total sequence reads**	99.8 Bn	--	--	1.03 Bn	--
**Mean coverage**	97 samples at 30X each	--	--	30X	--
**Variation**					
**SNPs**	13,010,979	477,564	3.7%(12.6%)	3,488,083	0.1% (0.7%)
**Bi-allelic**	12,667,283	470,667	3.7%(12.7%)	3,340,557	0.3%(0.6%)
**Multi-allelic**	343,696	6,897	2.0%(7.4%)	146,340	0.8%(4.7%)
**Small Indels** [¥]	2,727,604	76,484	2.8%(7.4%)	917,731	0.3% (1.0%)
**Deletions**	1,805,739	55,599	3.1% (9.0%)	624,919	0.3% (2.4%)
**Insertions**	1,4459,87	30,453	2.1%(6.7%)	571,461	0.2% (2.1%)

Structural Variants [$]					
Large Deletions	16,078	10,914	67.9(48.3%)	3,524	52.6%(19.1%)
Large Duplications	1,845	1,356	73.5%(42.3%)	562	89.4%(35.2%)
Inversions	337	314	93.2% (47.8%)	185	94.1%(48.6%)
Mobile Element Insertions					
Alu	2,316	1805	77.9%(38.1%)	473	68.1%(18.0%)
L1	451	289	64%(50.1%)	79	60.8%(27.8%)
SVA	100	75	75%(52.0%)	20	70%(50%)
NUMT	714	--	--	16	--

₴ Defined as "percent not reported in gnomAD(1000Genomes)"

[¥] Small indels are insertions and deletions < 50bp called by GATK [15].

[$] Large deletions and duplications are those called by *lumpy* [16] which are > 50 bp.

Variant calling and confirmation

For each sample in the database, we estimated the number of passing bi-allelic SNPs calls (i.e. locuses with non-reference genotypes compared to the most current major human genome assembly, GRCh38 [17])(**Table 1**). Approximately 12% of these were filtered out based on excess heterozygosity and low variant quality scores (**Table S2**). Similarly for the indels, we also estimated the number of passing calls compared to GRCh38, and excluded 4% of those which did not pass filtering. The total number of unique SNPs, small and large indels (**Table 1**) was calculated from the alignments of raw reads from all the 97 sequenced genomes (**Total Unique SNPs, Table S2**) minus those filtered out for containing excess heterozygosity and showing low variant quality scores (**Filtered Count**; **Table S2**). In addition, 4,135,903 variants that only appeared once in a single sample (for both indels and SNPs) were designated as "*singletons*" and filtered out as well.

The resulting database was compared to the existing global databases of population variation from whole genome sequencing data such as Genome Aggregation Database (gnomAD)[11] and the 1000Genomes Project (1KG) database [13]. Under our search criteria, the small variants (SNPs and Small Indels) were considered "*novel*" if they were not present in any of the samples in the two global datasets (gnomAD/1KG; **Table 1**). The large structural variants and Mobile Element Insertions were considered novel if there were no variants in gnomAD/1KG within 25 base pairs whose length is within 5% of the size of the variant discovered in the Ukrainian population. We observed no significant deviation of the rate at which reference bases were observed at REF/alt heterozygous SNP sites (reference bias was near 50%).

We report a good correspondence between the SNP calls made using DNBSEQ and NovaSeq data. A comparison of the variants detected using these three platforms for sample EG600036 are summarized in **Figure 1.A**. The SNP concordance for samples with both DNBSEQ and SNP array data is summarized in **Figure 1C**. The cross-platform comparison shows a very good overlap across

all three technologies: with more than 3.5 M SNPs (or **97.7%**) of the SNPs identified in the the DNBSEQ were also verified in the whole genome sequence of EG600036 sequenced by the Illumina NovaSeq. The correspondence with the Illumina SNP Array for sample EG600036 was also very good: **95.8%** of all the SNPs genotypes called by the Illumina method were also detected by the DNBSEQ (**Figure 1.A(Right), C(Right)**). The concordance between the non-reference alleles between the two platforms in all the 86 samples is nearly linear (*r^2*=0.985, **Figure 1.C(Left)**).

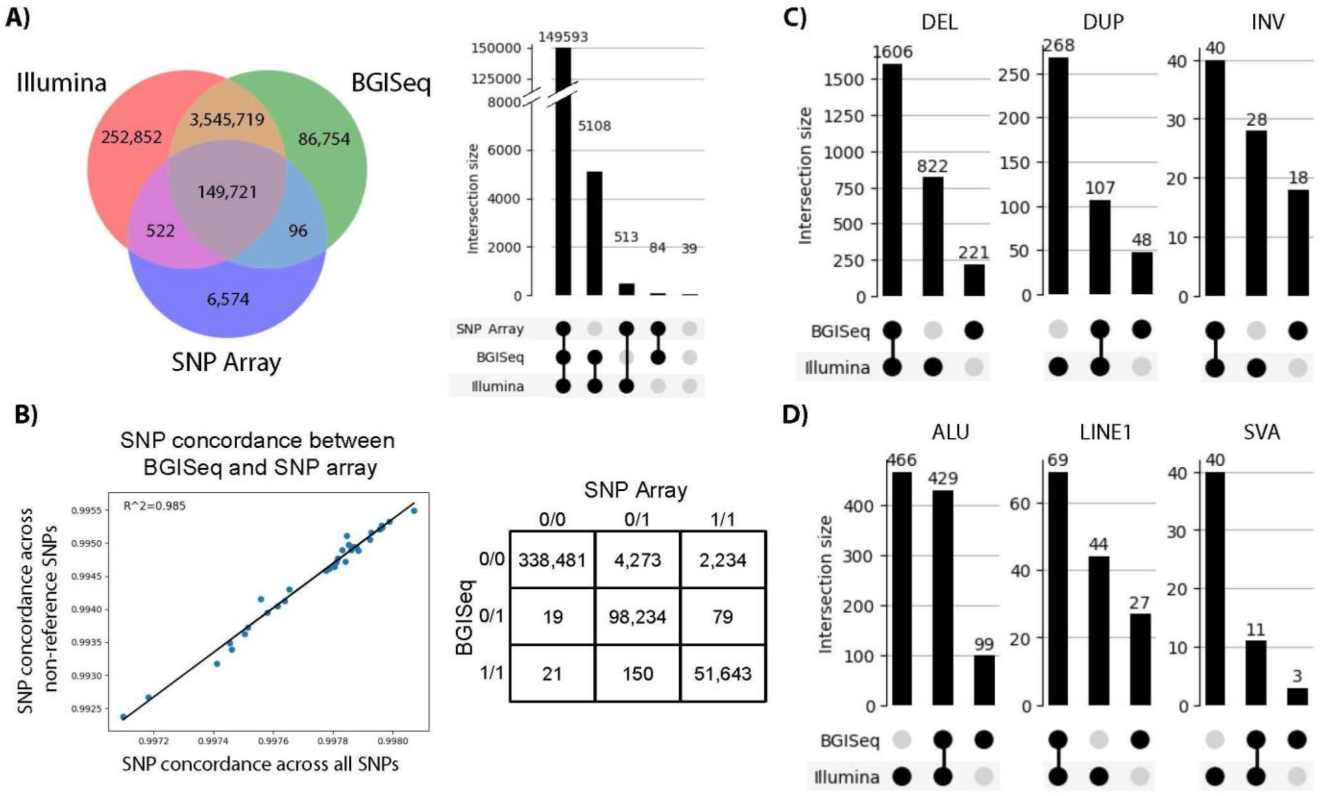

Figure 1. Variant concordance across the three sequencing/genotype methods **A) Left:** Overlap of SNP positions identified in one sample (EG600036) using each of the three platforms. **Right:** Concordance of SNP genotypes in one sample derived from each of the three platforms. This only includes the subset of SNPs with alternate alleles included in the Illumina genotyping array (the smallest of the three variant sets). The variants indicated as belonging to none of the categories are variants whose genotypes differ between all three platforms. **B) Left:** The percentage (%) of concordance between the Illumina SNP array and DNBSEQ for all SNPs compared to the % concordance of only SNPs with non-reference alleles in the Illumina SNP array for the 86 samples genotyped on both platforms. **Right:** Concordance of SNP genotypes between DNBSEQ and Illumina SNP Array for one sample (EG600036). **C)** Overlap within the numbers of the three major structural variants detected in one sample using the two whole genome sequencing datasets. **D)** Overlap within the numbers of the three major mobile element insertions detected in one sample using the two whole genome sequencing datasets.

Transition/Transversion ratio (or TITV ratio) for the novel SNPs (estimated with *TiTvtools* [18] and visualized by *plotTiTv* in **Figure S1**) was lower than the TITV ratio for SNPs in the dbSNPs database (**1.9 vs 2.2**; [19]). Since transversions are more likely to carry functional change, this finding supports the importance of this new dataset in providing the novel functional variation to the future research. Similarly, insertions to deletions (ins/del) ratio for novel indels is lower than for the indels already reported in the dbSNP database (**0.63 vs 0.75**). This observation likely reflects our improved ability

to detect small insertions in newer sequencing technologies compared to many platforms which historically submitted variation to dbSNP.

We have defined the multi-allelic SNPs as observations of genomic positions having two or more alternative alleles [20]. These are important variants that are overlooked or require special modifications in the commonly used resources and tools in genomic research and diagnostics. We report a total of 343,696 multiallelic sites in the sequences from our sample of which 2.0% are at locations unreported in the gnomAD database [11]. (**Table 1**).

In addition to the SNPs, we have identified and quantified major classes of structural variations in the Ukrainian population: small indels (insertions and deletions < 50bp), large structural variants (deletions, duplications and inversions > 50 bp) and Mobile Element Insertions (MEI)(Alu-s, L1 elements, non-autonomous retroelements (SVA), and nuclear mitochondrial DNA (NUMT) copies). A number of structural elements were reported, including common and novel ones. While among the small variants most were common (6-9%), a large proportion of large variants and MEIs (38-52%) have not been reported previously in the 1000Genomes Database (**Table 1**).

Once more, there is a significant correspondence between the calls made using BGI DNBSEQ and Illumina NovaSeq data. The two sequencing platforms show a significant overlap in calling indels (**DEL**): 87.9% of the variants called by the DNBSEQ were also detected by the Illumina. At the same time, there were 822 deletions, or 33.8% of all the indels called by the Illumina that were not detected by the DNBSEQ (**Figure 1.B**). A similar picture, where DNBSEQ performs competitively well, is also observed for inversions (**INV**)(**Figure 1.B**), and **LINE1** transposable elements (**Figure 1.D**). At the same time, more Duplications (**DUP**)(**Figure 1.B**), and the two classes of transposable elements evaluated: Alu elements (**ALU**) and the non-autonomous retroelements (**SVA**)(**Figure 1.D**). Evaluation tests show that current algorithms are platform dependent, in the sense that they exhibit their best performance for specific types of structural variation as well as for specific size ranges [21], and the algorithms designed for detection and archived datasets are predominantly for Illumina pair-end sequencing [22,23]. While it is possible that these results indicate Illumina's superiority at detecting structural variation, it also can also be the consequence of the bioinformatics tools for calling structural variants developed using mainly the Illumina data, as suggested by previous comparative evaluations of the two technologies [24,25].

Collection of functional variants

A particular interest in this study is the distribution of functional variation, not in the least due to the potential impact on phenotypes, especially to those with medical relevance [26]. As much as 97.5% of all annotated variation was discovered outside of the known functional elements (upstream, downstream, intron and intergenic). These results are similar to the expected distributions of mutations shown with the simulated data [27]. Nevertheless, there were more than 8 thousand mutations discovered within exons of each individual on average (**Table 2.A**). Within the coding regions we have annotated several classes of functional mutations (**Table 2.B**). Some of the mutations listed in the can be classified in more than one category (e.g. "*Synonymous variants*" can also be counted in "*Exonic variants*"). As expected, the nonsense mutations classified in the annotation file as "*Disruptive in-frame indel*", "*Start lost*", "*Stop gained*", and "*Stop loss*" were rare, while categories with minimal effect on the function, such as "*Synonymous*", "*Motiff*", "*Protein folding*", "*Missense*" were more common. In addition to the gene coding mutations, we report a number of regulatory variants. For example, the

database contains a total of 2,229 transcription factor binding site ablation (TFBS) mutations (**Table 2.B**). A summary of functional variation discovered in this study is presented in **Table 2.** The full list of high impact functional variants (including frameshift, start lost/stop lost or gained, transcript ablations and splice alterations) that had an allele count of two or more with their predicted function, number of gene transcripts of the gene affected, and frequencies is presented in **Table S3**. The full annotation database with classifications is available online as **Supplementary File 4** **(Link not currently available - it's a 11Gb VCF file).**

Table 2. Summary annotation of different genomic elements in the Ukrainian genomes annotated in BGISeq data from 97 Ukrainian samples

**A. Variants by Location**	**# of unique alleles ₴**	**Total allele #**	**Average /sample**
**Upstream**	2,023,920	6,716,794	69,246
**UTR 5 Prime**	31,026	122,417	1,263
**Exon**	320,979	839,045	8,650
**UTR 3 Prime**	150,302	389,528	4,016
**Downstream**	2,036,111	6,591,978	67,959
**Intergenic**	9,844,120	9,844,120	101,486
**Intron**	9,297,384	42,268,211	435,755
**Motif**	58,164	58,164	600
**B. Functional Variants by Type ₤**			
**Splice site acceptor**	1,105	3,844	40
**Splice site donor**	969	3,609	38
**Splice site region**	19,436	79,853	824
**Transcription factor binding site (TFBS) ablation**	2,229	2,229	23
**Conservative in-frame indels**	1544	2,475	26
**Gene Fusion**	98	1,482	16
**Disruptive in-frame indels**	978	4,093	43
**Missense**	61,181	169,454	1,747
**Start lost**	116	413	5
**Stop gained**	885	2,442	26
**Stop loss**	95	324	4
**Synonymous**	49,731	146,066	1,506
**Protein folding**	105,436	258,767	2,668

₴ Unique alleles represent mutations that were counted only once using the largest transcript, disregarding their frequency in the population

₤ Some of the mutations listed in the can be classified in more than one category

Collection of medically related variants

Many of the reported variants are already known to be medically related, and are listed either in Genome-wide association studies (GWAS) [28] or ClinVar (a NCBI archive of reports of the relationships among human variations and phenotypes with supporting evidence) [29] catalogues (**Table 3**). This database contains a total of 43,892 benign mutations in medically related genes, but also 189 unique pathogenic or likely pathogenic variants, as well as 20 protective or likely protective

alleles as defined in ClinVar [29,30]. While each of the individuals in this study was carrying 19 pathogenic and 12 protective mutations on average. While least some individuals were homozygous for the pathogenic allele, none of the associated disease phenotypes have been reported, which could be largely attributed to heterozygosity, age-dependent penetrance, expressivity and gene-by-environment interactions [31,32].

As expected, our study shared a lot more variants with the GWAS [28] than with the ClinVar [29] catalogue. However, while GWAS has recently become the tool of choice to identify genetic variants associated with complex disease and other phenotypes of interest [33], since the amount of genetic variance explained by these results is low, they are generally not very useful for prediction pathogenic phenotypes [34]. It is also important to note, that not all ClinVar variants carry the same weight of supporting evidence, attributing disease causation to prioritized variants remains an inexact process and some of the reported associations eventually are proven to be spurious [35]. Nevertheless, the importance of the unique set of mutations published here is difficult to overemphasize, as it constitutes the first published review of pathological variants in an understudied population, and will become an important step towards a local catalogue of medically relevant mutations that should be emphasized in personalized genomic tests for medical diagnostics in Ukraine. In addition, as the attention in the genomic community is shifting from monogenic to polygenic traits, many of these may become relevant in the future research and exploration [36]. Full list of the medically relevant functional markers found in the Ukrainian population and reported in GWAS [28] and ClinVar [29] databases. with alternative allele frequencies and annotations are presented in **Tables) S4**.

Table 3. Medically-relevant variants in the Ukrainian population included in GWAS [28] and ClinVar [29] databases

Source of Annotation	# Unique substitutions ₴	Total allele #	Average /sample
**GWAS catalog**	102551	6,479,953	66804
**ClinVar:** pathogenic (or likely pathogenic)	189	1,830	19
**ClinVar:** benign (or likely benign)	43,892	1,842,668	18997
**ClinVar:** protective (or likely protective)	20	1,209	12

₴ Unique variants represent substitutions that were counted only once, disregarding their frequency in the population

Disease variants with frequencies that differed between the Ukrainians and the neighboring populations are of particular interest to the medical community. It is well established that differences in allele frequencies are a consequence of evolutionary forces acting in populations (such as drift, mutation, migration, nonrandom mating and natural selection), the certain diseases and heritable traits display marked differences in frequency between populations [37]. With this in mind, we created a list of the known disease variants that whose frequencies differ between Ukrainians and other European populations (the combined European sample (EUR) from the 1000Genomes Project (Utah Residents (CEPH) with Northern and Western European Ancestry, Toscani in Italy (TSI), Finnish in Finland (FIN) British in England and Scotland (GBR), and Iberian Population in Spain (IBS)); [13,38]) and Russians from Russia (RUS)(Novgorod and Pskov; [12]). Several examples of these variants are presented in **Table 4**. Among these are variants involved in a number of medical conditions such as hyperglycinuria/iminoglycinuria (*rs35329108; SLC6A19*), bisphosphonates (*rs2297480; FDPS*),

warfarin (*rs7089580; CYP2C9*), and cisplatin (*rs2228001; XPC*) response/toxicity, longevity and diabetes type II susceptibility (rs1805097; *IRS2*), risk of asthma and lung function (*rs4950928; CHI3L1*)(**Table 4**).

Table 4. Examples of the functional SNPs with highly differentiating functional markers reported in ClinVar [29], with high differences in the Ukraininan population compared to the neighboring populations in other European populations (the combined sample from Western and Central Europe from 1000Genomes Project (EUR); [13,38] and Russians from Russia (RUS;) [12]. Non-reference allele frequency (NAF) is reported compared to the reference allele in GRCh38. Differences are evaluated by the Fisher Exact Test (FET). All the functional SNPs with significant population frequency differences are listed in **Table S5**.

SNP	Chr	Gene	REF/ alt ᵽ	Associated medical condition	Function	NAF UKR	NAF EUR	NAF RUS	FET vs. EUR (p-value)	FET vs. RUS (p-value)
rs35329108	*5*	*SLC6A19*	g/A	Hyperglycinuria | Iminoglycinuria	Pathogenic, Intronic	0.32	0.23	0.20	*0.004*	*0.044*
rs2297480	*1*	*FDPS*	t/G	Bisphosphonates response	Drug response, intronic	0.13	0.26	0.26	*5.36×10^{-5}*	*0.015*
rs7089580	*10*	*CYP2C9*	a/T	Warfarin response	Drug response, intronic	0.31	0.22	0.19	*0.006*	*0.04*
rs2228001	*3*	*XPC*	G/t	Cisplatin response-toxicity	Drug response, exonic, nonsynonymous	0.51	0.66	0.60	*0.04*	*0.03*
rs1805097	*13*	*IRS2*	c/T	Longevity, Diabetes type II susceptibility	Risk factor, exonic, nonsynonymous	0.39	0.31	0.26	*0.04*	*0.04*
rs4950928	*1*	*CHI3L1*	G/c	Risk of asthma and lung function	Risk factor, upstream	0.75	0.82	0.62	*0.03*	*0.03*

ᵽ The reference allele is set according to the reference allele in GrCH38.p13 [17].

Of course, not all the medically related variants are currently known, and many remain to be discovered and verified in local populations. This is, to some extent, a consequence underreporting of allelic endemism within understudied populations, particularly in Eastern Europe [10] but also elsewhere [39,40]. By offering public annotations of functional mutations in a population sampled across the territory of Ukraine, our database contributes a number of candidates to direct future research in medical genomics. We chose only the **markers with the highest non-reference allele frequency (NAF) differences** compared to the neighboring populations: the combined population from Europe (EUR; [13]) and Russians from Russia (RUS; [12]), evaluated by the Fisher Exact Test (FET) and listed them **Table 5**.

Table 5. Examples of the functional markers with the highest **non-reference allele frequency (NAF) differences** in the Ukraininan population evaluated by the Fisher Exact Test (**FET**) compared to the frequencies in the neighboring populations: the combined population from Europe (**EUR**; [13]) and Russians from Russia (**RUS**; [12]).

SNP	Chr	Gene	Ref/ Alt	Function	NAF UKR	NAF EUR	NAF RUS	FET vs. CEU (p-value)	FET vs. RUS (p-value)
rs3753045	20	*CDH4*	C/T	Exonic, synonymous	0.01	0.25	0.20	2.90E-06	9.89E-08
rs3975155	11	*OR9G1; OR9G9*	T/G	Exonic, nonsynonymous	0.97	0.59	0.70	1.95E-06	2.06E-07
rs2634041	12	*KRT2*	T/C	Exonic, nonsynonymous	0.03	0.28	0.33	2.02E-06	6.68E-07
rs13090	19	*MED16*	C/T	Exonic, nonsynonymous	0.03	0.28	0.27	1.72E-06	7.25E-07
rs11568188	22	*MLC1*	T/C	Exonic, nonsynonymous	0.006	0.13	0.17	1.60E-06	9.35E-07
rs536024292	17	*SMARCD2*	G/C	Exonic, nonsynonymous	0.35	0.001	0.05	1.27E-06	1.04E-06

Population structure and ancestry informative markers

We performed several population analyses, but only to demonstrate the uniqueness and usefulness of this new dataset. Therefore, we do not evaluate any historical hypotheses on the timing of origins, founding, migration and admixture of this population, and use only the naive approaches, choosing models based on the statistical models.

To demonstrate the extent to which our dataset contributes to the genetic map of Europe, we explored genetic relationships between Ukrainian individuals within our sample and evaluated genetic differences between this population and its immediate neighbors on the European continent for which population data of full genome sequences was publicly available . A Principal Component Analysis (PCA) of the merged dataset of 654 samples included European populations from the 1000Genomes Project (Utah Residents (CEU) with Northern and Western European Ancestry, Toscani in Italy (TSI), Finnish in Finland (FIN) British in England and Scotland (GBR), and Iberian Population in Spain (IBS)); [13,38]) and Russians from Russia (RUS)(Novgorod and Pskov; [12] as well as the relevant high-coverage human genomes from the Estonian Biocentre Human Genome Diversity Panel (EGDP: Croatians (CRO), Estonians (EST), Germans (GER), Moldovans (MOL), Polish (POL), and Ukrainians (UKR)[44], and Simmons Genome Diversity project (Czechs (CZ), Estonians (EST), French (FRA), Greeks (GRE), and Polish (POL) [45] (**Figure 2**). The latter paper also identifies "Cossacs" as a separate group within Russians (Cossacs (RUS) or Ukrainians (Cossacs (UKR)) [45] (**Supplementary File 5**).

Our results indicate that genetic diversity of the Ukrainian population is uniquely shaped by the evolutionary and demographic forces, and cannot be ignored in the future genetic studies. Ukrainan genomes from this and other studies [44,45] form a single cluster (**black shapes**) positioned

between the Northern and Eastern Europeans (**green and purple**; Estonians and Russians From Russia) on one side and Western European populations (**blue**; CEU, French, British and Germans) on the other **Figure 2**). There was a significant overlap with the other Central and Eastern European populations, such as Czechs and Polish (**red**), and the people from the Balkans (Croats, Greeks and Moldovans; **gold**). This is not surprising, in addition to the close geographic distance between these populations, this may also reflect the insufficient representation of samples from the surrounding populations (see **Supplementary Data 5**). Similarly, the admixture analysis demonstrates distinctiveness of our dataset, but also demonstrates unique combinations of genetic components that may have shaped this population (**Figure 3** and **Figure S3**).

Addition of the new genomic data will most likely add to the resolution of the genetic map this region and further reveal differences between the populations of Eastern and Central Europe. Meanwhile, our dataset showed a limited amount of inbreeding (**Figure S4**) and contains information for future population studies. A list of all the variants with significant difference in frequencies between Ukrainians and other European populations are listed in **Table S6**. This database can be a starting point for association studies, as ancestry informative markers (AIMs)[41], and to be used for mapping disease alleles by admixture disequilibrium [42,43].

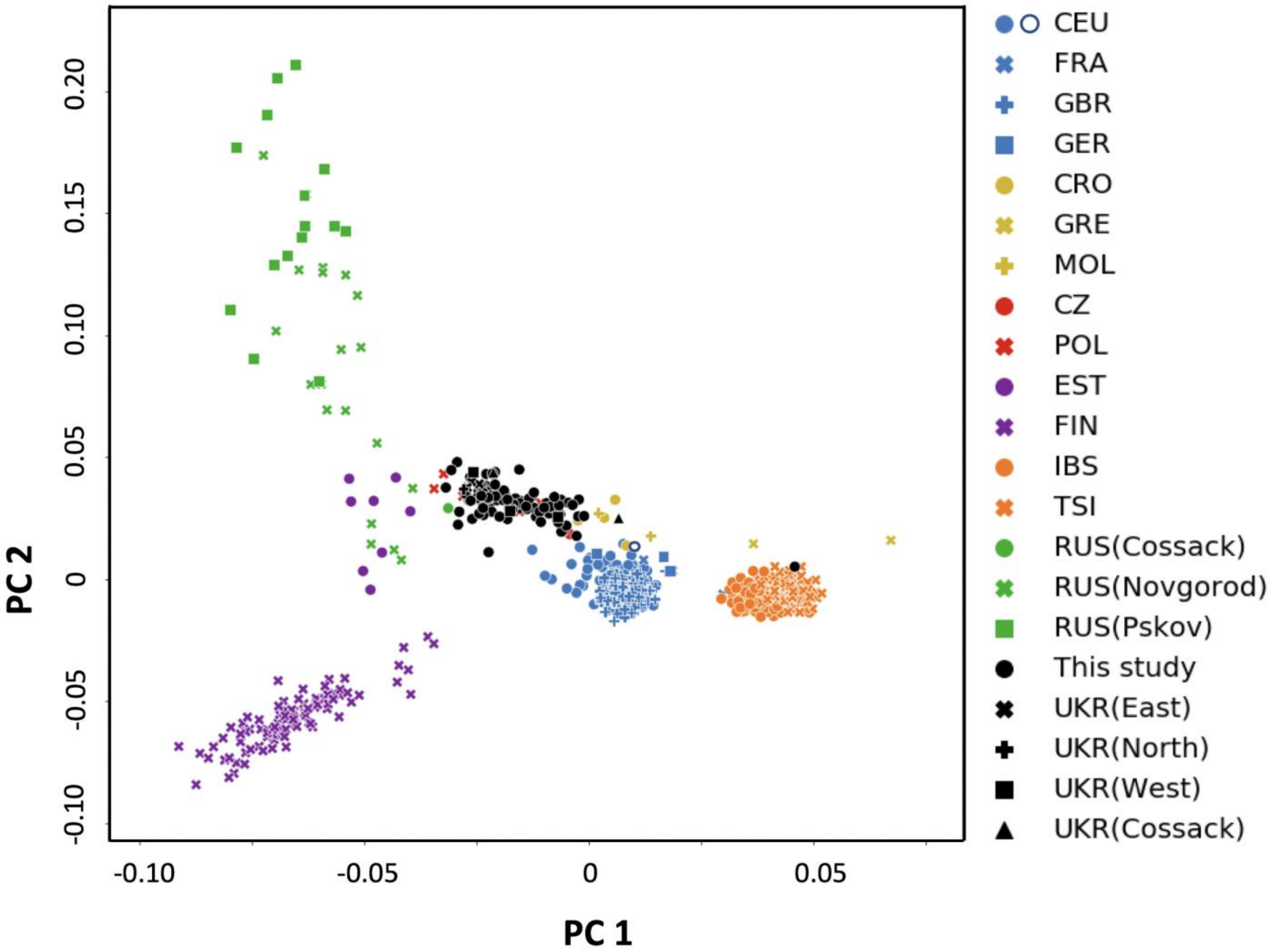

Figure 2. The Principal Component (PC) analysis of genetic merged dataset, containing European populations. Colors reflect prior population assignments from the European samples from the 1000Genomes Project (Utah Residents (CEPH) with Northern and Western European Ancestry, Toscani in Italy (TSI), Finnish in Finland (FIN), British in England and Scotland (GBR), and Iberian Population in Spain (IBS)); [13,38]) and Russians from Russia (RUS)(Novgorod and Pskov; [12] as well as the relevant high-coverage human genomes from the Estonian Biocentre Human Genome Diversity Panel (EGDP (EST; [44], and Simmons Genome Diversity project [45]. The analysis was performed with Eigensoft [46]. The hollow blue circle represents the internal positive CEU control.

To provide a more extended view of the genetic components contributing to the Ukrainian population, we used the population structure plots using the ADMIXTURE package [47]. This allowed us to construct a preliminary picture of putative ancestry contributions and population admixture. In order to identify the optimal K, we implied the 10-fold cross-validation function in range from K=2 to 8. The results with the optimal K=3 shown in **Figure 3** illustrate similarity and the difference of Ukrainian population compared to the other populations in Central and Eastern Europe (**Figure 3, second row**). While the higher values of K (K=3-8; **Figure S3**) show an increasing number of clusters, they also show an increasing amount of error in the cross validation function. This analysis already shows the potential of the current database in helping to resolve population structure in Eatern Europe, but additional genome wide data from neighboring populations would be very helpful to refine the picture in this geographical region.

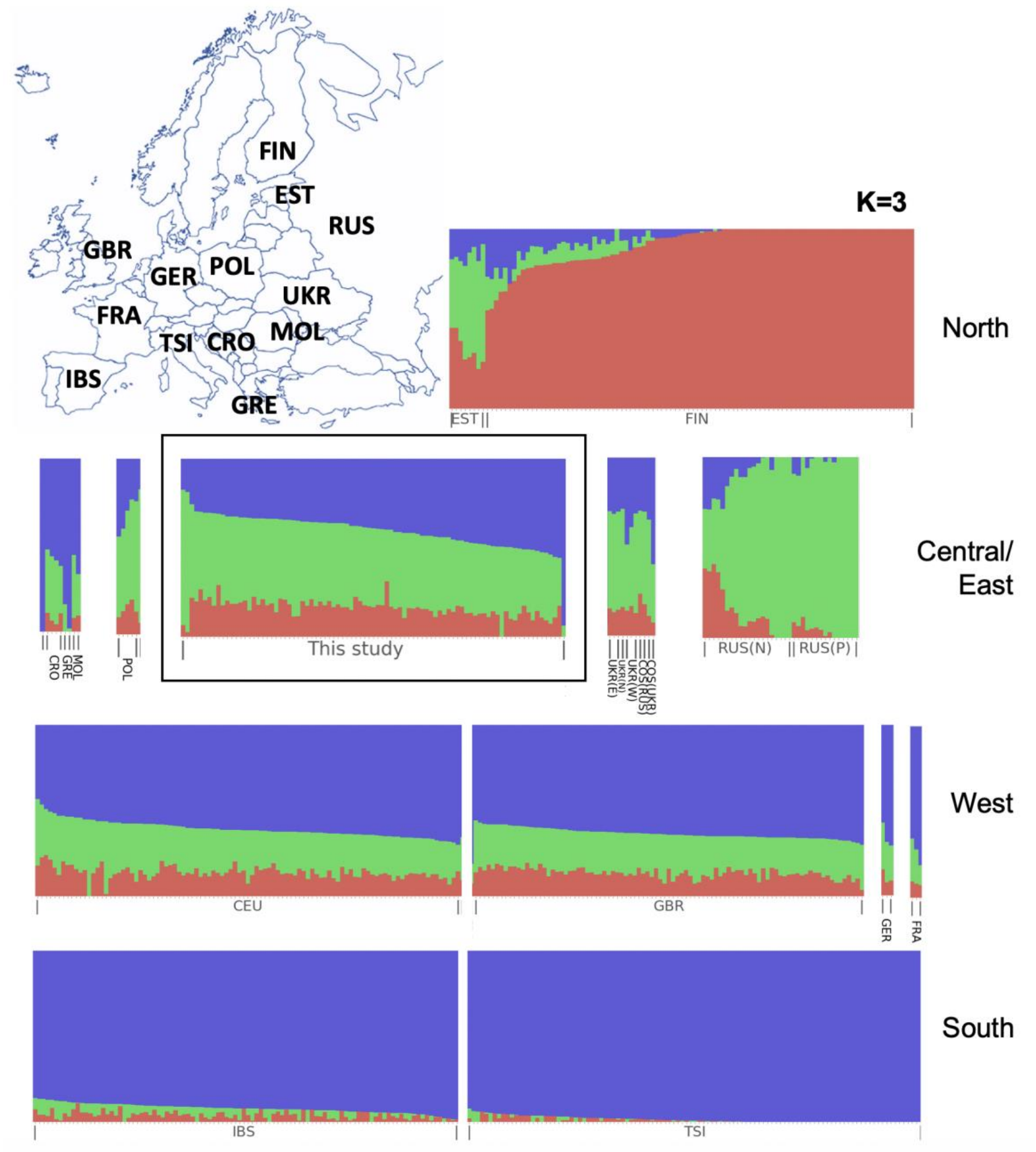

Figure 3. Genetic structure of Ukrainian population in comparison to other European populations. Structure plot constructed ADMIXTURE package [47] at K=3 illustrates similarity and differences between genomes from this study as well as samples from the 1000Genomes Project (Utah Residents (CEU) with Northern and Western European Ancestry, Toscani in Italy (TSI), Finnish in Finland (FIN), British in England and Scotland (GBR), and Iberian Population in Spain (IBS)); [13,38]) and Russians from Russia (RUS)(Novgorod and Pskov; [12] as well as the relevant high-coverage human genomes from the Estonian Biocentre Human Genome Diversity Panel (EGDP (EST; [44], and Simmons Genome Diversity project [45]. For identification of the optimal K parameter, we evaluated a range from 2 to 8, with K=3 resulting in the lowest error. Plots with K=4 to K=8 are presented in **Figure S3**.

Despite the fact that all of the samples were collected from self-identified ethnic Ukrainians, there were two notable outliers. Sample EG600048 that clustered with the Southern Europeans (Iberia and Italian populations), and EG6000xx clustered with the Western Europeans (CEU, French, British and Germans) (**Figure 2**). This illustrates an important point that while ignoring the unique composition of this population will result in ascertainment bias in biomedical studies, genetics is not a reliable determinant of ethnicity. In anticipating the future ancestry studies we contribute the full list of candidates for Ancestry Informative Markers differentiating Ukrainians with their neighboring populations in Europe (**Table S6**).

People of Ukraine carry many previously known and several novel genetic variants with clinical and functional importance that in many cases show allele frequencies different from neighboring populations in the rest of Europe, including Poland to the West, Romania to the South, the Baltics to the north and Russia to the northeast. While several large genome projects already exists contributing to the understanding of the global genetic variation, many of the rare and endemic alleles that have not been yet identified by the international databases such as the 1,000 Genomes project, and currently not available in standard genotyping panels for association testing for human diseases, and glaring white spots still exists on the genetic maps in local populations of Eastern Europe [10]. We fully expect that the future sampling and sequencing will continue to improve and complete the detailed picture of genomic diversity in people across the country and contribute to the further development of genetic approaches in biomedical research and applications.

Methods

a) Sampling strategy

The collection procedure was approved as part of the "*Genome Diversity in Ukraine* " project by the Institutional Review Board (IRB) of Uzhhorod National University in Uzhhorod, Ukraine (Protocol #1 from 09/18/2018, Supplementary File 1). We employed doctors and medical professionals from different regions of Ukraine to oversee collection of blood samples at hospitals. Healthy (non-hospitalized) volunteers were contacted through advertisements, and invited for personal interviews at outpatient offices. During the visit the volunteers were familiarized with the study and the collection procedure, and gave full consent to participate and have their genotypic and phenotypic data to be freely and publicly available. During each interview, the volunteer participants also completed a questionnaire indicating self-reported region of origin, place of birth of both grandparents (if remembered), sex and several phenotypical features, such as daily history of disease (**Supplementary File 3**). The hard copies of the consents and personal interviews remain sealed and stored at the Biology Department of Uzhhorod National University. After the conclusion of the interview and sample collection, all personal identifiers were removed from the vials containing blood samples, except for an alphanumeric identifier and a barcode. All the subsequent analysis and publication was done in a blind design where neither the participants nor the researchers could identify the person who donated the sample.

At the conclusion of the interview a whole blood sample was collected from a vein into two a 5 ml EDTA tubes by a certified nurse or a phlebotomist, assigned a barcode number, and shipped by courier on dry ice to a biomedical laboratory certified to handle blood samples in Uzhhorod, Ukraine (**Astra Dia Inc**.) for DNA extraction immediately on arrival. The excess of the blood and DNA from samples remaining after the genetic analysis is stored frozen at the biobank of the Biology Department, Uzhhorod National University, Ukraine. As a result, blood samples were collected from a total 113 individuals.

b) DNA extraction

Immediately upon arrival to the laboratory, DNA isolation from 200 uL of blood was attempted with innuPREP DNA Blood Minikit (Analitik Gena, Germany). High molecular weight genomic DNA was lightly fragmented by vortexing. The initial DNA concentration was measured with the Implen C40 Nanophotometer (München, Germany), and quality was verified visually on a 2% agarose gel. The 97 successfully extracted DNA samples were normalized to 20-30 ng/µl concentration for downstream application. After the extraction the samples were re-coded and sent to NIH for genotyping procedure, from where the aliquots were further shipped to BGI facility (BGI Shenzhen, CHINA) or to Psomagen Inc. (Gaithersburg, MD, USA) for the whole genome sequencing (WGS). The remaining ~2 ml was frozen for future use.

c) Sequencing and Genotyping

All the 97 individuals in this study were sequenced with DNBSEQ-G50 and 88 individuals were cross validated by genotyping using Illumina Global Screening Array. The record of which individual samples have been cross-validated by both technologies is presented in **Table S2**. In addition, a single sample (EG600036) was also sequenced on Illumina HiSeq (~60x coverage)..

Sequencing with BGI DNBSEQ-G50

All 97 DNA samples were sequenced on DNBSEQ-G50 (BGI Shenzhen, CHINA). Upon the receipt at the BGI facility, and prior to sequencing, samples were checked again for quality. Concentration was once more detected by fluorometer or Microplate Reader (e.g. Qubit Fluorometer, Invitrogen). Sample integrity and purity were detected by Agarose Gel Electrophoresis (Concentration of Agarose Gel:1% Voltage:150 V, Electrophoresis Time:40 min). 1μg genomic DNA was aliquoted and fragmented by Covaris. The fragmented genomic DNA was selected by Agencourt AMPure XP-Medium kit to an average size of 200-400bp. Fragments were end repaired and then 3' adenylated. Adaptors were ligated to the ends of these 3' adenylated fragments. PCR products were purified by the Agencourt AMPure XP-Medium kit. The double stranded PCR products were heat denatured and circularized by the splint oligo sequence. The single strand circle DNA (ssCir DNA) was formatted as the final library. The qualified libraries were sequenced by DNBSEQ-G50: ssCir DNA molecule formed a DNA nanoball (DNB) containing more than 300 copies through a rolling-cycle replication. The DNBs were loaded into the patterned nanoarray by using high density DNA nanochip technology. Finally, pair- end 100 bp reads were obtained by combinatorial Probe-Anchor Synthesis (cPAS). Raw reads were filtered removing adaptor sequences, contamination and low-quality reads. Sequencing of all the 97 full genome samples submitted for sequencing at BGI was successful.

Short Read Sequencing with Illumina NovaSeek6000

one individual was resequenced by Illumina NovaSeq6000 S4 at Psomagen Inc. (Gaithersburg, MD, USA). Library was prepared using TruSeq DNA PCR Free 350bp protocol by Illumina. The library was sequenced at approximately 64X depth, producing 150bp-long reads, resulting in 241.7G bp of data.

Genotyping with the Illumina Infinium Global Screening Array

We attempted to genotype all 97 of the collected samples using the Illumina Infinium Global Screening BeadChip Array-24 v1.0 (GSAMD-24v1-0) for 700,078 loci at the NCI's DCEG (Bethesda, MD; https://grcf.jhmi.edu/wp-content/uploads/2017/12/infinium-commercial-gsa-data-sheet-370-2016-016.pdf). Data was analyzed by using the standard Illumina microarray data analysis workflow. During QC, samples were filtered for contamination, completion rate, and relatedness. As part of QC, we performed ancestry assessment using SNPweights software [41] with a reference panel consisting of 3 populations (European, West African, and East Asian). All samples were attributed to the European ancestry group. After OC and sample exclusion, 87 (86 samples and 1 QC) samples with 689,918 loci and completion rate of 99.9 were retained for further analysis.

d). Variant Calling

Variant Calling of the BGISeq500 data

The sequencing data produced using the DNBSEQ platform for 97 samples were analyzed using the Sention tools (Sentieon Inc, San Jose, CA, USA) high-performance implementation of the BWA/GATK best practices pipeline on servers hosted by the Cornell University Biotechnology Resource Center. Reads were aligned to the GRCh38 human reference genome using BWA-MEM (Version: 0.7.16a-r1181), and mapped reads were prepared for variant calling using Genome Analysis

Toolkit (GATK) v3.8-1-0-gf15c1c3ef by Broad), including marking duplicates (picard MarkDuplicates, Version 2.12.1), indel realignment (GATK RealignerTargetCreator, IndelRealigner, Version 3.7-0), and base quality score recalibration (GATK BaseRecalibrator, PrintReads, Version 3.7-0). SNP and Indel discovery were performed for each individual using GATK HaplotypeCaller, and merged into a single pVCF using *bcftools*. Sample EG600036 was also run without joint calling which was used when calculating concordance between the Illumina and BGISeq variant callsets. estimated with *TiTvtools* and visualized by *plotTiTv* [18].

Repetitive variant calling

Mobile element discovery was performed using MELT (Version 2.2.0) [48] and structural variant discovery using *lumpy-sv* with *Smoove* (Version: 0.2.5) [16]. Short tandem repeats were called using *GangSTR* (Version: 2.4.2) [49] and nuclear mitochondrial DNA using *dinumt [50]*.

e) Data validation and quality control

Variant files were compared for consistency across the three different platforms: BGI DNBSEQ-G50 sequencing, Illumina genotyping, and Illumina ovaSeq6000 sequencing. Illumina genotyping was performed on 86 of the 97 samples previously sequenced with DNBSEQ-G50. Additionally, one sample (EG600036) was also sequenced with Illumina NovaSeq6000 S4. The variant detection programs were re-run without joint calling for the DNBSEQ-G50 sequencing for sample EG600036 for comparison with the single Illumina sequenced sample. In this sample, the SNPs derived from the WGS platforms were compared to those identified using the Illumina SNP array both for matching position and matching genotype. Structural variants and mobile element insertions were compared between the WGS platforms in EG600036. Variants were considered the same if they had 95% reciprocal overlap. Overall, we found Illumina identified a higher number of larger variants than DNBSEQ-G50. This could potentially be due to its higher coverage (~60X) compared to DNBSEQ-G50 (~30X). However, as both have high coverage, we may see diminishing returns for coverage over 30X. An alternative explanation is that the variant identification tools have been built to detect variation from Illumina sequencing data and therefore, may not be able detect variants DNBSEQ-G50 as accurately.

f) Annotation

Sequence variant files were annotated using *ANNOVAR* [51] and *SNPEff* [52] software using GRCh38 reference databases. The following databases were used for the For ANNOVAR annotations: RefSeq Gene, 1000 genomes superpopulation, dbSNP150 with allelic splitting and left-normalization. For annotation of the medically related and functional variants we used ClinVar version 20200316 [29], InterVar gnomeAd ver 3.0 [11], and *dbnsfp ver. 35c [53]*. For *SNPEff*, the default GRCh38 annotation database [54] was complemented with ClinVar [29] and GWAS catalog [28] database annotation using *snpSift* tool [55].

g) Population analysis

Principal Component analysis (PCA)

For principal component analysis, we used WGS variants of our samples and merged then with samples from neighbouring countries available from the European samples from the 1000Genomes

Project (Utah Residents (CEPH) with Northern and Western European Ancestry, Toscani in Italy (TSI), Finnish in Finland (FIN), British in England and Scotland (GBR), and Iberian Population in Spain (IBS)); [13,38]) and Russians from Russia (RUS)(Novgorod and Pskov; [12] as well as the relevant high-coverage human genomes from the Estonian Biocentre Human Genome Diversity Panel (EGDP (EST; [44], and the Simmons Genome Diversity project [45]. The analysis was performed with Eigensoft [46].

The resulting dataset was filtered by genotyping rate (> 0.9) and pruned for variants in LD by excluding those with high pairwise correlation within a moving window(*--indep-pairwise* 50 10 0.2). This resulted in 654 samples with 126,954 variants. We used *EIGENSOFT* [46] to calculate the eigenvectors, of which, PC1 and PC2 were visualized using Python programming language, with *pandas, matplotlib and seaborn* libraries [56] (**Figure 2 and S2**).

Model-based population structure analysis

For the naive (model-based) structure analysis, we used the same dataset described in the Principal Component Analysis (above). The analysis was performed using *ADMIXTURE* software [47]. For identification of the optimal K parameter, we used the 10-fold cross-validation function of *ADMIXTURE* in range from 2 to 8, with K=3 resulting in the lowest error, deeming it optimal. The results were visualized using Python programming language, with *pandas*, *matplotlib* and *seaborn* libraries [56,57] to construct a population structure plot using samples from the 1000Genomes Project (Utah Residents (CEU) with Northern and Western European Ancestry, Toscani in Italy (TSI), Finnish in Finland (FIN), British in England and Scotland (GBR), and Iberian Population in Spain (IBS)); [13,38]) and Russians from Russia (RUS)(Novgorod and Pskov; [12] as well as the relevant high-coverage human genomes from the Estonian Biocentre Human Genome Diversity Panel (EGDP (EST; [44], and Simmons Genome Diversity project [45]. The resulting plot with K=3 is presented in **Figure 3**, and plots with K=4 to K=8 are in the **Figure S3**.

Inbreeding estimates

We estimated inbreeding coefficients for all the genotype samples in the same dataset. For this analysis the samples were pruned for genotyping rate (>0.9) and linkage disequilibrium by excluding those with high pairwise correlation within a moving window (plink parameter*--indep-pairwise* 50 10 0.1). Using the resulting dataset containing the remaining 117,641 loci from 84 samples, we performed several inbreeding estimates: (a) method-of-moments F-coefficient estimates, (b) variance-standardized relationship minus 1 estimates, and (c) F-estimates based on correlation between uniting gametes [58]. All the resulting values are presented in **Table S7**, and the estimates for the of method method-of-moments F-coefficient estimates are visualized in a histogram (**Figure S4**).

Re-use potential

Since the publication of the first human genome [59,60] , and the first surveys of worldwide variation such as the 1,000 Genomes project [13,38], the efforts have been directed to expand outwards by expanding the exploration of the human diversity across the world, and filling out more and more "white spots" of genome variation [12,45], as well as inward, to fill the remaining white spots in the human genome itself: to map the remaining gaps in the chromosome assembly and identify new structural and functional variation [61] and to map the three dimensional structure of the human

genome [62]. The new data presents a valuable addition to the former and represents the first exploration of the genome landscape in the important component of European genomic diversity.

Genome diversity of Ukraine is an important puzzle to help modern genome studies of population history of Europe. The country is positioned in the crossroad of the early migration of modern humans and the westward expansion of the Indo-Europeans, and represents an aftermath of centuries of migration, admixture, demographic and selective processes. As wave after wave of great human migrations moved across this land for millennia, they were followed by exchange of cultural knowledge and technology along the great trade routes that transect this territory until this day.

The justifications for collecting, sequencing and analyzing populations from this part of Europe has been outlined earlier [10,63], and the new database is a step into that direction. Given its unique history, the genome diversity data from Ukraine will contribute a wealth of new information bringing forth different risk and/or protective alleles that do not exist nor associate with disease, elsewhere in the world. This project identified 13M variants in Ukrainians of which 478 K were novel genomic SNPs currently missing from the global surveys of genomic diversity [11][13]. We also report almost 1M (909,991) complex indels, regions of simultaneous deletions and insertions of DNA fragments of different sizes which lead to net a change in length, with only 713,858 previously reported in gnomAD [11] (**Table 1**). The newly discovered local variants can be used to augment the current genotyping arrays and used to screen individuals with genetic disorders in genome wide association studies (GWAS), in clinical trials, and in genome assessment of proliferating cancer cells.

The current project is built upon the open release/access philosophy. The data has been released and can be used to search from population ancestry markers and well as the medically related variants in the subsequent studies. The public nature of the data deposited on the specially created web resource located at Uzhhorod National University, will ensure that the biomedical researchers in the country will receive access to a useful information resource for future projects in genomics, bioinformatics and personalized medicine. Engaging local Ukrainian scientists in this collaborative international project like building the foundation for the future studies and ensuring their participation in the worldwide research community.

Availability of source code and requirements

Availability of the Supporting Data

The raw reads are available at the ENA (accession # ————————). Raw sequencing data, genotypes and the assembly parameters have been submitted to the GenBank (accession # ————————), and all data, including FASTA files of the assembled genomes,, corresponding assembly parameters, and annotation data are available in GigaDB (reference). The links to all the supplementary tables and databases are listed in (Additional files 2, 3, ... k-1 and k) and can also be accessed at GigaDB (http://------------------).

List of Supplementary Tables

Table S1. Sequencing summaries of output from DNBSEQ-G50 and Illumina NovaSeq6000. Full sequencing statistics for individual samples in **Table S1.2**

Table S2. Filtering summary of the data obtained from 97 whole genomes sequenced with DNBSeq-G50.

Table S3. The full list of high impact functional variants (including frameshift, start lost/stop lost or gained, transcript ablations and splice alterations) that had an allele count of two or more with their predicted function, number of gene transcripts of the gene affected, and frequencies.

Table S4. List of the medically relevant functional markers found in the Ukrainian population and reported in **A.** **GWAS catalog** [28] and B. **ClinVar** [29] databases. Allele frequency is reported compared to the reference allele in GRCh38 .

Table S5. Complete list of the highly differentiating markers, reported in ClinVar [29], with high differences in the Ukrainian population compared to the neighboring populations in other European populations (the combined sample from Western and Central Europe from 1000Genomes Project (EUR); [13,38] and Russians from Russia (RUS; [12]. Non-reference allele frequency (NAF) is reported compared to the reference allele in GRCh38. Differences are evaluated by the Fisher Exact Test (FET).

Table S6. A list of markers with the highest non-reference allele frequency (NAF) differences in the Ukrainian population evaluated by the Fisher Exact Test (FET) compared to the frequencies in the neighboring populations: the combined population from Europe (EUR; [13]) and Russians from Russia (RUS; [12]). This database contains candidate ancestry informative markers (or AIMs)[41], that can be used for mapping disease alleles by admixture disequilibrium [42,43].

Table S7. Inbreeding estimates in a dataset of 117,641 loci from 84 samples: (a) method-of-moments F-coefficient estimates, (b) variance-standardized relationship minus 1 estimates, and (c) F-estimates based on correlation between uniting gametes [58].

List of Supplementary Files

Supplementary File 1. IRB approval of the study "Genomic Diversity of Ukraine's Population" (*in Ukrainian*). Supplementary File 1. The IRB Approval.jpg

Supplementary File 2. Genomic Diversity of Ukraine's Population Project: Protocol description, questionnaire, and informed consent to participate (*in Ukrainian with English Translation*). Supplementary File 2. The Informed Consent

Supplementary File 3. The list of the samples in this study, their characteristics and geographical locations, and sources of genomic data for each (DNBSEQ-G50 sequencing (BGI Inc., Shenzhen, China), Illumina Global Screening Array genotyping, and Illumina HiSeq sequencing array (Illumina Inc., San Diego, USA). Supplementary File 3. The List of Samples

Supplementary File 4. The full annotation database with classifications of variants in the Ukrainian populations from 97 genomes fully sequenced on BGISeq500

Supplementary File 5. List of the samples from different studies used in the current population analysis. Supplementary File 5. Sample Sources

Supplementary Figures

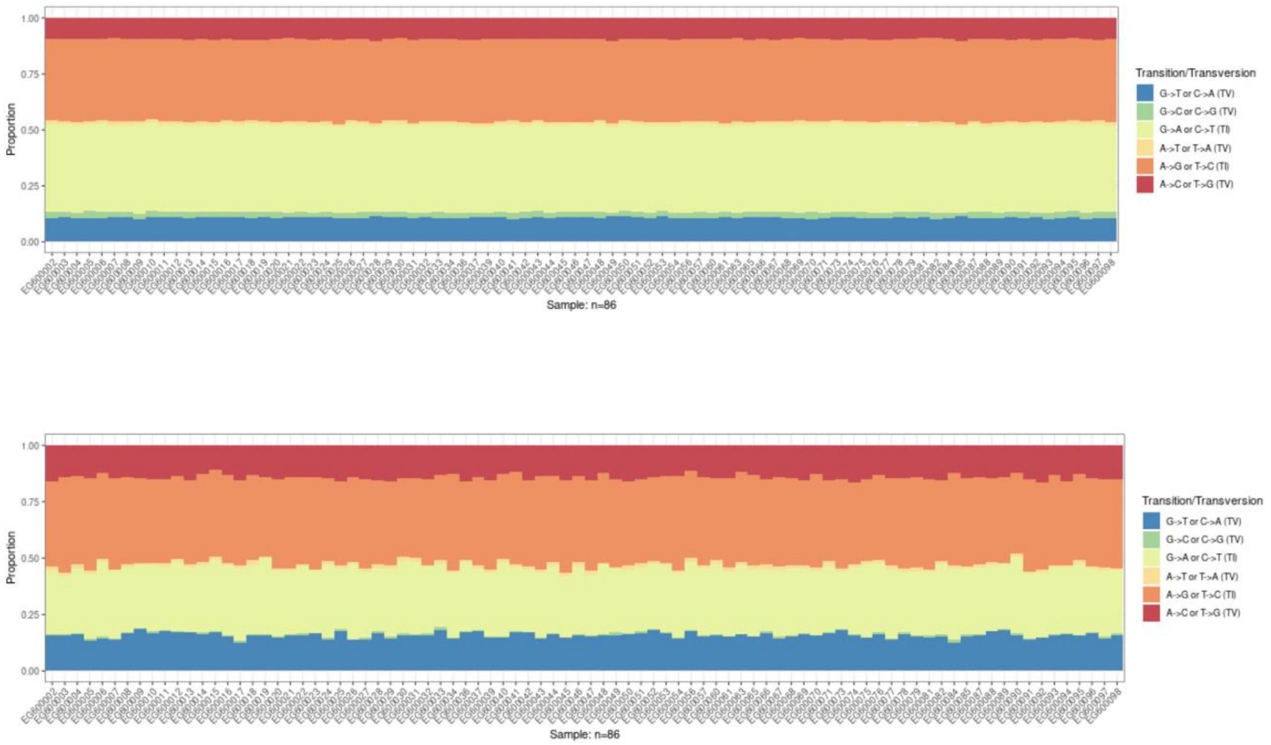

Figure S1. Transition/Transversion ratio (or TITV ratio) for the novel SNPs (estimated with *TiTvtools* [18] and visualized by *plotTiTv*) (top) for the SNPs where Illumina SNP array identified more alternate haplotypes than BGI (top right triangle in Figure 1C) and (bottom) for the SNPs where BGISeq identified more alternate haplotypes than Illumina SNP Array (bottom left triangle on Figure 1C table).

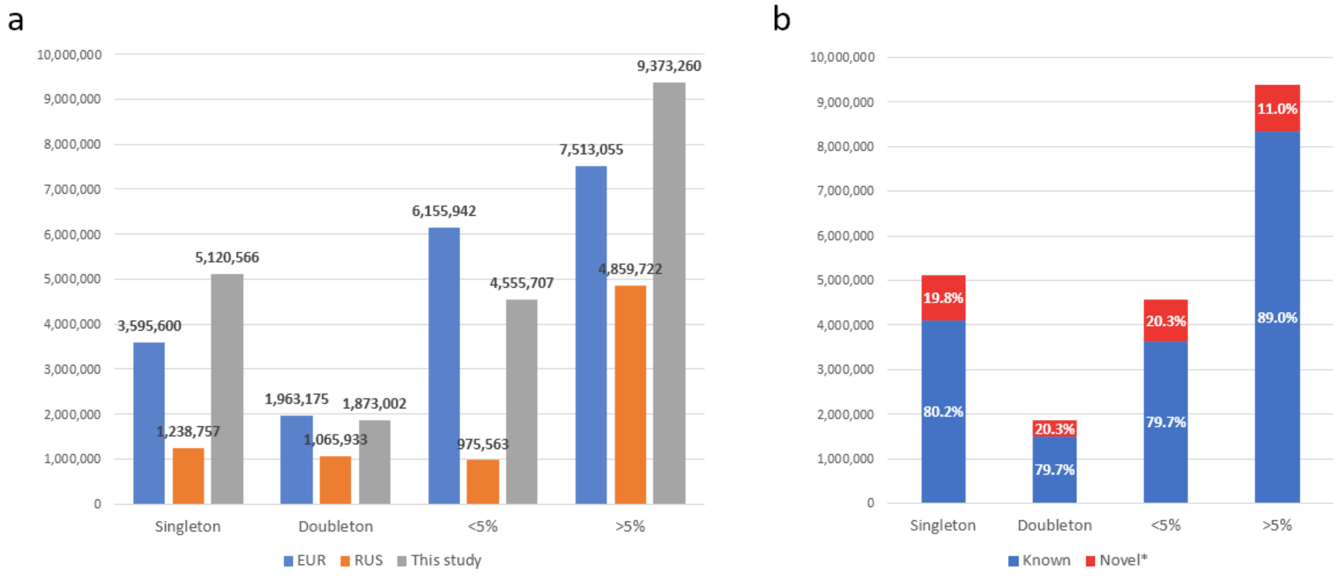

Figure S2. A. Frequencies of various classes of SNPs in the Ukrainian genome variation database. Definitions are as follows: Singleton (passed the GATK QC once), Doubleton, Rare (3-10 counts roughly equivalent to 1%< x < 5%) and Common (>5%) to make it closer to the 1KGP definitions. B. Percent novel mutations in various classes of SNPs.

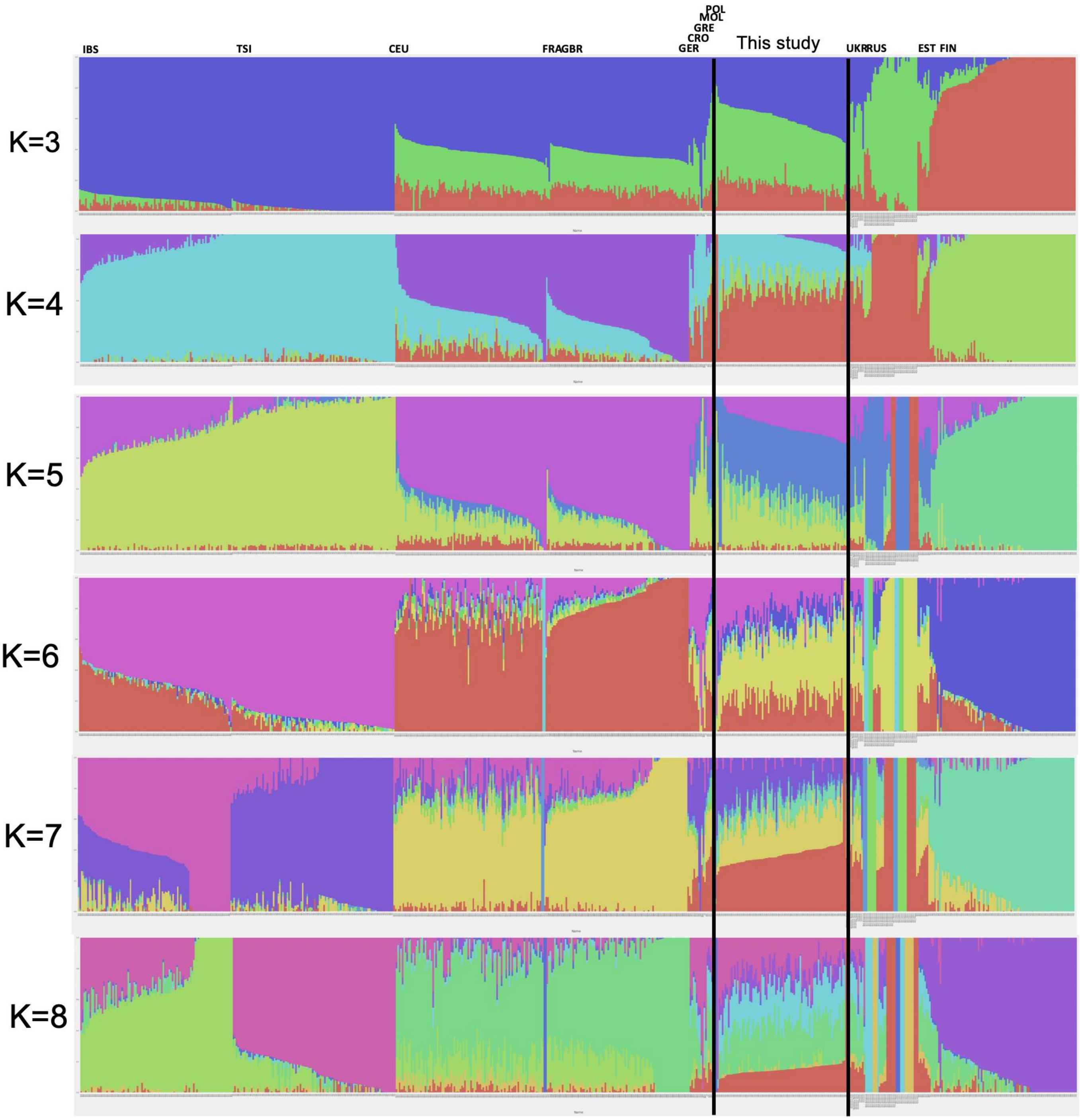

Figure S3. Genetic structure of Ukrainian population in comparison to other European populations. For identification of the optimal K parameter, we used the 10-fold cross-validation function of *ADMIXTURE* in range from 2 to 8, with K=3 resulting in the lowest error [47]. This analysis included genomes from this study as well as samples from the 1000Genomes Project (Utah Residents (CEPH) with Northern and Western European Ancestry, Toscani in Italy (TSI), Finnish in Finland (FIN), British in England and Scotland (GBR), and Iberian Population in Spain (IBS)); [13,38]) and Russians from Russia (RUS)(Novgorod and Pskov; [12] as well as the relevant high-coverage human genomes from the Estonian Biocentre Human Genome Diversity Panel (EGDP (EST; [44], and Simmons Genome Diversity project [45].

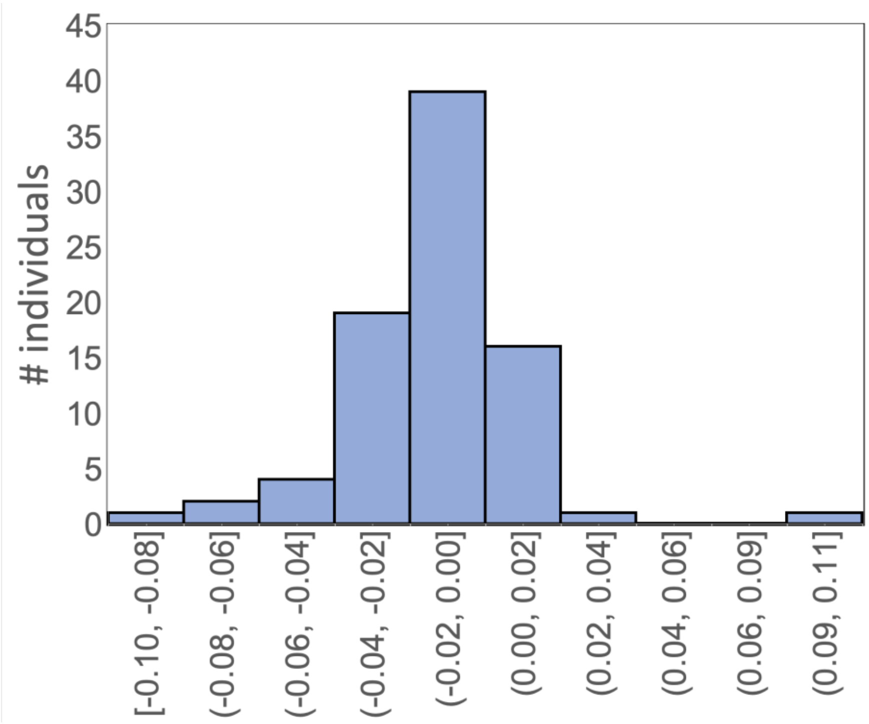

Figure S4. Distribution of inbreeding coefficients in the Ukrainian sample. The individual values corresponding to the samples are presented in **Table S7**

Supplementary Tables

Table S1. Sequencing summary of output from DNBSEQ-G50 and Illumina NovaSeq6000.

	DNBSeq-G50 ₴	Illumina NovaSeq6000 ¥
**Samples sequenced**	97	1
**Read length (bp)**	100	150
**Reads above Q20 (>99% quality score)**	97.85%	96.91 %
**Total Reads**	99,638,538,182	1,600,898,738
**Average reads/sample**	1,027,201,425	1,600,898,738
**Average GC content**	42.05%	41.07

₴ Sequencing of 97 samples were attempted on DNBSeq-G50 at BGI sequencing facility (BGI Shenzhen, CHINA), and all 97 were successful.

¥ One sample (EG600036) was sent to Illumina NovaSeq6000 S4 at Psomagen Inc. (Gaithersburg, MD, USA). In addition, 96 samples were genotyped using Illumina Global Screening Array array (Illumina Inc., San Diego, USA), and 87 were successful (86 individual samples and 1 internal QC) remained after filtering.

Table S2. Filtering summary of the data obtained from 97 whole genomes sequenced with DNBSeq-G50**.**

**Sequencing results**	**All samples**		
	**Total Unique SNPs #**	**Filtered Count**	**% Filtered₴**
**Variation**			
**SNPs**	14,738,063	1,727,084	11.7
**Bi-allelic**	14,254,070	1,586,787	11.1
**Multi-allelic**	483,993	140,297	29.0
**Small Indels ¥**	2,808,384	80,780	2.9
**Deletions**	1,864,698	57,959	3.1
**Insertions**	1,488,408	42,421	2.9
**Structural Variants $**			
**Large Deletions**	685,56	52,478	76.5
**Large Duplications**	3,374	52,478	45.3
**Inversions**	430	93	21.6
**Mobile Element Insertions**			
**Alu**	7550	1790	23.7
**L1**	3123	2672	85.6
**SVA**	222	122	55.0
**NUMT**	1169	455	38.9

Declarations

List of abbreviations

Consent for publication

The collection procedure was approved as part of the "*Genome Diversity in Ukraine*" project by the Institutional Review Board (IRB) of Uzhhorod National University, Uzhhorod Ukraine (**Supplementary File 1**). Each participant had an opportunity to review the informed consent (**Supplementary File 2**), have been explained the nature of the genome data and take a decision about making it public.

Competing interests

The following authors declare that they have no competing interests:

Taras K. Oleksyk, Walter W. Wolfsberger, Alexandra Weber, Khrystyna Shchubelka, Stephanie O. Castro-Marquez, Sarah Medley, Alina Urbanovych, Patricia Boldyzhar, Viktoriya Stakhovska, Kateryna Malyar, Yaroslava Hasynets, Juan L. Rodriguez-Flores, Fabia Battistuzzi, Siru Chen, Meredith Yeager, Michael Dean, Olga T. Oleksyk, Ryan E. Mills , and Volodymyr Smolanka

The following authors may have competing interests:

Yuan Liu, Huanming Yang, Olga Levchuk, Alla Patrus, Nelya Lazar

Funding

This research was funded in part by the internal funding from BGI (China), Uzhgorod National University (Ukraine), Division of Cancer Epidemiology and Genetics, National Cancer Institute (USA), and the startup fund of Oakland University, Rochester, Michigan.

Authors' contributions

Conceptualization: Taras K. Oleksyk, Khrystyna Shchubelka, Walter Wolfsberger, Siru Chen, Huanming Yang, Yuan Liu, Volodymyr Smolanka, Juan L. Rodriguez-Flores, Fabia Battistuzzi, Olga T. Oleksyk, Michael Dean, Meredith Yeager, Ryan Mills, and Volodymyr Smolanka

Data curation: Walter Wolfsberger, Khrystyna Shchubelka, Alexandra Weber, Alina Urbanovych, Patricia Boldyzhar, Viktoriya Stakhovska, Kateryna Malyar, Yaroslava Hasynets, Nelya Lazar, Olga T. Oleksyk

Formal analysis: Walter Wolfsberger, Alexandra Weber;

Funding acquisition: Taras K. Oleksyk;

Investigation: Khrystyna Shchubelka, Walter Wolfsberger, Alexandra Weber, Stephanie Castro-Marquez;

Methodology: Taras K. Oleksyk, Michael Dean, and Ryan Mills

Project administration: Taras K. Oleksyk, Michael Dean, and Volodymyr Smolanka;

Resources, Taras K. Oleksyk, Huanming Yang, Yuan Liu, Ryan Mills, Meredith Yeager, Michel Dean, Olga T. Oleksyk, and Volodymyr Smolanka

Software: Walter Wolfsberger, Alexandra Weber;

Supervision: Taras K. Oleksyk, Volodymyr Smolanka, Michael Dean, and Ryan Mills;

Visualization: Walter Wolfsberger, Khrystyna Shchubelka, Alexandra Weber;

Writing, Taras K. Oleksyk, Khrystyna Shchubelka, Walter Wolfsberger, Alexandra Weber (original draft), and Taras K. Oleksyk and Ryan Mills (review & editing).

Acknowledgements

We thank all the Ukrainian volunteers who contributed their data for the project.

Group authorship:

n/a

Authors' information

n/a

Endnotes

n/a

References

1. Subtelny O. Ukraine: A History, 4th Edition [Internet]. University of Toronto Press; 2009. Available from: https://play.google.com/store/books/details?id=ktyM07I9HXwC

2. Mathieson I, Alpaslan-Roodenberg S, Posth C, Szécsényi-Nagy A, Rohland N, Mallick S, et al. The genomic history of southeastern Europe. Nature [Internet]. 2018;555:197–203. Available from: http://dx.doi.org/10.1038/nature25778

3. Warmuth V, Eriksson A, Bower MA, Barker G, Barrett E, Hanks BK, et al. Reconstructing the origin and spread of horse domestication in the Eurasian steppe. Proc Natl Acad Sci U S A [Internet]. 2012;109:8202–6. Available from: http://dx.doi.org/10.1073/pnas.1111122109

4. Schubert M, Jónsson H, Chang D, Der Sarkissian C, Ermini L, Ginolhac A, et al. Prehistoric genomes reveal the genetic foundation and cost of horse domestication. Proceedings of the National Academy of Sciences [Internet]. 2014;201416991. Available from: http://www.pnas.org/content/early/2014/12/12/1416991111.abstract

5. Gaunitz C, Fages A, Hanghøj K, Albrechtsen A, Khan N, Schubert M, et al. Ancient genomes revisit the ancestry of domestic and Przewalski's horses. Science [Internet]. 2018;360:111–4. Available from: http://dx.doi.org/10.1126/science.aao3297

6. Librado P, Fages A, Gaunitz C, Leonardi M, Wagner S, Khan N, et al. The Evolutionary Origin and Genetic Makeup of Domestic Horses. Genetics [Internet]. 2016;204:423–34. Available from: http://dx.doi.org/10.1534/genetics.116.194860

7. Demay L, Péan S, Patou-Mathis M. Mammoths used as food and building resources by Neanderthals: Zooarchaeological study applied to layer 4, Molodova I (Ukraine). Quat Int [Internet]. 2012;276-277:212–26.

Available from: http://www.sciencedirect.com/science/article/pii/S1040618211006598

8. Seguin-Orlando A, Korneliussen TS, Sikora M, Malaspinas A-S, Manica A, Moltke I, et al. Genomic structure in Europeans dating back at least 36,200 years [Internet]. Science. 2014. p. 1113–8. Available from: http://dx.doi.org/10.1126/science.aaa0114

9. Eberhardt P, Owsinski J. Ethnic Groups and Population Changes in Twentieth Century Eastern Europe: History, Data and Analysis: History, Data and Analysis [Internet]. Routledge; 2015. Available from: https://play.google.com/store/books/details?id=ePsvCgAAQBAJ

10. Oleksyk TK, Brukhin V, O'Brien SJ. The Genome Russia project: closing the largest remaining omission on the world Genome map. Gigascience [Internet]. BioMed Central Ltd; 2015;4:53. Available from: http://www.gigasciencejournal.com/content/4/1/53

11. Karczewski KJ, Francioli LC, Tiao G, Cummings BB, Alföldi J, Wang Q, et al. The mutational constraint spectrum quantified from variation in 141,456 humans. Nature [Internet]. 2020;581:434–43. Available from: http://dx.doi.org/10.1038/s41586-020-2308-7

12. Zhernakova DV, Brukhin V, Malov S, Oleksyk TK, Koepfli KP, Zhuk A, et al. Genome-wide sequence analyses of ethnic populations across Russia. Genomics [Internet]. Academic Press; 2019; Available from: https://www.sciencedirect.com/science/article/pii/S0888754318307419

13. Auton A, Abecasis GRGR, Altshuler DMDM, Durbin RMRM, Bentley DRDR, Chakravarti A, et al. A global reference for human genetic variation. Nature [Internet]. 2015;526. Available from: http://dx.doi.org/10.1038/nature15393

14. Kim J, Weber JA, Jho S, Jang J, Jun J, Cho YS, et al. KoVariome: Korean National Standard Reference Variome database of whole genomes with comprehensive SNV, indel, CNV, and SV analyses. Sci Rep [Internet]. 2018;8:5677. Available from: http://dx.doi.org/10.1038/s41598-018-23837-x

15. Van der Auwera GA, Carneiro MO, Hartl C, Poplin R, del Angel G, Levy-Moonshine A, et al. From fastQ data to high-confidence variant calls: The genome analysis toolkit best practices pipeline. Curr Protoc Bioinformatics [Internet]. 2013; Available from: http://dx.doi.org/10.1002/0471250953.bi1110s43

16. Layer RM, Chiang C, Quinlan AR, Hall IM. LUMPY: a probabilistic framework for structural variant discovery. Genome Biol [Internet]. 2014;15:R84. Available from: http://dx.doi.org/10.1186/gb-2014-15-6-r84

17. Schneider VA, Graves-Lindsay T, Howe K, Bouk N, Chen H-C, Kitts PA, et al. Evaluation of GRCh38 and de novo haploid genome assemblies demonstrates the enduring quality of the reference assembly [Internet]. Genome Research. 2017. p. 849–64. Available from: http://dx.doi.org/10.1101/gr.213611.116

18. Mayakonda A, Lin D-C, Assenov Y, Plass C, Koeffler HP. Maftools: efficient and comprehensive analysis of somatic variants in cancer. Genome Res [Internet]. 2018;28:1747–56. Available from: http://dx.doi.org/10.1101/gr.239244.118

19. Sherry ST, Ward M, Sirotkin K. dbSNP—Database for Single Nucleotide Polymorphisms and Other Classes of Minor Genetic Variation. Genome Res [Internet]. 1999;9:677–9. Available from: http://genome.cshlp.org/content/9/8/677.short

20. Campbell IM, Gambin T, Jhangiani S, Grove ML, Veeraraghavan N, Muzny DM, et al. Multiallelic Positions in the Human Genome: Challenges for Genetic Analyses. Hum Mutat [Internet]. 2016;37:231–4. Available from: http://dx.doi.org/10.1002/humu.22944

21. Kosugi S, Momozawa Y, Liu X, Terao C, Kubo M, Kamatani Y. Comprehensive evaluation of structural variation detection algorithms for whole genome sequencing. Genome Biol [Internet]. 2019;20:117. Available from: http://dx.doi.org/10.1186/s13059-019-1720-5

22. Ye K, Hall G, Ning Z. Structural variation detection from next generation sequencing. Next Generat Sequenc & Applic. 2016;1.

23. MacDonald JR, Ziman R, Yuen RKC, Feuk L, Scherer SW. The Database of Genomic Variants: a curated collection of structural variation in the human genome. Nucleic Acids Res [Internet]. 2014;42:D986–92. Available from: http://dx.doi.org/10.1093/nar/gkt958

24. Mak SST, Gopalakrishnan S, Carøe C, Geng C, Liu S, Sinding M-HS, et al. Erratum to: Comparative performance of the BGISEQ-500 vs Illumina HiSeq2500 sequencing platforms for palaeogenomic sequencing. Gigascience [Internet]. 2018;7. Available from: http://dx.doi.org/10.1093/gigascience/giy151

25. Zhou Y, Liu C, Zhou R, Lu A, Huang B, Liu L, et al. SEQdata-BEACON: a comprehensive database of sequencing performance and statistical tools for performance evaluation and yield simulation in BGISEQ-500. BioData Min [Internet]. 2019;12:21. Available from: https://doi.org/10.1186/s13040-019-0209-9

26. Loewe L, Hill WG. The population genetics of mutations: good, bad and indifferent. Philos Trans R Soc Lond B Biol Sci [Internet]. 2010;365:1153–67. Available from: http://dx.doi.org/10.1098/rstb.2009.0317

27. Volfovsky N, Oleksyk TK, Cruz KC, Truelove AL, Stephens RM, Smith MWBHW. Genome and gene alterations by insertions and deletions in the evolution of human and chimpanzee chromosome 22. BMC Genomics [Internet]. 2009;10:51. Available from: http://dx.doi.org/10.1186/1471-2164-10-51

28. Buniello A, MacArthur JAL, Cerezo M, Harris LW, Hayhurst J, Malangone C, et al. The NHGRI-EBI GWAS Catalog of published genome-wide association studies, targeted arrays and summary statistics 2019. Nucleic Acids Res [Internet]. Oxford Academic; 2019 [cited 2020 Jul 7];47:D1005–12. Available from: https://academic.oup.com/nar/article/47/D1/D1005/5184712

29. Landrum MJ, Lee JM, Benson M, Brown GR, Chao C, Chitipiralla S, et al. ClinVar: improving access to variant interpretations and supporting evidence. Nucleic Acids Res [Internet]. 2018;46:D1062–7. Available from: http://dx.doi.org/10.1093/nar/gkx1153

30. Landrum MJ, Lee JM, Benson M, Brown G, Chao C, Chitipiralla S, et al. ClinVar: public archive of interpretations of clinically relevant variants. Nucleic Acids Res [Internet]. 2016;44:D862–8. Available from: http://dx.doi.org/10.1093/nar/gkv1222

31. Cooper DN, Krawczak M, Polychronakos C, Tyler-Smith C, Kehrer-Sawatzki H. Where genotype is not predictive of phenotype: towards an understanding of the molecular basis of reduced penetrance in human inherited disease. Hum Genet [Internet]. 2013;132:1077–130. Available from: http://dx.doi.org/10.1007/s00439-013-1331-2

32. Lobo I. Same genetic mutation, different genetic disease phenotype. Nature Education [Internet]. 2008;1. Available from: http://lms.bums.ac.ir/pluginfile.php/25862/mod_forum/attachment/3486/Same%20Genetic%20Mutatio1.pdf

33. Visscher PM, Wray NR, Zhang Q, Sklar P, McCarthy MI, Brown MA, et al. 10 Years of GWAS Discovery: Biology, Function, and Translation. Am J Hum Genet [Internet]. 2017;101:5–22. Available from: http://dx.doi.org/10.1016/j.ajhg.2017.06.005

34. Marigorta UM, Rodríguez JA, Gibson G, Navarro A. Replicability and Prediction: Lessons and Challenges from GWAS. Trends Genet [Internet]. 2018;34:504–17. Available from: http://dx.doi.org/10.1016/j.tig.2018.03.005

35. Eilbeck K, Quinlan A, Yandell M. Settling the score: variant prioritization and Mendelian disease. Nat Rev Genet [Internet]. 2017;18:599–612. Available from: http://dx.doi.org/10.1038/nrg.2017.52

36. Boyle EA, Li YI, Pritchard JK. An Expanded View of Complex Traits: From Polygenic to Omnigenic. Cell [Internet]. 2017;169:1177–86. Available from: http://dx.doi.org/10.1016/j.cell.2017.05.038

37. Oleksyk TK, Smith MW, O'Brien SJ. Genome-wide scans for footprints of natural selection. Philos Trans R Soc Lond B Biol Sci [Internet]. 2010;365:185–205. Available from: http://dx.doi.org/10.1098/rstb.2009.0219

38. Altshuler DM, Durbin RM, Abecasis GR, Bentley DR, Chakravarti A, Clark AG, et al. An integrated map of genetic variation from 1,092 human genomes. Nature [Internet]. 2012;491:56–65. Available from: http://dx.doi.org/10.1038/nature11632

39. Nugent A, Conatser KR, Turner LL, Nugent JT, Sarino EMB, Ricks-Santi LJ. Reporting of race in genome and exome sequencing studies of cancer: a scoping review of the literature. Genet Med [Internet]. 2019;21:2676–80. Available from: http://dx.doi.org/10.1038/s41436-019-0558-2

40. Spratt DE, Chan T, Waldron L, Speers C, Feng FY, Ogunwobi OO, et al. Racial/Ethnic Disparities in Genomic Sequencing. JAMA Oncol [Internet]. 2016;2:1070–4. Available from: http://dx.doi.org/10.1001/jamaoncol.2016.1854

41. Chen C-Y, Pollack S, Hunter DJ, Hirschhorn JN, Kraft P, Price AL. Improved ancestry inference using weights from external reference panels. Bioinformatics [Internet]. 2013;29:1399–406. Available from: http://dx.doi.org/10.1093/bioinformatics/btt144

42. Smith MW, O'Brien SJ. Mapping by admixture linkage disequilibrium: advances, limitations and guidelines. Nat Rev Genet [Internet]. 2005;6:623–32. Available from: http://dx.doi.org/10.1038/nrg1657

43. Stephens JC, Briscoe D, O'Brien SJ. Mapping by admixture linkage disequilibrium in human populations: limits and guidelines. Am J Hum Genet [Internet]. 1994;55:809–24. Available from: https://www.ncbi.nlm.nih.gov/pubmed/7942858

44. Pagani L, Lawson DJ, Jagoda E, Mörseburg A, Eriksson A, Mitt M, et al. Genomic analyses inform on migration events during the peopling of Eurasia. Nature [Internet]. Nature Publishing Group; 2016;538:238–42. Available from: http://dx.doi.org/10.1038/nature19792

45. Mallick S, Li H, Lipson M, Mathieson I, Gymrek M, Racimo F, et al. The Simons Genome Diversity Project: 300 genomes from 142 diverse populations. Nature [Internet]. Nature Publishing Group; 2016;538:201–6. Available from: http://dx.doi.org/10.1038/nature18964

46. Patterson N, Price AL, Reich D, Plenge RM, Weinblatt ME, Shadick NA, et al. EIGENSOFT version 5.01. Harvard University. 2013;

47. Alexander DH, Novembre J, Lange K. Fast model-based estimation of ancestry in unrelated individuals. Genome Res [Internet]. 2009;19:1655–64. Available from: http://dx.doi.org/10.1101/gr.094052.109

48. Gardner EJ, Lam VK, Harris DN, Chuang NT, Scott EC, Pittard WS, et al. The Mobile Element Locator Tool (MELT): population-scale mobile element discovery and biology. Genome Res [Internet]. 2017;27:1916–29. Available from: http://dx.doi.org/10.1101/gr.218032.116

49. Mousavi N, Shleizer-Burko S, Yanicky R, Gymrek M. Profiling the genome-wide landscape of tandem repeat expansions. Nucleic Acids Res [Internet]. 2019;47:e90. Available from: http://dx.doi.org/10.1093/nar/gkz501

50. Dayama G, Emery SB, Kidd JM, Mills RE. The genomic landscape of polymorphic human nuclear mitochondrial insertions. Nucleic Acids Res [Internet]. 2014;42:12640–9. Available from: http://dx.doi.org/10.1093/nar/gku1038

51. Wang K, Li M, Hakonarson H. ANNOVAR: functional annotation of genetic variants from high-throughput sequencing data. Nucleic Acids Res [Internet]. 2010;38:e164. Available from: http://dx.doi.org/10.1093/nar/gkq603

52. Cingolani P, Platts A, Wang LL, Coon M, Nguyen T, Wang L, et al. A program for annotating and

predicting the effects of single nucleotide polymorphisms, SnpEff: SNPs in the genome of Drosophila melanogaster strain w1118; iso-2; iso-3. Fly [Internet]. 2012;6:80–92. Available from: http://dx.doi.org/10.4161/fly.19695

53. Liu X, Wu C, Li C, Boerwinkle E. dbNSFP v3.0: A One-Stop Database of Functional Predictions and Annotations for Human Nonsynonymous and Splice-Site SNVs. Hum Mutat [Internet]. 2016;37:235–41. Available from: http://dx.doi.org/10.1002/humu.22932

54. Zheng-Bradley X, Streeter I, Fairley S, Richardson D, Clarke L, Flicek P, et al. Alignment of 1000 Genomes Project reads to reference assembly GRCh38. Gigascience [Internet]. 2017;6:1–8. Available from: http://dx.doi.org/10.1093/gigascience/gix038

55. Ruden D, Cingolani P, Patel V, Coon M, Nguyen T, Land S, et al. Using Drosophila melanogaster as a Model for Genotoxic Chemical Mutational Studies with a New Program, SnpSift. Front Genet [Internet]. 2012;3:35. Available from: https://www.frontiersin.org/article/10.3389/fgene.2012.00035

56. McKinney W, Others. Data structures for statistical computing in python. Proceedings of the 9th Python in Science Conference [Internet]. Austin, TX; 2010. p. 51–6. Available from: http://conference.scipy.org/proceedings/scipy2010/pdfs/mckinney.pdf

57. Virtanen P, Gommers R, Oliphant TE, Haberland M, Reddy T, Cournapeau D, et al. SciPy 1.0: fundamental algorithms for scientific computing in Python. Nat Methods [Internet]. 2020;17:261–72. Available from: http://dx.doi.org/10.1038/s41592-019-0686-2

58. Purcell S, Neale B, Todd-Brown K, Thomas L, Ferreira MAR, Bender D, et al. PLINK: a tool set for whole-genome association and population-based linkage analyses. Am J Hum Genet [Internet]. 2007;81:559–75. Available from: http://dx.doi.org/10.1086/519795

59. Lander ES, Linton LM, Birren B, Nusbaum C, Zody MC, Baldwin J, et al. Initial sequencing and analysis of the human genome. Nature [Internet]. 2001;409:860–921. Available from: http://dx.doi.org/10.1038/35057062

60. Venter JC, Adams MD, Myers EW, Li PW, Mural RJ, Sutton GG, et al. The sequence of the human genome. Science [Internet]. 2001;291:1304–51. Available from: http://dx.doi.org/10.1126/science.1058040

61. Sherman RM, Salzberg SL. Pan-genomics in the human genome era [Internet]. Nature Reviews Genetics. 2020. p. 243–54. Available from: http://dx.doi.org/10.1038/s41576-020-0210-7

62. Kempfer R, Pombo A. Methods for mapping 3D chromosome architecture [Internet]. Nature Reviews Genetics. 2020. p. 207–26. Available from: http://dx.doi.org/10.1038/s41576-019-0195-2

63. Oleksyk TKTK, Brukhin V, O'Brien SJSJ, Sills J. Genome Russia. Science [Internet]. 2015;350. Available from: http://dx.doi.org/10.1126/science.350.6262.747-a

Web links and URLs

GigaDB ______________
http://genomes.uzhhorod.edu.ua

Table S1-2

Click here to access/download;Table;Table S2. Sequencing and Filtering Summary.xlsx

Table S1. Sequencing summary of output from DNBSEQ-G50 and Illumina NovaSeq6000.

	DNBSeq-G50 ₴	llumina NovaSeq6000 ¥
**Samples sequenced**	97	1
**Read length (bp)**	100	150
**Reads above Q20 (>99% quality**	97.85%	96.91 %
**Total Reads**	99,638,538,182	1,600,898,738
**Average reads/sample**	1,027,201,425	1,600,898,738
**Average GC content**	42.05%	41.07

₴ Sequencing of 97 samples were attempted on DNBSeq-G50 at BGI sequencing facility (BGI Shenzhen, CHINA), and all 97 w

¥ One sample (EG600036) was sent to Illumina NovaSeq6000 S4 at Psomagen Inc. (Gaithersburg, MD, USA). In addition, 96 s

Table S2. Filtering summary of the data obtained from 97 whole genomes sequenced with DNBSeq-G50.

Sequencing results	All samples		
	**Total Unique SNPs #**	**Filtered Count**	**% Filtered₴**
Variation			
**SNPs**	14,738,063	1,727,084	11.7
**Bi-allelic**	14,254,070	1,586,787	11.1
**Multi-allelic**	483,993	140,297	29
**Small Indels ¥**	2,808,384	80,780	2.9
**Deletions**	1,864,698	57,959	3.1
**Insertions**	1,488,408	42,421	2.9
**Structural Variants $**			
**Large Deletions**	685,56	52,478	76.5
**Large Duplications**	3,374	52,478	45.3
**Inversions**	430	93	21.6
**Mobile Element Insertions**			
**Alu**	7550	1790	23.7
**L1**	3123	2672	85.6
**SVA**	222	122	55
**NUMT**	1169	455	38.9

vere successful.

amples were genotyped using Illumina Global Screening Array array (Illumina Inc., San Diego, USA), and 87 were successful (86 individual sa

ımples and 1 internal QC) remained after filtering.

Click here to access/download;Table;S1_sequencing_stats.xlsx

Sample	Clean Reads	Clean bases	Read length (bp)	Q20(%)	GC(%)
Illumina Hiseq Illumina NovaSeq6000					
EG600036	1,325,824,028	198873604200	150	96.91%	41.07%
MGI DNBSEQ-G50					
EG600001	1,010,900,376	101,090,037,600	100	98.23%	42.38%
EG600002	1,005,891,702	100,589,170,200	100	98.15%	42.11%
EG600003	1,009,303,880	100,930,388,000	100	98.29%	42.14%
EG600004	983,104,802	98,310,480,200	100	98.13%	42.47%
EG600005	1,008,570,176	100,857,017,600	100	98.22%	42.27%
EG600006	1,008,633,484	100,863,348,400	100	98.04%	41.99%
EG600007	1,010,997,442	101,099,744,200	100	98.23%	41.78%
EG600008	1,009,485,606	100,948,560,600	100	97.40%	41.94%
EG600009	1,012,169,820	101,216,982,000	100	97.40%	41.89%
EG600010	1,009,831,640	100,983,164,000	100	97.68%	41.85%
EG600011	1,009,233,444	100,923,344,400	100	97.74%	42.18%
EG600012	1,010,587,568	101,058,756,800	100	97.90%	42.02%
EG600013	1,012,157,562	101,215,756,200	100	97.71%	41.77%
EG600014	1,010,840,982	101,084,098,200	100	97.77%	41.68%
EG600015	1,008,524,246	100,852,424,600	100	97.92%	42.04%
EG600016	1,010,229,074	101,022,907,400	100	97.93%	42.03%
EG600017	1,008,034,326	100,803,432,600	100	97.29%	41.97%
EG600018	1,133,820,076	113,382,007,600	100	97.55%	42.99%
EG600019	1,158,654,942	115,865,494,200	100	97.40%	43.54%
EG600020	1,186,191,440	118,619,144,000	100	97.60%	43.02%
EG600021	1,184,420,884	118,442,088,400	100	97.71%	42.20%
EG600022	1,229,884,504	122,988,450,400	100	97.56%	42.70%
EG600023	1,091,677,340	109,167,734,000	100	97.51%	42.67%
EG600024	1,180,648,464	118,064,846,400	100	97.30%	42.59%
EG600025	1,208,015,100	120,801,510,000	100	97.45%	41.88%
EG600026	1,228,152,722	122,815,272,200	100	97.50%	42.46%
EG600027	1,194,303,528	119,430,352,800	100	97.84%	42.65%
EG600028	1,008,694,760	100,869,476,000	100	97.99%	42.00%
EG600029	1,012,550,690	101,255,069,000	100	98.37%	42.43%
EG600030	1,013,027,434	101,302,743,400	100	98.05%	42.40%
EG600031	1,009,762,124	100,976,212,400	100	98.25%	42.21%
EG600032	1,010,644,570	101,064,457,000	100	98.01%	42.11%
EG600033	1,008,814,996	100,881,499,600	100	98.07%	41.97%
EG600034	1,009,411,080	100,941,108,000	100	98.20%	41.27%
EG600035	1,009,685,742	100,968,574,200	100	98.08%	41.74%
EG600036	1,009,287,730	100,928,773,000	100	97.99%	41.95%
EG600037	1,011,969,670	101,196,967,000	100	97.90%	42.74%
EG600038	1,009,128,672	100,912,867,200	100	97.67%	42.22%
EG600039	1,009,728,626	100,972,862,600	100	97.91%	42.26%
EG600040	1,013,751,564	101,375,156,400	100	97.83%	41.98%
EG600041	1,008,864,382	100,886,438,200	100	98.01%	41.83%

EG600042	1,008,633,486	100,863,348,600	100	98.05%	41.99%
EG600043	1,008,293,364	100,829,336,400	100	97.29%	42.09%
EG600044	1,012,439,192	101,243,919,200	100	97.38%	43.09%
EG600045	1,008,240,536	100,824,053,600	100	97.48%	42.60%
EG600046	1,010,802,782	101,080,278,200	100	97.06%	42.14%
EG600047	1,007,913,186	100,791,318,600	100	96.50%	41.96%
EG600048	1,011,273,390	101,127,339,000	100	98.29%	42.78%
EG600049	1,011,046,570	101,104,657,000	100	98.51%	41.65%
EG600050	1,008,071,616	100,807,161,600	100	98.50%	41.93%
EG600051	1,008,499,644	100,849,964,400	100	98.20%	41.95%
EG600052	1,007,863,566	100,786,356,600	100	98.38%	42.46%
EG600053	1,013,284,478	101,328,447,800	100	98.35%	41.90%
EG600054	1,010,696,470	101,069,647,000	100	98.19%	43.03%
EG600055	1,011,911,780	101,191,178,000	100	98.49%	41.97%
EG600056	1,009,910,516	100,991,051,600	100	98.42%	42.40%
EG600057	1,007,895,902	100,789,590,200	100	98.43%	41.85%
EG600059	1,008,136,158	100,813,615,800	100	98.03%	41.75%
EG600060	1,012,021,200	101,202,120,000	100	97.95%	42.51%
EG600061	1,010,755,592	101,075,559,200	100	98.14%	41.94%
EG600062	1,008,287,740	100,828,774,000	100	97.84%	41.90%
EG600063	1,010,028,336	101,002,833,600	100	98.27%	42.28%
EG600064	1,007,985,758	100,798,575,800	100	98.60%	42.11%
EG600065	1,010,518,810	101,051,881,000	100	98.49%	42.06%
EG600066	1,013,147,842	101,314,784,200	100	98.55%	41.77%
EG600067	1,006,962,436	100,696,243,600	100	98.74%	41.89%
EG600068	1,011,508,960	101,150,896,000	100	98.33%	41.81%
EG600069	1,008,409,178	100,840,917,800	100	98.70%	41.78%
EG600070	1,010,040,160	101,004,016,000	100	98.26%	41.60%
EG600071	1,011,258,760	101,125,876,000	100	98.35%	41.14%
EG600072	1,008,358,214	100,835,821,400	100	98.47%	41.89%
EG600073	1,010,128,780	101,012,878,000	100	98.23%	41.73%
EG600074	1,009,981,420	100,998,142,000	100	98.27%	41.63%
EG600075	1,012,386,076	101,238,607,600	100	98.20%	41.97%
EG600076	1,008,349,368	100,834,936,800	100	98.17%	41.97%
EG600077	1,008,480,614	100,848,061,400	100	98.13%	41.87%
EG600078	1,007,816,616	100,781,661,600	100	98.19%	41.71%
EG600079	1,013,157,480	101,315,748,000	100	98.07%	41.63%
EG600080	1,010,606,938	101,060,693,800	100	98.27%	42.13%
EG600081	1,013,321,818	101,332,181,800	100	98.27%	42.15%
EG600082	1,010,548,796	101,054,879,600	100	98.14%	41.89%
EG600083	1,008,765,946	100,876,594,600	100	97.97%	41.88%
EG600084	1,008,966,594	100,896,659,400	100	97.90%	41.79%
EG600085	1,012,013,584	101,201,358,400	100	96.31%	41.62%
EG600086	1,009,547,738	100,954,773,800	100	96.80%	41.55%
EG600087	1,009,776,660	100,977,666,000	100	96.46%	41.43%
EG600088	1,009,402,846	100,940,284,600	100	96.55%	41.44%
EG600089	1,008,733,502	100,873,350,200	100	96.72%	41.76%

EG600090	1,011,320,438	101,132,043,800	100	96.03%	41.61%
EG600091	1,006,778,018	100,677,801,800	100	96.82%	41.64%
EG600092	1,009,914,806	100,991,480,600	100	96.59%	41.62%
EG600093	1,009,821,386	100,982,138,600	100	96.68%	41.61%
EG600094	1,011,965,490	101,196,549,000	100	97.05%	41.77%
EG600095	1,011,431,164	101,143,116,400	100	97.73%	41.82%
EG600096	1,010,529,336	101,052,933,600	100	97.91%	41.93%
EG600097	1,013,270,446	101,327,044,600	100	97.99%	41.97%
EG600098	1,007,739,530	100,773,953,000	100	98.06%	41.97%

Table S3 Click here to access/download;Table;S3_High_impact_full_2.xlsx

General information								
Id	Chr	Position	Ref	Alt	Gene	Allele Coun	Allele Freq	Type
769497	chr1	939398	GCCTCCCC/	G	SAMD11	9	0.046	INDEL
rs6176628	chr1	1014217	C	T	ISG15	2	0.01	SNP
rs1921;402	chr1	1014228	G	A	ISG15	77	0.397	SNP
rs3813194	chr1	1063202	G	C	RP11-465B	100	0.515	SNP
rs6262358	chr1	1318875	G	A	CPSF3L	2	0.01	SNP
rs3831366	chr1	1398672	CTAGAG	C	CCNL2	4	0.021	INDEL
rs2014019	chr1	1580020	AT	ATT	AL645728.:	5	0.026	INDEL
rs2014019	chr1	1580020	AT	A	AL645728.:	7	0.036	INDEL
rs2014019	chr1	1580020	AT	AATT	AL645728.:	2	0.01	INDEL
rs1275508	chr1	1623412	T	C	MIB2	185	0.954	SNP
.	chr1	1641667	GA	G	CDK11B	3	0.017	INDEL
.	chr1	1658912	A	C	CDK11B	3	0.015	SNP
.	chr1	2212536	CAGGCAGC	C	FAAP20	2	0.01	INDEL
rs1801174	chr1	3722110	C	T	TP73	177	0.912	SNP
rs5570471	chr1	3741219	C	T	TP73-AS1	49	0.253	SNP
rs3612427	chr1	3783866	G	T	LRRC47	10	0.052	SNP
rs241211	chr1	4572468	A	G	RP1-37J18.	27	0.139	SNP
.	chr1	5875102	T	A	NPHP4	31	0.16	SNP
rs2227911	chr1	6082248	T	C	KCNAB2	25	0.129	SNP
rs1202315	chr1	7830004	A	G	PER3	67	0.353	SNP
rs7165361	chr1	7970934	G	A	PARK7	3	0.015	SNP
rs2274330	chr1	8949411	G	C	CA6	19	0.098	SNP
rs1398556	chr1	9244919	CCCCAGGC	C	H6PD	36	0.186	INDEL
rs1281013	chr1	10949622	G	A	C1orf127	14	0.072	SNP
rs1481154	chr1	10991092	C	G	CFL1P6	3	0.015	SNP
rs2233019	chr1	11676807	A	C	MAD2L2	7	0.036	SNP
rs2233004	chr1	11682017	C	T	MAD2L2	147	0.758	SNP
rs4127546	chr1	11784463	C	T	C1orf167	3	0.015	SNP
rs5065;226	chr1	11846011	A	G	NPPA	22	0.113	SNP
.	chr1	12716215	C	A	AADACL3	17	0.088	SNP
rs7541167	chr1	12860036	G	T	PRAMEF2	6	0.031	SNP
rs2006527	chr1	12879725	C	CG	PRAMEF4	18	0.093	INDEL
rs3676951	chr1	15192442	CTTTCTTTC	C	RP1-21O18	4	0.023	INDEL
rs2234723	chr1	15493812	A	AC	CASP9	40	0.206	INDEL
.	chr1	15569631	CTT	CT	DNAJC16	22	0.115	INDEL
.	chr1	15569631	CTT	CTTTT	DNAJC16	41	0.214	INDEL
.	chr1	15569631	CTT	CTTT	DNAJC16	10	0.052	INDEL
.	chr1	15569631	CTT	C	DNAJC16	3	0.016	INDEL
rs2014668	chr1	15747029	C	CGA	TMEM82	7	0.036	INDEL
rs1999085	chr1	15747032	T	TG	TMEM82	7	0.036	INDEL
.	chr1	15935987	CGT	C	SPEN	2	0.01	INDEL
rs4126915	chr1	15939409	T	A	SPEN	14	0.072	SNP
rs5570000	chr1	16135769	C	T	EPHA2	5	0.026	SNP
rs2230597;	chr1	16138178	G	A	EPHA2	76	0.392	SNP

rs6678616;	chr1	16148628	C	T	EPHA2	54	0.278	SNP
rs6678618;	chr1	16148631	C	T	EPHA2	54	0.278	SNP
.	chr1	16705437	ATT	AT	RP1-163M9	89	0.464	INDEL
.	chr1	16705437	ATT	ATTTTTTTT	RP1-163M9	14	0.073	INDEL
.	chr1	16705437	ATT	A	RP1-163M9	8	0.042	INDEL
.	chr1	16705437	ATT	ATTTTTTTT	RP1-163M9	10	0.052	INDEL
.	chr1	16705437	ATT	ATTTTTTT	RP1-163M9	4	0.021	INDEL
.	chr1	16705437	ATT	ATTTTTTTT	RP1-163M9	3	0.016	INDEL
.	chr1	16873501	C	CG	RP11-108M	3	0.015	INDEL
.	chr1	16873501	C	CGG	RP11-108M	3	0.015	INDEL
rs3818032	chr1	17075760	C	T	PADI2	125	0.644	SNP
rs6787910:	chr1	17331629	GTGGCC	G	AC004824.	113	0.582	INDEL
rs874881	chr1	17334004	G	C	PADI4	102	0.526	SNP
rs3398138:	chr1	17342393	A	G	PADI4	2	0.01	SNP
rs2240335	chr1	17348042	C	A	PADI4	72	0.371	SNP
rs6175768:	chr1	18872954	G	T	ALDH4A1	4	0.021	SNP
rs6695033;	chr1	18875425	T	C	ALDH4A1	5	0.026	SNP
rs2230709;	chr1	18875434	C	T	ALDH4A1	43	0.222	SNP
rs2230708;	chr1	18875462	A	G	ALDH4A1	124	0.639	SNP
rs7550938;	chr1	18876423	T	C	ALDH4A1	119	0.613	SNP
rs2230705;	chr1	18877503	C	G	ALDH4A1	132	0.68	SNP
rs6723297(	chr1	19081800	G	GC	UBR4	75	0.387	INDEL
rs1405869:	chr1	19270780	TC	T	AKR7L	9	0.046	INDEL
rs1907477:	chr1	19270834	A	G	AKR7L	9	0.046	SNP
.	chr1	19273984	CTGCGGCG	C	AKR7L	9	0.046	INDEL
.	chr1	19327559	CTGTTCAC(	C	PQLC2	3	0.016	INDEL
rs5837498	chr1	19617327	CACAG	C	MINOS1-NI	15	0.077	INDEL
rs3530755:	chr1	20063483	G	A	PLA2G5	10	0.052	SNP
rs1213910(	chr1	20175089	G	A	PLA2G2C	26	0.134	SNP
.	chr1	20692776	CTT	CT	RP5-930J4.	75	0.387	INDEL
.	chr1	20692776	CTT	C	RP5-930J4.	6	0.031	INDEL
.	chr1	20692776	CTT	CTTT	RP5-930J4.	10	0.052	INDEL
rs6177597	chr1	21598419	A	G	RAP1GAP	2	0.01	SNP
rs7406002(	chr1	21608865	C	T	RAP1GAP	5	0.026	SNP
.	chr1	21706892	GA	G	USP48	21	0.108	INDEL
.	chr1	21706892	GA	GAA	USP48	4	0.021	INDEL
rs1449942:	chr1	21978455	G	A	CELA3B	2	0.01	SNP
rs7523572	chr1	22028796	C	G	LINC00339	146	0.753	SNP
rs3841849	chr1	22030938	CAGAA	C	LINC00339	142	0.732	INDEL
rs3841849	chr1	22030938	CAGAA	CGAA	LINC00339	4	0.021	INDEL
.	chr1	22030940	GAAA	*	LINC00339	142	0.732	INDEL
.	chr1	22030940	GAAA	G	LINC00339	4	0.021	INDEL
rs2235527	chr1	22127482	C	G	WNT4	31	0.16	SNP
rs1384319(	chr1	22953998	G	T	LACTBL1	7	0.036	SNP
rs7441526	chr1	23434535	G	A	ASAP3	15	0.077	SNP
rs1155159:	chr1	23436956	G	A	ASAP3	12	0.062	SNP
rs3905358	chr1	23670909	A	T	RP11-223J1	59	0.304	SNP

.	chr1	23838140	C	T	HMGCL	2	0.01	SNP
rs1090302	chr1	24138623	C	T	IL22RA1	73	0.376	SNP
.	chr1	25208512	CT	C	RP4-706G2	32	0.165	INDEL
.	chr1	25208512	CT	CTT	RP4-706G2	11	0.057	INDEL
rs2013054	chr1	26183819	AC	A	CNKSR1	9	0.046	INDEL
.	chr1	26282334	TGGGGCCC	T	UBXN11	88	0.463	INDEL
.	chr1	26282334	TGGGGCCC	*	UBXN11	10	0.053	INDEL
.	chr1	26282344	ACCGGGAC	A	UBXN11	89	0.484	INDEL
.	chr1	26282344	ACCGGGAC	*	UBXN11	10	0.054	INDEL
.	chr1	26282357	CTGGGGCC	*	UBXN11	94	0.49	INDEL
.	chr1	26282357	CTGGGGCC	C	UBXN11	12	0.063	INDEL
.	chr1	26282368	ACCGGGAC	A	UBXN11	12	0.063	INDEL
.	chr1	26282374	ACC	A	UBXN11	12	0.063	INDEL
.	chr1	26282374	ACC	*	UBXN11	13	0.068	INDEL
.	chr1	26282377	GGGACTGG	G	UBXN11	12	0.064	INDEL
.	chr1	26345031	GAAATGAG	G	AIM1L	139	0.716	INDEL
.	chr1	26345047	AGCAC	A	AIM1L	142	0.732	INDEL
.	chr1	26345053	GGGGCCCT	G	AIM1L	142	0.732	INDEL
.	chr1	26552881	CTATT	C	RPS6KA1	2	0.01	INDEL
rs4970490	chr1	26553429	T	C	RPS6KA1	194	1	SNP
rs1064196	chr1	26561101	T	C	RPS6KA1	50	0.258	SNP
.	chr1	27350103	G	GGCCGCCG	SYTL1	2	0.01	INDEL
rs2835709	chr1	27373179	AG	A	FCN3	3	0.015	INDEL
rs1384665	chr1	27942318	T	TC	SMPDL3B	51	0.263	INDEL
rs1138605	chr1	27942322	C	CG	SMPDL3B	88	0.454	INDEL
rs1380911	chr1	29202029	G	A	MECR	4	0.021	SNP
rs7151632	chr1	31764679	C	T	ADGRB2	4	0.021	SNP
.	chr1	31937840	A	C	PTP4A2	3	0.015	SNP
rs2000868	chr1	32209141	T	TG	DCDC2B	3	0.015	INDEL
rs2762904	chr1	32668367	T	C	RBBP4	194	1	SNP
.	chr1	32771486	G	GGC	KIAA1522	2	0.01	INDEL
rs6175096	chr1	33021406	C	T	AK2	2	0.01	SNP
rs7606164	chr1	33036775	G	A	AK2	8	0.041	SNP
rs2017159	chr1	33312068	TAGTC	T	A3GALT2	2	0.01	INDEL
.	chr1	33864353	G	GCATGAAA	HMGB4	2	0.01	INDEL
rs1468128	chr1	34761406	TTGTC	T	GJB4	11	0.057	INDEL
.	chr1	34978676	A	C	RP11-244H	2	0.01	SNP
rs7517651	chr1	37722932	C	T	EPHA10	8	0.041	SNP
rs2858014	chr1	37888269	G	T	INPP5B	5	0.026	SNP
rs6688020	chr1	39254140	G	A	RP11-420K	35	0.18	SNP
rs1447077	chr1	39876090	C	CA	TRIT1	34	0.175	INDEL
rs1800205	chr1	40091361	A	G	PPT1	20	0.103	SNP
.	chr1	40247314	CTT	CT	TMCO2	35	0.18	INDEL
.	chr1	40247314	CTT	CTTT	TMCO2	16	0.082	INDEL
.	chr1	40247314	CTT	C	TMCO2	2	0.01	INDEL
.	chr1	40268501	TTG	T	ZMPSTE24	7	0.036	INDEL
rs1500189	chr1	40515573	C	CG	EXO5	7	0.036	INDEL

rs5030799;	chr1	40988656	A	G	CTPS1	2	0.01	SNP
.	chr1	41382209	C	CGGGACG(	FOXO6	11	0.057	INDEL
.	chr1	41382209	C	CGGGACG(	FOXO6	8	0.041	INDEL
rs2069375	chr1	43358418	C	T	RP1-92O14	2	0.01	SNP
rs3215843	chr1	43660291	CA	C	KDM4A	83	0.428	INDEL
rs1740285	chr1	43956950	C	T	IPO13	4	0.021	SNP
.	chr1	43992150	CAG	C	CCDC24	2	0.01	INDEL
rs1458304	chr1	44218326	G	C	DMAP1	2	0.01	SNP
rs3014212	chr1	45592753	G	A	AL355480.:	138	0.711	SNP
rs1622208	chr1	46032703	A	G	MAST2	136	0.701	SNP
rs1736176	chr1	46344998	T	C	NSUN4	45	0.232	SNP
rs6671527	chr1	46615007	G	A	MOB3C	98	0.505	SNP
rs3215983;	chr1	46815074	GAT	G	CYP4B1	33	0.17	INDEL
rs2297810	chr1	46815187	G	A	CYP4B1	34	0.175	SNP
.	chr1	48055190	GAGCAGG(	G	RP4-683M8	64	0.33	INDEL
rs1414092	chr1	48103806	CTG	CTGTGTG	AL356289.:	11	0.057	INDEL
rs1414092	chr1	48103806	CTG	C	AL356289.:	5	0.026	INDEL
rs850763	chr1	48242556	G	T	SLC5A9	45	0.232	SNP
rs3753056	chr1	51302240	GC	G	TTC39A	3	0.015	INDEL
rs3753056	chr1	51302240	GC	GCC	TTC39A	5	0.026	INDEL
rs3753056	chr1	51302240	GC	GCCCCCC	TTC39A	4	0.021	INDEL
rs1444491	chr1	52252667	G	A	DNAJC19P7	2	0.01	SNP
rs6174430	chr1	52860749	A	G	ZYG11A	2	0.01	SNP
rs6176958	chr1	53181024	T	A	RP5-1024G	2	0.01	SNP
rs7918948	chr1	53186284	G	A	RP5-1024G	2	0.01	SNP
rs1427562	chr1	53871370	A	G	YIPF1	2	0.01	SNP
rs3841798	chr1	54139645	T	TGG	CDCP2	28	0.144	INDEL
rs3841798	chr1	54139645	T	TG	CDCP2	21	0.108	INDEL
rs3601310	chr1	54139646	G	GC	CDCP2	136	0.701	INDEL
rs3414824	chr1	54604416	C	T	ACOT11	2	0.01	SNP
rs3591400	chr1	54607242	G	A	ACOT11	3	0.015	SNP
rs4554264	chr1	54608974	G	A	ACOT11	2	0.01	SNP
rs3737390	chr1	54610464	CCACTCCA(	C	FAM151A	15	0.077	INDEL
rs1147990	chr1	54716627	T	A	MROH7-TT	93	0.479	SNP
rs2270002	chr1	54785641	G	A	TTC22	16	0.082	SNP
rs2000622	chr1	54813813	G	GA	LEXM	4	0.021	INDEL
rs540796;2	chr1	55058524	A	G	PCSK9	164	0.845	SNP
rs1056868	chr1	55217930	CAAGT	C	RP11-101C	38	0.196	INDEL
rs5774268	chr1	56249200	T	TC	RP11-504A	193	0.995	INDEL
rs1784859	chr1	56692378	G	A	PRKAA2	11	0.057	SNP
rs4128684	chr1	56940965	G	A	C8B	2	0.01	SNP
rs6177027	chr1	57880271	T	C	DAB1-AS1	69	0.356	SNP
rs3015311	chr1	58896546	C	G	RP4-794H1	92	0.474	SNP
rs1383894	chr1	59789075	A	C	RP4-782L2:	3	0.015	SNP
.	chr1	61406560	A	C	NFIA	2	0.011	SNP
.	chr1	61918051	CAAA	CAA	PATJ	11	0.057	INDEL
.	chr1	61918051	CAAA	CAAAA	PATJ	9	0.046	INDEL

rs4073681	chr1	62038496	G	T	PATJ	46	0.237	SNP
rs2020296!	chr1	62210575	GAACT	G	L1TD1	2	0.01	INDEL
rs1712439!	chr1	63024312	C	T	RP11-5P4.2	29	0.149	SNP
rs7519254	chr1	63170150	T	A	LINC00466	55	0.284	SNP
rs7704313	chr1	63623761	G	A	PGM1	8	0.041	SNP
rs2230588	chr1	64844806	T	C	JAK1	66	0.34	SNP
rs1712706	chr1	64846659	G	A	JAK1	9	0.046	SNP
rs1712719	chr1	64995089	C	G	LINC01359	5	0.026	SNP
rs783036	chr1	66365687	G	A	PDE4B	90	0.464	SNP
rs3816989	chr1	66776404	G	A	TCTEX1D1	31	0.16	SNP
rs2001048	chr1	67093186	CTCATT	C	C1orf141	2	0.01	INDEL
rs4128785	chr1	68138451	G	A	GNG12-AS1	4	0.021	SNP
.	chr1	70363782	G	GT	AL158839.1	59	0.304	INDEL
rs2894178!	chr1	70415987	C	T	CTH	2	0.01	SNP
.	chr1	71203810	A	T	ZRANB2-AS	2	0.01	SNP
rs6424545	chr1	73307365	T	C	LINC01360	111	0.572	SNP
rs7527057	chr1	74706317	T	A	CRYZ	84	0.433	SNP
rs1133891	chr1	74748757	A	G	TYW3	20	0.103	SNP
rs1438218	chr1	77746398	C	T	USP33	3	0.015	SNP
.	chr1	78662885	G	GT	IFI44	86	0.443	INDEL
.	chr1	81514046	CTGAAAGA	C	RP5-837I24	12	0.062	INDEL
rs323942	chr1	83096978	A	G	LINC01362	192	0.99	SNP
rs2001678	chr1	84408479	A	T	DNASE2B	2	0.01	SNP
.	chr1	86445356	CTTTT	CTTT	CLCA2	23	0.124	INDEL
.	chr1	86445356	CTTTT	CTT	CLCA2	6	0.032	INDEL
.	chr1	86445356	CTTTT	CTTTTT	CLCA2	25	0.134	INDEL
.	chr1	86445356	CTTTT	CT	CLCA2	5	0.027	INDEL
rs4128659	chr1	86636910	T	C	CLCA3P	34	0.175	SNP
rs535037	chr1	86650239	T	C	RP4-651E1	105	0.541	SNP
rs101028	chr1	86914313	G	T	44089	41	0.211	SNP
rs6671949	chr1	88807604	G	A	PKN2	5	0.026	SNP
rs2009070	chr1	88983185	A	T	RBMXL1	6	0.031	SNP
.	chr1	88983825	AT	A	RBMXL1	3	0.015	INDEL
rs1485260	chr1	89058073	G	T	GBP1	6	0.031	SNP
rs1048401	chr1	89060283	T	C	GBP1	8	0.041	SNP
.	chr1	89133282	TC	T	GBP7	2	0.01	INDEL
.	chr1	89133286	A	AT	GBP7	4	0.021	INDEL
.	chr1	89263883	GC	G	GBP5	2	0.01	INDEL
.	chr1	89267076	G	GAGTC	GBP5	2	0.01	INDEL
rs1181074	chr1	96006362	G	C	RP11-147C	32	0.165	SNP
rs1209751!	chr1	98210811	G	A	RP5-1070A	87	0.448	SNP
.	chr1	98698690	CT	C	SNX7	12	0.062	INDEL
.	chr1	98698690	CT	CTT	SNX7	3	0.015	INDEL
rs2307130;	chr1	99851033	A	G	AGL	86	0.443	SNP
rs1274758	chr1	1.01E+08	C	T	LINC01349	27	0.139	SNP
rs3835397	chr1	1.01E+08	AG	A	S1PR1	8	0.041	INDEL
rs1925562!	chr1	1.04E+08	T	C	AMY2B	2	0.01	SNP

rs1467077:	chr1	1.04E+08	T	TTCAAA	RP11-364B	36	0.186	INDEL
rs2232016	chr1	1.07E+08	C	T	PRMT6	36	0.186	SNP
rs1454651(	chr1	1.09E+08	G	T	SARS	2	0.01	SNP
rs1163890:	chr1	1.09E+08	T	A	PSRC1	2	0.01	SNP
rs1398495:	chr1	1.09E+08	G	A	MYBPHL	3	0.015	SNP
rs2230350	chr1	1.1E+08	G	A	GNAI3	3	0.015	SNP
rs1390532:	chr1	1.1E+08	CTTT	CTT	LINC01397	2	0.027	INDEL
rs1390532:	chr1	1.1E+08	CTTT	C	LINC01397	4	0.054	INDEL
rs1390532:	chr1	1.1E+08	CTTT	CTTTT	LINC01397	2	0.027	INDEL
rs6674775	chr1	1.1E+08	C	T	LAMTOR5-/	97	0.5	SNP
rs644081	chr1	1.11E+08	C	T	DRAM2	48	0.247	SNP
.	chr1	1.11E+08	CT	C	CHI3L2	13	0.067	INDEL
.	chr1	1.11E+08	CT	CTT	CHI3L2	3	0.015	INDEL
rs1155686:	chr1	1.11E+08	C	T	CHI3L2	33	0.17	SNP
rs13721;40	chr1	1.11E+08	C	A	CHI3L2	56	0.289	SNP
rs13721;40	chr1	1.11E+08	C	T	CHI3L2	32	0.165	SNP
rs3469801(	chr1	1.11E+08	C	CCT	CHIA	81	0.418	INDEL
rs2275253	chr1	1.11E+08	A	G	CHIA	142	0.732	SNP
.	chr1	1.12E+08	G	GT	DDX20	2	0.01	INDEL
rs1760272!	chr1	1.15E+08	G	A	AMPD1	28	0.144	SNP
rs4127556(	chr1	1.16E+08	T	A	RP4-655J12	4	0.021	SNP
rs4129618:	chr1	1.17E+08	G	T	TRIM45	2	0.01	SNP
rs1142866(	chr1	1.2E+08	A	AG	HSD3BP3	146	0.753	INDEL
rs1911897(	chr1	1.22E+08	C	A	RP11-344P	2	0.01	SNP
.	chr1	1.44E+08	GT	G	NBPF15	5	0.026	MIXED
.	chr1	1.45E+08	TG	T	NBPF20	11	0.057	INDEL
rs1157489(	chr1	1.46E+08	G	A	POLR3C	4	0.021	SNP
.	chr1	1.46E+08	CG	C	NUDT17	6	0.031	MIXED
.	chr1	1.46E+08	CG	TG	NUDT17	3	0.015	MIXED
.	chr1	1.46E+08	GTAAAA	G	NUDT17	6	0.031	INDEL
.	chr1	1.46E+08	G	C	ANKRD35	76	0.392	SNP
.	chr1	1.46E+08	G	GC	ANKRD35	2	0.01	INDEL
rs6181639-	chr1	1.46E+08	G	C	NBPF10	79	0.407	SNP
.	chr1	1.47E+08	C	T	NBPF12	6	0.031	SNP
.	chr1	1.47E+08	AGCCTG	A	CHD1L	2	0.01	INDEL
.	chr1	1.48E+08	C	G	ACP6	155	0.799	SNP
.	chr1	1.48E+08	A	T	RP11-495P	67	0.345	SNP
.	chr1	1.49E+08	T	TTTGCCGT	PDE4DIP	4	0.091	INDEL
.	chr1	1.49E+08	T	TA	PDE4DIP	6	0.031	INDEL
.	chr1	1.5E+08	C	CTT	PRPF3	14	0.072	INDEL
.	chr1	1.5E+08	C	CTTT	PRPF3	74	0.381	INDEL
.	chr1	1.5E+08	C	CTTTT	PRPF3	105	0.541	INDEL
.	chr1	1.5E+08	G	GGTATACT.	PRPF3	25	0.129	INDEL
rs1212627:	chr1	1.51E+08	G	A	ADAMTSL4	99	0.51	SNP
rs1030567:	chr1	1.51E+08	G	A	ARNT	69	0.356	SNP
rs267731	chr1	1.51E+08	A	G	ANXA9	6	0.031	SNP
rs4971016	chr1	1.51E+08	G	A	PIP5K1A	122	0.629	SNP

.	chr1	1.52E+08	CTT	CTTT	SNX27	59	0.307	INDEL
.	chr1	1.52E+08	CTT	CT	SNX27	8	0.042	INDEL
.	chr1	1.52E+08	CTT	CTTTT	SNX27	41	0.214	INDEL
.	chr1	1.52E+08	CTT	CTTTTT	SNX27	8	0.042	INDEL
.	chr1	1.52E+08	CTT	C	SNX27	2	0.01	INDEL
.	chr1	1.52E+08	A	ACCTC	RP11-98D1	6	0.031	INDEL
rs1400764(	chr1	1.52E+08	GGGAGAC(	G	OAZ3&TDR	99	0.51	INDEL
.	chr1	1.52E+08	ATT	ATTT	RP11-98D1	118	0.621	INDEL
.	chr1	1.52E+08	ATT	AT	RP11-98D1	3	0.016	INDEL
.	chr1	1.52E+08	ATT	ATTTT	RP11-98D1	4	0.021	INDEL
rs6587625	chr1	1.52E+08	T	C	THEM5	139	0.716	SNP
rs1419508(	chr1	1.52E+08	G	A	HRNR	2	0.01	SNP
rs3406171!	chr1	1.52E+08	AT	A	HRNR	193	0.995	INDEL
16320	chr1	1.52E+08	CACTG	C	FLG	4	0.021	INDEL
rs6181676:	chr1	1.52E+08	G	A	FLG	2	0.01	SNP
rs7547738:	chr1	1.52E+08	A	T	FLG-AS1	4	0.021	SNP
rs1256878(	chr1	1.52E+08	G	T	FLG2	31	0.16	SNP
rs4126850(	chr1	1.53E+08	T	G	LCE1D	14	0.076	SNP
rs1762521!	chr1	1.53E+08	T	C	LCE6A	2	0.01	SNP
rs1139014(	chr1	1.54E+08	AC	A	S100A3	2	0.01	INDEL
rs1162084!	chr1	1.54E+08	G	C	S100A3	7	0.036	SNP
.	chr1	1.54E+08	CTT	CTTT	GATAD2B	32	0.165	INDEL
.	chr1	1.54E+08	CTT	CT	GATAD2B	38	0.196	INDEL
.	chr1	1.54E+08	CTT	C	GATAD2B	6	0.031	INDEL
rs3590220(	chr1	1.54E+08	G	GC	DENND4B	12	0.062	INDEL
rs2275483	chr1	1.54E+08	T	TGC	DENND4B	10	0.052	MIXED
.	chr1	1.55E+08	CGCAGCAT	C	EFNA3	2	0.01	INDEL
.	chr1	1.55E+08	A	C	EFNA3	3	0.015	SNP
.	chr1	1.55E+08	A	*	EFNA3	2	0.01	SNP
.	chr1	1.55E+08	G	C	EFNA3	3	0.015	SNP
.	chr1	1.55E+08	G	*	EFNA3	2	0.01	SNP
rs4072037	chr1	1.55E+08	C	T	MUC1	82	0.423	SNP
.	chr1	1.56E+08	CTT	C	RP11-243J1	8	0.041	INDEL
.	chr1	1.56E+08	CTT	CT	RP11-243J1	34	0.175	INDEL
.	chr1	1.56E+08	CTT	CTTT	RP11-243J1	11	0.057	INDEL
.	chr1	1.56E+08	CGTGGGG(	C	MEX3A	3	0.015	INDEL
.	chr1	1.56E+08	CT	C	RHBG	20	0.103	INDEL
.	chr1	1.56E+08	CT	CTT	RHBG	8	0.041	INDEL
.	chr1	1.56E+08	ATT	ATTT	RHBG	12	0.062	INDEL
.	chr1	1.56E+08	ATT	ATTTT	RHBG	65	0.335	INDEL
.	chr1	1.56E+08	ATT	A	RHBG	9	0.046	INDEL
.	chr1	1.56E+08	ATT	AT	RHBG	76	0.392	INDEL
rs2245623	chr1	1.56E+08	G	A	RHBG	78	0.402	SNP
rs7159193!	chr1	1.56E+08	T	TC	RHBG	134	0.691	INDEL
rs1408534:	chr1	1.57E+08	GC	G	TTC24	6	0.031	INDEL
rs7255912!	chr1	1.57E+08	A	AAC	GPATCH4	194	1	INDEL
rs1114398(	chr1	1.57E+08	C	T	GPATCH4	3	0.015	SNP

rs6334;292	chr1	1.57E+08	G	A	NTRK1	40	0.206	SNP
rs1429575	chr1	1.57E+08	C	T	ARHGEF11	2	0.01	SNP
rs2001675	chr1	1.58E+08	T	TG	FCRL5	2	0.01	INDEL
rs2758628	chr1	1.58E+08	T	C	RP4-801G2	91	0.469	SNP
rs1428701	chr1	1.58E+08	C	A	CD5L	2	0.01	SNP
rs1490193	chr1	1.58E+08	G	A	CD1A	2	0.01	SNP
rs863362	chr1	1.59E+08	C	T	OR10X1	94	0.485	SNP
rs1434843	chr1	1.59E+08	C	T	PYHIN1	2	0.01	SNP
rs3737523	chr1	1.59E+08	C	T	IFI16	3	0.015	SNP
rs2276405	chr1	1.59E+08	C	T	AIM2	2	0.01	SNP
rs1141954	chr1	1.59E+08	G	GT	OR10J4	190	0.979	INDEL
rs1240954	chr1	1.59E+08	T	A	OR10J1	29	0.149	SNP
rs1996659	chr1	1.6E+08	C	G	DUSP23	3	0.015	SNP
rs1129923	chr1	1.6E+08	G	A	DUSP23	26	0.134	SNP
rs4301626	chr1	1.6E+08	T	C	FCRL6	13	0.067	SNP
rs6182316	chr1	1.6E+08	C	T	FCRL6	26	0.134	SNP
rs1421000	chr1	1.61E+08	AC	A	CD48	9	0.046	INDEL
rs7271286	chr1	1.61E+08	C	T	F11R	2	0.01	SNP
.	chr1	1.61E+08	AC	A	RP11-544M	10	0.052	INDEL
.	chr1	1.61E+08	CTTATTTAT	CTTATTTA	AL591806.	2	0.01	INDEL
.	chr1	1.61E+08	CTTATTTAT	CTTATTTAT	AL591806.	26	0.134	INDEL
.	chr1	1.61E+08	CTTATTTAT	CTTATTTAT	AL591806.	27	0.139	INDEL
.	chr1	1.61E+08	CTTATTTAT	CTTATTTAT	AL591806.	3	0.015	INDEL
rs7744410	chr1	1.61E+08	G	T	NECTIN4	4	0.021	SNP
rs3737786	chr1	1.61E+08	A	G	NECTIN4	3	0.015	SNP
rs9427397;	chr1	1.62E+08	C	T	FCGR2A	21	0.108	SNP
rs6374909	chr1	1.62E+08	C	T	HSPA6	24	0.124	SNP
rs7627741	chr1	1.62E+08	A	G	FCGR2C	25	0.129	SNP
rs1063178	chr1	1.62E+08	C	T	DUSP12	60	0.309	SNP
rs1169744	chr1	1.62E+08	C	A	C1orf111	3	0.015	SNP
rs3494280	chr1	1.63E+08	A	G	UAP1	2	0.01	SNP
rs6695491	chr1	1.63E+08	G	T	RP11-267N	48	0.247	SNP
rs3750540	chr1	1.63E+08	G	T	NUF2	4	0.021	SNP
rs9333378	chr1	1.66E+08	G	A	MGST3	122	0.629	SNP
rs1155330	chr1	1.66E+08	G	A	UCK2	36	0.186	SNP
.	chr1	1.66E+08	AT	A	RP11-375H	15	0.077	INDEL
.	chr1	1.66E+08	AT	ATT	RP11-375H	3	0.015	INDEL
rs1747244	chr1	1.67E+08	T	C	FMO9P	28	0.144	SNP
rs1890128	chr1	1.68E+08	G	A	RCSD1	7	0.036	SNP
.	chr1	1.68E+08	G	GCCGAGG	MPZL1	3	0.015	INDEL
rs7757417	chr1	1.68E+08	G	A	MPZL1	2	0.01	SNP
rs203849	chr1	1.68E+08	A	G	ADCY10	92	0.474	SNP
rs2006649	chr1	1.68E+08	A	AG	GPR161	6	0.031	INDEL
rs2021668	chr1	1.7E+08	C	T	C1orf112	2	0.01	SNP
rs2836986	chr1	1.71E+08	TG	T	FMO2	8	0.041	INDEL
769222	chr1	1.71E+08	C	T	FMO2	193	0.995	SNP
rs2234889;	chr1	1.71E+08	C	CT	FMO2	15	0.077	INDEL

rs6173097	chr1	1.72E+08	A	G	MYOC	6	0.031	SNP
rs1466066	chr1	1.72E+08	C	A	MYOC	2	0.01	SNP
rs4131089	chr1	1.72E+08	G	A	C1orf105	7	0.036	SNP
rs1997980	chr1	1.74E+08	GAGGCA	G	ZBTB37	30	0.155	INDEL
rs1481842	chr1	1.75E+08	TTG	T	KIAA0040	2	0.01	INDEL
rs6019588	chr1	1.75E+08	TC	T	KIAA0040	92	0.474	INDEL
.	chr1	1.75E+08	TTCTTCTTG	T	KIAA0040	92	0.474	INDEL
rs1214115	chr1	1.79E+08	C	T	C1orf220	73	0.376	SNP
rs2245425;	chr1	1.8E+08	G	A	TOR1AIP1	124	0.639	SNP
rs1573247	chr1	1.82E+08	C	T	LINC01344	191	0.985	SNP
rs2274064;	chr1	1.84E+08	T	C	NCF2	84	0.433	SNP
rs1686125	chr1	1.84E+08	A	G	ARPC5	7	0.036	SNP
rs1135492	chr1	1.85E+08	C	T	GS1-279B7	2	0.01	SNP
.	chr1	1.86E+08	TATAG	TATAGATA	AL596220.	28	0.144	INDEL
.	chr1	1.86E+08	TATAG	TATAGATA	AL596220.	50	0.258	INDEL
.	chr1	1.86E+08	TATAG	TATAGATA	AL596220.	12	0.062	INDEL
.	chr1	1.86E+08	T	TAG	AL596220.	10	0.052	INDEL
rs3124816	chr1	1.86E+08	T	TAG	AL596220.	9	0.046	MIXED
rs3124816	chr1	1.86E+08	T	G	AL596220.	4	0.021	MIXED
rs1482125	chr1	1.93E+08	A	G	GLRX2	2	0.01	SNP
rs387111	chr1	1.97E+08	G	A	CFHR3	43	0.229	SNP
rs481759	chr1	1.97E+08	T	C	CFHR3	29	0.163	SNP
rs3514175	chr1	1.97E+08	GT	G	CFHR3	25	0.142	INDEL
294541;40	chr1	1.97E+08	C	CA	CFHR5	2	0.01	INDEL
294541;40	chr1	1.97E+08	C	CAA	CFHR5	5	0.026	INDEL
rs1213496	chr1	1.97E+08	G	C	F13B	36	0.186	SNP
rs7273191	chr1	1.98E+08	G	A	NEK7	18	0.093	SNP
rs1134462	chr1	2.01E+08	C	CT	RP11-92G1	108	0.557	INDEL
rs1134462	chr1	2.01E+08	C	CTT	RP11-92G1	3	0.015	INDEL
.	chr1	2.01E+08	CTT	CT	RP11-92G1	29	0.154	INDEL
.	chr1	2.01E+08	CTT	C	RP11-92G1	5	0.027	INDEL
.	chr1	2.01E+08	CTT	CTTTTTTTT	RP11-92G1	9	0.048	INDEL
.	chr1	2.01E+08	CTT	CTTTTTTTT	RP11-92G1	5	0.027	INDEL
rs3501653	chr1	2.01E+08	GC	G	LAD1	7	0.036	INDEL
780294	chr1	2.01E+08	ACC	*	LAD1	17	0.088	INDEL
780294	chr1	2.01E+08	ACC	A	LAD1	7	0.036	INDEL
rs5674382	chr1	2.02E+08	T	G	PTPRVP	14	0.072	SNP
rs7435547	chr1	2.02E+08	TG	T	LGR6	17	0.088	INDEL
rs14451	chr1	2.02E+08	T	C	UBE2T	90	0.464	SNP
rs3458326	chr1	2.02E+08	CT	CTT	CYCSP4	3	0.015	INDEL
rs3458326	chr1	2.02E+08	CT	C	CYCSP4	106	0.546	INDEL
rs1892164	chr1	2.03E+08	G	A	KDM5B	140	0.722	SNP
rs1063529	chr1	2.03E+08	G	GGC	PPFIA4	46	0.237	INDEL
rs1492005	chr1	2.03E+08	TGGGATGC	T	ADORA1&F	112	0.577	INDEL
rs4950927	chr1	2.03E+08	A	G	CHI3L1	194	1	SNP
rs880633	chr1	2.03E+08	T	C	CHI3L1	108	0.557	SNP
rs1501923	chr1	2.03E+08	C	CAGACCAT	CHIT1	29	0.149	INDEL

rs4126427	chr1	2.04E+08	A	G	LINC00303	62	0.32	SNP
rs7683424	chr1	2.04E+08	C	T	REN	10	0.052	SNP
rs7174562	chr1	2.04E+08	CT	C	KISS1	47	0.242	INDEL
rs1172147	chr1	2.05E+08	G	A	TMCC2	72	0.371	SNP
rs1205916	chr1	2.05E+08	C	T	KLHDC8A	43	0.222	SNP
.	chr1	2.06E+08	T	*	RP11-6B6.	2	0.01	SNP
.	chr1	2.06E+08	T	C	RP11-6B6.	2	0.01	SNP
.	chr1	2.06E+08	GAC	G	C1orf186	2	0.01	INDEL
rs3452686	chr1	2.07E+08	C	T	MAPKAPK2	2	0.01	SNP
rs2490257	chr1	2.07E+08	T	C	CD55	194	1	SNP
rs3043874	chr1	2.08E+08	GA	GAA	CD34	107	0.552	INDEL
rs3043874	chr1	2.08E+08	GA	GAAA	CD34	10	0.052	INDEL
.	chr1	2.1E+08	CT	C	AL022397.	12	0.062	INDEL
.	chr1	2.1E+08	CT	CTT	AL022397.	5	0.026	INDEL
rs701941	chr1	2.1E+08	G	A	ST13P19	16	0.082	SNP
rs701929;4	chr1	2.12E+08	A	G	NEK2	104	0.536	SNP
rs701923	chr1	2.12E+08	C	T	RP11-122M	148	0.763	SNP
rs6661565	chr1	2.12E+08	G	A	RP11-122M	31	0.16	SNP
rs6173692	chr1	2.13E+08	C	G	TATDN3	26	0.134	SNP
rs7809500	chr1	2.13E+08	G	A	TATDN3	3	0.015	SNP
.	chr1	2.16E+08	C	CTAG	KCTD3	3	0.015	INDEL
rs4130304	chr1	2.18E+08	G	C	SPATA17	21	0.108	SNP
rs1774080	chr1	2.18E+08	T	A	LINC00210	140	0.722	SNP
709022	chr1	2.18E+08	GC	G	RRP15	3	0.015	INDEL
rs7550232	chr1	2.18E+08	A	C	TGFB2-AS1	3	0.015	SNP
rs1475332	chr1	2.2E+08	C	T	BPNT1	5	0.026	SNP
rs3693757	chr1	2.2E+08	G	A	BPNT1	2	0.01	SNP
rs2005106	chr1	2.2E+08	C	CT	RP11-410C	12	0.062	INDEL
rs1427304	chr1	2.22E+08	C	T	RP11-378J	13	0.067	SNP
rs5744168;	chr1	2.23E+08	G	A	TLR5	7	0.036	SNP
rs2230083	chr1	2.24E+08	A	G	CAPN2	2	0.01	SNP
rs17598	chr1	2.24E+08	C	T	CAPN2	13	0.067	SNP
rs1505098	chr1	2.24E+08	C	T	NVL	3	0.015	SNP
rs1213057	chr1	2.25E+08	T	TAG	DNAH14	4	0.021	MIXED
rs1213057	chr1	2.25E+08	T	G	DNAH14	12	0.062	MIXED
rs1443398	chr1	2.25E+08	CAAAG	C	DNAH14	14	0.072	INDEL
.	chr1	2.26E+08	CT	C	SRP9	23	0.119	INDEL
.	chr1	2.26E+08	CT	CTT	SRP9	33	0.17	INDEL
.	chr1	2.26E+08	AAAAC	A	TMEM63A	9	0.047	INDEL
.	chr1	2.26E+08	AAAC	A	TMEM63A	32	0.167	INDEL
.	chr1	2.26E+08	AAAC	*	TMEM63A	8	0.042	INDEL
rs1387770	chr1	2.26E+08	AAC	*	TMEM63A	38	0.198	INDEL
rs1387770	chr1	2.26E+08	AAC	A	TMEM63A	111	0.578	INDEL
rs3755158	chr1	2.26E+08	AC	*	TMEM63A	143	0.745	INDEL
rs3755158	chr1	2.26E+08	AC	A	TMEM63A	35	0.182	INDEL
rs1079932	chr1	2.26E+08	C	*	TMEM63A	177	0.912	SNP
rs1079932	chr1	2.26E+08	C	A	TMEM63A	11	0.057	SNP

rs1805404	chr1	2.26E+08	G	A	PARP1	41	0.211	SNP
rs4130312!	chr1	2.27E+08	C	T	COQ8A	3	0.015	SNP
rs5595823:	chr1	2.27E+08	C	T	COQ8A	2	0.01	SNP
rs1784992:	chr1	2.27E+08	C	T	COQ8A	2	0.01	SNP
rs12593;12	chr1	2.27E+08	C	T	COQ8A	84	0.433	SNP
rs3738725;	chr1	2.27E+08	T	C	COQ8A	93	0.479	SNP
rs1180030!	chr1	2.28E+08	G	T	C1orf145	53	0.273	SNP
rs1174841:	chr1	2.28E+08	G	A	OBSCN	2	0.01	SNP
rs3795786	chr1	2.28E+08	A	T	OBSCN	3	0.015	SNP
rs3738073	chr1	2.29E+08	C	T	RHOU	50	0.258	SNP
rs1403115!	chr1	2.29E+08	TTC	T	TMEM78	32	0.167	INDEL
.	chr1	2.3E+08	C	T	GALNT2	2	0.01	SNP
rs2282319	chr1	2.31E+08	C	T	CAPN9	67	0.345	SNP
rs3828126	chr1	2.31E+08	T	C	CAPN9	57	0.294	SNP
rs1439919(	chr1	2.31E+08	G	A	EGLN1	2	0.01	SNP
rs6692987:	chr1	2.35E+08	T	A	ARID4B	18	0.093	SNP
rs650692	chr1	2.36E+08	A	G	EDARADD	58	0.299	SNP
rs1126407	chr1	2.37E+08	T	A	LGALS8	142	0.732	SNP
rs1041935	chr1	2.37E+08	C	T	LGALS8	142	0.732	SNP
.	chr1	2.37E+08	GTTCCTGG	G	RP11-385F	2	0.01	INDEL
rs2273865	chr1	2.37E+08	T	A	LGALS8	4	0.021	SNP
rs2243525	chr1	2.37E+08	G	C	LGALS8	154	0.794	SNP
rs1135510(	chr1	2.37E+08	TC	T	ACTN2	137	0.706	INDEL
.	chr1	2.4E+08	C	CA	FMN2	2	0.01	INDEL
rs7244456:	chr1	2.4E+08	CA	C	FMN2	2	0.01	MIXED
rs4149909	chr1	2.42E+08	A	G	EXO1	8	0.041	SNP
rs5581323:	chr1	2.42E+08	C	T	MAP1LC3C	5	0.026	SNP
rs5676849(	chr1	2.42E+08	A	G	FAM166AP	12	0.062	SNP
rs5564821(	chr1	2.44E+08	T	C	ADSS	5	0.026	SNP
rs1053664!	chr1	2.45E+08	GGC	G	EFCAB2	109	0.562	INDEL
rs1458354:	chr1	2.45E+08	T	TCCTCC	EFCAB2	110	0.567	INDEL
rs1138164!	chr1	2.45E+08	C	CTTATT	EFCAB2	12	0.062	INDEL
.	chr1	2.47E+08	CGG	C	ZNF669	2	0.01	INDEL
rs3530598(	chr1	2.47E+08	GA	G	OR2B11	64	0.33	INDEL
rs5604307(	chr1	2.48E+08	G	A	GCSAML	11	0.057	SNP
rs1126966	chr1	2.48E+08	G	GA	OR2W3	26	0.134	INDEL
rs1088828:	chr1	2.48E+08	T	A	OR2L8	186	0.959	SNP
rs1133945:	chr1	2.48E+08	CT	C	OR2T12	28	0.144	INDEL
.	chr1	2.49E+08	T	TCA	OR2T35	2	0.018	INDEL
rs1430105	chr1	2.49E+08	TCAGCACG	T	OR2T35	176	0.907	INDEL
rs2006698(	chr1	2.49E+08	T	TC	OR2T27	3	0.015	INDEL
rs1018105:	chr2	242800	T	C	SH3YL1	73	0.376	SNP
.	chr2	306524	CAGCGAG(	TAGCGAG(	AC079779.	4	0.025	MIXED
.	chr2	306524	CAGCGAG(	C	AC079779.	2	0.013	MIXED
rs1898095:	chr2	312952	C	A	AC079779.	2	0.01	SNP
rs1126672!	chr2	313885	CTGATTTG(	C	AC079779.	164	0.845	INDEL
rs1118395!	chr2	1669834	C	CTAAA	PXDN	42	0.216	INDEL

rs7595702	chr2	3208933	C	A	TSSC1	84	0.433	SNP
rs7716583	chr2	3769143	C	T	DCDC2C	18	0.093	SNP
rs4395236	chr2	5973430	G	A	LINC01105	9	0.046	SNP
rs1169469	chr2	6633788	C	T	MIR7515H	15	0.077	SNP
rs1117976	chr2	6649720	AGTCTTAG	A	MIR7515H	107	0.552	INDEL
rs7558716	chr2	7076881	C	T	AC019048.	29	0.149	SNP
rs1092953	chr2	8578568	A	G	AC011747.	63	0.325	SNP
rs1124627	chr2	9412254	A	AT	ITGB1BP1	70	0.361	INDEL
rs1269239	chr2	9693569	T	G	RP11-521D	68	0.351	SNP
.	chr2	9849441	T	TA	TAF1B	4	0.021	INDEL
rs7578338	chr2	10719033	A	G	AC092687.	51	0.263	SNP
rs4807	chr2	10790777	T	C	PDIA6	62	0.32	SNP
rs1106184	chr2	10823401	T	C	PDIA6	131	0.675	SNP
rs4669643	chr2	10884833	G	A	RP11-245G	2	0.01	SNP
rs1406458	chr2	11129453	C	CTAAA	AC062028.	65	0.335	INDEL
rs6139988	chr2	11133332	A	AC	C2orf50	3	0.015	INDEL
rs1339461	chr2	11587381	G	A	GREB1	79	0.407	SNP
rs3408012	chr2	15238684	C	G	NBAS	2	0.01	SNP
rs1746695	chr2	15599169	T	C	DDX1	5	0.026	SNP
.	chr2	17567353	CTT	CTTTT	VSNL1	78	0.411	INDEL
.	chr2	17567353	CTT	CTTTTT	VSNL1	15	0.079	INDEL
.	chr2	17567353	CTT	CTTTTTT	VSNL1	40	0.211	INDEL
.	chr2	17567353	CTT	C	VSNL1	4	0.021	INDEL
.	chr2	17567353	CTT	CTTT	VSNL1	18	0.095	INDEL
.	chr2	17567353	CTT	CTTTTTTT	VSNL1	2	0.011	INDEL
rs1499369	chr2	17781726	CAAGTT	C	GEN1	32	0.165	INDEL
rs6174259	chr2	18584890	G	T	NT5C1B	18	0.093	SNP
rs3475002	chr2	20598246	CT	C	HS1BP3	35	0.18	INDEL
rs7498967	chr2	20598263	C	T	HS1BP3	4	0.021	SNP
rs1450787	chr2	24079505	G	C	TP53I3	4	0.021	SNP
.	chr2	24164308	GC	G	FAM228B	13	0.067	INDEL
rs1898101	chr2	24165385	C	A	FAM228B	3	0.015	SNP
rs6119301	chr2	24179258	C	CAA	FAM228A	181	0.933	INDEL
.	chr2	24335062	CAAAAAAA	CAAAA	RPL36AP13	131	0.682	INDEL
.	chr2	24335062	CAAAAAAA	CAAA	RPL36AP13	34	0.177	INDEL
.	chr2	24335062	CAAAAAAA	CA	RPL36AP13	4	0.021	INDEL
.	chr2	24335062	CAAAAAAA	CAA	RPL36AP13	8	0.042	INDEL
.	chr2	24335062	CAAAAAAA	CAAAAA	RPL36AP13	12	0.063	INDEL
rs4665809	chr2	26028492	A	G	TRMT112P	147	0.758	SNP
rs1141313	chr2	27238100	A	G	CAD	44	0.227	SNP
rs1446927	chr2	27238500	T	C	CAD	2	0.01	SNP
rs6175764	chr2	27238628	C	T	CAD	2	0.01	SNP
rs3739092	chr2	27239208	C	T	CAD	34	0.175	SNP
rs1409486	chr2	27858572	C	G	RBKS	4	0.021	SNP
rs1503025	chr2	28310080	A	C	BRE	7	0.036	SNP
rs1017611	chr2	28387760	T	C	FLJ31356	35	0.18	SNP
rs7570234	chr2	29115150	G	T	CLIP4	62	0.32	SNP

rs1569156	chr2	29221229	C	T	ALK	194	1	SNP
rs207440;2	chr2	31339546	C	T	XDH	13	0.067	SNP
rs2295475;	chr2	31366981	G	A	XDH	53	0.273	SNP
rs1732322!	chr2	31370399	T	C	XDH	5	0.026	SNP
rs4407290;	chr2	31383804	G	A	XDH	6	0.031	SNP
rs4557503:	chr2	31387835	C	T	XDH	8	0.041	SNP
rs249097	chr2	34297467	A	G	LINC01317	193	0.995	SNP
rs848637	chr2	36581589	T	G	FEZ2	122	0.629	SNP
rs2307469	chr2	37109260	G	C	EIF2AK2	10	0.052	SNP
rs1510341	chr2	37253176	A	AT	PRKD3	13	0.067	INDEL
rs4670696	chr2	37372820	A	C	QPCT	9	0.046	SNP
rs7233236	chr2	37952271	CTG	C	RMDN2	82	0.423	INDEL
rs7920436:	chr2	38071251	C	T	CYP1B1	2	0.01	SNP
rs151257	chr2	38098758	G	T	CYP1B1-AS	46	0.237	SNP
rs6741892	chr2	38689828	A	T	GALM	9	0.046	SNP
rs1019229:	chr2	41938614	C	T	C2orf91	49	0.253	SNP
rs3816183	chr2	42788579	T	C	HAAO	143	0.737	SNP
.	chr2	42798208	CTTTTTTT	CTTTTT	FTOP1	31	0.17	INDEL
.	chr2	42798208	CTTTTTTT	CTTTTTT	FTOP1	22	0.121	INDEL
.	chr2	42798208	CTTTTTTT	CTTTTTTTT	FTOP1	10	0.055	INDEL
.	chr2	42798208	CTTTTTTT	CT	FTOP1	28	0.154	INDEL
.	chr2	42798208	CTTTTTTT	C	FTOP1	4	0.022	INDEL
.	chr2	42798208	CTTTTTTT	CTTTTTTTT	FTOP1	21	0.115	INDEL
.	chr2	43775692	CT	CTT	DYNC2LI1	8	0.042	INDEL
.	chr2	43775692	CT	C	DYNC2LI1	4	0.021	INDEL
rs6117982	chr2	44301128	GT	G	SLC3A1	24	0.124	INDEL
.	chr2	45845375	ATT	A	PRKCE	32	0.165	INDEL
.	chr2	45845375	ATT	AT	PRKCE	112	0.577	INDEL
.	chr2	46480744	C	CGG	TMEM247	5	0.026	INDEL
rs1703652	chr2	47345224	G	C	EPCAM	25	0.129	SNP
rs1440776:	chr2	47369714	C	CG	EPCAM	12	0.062	INDEL
.	chr2	47907303	AT	A	AC079807.	6	0.031	INDEL
rs3732050	chr2	51202315	C	A	NRXN1	59	0.304	SNP
rs1704303:	chr2	52495838	T	A	AC139712.	11	0.057	SNP
rs1363061	chr2	54057304	G	A	ACYP2	23	0.119	SNP
rs1149311!	chr2	55180508	A	T	CLHC1	8	0.041	SNP
rs2016817	chr2	55643394	C	T	PNPT1	2	0.01	SNP
rs2627765;	chr2	55656182	G	T	PNPT1	112	0.577	SNP
rs1128753!	chr2	61074967	CT	C	KIAA1841	13	0.067	INDEL
rs1128753!	chr2	61074967	CT	CTT	KIAA1841	17	0.088	INDEL
rs7470675	chr2	61081434	ATT	AT	KIAA1841	14	0.072	INDEL
rs7470675	chr2	61081434	ATT	ATTT	KIAA1841	15	0.077	INDEL
rs7470675	chr2	61081434	ATT	A	KIAA1841	2	0.01	INDEL
rs1422695!	chr2	61134190	TG	T	KIAA1841	17	0.088	INDEL
.	chr2	62146282	TA	T	COMMD1	8	0.041	INDEL
.	chr2	62146282	TA	TAA	COMMD1	5	0.026	INDEL
rs7576859	chr2	64189016	C	T	LINC00309	7	0.036	SNP

rs1147408:	chr2	64201360	T	C	LINC00309	6	0.031	SNP
rs3464198:	chr2	65899310	G	T	AC074391.	45	0.232	SNP
rs4671787	chr2	67181225	T	G	AC023115.	92	0.474	SNP
rs6057312:	chr2	68365414	C	A	AC015969.	2	0.01	SNP
rs1018725(	chr2	68922849	A	G	GKN3P	15	0.077	SNP
rs4453725;	chr2	69431994	A	T	NFU1	77	0.397	SNP
rs1338383(	chr2	70831175	T	C	CD207	11	0.057	SNP
rs3213749	chr2	70831775	G	T	CD207	11	0.057	SNP
rs6712863	chr2	70832906	A	G	CD207	19	0.098	SNP
rs1168164:	chr2	70935956	T	C	ATP6V1B1	78	0.402	SNP
rs1122919!	chr2	70943568	GGA	G	ATP6V1B1	2	0.01	INDEL
.	chr2	70943656	A	C	ATP6V1B1	2	0.01	SNP
.	chr2	70943657	G	C	ATP6V1B1	2	0.01	SNP
rs1383977(	chr2	70994746	C	T	AC007040.	2	0.01	SNP
rs2287328	chr2	71070565	T	C	NAGK	24	0.124	SNP
rs1043701	chr2	71077608	G	A	NAGK	26	0.134	SNP
rs5832083	chr2	72465143	A	AAG	EXOC6B	191	0.985	INDEL
rs4852974	chr2	73700804	A	G	NAT8B	151	0.778	SNP
rs7700035:	chr2	73850429	G	T	STAMBP	2	0.01	SNP
rs1019465	chr2	74887981	A	G	HK2	118	0.608	SNP
rs7609047	chr2	79319971	A	T	CTNNA2	42	0.216	SNP
rs4240199	chr2	85322745	A	G	TGOLN2	116	0.598	SNP
rs6262345:	chr2	85402143	C	T	CAPG	3	0.015	SNP
rs7141183:	chr2	85621327	G	A	USP39	2	0.01	SNP
rs1805164;	chr2	88579613	T	C	EIF2AK3	56	0.289	SNP
rs1883313:	chr2	89196390	C	T	IGKV2-26	3	0.015	SNP
.	chr2	89213535	A	AC	IGKV1-27	3	0.015	INDEL
rs1839493:	chr2	90173128	G	T	IGKV3D-11	10	0.052	SNP
rs3744289(	chr2	95859918	ATTTC	A	ANKRD36C	3	0.015	INDEL
rs3171927;	chr2	96287095	A	G	SNRNP200	59	0.304	SNP
rs1388747.	chr2	96687723	G	GGGGAA	FER1L5	8	0.041	INDEL
rs1414473(	chr2	97113750	GC	G	ANKRD36	35	0.18	INDEL
rs1478346(	chr2	97669801	A	G	LINC01125	2	0.01	SNP
rs3192177;	chr2	97738048	G	A	ZAP70	74	0.381	SNP
rs6215492:	chr2	98162976	G	T	VWA3B	11	0.057	SNP
rs7585747	chr2	98602608	T	C	COA5	32	0.165	SNP
rs1062847	chr2	98609710	T	A	UNC50	32	0.165	SNP
rs3087399	chr2	99438696	T	C	REV1	22	0.113	SNP
rs2017546!	chr2	99593872	CG	C	AFF3	5	0.026	INDEL
rs1995572:	chr2	99593875	CTGAGG	C	AFF3	5	0.026	INDEL
rs5902902(	chr2	1E+08	A	G	NMS	2	0.01	SNP
rs1513629	chr2	1.01E+08	C	A	AC092168.	154	0.794	SNP
rs1776645.	chr2	1.02E+08	T	G	IL1R1	4	0.021	SNP
rs7095350.	chr2	1.05E+08	TTTCCATG	T	TGFBRAP1	134	0.691	INDEL
rs2005274(	chr2	1.06E+08	C	A	RGPD3	2	0.01	SNP
rs1120502(	chr2	1.08E+08	G	A	SULT1C3	3	0.015	SNP
rs2219078	chr2	1.08E+08	G	A	SULT1C3	43	0.222	SNP

rs1114554	chr2	1.08E+08	T	C	SULT1C3	3	0.015	SNP
.	chr2	1.08E+08	AT	A	SULT1C2	8	0.041	INDEL
.	chr2	1.08E+08	AT	ATT	SULT1C2	12	0.062	INDEL
rs2011350	chr2	1.09E+08	C	T	LIMS1	38	0.196	SNP
rs1423704	chr2	1.09E+08	G	T	LIMS1	9	0.046	SNP
rs5636191	chr2	1.09E+08	T	TA	LIMS1-AS1	90	0.464	INDEL
rs5833305	chr2	1.09E+08	C	CT	CCDC138	47	0.242	INDEL
rs2009150	chr2	1.09E+08	GCT	G	AC109344.	7	0.036	INDEL
rs1894293	chr2	1.11E+08	G	T	ACOXL	2	0.01	SNP
rs724710	chr2	1.11E+08	T	C	BCL2L11	136	0.701	SNP
rs7293624	chr2	1.12E+08	G	A	ANAPC1	36	0.186	SNP
.	chr2	1.12E+08	CA	CAAA	AC093166.	77	0.397	INDEL
.	chr2	1.12E+08	CA	CAAAA	AC093166.	37	0.191	INDEL
.	chr2	1.12E+08	CA	CAA	AC093166.	5	0.026	INDEL
.	chr2	1.12E+08	CA	C	AC093166.	4	0.021	INDEL
rs7778175	chr2	1.12E+08	A	G	MERTK	37	0.191	SNP
rs2003638	chr2	1.12E+08	G	C	MERTK	2	0.01	SNP
rs1302160	chr2	1.12E+08	A	C	RGPD8	25	0.129	SNP
rs1388962	chr2	1.13E+08	TTCCGGCG	T	POLR1B	4	0.021	INDEL
rs2015283	chr2	1.14E+08	CG	C	RABL2A	2	0.01	INDEL
.	chr2	1.17E+08	CTTT	CT	AC092170.	44	0.229	INDEL
.	chr2	1.17E+08	CTTT	CTT	AC092170.	32	0.167	INDEL
.	chr2	1.17E+08	CTTT	C	AC092170.	7	0.036	INDEL
.	chr2	1.19E+08	CACACACA	C	EN1	2	0.01	INDEL
rs1701337	chr2	1.19E+08	G	A	STEAP3	3	0.015	SNP
rs3731608	chr2	1.19E+08	C	G	DBI	37	0.191	SNP
rs2001656	chr2	1.2E+08	G	C	CFAP221	30	0.155	SNP
rs3214717	chr2	1.2E+08	A	AT	PTPN4	124	0.639	INDEL
.	chr2	1.2E+08	A	AT	PTPN4	5	0.026	INDEL
.	chr2	1.2E+08	CT	C	PTPN4	31	0.16	INDEL
.	chr2	1.2E+08	CT	CTT	PTPN4	6	0.031	INDEL
rs2304560	chr2	1.21E+08	A	G	CLASP1	9	0.046	SNP
rs3485745	chr2	1.24E+08	C	G	AC079154.	12	0.062	SNP
rs6174278	chr2	1.27E+08	A	G	AC114783.	26	0.134	SNP
rs6173022	chr2	1.27E+08	C	T	AC114783.	26	0.134	SNP
rs2276683	chr2	1.27E+08	G	C	AC068282.	163	0.84	SNP
rs1021069	chr2	1.28E+08	T	C	POLR2D	32	0.165	SNP
.	chr2	1.3E+08	CA	C	POTEI	2	0.01	INDEL
rs7226200	chr2	1.31E+08	AC	A	MZT2A	17	0.088	INDEL
rs1348793	chr2	1.35E+08	T	C	CCNT2	63	0.325	SNP
.	chr2	1.36E+08	GT	G	R3HDM1	9	0.046	INDEL
.	chr2	1.36E+08	GT	GTT	R3HDM1	19	0.098	INDEL
rs9013	chr2	1.43E+08	A	G	KYNU	24	0.124	SNP
.	chr2	1.45E+08	AT	A	TEX41	7	0.036	INDEL
.	chr2	1.45E+08	AT	ATT	TEX41	16	0.082	INDEL
rs1112575	chr2	1.48E+08	GT	G	MBD5	5	0.026	INDEL
rs1112575	chr2	1.48E+08	GT	GTT	MBD5	8	0.041	INDEL

rs1046668	chr2	1.51E+08	A	G	TNFAIP6	35	0.18	SNP
.	chr2	1.52E+08	T	C	ARL5A	2	0.01	SNP
587435	chr2	1.53E+08	C	CA	FMNL2	2	0.01	INDEL
rs7097758	chr2	1.53E+08	ATTTTTTTT	A	AC079150.	166	0.856	INDEL
rs7097758	chr2	1.53E+08	ATTTTTTTT	ATTTTTTTT	AC079150.	2	0.01	INDEL
rs1127852	chr2	1.57E+08	C	A	CYTIP	3	0.015	SNP
rs1146031	chr2	1.58E+08	C	T	ACVR1	190	0.979	SNP
rs1136850	chr2	1.58E+08	G	GA	UPP2	7	0.036	INDEL
rs1509487	chr2	1.58E+08	C	G	UPP2	2	0.01	SNP
rs6760610	chr2	1.58E+08	G	C	CCDC148-A	68	0.351	SNP
rs1303199	chr2	1.6E+08	G	A	43897	22	0.113	SNP
rs2302872	chr2	1.62E+08	G	A	DPP4	2	0.01	SNP
rs5617912	chr2	1.62E+08	C	T	DPP4	2	0.01	SNP
rs1163027	chr2	1.62E+08	T	C	DPP4	6	0.031	SNP
rs3574460	chr2	1.62E+08	C	A	IFIH1	3	0.015	SNP
rs3533754	chr2	1.62E+08	C	G	IFIH1	2	0.01	SNP
rs7287770	chr2	1.64E+08	C	T	AC092684.	21	0.108	SNP
rs6217395	chr2	1.65E+08	T	C	COBLL1	35	0.18	SNP
rs1379471	chr2	1.65E+08	CAAGTTAG	C	AC013463.	10	0.052	INDEL
rs1946892	chr2	1.65E+08	T	A	AC013463.	81	0.418	SNP
rs1718381	chr2	1.65E+08	G	A	SCN2A	17	0.088	SNP
rs2020102	chr2	1.65E+08	ATT	AT	SCN2A	48	0.247	INDEL
rs2020102	chr2	1.65E+08	ATT	A	SCN2A	50	0.258	INDEL
rs1968294	chr2	1.66E+08	C	T	GALNT3	96	0.495	SNP
rs6262279	chr2	1.66E+08	T	C	SCN7A	4	0.021	SNP
.	chr2	1.67E+08	TG	T	XIRP2	3	0.015	INDEL
rs1685316	chr2	1.67E+08	T	C	XIRP2	18	0.093	SNP
rs1448124	chr2	1.69E+08	G	C	DHRS9	4	0.021	SNP
708750	chr2	1.7E+08	G	GT	FASTKD1	3	0.015	INDEL
rs2276611	chr2	1.7E+08	G	A	PPIG	25	0.129	SNP
rs2114646	chr2	1.7E+08	C	T	PTCHD3P2	45	0.232	SNP
rs1506735	chr2	1.7E+08	C	CA	KLHL23	117	0.603	INDEL
rs1861464	chr2	1.71E+08	T	C	SP5	74	0.381	SNP
rs1928611	chr2	1.71E+08	C	T	DCAF17	38	0.196	MIXED
rs1928611	chr2	1.71E+08	C	CT	DCAF17	7	0.036	MIXED
rs1299473	chr2	1.74E+08	G	A	SCRN3	46	0.237	SNP
rs1456990	chr2	1.74E+08	TTCAAATTT	T	SCRN3	60	0.309	INDEL
rs1017973	chr2	1.77E+08	C	T	AC074286.	155	0.799	SNP
rs7904582	chr2	1.79E+08	CT	C	TTN-AS1	19	0.098	INDEL
rs7904582	chr2	1.79E+08	CT	CTT	TTN-AS1	4	0.021	INDEL
rs7558237	chr2	1.81E+08	C	T	AC068196.	6	0.031	SNP
.	chr2	1.82E+08	CT	C	PPP1R1C	34	0.175	INDEL
.	chr2	1.82E+08	CT	CTT	PPP1R1C	17	0.088	INDEL
rs7921780	chr2	1.83E+08	T	G	AC064871.	17	0.088	SNP
rs1452076	chr2	1.83E+08	T	A	NUP35	6	0.031	SNP
rs7692139	chr2	1.83E+08	C	T	NUP35	21	0.108	SNP
rs925806	chr2	1.83E+08	G	A	NUP35	92	0.474	SNP

rs3561728:	chr2	1.86E+08	A	AG	FSIP2	66	0.34	INDEL
rs3579513(	chr2	1.87E+08	C	CT	AC018867.	62	0.32	INDEL
rs3214425	chr2	1.9E+08	T	TA	PMS1	39	0.201	INDEL
rs1440746(	chr2	1.9E+08	A	C	HIBCH	2	0.01	SNP
rs291466;3	chr2	1.9E+08	A	G	HIBCH	104	0.536	SNP
rs1683700(	chr2	1.96E+08	T	C	AC064834.	40	0.206	SNP
rs1093172(	chr2	1.96E+08	T	C	STK17B	124	0.639	SNP
rs1439042!	chr2	1.97E+08	CTG	C	CCDC150	3	0.015	INDEL
rs1392949!	chr2	1.97E+08	ATG	A	ANKRD44	3	0.015	INDEL
.	chr2	2E+08	TA	T	FTCDNL1	14	0.072	INDEL
.	chr2	2E+08	TA	TAA	FTCDNL1	25	0.129	INDEL
rs2540051	chr2	2.01E+08	G	A	AOX3P	36	0.186	SNP
rs6262359!	chr2	2.01E+08	G	A	AOX2P	31	0.16	SNP
rs1738310!	chr2	2.01E+08	C	A	CLK1	20	0.103	SNP
rs1019075:	chr2	2.01E+08	G	A	CFLAR	47	0.242	SNP
rs3769824;	chr2	2.01E+08	T	C	CASP8	8	0.041	SNP
rs1045485;	chr2	2.01E+08	G	C	CASP8	21	0.108	SNP
rs1045487;	chr2	2.01E+08	G	A	CASP8	11	0.057	SNP
rs2021242(	chr2	2.01E+08	G	C	ALS2CR12	2	0.01	SNP
rs1019461!	chr2	2.02E+08	T	C	ALS2	27	0.139	SNP
.	chr2	2.02E+08	C	T	KIAA2012	3	0.015	SNP
.	chr2	2.03E+08	ATTT	ATTTT	ICA1L	88	0.454	INDEL
.	chr2	2.03E+08	ATTT	AT	ICA1L	21	0.108	INDEL
.	chr2	2.03E+08	ATTT	ATTTTT	ICA1L	4	0.021	INDEL
rs2015206!	chr2	2.03E+08	C	CA	CARF	2	0.01	INDEL
rs2006898(	chr2	2.03E+08	G	T	NBEAL1	2	0.01	SNP
rs2469953	chr2	2.03E+08	T	C	RAPH1	111	0.572	SNP
rs1262363(	chr2	2.04E+08	G	A	AC009965.	72	0.371	SNP
.	chr2	2.06E+08	TCGCA	T	NRP2	8	0.041	INDEL
rs2004835:	chr2	2.06E+08	T	TA	NRP2	8	0.041	INDEL
rs1016550(	chr2	2.07E+08	C	T	AC007879.	94	0.485	SNP
rs3459917!	chr2	2.08E+08	T	C	IDH1	6	0.031	SNP
rs3421884(	chr2	2.08E+08	C	T	IDH1	8	0.041	SNP
rs1155413:	chr2	2.08E+08	G	A	IDH1	8	0.041	SNP
rs6174232!	chr2	2.08E+08	C	A	PTH2R	8	0.041	SNP
rs6738070	chr2	2.13E+08	A	C	IKZF2	182	0.938	MIXED
rs2070094;	chr2	2.15E+08	C	T	BARD1	72	0.371	SNP
rs2070093;	chr2	2.15E+08	A	G	BARD1	155	0.799	SNP
.	chr2	2.15E+08	G	GA	ABCA12	8	0.041	INDEL
rs1386863:	chr2	2.15E+08	G	A	ATIC	2	0.01	SNP
rs1409888:	chr2	2.16E+08	G	C	AC012668.	4	0.021	SNP
rs1685535:	chr2	2.16E+08	C	T	PECR	23	0.119	SNP
rs1489613(	chr2	2.18E+08	AC	A	DIRC3	18	0.093	INDEL
rs1152548:	chr2	2.18E+08	G	C	CTDSP1	14	0.073	SNP
rs832812	chr2	2.19E+08	T	C	CNOT9	194	1	SNP
rs908197;6	chr2	2.19E+08	G	C	DNAJB2	25	0.129	SNP
.	chr2	2.19E+08	CA	C	SPEG	2	0.01	INDEL

.	chr2	2.19E+08	CCACA	C	SPEG	2	0.01	INDEL
rs1143706!	chr2	2.19E+08	T	TA	SPEG	10	0.052	INDEL
rs1995142(	chr2	2.23E+08	AG	A	MOGAT1	5	0.026	INDEL
rs1392201:	chr2	2.23E+08	T	C	ACSL3	2	0.01	SNP
rs5911886:	chr2	2.24E+08	C	CAA	AP1S3	35	0.18	INDEL
.	chr2	2.25E+08	A	ACATTG	RP11-811O	2	0.01	INDEL
rs3731597	chr2	2.27E+08	G	A	IRS1	2	0.01	SNP
rs6729258	chr2	2.27E+08	A	C	AC097662.	116	0.598	SNP
rs1411377!	chr2	2.27E+08	C	A	AC097662.	7	0.036	SNP
.	chr2	2.27E+08	T	TCTGGAAA	MFF	4	0.021	INDEL
.	chr2	2.27E+08	G	GGTGAGCA	MFF	2	0.01	INDEL
.	chr2	2.27E+08	G	GGTGAGCA	MFF	2	0.01	INDEL
.	chr2	2.27E+08	G	GAAA	MFF	4	0.021	INDEL
rs2176186	chr2	2.28E+08	C	T	C2orf83	65	0.335	SNP
rs1405708!	chr2	2.28E+08	C	CTAAAATA.	AC064853.	32	0.165	INDEL
rs1405708!	chr2	2.28E+08	C	CTAAAA	AC064853.	18	0.093	INDEL
rs3483426!	chr2	2.28E+08	CT	C	CCL20	17	0.088	INDEL
rs3483426!	chr2	2.28E+08	CT	CTT	CCL20	10	0.052	INDEL
rs1302632:	chr2	2.3E+08	G	A	AC009950.	22	0.113	SNP
rs4973318	chr2	2.3E+08	T	C	SP140L	31	0.16	SNP
rs7236278(	chr2	2.31E+08	TCAGCAGC	T	SPATA3	101	0.521	INDEL
rs3765311!	chr2	2.31E+08	C	A	SPATA3	2	0.01	SNP
rs2868516:	chr2	2.32E+08	C	T	LINC00471	47	0.242	SNP
rs1493436!	chr2	2.32E+08	C	A	PDE6D	4	0.021	SNP
rs1164705!	chr2	2.32E+08	G	A	DIS3L2	4	0.021	SNP
rs9678004	chr2	2.32E+08	A	G	DIS3L2	19	0.098	SNP
rs1190441	chr2	2.33E+08	A	G	EIF4E2	59	0.304	SNP
402899	chr2	2.33E+08	C	*	GIGYF2	6	0.031	MIXED
402899	chr2	2.33E+08	C	CCG	GIGYF2	3	0.015	MIXED
402900	chr2	2.33E+08	ACAG	*	GIGYF2	139	0.716	INDEL
402900	chr2	2.33E+08	ACAG	AGCAG	GIGYF2	3	0.015	INDEL
rs2233385	chr2	2.33E+08	G	A	NEU2	2	0.01	SNP
rs2233394	chr2	2.33E+08	C	T	NEU2	24	0.124	SNP
rs3392688:	chr2	2.33E+08	T	TTG	INPP5D	38	0.196	INDEL
rs6219289!	chr2	2.33E+08	G	C	INPP5D	52	0.268	SNP
rs2021789!	chr2	2.33E+08	CGTGTGCG	C	DGKD	5	0.026	INDEL
rs1786836:	chr2	2.34E+08	G	A	MROH2A	33	0.17	SNP
rs1423416:	chr2	2.36E+08	C	CCAGG	AGAP1	25	0.129	INDEL
rs6740125	chr2	2.37E+08	G	A	COPS8	4	0.021	SNP
rs7711643:	chr2	2.38E+08	CTT	C	UBE2F-SCL'	10	0.052	INDEL
rs7711643:	chr2	2.38E+08	CTT	CT	UBE2F-SCL'	23	0.119	INDEL
rs7711643:	chr2	2.38E+08	CTT	CTTT	UBE2F-SCL'	10	0.052	INDEL
rs6680228!	chr2	2.38E+08	T	G	RP11-5O24	8	0.041	SNP
rs9711321	chr2	2.39E+08	T	C	AC144525.	118	0.608	SNP
.	chr2	2.4E+08	GTGGGTGA	G	PRR21	4	0.021	INDEL
.	chr2	2.4E+08	G	GGCCGTGC	PRR21	7	0.036	INDEL
rs1506811:	chr2	2.4E+08	G	A	GPC1	5	0.026	SNP

rs6262119	chr2	2.41E+08	C	A	ANKMY1	37	0.191	SNP
rs2019170	chr2	2.41E+08	GC	G	AQP12B	32	0.165	INDEL
rs5839795	chr2	2.41E+08	CG	C	AQP12A	9	0.046	INDEL
rs3395804	chr2	2.41E+08	G	A	AGXT	26	0.134	SNP
rs3410859	chr2	2.41E+08	T	C	CROCC2	2	0.01	SNP
rs2286323	chr2	2.41E+08	A	G	MTERF4	22	0.113	SNP
rs1131293	chr2	2.41E+08	A	C	PASK	26	0.134	SNP
rs2302050	chr2	2.41E+08	C	T	PASK	24	0.124	SNP
.	chr2	2.41E+08	TG	T	PASK	2	0.01	INDEL
rs2074828	chr2	2.41E+08	A	G	PASK	72	0.371	SNP
.	chr2	2.41E+08	GTTTTTTTT	GTTTTTTTT	FARP2	6	0.033	INDEL
.	chr2	2.41E+08	GTTTTTTTT	GTTTTTTTT	FARP2	27	0.147	INDEL
.	chr2	2.42E+08	GTGTCCTT(	G	D2HGDH	26	0.134	INDEL
rs1114629	chr3	197664	C	CG	CHL1	39	0.201	MIXED
rs1114629	chr3	197664	C	G	CHL1	11	0.057	MIXED
.	chr3	197872	CT	C	CHL1	14	0.072	INDEL
.	chr3	197872	CT	CTT	CHL1	113	0.582	INDEL
.	chr3	197872	CT	CTTT	CHL1	3	0.015	INDEL
.	chr3	197872	CT	CTTTT	CHL1	14	0.072	INDEL
rs6807118	chr3	1092761	C	A	CNTN6	28	0.144	SNP
rs163579	chr3	3059381	C	T	CNTN4-AS1	79	0.407	SNP
.	chr3	3145502	CAAAA	CA	AC024060.	25	0.129	INDEL
.	chr3	3145502	CAAAA	CAA	AC024060.	130	0.67	INDEL
.	chr3	3145502	CAAAA	CAAA	AC024060.	30	0.155	INDEL
.	chr3	3145502	CAAAA	C	AC024060.	6	0.031	INDEL
rs9858984	chr3	5963082	G	C	AC027119.	48	0.247	SNP
rs3510671	chr3	7146493	T	C	GRM7	4	0.021	SNP
rs5846531	chr3	7691039	AT	A	GRM7	177	0.912	INDEL
rs9784344	chr3	8611640	C	T	LMCD1-AS	10	0.052	SNP
rs1115203	chr3	9049260	C	G	SRGAP3	20	0.103	SNP
.	chr3	9602282	AATATATA	*	DUSP5P2	76	0.567	MIXED
.	chr3	9602282	AATATATA	AAT	DUSP5P2	4	0.03	MIXED
.	chr3	9602282	AATATATA	AATATAT	DUSP5P2	7	0.052	MIXED
.	chr3	9602282	AATATATA	AATATATA	DUSP5P2	2	0.015	MIXED
.	chr3	9602282	AATATATA	TATATATA	DUSP5P2	2	0.015	MIXED
.	chr3	9729662	CAG	C	CPNE9	2	0.01	INDEL
rs9879307	chr3	9828381	C	T	TTLL3	143	0.737	SNP
rs1159171	chr3	9833230	C	T	TTLL3	8	0.041	SNP
rs1441669	chr3	11807449	CCACT	C	TAMM41	5	0.026	INDEL
rs9876026	chr3	11808333	A	C	TAMM41	80	0.412	SNP
rs3856806;	chr3	12434058	C	T	PPARG	33	0.17	SNP
rs1995821	chr3	14540371	CT	C	GRIP2	2	0.01	INDEL
rs6153099	chr3	15264291	T	TC	SH3BP5-AS	2	0.01	MIXED
rs14576	chr3	16227479	C	A	GALNT15	63	0.325	SNP
.	chr3	18423727	CT	C	SATB1	11	0.057	INDEL
rs6790734	chr3	21034445	C	T	SGO1-AS1	91	0.469	SNP
rs1156725	chr3	21777607	C	T	ZNF385D	11	0.057	SNP

rs6550714	chr3	22904655	A	G	RANP7	18	0.093	SNP
rs1550769	chr3	27255305	C	G	NEK10	49	0.253	SNP
rs6223488(	chr3	29641148	C	T	RBMS3-AS2	11	0.057	SNP
rs4639011	chr3	31989506	C	T	ZNF860	3	0.015	SNP
.	chr3	32704796	G	GT	CNOT10	8	0.041	INDEL
.	chr3	32704796	G	GTT	CNOT10	4	0.021	INDEL
rs1127544:	chr3	33845516	G	A	PDCD6IP	15	0.077	SNP
rs6050037:	chr3	37984259	CA	C	CTDSPL	43	0.222	INDEL
rs1382572:	chr3	38307655	ATTTG	A	SLC22A14	3	0.015	INDEL
rs1461344:	chr3	38348565	G	C	XYLB	6	0.031	SNP
rs818850	chr3	38360348	T	C	XYLB	194	1	SNP
rs704943	chr3	38365282	G	T	XYLB	188	0.969	SNP
rs843890	chr3	38366870	G	A	XYLB	188	0.969	SNP
.	chr3	41977517	CA	CAA	GEMIN2P2	36	0.186	INDEL
.	chr3	41977517	CA	C	GEMIN2P2	17	0.088	INDEL
rs413042	chr3	42519391	C	G	VIPR1-AS1	2	0.01	SNP
rs7308530:	chr3	42619075	G	A	NKTR	5	0.026	SNP
rs3604672:	chr3	44499299	TTC	T	ZNF852	194	1	INDEL
rs1398609!	chr3	44901672	G	A	TGM4	7	0.036	SNP
rs5848762	chr3	45636146	CA	C	LIMD1	194	1	INDEL
rs333;8184	chr3	46373452	TACAGTCA	T	CCR5	15	0.077	INDEL
rs1488709!	chr3	46617285	C	T	AC104304.:	5	0.026	SNP
rs4075012	chr3	46743865	T	C	PRSS45	21	0.108	SNP
rs7532404(	chr3	46833768	A	G	PRSS42	6	0.031	SNP
.	chr3	47407694	CAGGAG	C	PTPN23	2	0.01	INDEL
.	chr3	47410664	CAG	C	PTPN23	2	0.01	INDEL
.	chr3	48468965	AGGACACA	A	SHISA5	6	0.031	INDEL
rs1449234:	chr3	49280775	G	A	USP4	3	0.015	SNP
rs4129070(	chr3	49283991	T	C	USP4	2	0.01	SNP
.	chr3	49312474	CA	C	USP4	4	0.021	INDEL
rs1426900:	chr3	49684379	G	A	MST1	4	0.021	SNP
rs2011392!	chr3	49686483	T	G	MST1	5	0.026	SNP
.	chr3	50061560	CTTTT	CTTT	RBM6	75	0.403	INDEL
.	chr3	50061560	CTTTT	CTTTTT	RBM6	6	0.032	INDEL
.	chr3	50061560	CTTTT	CTTTTTT	RBM6	28	0.151	INDEL
.	chr3	50061560	CTTTT	CTT	RBM6	14	0.075	INDEL
.	chr3	50061560	CTTTT	C	RBM6	2	0.011	INDEL
.	chr3	50061560	CTTTT	CTTTTTTT	RBM6	6	0.032	INDEL
rs3766575(	chr3	50075156	CA	C	RBM6	35	0.18	INDEL
rs3766575(	chr3	50075156	CA	CAA	RBM6	7	0.036	INDEL
.	chr3	50218267	CAG	C	SLC38A3	2	0.01	INDEL
.	chr3	50218269	G	C	SLC38A3	9	0.047	SNP
rs762707	chr3	50252421	C	T	GNAI2	6	0.031	SNP
rs7187732:	chr3	51385310	CGGAGGA	CGGA	MANF	6	0.031	INDEL
.	chr3	51385329	GGAGGAT	*	MANF	2	0.01	INDEL
.	chr3	51385332	GGAT	*	MANF	3	0.015	INDEL
.	chr3	51385332	GGAT	G	MANF	2	0.01	INDEL

.	chr3	51385335	T	*	MANF	5	0.026	SNP
.	chr3	51385335	T	G	MANF	15	0.077	SNP
rs3403885(	chr3	52229951	G	A	TWF2	19	0.098	SNP
rs2276834	chr3	52291743	A	G	GLYCTK	115	0.593	SNP
.	chr3	52322632	CTG	C	DNAH1	2	0.01	INDEL
.	chr3	52322639	CGG	C	DNAH1	2	0.01	INDEL
rs2015395:	chr3	52423087	C	CCA	PHF7	2	0.01	INDEL
rs1463284(	chr3	52519256	G	GC	STAB1	5	0.026	INDEL
rs6809412:	chr3	52787976	CT	C	ITIH1	104	0.536	INDEL
rs2230493;	chr3	53179641	T	C	PRKCD	21	0.108	SNP
rs1388873:	chr3	53231451	T	A	TKT	2	0.01	SNP
rs1705292(	chr3	53235071	T	C	TKT	2	0.01	SNP
rs1051483	chr3	53235147	G	A	TKT	38	0.196	SNP
rs1043261	chr3	53865249	C	T	IL17RB	15	0.077	SNP
rs4687757	chr3	53872486	G	A	ACTR8	78	0.402	SNP
rs3733082	chr3	53880066	G	A	ACTR8	5	0.026	SNP
rs7928276:	chr3	57227919	G	C	APPL1	9	0.046	SNP
.	chr3	58632518	ATT	ATTTTT	FAM3D-AS:	43	0.222	INDEL
.	chr3	58632518	ATT	ATTTTTT	FAM3D-AS:	42	0.216	INDEL
.	chr3	58632518	ATT	ATTT	FAM3D-AS:	64	0.33	INDEL
rs1447215!	chr3	62168090	C	T	PTPRG	2	0.01	SNP
rs6175421(	chr3	62267477	T	C	PTPRG	2	0.01	SNP
rs6768504	chr3	63427431	A	G	SYNPR-AS1	121	0.624	SNP
.	chr3	67007905	CT	C	KBTBD8	13	0.067	INDEL
.	chr3	67007905	CT	CTT	KBTBD8	6	0.031	INDEL
rs1052906	chr3	67947115	T	TTTTATTTA	SUCLG2-AS	90	0.464	INDEL
rs1052906	chr3	67947115	T	TTTTATTTA	SUCLG2-AS	63	0.325	INDEL
rs1052906	chr3	67947115	T	TTTTATTTA	SUCLG2-AS	2	0.01	INDEL
rs1052906	chr3	67947115	T	TTTTATTTA	SUCLG2-AS	7	0.036	INDEL
.	chr3	68250777	G	GT	FAM19A1	10	0.052	INDEL
.	chr3	71583983	ACCCCCCT(	A	FOXP1&RP	2	0.01	INDEL
rs3832186	chr3	73062330	C	CA	EBLN2	27	0.139	INDEL
rs1148607:	chr3	75337042	C	T	MYLKP1	3	0.015	SNP
rs6103255(	chr3	75435013	C	A	FAM86DP	6	0.031	SNP
rs3559483:	chr3	75491037	C	G	ENPP7P2	67	0.345	SNP
rs2019808:	chr3	75738008	C	A	ZNF717	3	0.015	SNP
rs5856475:	chr3	75784914	C	T	ZNF717	5	0.026	SNP
rs1812953!	chr3	78717842	A	G	ROBO1	2	0.01	SNP
rs2228389;	chr3	81578024	T	C	GBE1	4	0.021	SNP
rs2229519;	chr3	81648979	T	C	GBE1	60	0.309	SNP
rs7311865:	chr3	82458149	T	C	RP11-260O	30	0.155	SNP
rs1308916:	chr3	84672620	C	G	LINC00971	62	0.32	SNP
rs1479305!	chr3	88467364	TA	T	RP11-249L:	20	0.103	INDEL
rs7635824	chr3	88467516	G	A	RP11-249L:	143	0.737	SNP
rs1398197	chr3	89449278	G	A	EPHA3	12	0.062	SNP
rs832079	chr3	97954424	A	C	MINA	194	1	SNP
rs5590765(	chr3	97959053	T	G	MINA	2	0.01	SNP

.	chr3	98007903	A	T	GABRR3	35	0.18	SNP
rs5851097	chr3	98064722	AT	A	OR5AC1	82	0.423	INDEL
rs8022095	chr3	98088100	T	C	OR5AC2	41	0.211	SNP
rs3776594	chr3	98168999	AT	A	OR5H15	11	0.057	INDEL
rs2316271	chr3	98312463	T	A	OR5H8	65	0.335	SNP
rs1128861	chr3	98354747	TA	T	OR5K4	99	0.51	INDEL
rs1447590	chr3	98391562	G	GA	OR5K3	100	0.515	INDEL
rs5563937	chr3	98498334	T	A	OR5K2	48	0.247	SNP
rs1729995;	chr3	98585623	T	C	CPOX	134	0.691	SNP
rs1306527	chr3	98715649	C	T	ST3GAL6-A	86	0.443	SNP
rs3830303	chr3	1E+08	C	CG	NIT2	133	0.686	INDEL
rs3732895	chr3	1.01E+08	T	C	ABI3BP	36	0.186	SNP
rs2305035	chr3	1.06E+08	G	A	CBLB	39	0.201	SNP
.	chr3	1.07E+08	CTT	CT	DUBR	105	0.541	INDEL
.	chr3	1.07E+08	CTT	C	DUBR	4	0.021	INDEL
.	chr3	1.07E+08	CTT	CTTT	DUBR	16	0.082	INDEL
rs9832228	chr3	1.07E+08	T	C	RP11-446H	18	0.093	SNP
rs9856626	chr3	1.09E+08	G	C	DZIP3	139	0.716	SNP
rs5851607	chr3	1.09E+08	G	GGGGGAT	RETNLB	15	0.077	INDEL
rs1093397	chr3	1.09E+08	C	A	GUCA1C	58	0.299	SNP
rs1431744	chr3	1.09E+08	C	A	GUCA1C	2	0.01	SNP
rs1685556	chr3	1.09E+08	A	G	DPPA4	2	0.01	SNP
rs5851818	chr3	1.12E+08	G	GT	C3orf52	72	0.371	INDEL
rs3556066	chr3	1.13E+08	C	CA	ATG3	173	0.892	INDEL
rs5816163	chr3	1.13E+08	GT	G	CD200R1L	50	0.258	INDEL
rs1462815	chr3	1.13E+08	TAGA	T	BOC	8	0.041	INDEL
rs810209	chr3	1.13E+08	G	A	CFAP44	139	0.716	SNP
rs3732781	chr3	1.14E+08	A	C	ZNF80	55	0.284	SNP
.	chr3	1.14E+08	GAAAAAAA	GAAAAAAA	MIR568&Z	64	0.33	INDEL
.	chr3	1.14E+08	GAAAAAAA	GAAAAAAA	MIR568&Z	8	0.041	INDEL
.	chr3	1.14E+08	GAAAAAAA	GAAAAAAA	MIR568&Z	49	0.253	INDEL
.	chr3	1.14E+08	GAAAAAAA	G	MIR568&Z	5	0.026	INDEL
.	chr3	1.14E+08	GAAAAAAA	GAAAAAAA	MIR568&Z	2	0.01	INDEL
rs2272277	chr3	1.14E+08	G	A	ZBTB20-AS	2	0.01	SNP
rs1420457	chr3	1.17E+08	AC	A	LSAMP	4	0.021	INDEL
.	chr3	1.19E+08	C	G	B4GALT4	2	0.01	SNP
rs1131265	chr3	1.2E+08	G	C	TIMMDC1	32	0.165	SNP
rs1379230	chr3	1.21E+08	T	G	HGD	12	0.062	SNP
rs7910616	chr3	1.21E+08	C	A	RABL3	8	0.041	SNP
.	chr3	1.21E+08	CAATAGTA	C	POLQ	4	0.021	INDEL
rs1405379	chr3	1.22E+08	C	T	ARGFX	3	0.015	SNP
rs6175034	chr3	1.22E+08	C	T	KPNA1	3	0.015	SNP
rs6173276	chr3	1.23E+08	C	T	PARP14	10	0.052	SNP
rs1683346	chr3	1.23E+08	G	A	PARP14	16	0.082	SNP
.	chr3	1.25E+08	A	G	KALRN	2	0.01	SNP
rs13146;25	chr3	1.25E+08	C	T	UMPS	33	0.17	SNP
rs3772809;	chr3	1.25E+08	A	G	UMPS	2	0.01	SNP

rs6767618	chr3	1.26E+08	C	G	RP11-379B	44	0.227	SNP
rs2004728.	chr3	1.26E+08	CAGGGCAA	C	ROPN1B	8	0.054	INDEL
rs3776574	chr3	1.26E+08	TAACA	T	SLC41A3	43	0.222	INDEL
rs1044215	chr3	1.26E+08	C	G	SLC41A3	43	0.222	SNP
rs1426159	chr3	1.26E+08	G	A	CFAP100	2	0.01	SNP
rs3708206	chr3	1.27E+08	ACCCTGCA	A	C3orf22	17	0.088	INDEL
rs4881	chr3	1.28E+08	T	C	MGLL	17	0.088	SNP
rs4548;138	chr3	1.29E+08	C	T	RAB7A	7	0.036	SNP
rs5891595	chr3	1.29E+08	A	AG	RP11-529F	29	0.149	INDEL
rs140696	chr3	1.29E+08	G	A	MBD4	16	0.082	SNP
rs9870300	chr3	1.3E+08	A	G	RP11-93K2	118	0.608	SNP
rs5580001	chr3	1.3E+08	GC	G	ALG1L2	138	0.711	INDEL
rs1683462	chr3	1.3E+08	T	G	FAM86HP	22	0.113	SNP
rs1153758	chr3	1.3E+08	G	T	COL6A5	3	0.015	SNP
rs1135579	chr3	1.3E+08	AT	A	COL6A5	158	0.814	INDEL
rs4127231	chr3	1.33E+08	C	A	ACAD11	6	0.031	SNP
rs7622700	chr3	1.33E+08	G	T	NPHP3-AS1	81	0.418	SNP
rs1398722	chr3	1.33E+08	G	A	NPHP3-AS1	107	0.552	SNP
rs1730188	chr3	1.34E+08	A	G	TOPBP1	33	0.17	SNP
rs6174811	chr3	1.34E+08	C	T	RAB6B	2	0.01	SNP
rs2000240	chr3	1.36E+08	C	CCAAT	PPP2R3A	8	0.041	INDEL
496411	chr3	1.38E+08	CA	C	MRAS	4	0.021	INDEL
rs1412826	chr3	1.39E+08	CGGCATTC	C	FOXL2NB	4	0.021	INDEL
rs1093532	chr3	1.39E+08	G	A	MRPS22	116	0.598	SNP
rs7904340	chr3	1.4E+08	G	A	NMNAT3	4	0.021	SNP
rs7615158	chr3	1.41E+08	A	G	CLSTN2-AS	28	0.144	SNP
rs890529	chr3	1.41E+08	C	A	SPSB4	39	0.201	SNP
rs2291039	chr3	1.41E+08	T	G	PXYLP1	121	0.624	SNP
.	chr3	1.43E+08	CTT	CT	PAQR9-AS1	22	0.113	INDEL
.	chr3	1.43E+08	CTT	C	PAQR9-AS1	5	0.026	INDEL
.	chr3	1.43E+08	CTT	CTTT	PAQR9-AS1	9	0.046	INDEL
rs1457209	chr3	1.43E+08	A	T	U2SURP	2	0.01	SNP
rs1431377	chr3	1.49E+08	G	C	GYG1	3	0.015	SNP
.	chr3	1.49E+08	C	T	HLTF	2	0.01	SNP
rs1123999	chr3	1.5E+08	C	CTTAA	WWTR1	16	0.082	INDEL
rs6783790	chr3	1.5E+08	G	A	WWTR1	94	0.485	SNP
rs1833522	chr3	1.5E+08	C	T	RP11-167H	4	0.021	SNP
rs3821661	chr3	1.51E+08	C	T	RP11-373I8	40	0.206	SNP
rs6440742	chr3	1.51E+08	G	T	IGSF10	147	0.758	SNP
rs7315397	chr3	1.54E+08	G	A	RP11-217E	4	0.021	SNP
rs5620827	chr3	1.55E+08	G	A	MME	38	0.196	SNP
rs6175037	chr3	1.56E+08	A	C	GMPS	2	0.01	SNP
rs1159068	chr3	1.56E+08	T	A	KCNAB1	11	0.057	SNP
rs2008344	chr3	1.57E+08	A	AT	LEKR1	7	0.036	INDEL
rs1488388	chr3	1.58E+08	A	G	VEPH1	5	0.026	SNP
rs1151080	chr3	1.59E+08	C	T	LXN	2	0.01	SNP
.	chr3	1.6E+08	CAGAT	C	TRIM59	5	0.026	INDEL

rs7860066.	chr3	1.61E+08	G	A	RP11-3P17	9	0.046	SNP
rs2229697;	chr3	1.68E+08	G	A	SERPINI1	20	0.103	SNP
rs1263200	chr3	1.7E+08	T	C	SAMD7	2	0.01	SNP
rs5953502(	chr3	1.72E+08	G	C	FNDC3B	9	0.046	SNP
rs1131532	chr3	1.73E+08	A	G	TNFSF10	137	0.706	SNP
rs5611911(	chr3	1.73E+08	A	G	TNFSF10	2	0.01	SNP
.	chr3	1.73E+08	GT	GTT	ECT2	24	0.124	INDEL
.	chr3	1.73E+08	GT	G	ECT2	8	0.041	INDEL
rs7117084	chr3	1.77E+08	TCATCCAT(	TCATCCAT(	TBL1XR1	16	0.082	INDEL
rs7117084	chr3	1.77E+08	TCATCCAT(	TCATCCAT(	TBL1XR1	44	0.227	INDEL
rs7117084	chr3	1.77E+08	TCATCCAT(	TCATCCAT(	TBL1XR1	22	0.113	INDEL
rs7117084	chr3	1.77E+08	TCATCCAT(	T	TBL1XR1	21	0.108	INDEL
rs7117084	chr3	1.77E+08	TCATCCAT(	TCATCCAT(	TBL1XR1	4	0.021	INDEL
.	chr3	1.78E+08	CTATATAT/	CTATATAT/	RP11-33A1	18	0.093	INDEL
.	chr3	1.78E+08	CTATATAT/	CTATATAT/	RP11-33A1	25	0.129	INDEL
.	chr3	1.78E+08	CTATATAT/	C	RP11-33A1	13	0.067	INDEL
.	chr3	1.78E+08	CTATATAT/	CTATATAT/	RP11-33A1	14	0.072	INDEL
.	chr3	1.78E+08	CTATATAT/	CTATATAT/	RP11-33A1	15	0.077	INDEL
.	chr3	1.78E+08	CTATATAT/	CTATATAT/	RP11-33A1	7	0.036	INDEL
rs3680291(	chr3	1.79E+08	ATTT	A	KCNMB2	52	0.268	INDEL
rs3680291(	chr3	1.79E+08	ATTT	ATT	KCNMB2	8	0.041	INDEL
rs3680291(	chr3	1.79E+08	ATTT	ATTTT	KCNMB2	10	0.052	INDEL
rs2230461;	chr3	1.79E+08	A	G	PIK3CA	8	0.041	SNP
rs1439622	chr3	1.79E+08	CT	C	KCNMB3	8	0.041	INDEL
rs5923822	chr3	1.8E+08	G	GCTGT	NDUFB5	17	0.088	INDEL
rs2003539	chr3	1.81E+08	T	TA	CCDC39	2	0.01	INDEL
rs4855066	chr3	1.82E+08	T	A	LINC01206	131	0.675	SNP
rs3678494	chr3	1.83E+08	GT	G	RP11-531F	19	0.098	INDEL
rs3678494	chr3	1.83E+08	GT	GTT	RP11-531F	5	0.026	INDEL
.	chr3	1.83E+08	CT	CTT	RP11-531F	130	0.67	INDEL
.	chr3	1.83E+08	CT	C	RP11-531F	5	0.026	INDEL
rs3559716	chr3	1.83E+08	G	A	LAMP3	2	0.01	SNP
rs5706737(	chr3	1.83E+08	C	T	MCF2L2	34	0.175	SNP
rs7131833	chr3	1.84E+08	G	A	KLHL24	32	0.165	SNP
rs1012987	chr3	1.84E+08	T	G	CYP2AB1P	40	0.206	SNP
rs7709958(	chr3	1.84E+08	A	C	HTR3D	7	0.036	SNP
rs6443930	chr3	1.84E+08	G	C	HTR3D	78	0.402	SNP
rs7648737	chr3	1.84E+08	G	A	RP11-778D	104	0.536	SNP
rs5855015	chr3	1.84E+08	AG	A	HTR3E	14	0.072	INDEL
rs1154687	chr3	1.84E+08	C	T	ECE2	44	0.227	SNP
rs902415	chr3	1.84E+08	T	C	ECE2	42	0.216	SNP
rs1306966	chr3	1.85E+08	A	G	EPHB3	37	0.191	SNP
rs9862375	chr3	1.85E+08	G	A	EPHB3	22	0.113	SNP
rs1122472	chr3	1.85E+08	C	T	EPHB3	2	0.01	SNP
rs7643893	chr3	1.87E+08	C	T	KNG1	9	0.046	SNP
rs3408276	chr3	1.87E+08	AG	A	PSMD10P2	49	0.253	INDEL
rs3537615	chr3	1.87E+08	T	A	ST6GAL1	29	0.149	SNP

rs850312	chr3	1.87E+08	C	T	MASP1	74	0.381	SNP
rs7254915	chr3	1.87E+08	C	A	MASP1	7	0.036	SNP
rs5608631	chr3	1.9E+08	AG	A	CLDN16	43	0.222	INDEL
rs1166531	chr3	1.93E+08	G	A	HRASLS	6	0.031	SNP
rs735930	chr3	1.93E+08	C	T	ATP13A5	6	0.031	SNP
rs7443735	chr3	1.93E+08	G	A	ATP13A5	6	0.031	SNP
rs1697343	chr3	1.94E+08	A	G	RP11-528A	160	0.825	SNP
rs4974539	chr3	1.94E+08	G	A	CPN2	75	0.387	SNP
.	chr3	1.94E+08	C	A	LRRC15	2	0.01	SNP
rs2010052	chr3	1.96E+08	T	C	ZDHHC19	2	0.01	SNP
rs6775861	chr3	1.96E+08	C	T	TCTEX1D2	11	0.057	SNP
rs1117706	chr3	1.96E+08	G	A	TM4SF19	23	0.119	SNP
rs1134986	chr3	1.97E+08	C	T	DLG1	39	0.201	SNP
.	chr3	1.98E+08	C	T	IQCG	2	0.01	SNP
.	chr3	1.98E+08	ATCAGCAA	A	LMLN-AS1	2	0.01	INDEL
rs2852097	chr4	651759	T	C	PDE6B	26	0.134	SNP
rs3742324	chr4	734151	TTCACACC	T	PCGF3	3	0.015	INDEL
rs1457864	chr4	769256	CCTTG	C	PCGF3	7	0.036	INDEL
rs3488421	chr4	950422	A	C	TMEM175	23	0.119	SNP
rs1079453	chr4	987183	T	G	IDUA	161	0.83	SNP
rs6815946;	chr4	1001517	T	C	IDUA	24	0.124	SNP
rs1148068	chr4	1002080	C	T	IDUA	13	0.067	SNP
rs6830825;	chr4	1002131	G	C	IDUA	24	0.124	SNP
rs6831280;	chr4	1002377	G	A	IDUA	24	0.124	SNP
350230	chr4	1002887	C	A	IDUA	9	0.046	SNP
rs7306647	chr4	1002902	G	A	IDUA	24	0.124	SNP
.	chr4	1058312	GGGCACTA	AGGCACTA	RNF212	11	0.061	MIXED
.	chr4	1058312	GGGCACTA	GTTGGCAC	RNF212	2	0.011	MIXED
rs6003526	chr4	1093699	G	A	RNF212	27	0.139	SNP
rs4045481	chr4	1096837	G	A	RNF212	137	0.706	SNP
.	chr4	1194932	CAGCCGGC	C	SPON2	2	0.01	INDEL
rs1155478	chr4	1250894	A	C	CTBP1-AS2	4	0.021	SNP
.	chr4	1394562	G	GTGCCCAT	CRIPAK	87	0.458	INDEL
.	chr4	1394589	ATG	A	CRIPAK	5	0.026	INDEL
.	chr4	1394715	ACG	A	CRIPAK	10	0.052	INDEL
.	chr4	1394805	ACACGTGC	A	CRIPAK	14	0.072	INDEL
.	chr4	1394834	T	*	CRIPAK	14	0.074	MIXED
.	chr4	1394834	T	TCA	CRIPAK	6	0.032	MIXED
.	chr4	1394971	C	CGGAGTGC	CRIPAK	14	0.074	INDEL
.	chr4	1395000	C	CGTGGAGT	CRIPAK	2	0.01	INDEL
.	chr4	1395155	TGTGG	T	CRIPAK	33	0.201	INDEL
.	chr4	1395160	AGTGTTCG	A	CRIPAK	26	0.138	MIXED
.	chr4	1395160	AGTGTTCG	CGTGTTCG	CRIPAK	2	0.011	MIXED
.	chr4	1395173	TCA	*	CRIPAK	26	0.135	INDEL
.	chr4	1395173	TCA	T	CRIPAK	4	0.021	INDEL
rs2022302	chr4	2174006	A	G	POLN	20	0.103	SNP
rs6050471	chr4	2965260	C	T	GRK4	14	0.072	SNP

.	chr4	3074908	AGC	A	HTT	2	0.01	INDEL
.	chr4	3074911	AGCAGCAG	A	HTT	2	0.01	INDEL
.	chr4	3074917	AGC	*	HTT	2	0.01	INDEL
.	chr4	3074920	AGCAGCAG	*	HTT	2	0.01	INDEL
402952	chr4	3074923	AGC	A	HTT	4	0.021	INDEL
402952	chr4	3074923	AGC	*	HTT	3	0.015	INDEL
402953	chr4	3074926	AGCAG	A	HTT	2	0.01	INDEL
402953	chr4	3074926	AGCAG	*	HTT	3	0.015	INDEL
402953	chr4	3074926	AGCAG	ACAG	HTT	2	0.01	INDEL
.	chr4	3074943	C	CAG	HTT	2	0.01	INDEL
.	chr4	3074945	ACC	ACCGCCGC	HTT	5	0.026	MIXED
.	chr4	3074945	ACC	GCC	HTT	32	0.165	MIXED
.	chr4	3074945	ACC	ACCGCCGC	HTT	8	0.041	MIXED
.	chr4	3074945	ACC	A	HTT	2	0.01	MIXED
rs2846842	chr4	3448159	G	A	HGFAC	45	0.232	SNP
rs2863310	chr4	3548673	A	G	RP11-529E	45	0.232	SNP
rs3451695	chr4	5345310	CTT	CT	STK32B	75	0.387	INDEL
rs3451695	chr4	5345310	CTT	CTTT	STK32B	8	0.041	INDEL
rs3451695	chr4	5345310	CTT	C	STK32B	5	0.026	INDEL
rs1004374	chr4	5438081	T	A	STK32B	132	0.68	SNP
rs1389287	chr4	5973658	C	T	C4orf50	3	0.015	SNP
rs1438598	chr4	5989141	CCT	C	C4orf50	5	0.026	INDEL
rs1145543	chr4	6674992	G	A	AC093323.	2	0.01	SNP
rs2854578	chr4	8041348	A	G	ABLIM2	10	0.052	SNP
rs6852348	chr4	8394849	A	T	ACOX3	5	0.026	SNP
.	chr4	8441116	C	A	TRMT44	2	0.01	SNP
.	chr4	13632182	CTT	CT	AC006445.	17	0.088	INDEL
.	chr4	13632182	CTT	CTTT	AC006445.	14	0.072	INDEL
rs1908446	chr4	15001160	C	T	CPEB2-AS1	3	0.015	SNP
.	chr4	15003229	CACCTT	C	CPEB2	4	0.021	INDEL
rs1861050	chr4	15480736	C	T	CC2D2A	15	0.077	SNP
rs4320134	chr4	15737805	A	T	BST1	28	0.144	SNP
rs1800051	chr4	15834221	A	C	CD38	4	0.021	SNP
rs1386302	chr4	15834298	G	A	CD38	2	0.01	SNP
.	chr4	16256236	CTT	CT	TAPT1-AS1	38	0.196	INDEL
.	chr4	16256236	CTT	C	TAPT1-AS1	13	0.067	INDEL
.	chr4	16256236	CTT	CTTT	TAPT1-AS1	46	0.237	INDEL
rs1002017	chr4	17509972	A	T	QDPR	43	0.222	SNP
rs2315552	chr4	17625671	A	G	MED28	123	0.634	SNP
rs4380521	chr4	19533620	C	T	RP11-608O	67	0.345	SNP
.	chr4	20523838	A	T	SLIT2	2	0.01	SNP
rs7548032	chr4	22610340	CA	C	RP11-10G1	47	0.242	INDEL
rs358231	chr4	22818881	A	T	GBA3	176	0.907	SNP
rs5728416	chr4	23122373	A	G	RP11-552M	2	0.01	SNP
rs764497	chr4	24980067	A	T	CCDC149	49	0.253	SNP
rs3399268	chr4	26735441	C	CT	TBC1D19	185	0.954	INDEL
rs1388561	chr4	31176597	CTTTACT	C	RP11-617I1	72	0.371	INDEL

.	chr4	36245152	CT	C	RP11-431M	35	0.18	INDEL
.	chr4	36245152	CT	CTT	RP11-431M	10	0.052	INDEL
rs2003763(	chr4	37960987	CAT	C	PTTG2	5	0.026	INDEL
rs6659145{	chr4	38118393	C	CT	TBC1D1	142	0.732	INDEL
rs4129009	chr4	38773268	T	C	TLR10	33	0.17	SNP
rs1077648:	chr4	38773419	A	G	TLR10	39	0.201	SNP
rs5743818	chr4	38827542	A	C	TLR6	78	0.402	SNP
rs1129052	chr4	39508644	A	G	UGDH	22	0.113	SNP
rs1154485!	chr4	39510727	T	C	UGDH	34	0.175	SNP
rs1165096!	chr4	41939720	G	C	TMEM33	8	0.041	SNP
.	chr4	42018500	ATCCAGGT	A	SLC30A9	2	0.01	INDEL
.	chr4	44690781	CAG	C	GUF1	2	0.01	INDEL
rs963694	chr4	44944075	A	C	PRDX4P1	20	0.103	SNP
rs1477507!	chr4	49032652	CA	C	CWH43	4	0.021	INDEL
rs1996192{	chr4	49244271	ACTTC	A	RP11-1281	2	0.011	INDEL
rs2005297(	chr4	49246710	TATATATA	T	MTND3P22	75	0.387	MIXED
rs2005297(	chr4	49246710	TATATATA	AATATATA	MTND3P22	4	0.021	MIXED
rs9994643	chr4	52685545	G	T	RP11-177B	4	0.021	SNP
rs1436716!	chr4	53453080	CAG	C	FIP1L1	5	0.026	INDEL
rs1510163:	chr4	54725893	A	G	KIT	4	0.021	SNP
rs5578961!	chr4	54733102	C	T	KIT	7	0.036	SNP
rs2305948;	chr4	55113391	C	T	KDR	14	0.072	SNP
rs1154997(	chr4	56448739	A	C	PAICS	17	0.088	SNP
.	chr4	65145338	GGGTTTAT	G	EFTUD1P2	63	0.325	INDEL
rs5586055	chr4	65669566	G	A	EPHA5	40	0.206	SNP
rs977728	chr4	67963391	C	T	TMPRSS11/	26	0.134	SNP
rs1193595:	chr4	69051603	G	T	UGT2B7	35	0.18	SNP
rs1116966!	chr4	69647069	A	T	UGT2A1	5	0.026	SNP
rs2306949	chr4	70474006	G	A	MUC7	30	0.155	SNP
rs3491894:	chr4	70480983	A	AC	MUC7	4	0.021	INDEL
rs6743726!	chr4	71022523	C	T	DCK	2	0.01	SNP
rs7678112:	chr4	71803944	C	A	GC	5	0.026	SNP
rs6535531	chr4	75549656	T	C	THAP6	56	0.289	SNP
rs5622901:	chr4	75596341	C	T	CDKL2	3	0.015	SNP
rs7654738	chr4	75866520	T	C	PPEF2	194	1	SNP
rs1722080:	chr4	75884592	A	G	PPEF2	6	0.031	SNP
rs6001516	chr4	76145982	C	A	NUP54	29	0.149	SNP
.	chr4	77819049	G	GACACACA	CNOT6L	37	0.195	INDEL
.	chr4	77819049	G	GAC	CNOT6L	11	0.058	INDEL
.	chr4	77819049	G	GACACACA	CNOT6L	6	0.032	INDEL
.	chr4	77819049	G	GACACACA	CNOT6L	76	0.4	INDEL
.	chr4	77819049	G	GACACACA	CNOT6L	9	0.047	INDEL
.	chr4	77819049	G	GACAC	CNOT6L	6	0.032	INDEL
rs1000848!	chr4	78068562	T	C	FRAS1	43	0.222	SNP
rs1152781:	chr4	79690077	G	A	RP11-452C	2	0.01	SNP
rs1051666:	chr4	81802891	T	C	RASGEF1B	2	0.01	SNP
rs1120333(	chr4	83284851	T	A	COQ2	8	0.041	SNP

rs1398631	chr4	83484938	A	AG	FAM175A	3	0.015	INDEL
rs1003433	chr4	86892061	A	T	C4orf36	78	0.402	SNP
rs7261356	chr4	87310240	T	TA	HSD17B13	34	0.175	INDEL
rs1426481	chr4	87313916	G	C	HSD17B13	2	0.01	SNP
.	chr4	87615923	CA	*	DSPP	87	0.649	INDEL
.	chr4	87615925	GTGACAGC	*	DSPP	87	0.649	INDEL
rs4693959	chr4	88524755	G	T	HERC3	13	0.067	SNP
rs1418034	chr4	88991152	T	C	FAM13A	3	0.015	SNP
rs3833895	chr4	89836790	C	CT	SNCA	14	0.072	INDEL
.	chr4	89894722	A	G	MMRN1	6	0.031	SNP
rs3733453	chr4	90727202	A	G	CCSER1	11	0.057	SNP
rs1444196	chr4	94299483	G	C	HPGDS	2	0.01	SNP
rs7584170	chr4	94455353	A	G	PDLIM5	4	0.021	SNP
rs5851451	chr4	97120767	T	G	RP11-431J	21	0.108	SNP
rs6830125	chr4	98890785	G	C	EIF4E	83	0.428	SNP
rs1432809	chr4	99005871	G	A	METAP1	2	0.01	SNP
rs1126672	chr4	99126661	G	A	ADH4	66	0.34	SNP
rs2032349	chr4	99141668	A	G	ADH4	187	0.964	SNP
rs3919370	chr4	99143175	A	T	ADH4	66	0.34	SNP
rs1970704	chr4	99188644	G	C	RP11-696N	58	0.299	SNP
rs4699738	chr4	99254373	G	A	RP11-696N	58	0.299	SNP
rs2836431	chr4	99284672	T	G	ADH1A	65	0.335	SNP
18181	chr4	99347033	C	A	ADH1C	5	0.026	SNP
rs1118696	chr4	99535960	A	ACAGT	C4orf17	54	0.278	INDEL
rs1194475	chr4	99564098	G	A	MTTP	49	0.253	SNP
rs5870665	chr4	1E+08	GT	G	DDIT4L	12	0.062	INDEL
rs3765637	chr4	1E+08	A	T	RP11-588P	49	0.253	SNP
.	chr4	1.02E+08	GA	G	BANK1	2	0.01	INDEL
rs2272676	chr4	1.03E+08	G	T	NFKB1	65	0.335	SNP
rs1883172	chr4	1.03E+08	A	G	NFKB1	2	0.01	SNP
rs4648039	chr4	1.03E+08	C	T	NFKB1	2	0.01	SNP
rs3473309	chr4	1.03E+08	C	CTT	UBE2D3	25	0.136	INDEL
rs3473309	chr4	1.03E+08	C	CTTT	UBE2D3	40	0.217	INDEL
rs3473309	chr4	1.03E+08	C	CT	UBE2D3	4	0.022	INDEL
rs1721725	chr4	1.03E+08	T	C	CENPE	57	0.294	SNP
rs2290943	chr4	1.03E+08	A	G	CENPE	57	0.294	SNP
rs4639077	chr4	1.04E+08	G	A	RP11-297P	144	0.742	SNP
rs1388036	chr4	1.05E+08	A	G	RP11-556I1	4	0.021	SNP
rs3496121	chr4	1.06E+08	C	T	TBCK	28	0.144	SNP
rs3558099	chr4	1.08E+08	T	C	PAPSS1	2	0.01	SNP
rs3775974	chr4	1.08E+08	G	C	HADH	168	0.866	SNP
rs7383858	chr4	1.09E+08	C	T	SEC24B-AS	3	0.015	SNP
rs7978616	chr4	1.1E+08	G	GC	EGF	2	0.01	INDEL
rs3396635	chr4	1.11E+08	G	A	ENPEP	4	0.021	SNP
rs2278782	chr4	1.11E+08	G	A	PITX2	24	0.124	SNP
.	chr4	1.13E+08	CATCAG	C	LARP7	2	0.01	INDEL
rs2858029	chr4	1.19E+08	C	G	CEP170P1	28	0.144	SNP

rs1109853:	chr4	1.2E+08	G	A	RP11-33B1	53	0.273	SNP	
rs1705127(	chr4	1.2E+08	A	C	PDE5A	11	0.057	SNP	
rs6175260{	chr4	1.2E+08	T	C	MAD2L1	2	0.01	SNP	
rs6175260!	chr4	1.2E+08	T	C	MAD2L1	13	0.067	SNP	
.	chr4	1.21E+08	TCACACA	TCACACAC/	SETP12	89	0.459	INDEL	
.	chr4	1.21E+08	TCACACA	TCACACAC/	SETP12	7	0.036	INDEL	
.	chr4	1.21E+08	TCACACA	TCACA	SETP12	21	0.108	INDEL	
.	chr4	1.21E+08	TCACACA	T	SETP12	2	0.01	INDEL	
rs769243	chr4	1.22E+08	C	T	CCNA2	5	0.026	SNP	
rs7524114(	chr4	1.22E+08	AT	ATT	KIAA1109	10	0.052	INDEL	
rs7524114(	chr4	1.22E+08	AT	A	KIAA1109	9	0.046	INDEL	
rs2006154{	chr4	1.28E+08	G	GT	PLK4	19	0.098	INDEL	
rs5799564:	chr4	1.28E+08	A	AT	PLK4	8	0.041	INDEL	
.	chr4	1.28E+08	CGG	C	PGRMC2	2	0.01	INDEL	
rs7671638-	chr4	1.3E+08	A	AT	RP11-422J:	3	0.015	INDEL	
rs1314473-	chr4	1.32E+08	C	T	RP11-789C	23	0.119	SNP	
rs3481983-	chr4	1.35E+08	G	A	RP11-553P	18	0.093	SNP	
rs7976748!	chr4	1.38E+08	C	T	LINC00616	11	0.057	SNP	
.	chr4	1.4E+08	TTGCTGCT(	TTGCTGCT(	MAML3	4	0.021	INDEL	
.	chr4	1.4E+08	TTGCTGCT(	T	MAML3	2	0.01	INDEL	
.	chr4	1.4E+08	GC	*	MAML3	2	0.032	INDEL	
.	chr4	1.4E+08	TGCTGCTG	*	MAML3	2	0.01	INDEL	
rs3738040(	chr4	1.4E+08	GC	G	MAML3	167	0.861	INDEL	
rs3738040(	chr4	1.4E+08	GC	*	MAML3	3	0.015	INDEL	
.	chr4	1.4E+08	TGCTGCTG	TTGC	MAML3	166	0.856	INDEL	
.	chr4	1.4E+08	TGCTGCTG	*	MAML3	3	0.015	INDEL	
.	chr4	1.4E+08	C	*	MAML3	169	0.871	MIXED	
rs1194507{	chr4	1.41E+08	G	A	ELMOD2	54	0.278	SNP	
rs1110080(	chr4	1.44E+08	G	C	RP11-780M	63	0.325	SNP	
.	chr4	1.44E+08	TTAGAATA	T	RP11-673E	4	0.021	INDEL	
rs1132783	chr4	1.44E+08	C	G	GYPB	56	0.289	SNP	
rs5862681	chr4	1.44E+08	A	AT	GYPB	165	0.982	INDEL	
rs1997881!	chr4	1.45E+08	A	C	ANAPC10	2	0.01	SNP	
.	chr4	1.45E+08	AT	A	ABCE1	29	0.149	INDEL	
.	chr4	1.45E+08	AT	ATT	ABCE1	5	0.026	INDEL	
rs1249826:	chr4	1.49E+08	T	C	RP11-24I21	5	0.026	SNP	
rs1391954!	chr4	1.5E+08	TGG	T	RP11-526A	2	0.01	INDEL	
rs1161279:	chr4	1.5E+08	C	T	RP11-526A	3	0.015	SNP	
rs1488619:	chr4	1.51E+08	G	GCAGGT	PRSS48	92	0.474	INDEL	
rs2407221	chr4	1.51E+08	T	G	PRSS48	186	0.959	SNP	
.	chr4	1.53E+08	TC	T	MND1	5	0.026	INDEL	
rs1998403:	chr4	1.54E+08	TTTTG	T	DCHS2	179	0.923	INDEL	
rs7671792	chr4	1.55E+08	T	C	PLRG1	66	0.34	SNP	
rs6536068	chr4	1.55E+08	G	T	AC097467.	44	0.227	SNP	
rs1497907	chr4	1.55E+08	G	A	AC097467.	2	0.01	SNP	
rs1459853	chr4	1.56E+08	G	A	GUCY1B3	108	0.557	SNP	
rs7504214-	chr4	1.57E+08	G	C	RP11-171N	16	0.082	SNP	

.	chr4	1.57E+08	G	GT	GRIA2	2	0.01	INDEL
rs2881373	chr4	1.58E+08	A	G	RP11-597D	167	0.861	SNP
.	chr4	1.58E+08	G	A	TMEM144	3	0.015	SNP
rs7465178!	chr4	1.65E+08	C	T	TRIM61	7	0.036	SNP
rs1452465:	chr4	1.65E+08	C	A	RP11-668G	13	0.067	SNP
rs3857035	chr4	1.68E+08	C	T	DDX60L	114	0.588	SNP
.	chr4	1.69E+08	C	CT	CBR4	2	0.01	INDEL
rs2001617(	chr4	1.7E+08	C	T	NEK1	2	0.01	SNP
rs3580175	chr4	1.7E+08	A	AT	CLCN3	32	0.165	INDEL
rs3681793!	chr4	1.76E+08	TTCTC	T	SPATA4	27	0.139	INDEL
rs3537003(	chr4	1.77E+08	GT	GTT	NEIL3	13	0.067	INDEL
rs3537003(	chr4	1.77E+08	GT	G	NEIL3	40	0.206	INDEL
rs4742	chr4	1.83E+08	A	G	DCTD	62	0.32	SNP
rs1075695	chr4	1.83E+08	C	G	FAM92A1P	73	0.376	SNP
.	chr4	1.85E+08	AT	ATT	RP11-386B	45	0.232	INDEL
.	chr4	1.85E+08	AT	A	RP11-386B	10	0.052	INDEL
rs3571526(	chr4	1.85E+08	TG	T	C4orf47	2	0.01	INDEL
rs3775291;	chr4	1.86E+08	C	T	TLR3	56	0.289	SNP
rs3775290;	chr4	1.86E+08	C	T	TLR3	66	0.34	SNP
rs1438216	chr4	1.86E+08	G	T	KLKB1	2	0.01	SNP
rs5976;255	chr4	1.86E+08	G	A	F11	9	0.046	SNP
rs6765032:	chr4	1.88E+08	C	T	TRIML2	61	0.314	SNP
rs7418955:	chr4	1.88E+08	C	A	RP11-366H	3	0.015	SNP
rs1113252:	chr4	1.88E+08	G	A	RP11-366H	70	0.361	SNP
rs5603554	chr5	220318	T	TG	SDHA	8	0.041	INDEL
rs1123078	chr5	256368	CTT	C	SDHA	4	0.021	INDEL
rs2244029	chr5	311363	T	C	PDCD6	150	0.773	SNP
rs6262278:	chr5	468611	T	C	EXOC3	39	0.201	SNP
.	chr5	635522	ACGGAG	A	CEP72	2	0.01	INDEL
rs1454372!	chr5	733800	T	TAA	ZDHHC11B	7	0.036	MIXED
.	chr5	733804	CAT	C	ZDHHC11B	6	0.031	INDEL
rs1809932	chr5	766918	A	C	ZDHHC11B	146	0.753	SNP
rs1057010	chr5	795832	C	T	ZDHHC11	19	0.099	SNP
.	chr5	795839	CAGTACTA	C	ZDHHC11	19	0.099	INDEL
rs3392941	chr5	795932	CTG	C	ZDHHC11	144	0.75	INDEL
rs3842081	chr5	840245	G	GT	ZDHHC11	2	0.01	INDEL
rs1507417!	chr5	1216718	G	A	SLC6A19	2	0.01	SNP
rs7447815	chr5	1240642	C	G	SLC6A18	69	0.356	SNP
rs1440567(	chr5	1799789	T	TACCCGGC	MRPL36	30	0.155	INDEL
rs4147773;	chr5	1814669	T	C	NDUFS6	174	0.897	SNP
rs6233323!	chr5	2755371	C	T	C5orf38	83	0.428	SNP
rs1687492!	chr5	7301896	T	C	RP11-404K	78	0.402	SNP
.	chr5	7856774	AT	ATT	MTRR	73	0.376	INDEL
.	chr5	7856774	AT	A	MTRR	2	0.01	INDEL
rs10380;13	chr5	7897078	C	T	MTRR	7	0.036	SNP
rs1160006!	chr5	9658423	C	T	CTD-2001E	5	0.026	SNP
rs4128036:	chr5	10448263	T	A	ROPN1L	2	0.01	SNP

rs1385132:	chr5	10521708	TTTTCCATT	T	RP11-1C1.5	14	0.072	INDEL
rs5624918:	chr5	16671482	G	A	MYO10	2	0.01	SNP
rs6874374	chr5	24177835	A	G	C5orf17	128	0.66	SNP
rs3736393	chr5	33461929	G	A	TARS	4	0.021	SNP
rs1530507	chr5	33751349	A	T	ADAMTS12	148	0.763	SNP
rs1047290!	chr5	33994011	A	T	AMACR	97	0.5	SNP
rs1689209(	chr5	33994077	T	C	AMACR	8	0.041	SNP
rs3217251	chr5	34008101	CCGGCGCC	C	AMACR&C:	98	0.505	INDEL
rs1137096(	chr5	34685020	A	ATT	RAI14	114	0.588	INDEL
rs1137096(	chr5	34685020	A	AT	RAI14	80	0.412	INDEL
.	chr5	34925207	CTT	CT	BRIX1	23	0.119	INDEL
.	chr5	34925207	CTT	CTTT	BRIX1	27	0.139	INDEL
.	chr5	34925207	CTT	C	BRIX1	5	0.026	INDEL
rs6687551!	chr5	34925223	T	A	BRIX1	6	0.031	MIXED
rs6687551!	chr5	34925223	T	TA	BRIX1	39	0.201	MIXED
rs1689997:	chr5	34998673	C	A	AGXT2	106	0.546	SNP
rs1142861(	chr5	35012953	C	T	AGXT2	2	0.01	SNP
rs1457984!	chr5	36649326	CAACAAAA	C	CTD-2282P	91	0.469	INDEL
rs4371784	chr5	37687908	A	G	WDR70	70	0.361	SNP
rs3043270	chr5	40241414	AT	A	LINC00604	11	0.057	INDEL
rs3043270	chr5	40241414	AT	ATT	LINC00604	45	0.232	INDEL
rs1421382(	chr5	40569445	TA	T	TTC33	11	0.057	INDEL
rs1179486:	chr5	40569476	C	T	TTC33	2	0.01	SNP
rs1412445:	chr5	40841469	CTT	C	CARD6	5	0.026	INDEL
rs1023840	chr5	41061613	C	T	MROH2B	39	0.201	SNP
rs7620290!	chr5	41149933	A	G	C6	2	0.01	SNP
rs3592123:	chr5	41909728	CT	C	C5orf51	32	0.165	INDEL
rs3592123:	chr5	41909728	CT	CTT	C5orf51	6	0.031	INDEL
rs2972411	chr5	42466014	G	T	SERBP1P6	29	0.149	SNP
.	chr5	43179811	AT	A	ZNF131	24	0.124	INDEL
.	chr5	43179811	AT	ATTT	ZNF131	22	0.113	INDEL
rs5625714	chr5	43294090	C	T	HMGCS1	19	0.098	SNP
rs3733769	chr5	43297042	A	G	HMGCS1	100	0.515	SNP
rs1431811:	chr5	43602754	G	T	NNT	2	0.01	SNP
.	chr5	50741814	CT	CTT	PARP8	11	0.057	INDEL
.	chr5	50741814	CT	C	PARP8	6	0.031	INDEL
rs2233213;	chr5	53102175	T	C	MOCS2	9	0.046	SNP
rs1124062	chr5	55329343	C	CT	SKIV2L2	3	0.015	INDEL
rs1145827:	chr5	55767993	G	A	DDX4	3	0.015	SNP
.	chr5	55928656	GTT	GT	AC008914.	73	0.451	INDEL
.	chr5	55928656	GTT	GTTT	AC008914.	3	0.019	INDEL
.	chr5	55928656	GTT	G	AC008914.	11	0.068	INDEL
.	chr5	56100204	GT	G	ANKRD55	2	0.01	INDEL
rs7546447	chr5	56959385	C	A	MIER3	6	0.031	SNP
rs702722	chr5	58459024	A	G	PLK2	14	0.072	SNP
rs6787331!	chr5	58496722	G	T	GAPT	31	0.16	SNP
rs7276336!	chr5	61751668	A	G	C5orf64	8	0.041	SNP

.	chr5	65210450	CAA	CAAAA	CTD-2194F	10	0.052	INDEL
.	chr5	65210450	CAA	CAAA	CTD-2194F	44	0.227	INDEL
.	chr5	65210450	CAA	CA	CTD-2194F	45	0.232	INDEL
.	chr5	65210450	CAA	C	CTD-2194F	7	0.036	INDEL
.	chr5	65624708	CTCTTTTTT	CT	TRAPPC13E	41	0.211	INDEL
.	chr5	65624708	CTCTTTTTT	CTTTTTCTT	TRAPPC13E	17	0.088	INDEL
.	chr5	65624708	CTCTTTTTT	C	TRAPPC13E	4	0.021	INDEL
.	chr5	65624708	CTCTTTTTT	CTTTTTTCT	TRAPPC13E	30	0.155	INDEL
.	chr5	65624708	CTCTTTTTT	CTTTTTTTC	TRAPPC13E	9	0.046	INDEL
.	chr5	65624708	CTCTTTTTT	CTTT	TRAPPC13E	3	0.015	INDEL
rs1697144	chr5	67184176	T	C	CD180	194	1	SNP
rs706713;1	chr5	68226894	C	T	PIK3R1	48	0.247	SNP
.	chr5	70905179	T	C	SERF1A	3	0.025	MIXED
rs17606	chr5	72888271	C	T	TNPO1	175	0.902	SNP
rs7725317	chr5	75666988	G	A	ANKDD1B	3	0.015	SNP
rs34358	chr5	75669297	G	A	ANKDD1B	129	0.665	SNP
rs2003503	chr5	76173982	T	TC	AC113404.	6	0.031	INDEL
.	chr5	76173988	C	CA	AC113404.	6	0.031	INDEL
rs2005421	chr5	76173990	G	GTA	AC113404.	6	0.031	INDEL
rs31244	chr5	76298918	G	A	SV2C	16	0.082	SNP
rs3570719	chr5	77620503	A	T	WDR41	3	0.015	SNP
rs5868876	chr5	77620510	G	GC	WDR41	126	0.649	INDEL
rs1071598;	chr5	78885600	C	T	ARSB	30	0.155	SNP
rs6034083	chr5	79127798	C	T	BHMT	2	0.01	SNP
rs259120	chr5	79777993	A	G	CTC-431G1	52	0.268	SNP
rs1316773	chr5	80074424	G	T	THBS4	11	0.057	SNP
rs1115992	chr5	80300545	G	GTCCCTCT	CTC-512J1	23	0.119	INDEL
rs1115992	chr5	80300545	G	GTCCCTC	CTC-512J1	35	0.18	INDEL
rs1484107	chr5	81216963	G	A	RASGRF2	3	0.015	SNP
rs3460717	chr5	81345970	C	T	ACOT12	7	0.036	SNP
rs1138408	chr5	82275296	C	A	RPS23	2	0.01	SNP
rs1429983	chr5	82976282	C	T	ST13P12	2	0.01	SNP
rs1805377	chr5	83353124	G	A	XRCC4	26	0.134	SNP
rs1445495	chr5	85210191	CTTTATT	C	RP11-1072	3	0.015	INDEL
rs1363339	chr5	86749115	G	A	CTC-493L2	123	0.634	SNP
.	chr5	88270511	G	GT	TMEM161E	4	0.021	INDEL
rs3489413	chr5	90756446	T	TC	ADGRV1	79	0.407	INDEL
rs1457154	chr5	92913718	C	G	CTC-458G6	6	0.031	SNP
.	chr5	93741557	C	CG	POU5F2	2	0.01	INDEL
rs3539143	chr5	95414083	C	T	FAM81B	3	0.015	SNP
rs7906139	chr5	95793038	G	T	RHOBTB3	5	0.026	SNP
rs1748207	chr5	96783162	C	T	ERAP1	30	0.155	SNP
rs30187	chr5	96788627	T	C	ERAP1	134	0.691	SNP
rs4150665	chr5	96879976	C	T	ERAP2	22	0.113	SNP
rs3733905	chr5	96883857	C	T	ERAP2	3	0.015	SNP
rs2549782	chr5	96895296	G	T	ERAP2	107	0.552	SNP
rs1827808	chr5	97101758	G	A	CTD-2215E	2	0.01	SNP

.	chr5	97521189	ACCTATTA1	A	LINC01340	5	0.026	INDEL
rs2617354	chr5	1E+08	C	A	RP11-346J1	33	0.17	SNP
.	chr5	1.03E+08	CAAAAAAA	CAAAAAAA	CTD-2340E	42	0.219	INDEL
.	chr5	1.03E+08	CAAAAAAA	CA	CTD-2340E	64	0.333	INDEL
.	chr5	1.03E+08	CAAAAAAA	CAAAAAAA	CTD-2340E	10	0.052	INDEL
.	chr5	1.03E+08	CAAAAAAA	CAA	CTD-2340E	3	0.016	INDEL
rs7576137	chr5	1.09E+08	C	A	PJA2	7	0.036	SNP
rs1461923	chr5	1.09E+08	A	ATT	CTD-2128F	14	0.072	INDEL
rs1461923	chr5	1.09E+08	A	AT	CTD-2128F	5	0.026	INDEL
rs1461923	chr5	1.09E+08	A	ATTT	CTD-2128F	84	0.433	INDEL
rs1713415	chr5	1.12E+08	C	T	EPB41L4A	33	0.17	SNP
rs1457080	chr5	1.12E+08	C	CTAAAA	EPB41L4A	69	0.356	INDEL
rs2229992;	chr5	1.13E+08	T	C	APC	102	0.526	SNP
rs7254180	chr5	1.13E+08	G	A	APC	3	0.015	SNP
rs41115;42	chr5	1.13E+08	G	A	APC	105	0.541	SNP
rs6262357	chr5	1.13E+08	G	C	CTC-487M2	2	0.01	SNP
rs1138258	chr5	1.13E+08	GGTCACTG	G	MCC	54	0.278	INDEL
rs1477060	chr5	1.16E+08	A	T	ATG12	6	0.031	SNP
rs1872252	chr5	1.16E+08	G	A	AC026449.	2	0.01	SNP
.	chr5	1.16E+08	T	TATATATA1	LVRN	2	0.01	INDEL
.	chr5	1.19E+08	CT	CTT	TNFAIP8	16	0.082	INDEL
.	chr5	1.19E+08	CT	C	TNFAIP8	18	0.093	INDEL
rs25640;19	chr5	1.19E+08	G	A	HSD17B4	99	0.51	SNP
rs3217313	chr5	1.22E+08	AT	A	ZNF474	7	0.036	INDEL
rs451195	chr5	1.23E+08	T	C	PPIC	73	0.376	SNP
rs3434137	chr5	1.23E+08	T	C	PPIC	2	0.01	SNP
rs4544949	chr5	1.23E+08	TC	T	PPIC	2	0.01	INDEL
.	chr5	1.23E+08	TCTCTCTCT	T	AC106786.	4	0.022	INDEL
.	chr5	1.23E+08	TCTCTCTCT	*	AC106786.	6	0.033	INDEL
.	chr5	1.24E+08	T	C	CTD-2308B	5	0.026	SNP
rs6897488	chr5	1.26E+08	C	T	RP11-517I3	96	0.495	SNP
rs3401138	chr5	1.27E+08	G	GA	ALDH7A1	122	0.629	INDEL
.	chr5	1.29E+08	CT	CTTT	CTC-573M9	6	0.032	INDEL
.	chr5	1.29E+08	CT	CTT	CTC-573M9	18	0.097	INDEL
.	chr5	1.29E+08	CT	CTTTT	CTC-573M9	21	0.113	INDEL
.	chr5	1.29E+08	CT	C	CTC-573M9	13	0.07	INDEL
rs2278060;	chr5	1.31E+08	A	C	HINT1	48	0.247	SNP
rs3043838	chr5	1.32E+08	C	CTG	ACSL6	148	0.763	INDEL
rs1716576	chr5	1.32E+08	T	C	AC063976.	9	0.046	SNP
.	chr5	1.34E+08	G	GAACCTTG	FSTL4	4	0.022	INDEL
rs1071686	chr5	1.35E+08	CA	C	PITX1	75	0.387	INDEL
.	chr5	1.35E+08	CCCCCATC(	C	C5orf66&C	15	0.077	INDEL
.	chr5	1.35E+08	CCCCCATC(	CCCCCATC(	C5orf66&C	5	0.026	INDEL
.	chr5	1.35E+08	CCCCCATC(	CCCCCAT	C5orf66&C	2	0.01	INDEL
rs2078358	chr5	1.35E+08	A	G	C5orf66-AS	6	0.031	SNP
rs585063	chr5	1.35E+08	C	T	C5orf66-AS	10	0.052	SNP
rs3733728	chr5	1.35E+08	C	T	H2AFY	8	0.041	SNP

rs1252079!	chr5	1.35E+08	T	A	DCANP1	81	0.418	SNP
rs2001647!	chr5	1.36E+08	A	G	SLC25A48	3	0.015	SNP
rs4669;255	chr5	1.36E+08	T	C	TGFBI	55	0.284	SNP
rs1927182(	chr5	1.36E+08	A	AT	SMAD5	42	0.216	MIXED
rs1927182(	chr5	1.36E+08	A	T	SMAD5	3	0.015	MIXED
rs7999694!	chr5	1.38E+08	A	G	KLHL3	7	0.036	SNP
rs2301708;	chr5	1.38E+08	C	T	KLHL3	28	0.144	SNP
.	chr5	1.38E+08	CTTTT	CTTT	RPS27AP18	51	0.263	INDEL
.	chr5	1.38E+08	CTTTT	CTT	RPS27AP18	56	0.289	INDEL
.	chr5	1.38E+08	CTTTT	CT	RPS27AP18	6	0.031	INDEL
.	chr5	1.38E+08	CTTTT	CTTTTT	RPS27AP18	13	0.067	INDEL
.	chr5	1.38E+08	CTTTT	C	RPS27AP18	3	0.015	INDEL
.	chr5	1.39E+08	AT	ATT	HSPA9	60	0.309	INDEL
.	chr5	1.39E+08	AT	A	HSPA9	5	0.026	INDEL
.	chr5	1.39E+08	AT	ATTT	HSPA9	10	0.052	INDEL
rs1042665	chr5	1.39E+08	T	C	HSPA9	44	0.227	SNP
rs3491663:	chr5	1.39E+08	G	A	HSPA9	3	0.015	SNP
.	chr5	1.39E+08	CTTT	CTT	RPL12P21	15	0.077	INDEL
.	chr5	1.39E+08	CTTT	CTTTT	RPL12P21	4	0.021	INDEL
.	chr5	1.39E+08	CTTT	CT	RPL12P21	2	0.01	INDEL
rs1124246:	chr5	1.39E+08	C	T	SLC23A1	47	0.242	SNP
rs3085220	chr5	1.41E+08	A	AGT	SRA1	89	0.459	INDEL
rs5871740;	chr5	1.41E+08	C	CG	SRA1	89	0.459	INDEL
rs801175	chr5	1.41E+08	C	T	RP11-515C	40	0.206	SNP
.	chr5	1.41E+08	A	AAAGACAC	PCDHA4	2	0.01	INDEL
.	chr5	1.41E+08	GC	G	PCDHA4	76	0.396	INDEL
.	chr5	1.41E+08	AGG	A	PCDHA4	76	0.396	INDEL
rs1317288!	chr5	1.41E+08	G	A	CH17-140K	19	0.098	SNP
.	chr5	1.41E+08	CCCGGGCG	C	PCDHB3	2	0.01	INDEL
.	chr5	1.41E+08	T	TCC	PCDHB8	17	0.088	INDEL
.	chr5	1.41E+08	GTC	G	PCDHB8	17	0.088	INDEL
rs4912742	chr5	1.41E+08	T	C	CH17-140K	69	0.356	SNP
.	chr5	1.41E+08	G	GT	SLC25A2	2	0.01	INDEL
rs3214276	chr5	1.41E+08	GC	G	PCDHGA8	13	0.067	INDEL
.	chr5	1.41E+08	TA	TAA	PCDHGA10	8	0.041	INDEL
rs2005550!	chr5	1.41E+08	A	T	PCDHGA10	2	0.01	SNP
.	chr5	1.41E+08	CT	C	PCDHGA12	4	0.021	INDEL
rs2003094	chr5	1.41E+08	G	A	PCDHGB9P	11	0.057	SNP
rs164080	chr5	1.42E+08	G	A	GNPDA1	99	0.51	SNP
rs6878628	chr5	1.44E+08	G	A	CTD-2050E	22	0.113	SNP
rs2304066	chr5	1.46E+08	C	T	CTB-1H10.1	168	0.866	SNP
rs3703086!	chr5	1.47E+08	A	G	DPYSL3	2	0.01	SNP
rs1475412!	chr5	1.47E+08	T	C	DPYSL3	7	0.036	SNP
.	chr5	1.49E+08	TTG	T	HTR4	2	0.01	INDEL
rs250676	chr5	1.49E+08	A	T	SH3TC2	37	0.191	SNP
.	chr5	1.49E+08	A	G	ABLIM3	2	0.01	SNP
.	chr5	1.49E+08	TA	T	CSNK1A1	14	0.072	INDEL

.	chr5	1.49E+08	TA	TAA	CSNK1A1	52	0.268	INDEL
rs3832324	chr5	1.5E+08	CT	C	TIGD6	138	0.711	INDEL
rs4128709	chr5	1.5E+08	G	A	CSF1R	3	0.015	SNP
rs5632760	chr5	1.5E+08	G	C	CSF1R	4	0.021	SNP
rs1007925	chr5	1.5E+08	T	C	CSF1R	15	0.077	SNP
rs1765200	chr5	1.5E+08	G	A	CSF1R	2	0.01	SNP
rs4099171	chr5	1.51E+08	C	T	CTB-23I7.1	96	0.495	SNP
.	chr5	1.52E+08	A	C	FAT2	2	0.011	SNP
rs6890099	chr5	1.52E+08	A	G	ATOX1	40	0.206	SNP
rs3841128	chr5	1.53E+08	T	TC	GRIA1	4	0.021	INDEL
rs3734085	chr5	1.54E+08	G	A	GALNT10	2	0.01	SNP
rs1471921	chr5	1.55E+08	A	C	LARP1	3	0.015	SNP
rs1831720	chr5	1.56E+08	A	G	SGCD	2	0.01	SNP
rs1862874	chr5	1.57E+08	T	A	CYFIP2	59	0.304	SNP
rs1006341	chr5	1.57E+08	G	A	CTB-47B11	19	0.098	SNP
rs6067963	chr5	1.57E+08	CAAAAAAA	CAAAAAAA	CTB-109A1	58	0.299	INDEL
rs6067963	chr5	1.57E+08	CAAAAAAA	CAAAAAAA	CTB-109A1	17	0.088	INDEL
rs6067963	chr5	1.57E+08	CAAAAAAA	CAAAAAAA	CTB-109A1	12	0.062	INDEL
rs6067963	chr5	1.57E+08	CAAAAAAA	CAAAAAAA	CTB-109A1	15	0.077	INDEL
rs6067963	chr5	1.57E+08	CAAAAAAA	C	CTB-109A1	8	0.041	INDEL
rs1195529	chr5	1.58E+08	C	T	CLINT1	19	0.098	SNP
rs1368298	chr5	1.59E+08	A	G	EBF1	80	0.412	SNP
.	chr5	1.59E+08	C	CGG	RNF145	2	0.01	INDEL
rs4921230	chr5	1.59E+08	C	T	AC008703.	37	0.191	SNP
.	chr5	1.6E+08	CTT	CTTT	AC008697.	6	0.031	INDEL
.	chr5	1.6E+08	CTT	CT	AC008697.	36	0.186	INDEL
.	chr5	1.6E+08	CTT	C	AC008697.	20	0.103	INDEL
rs1436192	chr5	1.65E+08	C	CT	CTC-340A1	2	0.01	INDEL
rs264843	chr5	1.7E+08	G	A	DOCK2	158	0.814	SNP
rs1764749	chr5	1.7E+08	C	T	DOCK2	6	0.031	SNP
rs1007654	chr5	1.7E+08	A	G	KCNIP1	18	0.093	SNP
rs2306962	chr5	1.72E+08	C	T	STK10	134	0.691	SNP
rs3214436	chr5	1.73E+08	TC	T	ERGIC1	8	0.041	INDEL
rs3594189	chr5	1.73E+08	G	GT	ERGIC1	48	0.247	INDEL
rs1125058	chr5	1.73E+08	C	CA	CTC-308K2	6	0.031	INDEL
rs3749282	chr5	1.73E+08	AT	A	BNIP1	45	0.232	INDEL
rs3749282	chr5	1.73E+08	AT	ATT	BNIP1	8	0.041	INDEL
.	chr5	1.74E+08	C	CTGAACCA	BOD1	3	0.015	INDEL
.	chr5	1.74E+08	C	CTGAA	BOD1	4	0.021	INDEL
.	chr5	1.74E+08	C	CCACACTC	BOD1	2	0.01	INDEL
rs6158719	chr5	1.74E+08	CTGTGTG	CTGTG	LINC01484	94	0.485	INDEL
rs6158719	chr5	1.74E+08	CTGTGTG	CTG	LINC01484	42	0.216	INDEL
rs6158719	chr5	1.74E+08	CTGTGTG	C	LINC01484	14	0.072	INDEL
rs7589092	chr5	1.75E+08	CT	C	SFXN1	37	0.191	INDEL
rs7589092	chr5	1.75E+08	CT	CTT	SFXN1	9	0.046	INDEL
rs2241584	chr5	1.77E+08	G	A	RNF44	69	0.356	SNP
.	chr5	1.77E+08	TCAAAGAC	T	GPRIN1	47	0.242	INDEL

768051	chr5	1.77E+08	TCCTCCTTC	T	GPRIN1	47	0.242	INDEL
.	chr5	1.77E+08	CGGAGGA(	C	FGFR4	4	0.021	INDEL
rs1397309(	chr5	1.78E+08	C	T	B4GALT7	2	0.01	SNP
rs1487107:	chr5	1.78E+08	C	A	FAM153A	63	0.325	SNP
rs1174638:	chr5	1.79E+08	G	C	ZFP2	73	0.376	SNP
.	chr5	1.8E+08	CT	C	CANX	33	0.17	MIXED
.	chr5	1.8E+08	CT	CTT	CANX	31	0.16	MIXED
.	chr5	1.8E+08	CT	TT	CANX	2	0.01	MIXED
rs5603000:	chr5	1.8E+08	T	C	CANX	5	0.026	SNP
353157	chr5	1.8E+08	C	T	SQSTM1	3	0.015	SNP
rs3736062;	chr5	1.81E+08	G	A	FLT4	2	0.01	SNP
rs705441	chr5	1.81E+08	C	G	ZFP62	153	0.789	SNP
rs1405983(	chr5	1.81E+08	TTGTC	T	OR2V2	5	0.026	INDEL
rs7595335!	chr5	1.81E+08	G	A	CTC-338M:	12	0.062	SNP
.	chr5	1.81E+08	AC	A	TRIM52	2	0.01	INDEL
rs1159270:	chr6	1624644	C	T	GMDS	2	0.01	SNP
rs1221028(	chr6	2398865	G	A	GMDS-AS1	33	0.17	SNP
rs5908192:	chr6	3056478	AT	A	RP1-40E16	21	0.108	INDEL
rs5908192:	chr6	3056478	AT	ATT	RP1-40E16	13	0.067	INDEL
rs2272990	chr6	3076907	T	C	RIPK1	186	0.959	SNP
rs9503497	chr6	3255093	G	A	PSMG4	165	0.851	SNP
.	chr6	3754701	AAAGAC	A	RP11-420L!	7	0.036	INDEL
rs1713351:	chr6	7541915	C	CA	DSP	29	0.149	INDEL
rs2076300;	chr6	7584384	C	T	DSP	72	0.371	SNP
rs1155873:	chr6	7585437	C	A	DSP	13	0.067	SNP
rs1113311!	chr6	7883235	C	T	TXNDC5	6	0.031	SNP
rs9392961	chr6	8095396	G	A	EEF1E1	52	0.268	SNP
rs1118819:	chr6	9610828	T	C	OFCC1	4	0.021	SNP
rs2865183!	chr6	11490311	A	G	RP11-716O	7	0.036	SNP
rs7909121(	chr6	12583504	GAGAC	G	RP11-125M	7	0.036	INDEL
.	chr6	12749523	A	ACCCCTTC(	PHACTR1	2	0.014	INDEL
rs3430781!	chr6	13318096	A	G	TBC1D7	23	0.119	SNP
rs3757261	chr6	13599071	C	T	SIRT5	29	0.149	SNP
rs1142345;	chr6	18130687	T	C	TPMT	2	0.01	SNP
.	chr6	18367055	T	TTTTTG	RP11-528A	2	0.01	INDEL
rs6939160	chr6	19802086	C	G	RP4-625H1	46	0.237	SNP
rs7941930!	chr6	19804279	T	C	RP4-625H1	16	0.082	SNP
rs6173714!	chr6	20212179	T	C	RP11-239H	8	0.041	SNP
rs1139043(	chr6	21758380	A	AT	CASC15	105	0.541	INDEL
rs6696991-	chr6	24435815	ACG	A	GPLD1	6	0.031	INDEL
rs1052677(	chr6	24645840	TACACACA	TAC	KIAA0319	33	0.172	INDEL
rs1052677(	chr6	24645840	TACACACA	TACACACA	KIAA0319	19	0.099	INDEL
rs1052677(	chr6	24645840	TACACACA	TACACACA	KIAA0319	14	0.073	INDEL
rs1052677(	chr6	24645840	TACACACA	TACACACA	KIAA0319	12	0.063	INDEL
rs1052677(	chr6	24645840	TACACACA	T	KIAA0319	45	0.234	INDEL
rs1052677(	chr6	24645840	TACACACA	TACACACA	KIAA0319	24	0.125	INDEL
rs1155103!	chr6	24797412	A	G	C6orf229	5	0.026	SNP

rs9358799;	chr6	24806366	C	T	RP3-369A1	111	0.572	SNP
rs2328894	chr6	25777954	C	T	SLC17A4	2	0.01	SNP
rs7675783:	chr6	25969403	C	T	TRIM38	2	0.01	SNP
rs7284150!	chr6	26368051	G	A	BTN3A2	12	0.062	SNP
rs5836759!	chr6	26370605	T	G	BTN3A2	11	0.057	SNP
rs1380652!	chr6	26445783	G	A	BTN3A3	2	0.01	SNP
rs1422030(	chr6	26458639	G	A	BTN2A1	2	0.01	SNP
.	chr6	27831384	G	GA	HIST1H4K	2	0.01	INDEL
rs7654221	chr6	28230344	C	T	ZSCAN9	6	0.031	SNP
rs7338781(	chr6	28350389	G	A	ZKSCAN3	2	0.01	SNP
.	chr6	28382580	AG	A	ZSCAN12	2	0.01	INDEL
rs1384749!	chr6	31138723	T	TC	PSORS1C1	6	0.041	INDEL
.	chr6	32530181	A	AGT	HLA-DRB5	2	0.023	INDEL
.	chr6	32530182	GCTT	GT	HLA-DRB5	9	0.102	INDEL
.	chr6	32530182	GCTT	G	HLA-DRB5	2	0.023	INDEL
rs701884	chr6	32530185	T	C	HLA-DRB5	27	0.314	MIXED
rs701884	chr6	32530185	T	TGC	HLA-DRB5	8	0.093	MIXED
rs701884	chr6	32530185	T	*	HLA-DRB5	2	0.023	MIXED
.	chr6	33440599	CCT	C	SYNGAP1	2	0.012	INDEL
rs561276	chr6	33575339	C	T	BAK1	2	0.01	SNP
rs7874861(	chr6	33746635	T	C	IP6K3	8	0.041	SNP
rs1429936(	chr6	35141212	TC	T	TCP11	20	0.103	INDEL
rs662713	chr6	35920290	G	C	SRPK1	73	0.376	SNP
rs3761302	chr6	35989900	TTTTTG	T	DPRXP2	6	0.037	INDEL
rs1421710(	chr6	36225308	G	A	BRPF3	3	0.015	SNP
rs4562103:	chr6	36306371	T	A	PNPLA1	2	0.01	SNP
rs1995881	chr6	36452541	CTTTTTTT	C	KCTD20	50	0.26	INDEL
rs1995881	chr6	36452541	CTTTTTTT	CTTTTTT	KCTD20	7	0.036	INDEL
rs1995881	chr6	36452541	CTTTTTTT	CTTTTT	KCTD20	2	0.01	INDEL
rs1995881	chr6	36452541	CTTTTTTT	CTTTTTTTT	KCTD20	3	0.016	INDEL
.	chr6	36601940	CTT	CTTT	SRSF3	55	0.284	INDEL
.	chr6	36601940	CTT	CT	SRSF3	16	0.082	INDEL
.	chr6	36601940	CTT	C	SRSF3	3	0.015	INDEL
.	chr6	36601940	CTT	CTTTT	SRSF3	4	0.021	INDEL
.	chr6	36601940	CTT	CTTTTT	SRSF3	8	0.041	INDEL
rs2071823	chr6	36856623	C	T	PPIL1	41	0.211	SNP
rs1219440!	chr6	36871822	C	G	PPIL1	10	0.052	SNP
rs3553895!	chr6	36986143	AG	A	MTCH1	14	0.072	INDEL
rs2284922	chr6	37381257	G	A	RNF8	62	0.32	SNP
rs1385739!	chr6	39315040	G	A	KCNK16	3	0.015	SNP
rs4127335(	chr6	41042779	G	A	TSPO2	3	0.015	SNP
rs2073014	chr6	41061370	T	C	APOBEC2	46	0.237	SNP
rs1319132:	chr6	41071106	C	T	OARD1	49	0.253	SNP
rs5624671:	chr6	41137356	T	G	ADCY10P1	96	0.495	SNP
rs2234237	chr6	41282728	T	A	TREM1	21	0.108	SNP
rs7476886(	chr6	41753896	A	T	PGC	9	0.046	SNP
rs5964469	chr6	41753944	G	GT	PGC	81	0.418	INDEL

rs2013388	chr6	41798694	C	CCCCCA	USP49	11	0.057	INDEL
rs9471775	chr6	42126815	C	T	C6orf132	32	0.165	SNP
rs6241461	chr6	42592188	G	A	UBR2	11	0.057	SNP
.	chr6	42644198	TA	TAA	UBR2	6	0.031	INDEL
.	chr6	42644198	TA	T	UBR2	16	0.082	INDEL
rs6173235	chr6	42890786	C	T	C6orf226	4	0.021	SNP
rs7832	chr6	43226139	G	A	DNPH1	18	0.093	SNP
rs3588980	chr6	44168930	A	AT	CAPN11	70	0.361	INDEL
rs5728879	chr6	44172317	T	TGGCTGCC	CAPN11	11	0.057	INDEL
rs7451713	chr6	46140015	C	G	ENPP4	100	0.515	SNP
rs9472696	chr6	46141179	C	G	ENPP4	12	0.062	SNP
rs1805017;	chr6	46716485	C	T	PLA2G7	57	0.294	SNP
rs1234122	chr6	46905257	A	G	RP3-365O1	99	0.51	SNP
.	chr6	52423657	ATT	AT	EFHC1	20	0.128	INDEL
.	chr6	52423657	ATT	ATTTT	EFHC1	3	0.019	INDEL
.	chr6	52423657	ATT	A	EFHC1	5	0.032	INDEL
.	chr6	52423657	ATT	ATTT	EFHC1	5	0.032	INDEL
rs1051775	chr6	52794164	T	C	GSTA1	80	0.412	SNP
rs4127385	chr6	52902401	T	C	GSTA3	3	0.015	SNP
rs4127387	chr6	53269166	C	A	ELOVL5	3	0.015	SNP
rs2005396	chr6	57320446	C	CT	PRIM2	2	0.01	INDEL
rs2230042	chr6	57382049	C	T	PRIM2	13	0.067	SNP
.	chr6	62548007	TA	T	RP11-448N	11	0.057	INDEL
rs701655	chr6	69232614	G	A	ADGRB3	17	0.088	SNP
rs2000426	chr6	70668651	AC	A	SMAP1	9	0.046	INDEL
rs7748778	chr6	70837869	A	T	SMAP1	8	0.041	SNP
rs4707908	chr6	71304347	C	T	RP11-154D	72	0.371	SNP
rs7977242	chr6	72390886	G	A	RIMS1	8	0.041	SNP
rs7232301	chr6	73263055	GGCGGCGG	G	KHDC1	150	0.773	INDEL
rs1688357	chr6	73309615	G	A	KHDC1	18	0.093	SNP
rs311678	chr6	73425293	C	T	MB21D1	135	0.696	SNP
.	chr6	75646835	CAA	CA	SENP6	20	0.103	INDEL
.	chr6	75646835	CAA	CAAA	SENP6	8	0.041	INDEL
.	chr6	75934969	AG	A	IMPG1	2	0.01	INDEL
rs3553361	chr6	78903027	CA	C	IRAK1BP1	38	0.196	INDEL
rs6788067	chr6	81728255	A	G	RP5-991C6	9	0.046	SNP
rs1487154	chr6	82365314	C	T	TPBG	3	0.015	SNP
rs2230900	chr6	83398372	C	T	ME1	13	0.067	SNP
rs1266542	chr6	85156971	A	T	RP3-455E7	23	0.119	SNP
rs2229524	chr6	85489525	T	C	NT5E	12	0.062	SNP
rs2011192	chr6	87086515	G	A	CGA	93	0.479	SNP
rs2838146	chr6	87595171	G	A	ORC3	2	0.01	SNP
rs4590242	chr6	89203490	C	T	GABRR1	120	0.619	SNP
.	chr6	89867987	TG	T	CASP8AP2	23	0.119	INDEL
.	chr6	89867992	TCTTTGCCC	T	CASP8AP2	23	0.119	INDEL
rs2236181	chr6	89951857	T	C	BACH2	67	0.345	SNP
rs598218	chr6	91685047	C	T	CASC6	32	0.165	SNP

.	chr6	95586993	G	GTATA	MANEA	2	0.01	INDEL
rs1489676(	chr6	95587005	A	ATATG	MANEA	77	0.397	INDEL
rs1489676(	chr6	95587005	A	ATATATG	MANEA	17	0.088	INDEL
rs1489676(	chr6	95587005	A	ATATATGT(	MANEA	3	0.015	INDEL
rs1489676(	chr6	95587005	A	ATATATAT(	MANEA	3	0.015	INDEL
rs2009880(	chr6	95587019	CTG	C	MANEA	15	0.077	INDEL
.	chr6	96552465	CT	C	UFL1	18	0.093	INDEL
.	chr6	96552465	CT	CTT	UFL1	7	0.036	INDEL
rs9386930	chr6	96896083	C	T	NDUFAF4	62	0.32	SNP
rs1457310!	chr6	97373915	G	A	RP11-436D	5	0.026	SNP
.	chr6	99558419	TA	T	CCNC	6	0.031	INDEL
rs9486410	chr6	1.07E+08	A	G	RTN4IP1	10	0.052	SNP
.	chr6	1.07E+08	CTGTACTT(	C	PDSS2	8	0.041	INDEL
rs9386758	chr6	1.09E+08	T	C	ARMC2	4	0.021	SNP
rs1731611(	chr6	1.09E+08	A	C	ARMC2	66	0.34	SNP
.	chr6	1.09E+08	AT	ATT	CEP57L1	89	0.459	INDEL
.	chr6	1.09E+08	AT	A	CEP57L1	6	0.031	INDEL
rs6174358!	chr6	1.09E+08	A	T	CEP57L1	2	0.01	SNP
rs9480921	chr6	1.09E+08	G	A	CCDC162P	106	0.546	SNP
rs3544491	chr6	1.09E+08	TC	T	SMPD2	12	0.062	INDEL
rs7233139	chr6	1.1E+08	AAC	A	AK9	118	0.608	INDEL
rs3060758	chr6	1.1E+08	AC	*	AK9	119	0.613	INDEL
rs3060758	chr6	1.1E+08	AC	A	AK9	3	0.015	INDEL
rs1137710(	chr6	1.1E+08	G	GT	FIG4	128	0.66	MIXED
rs1137710(	chr6	1.1E+08	G	GTT	FIG4	10	0.052	MIXED
rs5581792	chr6	1.1E+08	G	A	WASF1	4	0.021	SNP
rs3463791	chr6	1.11E+08	T	TC	AMD1	110	0.567	INDEL
rs3463791	chr6	1.11E+08	T	TCC	AMD1	33	0.17	INDEL
.	chr6	1.11E+08	CTT	CT	RPF2	49	0.253	INDEL
.	chr6	1.11E+08	CTT	C	RPF2	5	0.026	INDEL
rs1175057	chr6	1.11E+08	T	A	MFSD4B	3	0.015	SNP
rs9481421	chr6	1.14E+08	G	A	RP3-399L1!	27	0.139	SNP
rs9488412	chr6	1.15E+08	C	T	RP3-399L1!	10	0.052	SNP
rs2228548;	chr6	1.16E+08	C	G	COL10A1	13	0.067	SNP
rs1173613(	chr6	1.16E+08	T	G	FAM26F	25	0.129	SNP
rs7716507!	chr6	1.17E+08	CT	C	RWDD1	54	0.278	INDEL
rs7716507!	chr6	1.17E+08	CT	CTT	RWDD1	7	0.036	INDEL
769689	chr6	1.17E+08	A	AGG	GPRC6A	11	0.057	INDEL
769690	chr6	1.17E+08	T	TA	GPRC6A	11	0.057	INDEL
rs6907580	chr6	1.17E+08	G	A	GPRC6A	10	0.052	SNP
rs1451044(	chr6	1.17E+08	C	A	ROS1	2	0.01	SNP
rs552183	chr6	1.17E+08	T	G	ROS1	23	0.119	SNP
rs1493905!	chr6	1.21E+08	CCTTA	C	TBC1D32	3	0.015	INDEL
rs1991642	chr6	1.23E+08	C	G	PKIB	54	0.278	SNP
rs1266508	chr6	1.23E+08	G	A	RP11-532N	93	0.479	SNP
rs1049913	chr6	1.25E+08	T	C	RP11-138M	41	0.211	SNP
rs5600606	chr6	1.26E+08	A	T	RP1-293L8.	4	0.021	SNP

.	chr6	1.3E+08	A	G	SAMD3	2	0.01	SNP
rs3450448:	chr6	1.32E+08	C	T	ARG1	13	0.067	SNP
.	chr6	1.33E+08	AC	A	STX7	7	0.036	INDEL
.	chr6	1.33E+08	A	T	TAAR9	48	0.247	SNP
rs8192646	chr6	1.33E+08	C	T	TAAR2	4	0.021	SNP
rs3449243	chr6	1.33E+08	C	A	VNN2	7	0.036	SNP
rs1138523(	chr6	1.33E+08	A	AG	LINC00326	30	0.155	INDEL
rs1476574	chr6	1.34E+08	T	C	SGK1	3	0.015	SNP
rs1175907	chr6	1.35E+08	A	G	CTA-212D2	90	0.464	SNP
rs1390549(	chr6	1.38E+08	T	G	RP11-356I2	2	0.01	SNP
.	chr6	1.38E+08	GCCCGGAG	G	TNFAIP3	7	0.036	INDEL
rs1465346	chr6	1.38E+08	A	G	TNFAIP3	2	0.01	SNP
rs3841283	chr6	1.38E+08	A	AG	PBOV1	23	0.119	INDEL
rs1180467	chr6	1.38E+08	G	A	HEBP2	2	0.01	SNP
rs1489201(	chr6	1.39E+08	G	A	RP1-225E1	2	0.01	SNP
rs4128981	chr6	1.39E+08	G	A	TXLNB	30	0.155	SNP
rs1338532	chr6	1.43E+08	G	A	VDAC1P8	3	0.015	SNP
.	chr6	1.44E+08	CAT	C	ZC2HC1B	2	0.01	INDEL
.	chr6	1.44E+08	CTTGAA	C	ZC2HC1B	3	0.015	INDEL
rs3737260	chr6	1.45E+08	G	C	UTRN	14	0.072	SNP
rs7250173	chr6	1.5E+08	G	C	ULBP3	3	0.015	SNP
rs7171354	chr6	1.53E+08	GAGAT	G	MYCT1	9	0.046	INDEL
rs677830;4	chr6	1.54E+08	C	T	OPRM1	60	0.309	SNP
rs3442788	chr6	1.54E+08	C	T	OPRM1	13	0.067	SNP
rs3211067	chr6	1.58E+08	G	A	SNX9	49	0.253	SNP
rs1494007(	chr6	1.58E+08	G	A	SYNJ2-IT1	4	0.021	SNP
rs1127804	chr6	1.58E+08	A	T	SERAC1	3	0.015	SNP
.	chr6	1.59E+08	CACCA	C	FNDC1	3	0.016	INDEL
rs3464	chr6	1.6E+08	C	T	ACAT2	38	0.196	SNP
rs1464374	chr6	1.6E+08	C	G	ACAT2	2	0.01	SNP
rs1094564	chr6	1.6E+08	A	T	AIRN	24	0.124	SNP
rs2048328	chr6	1.6E+08	C	T	LPAL2	35	0.18	SNP
rs4127211	chr6	1.61E+08	C	T	LPA	7	0.036	SNP
rs1801474;	chr6	1.62E+08	C	T	PARK2	12	0.062	SNP
.	chr6	1.62E+08	T	A	PARK2	2	0.01	SNP
rs9364644	chr6	1.62E+08	G	C	PARK2	2	0.01	SNP
rs1488452	chr6	1.65E+08	A	G	PDE10A	4	0.021	SNP
rs1511746	chr6	1.65E+08	G	A	PDE10A	31	0.16	SNP
rs220740	chr6	1.65E+08	C	T	PDE10A	58	0.299	SNP
rs11159;35	chr6	1.67E+08	G	A	RNASET2	11	0.057	SNP
rs2235197	chr6	1.67E+08	G	A	UNC93A	23	0.119	SNP
rs1139066	chr6	1.67E+08	G	C	UNC93A	2	0.01	SNP
rs1453608	chr6	1.67E+08	C	T	UNC93A	2	0.01	SNP
rs3725360	chr6	1.67E+08	CA	C	TCP10	10	0.053	INDEL
.	chr6	1.67E+08	A	T	TCP10	10	0.054	SNP
rs9457321	chr6	1.67E+08	A	C	TCP10	150	0.773	SNP
.	chr6	1.68E+08	GAATGCAA	GAATGCAA	RP11-503C	32	0.165	INDEL

rs6925614	chr6	1.68E+08	T	C	DACT2	68	0.351	SNP
.	chr6	1.7E+08	AC	A	WDR27	4	0.021	INDEL
rs3055	chr6	1.7E+08	C	T	WDR27	62	0.32	SNP
403524	chr6	1.71E+08	AGC	*	TBP	3	0.015	INDEL
403524	chr6	1.71E+08	AGC	A	TBP	61	0.314	INDEL
.	chr6	1.71E+08	AGCAGCAG	ACAGCAGC	TBP	16	0.082	INDEL
.	chr6	1.71E+08	AGCAGCAG	ACAGCAGC	TBP	38	0.196	INDEL
.	chr6	1.71E+08	AGCAGCAG	ACAGCAG	TBP	6	0.031	INDEL
rs1042327;	chr6	1.71E+08	T	C	TBP	120	0.619	SNP
rs1438965	chr7	887154	G	GCGTC	GET4	4	0.021	INDEL
rs6173095	chr7	904231	G	A	ADAP1	16	0.082	SNP
rs7664917	chr7	1093188	TTC	T	GPER1	4	0.021	INDEL
.	chr7	2288347	TA	T	IMMP1LP3	32	0.165	INDEL
.	chr7	2288347	TA	TAA	IMMP1LP3	11	0.057	INDEL
.	chr7	2403653	GCGCGAGC	G	CHST12	4	0.021	INDEL
591987	chr7	2513247	AGATGGAT	AGATGGAT	LFNG	62	0.32	INDEL
591987	chr7	2513247	AGATGGAT	AGATG	LFNG	2	0.01	INDEL
rs5591942	chr7	2712425	C	T	AMZ1	10	0.052	SNP
rs798489	chr7	2762169	C	T	GNA12	49	0.253	SNP
rs798488	chr7	2762888	T	C	GNA12	52	0.268	SNP
rs9689983	chr7	4984909	G	A	RNF216P1	13	0.067	SNP
.	chr7	5390358	CAAAA	CAAA	TNRC18	92	0.484	INDEL
.	chr7	5390358	CAAAA	C	TNRC18	4	0.021	INDEL
.	chr7	5390358	CAAAA	CA	TNRC18	25	0.132	INDEL
.	chr7	5390358	CAAAA	CAA	TNRC18	28	0.147	INDEL
rs1640233	chr7	5603514	T	C	FSCN1	27	0.139	SNP
rs1461760	chr7	6005969	C	G	PMS2	3	0.015	SNP
rs2301910	chr7	6165368	A	C	CYTH3	135	0.696	SNP
.	chr7	6170826	TCA	T	CYTH3	2	0.01	INDEL
rs1638213	chr7	7277584	G	C	AC005532.	78	0.402	SNP
rs7240228	chr7	7673310	TAGCAGCA	TAGCAGC	UMAD1	83	0.428	INDEL
rs7240228	chr7	7673310	TAGCAGCA	TAGCAGCA	UMAD1	26	0.134	INDEL
rs7240228	chr7	7673310	TAGCAGCA	TAGCAGCA	UMAD1	12	0.062	INDEL
rs7240228	chr7	7673310	TAGCAGCA	TAGCAGCA	UMAD1	16	0.082	INDEL
rs7240228	chr7	7673310	TAGCAGCA	TAGC	UMAD1	9	0.046	INDEL
rs7240228	chr7	7673310	TAGCAGCA	T	UMAD1	9	0.046	INDEL
rs7101476	chr7	8156929	TAAAAAAA	T	ICA1	30	0.155	INDEL
.	chr7	8156936	AAAAAAAA	A	ICA1	4	0.021	INDEL
.	chr7	8156936	AAAAAAAA	*	ICA1	30	0.155	INDEL
rs1145453	chr7	12351642	T	TA	VWDE	25	0.129	INDEL
rs1716593	chr7	12375099	G	A	VWDE	24	0.124	SNP
rs1808802	chr7	12523125	T	C	AC005281.	2	0.01	SNP
rs1142906	chr7	12523234	G	GA	AC005281.	21	0.108	INDEL
rs6460967	chr7	12571627	T	C	SCIN	109	0.562	SNP
rs6943959	chr7	13102006	A	G	AC011288.	35	0.18	SNP
791269	chr7	16462576	CT	C	SOSTDC1	3	0.015	INDEL
rs3405231	chr7	16682767	G	GT	BZW2	4	0.021	INDEL

rs4719497	chr7	17295275	T	C	AC003075.	21	0.108	SNP
rs1427894	chr7	18027344	CATACA	C	PRPS1L1	2	0.01	INDEL
rs1714318	chr7	20626612	G	C	ABCB5	2	0.01	SNP
rs3460355	chr7	20651424	T	C	ABCB5	30	0.155	SNP
rs2285943;	chr7	21543345	G	T	DNAH11	73	0.376	SNP
rs1467720	chr7	21851321	G	A	DNAH11	9	0.046	SNP
.	chr7	22817411	CT	CTT	TOMM7	6	0.031	INDEL
.	chr7	22817411	CT	C	TOMM7	8	0.041	INDEL
rs1405084	chr7	23194941	GCCTC	G	NUPL2	10	0.052	INDEL
.	chr7	23686292	CT	C	FAM221A	5	0.026	INDEL
.	chr7	23686294	T	A	FAM221A	5	0.026	SNP
rs7802834	chr7	23688325	T	C	FAM221A	158	0.814	SNP
rs6945306	chr7	23717543	G	C	STK31	51	0.263	SNP
rs1126901	chr7	29001025	AAGGAGG	A	CTB-113D1	132	0.68	INDEL
.	chr7	29660294	TAA	T	DPY19L2P3	19	0.098	INDEL
.	chr7	30115970	GTGCATGC	*	Clostridiale	4	0.023	MIXED
.	chr7	30115970	GTGCATGC	ATGCATGC	Clostridiale	3	0.017	MIXED
rs7521040	chr7	30533395	G	C	RP4-777O2	3	0.015	SNP
rs1253396	chr7	30572998	A	G	AC005154.	127	0.655	SNP
rs2302339	chr7	30755672	A	G	INMT	70	0.361	SNP
rs6970605	chr7	30755832	G	A	INMT	3	0.015	SNP
rs6174173	chr7	30755850	G	C	INMT	2	0.01	SNP
rs3558977	chr7	31658299	C	CT	CCDC129	60	0.309	INDEL
rs2014823	chr7	32755409	C	CT	DPY19L1P1	33	0.17	INDEL
198295	chr7	33095235	CA	C	RP9	2	0.01	INDEL
rs1027502	chr7	34849610	T	C	NPSR1	59	0.304	SNP
rs7809642	chr7	34878128	C	T	NPSR1	37	0.191	SNP
rs7809642	chr7	34878128	C	A	NPSR1	17	0.088	SNP
rs3753985	chr7	36425997	CT	C	ANLN	42	0.216	INDEL
rs3753985	chr7	36425997	CT	CTT	ANLN	10	0.052	INDEL
rs5588940	chr7	38201712	A	G	STARD3NL	54	0.278	SNP
rs7680745	chr7	38291815	C	T	TRGV11	23	0.119	SNP
.	chr7	39339680	C	CA	POU6F2	2	0.01	INDEL
.	chr7	39339682	C	CAG	POU6F2	2	0.01	INDEL
rs5954898	chr7	42802330	AAAAT	A	TCP1P1	145	0.747	INDEL
rs5954898	chr7	42802330	AAAAT	AAAATAAA	TCP1P1	10	0.052	INDEL
rs1902209	chr7	43044546	C	T	AC005537.	2	0.01	SNP
rs2730616	chr7	43879667	C	T	URGCP	140	0.722	SNP
rs2838264	chr7	44078724	GC	G	POLM	10	0.052	INDEL
rs5883888	chr7	44140480	A	AG	MYL7	194	1	INDEL
rs1154222	chr7	44243269	A	G	CAMK2B	101	0.521	SNP
rs1154222	chr7	44243278	A	G	CAMK2B	88	0.454	SNP
rs1143766	chr7	44539736	G	A	NPC1L1	8	0.041	SNP
rs1482441	chr7	45063959	C	T	CCM2	5	0.026	SNP
rs4988515	chr7	45893001	C	T	IGFBP1	4	0.021	SNP
rs1176557	chr7	45921672	G	A	IGFBP3	34	0.175	SNP
rs1120147	chr7	46674252	C	T	AC011294.	19	0.098	SNP

rs6975704	chr7	46678671	A	G	AC011294.	55	0.284	SNP
rs1415769	chr7	48412584	G	T	ABCA13	2	0.01	SNP
rs1157554	chr7	50463289	C	T	DDC	2	0.01	SNP
rs1157537	chr7	50504057	C	A	DDC	3	0.015	SNP
rs1157530	chr7	50539996	G	A	DDC	2	0.01	SNP
rs6175658	chr7	50604057	G	A	GRB10	3	0.015	SNP
rs7818255	chr7	54557296	G	C	VSTM2A-O	10	0.052	SNP
rs1050171;	chr7	55181370	G	A	EGFR	128	0.66	SNP
rs2229066;	chr7	55191757	C	T	EGFR	5	0.026	SNP
rs1391905	chr7	55764950	A	T	PSPHP1	3	0.015	SNP
.	chr7	56289869	CAAA	CAAAA	RP11-700P	19	0.112	INDEL
.	chr7	56289869	CAAA	CAAAAA	RP11-700P	15	0.088	INDEL
.	chr7	56289869	CAAA	CAAAAAA	RP11-700P	7	0.041	INDEL
.	chr7	56289869	CAAA	CAAAAAAA	RP11-700P	2	0.012	INDEL
.	chr7	56289869	CAAA	CAA	RP11-700P	2	0.012	INDEL
.	chr7	56289869	CAAA	C	RP11-700P	2	0.012	INDEL
rs1849674	chr7	56632136	T	C	TRIM60P16	162	0.835	SNP
rs7495303	chr7	57061175	C	G	PHKG1P4	14	0.072	SNP
.	chr7	57416007	G	A	RP11-114G	180	1	SNP
rs1609527	chr7	63138879	C	T	SEPT14P1	134	0.691	SNP
rs5758515	chr7	63148830	TG	T	SEPT14P1	50	0.26	INDEL
.	chr7	63148831	GT	*	SEPT14P1	50	0.26	MIXED
.	chr7	63148831	GT	TT	SEPT14P1	3	0.016	MIXED
rs3738439	chr7	63574705	G	A	TNRC18P2	5	0.026	SNP
rs1404453	chr7	64978289	G	A	ZNF117	185	0.954	SNP
rs6704782	chr7	64992360	G	A	ERV3-1	26	0.134	SNP
.	chr7	65140971	GC	G	AC104073.	9	0.057	INDEL
.	chr7	65140971	GC	*	AC104073.	2	0.013	INDEL
.	chr7	65292469	CAAAAAA	CAAAAAAA	RP11-746P	6	0.038	INDEL
.	chr7	65292469	CAAAAAA	CAA	RP11-746P	43	0.276	INDEL
.	chr7	65292469	CAAAAAA	CA	RP11-746P	4	0.026	INDEL
.	chr7	65292469	CAAAAAA	CAAAAAAA	RP11-746P	6	0.038	INDEL
rs3775933	chr7	65555008	CG	C	AC104057.	14	0.538	INDEL
.	chr7	65647610	GC	G	RP11-479O	99	0.569	INDEL
.	chr7	65647610	GC	*	RP11-479O	35	0.201	INDEL
.	chr7	65647611	CCCCCCGT	*	RP11-479O	125	0.753	INDEL
.	chr7	65647611	CCCCCCGT	C	RP11-479O	2	0.012	INDEL
rs1061361;	chr7	65964372	G	A	GUSB	33	0.17	SNP
rs6963583	chr7	67339574	G	A	LINC01372	62	0.32	SNP
rs3491852	chr7	68097860	A	AT	MTCO1P57	114	0.588	MIXED
rs3491852	chr7	68097860	A	ATTT	MTCO1P57	19	0.098	MIXED
.	chr7	72728896	C	T	TYW1B	142	0.732	SNP
.	chr7	72744551	C	CA	TYW1B	147	0.758	INDEL
.	chr7	72942080	CGT	C	POM121	8	0.041	INDEL
rs4146324	chr7	75771945	C	T	CCL26	4	0.021	SNP
rs1173154	chr7	75823321	C	A	CCL24	11	0.057	SNP
rs2072435	chr7	76329871	G	A	YWHAG	64	0.33	SNP

.	chr7	76393371	CCGCTGTC.	C	SSC4D	3	0.015	INDEL
rs2428937	chr7	78039633	T	C	MAGI2	23	0.119	SNP
rs1177337!	chr7	78158312	G	A	MAGI2	15	0.077	SNP
rs1715355!	chr7	80188961	A	G	GNAI1	4	0.021	SNP
rs5745635;	chr7	81758726	T	C	HGF	3	0.015	SNP
rs274624	chr7	86644973	T	C	GRM3	112	0.577	SNP
rs3214245	chr7	87184806	CT	C	DMTF1	2	0.01	INDEL
rs3214245	chr7	87184806	CT	CTT	DMTF1	7	0.036	INDEL
rs1786582(	chr7	90487326	G	A	CLDN12	12	0.062	SNP
rs7322813:	chr7	91556735	G	A	RP11-142A	26	0.134	SNP
rs2521434	chr7	91693396	G	A	MTERF1	133	0.686	SNP
rs1154268:	chr7	92213240	T	C	KRIT1	26	0.134	SNP
rs6714260!	chr7	92535004	C	T	RBM48	6	0.031	SNP
rs3425756!	chr7	92725719	T	A	CDK6	5	0.026	SNP
rs1500706!	chr7	93134756	G	A	SAMD9L	6	0.031	SNP
rs2214098	chr7	93246118	A	G	VPS50	2	0.01	SNP
.	chr7	93320362	CT	C	VPS50	2	0.011	INDEL
.	chr7	93320362	CT	CTT	VPS50	2	0.011	INDEL
rs2106735	chr7	93441499	C	T	CALCR	41	0.211	SNP
rs1770448(	chr7	95587034	T	C	PDK4	2	0.01	SNP
rs1154129!	chr7	95587109	G	A	PDK4	16	0.082	SNP
rs1095328!	chr7	99415262	G	C	BUD31	7	0.036	SNP
rs1172431!	chr7	99559023	T	C	ZNF655	4	0.021	SNP
rs6977165	chr7	99671774	T	C	CYP3A5	5	0.026	SNP
.	chr7	99672916	T	C	CYP3A5	188	0.969	SNP
rs6146981(	chr7	99836454	TA	T	CYP3A43	9	0.046	INDEL
.	chr7	99849955	ATT	AT	CYP3A43	28	0.144	INDEL
.	chr7	99849955	ATT	ATTT	CYP3A43	12	0.062	INDEL
.	chr7	99849955	ATT	A	CYP3A43	7	0.036	INDEL
rs1176742	chr7	99902176	T	C	TRIM4	4	0.021	SNP
rs7168966	chr7	1E+08	GTAGT	G	ZNF3	52	0.268	INDEL
rs4128096	chr7	1E+08	G	A	AP4M1	19	0.098	SNP
rs3835191	chr7	1E+08	C	CAGTG	STAG3	42	0.216	INDEL
rs7786505	chr7	1E+08	G	T	PVRIG	48	0.247	SNP
.	chr7	1E+08	CTG	C	NYAP1	2	0.01	INDEL
.	chr7	1E+08	CGG	C	NYAP1	2	0.01	INDEL
.	chr7	1E+08	CGCCTTGC	C	NYAP1	2	0.01	INDEL
rs1174067(	chr7	1.01E+08	G	A	ZAN	4	0.021	SNP
rs2293766	chr7	1.01E+08	G	A	ZAN	3	0.015	SNP
.	chr7	1.01E+08	TG	T	ZAN	12	0.062	INDEL
.	chr7	1.01E+08	G	GTT	ZAN	92	0.474	MIXED
.	chr7	1.01E+08	G	*	ZAN	12	0.062	MIXED
rs482308	chr7	1.01E+08	G	A	ZAN	87	0.448	SNP
rs7236464	chr7	1.01E+08	GGCTTCAG	G	ZAN	96	0.495	INDEL
rs5617307	chr7	1.01E+08	A	G	EPHB4	6	0.031	SNP
.	chr7	1.01E+08	AGC	A	MUC3A	14	0.072	INDEL
.	chr7	1.01E+08	A	AGT	MUC3A	14	0.072	INDEL

.	chr7	1.01E+08	TCCTAC	T	MUC3A	4	0.056	INDEL
.	chr7	1.01E+08	TCCTAC	*	MUC3A	14	0.194	INDEL
.	chr7	1.01E+08	C	CACAT	MUC3A	3	0.015	INDEL
.	chr7	1.01E+08	CCTTA	C	MUC3A	3	0.015	INDEL
rs1129646	chr7	1.01E+08	A	ACCCCC	MUC3A	4	0.021	MIXED
.	chr7	1.01E+08	ATC	A	MUC3A	47	0.242	INDEL
.	chr7	1.01E+08	C	CGT	MUC3A	47	0.242	INDEL
.	chr7	1.01E+08	C	G	RP11-395B	21	0.108	SNP
.	chr7	1.01E+08	T	TCAGAA	MUC17	2	0.01	INDEL
.	chr7	1.01E+08	C	CAG	MUC17	3	0.015	INDEL
rs1466657	chr7	1.01E+08	A	AACAGGG	FIS1	6	0.031	INDEL
.	chr7	1.02E+08	TGCCCTGG	T	RASA4B	11	0.067	INDEL
rs1172749	chr7	1.03E+08	G	A	NFE4	8	0.041	SNP
.	chr7	1.06E+08	CTT	CTTT	CDHR3	3	0.018	INDEL
.	chr7	1.06E+08	CTT	CT	CDHR3	5	0.029	INDEL
rs1445657	chr7	1.07E+08	G	A	PIK3CG	4	0.021	SNP
rs6174991	chr7	1.07E+08	A	G	PIK3CG	2	0.01	SNP
rs2230460	chr7	1.07E+08	C	T	PIK3CG	17	0.088	SNP
771727	chr7	1.08E+08	AT	A	LAMB4	2	0.01	INDEL
rs428459	chr7	1.08E+08	T	C	NRCAM	164	0.845	SNP
rs1457046	chr7	1.13E+08	AAAAAC	A	NPM1P14	92	0.474	INDEL
rs1457046	chr7	1.13E+08	AAAAAC	AAAAACAA	NPM1P14	18	0.093	INDEL
rs1457046	chr7	1.13E+08	AAAAAC	AAAAACAA	NPM1P14	10	0.052	INDEL
436408	chr7	1.14E+08	CCT	C	PPP1R3A	3	0.015	INDEL
.	chr7	1.16E+08	TA	TAA	RP11-222O	5	0.026	INDEL
.	chr7	1.16E+08	TA	T	RP11-222O	5	0.026	INDEL
rs4710	chr7	1.16E+08	G	C	TES	51	0.263	SNP
rs1504961	chr7	1.17E+08	G	GCC	CAV1	21	0.108	INDEL
rs1176221	chr7	1.17E+08	G	A	MET	8	0.041	SNP
rs3577572	chr7	1.17E+08	C	T	MET	16	0.082	SNP
rs3482218	chr7	1.17E+08	CA	C	MET	68	0.351	INDEL
rs3558325	chr7	1.17E+08	G	GT	AC002543.	54	0.278	INDEL
rs1066806	chr7	1.21E+08	G	GCACC	WNT16	62	0.32	INDEL
rs1066806	chr7	1.21E+08	G	GTACC	WNT16	2	0.01	INDEL
rs1021601	chr7	1.22E+08	G	A	RP5-1101C	29	0.149	SNP
rs7866609	chr7	1.22E+08	G	A	RP5-1101C	3	0.015	SNP
rs7157471	chr7	1.23E+08	C	T	RNF133	21	0.108	SNP
rs1382151	chr7	1.24E+08	CG	C	ASB15	4	0.021	INDEL
rs1267220	chr7	1.24E+08	A	G	HYAL4	6	0.031	SNP
rs6977407;	chr7	1.25E+08	A	C	POT1	123	0.634	SNP
rs5960435	chr7	1.26E+08	G	A	AC005276.	45	0.232	SNP
rs1786674	chr7	1.27E+08	C	T	GRM8	5	0.026	SNP
rs712700;3	chr7	1.28E+08	T	C	PAX4	142	0.732	SNP
rs3823994	chr7	1.28E+08	A	T	LRRC4	57	0.294	SNP
rs322825	chr7	1.28E+08	T	C	SND1	121	0.624	SNP
rs7755465	chr7	1.29E+08	T	C	RP11-274B	20	0.103	SNP
rs1927256	chr7	1.29E+08	G	A	FLNC	2	0.01	SNP

.	chr7	1.29E+08	T	G	IRF5	102	0.526	SNP
rs1995089(	chr7	1.29E+08	CACTCTGC/	C	IRF5	89	0.459	INDEL
rs2305324;	chr7	1.29E+08	G	C	TNPO3	83	0.428	SNP
.	chr7	1.29E+08	CTGGA	C	RP11-286H	15	0.077	INDEL
.	chr7	1.32E+08	GTT	GT	AC009518.	36	0.186	INDEL
.	chr7	1.32E+08	GTT	G	AC009518.	5	0.026	INDEL
.	chr7	1.32E+08	GTT	GTTT	AC009518.	22	0.113	INDEL
rs3577893(	chr7	1.32E+08	A	G	AC105443.	30	0.155	SNP
.	chr7	1.35E+08	TAG	T	TMEM140	2	0.01	INDEL
rs2862572:	chr7	1.36E+08	T	C	AC024084.	22	0.113	SNP
rs7997012(	chr7	1.4E+08	G	A	PARP12	13	0.067	SNP
rs3732399‹	chr7	1.41E+08	CT	CTT	ADCK2	20	0.103	INDEL
rs3732399‹	chr7	1.41E+08	CT	C	ADCK2	18	0.093	INDEL
rs1181620(	chr7	1.42E+08	G	T	CLEC5A	12	0.062	SNP
rs5596027:	chr7	1.43E+08	C	T	CLCN1	3	0.015	SNP
.	chr7	1.44E+08	CT	C	OR10AC1	3	0.015	INDEL
rs3433319:	chr7	1.44E+08	GC	G	OR10AC1	146	0.753	INDEL
.	chr7	1.44E+08	GCT	G	TCAF2	4	0.023	INDEL
.	chr7	1.44E+08	A	ATGGAGG(	ARHGEF5	38	0.196	INDEL
rs1462773!	chr7	1.45E+08	C	CT	TPK1	10	0.052	INDEL
rs6764476‹	chr7	1.45E+08	G	T	TPK1	10	0.052	SNP
rs7316274!	chr7	1.45E+08	C	T	TPK1	9	0.046	SNP
rs2371641	chr7	1.45E+08	C	T	TPK1	114	0.588	SNP
rs7735816:	chr7	1.45E+08	C	T	TPK1	2	0.01	SNP
rs6262449:	chr7	1.49E+08	G	T	C7orf33	3	0.015	SNP
rs7227554:	chr7	1.49E+08	TTTTA	TTTTATTTA	RP4-800G7	33	0.17	INDEL
rs7227554:	chr7	1.49E+08	TTTTA	TTTTATTTA	RP4-800G7	18	0.093	INDEL
rs7227554:	chr7	1.49E+08	TTTTA	TTTTATTTA	RP4-800G7	2	0.01	INDEL
rs7227554:	chr7	1.49E+08	TTTTA	T	RP4-800G7	8	0.041	INDEL
rs7227554:	chr7	1.49E+08	TTTTA	TTTTATTTA	RP4-800G7	18	0.093	INDEL
rs7227554:	chr7	1.49E+08	TTTTA	TTTTATTTA	RP4-800G7	2	0.01	INDEL
rs1146234(	chr7	1.5E+08	C	CT	KRBA1	124	0.639	INDEL
.	chr7	1.5E+08	TG	T	SSPO	3	0.015	INDEL
rs1635798	chr7	1.5E+08	T	G	SSPO	14	0.072	SNP
rs7235981:	chr7	1.5E+08	GGATA	G	SSPO	36	0.186	INDEL
rs7119466:	chr7	1.5E+08	G	GC	SSPO	194	1	INDEL
.	chr7	1.5E+08	T	TA	SSPO	130	0.67	INDEL
rs1135384!	chr7	1.5E+08	TC	T	SSPO	39	0.201	INDEL
rs2074697	chr7	1.5E+08	T	G	SSPO	67	0.345	SNP
rs1026197:	chr7	1.5E+08	C	T	SSPO	39	0.201	SNP
.	chr7	1.5E+08	A	G	SSPO	146	0.753	SNP
rs1819756:	chr7	1.5E+08	G	A	ACTR3C	11	0.057	SNP
rs1155805‹	chr7	1.51E+08	G	T	GIMAP2	6	0.031	SNP
rs6952002	chr7	1.51E+08	G	C	GIMAP2	59	0.304	SNP
rs4146514!	chr7	1.51E+08	C	T	AOC1	10	0.052	SNP
.	chr7	1.51E+08	C	CG	AGAP3	190	0.979	INDEL
.	chr7	1.51E+08	C	CG	AGAP3	192	1	INDEL

rs2000335	chr7	1.51E+08	GC	G	AGAP3	10	0.052	INDEL
.	chr7	1.51E+08	TC	T	IQCA1L	115	0.593	INDEL
rs7119871(	chr7	1.51E+08	TCAAAGTG	T	NUB1	6	0.031	INDEL
rs1324084	chr7	1.52E+08	A	G	GALNTL5	117	0.603	SNP
rs1717333	chr7	1.52E+08	T	G	GALNT11	5	0.026	SNP
rs3807218	chr7	1.55E+08	A	G	DPP6	169	0.871	SNP
rs5609148	chr7	1.55E+08	C	T	DPP6	14	0.072	SNP
rs3817522	chr7	1.55E+08	T	C	DPP6	30	0.155	SNP
rs1129300	chr7	1.55E+08	C	T	DPP6	25	0.129	SNP
rs3823617	chr7	1.57E+08	T	C	RNF32	4	0.021	SNP
rs3773927	chr7	1.57E+08	CTAACACC	C	RP5-1142J	21	0.108	INDEL
rs7317150	chr7	1.58E+08	G	C	AC011899.	11	0.057	SNP
rs842695	chr7	1.59E+08	G	A	WDR60	46	0.237	SNP
rs336433	chr8	627101	A	G	ERICH1	116	0.598	SNP
.	chr8	1246858	CCTTTGAG	C	CTD-2281E	3	0.052	INDEL
rs1326574	chr8	2822392	A	G	GS1-57L11	12	0.062	SNP
rs2916747;	chr8	6513770	A	G	ANGPT2	8	0.041	SNP
rs1706343	chr8	6513782	A	G	ANGPT2	10	0.052	SNP
rs2017466	chr8	7296399	T	C	FAM90A20	3	0.016	SNP
rs3177011	chr8	7860665	T	G	SPAG11A	87	0.489	SNP
rs7744182	chr8	8206952	C	T	ENPP7P1	2	0.012	SNP
rs5715331	chr8	8232793	G	A	FAM86B3P	6	0.031	SNP
rs2839301	chr8	9096861	G	A	ERI1	25	0.129	SNP
rs1008912(	chr8	9351551	T	G	RP11-115J	43	0.222	SNP
rs1870397	chr8	9403944	C	A	RP11-115J	17	0.088	SNP
rs1198946(	chr8	9436031	C	T	RP11-115J	41	0.211	SNP
rs1124991	chr8	9454303	A	G	RP11-375N	6	0.031	SNP
rs1439450(	chr8	9454304	A	G	RP11-375N	2	0.01	SNP
rs7015700	chr8	9670197	G	A	TNKS	28	0.144	SNP
rs1425512	chr8	10554002	TG	T	PRSS55	11	0.057	INDEL
225459	chr8	10610141	C	CCTCTCTTC	RP1L1	7	0.036	INDEL
rs4840501;	chr8	10610142	G	C	RP1L1	40	0.25	MIXED
rs4840501;	chr8	10610142	G	GCAGCCCT	RP1L1	6	0.038	MIXED
rs7825250(	chr8	10822125	C	A	PINX1	8	0.041	SNP
rs2736277	chr8	11361384	A	G	TDH	81	0.418	SNP
rs8417	chr8	11809670	C	T	FDFT1	30	0.155	SNP
rs2229247	chr8	11826113	T	C	FDFT1	8	0.041	SNP
rs904011	chr8	11826144	T	C	FDFT1	194	1	SNP
rs1860718	chr8	12177069	C	T	FAM90A2P	2	0.01	SNP
.	chr8	12186440	GCTGT	G	FAM86B1	7	0.036	INDEL
.	chr8	12428721	GCTGT	G	FAM86B2	3	0.016	INDEL
.	chr8	12961715	AAAAG	A	RP11-369K	4	0.021	INDEL
rs658856	chr8	13094784	C	T	DLC1	8	0.041	SNP
rs435815	chr8	16186158	C	T	MSR1	25	0.129	SNP
rs5884867	chr8	17081790	C	CA	MICU3	19	0.098	INDEL
.	chr8	17246516	CAGTTA	C	CNOT7	4	0.021	MIXED
.	chr8	17246516	CAGTTA	TAGTTA	CNOT7	8	0.041	MIXED

rs1505366(	chr8	17279811	TTA	T	VPS37A	5	0.026	INDEL
rs3552285!	chr8	17891808	C	G	FGL1	14	0.072	SNP
rs426372	chr8	17905856	G	A	RP11-156K	6	0.031	SNP
rs5564603(	chr8	18067100	T	TGCACCTG	ASAH1	113	0.582	INDEL
rs1386771:	chr8	18067187	CT	C	ASAH1	4	0.021	INDEL
rs3446655!	chr8	18084285	A	C	ASAH1	14	0.072	SNP
.	chr8	18211192	G	C	NAT1	2	0.01	SNP
rs4986782	chr8	18222607	G	A	NAT1	2	0.01	SNP
.	chr8	18865224	TTATATAT/	TTATATAT/	PSD3&RP1	2	0.011	INDEL
.	chr8	18865224	TTATATAT/	TTATATA	PSD3&RP1	9	0.048	INDEL
.	chr8	18865224	TTATATAT/	TTATATAT/	PSD3&RP1	7	0.038	INDEL
.	chr8	18865224	TTATATAT/	TTATATAT/	PSD3&RP1	2	0.011	INDEL
.	chr8	18865240	ATATATAT/	*	PSD3&RP1	19	0.148	INDEL
.	chr8	18865240	ATATATAT/	A	PSD3&RP1	2	0.016	INDEL
.	chr8	18865246	ATATATAT/	ATT	PSD3&RP1	9	0.056	INDEL
.	chr8	18865246	ATATATAT/	ATTT	PSD3&RP1	8	0.05	INDEL
.	chr8	18865246	ATATATAT/	ATTTT	PSD3&RP1	3	0.019	INDEL
.	chr8	18865246	ATATATAT/	*	PSD3&RP1	4	0.025	INDEL
rs4921665	chr8	19682926	A	G	CSGALNAC	48	0.247	SNP
rs328;1534	chr8	19962213	C	G	LPL	23	0.119	SNP
rs1497024	chr8	20203958	G	C	ATP6V1B2	30	0.155	SNP
rs491797	chr8	20955835	A	G	RP11-369E	193	0.995	SNP
rs7768922!	chr8	22121221	G	A	HR	2	0.01	SNP
rs8005512(	chr8	22196188	T	G	BMP1	3	0.015	SNP
rs1393962(	chr8	22579211	T	G	PDLIM2	3	0.015	SNP
rs3512155:	chr8	22713394	GT	G	PEBP4	51	0.263	INDEL
rs3808536	chr8	23290051	G	A	R3HCC1	103	0.531	SNP
.	chr8	23425049	A	AGTATT	LOXL2	13	0.067	INDEL
.	chr8	23844813	TCCTCCTC/	T	STC1	20	0.103	INDEL
.	chr8	23844816	TCCTCA	*	STC1	13	0.067	INDEL
.	chr8	23844816	TCCTCA	T	STC1	39	0.201	INDEL
rs3758098	chr8	26381867	C	A	SDAD1P1	25	0.129	SNP
rs327222	chr8	26624255	T	C	DPYSL2	193	0.995	SNP
rs1391232:	chr8	26624306	C	T	DPYSL2	5	0.026	SNP
rs4150795:	chr8	27500988	A	G	EPHX2	17	0.088	SNP
rs2234913	chr8	27516337	G	A	EPHX2	3	0.015	SNP
rs751141;1	chr8	27516348	G	A	EPHX2	28	0.144	SNP
rs7155386	chr8	27536817	A	AGTC	EPHX2	13	0.067	INDEL
rs1343945!	chr8	27536849	C	T	EPHX2	18	0.093	SNP
rs1011362:	chr8	27833040	A	C	PBK	13	0.067	SNP
rs6993934	chr8	28415871	T	C	RP11-181B	19	0.098	SNP
rs6994291	chr8	28631551	G	A	EXTL3	69	0.356	SNP
.	chr8	29908532	A	G	RP11-489E	3	0.015	SNP
.	chr8	30070061	T	A	SARAF	2	0.01	MIXED
rs3574439	chr8	30732715	TG	T	UBXN8	53	0.273	INDEL
.	chr8	30843317	TTC	T	TEX15	3	0.015	INDEL
rs1198970!	chr8	33593612	G	A	DUSP26	2	0.01	SNP

rs7495935!	chr8	34038342	CT	CTT	RP11-317N	20	0.103	INDEL
rs7495935!	chr8	34038342	CT	C	RP11-317N	29	0.149	INDEL
.	chr8	37698479	CGGCAG	C	ZNF703	2	0.01	INDEL
rs8031567!	chr8	37784262	C	G	ADGRA2	4	0.021	SNP
rs1507219(	chr8	38512464	C	G	C8orf86	2	0.01	SNP
rs7121669:	chr8	38901439	C	CG	PLEKHA2	184	0.948	INDEL
rs6251050(	chr8	39505340	C	T	ADAM3A	29	0.177	SNP
rs4503083	chr8	40015416	T	A	IDO2	50	0.258	SNP
rs1175162(	chr8	41694031	G	A	ANK1	6	0.031	SNP
rs6175367!	chr8	41714174	G	T	ANK1	3	0.015	SNP
rs2020367ʻ	chr8	43103684	C	T	POMK	2	0.01	SNP
.	chr8	50163481	CTT	CT	SNTG1	23	0.119	INDEL
.	chr8	50163481	CTT	CTTT	SNTG1	12	0.062	INDEL
.	chr8	50163481	CTT	C	SNTG1	5	0.026	INDEL
rs1177523!	chr8	51372000	A	T	PXDNL	2	0.01	SNP
rs1181644(	chr8	52150972	T	C	RP11-26M!	3	0.015	SNP
.	chr8	55746039	GTGCGGTG	G	TMEM68	2	0.01	INDEL
rs4478561	chr8	58271548	C	A	RP11-716D	187	0.964	SNP
rs7571298(	chr8	58637233	C	T	NSMAF	13	0.067	SNP
rs7756885!	chr8	59612890	G	C	RP11-379I1	13	0.067	SNP
rs7464181;	chr8	60266015	T	C	CA8	75	0.387	SNP
rs6983123	chr8	61465806	T	C	CLVS1	24	0.124	SNP
rs7747619!	chr8	65172513	C	T	LINC00251	17	0.088	SNP
.	chr8	65707072	CTG	C	MTFR1	3	0.015	INDEL
rs3771823!	chr8	66677787	CG	C	C8orf44	2	0.01	INDEL
rs2006530!	chr8	68331068	TCCGGCTC	T	C8orf34	2	0.01	INDEL
rs1325709!	chr8	69944575	G	A	SDCBPP2	58	0.299	SNP
rs6652771(	chr8	72020024	C	T	TRPA1	41	0.211	SNP
.	chr8	72881595	TCTTAAAA(	T	RP11-1145	4	0.021	INDEL
rs1141913:	chr8	73669055	A	AG	STAU2	189	0.974	INDEL
rs1441743!	chr8	73794116	CT	C	UBE2W	8	0.041	INDEL
rs1095773ʻ	chr8	74625343	T	C	MIR2052H(	179	0.923	SNP
.	chr8	78686431	GTTTATTTA	GTTTATTTA	ZC2HC1A	161	0.83	INDEL
.	chr8	78686431	GTTTATTTA	GTTTATTTA	ZC2HC1A	23	0.119	INDEL
rs1426136:	chr8	79766258	AGGCATGT	A	HEY1	21	0.108	INDEL
.	chr8	80485781	CA	C	ZBTB10	3	0.015	INDEL
rs7327547(	chr8	81525965	G	A	RP11-257P	6	0.031	SNP
.	chr8	82160390	CAGTAAGA	C	RP11-99H2	3	0.015	INDEL
rs1254137!	chr8	82863112	T	C	RP11-731N	35	0.18	SNP
rs6653217:	chr8	84164024	CT	CTTTTT	RP11-120I2	3	0.034	INDEL
rs6653217:	chr8	84164024	CT	C	RP11-120I2	3	0.034	INDEL
rs6653217:	chr8	84164024	CT	CTTTT	RP11-120I2	2	0.023	INDEL
rs6653217:	chr8	84164024	CT	CTTTTTTT	RP11-120I2	4	0.045	INDEL
rs1071225!	chr8	84887592	TC	T	RALYL	59	0.304	INDEL
rs1071225!	chr8	84887592	TC	TCC	RALYL	2	0.01	INDEL
.	chr8	85110101	CT	C	LRRCC1	3	0.015	INDEL
.	chr8	85110101	CT	CTT	LRRCC1	4	0.021	INDEL

rs6757381:	chr8	85219234	A	T	C8orf59	145	0.747	SNP
rs7535321!	chr8	85268490	A	G	CA13	2	0.01	SNP
rs1496532	chr8	85341753	C	T	CA1	120	0.619	SNP
rs703;2547	chr8	85477174	T	C	CA2	121	0.624	SNP
rs5699377!	chr8	86214405	TCCGACAT(	T	SLC7A13	33	0.17	INDEL
rs7814198	chr8	86229618	G	A	SLC7A13	60	0.309	SNP
.	chr8	86524874	TAGACGGA	T	CPNE3	2	0.01	INDEL
rs1458116	chr8	86760834	A	G	RP11-386D	96	0.495	SNP
rs1690227:	chr8	90006279	T	A	DECR1	9	0.046	SNP
rs1010989!	chr8	90593972	C	T	LINC01030	18	0.093	SNP
.	chr8	91133094	GT	GTT	LRRC69	31	0.16	INDEL
.	chr8	91133094	GT	G	LRRC69	7	0.036	INDEL
rs7266605(	chr8	91200794	T	A	LRRC69	7	0.036	SNP
.	chr8	93755751	CT	C	TMEM67	20	0.106	INDEL
.	chr8	93755751	CT	CTTT	TMEM67	2	0.011	INDEL
.	chr8	93755751	CT	CTT	TMEM67	6	0.032	INDEL
rs1494694(	chr8	93762947	CAG	C	TMEM67	19	0.098	INDEL
rs1132692!	chr8	94868680	C	CT	INTS8	42	0.216	INDEL
403028	chr8	97775937	C	CGGCGGG(	LAPTM4B	36	0.186	INDEL
rs7818775	chr8	97815303	G	A	LAPTM4B	99	0.51	SNP
rs1783116(	chr8	98033638	G	A	MATN2	8	0.041	SNP
rs2255464	chr8	98033750	A	C	MATN2	85	0.438	SNP
rs3735887	chr8	98193384	C	T	NIPAL2	90	0.464	SNP
rs7460625;	chr8	99121478	T	G	VPS13B	161	0.83	SNP
.	chr8	99832341	AT	ATTTT	VPS13B	32	0.167	INDEL
.	chr8	99832341	AT	A	VPS13B	30	0.156	INDEL
.	chr8	99832341	AT	ATT	VPS13B	25	0.13	INDEL
.	chr8	99832341	AT	ATTT	VPS13B	3	0.016	INDEL
.	chr8	99832341	AT	ATTTTT	VPS13B	3	0.016	INDEL
.	chr8	99832341	AT	ATTTTTTTT	VPS13B	2	0.01	INDEL
rs1130474	chr8	99892049	T	C	COX6C	31	0.16	SNP
.	chr8	1.01E+08	GCTAAAAA	G	PABPC1	46	0.237	INDEL
.	chr8	1.01E+08	G	GGATGAG(	PABPC1	12	0.062	INDEL
.	chr8	1.01E+08	G	GGATGAG(	PABPC1	2	0.01	INDEL
.	chr8	1.01E+08	G	GGATGAG(	PABPC1	5	0.026	INDEL
.	chr8	1.01E+08	G	GGATGAG(	PABPC1	5	0.026	INDEL
rs1129668!	chr8	1.01E+08	CT	C	PABPC1	23	0.119	INDEL
.	chr8	1.01E+08	T	TC	PABPC1	32	0.165	INDEL
rs7268144:	chr8	1.01E+08	T	C	PABPC1	33	0.17	SNP
.	chr8	1.01E+08	C	CTTTACAT(	PABPC1	3	0.015	INDEL
rs4147314	chr8	1.01E+08	C	T	YWHAZ	6	0.031	SNP
rs1325340:	chr8	1.01E+08	G	C	FLJ42969	36	0.186	SNP
rs5893603;	chr8	1.02E+08	C	CG	RRM2B	11	0.057	INDEL
776368	chr8	1.03E+08	AGCCCCTG	*	ODF1	45	0.232	INDEL
776369	chr8	1.03E+08	AACCCGTG	*	ODF1	45	0.232	INDEL
rs7266727(	chr8	1.03E+08	G	A	AZIN1-AS1	26	0.134	SNP
.	chr8	1.03E+08	ATT	*	MTCO1P4	87	0.448	INDEL

.	chr8	1.03E+08	ATT	A	MTCO1P4	58	0.299	INDEL
rs827528	chr8	1.03E+08	A	G	FZD6	57	0.294	SNP
rs6995597	chr8	1.03E+08	A	G	CTHRC1	7	0.036	SNP
.	chr8	1.07E+08	TA	T	OXR1	15	0.077	INDEL
rs1457398	chr8	1.07E+08	G	A	OXR1	2	0.01	SNP
rs1736831	chr8	1.09E+08	G	C	PKHD1L1	13	0.067	SNP
rs7130595	chr8	1.1E+08	CTT	CT	PKHD1L1	20	0.103	INDEL
rs7130595	chr8	1.1E+08	CTT	C	PKHD1L1	35	0.18	INDEL
rs7130595	chr8	1.1E+08	CTT	CTTT	PKHD1L1	12	0.062	INDEL
rs1010162	chr8	1.23E+08	G	T	TBC1D31	28	0.144	SNP
.	chr8	1.24E+08	CTTA	C	TMEM65	2	0.01	INDEL
rs1154267	chr8	1.25E+08	G	A	TATDN1	10	0.052	SNP
rs6470252;	chr8	1.25E+08	C	T	NDUFB9	140	0.722	SNP
.	chr8	1.26E+08	CT	C	RP11-136O	3	0.015	INDEL
.	chr8	1.26E+08	CT	CTT	RP11-136O	116	0.598	INDEL
.	chr8	1.26E+08	CT	CTTT	RP11-136O	5	0.026	INDEL
rs1690405	chr8	1.29E+08	T	C	CCDC26	12	0.062	SNP
rs1009235	chr8	1.3E+08	A	C	MTRF1LP2	37	0.191	SNP
rs3721708	chr8	1.31E+08	G	T	CTD-2501M	8	0.041	SNP
rs7334545	chr8	1.31E+08	A	G	CTD-2501M	83	0.428	SNP
rs3739261	chr8	1.33E+08	T	C	WISP1	67	0.345	SNP
.	chr8	1.35E+08	A	C	ZFAT	3	0.015	SNP
.	chr8	1.35E+08	CTG	C	ZFAT	2	0.01	INDEL
rs7339813	chr8	1.37E+08	A	G	RP11-30J2	2	0.01	SNP
rs2292781	chr8	1.41E+08	G	A	AGO2	77	0.397	SNP
rs307772	chr8	1.41E+08	T	C	DENND3	134	0.691	SNP
.	chr8	1.41E+08	GTCTGTGA	G	RP11-10J2	2	0.01	INDEL
.	chr8	1.41E+08	C	T	MROH5	39	0.201	SNP
.	chr8	1.43E+08	GCCT	G	JRK	18	0.093	INDEL
.	chr8	1.43E+08	C	*	JRK	18	0.093	MIXED
rs2016282	chr8	1.43E+08	T	TGGAGGA	JRK	115	0.593	MIXED
rs2016282	chr8	1.43E+08	T	*	JRK	18	0.093	MIXED
rs1383779	chr8	1.43E+08	G	A	PSCA	3	0.015	SNP
rs6175729	chr8	1.43E+08	A	G	CYP11B2	2	0.01	SNP
rs4538;362	chr8	1.43E+08	G	T	CYP11B2	113	0.582	SNP
rs4539;362	chr8	1.43E+08	T	C	CYP11B2	85	0.438	SNP
rs6558339	chr8	1.43E+08	T	C	RP13-582O	171	0.881	SNP
rs1142416	chr8	1.43E+08	A	AG	TOP1MT	154	0.794	INDEL
.	chr8	1.43E+08	A	AG	ZC3H3	9	0.046	INDEL
rs2272754	chr8	1.43E+08	G	T	ZC3H3	29	0.149	SNP
rs6993938;	chr8	1.44E+08	A	G	PLEC	69	0.356	SNP
rs1113625	chr8	1.44E+08	G	T	SHARPIN	182	0.938	SNP
rs3417306	chr8	1.44E+08	G	A	SHARPIN	18	0.093	SNP
.	chr8	1.44E+08	CT	CTT	HSF1	6	0.031	INDEL
.	chr8	1.44E+08	CT	C	HSF1	23	0.119	INDEL
.	chr8	1.44E+08	CGT	C	KIFC2	3	0.015	INDEL
rs3496620	chr8	1.45E+08	GC	G	ZNF7	3	0.015	INDEL

rs7922001	chr9	116800	C	G	FOXD4	50	0.258	SNP
.	chr9	117366	C	CCG	FOXD4	28	0.144	INDEL
.	chr9	117370	GCC	G	FOXD4	28	0.144	INDEL
rs7911952	chr9	454608	T	C	RP11-165F	6	0.031	SNP
.	chr9	2045826	GACACACA	GACACACA	RP11-264I1	55	0.284	INDEL
.	chr9	2045826	GACACACA	GACACACA	RP11-264I1	11	0.057	INDEL
.	chr9	2045826	GACACACA	GACACACA	RP11-264I1	14	0.072	INDEL
.	chr9	2045826	GACACACA	GACACACA	RP11-264I1	11	0.057	INDEL
.	chr9	2045826	GACACACA	G	RP11-264I1	4	0.021	INDEL
.	chr9	2045826	GACACACA	GACACACA	RP11-264I1	8	0.041	INDEL
.	chr9	3453710	CAA	CA	AL365202.	64	0.33	INDEL
.	chr9	3453710	CAA	CAAA	AL365202.	6	0.031	INDEL
.	chr9	3453710	CAA	C	AL365202.	9	0.046	INDEL
rs1177852	chr9	3671473	G	C	RFX3-AS1	4	0.021	SNP
.	chr9	4945351	CTTTTTTTT	CTTTTTTTT	HNRNPA1P	19	0.158	INDEL
.	chr9	4945351	CTTTTTTTT	CT	HNRNPA1P	8	0.067	INDEL
.	chr9	4945351	CTTTTTTTT	CTTTTTTTT	HNRNPA1P	4	0.033	INDEL
.	chr9	4945351	CTTTTTTTT	CTTTTTTTT	HNRNPA1P	3	0.025	INDEL
rs2020291	chr9	5164066	C	T	INSL6	2	0.01	SNP
.	chr9	5832788	G	T	ERMP1	2	0.01	SNP
rs2022446	chr9	6329010	TG	T	TPD52L3	2	0.01	INDEL
rs3818899	chr9	6941816	A	G	KDM4C	57	0.294	SNP
rs1075645	chr9	13150532	T	C	MPDZ	64	0.33	SNP
rs1387576	chr9	15214193	A	T	TTC39B	2	0.01	SNP
rs1858109	chr9	17298293	C	T	CNTLN	3	0.015	SNP
rs3539902	chr9	17457514	TA	T	CNTLN	183	0.943	INDEL
rs3552518	chr9	18826263	GT	G	ADAMTSL1	149	0.768	INDEL
rs1011991	chr9	21207038	A	T	IFNA10	40	0.206	SNP
rs2004005	chr9	21228003	A	AT	IFNA17	2	0.01	INDEL
rs4129819	chr9	21304914	G	A	IFNA5	7	0.036	SNP
rs2039381	chr9	21481484	G	A	IFNE	3	0.015	SNP
rs7023954;	chr9	21816759	G	A	MTAP	90	0.464	SNP
rs1096516	chr9	21854741	C	T	MTAP	9	0.046	SNP
.	chr9	21995503	GGCGTAC	G	RP11-149I2	2	0.01	INDEL
rs7344021	chr9	27104610	A	T	RP11-56F1	8	0.041	SNP
rs1440601	chr9	27524366	G	GTGTT	IFNK	8	0.041	INDEL
rs6175294	chr9	32481341	C	T	DDX58	2	0.01	SNP
rs1065765;	chr9	33135240	G	A	B4GALT1	11	0.057	SNP
.	chr9	34372875	G	C	KIAA1161	41	0.211	SNP
.	chr9	34376791	A	C	KIAA1161	2	0.01	SNP
rs4127486	chr9	34648091	G	A	GALT	12	0.062	SNP
rs1512572	chr9	34893048	GC	G	FAM205C	71	0.366	INDEL
rs1480676	chr9	34990342	TCA	T	DNAJB5	2	0.01	INDEL
rs3068510	chr9	35562396	A	ATAGT	FAM166B	7	0.036	INDEL
rs1381637	chr9	35682767	A	AG	TPM2	37	0.191	INDEL
rs1951433	chr9	35757196	T	C	RP11-112J	122	0.629	SNP
rs7866903	chr9	35790154	A	G	RP11-112J	29	0.149	SNP

rs2381409	chr9	35829393	C	T	TMEM8B	115	0.593	SNP
rs1097268	chr9	36002932	T	C	OR13C7	46	0.237	SNP
rs1081431	chr9	36003309	A	T	OR13C7	46	0.237	SNP
rs1116060	chr9	36003431	CTT	C	OR13C7	46	0.237	INDEL
rs7846941	chr9	38394566	G	A	ALDH1B1	39	0.201	SNP
rs2013260	chr9	39028169	TTATG	T	VN2R3P	167	0.861	INDEL
402551	chr9	41960804	CA	C	CNTNAP3B	2	0.01	INDEL
.	chr9	41970123	GC	G	CNTNAP3B	43	0.224	INDEL
.	chr9	64377588	AAAAG	A	ANKRD20A	5	0.026	INDEL
.	chr9	65283162	AG	A	FOXD4L5	28	0.255	INDEL
.	chr9	65283489	GGA	G	FOXD4L5	2	0.01	INDEL
.	chr9	65733001	GTCAA	G	CBWD5	2	0.01	INDEL
.	chr9	66141076	TTAAG	T	ANKRD20A	11	0.074	INDEL
.	chr9	67867344	AAAAG	A	ANKRD20A	10	0.052	INDEL
rs2004980	chr9	68285995	G	GA	CBWD3	44	0.275	INDEL
.	chr9	68322903	CTCAGTCC	C	RP11-561O	6	0.036	MIXED
.	chr9	68322903	CTCAGTCC	GTCAGTCC	RP11-561O	12	0.072	MIXED
.	chr9	68322918	GCGAGGG	*	RP11-561O	6	0.037	INDEL
.	chr9	72149619	C	T	GDA	2	0.01	SNP
rs3752955	chr9	75069774	A	C	NMRK1	100	0.515	SNP
.	chr9	76175201	AGAATG	AGAATGGA	PCSK5	6	0.031	INDEL
.	chr9	76175222	AAATGGAA	AAATGGAA	PCSK5	9	0.046	INDEL
.	chr9	76175222	AAATGGAA	*	PCSK5	3	0.015	INDEL
.	chr9	76175232	GAATGA	*	PCSK5	11	0.057	INDEL
.	chr9	76175232	GAATGA	G	PCSK5	40	0.206	INDEL
.	chr9	76175235	T	*	PCSK5	50	0.258	MIXED
.	chr9	76175235	T	TG	PCSK5	20	0.103	MIXED
.	chr9	76175237	AAATGGAA	*	PCSK5	63	0.325	MIXED
.	chr9	76175237	AAATGGAA	GAATGGAA	PCSK5	75	0.387	MIXED
.	chr9	76175237	AAATGGAA	AATGGAAT	PCSK5	15	0.077	MIXED
.	chr9	76175237	AAATGGAA	AATGGAAT	PCSK5	7	0.036	MIXED
.	chr9	76175255	T	TC	PCSK5	5	0.026	INDEL
rs7724976	chr9	76175256	G	C	PCSK5	6	0.031	MIXED
rs7724976	chr9	76175256	G	GAATCGAA	PCSK5	4	0.021	MIXED
rs1011832	chr9	76175271	G	C	PCSK5	39	0.201	MIXED
rs3750908	chr9	76175286	C	A	PCSK5	2	0.01	SNP
rs1011613	chr9	76175300	T	C	PCSK5	4	0.021	SNP
rs6255662	chr9	76175301	A	C	PCSK5	18	0.093	SNP
rs7615799	chr9	76613379	T	C	PRUNE2	2	0.01	SNP
rs1234809	chr9	77337552	A	T	VPS13A	28	0.144	SNP
rs1778778	chr9	78253943	G	A	CEP78	10	0.052	SNP
rs3739474	chr9	78304840	T	G	PSAT1	126	0.649	SNP
rs1467060	chr9	83830941	C	T	RP11-575L	7	0.036	SNP
rs1011581	chr9	83859676	T	C	RP11-575L	48	0.247	SNP
.	chr9	85935344	CAA	CA	RP11-213G	12	0.062	INDEL
.	chr9	85935344	CAA	CAAA	RP11-213G	16	0.082	INDEL
.	chr9	85935344	CAA	C	RP11-213G	4	0.021	INDEL

.	chr9	85956346	CTT	CTTT	NAA35	6	0.031	INDEL
.	chr9	85956346	CTT	CT	NAA35	7	0.036	INDEL
rs3620742	chr9	87605005	G	A	DAPK1	12	0.062	SNP
rs5580026	chr9	87638051	C	T	DAPK1	8	0.041	SNP
rs2378757	chr9	87728865	A	C	CTSL	129	0.665	SNP
rs1384968	chr9	89315170	G	C	CKS2	2	0.01	SNP
rs1152646	chr9	89388764	C	T	SEMA4D	64	0.33	SNP
rs9802603	chr9	89803214	G	T	RP5-1050E	73	0.376	SNP
rs3575816	chr9	90844027	G	A	SYK	45	0.232	SNP
rs2290888	chr9	90877691	G	C	SYK	28	0.144	SNP
rs2290887	chr9	90877727	G	A	SYK	28	0.144	SNP
rs2306041	chr9	90878893	C	T	SYK	28	0.144	SNP
rs2306040	chr9	90878917	T	C	SYK	15	0.077	SNP
rs1099206	chr9	91724406	G	A	ROR2	81	0.418	SNP
rs1173605	chr9	92877889	C	T	ZNF484	4	0.021	SNP
rs1170492	chr9	92917574	T	C	ALOX15P2	4	0.021	SNP
rs1759349	chr9	93110603	C	T	CARD19	23	0.119	SNP
rs690528	chr9	95876006	A	G	ERCC6L2	60	0.309	SNP
rs7026211	chr9	96948911	T	C	MFSD14C	4	0.021	SNP
rs1155464	chr9	97633890	G	A	NCBP1	3	0.015	SNP
rs2021845	chr9	97930260	C	A	HEMGN	2	0.01	SNP
rs4237190	chr9	98081135	A	G	NANS	92	0.474	SNP
.	chr9	98854332	C	CTAGGGAC	RP11-92C4	2	0.01	INDEL
rs3749628	chr9	1.02E+08	GAGCTAGA	G	GRIN3A	60	0.309	INDEL
rs1099043	chr9	1.03E+08	G	A	CYLC2	55	0.284	SNP
rs1082034	chr9	1.03E+08	G	A	CYLC2	128	0.66	SNP
rs1131421	chr9	1.05E+08	GT	G	OR13C5	42	0.216	INDEL
rs3761078	chr9	1.05E+08	AGC	A	OR13C5	2	0.01	INDEL
rs1431981	chr9	1.05E+08	TGTTA	T	OR13C2	41	0.211	INDEL
rs1437607	chr9	1.05E+08	AGC	A	OR13C2	41	0.211	INDEL
rs1436306	chr9	1.05E+08	G	A	NIPSNAP3E	2	0.01	SNP
.	chr9	1.07E+08	CGGCCA	CCCA	ZNF462	2	0.01	INDEL
rs3056494	chr9	1.07E+08	CA	C	RAD23B	125	0.644	INDEL
rs3056494	chr9	1.07E+08	CA	CAA	RAD23B	55	0.284	INDEL
rs1772885	chr9	1.09E+08	A	G	ACTL7B	42	0.216	SNP
rs1408029	chr9	1.09E+08	G	A	PTPN3	4	0.021	SNP
.	chr9	1.1E+08	GTTTTTTTT	G	YBX1P6	43	0.272	INDEL
.	chr9	1.1E+08	GTTTTTTTT	GTTTTTTTT	YBX1P6	15	0.095	INDEL
.	chr9	1.1E+08	GTTTTTTTT	GTTTTTTTT	YBX1P6	6	0.038	INDEL
.	chr9	1.1E+08	GTTTTTTTT	GTTTTTTTT	YBX1P6	2	0.013	INDEL
rs2010811	chr9	1.1E+08	AT	A	C9orf152	2	0.01	INDEL
rs2002718	chr9	1.12E+08	T	TA	C9orf84	2	0.01	INDEL
.	chr9	1.13E+08	TGGTGTAC	T	RP11-276E	5	0.026	INDEL
rs1098158	chr9	1.13E+08	G	A	ZNF883	12	0.062	SNP
rs1776561	chr9	1.13E+08	G	A	HDHD3	6	0.031	SNP
rs1383138	chr9	1.13E+08	C	T	ALAD	2	0.01	SNP
rs1139488	chr9	1.13E+08	A	G	ALAD	89	0.459	SNP

rs7873844	chr9	1.13E+08	G	A	C9orf43	22	0.113	SNP
rs3810936	chr9	1.15E+08	T	C	TNFSF15	136	0.701	SNP
rs7365541	chr9	1.17E+08	G	A	RP11-67K1	31	0.16	SNP
.	chr9	1.2E+08	CT	C	CDK5RAP2	7	0.036	INDEL
.	chr9	1.2E+08	CT	CTT	CDK5RAP2	5	0.026	INDEL
rs1927626	chr9	1.21E+08	G	A	GSN	5	0.026	SNP
rs4557933	chr9	1.22E+08	G	T	OR1J1	7	0.036	SNP
rs1459118	chr9	1.23E+08	A	AT	OR1J2	19	0.098	INDEL
rs1476860	chr9	1.23E+08	G	A	OR1B1	79	0.407	SNP
rs7812604	chr9	1.23E+08	C	CA	OR1B1	78	0.402	INDEL
rs2596698	chr9	1.23E+08	T	C	RC3H2	194	1	SNP
rs3214358	chr9	1.23E+08	CA	C	RABGAP1	5	0.026	INDEL
rs3214358	chr9	1.23E+08	CA	CAA	RABGAP1	16	0.082	INDEL
.	chr9	1.23E+08	A	AG	CRB2	3	0.015	INDEL
.	chr9	1.25E+08	CACCA	C	OLFML2A	2	0.01	INDEL
rs1098646	chr9	1.25E+08	A	T	ARPC5L	74	0.381	SNP
rs2070113	chr9	1.25E+08	G	A	MAPKAP1	45	0.232	SNP
rs2286889	chr9	1.26E+08	A	G	MVB12B	24	0.124	SNP
rs6174512	chr9	1.28E+08	G	C	PTRH1	9	0.046	SNP
rs2233513;	chr9	1.28E+08	C	T	SH2D3C	3	0.015	SNP
rs1437187	chr9	1.28E+08	G	A	CDK9	5	0.026	SNP
rs2229917;	chr9	1.28E+08	G	A	DNM1	19	0.098	SNP
rs1412567	chr9	1.28E+08	G	A	DNM1	4	0.021	SNP
rs1076056	chr9	1.29E+08	A	G	SPTAN1	194	1	SNP
rs1098810	chr9	1.29E+08	A	G	ZDHHC12	12	0.062	MIXED
rs2011610	chr9	1.29E+08	T	C	KYAT1	2	0.01	SNP
.	chr9	1.29E+08	ATT	AT	KYAT1	11	0.057	INDEL
.	chr9	1.29E+08	ATT	ATTT	KYAT1	5	0.026	INDEL
rs2273866	chr9	1.29E+08	A	G	PHYHD1	90	0.464	SNP
167006	chr9	1.29E+08	A	AT	DOLK	4	0.021	INDEL
rs2254230	chr9	1.29E+08	T	G	PTPA	57	0.294	SNP
rs1433785	chr9	1.3E+08	C	A	HMCN2	7	0.036	SNP
.	chr9	1.3E+08	G	GA	HMCN2	14	0.072	INDEL
rs1090107	chr9	1.3E+08	C	T	ASS1	29	0.149	SNP
rs1390311	chr9	1.3E+08	G	A	ASS1	3	0.015	SNP
rs2227985	chr9	1.31E+08	A	G	ABL1	22	0.113	SNP
.	chr9	1.31E+08	CTT	CTTT	NUP214	5	0.029	INDEL
.	chr9	1.31E+08	CTT	CT	NUP214	12	0.071	INDEL
.	chr9	1.31E+08	CTT	C	NUP214	2	0.012	INDEL
.	chr9	1.31E+08	AC	A	RP11-544A	2	0.01	INDEL
rs6175147	chr9	1.31E+08	A	G	NUP214	3	0.015	SNP
rs2866531	chr9	1.31E+08	A	G	NUP214	4	0.021	SNP
rs3887873;	chr9	1.32E+08	C	T	POMT1	35	0.18	SNP
rs3824574	chr9	1.32E+08	C	T	NTNG2	60	0.309	SNP
rs3739930	chr9	1.32E+08	C	T	NTNG2	21	0.108	SNP
rs2149171	chr9	1.32E+08	C	T	NTNG2	107	0.552	SNP
rs6059582	chr9	1.32E+08	TGAGAATC	T	CFAP77&RI	194	1	INDEL

rs1062356	chr9	1.33E+08	G	C	RALGDS	169	0.871	SNP
rs3589852	chr9	1.33E+08	G	T	GBGT1	9	0.046	SNP
.	chr9	1.33E+08	CA	C	OBP2B	2	0.01	INDEL
rs5639230	chr9	1.33E+08	CG	C	ABO	14	0.072	INDEL
rs5572730	chr9	1.33E+08	C	T	ABO	7	0.036	SNP
rs8176719	chr9	1.33E+08	T	TC	ABO	86	0.443	INDEL
769359	chr9	1.33E+08	G	A	ADAMTS13	125	0.644	SNP
rs3452325	chr9	1.34E+08	A	G	WDR5	8	0.041	SNP
rs6175147	chr9	1.34E+08	G	A	RXRA	5	0.026	SNP
rs6104355	chr9	1.35E+08	G	GAA	OLFM1	187	0.964	INDEL
.	chr9	1.35E+08	G	GCA	OLFM1	3	0.015	INDEL
rs7875358	chr9	1.35E+08	T	A	SOCS5P2	147	0.758	SNP
rs1175422	chr9	1.36E+08	G	C	MRPS2	3	0.015	SNP
rs1128515	chr9	1.36E+08	G	GC	OBP2A	43	0.222	INDEL
rs3421744	chr9	1.36E+08	CA	C	GLT6D1	40	0.206	INDEL
rs3622168	chr9	1.36E+08	CGCCCT	C	KCNT1	53	0.273	INDEL
rs3622168	chr9	1.36E+08	CGCCCT	CGCCCTGC	KCNT1	2	0.01	INDEL
rs1457291	chr9	1.36E+08	TC	T	GPSM1	30	0.155	INDEL
rs5987390	chr9	1.36E+08	C	A	GPSM1	34	0.175	SNP
rs1419923	chr9	1.36E+08	C	G	CARD9	4	0.021	SNP
rs3398266	chr9	1.36E+08	C	A	INPP5E	58	0.299	SNP
rs1087018	chr9	1.36E+08	G	A	INPP5E	19	0.098	SNP
rs1078154	chr9	1.36E+08	A	G	INPP5E	85	0.438	SNP
rs9886752	chr9	1.37E+08	G	A	LCN10	26	0.134	SNP
rs2297723	chr9	1.37E+08	A	C	LCN15	147	0.758	SNP
rs8666	chr9	1.37E+08	C	T	PHPT1	11	0.057	SNP
rs2019743	chr9	1.37E+08	G	GC	C8G	10	0.052	INDEL
rs1078152	chr9	1.37E+08	A	G	LCN12	186	0.959	SNP
rs1078152	chr9	1.37E+08	A	*	LCN12	2	0.01	SNP
rs1078153	chr9	1.37E+08	G	A	NPDC1	194	1	SNP
rs4880091	chr9	1.37E+08	T	C	MAN1B1	140	0.722	SNP
rs9775264	chr9	1.37E+08	G	A	FAM166A	194	1	SNP
.	chr9	1.38E+08	C	CT	PNPLA7	2	0.01	INDEL
rs2003509	chr9	1.38E+08	TGGCCACC	T	ARRDC1	2	0.01	INDEL
rs4131462	chr10	1019517	T	A	IDI2	3	0.015	SNP
rs1044261	chr10	1019770	C	T	IDI2	11	0.057	SNP
rs1337703	chr10	1022699	C	T	IDI2	13	0.067	SNP
.	chr10	1048262	AC	A	IDI1	2	0.01	INDEL
rs1079471	chr10	1096268	T	C	WDR37	194	1	SNP
.	chr10	1292983	AGAGAGA	A	ADARB2&R	2	0.011	INDEL
rs1052333	chr10	3129929	C	T	PFKP	64	0.33	SNP
rs3577934	chr10	3138096	C	T	PITRM1	3	0.015	SNP
rs11414	chr10	3138124	T	C	PITRM1	110	0.567	SNP
rs2279217	chr10	3138811	A	G	PITRM1	125	0.644	SNP
rs3814596	chr10	3165440	A	G	PITRM1	31	0.16	SNP
rs1235903	chr10	3166365	A	G	PITRM1	129	0.665	SNP
rs1484728	chr10	3166375	T	TGCACGCT	PITRM1	2	0.01	INDEL

rs1224027(	chr10	4847211	C	T	AKR1E2	25	0.129	SNP
rs1395882(	chr10	4968883	G	A	AKR1C1	4	0.021	SNP
rs6724580	chr10	5007406	C	G	AKR1C2	36	0.186	SNP
rs4881396	chr10	5077965	G	T	AKR1C3	164	0.845	SNP
rs4567355	chr10	5453255	T	C	NET1	194	1	SNP
rs7138849(	chr10	5861897	CTTTTTTTT	CTTTTTTTT	RPL12P28	16	0.082	INDEL
rs7138849(	chr10	5861897	CTTTTTTTT	C	RPL12P28	73	0.376	INDEL
rs7138849(	chr10	5861897	CTTTTTTTT	CTTTTTTTT	RPL12P28	8	0.041	INDEL
.	chr10	5888024	TCA	T	ANKRD16	2	0.01	INDEL
rs8177786	chr10	5958223	TGGAAACA	T	IL15RA	6	0.031	INDEL
rs1125874	chr10	6430929	G	T	PRKCQ	37	0.191	SNP
rs1125894	chr10	6456761	G	A	PRKCQ	37	0.191	SNP
rs1730118	chr10	6479019	C	T	PRKCQ	3	0.015	SNP
.	chr10	11755323	CAAAA	CAAA	ECHDC3	68	0.351	INDEL
.	chr10	11755323	CAAAA	CAA	ECHDC3	69	0.356	INDEL
.	chr10	11755323	CAAAA	CA	ECHDC3	23	0.119	INDEL
.	chr10	11755323	CAAAA	C	ECHDC3	3	0.015	INDEL
rs7899215	chr10	11755437	T	C	ECHDC3	194	1	SNP
rs3598648	chr10	11755501	G	A	ECHDC3	14	0.072	SNP
rs1454536	chr10	11755518	C	T	ECHDC3	3	0.015	SNP
rs1090601	chr10	11763358	G	A	ECHDC3	118	0.608	SNP
rs1177066(	chr10	11763400	C	T	ECHDC3	5	0.026	SNP
rs6264168	chr10	13322914	C	G	SEPHS1	12	0.062	SNP
rs6264168	chr10	13336273	G	C	SEPHS1	2	0.01	SNP
rs13005	chr10	13643766	G	A	RP11-295P	120	0.619	SNP
.	chr10	16295671	CT	CTT	RP11-461K	133	0.693	INDEL
.	chr10	16295671	CT	C	RP11-461K	2	0.01	INDEL
.	chr10	16295671	CT	CTTT	RP11-461K	8	0.042	INDEL
rs1125440	chr10	17157779	C	A	TRDMT1	25	0.129	SNP
rs1125441	chr10	17162188	G	A	TRDMT1	83	0.428	SNP
522255	chr10	18150855	CTTTTTTT	CTTTTTT	CACNB2	29	0.167	INDEL
522255	chr10	18150855	CTTTTTTT	CTTTTT	CACNB2	4	0.023	INDEL
522255	chr10	18150855	CTTTTTTT	CTTTTTTTT	CACNB2	7	0.04	INDEL
522255	chr10	18150855	CTTTTTTT	C	CACNB2	4	0.023	INDEL
rs7412023	chr10	18150879	T	TTTTG	CACNB2	3	0.016	MIXED
rs7412023	chr10	18150879	T	TTTTTG	CACNB2	14	0.073	MIXED
rs7412023	chr10	18150879	T	G	CACNB2	2	0.01	MIXED
rs7412023	chr10	18150879	T	TTTTTTG	CACNB2	2	0.01	MIXED
rs1430601	chr10	18539262	C	T	CACNB2	4	0.021	SNP
rs7210276	chr10	21146411	ACACT	A	C10orf113	48	0.247	INDEL
.	chr10	21551800	CT	C	MLLT10	17	0.088	INDEL
.	chr10	21551800	CT	CTT	MLLT10	6	0.031	INDEL
.	chr10	22165707	TAAAAAAA	TAAAAAAA	ADIPOR1P1	8	0.042	INDEL
.	chr10	22165707	TAAAAAAA	TAAAAAAA	ADIPOR1P1	17	0.089	INDEL
.	chr10	22165707	TAAAAAAA	T	ADIPOR1P1	4	0.021	INDEL
.	chr10	22165707	TAAAAAAA	TAAAAAAA	ADIPOR1P1	2	0.011	INDEL
.	chr10	22165707	TAAAAAAA	TAAAAAAA	ADIPOR1P1	3	0.016	INDEL

rs1805397	chr10	26270681	C	T	GAD2	2	0.01	SNP
rs7902245	chr10	26667566	G	A	RP13-16H1	63	0.325	SNP
rs1042058	chr10	30439172	T	C	MAP3K8	116	0.598	SNP
.	chr10	30691072	A	T	SVILP1	2	0.01	SNP
rs3756172(	chr10	30723411	GAAGAA	G	RP11-14C2	160	0.825	INDEL
rs1693201(	chr10	30857686	T	C	ZNF438	33	0.17	SNP
rs1466799:	chr10	32451870	C	CT	CCDC7	2	0.01	INDEL
rs41276068	chr10	32518506	G	T	CCDC7	16	0.082	SNP
rs3714663:	chr10	32847890	TAA	T	AL356053.:	11	0.057	INDEL
rs2228637	chr10	33207648	G	C	NRP1	2	0.01	SNP
rs2229935	chr10	33221735	G	A	NRP1	62	0.32	SNP
rs1169390:	chr10	37130364	G	T	ANKRD30A	6	0.031	SNP
.	chr10	37193254	AG	A	ANKRD30A	7	0.036	INDEL
rs6143878	chr10	42429112	TCACCTTT(	T	CCNYL2	69	0.356	INDEL
.	chr10	42429113	CACCTTTG(	*	CCNYL2	69	0.356	INDEL
.	chr10	42429113	CACCTTTG(	C	CCNYL2	5	0.026	INDEL
rs7772490:	chr10	43118460	A	T	RET	6	0.031	SNP
.	chr10	43202662	CAG	C	RASGEF1A	2	0.01	INDEL
rs2002516	chr10	44292818	AC	ACC	C10orf142	4	0.021	INDEL
rs6593456	chr10	45015960	A	G	CEP164P1	141	0.727	SNP
.	chr10	45070664	CT	C	CEP164P1	2	0.01	INDEL
.	chr10	45847219	CGCTCCTG(	C	AGAP4	3	0.015	INDEL
rs6682548:	chr10	46263509	C	T	ANTXRLP1	11	0.057	SNP
rs3747414	chr10	46270980	ATTTTCTTC	A	ANTXRLP1	11	0.057	INDEL
rs1769672	chr10	46293101	G	A	ANTXRL	12	0.062	SNP
.	chr10	46583722	T	TC	SYT15	66	0.34	INDEL
.	chr10	47502280	CG	C	AGAP9	3	0.015	INDEL
rs2837744!	chr10	47726840	G	C	FAM35DP	4	0.021	SNP
.	chr10	48171180	CT	C	FRMPD2	13	0.068	INDEL
.	chr10	49551810	CAA	CA	RP11-140C	53	0.279	INDEL
.	chr10	49551810	CAA	C	RP11-140C	12	0.063	INDEL
rs8178992;	chr10	49655101	T	C	CHAT	168	0.866	SNP
rs1456898	chr10	49693871	C	A	C10orf53	2	0.01	SNP
rs1475845!	chr10	49709945	CTGTGTG	C	C10orf53	7	0.036	INDEL
.	chr10	49709959	GTGTGTGT	G	C10orf53	7	0.036	INDEL
.	chr10	49709961	GTGTGTGT	G	C10orf53	101	0.521	INDEL
.	chr10	49709961	GTGTGTGT	*	C10orf53	6	0.031	INDEL
.	chr10	49709963	GTGTGTGT	*	C10orf53	107	0.552	INDEL
.	chr10	49709963	GTGTGTGT	G	C10orf53	3	0.015	INDEL
.	chr10	49709967	GTGTGTC	*	C10orf53	110	0.567	MIXED
.	chr10	49709967	GTGTGTC	G	C10orf53	9	0.046	MIXED
.	chr10	49709967	GTGTGTC	CTGTGTC	C10orf53	54	0.278	MIXED
.	chr10	49988768	A	AC	AGAP6	49	0.253	INDEL
.	chr10	49988921	AC	A	AGAP6	15	0.077	INDEL
rs1412178(	chr10	50008914	CAA	C	AGAP6	63	0.325	INDEL
rs6026732(	chr10	50679172	A	G	NUTM2HP	2	0.01	SNP
rs5575465	chr10	51153278	C	T	PRKG1	2	0.01	SNP

rs3499749	chr10	52062541	A	G	PRKG1	7	0.036	SNP
.	chr10	52451518	C	A	LINC01468	2	0.01	SNP
rs1407437	chr10	59282560	A	G	RP11-443O	2	0.01	SNP
rs2456777	chr10	60792131	A	G	CDK1	130	0.67	SNP
rs2456778	chr10	60792132	A	T	CDK1	132	0.68	SNP
.	chr10	61412992	C	CATGGGTC	TMEM26	5	0.026	INDEL
rs3727476	chr10	62241190	G	A	RTKN2	2	0.01	SNP
rs3211105	chr10	63185604	G	A	JMJD1C	148	0.763	SNP
.	chr10	63664772	G	GCTTCTTC(	RP11-170M	2	0.01	INDEL
rs1902387	chr10	65585568	G	A	LINC01515	124	0.639	SNP
rs1099665	chr10	65766624	G	C	LINC01515	12	0.062	SNP
rs2273773	chr10	67906841	T	C	SIRT1	12	0.062	SNP
rs1512596	chr10	68788131	TTTATA	T	CCAR1	5	0.026	INDEL
rs977096	chr10	69630637	A	G	C10orf35	37	0.191	SNP
rs6018857	chr10	70529208	GCCCCC	G	PALD1	3	0.016	INDEL
rs6018857	chr10	70529208	GCCCCC	GCCCC	PALD1	17	0.092	INDEL
rs6018857	chr10	70529208	GCCCCC	GCC	PALD1	26	0.141	INDEL
rs6018857	chr10	70529208	GCCCCC	GCCC	PALD1	13	0.071	INDEL
rs6018857	chr10	70529208	GCCCCC	GC	PALD1	29	0.158	INDEL
rs6018857	chr10	70529208	GCCCCC	GCCCCCC	PALD1	9	0.049	INDEL
rs865832	chr10	70871869	C	T	SGPL1	40	0.206	SNP
rs1221863	chr10	72215835	T	C	ASCC1	8	0.041	SNP
rs3793927;	chr10	72508273	T	C	MICU1	122	0.629	SNP
rs1426545	chr10	72692883	CTG	C	MCU	6	0.031	INDEL
.	chr10	72861656	AT	A	MCU	42	0.216	INDEL
.	chr10	72861656	AT	ATT	MCU	11	0.057	INDEL
rs3755243	chr10	72861777	G	A	MCU	2	0.01	SNP
rs3833730	chr10	73126634	GC	G	NUDT13	10	0.052	INDEL
.	chr10	73168308	TGAG	T	FAM149B1	3	0.015	INDEL
.	chr10	73648134	T	G	SYNPO2L	2	0.013	MIXED
rs3740316	chr10	75095663	G	A	DUSP13	35	0.18	SNP
rs3832680	chr10	75099203	CT	C	DUSP13	45	0.232	INDEL
rs7489244	chr10	75298933	CTT	CT	ZNF503-AS	22	0.113	INDEL
rs7489244	chr10	75298933	CTT	CTTT	ZNF503-AS	13	0.067	INDEL
rs7489244	chr10	75298933	CTT	C	ZNF503-AS	4	0.021	INDEL
.	chr10	77376558	TTTTTTTTC	T	KCNMA1&I	23	0.119	INDEL
rs1175599	chr10	78549699	C	T	RP11-17G2	7	0.036	SNP
rs2233719	chr10	79349702	C	T	PPIF	3	0.015	SNP
rs1480357	chr10	79665595	C	T	NUTM2B-A	49	0.253	SNP
rs2275190	chr10	80055914	G	A	TMEM254-	92	0.474	SNP
rs1131725	chr10	80081665	C	CA	TMEM254	14	0.072	INDEL
rs1131725	chr10	80081665	C	CAA	TMEM254	13	0.067	INDEL
rs1435387	chr10	80081672	AAAAG	A	TMEM254	14	0.072	INDEL
rs1495064	chr10	80144341	G	GAGA	PLAC9	3	0.015	INDEL
rs1120196	chr10	80171611	A	G	ANXA11	16	0.082	SNP
rs2993763;	chr10	80273838	G	A	MAT1A	81	0.418	SNP
rs6173447	chr10	80275179	G	A	MAT1A	2	0.01	SNP

rs1143694;	chr10	80280296	A	G	MAT1A	128	0.66	SNP
rs2351556	chr10	85461594	A	G	LINC01520	105	0.541	SNP
rs7094132	chr10	86443311	T	C	WAPL	184	0.948	SNP
rs9864	chr10	86962641	A	T	SNCG	51	0.263	SNP
rs1828122(	chr10	87245856	C	G	NUTM2A-A	4	0.021	SNP
rs2180969	chr10	87658954	A	G	RP11-57C1	4	0.021	SNP
229653	chr10	87960876	CT	CTT	PTEN	30	0.155	INDEL
229653	chr10	87960876	CT	C	PTEN	20	0.103	INDEL
229653	chr10	87960876	CT	CTTT	PTEN	2	0.01	INDEL
rs7477768	chr10	88594666	G	C	LIPJ	2	0.01	SNP
rs1385360(	chr10	88596265	CTTATT	C	LIPJ	8	0.041	INDEL
rs1381704!	chr10	88815223	AG	A	LIPM	2	0.01	INDEL
rs7924289(	chr10	89412799	AG	A	LIPA	32	0.165	INDEL
rs3542665	chr10	92487235	C	A	IDE	6	0.031	SNP
rs3404893!	chr10	93323125	G	A	MYOF	18	0.093	SNP
rs1924699	chr10	93805497	G	T	LGI1	179	0.923	SNP
rs1799853;	chr10	94942290	C	T	CYP2C9	21	0.108	SNP
rs1057910;	chr10	94981296	A	C	CYP2C9	14	0.072	SNP
rs1057911	chr10	94988980	A	T	CYP2C9	14	0.072	SNP
rs1058930;	chr10	95058362	G	C	CYP2C8	9	0.046	SNP
rs1157207	chr10	95067362	T	TA	CYP2C8	41	0.211	INDEL
rs2071426;	chr10	95068566	T	C	CYP2C8	68	0.351	SNP
rs4129164	chr10	96633059	T	C	PIK3AP1	10	0.052	SNP
.	chr10	97583573	CAGGCAG(	C	ANKRD2	3	0.015	INDEL
rs4129045(	chr10	97599144	G	A	HOGA1	4	0.021	SNP
rs3814556	chr10	97616395	A	G	MORN4	46	0.237	SNP
rs3818780	chr10	97679916	C	G	AVPI1	27	0.139	SNP
rs1181501	chr10	97771814	C	G	SFRP5	165	0.851	MIXED
rs1119030	chr10	99877160	C	T	DNMBP	4	0.021	SNP
rs3750715	chr10	99956727	G	A	DNMBP	3	0.015	SNP
rs4604805	chr10	1.01E+08	G	C	SEC31B	35	0.18	SNP
rs2295778	chr10	1.01E+08	C	G	HIF1AN	48	0.247	SNP
rs1711480	chr10	1.03E+08	T	C	SUFU	20	0.103	SNP
.	chr10	1.03E+08	TCCTGAGT(	T	AS3MT&BC	12	0.062	INDEL
rs6174613(	chr10	1.03E+08	C	T	RPEL1	2	0.01	SNP
.	chr10	1.04E+08	A	C	SH3PXD2A	2	0.01	SNP
rs1088396(	chr10	1.04E+08	A	G	SLK	44	0.227	SNP
rs7232378	chr10	1.04E+08	AGAG	A	GSTO1	5	0.026	INDEL
rs1889567	chr10	1.11E+08	C	T	DUSP5	57	0.294	SNP
rs2010846	chr10	1.14E+08	G	A	NRAP	2	0.01	SNP
rs1055359(	chr10	1.14E+08	CTT	C	CASP7	51	0.263	INDEL
rs1412669	chr10	1.14E+08	T	C	CASP7	4	0.021	SNP
rs2227310	chr10	1.14E+08	C	G	CASP7	48	0.247	SNP
.	chr10	1.14E+08	CAAAA	C	CTB-1144G	11	0.057	INDEL
.	chr10	1.14E+08	CAAAA	CAAAAA	CTB-1144G	29	0.149	INDEL
.	chr10	1.14E+08	CAAAA	CAAAAAA	CTB-1144G	10	0.052	INDEL
.	chr10	1.14E+08	CAAAA	CA	CTB-1144G	7	0.036	INDEL

.	chr10	1.14E+08	CAAAA	CAAA	CTB-1144G	12	0.062	INDEL
rs1458431	chr10	1.16E+08	G	GA	PNLIPRP3	15	0.077	INDEL
rs1119774	chr10	1.17E+08	A	G	PNLIPRP1	4	0.021	SNP
rs1166101	chr10	1.17E+08	T	C	PNLIPRP1	2	0.01	SNP
rs4751995	chr10	1.17E+08	A	G	PNLIPRP2	104	0.536	SNP
.	chr10	1.19E+08	CTT	CT	FAM45A	24	0.124	INDEL
.	chr10	1.19E+08	CTT	CTTT	FAM45A	7	0.036	INDEL
rs1155021	chr10	1.2E+08	G	A	RGS10	7	0.036	SNP
rs1047100;	chr10	1.22E+08	T	C	FGFR2	134	0.691	SNP
rs2736911;	chr10	1.22E+08	C	T	ARMS2	42	0.216	SNP
.	chr10	1.24E+08	CGG	CG	CHST15	99	0.51	INDEL
.	chr10	1.24E+08	CGG	CGGG	CHST15	16	0.082	INDEL
.	chr10	1.24E+08	CGG	C	CHST15	12	0.062	INDEL
.	chr10	1.24E+08	CGG	CGGGG	CHST15	16	0.082	INDEL
.	chr10	1.24E+08	CGG	CGGGGGG	CHST15	2	0.01	INDEL
.	chr10	1.24E+08	CGG	CGGGGG	CHST15	6	0.031	INDEL
rs11461;16	chr10	1.24E+08	G	A	OAT	88	0.454	SNP
rs1179480	chr10	1.25E+08	G	T	NPM1P31	6	0.031	SNP
rs1418647	chr10	1.25E+08	CCT	C	CTBP2	2	0.01	INDEL
.	chr10	1.3E+08	C	G	RP11-109A	2	0.01	SNP
rs728900	chr10	1.3E+08	T	A	RP11-109A	84	0.433	SNP
rs5624344	chr10	1.3E+08	G	A	RP11-109A	6	0.031	SNP
.	chr10	1.32E+08	C	CCTAT	STK32C	3	0.015	INDEL
rs1846315	chr10	1.32E+08	C	T	PWWP2B	2	0.01	SNP
rs1458118	chr10	1.33E+08	T	TG	ADGRA1	2	0.01	INDEL
rs1131720	chr10	1.33E+08	C	T	ADAM8	177	0.912	SNP
.	chr10	1.33E+08	CGTTTTCCT	C	RP11-108K	13	0.067	INDEL
rs731946	chr10	1.33E+08	G	C	SCART1	3	0.015	SNP
rs5789167	chr10	1.33E+08	CT	C	SCART1	19	0.098	INDEL
rs5789167	chr10	1.33E+08	CT	CTT	SCART1	9	0.046	INDEL
rs6413419	chr10	1.34E+08	G	A	CYP2E1	8	0.041	SNP
rs2149616	chr10	1.34E+08	A	G	CYP2E1	8	0.041	SNP
.	chr10	1.34E+08	AC	A	FRG2B	50	0.258	INDEL
rs1124602	chr11	233067	C	T	SIRT3	46	0.237	SNP
rs1155523	chr11	233212	C	A	SIRT3	46	0.237	SNP
.	chr11	433356	TG	T	ANO9	2	0.01	INDEL
.	chr11	552565	CTAG	C	LRRC56	4	0.067	INDEL
.	chr11	640098	C	CGGCCTCC	DRD4	2	0.022	INDEL
rs3466205	chr11	640099	A	G	DRD4	8	0.4	MIXED
rs3466205	chr11	640099	A	ACCCCTGC	DRD4	2	0.1	MIXED
rs3578249	chr11	704604	GA	G	TMEM80	90	0.464	INDEL
.	chr11	752245	AT	A	TALDO1	17	0.088	INDEL
.	chr11	752245	AT	ATT	TALDO1	33	0.17	INDEL
.	chr11	752245	AT	ATTT	TALDO1	2	0.01	INDEL
rs6187674	chr11	827713	A	T	CRACR2B	81	0.418	SNP
rs1124632	chr11	843713	C	T	TSPAN4	53	0.273	MIXED
rs1124632	chr11	843713	C	CTCCTTGG	TSPAN4	138	0.711	MIXED

.	chr11	1191643	G	GGT	MUC5AC	5	0.026	MIXED
.	chr11	1191643	G	*	MUC5AC	10	0.052	MIXED
.	chr11	1195871	CACAGGGT	C	MUC5AC	2	0.01	INDEL
.	chr11	1195892	CACACTGT(	C	MUC5AC	2	0.01	INDEL
rs1181755	chr11	2164329	G	A	TH	2	0.01	SNP
rs1156471(	chr11	2167446	C	T	TH	2	0.01	SNP
rs1888391(	chr11	2301859	C	T	C11orf21	5	0.026	SNP
rs2074022	chr11	2302811	A	G	TSPAN32	186	0.959	SNP
rs3764892	chr11	2917012	C	T	SLC22A18	25	0.164	MIXED
rs183484	chr11	4119902	C	A	RRM1	86	0.443	SNP
rs9937	chr11	4138227	A	G	RRM1	86	0.443	SNP
rs1131040	chr11	4368174	AG	A	OR52B4	83	0.428	INDEL
rs1416004(	chr11	4571476	T	TAC	C11orf40	34	0.175	INDEL
rs7128081	chr11	4571478	C	CAG	C11orf40	70	0.361	INDEL
rs1467116	chr11	4573328	T	TG	C11orf40	10	0.052	INDEL
rs7944853(	chr11	4577726	C	T	C11orf40	10	0.052	SNP
rs3467292	chr11	4769643	CG	C	OR51F1	30	0.155	INDEL
.	chr11	4882443	T	TACCACCC(	OR51T1	2	0.01	INDEL
rs5702647	chr11	5047432	C	T	OR52J3	25	0.129	SNP
rs5789358	chr11	5070346	A	AC	OR52E1	83	0.428	INDEL
rs1120989	chr11	5151565	A	AC	OR52A1	47	0.242	INDEL
rs5789371	chr11	5178587	GC	G	OR52Z1	142	0.732	INDEL
rs1280521	chr11	5205088	G	A	AC104389.	20	0.103	SNP
rs713040;1	chr11	5227013	A	G	HBB	168	0.866	SNP
rs1470626(	chr11	5343311	CCAGCCCC.	C	OR51B5	5	0.026	INDEL
rs1130473	chr11	5403157	T	TTATC	OR51J1	12	0.062	INDEL
rs2647574	chr11	5422906	C	T	OR51Q1	88	0.454	SNP
rs1693099	chr11	5441472	G	A	OR51I1	8	0.041	SNP
rs3530158	chr11	5454201	T	TCA	OR51I2	74	0.381	INDEL
rs3606620	chr11	5489310	G	GGGCT	OR52D1	28	0.144	INDEL
rs4910844	chr11	5755254	A	T	OR52N4	45	0.232	SNP
rs1424427	chr11	5788576	GA	G	OR52N1	11	0.057	INDEL
rs1241960	chr11	5856749	T	A	OR52E8	91	0.469	SNP
rs6173042	chr11	5967993	G	A	OR56A5	18	0.093	SNP
.	chr11	6319672	C	A	PRKCDBP	4	0.021	SNP
.	chr11	6402571	CTTACTT	C	APBB1	2	0.01	INDEL
rs1997520(	chr11	6558077	C	T	DNHD1	2	0.01	SNP
rs1043388;	chr11	6608435	C	T	ILK	55	0.284	SNP
rs5926461(	chr11	6921357	TC	T	OR2D3	10	0.052	INDEL
rs6716729	chr11	6926396	GCGGGGC(	G	RP11-413N	185	0.954	INDEL
rs6106308	chr11	7038750	G	A	NLRP14	32	0.165	SNP
rs2009621	chr11	8392542	C	CA	STK33	3	0.015	INDEL
rs2019273	chr11	8392543	G	GAACCA	STK33	3	0.015	INDEL
rs7766371	chr11	8680666	G	C	RP11-152H	4	0.021	SNP
.	chr11	9438045	GTTTTTTTT	GT	IPO7	73	0.376	INDEL
.	chr11	9438045	GTTTTTTTT	GTT	IPO7	6	0.031	INDEL
.	chr11	9438045	GTTTTTTTT	GTTTTTTTT	IPO7	44	0.227	INDEL

.	chr11	9438045	GTTTTTTTT	GTTTTTTTT	IPO7	12	0.062	INDEL
.	chr11	9438045	GTTTTTTTT	GTTTTTTTT	IPO7	7	0.036	INDEL
rs2020819	chr11	9783105	GACTGGT	G	SBF2-AS1	92	0.474	INDEL
rs7938316	chr11	10450517	C	T	AMPD3	81	0.418	SNP
rs2001244	chr11	10499234	G	GT	AMPD3	6	0.031	INDEL
rs1462941	chr11	10541543	G	T	MRVI1-AS1	8	0.041	SNP
rs1104290	chr11	10634076	C	T	MRVI1	62	0.32	SNP
.	chr11	12072170	CT	CTTT	RP13-631K	76	0.396	INDEL
.	chr11	12072170	CT	CTTTT	RP13-631K	6	0.031	INDEL
.	chr11	12072170	CT	CTTTTT	RP13-631K	21	0.109	INDEL
.	chr11	12072170	CT	CTT	RP13-631K	11	0.057	INDEL
.	chr11	12072170	CT	C	RP13-631K	8	0.042	INDEL
.	chr11	12294624	C	CA	MICALCL	2	0.01	INDEL
.	chr11	12477099	CTT	CTTT	PARVA	22	0.113	INDEL
.	chr11	12477099	CTT	CT	PARVA	9	0.046	INDEL
.	chr11	12477099	CTT	C	PARVA	4	0.021	INDEL
rs3761859	chr11	13963036	C	T	SPON1	45	0.232	SNP
rs2303973	chr11	14243370	A	G	SPON1	76	0.392	SNP
.	chr11	14499598	CGTA	C	COPB1	2	0.01	INDEL
.	chr11	14499607	A	C	COPB1	2	0.01	SNP
rs1279471	chr11	14892029	G	A	CYP2R1	86	0.443	SNP
.	chr11	17193847	AAAAAGAA	AAAAAGAA	RP11-452G	2	0.011	INDEL
rs6140681	chr11	17372490	CTT	C	NCR3LG1	11	0.057	INDEL
rs2074308	chr11	17431033	C	T	ABCC8	34	0.175	SNP
rs1450136	chr11	17527316	C	T	USH1C	2	0.01	SNP
417941	chr11	17574890	C	T	OTOG	3	0.015	SNP
rs1396798	chr11	18006658	G	T	SERGEF	2	0.01	SNP
rs7840823	chr11	18138122	G	A	MRGPRX3	18	0.093	SNP
rs1512193	chr11	18197166	C	CT	MRGPRX4	7	0.036	INDEL
rs6175291	chr11	18400979	G	A	LDHA	3	0.015	SNP
rs5030621;	chr11	18402904	C	T	LDHA	133	0.686	SNP
rs6175231	chr11	18578736	T	C	UEVLD	6	0.031	SNP
rs3824886	chr11	20383067	T	G	HTATIP2	186	0.959	SNP
rs7812658	chr11	22113483	T	C	CTD-2019C	23	0.119	SNP
rs6262136	chr11	22480071	A	G	LINC01495	23	0.119	SNP
rs7703519	chr11	27127300	G	A	BBOX1	7	0.036	SNP
rs3491348	chr11	28084706	G	A	KIF18A	3	0.015	SNP
rs1867930	chr11	28125509	G	A	METTL15	2	0.01	SNP
rs1430388	chr11	30584592	G	A	RP5-1024C	2	0.01	SNP
rs5756931	chr11	33074141	CG	C	AC131263.	4	0.021	INDEL
rs3758741	chr11	33085070	C	T	TCP11L1	61	0.314	SNP
.	chr11	33335644	C	CT	HIPK3	4	0.021	INDEL
rs7348098	chr11	33699576	G	A	C11orf91	5	0.026	SNP
.	chr11	34431229	CA	C	CIR1P3	12	0.062	INDEL
rs769217;5	chr11	34461361	C	T	CAT	44	0.227	SNP
rs1571133	chr11	34888379	T	G	APIP	134	0.691	SNP
rs1977420	chr11	34888850	C	T	APIP	69	0.356	SNP

rs3763930;	chr11	34915939	G	C	PDHX	24	0.124	SNP
rs7104194	chr11	35020187	CTT	C	PDHX	30	0.155	INDEL
rs7104194	chr11	35020187	CTT	CT	PDHX	28	0.144	INDEL
rs7104194	chr11	35020187	CTT	CTTT	PDHX	36	0.186	INDEL
rs1401840	chr11	35222374	G	A	CD44	2	0.01	SNP
rs5620561	chr11	35322560	C	T	SLC1A2	9	0.046	SNP
rs6090477	chr11	36536035	G	A	RAG1	31	0.16	SNP
rs1877022	chr11	41821394	G	C	LINC01499	44	0.227	SNP
rs1048928	chr11	43919094	G	T	ALKBH3	117	0.603	SNP
rs1973716	chr11	43925029	G	C	RP11-613D	21	0.108	SNP
rs1103778	chr11	43995055	G	T	RP11-613D	97	0.5	SNP
rs1446726	chr11	44082189	GAGTGTAA	G	ACCS	66	0.34	INDEL
rs1083834	chr11	44736403	G	A	RP11-45A1	153	0.789	SNP
rs5791652	chr11	44903399	GC	G	TP53I11	69	0.356	INDEL
rs1145636	chr11	44903808	C	CT	TP53I11	124	0.639	INDEL
rs1389823	chr11	44936625	GC	G	TP53I11	9	0.046	INDEL
.	chr11	46315170	G	C	CREB3L1	2	0.01	SNP
.	chr11	46382790	GCCC	GC	MDK	11	0.057	INDEL
.	chr11	46382790	GCCC	GCC	MDK	33	0.17	INDEL
.	chr11	46382790	GCCC	GCCCC	MDK	14	0.072	INDEL
.	chr11	46382790	GCCC	GCCCCC	MDK	3	0.015	INDEL
.	chr11	46382790	GCCC	G	MDK	10	0.052	INDEL
.	chr11	46644265	CT	CTT	ATG13	7	0.036	INDEL
.	chr11	46644265	CT	C	ATG13	5	0.026	INDEL
rs3136532;	chr11	46739363	C	T	F2	3	0.015	SNP
.	chr11	47182999	CATA	C	PACSIN3	3	0.015	INDEL
rs1442666	chr11	47234792	G	A	DDB2	2	0.01	SNP
rs3781620	chr11	47237713	G	C	DDB2	26	0.134	SNP
rs3523310	chr11	47285079	C	T	MADD	4	0.021	SNP
rs3729989;	chr11	47348490	T	C	MYBPC3	27	0.139	SNP
rs3218719;	chr11	47350027	G	A	MYBPC3	5	0.026	SNP
rs3528203	chr11	47358725	G	GCA	SPI1	15	0.077	INDEL
.	chr11	47400952	TCTGAAGG	T	MIR4487&	11	0.057	INDEL
.	chr11	47581159	AT	A	NDUFS3	7	0.036	INDEL
.	chr11	47581159	AT	ATT	NDUFS3	4	0.021	INDEL
.	chr11	47638806	CTA	C	MTCH2	9	0.046	INDEL
.	chr11	47638806	CTA	CATAACAA	MTCH2	3	0.015	INDEL
rs7120775	chr11	48245184	C	G	OR4X2	16	0.082	SNP
rs7120775	chr11	48245184	C	T	OR4X2	3	0.015	SNP
rs1083885	chr11	48264679	T	A	OR4X1	153	0.789	SNP
rs1965370	chr11	48978871	C	G	TRIM51GP	169	0.871	SNP
rs202716	chr11	49174938	T	C	FOLH1	35	0.18	SNP
rs202680	chr11	49200333	T	A	FOLH1	35	0.18	SNP
rs202676	chr11	49206068	A	G	FOLH1	24	0.124	SNP
rs7145594	chr11	49851888	CTGGACCA	C	RP11-163O	136	0.701	INDEL
rs6189489	chr11	55269336	T	C	TRIM48	17	0.089	SNP
rs1459101	chr11	55572176	C	T	OR4C16	56	0.289	SNP

rs7542353	chr11	55603905	G	A	OR4C11	16	0.09	SNP
rs7616013	chr11	55638546	C	G	OR4P4	16	0.09	SNP
.	chr11	55872879	C	CGGGCGCC	RP11-738O	13	0.067	INDEL
rs1383724	chr11	56093801	GT	G	OR8I2	17	0.088	INDEL
rs1446908	chr11	56093830	CA	C	OR8I2	17	0.088	INDEL
rs6188709	chr11	56094174	C	G	OR8I2	17	0.088	SNP
rs1063338	chr11	56413160	A	ACT	OR5AL1	180	0.928	INDEL
rs1754728	chr11	56542880	A	T	OR5M11	17	0.088	SNP
rs1484381	chr11	56577367	CCATTGAA	C	OR5M10	17	0.088	INDEL
rs7200305	chr11	56613070	CCAGA	C	OR5M1	55	0.284	INDEL
rs1122871	chr11	56663740	C	T	OR5AR1	134	0.691	SNP
.	chr11	56700566	TTTTCACT	T	OR9G1	2	0.013	INDEL
rs6188888	chr11	57325698	G	A	RP11-872D	15	0.077	SNP
rs1435755	chr11	57753543	G	A	CTNND1	3	0.015	SNP
rs3484625	chr11	58031636	AC	A	OR6Q1	29	0.149	INDEL
rs7103033	chr11	58215722	A	G	OR1S1	63	0.325	SNP
rs2007991	chr11	58403319	AG	A	OR5B3	10	0.052	INDEL
rs2015671	chr11	58439562	AT	A	OR5B12	6	0.031	INDEL
rs7913582	chr11	58610949	G	A	ZFP91	8	0.041	SNP
rs1998511	chr11	59124488	GT	G	FAM111B	11	0.057	INDEL
770974	chr11	59152449	G	GGCAGATA	FAM111A	2	0.01	INDEL
rs6168913	chr11	59255552	G	A	WARSP1	121	0.624	SNP
rs7704857	chr11	59457656	GC	G	OR4D6	2	0.01	INDEL
rs7589855	chr11	59478120	G	T	OR4D10	2	0.01	SNP
rs499037	chr11	59713479	G	A	OR10V1	2	0.01	SNP
rs1241972	chr11	59971194	G	A	OOSP1	10	0.052	SNP
rs1121930	chr11	60078004	TCAACAAC.	TCAA	RP11-736I1	58	0.299	INDEL
rs1121930	chr11	60078004	TCAACAAC.	T	RP11-736I1	61	0.314	INDEL
rs1121930	chr11	60078004	TCAACAAC.	TCAACAAC.	RP11-736I1	37	0.191	INDEL
rs1121930	chr11	60078004	TCAACAAC.	TCAACAA	RP11-736I1	31	0.16	INDEL
rs598862	chr11	60178829	C	T	MS4A6A	189	0.974	SNP
rs3217518	chr11	60397879	ATT	A	MS4A14	139	0.716	INDEL
rs4938941	chr11	60405887	A	G	MS4A14	139	0.716	SNP
rs2298553	chr11	60497529	C	T	MS4A12	95	0.49	SNP
rs1474833	chr11	60899179	G	A	PRPF19	4	0.021	SNP
rs2872029	chr11	61322299	C	T	DDB1	3	0.015	SNP
rs2230356	chr11	61331600	G	A	DDB1	23	0.119	SNP
rs1138254	chr11	61398259	C	CA	TMEM216	166	0.856	INDEL
rs1089715	chr11	61398269	G	C	TMEM216	162	0.835	SNP
rs4963390	chr11	61747349	A	G	DKFZP434K	31	0.16	SNP
rs3515667	chr11	62602409	G	A	EML3	62	0.32	SNP
rs4693	chr11	62796537	T	C	NXF1	127	0.655	SNP
rs7105855	chr11	62796941	C	T	NXF1	63	0.325	SNP
rs1123134	chr11	63081015	A	C	SLC22A24	159	0.82	SNP
rs7700218	chr11	63083303	G	A	SLC22A24	4	0.021	SNP
.	chr11	63143377	C	T	SLC22A24	31	0.16	SNP
rs1123139	chr11	63183749	C	G	SLC22A25	60	0.309	SNP

rs1790218	chr11	63290453	G	A	SLC22A10	127	0.655	SNP
.	chr11	63382198	C	CA	SLC22A9	5	0.026	INDEL
.	chr11	63490965	T	TGG	HRASLS5	2	0.01	INDEL
rs1127101	chr11	63850023	ACTCTAGG	A	MARK2	11	0.057	INDEL
rs1160270	chr11	63987039	G	T	OTUB1	13	0.067	SNP
.	chr11	64231883	TACTTATTA	T	DNAJC4	2	0.01	INDEL
rs1241966	chr11	64255798	C	T	PLCB3	2	0.01	SNP
rs2244625	chr11	64258672	G	A	PLCB3	136	0.701	SNP
rs3516979	chr11	64263769	C	T	PLCB3	13	0.067	SNP
.	chr11	64341843	GGTTGTGG	G	MIR7155	50	0.258	INDEL
rs2071313;	chr11	64805130	G	A	MEN1	96	0.495	SNP
.	chr11	64903579	GCCCTGCC	G	ATG2A	9	0.046	INDEL
rs6173663	chr11	65021750	C	T	ARL2	3	0.015	SNP
.	chr11	65025779	CTTT	CTT	AP000436.	92	0.484	INDEL
.	chr11	65025779	CTTT	CT	AP000436.	13	0.068	INDEL
.	chr11	65025779	CTTT	CTTTT	AP000436.	4	0.021	INDEL
.	chr11	65025779	CTTT	C	AP000436.	4	0.021	INDEL
.	chr11	65557803	CGCCTGCG	C	LTBP3	2	0.01	INDEL
rs6175989	chr11	65658293	C	T	RELA	5	0.026	SNP
rs667555	chr11	65857091	A	C	CFL1	132	0.68	SNP
rs1939212	chr11	65859230	C	T	CFL1	62	0.32	SNP
rs575085	chr11	65861589	C	T	CFL1	3	0.015	SNP
rs630303;4	chr11	65864502	G	A	MUS81	125	0.644	SNP
rs558114;4	chr11	65864791	G	T	MUS81	125	0.644	SNP
.	chr11	65865802	G	GC	MUS81	2	0.01	INDEL
.	chr11	65879879	TGCCTCCT	T	CTSW	5	0.026	INDEL
rs2305535	chr11	66504766	G	A	DPP3	52	0.268	SNP
18312	chr11	66560624	C	T	ACTN3	80	0.412	SNP
rs3741189	chr11	67228132	T	C	KDM2A	185	0.954	SNP
rs2228418	chr11	67266795	C	A	GRK2	170	0.876	SNP
rs1385188	chr11	67396309	G	A	RAD9A	2	0.01	SNP
rs2228522	chr11	67398839	G	A	PPP1CA	9	0.046	SNP
.	chr11	67451597	C	CAGCTGTG	GPR152	3	0.015	INDEL
rs1431995	chr11	67467580	G	A	TMEM134	5	0.026	SNP
rs7947754	chr11	67674435	A	C	ALDH3B2	194	1	SNP
rs1481575	chr11	68929226	C	T	IGHMBP2	2	0.01	SNP
rs3862792	chr11	69648088	C	T	CCND1	5	0.026	SNP
rs552282	chr11	70383065	G	A	PPFIA1	61	0.314	SNP
rs643301	chr11	70433660	C	T	CTTN	34	0.175	SNP
rs9888234	chr11	70571343	C	T	SHANK2	146	0.753	SNP
rs3513227	chr11	70952751	CG	C	SHANK2	55	0.284	INDEL
.	chr11	70952780	A	AACTCGCC	SHANK2	194	1	INDEL
rs1155521	chr11	71441401	C	T	DHCR7	2	0.01	SNP
.	chr11	71568831	GGTCCAGC	G	KRTAP5-10	17	0.088	INDEL
rs5595467	chr11	72050661	C	T	NUMA1	2	0.01	SNP
rs1880809	chr11	72096721	A	C	LRTOMT	3	0.015	SNP
768461	chr11	72139110	CTA	C	FOLR3	181	0.933	INDEL

rs1174680:	chr11	72582446	T	C	PDE2A	3	0.015	SNP
rs1280521{	chr11	72685945	G	A	ARAP1	100	0.515	SNP
.	chr11	73384343	CAGATT	C	RELT	2	0.01	INDEL
.	chr11	73722325	ATATATAT/	ATT	RAB6A&RP	5	0.026	INDEL
.	chr11	73722327	ATATATAT/	A	RAB6A&RP	2	0.011	INDEL
.	chr11	73722327	ATATATAT/	*	RAB6A&RP	5	0.026	INDEL
.	chr11	73722329	ATATATAT/	*	RAB6A&RP	8	0.042	INDEL
.	chr11	73722329	ATATATAT/	AT	RAB6A&RP	2	0.011	INDEL
.	chr11	73722329	ATATATAT/	ATTTT	RAB6A&RP	4	0.021	INDEL
.	chr11	73722329	ATATATAT/	ATT	RAB6A&RP	4	0.021	INDEL
.	chr11	73722331	ATATATAT/	ATTTTTT	RAB6A&RP	4	0.021	INDEL
.	chr11	73722331	ATATATAT/	AT	RAB6A&RP	31	0.161	INDEL
.	chr11	73722331	ATATATAT/	ATTTTT	RAB6A&RP	26	0.135	INDEL
.	chr11	73722331	ATATATAT/	*	RAB6A&RP	21	0.109	INDEL
.	chr11	73722331	ATATATAT/	A	RAB6A&RP	4	0.021	INDEL
.	chr11	73722333	ATATATAT/	ATTTT	RAB6A&RP	4	0.021	INDEL
.	chr11	73722333	ATATATAT/	*	RAB6A&RP	83	0.432	INDEL
.	chr11	73722333	ATATATAT/	ATTTTTT	RAB6A&RP	10	0.052	INDEL
.	chr11	73722333	ATATATAT/	ATTTTT	RAB6A&RP	7	0.036	INDEL
.	chr11	73722335	ATATATAT/	*	RAB6A&RP	100	0.526	INDEL
.	chr11	73722335	ATATATAT/	ATT	RAB6A&RP	3	0.016	INDEL
.	chr11	73722335	ATATATAT/	ATTT	RAB6A&RP	3	0.016	INDEL
.	chr11	73722335	ATATATAT/	AT	RAB6A&RP	6	0.032	INDEL
.	chr11	73722337	ATATATAT	*	RAB6A&RP	105	0.565	INDEL
.	chr11	73722337	ATATATAT	ATT	RAB6A&RP	4	0.022	INDEL
.	chr11	73722337	ATATATAT	A	RAB6A&RP	3	0.016	INDEL
.	chr11	73722337	ATATATAT	AT	RAB6A&RP	6	0.032	INDEL
.	chr11	73722339	ATATATTTI	*	RAB6A&RP	110	0.591	INDEL
.	chr11	73722339	ATATATTTI	AT	RAB6A&RP	4	0.022	INDEL
rs1481477(	chr11	73817842	T	A	MRPL48	4	0.021	SNP
rs1059655(	chr11	74108144	CTG	C	C2CD3	34	0.175	INDEL
rs7831544!	chr11	74171045	T	TGG	C2CD3	4	0.021	INDEL
rs7831544!	chr11	74171045	T	TG	C2CD3	3	0.015	INDEL
rs3741132	chr11	74267198	G	A	P4HA3	35	0.18	SNP
rs2304683	chr11	74852032	G	A	XRRA1	51	0.263	SNP
rs1123643(	chr11	75430585	T	C	KLHL35	104	0.536	MIXED
rs3420367(	chr11	76206872	A	AG	WNT11	156	0.804	INDEL
rs1439539!	chr11	77184725	GGGAGGC(	G	MYO7A	98	0.505	INDEL
rs7622920:	chr11	77243743	T	TA	GDPD4	54	0.278	INDEL
rs6040027/	chr11	77918086	A	AT	AAMDC	128	0.66	INDEL
rs2228312	chr11	82825066	G	C	PRCP	7	0.036	SNP
rs4843	chr11	82839345	A	G	PRCP	3	0.015	SNP
rs2229437	chr11	82853252	T	G	PRCP	32	0.165	SNP
rs6023717(	chr11	83273727	T	TA	CCDC90B	11	0.057	INDEL
rs3434347:	chr11	86727524	C	T	CTD-2005H	42	0.216	SNP
.	chr11	87025335	CAAAAAA	CAAAAA	HNRNPCP8	10	0.072	INDEL
.	chr11	87025335	CAAAAAA	CAAAA	HNRNPCP8	20	0.145	INDEL

.	chr11	87025335	CAAAAAA	CAAAAAAA	HNRNPCP8	4	0.029	INDEL
.	chr11	87025335	CAAAAAA	CAAAAAAA	HNRNPCP8	5	0.036	INDEL
.	chr11	87025335	CAAAAAA	CAAAAAAA	HNRNPCP8	2	0.014	INDEL
rs6190550	chr11	89687439	G	A	FOLH1B	9	0.046	SNP
rs3710456	chr11	89875640	GC	G	TRIM64B	8	0.043	INDEL
.	chr11	90086720	C	CT	UBTFL1	37	0.195	INDEL
.	chr11	90086945	TCTGCAAG	T	UBTFL1	3	0.016	INDEL
rs1101887	chr11	90147436	G	A	NAALAD2	6	0.031	SNP
rs7621746	chr11	92915856	C	G	RP11-676F	3	0.015	SNP
rs496797	chr11	94492641	C	T	MRE11A	95	0.49	SNP
rs7712224	chr11	95794361	T	G	CEP57	9	0.046	SNP
rs6173557	chr11	95838183	C	G	MTMR2	9	0.046	SNP
rs1138979	chr11	95845106	C	T	MTMR2	8	0.041	SNP
.	chr11	95847900	C	CTTGT	MTMR2	2	0.01	INDEL
.	chr11	95849862	C	CTGGGATA	MTMR2	2	0.01	INDEL
.	chr11	95857636	C	CAGGT	MTMR2	2	0.01	INDEL
.	chr11	95882497	CTAAAATA	CTAAAA	MTMR2	141	0.727	INDEL
.	chr11	95882497	CTAAAATA	C	MTMR2	2	0.01	INDEL
.	chr11	95882497	CTAAAATA	CTAAAATA	MTMR2	5	0.026	INDEL
.	chr11	95887685	C	CCTCAAAT	MTMR2	2	0.01	INDEL
rs7598603	chr11	96512082	T	C	RP11-49K4	29	0.149	SNP
rs1229265	chr11	99819645	C	T	CNTN5	54	0.278	SNP
.	chr11	99819753	T	TGAGGAAC	CNTN5	2	0.01	INDEL
rs500760	chr11	1.01E+08	T	C	PGR	34	0.175	SNP
rs3523146	chr11	1.03E+08	G	A	MMP8	5	0.026	SNP
rs1786095	chr11	1.03E+08	T	C	MMP10	4	0.021	SNP
rs1786095	chr11	1.03E+08	A	G	MMP10	15	0.077	SNP
rs1786094	chr11	1.03E+08	G	A	MMP10	20	0.103	SNP
rs1051121	chr11	1.03E+08	C	T	MMP1	5	0.026	SNP
rs1787974	chr11	1.03E+08	CA	C	MMP1	7	0.036	INDEL
rs1383143	chr11	1.05E+08	G	A	CASP1	3	0.015	SNP
rs3518804	chr11	1.08E+08	C	A	ACAT1	32	0.165	SNP
.	chr11	1.08E+08	CTT	CT	ATM	8	0.041	INDEL
.	chr11	1.08E+08	CTT	CTTT	ATM	89	0.459	INDEL
.	chr11	1.08E+08	CTT	C	ATM	2	0.01	INDEL
rs7105857	chr11	1.11E+08	C	T	COLCA2	132	0.68	SNP
.	chr11	1.12E+08	CACCGCCA	CCCGCCA	SIK2	2	0.01	INDEL
.	chr11	1.12E+08	CAG	C	SIK2	2	0.01	INDEL
rs5903886	chr11	1.12E+08	G	A	C11orf57	2	0.01	SNP
rs584427	chr11	1.13E+08	T	G	NCAM1	79	0.407	SNP
rs2236709	chr11	1.13E+08	G	A	TTC12	142	0.732	SNP
rs1078994	chr11	1.13E+08	A	G	TTC12	98	0.505	SNP
rs1156968	chr11	1.14E+08	T	C	NNMT	2	0.01	SNP
rs1121498	chr11	1.14E+08	C	T	RBM7	35	0.18	SNP
.	chr11	1.17E+08	T	TTATA	AP000936.	10	0.052	INDEL
.	chr11	1.17E+08	T	TTATATA	AP000936.	6	0.031	INDEL
.	chr11	1.18E+08	TGG	*	TMPRSS13	140	0.722	INDEL

.	chr11	1.18E+08	TGG	T	TMPRSS13	2	0.01	INDEL
.	chr11	1.18E+08	AGATGCCT	*	TMPRSS13	140	0.722	INDEL
.	chr11	1.18E+08	AGATGCCT	A	TMPRSS13	2	0.01	INDEL
rs1125705	chr11	1.18E+08	C	T	AP002962.	7	0.036	SNP
rs2276122	chr11	1.18E+08	A	G	TMPRSS4	79	0.407	SNP
585268	chr11	1.18E+08	CT	C	MPZL2	2	0.01	INDEL
rs1418379	chr11	1.19E+08	CAGTGTGG	C	TMEM25	21	0.108	INDEL
rs5696611	chr11	1.19E+08	AC	A	SLC37A4	194	1	INDEL
.	chr11	1.19E+08	CACCAGAC	C	C2CD2L	2	0.01	INDEL
rs4245191	chr11	1.19E+08	C	A	NLRX1	96	0.495	SNP
rs2227987;	chr11	1.19E+08	T	C	CBL	3	0.015	SNP
rs2241646	chr11	1.19E+08	G	A	USP2	125	0.644	SNP
.	chr11	1.2E+08	GGGCCGG(	G	TRIM29	4	0.021	INDEL
.	chr11	1.2E+08	G	*	TRIM29	3	0.015	SNP
rs1236498	chr11	1.21E+08	T	C	SORL1	109	0.562	SNP
rs1484304	chr11	1.22E+08	G	A	SORL1	3	0.015	SNP
.	chr11	1.23E+08	G	T	CTD-2216M	2	0.01	SNP
.	chr11	1.23E+08	A	G	GRAMD1B	3	0.015	SNP
rs2512227	chr11	1.24E+08	T	G	OR10D3	105	0.541	SNP
.	chr11	1.24E+08	C	G	OR8G1	86	0.443	SNP
rs2016614	chr11	1.24E+08	A	AG	OR8B3	14	0.072	INDEL
rs6191062	chr11	1.25E+08	C	T	ESAM	3	0.015	SNP
rs558814	chr11	1.25E+08	A	G	RP11-677M	56	0.289	SNP
rs3433203	chr11	1.25E+08	C	T	PKNOX2	44	0.227	SNP
rs3443509	chr11	1.25E+08	G	A	FEZ1	78	0.402	SNP
.	chr11	1.26E+08	G	A	CHEK1	3	0.015	SNP
.	chr11	1.26E+08	A	AT	CHEK1	3	0.015	INDEL
rs1122023	chr11	1.26E+08	A	C	PATE4	127	0.655	SNP
rs1155332	chr11	1.26E+08	T	C	CDON	5	0.026	SNP
rs8177374;	chr11	1.26E+08	C	T	TIRAP	27	0.139	SNP
.	chr11	1.26E+08	CTT	C	TIRAP	6	0.031	INDEL
.	chr11	1.26E+08	CTT	CT	TIRAP	27	0.139	INDEL
.	chr11	1.26E+08	CTT	CTTT	TIRAP	11	0.057	INDEL
rs2010955	chr11	1.26E+08	C	CTGGGGA	DCPS	3	0.015	INDEL
rs1137729	chr11	1.27E+08	G	GC	KIRREL3-AS	194	1	INDEL
rs2230004	chr11	1.28E+08	T	C	ETS1	99	0.51	SNP
rs1401917	chr11	1.29E+08	G	T	TP53AIP1	2	0.01	SNP
.	chr11	1.3E+08	AT	A	APLP2	12	0.062	INDEL
.	chr11	1.3E+08	AT	ATTTT	APLP2	10	0.052	INDEL
.	chr11	1.3E+08	AT	ATT	APLP2	9	0.046	INDEL
.	chr11	1.3E+08	CTTTTTTTT	CTTTTTTTT	RP11-567O	10	0.052	MIXED
.	chr11	1.3E+08	CTTTTTTTT	CTTTTTTTT	RP11-567O	16	0.083	MIXED
rs1160249	chr11	1.31E+08	A	G	RP11-890B	37	0.191	SNP
rs6590557	chr11	1.31E+08	G	A	AP002856.	87	0.448	SNP
rs4436560	chr11	1.35E+08	A	C	AP005135.	25	0.129	SNP
rs216252	chr12	203267	G	A	SLC6A12	96	0.495	SNP
.	chr12	259833	CA	C	SLC6A13	2	0.01	INDEL

rs7144749	chr12	555328	G	A	B4GALNT3	21	0.108	SNP
rs1418234	chr12	865142	T	TC	WNK1	66	0.34	INDEL
rs1144189	chr12	865189	T	TC	WNK1	104	0.536	INDEL
.	chr12	865231	T	TC	WNK1	2	0.01	INDEL
rs4987208;	chr12	913403	A	C	RAD52	4	0.021	SNP
rs4987207	chr12	914052	G	T	RAD52	4	0.021	SNP
rs3527821	chr12	929812	C	CT	RAD52	23	0.119	INDEL
rs215994;7	chr12	2607994	T	C	CACNA1C	44	0.227	SNP
rs5619686	chr12	2799164	C	A	FKBP4	6	0.031	SNP
rs4766003	chr12	2878114	G	A	RHNO1	74	0.381	SNP
.	chr12	2959481	GGTGAGA	G	TEAD4	6	0.031	INDEL
.	chr12	3540624	CAG	C	PRMT8	2	0.01	INDEL
rs2001787	chr12	4626799	T	TC	AKAP3	17	0.088	INDEL
rs6751258	chr12	4626803	GA	G	AKAP3	17	0.088	INDEL
rs3485528	chr12	4850738	G	GA	GALNT8	112	0.577	INDEL
rs216310;2	chr12	6018777	A	G	VWF	97	0.5	SNP
rs1106406	chr12	6160496	C	T	RP3-454B2	127	0.655	SNP
.	chr12	6327491	CGA	C	PLEKHG6	2	0.01	INDEL
rs1065691	chr12	6537154	T	C	GAPDH	41	0.211	SNP
rs1479709	chr12	6537922	C	T	GAPDH	2	0.01	SNP
rs1803621	chr12	6537943	T	C	GAPDH	41	0.211	SNP
.	chr12	6834938	CA	CAA	P3H3	8	0.041	INDEL
.	chr12	6834938	CA	C	P3H3	30	0.155	INDEL
rs1491459	chr12	6856096	C	T	USP5	2	0.01	SNP
rs7266110	chr12	6870035	G	A	TPI1	2	0.01	SNP
rs1106443	chr12	6872998	C	T	SPSB2	2	0.01	SNP
rs1890021	chr12	6873638	G	A	SPSB2	4	0.021	SNP
rs2110072	chr12	6956472	T	C	PTPN6	182	0.938	SNP
.	chr12	6981836	ATG	A	LPCAT3	5	0.026	INDEL
rs3752772	chr12	7108113	G	C	C1RL-AS1	18	0.093	SNP
rs7485773	chr12	7322485	C	T	ACSM4	7	0.036	SNP
rs1484149	chr12	7368967	TGA	T	CD163L1	13	0.067	INDEL
rs1484149	chr12	7368967	TGA	TGAGAGA	CD163L1	5	0.026	INDEL
rs3427547	chr12	7652818	C	T	APOBEC1	2	0.01	SNP
.	chr12	7795089	CGT	C	NANOG	7	0.036	INDEL
.	chr12	8224771	TG	T	FAM90A1	6	0.031	INDEL
rs2968742	chr12	8234275	C	G	FAM86FP	152	0.784	SNP
rs1161187	chr12	8648486	C	A	MFAP5	15	0.077	SNP
rs1998714	chr12	8788704	AAGCGGTG	A	RP11-20D1	154	0.794	INDEL
rs1169126	chr12	8788762	C	T	RP11-20D1	4	0.021	SNP
rs1427948	chr12	8857942	G	GT	A2ML1	2	0.01	INDEL
rs1446863	chr12	8863966	GGC	G	A2ML1	17	0.088	INDEL
rs1799759;	chr12	9093580	CTATGG	C	A2M	25	0.129	INDEL
rs1178897	chr12	9181030	G	A	PZP	2	0.01	SNP
rs1122156	chr12	9253604	C	T	A2MP1	25	0.129	SNP
rs5591353	chr12	9270369	C	A	A2MP1	62	0.32	SNP
rs7302081	chr12	9648330	C	T	RP11-705C	51	0.263	SNP

rs3564290!	chr12	9653809	G	A	RP11-705C	20	0.103	SNP
rs3536455!	chr12	9656549	G	GT	RP11-705C	62	0.32	INDEL
rs3536455!	chr12	9656549	G	GTT	RP11-705C	5	0.026	INDEL
rs1132226:	chr12	9733111	A	ATAAGT	CLECL1	102	0.526	INDEL
rs1691052(	chr12	10118488	A	C	CLEC7A	9	0.046	SNP
rs2418102	chr12	10703620	C	T	YBX3	50	0.258	SNP
rs2302685	chr12	12148964	C	T	LRP6	158	0.814	SNP
rs1008283	chr12	12181072	G	C	LRP6	22	0.113	SNP
.	chr12	12330483	C	CAA	MANSC1	2	0.01	INDEL
.	chr12	12330484	G	GA	MANSC1	2	0.01	INDEL
.	chr12	12615499	C	CT	RPL21P136	10	0.052	INDEL
rs2021164:	chr12	12726097	G	A	APOLD1	2	0.01	SNP
rs1161297!	chr12	12820780	C	T	DDX47	37	0.191	SNP
rs1415090!	chr12	13087228	G	A	GSG1	7	0.036	SNP
.	chr12	13376513	C	A	LINC01559	3	0.015	SNP
rs1055601(	chr12	14823483	CTA	C	C12orf60	33	0.17	INDEL
rs7998939!	chr12	14917253	T	C	ERP27	2	0.01	SNP
rs3748299	chr12	15580735	G	A	PTPRO	20	0.103	SNP
rs1873520(	chr12	18562893	G	A	PIK3C2G	2	0.01	SNP
rs6175771!	chr12	18648027	G	C	PIK3C2G	7	0.036	SNP
712105	chr12	20855146	T	TAATTG	SLCO1B3	4	0.021	INDEL
.	chr12	21306938	CTGTT	C	SLCO1A2	4	0.021	INDEL
rs1104605!	chr12	21447705	T	G	TCEB1P31	105	0.541	SNP
rs7199449!	chr12	26326465	GATGACTT	G	RP11-283G	23	0.119	INDEL
rs5919936!	chr12	26681871	T	TACTC	ITPR2	114	0.588	INDEL
rs1755206(	chr12	27710598	T	C	RP11-1060.	7	0.036	SNP
rs1133028	chr12	28450179	A	G	CCDC91	2	0.01	SNP
rs53024	chr12	29521087	G	T	RP11-310I2	135	0.696	SNP
.	chr12	30754687	A	T	CAPRIN2	4	0.021	SNP
rs3414357:	chr12	30798125	AT	A	LINC00941	29	0.149	INDEL
rs3414357:	chr12	30798125	AT	ATT	LINC00941	12	0.062	INDEL
rs1177755:	chr12	30995986	C	T	RP11-551L:	3	0.015	SNP
rs1077180:	chr12	31102329	G	A	DDX11	90	0.464	SNP
rs7496397!	chr12	38084087	G	A	CLUHP8	14	0.072	SNP
rs1118221	chr12	38086228	G	A	CLUHP8	71	0.366	SNP
rs1117657!	chr12	40426406	G	A	MUC19	86	0.443	SNP
rs1156423(	chr12	40443462	T	C	MUC19	20	0.103	SNP
rs1494001:	chr12	40516466	AG	A	MUC19	8	0.041	INDEL
rs7668401!	chr12	40537853	G	A	MUC19	36	0.186	SNP
.	chr12	42374866	CATG	C	PPHLN1	2	0.01	INDEL
rs1175214:	chr12	42589597	C	T	PRICKLE1	12	0.062	SNP
.	chr12	45554579	AACTGGAA	A	U6	106	0.546	INDEL
rs1508945!	chr12	45776079	A	G	ARID2	2	0.01	SNP
.	chr12	47793367	A	C	HDAC7	7	0.036	SNP
rs2228570;	chr12	47879112	A	G	VDR	123	0.634	SNP
rs1116835!	chr12	48017195	G	A	RP1-228P1	40	0.206	SNP
rs1492277(	chr12	48125405	C	T	PFKM	3	0.015	SNP

rs7965021	chr12	48203092	C	CA	OR10AD1	40	0.206	INDEL
.	chr12	48357431	CTT	CT	RP11-370I1	27	0.139	INDEL
.	chr12	48357431	CTT	CTTT	RP11-370I1	11	0.057	INDEL
.	chr12	48357431	CTT	C	RP11-370I1	16	0.082	INDEL
rs1172708	chr12	48727560	G	A	LINC00935	5	0.026	SNP
rs1125629	chr12	48923041	G	A	FKBP11	19	0.098	SNP
rs1175279	chr12	49091150	G	A	DHH	2	0.01	SNP
rs385988	chr12	49929499	G	A	RP11-70F1	4	0.021	SNP
rs426496;5	chr12	49954295	T	C	AQP2	160	0.825	SNP
.	chr12	50352078	T	TGA	FAM186A	3	0.015	INDEL
rs1231733	chr12	50352080	C	G	FAM186A	22	0.224	MIXED
rs1231733	chr12	50352080	C	CCTGCTGA	FAM186A	3	0.031	MIXED
rs224590	chr12	51006214	A	G	SLC11A2	186	0.959	SNP
rs1786036	chr12	51329814	A	AG	CELA1	33	0.17	INDEL
.	chr12	51346623	CATAA	C	CELA1	53	0.273	INDEL
.	chr12	51346631	A	AAG	CELA1	53	0.273	INDEL
.	chr12	51346632	C	CG	CELA1	53	0.273	INDEL
rs7303111	chr12	51890490	G	A	ANKRD33	51	0.263	SNP
rs1870218	chr12	52258169	C	A	KRT87P	10	0.052	SNP
rs2857667;	chr12	52317765	G	A	KRT83	68	0.351	SNP
rs2857667;	chr12	52317765	G	T	KRT83	6	0.031	SNP
rs1467534	chr12	52317963	C	A	KRT83	4	0.021	SNP
rs1498688	chr12	52773632	C	A	KRT76	5	0.026	SNP
rs1406537	chr12	52790835	T	TA	KRT3	17	0.088	INDEL
rs1179282	chr12	53063801	C	T	TNS2	2	0.01	SNP
rs1480369	chr12	53160142	C	T	CSAD	3	0.015	SNP
.	chr12	53446049	AT	A	PRR13	4	0.021	INDEL
rs1172475	chr12	53493697	A	T	MAP3K12	6	0.031	SNP
.	chr12	54325081	A	G	COPZ1	2	0.01	SNP
rs1117087	chr12	54340505	A	G	COPZ1	21	0.108	SNP
rs1460668	chr12	54472135	GCAGGTTA	G	GTSF1	31	0.16	INDEL
rs1249950	chr12	54576670	T	C	PDE1B	93	0.479	SNP
rs5798318	chr12	54646033	TG	T	DCD	45	0.232	INDEL
rs1117101	chr12	54687078	A	G	RP11-850F	140	0.722	SNP
rs1801876	chr12	54950240	G	A	TESPA1	11	0.057	SNP
rs5803602	chr12	55129802	AT	A	OR9K2	64	0.33	INDEL
rs4522268	chr12	55247471	C	T	OR6C74	63	0.325	SNP
rs5798345	chr12	55320622	C	CA	OR6C1	76	0.392	INDEL
rs5738718	chr12	55427174	CA	C	OR6C76	43	0.222	INDEL
rs2069398	chr12	55967092	G	A	CDK2	10	0.052	SNP
rs703822	chr12	56206744	C	T	RNF41	5	0.026	SNP
rs6054295	chr12	56267121	G	T	COQ10A	33	0.17	SNP
rs3747102	chr12	56686880	CAT	C	PTGES3	143	0.737	INDEL
rs1131514	chr12	56739356	T	G	PRIM1	143	0.737	SNP
rs3725811	chr12	56997074	AAC	A	GPR182	16	0.082	INDEL
.	chr12	56997398	G	A	GPR182	3	0.015	SNP
rs3825080	chr12	57477772	A	G	ARHGAP9	165	0.851	SNP

rs774895	chr12	57625689	T	G	SLC26A10	194	1	SNP
rs1112998	chr12	57780492	T	C	METTL21B	6	0.031	SNP
rs2014886	chr12	57783654	C	T	RP11-571M	75	0.387	SNP
rs1113844	chr12	58616933	A	G	RP11-767I2	129	0.665	SNP
rs3516646	chr12	59768160	G	GA	SLC16A7	49	0.253	INDEL
rs7107760	chr12	60095105	C	CTTT	RP11-316A	5	0.028	INDEL
rs7107760	chr12	60095105	C	CTT	RP11-316A	95	0.534	INDEL
rs7107760	chr12	60095105	C	CT	RP11-316A	31	0.174	INDEL
rs5798648	chr12	62578430	G	GT	MON2	63	0.325	INDEL
rs3864455	chr12	63724778	A	G	RP11-415I1	98	0.505	SNP
rs7216152	chr12	64097222	GT	G	SRGAP1	10	0.052	INDEL
rs7216152	chr12	64097222	GT	GTT	SRGAP1	31	0.16	INDEL
.	chr12	65019331	GT	GTT	RP11-168J1	38	0.196	INDEL
.	chr12	65019331	GT	G	RP11-168J1	2	0.01	INDEL
rs3410581	chr12	65068843	A	G	WIF1	2	0.01	SNP
rs1996726	chr12	66310493	AG	A	HELB	9	0.046	INDEL
rs490872	chr12	69252084	A	G	CPSF6	141	0.727	SNP
rs2305641	chr12	69253134	G	A	CPSF6	86	0.443	SNP
.	chr12	69694439	GT	G	BEST3	11	0.057	INDEL
rs1161106	chr12	69739168	C	T	RAB3IP	84	0.433	SNP
rs5935962	chr12	70285384	AT	A	CNOT2	58	0.299	INDEL
rs5935962	chr12	70285384	AT	ATT	CNOT2	2	0.01	INDEL
rs3752703	chr12	70534836	G	A	PTPRB	56	0.289	SNP
rs582954	chr12	70534944	A	G	PTPRB	183	0.943	SNP
rs1173243	chr12	71440257	G	A	LGR5	4	0.021	SNP
rs1175351	chr12	71556627	A	G	LGR5	4	0.021	SNP
rs1797372	chr12	71566428	G	A	LGR5	191	0.985	SNP
rs1087929	chr12	71567316	G	C	LGR5	3	0.015	SNP
rs1382155	chr12	71577986	C	G	LGR5	2	0.01	SNP
rs1461101	chr12	71578805	G	A	LGR5	2	0.01	SNP
rs4290270;	chr12	72022455	A	T	TPH2	121	0.624	SNP
rs4760757	chr12	72309699	A	G	TRHDE	98	0.505	SNP
.	chr12	75410481	AT	A	GLIPR1L2	2	0.01	INDEL
rs2018294	chr12	77947410	AT	A	NAV3	7	0.036	INDEL
rs2018294	chr12	77947410	AT	ATT	NAV3	53	0.273	INDEL
rs2018294	chr12	77947410	AT	ATTTT	NAV3	2	0.01	INDEL
rs2018294	chr12	77947410	AT	ATTT	NAV3	8	0.041	INDEL
rs1367352	chr12	78465516	G	A	RP11-754N	73	0.376	SNP
rs3529258	chr12	78955567	G	A	SYT1	19	0.098	SNP
rs1281291	chr12	79448929	G	T	SYT1	18	0.093	SNP
rs5799725	chr12	85244867	GA	G	LRRIQ1	66	0.34	INDEL
.	chr12	89013317	TC	T	RP11-13A1	2	0.01	INDEL
rs808820	chr12	89351049	A	G	DUSP6	25	0.129	SNP
rs6028377	chr12	93106283	TTTTTG	T	RP11-202G	40	0.206	INDEL
rs6028377	chr12	93106283	TTTTTG	TTTTTGTTT	RP11-202G	8	0.041	INDEL
rs7976742	chr12	93328025	A	G	RP11-486A	86	0.443	SNP
.	chr12	93501029	G	GTTTTACT	MRPL42	7	0.036	INDEL

.	chr12	93501029	G	GTTTTACTA	MRPL42	5	0.026	INDEL
.	chr12	93837277	G	GT	RP11-887P	6	0.031	INDEL
rs2230754	chr12	94149730	C	T	PLXNC1	60	0.309	SNP
rs6764831	chr12	94303749	T	C	PLXNC1	35	0.18	MIXED
rs6764831	chr12	94303749	T	TC	PLXNC1	7	0.036	MIXED
rs7336873	chr12	94928016	C	T	NDUFA12	19	0.098	SNP
rs159853	chr12	95436562	C	T	RP11-167N	76	0.392	SNP
rs2305293	chr12	95485958	C	T	METAP2	9	0.046	SNP
rs3794261	chr12	95512827	C	A	METAP2	19	0.098	SNP
rs1110910	chr12	97564330	T	C	RMST	134	0.691	SNP
rs1111026	chr12	1E+08	T	C	GOLGA2P5	14	0.072	SNP
rs1697479	chr12	1.02E+08	A	G	MYBPC1	32	0.165	SNP
rs1544921	chr12	1.02E+08	G	C	CHPT1	110	0.567	SNP
rs1475725	chr12	1.02E+08	TTGAC	T	CHPT1	11	0.057	INDEL
rs1042503	chr12	1.03E+08	C	T	PAH	51	0.263	SNP
.	chr12	1.03E+08	CGCAGCAG	CGCAGCAG	ASCL1&PAI	62	0.32	INDEL
.	chr12	1.03E+08	CGCAGCAG	C	ASCL1&PAI	2	0.01	INDEL
.	chr12	1.03E+08	CGCAGCAG	CGCAGCAG	ASCL1&PAI	2	0.01	INDEL
.	chr12	1.03E+08	CGCAGCAG	CGCAGCAG	ASCL1&PAI	2	0.01	INDEL
rs1161342	chr12	1.04E+08	A	G	TTC41P	97	0.5	SNP
.	chr12	1.04E+08	G	GA	TDG	5	0.026	INDEL
.	chr12	1.04E+08	T	G	TDG	5	0.026	SNP
.	chr12	1.04E+08	A	ATT	TDG	6	0.031	INDEL
.	chr12	1.04E+08	A	ATTGAGAG	TDG	12	0.062	INDEL
.	chr12	1.04E+08	A	ATTGAGAG	TDG	3	0.015	INDEL
.	chr12	1.04E+08	G	GAGAGCGT	TDG	5	0.026	MIXED
.	chr12	1.04E+08	G	T	TDG	7	0.036	MIXED
rs1406966	chr12	1.04E+08	G	T	HCFC2	7	0.036	SNP
.	chr12	1.06E+08	CTT	C	POLR3B	5	0.026	INDEL
.	chr12	1.06E+08	CTT	CT	POLR3B	49	0.253	INDEL
.	chr12	1.06E+08	CTT	CTTT	POLR3B	52	0.268	INDEL
rs2015138	chr12	1.08E+08	AG	A	ASCL4	3	0.015	INDEL
rs7724167	chr12	1.09E+08	A	C	FICD	4	0.021	SNP
rs6374899	chr12	1.09E+08	GGAGTGGT	G	SELPLG	50	0.258	INDEL
rs7980427	chr12	1.09E+08	G	A	DAO	17	0.088	SNP
rs3396231	chr12	1.09E+08	C	T	ALKBH2	4	0.021	SNP
rs1106577	chr12	1.09E+08	T	C	ACACB	175	0.902	SNP
rs2300455	chr12	1.09E+08	G	A	ACACB	52	0.268	SNP
rs2075438	chr12	1.09E+08	G	A	FOXN4	76	0.392	SNP
.	chr12	1.09E+08	G	A	UBE3B	2	0.01	SNP
rs7957619	chr12	1.1E+08	G	A	MVK	41	0.211	SNP
rs3436809	chr12	1.1E+08	G	A	MVK	28	0.144	SNP
rs1344554	chr12	1.1E+08	G	A	TRPV4	10	0.052	SNP
rs3742034	chr12	1.1E+08	A	G	TRPV4	18	0.093	SNP
rs1920855	chr12	1.1E+08	C	A	ANAPC7	2	0.01	SNP
rs6174807	chr12	1.11E+08	C	T	PPP1CC	3	0.015	SNP
.	chr12	1.11E+08	GA	G	CCDC63	3	0.015	INDEL

.	chr12	1.12E+08	GC	G	ATXN2	3	0.015	INDEL
.	chr12	1.12E+08	GC	G	ATXN2	3	0.015	INDEL
.	chr12	1.12E+08	TGCTGCTG	T	ATXN2	2	0.01	INDEL
rs1258024(	chr12	1.12E+08	G	A	MAPKAPK5	18	0.093	SNP
rs1106611.	chr12	1.12E+08	C	T	TMEM116	42	0.216	SNP
rs1077467.	chr12	1.13E+08	G	A	OAS1	129	0.665	SNP
rs1135283!	chr12	1.13E+08	TA	T	OAS1	127	0.655	INDEL
rs1281976.	chr12	1.13E+08	C	T	OAS3	4	0.021	SNP
rs6194223.	chr12	1.13E+08	C	T	OAS3	2	0.01	SNP
rs15895	chr12	1.13E+08	A	G	OAS2	138	0.711	SNP
rs1379863	chr12	1.13E+08	C	T	DTX1	186	0.959	SNP
rs1005225	chr12	1.14E+08	G	C	RP11-100F	21	0.108	SNP
rs3736839.	chr12	1.17E+08	GGAAA	G	RNFT2	24	0.124	INDEL
.	chr12	1.17E+08	T	TG	RNFT2	2	0.01	INDEL
rs9658356;	chr12	1.17E+08	T	G	NOS1	2	0.01	SNP
rs1085092.	chr12	1.18E+08	G	A	RP11-465L!	41	0.211	SNP
rs5810589.	chr12	1.2E+08	AGGCACCT	A	PRKAB1	16	0.082	INDEL
rs1171920.	chr12	1.21E+08	G	A	COQ5	3	0.015	SNP
rs2018808.	chr12	1.21E+08	AGTGC	A	CABP1	23	0.119	INDEL
.	chr12	1.21E+08	CGTGCGTG	CGT	CABP1	101	0.521	INDEL
rs2002015.	chr12	1.21E+08	TGC	*	CABP1	102	0.526	INDEL
rs2002015.	chr12	1.21E+08	TGC	T	CABP1	12	0.062	INDEL
.	chr12	1.21E+08	TGTGC	T	CABP1	6	0.031	INDEL
rs3914;136	chr12	1.21E+08	T	C	ACADS	85	0.438	SNP
rs7654364(	chr12	1.21E+08	C	T	ACADS	4	0.021	SNP
rs3213545	chr12	1.21E+08	G	A	OASL	61	0.314	SNP
.	chr12	1.21E+08	CT	C	CAMKK2	7	0.036	INDEL
.	chr12	1.21E+08	CT	CTTT	CAMKK2	3	0.015	INDEL
.	chr12	1.21E+08	CT	CTTTT	CAMKK2	56	0.289	INDEL
.	chr12	1.21E+08	CT	CTTTTT	CAMKK2	13	0.067	INDEL
rs7962143	chr12	1.21E+08	G	T	RP11-711D	74	0.381	SNP
777690	chr12	1.22E+08	CTG	C	KDM2B	3	0.015	INDEL
.	chr12	1.22E+08	CGG	C	SETD1B	2	0.01	MIXED
.	chr12	1.22E+08	T	TC	MLXIP	3	0.015	INDEL
rs1074415!	chr12	1.22E+08	C	T	RP11-512M	194	1	SNP
rs1394457(	chr12	1.22E+08	T	C	CLIP1	3	0.015	SNP
rs3603730.	chr12	1.24E+08	AC	A	CCDC92	22	0.113	INDEL
.	chr12	1.24E+08	G	GACCT	ZNF664	6	0.031	INDEL
.	chr12	1.24E+08	G	GACCTGCA	ZNF664	3	0.015	INDEL
.	chr12	1.24E+08	G	GACCTGCA	ZNF664	2	0.01	INDEL
.	chr12	1.24E+08	G	GCAAATCC	ZNF664	5	0.026	INDEL
.	chr12	1.24E+08	AAGGTTTG	A	ZNF664	32	0.165	INDEL
rs1084678.	chr12	1.25E+08	G	A	DHX37	8	0.041	SNP
rs1871413(	chr12	1.27E+08	T	C	LINC00943	2	0.01	SNP
rs3546127(	chr12	1.32E+08	C	T	PUS1	2	0.01	SNP
.	chr12	1.32E+08	CAG	C	EP400	2	0.01	INDEL
.	chr12	1.32E+08	CAGCCGCA	C	EP400	2	0.01	INDEL

rs3689856	chr12	1.32E+08	TC	T	GALNT9	24	0.124	INDEL
rs2018248	chr12	1.32E+08	GC	G	GALNT9	18	0.093	INDEL
.	chr12	1.33E+08	G	GC	FBRSL1	5	0.026	INDEL
.	chr12	1.33E+08	GCCACC	G	PXMP2	2	0.01	INDEL
rs7958567	chr12	1.33E+08	T	C	PXMP2	186	0.959	SNP
rs6195282	chr12	1.33E+08	T	C	CHFR	50	0.258	SNP
.	chr13	18261323	A	AC	GGT4P	2	0.01	INDEL
rs5635312	chr13	18954927	C	A	PHF2P2	23	0.119	SNP
rs7342580	chr13	19026389	G	A	GTF2IP3	6	0.031	SNP
rs2343712	chr13	19028574	G	A	RP11-301J	130	0.67	SNP
rs1121305	chr13	19204338	A	G	PSPC1P1	14	0.072	SNP
rs1115654	chr13	19290752	T	C	ANKRD26P	10	0.052	SNP
rs5586001	chr13	19366574	T	G	PARP4P2	83	0.428	SNP
rs1442765	chr13	19381178	TAGAC	T	PARP4P2	6	0.031	INDEL
rs2497213	chr13	19425773	G	A	TPTE2	27	0.139	SNP
rs2003538	chr13	19425814	T	TGCGAA	TPTE2	147	0.758	INDEL
rs8033893	chr13	20189546	AC	A	GJB2	3	0.015	INDEL
rs14236	chr13	20489385	A	G	CRYL1	37	0.191	SNP
.	chr13	20704042	G	GGGCCGCC	IL17D	3	0.015	INDEL
.	chr13	21155148	T	TTTCAGTT	SKA3	5	0.026	INDEL
.	chr13	21155151	C	CAGTTTTC	SKA3	8	0.041	INDEL
.	chr13	21155151	C	CAGTTTTC	SKA3	3	0.015	INDEL
.	chr13	21155151	C	CAGTTTTC	SKA3	4	0.021	INDEL
.	chr13	21158125	C	CCAAAG	SKA3	11	0.057	INDEL
.	chr13	21158126	C	CAAAGCTA	SKA3	4	0.021	INDEL
.	chr13	21159987	T	TCA	SKA3	6	0.031	INDEL
.	chr13	21159988	C	CACTTTTTT	SKA3	3	0.015	INDEL
rs1512722	chr13	21172461	TG	T	SKA3	28	0.144	INDEL
.	chr13	21172504	C	CCG	SKA3	5	0.026	INDEL
.	chr13	21172505	C	CGCTTTCC	SKA3	3	0.015	INDEL
rs6195035	chr13	21176399	G	A	SKA3	25	0.129	SNP
.	chr13	21377196	GGCCCGAC	G	ZDHHC20-I	3	0.015	INDEL
rs7992360	chr13	22670991	T	C	AL354828.	2	0.01	SNP
rs3585625	chr13	22676877	CT	C	AL354828.	188	0.969	INDEL
rs1129555	chr13	23897001	A	ACT	C1QTNF9B	2	0.01	INDEL
rs1493671	chr13	25258693	CTAAAAT	C	MTMR6	11	0.057	INDEL
rs4769468	chr13	26118923	C	T	PRUNEP1	160	0.825	SNP
rs9507748	chr13	26619456	C	G	WASF3-AS	70	0.361	SNP
rs3485660	chr13	27203206	C	CTAAA	RP11-428O	168	0.866	INDEL
rs7108688	chr13	28723368	CT	C	CYP51A1P	112	0.577	INDEL
rs1285747	chr13	30956872	G	A	TEX26	75	0.387	SNP
rs7350700	chr13	31067428	T	C	WDR95P	99	0.51	SNP
rs3410552	chr13	31077310	TCTGC	T	WDR95P	162	0.835	INDEL
rs1409048	chr13	31786554	T	TAAA	RXFP2	19	0.098	INDEL
rs1409048	chr13	31786554	T	TA	RXFP2	23	0.119	INDEL
rs1409048	chr13	31786554	T	TAA	RXFP2	63	0.325	INDEL
.	chr13	32395960	CT	CTT	BRCA2	9	0.07	INDEL

.	chr13	32395960	CT	C	BRCA2	6	0.047	INDEL
rs1157183:	chr13	32398489	A	T	BRCA2	2	0.01	SNP
rs206321	chr13	32416643	A	C	N4BP2L1	32	0.165	SNP
rs3766984!	chr13	32758070	CT	C	PDS5B	12	0.062	INDEL
rs3766984!	chr13	32758070	CT	CTT	PDS5B	9	0.046	INDEL
rs9315629	chr13	38923743	G	A	ANKRD26P	185	0.954	SNP
rs1141475:	chr13	39679963	A	AT	COG6	42	0.216	INDEL
rs3693226	chr13	42159244	C	CTTTTTTTT	DGKH	2	0.016	INDEL
rs3693226	chr13	42159244	C	CTTTTTTTT	DGKH	13	0.107	INDEL
rs3693226	chr13	42159244	C	CTTTTTTTT	DGKH	18	0.148	INDEL
rs3693226	chr13	42159244	C	CTTTTTTTT	DGKH	10	0.082	INDEL
rs3693226	chr13	42159244	C	CTTTTTTT	DGKH	7	0.057	INDEL
rs3693226	chr13	42159244	C	CTTTTTTTT	DGKH	6	0.049	INDEL
rs6009522(	chr13	42195961	T	C	DGKH	22	0.113	SNP
rs9562415;	chr13	42606888	T	C	TNFSF11	3	0.015	SNP
rs6138934(	chr13	42888298	T	TTCAGG	EPSTI1	4	0.021	INDEL
rs2234216	chr13	45340822	T	C	TPT1	117	0.603	SNP
rs7997422	chr13	45351896	G	A	TPT1-AS1	6	0.031	SNP
rs8007237:	chr13	45713238	C	A	SPERT	12	0.062	SNP
rs7970784:	chr13	45713882	C	A	SPERT	15	0.077	SNP
rs9316179	chr13	46067331	A	G	CPB2	68	0.351	SNP
rs1400996!	chr13	48093561	CAT	C	MED4	4	0.021	INDEL
rs3727637	chr13	49220340	G	A	MLNR	4	0.021	SNP
rs3430134	chr13	49630893	G	A	ARL11	7	0.036	SNP
rs6747325(	chr13	50527437	ACTGAAAT	A	DLEU1&DL	33	0.17	INDEL
rs9526704	chr13	50910481	A	T	RNASEH2B-	62	0.32	SNP
rs2003207:	chr13	50956450	T	TA	RNASEH2B	4	0.021	INDEL
rs1286826:	chr13	51080705	A	T	GUCY1B2	41	0.211	SNP
.	chr13	51461948	CT	C	INTS6-AS1	34	0.175	INDEL
.	chr13	51461948	CT	CTT	INTS6-AS1	10	0.052	INDEL
rs9568618	chr13	51515647	A	G	INTS6-AS1	147	0.758	SNP
.	chr13	52143914	CT	C	NEK3	66	0.34	INDEL
.	chr13	52571293	G	A	TPTE2P3	2	0.01	SNP
rs6195807!	chr13	52583187	C	T	TPTE2P3	9	0.046	SNP
rs1128296(	chr13	52643320	GA	G	HNRNPA1L	2	0.01	INDEL
rs2050536	chr13	54120939	A	C	LINC00458	176	0.907	SNP
.	chr13	58206916	CTTTT	CTTT	RP11-538C	63	0.325	INDEL
.	chr13	58206916	CTTTT	CT	RP11-538C	23	0.119	INDEL
.	chr13	58206916	CTTTT	CTT	RP11-538C	70	0.361	INDEL
.	chr13	58206916	CTTTT	CTTTTT	RP11-538C	17	0.088	INDEL
.	chr13	58206916	CTTTT	C	RP11-538C	3	0.015	INDEL
rs1169179:	chr13	71015250	T	C	LINC00348	5	0.026	SNP
rs1008749	chr13	71167684	A	C	LINC00348	13	0.067	SNP
rs1496957	chr13	75549765	CGGCGGC(	C	RP11-29G8	34	0.175	INDEL
rs3543912:	chr13	77017068	CT	C	CLN5	141	0.727	INDEL
rs2013804:	chr13	77698132	T	TGG	SLAIN1	194	1	INDEL
rs7110277:	chr13	77698141	G	GC	SLAIN1	194	1	INDEL

.	chr13	77939994	CTAAA	CTAAATAA	RP11-318G	80	0.412	INDEL
.	chr13	77939994	CTAAA	CTAAATAA	RP11-318G	42	0.216	INDEL
.	chr13	77939994	CTAAA	C	RP11-318G	10	0.052	INDEL
.	chr13	77939994	CTAAA	CTAAATAA	RP11-318G	8	0.041	INDEL
rs6261875	chr13	78605446	T	G	RNF219-AS	4	0.021	SNP
rs7691508	chr13	84562677	G	A	LINC00333	15	0.077	SNP
rs1258608	chr13	86935771	G	A	LINC00430	41	0.211	SNP
.	chr13	94711404	C	CAG	SOX21	2	0.01	INDEL
rs1875326	chr13	94711405	C	G	SOX21	3	0.016	MIXED
rs1875326	chr13	94711405	C	CG	SOX21	2	0.011	MIXED
.	chr13	94711407	C	A	SOX21	2	0.01	MIXED
.	chr13	94711409	G	GA	SOX21	3	0.015	INDEL
rs1457930	chr13	96986746	T	A	OXGR1	3	0.015	SNP
rs5712672	chr13	99864941	CTG	C	CLYBL	29	0.149	INDEL
rs5712672	chr13	99864941	CTG	CTGTG	CLYBL	2	0.01	INDEL
rs4128111	chr13	99866380	C	T	CLYBL	7	0.036	SNP
.	chr13	99982755	CACCACCA	C	ZIC2	2	0.01	INDEL
.	chr13	1E+08	GTTCATTC	GTTCATTC	PCCA&RP1	98	0.505	INDEL
.	chr13	1E+08	GTTCATTC	GTTCA	PCCA&RP1	18	0.093	INDEL
.	chr13	1E+08	GTTCATTC	GTTCATTC	PCCA&RP1	8	0.041	INDEL
.	chr13	1E+08	GTTCATTC	GTTCATTC	PCCA&RP1	5	0.026	INDEL
.	chr13	1E+08	GTTCATTC	G	PCCA&RP1	4	0.021	INDEL
rs660207	chr13	1.03E+08	A	G	METTL21C	81	0.418	SNP
rs1401377	chr13	1.06E+08	G	A	LINC00343	4	0.021	SNP
rs1805386;	chr13	1.08E+08	A	G	LIG4	30	0.155	SNP
rs1805388;	chr13	1.08E+08	G	A	LIG4	40	0.206	SNP
rs881735	chr13	1.08E+08	G	A	RP11-153I2	44	0.227	SNP
rs7642834	chr13	1.11E+08	GGAGTC	G	COL4A2-AS	8	0.041	INDEL
rs7952960	chr13	1.11E+08	C	G	ANKRD10-I	9	0.046	SNP
.	chr13	1.11E+08	AT	ATTT	LINC00368	45	0.232	INDEL
.	chr13	1.11E+08	AT	ATT	LINC00368	81	0.418	INDEL
.	chr13	1.11E+08	AT	A	LINC00368	3	0.015	INDEL
rs5769340	chr13	1.13E+08	TC	T	MCF2L	115	0.593	INDEL
.	chr13	1.13E+08	AG	A	ADPRHL1	7	0.036	INDEL
.	chr13	1.13E+08	CCCGTT	C	ADPRHL1	7	0.036	INDEL
rs7558660	chr13	1.14E+08	G	C	LINC00452	5	0.026	SNP
.	chr13	1.14E+08	A	G	ABC13-474	194	1	SNP
rs2017356	chr14	18601741	AG	A	OR11H12	3	0.015	INDEL
.	chr14	18974742	CT	CTTT	POTEM	15	0.086	INDEL
.	chr14	18974742	CT	C	POTEM	12	0.069	INDEL
.	chr14	18974742	CT	CTT	POTEM	15	0.086	INDEL
rs4410000	chr14	19822253	G	A	OR4N2	2	0.01	SNP
rs3431594	chr14	20002693	CT	C	OR4Q2	77	0.397	INDEL
rs3396569	chr14	20060289	TCATAGAT	T	OR4L1	65	0.335	INDEL
rs5578122	chr14	20198016	C	CA	OR11G2	138	0.711	INDEL
rs1953558	chr14	20230080	T	C	OR11H7	89	0.459	SNP
rs1048945	chr14	20456008	G	C	APEX1	9	0.046	SNP

rs6175283	chr14	20457496	C	T	APEX1	4	0.021	SNP
rs1049562	chr14	20472356	C	T	PNP	37	0.191	SNP
rs1130650	chr14	20472467	C	T	PNP	40	0.206	SNP
rs891297	chr14	20558614	C	T	RNASE9	35	0.18	SNP
rs7573210	chr14	20731742	G	T	EDDM3A	22	0.113	SNP
rs9671404	chr14	20734667	G	C	EDDM3A	16	0.082	SNP
rs1470381	chr14	20990161	CCCCG	C	METTL17	62	0.32	INDEL
rs1489612	chr14	20990192	T	TTG	METTL17	3	0.015	INDEL
rs4981349	chr14	21018228	G	A	NDRG2	6	0.031	SNP
.	chr14	21075702	A	G	ARHGEF40	2	0.01	SNP
rs7141919	chr14	22007907	CA	C	TRAV19	46	0.237	INDEL
rs1711320	chr14	22008151	C	A	TRAV19	4	0.021	SNP
rs2000835	chr14	22124026	G	GC	TRAV26-1	29	0.149	INDEL
.	chr14	22185771	C	G	TRAV32	179	0.923	SNP
rs2242542	chr14	22203081	G	A	TRAV26-2	43	0.222	SNP
710367	chr14	23042460	GC	G	PSMB11	2	0.01	INDEL
rs5733196	chr14	23104903	CA	C	RP11-244E	144	0.742	INDEL
rs5733196	chr14	23104903	CA	*	C14orf119	2	0.01	INDEL
rs5568679	chr14	23104905	GCA	G	RP11-244E	145	0.747	INDEL
rs1718386	chr14	23129767	G	A	SLC7A8	15	0.077	SNP
rs2231124	chr14	23430584	G	A	MYH7	5	0.026	SNP
rs1127279	chr14	23955080	T	C	DHRS4	3	0.015	SNP
rs1214722	chr14	23955158	G	A	DHRS4	12	0.062	SNP
rs7675483	chr14	23965935	C	T	DHRS4	7	0.036	SNP
rs1439735	chr14	23990954	T	C	DHRS4L2	9	0.046	SNP
rs1811890	chr14	24000929	C	T	DHRS4L2	15	0.077	SNP
.	chr14	24001481	T	TA	DHRS4L2	14	0.072	INDEL
rs2273913	chr14	24148029	C	T	RNF31	40	0.206	SNP
rs4546659	chr14	24148333	G	A	RNF31	4	0.021	SNP
.	chr14	24164585	A	G	IRF9	2	0.01	SNP
rs1496439	chr14	24182418	CAG	C	IPO4	20	0.103	INDEL
.	chr14	24183454	A	C	IPO4	4	0.021	SNP
.	chr14	24212554	ATT	AT	CHMP4A	127	0.655	INDEL
.	chr14	24212554	ATT	A	CHMP4A	33	0.17	INDEL
rs3215610	chr14	24214060	CA	C	MDP1	13	0.067	INDEL
rs3435410	chr14	24238273	G	A	GMPR2	17	0.088	SNP
rs6573921	chr14	24654708	G	A	RP11-104E	76	0.392	SNP
rs1241484	chr14	25126917	C	T	RP11-89K2	2	0.01	SNP
.	chr14	28812952	CTTTTTT	CTTTTTTT	LINC01551	108	0.563	INDEL
.	chr14	28812952	CTTTTTT	CTTTTTTTT	LINC01551	3	0.016	INDEL
.	chr14	28812952	CTTTTTT	CTTTTT	LINC01551	9	0.047	INDEL
rs7264253	chr14	28830125	C	A	RP11-966I7	2	0.01	SNP
210220	chr14	31084758	G	GT	AP4S1	2	0.01	INDEL
rs1014596	chr14	31427794	G	T	RP11-176H	194	1	SNP
.	chr14	31483548	G	A	GPR33	193	0.995	SNP
rs3432024	chr14	34768733	CT	C	BAZ1A	20	0.103	INDEL
rs7144852	chr14	36809117	G	A	RP11-81F1	67	0.345	SNP

rs1155621(	chr14	39048721	T	C	SEC23A	80	0.412	SNP
.	chr14	39128266	C	CT	GEMIN2	8	0.041	INDEL
rs2002712	chr14	39131946	A	AT	GEMIN2	2	0.01	INDEL
rs1386850(	chr14	45042296	TCTCTGGA	T	FAM179B&	12	0.062	INDEL
rs8009193	chr14	45135258	C	T	FKBP3	13	0.067	SNP
rs1123267!	chr14	45159080	TTA	T	FANCM	26	0.134	INDEL
rs3425235(	chr14	45185207	TCTTA	T	FANCM	27	0.139	INDEL
rs2008327!	chr14	50156936	A	AAT	SOS2	9	0.046	INDEL
rs1119709(	chr14	50156978	TAA	T	SOS2	9	0.046	INDEL
rs1060197;	chr14	50591009	G	A	ATL1	142	0.732	SNP
rs1135603!	chr14	50911872	CT	C	PYGL	77	0.397	INDEL
rs3764156	chr14	51352580	A	G	RP11-255G	46	0.237	SNP
rs7112170(	chr14	52287957	CAAAAAA	C	RP11-452D	4	0.021	INDEL
rs7112170(	chr14	52287957	CAAAAAA	CAAAAA	RP11-452D	46	0.24	INDEL
rs7112170(	chr14	52287957	CAAAAAA	CAAA	RP11-452D	4	0.021	INDEL
rs7112170(	chr14	52287957	CAAAAAA	CAAAA	RP11-452D	9	0.047	INDEL
rs7112170(	chr14	52287957	CAAAAAA	CAAAAAAA	RP11-452D	16	0.083	INDEL
rs4898777	chr14	53037075	T	C	RP11-368P	167	0.861	SNP
rs7712707(	chr14	53369433	CTT	CT	AL163953.:	50	0.258	INDEL
rs7712707(	chr14	53369433	CTT	CTTT	AL163953.:	45	0.232	INDEL
rs7712707(	chr14	53369433	CTT	C	AL163953.:	2	0.01	INDEL
rs17563;19	chr14	53950804	A	G	BMP4	107	0.552	SNP
.	chr14	54760360	CCACGGCC	C	SAMD4A	26	0.134	INDEL
rs11125	chr14	55145121	A	T	LGALS3	15	0.077	SNP
.	chr14	55438906	CAA	C	TBPL2	12	0.062	INDEL
.	chr14	55438906	CAA	CA	TBPL2	30	0.155	INDEL
.	chr14	55438906	CAA	CAAA	TBPL2	9	0.046	INDEL
rs1288820(	chr14	55962278	G	A	RP11-1012	8	0.041	SNP
rs3496043(	chr14	57480703	G	A	C14orf105	2	0.01	SNP
rs1152522	chr14	57481662	T	C	C14orf105	133	0.686	SNP
rs1497989	chr14	57483127	G	A	C14orf105	2	0.01	SNP
rs1119324	chr14	58231077	C	CAG	ACTR10	123	0.634	INDEL
rs3783694	chr14	58427859	T	C	KIAA0586	110	0.567	SNP
rs2014561	chr14	59483488	AC	A	L3HYPDH	5	0.026	INDEL
rs3542717!	chr14	60008141	G	A	LRRC9	5	0.026	SNP
rs1014320	chr14	60658222	A	G	SIX1	179	0.923	SNP
372246	chr14	60979582	CTATT	C	TRMT5	2	0.01	INDEL
rs1876404(	chr14	61570761	G	A	FLJ22447	3	0.015	SNP
.	chr14	63299320	CG	C	RP11-696D	2	0.01	INDEL
.	chr14	63599645	G	A	WDR89	33	0.17	SNP
rs6059646	chr14	63993818	A	AT	SYNE2	92	0.474	INDEL
rs2781377;	chr14	64093374	G	A	SYNE2	19	0.098	SNP
rs1473730	chr14	64354717	TTACTTTC	T	TEX21P	3	0.015	INDEL
rs1063391	chr14	64541533	C	T	HSPA2	132	0.68	SNP
rs2844078	chr14	65222218	C	T	CTD-2509G	10	0.052	SNP
.	chr14	66922784	CTCTTT	C	GPHN	3	0.015	INDEL
rs1512566!	chr14	67204765	C	CT	FAM71D	4	0.021	INDEL

.	chr14	67254182	CT	C	MPP5	7	0.042	INDEL
.	chr14	67254182	CT	CTT	MPP5	4	0.024	INDEL
rs1114624	chr14	67587182	C	T	PLEKHH1	2	0.01	SNP
rs5567060	chr14	68540199	G	A	RAD51B	17	0.088	SNP
.	chr14	68868952	CTTTT	CT	BLZF2P	80	0.412	INDEL
.	chr14	68868952	CTTTT	CTT	BLZF2P	70	0.361	INDEL
.	chr14	68868952	CTTTT	CTTT	BLZF2P	29	0.149	INDEL
.	chr14	68868952	CTTTT	C	BLZF2P	8	0.041	INDEL
rs1174091	chr14	69351742	G	A	GALNT16	3	0.015	SNP
rs1114954	chr14	69461696	G	A	SLC39A9	2	0.01	SNP
rs1747533	chr14	70359741	T	G	SYNJ2BP-C(	25	0.129	SNP
rs4903046	chr14	72679200	G	A	DPF3	85	0.438	SNP
.	chr14	73064954	CT	CTT	RBM25	16	0.083	INDEL
.	chr14	73064954	CT	C	RBM25	11	0.057	INDEL
rs1509882(	chr14	73105898	AAG	A	RBM25	5	0.026	INDEL
rs6198693	chr14	73245245	C	A	RP4-647C1	13	0.067	SNP
.	chr14	73263953	GGCCCCCC	G	PAPLN	20	0.103	MIXED
.	chr14	73263953	GGCCCCCC	AGCCCCCC	PAPLN	2	0.01	MIXED
rs1059560	chr14	73264585	CAG	C	PAPLN	4	0.021	INDEL
.	chr14	73477094	ATTTTTTTT	*	RP1-240K6	26	0.351	MIXED
.	chr14	73477094	ATTTTTTTT	ATTT	RP1-240K6	3	0.041	MIXED
.	chr14	73477094	ATTTTTTTT	ATTTTT	RP1-240K6	3	0.041	MIXED
.	chr14	73477094	ATTTTTTTT	TTTTTTTTT	RP1-240K6	3	0.041	MIXED
.	chr14	73477094	ATTTTTTTT	ATTTTTTTT	RP1-240K6	2	0.027	MIXED
rs5606962	chr14	73569512	A	G	ACOT2	6	0.031	SNP
rs1469680	chr14	73569796	C	T	ACOT2	5	0.026	SNP
rs3572488(	chr14	73593804	C	A	ACOT4	59	0.304	SNP
.	chr14	73593807	T	TTCAA	ACOT4	59	0.304	INDEL
rs3758019	chr14	73593809	GCTTA	G	ACOT4	59	0.304	INDEL
rs1115904	chr14	73874094	A	T	PTGR2	104	0.536	SNP
rs1401300	chr14	74480701	C	T	NPC2	4	0.021	SNP
rs1456535	chr14	76196033	G	GT	GPATCH2L	39	0.201	INDEL
rs6041519	chr14	76828758	T	C	LRRC74A	12	0.062	SNP
.	chr14	77027444	GC	G	RP11-7F17.	2	0.01	INDEL
.	chr14	77027446	TGCTGTTG	*	IRF2BPL	5	0.026	INDEL
.	chr14	77027487	CGCGGCGC	C	IRF2BPL	5	0.026	INDEL
rs3548451	chr14	77181894	TGTG	T	TMEM63C	91	0.469	INDEL
.	chr14	77416654	TC	T	AF111168.	4	0.021	INDEL
rs1511080	chr14	80831096	GAT	G	CEP128	7	0.036	INDEL
rs1289599(	chr14	81209938	T	C	GTF2A1	18	0.093	SNP
rs7818775	chr14	87992401	C	T	GALC	22	0.113	SNP
rs5810413;	chr14	88385935	TG	T	SPATA7	194	1	INDEL
rs3179969;	chr14	88396185	G	A	SPATA7	74	0.381	SNP
rs3469435	chr14	90023075	CTGG	C	TDP1	4	0.021	INDEL
rs4131350	chr14	91114996	T	C	C14orf159	15	0.077	SNP
rs4900072	chr14	91170188	C	T	C14orf159	58	0.299	SNP
.	chr14	91215616	GC	G	C14orf159	2	0.01	INDEL

rs7158733	chr14	92070879	G	T	ATXN3	35	0.18	SNP
402402	chr14	92071009	C	CG	ATXN3	32	0.167	INDEL
218660	chr14	92071010	C	CTGCTGCT(	ATXN3	26	0.134	INDEL
218660	chr14	92071010	C	CCTGCTGC	ATXN3	72	0.371	INDEL
218660	chr14	92071010	C	CCTGCTGC	ATXN3	12	0.062	INDEL
218660	chr14	92071010	C	CCTGCTGC	ATXN3	10	0.052	INDEL
218660	chr14	92071010	C	CTGCTGCT(	ATXN3	8	0.041	INDEL
218660	chr14	92071010	C	CCTGCTGC	ATXN3	2	0.01	INDEL
.	chr14	92071034	G	GC	ATXN3	11	0.057	INDEL
rs1289658	chr14	92071035	T	C	ATXN3	6	0.032	MIXED
rs1289658	chr14	92071035	T	TGCTGCTG	ATXN3	9	0.047	MIXED
rs3494905	chr14	92121194	A	G	NDUFB1	69	0.356	SNP
rs5922349	chr14	92714382	A	G	LGMN	2	0.01	SNP
rs2020703	chr14	94464705	A	G	SERPINA9	2	0.01	SNP
rs7837984	chr14	95260604	T	C	CLMN	26	0.134	SNP
rs1162890	chr14	95544087	G	A	GLRX5	22	0.113	SNP
.	chr14	96555831	AT	A	PAPOLA	20	0.103	INDEL
.	chr14	96555831	AT	ATT	PAPOLA	7	0.036	INDEL
rs1065186	chr14	96860761	T	TTG	VRK1	14	0.072	INDEL
rs1398271	chr14	98716189	TGGATGCA	T	C14orf177	18	0.093	INDEL
rs9453	chr14	1E+08	G	A	WARS	143	0.737	SNP
rs5878171	chr14	1E+08	T	A	WARS	7	0.036	SNP
rs1014055	chr14	1.01E+08	C	G	BEGAIN	22	0.113	SNP
rs1169076	chr14	1.01E+08	G	C	MEG3	5	0.026	SNP
rs3742402	chr14	1.01E+08	G	C	MEG9	16	0.082	SNP
rs1131030	chr14	1.01E+08	AC	A	AL132709.	6	0.031	INDEL
rs3464499	chr14	1.02E+08	T	G	HSP90AA1	6	0.031	SNP
rs4947	chr14	1.02E+08	G	A	HSP90AA1	165	0.851	SNP
.	chr14	1.02E+08	TAC	T	MOK	2	0.01	INDEL
rs5637716	chr14	1.02E+08	A	G	MOK	14	0.072	SNP
rs3493175	chr14	1.02E+08	G	A	MOK	10	0.052	SNP
rs1135542	chr14	1.03E+08	CG	C	EXOC3L4	92	0.484	INDEL
.	chr14	1.03E+08	A	C	RPL21P12	3	0.015	SNP
rs6079146	chr14	1.03E+08	CAA	CA	GCSHP2	4	0.035	INDEL
rs6079146	chr14	1.03E+08	CAA	CAAA	GCSHP2	6	0.053	INDEL
.	chr14	1.03E+08	G	GT	MARK3	6	0.031	INDEL
rs1803283	chr14	1.04E+08	C	T	CKB	117	0.603	SNP
rs1136165	chr14	1.04E+08	G	T	CKB	113	0.582	SNP
rs7750559	chr14	1.04E+08	T	G	RP11-260M	9	0.046	SNP
rs1392976	chr14	1.05E+08	G	A	AKT1	2	0.01	SNP
rs1440885	chr14	1.05E+08	G	A	AKT1	2	0.01	SNP
.	chr14	1.05E+08	TTGGC	T	AHNAK2	2	0.014	INDEL
.	chr14	1.05E+08	A	C	JAG2	2	0.01	SNP
rs2816606	chr14	1.05E+08	C	T	BTBD6	47	0.242	SNP
rs1998076	chr15	20535014	C	A	GOLGA6L6	55	0.444	SNP
rs1400599	chr15	23364403	G	T	GOLGA8S	29	0.149	SNP
.	chr15	23440078	T	TG	GOLGA6L2	8	0.045	INDEL

rs3759819	chr15	23440394	CT	C	GOLGA6L2	180	0.968	INDEL
.	chr15	23440457	TC	T	GOLGA6L2	56	0.289	INDEL
.	chr15	23440459	TCTTCCTG(	T	GOLGA6L2	55	0.284	INDEL
rs1290505	chr15	24568183	G	A	PWRN1	73	0.376	SNP
rs7688851	chr15	27271593	GAGTC	G	GABRG3	120	0.619	INDEL
rs1996614	chr15	28845789	A	AT	GOLGA6L7I	3	0.015	INDEL
rs1503002	chr15	34520788	A	G	RP11-1H8.(	4	0.021	SNP
rs1384026	chr15	34520789	G	T	RP11-1H8.(	4	0.021	SNP
rs8009310	chr15	34906678	G	A	AQR	14	0.072	SNP
rs3692276	chr15	35084193	TCA	T	NANOGP8	11	0.057	INDEL
rs7808751	chr15	39071501	A	G	RP11-624L	3	0.015	SNP
rs4133834	chr15	39588617	C	T	THBS1	9	0.046	SNP
rs2022252	chr15	40292440	T	C	PLCB2	2	0.01	SNP
.	chr15	40407805	GTT	G	IVD	56	0.289	INDEL
rs3803354	chr15	40564790	C	T	C15orf57	163	0.84	SNP
rs1427305	chr15	40807701	T	TGGGGC	ZFYVE19	73	0.376	INDEL
rs7839514	chr15	40999441	G	A	RP11-540O	12	0.062	SNP
rs1467116	chr15	41287959	C	CA	OIP5-AS1	55	0.284	INDEL
rs2004462	chr15	41351947	CT	C	NUSAP1	55	0.284	INDEL
rs2004462	chr15	41351947	CT	CTT	NUSAP1	3	0.015	INDEL
rs4924549	chr15	41507513	C	T	LTK	21	0.108	SNP
.	chr15	41511863	CAGCGCCG	C	LTK	3	0.015	INDEL
.	chr15	41561310	G	GCCTGAA	TYRO3	10	0.052	INDEL
.	chr15	41570155	GGGTAAG(	G	TYRO3	12	0.062	INDEL
.	chr15	41570340	TGTGAG	TTGGACAG	TYRO3	3	0.015	INDEL
.	chr15	41570340	TGTGAG	TTGGACAG	TYRO3	2	0.01	INDEL
.	chr15	41570699	G	GGAGA	TYRO3	4	0.021	INDEL
.	chr15	41571686	T	TGG	TYRO3	3	0.015	INDEL
.	chr15	41572564	T	TAACCTAC(	TYRO3	2	0.01	INDEL
.	chr15	41573466	T	TGTGGGCG	TYRO3	4	0.021	INDEL
.	chr15	41573467	G	GTGGGCGT	TYRO3	2	0.01	INDEL
rs1997115	chr15	41861419	C	T	SPTBN5	2	0.01	SNP
rs6175083	chr15	41870269	G	A	SPTBN5	9	0.046	SNP
.	chr15	42801163	T	TTGACTGG	TTBK2	3	0.016	INDEL
rs4557624	chr15	43330860	G	A	ADAL	3	0.015	SNP
rs3917221	chr15	43366737	C	T	ZSCAN29	33	0.17	SNP
rs3809482	chr15	43369604	T	C	ZSCAN29	46	0.237	SNP
rs7169097;	chr15	43632936	T	A	CATSPER2	47	0.242	SNP
rs1170913	chr15	43744570	T	A	CATSPER2F	2	0.01	SNP
.	chr15	43783008	CTT	CT	AC018512.	26	0.134	INDEL
.	chr15	43783008	CTT	C	AC018512.	3	0.015	INDEL
.	chr15	43783008	CTT	CTTT	AC018512.	19	0.098	INDEL
rs8026845	chr15	44674191	T	C	PATL2	194	1	SNP
rs5744534	chr15	44924659	G	A	CTD-2014N	4	0.021	SNP
189229	chr15	45101227	TGAAC	T	DUOX2	2	0.01	INDEL
.	chr15	45199953	CAAAAAAA	CAA	RP11-519G	112	0.577	INDEL
.	chr15	45199953	CAAAAAAA	C	RP11-519G	3	0.015	INDEL

.	chr15	45199953	CAAAAAAA	CA	RP11-519G	34	0.175	INDEL
.	chr15	45199953	CAAAAAAA	CAAA	RP11-519G	34	0.175	INDEL
.	chr15	45199953	CAAAAAAA	CAAAA	RP11-519G	4	0.021	INDEL
rs6682281	chr15	48331051	TCGGGCGC	T	RP11-154J2	167	0.861	INDEL
rs1055254	chr15	49328003	C	T	GALK2	46	0.237	SNP
rs1185751	chr15	50497226	G	A	USP8	9	0.046	SNP
rs1810098	chr15	50874570	C	A	CTD-2378E	188	0.969	SNP
rs1765184	chr15	52590675	G	A	RP11-23N2	12	0.062	SNP
.	chr15	54013186	C	T	UNC13C	2	0.01	SNP
rs5744929	chr15	54338354	T	TTGTC	UNC13C	50	0.258	INDEL
rs7479353	chr15	54416885	G	A	UNC13C	2	0.01	SNP
rs5780990	chr15	55430684	C	A	DYX1C1	7	0.036	SNP
rs2012957	chr15	55846988	C	T	NEDD4	2	0.01	SNP
rs1418138	chr15	56323706	GAGGAGG	G	RP11-844G	83	0.432	INDEL
.	chr15	56323715	AAGGAGG	*	RP11-844G	83	0.446	INDEL
rs7273914	chr15	58279202	A	T	ALDH1A2	25	0.129	SNP
.	chr15	58890364	GCT	G	SLTM	2	0.01	INDEL
rs8041223	chr15	59644327	C	G	AC092755.	76	0.392	SNP
rs4775262	chr15	60374487	C	T	ANXA2	33	0.17	SNP
rs2100431	chr15	60478651	C	A	ICE2	157	0.809	SNP
rs1827816	chr15	62248396	C	A	RP11-299H	3	0.015	SNP
.	chr15	62250582	C	CT	RP11-299H	3	0.015	INDEL
rs1181821	chr15	62843045	T	C	RP11-1069	7	0.036	SNP
rs332259	chr15	64093652	T	C	FAM96A	194	1	SNP
rs3575551	chr15	64355987	C	T	CSNK1G1	22	0.113	SNP
rs7208271	chr15	64393326	G	GT	TRIP4	9	0.046	INDEL
rs5994296	chr15	64393338	T	TA	TRIP4	86	0.443	INDEL
rs5994296	chr15	64393338	T	TTA	TRIP4	6	0.031	INDEL
rs7568353	chr15	64824191	C	A	PIF1	5	0.026	SNP
.	chr15	65392132	A	C	IGDCC4	2	0.01	SNP
.	chr15	65816864	CTT	CT	HNRNPA1P	40	0.206	INDEL
.	chr15	65816864	CTT	C	HNRNPA1P	4	0.021	INDEL
.	chr15	65816864	CTT	CTTT	HNRNPA1P	8	0.041	INDEL
rs1421744	chr15	67520778	C	T	IQCH-AS1	3	0.015	SNP
rs8024242	chr15	68750234	G	A	CARSP1	98	0.505	SNP
rs7114759	chr15	69196409	A	AT	GLCE	194	1	INDEL
rs1244030	chr15	69255999	A	G	GLCE	6	0.031	SNP
rs7486001	chr15	69295071	A	G	RP11-352D	23	0.119	SNP
.	chr15	70065994	CTG	C	TLE3	2	0.01	INDEL
rs7275549	chr15	70778780	A	C	RP11-138H	9	0.046	SNP
.	chr15	70872883	CTTTT	CTTT	LRRC49	34	0.175	INDEL
.	chr15	70872883	CTTTT	CTT	LRRC49	7	0.036	INDEL
.	chr15	70872883	CTTTT	CTTTTT	LRRC49	10	0.052	INDEL
.	chr15	72320629	A	C	RP11-106M	2	0.01	SNP
.	chr15	73126400	AT	A	NEO1	17	0.088	INDEL
.	chr15	73126400	AT	ATT	NEO1	4	0.021	INDEL
rs2680348	chr15	73270191	C	T	NEO1	18	0.093	SNP

rs7177088	chr15	73697704	T	G	CD276	14	0.072	SNP
rs7344638:	chr15	73928063	A	C	LOXL1-AS1	3	0.015	SNP
rs3399142:	chr15	74801081	C	T	CSK	6	0.031	SNP
.	chr15	75206850	CACTA	C	C15orf39	2	0.01	INDEL
.	chr15	75415854	GA	GAA	CTD-2562G	31	0.16	INDEL
.	chr15	75415854	GA	G	CTD-2562G	5	0.026	INDEL
.	chr15	75689500	ACCTCCAG	A	CSPG4	2	0.01	INDEL
rs1440372(	chr15	75783689	GGC	G	RP11-24M1	2	0.01	INDEL
rs3764189	chr15	76012301	C	T	NRG4	60	0.309	SNP
.	chr15	76228751	CT	C	TMEM266	31	0.165	INDEL
.	chr15	76228751	CT	CTT	TMEM266	7	0.037	INDEL
.	chr15	76228751	CT	CTTT	TMEM266	26	0.138	INDEL
.	chr15	76228751	CT	CTTTT	TMEM266	2	0.011	INDEL
rs3812908	chr15	76883817	T	C	SCAPER	66	0.34	SNP
rs907593	chr15	76904612	G	A	SCAPER	66	0.34	SNP
rs4886841	chr15	76938817	C	T	RCN2	191	0.985	SNP
rs4444305	chr15	77917094	C	T	RP11-114H	188	0.969	SNP
rs2289524	chr15	78098072	T	C	SH2D7	97	0.5	SNP
rs1420259	chr15	78266242	CAT	C	DNAJA4	9	0.046	INDEL
rs8053	chr15	78548878	T	C	PSMA4	115	0.593	SNP
.	chr15	78766678	T	TA	ADAMTS7	3	0.015	INDEL
rs1435163	chr15	78893556	C	A	MORF4L1	20	0.103	SNP
rs1411940!	chr15	79311418	G	A	TMED3	2	0.01	SNP
rs1482365:	chr15	81323836	C	CT	STARD5	12	0.062	INDEL
rs1508436	chr15	81332588	G	T	TMC3	2	0.01	SNP
rs7563247(	chr15	81342732	G	A	TMC3-AS1	8	0.041	SNP
.	chr15	82045432	CAT	C	MEX3B	3	0.015	INDEL
.	chr15	82045440	CTCGA	C	MEX3B	2	0.01	INDEL
.	chr15	82282392	AAC	A	SAXO2	2	0.01	INDEL
rs1500796:	chr15	84686514	G	A	RP11-245C	15	0.077	SNP
rs7952237!	chr15	86079762	A	G	LINC01584	14	0.072	SNP
rs1163588:	chr15	86079963	C	T	LINC01584	121	0.624	SNP
rs1105693	chr15	88253479	G	A	NTRK3-AS1	67	0.345	SNP
rs8023681	chr15	88604862	A	G	LINC01586	76	0.392	SNP
rs3500875!	chr15	88639471	T	C	ISG20	11	0.057	SNP
rs8039131	chr15	88893064	G	A	HAPLN3	50	0.258	SNP
rs1404174!	chr15	89335370	G	T	RP11-217B	2	0.01	SNP
.	chr15	89751073	C	CG	MESP1	54	0.278	INDEL
rs6496598	chr15	89751075	C	CACGGGG(	MESP1	54	0.278	MIXED
rs6496598	chr15	89751075	C	G	MESP1	45	0.232	MIXED
.	chr15	89753039	CAAAAAAA	CAA	MRPL15P1	3	0.016	INDEL
.	chr15	89753039	CAAAAAAA	CAAA	MRPL15P1	13	0.068	INDEL
.	chr15	89753039	CAAAAAAA	CAAAA	MRPL15P1	53	0.276	INDEL
.	chr15	89753039	CAAAAAAA	CAAAAAA	MRPL15P1	87	0.453	INDEL
.	chr15	89753039	CAAAAAAA	CAAAAA	MRPL15P1	10	0.052	INDEL
rs1413336	chr15	89785450	G	A	ANPEP	3	0.015	SNP
rs25651	chr15	89792557	C	T	ANPEP	55	0.284	SNP

rs4127692:	chr15	89806230	G	A	ANPEP	10	0.052	SNP
rs25653	chr15	89806327	C	T	ANPEP	102	0.526	SNP
rs1505741(	chr15	90084323	G	C	IDH2	4	0.021	SNP
rs4932305	chr15	90220987	G	A	SEMA4B	22	0.113	SNP
rs6225	chr15	90880109	G	T	FURIN	5	0.026	SNP
rs1133024(	chr15	90885291	CT	C	FES	68	0.351	INDEL
rs1379045	chr15	90966617	GACAC	G	PRC1	2	0.01	INDEL
rs9744253	chr15	93900350	C	A	LINC01579	40	0.206	SNP
rs2397454	chr15	95255889	C	A	LINC01197	51	0.263	SNP
rs6736981(	chr15	96118966	ATCCTGAT	A	NR2F2-AS1	10	0.052	INDEL
rs7139684:	chr15	96127160	A	G	NR2F2-AS1	3	0.015	SNP
rs3812907	chr15	96784163	C	T	SPATA8	19	0.098	SNP
rs1948348	chr15	97809082	C	A	LINC00923	103	0.531	SNP
rs1214848:	chr15	98953111	G	T	IGF1R	26	0.134	SNP
rs5814919	chr15	98968575	A	AC	PGPEP1L	87	0.448	INDEL
.	chr15	99028516	CTAGTTTA(	C	RP11-654A	9	0.046	INDEL
.	chr15	99028516	CTAGTTTA(	CTAGTTTA(	RP11-654A	90	0.464	INDEL
.	chr15	99028516	CTAGTTTA(	CTAGTTTA(	RP11-654A	33	0.17	INDEL
.	chr15	99028516	CTAGTTTA(	CTAGTT	RP11-654A	18	0.093	INDEL
.	chr15	99028516	CTAGTTTA(	CTAGTTTA(	RP11-654A	3	0.015	INDEL
.	chr15	99028516	CTAGTTTA(	CTAGTTTA(	RP11-654A	5	0.026	INDEL
rs2004932	chr15	99139508	G	A	TTC23	2	0.01	SNP
rs2021193(	chr15	99387067	TTTG	GTTG	LRRC28	94	0.485	MIXED
rs2021193(	chr15	99387067	TTTG	T	LRRC28	6	0.031	MIXED
rs2018667(	chr15	99716652	A	ACG	LYSMD4	6	0.031	INDEL
rs3764198	chr15	1.01E+08	C	T	LINS1	180	0.928	SNP
rs4965729	chr15	1.01E+08	T	C	RP11-66B2	4	0.021	SNP
rs7178773	chr15	1.01E+08	G	A	RP11-66B2	90	0.464	SNP
rs7169323	chr15	1.01E+08	C	G	RP11-505E	79	0.407	SNP
rs1489294:	chr15	1.01E+08	C	CACTT	LRRK1	84	0.433	INDEL
rs1168526!	chr15	1.01E+08	C	T	PCSK6	3	0.015	SNP
rs1466001!	chr16	79424	G	T	MPG	3	0.015	SNP
.	chr16	138304	AGAGGAG(	A	NPRL3	14	0.072	INDEL
rs1999390	chr16	254387	G	A	FAM234A	9	0.046	SNP
rs1501293:	chr16	260645	C	CACGG	FAM234A	2	0.01	INDEL
rs6175990:	chr16	270993	C	A	RGS11	6	0.031	SNP
rs1998871:	chr16	286888	C	CA	PDIA2	2	0.01	INDEL
.	chr16	369614	C	CCCGACCT(	MRPL28	6	0.031	INDEL
rs1441775:	chr16	372544	A	AG	TMEM8A	9	0.046	INDEL
rs3596349(	chr16	400140	C	CAG	NME4	78	0.402	INDEL
rs1133557!	chr16	536144	T	C	CAPN15	24	0.124	SNP
rs1442821:	chr16	572568	G	GATGT	PIGQ	29	0.149	INDEL
rs3838962	chr16	625087	AC	A	RAB40C	69	0.356	INDEL
rs3216838	chr16	682287	GC	G	STUB1	86	0.443	INDEL
rs5815052	chr16	717426	T	TG	METRN	41	0.211	MIXED
rs5815052	chr16	717426	T	G	METRN	6	0.031	MIXED
rs1999638:	chr16	717449	T	TG	METRN	4	0.021	INDEL

rs1054266:	chr16	788407	GCC	GC	CHTF18	53	0.282	MIXED
rs1054266:	chr16	788407	GCC	CCC	CHTF18	4	0.021	MIXED
rs1054266:	chr16	788407	GCC	GCCC	CHTF18	8	0.043	MIXED
rs1054266:	chr16	788407	GCC	G	CHTF18	26	0.138	MIXED
.	chr16	788418	CGG	C	CHTF18	2	0.011	INDEL
rs1387338:	chr16	813355	AGGACGCC	A	PRR25	6	0.031	INDEL
rs7275947	chr16	933990	C	T	LMF1	3	0.015	SNP
rs1485509	chr16	964046	C	T	LMF1	4	0.021	SNP
rs3554268	chr16	991240	CA	C	RP11-161M	113	0.582	INDEL
rs1503373	chr16	1093496	C	A	C1QTNF8	5	0.026	SNP
rs3830782	chr16	1256558	AG	A	TPSD1	34	0.175	INDEL
rs4610	chr16	1314364	A	G	UBE2I	14	0.072	SNP
.	chr16	1397263	TCCCCGTG	T	UNKL	92	0.474	INDEL
rs1173185	chr16	1434535	C	T	CCDC154	4	0.021	SNP
rs11890	chr16	1770991	T	A	NME3	159	0.82	SNP
rs4027362	chr16	1772796	C	CACCT	MRPS34	108	0.557	INDEL
rs3770746	chr16	1773388	CG	C	EME2	5	0.026	INDEL
rs7276117	chr16	1783507	G	A	NUBP2	16	0.082	SNP
.	chr16	1961657	CAA	C	NDUFB10	2	0.01	INDEL
rs1995639	chr16	1979537	G	A	NOXO1	2	0.01	SNP
rs3416586	chr16	2037784	T	C	SLC9A3R2	36	0.186	SNP
.	chr16	2228290	A	G	E4F1	2	0.01	SNP
.	chr16	2771012	C	CT	SRRM2	2	0.01	INDEL
rs7638146:	chr16	2857670	GGT	G	PRSS22	164	0.845	INDEL
rs2717692	chr16	2998390	T	C	LA16c-380I	125	0.644	SNP
rs3216961	chr16	3020707	TG	T	TNFRSF12A	125	0.644	INDEL
rs5882617	chr16	3081939	C	CGG	IL32	115	0.618	INDEL
rs1048950	chr16	3243403	T	C	MEFV	7	0.036	SNP
rs1231122;	chr16	3243888	C	T	MEFV	89	0.459	SNP
rs224207;2	chr16	3247175	T	C	MEFV	105	0.541	SNP
rs1423973	chr16	3356756	GT	G	OR2C1	20	0.103	INDEL
rs1136948;	chr16	3671772	G	C	TRAP1	37	0.191	SNP
rs1154171:	chr16	3674351	G	A	TRAP1	8	0.041	SNP
rs5825530	chr16	4206754	TC	T	SRL	83	0.428	INDEL
rs7838477	chr16	4714116	A	C	ANKS3	13	0.067	SNP
rs5872982	chr16	4766101	T	C	ZNF500	49	0.253	SNP
rs6203608	chr16	4796335	G	A	RP11-127I2	48	0.247	SNP
rs1126835:	chr16	5072071	G	GGTCT	ALG1	106	0.546	INDEL
rs7715988	chr16	5120673	C	A	ENPP7P14	3	0.015	SNP
rs1508118	chr16	6573891	G	A	RP11-468I1	2	0.01	SNP
.	chr16	7328627	ATT	A	RBFOX1	3	0.015	INDEL
.	chr16	7328627	ATT	AT	RBFOX1	36	0.186	INDEL
.	chr16	7328627	ATT	ATTT	RBFOX1	4	0.021	INDEL
rs1474361	chr16	8797614	T	C	TMEM186	3	0.015	SNP
rs3425828	chr16	8813057	A	C	PMM2	5	0.026	SNP
rs1411692:	chr16	8860127	G	C	RP11-77H9	2	0.01	SNP
.	chr16	9070823	ATTTTTTT	TTTTTTTT	RP11-473I1	3	0.034	MIXED

.	chr16	9070823	ATTTTTTT	ATTTTTTTT	RP11-473I1	3	0.034	MIXED
.	chr16	9070823	ATTTTTTT	*	RP11-473I1	14	0.159	MIXED
.	chr16	9070823	ATTTTTTT	ATTTTTTTT	RP11-473I1	9	0.102	MIXED
.	chr16	9070823	ATTTTTTT	ATATTTTTT	RP11-473I1	2	0.023	MIXED
.	chr16	9070823	ATTTTTTT	A	RP11-473I1	2	0.023	MIXED
rs7557300	chr16	10800340	T	G	TVP23A	8	0.041	SNP
.	chr16	11273377	C	CTT	PRM3	2	0.01	INDEL
rs1154533	chr16	11345639	G	A	RMI2	33	0.17	SNP
rs7204628	chr16	11350715	A	C	RMI2	33	0.17	SNP
.	chr16	11414495	CTGGGT	C	CTD-3088G	3	0.015	INDEL
.	chr16	11424261	CCG	C	CTD-3088G	2	0.01	INDEL
.	chr16	11908477	T	TAGCC	RP11-166B	9	0.047	INDEL
rs6084542	chr16	11908478	AG	A	RP11-166B	7	0.038	INDEL
.	chr16	11908480	G	C	RP11-166B	3	0.018	SNP
rs7113950	chr16	11933617	CAT	C	RP11-166B	194	1	INDEL
.	chr16	11966977	CATTTATTT	CATTT	TNFRSF17	76	0.392	INDEL
.	chr16	11966977	CATTTATTT	CATTTATTT	TNFRSF17	42	0.216	INDEL
.	chr16	11966977	CATTTATTT	C	TNFRSF17	4	0.021	INDEL
.	chr16	11966977	CATTTATTT	CATTTATTT	TNFRSF17	18	0.093	INDEL
.	chr16	11966977	CATTTATTT	CATTTATTT	TNFRSF17	3	0.015	INDEL
rs1244439	chr16	13203491	C	T	AC009134.	8	0.041	SNP
.	chr16	18347256	TCAC	T	RP11-1212	36	0.188	INDEL
rs1432443	chr16	19061798	C	A	TMC7	4	0.021	SNP
rs4496124	chr16	19087002	C	T	RP11-626G	185	0.954	SNP
rs5816059	chr16	19581668	T	TC	C16orf62	42	0.216	INDEL
rs5816059	chr16	19581668	T	TCCC	C16orf62	5	0.026	INDEL
rs5816059	chr16	19581668	T	TCCCC	C16orf62	4	0.021	INDEL
rs4782272	chr16	19717694	G	C	IQCK	35	0.18	SNP
rs7560245	chr16	20442389	C	T	CTD-2194A	12	0.062	SNP
rs5926176	chr16	20465682	C	T	ACSM2A	2	0.01	SNP
rs1700805	chr16	20477374	A	G	ACSM2A	51	0.263	SNP
rs7499739	chr16	20549868	G	T	ACSM2B	43	0.222	SNP
rs1692729	chr16	20627254	A	T	ACSM1	65	0.335	SNP
rs3438122	chr16	20790685	C	G	ACSM3	2	0.01	SNP
rs2204997	chr16	21177328	G	A	TMEM159	153	0.789	SNP
rs14122;44	chr16	21261270	G	C	CRYM	83	0.428	SNP
rs7943712	chr16	21527215	CT	C	CTB-31N19	7	0.036	INDEL
.	chr16	21612623	CTT	CT	METTL9	23	0.122	INDEL
.	chr16	21612623	CTT	CTTT	METTL9	14	0.074	INDEL
rs2010441	chr16	22008325	AG	A	C16orf52	19	0.098	INDEL
.	chr16	23493474	A	AC	GGA2	4	0.021	INDEL
rs6173114	chr16	23757554	C	T	CHP2	6	0.031	SNP
.	chr16	25020224	GAAGGAA	*	RP11-266L	12	0.062	MIXED
.	chr16	25020224	GAAGGAA	G	RP11-266L	5	0.026	MIXED
.	chr16	25020224	GAAGGAA	AAAGGAA	RP11-266L	3	0.015	MIXED
rs1407860	chr16	25020228	GAAGAAA	GAAGAAA	RP11-266L	27	0.144	MIXED
rs1407860	chr16	25020228	GAAGAAA	G	RP11-266L	30	0.16	MIXED

rs1407860(	chr16	25020228	GAAGAAA(	*	RP11-266L!	17	0.09	MIXED
rs1407860(	chr16	25020228	GAAGAAA(	AAAGAAA(	RP11-266L!	22	0.117	MIXED
rs1407860(	chr16	25020228	GAAGAAA(	GAAGGAA(	RP11-266L!	11	0.059	MIXED
rs1407860(	chr16	25020228	GAAGAAA(	GAAGGAA(	RP11-266L!	6	0.032	MIXED
rs3457737(	chr16	27220419	G	C	KDM8	31	0.16	SNP
.	chr16	28342966	GAGCAA	G	NPIPB6	4	0.021	INDEL
rs3433748:	chr16	28588681	C	CA	SGF29	10	0.052	INDEL
rs11401	chr16	28591670	A	G	SGF29	26	0.134	SNP
rs3194168	chr16	28591691	A	G	SGF29	15	0.077	SNP
rs1450081	chr16	28595800	C	T	SULT1A2	2	0.01	SNP
rs1079730(	chr16	28595875	G	A	SULT1A2	15	0.077	SNP
rs3451397:	chr16	28608799	C	T	SULT1A1	8	0.041	SNP
rs5571989(	chr16	28835545	G	A	ATXN2L	81	0.418	SNP
.	chr16	28863346	G	C	SH2B1	4	0.021	SNP
rs7465732!	chr16	29212907	G	A	RP11-426C	3	0.015	SNP
.	chr16	29449170	AATATTAT	A	BOLA2&RP	26	0.134	INDEL
rs2303255	chr16	29695123	C	T	QPRT	41	0.211	SNP
rs3935873	chr16	30007179	C	T	DOC2A	87	0.448	SNP
.	chr16	30652232	CT	C	PRR14	21	0.108	INDEL
.	chr16	30652232	CT	CTT	PRR14	12	0.062	INDEL
.	chr16	30654797	CGT	C	PRR14	2	0.01	INDEL
.	chr16	30654799	T	TCCCCCCC(	PRR14	2	0.013	INDEL
.	chr16	30654799	T	TCCCCCCC(	PRR14	2	0.013	INDEL
.	chr16	30664406	CACCA	C	FBRS	2	0.012	INDEL
.	chr16	30664422	CTCCTTGT	C	FBRS	2	0.011	INDEL
.	chr16	30664425	CTTGTTCT	C	FBRS	5	0.026	INDEL
.	chr16	30664425	CTTGTTCT	*	FBRS	2	0.011	INDEL
.	chr16	30965154	CAG	C	SETD1A	2	0.01	INDEL
rs2884737;	chr16	31094233	A	C	VKORC1	59	0.304	SNP
rs7578242(	chr16	31528606	A	T	AHSP	3	0.015	SNP
rs3751738	chr16	31709315	T	C	CTD-2358C	128	0.66	SNP
rs1998016:	chr16	33009518	G	GTT	IGHV3OR1(	4	0.021	INDEL
rs1909621:	chr16	33858533	C	T	RP11-812E	3	0.015	SNP
rs1417335!	chr16	47461389	G	A	PHKB	3	0.015	SNP
rs3610257!	chr16	48096870	C	T	ABCC12	4	0.021	SNP
.	chr16	50294637	CAGCAT	C	ADCY7	4	0.021	INDEL
rs1458963!	chr16	50334766	CCT	C	BRD7	5	0.026	INDEL
rs2066847;	chr16	50729867	G	GC	NOD2	11	0.057	INDEL
rs3135499;	chr16	50732216	A	C	RP11-327F:	81	0.418	SNP
rs6204282!	chr16	52095074	G	A	RP11-152O	18	0.093	SNP
rs8050739	chr16	52630885	T	C	RP11-297L:	45	0.232	SNP
rs2002361	chr16	54283671	G	GT	IRX3	2	0.01	INDEL
rs1164785:	chr16	55464826	G	C	MMP2	77	0.397	SNP
.	chr16	55493214	A	C	MMP2	2	0.01	SNP
.	chr16	55671848	T	G	SLC6A2	2	0.01	SNP
rs1181480(	chr16	56498694	C	G	BBS2	4	0.021	SNP
rs1924916	chr16	56643754	T	G	MT1DP	2	0.01	SNP

rs2298847	chr16	56667382	T	A	MT1G	25	0.129	SNP
rs1186190:	chr16	56740875	C	T	NUP93	46	0.237	SNP
rs3214652	chr16	56839701	T	TA	NUP93	20	0.103	INDEL
rs1421245:	chr16	57061930	AAAAG	A	NLRC5	58	0.299	INDEL
rs6499867	chr16	57153807	C	T	FAM192A	180	0.928	SNP
.	chr16	57321907	CAAAAAAA	CAAA	RPL23AP91	5	0.036	INDEL
.	chr16	57321907	CAAAAAAA	CAAAAAAA	RPL23AP91	10	0.072	INDEL
.	chr16	57321907	CAAAAAAA	CAAAAAAA	RPL23AP91	11	0.08	INDEL
.	chr16	57321907	CAAAAAAA	CAAAAAAA	RPL23AP91	9	0.065	INDEL
.	chr16	57321907	CAAAAAAA	C	RPL23AP91	2	0.014	INDEL
.	chr16	57321907	CAAAAAAA	CAAAAAAA	RPL23AP91	10	0.072	INDEL
rs170358	chr16	57430324	G	A	CIAPIN1	10	0.052	SNP
rs1174149:	chr16	57478543	C	A	DOK4	4	0.021	SNP
.	chr16	57923267	TCTCA	T	CNGB1	25	0.129	INDEL
rs1695964:	chr16	58020195	C	G	USB1	14	0.072	SNP
rs5574863(	chr16	58167727	G	A	CSNK2A2	2	0.01	SNP
rs4784938	chr16	58433949	G	A	RP11-481J2	130	0.67	SNP
rs4784046	chr16	58516028	T	C	SETD6	116	0.598	SNP
rs1154099:	chr16	58541585	G	A	CNOT1	59	0.304	SNP
rs5817153;	chr16	58543411	GA	G	CNOT1	57	0.294	INDEL
rs246258	chr16	58545370	C	T	CNOT1	57	0.294	SNP
rs1998531:	chr16	60358911	TG	T	RP11-457D	28	0.144	INDEL
rs1130821	chr16	64988331	C	T	CDH11	32	0.165	SNP
.	chr16	65233181	G	A	RP11-256I9	6	0.031	SNP
rs3913028	chr16	65363379	A	G	LINC00922	17	0.088	SNP
rs1164268!	chr16	65571769	A	G	LINC00922	7	0.036	SNP
rs2241619;	chr16	66513864	T	C	TK2	5	0.026	SNP
rs9932340	chr16	66752901	G	A	RP11-61A1	6	0.031	SNP
rs7195853	chr16	66827933	G	A	NAE1	6	0.031	SNP
rs1155501:	chr16	68054582	A	G	DUS2	26	0.134	SNP
.	chr16	69241928	CAAAAAAA	CAAAAAAA	RP11-70O5	41	0.216	INDEL
.	chr16	69241928	CAAAAAAA	CAAAAAAA	RP11-70O5	5	0.026	INDEL
.	chr16	69241928	CAAAAAAA	CAAAAAAA	RP11-70O5	7	0.037	INDEL
.	chr16	69241928	CAAAAAAA	C	RP11-70O5	3	0.016	INDEL
rs1131341	chr16	69714966	G	A	NQO1	6	0.031	SNP
rs1431115:	chr16	70654657	C	A	IL34	2	0.01	SNP
rs7776900(	chr16	70657013	C	T	IL34	5	0.026	SNP
rs8046424	chr16	70657086	G	C	IL34	86	0.443	SNP
rs4985556	chr16	70660097	C	A	IL34	24	0.124	SNP
rs3743953;	chr16	71230658	A	G	HYDIN	25	0.129	SNP
rs1488488!	chr16	71922608	AATGCCC	A	IST1	71	0.366	INDEL
rs1475050!	chr16	71933983	G	A	PKD1L3	5	0.026	SNP
769495	chr16	71947511	CTTTG	C	PKD1L3	51	0.263	INDEL
rs4788587	chr16	71967237	G	A	PKD1L3	32	0.165	SNP
rs1259695:	chr16	74315145	G	T	AC009120.	21	0.108	SNP
rs7504601:	chr16	75372570	T	C	CFDP1	6	0.031	SNP
rs1156187:	chr16	75464250	T	C	TMEM170/	3	0.015	SNP

.	chr16	79106618	A	G	RP11-556H	2	0.01	SNP
rs1997192!	chr16	79211976	G	GGTAAA	WWOX	9	0.046	INDEL
rs5671323:	chr16	80632929	T	C	CDYL2	24	0.124	SNP
rs9937731	chr16	80978406	C	A	CMC2	41	0.211	SNP
rs5929900	chr16	80978410	TG	T	CMC2	10	0.052	INDEL
rs3743503	chr16	81022836	T	G	CENPN	21	0.108	SNP
.	chr16	81032554	GT	G	CENPN	7	0.036	INDEL
.	chr16	81032554	GT	GTT	CENPN	6	0.031	INDEL
rs8054182	chr16	81112202	C	G	PKD1L2	193	0.995	SNP
rs1456029!	chr16	81127868	G	GTACTGCC.	PKD1L2	30	0.155	INDEL
.	chr16	81141373	G	A	PKD1L2	186	0.959	SNP
rs5998097	chr16	81149720	T	A	PKD1L2	13	0.067	SNP
rs1292577:	chr16	81165939	G	A	PKD1L2	48	0.247	SNP
rs5598034!	chr16	81208543	GTT	G	PKD1L2	74	0.381	INDEL
rs7499011	chr16	81208593	G	A	PKD1L2	82	0.423	SNP
rs2014608	chr16	81988002	AGAGG	A	RP11-510J:	5	0.026	INDEL
rs1154246:	chr16	82000205	G	A	SDR42E1	20	0.103	SNP
rs1164788:	chr16	82170348	G	T	CTD-2588J(	3	0.015	SNP
rs1169305(	chr16	83397345	C	G	RP11-543N	3	0.015	SNP
rs4782865	chr16	83949466	C	T	RP11-505K	128	0.66	SNP
rs9927826	chr16	84041066	C	G	SLC38A8	18	0.093	SNP
rs3826151;	chr16	84174729	G	T	DNAAF1	53	0.273	SNP
rs4782970;	chr16	84461712	A	C	ATP2C2	3	0.015	SNP
rs8055536	chr16	84495776	G	C	RP11-517C	4	0.021	SNP
rs1859681:	chr16	84842267	T	C	CRISPLD2	2	0.01	SNP
.	chr16	85071765	CTT	CT	KIAA0513	13	0.067	INDEL
.	chr16	85071765	CTT	CTTT	KIAA0513	25	0.129	INDEL
.	chr16	85071765	CTT	C	KIAA0513	3	0.015	INDEL
rs8050680	chr16	85185117	A	G	GSE1	109	0.562	SNP
rs2930230	chr16	85283559	C	T	LINC00311	83	0.428	SNP
.	chr16	85654982	CGG	C	GSE1	2	0.01	INDEL
.	chr16	85654986	CTTCAGGT(	C	GSE1	2	0.01	INDEL
rs1293529!	chr16	85743682	G	C	RP11-568J:	45	0.232	SNP
rs903202	chr16	85914173	T	C	IRF8	76	0.392	SNP
rs1123498:	chr16	86347450	G	C	RP11-158I3	32	0.165	SNP
rs1626257	chr16	86349157	T	A	LINC00917	118	0.608	SNP
rs7789705!	chr16	86349548	T	TA	LINC00917	23	0.119	INDEL
rs5589344:	chr16	88530440	A	T	RP11-21B2	15	0.077	SNP
rs6754943!	chr16	88533287	GGA	G	ZFPM1	194	1	INDEL
rs6732292!	chr16	88533292	CT	C	ZFPM1	194	1	INDEL
rs1488334:	chr16	88653318	T	C	MVD	6	0.031	SNP
.	chr16	88660842	C	A	MVD	3	0.015	SNP
.	chr16	88736406	TACCTGCG	T	PIEZO1	2	0.01	INDEL
rs3523274!	chr16	88835265	G	A	GALNS	3	0.015	SNP
rs1064315;	chr16	88835775	G	A	GALNS	42	0.216	SNP
rs3474533!	chr16	88835791	G	C	GALNS	14	0.072	SNP
rs3427879:	chr16	88841898	G	A	GALNS	3	0.015	SNP

.	chr16	88859980	C	CTACTTTCT	TRAPPC2L	2	0.01	INDEL
rs2270416;	chr16	89195074	C	A	CDH15	7	0.036	SNP
rs3830555	chr16	89584767	T	TC	CPNE7	9	0.046	INDEL
rs1477163:	chr16	89669483	C	CTG	SPATA33	4	0.021	INDEL
rs1259827(	chr16	89762788	G	C	FANCA	19	0.098	SNP
rs886950	chr16	89770464	T	C	FANCA	84	0.433	SNP
.	chr16	89885977	C	CGCCCTTC	TCF25	11	0.057	INDEL
.	chr16	89885977	C	CGCCCTTC	TCF25	7	0.036	INDEL
.	chr16	89885977	C	CGCCCTTC	TCF25	14	0.072	INDEL
rs1512890(	chr16	89971820	C	CA	CENPBD1	3	0.015	INDEL
rs3396965	chr16	89979713	A	G	AFG3L1P	17	0.088	SNP
rs4545640:	chr16	89981919	G	A	AFG3L1P	14	0.072	SNP
rs1410631	chr16	90031264	C	A	GAS8	3	0.015	SNP
rs2302513	chr16	90031340	C	T	GAS8	22	0.113	SNP
.	chr16	90075897	C	CT	PRDM7	2	0.01	INDEL
.	chr17	234378	CGAGCAG(	C	RPH3AL	9	0.048	INDEL
rs3830920	chr17	733026	A	AGGGCC	FAM57A	100	0.515	INDEL
rs7727485:	chr17	896885	C	T	NXN	22	0.113	SNP
rs2230463	chr17	1553024	G	A	PITPNA	26	0.134	SNP
rs1426751	chr17	1562543	C	T	PITPNA	3	0.015	SNP
rs5030749	chr17	1830105	A	G	RPA1	139	0.716	SNP
rs5030755	chr17	1879658	A	G	RPA1	24	0.124	SNP
rs2230930	chr17	1879663	C	T	RPA1	43	0.222	SNP
.	chr17	2694855	A	C	CLUH	3	0.015	SNP
rs7209613	chr17	3468885	A	G	SPATA22	163	0.84	SNP
rs1507700(	chr17	3471678	GCA	G	SPATA22	8	0.041	INDEL
rs1294821	chr17	3494408	C	T	ASPA	61	0.314	SNP
rs1294688:	chr17	3518783	G	A	TRPV3	33	0.17	SNP
rs390804	chr17	3546713	C	T	TRPV3	74	0.381	SNP
rs3215407;	chr17	3690982	TG	T	P2RX5	132	0.68	INDEL
rs7215121	chr17	4558453	G	A	GGT6	70	0.361	SNP
rs1442072(	chr17	4732229	GC	G	RP11-314A	54	0.278	INDEL
.	chr17	4745923	CT	*	ZMYND15	2	0.01	INDEL
.	chr17	4745926	CTG	*	ZMYND15	2	0.01	INDEL
rs3215170	chr17	4785899	AC	A	VMO1	81	0.418	INDEL
.	chr17	4890214	T	*	MINK1	2	0.024	MIXED
.	chr17	4890214	T	TCCCCC	MINK1	32	0.381	MIXED
.	chr17	4890214	T	TCCCCCCC(	MINK1	2	0.024	MIXED
rs3540027	chr17	4900416	G	A	C17orf107	25	0.129	SNP
255466	chr17	4933876	GGAGCCCA	G	GP1BA	17	0.144	INDEL
435345	chr17	4933907	ACCACCCC	*	GP1BA	17	0.12	INDEL
435345	chr17	4933907	ACCACCCC	A	GP1BA	3	0.021	INDEL
435346	chr17	4933925	TCAGAGCC	T	GP1BA	2	0.014	INDEL
.	chr17	4934203	CTA	C	GP1BA	2	0.01	INDEL
.	chr17	5240944	CAACTAAT(	C	RP11-333E	20	0.103	INDEL
.	chr17	5241001	TTGTGACA	*	RP11-333E	95	0.49	INDEL
.	chr17	5241001	TTGTGACA	T	RP11-333E	5	0.026	INDEL

rs5581990	chr17	5241041	C	*	RP11-333E	12	0.063	MIXED
rs5581990	chr17	5241041	C	CA	RP11-333E	8	0.042	MIXED
rs5564598	chr17	5241042	T	*	RP11-333E	12	0.063	SNP
rs5564598	chr17	5241042	T	C	RP11-333E	8	0.042	SNP
rs5601402	chr17	5434961	G	A	C1QBP	3	0.015	SNP
rs7215856	chr17	5536877	T	C	NLRP1	15	0.077	SNP
rs7215868	chr17	5536901	T	C	NLRP1	15	0.077	SNP
rs1165774	chr17	5541923	G	A	NLRP1	15	0.077	SNP
rs1175692	chr17	5584501	C	G	NLRP1	6	0.031	SNP
rs4985639	chr17	5837217	G	A	WSCD1	155	0.799	SNP
.	chr17	6418178	GAA	GAAA	RP11-636N	22	0.113	INDEL
.	chr17	6418178	GAA	GA	RP11-636N	46	0.237	INDEL
.	chr17	6418178	GAA	G	RP11-636N	14	0.072	INDEL
.	chr17	6457592	A	C	PITPNM3	13	0.067	SNP
rs1422941	chr17	6641101	G	A	TXNDC17	2	0.01	SNP
rs4796550	chr17	6758265	T	C	XAF1	77	0.397	SNP
rs3717565	chr17	7014470	CTGTTCCG	C	C17orf49&	15	0.077	INDEL
rs1720328	chr17	7196492	G	A	DLG4	65	0.335	SNP
.	chr17	7234244	CAGTGCTG	C	DVL2	2	0.01	INDEL
rs402514	chr17	7254884	T	G	ELP5	122	0.629	SNP
rs7613258	chr17	7258766	G	A	ELP5	2	0.01	SNP
rs5621910	chr17	7262076	CG	C	CLDN7	120	0.619	INDEL
.	chr17	7329246	CA	C	NEURL4	2	0.01	INDEL
.	chr17	7329256	CGG	C	NEURL4	3	0.015	INDEL
rs3809828	chr17	7343532	C	T	ACAP1	25	0.129	SNP
rs3414079	chr17	7380749	T	G	TNK1	80	0.412	SNP
rs7220814	chr17	7387376	A	G	TNK1	10	0.052	SNP
.	chr17	7436175	ATCCGTTC	A	TMEM102	4	0.021	INDEL
rs7214088	chr17	7482961	G	A	SLC35G6	162	0.835	SNP
.	chr17	7513774	CCA	C	POLR2A	188	1	INDEL
.	chr17	7571837	TTATATAT	TTA	SENP3	4	0.023	INDEL
.	chr17	7571837	TTATATAT	T	SENP3	2	0.011	INDEL
.	chr17	7571837	TTATATAT	TTATA	SENP3	3	0.017	INDEL
.	chr17	7571837	TTATATAT	TTATATAT	SENP3	3	0.017	INDEL
.	chr17	7571842	TATATATA	TTATATA	SENP3	8	0.052	INDEL
.	chr17	7571842	TATATATA	TTATATAT	SENP3	4	0.026	INDEL
.	chr17	7571842	TATATATA	*	SENP3	13	0.084	INDEL
.	chr17	7571842	TATATATA	TTATATAT	SENP3	3	0.019	INDEL
.	chr17	7571842	TATATATA	TTATATAT	SENP3	2	0.013	INDEL
.	chr17	7571870	TATATATA	*	SENP3	30	0.172	INDEL
.	chr17	7571870	TATATATA	T	SENP3	6	0.034	INDEL
.	chr17	7571872	TATATATA	*	SENP3	32	0.184	MIXED
.	chr17	7571872	TATATATA	AATATATA	SENP3	2	0.011	MIXED
.	chr17	7571872	TATATATA	T	SENP3	8	0.046	MIXED
.	chr17	7571874	TATATATA	*	SENP3	36	0.212	MIXED
.	chr17	7571874	TATATATA	T	SENP3	2	0.012	MIXED
.	chr17	7571876	TATATATA	*	SENP3	24	0.14	MIXED

.	chr17	7571876	TATATATA	T	SENP3	2	0.012	MIXED
.	chr17	7571878	TATATATA	*	SENP3	25	0.144	INDEL
.	chr17	7571885	ATATATAT	*	SENP3	24	0.14	INDEL
.	chr17	7571886	TATATATA	*	SENP3	25	0.145	INDEL
.	chr17	7571886	TATATATA	T	SENP3	2	0.012	INDEL
.	chr17	7571888	TATATATA	*	SENP3	27	0.161	INDEL
.	chr17	7571894	TATATATA	*	SENP3	30	0.181	MIXED
.	chr17	7571894	TATATATA	AATATATA	SENP3	5	0.03	MIXED
.	chr17	7571895	ATATATAT	*	SENP3	29	0.177	INDEL
.	chr17	7571895	ATATATAT	A	SENP3	2	0.012	INDEL
.	chr17	7571895	ATATATAT	AATATATA	SENP3	2	0.012	INDEL
.	chr17	7571896	TATATATA	*	SENP3	33	0.204	INDEL
.	chr17	7571896	TATATATA	T	SENP3	2	0.012	INDEL
.	chr17	7571897	ATATATAT	*	SENP3	33	0.201	INDEL
.	chr17	7571897	ATATATAT	A	SENP3	2	0.012	INDEL
.	chr17	7571898	TATATATA	*	SENP3	35	0.211	MIXED
.	chr17	7571898	TATATATA	T	SENP3	4	0.024	MIXED
.	chr17	7571902	TATATATA	*	SENP3	39	0.238	MIXED
.	chr17	7571902	TATATATA	AATATATA	SENP3	6	0.037	MIXED
.	chr17	7571904	TATATAA	*	SENP3	40	0.247	MIXED
.	chr17	7571904	TATATAA	AATATAA	SENP3	3	0.019	MIXED
.	chr17	7571904	TATATAA	T	SENP3	3	0.019	MIXED
.	chr17	7571906	TATAA	*	SENP3	43	0.279	MIXED
.	chr17	7571906	TATAA	T	SENP3	6	0.039	MIXED
.	chr17	7571906	TATAA	AATAA	SENP3	2	0.013	MIXED
rs1107869	chr17	7576574	C	T	EIF4A1	3	0.015	SNP
.	chr17	7586926	TG	T	MPDU1	2	0.01	INDEL
rs13894	chr17	7626584	G	A	SAT2	18	0.093	SNP
.	chr17	7667260	TAA	TA	TP53	73	0.376	INDEL
.	chr17	7667260	TAA	TAAA	TP53	72	0.371	INDEL
.	chr17	7667260	TAA	T	TP53	3	0.015	INDEL
rs7397953	chr17	7667261	A	*	TP53	78	0.402	SNP
rs7397953	chr17	7667261	A	T	TP53	6	0.031	SNP
rs1800372	chr17	7674892	T	C	TP53	3	0.015	SNP
.	chr17	7847928	CAA	C	KDM6B	4	0.021	INDEL
rs1441630	chr17	7857256	C	T	NAA38	4	0.021	SNP
rs7469472	chr17	8039496	C	T	ALOX15B	3	0.015	SNP
rs7658924	chr17	8039583	C	G	ALOX15B	53	0.273	SNP
rs6503070	chr17	8044857	C	T	ALOX15B	105	0.541	SNP
rs1154108	chr17	8047059	C	T	ALOX15B	88	0.454	SNP
rs9898751	chr17	8047634	C	A	ALOX15B	100	0.515	SNP
rs6131286	chr17	8047640	G	A	ALOX15B	10	0.052	SNP
rs7225107	chr17	8048560	A	G	ALOX15B	2	0.01	SNP
rs8067606	chr17	8163465	A	G	VAMP2	119	0.613	SNP
rs8064449	chr17	8175711	G	C	TMEM107	115	0.593	SNP
rs6206072	chr17	10724454	C	T	TMEM220	10	0.052	SNP
774512	chr17	11598539	A	ACGCGGAT	DNAH9	6	0.031	INDEL

.	chr17	12739304	CATAG	C	MYOCD	2	0.01	INDEL
.	chr17	12984651	CGT	C	ARHGAP44	3	0.015	INDEL
rs6205231	chr17	14024776	G	A	CDRT15P1	42	0.216	SNP
rs2159132;	chr17	14102122	G	A	COX10	103	0.531	SNP
rs1418646	chr17	16787977	G	A	SRP68P1	9	0.046	SNP
rs1998294	chr17	16830094	CTG	C	KRT16P2	2	0.032	INDEL
rs3752001	chr17	16925845	C	T	TBC1D27	4	0.021	SNP
rs3506802	chr17	17793784	AGC	A	RAI1	71	0.366	INDEL
rs3506802	chr17	17793784	AGC	*	RAI1	8	0.041	INDEL
587783	chr17	17793787	AGCAG	ACAG	RAI1	71	0.366	INDEL
587783	chr17	17793787	AGCAG	*	RAI1	8	0.041	INDEL
.	chr17	18263316	TG	T	MIEF2	2	0.01	INDEL
rs2230154	chr17	18290627	G	A	TOP3A	62	0.32	SNP
.	chr17	18420181	G	A	KRT17P5	3	0.015	MIXED
rs3764125	chr17	18476849	GGA	G	LGALS9C	67	0.345	INDEL
rs2472693	chr17	18953558	A	G	AC090286.	147	0.758	SNP
.	chr17	19329649	T	A	EPN2	2	0.01	SNP
rs7216;322	chr17	19675560	A	T	ALDH3A2	105	0.541	SNP
rs3874846	chr17	20394232	T	G	CCDC144CI	97	0.5	SNP
rs1068769	chr17	20467469	A	ATC	LGALS9B	47	0.309	INDEL
.	chr17	20501764	CTG	C	KRT16P3	4	0.021	INDEL
rs9910790	chr17	21123445	G	A	DHRS7B	5	0.026	SNP
rs9989453	chr17	22299387	T	G	FAM27E5	55	0.284	SNP
rs1402034	chr17	27241612	C	A	RP11-663N	10	0.052	SNP
rs1993829	chr17	27426561	C	T	TBC1D3P5	102	0.526	SNP
rs9897292	chr17	27655825	A	T	NOS2P1	39	0.201	SNP
rs1457103	chr17	28548659	G	C	UNC119	2	0.01	SNP
rs1431024	chr17	28592479	T	G	SPAG5	2	0.01	SNP
rs1469018	chr17	28723695	CAT	C	RPL23A	3	0.015	INDEL
.	chr17	28849258	CA	C	RP11-20B2	23	0.119	INDEL
.	chr17	28849258	CA	CAA	RP11-20B2	24	0.124	INDEL
rs7891750	chr17	28952456	G	T	PIPOX	2	0.01	SNP
rs1145063	chr17	28963951	T	TG	SEZ6	99	0.51	INDEL
rs497993	chr17	29568510	T	C	TP53I13	137	0.706	SNP
rs7955640	chr17	29941799	G	A	EFCAB5	4	0.021	SNP
rs2011972	chr17	29941839	G	A	EFCAB5	12	0.062	SNP
rs1117801	chr17	30560116	G	A	TBC1D29	23	0.119	SNP
rs1056170	chr17	30633973	CTTTTT	CTTTT	LRRC37BP1	27	0.139	INDEL
rs1056170	chr17	30633973	CTTTTT	CTTTTTT	LRRC37BP1	3	0.015	INDEL
rs1056170	chr17	30633973	CTTTTT	CTTT	LRRC37BP1	16	0.082	INDEL
rs1056170	chr17	30633973	CTTTTT	CTTTTTTCT	LRRC37BP1	15	0.077	INDEL
rs1056170	chr17	30633973	CTTTTT	CTT	LRRC37BP1	4	0.021	INDEL
rs2525574	chr17	31378929	T	C	NF1	120	0.619	SNP
rs1849540	chr17	31379024	G	A	NF1	2	0.01	SNP
rs7114209	chr17	31856644	G	GC	COPRS	178	0.918	INDEL
.	chr17	31966130	CT	C	SUZ12	16	0.082	INDEL
.	chr17	31966130	CT	CTT	SUZ12	39	0.201	INDEL

.	chr17	32142451	CCCGCCGC	CCCG	AC090616.	9	0.046	INDEL
.	chr17	32142451	CCCGCCGC	CCCGCCGC	AC090616.	24	0.124	INDEL
.	chr17	32142451	CCCGCCGC	CCCGCCGC	AC090616.	22	0.113	INDEL
.	chr17	32142451	CCCGCCGC	CCCGCCGC	AC090616.	21	0.108	INDEL
.	chr17	32142451	CCCGCCGC	C	AC090616.	26	0.134	INDEL
.	chr17	32142451	CCCGCCGC	CCCGCCGC	AC090616.	12	0.062	INDEL
.	chr17	33932642	GGCGGAG(	G	CTC-304I17	4	0.021	INDEL
.	chr17	33932642	GGCGGAG(	GAA	CTC-304I17	2	0.01	INDEL
rs8072510	chr17	35445639	G	T	SLFN13	27	0.139	SNP
rs1434710:	chr17	35479822	AG	A	SLFN12L	2	0.01	INDEL
rs4796089	chr17	35487739	T	C	SLFN12L	24	0.124	SNP
.	chr17	35552729	C	T	SLFN14	2	0.01	SNP
rs1767058	chr17	35671885	T	C	AP2B1	14	0.072	SNP
rs3744075	chr17	38918679	C	T	LASP1	13	0.067	SNP
rs1495350:	chr17	39173562	GGT	G	RP5-906A2	8	0.041	INDEL
rs2240083	chr17	39195150	C	T	CACNB1	59	0.304	SNP
.	chr17	39471445	CTCTACCA/	C	CDK12	2	0.01	INDEL
rs2952149	chr17	39660208	T	C	STARD3	7	0.036	SNP
rs2941513	chr17	39662308	T	C	STARD3	7	0.036	SNP
rs5638	chr17	39669996	A	G	PNMT	8	0.041	SNP
rs3434149(	chr17	39670063	G	A	PNMT	2	0.01	SNP
rs1107892	chr17	39908216	T	C	GSDMB	77	0.397	SNP
rs5820318	chr17	40019175	A	AACACAC	MED24	70	0.361	MIXED
rs5820318	chr17	40019175	A	AACACACA	MED24	3	0.015	MIXED
rs5820318	chr17	40019175	A	AACAC	MED24	2	0.01	MIXED
rs3711968	chr17	40019203	C	CACACACA	MED24	39	0.201	INDEL
rs3711968	chr17	40019203	C	CACACACA	MED24	21	0.108	INDEL
rs3711968	chr17	40019203	C	CACACAT	MED24	5	0.026	INDEL
rs3711968	chr17	40019203	C	CACAT	MED24	2	0.01	INDEL
.	chr17	40022659	G	GC	MED24	2	0.01	INDEL
rs1474463:	chr17	40141200	CT	C	CASC3	13	0.067	INDEL
rs1116043(	chr17	40163475	T	TTG	CASC3	3	0.015	INDEL
rs2228015	chr17	40558934	T	C	CCR7	10	0.052	SNP
rs1130987:	chr17	40701882	CA	C	KRT24	92	0.474	INDEL
.	chr17	41084529	CCTGCTGC(	C	KRTAP4-7	10	0.072	INDEL
.	chr17	41168104	GCTCTC	G	KRTAP4-3	3	0.015	INDEL
.	chr17	41168106	T	*	KRTAP4-3	3	0.015	MIXED
rs7115512(	chr17	41184543	ACGGCAG(	A	KRTAP4-1	165	0.851	INDEL
rs6174354(	chr17	41189887	A	G	KRTAP9-1	41	0.211	SNP
rs1128384	chr17	41190343	T	TGCTGTGG	KRTAP9-1	176	0.907	INDEL
rs7283002:	chr17	41449102	C	T	KRT43P	21	0.108	SNP
rs1481683(	chr17	41886139	T	C	ACLY	2	0.01	SNP
rs8065502	chr17	41892360	A	G	ACLY	183	0.943	SNP
rs2005649(	chr17	42011790	AG	A	NKIRAS2	2	0.01	INDEL
rs1172536:	chr17	42101837	C	T	DHX58	2	0.01	SNP
775163	chr17	42118011	ACTGAAGC	A	KAT2A	3	0.015	INDEL
.	chr17	42120933	GCCCCGCC	G	KAT2A	2	0.01	INDEL

.	chr17	42120964	A	*	KAT2A	4	0.021	SNP
.	chr17	42120966	CTTGCAAG	*	KAT2A	4	0.021	INDEL
.	chr17	42126631	CAA	CAAAA	RAB5C	3	0.016	INDEL
.	chr17	42126631	CAA	CA	RAB5C	15	0.081	INDEL
.	chr17	42126631	CAA	C	RAB5C	6	0.032	INDEL
.	chr17	42126631	CAA	CAAA	RAB5C	14	0.075	INDEL
rs2018947!	chr17	42478928	T	TA	ATP6V0A1	4	0.021	INDEL
.	chr17	42502811	GGCCCCAG	G	ATP6V0A1	3	0.015	INDEL
rs3398694:	chr17	42852620	G	A	AOC3	11	0.057	SNP
rs455055	chr17	43015940	C	A	VAT1	134	0.691	SNP
rs1799967;	chr17	43070958	C	T	BRCA1	8	0.041	SNP
rs6503726	chr17	43132725	T	C	NBR2	63	0.325	SNP
rs3562063!	chr17	43520862	G	A	DHX8	2	0.01	SNP
.	chr17	43609867	CT	C	RP11-392O	42	0.216	INDEL
.	chr17	43609867	CT	CTT	RP11-392O	5	0.026	INDEL
rs1483123(	chr17	43774881	G	A	DUSP3	3	0.015	SNP
.	chr17	43884083	C	T	MPP2	35	0.18	SNP
rs7220138	chr17	44176643	C	G	ASB16-AS1	81	0.418	SNP
rs2285953	chr17	44190887	G	C	TMUB2	12	0.062	SNP
rs2271803	chr17	47323542	G	A	EFCAB13	141	0.727	SNP
rs7137730(	chr17	47347921	C	T	EFCAB13	7	0.036	SNP
rs7886564	chr17	47361422	C	T	EFCAB13	3	0.015	SNP
rs7629962(	chr17	47370436	G	A	EFCAB13	2	0.01	SNP
rs7496948!	chr17	47374891	A	T	EFCAB13	2	0.01	SNP
rs1180047	chr17	47391492	T	G	EFCAB13	9	0.046	SNP
rs1136613	chr17	47658609	G	A	KPNB1	4	0.021	SNP
rs1136614	chr17	47673102	T	C	KPNB1	4	0.021	SNP
769921	chr17	48804924	GCTTTCCA/	G	TTLL6	2	0.01	INDEL
rs5820737	chr17	48937289	CT	C	SNF8	53	0.273	INDEL
rs7135361	chr17	50094891	GAGGAT	G	PDK2	72	0.371	INDEL
rs9839	chr17	50097544	C	T	PDK2	68	0.351	SNP
rs2014867!	chr17	50175832	GA	G	SGCA	3	0.015	INDEL
rs1143745!	chr17	50211943	C	CA	RP11-893F:	44	0.227	INDEL
.	chr17	50214083	CTT	CT	RP11-893F:	57	0.294	INDEL
.	chr17	50214083	CTT	C	RP11-893F:	10	0.052	INDEL
.	chr17	50214083	CTT	CTTT	RP11-893F:	2	0.01	INDEL
rs1475929	chr17	50368313	CCTGGTCA	C	MRPL27	72	0.371	INDEL
rs2189712	chr17	50378624	A	T	EME1	32	0.165	SNP
rs2006377	chr17	50547946	GGGGGAG(	G	SPATA20	8	0.041	INDEL
rs1158482	chr17	51166498	G	A	NME2	11	0.057	SNP
rs1165871	chr17	54999625	G	A	STXBP4	137	0.706	SNP
rs1294173!	chr17	55751132	A	C	PCTP	24	0.124	SNP
rs2960062	chr17	55773867	C	T	PCTP	190	0.979	SNP
.	chr17	56499065	T	TCCCCCC	ANKFN1	4	0.021	INDEL
.	chr17	56499065	T	TCCCCC	ANKFN1	2	0.01	INDEL
.	chr17	57450283	GAAAGAA/	GGAAAGA/	MSI2&RP1:	43	0.239	INDEL
.	chr17	57450283	GAAAGAA/	*	MSI2&RP1:	4	0.022	INDEL

.	chr17	57450283	GAAAGAA/	GGAAAGA/	MSI2&RP1:	6	0.033	INDEL
.	chr17	57450283	GAAAGAA/	GGAAAGA/	MSI2&RP1:	2	0.011	INDEL
.	chr17	57450287	GAAAGAA/	*	MSI2&RP1:	49	0.272	INDEL
rs3561769:	chr17	58195140	C	A	EPX	2	0.01	SNP
rs3589705:	chr17	58270865	T	G	MPO	2	0.01	SNP
rs2526375	chr17	58351733	A	G	TSPOAP1-A	23	0.119	SNP
rs1490787(	chr17	58352122	G	T	SUPT4H1	3	0.015	SNP
rs6175810(	chr17	59081191	G	A	TRIM37	2	0.01	SNP
rs1438039(	chr17	59891037	AC	A	TUBD1	11	0.057	INDEL
rs1197060	chr17	60565440	G	A	RP11-15E1	6	0.031	SNP
.	chr17	61411515	C	T	TBX2-AS1	2	0.01	SNP
rs1124244(	chr17	61481810	A	C	TBX4	18	0.093	SNP
rs1761018:	chr17	61590592	G	A	NACA2	27	0.139	SNP
rs1473825	chr17	62354818	A	G	EFCAB3	194	1	SNP
.	chr17	62398025	CAA	CAAA	RPS23P7	110	0.573	INDEL
.	chr17	62398025	CAA	CA	RPS23P7	4	0.021	INDEL
.	chr17	62398025	CAA	C	RPS23P7	2	0.01	INDEL
rs1774615:	chr17	62746863	A	G	43900	76	0.392	SNP
rs4330	chr17	63486300	C	A	ACE	98	0.505	SNP
rs1272074:	chr17	63497005	CT	C	ACE	2	0.01	INDEL
rs2727287	chr17	63827368	G	A	FTSJ3	120	0.619	SNP
.	chr17	63838671	CGCCT	C	SMARCD2	2	0.01	INDEL
rs196912	chr17	64044120	T	C	ERN1	159	0.82	SNP
rs5716610(	chr17	64510162	T	C	CEP95	16	0.082	MIXED
rs5716610(	chr17	64510162	T	TC	CEP95	5	0.026	MIXED
.	chr17	64815246	A	C	PLEKHM1P	28	0.144	SNP
rs1801689	chr17	66214462	A	C	APOH	13	0.067	SNP
rs1903132:	chr17	67992921	A	G	C17orf58	5	0.026	SNP
rs4638	chr17	68043234	A	G	KPNA2	146	0.753	SNP
rs7874649	chr17	68045755	AT	A	KPNA2	48	0.247	INDEL
rs7874649	chr17	68045755	AT	ATT	KPNA2	2	0.01	INDEL
rs3746063	chr17	68256818	TGGTGA	T	AMZ2	2	0.01	INDEL
rs3572385:	chr17	68523716	C	CT	PRKAR1A	41	0.211	INDEL
rs3214623	chr17	68677585	T	TC	LINC01482	53	0.273	INDEL
rs3842375;	chr17	69149049	TGA	T	ABCA10	9	0.046	INDEL
rs1049117:	chr17	69153832	G	A	ABCA10	9	0.046	SNP
rs1107739(	chr17	69179009	T	C	ABCA10	133	0.686	SNP
rs1130826!	chr17	69194395	AACAG	A	ABCA10	9	0.046	INDEL
.	chr17	69196283	CACTTCTC/	C	ABCA10	7	0.036	INDEL
rs1107741	chr17	69215890	A	G	ABCA10	132	0.68	SNP
rs1468512	chr17	69223627	C	T	ABCA10	20	0.103	SNP
rs7224070	chr17	70096903	A	G	KCNJ16	162	0.835	SNP
.	chr17	70100767	G	A	KCNJ16	32	0.165	SNP
.	chr17	72123571	CACCA	C	SOX9	2	0.01	INDEL
.	chr17	72339501	C	A	LINC00511	2	0.01	SNP
.	chr17	73192822	GCCCGCCC	*	COG1	31	0.163	INDEL
.	chr17	73192822	GCCCGCCC	G	COG1	2	0.011	INDEL

.	chr17	73192830	CCGCG	*	COG1	33	0.25	INDEL
.	chr17	73192856	TCGCGCCC	T	COG1	34	0.63	INDEL
.	chr17	73192856	TCGCGCCC	*	COG1	2	0.037	INDEL
rs2022200	chr17	73245182	CGTAA	C	C17orf80	6	0.031	INDEL
rs7284405	chr17	74106878	A	C	RP11-101O	10	0.052	SNP
rs3453568	chr17	74112645	C	T	RP11-101O	38	0.196	SNP
rs1489461	chr17	74213114	C	T	MGC16275	4	0.021	SNP
rs1421919	chr17	74215706	GT	G	TTYH2	5	0.026	INDEL
.	chr17	74541596	TTCTCA	T	CD300C	2	0.01	INDEL
.	chr17	74772073	CAG	C	NAT9	3	0.015	INDEL
rs7136163	chr17	74772558	G	GA	NAT9	23	0.119	INDEL
.	chr17	74893497	CGGTTCCA	CGGTTCCA	FADS6	13	0.067	MIXED
.	chr17	74893524	GGGCTCCG	G	FADS6	16	0.082	INDEL
rs3683670	chr17	74935803	T	C	OTOP3	2	0.01	SNP
rs2019509	chr17	75131663	C	T	NT5C	2	0.01	SNP
rs3400816	chr17	75239397	C	G	GGA3	5	0.026	SNP
rs7874066	chr17	75248849	G	C	GGA3	22	0.113	SNP
rs2002267	chr17	75588737	C	CG	MYO15B	19	0.098	INDEL
rs1179402	chr17	75589275	G	A	MYO15B	19	0.098	SNP
rs7399836	chr17	75590244	G	A	MYO15B	6	0.031	SNP
rs6208922	chr17	75590246	T	G	MYO15B	19	0.098	SNP
rs1452542	chr17	75596487	CTG	C	MYO15B	17	0.088	INDEL
rs3713539	chr17	75600607	CGGAG	C	MYO15B	72	0.462	INDEL
rs1670993	chr17	75607005	T	C	MYO15B	182	0.938	SNP
rs6173528	chr17	75751047	G	A	ITGB4	14	0.072	SNP
rs1043149	chr17	76081716	C	T	ZACN	33	0.17	SNP
rs1119127	chr17	76153122	CA	C	RP11-449J	50	0.258	INDEL
rs1147516	chr17	76153124	G	C	RP11-449J	50	0.258	SNP
rs3744037	chr17	76387394	T	C	SPHK1	34	0.175	SNP
rs1169332	chr17	76387428	G	A	SPHK1	3	0.015	SNP
rs346802	chr17	76387520	C	T	SPHK1	6	0.031	SNP
rs7288310	chr17	76633816	C	G	ST6GALNA	13	0.067	SNP
rs1475034	chr17	77400591	G	A	44083	3	0.015	SNP
rs877157	chr17	77533267	G	C	RP11-13K1	6	0.031	SNP
rs7289629	chr17	78157496	T	C	EIF5AP2	6	0.031	SNP
rs1143696	chr17	78182670	C	T	TK1	6	0.031	SNP
rs1154845	chr17	78976149	G	A	LGALS3BP	4	0.021	SNP
rs2377301	chr17	78998287	C	T	CANT1	12	0.062	SNP
rs3758582	chr17	80090152	CAACACAG	C	CCDC40	2	0.01	INDEL
.	chr17	80090349	GAC	G	CCDC40	2	0.01	INDEL
rs6080743	chr17	80090387	A	*	CCDC40	42	0.216	MIXED
rs5624184	chr17	80090397	T	*	CCDC40	42	0.216	SNP
rs2241886	chr17	80140033	G	A	EIF4A3	37	0.191	SNP
rs7495192	chr17	80191403	C	T	CARD14	7	0.036	SNP
rs1165846	chr17	80192586	C	T	CARD14	20	0.103	SNP
rs6262023	chr17	80210802	C	T	SGSH	4	0.021	SNP
rs3554908	chr17	80415678	G	A	ENDOV	10	0.052	SNP

rs4129871:	chr17	80422204	A	G	ENDOV	5	0.026	SNP
.	chr17	80960744	ACTGAGCA	A	CTD-2561B	9	0.046	INDEL
rs3482416(	chr17	80969713	C	T	CTD-2561B	6	0.031	SNP
rs1178500(	chr17	81230241	G	T	AC027601.	3	0.015	SNP
.	chr17	81238825	CG	C	TEPSIN	2	0.01	INDEL
.	chr17	81251213	TTTTAAAG(	T	SLC38A10	15	0.077	INDEL
rs1460492(	chr17	81389325	T	C	RP11-1055	2	0.01	SNP
rs1140892	chr17	81510330	C	T	ACTG1	2	0.01	SNP
rs2009258(	chr17	81528661	C	T	FSCN2	2	0.01	SNP
rs9906358	chr17	81647455	A	G	TSPAN10	79	0.407	SNP
.	chr17	81647906	T	TTAAC	TSPAN10	76	0.392	INDEL
rs6024388:	chr17	81653317	CG	C	PDE6G	16	0.082	INDEL
rs1043881	chr17	81670319	T	C	CCDC137	187	0.964	SNP
rs2070871	chr17	81847258	G	A	P4HB	39	0.201	SNP
rs7900183	chr17	81923417	C	G	MAFG	4	0.021	SNP
rs7906645	chr17	81954288	G	A	NOTUM	11	0.057	SNP
rs7582655	chr17	82019339	G	A	STRA13	2	0.01	SNP
rs1400854(	chr17	82022838	G	T	STRA13	3	0.015	SNP
rs4969478	chr17	82033727	C	T	RAC3	8	0.041	SNP
rs6173974(	chr17	82036724	C	T	DCXR	24	0.124	SNP
rs1133641(	chr17	82037614	C	CTT	DCXR	24	0.124	INDEL
rs1442122!	chr17	82083297	G	A	FASN	2	0.01	SNP
rs2229425	chr17	82083590	G	A	FASN	29	0.149	SNP
rs2229422	chr17	82085863	A	G	FASN	60	0.309	SNP
rs2228309	chr17	82093307	A	G	FASN	99	0.51	SNP
rs1165366:	chr17	82113574	G	A	CCDC57	44	0.227	SNP
.	chr17	82160327	AAGACAGC	A	RP11-1376	92	0.474	INDEL
rs5618160!	chr17	82251406	G	A	CSNK1D	2	0.01	SNP
rs1205165	chr17	82459274	G	T	NARF	68	0.351	SNP
rs2003930:	chr17	82797739	CT	CTT	TBCD	55	0.284	INDEL
rs2003930:	chr17	82797739	CT	C	TBCD	12	0.062	INDEL
rs2003930:	chr17	82797739	CT	CTTT	TBCD	2	0.01	INDEL
.	chr18	45883	GTGGGGG/	G	FAM166AP	33	0.172	INDEL
rs2009766:	chr18	48615	CCT	C	TUBB8P12	3	0.015	INDEL
.	chr18	109387	GATGCAAC	G	ROCK1P1	20	0.103	INDEL
rs3217715	chr18	690702	A	AAGCTGTT	ENOSF1	34	0.175	INDEL
rs2846474:	chr18	1656496	G	A	CTD-2015H	72	0.371	SNP
rs1143658;	chr18	3067544	G	C	MYOM1	2	0.01	SNP
rs1143657;	chr18	3075748	G	A	MYOM1	40	0.206	SNP
rs3765623;	chr18	3086067	C	T	MYOM1	6	0.031	SNP
rs3501277:	chr18	3262786	C	T	MYL12B	135	0.696	SNP
rs238533;6	chr18	3451764	C	T	TGIF1	29	0.149	SNP
rs1157151(	chr18	3452224	CT	C	TGIF1	62	0.32	INDEL
rs1380891(	chr18	3456681	GAC	G	TGIF1	11	0.057	INDEL
rs2298544	chr18	5570219	T	C	RP11-286N	62	0.32	SNP
rs1172953:	chr18	8018096	G	A	PTPRM	2	0.01	SNP
rs632423	chr18	8809447	G	C	MTCL1	54	0.278	SNP

rs1390097	chr18	9104206	G	GC	NDUFV2	18	0.093	INDEL
.	chr18	9132221	AC	A	NDUFV2-AS	4	0.021	INDEL
rs1052641	chr18	9844832	AGTTTTGT	A	RAB31&RN	130	0.67	INDEL
rs1052641	chr18	9844832	AGTTTTGT	AGTTTTGT	RAB31&RN	29	0.149	INDEL
rs1052641	chr18	9844832	AGTTTTGT	AGTTTTGT	RAB31&RN	22	0.113	INDEL
.	chr18	9887391	GGAAGCCA	G	TXNDC2	17	0.088	INDEL
rs659485	chr18	11885913	C	A	MPPE1	12	0.062	SNP
rs8097892	chr18	11905954	C	T	MPPE1	86	0.443	SNP
rs1295627	chr18	12014326	G	A	IMPA2	6	0.031	SNP
.	chr18	12211820	CTTTTTTT	C	RP11-64C1	28	0.144	INDEL
.	chr18	12211820	CTTTTTTT	CT	RP11-64C1	48	0.247	INDEL
.	chr18	12211820	CTTTTTTT	CTTTTTTTT	RP11-64C1	44	0.227	INDEL
.	chr18	12211820	CTTTTTTT	CTTTTTTTT	RP11-64C1	22	0.113	INDEL
.	chr18	12211820	CTTTTTTT	CTTTTTT	RP11-64C1	7	0.036	INDEL
.	chr18	12211820	CTTTTTTT	CTTTTTTTT	RP11-64C1	6	0.031	INDEL
rs5712250	chr18	12213598	A	G	RP11-64C1	78	0.402	SNP
rs7136991	chr18	12254563	C	CCCGCGCA	CIDEA	91	0.469	INDEL
rs3493937	chr18	12254957	G	A	CIDEA	41	0.211	SNP
.	chr18	12437550	ATTT	AT	RP11-861E	99	0.51	INDEL
.	chr18	12437550	ATTT	ATT	RP11-861E	19	0.098	INDEL
.	chr18	12437550	ATTT	A	RP11-861E	18	0.093	INDEL
rs1245526	chr18	12686674	G	A	PSMG2	62	0.32	SNP
rs1487546	chr18	13491470	G	T	LDLRAD4	3	0.015	SNP
788831	chr18	14105166	CTTCTCTCC	C	RP11-411B	3	0.015	INDEL
rs1176577	chr18	14188004	G	A	ANKRD20A	9	0.046	SNP
rs5608523	chr18	21042578	G	A	ROCK1	6	0.031	SNP
rs4562784	chr18	23134671	G	A	CABLES1	6	0.031	SNP
.	chr18	24148698	CTCAGGAG	C	CABYR	2	0.01	INDEL
rs7255796	chr18	26862263	C	T	AQP4	4	0.021	SNP
rs1944294	chr18	28036487	A	T	CDH2	27	0.139	SNP
rs2000560	chr18	31068033	T	TTC	DSC2	2	0.01	INDEL
rs1449080	chr18	31556555	A	C	DSG2-AS1	103	0.531	SNP
rs2289544	chr18	32192796	G	A	MEP1B	8	0.041	SNP
rs7136137	chr18	32202990	A	G	MEP1B	4	0.021	SNP
rs4799640	chr18	32208144	G	A	MEP1B	52	0.268	SNP
rs1914995	chr18	40082650	A	T	LINC01477	3	0.015	SNP
.	chr18	41564899	C	T	KC6	2	0.01	SNP
rs3420513	chr18	42004448	T	C	PIK3C3	20	0.103	SNP
rs3085861;	chr18	44876705	C	CTCTT	SETBP1	110	0.567	INDEL
rs8083998	chr18	46093178	A	C	ATP5A1	79	0.407	SNP
rs1166544	chr18	46555180	T	C	LOXHD1	130	0.67	SNP
rs3602459	chr18	46657032	A	T	LOXHD1	5	0.026	SNP
rs9973035	chr18	46946874	T	C	KATNAL2	9	0.046	SNP
.	chr18	46963135	T	TGC	TCEB3CL2	6	0.043	INDEL
.	chr18	46969062	T	TGC	TCEB3CL	29	0.204	INDEL
rs3567887	chr18	47098221	C	CA	KATNAL2	9	0.046	INDEL
rs3567887	chr18	47098221	C	CAA	KATNAL2	5	0.026	INDEL

rs2872345(	chr18	47574947	G	C	CTD-2130C	25	0.129	SNP
rs7482396!	chr18	49321379	T	G	DYM	19	0.098	SNP
rs1474385:	chr18	51196775	CGCCGCCG	C	MEX3C	73	0.376	INDEL
.	chr18	51613108	CTTTTTTTT	CTTTTTTTT	LINC01630.	3	0.016	INDEL
.	chr18	51613108	CTTTTTTTT	CTTTTTTTT	LINC01630.	21	0.109	INDEL
.	chr18	51613108	CTTTTTTTT	CTTTTTTTT	LINC01630.	38	0.198	INDEL
.	chr18	51613108	CTTTTTTTT	CTTTTTTTT	LINC01630.	8	0.042	INDEL
.	chr18	51613108	CTTTTTTTT	CT	LINC01630.	3	0.016	INDEL
.	chr18	51613108	CTTTTTTTT	C	LINC01630.	8	0.042	INDEL
rs6208350(	chr18	52338447	C	T	RP11-25O3	4	0.021	SNP
rs1729272!	chr18	54354519	G	A	STARD6	3	0.015	SNP
rs2080681	chr18	56721671	T	G	WDR7	160	0.825	SNP
.	chr18	57641542	CTT	CT	RP11-35G9	34	0.175	INDEL
.	chr18	57641542	CTT	CTTT	RP11-35G9	2	0.01	INDEL
.	chr18	57641542	CTT	C	RP11-35G9	4	0.021	INDEL
.	chr18	58696337	ATT	AT	MALT1	9	0.049	INDEL
.	chr18	58696337	ATT	ATTT	MALT1	11	0.06	INDEL
.	chr18	58696337	ATT	A	MALT1	9	0.049	INDEL
.	chr18	58696337	ATT	ATTTTT	MALT1	2	0.011	INDEL
rs2007887	chr18	59052668	A	G	OACYLP	29	0.149	SNP
rs1087176:	chr18	59083781	T	C	RP11-685A	174	0.897	SNP
rs1499620(	chr18	59230435	TGAAG	T	GRP	7	0.036	INDEL
rs1127220;	chr18	59355522	T	C	LMAN1	56	0.289	SNP
rs1260814(	chr18	59824674	C	T	RP11-824M	44	0.227	SNP
rs6650731	chr18	60804343	C	T	RP11-325K	2	0.01	SNP
.	chr18	62275513	AT	ATT	KIAA1468	47	0.242	INDEL
.	chr18	62275513	AT	A	KIAA1468	5	0.026	INDEL
rs1455555	chr18	63503549	A	G	SERPINB5	110	0.567	SNP
.	chr18	63712604	G	T	SERPINB11	146	0.753	SNP
rs6174007!	chr18	63720043	C	T	SERPINB11	7	0.036	SNP
rs6103	chr18	63903269	C	G	SERPINB2	51	0.263	SNP
rs6104	chr18	63903295	C	G	SERPINB2	51	0.263	SNP
.	chr18	65866018	G	GAAGTACC	RP11-389J2	8	0.066	INDEL
.	chr18	65866018	G	GAAGTACC	RP11-389J2	8	0.066	INDEL
.	chr18	65866018	G	GAAGTACC	RP11-389J2	4	0.033	INDEL
rs1477966!	chr18	65866020	C	A	RP11-389J2	2	0.021	SNP
rs1436278(	chr18	68691277	TAA	T	TMX3	18	0.093	INDEL
rs1404148:	chr18	68846375	G	A	CCDC102B	3	0.015	SNP
rs1127133	chr18	69659822	TTATATAT/	T	DOK6&RP1	18	0.127	INDEL
rs7237740	chr18	74495925	C	T	CNDP2	83	0.428	SNP
rs2278160	chr18	74509174	A	T	CNDP2	2	0.01	SNP
rs5826329	chr18	74518940	T	TC	CNDP2	140	0.722	INDEL
rs6191071	chr18	75201965	C	T	ZADH2	5	0.026	SNP
rs7230037	chr18	75202325	G	A	ZADH2	88	0.454	SNP
rs1115090(	chr18	75712015	C	T	RP11-704C	55	0.284	SNP
rs1051420:	chr18	76127016	C	T	RP11-1O2.:	43	0.222	SNP
rs1429078	chr18	77016816	A	G	MBP	4	0.021	SNP

rs5639521	chr18	77095552	T	A	MBP	14	0.072	SNP
rs2016994	chr18	79193239	C	T	ATP9B	2	0.01	SNP
.	chr19	110747	G	GT	OR4F17	3	0.015	INDEL
rs4897951	chr19	330263	C	T	MIER2	14	0.072	SNP
.	chr19	464140	C	CG	ODF3L2	2	0.01	INDEL
rs6109556	chr19	490040	G	A	MADCAM1	4	0.021	SNP
.	chr19	501697	T	TCCCAACA	MADCAM1	17	0.088	MIXED
rs7297025	chr19	501701	G	GACACCAC	MADCAM1	77	0.397	MIXED
rs7297025	chr19	501701	G	A	MADCAM1	5	0.026	MIXED
rs7297025	chr19	501701	G	GGAGCCTC	MADCAM1	17	0.088	MIXED
rs2003983	chr19	549171	C	T	GZMM	2	0.01	SNP
rs1054786	chr19	613898	T	C	HCN2	122	0.629	SNP
rs7780940	chr19	621070	G	A	POLRMT	18	0.105	SNP
rs6213130	chr19	735111	G	A	PALM	33	0.181	SNP
rs3170451	chr19	804642	C	T	PTBP1	48	0.247	SNP
.	chr19	868325	CAG	C	MED16	2	0.01	INDEL
rs3217342	chr19	868340	G	GGTAGCTG	MED16	12	0.062	INDEL
.	chr19	1004590	CAT	C	GRIN3B	2	0.01	INDEL
rs1066658	chr19	1004897	G	GCGTT	GRIN3B	63	0.325	INDEL
rs3752229	chr19	1041353	A	G	ABCA7	12	0.062	SNP
.	chr19	1041588	CTGGAGCA	C	ABCA7	2	0.01	INDEL
rs1121310	chr19	1066626	AGACGGA	A	ARHGAP45	22	0.113	INDEL
rs1132986	chr19	1236081	A	G	CBARP	5	0.026	SNP
rs3732669	chr19	1267293	G	A	CIRBP	2	0.01	SNP
rs1785158	chr19	1397444	G	A	GAMT	14	0.072	SNP
rs1443580	chr19	1474933	TG	T	C19orf25	9	0.046	INDEL
rs806528	chr19	1490421	A	C	PCSK4	62	0.32	SNP
rs1085395	chr19	1534043	T	C	PLK5	43	0.222	SNP
rs6048262	chr19	1783028	T	C	ATP8B3	10	0.052	SNP
.	chr19	1799764	C	CA	ATP8B3	6	0.031	INDEL
rs1155045	chr19	1979387	C	T	CSNK1G2	13	0.067	SNP
rs7251590	chr19	1989868	G	A	AC005306.	12	0.062	SNP
.	chr19	1991915	G	GT	BTBD2	3	0.015	INDEL
rs7250822	chr19	2255312	C	G	JSRP1	2	0.01	SNP
rs7117654	chr19	2330275	A	ATTTT	SPPL2B	87	0.448	INDEL
rs7117654	chr19	2330275	A	ATTTTTTT	SPPL2B	13	0.067	INDEL
rs7117654	chr19	2330275	A	ATTT	SPPL2B	20	0.103	INDEL
rs7117654	chr19	2330275	A	ATTTTTT	SPPL2B	7	0.036	INDEL
rs7117654	chr19	2330275	A	ATTTTT	SPPL2B	5	0.026	INDEL
rs2043336	chr19	2915740	T	A	ZNF57	177	0.912	SNP
rs3569917	chr19	2936537	G	A	ZNF77	14	0.072	SNP
rs5591979	chr19	3047562	C	T	TLE2	137	0.706	SNP
rs1781004	chr19	3855479	T	C	ZFR2	60	0.309	SNP
rs1998012	chr19	4207296	T	C	ANKRD24	6	0.031	SNP
rs7965764	chr19	4328758	G	C	STAP2	4	0.021	SNP
.	chr19	4343629	C	CGGGGCG	MPND	42	0.216	INDEL
.	chr19	4343629	C	CGGGGCG	MPND	2	0.01	INDEL

rs1040711	chr19	4470614	C	T	CTB-50L17.	8	0.041	SNP
.	chr19	4511363	CCA	C	PLIN4	31	0.16	INDEL
.	chr19	4511367	G	GAC	PLIN4	31	0.16	INDEL
rs7262052	chr19	4659912	C	T	MYDGF	10	0.052	SNP
rs1380885	chr19	4722194	C	T	DPP9	2	0.01	SNP
rs2123731	chr19	4929461	A	G	UHRF1	39	0.201	SNP
rs2250978	chr19	4945962	T	C	UHRF1	180	0.928	SNP
rs2307213	chr19	4950758	C	T	UHRF1	69	0.356	SNP
rs2230611	chr19	5212471	G	A	PTPRS	15	0.077	SNP
rs6175781	chr19	5245966	G	A	PTPRS	2	0.01	SNP
rs1141371	chr19	5273560	T	G	PTPRS	65	0.335	SNP
rs1420688	chr19	5692076	C	T	LONP1	2	0.01	SNP
rs1062373	chr19	5692181	C	T	LONP1	16	0.082	SNP
rs8033632	chr19	6375357	A	G	PSPN	10	0.052	SNP
rs4561663	chr19	6476076	C	T	DENND1C	7	0.036	SNP
.	chr19	6553958	CT	C	CTD-2396E	41	0.211	INDEL
.	chr19	6553958	CT	CTT	CTD-2396E	13	0.067	INDEL
rs7417481	chr19	6562238	C	CT	CLIC4P2	139	0.732	INDEL
rs1178194	chr19	6826619	G	A	VAV1	4	0.021	SNP
rs330880	chr19	6897453	C	G	ADGRE1	83	0.428	SNP
.	chr19	6921702	GT	GTT	ADGRE1	52	0.268	INDEL
.	chr19	6921702	GT	G	ADGRE1	2	0.01	INDEL
.	chr19	7612073	CGAAA	C	CAMSAP3	3	0.015	INDEL
rs1127881	chr19	7639929	TATGTGTG	T	STXBP2	65	0.335	INDEL
rs3719004	chr19	7639992	ATG	A	STXBP2	4	0.021	INDEL
rs1411909	chr19	7640132	ATG	A	STXBP2	54	0.303	INDEL
rs2021019	chr19	7640173	CGTCT	C	STXBP2	8	0.048	INDEL
.	chr19	7640202	GTGTA	G	STXBP2	2	0.011	INDEL
rs3709135	chr19	7640231	CGT	C	STXBP2	3	0.016	INDEL
rs3504205	chr19	7640350	C	CTG	STXBP2	76	0.392	INDEL
rs3736269	chr19	7640414	ATG	ATGTG	STXBP2	74	0.381	INDEL
rs7693727	chr19	7640497	CATCTGTG	C	STXBP2	22	0.113	INDEL
.	chr19	7640517	TGTGCATG	T	STXBP2	2	0.01	INDEL
rs1177618	chr19	7643172	C	T	STXBP2	2	0.01	SNP
rs3549040	chr19	7647401	G	C	STXBP2	2	0.01	SNP
rs8102872	chr19	7689308	C	T	FCER2	7	0.036	SNP
rs2228138	chr19	7690170	G	A	FCER2	54	0.278	SNP
rs2303701	chr19	7910701	C	T	MAP2K7	4	0.021	SNP
.	chr19	7922759	AGGTGAG	A	SNAPC2	8	0.041	INDEL
rs1117512	chr19	8334065	CTCGCTGT	C	KANK3	74	0.381	INDEL
rs968502	chr19	8502591	T	C	PRAM1	35	0.18	SNP
.	chr19	8896085	CGG	C	MUC16	12	0.062	INDEL
.	chr19	8910509	C	A	MUC16	17	0.088	SNP
rs7705723	chr19	8910510	T	G	MUC16	17	0.088	SNP
rs1112795	chr19	9126022	G	GATGGT	OR7G3	63	0.325	INDEL
rs7526699	chr19	9126240	AG	A	OR7G3	14	0.072	INDEL
rs5583749	chr19	9341350	ACT	A	ZNF559	119	0.613	INDEL

.	chr19	9413595	CT	C	ZNF266	3	0.015	INDEL
rs1120142	chr19	9690764	TCTCTC	T	ZNF812P	6	0.031	INDEL
rs7783389	chr19	10021324	G	A	RDH8	20	0.103	SNP
rs1499916	chr19	10119050	TGGCAGTC	T	EIF3G	73	0.376	INDEL
rs3745262	chr19	10328864	A	G	RAVER1	4	0.021	SNP
rs3745261	chr19	10329035	A	G	RAVER1	4	0.021	SNP
rs3501880	chr19	10354167	G	A	TYK2	2	0.01	SNP
rs5576274	chr19	10378250	C	T	TYK2	7	0.036	SNP
rs2011687	chr19	10463871	C	T	PDE4A	2	0.01	SNP
rs3217392	chr19	10572874	CAG	C	AP1M2	16	0.082	INDEL
rs1502889	chr19	11326797	T	TG	TSPAN16	42	0.216	INDEL
.	chr19	11337325	C	G	RAB3D	2	0.01	SNP
rs7873875	chr19	11348989	G	GA	CCDC159	30	0.155	INDEL
rs8108598	chr19	11507951	A	C	ECSIT	8	0.041	SNP
rs1168936	chr19	11510616	C	T	ECSIT	9	0.046	SNP
rs7663950	chr19	11578074	A	T	ACP5	25	0.129	SNP
rs6136978	chr19	12034830	CT	C	ZNF433	8	0.041	INDEL
rs1188071	chr19	12046080	A	G	CTD-2006C	8	0.041	SNP
rs3726284	chr19	12076155	ATTATC	A	ZNF844	3	0.015	INDEL
rs8109273	chr19	12113357	T	C	ZNF788	8	0.041	SNP
rs2855984	chr19	12430157	T	A	ZNF443	33	0.17	SNP
.	chr19	12430727	C	CTG	ZNF443	8	0.041	INDEL
.	chr19	12430728	CAA	TAA	ZNF443	38	0.196	MIXED
.	chr19	12430728	CAA	C	ZNF443	8	0.041	MIXED
rs4804711	chr19	12506587	A	C	MTATP6P2	140	0.722	SNP
rs1298146	chr19	12506671	A	G	MTATP6P2	140	0.722	SNP
rs1382758	chr19	12826502	T	G	HOOK2	2	0.01	SNP
.	chr19	13144423	AG	A	STX10	3	0.015	INDEL
rs2000632	chr19	13765090	G	A	MRI1	2	0.01	SNP
rs1382454	chr19	13773815	G	A	CTB-5E10.3	2	0.01	SNP
.	chr19	13788227	CTTTTTTTT	CTTT	AC008686.	5	0.026	INDEL
.	chr19	13788227	CTTTTTTTT	CTTTT	AC008686.	28	0.144	INDEL
.	chr19	13788227	CTTTTTTTT	CTT	AC008686.	95	0.49	INDEL
.	chr19	13788227	CTTTTTTTT	CTTTTTT	AC008686.	24	0.124	INDEL
.	chr19	13788227	CTTTTTTTT	C	AC008686.	7	0.036	INDEL
.	chr19	13788227	CTTTTTTTT	CT	AC008686.	18	0.093	INDEL
.	chr19	13931963	C	A	PODNL1	3	0.015	SNP
.	chr19	14054992	G	GGC	PALM3	2	0.01	INDEL
.	chr19	14090090	G	GCTGT	SAMD1	2	0.01	INDEL
.	chr19	14090093	C	CGG	SAMD1	2	0.016	INDEL
.	chr19	14090099	G	GCAGC	SAMD1	2	0.01	INDEL
.	chr19	14090108	C	CCGGGCGG	SAMD1	2	0.013	INDEL
rs3541638	chr19	14467895	G	A	PKN1	5	0.026	SNP
rs1042728	chr19	14470251	A	G	PKN1	13	0.067	SNP
.	chr19	14471083	CTT	CT	PKN1	51	0.263	INDEL
.	chr19	14471083	CTT	CTTT	PKN1	7	0.036	INDEL
.	chr19	14471083	CTT	C	PKN1	8	0.041	INDEL

rs7300284:	chr19	14536696	T	A	TECR	101	0.521	SNP
.	chr19	14765708	C	T	ADGRE2	2	0.01	SNP
rs1494265!	chr19	15125068	A	G	ILVBL	4	0.021	SNP
rs2366057	chr19	15480241	A	G	PGLYRP2	110	0.567	SNP
.	chr19	15678330	G	A	CYP4F12	10	0.052	SNP
rs2233144	chr19	16111928	C	T	RAB8A	3	0.015	SNP
rs1043308	chr19	16127469	C	T	RAB8A	58	0.299	SNP
rs2837275!	chr19	16197975	T	TCGCTGCC(	AP1M1	15	0.077	INDEL
rs1139628:	chr19	16628703	G	GC	MED26	10	0.052	INDEL
rs1124186	chr19	17274565	CT	C	USHBP1	33	0.17	INDEL
rs1396158	chr19	17281821	G	GC	ANKLE1	11	0.057	MIXED
.	chr19	17286647	GGTGTGTG	G	ANKLE1	5	0.026	INDEL
.	chr19	17286647	GGTGTGTG	GGTGTGTG	ANKLE1	10	0.052	INDEL
.	chr19	17286647	GGTGTGTG	GGTGTGTG	ANKLE1	2	0.01	INDEL
.	chr19	17286647	GGTGTGTG	GGTGTGTG	ANKLE1	5	0.026	INDEL
.	chr19	17286666	G	*	ANKLE1	6	0.031	MIXED
.	chr19	17286668	G	GTGTT	ANKLE1	4	0.021	INDEL
.	chr19	17286672	GTGTGTGT	TTGTGTGT(	ANKLE1	55	0.286	MIXED
.	chr19	17286676	GTGTGTGT	*	ANKLE1	2	0.01	INDEL
.	chr19	17286676	GTGTGTGT	G	ANKLE1	4	0.021	INDEL
.	chr19	17286678	GTGTGTGT	*	ANKLE1	3	0.015	INDEL
.	chr19	17286678	GTGTGTGT	G	ANKLE1	33	0.17	INDEL
402368	chr19	17286684	GTGTGTGT	*	ANKLE1	35	0.18	MIXED
402368	chr19	17286684	GTGTGTGT	TTGTGTGT	ANKLE1	13	0.067	MIXED
402368	chr19	17286684	GTGTGTGT	G	ANKLE1	3	0.015	MIXED
rs1060640(	chr19	17286688	GTGTT	*	ANKLE1	47	0.242	MIXED
rs1060640(	chr19	17286688	GTGTT	G	ANKLE1	29	0.149	MIXED
rs1060640(	chr19	17286688	GTGTT	TTGTT	ANKLE1	3	0.015	MIXED
.	chr19	17286690	GTT	*	ANKLE1	72	0.371	MIXED
.	chr19	17286690	GTT	G	ANKLE1	24	0.124	MIXED
rs1465582	chr19	17286692	T	G	ANKLE1	52	0.274	MIXED
rs1465582	chr19	17286692	T	TTGTGTGT(	ANKLE1	2	0.011	MIXED
rs1465582	chr19	17286692	T	*	ANKLE1	105	0.553	MIXED
rs2007579(	chr19	17719268	AAATG	A	MAP1S	4	0.021	INDEL
rs6167157!	chr19	17719968	T	G	MAP1S	11	0.057	SNP
.	chr19	17998328	CGG	C	KCNN1	2	0.01	INDEL
rs393548;4	chr19	18086934	T	A	IL12RB1	51	0.263	SNP
rs1456233:	chr19	18145130	C	T	MAST3	6	0.031	SNP
rs1042050	chr19	18218430	T	C	PDE4C	125	0.644	SNP
rs2229228	chr19	18218974	C	T	PDE4C	71	0.366	SNP
rs5827400	chr19	18220547	AC	A	PDE4C	125	0.644	INDEL
rs2232965	chr19	18316140	T	C	LSM4	139	0.716	SNP
rs7878357!	chr19	18433867	G	GC	SSBP4	194	1	INDEL
.	chr19	18614002	CTG	C	TMEM59L	3	0.015	INDEL
.	chr19	18938723	CTGA	C	HOMER3	2	0.01	INDEL
rs1297956!	chr19	19039374	C	T	ARMC6	137	0.706	SNP
rs7117251	chr19	19680067	CAT	C	ZNF101	7	0.036	INDEL

.	chr19	19926078	ATT	AT	ZNF93	30	0.155	INDEL
.	chr19	19926078	ATT	A	ZNF93	6	0.031	INDEL
.	chr19	19926078	ATT	ATTT	ZNF93	15	0.077	INDEL
rs7254200	chr19	19926128	A	G	ZNF93	9	0.046	SNP
.	chr19	20624327	GGCTTTGC	G	ZNF626	5	0.026	INDEL
rs3557580:	chr19	20624371	G	GA	ZNF626	86	0.672	MIXED
rs3557580:	chr19	20624371	G	*	ZNF626	5	0.039	MIXED
.	chr19	20799061	C	CT	ZNF66	13	0.072	INDEL
.	chr19	20799061	C	CTT	ZNF66	2	0.011	INDEL
rs7417237(	chr19	20799064	TC	T	ZNF66	4	0.023	INDEL
.	chr19	20799065	CT	TT	ZNF66	94	0.595	MIXED
.	chr19	20799065	CT	CTT	ZNF66	13	0.082	MIXED
.	chr19	20799065	CT	*	ZNF66	4	0.025	MIXED
rs7248410	chr19	21109642	C	A	ZNF714	189	0.974	SNP
.	chr19	21125078	CA	C	ZNF714	25	0.129	INDEL
.	chr19	21125078	CA	CAA	ZNF714	20	0.103	INDEL
rs1996797:	chr19	21537632	CA	C	ZNF429	2	0.01	INDEL
rs1409381!	chr19	21812018	G	A	ZNF43	11	0.057	SNP
rs3599974(	chr19	21933213	A	T	ZNF208	3	0.015	SNP
rs1260930	chr19	22514375	G	A	ZNF98	37	0.191	SNP
rs2304141	chr19	22603218	G	T	GOLGA2P9	35	0.18	SNP
rs3704100(	chr19	22634475	GT	G	ZNF492	20	0.103	INDEL
rs7135598!	chr19	22663799	G	T	ZNF492	5	0.026	SNP
rs2019701	chr19	22756205	T	G	ZNF99	4	0.021	SNP
.	chr19	22756224	TG	T	ZNF99	2	0.01	INDEL
.	chr19	22756653	GTTTCATA/	G	ZNF99	10	0.052	INDEL
.	chr19	22756662	GGGTCGA(	G	ZNF99	10	0.052	INDEL
rs2005181	chr19	22757390	A	AG	ZNF99	15	0.077	INDEL
rs6139775!	chr19	23744939	CCA	C	ZNF681	25	0.129	INDEL
rs1041520!	chr19	28612156	T	G	AC079466.	2	0.01	SNP
rs7941638!	chr19	28873799	T	C	AC007795.	7	0.036	SNP
rs7846924:	chr19	29412968	T	A	CTC-525D6	6	0.031	SNP
rs1428127	chr19	31360363	G	GT	AC007796.	29	0.149	INDEL
rs7932341(	chr19	31592344	T	C	AC011525.	6	0.031	SNP
rs4553863:	chr19	32604407	G	A	ANKRD27	2	0.01	SNP
rs7248273	chr19	32605910	C	T	ANKRD27	25	0.129	SNP
rs6759765	chr19	32826678	AAC	A	TDRD12	63	0.325	INDEL
rs745961	chr19	32879164	A	G	CEP89	49	0.253	SNP
rs2304103	chr19	32976507	C	T	FAAP24	5	0.026	SNP
rs3721206	chr19	34533668	CT	C	RPS26P55	24	0.124	INDEL
.	chr19	34608684	T	TATATATA1	SCGB2B2	8	0.041	INDEL
.	chr19	34608684	T	TATATATA1	SCGB2B2	8	0.041	INDEL
.	chr19	34608684	T	TATATATA1	SCGB2B2	4	0.021	INDEL
.	chr19	34608684	T	TATATATA1	SCGB2B2	2	0.01	INDEL
.	chr19	34608684	T	TATATATA1	SCGB2B2	7	0.036	INDEL
.	chr19	34684171	TAAAAAAA	T	ZNF302	2	0.014	INDEL
.	chr19	34684171	TAAAAAAA	TAAAAAAA	ZNF302	9	0.063	INDEL

.	chr19	34684171	TAAAAAAA	TAAAAAAA	ZNF302	9	0.063	INDEL
.	chr19	34684171	TAAAAAAA	TAAAAAAA	ZNF302	5	0.035	INDEL
.	chr19	34684171	TAAAAAAA	TAAAAAAA	ZNF302	7	0.049	INDEL
rs1128952!	chr19	34759543	C	A	ZNF599	4	0.021	SNP
rs1482275:	chr19	34759785	TAG	T	ZNF599	3	0.015	INDEL
rs4551269(	chr19	35059974	C	T	HPN	24	0.124	SNP
.	chr19	35105964	AATCACCG	*	CTD-2527I2	7	0.036	INDEL
.	chr19	35105964	AATCACCG	A	CTD-2527I2	2	0.01	INDEL
rs3500180!	chr19	35227988	C	T	FAM187B	8	0.041	SNP
rs541169	chr19	35228117	C	T	FAM187B	62	0.32	SNP
rs1500533	chr19	35270027	TG	T	USF2	24	0.125	INDEL
rs7943872:	chr19	35346193	C	T	CD22	8	0.041	SNP
rs3513870	chr19	35407079	A	C	LINC01531	14	0.072	SNP
.	chr19	35511478	C	*	DMKN	36	0.186	MIXED
.	chr19	35511479	C	*	DMKN	36	0.186	MIXED
rs3835005	chr19	35644377	C	CT	ETV2	120	0.619	INDEL
623943	chr19	35725212	T	TC	KMT2B	2	0.01	INDEL
rs7626094!	chr19	35778700	G	C	ARHGAP33	8	0.041	SNP
rs1173230	chr19	35790308	A	T	LINC01529	3	0.015	SNP
rs3488586(	chr19	35792387	C	G	LINC01529	7	0.036	SNP
rs2285425	chr19	36032767	C	G	CLIP3	26	0.134	SNP
rs1456439	chr19	36146302	C	T	CAPNS1	4	0.021	SNP
.	chr19	36151667	A	C	COX7A1	2	0.011	SNP
.	chr19	36802440	CTT	CT	ZNF790-AS	18	0.093	INDEL
.	chr19	36802440	CTT	CTTT	ZNF790-AS	12	0.062	INDEL
.	chr19	36802440	CTT	C	ZNF790-AS	5	0.026	INDEL
rs547483	chr19	36950463	T	C	ZNF568	63	0.325	SNP
rs1667366	chr19	36997597	T	C	ZNF568	86	0.443	SNP
.	chr19	37199055	C	A	ZNF585B	5	0.026	SNP
rs3604106:	chr19	37224802	G	GT	ZNF383	32	0.165	INDEL
.	chr19	37359409	CT	CTT	HKR1	28	0.144	INDEL
.	chr19	37359409	CT	C	HKR1	16	0.082	INDEL
rs3769315:	chr19	37388950	C	CTGTG	ZNF527	31	0.16	INDEL
rs2004202	chr19	37388952	AT	A	ZNF527	31	0.16	INDEL
.	chr19	37560376	AT	A	ZNF571-AS	23	0.119	INDEL
.	chr19	37560376	AT	ATT	ZNF571-AS	2	0.01	INDEL
rs1126719	chr19	37563751	AATATATT(	A	ZNF571	54	0.278	INDEL
rs1507537	chr19	37563777	AATATATG	*	ZNF571	54	0.278	INDEL
rs1507537	chr19	37563777	AATATATG	A	ZNF571	42	0.216	INDEL
rs1507537	chr19	37563777	AATATATG	AATATATT(	ZNF571	8	0.041	INDEL
rs1162085	chr19	37564828	G	A	ZNF571	2	0.01	SNP
rs6212348:	chr19	38384432	G	C	GGN	2	0.01	SNP
rs6212348:	chr19	38384432	G	A	GGN	2	0.01	SNP
rs3745859;	chr19	38706105	C	T	ACTN4	79	0.407	SNP
rs4801861	chr19	38733773	A	G	CAPN12	143	0.737	SNP
rs1443738!	chr19	38889130	C	T	SIRT2	2	0.01	SNP
rs4803206	chr19	39125881	T	C	PAK4	38	0.196	SNP

rs1132278:	chr19	39248513	CT	C	IFNL4	58	0.299	INDEL
rs2304217	chr19	39464893	G	A	SUPT5H	46	0.237	SNP
rs384138	chr19	39734346	A	G	CLC	139	0.716	SNP
.	chr19	39886263	CTCCCCGG	TTCCCCGG(	FCGBP	5	0.026	MIXED
.	chr19	39906057	CCT	C	FCGBP	6	0.031	INDEL
.	chr19	39906061	A	AGG	FCGBP	6	1	INDEL
.	chr19	39906132	AGC	A	FCGBP	6	0.031	INDEL
.	chr19	39906137	T	TGC	FCGBP	6	1	INDEL
.	chr19	39906156	ACT	A	FCGBP	6	0.032	INDEL
.	chr19	39906162	C	CAGGGG	FCGBP	6	0.031	INDEL
.	chr19	39906164	A	AGG	FCGBP	6	0.031	INDEL
.	chr19	39906168	GT	G	FCGBP	6	0.031	INDEL
rs1122936	chr19	40048798	T	C	ZNF780B	4	0.021	SNP
rs1061197	chr19	40458405	G	A	BLVRB	17	0.088	SNP
rs4548729	chr19	40458433	G	A	BLVRB	2	0.01	SNP
rs1697436	chr19	40576962	T	C	SHKBP1	44	0.227	SNP
rs2230694	chr19	40757498	A	G	SNRPA	11	0.057	SNP
rs7135894	chr19	40875778	G	T	CYP2A7	14	0.072	SNP
rs3745274;	chr19	41006936	G	T	CYP2B6	44	0.227	SNP
rs3211371	chr19	41016810	C	T	CYP2B6	30	0.155	SNP
rs7570307	chr19	41090053	C	T	CYP2A13	37	0.191	SNP
rs1156989	chr19	41094406	A	C	CYP2A13	8	0.041	SNP
rs1477971	chr19	41094412	C	T	CYP2A13	2	0.01	SNP
rs3833221;	chr19	41116202	G	GC	CYP2F1	38	0.196	INDEL
rs5636805	chr19	41354211	GAA	G	TMEM91	3	0.015	INDEL
rs1387319	chr19	41382945	AGGGGACT	A	TMEM91	18	0.093	INDEL
rs3217385;	chr19	41422962	G	GC	BCKDHA	99	0.51	INDEL
.	chr19	42321227	CTTT	CTTTT	TMEM145	8	0.041	INDEL
.	chr19	42321227	CTTT	CTT	TMEM145	30	0.155	INDEL
.	chr19	42321227	CTTT	CT	TMEM145	4	0.021	INDEL
.	chr19	42359092	CAACAG	C	MEGF8	4	0.021	INDEL
rs1851322	chr19	42626450	A	G	CEACAMP1	6	0.031	SNP
rs1135550	chr19	42763988	AG	A	PSG8	12	0.062	INDEL
rs1298607	chr19	42868882	G	A	PSG1	53	0.273	SNP
.	chr19	42935542	G	A	PSG7	165	0.851	SNP
rs1108368	chr19	43015210	C	T	PSG11	115	0.593	SNP
rs6718369	chr19	43204826	TC	T	PSG4	134	0.705	INDEL
rs2072284	chr19	43258164	A	C	PSG9	10	0.053	SNP
rs3746297	chr19	43268150	C	T	PSG9	21	0.108	SNP
rs7511447	chr19	43661400	CTTTTTTT	C	PLAUR	102	0.526	INDEL
rs7511447	chr19	43661400	CTTTTTTT	CTTTTTT	PLAUR	30	0.155	INDEL
rs7190716	chr19	43847014	GGGAGAT	G	ZNF283	78	0.402	INDEL
rs5966044	chr19	44274642	CG	C	ZNF233	28	0.144	INDEL
rs3510691	chr19	44781009	A	C	CBLC	10	0.052	SNP
.	chr19	44781206	G	C	CBLC	2	0.011	SNP
rs11615;22	chr19	45420395	A	G	ERCC1	75	0.387	SNP
.	chr19	45607069	C	T	EML2	2	0.01	SNP

rs2286756	chr19	45641767	G	T	EML2-AS1	104	0.536	SNP
rs1419442:	chr19	45768113	C	T	SIX5	3	0.015	SNP
rs1441316!	chr19	45770204	CCAGCAGC	C	DMPK	83	0.428	INDEL
rs1441316!	chr19	45770204	CCAGCAGC	CCAGCAGC	DMPK	27	0.139	INDEL
rs1441316!	chr19	45770204	CCAGCAGC	CCAGCAGC	DMPK	16	0.082	INDEL
rs1441316!	chr19	45770204	CCAGCAGC	CCAGCAGC	DMPK	4	0.021	INDEL
rs1441316!	chr19	45770204	CCAGCAGC	CCAGCAGC	DMPK	24	0.124	INDEL
rs1441316!	chr19	45770204	CCAGCAGC	CCAGCAGC	DMPK	23	0.119	INDEL
rs3549675:	chr19	46030107	C	CT	CTC-344H1	118	0.608	INDEL
rs3549675:	chr19	46030107	C	CTT	CTC-344H1	2	0.01	INDEL
rs1384650:	chr19	46041049	T	TA	IGFL4	2	0.01	INDEL
rs1177061:	chr19	46179484	A	C	AC006262.·	3	0.015	SNP
rs1173576·	chr19	46229127	C	T	AC006262.	2	0.01	SNP
.	chr19	46303676	G	A	HIF3A	2	0.01	SNP
rs3764609	chr19	46320445	A	G	HIF3A	36	0.186	SNP
rs917948	chr19	46353758	C	T	PPP5C	27	0.139	SNP
rs2279517;	chr19	46411290	C	G	CCDC8	5	0.026	SNP
rs1173064(	chr19	46600969	T	C	PPP5D1	3	0.015	SNP
.	chr19	46745756	C	A	STRN4	2	0.01	SNP
rs1124984!	chr19	47607610	G	T	CTD-2571L	3	0.015	SNP
rs8101879	chr19	47746031	T	G	GLTSCR2	2	0.01	SNP
rs1153866·	chr19	47756530	C	T	GLTSCR2	5	0.026	SNP
rs1421442:	chr19	47757038	T	TA	GLTSCR2	14	0.072	INDEL
rs1156459!	chr19	48077775	TG	T	PLA2G4C	2	0.01	INDEL
rs1458216:	chr19	48117686	C	A	LIG1	2	0.01	SNP
rs4987068	chr19	48137550	C	T	LIG1	5	0.026	SNP
rs1408266:	chr19	48231760	C	CTT	CARD8	13	0.067	INDEL
rs3428292:	chr19	48373572	T	C	SYNGR4	2	0.01	SNP
rs3452721·	chr19	48481921	TA	T	CYTH2	171	0.881	INDEL
rs3848543	chr19	48638275	T	C	CA11	146	0.753	SNP
rs601338;1	chr19	48703417	G	A	FUT2	79	0.407	SNP
rs3529902(	chr19	48815123	G	A	HSD17B14	11	0.057	SNP
rs1846058:	chr19	48832753	C	G	HSD17B14	5	0.026	SNP
rs8110220	chr19	48835841	T	C	HSD17B14	52	0.268	SNP
.	chr19	48874923	AT	A	PPP1R15A	21	0.108	INDEL
.	chr19	48874923	AT	ATTTT	PPP1R15A	30	0.155	INDEL
.	chr19	48874923	AT	ATT	PPP1R15A	4	0.021	INDEL
rs2270940	chr19	48939478	C	T	DHDH	4	0.021	SNP
rs3830420	chr19	48939592	T	TG	DHDH	40	0.206	INDEL
rs1042325!	chr19	48942517	C	T	DHDH	10	0.052	SNP
rs3835153	chr19	48944491	GGA	G	DHDH	21	0.108	INDEL
rs3840912	chr19	49057663	AC	A	CGB7	58	0.299	INDEL
rs1480338:	chr19	49058611	C	T	CTB-60B18	5	0.026	SNP
.	chr19	49115829	AC	A	LIN7B	2	0.01	INDEL
.	chr19	49115830	CAA	*	LIN7B	2	0.01	INDEL
.	chr19	49115830	CAA	CA	LIN7B	10	0.052	INDEL
.	chr19	49115830	CAA	CAAA	LIN7B	4	0.021	INDEL

rs1995759	chr19	49115844	AAG	*	LIN7B	4	0.021	INDEL
rs1995759	chr19	49115844	AAG	A	LIN7B	56	0.289	INDEL
rs6685877	chr19	49115845	AG	*	LIN7B	60	0.309	INDEL
rs6685877	chr19	49115845	AG	A	LIN7B	5	0.026	INDEL
.	chr19	49116009	CA	C	LIN7B	17	0.088	INDEL
.	chr19	49116009	CA	CAA	LIN7B	9	0.046	INDEL
.	chr19	49428625	A	AAC	GFY	5	0.026	INDEL
.	chr19	49428630	GACGC	G	GFY	5	0.026	INDEL
rs5599114	chr19	49490126	G	T	RPL13A	28	0.144	SNP
rs2304205	chr19	49665670	A	C	IRF3	62	0.32	SNP
.	chr19	49836271	CAG	C	MED25	3	0.015	INDEL
rs1999195	chr19	49862369	A	G	PNKP	2	0.01	SNP
rs3739172	chr19	49866530	T	C	PNKP	5	0.026	SNP
rs4802648	chr19	50025797	C	T	ZNF473	62	0.32	SNP
.	chr19	50395002	AT	A	POLD1	44	0.227	INDEL
.	chr19	50395002	AT	ATT	POLD1	10	0.052	INDEL
rs2002060	chr19	50481874	T	TG	EMC10	2	0.01	INDEL
rs1379836	chr19	50725224	C	T	CLEC11A	3	0.015	SNP
rs11573	chr19	50856241	T	C	KLK3	84	0.433	SNP
rs1155309	chr19	50952759	G	A	CTB-147C2	71	0.366	SNP
rs3745540	chr19	51031874	A	G	KLK12	114	0.588	SNP
rs1172293	chr19	51082721	C	T	KLK14	5	0.026	SNP
rs2010747	chr19	51225847	CCCGG	C	CD33	4	0.021	INDEL
rs1140225	chr19	51332638	T	TG	VSIG10L	194	1	INDEL
rs5587494	chr19	51354360	T	TG	ETFB	4	0.021	INDEL
rs1010425	chr19	51417359	C	T	SIGLEC10	162	0.835	SNP
rs6694984	chr19	51501537	G	GC	SIGLEC12	139	0.716	INDEL
rs1698274	chr19	51501649	G	A	SIGLEC12	31	0.16	SNP
rs1492433	chr19	51628003	A	AG	AC018755.	2	0.01	INDEL
rs5616691	chr19	51719927	A	G	HAS1	86	0.443	SNP
rs1246001	chr19	51839881	T	C	AC006272.	59	0.304	SNP
rs1153769	chr19	52211466	C	T	PPP2R1A	6	0.031	SNP
rs1041343	chr19	52225777	C	T	PPP2R1A	12	0.062	SNP
rs3422253	chr19	52297769	ACT	A	CTD-2525I3	3	0.015	INDEL
rs3217319;	chr19	52300416	CTG	C	ZNF480	144	0.742	INDEL
rs3447061	chr19	52383892	TA	T	ZNF880	67	0.345	INDEL
.	chr19	52384819	T	A	ZNF880	2	0.01	SNP
.	chr19	52384823	A	G	ZNF880	4	0.021	MIXED
.	chr19	52384823	A	ATG	ZNF880	3	0.015	MIXED
.	chr19	52384823	A	ATGAGGTC	ZNF880	3	0.015	MIXED
.	chr19	52401662	CT	C	ZNF528	24	0.125	INDEL
.	chr19	52401662	CT	CTT	ZNF528	109	0.568	INDEL
rs1245917	chr19	52456738	C	T	ZNF578	185	0.954	SNP
rs2017870	chr19	52854053	C	CA	ZNF468	18	0.093	INDEL
rs6211543	chr19	53091412	C	T	ZNF160	2	0.01	SNP
rs3544376	chr19	53123541	C	T	ZNF415	14	0.072	SNP
rs1720693	chr19	53200623	G	A	CTD-2245F	60	0.309	SNP

rs2015265	chr19	53491567	CAA	C	ZNF813	2	0.01	INDEL
.	chr19	54574889	C	CGT	LILRA2	2	0.071	INDEL
.	chr19	54574891	TGC	T	LILRA2	2	0.077	INDEL
rs3729711;	chr19	55156279	C	A	TNNI3	4	0.021	SNP
rs890871;2	chr19	55160687	A	G	DNAAF3	9	0.046	SNP
.	chr19	55304657	GTGGGGG	G	BRSK1	2	0.01	INDEL
rs617073	chr19	55669602	C	T	U2AF2	182	0.938	SNP
rs4544489	chr19	55836709	C	T	NLRP11	19	0.098	SNP
rs1471012	chr19	55912558	TGAAA	T	NLRP13	4	0.021	INDEL
rs306457	chr19	55987913	G	C	NLRP8	158	0.814	SNP
rs1720748	chr19	56495046	A	G	ZNF667-AS	41	0.211	SNP
.	chr19	56790080	T	A	ZIM2-AS1	3	0.015	SNP
rs9973206	chr19	57131414	C	A	USP29	155	0.799	SNP
rs1113501	chr19	57135025	T	A	ZIM3	4	0.021	SNP
rs5826428	chr19	57231121	C	T	AURKC	162	0.835	SNP
rs1143089	chr19	57370957	G	GT	ZNF547	80	0.412	INDEL
rs6674741	chr19	57428458	G	A	AC004076.	22	0.113	SNP
rs5925895	chr19	57445372	C	CA	ZNF749	2	0.01	INDEL
rs7306711	chr19	57475736	C	T	ZNF772	2	0.01	SNP
rs2067055	chr19	57476537	G	A	ZNF772	57	0.294	SNP
.	chr19	57492212	G	A	ZNF419	65	0.335	SNP
rs6705406	chr19	57508524	G	A	ZNF773	47	0.242	SNP
rs9749449	chr19	57633347	A	G	ZNF211	29	0.149	SNP
rs3441807	chr19	57639970	TG	T	ZNF211	70	0.361	INDEL
rs7493950	chr19	57702375	G	A	ZNF154	5	0.026	SNP
rs7253069	chr19	57790398	TG	T	ZNF586	73	0.376	INDEL
rs5972397	chr19	57901703	T	TG	CTD-2583A	194	1	INDEL
.	chr19	57926276	AGT	A	ZNF418	4	0.021	INDEL
.	chr19	57926279	A	AGG	ZNF418	4	0.021	INDEL
rs5683970	chr19	58267676	TAAAAAAA	T	CTD-3138B	129	0.672	INDEL
rs5683970	chr19	58267676	TAAAAAAA	TAAAAAAA	CTD-3138B	5	0.026	INDEL
rs5683970	chr19	58267676	TAAAAAAA	TAAAAAAA	CTD-3138B	15	0.078	INDEL
.	chr19	58368733	G	T	ZNF837	2	0.01	SNP
.	chr19	58404775	GGGCAGA	G	CTD-2619J	17	0.088	INDEL
rs2006952	chr19	58591492	T	TCTC	CENPBD1P	85	0.438	INDEL
rs1146749	chr20	145514	GCAAA	G	DEFB126	26	0.134	INDEL
rs1146741	chr20	145669	ACC	A	DEFB126	111	0.572	INDEL
rs1139605	chr20	187984	G	GT	DEFB128	3	0.015	INDEL
rs3835237	chr20	290584	T	TTTA	C20orf96	55	0.286	INDEL
rs3835237	chr20	290584	T	TTA	C20orf96	17	0.089	INDEL
rs3835237	chr20	290584	T	TTTTA	C20orf96	13	0.068	INDEL
rs3835237	chr20	290584	T	TTTTTTA	C20orf96	2	0.01	INDEL
rs3835237	chr20	290584	T	TTTTTA	C20orf96	3	0.016	INDEL
rs7307162	chr20	1118382	A	G	RP4-545L1	2	0.01	SNP
rs200892	chr20	1811114	C	G	RP5-968J1.	102	0.526	SNP
rs2019899	chr20	2207385	TG	T	RP11-128M	3	0.015	INDEL
rs6172922	chr20	2865406	T	C	VPS16	2	0.01	SNP

rs1114877(	chr20	3110719	T	C	UBOX5-AS1	8	0.041	SNP
rs1127354;	chr20	3213196	C	A	ITPA	14	0.072	SNP
.	chr20	3229093	A	C	SLC4A11	2	0.01	SNP
rs1465766:	chr20	3668985	ATCTGGAC	A	ADAM33	8	0.041	INDEL
rs1503582!	chr20	3706494	C	A	SIGLEC1	2	0.01	SNP
rs1056720	chr20	3803463	C	T	CDC25B	48	0.247	SNP
rs1799990;	chr20	4699605	A	G	PRNP	68	0.351	SNP
.	chr20	4869922	T	TGGGGGG(	SLC23A2	3	0.016	INDEL
.	chr20	4869922	T	TGGGGGG(	SLC23A2	2	0.011	INDEL
.	chr20	4869922	T	TGGGGGG(	SLC23A2	2	0.011	INDEL
rs6108191	chr20	8792114	G	A	PLCB1	59	0.304	SNP
rs4557513(	chr20	10652213	G	A	JAG1	4	0.021	SNP
rs1801138;	chr20	10658574	G	A	JAG1	8	0.041	SNP
rs7360641!	chr20	10987532	G	A	RP11-103J8	12	0.062	SNP
rs3908399	chr20	12920627	G	A	RP5-1069C	48	0.247	SNP
rs1768643	chr20	16748403	T	C	OTOR	10	0.052	SNP
68470	chr20	17494075	TAA	T	BFSP1	3	0.015	INDEL
rs3593814!	chr20	18138400	G	T	KAT14	64	0.33	SNP
rs1168840	chr20	18138445	G	T	KAT14	21	0.108	SNP
rs8011324!	chr20	18161818	A	G	KAT14	18	0.093	SNP
rs6075338	chr20	18412855	C	T	DZANK1	152	0.784	SNP
rs7487592	chr20	18465378	C	A	DZANK1	23	0.119	SNP
rs2273057	chr20	20052598	A	T	CRNKL1	90	0.464	SNP
rs7508949	chr20	20052723	C	G	CRNKL1	79	0.407	SNP
rs5840900	chr20	21126073	C	CG	KIZ	127	0.655	INDEL
rs1903377!	chr20	22685115	C	G	RP11-359G	4	0.021	SNP
rs7331813!	chr20	23637934	T	G	CST3	34	0.175	SNP
rs1056033	chr20	24958954	G	C	CST7	95	0.49	SNP
rs1046073;	chr20	25300697	G	A	ABHD12	86	0.443	SNP
rs3215644	chr20	25303302	A	AG	ABHD12	86	0.443	INDEL
.	chr20	25775308	CA	C	FAM182B	2	0.01	INDEL
rs6057289	chr20	30323948	T	G	LINC01598	18	0.093	SNP
rs2233832	chr20	31482436	C	A	REM1	11	0.057	SNP
rs1304518(	chr20	32943738	C	T	EFCAB8	40	0.206	SNP
rs1475768	chr20	33329563	G	A	BPIFB5P	70	0.361	SNP
.	chr20	33912756	CT	CTTTTCTTC	TPM3P2	108	0.557	INDEL
.	chr20	33912756	CT	CTTTTCTTC	TPM3P2	10	0.052	INDEL
.	chr20	33912756	CT	CTTTTCTTC	TPM3P2	12	0.062	INDEL
.	chr20	33912756	CT	CTTTTCTTC	TPM3P2	4	0.021	INDEL
.	chr20	33912756	CT	CTTTTCTTC	TPM3P2	5	0.026	INDEL
.	chr20	33912756	CT	C	TPM3P2	4	0.021	INDEL
rs1404601	chr20	34417100	CT	C	ITCH	16	0.082	INDEL
rs1404601	chr20	34417100	CT	CTT	ITCH	6	0.031	INDEL
.	chr20	34708787	CGAAGA	C	TP53INP2	5	0.026	INDEL
.	chr20	35176156	G	GTCTATCC/	PROCR	2	0.01	INDEL
rs2019859(	chr20	35616276	T	A	SPAG4	3	0.015	SNP
rs2425068	chr20	35626801	T	C	CPNE1	14	0.072	SNP

rs1470191:	chr20	35627312	C	CA	CPNE1	28	0.144	INDEL
.	chr20	36270512	CTT	CTTT	AAR2	18	0.093	INDEL
.	chr20	36270512	CTT	C	AAR2	11	0.057	INDEL
.	chr20	36270512	CTT	CT	AAR2	37	0.191	INDEL
.	chr20	36270536	CAG	C	AAR2	2	0.01	INDEL
rs5741598(	chr20	37179387	G	GCTTATAG,	MROH8	151	0.778	INDEL
rs5741598(	chr20	37179387	G	GCTTACAG	MROH8	42	0.216	INDEL
rs2235581	chr20	38131119	C	T	TGM2	2	0.01	SNP
rs2076390	chr20	38142186	C	T	TGM2	26	0.134	SNP
rs4128282(	chr20	38240603	G	A	KIAA1755	3	0.015	SNP
.	chr20	43653011	CAAAAAAA	CAAAAAAA	RPL27AP	44	0.282	INDEL
.	chr20	43653011	CAAAAAAA	CAAAAAAA	RPL27AP	9	0.058	INDEL
.	chr20	43653011	CAAAAAAA	CAAAAA	RPL27AP	2	0.013	INDEL
.	chr20	43653011	CAAAAAAA	CA	RPL27AP	2	0.013	INDEL
.	chr20	44484462	GGAGT	G	TTPAL	2	0.01	INDEL
rs244076;2	chr20	44624274	T	C	ADA	34	0.175	SNP
rs1155556(	chr20	44626579	T	C	ADA	15	0.077	SNP
rs394105;4	chr20	44636286	C	T	ADA	194	1	SNP
.	chr20	45843741	GC	G	ACOT8	2	0.01	INDEL
.	chr20	46022975	GA	*	SLC12A5	2	0.01	INDEL
.	chr20	46022975	GA	G	SLC12A5	2	0.01	INDEL
.	chr20	46022978	GAGGAGG/	*	SLC12A5	2	0.01	INDEL
.	chr20	46022978	GAGGAGG/	G	SLC12A5	2	0.01	INDEL
.	chr20	46128304	CTT	CT	CD40	49	0.255	INDEL
.	chr20	46128304	CTT	CTTT	CD40	8	0.042	INDEL
.	chr20	46128304	CTT	C	CD40	10	0.052	INDEL
.	chr20	46128304	CTT	CTTTT	CD40	25	0.13	INDEL
rs4128280(	chr20	47188085	C	T	EYA2	4	0.021	SNP
.	chr20	47630457	CTTT	CTT	RP5-1049G	18	0.097	INDEL
.	chr20	47630457	CTTT	CTTTT	RP5-1049G	17	0.091	INDEL
.	chr20	47630457	CTTT	CT	RP5-1049G	3	0.016	INDEL
.	chr20	47630457	CTTT	CTTTTTT	RP5-1049G	2	0.011	INDEL
.	chr20	47630457	CTTT	CTTTTTTT	RP5-1049G	6	0.032	INDEL
rs1419107	chr20	47678506	A	AGGGGAC(	CTD-2653D	23	0.119	INDEL
rs1148096(	chr20	49158937	A	AC	STAU1	73	0.376	INDEL
rs1141260:	chr20	49438576	G	GT	RP4-791K1	22	0.113	INDEL
rs6132288	chr20	49539712	G	A	PTGIS	5	0.026	SNP
rs7684604!	chr20	49812925	G	T	SLC9A8	3	0.015	SNP
.	chr20	49850763	G	GT	SLC9A8	3	0.015	INDEL
rs2230605	chr20	50578524	A	G	PTPN1	2	0.01	SNP
rs6013193	chr20	51475490	T	G	NFATC2	99	0.51	SNP
rs212600	chr20	55438127	A	G	LINC01440	30	0.155	SNP
rs6024911	chr20	56497416	A	G	GCNT7	146	0.753	SNP
.	chr20	56524917	CAATA	C	FAM209A	2	0.01	INDEL
.	chr20	56524922	C	CGTGT	FAM209A	2	0.01	INDEL
.	chr20	56533450	C	CAATA	FAM209B	4	0.021	INDEL
.	chr20	56533451	CGTGT	C	FAM209B	4	0.021	INDEL

rs3761201	chr20	57106087	G	A	RP11-560A	92	0.474	SNP
rs5566282!	chr20	57272053	G	C	RP4-813D1	6	0.031	SNP
rs6070157;	chr20	57562128	C	T	PCK1	53	0.273	SNP
rs1062600;	chr20	57562742	G	A	PCK1	76	0.392	SNP
rs2835954	chr20	57563122	C	T	PCK1	15	0.077	SNP
rs1155214!	chr20	57563592	G	A	PCK1	53	0.273	SNP
rs1804160;	chr20	57564309	G	A	PCK1	15	0.077	SNP
rs1400927!	chr20	57607192	GA	G	ZBP1	4	0.021	INDEL
rs4552163	chr20	57620293	C	T	ZBP1	3	0.015	SNP
.	chr20	58741187	A	C	PIEZO1P2	3	0.015	SNP
rs1434989!	chr20	62143295	G	A	PSMA7	6	0.031	SNP
rs2890083	chr20	62336902	G	A	LAMA5	128	0.66	SNP
rs911077	chr20	62387999	T	C	RPS21	27	0.139	SNP
rs744533	chr20	62427975	T	C	RP5-908M1	117	0.603	SNP
rs5842407	chr20	62926430	ACCGCAGG	A	DIDO1	38	0.196	INDEL
rs6011548	chr20	63034711	C	T	LINC00029	116	0.598	SNP
rs4128299	chr20	63202740	G	A	YTHDF1	9	0.046	SNP
rs2273500	chr20	63355597	T	C	CHRNA4	29	0.149	SNP
rs2738787;	chr20	63697022	A	G	TNFRSF6B	177	0.912	SNP
rs2258056	chr20	63697100	T	C	TNFRSF6B	36	0.186	SNP
rs909341	chr20	63697389	C	T	TNFRSF6B	35	0.18	SNP
.	chr21	10482858	TG	T	BAGE2	36	0.186	INDEL
rs1118413!	chr21	10542449	G	A	TPTE	4	0.021	SNP
rs1262737!	chr21	10605416	G	T	TPTE	10	0.052	SNP
rs3115460	chr21	13916746	C	T	ANKRD20A	39	0.201	SNP
rs1304801(	chr21	14353681	G	A	ABCC13	24	0.124	SNP
rs1453390	chr21	14853273	C	T	AF127936.	3	0.015	SNP
rs3739020	chr21	14902961	TTGCAGTG	T	AF127936.	108	0.557	INDEL
.	chr21	16284686	GTGTGTGT	G	MIR99AHG	2	0.01	INDEL
rs1495370!	chr21	16866677	C	A	AF212831.	2	0.01	SNP
rs1826426	chr21	21284309	T	C	NCAM2	4	0.021	SNP
rs1464223!	chr21	25101321	G	A	AP000233.	12	0.062	SNP
rs2829553	chr21	25135053	C	T	AP000233.	95	0.49	SNP
rs7783771!	chr21	26215685	CTT	CT	AP000230.	14	0.072	INDEL
rs7783771!	chr21	26215685	CTT	CTTT	AP000230.	17	0.088	INDEL
rs7783771!	chr21	26215685	CTT	C	AP000230.	6	0.031	INDEL
rs3838077	chr21	26843517	GC	G	ADAMTS1	153	0.789	INDEL
rs400852	chr21	26843740	A	G	ADAMTS1	28	0.144	SNP
rs370850	chr21	26843747	C	T	ADAMTS1	153	0.789	SNP
.	chr21	27745812	GTGTAGT	G	LINC00113	2	0.01	INDEL
.	chr21	27977885	GTTT	GT	AJ006995.3	30	0.155	INDEL
.	chr21	27977885	GTTT	GTT	AJ006995.3	75	0.387	INDEL
.	chr21	27977885	GTTT	GTTTT	AJ006995.3	5	0.026	INDEL
.	chr21	27977885	GTTT	G	AJ006995.3	2	0.01	INDEL
rs6154890!	chr21	28888333	C	CT	HSPD1P7	18	0.093	INDEL
rs6154890!	chr21	28888333	C	CTT	HSPD1P7	146	0.753	INDEL
rs6154890!	chr21	28888333	C	CTTT	HSPD1P7	5	0.026	INDEL

rs3754260:	chr21	28902054	AT	A	THUMPD1I	11	0.057	INDEL
rs3754260:	chr21	28902054	AT	ATT	THUMPD1I	14	0.072	INDEL
.	chr21	29373240	A	G	BACH1-IT2	2	0.01	SNP
rs2840208	chr21	30089992	A	G	AF096876.:	11	0.057	SNP
rs877346	chr21	30371809	A	T	KRTAP13-2	92	0.474	SNP
rs5843453	chr21	30541662	AG	A	KRTAP19-7	131	0.675	INDEL
rs1423963	chr21	32577592	A	T	TCP10L	2	0.01	SNP
rs1463913	chr21	32592412	CA	C	C21orf59	5	0.026	INDEL
.	chr21	32612257	AGGTACTG	A	AP000275.	4	0.021	INDEL
rs2834090	chr21	33114384	A	G	AP000289.	77	0.397	SNP
rs3437718	chr21	33576378	G	GA	SON	194	1	INDEL
rs3437312:	chr21	33576390	GA	G	SON	194	1	INDEL
.	chr21	33915730	G	A	ATP5O	2	0.01	SNP
rs8129054	chr21	33931469	A	G	AP000304.	57	0.294	SNP
rs9282628	chr21	36135442	C	G	CBR3	2	0.01	SNP
rs881711	chr21	36135447	C	T	CBR3	172	0.887	SNP
rs881712	chr21	36135471	C	T	CBR3	73	0.376	SNP
rs1784967:	chr21	36146284	G	A	CBR3	9	0.046	SNP
rs1063308	chr21	36375309	G	A	MORC3	5	0.026	SNP
rs1453631	chr21	36719946	GA	G	SIM2	57	0.294	INDEL
rs1453631	chr21	36719946	GA	GAA	SIM2	2	0.01	INDEL
rs3171465	chr21	39180381	A	G	PSMG1	90	0.464	SNP
rs1178419	chr21	39746251	G	A	IGSF5	3	0.015	SNP
rs2838010	chr21	41322706	A	T	FAM3B	17	0.088	SNP
rs2021078	chr21	41878845	C	CG	PRDM15	194	1	INDEL
.	chr21	42024305	G	C	ZNF295-AS	123	0.634	SNP
rs1143581	chr21	42111215	C	T	UMODL1	2	0.01	SNP
rs5628396	chr21	42383199	C	CTA	TMPRSS3	11	0.057	INDEL
rs1304795:	chr21	42760333	C	T	PDE9A	94	0.485	SNP
rs4148974	chr21	42903610	C	T	NDUFV3	12	0.062	SNP
rs1153953	chr21	43749003	G	A	PDXK	24	0.124	SNP
rs6173706:	chr21	43749042	G	A	PDXK	4	0.021	SNP
.	chr21	44046506	GTT	GTTT	H2AFZP1	28	0.144	INDEL
rs6002738	chr21	44339472	T	C	AP001062.	36	0.186	SNP
.	chr21	44539673	A	ATG	KRTAP10-1	131	0.675	INDEL
.	chr21	44539674	ATC	A	KRTAP10-1	131	0.675	INDEL
rs7276273	chr21	44574964	A	C	KRTAP10-4	2	0.01	SNP
rs233303	chr21	44591702	G	T	KRTAP10-6	53	0.273	SNP
.	chr21	44592350	G	GCT	KRTAP10-6	8	0.042	INDEL
.	chr21	44600767	C	CCAGCTGC	KRTAP10-7	2	0.01	MIXED
rs1166730	chr21	44637501	G	C	KRTAP10-1	74	0.381	MIXED
rs760462;8	chr21	44908184	T	C	ITGB2	163	0.84	SNP
.	chr21	45285671	GCCTCA	G	POFUT2	14	0.072	INDEL
.	chr21	45456647	T	TG	COL18A1	3	0.015	INDEL
.	chr21	45498344	A	G	SLC19A1	2	0.01	SNP
rs1148324	chr21	45498363	G	A	SLC19A1	24	0.124	SNP
.	chr21	45498370	C	CGGTCCCC	SLC19A1	6	0.031	INDEL

.	chr21	45504512	GGCCCCCC	*	COL18A1	192	0.99	INDEL
.	chr21	45918811	TTGGTGTA	T	PCBP3-OT1	5	0.026	INDEL
rs1499543!	chr21	46125455	A	AC	COL6A2	23	0.119	INDEL
rs2839158;	chr21	46221880	C	T	LSS	31	0.16	SNP
rs1051169	chr21	46602317	C	G	S100B	138	0.711	SNP
.	chr22	15698738	CAAATT	C	POTEH	2	0.01	INDEL
rs4008588	chr22	16573213	G	A	KB-67B5.12	37	0.191	SNP
rs1901501	chr22	16611656	A	G	AC005301.	2	0.01	SNP
rs2850215	chr22	16988159	C	A	GAB4	72	0.371	SNP
rs1474614	chr22	17740102	GGCCACGC	G	BID	36	0.186	INDEL
rs361534	chr22	18145948	C	T	TUBA8	86	0.443	SNP
rs2015889	chr22	18608005	C	CA	RIMBP3	3	0.015	INDEL
.	chr22	18609475	C	CGG	RIMBP3	14	0.072	INDEL
rs1492179	chr22	19448439	CAG	C	AC000068.	46	0.237	INDEL
rs885985	chr22	19524402	G	A	CLDN5	104	0.536	SNP
rs4129881	chr22	19763250	T	C	TBX1	52	0.268	SNP
rs5933936	chr22	19875633	TG	T	TXNRD2	36	0.186	INDEL
rs4633;256	chr22	19962712	C	T	COMT	96	0.495	SNP
rs4818;256	chr22	19963684	C	G	COMT	84	0.433	SNP
rs4680;175	chr22	19963748	G	A	COMT	97	0.5	SNP
rs165631	chr22	19964293	C	T	COMT	4	0.021	SNP
rs6114320	chr22	19967393	AT	A	COMT	7	0.036	INDEL
rs6114320	chr22	19967393	AT	ATT	COMT	12	0.062	INDEL
.	chr22	20112643	G	GC	TRMT2A	2	0.01	INDEL
.	chr22	20116969	GGGCCTGT	G	TRMT2A	6	0.031	INDEL
rs1485349	chr22	20537211	C	CCCTA	MED15	14	0.072	INDEL
rs7176981	chr22	20566526	ACAG	ACAGCAG	MED15	42	0.216	INDEL
rs7176981	chr22	20566526	ACAG	ACAGCAGC	MED15	5	0.026	INDEL
rs1392451	chr22	20702829	G	T	TMEM191	3	0.015	SNP
rs1395982	chr22	20709921	TTGTC	T	PI4KA	4	0.021	INDEL
rs7131475	chr22	21002830	G	A	THAP7-AS1	17	0.088	SNP
rs2075276	chr22	21009455	T	C	TUBA3FP	77	0.397	SNP
.	chr22	21183790	ACGAGGA	GCGAGGA	FAM230B	13	0.068	MIXED
.	chr22	21183790	ACGAGGA	A	FAM230B	10	0.052	MIXED
rs3422156	chr22	22162490	TC	T	IGLV4-60	57	0.294	INDEL
.	chr22	22353380	T	C	IGLV5-48	146	0.753	SNP
rs3773681	chr22	22646181	A	G	GGTLC2	5	0.026	SNP
.	chr22	22704658	GCC	G	IGLV3-22	28	0.144	INDEL
rs7736496	chr22	22704663	T	TGATATAC	IGLV3-22	28	0.144	INDEL
rs3087123	chr22	22747454	ACT	A	IGLV3-16	107	0.552	INDEL
rs6003299	chr22	22771834	T	C	IGLV3-12	75	0.387	SNP
rs2009176	chr22	22792536	GC	G	IGLV2-11	7	0.036	INDEL
.	chr22	22895417	CCT	C	IGLL5	2	0.01	INDEL
rs2020362	chr22	22899573	T	TA	IGLJ2	79	0.407	INDEL
.	chr22	23070353	TGGCGGC	TGGCGGC	GNAZ&RSP	82	0.423	INDEL
.	chr22	23070353	TGGCGGC	TGGCGGC	GNAZ&RSP	6	0.031	INDEL
rs3680361	chr22	23070756	G	C	GNAZ	2	0.01	SNP

rs5639621	chr22	23156147	C	CA	RAB36	63	0.325	INDEL
rs2227939	chr22	23289614	T	C	BCR	69	0.356	SNP
rs1506085	chr22	23567325	G	A	AP000345.	3	0.015	SNP
rs8138979	chr22	23568559	G	A	AP000345.	49	0.253	SNP
rs3747107	chr22	23653353	C	G	KB-1572G7	45	0.232	SNP
rs2070445	chr22	23688720	G	A	RGL4	139	0.716	SNP
.	chr22	24167061	CA	C	CABIN1	3	0.015	INDEL
.	chr22	24167075	CAGCCCAG	C	CABIN1	2	0.01	INDEL
.	chr22	24167091	CG	C	CABIN1	3	0.015	INDEL
.	chr22	24167096	CCG	C	CABIN1	2	0.01	INDEL
rs1809511	chr22	24253399	G	C	POM121L9	4	0.021	SNP
rs1434930	chr22	24523618	G	A	UPB1	2	0.01	SNP
.	chr22	24593038	A	G	LRRC75B	2	0.01	SNP
rs5621410	chr22	24621069	C	T	GGT1	3	0.015	SNP
rs2020876	chr22	24627452	C	T	GGT1	11	0.057	SNP
rs7131899	chr22	24627525	G	A	GGT1	3	0.015	SNP
rs5996763	chr22	24813757	T	C	SGSM1	28	0.144	SNP
rs1767050	chr22	25203882	G	A	CRYBB3	10	0.052	SNP
rs1763374	chr22	25892225	A	C	CTA-125H2	10	0.052	SNP
rs3747129	chr22	26466075	G	A	HPS4	37	0.191	SNP
rs1216950	chr22	26540931	G	A	TPST2	46	0.237	SNP
rs5761587	chr22	26541121	C	A	TPST2	16	0.082	SNP
rs3552067	chr22	26623300	G	A	CRYBA4	3	0.015	SNP
rs5761637	chr22	26625493	T	C	CRYBA4	163	0.84	SNP
rs1734484	chr22	27050117	C	A	CTA-992D9	6	0.031	SNP
rs2004521	chr22	27721309	A	T	RP11-375H	108	0.557	SNP
rs1807641	chr22	28679663	G	A	TTC28	52	0.268	SNP
rs1460522	chr22	28737324	C	T	CHEK2	2	0.01	SNP
rs1248408	chr22	29441895	C	T	RFPL1	44	0.227	SNP
.	chr22	29767548	G	GAAGCTGT	UQCR10	5	0.026	INDEL
.	chr22	30388280	C	CTAAA	RNF215	4	0.021	INDEL
.	chr22	30388280	C	CTAAATAA	RNF215	5	0.026	INDEL
rs757660	chr22	30397148	G	A	SEC14L2	76	0.392	SNP
rs3576412	chr22	30460134	G	A	SEC14L3	11	0.057	SNP
rs5749104	chr22	30461461	A	G	SEC14L3	136	0.701	SNP
rs4820853	chr22	30468623	A	G	SEC14L3	76	0.392	SNP
rs5565069	chr22	30494899	G	A	SEC14L4	3	0.015	SNP
rs9606738	chr22	30495263	T	C	SEC14L4	29	0.149	SNP
rs9606739	chr22	30495307	G	C	SEC14L4	28	0.144	SNP
rs7767934	chr22	30495313	G	A	SEC14L4	3	0.015	SNP
rs9608956	chr22	30505605	T	C	SEC14L4	36	0.186	SNP
rs5753396	chr22	30929997	C	T	MORC2-AS	63	0.325	SNP
rs1928963	chr22	31093448	A	G	SMTN	2	0.01	SNP
rs2228619	chr22	31267856	C	G	LIMK2	131	0.675	SNP
rs1432983	chr22	31676814	C	T	PRR14L	4	0.021	SNP
rs3709940	chr22	32247460	AC	A	SLC5A4	2	0.01	INDEL
rs6223905	chr22	32247473	C	A	SLC5A4	4	0.021	SNP

rs11107;19	chr22	32479203	G	A	FBXO7	71	0.366	SNP
rs1175139:	chr22	32527686	C	T	SYN3	3	0.015	SNP
rs133416	chr22	35410383	T	C	MCM5	137	0.706	SNP
.	chr22	36191797	ACT	A	APOL4	82	0.423	INDEL
rs9610445	chr22	36201698	A	C	APOL4	24	0.124	SNP
rs6000181	chr22	36202035	A	T	APOL4	24	0.124	SNP
rs7967481:	chr22	36396117	G	A	RP4-633O1	9	0.046	SNP
rs1801117;	chr22	36933957	C	T	CSF2RB	33	0.17	SNP
rs1063741	chr22	36967952	T	TTTTA	LL22NC01-8	19	0.098	INDEL
rs1063741	chr22	36967952	T	TTTTATTTA	LL22NC01-8	38	0.196	INDEL
rs1063741	chr22	36967952	T	TTTTATTTA	LL22NC01-8	2	0.01	INDEL
rs2004349:	chr22	37069345	CTGGGG	C	TMPRSS6	32	0.17	INDEL
rs1064498;	chr22	37226775	A	G	RAC2	34	0.175	SNP
rs1156862	chr22	37570642	G	A	LGALS2	2	0.01	SNP
rs1305534:	chr22	37609137	T	C	GGA1	4	0.021	SNP
rs1420283	chr22	37665630	A	T	PDXP	3	0.015	SNP
rs7796801	chr22	38082659	C	G	SLC16A8	2	0.01	SNP
rs6650063	chr22	38086345	GTGCGGG/	G	BAIAP2L2	71	0.366	INDEL
rs1062687	chr22	38738710	T	C	SUN2	58	0.299	SNP
rs1749604	chr22	39086366	C	G	APOBEC3G	13	0.067	SNP
rs20552;93	chr22	41155035	T	A	EP300	113	0.582	SNP
rs5751058	chr22	41169281	A	G	EP300	73	0.376	SNP
.	chr22	41238083	A	ACGCGCGC	CHADL	5	0.026	INDEL
.	chr22	41589051	CCCTCA	C	PMM1	4	0.021	INDEL
rs5758511	chr22	41940168	G	A	CENPM	69	0.356	SNP
rs3554250	chr22	41945930	A	G	CENPM	17	0.088	SNP
rs5030655;	chr22	42129083	CA	C	CYP2D6	5	0.037	INDEL
rs137070	chr22	42576509	T	TA	RRP7BP	23	0.119	INDEL
rs3833393	chr22	43537404	CCT	C	EFCAB6	6	0.031	INDEL
rs132852	chr22	45164760	T	C	NUP50	42	0.216	SNP
rs132876	chr22	45171154	C	T	NUP50	42	0.216	SNP
rs1042311	chr22	46231883	C	T	PPARA	4	0.021	SNP
rs2879000	chr22	47363710	C	G	CITF22-49D	116	0.598	SNP
.	chr22	48892665	G	A	LINC01310	3	0.015	SNP
.	chr22	49758002	CT	CTT	RPL5P35	4	0.021	INDEL
.	chr22	49758002	CT	C	RPL5P35	7	0.036	INDEL
770820	chr22	49922288	CCTCAGCA	C	CRELD2	24	0.124	INDEL
rs1129880	chr22	50255868	A	G	MAPK12	148	0.763	SNP
rs2072876	chr22	50256941	G	A	MAPK12	12	0.062	SNP
rs2076139	chr22	50266232	T	C	MAPK11	150	0.773	SNP
.	chr22	50437479	G	GTCCC	PPP6R2	2	0.01	INDEL
.	chr22	50437503	CAG	C	PPP6R2	2	0.01	INDEL
rs4128056	chr22	50489583	G	A	MIOX	2	0.01	SNP
rs2232885	chr22	50489597	G	A	MIOX	45	0.232	SNP
rs140524	chr22	50522253	C	T	NCAPH2	47	0.242	SNP
rs11479;13	chr22	50525807	G	A	TYMP	27	0.139	SNP
rs1138404;	chr22	50526017	A	T	TYMP	18	0.093	SNP

.	chr22	50526574	A	C	TYMP	2	0.01	SNP
rs1904786(	chr22	50623773	C	T	ARSA	3	0.015	SNP
.	chr22	50697561	GTT	G	SHANK3	132	0.957	INDEL
.	chr22	50720550	CTCGA	C	SHANK3	3	0.015	INDEL
.	chrX	1403270	CA	C	ASMTL	35	0.18	INDEL
.	chrX	1435756	T	C	ASMTL	62	0.32	SNP
.	chrX	1629941	T	C	ASMT	2	0.01	SNP
.	chrX	1636511	C	T	ASMT	11	0.057	SNP
.	chrX	1642840	A	T	ASMT	3	0.015	SNP
rs7049300;	chrX	5903745	G	A	NLGN4X	14	0.072	SNP
rs1803675	chrX	7077397	C	T	PUDP	49	0.253	SNP
rs1462457	chrX	7842742	G	A	VCX	11	0.057	SNP
.	chrX	7843768	A	ACT	VCX	2	0.012	INDEL
.	chrX	7843768	A	ACTGTGT	VCX	4	0.024	INDEL
rs4066728	chrX	8171106	T	C	VCX2	5	0.027	SNP
rs2859263(	chrX	8464991	G	A	VCX3B	10	0.054	SNP
.	chrX	9644659	CT	C	TBL1X	5	0.026	INDEL
.	chrX	9644659	CT	CTT	TBL1X	14	0.072	INDEL
.	chrX	12826648	GAAAA	GAAAAAAA	PSMA6P2	5	0.028	INDEL
.	chrX	12826648	GAAAA	GAAA	PSMA6P2	8	0.045	INDEL
.	chrX	12826648	GAAAA	GAAAAAAA	PSMA6P2	2	0.011	INDEL
.	chrX	12826648	GAAAA	G	PSMA6P2	4	0.023	INDEL
.	chrX	12826648	GAAAA	GAAAAAAA	PSMA6P2	3	0.017	INDEL
rs3764880	chrX	12906707	A	G	TLR8	44	0.227	SNP
rs2159377	chrX	12919394	C	T	TLR8	33	0.17	SNP
rs2407992	chrX	12920993	G	C	TLR8	89	0.459	SNP
rs3747414	chrX	12921293	C	A	TLR8	88	0.454	SNP
rs2013810(	chrX	13319859	C	A	ATXN3L	3	0.015	SNP
rs5935650	chrX	13663560	G	T	TCEANC	99	0.51	SNP
.	chrX	15327233	TG	T	PIGA	2	0.01	INDEL
rs8094	chrX	15397461	C	T	PIR	115	0.593	SNP
rs6160388	chrX	17557196	A	C	NHS-AS1	50	0.258	SNP
rs5901624;	chrX	17687730	C	CT	NHS	194	1	INDEL
rs3213544	chrX	18323914	G	A	SCML2	42	0.216	SNP
rs1061675	chrX	19345745	TCC	TC	PDHA1	14	0.072	INDEL
rs1061675	chrX	19345745	TCC	T	PDHA1	4	0.021	INDEL
rs1061675	chrX	19345745	TCC	TCCC	PDHA1	2	0.01	INDEL
rs1061675	chrX	19345745	TCC	TCCCCC	PDHA1	3	0.015	INDEL
rs1483121	chrX	19369214	G	A	MAP3K15	2	0.01	SNP
.	chrX	19497163	CT	C	EIF5P2	18	0.093	INDEL
.	chrX	19497163	CT	CTT	EIF5P2	28	0.144	INDEL
rs5925658	chrX	22743228	C	T	PTCHD1-AS	128	0.66	SNP
rs2250315	chrX	24650150	G	A	PCYT1B-AS	158	0.814	SNP
rs3530574	chrX	29955334	G	T	IL1RAPL1	2	0.01	SNP
rs5927639	chrX	30899069	G	A	TAB3	123	0.634	SNP
rs4829392	chrX	35803010	C	T	MAGEB16	111	0.572	SNP
rs1127003	chrX	36144567	C	CTG	CFAP47	22	0.113	INDEL

rs1383589;	chrX	36431472	T	G	RP11-87M1	7	0.036	SNP
rs1466848	chrX	38006669	G	A	SYTL5	3	0.015	SNP
rs4827349	chrX	38298370	C	A	RPGR	6	0.031	SNP
.	chrX	40072336	TG	T	BCOR	2	0.01	INDEL
.	chrX	40603355	CAA	CA	ATP6AP2	157	0.809	INDEL
.	chrX	40603355	CAA	C	ATP6AP2	8	0.041	INDEL
.	chrX	40603355	CAA	CAAAAAAA	ATP6AP2	3	0.015	INDEL
rs6520743	chrX	41357831	A	T	DDX3X	71	0.366	SNP
rs1820047(	chrX	46500988	G	A	ZNF674	2	0.01	SNP
.	chrX	46560996	CTTTTTTT	CTTTTTT	MKI67P1	28	0.179	INDEL
.	chrX	46560996	CTTTTTTT	CTTTTT	MKI67P1	3	0.019	INDEL
.	chrX	46560996	CTTTTTTT	C	MKI67P1	4	0.026	INDEL
.	chrX	46560996	CTTTTTTT	CTTTT	MKI67P1	2	0.013	INDEL
.	chrX	46560996	CTTTTTTT	CTTTTTTTT	MKI67P1	2	0.013	INDEL
rs1805147;	chrX	46860063	C	T	RP2	3	0.015	SNP
.	chrX	47439219	CT	C	NPM1P49	27	0.139	INDEL
.	chrX	47439219	CT	CTT	NPM1P49	11	0.057	INDEL
rs3508495	chrX	47836343	CT	C	AL022578.:	101	0.521	INDEL
rs1825859	chrX	48158446	A	G	SSXP3	2	0.01	SNP
.	chrX	48281284	A	AAAAAAAT	RP11-344N	33	0.188	INDEL
.	chrX	49067832	CA	C	CCDC120	2	0.01	INDEL
.	chrX	49183660	AGTGT	AGT	PRICKLE3	190	0.979	INDEL
.	chrX	49183660	AGTGT	A	PRICKLE3	3	0.015	INDEL
rs7818239:	chrX	51496443	ATCCTCGA(	A	NUDT11	194	1	INDEL
.	chrX	53070969	CT	CTT	GPR173	34	0.175	INDEL
.	chrX	53070969	CT	CTTT	GPR173	2	0.01	INDEL
.	chrX	53070969	CT	C	GPR173	37	0.191	INDEL
769214	chrX	53534126	T	C	HUWE1	194	1	SNP
.	chrX	54182866	A	AT	FAM120C	3	0.015	INDEL
.	chrX	54182869	G	GGGCGGC	FAM120C	52	0.268	INDEL
.	chrX	54182869	G	GGCGGC	FAM120C	3	0.015	INDEL
.	chrX	54759753	T	TA	ITIH6	3	0.015	INDEL
rs6174511	chrX	55221384	T	C	PAGE5	7	0.036	SNP
.	chrX	57910016	GCA	G	ZXDA	15	0.079	INDEL
.	chrX	57910019	G	GGC	ZXDA	15	0.086	INDEL
.	chrX	65328521	C	CT	MORF4L1P	3	0.016	INDEL
rs1146174	chrX	65497128	T	TA	ZC3H12B	3	0.015	INDEL
rs4545779(	chrX	65731167	T	C	MSN	2	0.01	SNP
rs4131066	chrX	70258582	C	T	P2RY4	3	0.015	SNP
.	chrX	70430952	ATT	AT	GDPD2	48	0.247	INDEL
.	chrX	70430952	ATT	ATTT	GDPD2	12	0.062	INDEL
.	chrX	70430952	ATT	A	GDPD2	9	0.046	INDEL
rs7050748	chrX	71407627	G	A	TAF1	2	0.01	SNP
rs2010190(	chrX	72186217	AT	A	PIN4	9	0.046	INDEL
rs2010190(	chrX	72186217	AT	ATT	PIN4	3	0.015	INDEL
rs1109337	chrX	72572062	C	T	HDAC8	2	0.01	SNP
.	chrX	74925026	T	C	KIAA2022	2	0.01	SNP

rs5938014	chrX	75294621	G	A	UPRT	13	0.067	SNP
rs1343879	chrX	75784694	C	A	MAGEE2	9	0.046	SNP
rs2021134	chrX	81413642	G	GT	RP13-52K8	6	0.031	INDEL
.	chrX	85268268	CT	C	ZNF711	17	0.102	INDEL
.	chrX	85268268	CT	CTTT	ZNF711	6	0.036	INDEL
.	chrX	85268268	CT	CTT	ZNF711	3	0.018	INDEL
.	chrX	85863095	CT	C	CHM	15	0.077	INDEL
.	chrX	85863095	CT	CTT	CHM	8	0.041	INDEL
.	chrX	85863096	T	*	CHM	17	0.088	SNP
.	chrX	85863096	T	C	CHM	2	0.01	SNP
rs1506679	chrX	92468314	T	C	PCDH11X	2	0.01	SNP
rs5668227	chrX	96916417	G	GT	DIAPH2	92	0.474	INDEL
rs5668227	chrX	96916417	G	GTT	DIAPH2	11	0.057	INDEL
.	chrX	1.01E+08	CTTT	CTT	CENPI	37	0.208	INDEL
.	chrX	1.01E+08	CTTT	CT	CENPI	58	0.326	INDEL
.	chrX	1.01E+08	CTTT	CTTTT	CENPI	5	0.028	INDEL
.	chrX	1.01E+08	CTTT	C	CENPI	9	0.051	INDEL
rs1135363;	chrX	1.01E+08	G	A	BTK	38	0.196	SNP
rs1140812	chrX	1.02E+08	T	TG	TCEAL6	194	1	INDEL
.	chrX	1.02E+08	GC	G	TCP11X2	31	0.16	INDEL
rs2301384	chrX	1.04E+08	C	T	H2BFM	82	0.423	SNP
.	chrX	1.07E+08	ATTTTT	ATTTT	TBC1D8B	24	0.136	INDEL
.	chrX	1.07E+08	ATTTTT	ATTT	TBC1D8B	5	0.028	INDEL
.	chrX	1.07E+08	ATTTTT	ATTTTTT	TBC1D8B	2	0.011	INDEL
rs2022325	chrX	1.07E+08	A	AT	CLDN2	3	0.015	INDEL
.	chrX	1.15E+08	CAG	C	LRCH2	2	0.01	INDEL
.	chrX	1.24E+08	CT	C	STAG2	22	0.113	INDEL
.	chrX	1.24E+08	CT	CTT	STAG2	31	0.16	INDEL
.	chrX	1.24E+08	CTT	CT	STAG2	29	0.161	INDEL
.	chrX	1.24E+08	CTT	CTTTT	STAG2	7	0.039	INDEL
.	chrX	1.24E+08	CTT	C	STAG2	8	0.044	INDEL
.	chrX	1.24E+08	CTT	CTTTTT	STAG2	3	0.017	INDEL
.	chrX	1.24E+08	CTT	CTTT	STAG2	4	0.022	INDEL
rs1139851;	chrX	1.3E+08	A	G	AIFM1	78	0.402	SNP
rs2411856	chrX	1.3E+08	C	A	RP1-274L7.	19	0.098	SNP
.	chrX	1.3E+08	CT	C	RP1-274L7.	7	0.036	INDEL
.	chrX	1.3E+08	CT	CTT	RP1-274L7.	20	0.103	INDEL
rs1886894	chrX	1.3E+08	A	G	RP1-274L7.	7	0.036	SNP
.	chrX	1.33E+08	GA	G	HS6ST2	2	0.01	INDEL
.	chrX	1.33E+08	CTTTTTTTT	CTTT	RP3-358H7	73	0.415	INDEL
.	chrX	1.33E+08	CTTTTTTTT	CT	RP3-358H7	6	0.034	INDEL
.	chrX	1.33E+08	CTTTTTTTT	CTT	RP3-358H7	14	0.08	INDEL
.	chrX	1.33E+08	CTTTTTTTT	CTTTTTTTT	RP3-358H7	3	0.017	INDEL
rs5930742	chrX	1.36E+08	T	C	RP11-432N	186	0.959	SNP
.	chrX	1.36E+08	G	C	FHL1	2	0.01	SNP
.	chrX	1.37E+08	G	GAA	RBMX	8	0.041	INDEL
.	chrX	1.37E+08	A	AT	RBMX	10	0.052	INDEL

rs2016735	chrX	1.37E+08	TG	T	RBMX	12	0.062	INDEL
.	chrX	1.42E+08	GTCTCC	*	MAGEC1	5	0.027	INDEL
.	chrX	1.42E+08	AGATTCCT	*	MAGEC1	25	0.133	INDEL
.	chrX	1.42E+08	TCTTTTCC	*	MAGEC1	5	0.028	INDEL
.	chrX	1.42E+08	TCTTTTCC	T	MAGEC1	2	0.011	INDEL
.	chrX	1.42E+08	AGAGTTCC	*	MAGEC1	2	0.011	INDEL
.	chrX	1.42E+08	AGAGTTCC	A	MAGEC1	2	0.011	INDEL
.	chrX	1.44E+08	AT	A	SPANXN2	3	0.015	INDEL
.	chrX	1.44E+08	G	T	UBE2NL	68	0.351	SNP
rs2002701	chrX	1.52E+08	CG	C	PRRG3	3	0.015	INDEL
.	chrX	1.52E+08	A	C	RP11-366F	2	0.01	SNP
rs6174613	chrX	1.52E+08	C	T	MAGEA4	7	0.036	SNP
rs1894360	chrX	1.53E+08	G	C	CSAG1	38	0.196	SNP
rs5987128	chrX	1.54E+08	C	T	PNCK	17	0.088	SNP
rs1994980	chrX	1.54E+08	C	CA	SLC6A8	15	0.077	INDEL
rs1847073	chrX	1.54E+08	C	A	BCAP31	14	0.072	SNP
rs2012536	chrX	1.54E+08	G	GTCCCC	SRPK3	55	0.284	INDEL
.	chrX	1.54E+08	A	C	ARHGAP4	2	0.01	SNP
470162	chrX	1.55E+08	A	G	G6PD	172	0.887	SNP
rs7784554	chrY	11157053	C	T	MAFIP	21	0.125	SNP
.	chrY	13160117	CTA	C	TAB3P1	3	0.044	INDEL
.	chrY	13160117	CTA	CTATA	TAB3P1	3	0.044	INDEL
.	chrY	13323634	C	T	UTY	2	0.029	SNP

Public databases						Cannonical transcript SC		
GWAS cata	Clinvar ass	Clinvar ass	AF_Exac	AF_IGP	Annotatioı	Gene ID	Feature Ty	FeatureID
NA	Benign	not_provid	NA	NA	splice_don	ENSG0000(	transcript	ENST0000C
NA	Benign	Immunode	0.00831	0.00339	structural_	ENSG0000(	interaction	1Z2M:A_79
NA	Benign	not_specifi	0.37025	0.33886	structural_	ENSG0000(	interaction	1Z2M:A_83
NA	NA	NA	NA	NA	splice_don	ENSG0000(	transcript	ENST0000C
NA	NA	NA	NA	NA	stop_gaine	ENSG0000(	transcript	ENST0000C
NA	NA	NA	NA	NA	splice_acce	ENSG0000(	transcript	ENST0000C
NA	NA	NA	NA	NA	frameshift_	ENSG0000(	transcript	ENST0000C
NA	NA	NA	NA	NA	frameshift_	ENSG0000(	transcript	ENST0000C
NA	NA	NA	NA	NA	frameshift_	ENSG0000(	transcript	ENST0000C
NA	NA	NA	NA	NA	start_lost	ENSG0000(	transcript	ENST0000C
NA	NA	NA	NA	NA	frameshift_	ENSG0000(	transcript	ENST0000C
NA	NA	NA	NA	NA	splice_don	ENSG0000(	transcript	ENST0000C
NA	NA	NA	NA	NA	splice_don	ENSG0000(	transcript	ENST0000C
NA	NA	NA	NA	NA	structural_	ENSG0000(	interaction	2XWC:A_1
NA	NA	NA	NA	NA	splice_don	ENSG0000(	transcript	ENST0000C
NA	NA	NA	NA	NA	stop_gaine	ENSG0000(	transcript	ENST0000C
NA	NA	NA	NA	NA	splice_acce	ENSG0000(	transcript	ENST0000C
NA	NA	NA	NA	NA	splice_acce	ENSG0000(	transcript	ENST0000C
NA	NA	NA	NA	NA	structural_	ENSG0000(	interaction	1ZSX:A_42-
NA	NA	NA	NA	NA	splice_acce	ENSG0000(	transcript	ENST0000C
NA	Benign	Parkinson_	0.00815	0.00619	structural_	ENSG0000(	interaction	1PDW:A_6
NA	NA	NA	NA	NA	structural_	ENSG0000(	interaction	3FE4:A_76-
Blood_prot	Benign	not_specifi	NA	NA	frameshift_	ENSG0000(	transcript	ENST0000C
NA	NA	NA	NA	NA	splice_don	ENSG0000(	transcript	ENST0000C
NA	NA	NA	NA	NA	splice_acce	ENSG0000(	transcript	ENST0000C
NA	NA	NA	NA	NA	splice_don	ENSG0000(	transcript	ENST0000C
NA	NA	NA	NA	NA	splice_acce	ENSG0000(	transcript	ENST0000C
NA	NA	NA	NA	NA	stop_gaine	ENSG0000(	transcript	ENST0000C
NA	Benign	not_specifi	0.14369	0.17911	stop_lost	ENSG0000(	transcript	ENST0000C
NA	NA	NA	NA	NA	stop_gaine	ENSG0000(	transcript	ENST0000C
NA	NA	NA	NA	NA	stop_gaine	ENSG0000(	transcript	ENST0000C
NA	NA	NA	NA	NA	frameshift_	ENSG0000(	transcript	ENST0000C
NA	NA	NA	NA	NA	splice_acce	ENSG0000(	transcript	ENST0000C
NA	NA	NA	NA	NA	frameshift_	ENSG0000(	transcript	ENST0000C
NA	NA	NA	NA	NA	splice_acce	ENSG0000(	transcript	ENST0000C
NA	NA	NA	NA	NA	splice_acce	ENSG0000(	transcript	ENST0000C
NA	NA	NA	NA	NA	splice_acce	ENSG0000(	transcript	ENST0000C
NA	NA	NA	NA	NA	splice_acce	ENSG0000(	transcript	ENST0000C
NA	NA	NA	NA	NA	frameshift_	ENSG0000(	transcript	ENST0000C
NA	NA	NA	NA	NA	frameshift_	ENSG0000(	transcript	ENST0000C
NA	NA	NA	NA	NA	frameshift_	ENSG0000(	transcript	ENST0000C
NA	NA	NA	NA	NA	structural_	ENSG0000(	interaction	1OW1:A_3
NA	Benign/Lik	Age-relate	0.00331	0.00160	structural_	ENSG0000(	interaction	2X10:A_43
NA	Benign	Age-relate	0.37871	0.34944	structural_	ENSG0000(	interaction	3FL7:A_329

NA	Benign	Age-relatec	0.28999	0.22644	structural_	ENSG0000(	interaction	2X10:A_67
NA	Benign	Age-relatec	NA	0.22664	structural_	ENSG0000(	interaction	2X10:A_10
NA	NA	NA	NA	NA	splice_acce	ENSG0000(	transcript	ENST0000C
NA	NA	NA	NA	NA	splice_acce	ENSG0000(	transcript	ENST0000C
NA	NA	NA	NA	NA	splice_acce	ENSG0000(	transcript	ENST0000C
NA	NA	NA	NA	NA	splice_acce	ENSG0000(	transcript	ENST0000C
NA	NA	NA	NA	NA	splice_acce	ENSG0000(	transcript	ENST0000C
NA	NA	NA	NA	NA	splice_acce	ENSG0000(	transcript	ENST0000C
NA	NA	NA	NA	NA	splice_don	ENSG0000(	transcript	ENST0000C
NA	NA	NA	NA	NA	splice_don	ENSG0000(	transcript	ENST0000C
NA	NA	NA	NA	NA	structural_	ENSG0000(	interaction	4N20:A_42
NA	NA	NA	NA	NA	frameshift_	ENSG0000(	transcript	ENST0000C
NA	NA	NA	NA	NA	structural_	ENSG0000(	interaction	3APM:A_8(
NA	Benign	not_provid	NA	NA	structural_	ENSG0000(	interaction	1WDA:A_3
Rheumatoi	NA	NA	NA	NA	structural_	ENSG0000(	interaction	1WDA:A_3
NA	Benign	Deficiency_	NA	0.00459	structural_	ENSG0000(	interaction	3V9G:A_31
Alzheimer's	Benign/Like	Hyperproli	0.03354	0.06550	structural_	ENSG0000(	interaction	3V9H:A_44
NA	Likely_beni	Hyperproli	0.10439	0.04792	structural_	ENSG0000(	interaction	3V9G:A_35
NA	Benign	Hyperproli	0.72982	0.73722	structural_	ENSG0000(	interaction	3V9G:A_35
NA	Benign	Hyperproli	0.70245	0.66094	structural_	ENSG0000(	interaction	3V9G:A_41
NA	Benign	Hyperproli	0.70717	0.64157	structural_	ENSG0000(	interaction	3V9G:A_31
NA	NA	NA	NA	NA	frameshift_	ENSG0000(	transcript	ENST0000C
NA	NA	NA	NA	NA	frameshift_	ENSG0000(	transcript	ENST0000C
NA	NA	NA	NA	NA	stop_lost	ENSG0000(	transcript	ENST0000C
NA	NA	NA	NA	NA	frameshift_	ENSG0000(	transcript	ENST0000C
NA	NA	NA	NA	NA	splice_acce	ENSG0000(	transcript	ENST0000C
NA	NA	NA	NA	NA	frameshift_	ENSG0000(	transcript	ENST0000C
NA	NA	NA	NA	NA	splice_acce	ENSG0000(	transcript	ENST0000C
NA	NA	NA	NA	NA	stop_gaine	ENSG0000(	transcript	ENST0000C
NA	NA	NA	NA	NA	splice_acce	ENSG0000(	transcript	ENST0000C
NA	NA	NA	NA	NA	splice_acce	ENSG0000(	transcript	ENST0000C
NA	NA	NA	NA	NA	splice_acce	ENSG0000(	transcript	ENST0000C
NA	NA	NA	NA	NA	stop_lost	ENSG0000(	transcript	ENST0000C
NA	NA	NA	NA	NA	structural_	ENSG0000(	interaction	1SRQ:C_31
NA	NA	NA	NA	NA	frameshift_	ENSG0000(	transcript	ENST0000C
NA	NA	NA	NA	NA	frameshift_	ENSG0000(	transcript	ENST0000C
NA	NA	NA	NA	NA	splice_don	ENSG0000(	transcript	ENST0000C
NA	NA	NA	NA	NA	splice_don	ENSG0000(	transcript	ENST0000C
NA	NA	NA	NA	NA	splice_acce	ENSG0000(	transcript	ENST0000C
NA	NA	NA	NA	NA	splice_acce	ENSG0000(	transcript	ENST0000C
NA	NA	NA	NA	NA	splice_don	ENSG0000(	transcript	ENST0000C
NA	NA	NA	NA	NA	splice_don	ENSG0000(	transcript	ENST0000C
NA	NA	NA	NA	NA	splice_acce	ENSG0000(	transcript	ENST0000C
NA	NA	NA	NA	NA	stop_gaine	ENSG0000(	transcript	ENST0000C
NA	NA	NA	NA	NA	structural_	ENSG0000(	interaction	2B0O:E_45
NA	NA	NA	NA	NA	structural_	ENSG0000(	interaction	2B0O:E_45
NA	NA	NA	NA	NA	splice_acce	ENSG0000(	transcript	ENST0000C

NA	NA	NA	NA	NA	stop_gaine	ENSG0000(	transcript	ENST0000C
NA	NA	NA	NA	NA	structural_	ENSG0000(	interaction	3DGC:R_23
NA	NA	NA	NA	NA	splice_acce	ENSG0000(	transcript	ENST0000C
NA	NA	NA	NA	NA	splice_regi	ENSG0000(	transcript	ENST0000C
NA	Benign	not_specifi	NA	NA	frameshift_	ENSG0000(	transcript	ENST0000C
NA	NA	NA	NA	NA	frameshift_	ENSG0000(	transcript	ENST0000C
NA	NA	NA	NA	NA	frameshift_	ENSG0000(	transcript	ENST0000C
NA	NA	NA	NA	NA	frameshift_	ENSG0000(	transcript	ENST0000C
NA	NA	NA	NA	NA	frameshift_	ENSG0000(	transcript	ENST0000C
NA	NA	NA	NA	NA	frameshift_	ENSG0000(	transcript	ENST0000C
NA	NA	NA	NA	NA	frameshift_	ENSG0000(	transcript	ENST0000C
NA	NA	NA	NA	NA	frameshift_	ENSG0000(	transcript	ENST0000C
NA	NA	NA	NA	NA	frameshift_	ENSG0000(	transcript	ENST0000C
NA	NA	NA	NA	NA	frameshift_	ENSG0000(	transcript	ENST0000C
NA	NA	NA	NA	NA	frameshift_	ENSG0000(	transcript	ENST0000C
NA	NA	NA	NA	NA	frameshift_	ENSG0000(	transcript	ENST0000C
NA	NA	NA	NA	NA	frameshift_	ENSG0000(	transcript	ENST0000C
NA	NA	NA	NA	NA	frameshift_	ENSG0000(	transcript	ENST0000C
NA	NA	NA	NA	NA	frameshift_	ENSG0000(	transcript	ENST0000C
NA	NA	NA	NA	NA	stop_lost	ENSG0000(	transcript	ENST0000C
NA	NA	NA	NA	NA	structural_	ENSG0000(	interaction	3RNY:B_46
NA	NA	NA	NA	NA	frameshift_	ENSG0000(	transcript	ENST0000C
NA	Uncertain_	Immunode	NA	NA	frameshift_	ENSG0000(	transcript	ENST0000C
NA	NA	NA	NA	NA	frameshift_	ENSG0000(	transcript	ENST0000C
NA	NA	NA	NA	NA	frameshift_	ENSG0000(	transcript	ENST0000C
NA	Benign	not_provid	NA	NA	structural_	ENSG0000(	interaction	2VCY:B_19
NA	NA	NA	NA	NA	splice_don	ENSG0000(	transcript	ENST0000C
NA	NA	NA	NA	NA	splice_don	ENSG0000(	transcript	ENST0000C
NA	NA	NA	NA	NA	frameshift_	ENSG0000(	transcript	ENST0000C
NA	NA	NA	NA	NA	structural_	ENSG0000(	interaction	2XU7:B_15
NA	NA	NA	NA	NA	frameshift_	ENSG0000(	transcript	ENST0000C
NA	Conflicting_	Reticular_c	0.00423	0.00220	structural_	ENSG0000(	interaction	2C9Y:A_12
NA	NA	NA	NA	NA	structural_	ENSG0000(	interaction	2C9Y:A_18
NA	NA	NA	NA	NA	frameshift_	ENSG0000(	transcript	ENST0000C
NA	NA	NA	NA	NA	frameshift_	ENSG0000(	transcript	ENST0000C
NA	NA	NA	NA	NA	frameshift_	ENSG0000(	transcript	ENST0000C
NA	NA	NA	NA	NA	splice_don	ENSG0000(	transcript	ENST0000C
NA	NA	NA	NA	NA	splice_acce	ENSG0000(	transcript	ENST0000C
NA	NA	NA	NA	NA	structural_	ENSG0000(	interaction	3MTC:A_2
NA	NA	NA	NA	NA	splice_don	ENSG0000(	transcript	ENST0000C
NA	NA	NA	NA	NA	frameshift_	ENSG0000(	transcript	ENST0000C
NA	Benign/Like	History_of_	0.06666	0.07388	structural_	ENSG0000(	interaction	3GRO:A_1
NA	NA	NA	NA	NA	splice_acce	ENSG0000(	transcript	ENST0000C
NA	NA	NA	NA	NA	splice_acce	ENSG0000(	transcript	ENST0000C
NA	NA	NA	NA	NA	splice_acce	ENSG0000(	transcript	ENST0000C
NA	NA	NA	NA	NA	frameshift_	ENSG0000(	transcript	ENST0000C
NA	NA	NA	NA	NA	frameshift_	ENSG0000(	transcript	ENST0000C

NA	Benign	Immunode	0.02680	0.04493	structural_	ENSG0000(	interaction	2VO1:A_16
NA	NA	NA	NA	NA	frameshift_	ENSG0000(	transcript	ENST0000C
NA	NA	NA	NA	NA	frameshift_	ENSG0000(	transcript	ENST0000C
NA	NA	NA	NA	NA	splice_don	ENSG0000(	transcript	ENST0000C
NA	NA	NA	NA	NA	splice_acce	ENSG0000(	transcript	ENST0000C
NA	NA	NA	NA	NA	protein_pr	ENSG0000(	interaction	2XWU:A_5
NA	NA	NA	NA	NA	splice_acce	ENSG0000(	transcript	ENST0000C
NA	NA	NA	NA	NA	structural_	ENSG0000(	interaction	3HM5:A_1
NA	NA	NA	NA	NA	splice_don	ENSG0000(	transcript	ENST0000C
NA	NA	NA	NA	NA	splice_acce	ENSG0000(	transcript	ENST0000C
NA	NA	NA	NA	NA	structural_	ENSG0000(	interaction	4FP9:A_97
NA	NA	NA	NA	NA	stop_gaine	ENSG0000(	transcript	ENST0000C
NA	Benign	not_specifi	0.14745	0.13538	frameshift_	ENSG0000(	transcript	ENST0000C
NA	NA	NA	NA	NA	stop_gaine	ENSG0000(	transcript	ENST0000C
NA	NA	NA	NA	NA	splice_don	ENSG0000(	transcript	ENST0000C
NA	NA	NA	NA	NA	frameshift_	ENSG0000(	transcript	ENST0000C
NA	NA	NA	NA	NA	frameshift_	ENSG0000(	transcript	ENST0000C
NA	NA	NA	NA	NA	stop_gaine	ENSG0000(	transcript	ENST0000C
NA	NA	NA	NA	NA	frameshift_	ENSG0000(	transcript	ENST0000C
NA	NA	NA	NA	NA	frameshift_	ENSG0000(	transcript	ENST0000C
NA	NA	NA	NA	NA	frameshift_	ENSG0000(	transcript	ENST0000C
NA	NA	NA	NA	NA	splice_don	ENSG0000(	transcript	ENST0000C
NA	NA	NA	NA	NA	start_lost	ENSG0000(	transcript	ENST0000C
NA	NA	NA	NA	NA	splice_don	ENSG0000(	transcript	ENST0000C
NA	NA	NA	NA	NA	splice_acce	ENSG0000(	transcript	ENST0000C
NA	NA	NA	NA	NA	splice_don	ENSG0000(	transcript	ENST0000C
NA	NA	NA	NA	NA	frameshift_	ENSG0000(	transcript	ENST0000C
NA	NA	NA	NA	NA	frameshift_	ENSG0000(	transcript	ENST0000C
NA	NA	NA	NA	NA	frameshift_	ENSG0000(	transcript	ENST0000C
NA	NA	NA	NA	NA	structural_	ENSG0000(	interaction	3FO5:A_40
NA	NA	NA	NA	NA	structural_	ENSG0000(	interaction	3FO5:A_39
NA	NA	NA	NA	NA	structural_	ENSG0000(	interaction	3FO5:A_42
Blood_prot	Benign	not_provid	NA	NA	frameshift_	ENSG0000(	transcript	ENST0000C
NA	NA	NA	NA	NA	stop_gaine	ENSG0000(	transcript	ENST0000C
NA	NA	NA	NA	NA	stop_gaine	ENSG0000(	transcript	ENST0000C
NA	NA	NA	NA	NA	frameshift_	ENSG0000(	transcript	ENST0000C
NA	Benign/Like	Familial_hy	0.85440	0.86921	structural_	ENSG0000(	interaction	2P4E:A_46
NA	NA	NA	NA	NA	splice_don	ENSG0000(	transcript	ENST0000C
NA	NA	NA	NA	NA	splice_acce	ENSG0000(	transcript	ENST0000C
NA	NA	NA	NA	NA	structural_	ENSG0000(	interaction	2H6D:A_11
NA	Pathogenic	Compleme	0.00109	0.00120	stop_gaine	ENSG0000(	transcript	ENST0000C
NA	NA	NA	NA	NA	splice_don	ENSG0000(	transcript	ENST0000C
NA	NA	NA	NA	NA	splice_don	ENSG0000(	transcript	ENST0000C
NA	NA	NA	NA	NA	splice_don	ENSG0000(	transcript	ENST0000C
NA	NA	NA	NA	NA	splice_acce	ENSG0000(	transcript	ENST0000C
NA	NA	NA	NA	NA	frameshift_	ENSG0000(	transcript	ENST0000C
NA	NA	NA	NA	NA	frameshift_	ENSG0000(	transcript	ENST0000C

NA	NA	NA	NA	NA	stop_gaine	ENSG0000(	transcript	ENST0000C
NA	Benign	not_provid	NA	NA	frameshift_	ENSG0000(	transcript	ENST0000C
NA	NA	NA	NA	NA	splice_don	ENSG0000(	transcript	ENST0000C
NA	NA	NA	NA	NA	splice_don	ENSG0000(	transcript	ENST0000C
NA	Likely_beni	not_provid	NA	0.00359	splice_don	ENSG0000(	transcript	ENST0000C
NA	NA	NA	NA	NA	structural_	ENSG0000(	interaction	4L00:A_69
NA	NA	NA	NA	NA	structural_	ENSG0000(	interaction	4L00:A_58
NA	NA	NA	NA	NA	splice_acce	ENSG0000(	transcript	ENST0000C
NA	NA	NA	NA	NA	structural_	ENSG0000(	interaction	3G45:A_43
NA	NA	NA	NA	NA	splice_don	ENSG0000(	transcript	ENST0000C
NA	Benign	not_provid	NA	NA	frameshift_	ENSG0000(	transcript	ENST0000C
NA	NA	NA	NA	NA	splice_don	ENSG0000(	transcript	ENST0000C
NA	NA	NA	NA	NA	frameshift_	ENSG0000(	transcript	ENST0000C
NA	Conflicting_	Cystathioni	NA	0.00260	structural_	ENSG0000(	interaction	2NMP:B_4
NA	NA	NA	NA	NA	splice_acce	ENSG0000(	transcript	ENST0000C
NA	NA	NA	NA	NA	splice_don	ENSG0000(	transcript	ENST0000C
NA	NA	NA	NA	NA	structural_	ENSG0000(	interaction	1YB5:A_15
NA	NA	NA	NA	NA	start_lost	ENSG0000(	transcript	ENST0000C
NA	NA	NA	NA	NA	splice_don	ENSG0000(	transcript	ENST0000C
NA	NA	NA	NA	NA	frameshift_	ENSG0000(	transcript	ENST0000C
NA	NA	NA	NA	NA	splice_acce	ENSG0000(	transcript	ENST0000C
NA	NA	NA	NA	NA	splice_don	ENSG0000(	transcript	ENST0000C
NA	NA	NA	NA	NA	stop_gaine	ENSG0000(	transcript	ENST0000C
NA	NA	NA	NA	NA	splice_acce	ENSG0000(	transcript	ENST0000C
NA	NA	NA	NA	NA	splice_acce	ENSG0000(	transcript	ENST0000C
NA	NA	NA	NA	NA	splice_acce	ENSG0000(	transcript	ENST0000C
NA	NA	NA	NA	NA	splice_acce	ENSG0000(	transcript	ENST0000C
NA	NA	NA	NA	NA	splice_don	ENSG0000(	transcript	ENST0000C
NA	NA	NA	NA	NA	splice_don	ENSG0000(	transcript	ENST0000C
NA	NA	NA	NA	NA	splice_don	ENSG0000(	transcript	ENST0000C
NA	NA	NA	NA	NA	structural_	ENSG0000(	interaction	4CRS:A_67
NA	NA	NA	NA	NA	stop_gaine	ENSG0000(	transcript	ENST0000C
NA	NA	NA	NA	NA	frameshift_	ENSG0000(	transcript	ENST0000C
NA	NA	NA	NA	NA	structural_	ENSG0000(	interaction	1F5N:A_23
NA	NA	NA	NA	NA	structural_	ENSG0000(	interaction	1DG3:A_13
NA	NA	NA	NA	NA	frameshift_	ENSG0000(	transcript	ENST0000C
NA	NA	NA	NA	NA	frameshift_	ENSG0000(	transcript	ENST0000C
NA	NA	NA	NA	NA	frameshift_	ENSG0000(	transcript	ENST0000C
NA	NA	NA	NA	NA	frameshift_	ENSG0000(	transcript	ENST0000C
NA	NA	NA	NA	NA	splice_acce	ENSG0000(	transcript	ENST0000C
NA	NA	NA	NA	NA	splice_don	ENSG0000(	transcript	ENST0000C
NA	NA	NA	NA	NA	splice_acce	ENSG0000(	transcript	ENST0000C
NA	NA	NA	NA	NA	splice_acce	ENSG0000(	transcript	ENST0000C
NA	Benign	Glycogen_s	0.46865	0.42712	splice_acce	ENSG0000(	transcript	ENST0000C
NA	NA	NA	NA	NA	splice_don	ENSG0000(	transcript	ENST0000C
Lymphocyt	NA	NA	NA	NA	splice_don	ENSG0000(	transcript	ENST0000C
NA	NA	NA	NA	NA	splice_don	ENSG0000(	transcript	ENST0000C

NA	NA	NA	NA	NA	splice_acce	ENSG0000(	transcript	ENST0000C
Red_cell_d	NA	NA	NA	NA	structural_	ENSG0000(	interaction	4Y30:A_19
NA	Likely_beni	not_provid	NA	NA	structural_	ENSG0000(	interaction	3VBB:A_22
NA	NA	NA	NA	NA	stop_gaine	ENSG0000(	transcript	ENST0000C
NA	NA	NA	NA	NA	stop_gaine	ENSG0000(	transcript	ENST0000C
NA	NA	NA	NA	NA	structural_	ENSG0000(	interaction	2IHB:A_35
NA	NA	NA	NA	NA	splice_acce	ENSG0000(	transcript	ENST0000C
NA	NA	NA	NA	NA	splice_acce	ENSG0000(	transcript	ENST0000C
NA	NA	NA	NA	NA	splice_acce	ENSG0000(	transcript	ENST0000C
NA	NA	NA	NA	NA	splice_don	ENSG0000(	transcript	ENST0000C
NA	NA	NA	NA	NA	splice_don	ENSG0000(	transcript	ENST0000C
NA	NA	NA	NA	NA	splice_acce	ENSG0000(	transcript	ENST0000C
NA	NA	NA	NA	NA	splice_acce	ENSG0000(	transcript	ENST0000C
Blood_prot	NA	NA	NA	NA	structural_	ENSG0000(	interaction	4AY1:A_18
NA	Benign	not_specifi	NA	0.10883	structural_	ENSG0000(	interaction	4AY1:A_31
NA	Benign	not_specifi	NA	0.10883	structural_	ENSG0000(	interaction	4AY1:A_31
NA	NA	NA	NA	NA	frameshift_	ENSG0000(	transcript	ENST0000C
NA	NA	NA	NA	NA	structural_	ENSG0000(	interaction	2YBU:F_33
NA	NA	NA	NA	NA	splice_acce	ENSG0000(	transcript	ENST0000C
NA	Uncertain_	Muscle_AN	0.08714	0.03814	stop_gaine	ENSG0000(	transcript	ENST0000C
NA	NA	NA	NA	NA	splice_acce	ENSG0000(	transcript	ENST0000C
NA	NA	NA	NA	NA	stop_gaine	ENSG0000(	transcript	ENST0000C
NA	NA	NA	NA	NA	splice_don	ENSG0000(	transcript	ENST0000C
NA	NA	NA	NA	NA	splice_don	ENSG0000(	transcript	ENST0000C
NA	NA	NA	NA	NA	frameshift_	ENSG0000(	transcript	ENST0000C
NA	NA	NA	NA	NA	frameshift_	ENSG0000(	transcript	ENST0000C
NA	NA	NA	NA	NA	structural_	ENSG0000(	interaction	2XUB:A_84
NA	NA	NA	NA	NA	frameshift_	ENSG0000(	transcript	ENST0000C
NA	NA	NA	NA	NA	frameshift_	ENSG0000(	transcript	ENST0000C
NA	NA	NA	NA	NA	frameshift_	ENSG0000(	transcript	ENST0000C
NA	NA	NA	NA	NA	stop_gaine	ENSG0000(	transcript	ENST0000C
NA	NA	NA	NA	NA	frameshift_	ENSG0000(	transcript	ENST0000C
NA	NA	NA	NA	NA	splice_acce	ENSG0000(	transcript	ENST0000C
NA	NA	NA	NA	NA	stop_gaine	ENSG0000(	transcript	ENST0000C
NA	NA	NA	NA	NA	frameshift_	ENSG0000(	transcript	ENST0000C
NA	NA	NA	NA	NA	stop_lost	ENSG0000(	transcript	ENST0000C
NA	NA	NA	NA	NA	splice_acce	ENSG0000(	transcript	ENST0000C
NA	NA	NA	NA	NA	splice_acce	ENSG0000(	transcript	ENST0000C
NA	NA	NA	NA	NA	splice_acce	ENSG0000(	transcript	ENST0000C
NA	NA	NA	NA	NA	splice_acce	ENSG0000(	transcript	ENST0000C
NA	NA	NA	NA	NA	splice_acce	ENSG0000(	transcript	ENST0000C
NA	NA	NA	NA	NA	splice_acce	ENSG0000(	transcript	ENST0000C
NA	NA	NA	NA	NA	frameshift_	ENSG0000(	transcript	ENST0000C
NA	NA	NA	NA	NA	splice_don	ENSG0000(	transcript	ENST0000C
NA	NA	NA	NA	NA	splice_don	ENSG0000(	transcript	ENST0000C
NA	NA	NA	NA	NA	start_lost	ENSG0000(	transcript	ENST0000C
NA	NA	NA	NA	NA	splice_acce	ENSG0000(	transcript	ENST0000C

NA	NA	NA	NA	NA	splice_acce	ENSG0000(	transcript	ENST0000C
NA	NA	NA	NA	NA	splice_acce	ENSG0000(	transcript	ENST0000C
NA	NA	NA	NA	NA	splice_acce	ENSG0000(	transcript	ENST0000C
NA	NA	NA	NA	NA	splice_acce	ENSG0000(	transcript	ENST0000C
NA	NA	NA	NA	NA	splice_acce	ENSG0000(	transcript	ENST0000C
NA	NA	NA	NA	NA	splice_don	ENSG0000(	transcript	ENST0000C
NA	NA	NA	NA	NA	bidirection	ENSG0000(	gene_varia	ENSG0000C
NA	NA	NA	NA	NA	splice_acce	ENSG0000(	transcript	ENST0000C
NA	NA	NA	NA	NA	splice_acce	ENSG0000(	transcript	ENST0000C
NA	NA	NA	NA	NA	splice_acce	ENSG0000(	transcript	ENST0000C
NA	NA	NA	NA	NA	structural_	ENSG0000(	interaction	4AE7:A_14
NA	NA	NA	NA	NA	stop_gaine	ENSG0000(	transcript	ENST0000C
NA	NA	NA	NA	NA	frameshift_	ENSG0000(	transcript	ENST0000C
NA	Conflicting_	FLG-Relate	NA	NA	frameshift_	ENSG0000(	transcript	ENST0000C
Allergic_dis	Pathogenic	Dermatitis,	NA	0.00339	stop_gaine	ENSG0000(	transcript	ENST0000C
NA	NA	NA	NA	NA	splice_acce	ENSG0000(	transcript	ENST0000C
NA	NA	NA	NA	NA	stop_gaine	ENSG0000(	transcript	ENST0000C
NA	NA	NA	NA	NA	stop_lost	ENSG0000(	transcript	ENST0000C
NA	NA	NA	NA	NA	splice_don	ENSG0000(	transcript	ENST0000C
NA	NA	NA	NA	NA	frameshift_	ENSG0000(	transcript	ENST0000C
NA	NA	NA	NA	NA	structural_	ENSG0000(	interaction	3NSI:A_31-
NA	NA	NA	NA	NA	splice_acce	ENSG0000(	transcript	ENST0000C
NA	NA	NA	NA	NA	splice_acce	ENSG0000(	transcript	ENST0000C
NA	NA	NA	NA	NA	splice_acce	ENSG0000(	transcript	ENST0000C
NA	NA	NA	NA	NA	frameshift_	ENSG0000(	transcript	ENST0000C
NA	NA	NA	NA	NA	frameshift_	ENSG0000(	transcript	ENST0000C
NA	NA	NA	NA	NA	splice_acce	ENSG0000(	transcript	ENST0000C
NA	NA	NA	NA	NA	splice_acce	ENSG0000(	transcript	ENST0000C
NA	NA	NA	NA	NA	splice_acce	ENSG0000(	transcript	ENST0000C
NA	NA	NA	NA	NA	splice_acce	ENSG0000(	transcript	ENST0000C
NA	NA	NA	NA	NA	splice_acce	ENSG0000(	transcript	ENST0000C
Magnesiun	NA	NA	NA	NA	splice_acce	ENSG0000(	transcript	ENST0000C
NA	NA	NA	NA	NA	splice_acce	ENSG0000(	transcript	ENST0000C
NA	NA	NA	NA	NA	splice_acce	ENSG0000(	transcript	ENST0000C
NA	NA	NA	NA	NA	splice_acce	ENSG0000(	transcript	ENST0000C
NA	NA	NA	NA	NA	frameshift_	ENSG0000(	transcript	ENST0000C
NA	NA	NA	NA	NA	splice_acce	ENSG0000(	transcript	ENST0000C
NA	NA	NA	NA	NA	splice_acce	ENSG0000(	transcript	ENST0000C
NA	NA	NA	NA	NA	splice_acce	ENSG0000(	transcript	ENST0000C
NA	NA	NA	NA	NA	splice_acce	ENSG0000(	transcript	ENST0000C
NA	NA	NA	NA	NA	splice_acce	ENSG0000(	transcript	ENST0000C
NA	NA	NA	NA	NA	splice_acce	ENSG0000(	transcript	ENST0000C
NA	NA	NA	NA	NA	stop_gaine	ENSG0000(	transcript	ENST0000C
NA	NA	NA	NA	NA	frameshift_	ENSG0000(	transcript	ENST0000C
NA	NA	NA	NA	NA	frameshift_	ENSG0000(	transcript	ENST0000C
NA	NA	NA	NA	NA	frameshift_	ENSG0000(	transcript	ENST0000C
NA	NA	NA	NA	NA	stop_gaine	ENSG0000(	transcript	ENST0000C

NA	Benign	Hereditary_	NA	0.21286	structural_	ENSG0000(	interaction	4AOJ:A_55
NA	NA	NA	NA	NA	structural_	ENSG0000(	interaction	1HTJ:F_447
NA	NA	NA	NA	NA	frameshift_	ENSG0000(	transcript	ENST0000C
NA	NA	NA	NA	NA	splice_acce	ENSG0000(	transcript	ENST0000C
NA	NA	NA	NA	NA	stop_gaine	ENSG0000(	transcript	ENST0000C
NA	NA	NA	NA	NA	stop_gaine	ENSG0000(	transcript	ENST0000C
NA	NA	NA	NA	NA	stop_gaine	ENSG0000(	transcript	ENST0000C
NA	NA	NA	NA	NA	stop_gaine	ENSG0000(	transcript	ENST0000C
NA	NA	NA	NA	NA	structural_	ENSG0000(	interaction	3RLN:A_61
Psoriasis	NA	NA	NA	NA	structural_	ENSG0000(	interaction	4O7Q:A_32
NA	NA	NA	NA	NA	frameshift_	ENSG0000(	transcript	ENST0000C
NA	NA	NA	NA	NA	stop_gaine	ENSG0000(	transcript	ENST0000C
NA	NA	NA	NA	NA	structural_	ENSG0000(	interaction	4ERC:A_10
NA	NA	NA	NA	NA	structural_	ENSG0000(	interaction	4ERC:A_99
NA	NA	NA	NA	NA	stop_lost	ENSG0000(	transcript	ENST0000C
NA	NA	NA	NA	NA	stop_gaine	ENSG0000(	transcript	ENST0000C
NA	NA	NA	NA	NA	frameshift_	ENSG0000(	transcript	ENST0000C
NA	Benign	not_provid	NA	NA	structural_	ENSG0000(	interaction	1NBQ:A_14
NA	NA	NA	NA	NA	frameshift_	ENSG0000(	transcript	ENST0000C
NA	NA	NA	NA	NA	frameshift_	ENSG0000(	transcript	ENST0000C
NA	NA	NA	NA	NA	frameshift_	ENSG0000(	transcript	ENST0000C
NA	NA	NA	NA	NA	frameshift_	ENSG0000(	transcript	ENST0000C
NA	NA	NA	NA	NA	frameshift_	ENSG0000(	transcript	ENST0000C
NA	NA	NA	NA	NA	structural_	ENSG0000(	interaction	4JJH:A_42-.
NA	NA	NA	NA	NA	structural_	ENSG0000(	interaction	4JJH:A_53-.
NA	Benign	not_specifi	0.10317	0.05611	stop_gaine	ENSG0000(	transcript	ENST0000C
NA	NA	NA	NA	NA	structural_	ENSG0000(	interaction	3FE1:A_19
NA	NA	NA	NA	NA	splice_don	ENSG0000(	transcript	ENST0000C
NA	NA	NA	NA	NA	structural_	ENSG0000(	interaction	4JNB:A_13
NA	NA	NA	NA	NA	splice_don	ENSG0000(	transcript	ENST0000C
NA	NA	NA	NA	NA	structural_	ENSG0000(	interaction	1JV1:A_24
NA	NA	NA	NA	NA	splice_acce	ENSG0000(	transcript	ENST0000C
NA	NA	NA	NA	NA	splice_don	ENSG0000(	transcript	ENST0000C
NA	NA	NA	NA	NA	splice_acce	ENSG0000(	transcript	ENST0000C
NA	NA	NA	NA	NA	structural_	ENSG0000(	interaction	1UDW:A_2
NA	NA	NA	NA	NA	splice_acce	ENSG0000(	transcript	ENST0000C
NA	NA	NA	NA	NA	splice_acce	ENSG0000(	transcript	ENST0000C
NA	NA	NA	NA	NA	splice_don	ENSG0000(	transcript	ENST0000C
NA	NA	NA	NA	NA	splice_don	ENSG0000(	transcript	ENST0000C
NA	NA	NA	NA	NA	splice_acce	ENSG0000(	transcript	ENST0000C
NA	NA	NA	NA	NA	splice_acce	ENSG0000(	transcript	ENST0000C
NA	NA	NA	NA	NA	structural_	ENSG0000(	interaction	4CLF:A_29
NA	Benign	not_specifi	NA	NA	frameshift_	ENSG0000(	transcript	ENST0000C
NA	NA	NA	NA	NA	stop_gaine	ENSG0000(	transcript	ENST0000C
NA	Benign	not_specifi	0.06059	0.10284	frameshift_	ENSG0000(	transcript	ENST0000C
NA	Benign	not_provid	NA	NA	stop_gaine	ENSG0000(	transcript	ENST0000C
NA	Benign	not_provid	NA	NA	frameshift_	ENSG0000(	transcript	ENST0000C

NA	Benign/Like	Glaucoma|	NA	0.01218	structural_	ENSG0000(	interaction	4WXU:A_3
NA	Conflicting_	Glaucoma|	0.00388	0.00160	structural_	ENSG0000(	interaction	4WXU:A_2
NA	NA	NA	NA	NA	splice_don	ENSG0000(	transcript	ENST0000C
NA	NA	NA	NA	NA	splice_don	ENSG0000(	transcript	ENST0000C
NA	NA	NA	NA	NA	frameshift_	ENSG0000(	transcript	ENST0000C
NA	NA	NA	NA	NA	frameshift_	ENSG0000(	transcript	ENST0000C
NA	NA	NA	NA	NA	frameshift_	ENSG0000(	transcript	ENST0000C
NA	NA	NA	NA	NA	splice_don	ENSG0000(	transcript	ENST0000C
NA	Conflicting_	Muscular_c	0.64659	0.63538	splice_acce	ENSG0000(	transcript	ENST0000C
NA	NA	NA	NA	NA	splice_acce	ENSG0000(	transcript	ENST0000C
NA	Benign	Chronic_gr	0.48949	0.49161	structural_	ENSG0000(	interaction	1E96:B_11
NA	NA	NA	NA	NA	stop_lost	ENSG0000(	transcript	ENST0000C
NA	NA	NA	NA	NA	splice_don	ENSG0000(	transcript	ENST0000C
NA	NA	NA	NA	NA	frameshift_	ENSG0000(	transcript	ENST0000C
NA	NA	NA	NA	NA	frameshift_	ENSG0000(	transcript	ENST0000C
NA	NA	NA	NA	NA	frameshift_	ENSG0000(	transcript	ENST0000C
NA	NA	NA	NA	NA	frameshift_	ENSG0000(	transcript	ENST0000C
NA	NA	NA	NA	NA	frameshift_	ENSG0000(	transcript	ENST0000C
NA	NA	NA	NA	NA	frameshift_	ENSG0000(	transcript	ENST0000C
Blood_prot	NA	NA	NA	NA	start_lost	ENSG0000(	transcript	ENST0000C
NA	NA	NA	NA	NA	splice_acce	ENSG0000(	transcript	ENST0000C
NA	NA	NA	NA	NA	splice_acce	ENSG0000(	transcript	ENST0000C
NA	NA	NA	NA	NA	frameshift_	ENSG0000(	transcript	ENST0000C
NA	Conflicting_	CFHR5_def	NA	NA	frameshift_	ENSG0000(	transcript	ENST0000C
NA	Conflicting_	CFHR5_def	NA	NA	frameshift_	ENSG0000(	transcript	ENST0000C
NA	NA	NA	NA	NA	splice_acce	ENSG0000(	transcript	ENST0000C
NA	NA	NA	NA	NA	stop_gaine	ENSG0000(	transcript	ENST0000C
NA	NA	NA	NA	NA	splice_acce	ENSG0000(	transcript	ENST0000C
NA	NA	NA	NA	NA	splice_acce	ENSG0000(	transcript	ENST0000C
NA	NA	NA	NA	NA	splice_acce	ENSG0000(	transcript	ENST0000C
NA	NA	NA	NA	NA	splice_acce	ENSG0000(	transcript	ENST0000C
NA	NA	NA	NA	NA	splice_regi	ENSG0000(	transcript	ENST0000C
NA	NA	NA	NA	NA	splice_regi	ENSG0000(	transcript	ENST0000C
NA	NA	NA	NA	NA	frameshift_	ENSG0000(	transcript	ENST0000C
NA	Benign	not_provid	NA	NA	frameshift_	ENSG0000(	transcript	ENST0000C
NA	Benign	not_provid	NA	NA	frameshift_	ENSG0000(	transcript	ENST0000C
NA	NA	NA	NA	NA	splice_don	ENSG0000(	transcript	ENST0000C
NA	NA	NA	NA	NA	frameshift_	ENSG0000(	transcript	ENST0000C
NA	NA	NA	NA	NA	protein_pr	ENSG0000(	interaction	4CCG:A_5-
NA	NA	NA	NA	NA	splice_acce	ENSG0000(	transcript	ENST0000C
NA	NA	NA	NA	NA	splice_acce	ENSG0000(	transcript	ENST0000C
NA	NA	NA	NA	NA	structural_	ENSG0000(	interaction	5A1F:A_62
NA	NA	NA	NA	NA	frameshift_	ENSG0000(	transcript	ENST0000C
NA	NA	NA	NA	NA	bidirection	ENSG0000(	gene_varia	ENSG00000
NA	NA	NA	NA	NA	structural_	ENSG0000(	interaction	1HJV:A_30
NA	NA	NA	NA	NA	structural_	ENSG0000(	interaction	1HJV:C_10
NA	Benign/Like	Chitotriosic	NA	NA	stop_gaine	ENSG0000(	transcript	ENST0000C

NA	NA	NA	NA	NA	splice_don	ENSG0000	transcript	ENST0000
NA	NA	NA	NA	NA	splice_don	ENSG0000	transcript	ENST0000
NA	Benign	not_specifi	0.29830	0.22185	frameshift_	ENSG0000	transcript	ENST0000
NA	NA	NA	NA	NA	splice_acce	ENSG0000	transcript	ENST0000
NA	NA	NA	NA	NA	splice_don	ENSG0000	transcript	ENST0000
NA	NA	NA	NA	NA	splice_don	ENSG0000	transcript	ENST0000
NA	NA	NA	NA	NA	splice_don	ENSG0000	transcript	ENST0000
NA	NA	NA	NA	NA	frameshift_	ENSG0000	transcript	ENST0000
NA	NA	NA	NA	NA	structural_	ENSG0000	interaction	3KC3:B_17
NA	NA	NA	NA	NA	splice_don	ENSG0000	transcript	ENST0000
NA	NA	NA	NA	NA	frameshift_	ENSG0000	transcript	ENST0000
NA	NA	NA	NA	NA	frameshift_	ENSG0000	transcript	ENST0000
NA	NA	NA	NA	NA	splice_acce	ENSG0000	transcript	ENST0000
NA	NA	NA	NA	NA	splice_acce	ENSG0000	transcript	ENST0000
NA	NA	NA	NA	NA	splice_acce	ENSG0000	transcript	ENST0000
Red_cell_d	Benign	not_specifi	0.55836	0.60304	structural_	ENSG0000	interaction	2W5B:A_4
NA	NA	NA	NA	NA	splice_don	ENSG0000	transcript	ENST0000
NA	NA	NA	NA	NA	splice_don	ENSG0000	transcript	ENST0000
NA	NA	NA	NA	NA	structural_	ENSG0000	interaction	2Y1H:A_7-
NA	NA	NA	NA	NA	splice_acce	ENSG0000	transcript	ENST0000
NA	NA	NA	NA	NA	splice_don	ENSG0000	transcript	ENST0000
NA	NA	NA	NA	NA	splice_acce	ENSG0000	transcript	ENST0000
NA	NA	NA	NA	NA	splice_don	ENSG0000	transcript	ENST0000
NA	Benign	not_provid	NA	NA	frameshift_	ENSG0000	transcript	ENST0000
NA	NA	NA	NA	NA	splice_don	ENSG0000	transcript	ENST0000
NA	NA	NA	NA	NA	structural_	ENSG0000	interaction	2WEF:A_1
NA	NA	NA	NA	NA	structural_	ENSG0000	interaction	2WEF:A_6
NA	NA	NA	NA	NA	splice_acce	ENSG0000	transcript	ENST0000
NA	NA	NA	NA	NA	splice_don	ENSG0000	transcript	ENST0000
NA	protective,	Legionellos	0.05564	0.05032	stop_gaine	ENSG0000	transcript	ENST0000
NA	NA	NA	NA	NA	structural_	ENSG0000	interaction	1KFU:L_68-
NA	NA	NA	NA	NA	structural_	ENSG0000	interaction	1KFU:L_10
NA	NA	NA	NA	NA	structural_	ENSG0000	interaction	2X8A:A_68
NA	NA	NA	NA	NA	frameshift_	ENSG0000	transcript	ENST0000
NA	NA	NA	NA	NA	frameshift_	ENSG0000	transcript	ENST0000
NA	NA	NA	NA	NA	frameshift_	ENSG0000	transcript	ENST0000
NA	NA	NA	NA	NA	splice_acce	ENSG0000	transcript	ENST0000
NA	NA	NA	NA	NA	splice_acce	ENSG0000	transcript	ENST0000
NA	NA	NA	NA	NA	splice_don	ENSG0000	transcript	ENST0000
NA	NA	NA	NA	NA	splice_don	ENSG0000	transcript	ENST0000
NA	NA	NA	NA	NA	splice_don	ENSG0000	transcript	ENST0000
NA	NA	NA	NA	NA	splice_don	ENSG0000	transcript	ENST0000
NA	NA	NA	NA	NA	splice_don	ENSG0000	transcript	ENST0000
NA	NA	NA	NA	NA	splice_don	ENSG0000	transcript	ENST0000
NA	NA	NA	NA	NA	splice_don	ENSG0000	transcript	ENST0000
NA	NA	NA	NA	NA	splice_don	ENSG0000	transcript	ENST0000
NA	NA	NA	NA	NA	splice_don	ENSG0000	transcript	ENST0000

NA	NA	NA	NA	NA	structural_	ENSG0000(	interaction	3OD8:A_13
NA	Benign/Like	Coenzyme_	0.02091	0.00559	structural_	ENSG0000(	interaction	4PED:A_33
NA	Benign/Like	Coenzyme_	0.01909	0.00419	structural_	ENSG0000(	interaction	4PED:A_32
NA	Benign	Coenzyme_	NA	0.00180	structural_	ENSG0000(	interaction	4PED:A_37
NA	Benign	Coenzyme_	NA	0.23862	structural_	ENSG0000(	interaction	4PED:A_48
NA	Benign	Coenzyme_	0.40536	0.31390	structural_	ENSG0000(	interaction	4PED:A_57
NA	NA	NA	NA	NA	stop_gaine	ENSG0000(	transcript	ENST0000C
NA	NA	NA	NA	NA	structural_	ENSG0000(	interaction	4C4K:O_32
NA	NA	NA	NA	NA	stop_gaine	ENSG0000(	transcript	ENST0000C
NA	NA	NA	NA	NA	structural_	ENSG0000(	interaction	2Q3H:A_68
NA	NA	NA	NA	NA	frameshift_	ENSG0000(	transcript	ENST0000C
NA	NA	NA	NA	NA	structural_	ENSG0000(	interaction	2FFV:A_45
NA	NA	NA	NA	NA	structural_	ENSG0000(	interaction	1ZIV:A_74-
NA	NA	NA	NA	NA	structural_	ENSG0000(	interaction	1ZIV:A_115
NA	Benign/Like	Familial_er	0.00213	0.00080	structural_	ENSG0000(	interaction	2G19:A_29
NA	NA	NA	NA	NA	stop_lost	ENSG0000(	transcript	ENST0000C
NA	NA	NA	NA	NA	splice_acce	ENSG0000(	transcript	ENST0000C
NA	NA	NA	NA	NA	structural_	ENSG0000(	interaction	3AP4:A_19
NA	NA	NA	NA	NA	structural_	ENSG0000(	interaction	3AP4:A_36
NA	NA	NA	NA	NA	splice_acce	ENSG0000(	transcript	ENST0000C
NA	NA	NA	NA	NA	stop_gaine	ENSG0000(	transcript	ENST0000C
NA	NA	NA	NA	NA	structural_	ENSG0000(	interaction	3VKM:B_9-
NA	NA	NA	NA	NA	frameshift_	ENSG0000(	transcript	ENST0000C
NA	NA	NA	NA	NA	frameshift_	ENSG0000(	transcript	ENST0000C
NA	Likely_beni	not_provid	0.00430	NA	frameshift_	ENSG0000(	transcript	ENST0000C
Keratinocyt	NA	NA	NA	NA	structural_	ENSG0000(	interaction	3QE9:Y_21
NA	NA	NA	NA	NA	structural_	ENSG0000(	interaction	3VVW:B_6(
NA	NA	NA	NA	NA	splice_don	ENSG0000(	transcript	ENST0000C
NA	NA	NA	NA	NA	structural_	ENSG0000(	interaction	2V40:A_11
NA	NA	NA	NA	NA	frameshift_	ENSG0000(	transcript	ENST0000C
NA	NA	NA	NA	NA	frameshift_	ENSG0000(	transcript	ENST0000C
NA	NA	NA	NA	NA	splice_acce	ENSG0000(	transcript	ENST0000C
NA	NA	NA	NA	NA	frameshift_	ENSG0000(	transcript	ENST0000C
NA	NA	NA	NA	NA	frameshift_	ENSG0000(	transcript	ENST0000C
Mean_plat	NA	NA	NA	NA	splice_don	ENSG0000(	transcript	ENST0000C
NA	NA	NA	NA	NA	frameshift_	ENSG0000(	transcript	ENST0000C
NA	NA	NA	NA	NA	stop_gaine	ENSG0000(	transcript	ENST0000C
NA	NA	NA	NA	NA	frameshift_	ENSG0000(	transcript	ENST0000C
NA	NA	NA	NA	NA	frameshift_	ENSG0000(	transcript	ENST0000C
NA	NA	NA	NA	NA	frameshift_	ENSG0000(	transcript	ENST0000C
NA	NA	NA	NA	NA	frameshift_	ENSG0000(	transcript	ENST0000C
NA	NA	NA	NA	NA	start_lost&	ENSG0000(	transcript	ENST0000C
NA	NA	NA	NA	NA	splice_don	ENSG0000(	transcript	ENST0000C
NA	NA	NA	NA	NA	splice_don	ENSG0000(	transcript	ENST0000C
NA	NA	NA	NA	NA	splice_don	ENSG0000(	transcript	ENST0000C
NA	NA	NA	NA	NA	splice_acce	ENSG0000(	transcript	ENST0000C
NA	NA	NA	NA	NA	splice_acce	ENSG0000(	transcript	ENST0000C

NA	NA	NA	NA	NA	stop_gaine	ENSG0000(	transcript	ENST0000C
NA	NA	NA	NA	NA	splice_don	ENSG0000(	transcript	ENST0000C
NA	NA	NA	NA	NA	splice_don	ENSG0000(	transcript	ENST0000C
NA	NA	NA	NA	NA	splice_don	ENSG0000(	transcript	ENST0000C
NA	NA	NA	NA	NA	splice_acce	ENSG0000(	transcript	ENST0000C
NA	NA	NA	NA	NA	splice_don	ENSG0000(	transcript	ENST0000C
NA	NA	NA	NA	NA	splice_don	ENSG0000(	transcript	ENST0000C
NA	NA	NA	NA	NA	frameshift_	ENSG0000(	transcript	ENST0000C
NA	NA	NA	NA	NA	splice_don	ENSG0000(	transcript	ENST0000C
NA	NA	NA	NA	NA	frameshift_	ENSG0000(	transcript	ENST0000C
NA	NA	NA	NA	NA	splice_acce	ENSG0000(	transcript	ENST0000C
NA	NA	NA	NA	NA	structural_	ENSG0000(	interaction	4GWR:A_1
NA	NA	NA	NA	NA	splice_acce	ENSG0000(	transcript	ENST0000C
NA	NA	NA	NA	NA	splice_acce	ENSG0000(	transcript	ENST0000C
NA	NA	NA	NA	NA	splice_acce	ENSG0000(	transcript	ENST0000C
NA	NA	NA	NA	NA	frameshift_	ENSG0000(	transcript	ENST0000C
Endometri	NA	NA	NA	NA	splice_acce	ENSG0000(	transcript	ENST0000C
NA	NA	NA	NA	NA	start_lost	ENSG0000(	transcript	ENST0000C
NA	NA	NA	NA	NA	splice_don	ENSG0000(	transcript	ENST0000C
NA	NA	NA	NA	NA	splice_acce	ENSG0000(	transcript	ENST0000C
NA	NA	NA	NA	NA	splice_acce	ENSG0000(	transcript	ENST0000C
NA	NA	NA	NA	NA	splice_acce	ENSG0000(	transcript	ENST0000C
NA	NA	NA	NA	NA	splice_acce	ENSG0000(	transcript	ENST0000C
NA	NA	NA	NA	NA	splice_acce	ENSG0000(	transcript	ENST0000C
NA	NA	NA	NA	NA	splice_acce	ENSG0000(	transcript	ENST0000C
NA	NA	NA	NA	NA	frameshift_	ENSG0000(	transcript	ENST0000C
NA	NA	NA	NA	NA	stop_gaine	ENSG0000(	transcript	ENST0000C
NA	NA	NA	NA	NA	frameshift_	ENSG0000(	transcript	ENST0000C
NA	NA	NA	NA	NA	stop_gaine	ENSG0000(	transcript	ENST0000C
NA	NA	NA	NA	NA	stop_gaine	ENSG0000(	transcript	ENST0000C
NA	NA	NA	NA	NA	frameshift_	ENSG0000(	transcript	ENST0000C
NA	NA	NA	NA	NA	stop_gaine	ENSG0000(	transcript	ENST0000C
NA	NA	NA	NA	NA	frameshift_	ENSG0000(	transcript	ENST0000C
NA	NA	NA	NA	NA	splice_acce	ENSG0000(	transcript	ENST0000C
NA	NA	NA	NA	NA	splice_acce	ENSG0000(	transcript	ENST0000C
NA	NA	NA	NA	NA	splice_acce	ENSG0000(	transcript	ENST0000C
NA	NA	NA	NA	NA	splice_acce	ENSG0000(	transcript	ENST0000C
NA	NA	NA	NA	NA	splice_acce	ENSG0000(	transcript	ENST0000C
Gut_microl	NA	NA	NA	NA	splice_don	ENSG0000(	transcript	ENST0000C
NA	NA	NA	NA	NA	structural_	ENSG0000(	interaction	4BY3:A_15
NA	Likely_beni	Epileptic_e	NA	NA	structural_	ENSG0000(	interaction	4BY3:A_16
NA	Benign	not_provid	NA	NA	structural_	ENSG0000(	interaction	4BY3:A_14
NA	NA	NA	NA	NA	structural_	ENSG0000(	interaction	4BY3:A_14
NA	NA	NA	NA	NA	splice_acce	ENSG0000(	transcript	ENST0000C
NA	NA	NA	NA	NA	splice_acce	ENSG0000(	transcript	ENST0000C
NA	NA	NA	NA	NA	splice_don	ENSG0000(	transcript	ENST0000C
NA	NA	NA	NA	NA	splice_don	ENSG0000(	transcript	ENST0000C

NA	NA	NA	NA	NA	stop_gaine	ENSG0000(	transcript	ENST0000C
NA	Benign/Like	Xanthinuria	0.05097	0.04014	structural_	ENSG0000(	interaction	2E1Q:A_11
NA	Benign	Xanthinuria	0.30369	0.27017	structural_	ENSG0000(	interaction	2E1Q:A_73
NA	Likely_beni	Xanthinuria	NA	0.01937	structural_	ENSG0000(	interaction	2E1Q:A_64
NA	Likely_beni	Xanthinuria	0.03575	0.04393	structural_	ENSG0000(	interaction	2E1Q:A_25
NA	Likely_beni	Xanthinuria	0.05434	0.04034	structural_	ENSG0000(	interaction	2E1Q:A_12
NA	NA	NA	NA	NA	splice_acce	ENSG0000(	transcript	ENST0000C
NA	NA	NA	NA	NA	stop_lost&	ENSG0000(	transcript	ENST0000C
NA	NA	NA	NA	NA	structural_	ENSG0000(	interaction	3UIU:A_47
NA	NA	NA	NA	NA	frameshift_	ENSG0000(	transcript	ENST0000C
NA	NA	NA	NA	NA	structural_	ENSG0000(	interaction	2AFM:A_44
NA	NA	NA	NA	NA	frameshift_	ENSG0000(	transcript	ENST0000C
NA	Conflicting_	Congenital_	0.00616	0.00419	structural_	ENSG0000(	interaction	3PM0:A_36
NA	NA	NA	NA	NA	splice_don	ENSG0000(	transcript	ENST0000C
5-HTT_brai	NA	NA	NA	NA	structural_	ENSG0000(	interaction	1SNZ:A_19
NA	NA	NA	NA	NA	splice_acce	ENSG0000(	transcript	ENST0000C
Hypospadia	NA	NA	NA	NA	structural_	ENSG0000(	interaction	2QNK:A_37
NA	NA	NA	NA	NA	splice_acce	ENSG0000(	transcript	ENST0000C
NA	NA	NA	NA	NA	splice_acce	ENSG0000(	transcript	ENST0000C
NA	NA	NA	NA	NA	splice_regi	ENSG0000(	transcript	ENST0000C
NA	NA	NA	NA	NA	splice_regi	ENSG0000(	transcript	ENST0000C
NA	NA	NA	NA	NA	splice_regi	ENSG0000(	transcript	ENST0000C
NA	NA	NA	NA	NA	splice_regi	ENSG0000(	transcript	ENST0000C
NA	NA	NA	NA	NA	splice_acce	ENSG0000(	transcript	ENST0000C
NA	NA	NA	NA	NA	splice_acce	ENSG0000(	transcript	ENST0000C
NA	Benign/Like	Cystinuria |	NA	NA	frameshift_	ENSG0000(	transcript	ENST0000C
NA	NA	NA	NA	NA	splice_acce	ENSG0000(	transcript	ENST0000C
NA	NA	NA	NA	NA	splice_acce	ENSG0000(	transcript	ENST0000C
NA	NA	NA	NA	NA	frameshift_	ENSG0000(	transcript	ENST0000C
NA	NA	NA	NA	NA	splice_don	ENSG0000(	transcript	ENST0000C
NA	NA	NA	NA	NA	frameshift_	ENSG0000(	transcript	ENST0000C
NA	NA	NA	NA	NA	splice_acce	ENSG0000(	transcript	ENST0000C
NA	NA	NA	NA	NA	splice_acce	ENSG0000(	transcript	ENST0000C
NA	NA	NA	NA	NA	splice_acce	ENSG0000(	transcript	ENST0000C
NA	NA	NA	NA	NA	stop_gaine	ENSG0000(	transcript	ENST0000C
NA	NA	NA	NA	NA	splice_don	ENSG0000(	transcript	ENST0000C
NA	Benign	not_specifi	0.00100	0.00020	structural_	ENSG0000(	interaction	3U1K:B_61
NA	Benign	not_specifi	0.48046	0.35663	structural_	ENSG0000(	interaction	3U1K:A_42
NA	NA	NA	NA	NA	splice_acce	ENSG0000(	transcript	ENST0000C
NA	NA	NA	NA	NA	splice_acce	ENSG0000(	transcript	ENST0000C
NA	NA	NA	NA	NA	splice_acce	ENSG0000(	transcript	ENST0000C
NA	NA	NA	NA	NA	splice_acce	ENSG0000(	transcript	ENST0000C
NA	NA	NA	NA	NA	splice_acce	ENSG0000(	transcript	ENST0000C
NA	NA	NA	NA	NA	frameshift_	ENSG0000(	transcript	ENST0000C
NA	NA	NA	NA	NA	splice_acce	ENSG0000(	transcript	ENST0000C
NA	NA	NA	NA	NA	splice_acce	ENSG0000(	transcript	ENST0000C
NA	NA	NA	NA	NA	splice_don	ENSG0000(	transcript	ENST0000C

NA	NA	NA	NA	NA	splice_acce	ENSG0000(	transcript	ENST0000C
NA	NA	NA	NA	NA	splice_don	ENSG0000(	transcript	ENST0000C
NA	NA	NA	NA	NA	splice_don	ENSG0000(	transcript	ENST0000C
NA	NA	NA	NA	NA	splice_don	ENSG0000(	transcript	ENST0000C
NA	NA	NA	NA	NA	stop_lost	ENSG0000(	transcript	ENST0000C
Body_mass	Benign	Multiple_m	0.39284	0.31709	start_lost	ENSG0000(	transcript	ENST0000C
NA	NA	NA	NA	NA	structural_	ENSG0000(	interaction	3C22:A_28
NA	NA	NA	NA	NA	structural_	ENSG0000(	interaction	3C22:A_23
NA	NA	NA	NA	NA	structural_	ENSG0000(	interaction	3C22:B_20
NA	Benign	Renal_tubu	0.40949	0.36881	start_lost	ENSG0000(	transcript	ENST0000C
NA	NA	NA	NA	NA	frameshift_	ENSG0000(	transcript	ENST0000C
NA	NA	NA	NA	NA	splice_acce	ENSG0000(	transcript	ENST0000C
NA	NA	NA	NA	NA	splice_acce	ENSG0000(	transcript	ENST0000C
NA	NA	NA	NA	NA	start_lost	ENSG0000(	transcript	ENST0000C
NA	NA	NA	NA	NA	structural_	ENSG0000(	interaction	2CH5:A_31
NA	NA	NA	NA	NA	structural_	ENSG0000(	interaction	2CH5:D_12
NA	NA	NA	NA	NA	frameshift_	ENSG0000(	transcript	ENST0000C
NA	NA	NA	NA	NA	stop_lost	ENSG0000(	transcript	ENST0000C
NA	Likely_beni	not_specifi	0.01638	0.01118	structural_	ENSG0000(	interaction	3RZU:A_28
NA	NA	NA	NA	NA	structural_	ENSG0000(	interaction	2NZT:B_47
NA	NA	NA	NA	NA	splice_acce	ENSG0000(	transcript	ENST0000C
NA	NA	NA	NA	NA	stop_lost	ENSG0000(	transcript	ENST0000C
NA	NA	NA	NA	NA	start_lost	ENSG0000(	transcript	ENST0000C
NA	NA	NA	NA	NA	splice_don	ENSG0000(	transcript	ENST0000C
NA	Benign	Wolcott-Ra	0.28541	0.25819	structural_	ENSG0000(	interaction	4X7L:A_59
NA	NA	NA	NA	NA	splice_acce	ENSG0000(	transcript	ENST0000C
NA	NA	NA	NA	NA	frameshift_	ENSG0000(	transcript	ENST0000C
NA	NA	NA	NA	NA	stop_gaine	ENSG0000(	transcript	ENST0000C
NA	NA	NA	NA	NA	frameshift_	ENSG0000(	transcript	ENST0000C
NA	Likely_beni	Retinitis_Pi	0.31901	0.28414	structural_	ENSG0000(	interaction	4F92:B_11
NA	NA	NA	NA	NA	frameshift_	ENSG0000(	transcript	ENST0000C
NA	NA	NA	NA	NA	frameshift_	ENSG0000(	transcript	ENST0000C
NA	NA	NA	NA	NA	start_lost	ENSG0000(	transcript	ENST0000C
NA	Benign	not_specifi	0.30124	0.14836	structural_	ENSG0000(	interaction	2OZO:A_53
NA	NA	NA	NA	NA	stop_gaine	ENSG0000(	transcript	ENST0000C
NA	NA	NA	NA	NA	stop_lost	ENSG0000(	transcript	ENST0000C
NA	NA	NA	NA	NA	start_lost	ENSG0000(	transcript	ENST0000C
NA	NA	NA	NA	NA	structural_	ENSG0000(	interaction	3GQC:A_37
NA	NA	NA	NA	NA	frameshift_	ENSG0000(	transcript	ENST0000C
NA	NA	NA	NA	NA	frameshift_	ENSG0000(	transcript	ENST0000C
NA	Benign	not_provid	NA	NA	start_lost	ENSG0000(	transcript	ENST0000C
NA	NA	NA	NA	NA	splice_don	ENSG0000(	transcript	ENST0000C
NA	NA	NA	NA	NA	splice_don	ENSG0000(	transcript	ENST0000C
NA	NA	NA	NA	NA	splice_don	ENSG0000(	transcript	ENST0000C
NA	NA	NA	NA	NA	stop_gaine	ENSG0000(	transcript	ENST0000C
NA	Benign	not_specifi	0.02418	0.01877	stop_gaine	ENSG0000(	transcript	ENST0000C
NA	NA	NA	NA	NA	structural_	ENSG0000(	interaction	2REO:A_54

NA	NA	NA	NA	NA	structural_	ENSG0000(	interaction	2REO:A_13
NA	NA	NA	NA	NA	splice_acce	ENSG0000(	transcript	ENST0000C
NA	NA	NA	NA	NA	splice_acce	ENSG0000(	transcript	ENST0000C
NA	NA	NA	NA	NA	structural_	ENSG0000(	interaction	4HI8:B_38-
NA	NA	NA	NA	NA	splice_don	ENSG0000(	transcript	ENST0000C
NA	NA	NA	NA	NA	splice_don	ENSG0000(	transcript	ENST0000C
NA	NA	NA	NA	NA	splice_acce	ENSG0000(	transcript	ENST0000C
NA	NA	NA	NA	NA	frameshift_	ENSG0000(	transcript	ENST0000C
NA	NA	NA	NA	NA	stop_gaine	ENSG0000(	transcript	ENST0000C
NA	NA	NA	NA	NA	protein_pr	ENSG0000(	interaction	3IO8:C_97-
NA	Benign	not_specifi	0.44259	NA	stop_gaine	ENSG0000(	transcript	ENST0000C
NA	NA	NA	NA	NA	splice_acce	ENSG0000(	transcript	ENST0000C
NA	NA	NA	NA	NA	splice_acce	ENSG0000(	transcript	ENST0000C
NA	NA	NA	NA	NA	splice_acce	ENSG0000(	transcript	ENST0000C
NA	NA	NA	NA	NA	splice_acce	ENSG0000(	transcript	ENST0000C
NA	NA	NA	NA	NA	splice_acce	ENSG0000(	transcript	ENST0000C
NA	Uncertain_	Retinitis_Pi	0.00038	0.00020	structural_	ENSG0000(	interaction	4M3Q:A_6
NA	Benign	not_provid	NA	NA	start_lost	ENSG0000(	transcript	ENST0000C
NA	NA	NA	NA	NA	frameshift_	ENSG0000(	transcript	ENST0000C
NA	NA	NA	NA	NA	frameshift_	ENSG0000(	transcript	ENST0000C
NA	NA	NA	NA	NA	splice_acce	ENSG0000(	transcript	ENST0000C
NA	NA	NA	NA	NA	splice_regi	ENSG0000(	transcript	ENST0000C
NA	NA	NA	NA	NA	splice_regi	ENSG0000(	transcript	ENST0000C
NA	NA	NA	NA	NA	splice_don	ENSG0000(	transcript	ENST0000C
NA	NA	NA	NA	NA	structural_	ENSG0000(	interaction	2VQ3:B_15
NA	NA	NA	NA	NA	stop_gaine	ENSG0000(	transcript	ENST0000C
NA	NA	NA	NA	NA	stop_lost	ENSG0000(	transcript	ENST0000C
NA	NA	NA	NA	NA	frameshift_	ENSG0000(	transcript	ENST0000C
NA	NA	NA	NA	NA	splice_acce	ENSG0000(	transcript	ENST0000C
NA	NA	NA	NA	NA	splice_acce	ENSG0000(	transcript	ENST0000C
NA	NA	NA	NA	NA	splice_acce	ENSG0000(	transcript	ENST0000C
NA	NA	NA	NA	NA	structural_	ENSG0000(	interaction	4K92:A_43
NA	NA	NA	NA	NA	splice_acce	ENSG0000(	transcript	ENST0000C
NA	NA	NA	NA	NA	splice_don	ENSG0000(	transcript	ENST0000C
NA	NA	NA	NA	NA	stop_gaine	ENSG0000(	transcript	ENST0000C
NA	NA	NA	NA	NA	splice_don	ENSG0000(	transcript	ENST0000C
NA	NA	NA	NA	NA	structural_	ENSG0000(	interaction	2C35:A_35
NA	NA	NA	NA	NA	frameshift_	ENSG0000(	transcript	ENST0000C
NA	NA	NA	NA	NA	frameshift_	ENSG0000(	transcript	ENST0000C
NA	NA	NA	NA	NA	splice_don	ENSG0000(	transcript	ENST0000C
NA	NA	NA	NA	NA	splice_acce	ENSG0000(	transcript	ENST0000C
NA	NA	NA	NA	NA	splice_acce	ENSG0000(	transcript	ENST0000C
Blood_prot	NA	NA	NA	NA	structural_	ENSG0000(	interaction	3E9K:A_41
NA	NA	NA	NA	NA	splice_acce	ENSG0000(	transcript	ENST0000C
NA	NA	NA	NA	NA	splice_acce	ENSG0000(	transcript	ENST0000C
NA	NA	NA	NA	NA	splice_acce	ENSG0000(	transcript	ENST0000C
NA	NA	NA	NA	NA	splice_acce	ENSG0000(	transcript	ENST0000C

Blood_prot	NA	NA	NA	NA	structural_	ENSG0000(	interaction	2WNO:A_1
NA	NA	NA	NA	NA	splice_acce	ENSG0000(	transcript	ENST0000C
NA	Uncertain_	not_provid	0.00030	NA	frameshift_	ENSG0000(	transcript	ENST0000C
NA	NA	NA	NA	NA	transcript_	ENSG0000(	transcript	ENST0000C
NA	NA	NA	NA	NA	transcript_	ENSG0000(	transcript	ENST0000C
NA	NA	NA	NA	NA	splice_don	ENSG0000(	transcript	ENST0000C
NA	Benign	Progressive	0.96419	0.91394	structural_	ENSG0000(	interaction	4C02:A_18
NA	NA	NA	NA	NA	frameshift_	ENSG0000(	transcript	ENST0000C
NA	NA	NA	NA	NA	structural_	ENSG0000(	interaction	2XRF:A_62
NA	NA	NA	NA	NA	splice_acce	ENSG0000(	transcript	ENST0000C
NA	NA	NA	NA	NA	splice_don	ENSG0000(	transcript	ENST0000C
NA	NA	NA	NA	NA	structural_	ENSG0000(	interaction	1NU6:B_4C
NA	Likely_beni	not_provid	NA	NA	structural_	ENSG0000(	interaction	1PFQ:B_20
NA	NA	NA	NA	NA	splice_acce	ENSG0000(	transcript	ENST0000C
NA	Benign/Like	Aicardi-Gou	NA	0.00140	stop_gaine	ENSG0000(	transcript	ENST0000C
NA	Conflicting_	Aicardi-Gou	NA	0.00559	splice_don	ENSG0000(	transcript	ENST0000C
NA	NA	NA	NA	NA	splice_don	ENSG0000(	transcript	ENST0000C
NA	NA	NA	NA	NA	splice_acce	ENSG0000(	transcript	ENST0000C
NA	NA	NA	NA	NA	splice_don	ENSG0000(	transcript	ENST0000C
NA	Benign	not_provid	0.50676	0.63458	splice_acce	ENSG0000(	transcript	ENST0000C
Bipolar_dis	Benign	Benign_fan	0.08200	0.08387	splice_acce	ENSG0000(	transcript	ENST0000C
NA	NA	NA	NA	NA	frameshift_	ENSG0000(	transcript	ENST0000C
NA	NA	NA	NA	NA	frameshift_	ENSG0000(	transcript	ENST0000C
Total_body	NA	NA	NA	NA	splice_don	ENSG0000(	transcript	ENST0000C
NA	NA	NA	NA	NA	splice_acce	ENSG0000(	transcript	ENST0000C
NA	NA	NA	NA	NA	frameshift_	ENSG0000(	transcript	ENST0000C
NA	NA	NA	NA	NA	splice_don	ENSG0000(	transcript	ENST0000C
NA	NA	NA	NA	NA	splice_acce	ENSG0000(	transcript	ENST0000C
NA	Benign	not_provid	NA	NA	frameshift_	ENSG0000(	transcript	ENST0000C
NA	NA	NA	NA	NA	splice_don	ENSG0000(	transcript	ENST0000C
Obesity-rel	NA	NA	NA	NA	splice_don	ENSG0000(	transcript	ENST0000C
NA	NA	NA	NA	NA	frameshift_	ENSG0000(	transcript	ENST0000C
NA	NA	NA	NA	NA	splice_don	ENSG0000(	transcript	ENST0000C
NA	Benign	Hypogonac	0.28217	0.20707	splice_acce	ENSG0000(	transcript	ENST0000C
NA	Benign	Hypogonac	0.28217	0.20707	splice_acce	ENSG0000(	transcript	ENST0000C
NA	NA	NA	NA	NA	splice_don	ENSG0000(	transcript	ENST0000C
NA	Benign	not_provid	NA	NA	frameshift_	ENSG0000(	transcript	ENST0000C
NA	NA	NA	NA	NA	splice_don	ENSG0000(	transcript	ENST0000C
NA	NA	NA	NA	NA	splice_acce	ENSG0000(	transcript	ENST0000C
NA	NA	NA	NA	NA	splice_acce	ENSG0000(	transcript	ENST0000C
NA	NA	NA	NA	NA	splice_don	ENSG0000(	transcript	ENST0000C
NA	NA	NA	NA	NA	splice_acce	ENSG0000(	transcript	ENST0000C
NA	NA	NA	NA	NA	splice_acce	ENSG0000(	transcript	ENST0000C
NA	NA	NA	NA	NA	splice_acce	ENSG0000(	transcript	ENST0000C
NA	NA	NA	NA	NA	splice_don	ENSG0000(	transcript	ENST0000C
NA	NA	NA	NA	NA	stop_gaine	ENSG0000(	transcript	ENST0000C
NA	NA	NA	NA	NA	splice_don	ENSG0000(	transcript	ENST0000C

NA	NA	NA	NA	NA	frameshift_	ENSG0000(	transcript	ENST0000C
NA	NA	NA	NA	NA	frameshift_	ENSG0000(	transcript	ENST0000C
NA	NA	NA	NA	NA	frameshift_	ENSG0000(	transcript	ENST0000C
NA	Benign/Like	Beta-hydro	NA	0.00180	structural_	ENSG0000(	interaction	3BPT:A_81
NA	Benign	not_specifi	0.52787	0.43171	start_lost	ENSG0000(	transcript	ENST0000C
NA	NA	NA	NA	NA	splice_don	ENSG0000(	transcript	ENST0000C
NA	NA	NA	NA	NA	structural_	ENSG0000(	interaction	3LM0:A_74
NA	NA	NA	NA	NA	frameshift_	ENSG0000(	transcript	ENST0000C
NA	NA	NA	NA	NA	frameshift_	ENSG0000(	transcript	ENST0000C
NA	NA	NA	NA	NA	frameshift_	ENSG0000(	transcript	ENST0000C
NA	NA	NA	NA	NA	frameshift_	ENSG0000(	transcript	ENST0000C
NA	NA	NA	NA	NA	splice_don	ENSG0000(	transcript	ENST0000C
NA	NA	NA	NA	NA	splice_don	ENSG0000(	transcript	ENST0000C
NA	NA	NA	NA	NA	start_lost	ENSG0000(	transcript	ENST0000C
NA	NA	NA	NA	NA	splice_acce	ENSG0000(	transcript	ENST0000C
NA	Benign	not_specifi	0.04538	0.03454	start_lost	ENSG0000(	transcript	ENST0000C
NA	Benign/Like	Autoimmu	0.09069	0.05272	structural_	ENSG0000(	interaction	2Y1L:C_24(
NA	Benign	Autoimmu	0.10200	0.17532	structural_	ENSG0000(	interaction	2Y1L:A_32(
NA	NA	NA	NA	NA	stop_gaine	ENSG0000(	transcript	ENST0000C
NA	NA	NA	NA	NA	splice_don	ENSG0000(	transcript	ENST0000C
NA	NA	NA	NA	NA	stop_gaine	ENSG0000(	transcript	ENST0000C
NA	NA	NA	NA	NA	frameshift_	ENSG0000(	transcript	ENST0000C
NA	NA	NA	NA	NA	frameshift_	ENSG0000(	transcript	ENST0000C
NA	NA	NA	NA	NA	frameshift_	ENSG0000(	transcript	ENST0000C
NA	Likely_beni	not_provid	NA	NA	frameshift_	ENSG0000(	transcript	ENST0000C
NA	NA	NA	NA	NA	stop_gaine	ENSG0000(	transcript	ENST0000C
NA	NA	NA	NA	NA	structural_	ENSG0000(	interaction	4GMV:A_3
NA	NA	NA	NA	NA	splice_don	ENSG0000(	transcript	ENST0000C
NA	NA	NA	NA	NA	frameshift_	ENSG0000(	transcript	ENST0000C
NA	Benign	not_specifi	0.02093	0.02456	frameshift_	ENSG0000(	transcript	ENST0000C
NA	NA	NA	NA	NA	splice_don	ENSG0000(	transcript	ENST0000C
NA	not_provid	not_specifi	0.01055	0.00359	structural_	ENSG0000(	interaction	1T09:A_13
NA	Benign	not_specifi	0.05003	0.04932	structural_	ENSG0000(	interaction	3MAP:B_14
NA	Benign	not_specifi	0.05240	0.05691	structural_	ENSG0000(	interaction	4KZO:C_10
NA	NA	NA	NA	NA	stop_gaine	ENSG0000(	transcript	ENST0000C
Breast_can	NA	NA	NA	NA	frameshift_	ENSG0000(	transcript	ENST0000C
NA	Benign/Like	Neoplasm_	0.37833	0.36621	structural_	ENSG0000(	interaction	3C5R:A_50
NA	Benign/Like	Neoplasm_	0.81342	0.77057	structural_	ENSG0000(	interaction	3C5R:A_46
NA	NA	NA	NA	NA	frameshift_	ENSG0000(	transcript	ENST0000C
NA	NA	NA	NA	NA	splice_don	ENSG0000(	transcript	ENST0000C
NA	NA	NA	NA	NA	splice_acce	ENSG0000(	transcript	ENST0000C
NA	NA	NA	NA	NA	splice_acce	ENSG0000(	transcript	ENST0000C
NA	NA	NA	NA	NA	frameshift_	ENSG0000(	transcript	ENST0000C
NA	NA	NA	NA	NA	splice_don	ENSG0000(	transcript	ENST0000C
NA	NA	NA	NA	NA	stop_lost	ENSG0000(	transcript	ENST0000C
NA	Benign	not_provid	NA	NA	splice_don	ENSG0000(	transcript	ENST0000C
NA	NA	NA	NA	NA	frameshift_	ENSG0000(	transcript	ENST0000C

NA	NA	NA	NA	NA	frameshift_	ENSG0000(	transcript	ENST0000C
NA	NA	NA	NA	NA	frameshift_	ENSG0000(	transcript	ENST0000C
NA	NA	NA	NA	NA	frameshift_	ENSG0000(	transcript	ENST0000C
NA	NA	NA	NA	NA	splice_don	ENSG0000(	transcript	ENST0000C
NA	NA	NA	NA	NA	frameshift_	ENSG0000(	transcript	ENST0000C
NA	NA	NA	NA	NA	frameshift_	ENSG0000(	transcript	ENST0000C
NA	NA	NA	NA	NA	structural_	ENSG0000(	interaction	1QQG:A_9(
NA	NA	NA	NA	NA	splice_acce	ENSG0000(	transcript	ENST0000C
NA	NA	NA	NA	NA	splice_acce	ENSG0000(	transcript	ENST0000C
NA	NA	NA	NA	NA	splice_don	ENSG0000(	transcript	ENST0000C
NA	NA	NA	NA	NA	splice_don	ENSG0000(	transcript	ENST0000C
NA	NA	NA	NA	NA	splice_don	ENSG0000(	transcript	ENST0000C
NA	NA	NA	NA	NA	splice_don	ENSG0000(	transcript	ENST0000C
NA	NA	NA	NA	NA	stop_gaine	ENSG0000(	transcript	ENST0000C
NA	NA	NA	NA	NA	splice_acce	ENSG0000(	transcript	ENST0000C
NA	NA	NA	NA	NA	splice_acce	ENSG0000(	transcript	ENST0000C
NA	NA	NA	NA	NA	splice_acce	ENSG0000(	transcript	ENST0000C
NA	NA	NA	NA	NA	splice_acce	ENSG0000(	transcript	ENST0000C
NA	NA	NA	NA	NA	splice_acce	ENSG0000(	transcript	ENST0000C
NA	NA	NA	NA	NA	start_lost	ENSG0000(	transcript	ENST0000C
NA	NA	NA	NA	NA	frameshift_	ENSG0000(	transcript	ENST0000C
NA	NA	NA	NA	NA	stop_gaine	ENSG0000(	transcript	ENST0000C
NA	NA	NA	NA	NA	splice_don	ENSG0000(	transcript	ENST0000C
NA	NA	NA	NA	NA	splice_don	ENSG0000(	transcript	ENST0000C
NA	NA	NA	NA	NA	splice_don	ENSG0000(	transcript	ENST0000C
NA	NA	NA	NA	NA	splice_acce	ENSG0000(	transcript	ENST0000C
NA	NA	NA	NA	NA	splice_acce	ENSG0000(	transcript	ENST0000C
NA	Benign/Like	Parkinson_	NA	NA	frameshift_	ENSG0000(	transcript	ENST0000C
NA	Benign/Like	Parkinson_	NA	NA	frameshift_	ENSG0000(	transcript	ENST0000C
NA	Likely_beni	not_specifi	0.17396	NA	frameshift_	ENSG0000(	transcript	ENST0000C
NA	Likely_beni	not_specifi	0.17396	NA	frameshift_	ENSG0000(	transcript	ENST0000C
NA	NA	NA	NA	NA	structural_	ENSG0000(	interaction	1SNT:A_17
NA	NA	NA	NA	NA	structural_	ENSG0000(	interaction	1VCU:A_23
NA	NA	NA	NA	NA	frameshift_	ENSG0000(	transcript	ENST0000C
NA	NA	NA	NA	NA	splice_acce	ENSG0000(	transcript	ENST0000C
NA	NA	NA	NA	NA	frameshift_	ENSG0000(	transcript	ENST0000C
NA	NA	NA	NA	NA	splice_don	ENSG0000(	transcript	ENST0000C
NA	NA	NA	NA	NA	frameshift_	ENSG0000(	transcript	ENST0000C
NA	NA	NA	NA	NA	splice_acce	ENSG0000(	transcript	ENST0000C
NA	NA	NA	NA	NA	splice_acce	ENSG0000(	transcript	ENST0000C
NA	NA	NA	NA	NA	splice_acce	ENSG0000(	transcript	ENST0000C
NA	NA	NA	NA	NA	splice_acce	ENSG0000(	transcript	ENST0000C
NA	NA	NA	NA	NA	splice_don	ENSG0000(	transcript	ENST0000C
NA	NA	NA	NA	NA	splice_acce	ENSG0000(	transcript	ENST0000C
NA	NA	NA	NA	NA	frameshift_	ENSG0000(	transcript	ENST0000C
NA	NA	NA	NA	NA	frameshift_	ENSG0000(	transcript	ENST0000C
NA	NA	NA	NA	NA	structural_	ENSG0000(	interaction	4AD7:D_2(

NA	NA	NA	NA	NA	splice_acce	ENSG0000(	transcript	ENST0000C
NA	NA	NA	NA	NA	frameshift_	ENSG0000(	transcript	ENST0000C
NA	NA	NA	NA	NA	frameshift_	ENSG0000(	transcript	ENST0000C
NA	Benign/Like	Primary_hy	0.10850	0.15316	structural_	ENSG0000(	interaction	1H0C:A_78
NA	NA	NA	NA	NA	splice_don	ENSG0000(	transcript	ENST0000C
NA	NA	NA	NA	NA	start_lost	ENSG0000(	transcript	ENST0000C
NA	NA	NA	NA	NA	structural_	ENSG0000(	interaction	3DLS:E_109
NA	NA	NA	NA	NA	structural_	ENSG0000(	interaction	3DLS:A_99
NA	NA	NA	NA	NA	frameshift_	ENSG0000(	transcript	ENST0000C
NA	NA	NA	NA	NA	start_lost	ENSG0000(	transcript	ENST0000C
NA	NA	NA	NA	NA	splice_acce	ENSG0000(	transcript	ENST0000C
NA	NA	NA	NA	NA	splice_acce	ENSG0000(	transcript	ENST0000C
NA	NA	NA	NA	NA	frameshift_	ENSG0000(	transcript	ENST0000C
NA	NA	NA	NA	NA	frameshift_	ENSG0000(	transcript	ENST0000C
NA	NA	NA	NA	NA	frameshift_	ENSG0000(	transcript	ENST0000C
NA	NA	NA	NA	NA	frameshift_	ENSG0000(	transcript	ENST0000C
NA	NA	NA	NA	NA	frameshift_	ENSG0000(	transcript	ENST0000C
NA	NA	NA	NA	NA	frameshift_	ENSG0000(	transcript	ENST0000C
NA	NA	NA	NA	NA	frameshift_	ENSG0000(	transcript	ENST0000C
NA	NA	NA	NA	NA	stop_gaine	ENSG0000(	transcript	ENST0000C
NA	NA	NA	NA	NA	splice_acce	ENSG0000(	transcript	ENST0000C
NA	NA	NA	NA	NA	splice_acce	ENSG0000(	transcript	ENST0000C
NA	NA	NA	NA	NA	splice_acce	ENSG0000(	transcript	ENST0000C
NA	NA	NA	NA	NA	splice_acce	ENSG0000(	transcript	ENST0000C
NA	NA	NA	NA	NA	splice_acce	ENSG0000(	transcript	ENST0000C
NA	NA	NA	NA	NA	splice_don	ENSG0000(	transcript	ENST0000C
NA	Benign	not_provid	NA	NA	structural_	ENSG0000(	interaction	3MQ4:A_1
NA	NA	NA	NA	NA	frameshift_	ENSG0000(	transcript	ENST0000C
NA	NA	NA	NA	NA	splice_acce	ENSG0000(	transcript	ENST0000C
NA	NA	NA	NA	NA	splice_don	ENSG0000(	transcript	ENST0000C
NA	NA	NA	NA	NA	splice_acce	ENSG0000(	transcript	ENST0000C
NA	NA	NA	NA	NA	splice_acce	ENSG0000(	transcript	ENST0000C
NA	NA	NA	NA	NA	splice_acce	ENSG0000(	transcript	ENST0000C
NA	NA	NA	NA	NA	splice_acce	ENSG0000(	transcript	ENST0000C
NA	NA	NA	NA	NA	splice_acce	ENSG0000(	transcript	ENST0000C
NA	NA	NA	NA	NA	frameshift_	ENSG0000(	transcript	ENST0000C
NA	NA	NA	NA	NA	splice_don	ENSG0000(	transcript	ENST0000C
NA	NA	NA	NA	NA	stop_gaine	ENSG0000(	transcript	ENST0000C
NA	NA	NA	NA	NA	frameshift_	ENSG0000(	transcript	ENST0000C
NA	NA	NA	NA	NA	splice_don	ENSG0000(	transcript	ENST0000C
NA	Likely_beni	Obesity|Fa	0.13399	0.12660	structural_	ENSG0000(	interaction	3T03:A_35
NA	NA	NA	NA	NA	frameshift_	ENSG0000(	transcript	ENST0000C
NA	NA	NA	NA	NA	splice_acce	ENSG0000(	transcript	ENST0000C
NA	NA	NA	NA	NA	stop_gaine	ENSG0000(	transcript	ENST0000C
NA	NA	NA	NA	NA	splice_acce	ENSG0000(	transcript	ENST0000C
NA	NA	NA	NA	NA	splice_don	ENSG0000(	transcript	ENST0000C
NA	NA	NA	NA	NA	splice_don	ENSG0000(	transcript	ENST0000C

NA	NA	NA	NA	NA	splice_acce	ENSG0000(	transcript	ENST0000C
NA	NA	NA	NA	NA	splice_acce	ENSG0000(	transcript	ENST0000C
NA	NA	NA	NA	NA	splice_acce	ENSG0000(	transcript	ENST0000C
NA	NA	NA	NA	NA	stop_gaine	ENSG0000(	transcript	ENST0000C
NA	NA	NA	NA	NA	splice_acce	ENSG0000(	transcript	ENST0000C
NA	NA	NA	NA	NA	splice_acce	ENSG0000(	transcript	ENST0000C
NA	NA	NA	NA	NA	structural_	ENSG0000(	interaction	2OEX:A_42
NA	NA	NA	NA	NA	frameshift_	ENSG0000(	transcript	ENST0000C
NA	NA	NA	NA	NA	frameshift_	ENSG0000(	transcript	ENST0000C
NA	NA	NA	NA	NA	structural_	ENSG0000(	interaction	4BC2:A_25
NA	NA	NA	NA	NA	structural_	ENSG0000(	interaction	4BC2:A_17
NA	NA	NA	NA	NA	structural_	ENSG0000(	interaction	4BC2:A_10
NA	NA	NA	NA	NA	structural_	ENSG0000(	interaction	4BC2:A_10
NA	NA	NA	NA	NA	splice_acce	ENSG0000(	transcript	ENST0000C
NA	NA	NA	NA	NA	splice_regi	ENSG0000(	transcript	ENST0000C
NA	NA	NA	NA	NA	splice_acce	ENSG0000(	transcript	ENST0000C
NA	NA	NA	NA	NA	structural_	ENSG0000(	interaction	2HE9:A_63
NA	NA	NA	NA	NA	frameshift_	ENSG0000(	transcript	ENST0000C
NA	NA	NA	NA	NA	stop_gaine	ENSG0000(	transcript	ENST0000C
NA	NA	NA	NA	NA	splice_acce	ENSG0000(	transcript	ENST0000C
Blood_prot	Benign	West_nile_	NA	NA	frameshift_	ENSG0000(	transcript	ENST0000C
NA	NA	NA	NA	NA	stop_gaine	ENSG0000(	transcript	ENST0000C
NA	NA	NA	NA	NA	splice_don	ENSG0000(	transcript	ENST0000C
NA	NA	NA	NA	NA	start_lost	ENSG0000(	transcript	ENST0000C
NA	NA	NA	NA	NA	splice_acce	ENSG0000(	transcript	ENST0000C
NA	NA	NA	NA	NA	frameshift_	ENSG0000(	transcript	ENST0000C
NA	NA	NA	NA	NA	frameshift_	ENSG0000(	transcript	ENST0000C
NA	NA	NA	NA	NA	structural_	ENSG0000(	interaction	2Y6E:A_31
NA	Benign	not_provid	NA	NA	structural_	ENSG0000(	interaction	2Y6E:A_84
NA	NA	NA	NA	NA	frameshift_	ENSG0000(	transcript	ENST0000C
NA	NA	NA	NA	NA	stop_gaine	ENSG0000(	transcript	ENST0000C
NA	NA	NA	NA	NA	splice_acce	ENSG0000(	transcript	ENST0000C
NA	NA	NA	NA	NA	frameshift_	ENSG0000(	transcript	ENST0000C
NA	NA	NA	NA	NA	frameshift_	ENSG0000(	transcript	ENST0000C
NA	NA	NA	NA	NA	frameshift_	ENSG0000(	transcript	ENST0000C
NA	NA	NA	NA	NA	frameshift_	ENSG0000(	transcript	ENST0000C
NA	NA	NA	NA	NA	frameshift_	ENSG0000(	transcript	ENST0000C
NA	NA	NA	NA	NA	frameshift_	ENSG0000(	transcript	ENST0000C
NA	NA	NA	NA	NA	splice_acce	ENSG0000(	transcript	ENST0000C
NA	NA	NA	NA	NA	splice_acce	ENSG0000(	transcript	ENST0000C
NA	NA	NA	NA	NA	splice_acce	ENSG0000(	transcript	ENST0000C
NA	NA	NA	NA	NA	splice_acce	ENSG0000(	transcript	ENST0000C
NA	NA	NA	NA	NA	stop_gaine	ENSG0000(	transcript	ENST0000C
NA	NA	NA	NA	NA	start_lost&	ENSG0000(	transcript	ENST0000C
NA	NA	NA	NA	NA	start_lost&	ENSG0000(	transcript	ENST0000C
NA	NA	NA	NA	NA	start_lost&	ENSG0000(	transcript	ENST0000C
NA	NA	NA	NA	NA	start_lost&	ENSG0000(	transcript	ENST0000C

NA	NA	NA	NA	NA	start_lost	ENSG0000(	transcript	ENST0000C
NA	NA	NA	NA	NA	start_lost	ENSG0000(	transcript	ENST0000C
NA	NA	NA	NA	NA	structural_	ENSG0000(	interaction	2W0I:A_24
NA	NA	NA	NA	NA	splice_acce	ENSG0000(	transcript	ENST0000C
NA	NA	NA	NA	NA	frameshift_	ENSG0000(	transcript	ENST0000C
NA	NA	NA	NA	NA	frameshift_	ENSG0000(	transcript	ENST0000C
NA	Benign	not_provid	NA	NA	splice_acce	ENSG0000(	transcript	ENST0000C
NA	NA	NA	NA	NA	splice_acce	ENSG0000(	transcript	ENST0000C
NA	NA	NA	NA	NA	frameshift_	ENSG0000(	transcript	ENST0000C
NA	Benign	not_specifi	0.12815	0.08187	structural_	ENSG0000(	interaction	1YRK:A_6-
NA	Likely_beni	not_provid	NA	NA	structural_	ENSG0000(	interaction	3MOS:A_8
NA	Likely_beni	not_provid	NA	NA	structural_	ENSG0000(	interaction	3MOS:A_1
NA	NA	NA	NA	NA	splice_don	ENSG0000(	transcript	ENST0000C
NA	NA	NA	NA	NA	stop_gaine	ENSG0000(	transcript	ENST0000C
NA	NA	NA	NA	NA	structural_	ENSG0000(	interaction	4FO0:A_10
NA	NA	NA	NA	NA	structural_	ENSG0000(	interaction	4FO0:A_56
NA	NA	NA	NA	NA	structural_	ENSG0000(	interaction	2ELB:A_12-
NA	NA	NA	NA	NA	splice_acce	ENSG0000(	transcript	ENST0000C
NA	NA	NA	NA	NA	splice_acce	ENSG0000(	transcript	ENST0000C
NA	NA	NA	NA	NA	splice_acce	ENSG0000(	transcript	ENST0000C
NA	NA	NA	NA	NA	structural_	ENSG0000(	interaction	3JXH:C_24
NA	NA	NA	NA	NA	structural_	ENSG0000(	interaction	2H4V:A_87
NA	NA	NA	NA	NA	splice_don	ENSG0000(	transcript	ENST0000C
NA	NA	NA	NA	NA	splice_acce	ENSG0000(	transcript	ENST0000C
NA	NA	NA	NA	NA	splice_acce	ENSG0000(	transcript	ENST0000C
NA	NA	NA	NA	NA	splice_acce	ENSG0000(	transcript	ENST0000C
NA	NA	NA	NA	NA	splice_acce	ENSG0000(	transcript	ENST0000C
NA	NA	NA	NA	NA	splice_acce	ENSG0000(	transcript	ENST0000C
NA	NA	NA	NA	NA	splice_acce	ENSG0000(	transcript	ENST0000C
NA	NA	NA	NA	NA	splice_acce	ENSG0000(	transcript	ENST0000C
NA	NA	NA	NA	NA	bidirection	ENSG0000(	gene_varia	ENSG0000C
NA	NA	NA	NA	NA	frameshift_	ENSG0000(	transcript	ENST0000C
NA	NA	NA	NA	NA	splice_don	ENSG0000(	transcript	ENST0000C
NA	NA	NA	NA	NA	splice_don	ENSG0000(	transcript	ENST0000C
NA	NA	NA	NA	NA	splice_don	ENSG0000(	transcript	ENST0000C
NA	NA	NA	NA	NA	stop_gaine	ENSG0000(	transcript	ENST0000C
NA	NA	NA	NA	NA	splice_don	ENSG0000(	transcript	ENST0000C
NA	Benign	not_provid	NA	NA	structural_	ENSG0000(	interaction	2V9Q:A_2C
NA	Benign/Like	Glycogen_s	0.04853	0.07608	structural_	ENSG0000(	interaction	4BZY:A_50
NA	Benign	Glycogen_s	0.37460	0.32149	structural_	ENSG0000(	interaction	4BZY:B_19
NA	NA	NA	NA	NA	splice_don	ENSG0000(	transcript	ENST0000C
NA	NA	NA	NA	NA	splice_don	ENSG0000(	transcript	ENST0000C
NA	NA	NA	NA	NA	frameshift_	ENSG0000(	transcript	ENST0000C
NA	NA	NA	NA	NA	stop_gaine	ENSG0000(	transcript	ENST0000C
NA	NA	NA	NA	NA	structural_	ENSG0000(	interaction	4P5Z:A_80
NA	NA	NA	NA	NA	structural_	ENSG0000(	interaction	2XDV:A_16
NA	NA	NA	NA	NA	structural_	ENSG0000(	interaction	2XDV:A_68

NA	NA	NA	NA	NA	stop_gaine	ENSG0000(	transcript	ENST0000C
NA	NA	NA	NA	NA	frameshift_	ENSG0000(	transcript	ENST0000C
NA	NA	NA	NA	NA	stop_lost&	ENSG0000(	transcript	ENST0000C
NA	NA	NA	NA	NA	frameshift_	ENSG0000(	transcript	ENST0000C
NA	NA	NA	NA	NA	stop_gaine	ENSG0000(	transcript	ENST0000C
NA	NA	NA	NA	NA	frameshift_	ENSG0000(	transcript	ENST0000C
NA	NA	NA	NA	NA	frameshift_	ENSG0000(	transcript	ENST0000C
NA	NA	NA	NA	NA	stop_gaine	ENSG0000(	transcript	ENST0000C
NA	Benign	Hereditary_	0.70652	0.75399	structural_	ENSG0000(	interaction	2AEX:A_33
NA	NA	NA	NA	NA	splice_don	ENSG0000(	transcript	ENST0000C
NA	NA	NA	NA	NA	frameshift_	ENSG0000(	transcript	ENST0000C
NA	NA	NA	NA	NA	start_lost	ENSG0000(	transcript	ENST0000C
NA	NA	NA	NA	NA	structural_	ENSG0000(	interaction	3ZNI:A_35
NA	NA	NA	NA	NA	splice_acce	ENSG0000(	transcript	ENST0000C
NA	NA	NA	NA	NA	splice_acce	ENSG0000(	transcript	ENST0000C
NA	NA	NA	NA	NA	splice_acce	ENSG0000(	transcript	ENST0000C
NA	NA	NA	NA	NA	splice_acce	ENSG0000(	transcript	ENST0000C
NA	NA	NA	NA	NA	splice_don	ENSG0000(	transcript	ENST0000C
NA	NA	NA	NA	NA	frameshift_	ENSG0000(	transcript	ENST0000C
NA	NA	NA	NA	NA	splice_don	ENSG0000(	transcript	ENST0000C
NA	NA	NA	NA	NA	stop_gaine	ENSG0000(	transcript	ENST0000C
NA	NA	NA	NA	NA	start_lost	ENSG0000(	transcript	ENST0000C
NA	NA	NA	NA	NA	splice_acce	ENSG0000(	transcript	ENST0000C
NA	NA	NA	NA	NA	frameshift_	ENSG0000(	transcript	ENST0000C
NA	NA	NA	NA	NA	frameshift_	ENSG0000(	transcript	ENST0000C
NA	NA	NA	NA	NA	splice_don	ENSG0000(	transcript	ENST0000C
NA	NA	NA	NA	NA	splice_don	ENSG0000(	transcript	ENST0000C
NA	NA	NA	NA	NA	stop_gaine	ENSG0000(	transcript	ENST0000C
NA	NA	NA	NA	NA	gene_fusio	ENSG0000(	gene_varia	ENSG00000
NA	NA	NA	NA	NA	gene_fusio	ENSG0000(	gene_varia	ENSG00000
NA	NA	NA	NA	NA	gene_fusio	ENSG0000(	gene_varia	ENSG00000
NA	NA	NA	NA	NA	gene_fusio	ENSG0000(	gene_varia	ENSG00000
NA	NA	NA	NA	NA	gene_fusio	ENSG0000(	gene_varia	ENSG00000
NA	NA	NA	NA	NA	splice_don	ENSG0000(	transcript	ENST0000C
NA	NA	NA	NA	NA	frameshift_	ENSG0000(	transcript	ENST0000C
NA	NA	NA	NA	NA	splice_acce	ENSG0000(	transcript	ENST0000C
Multiple_s	NA	NA	NA	NA	stop_lost	ENSG0000(	transcript	ENST0000C
NA	Likely_beni	Alkaptonur	NA	0.00998	structural_	ENSG0000(	interaction	1EY2:A_31
NA	NA	NA	NA	NA	splice_don	ENSG0000(	transcript	ENST0000C
NA	NA	NA	NA	NA	frameshift_	ENSG0000(	transcript	ENST0000C
NA	NA	NA	NA	NA	stop_gaine	ENSG0000(	transcript	ENST0000C
NA	Benign	not_provid	NA	NA	protein_pr	ENSG0000(	interaction	3TJ3:B_395
NA	NA	NA	NA	NA	structural_	ENSG0000(	interaction	3Q6Z:A_88
NA	NA	NA	NA	NA	splice_don	ENSG0000(	transcript	ENST0000C
NA	NA	NA	NA	NA	start_lost	ENSG0000(	transcript	ENST0000C
NA	Benign	Orotic_acic	0.18874	0.18610	structural_	ENSG0000(	interaction	2V30:A_44
NA	Likely_beni	Orotic_acic	0.02159	0.02756	structural_	ENSG0000(	interaction	2JGY:A_41

NA	NA	NA	NA	NA	splice_acce	ENSG0000(	transcript	ENST0000C
NA	NA	NA	NA	NA	splice_acce	ENSG0000(	transcript	ENST0000C
NA	NA	NA	NA	NA	frameshift_	ENSG0000(	transcript	ENST0000C
NA	NA	NA	NA	NA	splice_don	ENSG0000(	transcript	ENST0000C
NA	NA	NA	NA	NA	stop_gaine	ENSG0000(	transcript	ENST0000C
NA	NA	NA	NA	NA	splice_don	ENSG0000(	transcript	ENST0000C
NA	NA	NA	NA	NA	structural_	ENSG0000(	interaction	3PE6:A_15
NA	Benign/Like	Charcot-Ma	0.04341	0.01937	protein_pr	ENSG0000(	interaction	1YHN:A_73
NA	NA	NA	NA	NA	splice_don	ENSG0000(	transcript	ENST0000C
NA	NA	NA	NA	NA	stop_gaine	ENSG0000(	transcript	ENST0000C
NA	NA	NA	NA	NA	splice_don	ENSG0000(	transcript	ENST0000C
NA	NA	NA	NA	NA	frameshift_	ENSG0000(	transcript	ENST0000C
NA	NA	NA	NA	NA	splice_acce	ENSG0000(	transcript	ENST0000C
NA	NA	NA	NA	NA	stop_gaine	ENSG0000(	transcript	ENST0000C
NA	NA	NA	NA	NA	frameshift_	ENSG0000(	transcript	ENST0000C
NA	NA	NA	NA	NA	splice_don	ENSG0000(	transcript	ENST0000C
NA	NA	NA	NA	NA	splice_acce	ENSG0000(	transcript	ENST0000C
NA	NA	NA	NA	NA	splice_don	ENSG0000(	transcript	ENST0000C
NA	NA	NA	NA	NA	structural_	ENSG0000(	interaction	3UEN:A_59
NA	NA	NA	NA	NA	structural_	ENSG0000(	interaction	2E9S:A_27-
NA	NA	NA	NA	NA	splice_acce	ENSG0000(	transcript	ENST0000C
NA	Benign	not_provid	NA	NA	splice_acce	ENSG0000(	transcript	ENST0000C
NA	NA	NA	NA	NA	frameshift_	ENSG0000(	transcript	ENST0000C
NA	NA	NA	NA	NA	splice_don	ENSG0000(	transcript	ENST0000C
NA	NA	NA	NA	NA	structural_	ENSG0000(	interaction	1NUP:A_50
NA	NA	NA	NA	NA	splice_don	ENSG0000(	transcript	ENST0000C
NA	NA	NA	NA	NA	structural_	ENSG0000(	interaction	2V24:A_77
NA	NA	NA	NA	NA	splice_don	ENSG0000(	transcript	ENST0000C
NA	NA	NA	NA	NA	splice_acce	ENSG0000(	transcript	ENST0000C
NA	NA	NA	NA	NA	splice_acce	ENSG0000(	transcript	ENST0000C
NA	NA	NA	NA	NA	splice_acce	ENSG0000(	transcript	ENST0000C
NA	NA	NA	NA	NA	stop_gaine	ENSG0000(	transcript	ENST0000C
NA	Pathogenic	Glycogen_s	0.00091	0.00040	structural_	ENSG0000(	interaction	3Q4S:A_10
NA	NA	NA	NA	NA	stop_gaine	ENSG0000(	transcript	ENST0000C
NA	NA	NA	NA	NA	frameshift_	ENSG0000(	transcript	ENST0000C
NA	NA	NA	NA	NA	splice_don	ENSG0000(	transcript	ENST0000C
NA	NA	NA	NA	NA	splice_don	ENSG0000(	transcript	ENST0000C
NA	NA	NA	NA	NA	splice_acce	ENSG0000(	transcript	ENST0000C
NA	NA	NA	NA	NA	stop_gaine	ENSG0000(	transcript	ENST0000C
NA	NA	NA	NA	NA	splice_don	ENSG0000(	transcript	ENST0000C
NA	NA	NA	NA	NA	splice_don	ENSG0000(	transcript	ENST0000C
Blood_met	Benign	not_provid	NA	NA	structural_	ENSG0000(	interaction	2VXO:A_45
NA	NA	NA	NA	NA	stop_gaine	ENSG0000(	transcript	ENST0000C
NA	NA	NA	NA	NA	frameshift_	ENSG0000(	transcript	ENST0000C
NA	NA	NA	NA	NA	splice_don	ENSG0000(	transcript	ENST0000C
NA	Benign	not_provid	NA	NA	structural_	ENSG0000(	interaction	2BO9:B_48
NA	NA	NA	NA	NA	frameshift_	ENSG0000(	transcript	ENST0000C

NA	NA	NA	NA	NA	splice_don	ENSG0000(	transcript	ENST0000C
NA	Benign	Encephalo	0.09846	0.11502	structural_	ENSG0000(	interaction	3F02:A_19
NA	NA	NA	NA	NA	splice_don	ENSG0000(	transcript	ENST0000C
NA	NA	NA	NA	NA	splice_acce	ENSG0000(	transcript	ENST0000C
NA	NA	NA	NA	NA	structural_	ENSG0000(	interaction	1D0G:A_12
NA	NA	NA	NA	NA	structural_	ENSG0000(	interaction	1D4V:B_14
NA	NA	NA	NA	NA	splice_acce	ENSG0000(	transcript	ENST0000C
NA	NA	NA	NA	NA	splice_acce	ENSG0000(	transcript	ENST0000C
NA	NA	NA	NA	NA	frameshift_	ENSG0000(	transcript	ENST0000C
NA	NA	NA	NA	NA	frameshift_	ENSG0000(	transcript	ENST0000C
NA	NA	NA	NA	NA	frameshift_	ENSG0000(	transcript	ENST0000C
NA	NA	NA	NA	NA	frameshift_	ENSG0000(	transcript	ENST0000C
NA	NA	NA	NA	NA	frameshift_	ENSG0000(	transcript	ENST0000C
NA	NA	NA	NA	NA	splice_acce	ENSG0000(	transcript	ENST0000C
NA	NA	NA	NA	NA	splice_acce	ENSG0000(	transcript	ENST0000C
NA	NA	NA	NA	NA	splice_acce	ENSG0000(	transcript	ENST0000C
NA	NA	NA	NA	NA	splice_acce	ENSG0000(	transcript	ENST0000C
NA	NA	NA	NA	NA	splice_acce	ENSG0000(	transcript	ENST0000C
NA	NA	NA	NA	NA	splice_acce	ENSG0000(	transcript	ENST0000C
NA	NA	NA	NA	NA	splice_acce	ENSG0000(	transcript	ENST0000C
NA	NA	NA	NA	NA	splice_acce	ENSG0000(	transcript	ENST0000C
NA	NA	NA	NA	NA	splice_acce	ENSG0000(	transcript	ENST0000C
NA	Benign	not_specifi	0.06488	0.08786	structural_	ENSG0000(	interaction	4JPS:A_334
NA	Benign	not_specifi	NA	NA	frameshift_	ENSG0000(	transcript	ENST0000C
NA	NA	NA	NA	NA	frameshift_	ENSG0000(	transcript	ENST0000C
NA	Benign/Lik	Primary_cil	0.00808	0.00539	frameshift_	ENSG0000(	transcript	ENST0000C
NA	NA	NA	NA	NA	splice_acce	ENSG0000(	transcript	ENST0000C
NA	NA	NA	NA	NA	splice_acce	ENSG0000(	transcript	ENST0000C
NA	NA	NA	NA	NA	splice_acce	ENSG0000(	transcript	ENST0000C
NA	NA	NA	NA	NA	splice_acce	ENSG0000(	transcript	ENST0000C
NA	NA	NA	NA	NA	splice_acce	ENSG0000(	transcript	ENST0000C
NA	NA	NA	NA	NA	structural_	ENSG0000(	interaction	4AKM:A_2
NA	NA	NA	NA	NA	splice_don	ENSG0000(	transcript	ENST0000C
NA	NA	NA	NA	NA	splice_acce	ENSG0000(	transcript	ENST0000C
NA	NA	NA	NA	NA	splice_acce	ENSG0000(	transcript	ENST0000C
NA	NA	NA	NA	NA	splice_acce	ENSG0000(	transcript	ENST0000C
NA	NA	NA	NA	NA	splice_don	ENSG0000(	transcript	ENST0000C
NA	NA	NA	NA	NA	splice_acce	ENSG0000(	transcript	ENST0000C
NA	NA	NA	NA	NA	frameshift_	ENSG0000(	transcript	ENST0000C
Body_mass	NA	NA	NA	NA	structural_	ENSG0000(	interaction	2PXX:A_12
NA	NA	NA	NA	NA	structural_	ENSG0000(	interaction	2PXX:A_17
NA	NA	NA	NA	NA	structural_	ENSG0000(	interaction	3P1I:A_55-
NA	NA	NA	NA	NA	structural_	ENSG0000(	interaction	3P1I:A_80-
NA	NA	NA	NA	NA	structural_	ENSG0000(	interaction	3P1I:A_10
NA	Benign	not_specifi	NA	0.02416	stop_gaine	ENSG0000(	transcript	ENST0000C
NA	NA	NA	NA	NA	splice_don	ENSG0000(	transcript	ENST0000C
NA	NA	NA	NA	NA	splice_don	ENSG0000(	transcript	ENST0000C

NA	NA	NA	NA	NA	structural_	ENSG0000	interaction	4KKD:A_52
NA	NA	NA	NA	NA	structural_	ENSG0000	interaction	4KKD:B_57
NA	Benign/Lik	Primary_hy	NA	NA	frameshift_	ENSG0000	transcript	ENST0000
NA	NA	NA	NA	NA	splice_don	ENSG0000	transcript	ENST0000
NA	NA	NA	NA	NA	splice_don	ENSG0000	transcript	ENST0000
NA	Likely_beni	not_specifi	0.02171	0.00958	stop_gaine	ENSG0000	transcript	ENST0000
NA	NA	NA	NA	NA	splice_don	ENSG0000	transcript	ENST0000
Height	NA	NA	NA	NA	stop_gaine	ENSG0000	transcript	ENST0000
NA	NA	NA	NA	NA	stop_gaine	ENSG0000	transcript	ENST0000
NA	NA	NA	NA	NA	splice_acce	ENSG0000	transcript	ENST0000
NA	NA	NA	NA	NA	splice_don	ENSG0000	transcript	ENST0000
NA	NA	NA	NA	NA	stop_gaine	ENSG0000	transcript	ENST0000
NA	NA	NA	NA	NA	structural_	ENSG0000	interaction	3RL7:D_27
NA	NA	NA	NA	NA	stop_gaine	ENSG0000	transcript	ENST0000
NA	NA	NA	NA	NA	splice_acce	ENSG0000	transcript	ENST0000
NA	NA	NA	NA	NA	splice_don	ENSG0000	transcript	ENST0000
NA	NA	NA	NA	NA	frameshift_	ENSG0000	transcript	ENST0000
NA	NA	NA	NA	NA	frameshift_	ENSG0000	transcript	ENST0000
Waist-to-hi	NA	NA	NA	NA	splice_acce	ENSG0000	transcript	ENST0000
NA	Benign	Mucopolys	0.91367	0.89197	structural_	ENSG0000	interaction	3W81:A_3
NA	Benign	Mucopolys	0.18198	0.21985	structural_	ENSG0000	interaction	3W81:A_8
NA	Benign/Lik	Mucopolys	0.08571	0.02995	structural_	ENSG0000	interaction	3W81:A_2
NA	Benign	Mucopolys	0.25599	0.21865	structural_	ENSG0000	interaction	4OBR:B_31
Total_body	Benign	Mucopolys	0.20557	0.21765	structural_	ENSG0000	interaction	3W81:A_3
NA	Conflicting_	Mucopolys	0.00083	0.00020	structural_	ENSG0000	interaction	3W81:A_4
NA	Benign	Mucopolys	0.34143	0.21865	structural_	ENSG0000	interaction	3W81:A_4
NA	NA	NA	NA	NA	splice_don	ENSG0000	transcript	ENST0000
NA	NA	NA	NA	NA	splice_don	ENSG0000	transcript	ENST0000
NA	Benign	not_specifi	0.07860	0.05212	stop_gaine	ENSG0000	transcript	ENST0000
NA	NA	NA	NA	NA	stop_gaine	ENSG0000	transcript	ENST0000
NA	NA	NA	NA	NA	splice_don	ENSG0000	transcript	ENST0000
NA	NA	NA	NA	NA	splice_acce	ENSG0000	transcript	ENST0000
NA	NA	NA	NA	NA	frameshift_	ENSG0000	transcript	ENST0000
NA	NA	NA	NA	NA	frameshift_	ENSG0000	transcript	ENST0000
NA	NA	NA	NA	NA	frameshift_	ENSG0000	transcript	ENST0000
NA	NA	NA	NA	NA	frameshift_	ENSG0000	transcript	ENST0000
NA	NA	NA	NA	NA	frameshift_	ENSG0000	transcript	ENST0000
NA	NA	NA	NA	NA	frameshift_	ENSG0000	transcript	ENST0000
NA	NA	NA	NA	NA	frameshift_	ENSG0000	transcript	ENST0000
NA	NA	NA	NA	NA	frameshift_	ENSG0000	transcript	ENST0000
NA	NA	NA	NA	NA	frameshift_	ENSG0000	transcript	ENST0000
NA	NA	NA	NA	NA	frameshift_	ENSG0000	transcript	ENST0000
NA	NA	NA	NA	NA	frameshift_	ENSG0000	transcript	ENST0000
NA	NA	NA	NA	NA	frameshift_	ENSG0000	transcript	ENST0000
NA	NA	NA	NA	NA	frameshift_	ENSG0000	transcript	ENST0000
NA	NA	NA	NA	NA	structural_	ENSG0000	interaction	4XVK:A_44
NA	NA	NA	NA	NA	stop_gaine	ENSG0000	transcript	ENST0000

NA	NA	NA	NA	NA	frameshift_	ENSG0000(	transcript	ENST0000C
NA	NA	NA	NA	NA	frameshift_	ENSG0000(	transcript	ENST0000C
NA	NA	NA	NA	NA	frameshift_	ENSG0000(	transcript	ENST0000C
NA	NA	NA	NA	NA	frameshift_	ENSG0000(	transcript	ENST0000C
NA	Benign	not_specifi	0.01386	NA	frameshift_	ENSG0000(	transcript	ENST0000C
NA	Benign	not_specifi	0.01386	NA	frameshift_	ENSG0000(	transcript	ENST0000C
NA	Benign	not_specifi	0.01390	NA	frameshift_	ENSG0000(	transcript	ENST0000C
NA	Benign	not_specifi	0.01390	NA	frameshift_	ENSG0000(	transcript	ENST0000C
NA	Benign	not_specifi	0.01390	NA	frameshift_	ENSG0000(	transcript	ENST0000C
NA	NA	NA	NA	NA	frameshift_	ENSG0000(	transcript	ENST0000C
NA	NA	NA	NA	NA	frameshift_	ENSG0000(	transcript	ENST0000C
NA	NA	NA	NA	NA	frameshift_	ENSG0000(	transcript	ENST0000C
NA	NA	NA	NA	NA	frameshift_	ENSG0000(	transcript	ENST0000C
NA	NA	NA	NA	NA	frameshift_	ENSG0000(	transcript	ENST0000C
NA	NA	NA	NA	NA	structural_	ENSG0000(	interaction	1YBW:A_4:
NA	NA	NA	NA	NA	splice_acce	ENSG0000(	transcript	ENST0000C
NA	NA	NA	NA	NA	splice_acce	ENSG0000(	transcript	ENST0000C
NA	NA	NA	NA	NA	splice_acce	ENSG0000(	transcript	ENST0000C
NA	NA	NA	NA	NA	splice_acce	ENSG0000(	transcript	ENST0000C
NA	NA	NA	NA	NA	splice_don	ENSG0000(	transcript	ENST0000C
NA	NA	NA	NA	NA	splice_don	ENSG0000(	transcript	ENST0000C
NA	NA	NA	NA	NA	frameshift_	ENSG0000(	transcript	ENST0000C
NA	NA	NA	NA	NA	splice_acce	ENSG0000(	transcript	ENST0000C
NA	NA	NA	NA	NA	splice_don	ENSG0000(	transcript	ENST0000C
NA	NA	NA	NA	NA	splice_don	ENSG0000(	transcript	ENST0000C
NA	NA	NA	NA	NA	stop_gaine	ENSG0000(	transcript	ENST0000C
NA	NA	NA	NA	NA	splice_acce	ENSG0000(	transcript	ENST0000C
NA	NA	NA	NA	NA	splice_acce	ENSG0000(	transcript	ENST0000C
NA	NA	NA	NA	NA	splice_don	ENSG0000(	transcript	ENST0000C
NA	NA	NA	NA	NA	frameshift_	ENSG0000(	transcript	ENST0000C
Conduct_d	Benign/Like	Joubert_sy	0.08823	0.11881	stop_gaine	ENSG0000(	transcript	ENST0000C
NA	NA	NA	NA	NA	stop_lost	ENSG0000(	transcript	ENST0000C
NA	NA	NA	NA	NA	stop_lost	ENSG0000(	transcript	ENST0000C
NA	NA	NA	NA	NA	structural_	ENSG0000(	interaction	1YH3:A_10
NA	NA	NA	NA	NA	splice_acce	ENSG0000(	transcript	ENST0000C
NA	NA	NA	NA	NA	splice_acce	ENSG0000(	transcript	ENST0000C
NA	NA	NA	NA	NA	splice_acce	ENSG0000(	transcript	ENST0000C
NA	NA	NA	NA	NA	stop_lost	ENSG0000(	transcript	ENST0000C
NA	NA	NA	NA	NA	stop_lost	ENSG0000(	transcript	ENST0000C
NA	NA	NA	NA	NA	splice_don	ENSG0000(	transcript	ENST0000C
NA	NA	NA	NA	NA	structural_	ENSG0000(	interaction	2V9S:A_40
NA	NA	NA	NA	NA	splice_acce	ENSG0000(	transcript	ENST0000C
Serum_me	NA	NA	NA	NA	stop_lost	ENSG0000(	transcript	ENST0000C
NA	NA	NA	NA	NA	splice_acce	ENSG0000(	transcript	ENST0000C
NA	NA	NA	NA	NA	splice_don	ENSG0000(	transcript	ENST0000C
NA	NA	NA	NA	NA	splice_acce	ENSG0000(	transcript	ENST0000C
NA	NA	NA	NA	NA	splice_acce	ENSG0000(	transcript	ENST0000C

NA	NA	NA	NA	NA	splice_acce	ENSG0000(	transcript	ENST0000C
NA	NA	NA	NA	NA	splice_acce	ENSG0000(	transcript	ENST0000C
NA	NA	NA	NA	NA	frameshift_	ENSG0000(	transcript	ENST0000C
NA	NA	NA	NA	NA	splice_acce	ENSG0000(	transcript	ENST0000C
NA	NA	NA	NA	NA	structural_	ENSG0000(	interaction	2J67:A_72(
NA	NA	NA	NA	NA	structural_	ENSG0000(	interaction	2J67:A_691
NA	NA	NA	NA	NA	structural_	ENSG0000(	interaction	4OM7:A_6
NA	NA	NA	NA	NA	structural_	ENSG0000(	interaction	3ITK:F_224
NA	NA	NA	NA	NA	structural_	ENSG0000(	interaction	3PTZ:F_13
NA	NA	NA	NA	NA	splice_acce	ENSG0000(	transcript	ENST0000C
NA	NA	NA	NA	NA	splice_don	ENSG0000(	transcript	ENST0000C
NA	NA	NA	NA	NA	frameshift_	ENSG0000(	transcript	ENST0000C
NA	NA	NA	NA	NA	splice_acce	ENSG0000(	transcript	ENST0000C
NA	NA	NA	NA	NA	frameshift_	ENSG0000(	transcript	ENST0000C
NA	NA	NA	NA	NA	splice_don	ENSG0000(	transcript	ENST0000C
NA	NA	NA	NA	NA	splice_acce	ENSG0000(	transcript	ENST0000C
NA	NA	NA	NA	NA	splice_acce	ENSG0000(	transcript	ENST0000C
NA	NA	NA	NA	NA	splice_don	ENSG0000(	transcript	ENST0000C
NA	NA	NA	NA	NA	frameshift_	ENSG0000(	transcript	ENST0000C
NA	Benign	Gastrointe	0.00277	0.00040	structural_	ENSG0000(	interaction	4K9E:C_43
NA	Benign/Like	Partial_albi	0.02110	0.01138	structural_	ENSG0000(	interaction	1PKG:A_67
Blood_prot	not_provid	not_specifi	0.10893	0.15256	structural_	ENSG0000(	interaction	2X1W:L_23
NA	NA	NA	NA	NA	structural_	ENSG0000(	interaction	2H31:A_15
NA	NA	NA	NA	NA	splice_acce	ENSG0000(	transcript	ENST0000C
NA	NA	NA	NA	NA	structural_	ENSG0000(	interaction	4ET7:A_59
NA	NA	NA	NA	NA	start_lost	ENSG0000(	transcript	ENST0000C
NA	NA	NA	NA	NA	splice_don	ENSG0000(	transcript	ENST0000C
NA	Likely_beni	not_specifi	0.03285	0.01458	stop_gaine	ENSG0000(	transcript	ENST0000C
NA	NA	NA	NA	NA	splice_acce	ENSG0000(	transcript	ENST0000C
NA	NA	NA	NA	NA	frameshift_	ENSG0000(	transcript	ENST0000C
NA	NA	NA	NA	NA	structural_	ENSG0000(	interaction	2NO6:B_22
NA	NA	NA	NA	NA	start_lost	ENSG0000(	transcript	ENST0000C
NA	NA	NA	NA	NA	stop_lost	ENSG0000(	transcript	ENST0000C
Monokine_	NA	NA	NA	NA	splice_acce	ENSG0000(	transcript	ENST0000C
NA	NA	NA	NA	NA	splice_acce	ENSG0000(	transcript	ENST0000C
NA	NA	NA	NA	NA	splice_don	ENSG0000(	transcript	ENST0000C
NA	NA	NA	NA	NA	splice_don	ENSG0000(	transcript	ENST0000C
NA	NA	NA	NA	NA	frameshift_	ENSG0000(	transcript	ENST0000C
NA	NA	NA	NA	NA	frameshift_	ENSG0000(	transcript	ENST0000C
NA	NA	NA	NA	NA	frameshift_	ENSG0000(	transcript	ENST0000C
NA	NA	NA	NA	NA	frameshift_	ENSG0000(	transcript	ENST0000C
NA	NA	NA	NA	NA	frameshift_	ENSG0000(	transcript	ENST0000C
NA	NA	NA	NA	NA	frameshift_	ENSG0000(	transcript	ENST0000C
NA	Benign	Fraser_syn	0.27001	0.28774	stop_lost	ENSG0000(	transcript	ENST0000C
NA	NA	NA	NA	NA	splice_don	ENSG0000(	transcript	ENST0000C
NA	NA	NA	NA	NA	splice_acce	ENSG0000(	transcript	ENST0000C
NA	Benign/Like	Coenzyme_	NA	0.00998	stop_gaine	ENSG0000(	transcript	ENST0000C

NA	NA	NA	NA	NA	frameshift_	ENSG0000(	transcript	ENST0000C
NA	NA	NA	NA	NA	splice_don	ENSG0000(	transcript	ENST0000C
NA	NA	NA	NA	NA	splice_don	ENSG0000(	transcript	ENST0000C
NA	NA	NA	NA	NA	stop_gaine	ENSG0000(	transcript	ENST0000C
NA	NA	NA	NA	NA	frameshift_	ENSG0000(	transcript	ENST0000C
NA	NA	NA	NA	NA	frameshift_	ENSG0000(	transcript	ENST0000C
NA	NA	NA	NA	NA	splice_acce	ENSG0000(	transcript	ENST0000C
NA	NA	NA	NA	NA	splice_acce	ENSG0000(	transcript	ENST0000C
NA	NA	NA	NA	NA	splice_acce	ENSG0000(	transcript	ENST0000C
NA	NA	NA	NA	NA	splice_acce	ENSG0000(	transcript	ENST0000C
NA	NA	NA	NA	NA	splice_acce	ENSG0000(	transcript	ENST0000C
NA	Benign	not_provid	NA	NA	structural_	ENSG0000(	interaction	1IYH:A_12-
NA	NA	NA	NA	NA	structural_	ENSG0000(	interaction	2UZC:A_22
NA	NA	NA	NA	NA	splice_don	ENSG0000(	transcript	ENST0000C
NA	NA	NA	NA	NA	splice_don	ENSG0000(	transcript	ENST0000C
NA	NA	NA	NA	NA	splice_don	ENSG0000(	transcript	ENST0000C
NA	NA	NA	NA	NA	structural_	ENSG0000(	interaction	3COS:A_35
NA	NA	NA	NA	NA	structural_	ENSG0000(	interaction	3COS:A_45
NA	NA	NA	NA	NA	stop_gaine	ENSG0000(	transcript	ENST0000C
NA	NA	NA	NA	NA	splice_acce	ENSG0000(	transcript	ENST0000C
NA	NA	NA	NA	NA	splice_acce	ENSG0000(	transcript	ENST0000C
NA	NA	NA	NA	NA	splice_don	ENSG0000(	transcript	ENST0000C
NA	risk_factor	Parkinson_	0.01516	0.00719	stop_gaine	ENSG0000(	transcript	ENST0000C
NA	NA	NA	NA	NA	frameshift_	ENSG0000(	transcript	ENST0000C
NA	Benign	Abetalipop	0.33191	0.25000	start_lost	ENSG0000(	transcript	ENST0000C
NA	NA	NA	NA	NA	frameshift_	ENSG0000(	transcript	ENST0000C
NA	NA	NA	NA	NA	splice_acce	ENSG0000(	transcript	ENST0000C
NA	NA	NA	NA	NA	frameshift_	ENSG0000(	transcript	ENST0000C
NA	NA	NA	NA	NA	splice_don	ENSG0000(	transcript	ENST0000C
NA	NA	NA	NA	NA	start_lost	ENSG0000(	transcript	ENST0000C
NA	NA	NA	NA	NA	structural_	ENSG0000(	interaction	1NFI:B_317
NA	NA	NA	NA	NA	frameshift_	ENSG0000(	transcript	ENST0000C
NA	NA	NA	NA	NA	frameshift_	ENSG0000(	transcript	ENST0000C
NA	NA	NA	NA	NA	frameshift_	ENSG0000(	transcript	ENST0000C
NA	NA	NA	NA	NA	structural_	ENSG0000(	interaction	1T5C:A_22
NA	NA	NA	NA	NA	structural_	ENSG0000(	interaction	1T5C:A_81-
NA	NA	NA	NA	NA	splice_don	ENSG0000(	transcript	ENST0000C
NA	NA	NA	NA	NA	splice_don	ENSG0000(	transcript	ENST0000C
NA	NA	NA	NA	NA	start_lost	ENSG0000(	transcript	ENST0000C
NA	NA	NA	NA	NA	structural_	ENSG0000(	interaction	1X6V:A_42
NA	NA	NA	NA	NA	splice_acce	ENSG0000(	transcript	ENST0000C
NA	NA	NA	NA	NA	splice_acce	ENSG0000(	transcript	ENST0000C
NA	Benign	Renal_Hyp	NA	NA	frameshift_	ENSG0000(	transcript	ENST0000C
Systolic_bl	NA	NA	NA	NA	stop_gaine	ENSG0000(	transcript	ENST0000C
NA	NA	NA	NA	NA	stop_gaine	ENSG0000(	transcript	ENST0000C
NA	NA	NA	NA	NA	frameshift_	ENSG0000(	transcript	ENST0000C
Body_mass	NA	NA	NA	NA	splice_acce	ENSG0000(	transcript	ENST0000C

NA	NA	NA	NA	NA	splice_don	ENSG0000(	transcript	ENST0000C
NA	NA	NA	NA	NA	structural_	ENSG0000(	interaction	3LFV:B_18
NA	Benign	not_provid	NA	NA	structural_	ENSG0000(	interaction	2QYF:A_10
NA	NA	NA	NA	NA	structural_	ENSG0000(	interaction	2QYF:A_35
NA	NA	NA	NA	NA	splice_acce	ENSG0000(	transcript	ENST0000C
NA	NA	NA	NA	NA	splice_acce	ENSG0000(	transcript	ENST0000C
NA	NA	NA	NA	NA	splice_acce	ENSG0000(	transcript	ENST0000C
NA	NA	NA	NA	NA	splice_acce	ENSG0000(	transcript	ENST0000C
NA	NA	NA	NA	NA	protein_pr	ENSG0000(	interaction	1GY3:C_15
NA	NA	NA	NA	NA	splice_acce	ENSG0000(	transcript	ENST0000C
NA	NA	NA	NA	NA	splice_acce	ENSG0000(	transcript	ENST0000C
NA	NA	NA	NA	NA	splice_acce	ENSG0000(	transcript	ENST0000C
NA	NA	NA	NA	NA	splice_acce	ENSG0000(	transcript	ENST0000C
NA	NA	NA	NA	NA	frameshift_	ENSG0000(	transcript	ENST0000C
NA	NA	NA	NA	NA	splice_acce	ENSG0000(	transcript	ENST0000C
NA	NA	NA	NA	NA	splice_acce	ENSG0000(	transcript	ENST0000C
NA	NA	NA	NA	NA	splice_don	ENSG0000(	transcript	ENST0000C
NA	NA	NA	NA	NA	splice_don	ENSG0000(	transcript	ENST0000C
NA	NA	NA	NA	NA	frameshift_	ENSG0000(	transcript	ENST0000C
NA	NA	NA	NA	NA	frameshift_	ENSG0000(	transcript	ENST0000C
NA	NA	NA	NA	NA	frameshift_	ENSG0000(	transcript	ENST0000C
NA	NA	NA	NA	NA	frameshift_	ENSG0000(	transcript	ENST0000C
NA	NA	NA	NA	NA	frameshift_	ENSG0000(	transcript	ENST0000C
NA	NA	NA	NA	NA	frameshift_	ENSG0000(	transcript	ENST0000C
NA	NA	NA	NA	NA	frameshift_	ENSG0000(	transcript	ENST0000C
NA	NA	NA	NA	NA	frameshift_	ENSG0000(	transcript	ENST0000C
NA	NA	NA	NA	NA	splice_don	ENSG0000(	transcript	ENST0000C
NA	NA	NA	NA	NA	splice_don	ENSG0000(	transcript	ENST0000C
NA	NA	NA	NA	NA	splice_acce	ENSG0000(	transcript	ENST0000C
NA	NA	NA	NA	NA	splice_don	ENSG0000(	transcript	ENST0000C
NA	NA	NA	NA	NA	splice_acce	ENSG0000(	transcript	ENST0000C
NA	NA	NA	NA	NA	frameshift_	ENSG0000(	transcript	ENST0000C
NA	NA	NA	NA	NA	structural_	ENSG0000(	interaction	1JHJ:A_64-
NA	NA	NA	NA	NA	splice_acce	ENSG0000(	transcript	ENST0000C
NA	NA	NA	NA	NA	splice_acce	ENSG0000(	transcript	ENST0000C
NA	NA	NA	NA	NA	splice_don	ENSG0000(	transcript	ENST0000C
NA	NA	NA	NA	NA	frameshift_	ENSG0000(	transcript	ENST0000C
NA	NA	NA	NA	NA	splice_don	ENSG0000(	transcript	ENST0000C
NA	NA	NA	NA	NA	frameshift_	ENSG0000(	transcript	ENST0000C
NA	NA	NA	NA	NA	stop_lost&	ENSG0000(	transcript	ENST0000C
NA	NA	NA	NA	NA	frameshift_	ENSG0000(	transcript	ENST0000C
NA	NA	NA	NA	NA	frameshift_	ENSG0000(	transcript	ENST0000C
NA	NA	NA	NA	NA	structural_	ENSG0000(	interaction	4YVD:A_29
NA	NA	NA	NA	NA	splice_don	ENSG0000(	transcript	ENST0000C
NA	NA	NA	NA	NA	splice_don	ENSG0000(	transcript	ENST0000C
NA	NA	NA	NA	NA	splice_don	ENSG0000(	transcript	ENST0000C
NA	NA	NA	NA	NA	splice_acce	ENSG0000(	transcript	ENST0000C

NA	NA	NA	NA	NA	splice_acce	ENSG0000(	transcript	ENST0000C
NA	NA	NA	NA	NA	splice_don	ENSG0000(	transcript	ENST0000C
NA	NA	NA	NA	NA	splice_acce	ENSG0000(	transcript	ENST0000C
NA	NA	NA	NA	NA	splice_don	ENSG0000(	transcript	ENST0000C
NA	NA	NA	NA	NA	stop_gaine	ENSG0000(	transcript	ENST0000C
NA	NA	NA	NA	NA	splice_acce	ENSG0000(	transcript	ENST0000C
NA	NA	NA	NA	NA	frameshift_	ENSG0000(	transcript	ENST0000C
NA	Conflicting_	Short_rib-p	NA	0.00080	structural_	ENSG0000(	interaction	4APC:A_10
NA	NA	NA	NA	NA	splice_acce	ENSG0000(	transcript	ENST0000C
NA	NA	NA	NA	NA	frameshift_	ENSG0000(	transcript	ENST0000C
NA	NA	NA	NA	NA	splice_acce	ENSG0000(	transcript	ENST0000C
NA	NA	NA	NA	NA	splice_acce	ENSG0000(	transcript	ENST0000C
NA	NA	NA	NA	NA	structural_	ENSG0000(	interaction	2W4L:A_1C
NA	NA	NA	NA	NA	splice_acce	ENSG0000(	transcript	ENST0000C
NA	NA	NA	NA	NA	splice_acce	ENSG0000(	transcript	ENST0000C
NA	NA	NA	NA	NA	splice_acce	ENSG0000(	transcript	ENST0000C
NA	Likely_beni	not_provid	NA	NA	frameshift_	ENSG0000(	transcript	ENST0000C
Medicatior	Benign/Like	Human_im	NA	0.23183	structural_	ENSG0000(	interaction	1ZIW:A_41
NA	Benign	not_specifi	0.30072	0.27217	structural_	ENSG0000(	interaction	1ZIW:A_43
NA	NA	NA	NA	NA	stop_gaine	ENSG0000(	transcript	ENST0000C
NA	Benign/Like	Hereditary_	0.05457	0.05831	structural_	ENSG0000(	interaction	3SOR:A_50
NA	NA	NA	NA	NA	splice_don	ENSG0000(	transcript	ENST0000C
NA	NA	NA	NA	NA	splice_don	ENSG0000(	transcript	ENST0000C
NA	NA	NA	NA	NA	splice_acce	ENSG0000(	transcript	ENST0000C
NA	NA	NA	NA	NA	frameshift_	ENSG0000(	transcript	ENST0000C
NA	NA	NA	NA	NA	frameshift_	ENSG0000(	transcript	ENST0000C
NA	NA	NA	NA	NA	structural_	ENSG0000(	interaction	2ZN8:A_11
NA	NA	NA	NA	NA	splice_don	ENSG0000(	transcript	ENST0000C
NA	NA	NA	NA	NA	frameshift_	ENSG0000(	transcript	ENST0000C
NA	NA	NA	NA	NA	frameshift_	ENSG0000(	transcript	ENST0000C
NA	NA	NA	NA	NA	frameshift_	ENSG0000(	transcript	ENST0000C
NA	NA	NA	NA	NA	start_lost	ENSG0000(	transcript	ENST0000C
NA	NA	NA	NA	NA	stop_gaine	ENSG0000(	transcript	ENST0000C
NA	NA	NA	NA	NA	frameshift_	ENSG0000(	transcript	ENST0000C
NA	NA	NA	NA	NA	frameshift_	ENSG0000(	transcript	ENST0000C
NA	NA	NA	NA	NA	frameshift_	ENSG0000(	transcript	ENST0000C
NA	NA	NA	NA	NA	stop_gaine	ENSG0000(	transcript	ENST0000C
NA	NA	NA	NA	NA	stop_gaine	ENSG0000(	transcript	ENST0000C
NA	NA	NA	NA	NA	splice_don	ENSG0000(	transcript	ENST0000C
NA	Benign	MITOCHON	NA	NA	stop_lost	ENSG0000(	transcript	ENST0000C
NA	NA	NA	NA	NA	stop_gaine	ENSG0000(	transcript	ENST0000C
NA	NA	NA	NA	NA	splice_acce	ENSG0000(	transcript	ENST0000C
NA	NA	NA	NA	NA	splice_acce	ENSG0000(	transcript	ENST0000C
NA	NA	NA	NA	NA	splice_acce	ENSG0000(	transcript	ENST0000C
NA	Benign	Gastrointe:	0.15090	0.21945	structural_	ENSG0000(	interaction	2QTL:A_56
NA	NA	NA	NA	NA	splice_don	ENSG0000(	transcript	ENST0000C
NA	NA	NA	NA	NA	stop_gaine	ENSG0000(	transcript	ENST0000C

NA	NA	NA	NA	NA	splice_don	ENSG0000(	transcript	ENST0000C
NA	Benign	not_provid	NA	NA	structural_	ENSG0000(	interaction	3AU5:B_17
NA	NA	NA	NA	NA	splice_acce	ENSG0000(	transcript	ENST0000C
NA	NA	NA	NA	NA	structural_	ENSG0000(	interaction	4P3N:A_55
NA	NA	NA	NA	NA	stop_gaine	ENSG0000(	transcript	ENST0000C
NA	NA	NA	NA	NA	stop_lost	ENSG0000(	transcript	ENST0000C
NA	NA	NA	NA	NA	splice_don	ENSG0000(	transcript	ENST0000C
NA	NA	NA	NA	NA	gene_fusio	ENSG0000(	gene_varia	ENSG0000C
NA	NA	NA	NA	NA	splice_acce	ENSG0000(	transcript	ENST0000C
NA	NA	NA	NA	NA	splice_acce	ENSG0000(	transcript	ENST0000C
NA	NA	NA	NA	NA	splice_acce	ENSG0000(	transcript	ENST0000C
NA	NA	NA	NA	NA	splice_acce	ENSG0000(	transcript	ENST0000C
NA	NA	NA	NA	NA	splice_acce	ENSG0000(	transcript	ENST0000C
NA	NA	NA	NA	NA	splice_acce	ENSG0000(	transcript	ENST0000C
NA	NA	NA	NA	NA	splice_acce	ENSG0000(	transcript	ENST0000C
NA	NA	NA	NA	NA	splice_don	ENSG0000(	transcript	ENST0000C
NA	Benign/Like	not_specifi	0.01073	0.00539	splice_don	ENSG0000(	transcript	ENST0000C
NA	NA	NA	NA	NA	bidirection	ENSG0000(	gene_varia	ENSG0000C
NA	NA	NA	NA	NA	splice_acce	ENSG0000(	transcript	ENST0000C
NA	NA	NA	NA	NA	splice_acce	ENSG0000(	transcript	ENST0000C
NA	NA	NA	NA	NA	splice_regi	ENSG0000(	transcript	ENST0000C
NA	NA	NA	NA	NA	frameshift_	ENSG0000(	transcript	ENST0000C
NA	NA	NA	NA	NA	stop_gaine	ENSG0000(	transcript	ENST0000C
NA	NA	NA	NA	NA	frameshift_	ENSG0000(	transcript	ENST0000C
NA	NA	NA	NA	NA	stop_gaine	ENSG0000(	transcript	ENST0000C
NA	Conflicting_	not_provid	0.00231	0.00120	splice_don	ENSG0000(	transcript	ENST0000C
NA	NA	NA	NA	NA	splice_acce	ENSG0000(	transcript	ENST0000C
NA	NA	NA	NA	NA	splice_acce	ENSG0000(	transcript	ENST0000C
NA	NA	NA	NA	NA	splice_acce	ENSG0000(	transcript	ENST0000C
NA	NA	NA	NA	NA	splice_acce	ENSG0000(	transcript	ENST0000C
NA	NA	NA	NA	NA	splice_acce	ENSG0000(	transcript	ENST0000C
NA	NA	NA	NA	NA	structural_	ENSG0000(	interaction	2P8U:A_19
NA	NA	NA	NA	NA	structural_	ENSG0000(	interaction	2P8U:A_23
NA	NA	NA	NA	NA	splice_don	ENSG0000(	transcript	ENST0000C
NA	NA	NA	NA	NA	splice_acce	ENSG0000(	transcript	ENST0000C
NA	NA	NA	NA	NA	splice_acce	ENSG0000(	transcript	ENST0000C
NA	Likely_beni	Combined_	0.03920	0.03634	structural_	ENSG0000(	interaction	4AP8:A_50
NA	NA	NA	NA	NA	splice_acce	ENSG0000(	transcript	ENST0000C
NA	NA	NA	NA	NA	stop_gaine	ENSG0000(	transcript	ENST0000C
NA	NA	NA	NA	NA	frameshift_	ENSG0000(	transcript	ENST0000C
NA	NA	NA	NA	NA	frameshift_	ENSG0000(	transcript	ENST0000C
NA	NA	NA	NA	NA	frameshift_	ENSG0000(	transcript	ENST0000C
NA	NA	NA	NA	NA	frameshift_	ENSG0000(	transcript	ENST0000C
NA	NA	NA	NA	NA	splice_don	ENSG0000(	transcript	ENST0000C
NA	NA	NA	NA	NA	structural_	ENSG0000(	interaction	4I5P:A_113
NA	NA	NA	NA	NA	splice_acce	ENSG0000(	transcript	ENST0000C
NA	NA	NA	NA	NA	splice_acce	ENSG0000(	transcript	ENST0000C

NA	NA	NA	NA	NA	splice_acce	ENSG0000(	transcript	ENST0000C
NA	NA	NA	NA	NA	splice_acce	ENSG0000(	transcript	ENST0000C
NA	NA	NA	NA	NA	splice_acce	ENSG0000(	transcript	ENST0000C
NA	NA	NA	NA	NA	splice_acce	ENSG0000(	transcript	ENST0000C
NA	NA	NA	NA	NA	bidirection	ENSG0000(	gene_varia	ENSG0000C
NA	NA	NA	NA	NA	bidirection	ENSG0000(	gene_varia	ENSG0000C
NA	NA	NA	NA	NA	bidirection	ENSG0000(	gene_varia	ENSG0000C
NA	NA	NA	NA	NA	bidirection	ENSG0000(	gene_varia	ENSG0000C
NA	NA	NA	NA	NA	bidirection	ENSG0000(	gene_varia	ENSG0000C
NA	NA	NA	NA	NA	bidirection	ENSG0000(	gene_varia	ENSG0000C
NA	NA	NA	NA	NA	structural_	ENSG0000(	interaction	3B2D:B_20
NA	Benign/Like	not_specifi	0.31294	0.41933	structural_	ENSG0000(	interaction	1PHT:A_11
NA	NA	NA	NA	NA	splice_acce	ENSG0000(	transcript	ENST0000C
NA	NA	NA	NA	NA	structural_	ENSG0000(	interaction	1QBK:B_32
NA	Benign	not_provid	NA	NA	stop_gaine	ENSG0000(	transcript	ENST0000C
NA	NA	NA	NA	NA	stop_gaine	ENSG0000(	transcript	ENST0000C
NA	NA	NA	NA	NA	frameshift_	ENSG0000(	transcript	ENST0000C
NA	NA	NA	NA	NA	frameshift_	ENSG0000(	transcript	ENST0000C
NA	NA	NA	NA	NA	frameshift_	ENSG0000(	transcript	ENST0000C
NA	NA	NA	NA	NA	structural_	ENSG0000(	interaction	4JRA:C_522
NA	NA	NA	NA	NA	stop_gaine	ENSG0000(	transcript	ENST0000C
NA	NA	NA	NA	NA	frameshift_	ENSG0000(	transcript	ENST0000C
NA	Benign/Like	Mucopolys	0.14636	0.10863	structural_	ENSG0000(	interaction	1FSU:A_35
NA	NA	NA	NA	NA	structural_	ENSG0000(	interaction	1LT7:A_246
NA	NA	NA	NA	NA	splice_don	ENSG0000(	transcript	ENST0000C
NA	NA	NA	NA	NA	splice_don	ENSG0000(	transcript	ENST0000C
NA	NA	NA	NA	NA	splice_acce	ENSG0000(	transcript	ENST0000C
NA	NA	NA	NA	NA	splice_acce	ENSG0000(	transcript	ENST0000C
NA	NA	NA	NA	NA	splice_acce	ENSG0000(	transcript	ENST0000C
NA	NA	NA	NA	NA	structural_	ENSG0000(	interaction	3B7K:B_59
NA	NA	NA	NA	NA	splice_acce	ENSG0000(	transcript	ENST0000C
NA	NA	NA	NA	NA	splice_acce	ENSG0000(	transcript	ENST0000C
NA	NA	NA	NA	NA	splice_acce	ENSG0000(	transcript	ENST0000C
NA	NA	NA	NA	NA	splice_acce	ENSG0000(	transcript	ENST0000C
NA	NA	NA	NA	NA	splice_acce	ENSG0000(	transcript	ENST0000C
NA	NA	NA	NA	NA	splice_acce	ENSG0000(	transcript	ENST0000C
NA	Benign	not_specifi	NA	NA	splice_acce	ENSG0000(	transcript	ENST0000C
NA	NA	NA	NA	NA	splice_don	ENSG0000(	transcript	ENST0000C
NA	NA	NA	NA	NA	frameshift_	ENSG0000(	transcript	ENST0000C
NA	NA	NA	NA	NA	stop_gaine	ENSG0000(	transcript	ENST0000C
NA	NA	NA	NA	NA	splice_acce	ENSG0000(	transcript	ENST0000C
Behcet's_d	NA	NA	NA	NA	structural_	ENSG0000(	interaction	3QNF:A_34
Ankylosing	NA	NA	NA	NA	structural_	ENSG0000(	interaction	2YD0:A_41
NA	NA	NA	NA	NA	structural_	ENSG0000(	interaction	4JBS:A_97-
NA	NA	NA	NA	NA	structural_	ENSG0000(	interaction	4JBS:A_214
NA	NA	NA	NA	NA	structural_	ENSG0000(	interaction	4JBS:A_200
NA	NA	NA	NA	NA	splice_acce	ENSG0000(	transcript	ENST0000C

NA	NA	NA	NA	NA	splice_acce	ENSG0000(	transcript	ENST0000C
NA	NA	NA	NA	NA	splice_don	ENSG0000(	transcript	ENST0000C
NA	NA	NA	NA	NA	gene_fusio	ENSG0000(	gene_varia	ENSG0000C
NA	NA	NA	NA	NA	gene_fusio	ENSG0000(	gene_varia	ENSG0000C
NA	NA	NA	NA	NA	gene_fusio	ENSG0000(	gene_varia	ENSG0000C
NA	NA	NA	NA	NA	gene_fusio	ENSG0000(	gene_varia	ENSG0000C
NA	NA	NA	NA	NA	splice_don	ENSG0000(	transcript	ENST0000C
NA	NA	NA	NA	NA	splice_acce	ENSG0000(	transcript	ENST0000C
NA	NA	NA	NA	NA	splice_acce	ENSG0000(	transcript	ENST0000C
NA	NA	NA	NA	NA	splice_acce	ENSG0000(	transcript	ENST0000C
NA	NA	NA	NA	NA	splice_acce	ENSG0000(	transcript	ENST0000C
NA	NA	NA	NA	NA	splice_acce	ENSG0000(	transcript	ENST0000C
NA	Benign	Hepatocell	0.57798	0.50998	structural_	ENSG0000(	interaction	3AU3:A_43
NA	Benign/Like	Hereditary_	0.00450	0.00120	structural_	ENSG0000(	interaction	3AU3:A_65
NA	Benign	Hereditary_	0.64857	0.66554	protein_pr	ENSG0000(	interaction	1T08:A_38
NA	NA	NA	NA	NA	splice_acce	ENSG0000(	transcript	ENST0000C
NA	NA	NA	NA	NA	frameshift_	ENSG0000(	transcript	ENST0000C
NA	NA	NA	NA	NA	splice_don	ENSG0000(	transcript	ENST0000C
NA	NA	NA	NA	NA	stop_gaine	ENSG0000(	transcript	ENST0000C
NA	NA	NA	NA	NA	frameshift_	ENSG0000(	transcript	ENST0000C
NA	NA	NA	NA	NA	splice_acce	ENSG0000(	transcript	ENST0000C
NA	NA	NA	NA	NA	splice_acce	ENSG0000(	transcript	ENST0000C
NA	Benign	Bifunctiona	0.44004	0.36262	structural_	ENSG0000(	interaction	1ZBQ:A_10
NA	NA	NA	NA	NA	frameshift_	ENSG0000(	transcript	ENST0000C
NA	NA	NA	NA	NA	structural_	ENSG0000(	interaction	2ESL:B_47-
NA	NA	NA	NA	NA	structural_	ENSG0000(	interaction	2ESL:B_86-
NA	NA	NA	NA	NA	frameshift_	ENSG0000(	transcript	ENST0000C
NA	NA	NA	NA	NA	bidirection	ENSG0000(	gene_varia	ENSG0000C
NA	NA	NA	NA	NA	bidirection	ENSG0000(	gene_varia	ENSG0000C
NA	NA	NA	NA	NA	splice_don	ENSG0000(	transcript	ENST0000C
NA	NA	NA	NA	NA	splice_acce	ENSG0000(	transcript	ENST0000C
NA	NA	NA	NA	NA	frameshift_	ENSG0000(	transcript	ENST0000C
NA	NA	NA	NA	NA	splice_acce	ENSG0000(	transcript	ENST0000C
NA	NA	NA	NA	NA	splice_acce	ENSG0000(	transcript	ENST0000C
NA	NA	NA	NA	NA	splice_acce	ENSG0000(	transcript	ENST0000C
NA	NA	NA	NA	NA	splice_acce	ENSG0000(	transcript	ENST0000C
NA	Benign	not_provid	NA	NA	splice_don	ENSG0000(	transcript	ENST0000C
NA	NA	NA	NA	NA	frameshift_	ENSG0000(	transcript	ENST0000C
NA	NA	NA	NA	NA	splice_don	ENSG0000(	transcript	ENST0000C
NA	NA	NA	NA	NA	frameshift_	ENSG0000(	transcript	ENST0000C
NA	NA	NA	NA	NA	frameshift_	ENSG0000(	transcript	ENST0000C
NA	NA	NA	NA	NA	gene_fusio	ENSG0000(	gene_varia	ENSG0000C
NA	NA	NA	NA	NA	gene_fusio	ENSG0000(	gene_varia	ENSG0000C
NA	NA	NA	NA	NA	gene_fusio	ENSG0000(	gene_varia	ENSG0000C
NA	NA	NA	NA	NA	splice_don	ENSG0000(	transcript	ENST0000C
NA	NA	NA	NA	NA	splice_don	ENSG0000(	transcript	ENST0000C
NA	NA	NA	NA	NA	structural_	ENSG0000(	interaction	1ZR3:B_30

NA	NA	NA	NA	NA	stop_gaine	ENSG0000(	transcript	ENST0000C
NA	NA	NA	NA	NA	splice_acce	ENSG0000(	transcript	ENST0000C
NA	Benign/Like	not_specifi	0.33120	0.42053	structural_	ENSG0000(	interaction	2VXP:A_54
NA	NA	NA	NA	NA	splice_acce	ENSG0000(	transcript	ENST0000C
NA	NA	NA	NA	NA	splice_acce	ENSG0000(	transcript	ENST0000C
NA	NA	NA	NA	NA	splice_don	ENSG0000(	transcript	ENST0000C
NA	Benign	Autosomal	0.21308	0.22644	structural_	ENSG0000(	interaction	4CH9:A_42
NA	NA	NA	NA	NA	splice_acce	ENSG0000(	transcript	ENST0000C
NA	NA	NA	NA	NA	splice_acce	ENSG0000(	transcript	ENST0000C
NA	NA	NA	NA	NA	splice_acce	ENSG0000(	transcript	ENST0000C
NA	NA	NA	NA	NA	splice_acce	ENSG0000(	transcript	ENST0000C
NA	NA	NA	NA	NA	splice_acce	ENSG0000(	transcript	ENST0000C
NA	NA	NA	NA	NA	frameshift_	ENSG0000(	transcript	ENST0000C
NA	NA	NA	NA	NA	frameshift_	ENSG0000(	transcript	ENST0000C
NA	NA	NA	NA	NA	frameshift_	ENSG0000(	transcript	ENST0000C
NA	NA	NA	NA	NA	structural_	ENSG0000(	interaction	4KBO:A_27
NA	NA	NA	NA	NA	stop_gaine	ENSG0000(	transcript	ENST0000C
NA	NA	NA	NA	NA	splice_acce	ENSG0000(	transcript	ENST0000C
NA	NA	NA	NA	NA	splice_acce	ENSG0000(	transcript	ENST0000C
NA	NA	NA	NA	NA	splice_acce	ENSG0000(	transcript	ENST0000C
NA	NA	NA	NA	NA	stop_gaine	ENSG0000(	transcript	ENST0000C
NA	NA	NA	NA	NA	frameshift_	ENSG0000(	transcript	ENST0000C
NA	Benign	not_provid	NA	NA	frameshift_	ENSG0000(	transcript	ENST0000C
NA	NA	NA	NA	NA	splice_don	ENSG0000(	transcript	ENST0000C
NA	NA	NA	NA	NA	frameshift_	ENSG0000(	transcript	ENST0000C
NA	NA	NA	NA	NA	frameshift_	ENSG0000(	transcript	ENST0000C
NA	NA	NA	NA	NA	frameshift_	ENSG0000(	transcript	ENST0000C
NA	NA	NA	NA	NA	splice_acce	ENSG0000(	transcript	ENST0000C
NA	NA	NA	NA	NA	frameshift_	ENSG0000(	transcript	ENST0000C
NA	NA	NA	NA	NA	frameshift_	ENSG0000(	transcript	ENST0000C
NA	NA	NA	NA	NA	frameshift_	ENSG0000(	transcript	ENST0000C
NA	NA	NA	NA	NA	splice_acce	ENSG0000(	transcript	ENST0000C
NA	NA	NA	NA	NA	frameshift_	ENSG0000(	transcript	ENST0000C
NA	NA	NA	NA	NA	frameshift_	ENSG0000(	transcript	ENST0000C
NA	NA	NA	NA	NA	frameshift_	ENSG0000(	transcript	ENST0000C
NA	NA	NA	NA	NA	stop_gaine	ENSG0000(	transcript	ENST0000C
NA	NA	NA	NA	NA	frameshift_	ENSG0000(	transcript	ENST0000C
NA	NA	NA	NA	NA	splice_acce	ENSG0000(	transcript	ENST0000C
NA	NA	NA	NA	NA	structural_	ENSG0000(	interaction	1NE7:A_23
NA	NA	NA	NA	NA	splice_don	ENSG0000(	transcript	ENST0000C
NA	NA	NA	NA	NA	splice_don	ENSG0000(	transcript	ENST0000C
NA	NA	NA	NA	NA	structural_	ENSG0000(	interaction	4BKN:A_17
NA	NA	NA	NA	NA	structural_	ENSG0000(	interaction	4BKN:A_14
NA	NA	NA	NA	NA	frameshift_	ENSG0000(	transcript	ENST0000C
NA	NA	NA	NA	NA	stop_lost	ENSG0000(	transcript	ENST0000C
NA	NA	NA	NA	NA	splice_acce	ENSG0000(	transcript	ENST0000C
NA	NA	NA	NA	NA	frameshift_	ENSG0000(	transcript	ENST0000C

NA	NA	NA	NA	NA	frameshift_	ENSG0000(	transcript	ENST0000C
NA	NA	NA	NA	NA	frameshift_	ENSG0000(	transcript	ENST0000C
NA	Benign/Like	Hereditary_	0.00917	0.00240	structural_	ENSG0000(	interaction	3DPK:A_76
NA	Benign/Like	Hereditary_	0.03271	0.01258	structural_	ENSG0000(	interaction	2I0V:A_759
NA	Benign	Hereditary_	0.11924	0.15336	structural_	ENSG0000(	interaction	4LIQ:E_325
NA	Benign	Hereditary_	0.04926	0.07768	structural_	ENSG0000(	interaction	4DKD:C_98
NA	NA	NA	NA	NA	splice_don	ENSG0000(	transcript	ENST0000C
NA	NA	NA	NA	NA	splice_don	ENSG0000(	transcript	ENST0000C
NA	NA	NA	NA	NA	splice_don	ENSG0000(	transcript	ENST0000C
NA	Benign	not_specifi	0.09833	0.11422	frameshift_	ENSG0000(	transcript	ENST0000C
NA	NA	NA	NA	NA	structural_	ENSG0000(	interaction	2D7I:A_464
NA	NA	NA	NA	NA	splice_acce	ENSG0000(	transcript	ENST0000C
NA	NA	NA	NA	NA	splice_acce	ENSG0000(	transcript	ENST0000C
NA	NA	NA	NA	NA	splice_don	ENSG0000(	transcript	ENST0000C
NA	NA	NA	NA	NA	splice_don	ENSG0000(	transcript	ENST0000C
NA	NA	NA	NA	NA	gene_fusio	ENSG0000(	gene_varia	ENSG0000C
NA	NA	NA	NA	NA	gene_fusio	ENSG0000(	gene_varia	ENSG0000C
NA	NA	NA	NA	NA	gene_fusio	ENSG0000(	gene_varia	ENSG0000C
NA	NA	NA	NA	NA	gene_fusio	ENSG0000(	gene_varia	ENSG0000C
NA	NA	NA	NA	NA	gene_fusio	ENSG0000(	gene_varia	ENSG0000C
NA	NA	NA	NA	NA	splice_acce	ENSG0000(	transcript	ENST0000C
NA	NA	NA	NA	NA	structural_	ENSG0000(	interaction	3MQI:A_31
NA	NA	NA	NA	NA	splice_acce	ENSG0000(	transcript	ENST0000C
Systemic_l	NA	NA	NA	NA	splice_don	ENSG0000(	transcript	ENST0000C
NA	NA	NA	NA	NA	splice_acce	ENSG0000(	transcript	ENST0000C
NA	NA	NA	NA	NA	splice_acce	ENSG0000(	transcript	ENST0000C
NA	NA	NA	NA	NA	splice_acce	ENSG0000(	transcript	ENST0000C
NA	NA	NA	NA	NA	splice_acce	ENSG0000(	transcript	ENST0000C
NA	NA	NA	NA	NA	splice_don	ENSG0000(	transcript	ENST0000C
NA	Benign	Immunode	0.01178	0.00559	structural_	ENSG0000(	interaction	2YIN:A_129
NA	NA	NA	NA	NA	splice_acce	ENSG0000(	transcript	ENST0000C
NA	NA	NA	NA	NA	structural_	ENSG0000(	interaction	2J7T:A_252
NA	NA	NA	NA	NA	frameshift_	ENSG0000(	transcript	ENST0000C
NA	NA	NA	NA	NA	frameshift_	ENSG0000(	transcript	ENST0000C
NA	NA	NA	NA	NA	splice_don	ENSG0000(	transcript	ENST0000C
NA	NA	NA	NA	NA	splice_acce	ENSG0000(	transcript	ENST0000C
NA	NA	NA	NA	NA	splice_acce	ENSG0000(	transcript	ENST0000C
NA	NA	NA	NA	NA	splice_acce	ENSG0000(	transcript	ENST0000C
NA	NA	NA	NA	NA	splice_acce	ENSG0000(	transcript	ENST0000C
NA	NA	NA	NA	NA	splice_acce	ENSG0000(	transcript	ENST0000C
NA	NA	NA	NA	NA	splice_acce	ENSG0000(	transcript	ENST0000C
NA	NA	NA	NA	NA	splice_acce	ENSG0000(	transcript	ENST0000C
NA	NA	NA	NA	NA	splice_acce	ENSG0000(	transcript	ENST0000C
NA	NA	NA	NA	NA	splice_acce	ENSG0000(	transcript	ENST0000C
NA	NA	NA	NA	NA	splice_acce	ENSG0000(	transcript	ENST0000C
Menopaus	NA	NA	NA	NA	stop_gaine	ENSG0000(	transcript	ENST0000C
NA	NA	NA	NA	NA	frameshift_	ENSG0000(	transcript	ENST0000C

NA	Benign	not_provid	NA	NA	frameshift_	ENSG0000(	transcript	ENST0000C
NA	NA	NA	NA	NA	splice_don	ENSG0000(	transcript	ENST0000C
NA	NA	NA	NA	NA	structural_	ENSG0000(	interaction	4IRP:A_234
NA	NA	NA	NA	NA	splice_don	ENSG0000(	transcript	ENST0000C
NA	NA	NA	NA	NA	splice_acce	ENSG0000(	transcript	ENST0000C
NA	NA	NA	NA	NA	splice_acce	ENSG0000(	transcript	ENST0000C
NA	NA	NA	NA	NA	splice_acce	ENSG0000(	transcript	ENST0000C
NA	NA	NA	NA	NA	splice_acce	ENSG0000(	transcript	ENST0000C
NA	NA	NA	NA	NA	splice_don	ENSG0000(	transcript	ENST0000C
NA	Benign/Lik	Paget_dise	0.00463	NA	structural_	ENSG0000(	interaction	4MJS:R_61
NA	Benign	not_specifi	0.03026	0.03415	structural_	ENSG0000(	interaction	4BSJ:A_391
NA	NA	NA	NA	NA	start_lost	ENSG0000(	transcript	ENST0000C
NA	NA	NA	NA	NA	frameshift_	ENSG0000(	transcript	ENST0000C
NA	NA	NA	NA	NA	splice_don	ENSG0000(	transcript	ENST0000C
NA	NA	NA	NA	NA	frameshift_	ENSG0000(	transcript	ENST0000C
NA	NA	NA	NA	NA	splice_don	ENSG0000(	transcript	ENST0000C
NA	NA	NA	NA	NA	splice_acce	ENSG0000(	transcript	ENST0000C
NA	NA	NA	NA	NA	splice_acce	ENSG0000(	transcript	ENST0000C
NA	NA	NA	NA	NA	splice_regi	ENSG0000(	transcript	ENST0000C
Preschool_	NA	NA	NA	NA	structural_	ENSG0000(	interaction	4ITI:A_28-A
NA	NA	NA	NA	NA	stop_gaine	ENSG0000(	transcript	ENST0000C
NA	NA	NA	NA	NA	splice_acce	ENSG0000(	transcript	ENST0000C
NA	Benign	Cardiomyo	0.19939	0.19968	frameshift_	ENSG0000(	transcript	ENST0000C
NA	Benign	Cardiomyo	0.28806	0.21166	structural_	ENSG0000(	interaction	1LM7:A_22
NA	Benign/Lik	Cardiomyo	0.04600	0.02396	structural_	ENSG0000(	interaction	1LM5:A_26
Blood_prot	Benign	not_provid	NA	NA	structural_	ENSG0000(	interaction	3UVT:A_40
NA	NA	NA	NA	NA	stop_gaine	ENSG0000(	transcript	ENST0000C
NA	NA	NA	NA	NA	splice_acce	ENSG0000(	transcript	ENST0000C
NA	NA	NA	NA	NA	splice_acce	ENSG0000(	transcript	ENST0000C
NA	NA	NA	NA	NA	splice_acce	ENSG0000(	transcript	ENST0000C
NA	NA	NA	NA	NA	frameshift_	ENSG0000(	transcript	ENST0000C
NA	NA	NA	NA	NA	splice_don	ENSG0000(	transcript	ENST0000C
NA	NA	NA	NA	NA	structural_	ENSG0000(	interaction	2B4Y:A_16
Thiopurine	Likely_beni	Thiopurine	0.03669	0.03914	structural_	ENSG0000(	interaction	2BZG:A_18
NA	NA	NA	NA	NA	splice_acce	ENSG0000(	transcript	ENST0000C
NA	NA	NA	NA	NA	splice_don	ENSG0000(	transcript	ENST0000C
NA	NA	NA	NA	NA	splice_acce	ENSG0000(	transcript	ENST0000C
NA	NA	NA	NA	NA	splice_don	ENSG0000(	transcript	ENST0000C
NA	NA	NA	NA	NA	splice_acce	ENSG0000(	transcript	ENST0000C
NA	NA	NA	NA	NA	splice_don	ENSG0000(	transcript	ENST0000C
NA	NA	NA	NA	NA	splice_acce	ENSG0000(	transcript	ENST0000C
NA	NA	NA	NA	NA	splice_acce	ENSG0000(	transcript	ENST0000C
NA	NA	NA	NA	NA	splice_acce	ENSG0000(	transcript	ENST0000C
NA	NA	NA	NA	NA	splice_acce	ENSG0000(	transcript	ENST0000C
NA	NA	NA	NA	NA	splice_acce	ENSG0000(	transcript	ENST0000C
NA	NA	NA	NA	NA	splice_acce	ENSG0000(	transcript	ENST0000C
NA	NA	NA	NA	NA	stop_lost	ENSG0000(	transcript	ENST0000C

NA	Benign	not_specifi	0.46084	0.35783	splice_don	ENSG0000(	transcript	ENST0000C
NA	NA	NA	NA	NA	stop_gaine	ENSG0000(	transcript	ENST0000C
NA	NA	NA	NA	NA	stop_gaine	ENSG0000(	transcript	ENST0000C
NA	NA	NA	NA	NA	splice_don	ENSG0000(	transcript	ENST0000C
NA	NA	NA	NA	NA	splice_don	ENSG0000(	transcript	ENST0000C
NA	NA	NA	NA	NA	stop_gaine	ENSG0000(	transcript	ENST0000C
NA	NA	NA	NA	NA	start_lost	ENSG0000(	transcript	ENST0000C
NA	NA	NA	NA	NA	frameshift_	ENSG0000(	transcript	ENST0000C
NA	NA	NA	NA	NA	stop_gaine	ENSG0000(	transcript	ENST0000C
NA	NA	NA	NA	NA	splice_don	ENSG0000(	transcript	ENST0000C
NA	NA	NA	NA	NA	frameshift_	ENSG0000(	transcript	ENST0000C
NA	NA	NA	NA	NA	frameshift_	ENSG0000(	transcript	ENST0000C
NA	NA	NA	NA	NA	frameshift_	ENSG0000(	transcript	ENST0000C
NA	NA	NA	NA	NA	frameshift_	ENSG0000(	transcript	ENST0000C
NA	NA	NA	NA	NA	conservati	ENSG0000(	transcript	ENST0000C
NA	NA	NA	NA	NA	frameshift_	ENSG0000(	transcript	ENST0000C
NA	NA	NA	NA	NA	frameshift_	ENSG0000(	transcript	ENST0000C
NA	NA	NA	NA	NA	frameshift_	ENSG0000(	transcript	ENST0000C
NA	NA	NA	NA	NA	frameshift_	ENSG0000(	transcript	ENST0000C
NA	NA	NA	NA	NA	structural_	ENSG0000(	interaction	4U2U:A_1C
NA	NA	NA	NA	NA	splice_acce	ENSG0000(	transcript	ENST0000C
NA	NA	NA	NA	NA	frameshift_	ENSG0000(	transcript	ENST0000C
NA	NA	NA	NA	NA	stop_gaine	ENSG0000(	transcript	ENST0000C
NA	NA	NA	NA	NA	splice_acce	ENSG0000(	transcript	ENST0000C
NA	NA	NA	NA	NA	stop_gaine	ENSG0000(	transcript	ENST0000C
NA	Benign/Lik	not_specifi	NA	0.00699	stop_gaine	ENSG0000(	transcript	ENST0000C
NA	NA	NA	NA	NA	splice_acce	ENSG0000(	transcript	ENST0000C
NA	NA	NA	NA	NA	splice_acce	ENSG0000(	transcript	ENST0000C
NA	NA	NA	NA	NA	splice_acce	ENSG0000(	transcript	ENST0000C
NA	NA	NA	NA	NA	splice_acce	ENSG0000(	transcript	ENST0000C
NA	NA	NA	NA	NA	splice_acce	ENSG0000(	transcript	ENST0000C
NA	NA	NA	NA	NA	splice_acce	ENSG0000(	transcript	ENST0000C
NA	NA	NA	NA	NA	splice_acce	ENSG0000(	transcript	ENST0000C
NA	NA	NA	NA	NA	splice_acce	ENSG0000(	transcript	ENST0000C
NA	NA	NA	NA	NA	splice_acce	ENSG0000(	transcript	ENST0000C
Adolescent	NA	NA	NA	NA	structural_	ENSG0000(	interaction	2X7K:A_81
Blood_prot	NA	NA	NA	NA	structural_	ENSG0000(	interaction	2X7K:A_36
NA	NA	NA	NA	NA	frameshift_	ENSG0000(	transcript	ENST0000C
NA	NA	NA	NA	NA	structural_	ENSG0000(	interaction	4AYC:A_41
NA	NA	NA	NA	NA	stop_gaine	ENSG0000(	transcript	ENST0000C
NA	NA	NA	NA	NA	splice_don	ENSG0000(	transcript	ENST0000C
NA	NA	NA	NA	NA	structural_	ENSG0000(	interaction	2NYT:C_58
NA	NA	NA	NA	NA	stop_gaine	ENSG0000(	transcript	ENST0000C
NA	NA	NA	NA	NA	splice_acce	ENSG0000(	transcript	ENST0000C
NA	NA	NA	NA	NA	structural_	ENSG0000(	interaction	1Q8M:D_2
NA	NA	NA	NA	NA	splice_don	ENSG0000(	transcript	ENST0000C
NA	NA	NA	NA	NA	frameshift_	ENSG0000(	transcript	ENST0000C

NA	NA	NA	NA	NA	frameshift_	ENSG0000(	transcript	ENST0000C
NA	NA	NA	NA	NA	stop_gaine	ENSG0000(	transcript	ENST0000C
NA	NA	NA	NA	NA	structural_	ENSG0000(	interaction	3NY2:D_12
NA	NA	NA	NA	NA	splice_acce	ENSG0000(	transcript	ENST0000C
NA	NA	NA	NA	NA	splice_acce	ENSG0000(	transcript	ENST0000C
NA	NA	NA	NA	NA	start_lost	ENSG0000(	transcript	ENST0000C
NA	NA	NA	NA	NA	structural_	ENSG0000(	interaction	4P5E:A_90-
NA	NA	NA	NA	NA	splice_acce	ENSG0000(	transcript	ENST0000C
NA	NA	NA	NA	NA	frameshift_	ENSG0000(	transcript	ENST0000C
NA	NA	NA	NA	NA	structural_	ENSG0000(	interaction	4LQY:A_11
NA	NA	NA	NA	NA	structural_	ENSG0000(	interaction	4LQY:A_26
Lipoprotein	not_provid	not_provid	0.30326	0.31749	structural_	ENSG0000(	interaction	3D59:B_92
NA	NA	NA	NA	NA	splice_acce	ENSG0000(	transcript	ENST0000C
NA	NA	NA	NA	NA	splice_acce	ENSG0000(	transcript	ENST0000C
NA	NA	NA	NA	NA	splice_acce	ENSG0000(	transcript	ENST0000C
NA	NA	NA	NA	NA	splice_acce	ENSG0000(	transcript	ENST0000C
NA	NA	NA	NA	NA	splice_acce	ENSG0000(	transcript	ENST0000C
NA	NA	NA	NA	NA	structural_	ENSG0000(	interaction	1PKW:B_1:
NA	Benign	not_provid	NA	NA	structural_	ENSG0000(	interaction	1TDI:A_73-
NA	NA	NA	NA	NA	stop_gaine	ENSG0000(	transcript	ENST0000C
NA	NA	NA	NA	NA	splice_acce	ENSG0000(	transcript	ENST0000C
NA	NA	NA	NA	NA	protein_pr	ENSG0000(	interaction	4BPU:A_18
NA	NA	NA	NA	NA	splice_acce	ENSG0000(	transcript	ENST0000C
NA	NA	NA	NA	NA	stop_gaine	ENSG0000(	transcript	ENST0000C
NA	NA	NA	NA	NA	frameshift_	ENSG0000(	transcript	ENST0000C
NA	NA	NA	NA	NA	stop_lost	ENSG0000(	transcript	ENST0000C
NA	NA	NA	NA	NA	splice_don	ENSG0000(	transcript	ENST0000C
NA	NA	NA	NA	NA	splice_don	ENSG0000(	transcript	ENST0000C
NA	NA	NA	NA	NA	splice_acce	ENSG0000(	transcript	ENST0000C
NA	NA	NA	NA	NA	stop_gaine	ENSG0000(	transcript	ENST0000C
NA	NA	NA	NA	NA	structural_	ENSG0000(	interaction	4KM5:A_4
NA	NA	NA	NA	NA	splice_don	ENSG0000(	transcript	ENST0000C
NA	NA	NA	NA	NA	splice_don	ENSG0000(	transcript	ENST0000C
NA	NA	NA	NA	NA	frameshift_	ENSG0000(	transcript	ENST0000C
NA	NA	NA	NA	NA	frameshift_	ENSG0000(	transcript	ENST0000C
NA	NA	NA	NA	NA	splice_don	ENSG0000(	transcript	ENST0000C
NA	NA	NA	NA	NA	structural_	ENSG0000(	interaction	4CNC:A_11
NA	NA	NA	NA	NA	structural_	ENSG0000(	interaction	2AW5:C_8(
NA	NA	NA	NA	NA	splice_don	ENSG0000(	transcript	ENST0000C
NA	NA	NA	NA	NA	structural_	ENSG0000(	interaction	4H2F:A_18
NA	NA	NA	NA	NA	splice_don	ENSG0000(	transcript	ENST0000C
NA	NA	NA	NA	NA	splice_acce	ENSG0000(	transcript	ENST0000C
NA	NA	NA	NA	NA	splice_acce	ENSG0000(	transcript	ENST0000C
NA	NA	NA	NA	NA	frameshift_	ENSG0000(	transcript	ENST0000C
NA	NA	NA	NA	NA	frameshift_	ENSG0000(	transcript	ENST0000C
NA	NA	NA	NA	NA	structural_	ENSG0000(	interaction	3OHU:A_8:
NA	NA	NA	NA	NA	splice_don	ENSG0000(	transcript	ENST0000C

NA	NA	NA	NA	NA	NA	frameshift_	ENSG0000(	transcript	ENST0000C
NA	NA	NA	NA	NA	NA	frameshift_	ENSG0000(	transcript	ENST0000C
NA	NA	NA	NA	NA	NA	frameshift_	ENSG0000(	transcript	ENST0000C
NA	NA	NA	NA	NA	NA	frameshift_	ENSG0000(	transcript	ENST0000C
NA	NA	NA	NA	NA	NA	frameshift_	ENSG0000(	transcript	ENST0000C
NA	NA	NA	NA	NA	NA	frameshift_	ENSG0000(	transcript	ENST0000C
NA	NA	NA	NA	NA	NA	splice_acce	ENSG0000(	transcript	ENST0000C
NA	NA	NA	NA	NA	NA	splice_regi	ENSG0000(	transcript	ENST0000C
NA	NA	NA	NA	NA	NA	splice_don	ENSG0000(	transcript	ENST0000C
NA	NA	NA	NA	NA	NA	splice_don	ENSG0000(	transcript	ENST0000C
NA	NA	NA	NA	NA	NA	frameshift_	ENSG0000(	transcript	ENST0000C
NA	NA	NA	NA	NA	NA	structural_	ENSG0000(	interaction	2VN8:A_17
NA	NA	NA	NA	NA	NA	splice_acce	ENSG0000(	transcript	ENST0000C
NA	NA	NA	NA	NA	NA	start_lost	ENSG0000(	transcript	ENST0000C
NA	NA	NA	NA	NA	NA	splice_acce	ENSG0000(	transcript	ENST0000C
NA	NA	NA	NA	NA	NA	splice_acce	ENSG0000(	transcript	ENST0000C
NA	NA	NA	NA	NA	NA	splice_acce	ENSG0000(	transcript	ENST0000C
NA	NA	NA	NA	NA	NA	splice_acce	ENSG0000(	transcript	ENST0000C
NA	NA	NA	NA	NA	NA	splice_acce	ENSG0000(	transcript	ENST0000C
NA	NA	NA	NA	NA	NA	frameshift_	ENSG0000(	transcript	ENST0000C
NA	NA	NA	NA	NA	NA	frameshift_	ENSG0000(	transcript	ENST0000C
NA	NA	NA	NA	NA	NA	frameshift_	ENSG0000(	transcript	ENST0000C
NA	NA	NA	NA	NA	NA	frameshift_	ENSG0000(	transcript	ENST0000C
NA	Uncertain_	Charcot-Ma	0.00507	NA		splice_acce	ENSG0000(	transcript	ENST0000C
NA	Uncertain_	Charcot-Ma	0.00507	NA		splice_acce	ENSG0000(	transcript	ENST0000C
NA	NA	NA	NA	NA	NA	protein_pr	ENSG0000(	interaction	4N78:D_21
NA	NA	NA	NA	NA	NA	splice_acce	ENSG0000(	transcript	ENST0000C
NA	NA	NA	NA	NA	NA	splice_acce	ENSG0000(	transcript	ENST0000C
NA	NA	NA	NA	NA	NA	splice_acce	ENSG0000(	transcript	ENST0000C
NA	NA	NA	NA	NA	NA	splice_acce	ENSG0000(	transcript	ENST0000C
NA	NA	NA	NA	NA	NA	stop_gaine	ENSG0000(	transcript	ENST0000C
NA	NA	NA	NA	NA	NA	splice_acce	ENSG0000(	transcript	ENST0000C
NA	NA	NA	NA	NA	NA	splice_don	ENSG0000(	transcript	ENST0000C
NA	Benign	Metaphyse	0.04402	0.02736		structural_	ENSG0000(	interaction	1GR3:A_60
NA	NA	NA	NA	NA	NA	splice_don	ENSG0000(	transcript	ENST0000C
NA	NA	NA	NA	NA	NA	splice_acce	ENSG0000(	transcript	ENST0000C
NA	NA	NA	NA	NA	NA	splice_acce	ENSG0000(	transcript	ENST0000C
NA	Benign	not_provid	NA	NA		frameshift_	ENSG0000(	transcript	ENST0000C
NA	Benign	not_provid	NA	NA		frameshift_	ENSG0000(	transcript	ENST0000C
NA	NA	NA	NA	NA	NA	stop_gaine	ENSG0000(	transcript	ENST0000C
NA	NA	NA	NA	NA	NA	structural_	ENSG0000(	interaction	3ZBF:A_21
NA	NA	NA	NA	NA	NA	structural_	ENSG0000(	interaction	4UXL:A_20
NA	NA	NA	NA	NA	NA	splice_don	ENSG0000(	transcript	ENST0000C
NA	NA	NA	NA	NA	NA	splice_don	ENSG0000(	transcript	ENST0000C
NA	NA	NA	NA	NA	NA	splice_don	ENSG0000(	transcript	ENST0000C
NA	NA	NA	NA	NA	NA	splice_acce	ENSG0000(	transcript	ENST0000C
NA	NA	NA	NA	NA	NA	splice_acce	ENSG0000(	transcript	ENST0000C

NA	NA	NA	NA	NA	stop_lost	ENSG0000(	transcript	ENST0000C
NA	Benign/Like	Arginase_d	0.04860	0.03075	structural_	ENSG0000(	interaction	1WVA:A_5(
NA	NA	NA	NA	NA	splice_don	ENSG0000(	transcript	ENST0000C
NA	NA	NA	NA	NA	stop_gaine	ENSG0000(	transcript	ENST0000C
NA	NA	NA	NA	NA	stop_gaine	ENSG0000(	transcript	ENST0000C
NA	NA	NA	NA	NA	splice_don	ENSG0000(	transcript	ENST0000C
NA	NA	NA	NA	NA	splice_don	ENSG0000(	transcript	ENST0000C
NA	NA	NA	NA	NA	start_lost	ENSG0000(	transcript	ENST0000C
NA	NA	NA	NA	NA	splice_acce	ENSG0000(	transcript	ENST0000C
NA	NA	NA	NA	NA	splice_acce	ENSG0000(	transcript	ENST0000C
NA	NA	NA	NA	NA	splice_don	ENSG0000(	transcript	ENST0000C
NA	Benign	Autoinflam	0.01073	0.01298	structural_	ENSG0000(	interaction	3ZJD:A_102
NA	NA	NA	NA	NA	frameshift_	ENSG0000(	transcript	ENST0000C
NA	NA	NA	NA	NA	structural_	ENSG0000(	interaction	3R8J:A_143
NA	NA	NA	NA	NA	splice_don	ENSG0000(	transcript	ENST0000C
NA	NA	NA	NA	NA	stop_gaine	ENSG0000(	transcript	ENST0000C
NA	NA	NA	NA	NA	splice_don	ENSG0000(	transcript	ENST0000C
NA	NA	NA	NA	NA	frameshift_	ENSG0000(	transcript	ENST0000C
NA	NA	NA	NA	NA	frameshift_	ENSG0000(	transcript	ENST0000C
NA	NA	NA	NA	NA	splice_don	ENSG0000(	transcript	ENST0000C
NA	NA	NA	NA	NA	stop_gaine	ENSG0000(	transcript	ENST0000C
NA	NA	NA	NA	NA	frameshift_	ENSG0000(	transcript	ENST0000C
NA	Benign	Tramadol_	0.19051	0.14996	stop_gaine	ENSG0000(	transcript	ENST0000C
NA	drug_respo	Tramadol_	NA	NA	stop_gaine	ENSG0000(	transcript	ENST0000C
NA	NA	NA	NA	NA	structural_	ENSG0000(	interaction	2RAJ:A_258
NA	NA	NA	NA	NA	splice_don	ENSG0000(	transcript	ENST0000C
NA	Benign	not_specifi	0.01546	0.01098	stop_gaine	ENSG0000(	transcript	ENST0000C
NA	NA	NA	NA	NA	frameshift_	ENSG0000(	transcript	ENST0000C
NA	NA	NA	NA	NA	structural_	ENSG0000(	interaction	1WL4:A_11
NA	Benign	not_provid	NA	NA	structural_	ENSG0000(	interaction	1WL4:A_28
NA	NA	NA	NA	NA	splice_don	ENSG0000(	transcript	ENST0000C
NA	NA	NA	NA	NA	splice_don	ENSG0000(	transcript	ENST0000C
Plasma_pla	Benign	not_specifi	NA	0.02336	splice_don	ENSG0000(	transcript	ENST0000C
NA	Benign/Like	Parkinson_	0.06758	0.11741	structural_	ENSG0000(	interaction	4BM9:A_16
NA	NA	NA	NA	NA	splice_don	ENSG0000(	transcript	ENST0000C
NA	NA	NA	NA	NA	splice_don	ENSG0000(	transcript	ENST0000C
NA	Likely_beni	not_provid	NA	NA	structural_	ENSG0000(	interaction	2OUN:A_67
NA	NA	NA	NA	NA	structural_	ENSG0000(	interaction	3UUO:A_51
NA	NA	NA	NA	NA	structural_	ENSG0000(	interaction	2ZMF:A_28
NA	Benign/Like	Leukoence	NA	0.07548	structural_	ENSG0000(	interaction	3T0O:A_49
NA	NA	NA	NA	NA	stop_gaine	ENSG0000(	transcript	ENST0000C
NA	NA	NA	NA	NA	splice_don	ENSG0000(	transcript	ENST0000C
NA	Likely_beni	not_provid	NA	NA	stop_gaine	ENSG0000(	transcript	ENST0000C
NA	NA	NA	NA	NA	frameshift_	ENSG0000(	transcript	ENST0000C
NA	NA	NA	NA	NA	stop_gaine	ENSG0000(	transcript	ENST0000C
NA	NA	NA	NA	NA	splice_don	ENSG0000(	transcript	ENST0000C
NA	NA	NA	NA	NA	splice_don	ENSG0000(	transcript	ENST0000C

NA	NA	NA	NA	NA	splice_acce	ENSG0000(	transcript	ENST0000C
NA	NA	NA	NA	NA	frameshift_	ENSG0000(	transcript	ENST0000C
NA	NA	NA	NA	NA	splice_don	ENSG0000(	transcript	ENST0000C
NA	Benign	not_specifi	0.02376	NA	frameshift_	ENSG0000(	transcript	ENST0000C
NA	Benign	not_specifi	0.02376	NA	frameshift_	ENSG0000(	transcript	ENST0000C
NA	NA	NA	NA	NA	frameshift_	ENSG0000(	transcript	ENST0000C
NA	NA	NA	NA	NA	frameshift_	ENSG0000(	transcript	ENST0000C
NA	NA	NA	NA	NA	frameshift_	ENSG0000(	transcript	ENST0000C
NA	Likely_beni	not_specifi	0.53191	0.61462	structural_	ENSG0000(	interaction	1C9B:B_16
NA	NA	NA	NA	NA	frameshift_	ENSG0000(	transcript	ENST0000C
NA	NA	NA	NA	NA	structural_	ENSG0000(	interaction	3FEH:A_18
NA	NA	NA	NA	NA	frameshift_	ENSG0000(	transcript	ENST0000C
NA	NA	NA	NA	NA	splice_acce	ENSG0000(	transcript	ENST0000C
NA	NA	NA	NA	NA	splice_acce	ENSG0000(	transcript	ENST0000C
Multiple_s	NA	NA	NA	NA	splice_don	ENSG0000(	transcript	ENST0000C
NA	Uncertain_	not_provid	NA	NA	frameshift_	ENSG0000(	transcript	ENST0000C
NA	Uncertain_	not_provid	NA	NA	frameshift_	ENSG0000(	transcript	ENST0000C
NA	NA	NA	NA	NA	stop_gaine	ENSG0000(	transcript	ENST0000C
Height,Wai	NA	NA	NA	NA	splice_don	ENSG0000(	transcript	ENST0000C
NA	NA	NA	NA	NA	start_lost	ENSG0000(	transcript	ENST0000C
NA	NA	NA	NA	NA	splice_acce	ENSG0000(	transcript	ENST0000C
NA	NA	NA	NA	NA	frameshift_	ENSG0000(	transcript	ENST0000C
NA	NA	NA	NA	NA	frameshift_	ENSG0000(	transcript	ENST0000C
NA	NA	NA	NA	NA	frameshift_	ENSG0000(	transcript	ENST0000C
NA	NA	NA	NA	NA	frameshift_	ENSG0000(	transcript	ENST0000C
NA	NA	NA	NA	NA	structural_	ENSG0000(	interaction	3LLP:A_305
NA	Conflicting_	Hereditary_	0.00048	0.00020	structural_	ENSG0000(	interaction	1H7S:A_29
NA	NA	NA	NA	NA	structural_	ENSG0000(	interaction	4KAX:B_30
NA	NA	NA	NA	NA	splice_don	ENSG0000(	transcript	ENST0000C
NA	NA	NA	NA	NA	splice_acce	ENSG0000(	transcript	ENST0000C
NA	NA	NA	NA	NA	start_lost&	ENSG0000(	transcript	ENST0000C
NA	NA	NA	NA	NA	start_lost&	ENSG0000(	transcript	ENST0000C
NA	NA	NA	NA	NA	start_lost&	ENSG0000(	transcript	ENST0000C
NA	NA	NA	NA	NA	start_lost&	ENSG0000(	transcript	ENST0000C
NA	NA	NA	NA	NA	start_lost&	ENSG0000(	transcript	ENST0000C
NA	NA	NA	NA	NA	start_lost&	ENSG0000(	transcript	ENST0000C
NA	NA	NA	NA	NA	frameshift_	ENSG0000(	transcript	ENST0000C
NA	NA	NA	NA	NA	frameshift_	ENSG0000(	transcript	ENST0000C
NA	NA	NA	NA	NA	frameshift_	ENSG0000(	transcript	ENST0000C
NA	NA	NA	NA	NA	frameshift_	ENSG0000(	transcript	ENST0000C
NA	NA	NA	NA	NA	stop_gaine	ENSG0000(	transcript	ENST0000C
NA	NA	NA	NA	NA	start_lost	ENSG0000(	transcript	ENST0000C
NA	NA	NA	NA	NA	frameshift_	ENSG0000(	transcript	ENST0000C
NA	NA	NA	NA	NA	splice_don	ENSG0000(	transcript	ENST0000C
NA	NA	NA	NA	NA	splice_acce	ENSG0000(	transcript	ENST0000C
NA	Benign	not_provid	NA	NA	frameshift_	ENSG0000(	transcript	ENST0000C
NA	NA	NA	NA	NA	splice_acce	ENSG0000(	transcript	ENST0000C

Coffee_cor	NA	NA	NA	NA	splice_acce	ENSG0000(	transcript	ENST0000C
NA	NA	NA	NA	NA	frameshift_	ENSG0000(	transcript	ENST0000C
NA	NA	NA	NA	NA	splice_don	ENSG0000(	transcript	ENST0000C
NA	NA	NA	NA	NA	start_lost	ENSG0000(	transcript	ENST0000C
NA	Benign	Primary_cil	0.50701	0.41194	stop_gaine	ENSG0000(	transcript	ENST0000C
NA	NA	NA	NA	NA	splice_don	ENSG0000(	transcript	ENST0000C
NA	NA	NA	NA	NA	frameshift_	ENSG0000(	transcript	ENST0000C
NA	NA	NA	NA	NA	frameshift_	ENSG0000(	transcript	ENST0000C
NA	NA	NA	NA	NA	frameshift_	ENSG0000(	transcript	ENST0000C
NA	NA	NA	NA	NA	frameshift_	ENSG0000(	transcript	ENST0000C
NA	NA	NA	NA	NA	stop_gaine	ENSG0000(	transcript	ENST0000C
NA	NA	NA	NA	NA	splice_don	ENSG0000(	transcript	ENST0000C
NA	NA	NA	NA	NA	splice_acce	ENSG0000(	transcript	ENST0000C
NA	NA	NA	NA	NA	splice_acce	ENSG0000(	transcript	ENST0000C
NA	NA	NA	NA	NA	splice_acce	ENSG0000(	transcript	ENST0000C
NA	NA	NA	NA	NA	bidirection	ENSG0000(	gene_varia	ENSG0000C
NA	NA	NA	NA	NA	bidirection	ENSG0000(	gene_varia	ENSG0000C
NA	NA	NA	NA	NA	splice_acce	ENSG0000(	transcript	ENST0000C
NA	NA	NA	NA	NA	splice_don	ENSG0000(	transcript	ENST0000C
NA	NA	NA	NA	NA	structural_	ENSG0000(	interaction	2A14:A_20
NA	NA	NA	NA	NA	structural_	ENSG0000(	interaction	2A14:A_23
NA	Benign	not_specifi	0.02492	0.01238	stop_lost	ENSG0000(	transcript	ENST0000C
NA	NA	NA	NA	NA	frameshift_	ENSG0000(	transcript	ENST0000C
NA	NA	NA	NA	NA	splice_acce	ENSG0000(	transcript	ENST0000C
NA	Benign/Like	Retinitis_pi	0.00781	0.00519	frameshift_	ENSG0000(	transcript	ENST0000C
NA	NA	NA	NA	NA	stop_lost	ENSG0000(	transcript	ENST0000C
NA	NA	NA	NA	NA	stop_gaine	ENSG0000(	transcript	ENST0000C
NA	NA	NA	NA	NA	stop_gaine	ENSG0000(	transcript	ENST0000C
NA	NA	NA	NA	NA	splice_acce	ENSG0000(	transcript	ENST0000C
NA	NA	NA	NA	NA	splice_acce	ENSG0000(	transcript	ENST0000C
NA	NA	NA	NA	NA	splice_acce	ENSG0000(	transcript	ENST0000C
NA	NA	NA	NA	NA	stop_gaine	ENSG0000(	transcript	ENST0000C
NA	NA	NA	NA	NA	frameshift_	ENSG0000(	transcript	ENST0000C
NA	NA	NA	NA	NA	frameshift_	ENSG0000(	transcript	ENST0000C
NA	NA	NA	NA	NA	splice_acce	ENSG0000(	transcript	ENST0000C
NA	NA	NA	NA	NA	splice_regi	ENSG0000(	transcript	ENST0000C
NA	NA	NA	NA	NA	splice_don	ENSG0000(	transcript	ENST0000C
NA	NA	NA	NA	NA	splice_don	ENSG0000(	transcript	ENST0000C
NA	NA	NA	NA	NA	frameshift_	ENSG0000(	transcript	ENST0000C
NA	NA	NA	NA	NA	frameshift_	ENSG0000(	transcript	ENST0000C
NA	NA	NA	NA	NA	structural_	ENSG0000(	interaction	3BHH:A_19
NA	NA	NA	NA	NA	structural_	ENSG0000(	interaction	3BHH:A_13
NA	NA	NA	NA	NA	structural_	ENSG0000(	interaction	3QNT:A_15
NA	Benign/Like	Cerebral_c	0.00167	0.00060	structural_	ENSG0000(	interaction	4WJ7:A_82
NA	NA	NA	NA	NA	structural_	ENSG0000(	interaction	1ZT3:A_23(
NA	NA	NA	NA	NA	stop_gaine	ENSG0000(	transcript	ENST0000C
NA	NA	NA	NA	NA	splice_acce	ENSG0000(	transcript	ENST0000C

NA	NA	NA	NA	NA	splice_don	ENSG0000(	transcript	ENST0000C
NA	NA	NA	NA	NA	splice_don	ENSG0000(	transcript	ENST0000C
NA	Likely_beni	Deficiency_	0.02672	0.05212	structural_	ENSG0000(	interaction	3RBF:A_75
NA	Benign	Deficiency_	NA	NA	structural_	ENSG0000(	interaction	3RBF:A_23
NA	Likely_beni	Deficiency_	0.02586	0.04473	structural_	ENSG0000(	interaction	3RCH:A_78
NA	NA	NA	NA	NA	structural_	ENSG0000(	interaction	1NRV:A_43
NA	NA	NA	NA	NA	splice_don	ENSG0000(	transcript	ENST0000C
NA	Benign/Like	Lung_canc	0.52278	0.43271	structural_	ENSG0000(	interaction	1XKK:A_71
NA	Likely_beni	Lung_canc	NA	0.00539	structural_	ENSG0000(	interaction	1XKK:A_83
NA	NA	NA	NA	NA	splice_don	ENSG0000(	transcript	ENST0000C
NA	NA	NA	NA	NA	splice_acce	ENSG0000(	transcript	ENST0000C
NA	NA	NA	NA	NA	splice_regi	ENSG0000(	transcript	ENST0000C
NA	NA	NA	NA	NA	splice_regi	ENSG0000(	transcript	ENST0000C
NA	NA	NA	NA	NA	splice_regi	ENSG0000(	transcript	ENST0000C
NA	NA	NA	NA	NA	splice_regi	ENSG0000(	transcript	ENST0000C
NA	NA	NA	NA	NA	splice_regi	ENSG0000(	transcript	ENST0000C
NA	NA	NA	NA	NA	splice_acce	ENSG0000(	transcript	ENST0000C
NA	NA	NA	NA	NA	splice_don	ENSG0000(	transcript	ENST0000C
NA	NA	NA	NA	NA	splice_acce	ENSG0000(	transcript	ENST0000C
NA	NA	NA	NA	NA	splice_don	ENSG0000(	transcript	ENST0000C
NA	NA	NA	NA	NA	splice_acce	ENSG0000(	transcript	ENST0000C
NA	NA	NA	NA	NA	splice_acce	ENSG0000(	transcript	ENST0000C
NA	NA	NA	NA	NA	splice_acce	ENSG0000(	transcript	ENST0000C
NA	NA	NA	NA	NA	splice_don	ENSG0000(	transcript	ENST0000C
NA	NA	NA	NA	NA	stop_gaine	ENSG0000(	transcript	ENST0000C
NA	NA	NA	NA	NA	stop_gaine	ENSG0000(	transcript	ENST0000C
NA	NA	NA	NA	NA	splice_acce	ENSG0000(	transcript	ENST0000C
NA	NA	NA	NA	NA	splice_acce	ENSG0000(	transcript	ENST0000C
NA	NA	NA	NA	NA	splice_acce	ENSG0000(	transcript	ENST0000C
NA	NA	NA	NA	NA	splice_acce	ENSG0000(	transcript	ENST0000C
NA	NA	NA	NA	NA	splice_acce	ENSG0000(	transcript	ENST0000C
NA	NA	NA	NA	NA	splice_acce	ENSG0000(	transcript	ENST0000C
NA	NA	NA	NA	NA	splice_acce	ENSG0000(	transcript	ENST0000C
NA	NA	NA	NA	NA	splice_acce	ENSG0000(	transcript	ENST0000C
NA	NA	NA	NA	NA	splice_acce	ENSG0000(	transcript	ENST0000C
NA	NA	NA	NA	NA	splice_acce	ENSG0000(	transcript	ENST0000C
NA	NA	NA	NA	NA	splice_acce	ENSG0000(	transcript	ENST0000C
NA	Benign/Like	Mucopolys	0.12886	0.14217	structural_	ENSG0000(	interaction	1BHG:A_53
NA	NA	NA	NA	NA	splice_don	ENSG0000(	transcript	ENST0000C
NA	NA	NA	NA	NA	splice_don	ENSG0000(	transcript	ENST0000C
NA	NA	NA	NA	NA	splice_don	ENSG0000(	transcript	ENST0000C
NA	NA	NA	NA	NA	stop_gaine	ENSG0000(	transcript	ENST0000C
NA	NA	NA	NA	NA	frameshift_	ENSG0000(	transcript	ENST0000C
NA	NA	NA	NA	NA	frameshift_	ENSG0000(	transcript	ENST0000C
NA	NA	NA	NA	NA	stop_gaine	ENSG0000(	transcript	ENST0000C
NA	NA	NA	NA	NA	splice_don	ENSG0000(	transcript	ENST0000C
NA	NA	NA	NA	NA	structural_	ENSG0000(	interaction	2B05:A_12

NA	NA	NA	NA	NA	splice_don	ENSG0000(	transcript	ENST0000C
NA	NA	NA	NA	NA	splice_acce	ENSG0000(	transcript	ENST0000C
NA	NA	NA	NA	NA	stop_gaine	ENSG0000(	transcript	ENST0000C
NA	NA	NA	NA	NA	structural_	ENSG0000(	interaction	1KJY:A_43-
NA	Benign/Like	not_specifi	0.04171	0.10344	structural_	ENSG0000(	interaction	1BHT:A_49
NA	NA	NA	NA	NA	splice_don	ENSG0000(	transcript	ENST0000C
NA	NA	NA	NA	NA	splice_acce	ENSG0000(	transcript	ENST0000C
NA	NA	NA	NA	NA	splice_acce	ENSG0000(	transcript	ENST0000C
NA	NA	NA	NA	NA	splice_acce	ENSG0000(	transcript	ENST0000C
NA	NA	NA	NA	NA	splice_don	ENSG0000(	transcript	ENST0000C
NA	NA	NA	NA	NA	splice_don	ENSG0000(	transcript	ENST0000C
NA	Benign	Angiokerat	0.12888	0.18051	structural_	ENSG0000(	interaction	4HDQ:A_6
NA	NA	NA	NA	NA	stop_gaine	ENSG0000(	transcript	ENST0000C
NA	Benign	not_provid	NA	NA	structural_	ENSG0000(	interaction	1XO2:B_10
NA	Benign	not_provid	NA	NA	stop_gaine	ENSG0000(	transcript	ENST0000C
NA	NA	NA	NA	NA	start_lost	ENSG0000(	transcript	ENST0000C
NA	NA	NA	NA	NA	splice_acce	ENSG0000(	transcript	ENST0000C
NA	NA	NA	NA	NA	splice_acce	ENSG0000(	transcript	ENST0000C
NA	NA	NA	NA	NA	splice_don	ENSG0000(	transcript	ENST0000C
NA	NA	NA	NA	NA	structural_	ENSG0000(	interaction	2ZDX:B_29
NA	NA	NA	NA	NA	structural_	ENSG0000(	interaction	2ZDX:A_13
NA	NA	NA	NA	NA	start_lost	ENSG0000(	transcript	ENST0000C
NA	NA	NA	NA	NA	splice_don	ENSG0000(	transcript	ENST0000C
NA	NA	NA	NA	NA	stop_lost	ENSG0000(	transcript	ENST0000C
Urinary_m	NA	NA	NA	NA	splice_acce	ENSG0000(	transcript	ENST0000C
NA	NA	NA	NA	NA	frameshift_	ENSG0000(	transcript	ENST0000C
NA	NA	NA	NA	NA	splice_acce	ENSG0000(	transcript	ENST0000C
NA	NA	NA	NA	NA	splice_acce	ENSG0000(	transcript	ENST0000C
NA	NA	NA	NA	NA	splice_acce	ENSG0000(	transcript	ENST0000C
NA	NA	NA	NA	NA	splice_acce	ENSG0000(	transcript	ENST0000C
NA	NA	NA	NA	NA	frameshift_	ENSG0000(	transcript	ENST0000C
NA	Benign	not_specifi	0.06865	0.03095	structural_	ENSG0000(	interaction	4MDR:A_2
NA	NA	NA	NA	NA	splice_don	ENSG0000(	transcript	ENST0000C
NA	NA	NA	NA	NA	stop_gaine	ENSG0000(	transcript	ENST0000C
NA	NA	NA	NA	NA	frameshift_	ENSG0000(	transcript	ENST0000C
NA	NA	NA	NA	NA	frameshift_	ENSG0000(	transcript	ENST0000C
NA	NA	NA	NA	NA	frameshift_	ENSG0000(	transcript	ENST0000C
NA	NA	NA	NA	NA	splice_don	ENSG0000(	transcript	ENST0000C
NA	NA	NA	NA	NA	stop_gaine	ENSG0000(	transcript	ENST0000C
NA	NA	NA	NA	NA	frameshift_	ENSG0000(	transcript	ENST0000C
NA	NA	NA	NA	NA	frameshift_	ENSG0000(	transcript	ENST0000C
NA	NA	NA	NA	NA	frameshift_	ENSG0000(	transcript	ENST0000C
NA	NA	NA	NA	NA	splice_don	ENSG0000(	transcript	ENST0000C
NA	Benign	not_provid	NA	NA	frameshift_	ENSG0000(	transcript	ENST0000C
NA	Benign	not_specifi	NA	NA	structural_	ENSG0000(	interaction	2BBA:A_10
NA	NA	NA	NA	NA	frameshift_	ENSG0000(	transcript	ENST0000C
NA	NA	NA	NA	NA	frameshift_	ENSG0000(	transcript	ENST0000C

NA	NA	NA	NA	NA	frameshift_	ENSG0000(	transcript	ENST0000C
NA	NA	NA	NA	NA	frameshift_	ENSG0000(	transcript	ENST0000C
NA	NA	NA	NA	NA	frameshift_	ENSG0000(	transcript	ENST0000C
NA	NA	NA	NA	NA	frameshift_	ENSG0000(	transcript	ENST0000C
NA	NA	NA	NA	NA	frameshift_	ENSG0000(	transcript	ENST0000C
NA	NA	NA	NA	NA	frameshift_	ENSG0000(	transcript	ENST0000C
NA	NA	NA	NA	NA	frameshift_	ENSG0000(	transcript	ENST0000C
NA	NA	NA	NA	NA	splice_acce	ENSG0000(	transcript	ENST0000C
NA	NA	NA	NA	NA	frameshift_	ENSG0000(	transcript	ENST0000C
NA	NA	NA	NA	NA	frameshift_	ENSG0000(	transcript	ENST0000C
NA	NA	NA	NA	NA	frameshift_	ENSG0000(	transcript	ENST0000C
NA	NA	NA	NA	NA	frameshift_	ENSG0000(	transcript	ENST0000C
NA	NA	NA	NA	NA	splice_don	ENSG0000(	transcript	ENST0000C
NA	NA	NA	NA	NA	splice_acce	ENSG0000(	transcript	ENST0000C
NA	NA	NA	NA	NA	splice_acce	ENSG0000(	transcript	ENST0000C
NA	NA	NA	NA	NA	structural_	ENSG0000(	interaction	1E8Y:A_35
NA	NA	NA	NA	NA	structural_	ENSG0000(	interaction	1HE8:A_35
NA	NA	NA	NA	NA	structural_	ENSG0000(	interaction	3ENE:A_88
NA	Benign	not_provid	NA	NA	frameshift_	ENSG0000(	transcript	ENST0000C
NA	NA	NA	NA	NA	splice_acce	ENSG0000(	transcript	ENST0000C
NA	NA	NA	NA	NA	splice_acce	ENSG0000(	transcript	ENST0000C
NA	NA	NA	NA	NA	non_coding	ENSG0000(	transcript	ENST0000C
NA	NA	NA	NA	NA	non_coding	ENSG0000(	transcript	ENST0000C
NA	Uncertain_	not_specifi	0.00249	0.00060	frameshift_	ENSG0000(	transcript	ENST0000C
NA	NA	NA	NA	NA	splice_acce	ENSG0000(	transcript	ENST0000C
NA	NA	NA	NA	NA	splice_regi	ENSG0000(	transcript	ENST0000C
NA	NA	NA	NA	NA	protein_pr	ENSG0000(	interaction	2XQN:A_34
NA	NA	NA	NA	NA	frameshift_	ENSG0000(	transcript	ENST0000C
NA	Benign/Like	Renal_cell_	0.03298	0.01538	structural_	ENSG0000(	interaction	2UZX:B_48
NA	Benign	Renal_cell_	0.05972	0.08846	structural_	ENSG0000(	interaction	2UZX:B_17
NA	NA	NA	NA	NA	splice_acce	ENSG0000(	transcript	ENST0000C
NA	NA	NA	NA	NA	splice_acce	ENSG0000(	transcript	ENST0000C
Heel_bone	NA	NA	NA	NA	frameshift_	ENSG0000(	transcript	ENST0000C
Heel_bone	NA	NA	NA	NA	frameshift_	ENSG0000(	transcript	ENST0000C
NA	NA	NA	NA	NA	splice_don	ENSG0000(	transcript	ENST0000C
NA	NA	NA	NA	NA	splice_don	ENSG0000(	transcript	ENST0000C
NA	NA	NA	NA	NA	start_lost	ENSG0000(	transcript	ENST0000C
NA	NA	NA	NA	NA	frameshift_	ENSG0000(	transcript	ENST0000C
NA	NA	NA	NA	NA	start_lost	ENSG0000(	transcript	ENST0000C
NA	Benign	not_specifi	0.59027	0.58207	stop_gaine	ENSG0000(	transcript	ENST0000C
NA	NA	NA	NA	NA	splice_acce	ENSG0000(	transcript	ENST0000C
NA	NA	NA	NA	NA	splice_don	ENSG0000(	transcript	ENST0000C
NA	Benign	Maturity_c	0.76323	0.66933	stop_lost	ENSG0000(	transcript	ENST0000C
NA	NA	NA	NA	NA	structural_	ENSG0000(	interaction	3ZYI:A_279
NA	NA	NA	NA	NA	structural_	ENSG0000(	interaction	3OMC:A_6
NA	NA	NA	NA	NA	splice_don	ENSG0000(	transcript	ENST0000C
NA	Likely_beni	Myofibrilla	0.00107	0.00020	structural_	ENSG0000(	interaction	3V8O:A_57

Height	NA	NA	NA	NA	splice_don	ENSG0000(	transcript	ENST0000C
NA	Benign	not_provid	NA	NA	splice_acce	ENSG0000(	transcript	ENST0000C
NA	Benign	not_specifi	0.40972	0.34185	structural_	ENSG0000(	interaction	4C0P:B_25
NA	NA	NA	NA	NA	frameshift_	ENSG0000(	transcript	ENST0000C
NA	NA	NA	NA	NA	splice_acce	ENSG0000(	transcript	ENST0000C
NA	NA	NA	NA	NA	splice_acce	ENSG0000(	transcript	ENST0000C
NA	NA	NA	NA	NA	splice_acce	ENSG0000(	transcript	ENST0000C
NA	NA	NA	NA	NA	splice_acce	ENSG0000(	transcript	ENST0000C
NA	NA	NA	NA	NA	frameshift_	ENSG0000(	transcript	ENST0000C
NA	NA	NA	NA	NA	splice_don	ENSG0000(	transcript	ENST0000C
NA	NA	NA	NA	NA	structural_	ENSG0000(	interaction	2PQF:B_55
NA	NA	NA	NA	NA	splice_acce	ENSG0000(	transcript	ENST0000C
NA	NA	NA	NA	NA	splice_acce	ENSG0000(	transcript	ENST0000C
NA	NA	NA	NA	NA	structural_	ENSG0000(	interaction	2YHF:F_12
NA	Pathogenic	Muscular_I	0.00287	0.00160	stop_gaine	ENSG0000(	transcript	ENST0000C
NA	NA	NA	NA	NA	frameshift_	ENSG0000(	transcript	ENST0000C
NA	NA	NA	NA	NA	frameshift_	ENSG0000(	transcript	ENST0000C
NA	NA	NA	NA	NA	frameshift_	ENSG0000(	transcript	ENST0000C
NA	NA	NA	NA	NA	frameshift_	ENSG0000(	transcript	ENST0000C
NA	NA	NA	NA	NA	frameshift_	ENSG0000(	transcript	ENST0000C
NA	NA	NA	NA	NA	stop_gaine	ENSG0000(	transcript	ENST0000C
NA	NA	NA	NA	NA	splice_don	ENSG0000(	transcript	ENST0000C
NA	NA	NA	NA	NA	splice_acce	ENSG0000(	transcript	ENST0000C
NA	Benign/Lik	Thiamine_r	0.00507	0.00180	structural_	ENSG0000(	interaction	3S4Y:A_33
NA	NA	NA	NA	NA	start_lost	ENSG0000(	transcript	ENST0000C
NA	NA	NA	NA	NA	splice_acce	ENSG0000(	transcript	ENST0000C
NA	NA	NA	NA	NA	splice_acce	ENSG0000(	transcript	ENST0000C
NA	NA	NA	NA	NA	splice_acce	ENSG0000(	transcript	ENST0000C
NA	NA	NA	NA	NA	splice_acce	ENSG0000(	transcript	ENST0000C
NA	NA	NA	NA	NA	splice_acce	ENSG0000(	transcript	ENST0000C
NA	NA	NA	NA	NA	splice_acce	ENSG0000(	transcript	ENST0000C
NA	NA	NA	NA	NA	splice_acce	ENSG0000(	transcript	ENST0000C
NA	NA	NA	NA	NA	frameshift_	ENSG0000(	transcript	ENST0000C
NA	NA	NA	NA	NA	splice_don	ENSG0000(	transcript	ENST0000C
NA	NA	NA	NA	NA	frameshift_	ENSG0000(	transcript	ENST0000C
NA	NA	NA	NA	NA	splice_acce	ENSG0000(	transcript	ENST0000C
NA	NA	NA	NA	NA	frameshift_	ENSG0000(	transcript	ENST0000C
NA	NA	NA	NA	NA	frameshift_	ENSG0000(	transcript	ENST0000C
NA	NA	NA	NA	NA	splice_acce	ENSG0000(	transcript	ENST0000C
NA	NA	NA	NA	NA	stop_gaine	ENSG0000(	transcript	ENST0000C
NA	NA	NA	NA	NA	splice_acce	ENSG0000(	transcript	ENST0000C
NA	Benign	not_specifi	0.05572	0.03275	stop_gaine	ENSG0000(	transcript	ENST0000C
NA	NA	NA	NA	NA	structural_	ENSG0000(	interaction	2XTM:A_2
NA	NA	NA	NA	NA	structural_	ENSG0000(	interaction	2XTM:A_6
NA	NA	NA	NA	NA	structural_	ENSG0000(	interaction	3HI7:A_32
NA	NA	NA	NA	NA	frameshift_	ENSG0000(	transcript	ENST0000C
NA	NA	NA	NA	NA	frameshift_	ENSG0000(	transcript	ENST0000C

NA	NA	NA	NA	NA	frameshift_	ENSG0000(	transcript	ENST0000C
NA	NA	NA	NA	NA	frameshift_	ENSG0000(	transcript	ENST0000C
NA	NA	NA	NA	NA	splice_don	ENSG0000(	transcript	ENST0000C
NA	NA	NA	NA	NA	splice_don	ENSG0000(	transcript	ENST0000C
NA	NA	NA	NA	NA	splice_don	ENSG0000(	transcript	ENST0000C
NA	NA	NA	NA	NA	structural_	ENSG0000(	interaction	1XFD:C_20
NA	NA	NA	NA	NA	structural_	ENSG0000(	interaction	1XFD:A_31
NA	NA	NA	NA	NA	structural_	ENSG0000(	interaction	1XFD:A_62
NA	NA	NA	NA	NA	structural_	ENSG0000(	interaction	1XFD:A_79
NA	NA	NA	NA	NA	splice_don	ENSG0000(	transcript	ENST0000C
NA	NA	NA	NA	NA	bidirection	ENSG0000(	gene_varia	ENSG0000C
NA	NA	NA	NA	NA	splice_don	ENSG0000(	transcript	ENST0000C
NA	NA	NA	NA	NA	splice_don	ENSG0000(	transcript	ENST0000C
NA	NA	NA	NA	NA	stop_lost	ENSG0000(	transcript	ENST0000C
NA	NA	NA	NA	NA	splice_acce	ENSG0000(	transcript	ENST0000C
NA	NA	NA	NA	NA	splice_acce	ENSG0000(	transcript	ENST0000C
NA	Likely_beni	not_specifi	0.08374	0.17532	structural_	ENSG0000(	interaction	1Z3S:A_28
NA	Likely_beni	not_specifi	0.05214	0.04433	structural_	ENSG0000(	interaction	1Z3S:A_36
NA	not_provid	not_provid	NA	NA	splice_don	ENSG0000(	transcript	ENST0000C
NA	NA	NA	NA	NA	stop_lost	ENSG0000(	transcript	ENST0000C
NA	NA	NA	NA	NA	splice_don	ENSG0000(	transcript	ENST0000C
NA	NA	NA	NA	NA	splice_acce	ENSG0000(	transcript	ENST0000C
NA	NA	NA	NA	NA	splice_don	ENSG0000(	transcript	ENST0000C
NA	NA	NA	NA	NA	splice_acce	ENSG0000(	transcript	ENST0000C
NA	NA	NA	NA	NA	splice_don	ENSG0000(	transcript	ENST0000C
NA	NA	NA	NA	NA	splice_don	ENSG0000(	transcript	ENST0000C
NA	NA	NA	NA	NA	splice_acce	ENSG0000(	transcript	ENST0000C
NA	NA	NA	NA	NA	splice_acce	ENSG0000(	transcript	ENST0000C
NA	NA	NA	NA	NA	splice_don	ENSG0000(	transcript	ENST0000C
NA	NA	NA	NA	NA	frameshift_	ENSG0000(	transcript	ENST0000C
NA	Uncertain_	Occult_ma	NA	NA	frameshift_	ENSG0000(	transcript	ENST0000C
NA	Benign	Occult_ma	NA	NA	frameshift_	ENSG0000(	transcript	ENST0000C
NA	Benign	Occult_ma	NA	NA	frameshift_	ENSG0000(	transcript	ENST0000C
NA	NA	NA	NA	NA	splice_acce	ENSG0000(	transcript	ENST0000C
NA	NA	NA	NA	NA	splice_acce	ENSG0000(	transcript	ENST0000C
NA	NA	NA	NA	NA	structural_	ENSG0000(	interaction	1EZF:A_44
NA	NA	NA	NA	NA	structural_	ENSG0000(	interaction	1EZF:A_20
NA	NA	NA	NA	NA	structural_	ENSG0000(	interaction	3WCH:A_2
NA	NA	NA	NA	NA	splice_acce	ENSG0000(	transcript	ENST0000C
NA	NA	NA	NA	NA	frameshift_	ENSG0000(	transcript	ENST0000C
NA	NA	NA	NA	NA	frameshift_	ENSG0000(	transcript	ENST0000C
NA	NA	NA	NA	NA	splice_acce	ENSG0000(	transcript	ENST0000C
NA	NA	NA	NA	NA	structural_	ENSG0000(	interaction	3KUQ:A_11
NA	NA	NA	NA	NA	splice_don	ENSG0000(	transcript	ENST0000C
NA	NA	NA	NA	NA	frameshift_	ENSG0000(	transcript	ENST0000C
NA	NA	NA	NA	NA	splice_don	ENSG0000(	transcript	ENST0000C
NA	NA	NA	NA	NA	splice_don	ENSG0000(	transcript	ENST0000C

NA	NA	NA	NA	NA	frameshift_	ENSG0000(	transcript	ENST0000C
NA	NA	NA	NA	NA	splice_acce	ENSG0000(	transcript	ENST0000C
NA	NA	NA	NA	NA	splice_don	ENSG0000(	transcript	ENST0000C
NA	Benign	not_provid	NA	NA	frameshift_	ENSG0000(	transcript	ENST0000C
NA	NA	NA	NA	NA	frameshift_	ENSG0000(	transcript	ENST0000C
NA	Likely_beni	Farber_dis	NA	0.07608	stop_lost&	ENSG0000(	transcript	ENST0000C
NA	NA	NA	NA	NA	splice_acce	ENSG0000(	transcript	ENST0000C
NA	NA	NA	NA	NA	structural_	ENSG0000(	interaction	2IJA:A_163
NA	NA	NA	NA	NA	gene_fusio	ENSG0000(	gene_varia	ENSG0000C
NA	NA	NA	NA	NA	gene_fusio	ENSG0000(	gene_varia	ENSG0000C
NA	NA	NA	NA	NA	gene_fusio	ENSG0000(	gene_varia	ENSG0000C
NA	NA	NA	NA	NA	gene_fusio	ENSG0000(	gene_varia	ENSG0000C
NA	NA	NA	NA	NA	gene_fusio	ENSG0000(	gene_varia	ENSG0000C
NA	NA	NA	NA	NA	gene_fusio	ENSG0000(	gene_varia	ENSG0000C
NA	NA	NA	NA	NA	gene_fusio	ENSG0000(	gene_varia	ENSG0000C
NA	NA	NA	NA	NA	gene_fusio	ENSG0000(	gene_varia	ENSG0000C
NA	NA	NA	NA	NA	gene_fusio	ENSG0000(	gene_varia	ENSG0000C
NA	NA	NA	NA	NA	gene_fusio	ENSG0000(	gene_varia	ENSG0000C
NA	NA	NA	NA	NA	splice_don	ENSG0000(	transcript	ENST0000C
Triglyceride	Likely_beni	LIPOPROTE	0.09350	0.09245	stop_gaine	ENSG0000(	transcript	ENST0000C
NA	NA	NA	NA	NA	splice_acce	ENSG0000(	transcript	ENST0000C
NA	NA	NA	NA	NA	splice_don	ENSG0000(	transcript	ENST0000C
NA	Uncertain_	Alopecia_u	0.01158	0.00659	stop_gaine	ENSG0000(	transcript	ENST0000C
NA	NA	NA	NA	NA	stop_gaine	ENSG0000(	transcript	ENST0000C
NA	NA	NA	NA	NA	splice_don	ENSG0000(	transcript	ENST0000C
NA	NA	NA	NA	NA	frameshift_	ENSG0000(	transcript	ENST0000C
NA	NA	NA	NA	NA	splice_acce	ENSG0000(	transcript	ENST0000C
NA	NA	NA	NA	NA	splice_don	ENSG0000(	transcript	ENST0000C
NA	NA	NA	NA	NA	frameshift_	ENSG0000(	transcript	ENST0000C
NA	NA	NA	NA	NA	frameshift_	ENSG0000(	transcript	ENST0000C
NA	NA	NA	NA	NA	frameshift_	ENSG0000(	transcript	ENST0000C
NA	NA	NA	NA	NA	splice_acce	ENSG0000(	transcript	ENST0000C
NA	NA	NA	NA	NA	structural_	ENSG0000(	interaction	2GSE:A_11
NA	Likely_beni	not_provid	NA	NA	structural_	ENSG0000(	interaction	2GSE:B_15
NA	NA	NA	NA	NA	structural_	ENSG0000(	interaction	3I1Y:A_55-
NA	NA	NA	NA	NA	structural_	ENSG0000(	interaction	5AIA:A_14
NA	risk_factor	Familial_hy	0.11532	0.12959	structural_	ENSG0000(	interaction	1S8O:A_25
NA	NA	NA	NA	NA	structural_	ENSG0000(	interaction	1S8O:A_40
NA	NA	NA	NA	NA	structural_	ENSG0000(	interaction	3ANS:B_41
NA	NA	NA	NA	NA	splice_don	ENSG0000(	transcript	ENST0000C
NA	NA	NA	NA	NA	splice_acce	ENSG0000(	transcript	ENST0000C
NA	NA	NA	NA	NA	splice_don	ENSG0000(	transcript	ENST0000C
NA	NA	NA	NA	NA	splice_acce	ENSG0000(	transcript	ENST0000C
NA	NA	NA	NA	NA	splice_acce	ENSG0000(	transcript	ENST0000C
NA	NA	NA	NA	NA	frameshift_	ENSG0000(	transcript	ENST0000C
NA	NA	NA	NA	NA	frameshift_	ENSG0000(	transcript	ENST0000C
NA	NA	NA	NA	NA	structural_	ENSG0000(	interaction	4B04:A_11

NA	NA	NA	NA	NA	splice_acce	ENSG0000(	transcript	ENST0000C
NA	NA	NA	NA	NA	splice_acce	ENSG0000(	transcript	ENST0000C
NA	NA	NA	NA	NA	frameshift_	ENSG0000(	transcript	ENST0000C
NA	NA	NA	NA	NA	stop_gaine	ENSG0000(	transcript	ENST0000C
NA	NA	NA	NA	NA	stop_lost	ENSG0000(	transcript	ENST0000C
NA	NA	NA	NA	NA	splice_don	ENSG0000(	transcript	ENST0000C
NA	NA	NA	NA	NA	splice_don	ENSG0000(	transcript	ENST0000C
NA	NA	NA	NA	NA	stop_gaine	ENSG0000(	transcript	ENST0000C
NA	Benign/Lik	Spherocytc	0.00474	0.00260	structural_	ENSG0000(	interaction	3UD1:A_11
NA	Benign/Lik	Spherocytc	0.02928	0.01098	structural_	ENSG0000(	interaction	1N11:A_56
NA	Uncertain_	Muscular_(	NA	NA	stop_gaine	ENSG0000(	transcript	ENST0000C
NA	NA	NA	NA	NA	splice_acce	ENSG0000(	transcript	ENST0000C
NA	NA	NA	NA	NA	splice_acce	ENSG0000(	transcript	ENST0000C
NA	NA	NA	NA	NA	splice_acce	ENSG0000(	transcript	ENST0000C
NA	NA	NA	NA	NA	stop_gaine	ENSG0000(	transcript	ENST0000C
NA	NA	NA	NA	NA	splice_don	ENSG0000(	transcript	ENST0000C
NA	NA	NA	NA	NA	splice_don	ENSG0000(	transcript	ENST0000C
NA	NA	NA	NA	NA	splice_acce	ENSG0000(	transcript	ENST0000C
NA	NA	NA	NA	NA	splice_don	ENSG0000(	transcript	ENST0000C
NA	NA	NA	NA	NA	splice_don	ENSG0000(	transcript	ENST0000C
Red_blood_	Benign	History_of_	0.42793	0.50100	structural_	ENSG0000(	interaction	2W2J:A_10
NA	NA	NA	NA	NA	splice_don	ENSG0000(	transcript	ENST0000C
NA	NA	NA	NA	NA	splice_acce	ENSG0000(	transcript	ENST0000C
NA	NA	NA	NA	NA	frameshift_	ENSG0000(	transcript	ENST0000C
NA	NA	NA	NA	NA	frameshift_	ENSG0000(	transcript	ENST0000C
NA	NA	NA	NA	NA	frameshift_	ENSG0000(	transcript	ENST0000C
NA	NA	NA	NA	NA	splice_acce	ENSG0000(	transcript	ENST0000C
NA	NA	NA	NA	NA	splice_acce	ENSG0000(	transcript	ENST0000C
NA	NA	NA	NA	NA	splice_acce	ENSG0000(	transcript	ENST0000C
NA	NA	NA	NA	NA	frameshift_	ENSG0000(	transcript	ENST0000C
NA	NA	NA	NA	NA	splice_acce	ENSG0000(	transcript	ENST0000C
NA	NA	NA	NA	NA	splice_don	ENSG0000(	transcript	ENST0000C
NA	NA	NA	NA	NA	splice_acce	ENSG0000(	transcript	ENST0000C
NA	NA	NA	NA	NA	splice_acce	ENSG0000(	transcript	ENST0000C
NA	NA	NA	NA	NA	frameshift_	ENSG0000(	transcript	ENST0000C
NA	NA	NA	NA	NA	frameshift_	ENSG0000(	transcript	ENST0000C
NA	NA	NA	NA	NA	splice_don	ENSG0000(	transcript	ENST0000C
NA	NA	NA	NA	NA	splice_don	ENSG0000(	transcript	ENST0000C
NA	NA	NA	NA	NA	splice_acce	ENSG0000(	transcript	ENST0000C
NA	NA	NA	NA	NA	splice_acce	ENSG0000(	transcript	ENST0000C
NA	NA	NA	NA	NA	splice_acce	ENSG0000(	transcript	ENST0000C
NA	NA	NA	NA	NA	splice_acce	ENSG0000(	transcript	ENST0000C
NA	NA	NA	NA	NA	splice_acce	ENSG0000(	transcript	ENST0000C
NA	NA	NA	NA	NA	splice_acce	ENSG0000(	transcript	ENST0000C
NA	NA	NA	NA	NA	splice_acce	ENSG0000(	transcript	ENST0000C
NA	NA	NA	NA	NA	splice_acce	ENSG0000(	transcript	ENST0000C
NA	NA	NA	NA	NA	splice_acce	ENSG0000(	transcript	ENST0000C

NA	NA	NA	NA	NA	splice_don	ENSG0000(	transcript	ENST0000C
NA	NA	NA	NA	NA	structural_	ENSG0000(	interaction	3DA2:A_57
NA	NA	NA	NA	NA	splice_don	ENSG0000(	transcript	ENST0000C
NA	Benign	Osteopetrc	0.58930	0.57768	structural_	ENSG0000(	interaction	4RFD:A_18
NA	NA	NA	NA	NA	frameshift_	ENSG0000(	transcript	ENST0000C
NA	NA	NA	NA	NA	splice_don	ENSG0000(	transcript	ENST0000C
NA	NA	NA	NA	NA	splice_acce	ENSG0000(	transcript	ENST0000C
NA	NA	NA	NA	NA	splice_acce	ENSG0000(	transcript	ENST0000C
NA	NA	NA	NA	NA	splice_don	ENSG0000(	transcript	ENST0000C
NA	NA	NA	NA	NA	splice_acce	ENSG0000(	transcript	ENST0000C
NA	NA	NA	NA	NA	splice_acce	ENSG0000(	transcript	ENST0000C
NA	NA	NA	NA	NA	splice_acce	ENSG0000(	transcript	ENST0000C
NA	NA	NA	NA	NA	splice_don	ENSG0000(	transcript	ENST0000C
NA	NA	NA	NA	NA	splice_acce	ENSG0000(	transcript	ENST0000C
NA	NA	NA	NA	NA	splice_acce	ENSG0000(	transcript	ENST0000C
NA	NA	NA	NA	NA	splice_acce	ENSG0000(	transcript	ENST0000C
NA	NA	NA	NA	NA	splice_acce	ENSG0000(	transcript	ENST0000C
NA	NA	NA	NA	NA	splice_acce	ENSG0000(	transcript	ENST0000C
NA	Benign	not_specifi	NA	NA	frameshift_	ENSG0000(	transcript	ENST0000C
NA	NA	NA	NA	NA	splice_acce	ENSG0000(	transcript	ENST0000C
NA	NA	NA	NA	NA	splice_don	ENSG0000(	transcript	ENST0000C
NA	NA	NA	NA	NA	stop_lost	ENSG0000(	transcript	ENST0000C
NA	NA	NA	NA	NA	splice_don	ENSG0000(	transcript	ENST0000C
NA	Benign	Cohen_syn	0.75035	0.70507	stop_gaine	ENSG0000(	transcript	ENST0000C
NA	NA	NA	NA	NA	splice_acce	ENSG0000(	transcript	ENST0000C
NA	NA	NA	NA	NA	splice_acce	ENSG0000(	transcript	ENST0000C
NA	NA	NA	NA	NA	splice_acce	ENSG0000(	transcript	ENST0000C
NA	NA	NA	NA	NA	splice_acce	ENSG0000(	transcript	ENST0000C
NA	NA	NA	NA	NA	splice_acce	ENSG0000(	transcript	ENST0000C
NA	NA	NA	NA	NA	splice_acce	ENSG0000(	transcript	ENST0000C
NA	NA	NA	NA	NA	splice_acce	ENSG0000(	transcript	ENST0000C
NA	NA	NA	NA	NA	splice_acce	ENSG0000(	transcript	ENST0000C
NA	NA	NA	NA	NA	splice_acce	ENSG0000(	transcript	ENST0000C
NA	NA	NA	NA	NA	splice_acce	ENSG0000(	transcript	ENST0000C
NA	NA	NA	NA	NA	splice_acce	ENSG0000(	transcript	ENST0000C
NA	NA	NA	NA	NA	splice_acce	ENSG0000(	transcript	ENST0000C
NA	NA	NA	NA	NA	frameshift_	ENSG0000(	transcript	ENST0000C
NA	NA	NA	NA	NA	frameshift_	ENSG0000(	transcript	ENST0000C
NA	NA	NA	NA	NA	structural_	ENSG0000(	interaction	1CVJ:G_10(
NA	NA	NA	NA	NA	splice_acce	ENSG0000(	transcript	ENST0000C
NA	NA	NA	NA	NA	structural_	ENSG0000(	interaction	1QJA:B_59
NA	NA	NA	NA	NA	splice_acce	ENSG0000(	transcript	ENST0000C
NA	Benign	not_specifi	0.05309	0.05132	frameshift_	ENSG0000(	transcript	ENST0000C
NA	Benign	not_provid	NA	NA	frameshift_	ENSG0000(	transcript	ENST0000C
NA	Benign	not_provid	NA	NA	frameshift_	ENSG0000(	transcript	ENST0000C
NA	NA	NA	NA	NA	splice_acce	ENSG0000(	transcript	ENST0000C
NA	NA	NA	NA	NA	splice_don	ENSG0000(	transcript	ENST0000C

NA	NA	NA	NA	NA	splice_don	ENSG0000(	transcript	ENST0000C
NA	NA	NA	NA	NA	start_lost	ENSG0000(	transcript	ENST0000C
NA	NA	NA	NA	NA	start_lost	ENSG0000(	transcript	ENST0000C
NA	NA	NA	NA	NA	splice_acce	ENSG0000(	transcript	ENST0000C
NA	NA	NA	NA	NA	stop_gaine	ENSG0000(	transcript	ENST0000C
NA	NA	NA	NA	NA	splice_don	ENSG0000(	transcript	ENST0000C
NA	NA	NA	NA	NA	splice_acce	ENSG0000(	transcript	ENST0000C
NA	NA	NA	NA	NA	splice_acce	ENSG0000(	transcript	ENST0000C
NA	NA	NA	NA	NA	splice_acce	ENSG0000(	transcript	ENST0000C
NA	NA	NA	NA	NA	splice_don	ENSG0000(	transcript	ENST0000C
NA	NA	NA	NA	NA	splice_don	ENSG0000(	transcript	ENST0000C
NA	NA	NA	NA	NA	structural_	ENSG0000(	interaction	2XIO:A_11
NA	Benign	MITOCHON	NA	NA	stop_gaine	ENSG0000(	transcript	ENST0000C
NA	NA	NA	NA	NA	splice_acce	ENSG0000(	transcript	ENST0000C
NA	NA	NA	NA	NA	splice_acce	ENSG0000(	transcript	ENST0000C
NA	NA	NA	NA	NA	splice_acce	ENSG0000(	transcript	ENST0000C
NA	NA	NA	NA	NA	splice_don	ENSG0000(	transcript	ENST0000C
NA	NA	NA	NA	NA	splice_acce	ENSG0000(	transcript	ENST0000C
NA	NA	NA	NA	NA	splice_don	ENSG0000(	transcript	ENST0000C
NA	NA	NA	NA	NA	splice_acce	ENSG0000(	transcript	ENST0000C
NA	NA	NA	NA	NA	stop_lost	ENSG0000(	transcript	ENST0000C
NA	NA	NA	NA	NA	splice_don	ENSG0000(	transcript	ENST0000C
NA	NA	NA	NA	NA	frameshift_	ENSG0000(	transcript	ENST0000C
NA	NA	NA	NA	NA	splice_acce	ENSG0000(	transcript	ENST0000C
NA	NA	NA	NA	NA	structural_	ENSG0000(	interaction	3LUC:A_44
NA	NA	NA	NA	NA	start_lost	ENSG0000(	transcript	ENST0000C
NA	NA	NA	NA	NA	splice_acce	ENSG0000(	transcript	ENST0000C
NA	NA	NA	NA	NA	splice_acce	ENSG0000(	transcript	ENST0000C
NA	NA	NA	NA	NA	splice_acce	ENSG0000(	transcript	ENST0000C
NA	NA	NA	NA	NA	splice_acce	ENSG0000(	transcript	ENST0000C
NA	NA	NA	NA	NA	splice_acce	ENSG0000(	transcript	ENST0000C
NA	NA	NA	NA	NA	splice_acce	ENSG0000(	transcript	ENST0000C
NA	NA	NA	NA	NA	stop_gaine	ENSG0000(	transcript	ENST0000C
NA	Benign/Like	Hyperaldos	0.00950	0.03994	structural_	ENSG0000(	interaction	4DVQ:A_38
NA	Benign	Hyperaldos	NA	0.60763	structural_	ENSG0000(	interaction	4DVQ:A_37
NA	Benign	Hyperaldos	0.41888	0.34645	structural_	ENSG0000(	interaction	4DVQ:E_17
NA	NA	NA	NA	NA	start_lost	ENSG0000(	transcript	ENST0000C
NA	NA	NA	NA	NA	frameshift_	ENSG0000(	transcript	ENST0000C
NA	NA	NA	NA	NA	frameshift_	ENSG0000(	transcript	ENST0000C
NA	NA	NA	NA	NA	stop_gaine	ENSG0000(	transcript	ENST0000C
NA	Benign	not_specifi	NA	0.23243	structural_	ENSG0000(	interaction	4Q59:A_71
NA	Benign	not_specifi	0.94716	0.88478	structural_	ENSG0000(	interaction	4EMO:D_5
Eosinophil_	NA	NA	NA	NA	structural_	ENSG0000(	interaction	4EMO:D_1
NA	NA	NA	NA	NA	splice_acce	ENSG0000(	transcript	ENST0000C
NA	NA	NA	NA	NA	splice_acce	ENSG0000(	transcript	ENST0000C
NA	NA	NA	NA	NA	frameshift_	ENSG0000(	transcript	ENST0000C
NA	NA	NA	NA	NA	frameshift_	ENSG0000(	transcript	ENST0000C

NA	NA	NA	NA	NA	stop_lost	ENSG0000(	transcript	ENST0000C
NA	NA	NA	NA	NA	frameshift_	ENSG0000(	transcript	ENST0000C
NA	NA	NA	NA	NA	frameshift_	ENSG0000(	transcript	ENST0000C
NA	NA	NA	NA	NA	splice_acce	ENSG0000(	transcript	ENST0000C
NA	NA	NA	NA	NA	splice_don	ENSG0000(	transcript	ENST0000C
NA	NA	NA	NA	NA	splice_don	ENSG0000(	transcript	ENST0000C
NA	NA	NA	NA	NA	splice_don	ENSG0000(	transcript	ENST0000C
NA	NA	NA	NA	NA	splice_don	ENSG0000(	transcript	ENST0000C
NA	NA	NA	NA	NA	splice_don	ENSG0000(	transcript	ENST0000C
NA	NA	NA	NA	NA	splice_don	ENSG0000(	transcript	ENST0000C
NA	NA	NA	NA	NA	splice_acce	ENSG0000(	transcript	ENST0000C
NA	NA	NA	NA	NA	splice_acce	ENSG0000(	transcript	ENST0000C
NA	NA	NA	NA	NA	splice_acce	ENSG0000(	transcript	ENST0000C
NA	NA	NA	NA	NA	splice_acce	ENSG0000(	transcript	ENST0000C
NA	NA	NA	NA	NA	splice_acce	ENSG0000(	transcript	ENST0000C
NA	NA	NA	NA	NA	splice_regi	ENSG0000(	transcript	ENST0000C
NA	NA	NA	NA	NA	intron_vari	ENSG0000(	transcript	ENST0000C
NA	NA	NA	NA	NA	intron_vari	ENSG0000(	transcript	ENST0000C
NA	NA	NA	NA	NA	stop_gaine	ENSG0000(	transcript	ENST0000C
NA	NA	NA	NA	NA	stop_gaine	ENSG0000(	transcript	ENST0000C
NA	NA	NA	NA	NA	frameshift_	ENSG0000(	transcript	ENST0000C
NA	NA	NA	NA	NA	splice_don	ENSG0000(	transcript	ENST0000C
NA	Benign	not_specifi	0.35526	0.47764	structural_	ENSG0000(	interaction	2FCF:A_12(
NA	NA	NA	NA	NA	stop_gaine	ENSG0000(	transcript	ENST0000C
NA	NA	NA	NA	NA	stop_gaine	ENSG0000(	transcript	ENST0000C
NA	NA	NA	NA	NA	splice_acce	ENSG0000(	transcript	ENST0000C
NA	NA	NA	NA	NA	frameshift_	ENSG0000(	transcript	ENST0000C
NA	NA	NA	NA	NA	stop_gaine	ENSG0000(	transcript	ENST0000C
NA	NA	NA	NA	NA	frameshift_	ENSG0000(	transcript	ENST0000C
NA	Benign	not_provid	NA	NA	stop_gaine	ENSG0000(	transcript	ENST0000C
NA	NA	NA	NA	NA	stop_gaine	ENSG0000(	transcript	ENST0000C
NA	Benign	Diaphyseal_	0.39442	0.38818	structural_	ENSG0000(	interaction	1CB0:A_12
NA	Benign	Diaphyseal_	0.06284	0.04073	structural_	ENSG0000(	interaction	1CB0:A_98
NA	NA	NA	NA	NA	splice_don	ENSG0000(	transcript	ENST0000C
NA	NA	NA	NA	NA	splice_acce	ENSG0000(	transcript	ENST0000C
NA	NA	NA	NA	NA	frameshift_	ENSG0000(	transcript	ENST0000C
NA	Benign	not_provid	NA	NA	structural_	ENSG0000(	interaction	2YKG:A_51
NA	Benign/Lik	Congenital_	0.05777	0.04732	structural_	ENSG0000(	interaction	2AE7:A_19
NA	NA	NA	NA	NA	stop_gaine	ENSG0000(	transcript	ENST0000C
NA	NA	NA	NA	NA	splice_don	ENSG0000(	transcript	ENST0000C
Blood_prot	Benign	Deficiency_	0.05365	0.04153	splice_acce	ENSG0000(	transcript	ENST0000C
NA	NA	NA	NA	NA	frameshift_	ENSG0000(	transcript	ENST0000C
NA	NA	NA	NA	NA	splice_acce	ENSG0000(	transcript	ENST0000C
NA	NA	NA	NA	NA	frameshift_	ENSG0000(	transcript	ENST0000C
NA	NA	NA	NA	NA	frameshift_	ENSG0000(	transcript	ENST0000C
NA	NA	NA	NA	NA	splice_acce	ENSG0000(	transcript	ENST0000C
NA	NA	NA	NA	NA	splice_acce	ENSG0000(	transcript	ENST0000C

NA	NA	NA	NA	NA	splice_don	ENSG0000(	transcript	ENST0000C
NA	NA	NA	NA	NA	stop_lost	ENSG0000(	transcript	ENST0000C
NA	NA	NA	NA	NA	stop_lost	ENSG0000(	transcript	ENST0000C
NA	NA	NA	NA	NA	frameshift_	ENSG0000(	transcript	ENST0000C
NA	NA	NA	NA	NA	splice_don	ENSG0000(	transcript	ENST0000C
NA	NA	NA	NA	NA	splice_acce	ENSG0000(	transcript	ENST0000C
NA	Benign	not_specifi	0.02602	NA	frameshift_	ENSG0000(	transcript	ENST0000C
NA	NA	NA	NA	NA	frameshift_	ENSG0000(	transcript	ENST0000C
NA	NA	NA	NA	NA	frameshift_	ENSG0000(	transcript	ENST0000C
NA	NA	NA	NA	NA	frameshift_	ENSG0000(	transcript	ENST0000C
NA	NA	NA	NA	NA	frameshift_	ENSG0000(	transcript	ENST0000C
NA	NA	NA	NA	NA	frameshift_	ENSG0000(	transcript	ENST0000C
NA	NA	NA	NA	NA	frameshift_	ENSG0000(	transcript	ENST0000C
NA	NA	NA	NA	NA	frameshift_	ENSG0000(	transcript	ENST0000C
NA	NA	NA	NA	NA	frameshift_	ENSG0000(	transcript	ENST0000C
NA	NA	NA	NA	NA	splice_don	ENSG0000(	transcript	ENST0000C
NA	NA	NA	NA	NA	splice_don	ENSG0000(	transcript	ENST0000C
NA	NA	NA	NA	NA	splice_don	ENSG0000(	transcript	ENST0000C
NA	NA	NA	NA	NA	structural_	ENSG0000(	interaction	2UZ9:A_20
NA	NA	NA	NA	NA	structural_	ENSG0000(	interaction	2QG6:A_11
NA	NA	NA	NA	NA	frameshift_	ENSG0000(	transcript	ENST0000C
NA	NA	NA	NA	NA	frameshift_	ENSG0000(	transcript	ENST0000C
NA	NA	NA	NA	NA	frameshift_	ENSG0000(	transcript	ENST0000C
NA	NA	NA	NA	NA	frameshift_	ENSG0000(	transcript	ENST0000C
NA	NA	NA	NA	NA	frameshift_	ENSG0000(	transcript	ENST0000C
NA	NA	NA	NA	NA	frameshift_	ENSG0000(	transcript	ENST0000C
NA	NA	NA	NA	NA	frameshift_	ENSG0000(	transcript	ENST0000C
NA	NA	NA	NA	NA	frameshift_	ENSG0000(	transcript	ENST0000C
NA	NA	NA	NA	NA	frameshift_	ENSG0000(	transcript	ENST0000C
NA	NA	NA	NA	NA	frameshift_	ENSG0000(	transcript	ENST0000C
NA	NA	NA	NA	NA	frameshift_	ENSG0000(	transcript	ENST0000C
NA	NA	NA	NA	NA	frameshift_	ENSG0000(	transcript	ENST0000C
NA	NA	NA	NA	NA	frameshift_	ENSG0000(	transcript	ENST0000C
NA	NA	NA	NA	NA	frameshift_	ENSG0000(	transcript	ENST0000C
NA	NA	NA	NA	NA	frameshift_	ENSG0000(	transcript	ENST0000C
NA	NA	NA	NA	NA	stop_gaine	ENSG0000(	transcript	ENST0000C
NA	NA	NA	NA	NA	stop_lost	ENSG0000(	transcript	ENST0000C
NA	NA	NA	NA	NA	stop_lost	ENSG0000(	transcript	ENST0000C
NA	NA	NA	NA	NA	splice_acce	ENSG0000(	transcript	ENST0000C
NA	Benign	Choreoaca	0.10943	0.12480	stop_lost	ENSG0000(	transcript	ENST0000C
NA	NA	NA	NA	NA	splice_don	ENSG0000(	transcript	ENST0000C
NA	Benign	Phosphose	0.62197	0.49940	structural_	ENSG0000(	interaction	3E77:A_99
NA	NA	NA	NA	NA	splice_don	ENSG0000(	transcript	ENST0000C
NA	NA	NA	NA	NA	splice_don	ENSG0000(	transcript	ENST0000C
NA	NA	NA	NA	NA	splice_acce	ENSG0000(	transcript	ENST0000C
NA	NA	NA	NA	NA	splice_regi	ENSG0000(	transcript	ENST0000C
NA	NA	NA	NA	NA	splice_regi	ENSG0000(	transcript	ENST0000C

NA	NA	NA	NA	NA	splice_acce	ENSG0000(	transcript	ENST0000C
NA	NA	NA	NA	NA	splice_acce	ENSG0000(	transcript	ENST0000C
NA	NA	NA	NA	NA	structural_	ENSG0000(	interaction	4PF4:A_38-
NA	NA	NA	NA	NA	structural_	ENSG0000(	interaction	1IG1:A_13
NA	NA	NA	NA	NA	stop_lost	ENSG0000(	transcript	ENST0000C
NA	NA	NA	NA	NA	structural_	ENSG0000(	interaction	1CKS:B_20-
High_light_	NA	NA	NA	NA	structural_	ENSG0000(	interaction	3OL2:A_32
NA	NA	NA	NA	NA	splice_don	ENSG0000(	transcript	ENST0000C
NA	NA	NA	NA	NA	structural_	ENSG0000(	interaction	1A81:E_18-
NA	NA	NA	NA	NA	structural_	ENSG0000(	interaction	4FL2:A_34
NA	NA	NA	NA	NA	structural_	ENSG0000(	interaction	1XBA:A_40
NA	NA	NA	NA	NA	structural_	ENSG0000(	interaction	1XBA:A_43
NA	NA	NA	NA	NA	structural_	ENSG0000(	interaction	1XBC:A_49
NA	Benign	Brachydact	0.42678	0.41534	structural_	ENSG0000(	interaction	3ZZW:A_65
Heel_bone_	NA	NA	NA	NA	splice_don	ENSG0000(	transcript	ENST0000C
NA	NA	NA	NA	NA	splice_acce	ENSG0000(	transcript	ENST0000C
NA	NA	NA	NA	NA	structural_	ENSG0000(	interaction	4DWN:A_1
NA	NA	NA	NA	NA	start_lost	ENSG0000(	transcript	ENST0000C
NA	NA	NA	NA	NA	stop_lost&	ENSG0000(	transcript	ENST0000C
NA	NA	NA	NA	NA	protein_pr	ENSG0000(	interaction	3FEY:A_3-C
NA	NA	NA	NA	NA	stop_gaine	ENSG0000(	transcript	ENST0000C
NA	NA	NA	NA	NA	stop_lost	ENSG0000(	transcript	ENST0000C
NA	NA	NA	NA	NA	splice_acce	ENSG0000(	transcript	ENST0000C
NA	NA	NA	NA	NA	splice_acce	ENSG0000(	transcript	ENST0000C
NA	NA	NA	NA	NA	splice_don	ENSG0000(	transcript	ENST0000C
NA	NA	NA	NA	NA	splice_don	ENSG0000(	transcript	ENST0000C
NA	NA	NA	NA	NA	frameshift_	ENSG0000(	transcript	ENST0000C
NA	NA	NA	NA	NA	frameshift_	ENSG0000(	transcript	ENST0000C
NA	NA	NA	NA	NA	frameshift_	ENSG0000(	transcript	ENST0000C
NA	NA	NA	NA	NA	frameshift_	ENSG0000(	transcript	ENST0000C
NA	NA	NA	NA	NA	splice_don	ENSG0000(	transcript	ENST0000C
NA	NA	NA	NA	NA	frameshift_	ENSG0000(	transcript	ENST0000C
NA	NA	NA	NA	NA	frameshift_	ENSG0000(	transcript	ENST0000C
NA	NA	NA	NA	NA	frameshift_	ENSG0000(	transcript	ENST0000C
NA	NA	NA	NA	NA	start_lost	ENSG0000(	transcript	ENST0000C
NA	Benign	not_provid	NA	NA	structural_	ENSG0000(	interaction	2B49:A_71
NA	NA	NA	NA	NA	splice_acce	ENSG0000(	transcript	ENST0000C
NA	NA	NA	NA	NA	intron_vari	ENSG0000(	transcript	ENST0000C
NA	NA	NA	NA	NA	intron_vari	ENSG0000(	transcript	ENST0000C
NA	NA	NA	NA	NA	intron_vari	ENSG0000(	transcript	ENST0000C
NA	NA	NA	NA	NA	frameshift_	ENSG0000(	transcript	ENST0000C
NA	NA	NA	NA	NA	splice_don	ENSG0000(	transcript	ENST0000C
NA	NA	NA	NA	NA	splice_acce	ENSG0000(	transcript	ENST0000C
NA	NA	NA	NA	NA	stop_gaine	ENSG0000(	transcript	ENST0000C
NA	NA	NA	NA	NA	structural_	ENSG0000(	interaction	3K1Z:A_13
NA	NA	NA	NA	NA	structural_	ENSG0000(	interaction	1E51:A_93-
NA	Benign	Porphobilir	0.34926	0.35224	structural_	ENSG0000(	interaction	1E51:A_36-

NA	NA	NA	NA	NA	splice_don	ENSG0000(	transcript	ENST0000C
Crohn's_di:	NA	NA	NA	NA	structural_	ENSG0000(	interaction	2O0O:A_17
NA	NA	NA	NA	NA	splice_don	ENSG0000(	transcript	ENST0000C
NA	NA	NA	NA	NA	splice_acce	ENSG0000(	transcript	ENST0000C
NA	NA	NA	NA	NA	splice_acce	ENSG0000(	transcript	ENST0000C
NA	NA	NA	NA	NA	splice_don	ENSG0000(	transcript	ENST0000C
NA	NA	NA	NA	NA	stop_gaine	ENSG0000(	transcript	ENST0000C
NA	NA	NA	NA	NA	frameshift_	ENSG0000(	transcript	ENST0000C
NA	NA	NA	NA	NA	stop_gaine	ENSG0000(	transcript	ENST0000C
NA	Benign	not_specifi	NA	NA	frameshift_	ENSG0000(	transcript	ENST0000C
NA	NA	NA	NA	NA	splice_acce	ENSG0000(	transcript	ENST0000C
NA	NA	NA	NA	NA	frameshift_	ENSG0000(	transcript	ENST0000C
NA	NA	NA	NA	NA	frameshift_	ENSG0000(	transcript	ENST0000C
NA	NA	NA	NA	NA	frameshift_	ENSG0000(	transcript	ENST0000C
NA	NA	NA	NA	NA	frameshift_	ENSG0000(	transcript	ENST0000C
NA	NA	NA	NA	NA	splice_don	ENSG0000(	transcript	ENST0000C
NA	NA	NA	NA	NA	structural_	ENSG0000(	interaction	3VOQ:A_38
NA	NA	NA	NA	NA	structural_	ENSG0000(	interaction	3TOW:A_5
NA	NA	NA	NA	NA	stop_gaine	ENSG0000(	transcript	ENST0000C
NA	Benign	not_provid	NA	NA	structural_	ENSG0000(	interaction	3T6G:A_42
NA	NA	NA	NA	NA	protein_pr	ENSG0000(	interaction	4IMY:B_11
NA	Benign	History_of_	0.07789	0.10124	structural_	ENSG0000(	interaction	2X2E:A_67
NA	NA	NA	NA	NA	stop_gaine	ENSG0000(	transcript	ENST0000C
NA	NA	NA	NA	NA	splice_acce	ENSG0000(	transcript	ENST0000C
NA	NA	NA	NA	NA	frameshift_	ENSG0000(	transcript	ENST0000C
NA	NA	NA	NA	NA	structural_	ENSG0000(	interaction	1W7L:A_12
NA	NA	NA	NA	NA	frameshift_	ENSG0000(	transcript	ENST0000C
NA	NA	NA	NA	NA	frameshift_	ENSG0000(	transcript	ENST0000C
NA	NA	NA	NA	NA	structural_	ENSG0000(	interaction	2OPW:A_1
NA	Benign/Like	Congenital_	0.00807	0.00260	frameshift_	ENSG0000(	transcript	ENST0000C
NA	NA	NA	NA	NA	stop_lost	ENSG0000(	transcript	ENST0000C
NA	NA	NA	NA	NA	stop_gaine	ENSG0000(	transcript	ENST0000C
NA	NA	NA	NA	NA	frameshift_	ENSG0000(	transcript	ENST0000C
NA	Benign/Like	Citrullinem	0.11478	0.10723	structural_	ENSG0000(	interaction	2NZ2:A_26
NA	Conflicting_	Citrullinem	0.00136	NA	structural_	ENSG0000(	interaction	2NZ2:A_34
NA	NA	NA	NA	NA	structural_	ENSG0000(	interaction	2G1T:B_46
NA	NA	NA	NA	NA	splice_acce	ENSG0000(	transcript	ENST0000C
NA	NA	NA	NA	NA	splice_acce	ENSG0000(	transcript	ENST0000C
NA	NA	NA	NA	NA	splice_acce	ENSG0000(	transcript	ENST0000C
NA	NA	NA	NA	NA	splice_don	ENSG0000(	transcript	ENST0000C
NA	NA	NA	NA	NA	start_lost	ENSG0000(	transcript	ENST0000C
NA	NA	NA	NA	NA	splice_acce	ENSG0000(	transcript	ENST0000C
NA	Benign/Like	Walker-Wa	0.10348	0.07867	stop_gaine	ENSG0000(	transcript	ENST0000C
NA	NA	NA	NA	NA	structural_	ENSG0000(	interaction	3ZYG:A_15
NA	NA	NA	NA	NA	structural_	ENSG0000(	interaction	3ZYG:A_10
NA	NA	NA	NA	NA	structural_	ENSG0000(	interaction	3ZYG:A_29
NA	NA	NA	NA	NA	gene_fusio	ENSG0000(	gene_varia	ENSG0000C

NA	NA	NA	NA	NA	structural_	ENSG0000(	interaction	3KH0:A_80
NA	NA	NA	NA	NA	stop_gaine	ENSG0000(	transcript	ENST0000C
NA	NA	NA	NA	NA	frameshift_	ENSG0000(	transcript	ENST0000C
Serum_alka	Affects	ABO_blooc	NA	NA	frameshift_	ENSG0000(	transcript	ENST0000C
NA	NA	NA	NA	NA	stop_gaine	ENSG0000(	transcript	ENST0000C
Malaria,Ve	NA	NA	NA	NA	splice_acce	ENSG0000(	transcript	ENST0000C
NA	Benign	not_provid	NA	NA	structural_	ENSG0000(	interaction	3GHM:A_5
NA	NA	NA	NA	NA	structural_	ENSG0000(	interaction	3EG6:A_16
NA	NA	NA	NA	NA	structural_	ENSG0000(	interaction	1XV9:A_25
NA	NA	NA	NA	NA	frameshift_	ENSG0000(	transcript	ENST0000C
NA	NA	NA	NA	NA	frameshift_	ENSG0000(	transcript	ENST0000C
NA	NA	NA	NA	NA	splice_don	ENSG0000(	transcript	ENST0000C
NA	NA	NA	NA	NA	splice_acce	ENSG0000(	transcript	ENST0000C
NA	NA	NA	NA	NA	frameshift_	ENSG0000(	transcript	ENST0000C
NA	NA	NA	NA	NA	frameshift_	ENSG0000(	transcript	ENST0000C
NA	Benign	not_specifi	NA	NA	splice_acce	ENSG0000(	transcript	ENST0000C
NA	Benign	not_specifi	NA	NA	splice_acce	ENSG0000(	transcript	ENST0000C
NA	NA	NA	NA	NA	frameshift_	ENSG0000(	transcript	ENST0000C
NA	NA	NA	NA	NA	stop_gaine	ENSG0000(	transcript	ENST0000C
Chronic_in	Conflicting_	Candidiasis	NA	0.00080	splice_don	ENSG0000(	transcript	ENST0000C
NA	Benign/Like	Joubert_sy	0.20473	0.17392	structural_	ENSG0000(	interaction	2XSW:A_28
NA	Benign/Like	Joubert_sy	NA	0.12141	structural_	ENSG0000(	interaction	2XSW:A_48
NA	Benign	Joubert_sy	0.41940	0.37840	structural_	ENSG0000(	interaction	2XSW:A_41
NA	NA	NA	NA	NA	stop_gaine	ENSG0000(	transcript	ENST0000C
NA	NA	NA	NA	NA	structural_	ENSG0000(	interaction	2XST:A_11
NA	NA	NA	NA	NA	structural_	ENSG0000(	interaction	2HW4:A_24
NA	NA	NA	NA	NA	splice_acce	ENSG0000(	transcript	ENST0000C
NA	NA	NA	NA	NA	splice_acce	ENSG0000(	transcript	ENST0000C
NA	NA	NA	NA	NA	splice_acce	ENSG0000(	transcript	ENST0000C
NA	NA	NA	NA	NA	stop_gaine	ENSG0000(	transcript	ENST0000C
NA	Benign	History_of_	NA	0.80990	structural_	ENSG0000(	interaction	1FMI:A_53
NA	NA	NA	NA	NA	splice_don	ENSG0000(	transcript	ENST0000C
NA	NA	NA	NA	NA	frameshift_	ENSG0000(	transcript	ENST0000C
NA	NA	NA	NA	NA	splice_acce	ENSG0000(	transcript	ENST0000C
NA	NA	NA	NA	NA	stop_lost	ENSG0000(	transcript	ENST0000C
Chronic_ki	NA	NA	NA	NA	stop_gaine	ENSG0000(	transcript	ENST0000C
NA	NA	NA	NA	NA	structural_	ENSG0000(	interaction	2PNY:A_73
NA	NA	NA	NA	NA	splice_don	ENSG0000(	transcript	ENST0000C
NA	NA	NA	NA	NA	stop_lost	ENSG0000(	transcript	ENST0000C
NA	NA	NA	NA	NA	gene_fusio	ENSG0000(	gene_varia	ENSG00000
NA	NA	NA	NA	NA	structural_	ENSG0000(	interaction	4U1R:B_56
NA	NA	NA	NA	NA	structural_	ENSG0000(	interaction	4L3T:A_86
NA	NA	NA	NA	NA	structural_	ENSG0000(	interaction	4NGE:A_87
NA	NA	NA	NA	NA	stop_lost	ENSG0000(	transcript	ENST0000C
NA	NA	NA	NA	NA	structural_	ENSG0000(	interaction	4L3T:A_11
NA	NA	NA	NA	NA	structural_	ENSG0000(	interaction	4L3T:B_94-
NA	Uncertain_	Irido-corne	NA	NA	frameshift_	ENSG0000(	transcript	ENST0000C

NA	NA	NA	NA	NA	stop_gaine	ENSG0000(	transcript	ENST0000C
NA	NA	NA	NA	NA	structural_	ENSG0000(	interaction	1MRQ:A_1
NA	NA	NA	NA	NA	splice_don	ENSG0000(	transcript	ENST0000C
NA	NA	NA	NA	NA	start_lost	ENSG0000(	transcript	ENST0000C
NA	NA	NA	NA	NA	structural_	ENSG0000(	interaction	3EO2:A_20
NA	NA	NA	NA	NA	splice_acce	ENSG0000(	transcript	ENST0000C
NA	NA	NA	NA	NA	splice_acce	ENSG0000(	transcript	ENST0000C
NA	NA	NA	NA	NA	splice_acce	ENSG0000(	transcript	ENST0000C
NA	NA	NA	NA	NA	frameshift_	ENSG0000(	transcript	ENST0000C
NA	NA	NA	NA	NA	frameshift_	ENSG0000(	transcript	ENST0000C
Type_1_di	NA	NA	NA	NA	structural_	ENSG0000(	interaction	1XJD:A_59
NA	NA	NA	NA	NA	structural_	ENSG0000(	interaction	1XJD:A_44
NA	Benign	not_provid	NA	NA	structural_	ENSG0000(	interaction	1XJD:A_44
NA	NA	NA	NA	NA	splice_acce	ENSG0000(	transcript	ENST0000C
NA	NA	NA	NA	NA	splice_acce	ENSG0000(	transcript	ENST0000C
NA	NA	NA	NA	NA	splice_acce	ENSG0000(	transcript	ENST0000C
NA	NA	NA	NA	NA	splice_acce	ENSG0000(	transcript	ENST0000C
NA	NA	NA	NA	NA	structural_	ENSG0000(	interaction	2VX2:A_14
NA	NA	NA	NA	NA	structural_	ENSG0000(	interaction	2VX2:A_14
NA	NA	NA	NA	NA	structural_	ENSG0000(	interaction	2VX2:A_14
NA	NA	NA	NA	NA	structural_	ENSG0000(	interaction	2VX2:B_24
NA	NA	NA	NA	NA	structural_	ENSG0000(	interaction	2VX2:A_16
NA	NA	NA	NA	NA	structural_	ENSG0000(	interaction	3FD5:A_29
NA	NA	NA	NA	NA	structural_	ENSG0000(	interaction	3FD5:A_12
NA	NA	NA	NA	NA	splice_don	ENSG0000(	transcript	ENST0000C
NA	NA	NA	NA	NA	splice_acce	ENSG0000(	transcript	ENST0000C
NA	NA	NA	NA	NA	splice_acce	ENSG0000(	transcript	ENST0000C
NA	NA	NA	NA	NA	splice_regi	ENSG0000(	transcript	ENST0000C
NA	NA	NA	NA	NA	structural_	ENSG0000(	interaction	1G55:A_14
NA	NA	NA	NA	NA	structural_	ENSG0000(	interaction	1G55:A_60
NA	Likely_beni	Brugada_s	NA	NA	splice_acce	ENSG0000(	transcript	ENST0000C
NA	Likely_beni	Brugada_s	NA	NA	splice_acce	ENSG0000(	transcript	ENST0000C
NA	Likely_beni	Brugada_s	NA	NA	splice_acce	ENSG0000(	transcript	ENST0000C
NA	Likely_beni	Brugada_s	NA	NA	splice_acce	ENSG0000(	transcript	ENST0000C
NA	Benign	not_specifi	0.00473	NA	splice_acce	ENSG0000(	transcript	ENST0000C
NA	Benign	not_specifi	0.00473	NA	splice_acce	ENSG0000(	transcript	ENST0000C
NA	Benign	not_specifi	0.00473	NA	splice_acce	ENSG0000(	transcript	ENST0000C
NA	Benign	not_specifi	0.00473	NA	splice_acce	ENSG0000(	transcript	ENST0000C
NA	Benign/Lik	Brugada_s	NA	0.00280	splice_acce	ENSG0000(	transcript	ENST0000C
NA	NA	NA	NA	NA	frameshift_	ENSG0000(	transcript	ENST0000C
NA	NA	NA	NA	NA	splice_acce	ENSG0000(	transcript	ENST0000C
NA	NA	NA	NA	NA	splice_acce	ENSG0000(	transcript	ENST0000C
NA	NA	NA	NA	NA	splice_acce	ENSG0000(	transcript	ENST0000C
NA	NA	NA	NA	NA	splice_acce	ENSG0000(	transcript	ENST0000C
NA	NA	NA	NA	NA	splice_acce	ENSG0000(	transcript	ENST0000C
NA	NA	NA	NA	NA	splice_acce	ENSG0000(	transcript	ENST0000C
NA	NA	NA	NA	NA	splice_acce	ENSG0000(	transcript	ENST0000C

NA	NA	NA	NA	NA	structural_	ENSG0000(	interaction	2OKK:A_33
NA	NA	NA	NA	NA	splice_don	ENSG0000(	transcript	ENST0000C
Inflammatc	NA	NA	NA	NA	structural_	ENSG0000(	interaction	4Y83:A_78
NA	NA	NA	NA	NA	splice_acce	ENSG0000(	transcript	ENST0000C
NA	NA	NA	NA	NA	splice_acce	ENSG0000(	transcript	ENST0000C
NA	NA	NA	NA	NA	start_lost	ENSG0000(	transcript	ENST0000C
NA	NA	NA	NA	NA	frameshift_	ENSG0000(	transcript	ENST0000C
NA	NA	NA	NA	NA	splice_don	ENSG0000(	transcript	ENST0000C
NA	NA	NA	NA	NA	frameshift_	ENSG0000(	transcript	ENST0000C
NA	NA	NA	NA	NA	structural_	ENSG0000(	interaction	2QQI:A_52
NA	NA	NA	NA	NA	structural_	ENSG0000(	interaction	2QQI:A_33
NA	NA	NA	NA	NA	stop_gaine	ENSG0000(	transcript	ENST0000C
NA	NA	NA	NA	NA	frameshift_	ENSG0000(	transcript	ENST0000C
NA	NA	NA	NA	NA	splice_don	ENSG0000(	transcript	ENST0000C
NA	NA	NA	NA	NA	splice_don	ENSG0000(	transcript	ENST0000C
NA	NA	NA	NA	NA	splice_don	ENSG0000(	transcript	ENST0000C
NA	Conflicting_	Pheochrom	0.00180	0.00020	structural_	ENSG0000(	interaction	2IVV:A_74
NA	NA	NA	NA	NA	frameshift_	ENSG0000(	transcript	ENST0000C
NA	NA	NA	NA	NA	frameshift_	ENSG0000(	transcript	ENST0000C
NA	NA	NA	NA	NA	splice_don	ENSG0000(	transcript	ENST0000C
NA	NA	NA	NA	NA	splice_acce	ENSG0000(	transcript	ENST0000C
NA	NA	NA	NA	NA	frameshift_	ENSG0000(	transcript	ENST0000C
NA	NA	NA	NA	NA	splice_don	ENSG0000(	transcript	ENST0000C
NA	NA	NA	NA	NA	splice_acce	ENSG0000(	transcript	ENST0000C
NA	NA	NA	NA	NA	splice_don	ENSG0000(	transcript	ENST0000C
NA	NA	NA	NA	NA	splice_acce	ENSG0000(	transcript	ENST0000C
NA	NA	NA	NA	NA	frameshift_	ENSG0000(	transcript	ENST0000C
NA	NA	NA	NA	NA	splice_acce	ENSG0000(	transcript	ENST0000C
NA	NA	NA	NA	NA	frameshift_	ENSG0000(	transcript	ENST0000C
NA	NA	NA	NA	NA	splice_acce	ENSG0000(	transcript	ENST0000C
NA	NA	NA	NA	NA	splice_acce	ENSG0000(	transcript	ENST0000C
NA	Benign	not_specifi	NA	0.79353	structural_	ENSG0000(	interaction	2FY2:A_42
NA	NA	NA	NA	NA	stop_gaine	ENSG0000(	transcript	ENST0000C
NA	NA	NA	NA	NA	splice_don	ENSG0000(	transcript	ENST0000C
NA	NA	NA	NA	NA	splice_acce	ENSG0000(	transcript	ENST0000C
NA	NA	NA	NA	NA	splice_acce	ENSG0000(	transcript	ENST0000C
NA	NA	NA	NA	NA	splice_acce	ENSG0000(	transcript	ENST0000C
NA	NA	NA	NA	NA	splice_acce	ENSG0000(	transcript	ENST0000C
NA	NA	NA	NA	NA	splice_acce	ENSG0000(	transcript	ENST0000C
NA	NA	NA	NA	NA	splice_don	ENSG0000(	transcript	ENST0000C
NA	NA	NA	NA	NA	splice_don	ENSG0000(	transcript	ENST0000C
NA	NA	NA	NA	NA	splice_don	ENSG0000(	transcript	ENST0000C
NA	NA	NA	NA	NA	frameshift_	ENSG0000(	transcript	ENST0000C
NA	NA	NA	NA	NA	frameshift_	ENSG0000(	transcript	ENST0000C
NA	Benign	not_provid	NA	NA	frameshift_	ENSG0000(	transcript	ENST0000C
NA	NA	NA	NA	NA	splice_acce	ENSG0000(	transcript	ENST0000C
NA	Benign/Like	Aortic_ane	0.00389	0.00100	structural_	ENSG0000(	interaction	3OCP:A_14

NA	Benign	Aortic_ane	0.04446	0.03215	structural_	ENSG0000(	interaction	4KU7:A_28
NA	NA	NA	NA	NA	splice_acce	ENSG0000(	transcript	ENST0000C
NA	NA	NA	NA	NA	splice_acce	ENSG0000(	transcript	ENST0000C
NA	NA	NA	NA	NA	splice_acce	ENSG0000(	transcript	ENST0000C
Cocaine_de	NA	NA	NA	NA	splice_acce	ENSG0000(	transcript	ENST0000C
NA	NA	NA	NA	NA	frameshift_	ENSG0000(	transcript	ENST0000C
NA	NA	NA	NA	NA	stop_gaine	ENSG0000(	transcript	ENST0000C
NA	NA	NA	NA	NA	structural_	ENSG0000(	interaction	2YPD:A_22
NA	NA	NA	NA	NA	splice_don	ENSG0000(	transcript	ENST0000C
NA	NA	NA	NA	NA	splice_don	ENSG0000(	transcript	ENST0000C
NA	NA	NA	NA	NA	splice_don	ENSG0000(	transcript	ENST0000C
NA	NA	NA	NA	NA	structural_	ENSG0000(	interaction	4IF6:A_332
NA	NA	NA	NA	NA	splice_acce	ENSG0000(	transcript	ENST0000C
NA	NA	NA	NA	NA	start_lost	ENSG0000(	transcript	ENST0000C
NA	NA	NA	NA	NA	splice_acce	ENSG0000(	transcript	ENST0000C
NA	NA	NA	NA	NA	splice_regi	ENSG0000(	transcript	ENST0000C
NA	NA	NA	NA	NA	splice_regi	ENSG0000(	transcript	ENST0000C
NA	NA	NA	NA	NA	splice_regi	ENSG0000(	transcript	ENST0000C
NA	NA	NA	NA	NA	splice_regi	ENSG0000(	transcript	ENST0000C
NA	NA	NA	NA	NA	splice_regi	ENSG0000(	transcript	ENST0000C
NA	NA	NA	NA	NA	structural_	ENSG0000(	interaction	4Q6R:A_31
NA	NA	NA	NA	NA	splice_don	ENSG0000(	transcript	ENST0000C
NA	Benign	not_provid	0.57981	0.29792	splice_acce	ENSG0000(	transcript	ENST0000C
NA	NA	NA	NA	NA	frameshift_	ENSG0000(	transcript	ENST0000C
NA	NA	NA	NA	NA	splice_acce	ENSG0000(	transcript	ENST0000C
NA	NA	NA	NA	NA	splice_acce	ENSG0000(	transcript	ENST0000C
NA	NA	NA	NA	NA	splice_don	ENSG0000(	transcript	ENST0000C
NA	NA	NA	NA	NA	frameshift_	ENSG0000(	transcript	ENST0000C
NA	NA	NA	NA	NA	start_lost&	ENSG0000(	transcript	ENST0000C
NA	NA	NA	NA	NA	frameshift_	ENSG0000(	transcript	ENST0000C
NA	NA	NA	NA	NA	structural_	ENSG0000(	interaction	2GWO:A_7
NA	NA	NA	NA	NA	frameshift_	ENSG0000(	transcript	ENST0000C
NA	NA	NA	NA	NA	splice_acce	ENSG0000(	transcript	ENST0000C
NA	NA	NA	NA	NA	splice_acce	ENSG0000(	transcript	ENST0000C
NA	NA	NA	NA	NA	splice_acce	ENSG0000(	transcript	ENST0000C
NA	NA	NA	NA	NA	bidirection	ENSG0000(	gene_varia	ENSG0000C
NA	NA	NA	NA	NA	splice_don	ENSG0000(	transcript	ENST0000C
NA	NA	NA	NA	NA	structural_	ENSG0000(	interaction	3RCG:A_88
NA	NA	NA	NA	NA	splice_acce	ENSG0000(	transcript	ENST0000C
NA	NA	NA	NA	NA	splice_don	ENSG0000(	transcript	ENST0000C
NA	NA	NA	NA	NA	frameshift_	ENSG0000(	transcript	ENST0000C
NA	NA	NA	NA	NA	frameshift_	ENSG0000(	transcript	ENST0000C
NA	NA	NA	NA	NA	frameshift_	ENSG0000(	transcript	ENST0000C
NA	NA	NA	NA	NA	splice_don	ENSG0000(	transcript	ENST0000C
NA	NA	NA	NA	NA	splice_don	ENSG0000(	transcript	ENST0000C
NA	Benign	Hepatic_m	0.51236	0.58686	structural_	ENSG0000(	interaction	2OBV:A_27
NA	Benign	Hepatic_m	0.00209	0.00100	structural_	ENSG0000(	interaction	2OBV:A_35

NA	Benign	Hepatic_m	0.75430	0.86342	structural_	ENSG0000(	interaction	2OBV:A_14
NA	NA	NA	NA	NA	splice_acce	ENSG0000(	transcript	ENST0000C
NA	NA	NA	NA	NA	structural_	ENSG0000(	interaction	4K6J:A_103
NA	NA	NA	NA	NA	stop_lost	ENSG0000(	transcript	ENST0000C
NA	NA	NA	NA	NA	splice_acce	ENSG0000(	transcript	ENST0000C
NA	NA	NA	NA	NA	splice_acce	ENSG0000(	transcript	ENST0000C
NA	Benign/Like	Hereditary_	NA	NA	splice_acce	ENSG0000(	transcript	ENST0000C
NA	Benign/Like	Hereditary_	NA	NA	splice_acce	ENSG0000(	transcript	ENST0000C
NA	Benign/Like	Hereditary_	NA	NA	splice_acce	ENSG0000(	transcript	ENST0000C
NA	NA	NA	NA	NA	splice_acce	ENSG0000(	transcript	ENST0000C
NA	NA	NA	NA	NA	splice_acce	ENSG0000(	transcript	ENST0000C
NA	NA	NA	NA	NA	splice_done	ENSG0000(	transcript	ENST0000C
NA	NA	NA	NA	NA	frameshift_	ENSG0000(	transcript	ENST0000C
NA	NA	NA	NA	NA	structural_	ENSG0000(	interaction	2G47:B_53
NA	NA	NA	NA	NA	stop_gaine	ENSG0000(	transcript	ENST0000C
NA	NA	NA	NA	NA	stop_lost	ENSG0000(	transcript	ENST0000C
Warfarin_r	drug_respo	warfarin_re	0.09144	0.04792	structural_	ENSG0000(	interaction	1OG2:A_14
Warfarin_r	drug_respo	acenocoum	0.06371	0.04852	structural_	ENSG0000(	interaction	4NZ2:B_35
NA	NA	NA	NA	NA	structural_	ENSG0000(	interaction	1OG2:B_48
NA	Benign	CYP2C8_PC	NA	0.01657	structural_	ENSG0000(	interaction	1PQ2:A_18
NA	NA	NA	NA	NA	frameshift_	ENSG0000(	transcript	ENST0000C
Blood_met	Benign	not_specifi	NA	0.20008	splice_done	ENSG0000(	transcript	ENST0000C
NA	NA	NA	NA	NA	start_lost	ENSG0000(	transcript	ENST0000C
NA	NA	NA	NA	NA	frameshift_	ENSG0000(	transcript	ENST0000C
NA	Likely_beni	Primary_hy	0.03999	0.06090	structural_	ENSG0000(	interaction	3S5N:A_10
NA	NA	NA	NA	NA	stop_lost	ENSG0000(	transcript	ENST0000C
NA	NA	NA	NA	NA	splice_acce	ENSG0000(	transcript	ENST0000C
NA	NA	NA	NA	NA	frameshift_	ENSG0000(	transcript	ENST0000C
NA	NA	NA	NA	NA	structural_	ENSG0000(	interaction	4CC7:C_15
NA	NA	NA	NA	NA	structural_	ENSG0000(	interaction	4GLM:A_24
NA	NA	NA	NA	NA	splice_done	ENSG0000(	transcript	ENST0000C
NA	NA	NA	NA	NA	structural_	ENSG0000(	interaction	2ILM:A_12
NA	Benign/Like	Medullobla	0.15868	0.24601	structural_	ENSG0000(	interaction	4KM8:A_40
NA	NA	NA	NA	NA	gene_fusio	ENSG0000(	gene_varia	ENSG0000C
NA	NA	NA	NA	NA	stop_gaine	ENSG0000(	transcript	ENST0000C
NA	NA	NA	NA	NA	splice_done	ENSG0000(	transcript	ENST0000C
NA	NA	NA	NA	NA	structural_	ENSG0000(	interaction	2J51:A_154
NA	NA	NA	NA	NA	frameshift_	ENSG0000(	transcript	ENST0000C
NA	NA	NA	NA	NA	structural_	ENSG0000(	interaction	2G6Z:A_20
NA	NA	NA	NA	NA	stop_gaine	ENSG0000(	transcript	ENST0000C
NA	NA	NA	NA	NA	frameshift_	ENSG0000(	transcript	ENST0000C
NA	NA	NA	NA	NA	structural_	ENSG0000(	interaction	1F1J:A_181
NA	NA	NA	NA	NA	structural_	ENSG0000(	interaction	1K88:B_25
NA	NA	NA	NA	NA	splice_acce	ENSG0000(	transcript	ENST0000C
NA	NA	NA	NA	NA	splice_acce	ENSG0000(	transcript	ENST0000C
NA	NA	NA	NA	NA	splice_acce	ENSG0000(	transcript	ENST0000C
NA	NA	NA	NA	NA	splice_acce	ENSG0000(	transcript	ENST0000C

NA	NA	NA	NA	NA	splice_acce	ENSG0000(	transcript	ENST0000C
NA	NA	NA	NA	NA	frameshift_	ENSG0000(	transcript	ENST0000C
NA	NA	NA	NA	NA	structural_	ENSG0000(	interaction	2PPL:A_61-
NA	NA	NA	NA	NA	structural_	ENSG0000(	interaction	2PPL:A_378
NA	NA	NA	NA	NA	stop_lost	ENSG0000(	transcript	ENST0000C
NA	NA	NA	NA	NA	splice_acce	ENSG0000(	transcript	ENST0000C
NA	NA	NA	NA	NA	splice_acce	ENSG0000(	transcript	ENST0000C
NA	NA	NA	NA	NA	protein_pr	ENSG0000(	interaction	2IHB:A_18(
NA	Benign	Craniosyno	NA	0.79533	structural_	ENSG0000(	interaction	1NUN:B_19
NA	Likely_beni	Macular_d	NA	0.11442	stop_gaine	ENSG0000(	transcript	ENST0000C
NA	NA	NA	NA	NA	frameshift_	ENSG0000(	transcript	ENST0000C
NA	NA	NA	NA	NA	frameshift_	ENSG0000(	transcript	ENST0000C
NA	NA	NA	NA	NA	frameshift_	ENSG0000(	transcript	ENST0000C
NA	NA	NA	NA	NA	frameshift_	ENSG0000(	transcript	ENST0000C
NA	NA	NA	NA	NA	frameshift_	ENSG0000(	transcript	ENST0000C
NA	NA	NA	NA	NA	frameshift_	ENSG0000(	transcript	ENST0000C
NA	Benign	Ornithine_	0.41803	0.40994	structural_	ENSG0000(	interaction	1GBN:A_35
NA	NA	NA	NA	NA	splice_don	ENSG0000(	transcript	ENST0000C
NA	Likely_beni	not_provid	NA	NA	frameshift_	ENSG0000(	transcript	ENST0000C
NA	NA	NA	NA	NA	splice_acce	ENSG0000(	transcript	ENST0000C
Age_at_me	NA	NA	NA	NA	splice_don	ENSG0000(	transcript	ENST0000C
NA	NA	NA	NA	NA	splice_acce	ENSG0000(	transcript	ENST0000C
NA	NA	NA	NA	NA	splice_acce	ENSG0000(	transcript	ENST0000C
NA	NA	NA	NA	NA	stop_gaine	ENSG0000(	transcript	ENST0000C
NA	NA	NA	NA	NA	frameshift_	ENSG0000(	transcript	ENST0000C
NA	NA	NA	NA	NA	structural_	ENSG0000(	interaction	4DD8:C_33
NA	NA	NA	NA	NA	frameshift_	ENSG0000(	transcript	ENST0000C
NA	NA	NA	NA	NA	splice_don	ENSG0000(	transcript	ENST0000C
NA	NA	NA	NA	NA	splice_acce	ENSG0000(	transcript	ENST0000C
NA	NA	NA	NA	NA	splice_regi	ENSG0000(	transcript	ENST0000C
NA	NA	NA	NA	NA	structural_	ENSG0000(	interaction	3GPH:A_17
NA	NA	NA	NA	NA	splice_acce	ENSG0000(	transcript	ENST0000C
NA	NA	NA	NA	NA	frameshift_	ENSG0000(	transcript	ENST0000C
NA	NA	NA	NA	NA	structural_	ENSG0000(	interaction	3GLS:C_208
NA	NA	NA	NA	NA	structural_	ENSG0000(	interaction	4BVH:A_15
NA	NA	NA	NA	NA	frameshift_	ENSG0000(	transcript	ENST0000C
NA	NA	NA	NA	NA	splice_acce	ENSG0000(	transcript	ENST0000C
NA	NA	NA	NA	NA	frameshift_	ENSG0000(	transcript	ENST0000C
NA	NA	NA	NA	NA	frameshift_	ENSG0000(	transcript	ENST0000C
NA	NA	NA	NA	NA	frameshift_	ENSG0000(	transcript	ENST0000C
NA	NA	NA	NA	NA	frameshift_	ENSG0000(	transcript	ENST0000C
NA	NA	NA	NA	NA	splice_acce	ENSG0000(	transcript	ENST0000C
NA	NA	NA	NA	NA	splice_acce	ENSG0000(	transcript	ENST0000C
NA	NA	NA	NA	NA	splice_acce	ENSG0000(	transcript	ENST0000C
NA	NA	NA	NA	NA	splice_don	ENSG0000(	transcript	ENST0000C
NA	NA	NA	NA	NA	splice_don	ENSG0000(	transcript	ENST0000C
NA	NA	NA	NA	NA	splice_don	ENSG0000(	transcript	ENST0000C

NA	NA	NA	NA	NA	frameshift_	ENSG0000(	transcript	ENST0000C
NA	NA	NA	NA	NA	frameshift_	ENSG0000(	transcript	ENST0000C
NA	NA	NA	NA	NA	frameshift_	ENSG0000(	transcript	ENST0000C
NA	NA	NA	NA	NA	frameshift_	ENSG0000(	transcript	ENST0000C
NA	Likely_beni	Dystonia	NA	NA	structural_	ENSG0000(	interaction	2XSN:A_19
NA	Conflicting_	Dystonia|S	0.00872	0.00120	structural_	ENSG0000(	interaction	2XSN:A_23
Pursuit_ma	NA	NA	NA	NA	start_lost	ENSG0000(	transcript	ENST0000C
NA	NA	NA	NA	NA	start_lost	ENSG0000(	transcript	ENST0000C
NA	NA	NA	NA	NA	splice_don	ENSG0000(	transcript	ENST0000C
NA	NA	NA	NA	NA	structural_	ENSG0000(	interaction	3HNC:B_28
NA	NA	NA	NA	NA	structural_	ENSG0000(	interaction	3HNC:A_71
NA	Benign	not_specifi	NA	NA	frameshift_	ENSG0000(	transcript	ENST0000C
NA	NA	NA	NA	NA	frameshift_	ENSG0000(	transcript	ENST0000C
NA	NA	NA	NA	NA	frameshift_	ENSG0000(	transcript	ENST0000C
NA	NA	NA	NA	NA	frameshift_	ENSG0000(	transcript	ENST0000C
NA	NA	NA	NA	NA	stop_gaine	ENSG0000(	transcript	ENST0000C
NA	Benign	not_specifi	0.21287	0.21586	frameshift_	ENSG0000(	transcript	ENST0000C
NA	NA	NA	NA	NA	frameshift_	ENSG0000(	transcript	ENST0000C
NA	NA	NA	NA	NA	stop_gaine	ENSG0000(	transcript	ENST0000C
NA	NA	NA	NA	NA	frameshift_	ENSG0000(	transcript	ENST0000C
NA	NA	NA	NA	NA	frameshift_	ENSG0000(	transcript	ENST0000C
NA	NA	NA	NA	NA	frameshift_	ENSG0000(	transcript	ENST0000C
NA	NA	NA	NA	NA	splice_don	ENSG0000(	transcript	ENST0000C
NA	Benign	Hb_SS_dise	NA	0.71426	structural_	ENSG0000(	interaction	1DXT:B_3-I
NA	NA	NA	NA	NA	frameshift_	ENSG0000(	transcript	ENST0000C
NA	NA	NA	NA	NA	frameshift_	ENSG0000(	transcript	ENST0000C
NA	NA	NA	NA	NA	stop_gaine	ENSG0000(	transcript	ENST0000C
NA	NA	NA	NA	NA	stop_gaine	ENSG0000(	transcript	ENST0000C
NA	NA	NA	NA	NA	frameshift_	ENSG0000(	transcript	ENST0000C
NA	NA	NA	NA	NA	frameshift_	ENSG0000(	transcript	ENST0000C
NA	NA	NA	NA	NA	stop_gaine	ENSG0000(	transcript	ENST0000C
NA	NA	NA	NA	NA	frameshift_	ENSG0000(	transcript	ENST0000C
NA	NA	NA	NA	NA	stop_lost	ENSG0000(	transcript	ENST0000C
NA	NA	NA	NA	NA	stop_gaine	ENSG0000(	transcript	ENST0000C
NA	NA	NA	NA	NA	splice_acce	ENSG0000(	transcript	ENST0000C
NA	NA	NA	NA	NA	frameshift_	ENSG0000(	transcript	ENST0000C
NA	NA	NA	NA	NA	stop_gaine	ENSG0000(	transcript	ENST0000C
NA	Benign	not_specifi	0.26771	0.31470	protein_pr	ENSG0000(	interaction	3F6Q:A_99
NA	NA	NA	NA	NA	frameshift_	ENSG0000(	transcript	ENST0000C
NA	NA	NA	NA	NA	bidirection	ENSG0000(	gene_varia	ENSG0000C
NA	NA	NA	NA	NA	structural_	ENSG0000(	interaction	4N1J:A_22-
NA	NA	NA	NA	NA	frameshift_	ENSG0000(	transcript	ENST0000C
NA	NA	NA	NA	NA	frameshift_	ENSG0000(	transcript	ENST0000C
NA	NA	NA	NA	NA	splice_acce	ENSG0000(	transcript	ENST0000C
NA	NA	NA	NA	NA	splice_acce	ENSG0000(	transcript	ENST0000C
NA	NA	NA	NA	NA	splice_acce	ENSG0000(	transcript	ENST0000C
NA	NA	NA	NA	NA	splice_acce	ENSG0000(	transcript	ENST0000C

NA	NA	NA	NA	NA	splice_acce	ENSG0000	transcript	ENST0000
NA	NA	NA	NA	NA	splice_acce	ENSG0000	transcript	ENST0000
NA	NA	NA	NA	NA	splice_don	ENSG0000	transcript	ENST0000
NA	NA	NA	NA	NA	splice_don	ENSG0000	transcript	ENST0000
NA	NA	NA	NA	NA	splice_acce	ENSG0000	transcript	ENST0000
NA	NA	NA	NA	NA	splice_don	ENSG0000	transcript	ENST0000
NA	NA	NA	NA	NA	splice_acce	ENSG0000	transcript	ENST0000
NA	NA	NA	NA	NA	splice_acce	ENSG0000	transcript	ENST0000
NA	NA	NA	NA	NA	splice_acce	ENSG0000	transcript	ENST0000
NA	NA	NA	NA	NA	splice_acce	ENSG0000	transcript	ENST0000
NA	NA	NA	NA	NA	splice_acce	ENSG0000	transcript	ENST0000
NA	NA	NA	NA	NA	splice_acce	ENSG0000	transcript	ENST0000
NA	NA	NA	NA	NA	frameshift_	ENSG0000	transcript	ENST0000
NA	NA	NA	NA	NA	splice_acce	ENSG0000	transcript	ENST0000
NA	NA	NA	NA	NA	splice_acce	ENSG0000	transcript	ENST0000
NA	NA	NA	NA	NA	splice_acce	ENSG0000	transcript	ENST0000
NA	NA	NA	NA	NA	structural_	ENSG0000	interaction	2ZOT:A_44
NA	NA	NA	NA	NA	structural_	ENSG0000	interaction	3Q13:A_23
NA	NA	NA	NA	NA	splice_don	ENSG0000	transcript	ENST0000
NA	NA	NA	NA	NA	splice_don	ENSG0000	transcript	ENST0000
NA	NA	NA	NA	NA	structural_	ENSG0000	interaction	3C6G:B_59
NA	NA	NA	NA	NA	splice_acce	ENSG0000	transcript	ENST0000
NA	NA	NA	NA	NA	frameshift_	ENSG0000	transcript	ENST0000
NA	NA	NA	NA	NA	splice_acce	ENSG0000	transcript	ENST0000
NA	Conflicting_	Retinitis_pi	0.00227	0.00100	structural_	ENSG0000	interaction	3K1R:A_13
NA	Pathogenic	Intellectual	0.00076	NA	stop_gaine	ENSG0000	transcript	ENST0000
NA	NA	NA	NA	NA	stop_gaine	ENSG0000	transcript	ENST0000
NA	NA	NA	NA	NA	stop_gaine	ENSG0000	transcript	ENST0000
NA	NA	NA	NA	NA	frameshift_	ENSG0000	transcript	ENST0000
NA	Conflicting_	Glycogen_	0.01017	0.00339	structural_	ENSG0000	interaction	4OJN:F_12
NA	Benign	Glycogen_	0.69779	0.63458	structural_	ENSG0000	interaction	4OJN:A_13
NA	NA	NA	NA	NA	start_lost	ENSG0000	transcript	ENST0000
NA	NA	NA	NA	NA	structural_	ENSG0000	interaction	2BKA:A_19
NA	NA	NA	NA	NA	splice_don	ENSG0000	transcript	ENST0000
NA	NA	NA	NA	NA	splice_don	ENSG0000	transcript	ENST0000
NA	NA	NA	NA	NA	structural_	ENSG0000	interaction	3MS5:A_15
NA	NA	NA	NA	NA	structural_	ENSG0000	interaction	3LRE:A_33
NA	NA	NA	NA	NA	splice_acce	ENSG0000	transcript	ENST0000
NA	NA	NA	NA	NA	splice_acce	ENSG0000	transcript	ENST0000
NA	NA	NA	NA	NA	splice_don	ENSG0000	transcript	ENST0000
NA	NA	NA	NA	NA	stop_gaine	ENSG0000	transcript	ENST0000
NA	NA	NA	NA	NA	splice_acce	ENSG0000	transcript	ENST0000
NA	NA	NA	NA	NA	stop_gaine	ENSG0000	transcript	ENST0000
NA	NA	NA	NA	NA	splice_acce	ENSG0000	transcript	ENST0000
NA	Benign	not_provid	0.24173	0.26278	structural_	ENSG0000	interaction	1DGG:C_3
NA	NA	NA	NA	NA	structural_	ENSG0000	interaction	4M6R:B_1
NA	NA	NA	NA	NA	structural_	ENSG0000	interaction	4M6R:A_5

NA	Benign	not_provid	NA	NA	splice_don	ENSG0000(	transcript	ENST0000C
NA	NA	NA	NA	NA	splice_acce	ENSG0000(	transcript	ENST0000C
NA	NA	NA	NA	NA	splice_regi	ENSG0000(	transcript	ENST0000C
NA	NA	NA	NA	NA	splice_regi	ENSG0000(	transcript	ENST0000C
NA	NA	NA	NA	NA	splice_acce	ENSG0000(	transcript	ENST0000C
NA	NA	NA	NA	NA	splice_don	ENSG0000(	transcript	ENST0000C
NA	NA	NA	NA	NA	splice_don	ENSG0000(	transcript	ENST0000C
NA	NA	NA	NA	NA	splice_don	ENSG0000(	transcript	ENST0000C
NA	NA	NA	NA	NA	structural_	ENSG0000(	interaction	2IUW:A_2C
NA	NA	NA	NA	NA	splice_acce	ENSG0000(	transcript	ENST0000C
NA	NA	NA	NA	NA	splice_don	ENSG0000(	transcript	ENST0000C
NA	NA	NA	NA	NA	splice_don	ENSG0000(	transcript	ENST0000C
NA	NA	NA	NA	NA	splice_don	ENSG0000(	transcript	ENST0000C
NA	NA	NA	NA	NA	frameshift_	ENSG0000(	transcript	ENST0000C
NA	NA	NA	NA	NA	frameshift_	ENSG0000(	transcript	ENST0000C
NA	NA	NA	NA	NA	frameshift_	ENSG0000(	transcript	ENST0000C
NA	NA	NA	NA	NA	splice_acce	ENSG0000(	transcript	ENST0000C
NA	NA	NA	NA	NA	frameshift_	ENSG0000(	transcript	ENST0000C
NA	NA	NA	NA	NA	frameshift_	ENSG0000(	transcript	ENST0000C
NA	NA	NA	NA	NA	frameshift_	ENSG0000(	transcript	ENST0000C
NA	NA	NA	NA	NA	frameshift_	ENSG0000(	transcript	ENST0000C
NA	NA	NA	NA	NA	frameshift_	ENSG0000(	transcript	ENST0000C
NA	NA	NA	NA	NA	splice_acce	ENSG0000(	transcript	ENST0000C
NA	NA	NA	NA	NA	splice_acce	ENSG0000(	transcript	ENST0000C
NA	Benign/Like	not_specifi	0.00531	0.00140	structural_	ENSG0000(	interaction	3P70:B_54(
NA	NA	NA	NA	NA	splice_don	ENSG0000(	transcript	ENST0000C
NA	Conflicting_	Xeroderma	0.00251	0.00260	structural_	ENSG0000(	interaction	4E54:B_24(
NA	NA	NA	NA	NA	splice_don	ENSG0000(	transcript	ENST0000C
Fasting_blc	NA	NA	NA	NA	stop_gaine	ENSG0000(	transcript	ENST0000C
NA	Benign/Like	Cardiomyo	0.10790	0.06709	structural_	ENSG0000(	interaction	2V6H:A_19
NA	Benign/Like	Cardiomyo	0.02879	0.00839	structural_	ENSG0000(	interaction	2V6H:A_16
NA	NA	NA	NA	NA	frameshift_	ENSG0000(	transcript	ENST0000C
NA	NA	NA	NA	NA	bidirection	ENSG0000(	gene_varia	ENSG0000C
NA	NA	NA	NA	NA	splice_acce	ENSG0000(	transcript	ENST0000C
NA	NA	NA	NA	NA	splice_acce	ENSG0000(	transcript	ENST0000C
NA	NA	NA	NA	NA	splice_acce	ENSG0000(	transcript	ENST0000C
NA	NA	NA	NA	NA	splice_acce	ENSG0000(	transcript	ENST0000C
NA	NA	NA	NA	NA	stop_gaine	ENSG0000(	transcript	ENST0000C
NA	NA	NA	NA	NA	synonymou	ENSG0000(	transcript	ENST0000C
NA	NA	NA	NA	NA	stop_gaine	ENSG0000(	transcript	ENST0000C
NA	NA	NA	NA	NA	splice_don	ENSG0000(	transcript	ENST0000C
NA	NA	NA	NA	NA	structural_	ENSG0000(	interaction	2C6C:A_11
NA	NA	NA	NA	NA	structural_	ENSG0000(	interaction	2C6C:A_11
Intraocular	NA	NA	NA	NA	stop_lost&	ENSG0000(	transcript	ENST0000C
NA	NA	NA	NA	NA	splice_don	ENSG0000(	transcript	ENST0000C
NA	NA	NA	NA	NA	stop_lost	ENSG0000(	transcript	ENST0000C
NA	NA	NA	NA	NA	stop_gaine	ENSG0000(	transcript	ENST0000C

NA	NA	NA	NA	NA	stop_gaine	ENSG0000(	transcript	ENST0000C
NA	NA	NA	NA	NA	stop_gaine	ENSG0000(	transcript	ENST0000C
NA	NA	NA	NA	NA	splice_don	ENSG0000(	transcript	ENST0000C
NA	NA	NA	NA	NA	frameshift_	ENSG0000(	transcript	ENST0000C
NA	NA	NA	NA	NA	frameshift_	ENSG0000(	transcript	ENST0000C
NA	NA	NA	NA	NA	stop_gaine	ENSG0000(	transcript	ENST0000C
NA	NA	NA	NA	NA	frameshift_	ENSG0000(	transcript	ENST0000C
NA	NA	NA	NA	NA	stop_gaine	ENSG0000(	transcript	ENST0000C
NA	NA	NA	NA	NA	frameshift_	ENSG0000(	transcript	ENST0000C
NA	NA	NA	NA	NA	frameshift_	ENSG0000(	transcript	ENST0000C
NA	NA	NA	NA	NA	stop_gaine	ENSG0000(	transcript	ENST0000C
NA	NA	NA	NA	NA	frameshift_	ENSG0000(	transcript	ENST0000C
NA	NA	NA	NA	NA	splice_don	ENSG0000(	transcript	ENST0000C
NA	NA	NA	NA	NA	splice_don	ENSG0000(	transcript	ENST0000C
NA	NA	NA	NA	NA	frameshift_	ENSG0000(	transcript	ENST0000C
NA	NA	NA	NA	NA	stop_lost&	ENSG0000(	transcript	ENST0000C
NA	NA	NA	NA	NA	frameshift_	ENSG0000(	transcript	ENST0000C
NA	NA	NA	NA	NA	frameshift_	ENSG0000(	transcript	ENST0000C
NA	NA	NA	NA	NA	splice_acce	ENSG0000(	transcript	ENST0000C
NA	NA	NA	NA	NA	frameshift_	ENSG0000(	transcript	ENST0000C
NA	Benign	not_provid	NA	NA	frameshift_	ENSG0000(	transcript	ENST0000C
NA	NA	NA	NA	NA	splice_don	ENSG0000(	transcript	ENST0000C
NA	NA	NA	NA	NA	frameshift_	ENSG0000(	transcript	ENST0000C
NA	NA	NA	NA	NA	stop_gaine	ENSG0000(	transcript	ENST0000C
NA	NA	NA	NA	NA	stop_gaine	ENSG0000(	transcript	ENST0000C
NA	NA	NA	NA	NA	splice_acce	ENSG0000(	transcript	ENST0000C
NA	NA	NA	NA	NA	splice_acce	ENSG0000(	transcript	ENST0000C
NA	NA	NA	NA	NA	splice_acce	ENSG0000(	transcript	ENST0000C
NA	NA	NA	NA	NA	splice_regi	ENSG0000(	transcript	ENST0000C
NA	NA	NA	NA	NA	non_codin	ENSG0000(	transcript	ENST0000C
NA	NA	NA	NA	NA	splice_acce	ENSG0000(	transcript	ENST0000C
NA	NA	NA	NA	NA	frameshift_	ENSG0000(	transcript	ENST0000C
NA	NA	NA	NA	NA	splice_acce	ENSG0000(	transcript	ENST0000C
NA	NA	NA	NA	NA	stop_gaine	ENSG0000(	transcript	ENST0000C
NA	NA	NA	NA	NA	structural_	ENSG0000(	interaction	4LG8:A_27
NA	Benign	not_provid	NA	NA	structural_	ENSG0000(	interaction	2B5L:B_37
NA	NA	NA	NA	NA	structural_	ENSG0000(	interaction	3EI1:A_51-
NA	Benign	Joubert_sy	0.85933	0.85583	splice_acce	ENSG0000(	transcript	ENST0000C
NA	Benign	Joubert_sy	NA	0.71965	splice_acce	ENSG0000(	transcript	ENST0000C
NA	NA	NA	NA	NA	splice_don	ENSG0000(	transcript	ENST0000C
NA	NA	NA	NA	NA	stop_gaine	ENSG0000(	transcript	ENST0000C
NA	NA	NA	NA	NA	structural_	ENSG0000(	interaction	1JKG:B_40
NA	NA	NA	NA	NA	splice_don	ENSG0000(	transcript	ENST0000C
NA	NA	NA	NA	NA	stop_gaine	ENSG0000(	transcript	ENST0000C
NA	NA	NA	NA	NA	stop_gaine	ENSG0000(	transcript	ENST0000C
NA	NA	NA	NA	NA	splice_don	ENSG0000(	transcript	ENST0000C
NA	NA	NA	NA	NA	stop_lost	ENSG0000(	transcript	ENST0000C

NA	NA	NA	NA	NA	stop_gaine	ENSG0000(	transcript	ENST0000C
NA	NA	NA	NA	NA	frameshift_	ENSG0000(	transcript	ENST0000C
NA	NA	NA	NA	NA	frameshift_	ENSG0000(	transcript	ENST0000C
NA	NA	NA	NA	NA	splice_don	ENSG0000(	transcript	ENST0000C
NA	NA	NA	NA	NA	splice_don	ENSG0000(	transcript	ENST0000C
NA	NA	NA	NA	NA	frameshift_	ENSG0000(	transcript	ENST0000C
NA	NA	NA	NA	NA	structural_	ENSG0000(	interaction	3OHM:B_2
NA	NA	NA	NA	NA	structural_	ENSG0000(	interaction	3OHM:B_4
High_densi	NA	NA	NA	NA	structural_	ENSG0000(	interaction	3OHM:B_7
NA	NA	NA	NA	NA	transcript_	ENSG0000(	transcript	ENST0000C
NA	Benign/Like	Hyperparat	0.39335	0.30791	structural_	ENSG0000(	interaction	3U84:A_41
NA	NA	NA	NA	NA	splice_don	ENSG0000(	transcript	ENST0000C
NA	NA	NA	NA	NA	structural_	ENSG0000(	interaction	3DOE:A_11
NA	NA	NA	NA	NA	splice_acce	ENSG0000(	transcript	ENST0000C
NA	NA	NA	NA	NA	splice_acce	ENSG0000(	transcript	ENST0000C
NA	NA	NA	NA	NA	splice_acce	ENSG0000(	transcript	ENST0000C
NA	NA	NA	NA	NA	splice_acce	ENSG0000(	transcript	ENST0000C
NA	NA	NA	NA	NA	frameshift_	ENSG0000(	transcript	ENST0000C
NA	Benign	not_provid	NA	NA	structural_	ENSG0000(	interaction	1NFI:A_207
NA	NA	NA	NA	NA	splice_don	ENSG0000(	transcript	ENST0000C
NA	NA	NA	NA	NA	splice_don	ENSG0000(	transcript	ENST0000C
NA	NA	NA	NA	NA	splice_acce	ENSG0000(	transcript	ENST0000C
NA	Benign	not_specifi	0.57928	0.42991	structural_	ENSG0000(	interaction	4P0P:A_30
NA	Benign	not_specifi	NA	0.42832	structural_	ENSG0000(	interaction	4P0P:A_31
NA	NA	NA	NA	NA	splice_acce	ENSG0000(	transcript	ENST0000C
NA	NA	NA	NA	NA	frameshift_	ENSG0000(	transcript	ENST0000C
NA	NA	NA	NA	NA	structural_	ENSG0000(	interaction	3FVY:A_67
Blood_prot	Conflicting_	ACTININ,_A	0.45197	0.40076	stop_gaine	ENSG0000(	transcript	ENST0000C
NA	NA	NA	NA	NA	structural_	ENSG0000(	interaction	2YU1:A_35
NA	NA	NA	NA	NA	structural_	ENSG0000(	interaction	3KRW:A_3
NA	NA	NA	NA	NA	structural_	ENSG0000(	interaction	3A1J:A_48
NA	NA	NA	NA	NA	protein_pr	ENSG0000(	interaction	3V4Y:A_25
NA	NA	NA	NA	NA	frameshift_	ENSG0000(	transcript	ENST0000C
NA	NA	NA	NA	NA	stop_gaine	ENSG0000(	transcript	ENST0000C
NA	NA	NA	NA	NA	splice_don	ENSG0000(	transcript	ENST0000C
NA	Conflicting_	Spinal_mus	0.00646	0.00479	structural_	ENSG0000(	interaction	4B3G:A_23
NA	NA	NA	NA	NA	structural_	ENSG0000(	interaction	2W9Z:A_17
NA	NA	NA	NA	NA	stop_gaine	ENSG0000(	transcript	ENST0000C
NA	NA	NA	NA	NA	stop_gaine	ENSG0000(	transcript	ENST0000C
NA	NA	NA	NA	NA	splice_acce	ENSG0000(	transcript	ENST0000C
NA	NA	NA	NA	NA	frameshift_	ENSG0000(	transcript	ENST0000C
NA	NA	NA	NA	NA	frameshift_	ENSG0000(	transcript	ENST0000C
NA	Conflicting_	Small_for_	0.00068	NA	stop_gaine	ENSG0000(	transcript	ENST0000C
NA	NA	NA	NA	NA	frameshift_	ENSG0000(	transcript	ENST0000C
NA	NA	NA	NA	NA	splice_don	ENSG0000(	transcript	ENST0000C
NA	NA	NA	NA	NA	splice_acce	ENSG0000(	transcript	ENST0000C
Blood_prot	Benign	not_provid	NA	NA	frameshift_	ENSG0000(	transcript	ENST0000C

NA	NA	NA	NA	NA	structural_	ENSG0000(	interaction	3ITU:C_591
NA	NA	NA	NA	NA	stop_gaine	ENSG0000(	transcript	ENST0000C
NA	NA	NA	NA	NA	splice_acce	ENSG0000(	transcript	ENST0000C
NA	NA	NA	NA	NA	bidirection	ENSG0000(	gene_varia	ENSG0000C
NA	NA	NA	NA	NA	bidirection	ENSG0000(	gene_varia	ENSG0000C
NA	NA	NA	NA	NA	bidirection	ENSG0000(	gene_varia	ENSG0000C
NA	NA	NA	NA	NA	bidirection	ENSG0000(	gene_varia	ENSG0000C
NA	NA	NA	NA	NA	bidirection	ENSG0000(	gene_varia	ENSG0000C
NA	NA	NA	NA	NA	bidirection	ENSG0000(	gene_varia	ENSG0000C
NA	NA	NA	NA	NA	bidirection	ENSG0000(	gene_varia	ENSG0000C
NA	NA	NA	NA	NA	bidirection	ENSG0000(	gene_varia	ENSG0000C
NA	NA	NA	NA	NA	bidirection	ENSG0000(	gene_varia	ENSG0000C
NA	NA	NA	NA	NA	bidirection	ENSG0000(	gene_varia	ENSG0000C
NA	NA	NA	NA	NA	bidirection	ENSG0000(	gene_varia	ENSG0000C
NA	NA	NA	NA	NA	bidirection	ENSG0000(	gene_varia	ENSG0000C
NA	NA	NA	NA	NA	bidirection	ENSG0000(	gene_varia	ENSG0000C
NA	NA	NA	NA	NA	bidirection	ENSG0000(	gene_varia	ENSG0000C
NA	NA	NA	NA	NA	bidirection	ENSG0000(	gene_varia	ENSG0000C
NA	NA	NA	NA	NA	bidirection	ENSG0000(	gene_varia	ENSG0000C
NA	NA	NA	NA	NA	bidirection	ENSG0000(	gene_varia	ENSG0000C
NA	NA	NA	NA	NA	bidirection	ENSG0000(	gene_varia	ENSG0000C
NA	NA	NA	NA	NA	bidirection	ENSG0000(	gene_varia	ENSG0000C
NA	NA	NA	NA	NA	bidirection	ENSG0000(	gene_varia	ENSG0000C
NA	NA	NA	NA	NA	bidirection	ENSG0000(	gene_varia	ENSG0000C
NA	NA	NA	NA	NA	bidirection	ENSG0000(	gene_varia	ENSG0000C
NA	NA	NA	NA	NA	bidirection	ENSG0000(	gene_varia	ENSG0000C
NA	NA	NA	NA	NA	bidirection	ENSG0000(	gene_varia	ENSG0000C
NA	NA	NA	NA	NA	bidirection	ENSG0000(	gene_varia	ENSG0000C
NA	NA	NA	NA	NA	bidirection	ENSG0000(	gene_varia	ENSG0000C
NA	NA	NA	NA	NA	stop_gaine	ENSG0000(	transcript	ENST0000C
NA	NA	NA	NA	NA	splice_acce	ENSG0000(	transcript	ENST0000C
NA	NA	NA	NA	NA	frameshift_	ENSG0000(	transcript	ENST0000C
NA	NA	NA	NA	NA	frameshift_	ENSG0000(	transcript	ENST0000C
NA	NA	NA	NA	NA	stop_gaine	ENSG0000(	transcript	ENST0000C
NA	NA	NA	NA	NA	stop_gaine	ENSG0000(	transcript	ENST0000C
NA	NA	NA	NA	NA	frameshift_	ENSG0000(	transcript	ENST0000C
NA	NA	NA	NA	NA	splice_don	ENSG0000(	transcript	ENST0000C
NA	Benign	Deafness,_	NA	NA	frameshift_	ENSG0000(	transcript	ENST0000C
NA	NA	NA	NA	NA	frameshift_	ENSG0000(	transcript	ENST0000C
NA	NA	NA	NA	NA	frameshift_	ENSG0000(	transcript	ENST0000C
NA	NA	NA	NA	NA	structural_	ENSG0000(	interaction	3N2Z:B_42
NA	NA	NA	NA	NA	structural_	ENSG0000(	interaction	3N2Z:B_29
Blood_prot	NA	NA	NA	NA	structural_	ENSG0000(	interaction	3N2Z:B_11
NA	NA	NA	NA	NA	frameshift_	ENSG0000(	transcript	ENST0000C
NA	NA	NA	NA	NA	splice_don	ENSG0000(	transcript	ENST0000C
NA	NA	NA	NA	NA	splice_acce	ENSG0000(	transcript	ENST0000C
NA	NA	NA	NA	NA	splice_acce	ENSG0000(	transcript	ENST0000C

NA	NA	NA	NA	NA	splice_acce	ENSG0000(	transcript	ENST0000C
NA	NA	NA	NA	NA	splice_acce	ENSG0000(	transcript	ENST0000C
NA	NA	NA	NA	NA	splice_acce	ENSG0000(	transcript	ENST0000C
NA	NA	NA	NA	NA	splice_don	ENSG0000(	transcript	ENST0000C
NA	NA	NA	NA	NA	frameshift_	ENSG0000(	transcript	ENST0000C
NA	NA	NA	NA	NA	frameshift_	ENSG0000(	transcript	ENST0000C
NA	NA	NA	NA	NA	frameshift_	ENSG0000(	transcript	ENST0000C
NA	NA	NA	NA	NA	structural_	ENSG0000(	interaction	3FEC:A_10
NA	NA	NA	NA	NA	splice_don	ENSG0000(	transcript	ENST0000C
NA	NA	NA	NA	NA	splice_don	ENSG0000(	transcript	ENST0000C
NA	NA	NA	NA	NA	splice_don	ENSG0000(	transcript	ENST0000C
NA	Benign/Like	Charcot-M	0.03619	0.04233	structural_	ENSG0000(	interaction	1LW3:A_5C
NA	Benign/Like	Charcot-M	0.01701	0.00619	structural_	ENSG0000(	interaction	1M7R:B_31
NA	NA	NA	NA	NA	splice_acce	ENSG0000(	transcript	ENST0000C
NA	NA	NA	NA	NA	splice_acce	ENSG0000(	transcript	ENST0000C
NA	NA	NA	NA	NA	splice_acce	ENSG0000(	transcript	ENST0000C
NA	NA	NA	NA	NA	splice_acce	ENSG0000(	transcript	ENST0000C
NA	NA	NA	NA	NA	splice_acce	ENSG0000(	transcript	ENST0000C
NA	NA	NA	NA	NA	splice_acce	ENSG0000(	transcript	ENST0000C
NA	NA	NA	NA	NA	splice_acce	ENSG0000(	transcript	ENST0000C
NA	NA	NA	NA	NA	splice_don	ENSG0000(	transcript	ENST0000C
NA	NA	NA	NA	NA	stop_gaine	ENSG0000(	transcript	ENST0000C
NA	NA	NA	NA	NA	stop_gaine	ENSG0000(	transcript	ENST0000C
NA	NA	NA	NA	NA	structural_	ENSG0000(	interaction	3G8O:A_8C
NA	Likely_beni	not_specifi	0.01609	0.00639	stop_gaine	ENSG0000(	transcript	ENST0000C
Blood_prot	NA	NA	NA	NA	splice_acce	ENSG0000(	transcript	ENST0000C
NA	NA	NA	NA	NA	structural_	ENSG0000(	interaction	1Q3A:A_11
NA	NA	NA	NA	NA	structural_	ENSG0000(	interaction	1Q3A:A_11
NA	Benign	not_specifi	0.02082	0.01198	structural_	ENSG0000(	interaction	1SU3:A_27
NA	NA	NA	NA	NA	frameshift_	ENSG0000(	transcript	ENST0000C
NA	Likely_beni	not_provid	NA	NA	structural_	ENSG0000(	interaction	3E4C:A_28
NA	Benign/Like	Deficiency_	0.15550	0.12700	structural_	ENSG0000(	interaction	2F2S:A_15
NA	NA	NA	NA	NA	splice_acce	ENSG0000(	transcript	ENST0000C
NA	NA	NA	NA	NA	splice_acce	ENSG0000(	transcript	ENST0000C
NA	NA	NA	NA	NA	splice_acce	ENSG0000(	transcript	ENST0000C
NA	NA	NA	NA	NA	splice_don	ENSG0000(	transcript	ENST0000C
NA	NA	NA	NA	NA	frameshift_	ENSG0000(	transcript	ENST0000C
NA	NA	NA	NA	NA	frameshift_	ENSG0000(	transcript	ENST0000C
NA	NA	NA	NA	NA	splice_acce	ENSG0000(	transcript	ENST0000C
NA	NA	NA	NA	NA	structural_	ENSG0000(	interaction	3MTR:A_54
NA	NA	NA	NA	NA	splice_don	ENSG0000(	transcript	ENST0000C
Feeling_gu	NA	NA	NA	NA	splice_acce	ENSG0000(	transcript	ENST0000C
NA	NA	NA	NA	NA	structural_	ENSG0000(	interaction	2IIP:A_8-A_
NA	NA	NA	NA	NA	stop_gaine	ENSG0000(	transcript	ENST0000C
NA	NA	NA	NA	NA	splice_acce	ENSG0000(	transcript	ENST0000C
NA	NA	NA	NA	NA	splice_acce	ENSG0000(	transcript	ENST0000C
NA	NA	NA	NA	NA	frameshift_	ENSG0000(	transcript	ENST0000C

NA	NA	NA	NA	NA	frameshift_	ENSG0000(	transcript	ENST0000C
NA	NA	NA	NA	NA	frameshift_	ENSG0000(	transcript	ENST0000C
NA	NA	NA	NA	NA	frameshift_	ENSG0000(	transcript	ENST0000C
NA	NA	NA	NA	NA	stop_gaine	ENSG0000(	transcript	ENST0000C
NA	NA	NA	NA	NA	splice_acce	ENSG0000(	transcript	ENST0000C
NA	Pathogenic	DEAFNESS,	0.00082	NA	frameshift_	ENSG0000(	transcript	ENST0000C
NA	Benign	not_provid	NA	NA	frameshift_	ENSG0000(	transcript	ENST0000C
NA	Benign	not_provid	NA	NA	frameshift_	ENSG0000(	transcript	ENST0000C
NA	NA	NA	NA	NA	splice_acce	ENSG0000(	transcript	ENST0000C
NA	NA	NA	NA	NA	structural_	ENSG0000(	interaction	3UN9:A_79
NA	Benign/Like	Rasopathy	0.00495	0.01098	structural_	ENSG0000(	interaction	1B47:B_10
NA	NA	NA	NA	NA	structural_	ENSG0000(	interaction	2IBI:A_346
NA	NA	NA	NA	NA	splice_don	ENSG0000(	transcript	ENST0000C
NA	NA	NA	NA	NA	splice_don	ENSG0000(	transcript	ENST0000C
NA	NA	NA	NA	NA	structural_	ENSG0000(	interaction	3WSX:A_24
NA	NA	NA	NA	NA	structural_	ENSG0000(	interaction	3WSX:A_68
NA	NA	NA	NA	NA	splice_don	ENSG0000(	transcript	ENST0000C
NA	NA	NA	NA	NA	start_lost	ENSG0000(	transcript	ENST0000C
NA	NA	NA	NA	NA	stop_gaine	ENSG0000(	transcript	ENST0000C
NA	NA	NA	NA	NA	stop_gaine	ENSG0000(	transcript	ENST0000C
NA	NA	NA	NA	NA	frameshift_	ENSG0000(	transcript	ENST0000C
NA	NA	NA	NA	NA	stop_gaine	ENSG0000(	transcript	ENST0000C
NA	NA	NA	NA	NA	splice_acce	ENSG0000(	transcript	ENST0000C
NA	NA	NA	NA	NA	splice_don	ENSG0000(	transcript	ENST0000C
NA	NA	NA	NA	NA	stop_gaine	ENSG0000(	transcript	ENST0000C
NA	NA	NA	NA	NA	stop_gaine	ENSG0000(	transcript	ENST0000C
NA	NA	NA	NA	NA	splice_acce	ENSG0000(	transcript	ENST0000C
NA	NA	NA	NA	NA	splice_acce	ENSG0000(	transcript	ENST0000C
NA	Benign/Like	Holoproser	0.01867	0.01957	protein_pr	ENSG0000(	interaction	3N1F:B_90
NA	protective	Mycobacte	0.12534	0.08586	structural_	ENSG0000(	interaction	4LQD:A_14
NA	NA	NA	NA	NA	splice_acce	ENSG0000(	transcript	ENST0000C
NA	NA	NA	NA	NA	splice_acce	ENSG0000(	transcript	ENST0000C
NA	NA	NA	NA	NA	splice_acce	ENSG0000(	transcript	ENST0000C
NA	NA	NA	NA	NA	structural_	ENSG0000(	interaction	1XMM:C_4
NA	NA	NA	NA	NA	splice_acce	ENSG0000(	transcript	ENST0000C
NA	NA	NA	NA	NA	structural_	ENSG0000(	interaction	3MFK:A_33
NA	NA	NA	NA	NA	stop_gaine	ENSG0000(	transcript	ENST0000C
NA	NA	NA	NA	NA	splice_acce	ENSG0000(	transcript	ENST0000C
NA	NA	NA	NA	NA	splice_acce	ENSG0000(	transcript	ENST0000C
NA	NA	NA	NA	NA	splice_acce	ENSG0000(	transcript	ENST0000C
NA	NA	NA	NA	NA	splice_acce	ENSG0000(	transcript	ENST0000C
NA	NA	NA	NA	NA	splice_acce	ENSG0000(	transcript	ENST0000C
NA	NA	NA	NA	NA	splice_don	ENSG0000(	transcript	ENST0000C
NA	NA	NA	NA	NA	splice_don	ENSG0000(	transcript	ENST0000C
NA	NA	NA	NA	NA	splice_don	ENSG0000(	transcript	ENST0000C
NA	NA	NA	NA	NA	splice_don	ENSG0000(	transcript	ENST0000C
NA	NA	NA	NA	NA	frameshift_	ENSG0000(	transcript	ENST0000C

NA	NA	NA	NA	NA	stop_gaine	ENSG0000(	transcript	ENST0000C
NA	NA	NA	NA	NA	frameshift_	ENSG0000(	transcript	ENST0000C
NA	NA	NA	NA	NA	frameshift_	ENSG0000(	transcript	ENST0000C
NA	NA	NA	NA	NA	frameshift_	ENSG0000(	transcript	ENST0000C
NA	Likely_beni	not_specifi	0.02945	0.02216	stop_gaine	ENSG0000(	transcript	ENST0000C
NA	NA	NA	NA	NA	stop_gaine	ENSG0000(	transcript	ENST0000C
NA	NA	NA	NA	NA	frameshift_	ENSG0000(	transcript	ENST0000C
NA	Benign	Long_QT_s	NA	NA	start_lost	ENSG0000(	transcript	ENST0000C
Heel_bone_	NA	NA	NA	NA	structural_	ENSG0000(	interaction	1QZ2:A_19
NA	NA	NA	NA	NA	splice_don	ENSG0000(	transcript	ENST0000C
NA	NA	NA	NA	NA	splice_don	ENSG0000(	transcript	ENST0000C
NA	NA	NA	NA	NA	frameshift_	ENSG0000(	transcript	ENST0000C
NA	NA	NA	NA	NA	frameshift_	ENSG0000(	transcript	ENST0000C
NA	NA	NA	NA	NA	frameshift_	ENSG0000(	transcript	ENST0000C
NA	NA	NA	NA	NA	frameshift_	ENSG0000(	transcript	ENST0000C
NA	Benign/Like	von_Willeb	0.68522	0.77656	structural_	ENSG0000(	interaction	3GXB:A_15
NA	NA	NA	NA	NA	splice_don	ENSG0000(	transcript	ENST0000C
NA	NA	NA	NA	NA	frameshift_	ENSG0000(	transcript	ENST0000C
NA	NA	NA	NA	NA	structural_	ENSG0000(	interaction	1U8F:O_10
NA	NA	NA	NA	NA	structural_	ENSG0000(	interaction	1U8F:O_28
NA	NA	NA	NA	NA	structural_	ENSG0000(	interaction	1U8F:O_27
NA	NA	NA	NA	NA	splice_don	ENSG0000(	transcript	ENST0000C
NA	NA	NA	NA	NA	splice_don	ENSG0000(	transcript	ENST0000C
NA	NA	NA	NA	NA	structural_	ENSG0000(	interaction	3IHP:A_59-
NA	Likely_beni	Triosephos	0.01599	0.02117	splice_acce	ENSG0000(	transcript	ENST0000C
Colorectal_	NA	NA	NA	NA	splice_acce	ENSG0000(	transcript	ENST0000C
NA	NA	NA	NA	NA	splice_don	ENSG0000(	transcript	ENST0000C
NA	NA	NA	NA	NA	structural_	ENSG0000(	interaction	2B3O:A_32
NA	NA	NA	NA	NA	frameshift_	ENSG0000(	transcript	ENST0000C
NA	NA	NA	NA	NA	splice_don	ENSG0000(	transcript	ENST0000C
NA	NA	NA	NA	NA	stop_gaine	ENSG0000(	transcript	ENST0000C
NA	NA	NA	NA	NA	frameshift_	ENSG0000(	transcript	ENST0000C
NA	NA	NA	NA	NA	frameshift_	ENSG0000(	transcript	ENST0000C
NA	NA	NA	NA	NA	stop_gaine	ENSG0000(	transcript	ENST0000C
NA	NA	NA	NA	NA	frameshift_	ENSG0000(	transcript	ENST0000C
NA	NA	NA	NA	NA	frameshift_	ENSG0000(	transcript	ENST0000C
NA	NA	NA	NA	NA	splice_don	ENSG0000(	transcript	ENST0000C
NA	Benign	not_provid	NA	NA	splice_don	ENSG0000(	transcript	ENST0000C
NA	NA	NA	NA	NA	splice_don	ENSG0000(	transcript	ENST0000C
NA	NA	NA	NA	NA	splice_don	ENSG0000(	transcript	ENST0000C
NA	Benign	not_specifi	0.02143	0.02596	splice_acce	ENSG0000(	transcript	ENST0000C
NA	Benign	not_specifi	0.04442	0.02696	frameshift_	ENSG0000(	transcript	ENST0000C
NA	Benign	ALPHA-2-M	NA	NA	splice_acce	ENSG0000(	transcript	ENST0000C
NA	NA	NA	NA	NA	stop_gaine	ENSG0000(	transcript	ENST0000C
NA	NA	NA	NA	NA	splice_don	ENSG0000(	transcript	ENST0000C
NA	NA	NA	NA	NA	splice_don	ENSG0000(	transcript	ENST0000C
NA	NA	NA	NA	NA	splice_don	ENSG0000(	transcript	ENST0000C

NA	NA	NA	NA	NA	splice_don	ENSG0000(	transcript	ENST0000C
NA	NA	NA	NA	NA	splice_acce	ENSG0000(	transcript	ENST0000C
NA	NA	NA	NA	NA	splice_acce	ENSG0000(	transcript	ENST0000C
NA	NA	NA	NA	NA	frameshift_	ENSG0000(	transcript	ENST0000C
NA	Benign	Familial_ch	NA	0.04093	stop_gaine	ENSG0000(	transcript	ENST0000C
NA	NA	NA	NA	NA	splice_acce	ENSG0000(	transcript	ENST0000C
NA	NA	NA	NA	NA	structural_	ENSG0000(	interaction	4A0P:A_10
NA	NA	NA	NA	NA	structural_	ENSG0000(	interaction	3S94:B_42(
NA	NA	NA	NA	NA	frameshift_	ENSG0000(	transcript	ENST0000C
NA	NA	NA	NA	NA	frameshift_	ENSG0000(	transcript	ENST0000C
NA	NA	NA	NA	NA	splice_acce	ENSG0000(	transcript	ENST0000C
NA	NA	NA	NA	NA	splice_don	ENSG0000(	transcript	ENST0000C
NA	NA	NA	NA	NA	splice_don	ENSG0000(	transcript	ENST0000C
NA	NA	NA	NA	NA	stop_gaine	ENSG0000(	transcript	ENST0000C
NA	NA	NA	NA	NA	splice_don	ENSG0000(	transcript	ENST0000C
NA	NA	NA	NA	NA	frameshift_	ENSG0000(	transcript	ENST0000C
NA	NA	NA	NA	NA	structural_	ENSG0000(	interaction	4F9Z:A_16
NA	NA	NA	NA	NA	structural_	ENSG0000(	interaction	2GJT:A_10
NA	Likely_beni	not_provid	NA	NA	splice_don	ENSG0000(	transcript	ENST0000C
NA	NA	NA	NA	NA	stop_lost	ENSG0000(	transcript	ENST0000C
NA	Conflicting_	Rotor_sync	NA	NA	frameshift_	ENSG0000(	transcript	ENST0000C
NA	NA	NA	NA	NA	frameshift_	ENSG0000(	transcript	ENST0000C
NA	NA	NA	NA	NA	splice_acce	ENSG0000(	transcript	ENST0000C
NA	NA	NA	NA	NA	splice_acce	ENSG0000(	transcript	ENST0000C
NA	NA	NA	NA	NA	splice_don	ENSG0000(	transcript	ENST0000C
NA	NA	NA	NA	NA	splice_don	ENSG0000(	transcript	ENST0000C
NA	NA	NA	NA	NA	start_lost	ENSG0000(	transcript	ENST0000C
NA	NA	NA	NA	NA	splice_don	ENSG0000(	transcript	ENST0000C
NA	NA	NA	NA	NA	splice_don	ENSG0000(	transcript	ENST0000C
NA	NA	NA	NA	NA	splice_acce	ENSG0000(	transcript	ENST0000C
NA	NA	NA	NA	NA	splice_acce	ENSG0000(	transcript	ENST0000C
NA	NA	NA	NA	NA	splice_don	ENSG0000(	transcript	ENST0000C
NA	NA	NA	NA	NA	stop_gaine	ENSG0000(	transcript	ENST0000C
NA	NA	NA	NA	NA	splice_don	ENSG0000(	transcript	ENST0000C
NA	NA	NA	NA	NA	splice_don	ENSG0000(	transcript	ENST0000C
NA	NA	NA	NA	NA	splice_acce	ENSG0000(	transcript	ENST0000C
NA	NA	NA	NA	NA	splice_don	ENSG0000(	transcript	ENST0000C
NA	NA	NA	NA	NA	splice_don	ENSG0000(	transcript	ENST0000C
NA	NA	NA	NA	NA	splice_acce	ENSG0000(	transcript	ENST0000C
NA	NA	NA	NA	NA	start_lost&	ENSG0000(	transcript	ENST0000C
NA	Likely_beni	Progressive	NA	0.01338	splice_don	ENSG0000(	transcript	ENST0000C
NA	NA	NA	NA	NA	transcript_	ENSG0000(	transcript	ENST0000C
NA	NA	NA	NA	NA	splice_acce	ENSG0000(	transcript	ENST0000C
NA	NA	NA	NA	NA	splice_don	ENSG0000(	transcript	ENST0000C
Eosinophil_	drug_respc	peginterfer	0.63756	0.67153	start_lost	ENSG0000(	transcript	ENST0000C
NA	NA	NA	NA	NA	splice_acce	ENSG0000(	transcript	ENST0000C
NA	NA	NA	NA	NA	stop_gaine	ENSG0000(	transcript	ENST0000C

NA	NA	NA	NA	NA	frameshift_	ENSG0000(	transcript	ENST0000C
NA	NA	NA	NA	NA	splice_acce	ENSG0000(	transcript	ENST0000C
NA	NA	NA	NA	NA	splice_acce	ENSG0000(	transcript	ENST0000C
NA	NA	NA	NA	NA	splice_acce	ENSG0000(	transcript	ENST0000C
NA	NA	NA	NA	NA	splice_don	ENSG0000(	transcript	ENST0000C
NA	NA	NA	NA	NA	stop_gaine	ENSG0000(	transcript	ENST0000C
NA	Uncertain_	46,XY_DSD	0.01103	0.00779	protein_pr	ENSG0000(	interaction	2WG3:A_1
NA	NA	NA	NA	NA	splice_don	ENSG0000(	transcript	ENST0000C
NA	Benign	not_specifi	0.75927	0.66713	structural_	ENSG0000(	interaction	4NEF:A_14
NA	NA	NA	NA	NA	frameshift_	ENSG0000(	transcript	ENST0000C
NA	NA	NA	NA	NA	frameshift_	ENSG0000(	transcript	ENST0000C
NA	NA	NA	NA	NA	frameshift_	ENSG0000(	transcript	ENST0000C
NA	NA	NA	NA	NA	stop_lost	ENSG0000(	transcript	ENST0000C
NA	NA	NA	NA	NA	frameshift_	ENSG0000(	transcript	ENST0000C
NA	NA	NA	NA	NA	frameshift_	ENSG0000(	transcript	ENST0000C
NA	NA	NA	NA	NA	frameshift_	ENSG0000(	transcript	ENST0000C
NA	NA	NA	NA	NA	frameshift_	ENSG0000(	transcript	ENST0000C
NA	NA	NA	NA	NA	splice_don	ENSG0000(	transcript	ENST0000C
NA	NA	NA	NA	NA	splice_don	ENSG0000(	transcript	ENST0000C
NA	Benign	Beaded_ha	NA	0.37620	stop_gaine	ENSG0000(	transcript	ENST0000C
NA	Benign	Beaded_ha	NA	0.37620	synonymou	ENSG0000(	transcript	ENST0000C
NA	Benign/Like	Beaded_ha	0.01186	0.00739	stop_gaine	ENSG0000(	transcript	ENST0000C
NA	NA	NA	NA	NA	stop_gaine	ENSG0000(	transcript	ENST0000C
NA	NA	NA	NA	NA	splice_don	ENSG0000(	transcript	ENST0000C
NA	Benign	not_provid	NA	NA	structural_	ENSG0000(	interaction	3HQC:A_12
NA	NA	NA	NA	NA	structural_	ENSG0000(	interaction	2JIS:A_283
NA	NA	NA	NA	NA	frameshift_	ENSG0000(	transcript	ENST0000C
NA	NA	NA	NA	NA	splice_don	ENSG0000(	transcript	ENST0000C
NA	NA	NA	NA	NA	splice_acce	ENSG0000(	transcript	ENST0000C
NA	NA	NA	NA	NA	start_lost	ENSG0000(	transcript	ENST0000C
NA	NA	NA	NA	NA	splice_don	ENSG0000(	transcript	ENST0000C
NA	NA	NA	NA	NA	structural_	ENSG0000(	interaction	1TAZ:A_37
NA	NA	NA	NA	NA	frameshift_	ENSG0000(	transcript	ENST0000C
NA	NA	NA	NA	NA	splice_acce	ENSG0000(	transcript	ENST0000C
NA	NA	NA	NA	NA	stop_gaine	ENSG0000(	transcript	ENST0000C
NA	NA	NA	NA	NA	frameshift_	ENSG0000(	transcript	ENST0000C
NA	NA	NA	NA	NA	stop_gaine	ENSG0000(	transcript	ENST0000C
NA	NA	NA	NA	NA	frameshift_	ENSG0000(	transcript	ENST0000C
NA	NA	NA	NA	NA	frameshift_	ENSG0000(	transcript	ENST0000C
NA	NA	NA	NA	NA	protein_pr	ENSG0000(	interaction	1H1P:A_28
NA	NA	NA	NA	NA	protein_pr	ENSG0000(	interaction	2GWF:A_2
NA	NA	NA	NA	NA	start_lost	ENSG0000(	transcript	ENST0000C
NA	Benign	not_provid	NA	NA	frameshift_	ENSG0000(	transcript	ENST0000C
NA	NA	NA	NA	NA	structural_	ENSG0000(	interaction	4BPU:A_33
NA	NA	NA	NA	NA	frameshift_	ENSG0000(	transcript	ENST0000C
NA	NA	NA	NA	NA	stop_gaine	ENSG0000(	transcript	ENST0000C
NA	NA	NA	NA	NA	start_lost	ENSG0000(	transcript	ENST0000C

NA	NA	NA	NA	NA	splice_don	ENSG0000(	transcript	ENST0000C
NA	NA	NA	NA	NA	structural_	ENSG0000(	interaction	4QPN:A_17
NA	NA	NA	NA	NA	splice_don	ENSG0000(	transcript	ENST0000C
NA	NA	NA	NA	NA	splice_don	ENSG0000(	transcript	ENST0000C
NA	NA	NA	NA	NA	frameshift_	ENSG0000(	transcript	ENST0000C
NA	NA	NA	NA	NA	splice_acce	ENSG0000(	transcript	ENST0000C
NA	NA	NA	NA	NA	splice_acce	ENSG0000(	transcript	ENST0000C
NA	NA	NA	NA	NA	splice_acce	ENSG0000(	transcript	ENST0000C
NA	NA	NA	NA	NA	splice_acce	ENSG0000(	transcript	ENST0000C
NA	NA	NA	NA	NA	splice_don	ENSG0000(	transcript	ENST0000C
NA	NA	NA	NA	NA	splice_acce	ENSG0000(	transcript	ENST0000C
NA	NA	NA	NA	NA	splice_acce	ENSG0000(	transcript	ENST0000C
NA	NA	NA	NA	NA	splice_acce	ENSG0000(	transcript	ENST0000C
NA	NA	NA	NA	NA	splice_regi	ENSG0000(	transcript	ENST0000C
NA	NA	NA	NA	NA	structural_	ENSG0000(	interaction	2YGN:A_1C
NA	NA	NA	NA	NA	frameshift_	ENSG0000(	transcript	ENST0000C
NA	NA	NA	NA	NA	splice_acce	ENSG0000(	transcript	ENST0000C
NA	NA	NA	NA	NA	protein_pr	ENSG0000(	interaction	3P5T:A_15
NA	NA	NA	NA	NA	frameshift_	ENSG0000(	transcript	ENST0000C
NA	NA	NA	NA	NA	splice_don	ENSG0000(	transcript	ENST0000C
NA	NA	NA	NA	NA	splice_acce	ENSG0000(	transcript	ENST0000C
NA	NA	NA	NA	NA	splice_acce	ENSG0000(	transcript	ENST0000C
NA	NA	NA	NA	NA	structural_	ENSG0000(	interaction	2AHS:A_18
NA	NA	NA	NA	NA	structural_	ENSG0000(	interaction	2AHS:A_17
NA	NA	NA	NA	NA	structural_	ENSG0000(	interaction	4KNG:B_59
NA	Likely_beni	not_provid	NA	NA	structural_	ENSG0000(	interaction	4KNG:A_19
NA	NA	NA	NA	NA	structural_	ENSG0000(	interaction	4KNG:A_27
NA	NA	NA	NA	NA	splice_don	ENSG0000(	transcript	ENST0000C
NA	Benign	not_provid	NA	NA	structural_	ENSG0000(	interaction	4KNG:A_4C
NA	Benign	not_provid	NA	NA	structural_	ENSG0000(	interaction	4KNG:A_4C
NA	Likely_beni	Tryptophar	0.57319	0.48722	structural_	ENSG0000(	interaction	4V06:A_23
NA	NA	NA	NA	NA	splice_acce	ENSG0000(	transcript	ENST0000C
NA	NA	NA	NA	NA	frameshift_	ENSG0000(	transcript	ENST0000C
NA	NA	NA	NA	NA	splice_acce	ENSG0000(	transcript	ENST0000C
NA	NA	NA	NA	NA	splice_acce	ENSG0000(	transcript	ENST0000C
NA	NA	NA	NA	NA	splice_acce	ENSG0000(	transcript	ENST0000C
NA	NA	NA	NA	NA	splice_acce	ENSG0000(	transcript	ENST0000C
NA	NA	NA	NA	NA	splice_don	ENSG0000(	transcript	ENST0000C
NA	NA	NA	NA	NA	splice_don	ENSG0000(	transcript	ENST0000C
NA	NA	NA	NA	NA	structural_	ENSG0000(	interaction	4V11:A_35
NA	NA	NA	NA	NA	frameshift_	ENSG0000(	transcript	ENST0000C
NA	NA	NA	NA	NA	splice_acce	ENSG0000(	transcript	ENST0000C
NA	NA	NA	NA	NA	start_lost	ENSG0000(	transcript	ENST0000C
NA	NA	NA	NA	NA	splice_acce	ENSG0000(	transcript	ENST0000C
NA	NA	NA	NA	NA	splice_acce	ENSG0000(	transcript	ENST0000C
NA	NA	NA	NA	NA	splice_don	ENSG0000(	transcript	ENST0000C
NA	NA	NA	NA	NA	splice_don	ENSG0000(	transcript	ENST0000C

NA	NA	NA	NA	NA	splice_don	ENSG0000(	transcript	ENST0000C
NA	NA	NA	NA	NA	splice_acce	ENSG0000(	transcript	ENST0000C
NA	NA	NA	NA	NA	structural_	ENSG0000(	interaction	3NVN:B_23
NA	Benign	not_provid	NA	NA	splice_acce	ENSG0000(	transcript	ENST0000C
NA	Benign	not_provid	NA	NA	splice_acce	ENSG0000(	transcript	ENST0000C
NA	NA	NA	NA	NA	splice_don	ENSG0000(	transcript	ENST0000C
NA	NA	NA	NA	NA	splice_don	ENSG0000(	transcript	ENST0000C
NA	NA	NA	NA	NA	structural_	ENSG0000(	interaction	1B59:A_13
NA	NA	NA	NA	NA	structural_	ENSG0000(	interaction	1B59:A_28
NA	NA	NA	NA	NA	splice_don	ENSG0000(	transcript	ENST0000C
NA	NA	NA	NA	NA	splice_acce	ENSG0000(	transcript	ENST0000C
NA	NA	NA	NA	NA	splice_don	ENSG0000(	transcript	ENST0000C
NA	NA	NA	NA	NA	splice_don	ENSG0000(	transcript	ENST0000C
NA	NA	NA	NA	NA	frameshift_	ENSG0000(	transcript	ENST0000C
NA	Benign	Phenylketo	0.29058	0.31490	structural_	ENSG0000(	interaction	1DMW:A_2
NA	NA	NA	NA	NA	bidirection	ENSG0000(	gene_varia	ENSG0000C
NA	NA	NA	NA	NA	bidirection	ENSG0000(	gene_varia	ENSG0000C
NA	NA	NA	NA	NA	bidirection	ENSG0000(	gene_varia	ENSG0000C
NA	NA	NA	NA	NA	bidirection	ENSG0000(	gene_varia	ENSG0000C
NA	NA	NA	NA	NA	splice_don	ENSG0000(	transcript	ENST0000C
NA	NA	NA	NA	NA	splice_don	ENSG0000(	transcript	ENST0000C
NA	NA	NA	NA	NA	splice_don	ENSG0000(	transcript	ENST0000C
NA	NA	NA	NA	NA	splice_don	ENSG0000(	transcript	ENST0000C
NA	NA	NA	NA	NA	splice_don	ENSG0000(	transcript	ENST0000C
NA	NA	NA	NA	NA	splice_don	ENSG0000(	transcript	ENST0000C
NA	NA	NA	NA	NA	splice_don	ENSG0000(	transcript	ENST0000C
NA	NA	NA	NA	NA	splice_don	ENSG0000(	transcript	ENST0000C
NA	NA	NA	NA	NA	splice_don	ENSG0000(	transcript	ENST0000C
NA	NA	NA	NA	NA	splice_acce	ENSG0000(	transcript	ENST0000C
NA	NA	NA	NA	NA	splice_acce	ENSG0000(	transcript	ENST0000C
NA	NA	NA	NA	NA	splice_acce	ENSG0000(	transcript	ENST0000C
NA	NA	NA	NA	NA	frameshift_	ENSG0000(	transcript	ENST0000C
NA	Benign	not_provid	NA	NA	structural_	ENSG0000(	interaction	4U07:A_14
NA	Benign	not_provid	NA	NA	frameshift_	ENSG0000(	transcript	ENST0000C
NA	NA	NA	NA	NA	structural_	ENSG0000(	interaction	2DU8:A_93
NA	NA	NA	NA	NA	structural_	ENSG0000(	interaction	3BTY:A_17
NA	NA	NA	NA	NA	structural_	ENSG0000(	interaction	2HJW:A_59
NA	NA	NA	NA	NA	structural_	ENSG0000(	interaction	2HJW:A_65
NA	NA	NA	NA	NA	stop_gaine	ENSG0000(	transcript	ENST0000C
NA	NA	NA	NA	NA	stop_gaine	ENSG0000(	transcript	ENST0000C
NA	Benign/Like	Methylmal	0.10167	0.07588	structural_	ENSG0000(	interaction	2R3V:A_52
NA	Benign/Like	Hyperimmu	0.04696	0.03215	structural_	ENSG0000(	interaction	2R3V:A_37
NA	Benign	Spondylom	0.07464	0.10503	structural_	ENSG0000(	interaction	4DX1:A_26
NA	Benign	Spondylom	0.11347	0.15635	structural_	ENSG0000(	interaction	4DX1:A_22
NA	NA	NA	NA	NA	splice_don	ENSG0000(	transcript	ENST0000C
NA	NA	NA	NA	NA	structural_	ENSG0000(	interaction	1IT6:B_64-
NA	NA	NA	NA	NA	frameshift_	ENSG0000(	transcript	ENST0000C

NA	NA	NA	NA	NA	frameshift_	ENSG0000(	transcript	ENST0000C
NA	NA	NA	NA	NA	frameshift_	ENSG0000(	transcript	ENST0000C
NA	NA	NA	NA	NA	frameshift_	ENSG0000(	transcript	ENST0000C
NA	NA	NA	NA	NA	splice_acce	ENSG0000(	transcript	ENST0000C
NA	NA	NA	NA	NA	stop_gaine	ENSG0000(	transcript	ENST0000C
NA	NA	NA	NA	NA	splice_acce	ENSG0000(	transcript	ENST0000C
NA	NA	NA	NA	NA	frameshift_	ENSG0000(	transcript	ENST0000C
NA	NA	NA	NA	NA	structural_	ENSG0000(	interaction	4S3N:A_65
NA	NA	NA	NA	NA	stop_gaine	ENSG0000(	transcript	ENST0000C
NA	NA	NA	NA	NA	stop_lost	ENSG0000(	transcript	ENST0000C
NA	NA	NA	NA	NA	splice_don	ENSG0000(	transcript	ENST0000C
NA	NA	NA	NA	NA	splice_don	ENSG0000(	transcript	ENST0000C
NA	NA	NA	NA	NA	frameshift_	ENSG0000(	transcript	ENST0000C
NA	NA	NA	NA	NA	frameshift_	ENSG0000(	transcript	ENST0000C
NA	Likely_beni	not_provid	NA	NA	structural_	ENSG0000(	interaction	4D1N:B_35
NA	NA	NA	NA	NA	splice_don	ENSG0000(	transcript	ENST0000C
NA	NA	NA	NA	NA	frameshift_	ENSG0000(	transcript	ENST0000C
NA	NA	NA	NA	NA	stop_gaine	ENSG0000(	transcript	ENST0000C
NA	NA	NA	NA	NA	frameshift_	ENSG0000(	transcript	ENST0000C
NA	NA	NA	NA	NA	frameshift_	ENSG0000(	transcript	ENST0000C
NA	NA	NA	NA	NA	frameshift_	ENSG0000(	transcript	ENST0000C
NA	NA	NA	NA	NA	frameshift_	ENSG0000(	transcript	ENST0000C
NA	NA	NA	NA	NA	frameshift_	ENSG0000(	transcript	ENST0000C
NA	Benign	Deficiency_	0.57060	0.59066	structural_	ENSG0000(	interaction	2VIG:B_10
NA	Benign/Like	Deficiency_	0.05600	0.07608	structural_	ENSG0000(	interaction	2VIG:A_79-
Cardiovasc	NA	NA	NA	NA	structural_	ENSG0000(	interaction	4XQ7:A_10
NA	NA	NA	NA	NA	frameshift_	ENSG0000(	transcript	ENST0000C
NA	NA	NA	NA	NA	frameshift_	ENSG0000(	transcript	ENST0000C
NA	NA	NA	NA	NA	frameshift_	ENSG0000(	transcript	ENST0000C
NA	NA	NA	NA	NA	frameshift_	ENSG0000(	transcript	ENST0000C
NA	NA	NA	NA	NA	splice_don	ENSG0000(	transcript	ENST0000C
NA	Likely_beni	not_provid	NA	NA	frameshift_	ENSG0000(	transcript	ENST0000C
NA	NA	NA	NA	NA	frameshift_	ENSG0000(	transcript	ENST0000C
NA	NA	NA	NA	NA	frameshift_	ENSG0000(	transcript	ENST0000C
NA	NA	NA	NA	NA	splice_acce	ENSG0000(	transcript	ENST0000C
NA	NA	NA	NA	NA	structural_	ENSG0000(	interaction	2E3I:A_84-
NA	NA	NA	NA	NA	splice_don	ENSG0000(	transcript	ENST0000C
NA	NA	NA	NA	NA	splice_don	ENSG0000(	transcript	ENST0000C
NA	NA	NA	NA	NA	splice_don	ENSG0000(	transcript	ENST0000C
NA	NA	NA	NA	NA	splice_don	ENSG0000(	transcript	ENST0000C
NA	NA	NA	NA	NA	splice_don	ENSG0000(	transcript	ENST0000C
NA	NA	NA	NA	NA	splice_acce	ENSG0000(	transcript	ENST0000C
NA	NA	NA	NA	NA	stop_gaine	ENSG0000(	transcript	ENST0000C
NA	NA	NA	NA	NA	splice_don	ENSG0000(	transcript	ENST0000C
NA	Conflicting_	Mitochond	0.00260	0.00020	structural_	ENSG0000(	interaction	4IQM:A_32
NA	NA	NA	NA	NA	frameshift_	ENSG0000(	transcript	ENST0000C
NA	NA	NA	NA	NA	frameshift_	ENSG0000(	transcript	ENST0000C

NA	NA	NA	NA	NA	frameshift_	ENSG0000(	transcript	ENST0000C
NA	NA	NA	NA	NA	frameshift_	ENSG0000(	transcript	ENST0000C
NA	NA	NA	NA	NA	splice_acce	ENSG0000(	transcript	ENST0000C
NA	NA	NA	NA	NA	frameshift_	ENSG0000(	transcript	ENST0000C
NA	NA	NA	NA	NA	stop_lost	ENSG0000(	transcript	ENST0000C
NA	NA	NA	NA	NA	splice_don	ENSG0000(	transcript	ENST0000C
NA	NA	NA	NA	NA	splice_don	ENSG0000(	transcript	ENST0000C
NA	NA	NA	NA	NA	splice_don	ENSG0000(	transcript	ENST0000C
NA	NA	NA	NA	NA	splice_acce	ENSG0000(	transcript	ENST0000C
NA	NA	NA	NA	NA	splice_acce	ENSG0000(	transcript	ENST0000C
NA	NA	NA	NA	NA	splice_don	ENSG0000(	transcript	ENST0000C
NA	NA	NA	NA	NA	splice_acce	ENSG0000(	transcript	ENST0000C
NA	NA	NA	NA	NA	splice_acce	ENSG0000(	transcript	ENST0000C
NA	NA	NA	NA	NA	splice_acce	ENSG0000(	transcript	ENST0000C
NA	NA	NA	NA	NA	stop_gaine	ENSG0000(	transcript	ENST0000C
NA	NA	NA	NA	NA	frameshift_	ENSG0000(	transcript	ENST0000C
NA	Pathogenic	Hearing_im	NA	NA	frameshift_	ENSG0000(	transcript	ENST0000C
NA	NA	NA	NA	NA	structural_	ENSG0000(	interaction	3F3S:A_7-A
NA	NA	NA	NA	NA	frameshift_	ENSG0000(	transcript	ENST0000C
NA	NA	NA	NA	NA	frameshift_	ENSG0000(	transcript	ENST0000C
NA	NA	NA	NA	NA	splice_acce	ENSG0000(	transcript	ENST0000C
NA	NA	NA	NA	NA	splice_acce	ENSG0000(	transcript	ENST0000C
NA	NA	NA	NA	NA	splice_acce	ENSG0000(	transcript	ENST0000C
NA	NA	NA	NA	NA	splice_acce	ENSG0000(	transcript	ENST0000C
NA	NA	NA	NA	NA	splice_acce	ENSG0000(	transcript	ENST0000C
NA	NA	NA	NA	NA	splice_acce	ENSG0000(	transcript	ENST0000C
NA	NA	NA	NA	NA	splice_acce	ENSG0000(	transcript	ENST0000C
NA	NA	NA	NA	NA	frameshift_	ENSG0000(	transcript	ENST0000C
NA	NA	NA	NA	NA	splice_acce	ENSG0000(	transcript	ENST0000C
NA	NA	NA	NA	NA	splice_acce	ENSG0000(	transcript	ENST0000C
NA	NA	NA	NA	NA	stop_gaine	ENSG0000(	transcript	ENST0000C
NA	NA	NA	NA	NA	splice_acce	ENSG0000(	transcript	ENST0000C
NA	NA	NA	NA	NA	splice_don	ENSG0000(	transcript	ENST0000C
NA	NA	NA	NA	NA	frameshift_	ENSG0000(	transcript	ENST0000C
NA	NA	NA	NA	NA	splice_acce	ENSG0000(	transcript	ENST0000C
NA	NA	NA	NA	NA	splice_acce	ENSG0000(	transcript	ENST0000C
NA	NA	NA	NA	NA	splice_don	ENSG0000(	transcript	ENST0000C
NA	NA	NA	NA	NA	splice_don	ENSG0000(	transcript	ENST0000C
NA	NA	NA	NA	NA	splice_acce	ENSG0000(	transcript	ENST0000C
NA	NA	NA	NA	NA	splice_acce	ENSG0000(	transcript	ENST0000C
NA	NA	NA	NA	NA	splice_acce	ENSG0000(	transcript	ENST0000C
NA	NA	NA	NA	NA	splice_don	ENSG0000(	transcript	ENST0000C
NA	NA	NA	NA	NA	splice_don	ENSG0000(	transcript	ENST0000C
NA	NA	NA	NA	NA	splice_acce	ENSG0000(	transcript	ENST0000C
NA	NA	NA	NA	NA	splice_acce	ENSG0000(	transcript	ENST0000C
NA	NA	NA	NA	NA	splice_acce	ENSG0000(	transcript	ENST0000C
NA	NA	NA	NA	NA	splice_acce	ENSG0000(	transcript	ENST0000C

NA	NA	NA	NA	NA	splice_acce	ENSG0000(	transcript	ENST0000C
Breast_can	Benign	Hereditary_	0.00702	0.00439	stop_gaine	ENSG0000(	transcript	ENST0000C
NA	NA	NA	NA	NA	stop_gaine	ENSG0000(	transcript	ENST0000C
NA	NA	NA	NA	NA	splice_acce	ENSG0000(	transcript	ENST0000C
NA	NA	NA	NA	NA	splice_acce	ENSG0000(	transcript	ENST0000C
NA	NA	NA	NA	NA	splice_acce	ENSG0000(	transcript	ENST0000C
NA	Benign/Like	Congenital_	NA	NA	splice_acce	ENSG0000(	transcript	ENST0000C
NA	NA	NA	NA	NA	splice_acce	ENSG0000(	transcript	ENST0000C
NA	NA	NA	NA	NA	splice_acce	ENSG0000(	transcript	ENST0000C
NA	NA	NA	NA	NA	splice_acce	ENSG0000(	transcript	ENST0000C
NA	NA	NA	NA	NA	splice_acce	ENSG0000(	transcript	ENST0000C
NA	NA	NA	NA	NA	splice_acce	ENSG0000(	transcript	ENST0000C
NA	NA	NA	NA	NA	splice_acce	ENSG0000(	transcript	ENST0000C
NA	NA	NA	NA	NA	splice_don	ENSG0000(	transcript	ENST0000C
NA	Likely_beni	Osteopetro	0.02182	0.04193	structural_	ENSG0000(	interaction	3URF:A_16
NA	NA	NA	NA	NA	frameshift_	ENSG0000(	transcript	ENST0000C
NA	NA	NA	NA	NA	splice_acce	ENSG0000(	transcript	ENST0000C
NA	NA	NA	NA	NA	splice_acce	ENSG0000(	transcript	ENST0000C
NA	NA	NA	NA	NA	stop_gaine	ENSG0000(	transcript	ENST0000C
NA	NA	NA	NA	NA	stop_gaine	ENSG0000(	transcript	ENST0000C
NA	NA	NA	NA	NA	structural_	ENSG0000(	interaction	4P10:A_15
NA	NA	NA	NA	NA	frameshift_	ENSG0000(	transcript	ENST0000C
NA	NA	NA	NA	NA	start_lost	ENSG0000(	transcript	ENST0000C
NA	Benign	not_provid	NA	NA	stop_gaine	ENSG0000(	transcript	ENST0000C
NA	NA	NA	NA	NA	bidirection	ENSG0000(	gene_varia	ENSG0000C
NA	NA	NA	NA	NA	splice_don	ENSG0000(	transcript	ENST0000C
NA	Benign/Like	Aicardi_Go	NA	NA	frameshift_	ENSG0000(	transcript	ENST0000C
NA	NA	NA	NA	NA	splice_don	ENSG0000(	transcript	ENST0000C
NA	NA	NA	NA	NA	splice_acce	ENSG0000(	transcript	ENST0000C
NA	NA	NA	NA	NA	splice_acce	ENSG0000(	transcript	ENST0000C
NA	NA	NA	NA	NA	splice_acce	ENSG0000(	transcript	ENST0000C
NA	NA	NA	NA	NA	frameshift_	ENSG0000(	transcript	ENST0000C
NA	NA	NA	NA	NA	splice_don	ENSG0000(	transcript	ENST0000C
NA	NA	NA	NA	NA	splice_don	ENSG0000(	transcript	ENST0000C
NA	NA	NA	NA	NA	frameshift_	ENSG0000(	transcript	ENST0000C
NA	NA	NA	NA	NA	splice_don	ENSG0000(	transcript	ENST0000C
NA	NA	NA	NA	NA	splice_acce	ENSG0000(	transcript	ENST0000C
NA	NA	NA	NA	NA	splice_acce	ENSG0000(	transcript	ENST0000C
NA	NA	NA	NA	NA	splice_acce	ENSG0000(	transcript	ENST0000C
NA	NA	NA	NA	NA	splice_acce	ENSG0000(	transcript	ENST0000C
NA	NA	NA	NA	NA	splice_acce	ENSG0000(	transcript	ENST0000C
NA	NA	NA	NA	NA	splice_don	ENSG0000(	transcript	ENST0000C
NA	NA	NA	NA	NA	splice_acce	ENSG0000(	transcript	ENST0000C
NA	NA	NA	NA	NA	gene_fusio	ENSG0000(	gene_varia	ENSG0000C
NA	NA	NA	NA	NA	frameshift_	ENSG0000(	transcript	ENST0000C
NA	Benign	not_provid	NA	NA	frameshift_	ENSG0000(	transcript	ENST0000C
NA	Benign	not_provid	NA	NA	frameshift_	ENSG0000(	transcript	ENST0000C

NA	NA	NA	NA	NA	splice_acce	ENSG0000(	transcript	ENST0000C
NA	NA	NA	NA	NA	splice_acce	ENSG0000(	transcript	ENST0000C
NA	NA	NA	NA	NA	splice_acce	ENSG0000(	transcript	ENST0000C
NA	NA	NA	NA	NA	splice_acce	ENSG0000(	transcript	ENST0000C
NA	NA	NA	NA	NA	splice_don	ENSG0000(	transcript	ENST0000C
NA	NA	NA	NA	NA	splice_don	ENSG0000(	transcript	ENST0000C
NA	NA	NA	NA	NA	splice_don	ENSG0000(	transcript	ENST0000C
NA	NA	NA	NA	NA	frameshift_	ENSG0000(	transcript	ENST0000C
NA	NA	NA	NA	NA	frameshift_	ENSG0000(	transcript	ENST0000C
NA	NA	NA	NA	NA	frameshift_	ENSG0000(	transcript	ENST0000C
NA	NA	NA	NA	NA	frameshift_	ENSG0000(	transcript	ENST0000C
NA	NA	NA	NA	NA	frameshift_	ENSG0000(	transcript	ENST0000C
NA	NA	NA	NA	NA	stop_lost&	ENSG0000(	transcript	ENST0000C
NA	NA	NA	NA	NA	frameshift_	ENSG0000(	transcript	ENST0000C
NA	NA	NA	NA	NA	frameshift_	ENSG0000(	transcript	ENST0000C
Vitamin_B1	NA	NA	NA	NA	stop_gaine	ENSG0000(	transcript	ENST0000C
NA	NA	NA	NA	NA	frameshift_	ENSG0000(	transcript	ENST0000C
NA	NA	NA	NA	NA	bidirection	ENSG0000(	gene_varia	ENSG0000C
NA	NA	NA	NA	NA	bidirection	ENSG0000(	gene_varia	ENSG0000C
NA	NA	NA	NA	NA	bidirection	ENSG0000(	gene_varia	ENSG0000C
NA	NA	NA	NA	NA	bidirection	ENSG0000(	gene_varia	ENSG0000C
NA	NA	NA	NA	NA	bidirection	ENSG0000(	gene_varia	ENSG0000C
NA	NA	NA	NA	NA	structural_	ENSG0000(	interaction	4MTL:A_1C
NA	NA	NA	NA	NA	splice_don	ENSG0000(	transcript	ENST0000C
NA	Benign/Like	Lig4_syndr	0.13443	0.06869	structural_	ENSG0000(	interaction	3W5O:A_5
NA	Benign	Lig4_syndr	0.17304	0.14577	structural_	ENSG0000(	interaction	3W1B:A_9-
NA	NA	NA	NA	NA	splice_acce	ENSG0000(	transcript	ENST0000C
NA	NA	NA	NA	NA	splice_acce	ENSG0000(	transcript	ENST0000C
NA	NA	NA	NA	NA	splice_don	ENSG0000(	transcript	ENST0000C
NA	NA	NA	NA	NA	splice_acce	ENSG0000(	transcript	ENST0000C
NA	NA	NA	NA	NA	splice_acce	ENSG0000(	transcript	ENST0000C
NA	NA	NA	NA	NA	splice_acce	ENSG0000(	transcript	ENST0000C
NA	NA	NA	NA	NA	frameshift_	ENSG0000(	transcript	ENST0000C
NA	NA	NA	NA	NA	frameshift_	ENSG0000(	transcript	ENST0000C
NA	NA	NA	NA	NA	frameshift_	ENSG0000(	transcript	ENST0000C
NA	NA	NA	NA	NA	splice_don	ENSG0000(	transcript	ENST0000C
NA	NA	NA	NA	NA	splice_don	ENSG0000(	transcript	ENST0000C
NA	NA	NA	NA	NA	frameshift_	ENSG0000(	transcript	ENST0000C
NA	NA	NA	NA	NA	splice_acce	ENSG0000(	transcript	ENST0000C
NA	NA	NA	NA	NA	splice_acce	ENSG0000(	transcript	ENST0000C
NA	NA	NA	NA	NA	splice_acce	ENSG0000(	transcript	ENST0000C
NA	NA	NA	NA	NA	splice_acce	ENSG0000(	transcript	ENST0000C
NA	NA	NA	NA	NA	frameshift_	ENSG0000(	transcript	ENST0000C
NA	NA	NA	NA	NA	frameshift_	ENSG0000(	transcript	ENST0000C
NA	NA	NA	NA	NA	frameshift_	ENSG0000(	transcript	ENST0000C
NA	NA	NA	NA	NA	stop_lost	ENSG0000(	transcript	ENST0000C
NA	NA	NA	NA	NA	structural_	ENSG0000(	interaction	1BIX:A_51-

NA	Likely_beni	not_provid	NA	NA	structural_	ENSG0000(	interaction	1BIX:A_50-
NA	Benign/Like	Purine-nuc	0.16640	0.17213	structural_	ENSG0000(	interaction	1M73:E_20
NA	Benign/Like	Purine-nuc	0.19324	0.20447	structural_	ENSG0000(	interaction	1M73:E_57
NA	NA	NA	NA	NA	splice_don	ENSG0000(	transcript	ENST0000C
NA	NA	NA	NA	NA	splice_don	ENSG0000(	transcript	ENST0000C
NA	NA	NA	NA	NA	splice_don	ENSG0000(	transcript	ENST0000C
NA	NA	NA	NA	NA	frameshift_	ENSG0000(	transcript	ENST0000C
NA	NA	NA	NA	NA	frameshift_	ENSG0000(	transcript	ENST0000C
NA	NA	NA	NA	NA	structural_	ENSG0000(	interaction	2XMQ:B_2
NA	NA	NA	NA	NA	stop_lost	ENSG0000(	transcript	ENST0000C
NA	NA	NA	NA	NA	frameshift_	ENSG0000(	transcript	ENST0000C
NA	NA	NA	NA	NA	stop_gaine	ENSG0000(	transcript	ENST0000C
NA	NA	NA	NA	NA	frameshift_	ENSG0000(	transcript	ENST0000C
NA	NA	NA	NA	NA	splice_acce	ENSG0000(	transcript	ENST0000C
NA	NA	NA	NA	NA	splice_acce	ENSG0000(	transcript	ENST0000C
NA	Benign	not_provid	NA	NA	frameshift_	ENSG0000(	transcript	ENST0000C
NA	NA	NA	NA	NA	frameshift_	ENSG0000(	transcript	ENST0000C
NA	NA	NA	NA	NA	downstrear	ENSG0000(	transcript	ENST0000C
NA	NA	NA	NA	NA	frameshift_	ENSG0000(	transcript	ENST0000C
NA	NA	NA	NA	NA	stop_gaine	ENSG0000(	transcript	ENST0000C
NA	Benign	Cardiomyo	0.02085	0.01458	structural_	ENSG0000(	interaction	4DB1:A_28
NA	NA	NA	NA	NA	structural_	ENSG0000(	interaction	3O4R:A_33
NA	NA	NA	NA	NA	structural_	ENSG0000(	interaction	3O4R:A_61
NA	NA	NA	NA	NA	structural_	ENSG0000(	interaction	3O4R:B_16
NA	NA	NA	NA	NA	splice_don	ENSG0000(	transcript	ENST0000C
NA	NA	NA	NA	NA	stop_gaine	ENSG0000(	transcript	ENST0000C
NA	NA	NA	NA	NA	frameshift_	ENSG0000(	transcript	ENST0000C
NA	NA	NA	NA	NA	protein_pr	ENSG0000(	interaction	4OYK:A_82
NA	NA	NA	NA	NA	structural_	ENSG0000(	interaction	4OYJ:L_96-
NA	NA	NA	NA	NA	splice_acce	ENSG0000(	transcript	ENST0000C
NA	NA	NA	NA	NA	frameshift_	ENSG0000(	transcript	ENST0000C
NA	NA	NA	NA	NA	splice_don	ENSG0000(	transcript	ENST0000C
NA	NA	NA	NA	NA	frameshift_	ENSG0000(	transcript	ENST0000C
NA	NA	NA	NA	NA	frameshift_	ENSG0000(	transcript	ENST0000C
NA	NA	NA	NA	NA	frameshift_	ENSG0000(	transcript	ENST0000C
Mean_corp	NA	NA	NA	NA	structural_	ENSG0000(	interaction	2A7R:A_55
NA	NA	NA	NA	NA	splice_don	ENSG0000(	transcript	ENST0000C
NA	NA	NA	NA	NA	splice_don	ENSG0000(	transcript	ENST0000C
NA	NA	NA	NA	NA	splice_acce	ENSG0000(	transcript	ENST0000C
NA	NA	NA	NA	NA	splice_acce	ENSG0000(	transcript	ENST0000C
NA	NA	NA	NA	NA	splice_acce	ENSG0000(	transcript	ENST0000C
NA	NA	NA	NA	NA	splice_don	ENSG0000(	transcript	ENST0000C
NA	Likely_beni	not_specifi	NA	NA	splice_acce	ENSG0000(	transcript	ENST0000C
NA	NA	NA	NA	NA	splice_acce	ENSG0000(	transcript	ENST0000C
NA	NA	NA	NA	NA	stop_gaine	ENSG0000(	transcript	ENST0000C
NA	NA	NA	NA	NA	frameshift_	ENSG0000(	transcript	ENST0000C
NA	NA	NA	NA	NA	splice_don	ENSG0000(	transcript	ENST0000C

NA	NA	NA	NA	NA	structural_	ENSG0000(	interaction	2NUP:A_33
NA	NA	NA	NA	NA	splice_acce	ENSG0000(	transcript	ENST0000C
NA	NA	NA	NA	NA	splice_acce	ENSG0000(	transcript	ENST0000C
NA	NA	NA	NA	NA	bidirection	ENSG0000(	gene_varia	ENSG0000C
NA	NA	NA	NA	NA	splice_don	ENSG0000(	transcript	ENST0000C
NA	Benign/Like	Fanconi_ar	NA	NA	splice_acce	ENSG0000(	transcript	ENST0000C
NA	Benign	Fanconi_ar	NA	NA	splice_acce	ENSG0000(	transcript	ENST0000C
NA	NA	NA	NA	NA	frameshift_	ENSG0000(	transcript	ENST0000C
NA	Benign/Like	Noonan_sy	NA	NA	frameshift_	ENSG0000(	transcript	ENST0000C
NA	Benign	Hereditary_	0.76327	0.82149	structural_	ENSG0000(	interaction	4IDN:B_77-
NA	Benign	Glycogen_s	NA	NA	splice_acce	ENSG0000(	transcript	ENST0000C
NA	NA	NA	NA	NA	splice_don	ENSG0000(	transcript	ENST0000C
NA	NA	NA	NA	NA	splice_acce	ENSG0000(	transcript	ENST0000C
NA	NA	NA	NA	NA	splice_acce	ENSG0000(	transcript	ENST0000C
NA	NA	NA	NA	NA	splice_regi	ENSG0000(	transcript	ENST0000C
NA	NA	NA	NA	NA	splice_regi	ENSG0000(	transcript	ENST0000C
NA	NA	NA	NA	NA	splice_regi	ENSG0000(	transcript	ENST0000C
NA	NA	NA	NA	NA	splice_don	ENSG0000(	transcript	ENST0000C
NA	NA	NA	NA	NA	splice_acce	ENSG0000(	transcript	ENST0000C
NA	NA	NA	NA	NA	splice_acce	ENSG0000(	transcript	ENST0000C
NA	NA	NA	NA	NA	splice_acce	ENSG0000(	transcript	ENST0000C
Primary_to	Benign/Like	Microphtha	0.45702	0.32568	stop_lost	ENSG0000(	transcript	ENST0000C
NA	NA	NA	NA	NA	frameshift_	ENSG0000(	transcript	ENST0000C
NA	NA	NA	NA	NA	structural_	ENSG0000(	interaction	3AYC:B_13
NA	NA	NA	NA	NA	splice_acce	ENSG0000(	transcript	ENST0000C
NA	NA	NA	NA	NA	splice_acce	ENSG0000(	transcript	ENST0000C
NA	NA	NA	NA	NA	splice_acce	ENSG0000(	transcript	ENST0000C
NA	NA	NA	NA	NA	splice_don	ENSG0000(	transcript	ENST0000C
NA	Benign	not_specifi	0.02565	0.01378	stop_gaine	ENSG0000(	transcript	ENST0000C
NA	NA	NA	NA	NA	splice_acce	ENSG0000(	transcript	ENST0000C
NA	NA	NA	NA	NA	stop_gaine	ENSG0000(	transcript	ENST0000C
NA	NA	NA	NA	NA	frameshift_	ENSG0000(	transcript	ENST0000C
NA	NA	NA	NA	NA	splice_don	ENSG0000(	transcript	ENST0000C
NA	NA	NA	NA	NA	splice_don	ENSG0000(	transcript	ENST0000C
NA	NA	NA	NA	NA	stop_gaine	ENSG0000(	transcript	ENST0000C
NA	NA	NA	NA	NA	start_lost	ENSG0000(	transcript	ENST0000C
NA	Conflicting_	Combined_	NA	NA	frameshift_	ENSG0000(	transcript	ENST0000C
NA	NA	NA	NA	NA	splice_don	ENSG0000(	transcript	ENST0000C
NA	NA	NA	NA	NA	splice_don	ENSG0000(	transcript	ENST0000C
NA	NA	NA	NA	NA	stop_gaine	ENSG0000(	transcript	ENST0000C
NA	NA	NA	NA	NA	splice_acce	ENSG0000(	transcript	ENST0000C
Psoriasis	Benign	Emery-Drei	0.08675	0.12240	stop_gaine	ENSG0000(	transcript	ENST0000C
NA	NA	NA	NA	NA	splice_don	ENSG0000(	transcript	ENST0000C
NA	NA	NA	NA	NA	structural_	ENSG0000(	interaction	3I33:A_208
NA	NA	NA	NA	NA	splice_don	ENSG0000(	transcript	ENST0000C
NA	NA	NA	NA	NA	frameshift_	ENSG0000(	transcript	ENST0000C
NA	NA	NA	NA	NA	frameshift_	ENSG0000(	transcript	ENST0000C

NA	NA	NA	NA	NA	splice_acce	ENSG0000(	transcript	ENST0000C
NA	NA	NA	NA	NA	splice_acce	ENSG0000(	transcript	ENST0000C
NA	Benign	not_provid	NA	NA	stop_gaine	ENSG0000(	transcript	ENST0000C
NA	NA	NA	NA	NA	splice_acce	ENSG0000(	transcript	ENST0000C
NA	NA	NA	NA	NA	splice_acce	ENSG0000(	transcript	ENST0000C
NA	NA	NA	NA	NA	splice_regi	ENSG0000(	transcript	ENST0000C
NA	NA	NA	NA	NA	splice_regi	ENSG0000(	transcript	ENST0000C
NA	NA	NA	NA	NA	splice_regi	ENSG0000(	transcript	ENST0000C
NA	NA	NA	NA	NA	splice_don	ENSG0000(	transcript	ENST0000C
NA	NA	NA	NA	NA	splice_acce	ENSG0000(	transcript	ENST0000C
NA	NA	NA	NA	NA	splice_acce	ENSG0000(	transcript	ENST0000C
NA	NA	NA	NA	NA	splice_don	ENSG0000(	transcript	ENST0000C
NA	NA	NA	NA	NA	splice_acce	ENSG0000(	transcript	ENST0000C
NA	NA	NA	NA	NA	splice_acce	ENSG0000(	transcript	ENST0000C
NA	NA	NA	NA	NA	frameshift_	ENSG0000(	transcript	ENST0000C
Red_cell_d	NA	NA	NA	NA	splice_acce	ENSG0000(	transcript	ENST0000C
NA	NA	NA	NA	NA	frameshift_	ENSG0000(	transcript	ENST0000C
NA	NA	NA	NA	NA	frameshift_	ENSG0000(	transcript	ENST0000C
NA	NA	NA	NA	NA	splice_acce	ENSG0000(	transcript	ENST0000C
NA	NA	NA	NA	NA	splice_acce	ENSG0000(	transcript	ENST0000C
NA	NA	NA	NA	NA	splice_acce	ENSG0000(	transcript	ENST0000C
NA	NA	NA	NA	NA	splice_acce	ENSG0000(	transcript	ENST0000C
NA	NA	NA	NA	NA	splice_acce	ENSG0000(	transcript	ENST0000C
NA	NA	NA	NA	NA	splice_acce	ENSG0000(	transcript	ENST0000C
NA	NA	NA	NA	NA	structural_	ENSG0000(	interaction	3HLK:A_91
NA	NA	NA	NA	NA	structural_	ENSG0000(	interaction	3HLK:A_16
NA	NA	NA	NA	NA	structural_	ENSG0000(	interaction	3K2I:A_147
NA	NA	NA	NA	NA	frameshift_	ENSG0000(	transcript	ENST0000C
NA	NA	NA	NA	NA	frameshift_	ENSG0000(	transcript	ENST0000C
NA	NA	NA	NA	NA	structural_	ENSG0000(	interaction	2VNA:A_76
Blood_prot	Conflicting_	Niemann-P	0.00363	0.00100	splice_don	ENSG0000(	transcript	ENST0000C
NA	NA	NA	NA	NA	frameshift_	ENSG0000(	transcript	ENST0000C
NA	NA	NA	NA	NA	splice_don	ENSG0000(	transcript	ENST0000C
NA	NA	NA	NA	NA	upstream_	ENSG0000(	transcript	ENST0000C
NA	NA	NA	NA	NA	frameshift_	ENSG0000(	transcript	ENST0000C
NA	NA	NA	NA	NA	frameshift_	ENSG0000(	transcript	ENST0000C
NA	NA	NA	NA	NA	splice_don	ENSG0000(	transcript	ENST0000C
NA	NA	NA	NA	NA	frameshift_	ENSG0000(	transcript	ENST0000C
NA	NA	NA	NA	NA	frameshift_	ENSG0000(	transcript	ENST0000C
NA	NA	NA	NA	NA	splice_acce	ENSG0000(	transcript	ENST0000C
NA	NA	NA	NA	NA	stop_gaine	ENSG0000(	transcript	ENST0000C
NA	Benign	not_provid	NA	NA	frameshift_	ENSG0000(	transcript	ENST0000C
NA	Benign/Like	Leber_cong	0.34728	0.40755	splice_don	ENSG0000(	transcript	ENST0000C
NA	NA	NA	NA	NA	frameshift_	ENSG0000(	transcript	ENST0000C
NA	NA	NA	NA	NA	splice_don	ENSG0000(	transcript	ENST0000C
NA	NA	NA	NA	NA	splice_don	ENSG0000(	transcript	ENST0000C
NA	NA	NA	NA	NA	frameshift_	ENSG0000(	transcript	ENST0000C

NA	NA	NA	NA	NA	stop_gaine	ENSG0000	transcript	ENST0000
NA	Uncertain_	not_specifi	NA	NA	frameshift_	ENSG0000	transcript	ENST0000
NA	Benign	not_specifi	NA	NA	frameshift_	ENSG0000	transcript	ENST0000
NA	Benign	not_specifi	NA	NA	frameshift_	ENSG0000	transcript	ENST0000
NA	Benign	not_specifi	NA	NA	frameshift_	ENSG0000	transcript	ENST0000
NA	Benign	not_specifi	NA	NA	frameshift_	ENSG0000	transcript	ENST0000
NA	Benign	not_specifi	NA	NA	frameshift_	ENSG0000	transcript	ENST0000
NA	Benign	not_specifi	NA	NA	frameshift_	ENSG0000	transcript	ENST0000
NA	NA	NA	NA	NA	frameshift_	ENSG0000	transcript	ENST0000
NA	Likely_beni	not_specifi	0.33422	NA	frameshift_	ENSG0000	transcript	ENST0000
NA	Likely_beni	not_specifi	0.33422	NA	frameshift_	ENSG0000	transcript	ENST0000
NA	NA	NA	NA	NA	splice_don	ENSG0000	transcript	ENST0000
NA	NA	NA	NA	NA	structural_	ENSG0000	interaction	4AWB:B_1
NA	NA	NA	NA	NA	splice_don	ENSG0000	transcript	ENST0000
NA	NA	NA	NA	NA	splice_acce	ENSG0000	transcript	ENST0000
NA	Benign	not_specifi	0.11975	0.08307	structural_	ENSG0000	interaction	2WUL:A_8
NA	NA	NA	NA	NA	splice_acce	ENSG0000	transcript	ENST0000
NA	NA	NA	NA	NA	splice_acce	ENSG0000	transcript	ENST0000
NA	NA	NA	NA	NA	frameshift_	ENSG0000	transcript	ENST0000
NA	NA	NA	NA	NA	start_lost&	ENSG0000	transcript	ENST0000
NA	NA	NA	NA	NA	structural_	ENSG0000	interaction	1O5T:A_30
NA	NA	NA	NA	NA	splice_acce	ENSG0000	transcript	ENST0000
NA	NA	NA	NA	NA	splice_don	ENSG0000	transcript	ENST0000
NA	NA	NA	NA	NA	splice_don	ENSG0000	transcript	ENST0000
NA	NA	NA	NA	NA	splice_acce	ENSG0000	transcript	ENST0000
NA	NA	NA	NA	NA	splice_don	ENSG0000	transcript	ENST0000
NA	NA	NA	NA	NA	structural_	ENSG0000	interaction	3Q6M:B_6
NA	NA	NA	NA	NA	structural_	ENSG0000	interaction	3Q6M:B_3
NA	NA	NA	NA	NA	frameshift_	ENSG0000	transcript	ENST0000
NA	NA	NA	NA	NA	splice_don	ENSG0000	transcript	ENST0000
NA	NA	NA	NA	NA	stop_gaine	ENSG0000	transcript	ENST0000
NA	NA	NA	NA	NA	frameshift_	ENSG0000	transcript	ENST0000
NA	NA	NA	NA	NA	splice_acce	ENSG0000	transcript	ENST0000
NA	NA	NA	NA	NA	splice_acce	ENSG0000	transcript	ENST0000
NA	NA	NA	NA	NA	splice_acce	ENSG0000	transcript	ENST0000
NA	NA	NA	NA	NA	splice_acce	ENSG0000	transcript	ENST0000
NA	NA	NA	NA	NA	structural_	ENSG0000	interaction	3B6R:A_30
Body_mass	NA	NA	NA	NA	structural_	ENSG0000	interaction	3B6R:A_15
NA	NA	NA	NA	NA	splice_don	ENSG0000	transcript	ENST0000
NA	Benign/Like	Cowden_sy	0.01313	0.00899	structural_	ENSG0000	interaction	4EJN:A_20
NA	Benign	Cowden_sy	0.00251	0.00060	structural_	ENSG0000	interaction	3CQU:A_3
NA	NA	NA	NA	NA	frameshift_	ENSG0000	transcript	ENST0000
NA	NA	NA	NA	NA	splice_don	ENSG0000	transcript	ENST0000
NA	NA	NA	NA	NA	structural_	ENSG0000	interaction	2VKP:A_56
NA	NA	NA	NA	NA	stop_gaine	ENSG0000	transcript	ENST0000
NA	NA	NA	NA	NA	stop_gaine	ENSG0000	transcript	ENST0000
NA	NA	NA	NA	NA	frameshift_	ENSG0000	transcript	ENST0000

NA	NA	NA	NA	NA	frameshift_	ENSG0000(	transcript	ENST0000C
NA	NA	NA	NA	NA	frameshift_	ENSG0000(	transcript	ENST0000C
NA	NA	NA	NA	NA	frameshift_	ENSG0000(	transcript	ENST0000C
NA	NA	NA	NA	NA	splice_don	ENSG0000(	transcript	ENST0000C
NA	NA	NA	NA	NA	frameshift_	ENSG0000(	transcript	ENST0000C
NA	NA	NA	NA	NA	frameshift_	ENSG0000(	transcript	ENST0000C
NA	NA	NA	NA	NA	splice_don	ENSG0000(	transcript	ENST0000C
NA	NA	NA	NA	NA	splice_don	ENSG0000(	transcript	ENST0000C
NA	NA	NA	NA	NA	structural_	ENSG0000(	interaction	4PJ3:A_56(
NA	NA	NA	NA	NA	frameshift_	ENSG0000(	transcript	ENST0000C
NA	NA	NA	NA	NA	splice_don	ENSG0000(	transcript	ENST0000C
NA	Benign	not_specifi	0.02406	0.01278	structural_	ENSG0000(	interaction	3R6B:A_49
NA	NA	NA	NA	NA	structural_	ENSG0000(	interaction	2FJU:B_24[illegible]
NA	NA	NA	NA	NA	frameshift_	ENSG0000(	transcript	ENST0000C
NA	NA	NA	NA	NA	splice_don	ENSG0000(	transcript	ENST0000C
NA	NA	NA	NA	NA	frameshift_	ENSG0000(	transcript	ENST0000C
NA	NA	NA	NA	NA	splice_don	ENSG0000(	transcript	ENST0000C
NA	NA	NA	NA	NA	splice_acce	ENSG0000(	transcript	ENST0000C
NA	NA	NA	NA	NA	splice_acce	ENSG0000(	transcript	ENST0000C
NA	NA	NA	NA	NA	splice_acce	ENSG0000(	transcript	ENST0000C
NA	NA	NA	NA	NA	splice_don	ENSG0000(	transcript	ENST0000C
NA	NA	NA	NA	NA	frameshift_	ENSG0000(	transcript	ENST0000C
NA	NA	NA	NA	NA	splice_don	ENSG0000(	transcript	ENST0000C
NA	NA	NA	NA	NA	frameshift_	ENSG0000(	transcript	ENST0000C
NA	NA	NA	NA	NA	splice_don	ENSG0000(	transcript	ENST0000C
NA	NA	NA	NA	NA	splice_don	ENSG0000(	transcript	ENST0000C
NA	NA	NA	NA	NA	splice_don	ENSG0000(	transcript	ENST0000C
NA	NA	NA	NA	NA	splice_don	ENSG0000(	transcript	ENST0000C
NA	NA	NA	NA	NA	splice_don	ENSG0000(	transcript	ENST0000C
NA	NA	NA	NA	NA	splice_don	ENSG0000(	transcript	ENST0000C
NA	NA	NA	NA	NA	splice_don	ENSG0000(	transcript	ENST0000C
NA	NA	NA	NA	NA	stop_gaine	ENSG0000(	transcript	ENST0000C
NA	NA	NA	NA	NA	stop_gaine	ENSG0000(	transcript	ENST0000C
NA	NA	NA	NA	NA	frameshift_	ENSG0000(	transcript	ENST0000C
NA	NA	NA	NA	NA	splice_don	ENSG0000(	transcript	ENST0000C
NA	NA	NA	NA	NA	stop_gaine	ENSG0000(	transcript	ENST0000C
NA	NA	NA	NA	NA	splice_acce	ENSG0000(	transcript	ENST0000C
NA	Benign	not_specifi	0.33037	0.45188	splice_acce	ENSG0000(	transcript	ENST0000C
NA	NA	NA	NA	NA	splice_acce	ENSG0000(	transcript	ENST0000C
NA	NA	NA	NA	NA	frameshift_	ENSG0000(	transcript	ENST0000C
NA	NA	NA	NA	NA	frameshift_	ENSG0000(	transcript	ENST0000C
NA	NA	NA	NA	NA	frameshift_	ENSG0000(	transcript	ENST0000C
NA	NA	NA	NA	NA	start_lost	ENSG0000(	transcript	ENST0000C
NA	NA	NA	NA	NA	splice_don	ENSG0000(	transcript	ENST0000C
NA	Pathogenic	Nongoitrou	NA	NA	frameshift_	ENSG0000(	transcript	ENST0000C
NA	NA	NA	NA	NA	bidirection	ENSG0000(	gene_varia	ENSG0000C
NA	NA	NA	NA	NA	bidirection	ENSG0000(	gene_varia	ENSG0000C

NA	NA	NA	NA	NA	bidirection	ENSG0000(	gene_varia	ENSG00000
NA	NA	NA	NA	NA	bidirection	ENSG0000(	gene_varia	ENSG00000
NA	NA	NA	NA	NA	bidirection	ENSG0000(	gene_varia	ENSG00000
NA	NA	NA	NA	NA	splice_acce	ENSG0000(	transcript	ENST0000C
NA	NA	NA	NA	NA	structural_	ENSG0000(	interaction	2A2C:A_14
NA	Benign	Hereditary_	NA	0.04054	structural_	ENSG0000(	interaction	2GFO:A_10
NA	NA	NA	NA	NA	splice_don	ENSG0000(	transcript	ENST0000C
NA	NA	NA	NA	NA	splice_acce	ENSG0000(	transcript	ENST0000C
NA	NA	NA	NA	NA	stop_gaine	ENSG0000(	transcript	ENST0000C
NA	NA	NA	NA	NA	splice_acce	ENSG0000(	transcript	ENST0000C
NA	NA	NA	NA	NA	splice_acce	ENSG0000(	transcript	ENST0000C
NA	Benign	Dyslexia_1	0.11236	0.17552	stop_gaine	ENSG0000(	transcript	ENST0000C
NA	NA	NA	NA	NA	structural_	ENSG0000(	interaction	2XBF:A_53
NA	NA	NA	NA	NA	gene_fusio	ENSG0000(	gene_varia	ENSG00000
NA	NA	NA	NA	NA	gene_fusio	ENSG0000(	gene_varia	ENSG00000
NA	NA	NA	NA	NA	start_lost	ENSG0000(	transcript	ENST0000C
NA	NA	NA	NA	NA	frameshift_	ENSG0000(	transcript	ENST0000C
NA	NA	NA	NA	NA	splice_acce	ENSG0000(	transcript	ENST0000C
NA	NA	NA	NA	NA	stop_gaine	ENSG0000(	transcript	ENST0000C
NA	NA	NA	NA	NA	splice_acce	ENSG0000(	transcript	ENST0000C
NA	NA	NA	NA	NA	splice_don	ENSG0000(	transcript	ENST0000C
NA	NA	NA	NA	NA	splice_acce	ENSG0000(	transcript	ENST0000C
NA	NA	NA	NA	NA	splice_acce	ENSG0000(	transcript	ENST0000C
NA	NA	NA	NA	NA	structural_	ENSG0000(	interaction	3UX3:A_39
Neuroticisr	NA	NA	NA	NA	splice_don	ENSG0000(	transcript	ENST0000C
NA	NA	NA	NA	NA	splice_acce	ENSG0000(	transcript	ENST0000C
NA	NA	NA	NA	NA	splice_acce	ENSG0000(	transcript	ENST0000C
NA	NA	NA	NA	NA	splice_acce	ENSG0000(	transcript	ENST0000C
NA	NA	NA	NA	NA	stop_gaine	ENSG0000(	transcript	ENST0000C
NA	NA	NA	NA	NA	splice_don	ENSG0000(	transcript	ENST0000C
NA	NA	NA	NA	NA	splice_acce	ENSG0000(	transcript	ENST0000C
NA	NA	NA	NA	NA	splice_acce	ENSG0000(	transcript	ENST0000C
NA	NA	NA	NA	NA	splice_acce	ENSG0000(	transcript	ENST0000C
NA	NA	NA	NA	NA	splice_don	ENSG0000(	transcript	ENST0000C
NA	NA	NA	NA	NA	splice_don	ENSG0000(	transcript	ENST0000C
NA	NA	NA	NA	NA	splice_acce	ENSG0000(	transcript	ENST0000C
NA	NA	NA	NA	NA	start_lost	ENSG0000(	transcript	ENST0000C
NA	NA	NA	NA	NA	splice_acce	ENSG0000(	transcript	ENST0000C
NA	NA	NA	NA	NA	splice_don	ENSG0000(	transcript	ENST0000C
NA	NA	NA	NA	NA	splice_don	ENSG0000(	transcript	ENST0000C
NA	NA	NA	NA	NA	splice_acce	ENSG0000(	transcript	ENST0000C
NA	NA	NA	NA	NA	splice_acce	ENSG0000(	transcript	ENST0000C
NA	NA	NA	NA	NA	splice_acce	ENSG0000(	transcript	ENST0000C
NA	NA	NA	NA	NA	stop_lost	ENSG0000(	transcript	ENST0000C
NA	NA	NA	NA	NA	splice_acce	ENSG0000(	transcript	ENST0000C
NA	NA	NA	NA	NA	splice_acce	ENSG0000(	transcript	ENST0000C
NA	NA	NA	NA	NA	structural_	ENSG0000(	interaction	3P4L:A_89

NA	NA	NA	NA	NA	splice_don	ENSG0000(	transcript	ENST0000C
NA	NA	NA	NA	NA	splice_don	ENSG0000(	transcript	ENST0000C
NA	NA	NA	NA	NA	structural_	ENSG0000(	interaction	1BYG:A_22
NA	NA	NA	NA	NA	frameshift_	ENSG0000(	transcript	ENST0000C
NA	NA	NA	NA	NA	splice_acce	ENSG0000(	transcript	ENST0000C
NA	NA	NA	NA	NA	splice_acce	ENSG0000(	transcript	ENST0000C
NA	NA	NA	NA	NA	frameshift_	ENSG0000(	transcript	ENST0000C
NA	NA	NA	NA	NA	splice_don	ENSG0000(	transcript	ENST0000C
NA	NA	NA	NA	NA	splice_don	ENSG0000(	transcript	ENST0000C
NA	NA	NA	NA	NA	splice_acce	ENSG0000(	transcript	ENST0000C
NA	NA	NA	NA	NA	splice_acce	ENSG0000(	transcript	ENST0000C
NA	NA	NA	NA	NA	splice_acce	ENSG0000(	transcript	ENST0000C
NA	NA	NA	NA	NA	splice_acce	ENSG0000(	transcript	ENST0000C
NA	NA	NA	NA	NA	start_lost	ENSG0000(	transcript	ENST0000C
NA	NA	NA	NA	NA	splice_don	ENSG0000(	transcript	ENST0000C
NA	NA	NA	NA	NA	splice_don	ENSG0000(	transcript	ENST0000C
NA	NA	NA	NA	NA	splice_acce	ENSG0000(	transcript	ENST0000C
NA	NA	NA	NA	NA	start_lost	ENSG0000(	transcript	ENST0000C
NA	Likely_beni	not_specifi	0.03216	0.01837	frameshift_	ENSG0000(	transcript	ENST0000C
NA	NA	NA	NA	NA	stop_lost	ENSG0000(	transcript	ENST0000C
NA	NA	NA	NA	NA	frameshift_	ENSG0000(	transcript	ENST0000C
NA	NA	NA	NA	NA	structural_	ENSG0000(	interaction	2AQL:B_18
NA	NA	NA	NA	NA	splice_don	ENSG0000(	transcript	ENST0000C
NA	NA	NA	NA	NA	frameshift_	ENSG0000(	transcript	ENST0000C
NA	Benign	not_specifi	NA	0.00938	stop_gaine	ENSG0000(	transcript	ENST0000C
NA	NA	NA	NA	NA	splice_acce	ENSG0000(	transcript	ENST0000C
NA	NA	NA	NA	NA	frameshift_	ENSG0000(	transcript	ENST0000C
NA	NA	NA	NA	NA	frameshift_	ENSG0000(	transcript	ENST0000C
NA	NA	NA	NA	NA	frameshift_	ENSG0000(	transcript	ENST0000C
NA	NA	NA	NA	NA	splice_acce	ENSG0000(	transcript	ENST0000C
NA	NA	NA	NA	NA	splice_don	ENSG0000(	transcript	ENST0000C
NA	NA	NA	NA	NA	splice_acce	ENSG0000(	transcript	ENST0000C
NA	NA	NA	NA	NA	splice_don	ENSG0000(	transcript	ENST0000C
NA	NA	NA	NA	NA	splice_don	ENSG0000(	transcript	ENST0000C
NA	NA	NA	NA	NA	structural_	ENSG0000(	interaction	1WLJ:A_35
NA	NA	NA	NA	NA	stop_gaine	ENSG0000(	transcript	ENST0000C
NA	NA	NA	NA	NA	splice_don	ENSG0000(	transcript	ENST0000C
NA	NA	NA	NA	NA	frameshift_	ENSG0000(	transcript	ENST0000C
NA	NA	NA	NA	NA	frameshift_	ENSG0000(	transcript	ENST0000C
NA	NA	NA	NA	NA	frameshift_	ENSG0000(	transcript	ENST0000C
NA	NA	NA	NA	NA	splice_acce	ENSG0000(	transcript	ENST0000C
NA	NA	NA	NA	NA	splice_acce	ENSG0000(	transcript	ENST0000C
NA	NA	NA	NA	NA	splice_acce	ENSG0000(	transcript	ENST0000C
NA	NA	NA	NA	NA	splice_acce	ENSG0000(	transcript	ENST0000C
NA	NA	NA	NA	NA	splice_acce	ENSG0000(	transcript	ENST0000C
NA	NA	NA	NA	NA	structural_	ENSG0000(	interaction	4FYQ:A_45
NA	NA	NA	NA	NA	structural_	ENSG0000(	interaction	4FYQ:A_69

NA	NA	NA	NA	NA	structural_	ENSG0000(	interaction	4FYQ:A_11
NA	NA	NA	NA	NA	structural_	ENSG0000(	interaction	4FYQ:A_86
NA	Benign	D-2-hydrox	NA	NA	structural_	ENSG0000(	interaction	4JA8:A_364
NA	NA	NA	NA	NA	stop_gaine	ENSG0000(	transcript	ENST0000C
NA	NA	NA	NA	NA	structural_	ENSG0000(	interaction	4OMD:C_4
NA	NA	NA	NA	NA	frameshift_	ENSG0000(	transcript	ENST0000C
NA	NA	NA	NA	NA	frameshift_	ENSG0000(	transcript	ENST0000C
NA	NA	NA	NA	NA	splice_don	ENSG0000(	transcript	ENST0000C
NA	NA	NA	NA	NA	splice_acce	ENSG0000(	transcript	ENST0000C
NA	NA	NA	NA	NA	splice_acce	ENSG0000(	transcript	ENST0000C
NA	NA	NA	NA	NA	splice_don	ENSG0000(	transcript	ENST0000C
NA	NA	NA	NA	NA	stop_gaine	ENSG0000(	transcript	ENST0000C
NA	NA	NA	NA	NA	splice_acce	ENSG0000(	transcript	ENST0000C
NA	NA	NA	NA	NA	splice_don	ENSG0000(	transcript	ENST0000C
NA	NA	NA	NA	NA	frameshift_	ENSG0000(	transcript	ENST0000C
NA	NA	NA	NA	NA	bidirection	ENSG0000(	gene_varia	ENSG0000C
NA	NA	NA	NA	NA	bidirection	ENSG0000(	gene_varia	ENSG0000C
NA	NA	NA	NA	NA	bidirection	ENSG0000(	gene_varia	ENSG0000C
NA	NA	NA	NA	NA	bidirection	ENSG0000(	gene_varia	ENSG0000C
NA	NA	NA	NA	NA	bidirection	ENSG0000(	gene_varia	ENSG0000C
NA	NA	NA	NA	NA	bidirection	ENSG0000(	gene_varia	ENSG0000C
NA	NA	NA	NA	NA	stop_gaine	ENSG0000(	transcript	ENST0000C
NA	NA	NA	NA	NA	splice_don	ENSG0000(	transcript	ENST0000C
NA	NA	NA	NA	NA	splice_don	ENSG0000(	transcript	ENST0000C
NA	NA	NA	NA	NA	frameshift_	ENSG0000(	transcript	ENST0000C
NA	NA	NA	NA	NA	splice_don	ENSG0000(	transcript	ENST0000C
NA	NA	NA	NA	NA	splice_don	ENSG0000(	transcript	ENST0000C
NA	NA	NA	NA	NA	splice_don	ENSG0000(	transcript	ENST0000C
NA	NA	NA	NA	NA	splice_acce	ENSG0000(	transcript	ENST0000C
NA	NA	NA	NA	NA	splice_acce	ENSG0000(	transcript	ENST0000C
NA	NA	NA	NA	NA	stop_gaine	ENSG0000(	transcript	ENST0000C
NA	NA	NA	NA	NA	splice_acce	ENSG0000(	transcript	ENST0000C
NA	NA	NA	NA	NA	splice_acce	ENSG0000(	transcript	ENST0000C
NA	NA	NA	NA	NA	splice_acce	ENSG0000(	transcript	ENST0000C
NA	NA	NA	NA	NA	frameshift_	ENSG0000(	transcript	ENST0000C
NA	NA	NA	NA	NA	stop_gaine	ENSG0000(	transcript	ENST0000C
NA	NA	NA	NA	NA	frameshift_	ENSG0000(	transcript	ENST0000C
NA	NA	NA	NA	NA	frameshift_	ENSG0000(	transcript	ENST0000C
NA	NA	NA	NA	NA	frameshift_	ENSG0000(	transcript	ENST0000C
NA	NA	NA	NA	NA	frameshift_	ENSG0000(	transcript	ENST0000C
NA	NA	NA	NA	NA	splice_don	ENSG0000(	transcript	ENST0000C
NA	NA	NA	NA	NA	frameshift_	ENSG0000(	transcript	ENST0000C
NA	NA	NA	NA	NA	frameshift_	ENSG0000(	transcript	ENST0000C
NA	NA	NA	NA	NA	frameshift_	ENSG0000(	transcript	ENST0000C
NA	NA	NA	NA	NA	frameshift_	ENSG0000(	transcript	ENST0000C
NA	NA	NA	NA	NA	frameshift_	ENSG0000(	transcript	ENST0000C
NA	NA	NA	NA	NA	frameshift_	ENSG0000(	transcript	ENST0000C

NA	NA	NA	NA	NA	frameshift_	ENSG0000	transcript	ENST00000
NA	NA	NA	NA	NA	frameshift_	ENSG0000	transcript	ENST00000
NA	NA	NA	NA	NA	frameshift_	ENSG0000	transcript	ENST00000
NA	NA	NA	NA	NA	frameshift_	ENSG0000	transcript	ENST00000
NA	NA	NA	NA	NA	frameshift_	ENSG0000	transcript	ENST00000
NA	NA	NA	NA	NA	frameshift_	ENSG0000	transcript	ENST00000
NA	NA	NA	NA	NA	splice_don	ENSG0000	transcript	ENST00000
NA	NA	NA	NA	NA	splice_don	ENSG0000	transcript	ENST00000
NA	NA	NA	NA	NA	splice_acce	ENSG0000	transcript	ENST00000
NA	NA	NA	NA	NA	splice_don	ENSG0000	transcript	ENST00000
NA	NA	NA	NA	NA	frameshift_	ENSG0000	transcript	ENST00000
NA	NA	NA	NA	NA	structural_	ENSG0000	interaction	1A3S:A_46
NA	NA	NA	NA	NA	frameshift_	ENSG0000	transcript	ENST00000
NA	NA	NA	NA	NA	splice_acce	ENSG0000	transcript	ENST00000
NA	NA	NA	NA	NA	structural_	ENSG0000	interaction	1ZS6:A_53
NA	NA	NA	NA	NA	splice_don	ENSG0000	transcript	ENST00000
NA	NA	NA	NA	NA	frameshift_	ENSG0000	transcript	ENST00000
NA	NA	NA	NA	NA	splice_don	ENSG0000	transcript	ENST00000
NA	NA	NA	NA	NA	frameshift_	ENSG0000	transcript	ENST00000
NA	NA	NA	NA	NA	stop_gaine	ENSG0000	transcript	ENST00000
NA	NA	NA	NA	NA	stop_lost	ENSG0000	transcript	ENST00000
NA	NA	NA	NA	NA	splice_acce	ENSG0000	transcript	ENST00000
NA	NA	NA	NA	NA	frameshift_	ENSG0000	transcript	ENST00000
NA	NA	NA	NA	NA	frameshift_	ENSG0000	transcript	ENST00000
NA	NA	NA	NA	NA	splice_don	ENSG0000	transcript	ENST00000
NA	NA	NA	NA	NA	frameshift_	ENSG0000	transcript	ENST00000
NA	NA	NA	NA	NA	frameshift_	ENSG0000	transcript	ENST00000
NA	Conflicting_	Abnormalit	0.00550	0.00180	structural_	ENSG0000	interaction	4CG4:A_66
NA	Benign	Familial_M	NA	0.35363	structural_	ENSG0000	interaction	2WL1:A_5
NA	Benign/Lik	Familial_M	0.60570	0.65056	structural_	ENSG0000	interaction	4CG4:A_47
NA	NA	NA	NA	NA	frameshift_	ENSG0000	transcript	ENST00000
NA	Benign	not_provid	0.15297	0.10823	structural_	ENSG0000	interaction	4Z1F:A_33
NA	Benign	not_provid	0.05649	0.04852	structural_	ENSG0000	interaction	4Z1F:A_31
NA	NA	NA	NA	NA	frameshift_	ENSG0000	transcript	ENST00000
NA	NA	NA	NA	NA	stop_lost	ENSG0000	transcript	ENST00000
NA	NA	NA	NA	NA	splice_acce	ENSG0000	transcript	ENST00000
NA	NA	NA	NA	NA	splice_don	ENSG0000	transcript	ENST00000
NA	Benign	ALG1-CDG|	NA	NA	frameshift_	ENSG0000	transcript	ENST00000
NA	NA	NA	NA	NA	splice_don	ENSG0000	transcript	ENST00000
NA	NA	NA	NA	NA	splice_don	ENSG0000	transcript	ENST00000
NA	NA	NA	NA	NA	splice_acce	ENSG0000	transcript	ENST00000
NA	NA	NA	NA	NA	splice_acce	ENSG0000	transcript	ENST00000
NA	NA	NA	NA	NA	splice_acce	ENSG0000	transcript	ENST00000
NA	Benign	not_provid	NA	NA	start_lost&	ENSG0000	transcript	ENST00000
NA	Benign/Lik	Congenital_	0.01930	0.00899	structural_	ENSG0000	interaction	2Q4R:A_19
NA	NA	NA	NA	NA	splice_acce	ENSG0000	transcript	ENST00000
NA	NA	NA	NA	NA	splice_acce	ENSG0000	transcript	ENST00000

NA	NA	NA	NA	NA	splice_acce	ENSG0000(	transcript	ENST0000C
NA	NA	NA	NA	NA	splice_acce	ENSG0000(	transcript	ENST0000C
NA	NA	NA	NA	NA	splice_acce	ENSG0000(	transcript	ENST0000C
NA	NA	NA	NA	NA	splice_acce	ENSG0000(	transcript	ENST0000C
NA	NA	NA	NA	NA	splice_acce	ENSG0000(	transcript	ENST0000C
NA	NA	NA	NA	NA	splice_acce	ENSG0000(	transcript	ENST0000C
NA	NA	NA	NA	NA	frameshift_	ENSG0000(	transcript	ENST0000C
NA	NA	NA	NA	NA	structural_	ENSG0000(	interaction	3MXN:B_5(
NA	NA	NA	NA	NA	structural_	ENSG0000(	interaction	3MXN:B_1(
NA	NA	NA	NA	NA	frameshift_	ENSG0000(	transcript	ENST0000C
NA	NA	NA	NA	NA	frameshift_	ENSG0000(	transcript	ENST0000C
NA	NA	NA	NA	NA	splice_don	ENSG0000(	transcript	ENST0000C
NA	NA	NA	NA	NA	splice_don	ENSG0000(	transcript	ENST0000C
NA	NA	NA	NA	NA	splice_don	ENSG0000(	transcript	ENST0000C
NA	NA	NA	NA	NA	frameshift_	ENSG0000(	transcript	ENST0000C
NA	NA	NA	NA	NA	splice_acce	ENSG0000(	transcript	ENST0000C
NA	NA	NA	NA	NA	splice_acce	ENSG0000(	transcript	ENST0000C
NA	NA	NA	NA	NA	splice_acce	ENSG0000(	transcript	ENST0000C
NA	NA	NA	NA	NA	splice_acce	ENSG0000(	transcript	ENST0000C
NA	NA	NA	NA	NA	splice_acce	ENSG0000(	transcript	ENST0000C
NA	NA	NA	NA	NA	splice_acce	ENSG0000(	transcript	ENST0000C
NA	NA	NA	NA	NA	splice_don	ENSG0000(	transcript	ENST0000C
NA	NA	NA	NA	NA	stop_gaine	ENSG0000(	transcript	ENST0000C
NA	NA	NA	NA	NA	splice_don	ENSG0000(	transcript	ENST0000C
NA	NA	NA	NA	NA	frameshift_	ENSG0000(	transcript	ENST0000C
NA	NA	NA	NA	NA	frameshift_	ENSG0000(	transcript	ENST0000C
NA	NA	NA	NA	NA	frameshift_	ENSG0000(	transcript	ENST0000C
NA	NA	NA	NA	NA	splice_acce	ENSG0000(	transcript	ENST0000C
NA	NA	NA	NA	NA	splice_acce	ENSG0000(	transcript	ENST0000C
NA	NA	NA	NA	NA	stop_gaine	ENSG0000(	transcript	ENST0000C
NA	NA	NA	NA	NA	structural_	ENSG0000(	interaction	2VZE:A_24
NA	NA	NA	NA	NA	stop_gaine	ENSG0000(	transcript	ENST0000C
NA	NA	NA	NA	NA	splice_don	ENSG0000(	transcript	ENST0000C
NA	NA	NA	NA	NA	stop_gaine	ENSG0000(	transcript	ENST0000C
NA	NA	NA	NA	NA	splice_don	ENSG0000(	transcript	ENST0000C
NA	Benign	not_specifi	NA	0.50260	structural_	ENSG0000(	interaction	2I99:A_222
NA	NA	NA	NA	NA	splice_acce	ENSG0000(	transcript	ENST0000C
NA	NA	NA	NA	NA	splice_acce	ENSG0000(	transcript	ENST0000C
NA	NA	NA	NA	NA	splice_acce	ENSG0000(	transcript	ENST0000C
NA	NA	NA	NA	NA	frameshift_	ENSG0000(	transcript	ENST0000C
NA	NA	NA	NA	NA	frameshift_	ENSG0000(	transcript	ENST0000C
NA	NA	NA	NA	NA	stop_gaine	ENSG0000(	transcript	ENST0000C
NA	NA	NA	NA	NA	splice_acce	ENSG0000(	transcript	ENST0000C
NA	NA	NA	NA	NA	splice_acce	ENSG0000(	transcript	ENST0000C
NA	NA	NA	NA	NA	splice_acce	ENSG0000(	transcript	ENST0000C
NA	NA	NA	NA	NA	splice_acce	ENSG0000(	transcript	ENST0000C
NA	NA	NA	NA	NA	splice_acce	ENSG0000(	transcript	ENST0000C

NA	NA	NA	NA	NA	splice_acce	ENSG0000(	transcript	ENST0000C
NA	NA	NA	NA	NA	splice_acce	ENSG0000(	transcript	ENST0000C
NA	NA	NA	NA	NA	splice_acce	ENSG0000(	transcript	ENST0000C
NA	NA	NA	NA	NA	splice_acce	ENSG0000(	transcript	ENST0000C
NA	NA	NA	NA	NA	structural_	ENSG0000(	interaction	3UYJ:A_34(
NA	NA	NA	NA	NA	frameshift_	ENSG0000(	transcript	ENST0000C
NA	NA	NA	NA	NA	frameshift_	ENSG0000(	transcript	ENST0000C
NA	NA	NA	NA	NA	structural_	ENSG0000(	interaction	3ME9:A_12
NA	NA	NA	NA	NA	structural_	ENSG0000(	interaction	3ME9:B_16
NA	NA	NA	NA	NA	structural_	ENSG0000(	interaction	1Z29:A_44-
NA	NA	NA	NA	NA	structural_	ENSG0000(	interaction	1Z29:A_19-
NA	NA	NA	NA	NA	structural_	ENSG0000(	interaction	1LS6:A_19-
NA	NA	NA	NA	NA	splice_acce	ENSG0000(	transcript	ENST0000C
NA	NA	NA	NA	NA	splice_don	ENSG0000(	transcript	ENST0000C
NA	NA	NA	NA	NA	splice_don	ENSG0000(	transcript	ENST0000C
NA	NA	NA	NA	NA	bidirection	ENSG0000(	gene_varia	ENSG0000C
NA	NA	NA	NA	NA	structural_	ENSG0000(	interaction	4KWV:A_1
NA	NA	NA	NA	NA	structural_	ENSG0000(	interaction	4MJJ:A_19
NA	NA	NA	NA	NA	splice_acce	ENSG0000(	transcript	ENST0000C
NA	NA	NA	NA	NA	splice_acce	ENSG0000(	transcript	ENST0000C
NA	NA	NA	NA	NA	frameshift_	ENSG0000(	transcript	ENST0000C
NA	NA	NA	NA	NA	frameshift_	ENSG0000(	transcript	ENST0000C
NA	NA	NA	NA	NA	frameshift_	ENSG0000(	transcript	ENST0000C
NA	NA	NA	NA	NA	frameshift_	ENSG0000(	transcript	ENST0000C
NA	NA	NA	NA	NA	frameshift_	ENSG0000(	transcript	ENST0000C
NA	NA	NA	NA	NA	frameshift_	ENSG0000(	transcript	ENST0000C
NA	NA	NA	NA	NA	frameshift_	ENSG0000(	transcript	ENST0000C
NA	NA	NA	NA	NA	frameshift_	ENSG0000(	transcript	ENST0000C
NA	drug_respo	warfarin_re	0.19190	0.09145	splice_don	ENSG0000(	transcript	ENST0000C
NA	NA	NA	NA	NA	structural_	ENSG0000(	interaction	1Y01:A_45-
NA	NA	NA	NA	NA	splice_acce	ENSG0000(	transcript	ENST0000C
NA	NA	NA	NA	NA	frameshift_	ENSG0000(	transcript	ENST0000C
NA	NA	NA	NA	NA	splice_don	ENSG0000(	transcript	ENST0000C
NA	Benign	Glycogen_s	0.00248	0.00140	stop_gaine	ENSG0000(	transcript	ENST0000C
NA	NA	NA	NA	NA	stop_gaine	ENSG0000(	transcript	ENST0000C
NA	NA	NA	NA	NA	splice_acce	ENSG0000(	transcript	ENST0000C
NA	NA	NA	NA	NA	frameshift_	ENSG0000(	transcript	ENST0000C
Crohn's_di	Conflicting_	Inflammato	NA	NA	frameshift_	ENSG0000(	transcript	ENST0000C
NA	Benign	Blau_syndr	NA	0.35823	splice_don	ENSG0000(	transcript	ENST0000C
NA	NA	NA	NA	NA	splice_don	ENSG0000(	transcript	ENST0000C
NA	NA	NA	NA	NA	splice_don	ENSG0000(	transcript	ENST0000C
NA	NA	NA	NA	NA	frameshift_	ENSG0000(	transcript	ENST0000C
NA	NA	NA	NA	NA	splice_don	ENSG0000(	transcript	ENST0000C
NA	NA	NA	NA	NA	structural_	ENSG0000(	interaction	1CK7:A_41
NA	NA	NA	NA	NA	start_lost	ENSG0000(	transcript	ENST0000C
NA	NA	NA	NA	NA	splice_acce	ENSG0000(	transcript	ENST0000C
NA	NA	NA	NA	NA	splice_don	ENSG0000(	transcript	ENST0000C

NA	NA	NA	NA	NA	splice_acce	ENSG0000(	transcript	ENST0000C
NA	NA	NA	NA	NA	splice_don	ENSG0000(	transcript	ENST0000C
NA	NA	NA	NA	NA	frameshift_	ENSG0000(	transcript	ENST0000C
NA	NA	NA	NA	NA	frameshift_	ENSG0000(	transcript	ENST0000C
NA	NA	NA	NA	NA	splice_acce	ENSG0000(	transcript	ENST0000C
NA	NA	NA	NA	NA	splice_acce	ENSG0000(	transcript	ENST0000C
NA	NA	NA	NA	NA	splice_acce	ENSG0000(	transcript	ENST0000C
NA	NA	NA	NA	NA	splice_acce	ENSG0000(	transcript	ENST0000C
NA	NA	NA	NA	NA	splice_acce	ENSG0000(	transcript	ENST0000C
NA	NA	NA	NA	NA	splice_acce	ENSG0000(	transcript	ENST0000C
NA	NA	NA	NA	NA	splice_acce	ENSG0000(	transcript	ENST0000C
NA	NA	NA	NA	NA	stop_gaine	ENSG0000(	transcript	ENST0000C
NA	NA	NA	NA	NA	splice_don	ENSG0000(	transcript	ENST0000C
NA	NA	NA	NA	NA	splice_don	ENSG0000(	transcript	ENST0000C
NA	Benign	not_specifi	0.06977	0.06010	structural_	ENSG0000(	interaction	4H7W:A_9
NA	NA	NA	NA	NA	structural_	ENSG0000(	interaction	3OFM:A_1
NA	NA	NA	NA	NA	splice_don	ENSG0000(	transcript	ENST0000C
NA	NA	NA	NA	NA	structural_	ENSG0000(	interaction	3QXY:A_37
NA	NA	NA	NA	NA	protein_pr	ENSG0000(	interaction	4CRU:A_15
NA	Benign	not_specifi	NA	NA	frameshift_	ENSG0000(	transcript	ENST0000C
NA	NA	NA	NA	NA	structural_	ENSG0000(	interaction	4CRU:A_13
NA	NA	NA	NA	NA	frameshift_	ENSG0000(	transcript	ENST0000C
NA	NA	NA	NA	NA	start_lost	ENSG0000(	transcript	ENST0000C
NA	NA	NA	NA	NA	splice_don	ENSG0000(	transcript	ENST0000C
NA	NA	NA	NA	NA	splice_don	ENSG0000(	transcript	ENST0000C
NA	NA	NA	NA	NA	splice_don	ENSG0000(	transcript	ENST0000C
NA	Benign	not_provid	NA	NA	splice_acce	ENSG0000(	transcript	ENST0000C
NA	NA	NA	NA	NA	splice_don	ENSG0000(	transcript	ENST0000C
NA	NA	NA	NA	NA	splice_don	ENSG0000(	transcript	ENST0000C
NA	NA	NA	NA	NA	structural_	ENSG0000(	interaction	4XP7:A_91
NA	NA	NA	NA	NA	gene_fusio	ENSG0000(	gene_varia	ENSG0000C
NA	NA	NA	NA	NA	gene_fusio	ENSG0000(	gene_varia	ENSG0000C
NA	NA	NA	NA	NA	gene_fusio	ENSG0000(	gene_varia	ENSG0000C
NA	NA	NA	NA	NA	gene_fusio	ENSG0000(	gene_varia	ENSG0000C
NA	NA	NA	NA	NA	structural_	ENSG0000(	interaction	4CF6:A_13
NA	NA	NA	NA	NA	structural_	ENSG0000(	interaction	4DKC:A_50
NA	NA	NA	NA	NA	structural_	ENSG0000(	interaction	4DKD:B_98
NA	NA	NA	NA	NA	structural_	ENSG0000(	interaction	4DKF:A_10
NA	NA	NA	NA	NA	stop_gaine	ENSG0000(	transcript	ENST0000C
NA	Benign	not_specifi	0.13774	0.16374	splice_don	ENSG0000(	transcript	ENST0000C
NA	NA	NA	NA	NA	frameshift_	ENSG0000(	transcript	ENST0000C
NA	NA	NA	NA	NA	stop_gaine	ENSG0000(	transcript	ENST0000C
NA	Benign	not_provid	NA	NA	frameshift_	ENSG0000(	transcript	ENST0000C
NA	NA	NA	NA	NA	stop_gaine	ENSG0000(	transcript	ENST0000C
NA	NA	NA	NA	NA	splice_acce	ENSG0000(	transcript	ENST0000C
NA	NA	NA	NA	NA	splice_acce	ENSG0000(	transcript	ENST0000C
NA	NA	NA	NA	NA	start_lost	ENSG0000(	transcript	ENST0000C

NA	NA	NA	NA	NA	splice_acce	ENSG0000(	transcript	ENST0000C
NA	NA	NA	NA	NA	frameshift_	ENSG0000(	transcript	ENST0000C
NA	NA	NA	NA	NA	splice_acce	ENSG0000(	transcript	ENST0000C
NA	NA	NA	NA	NA	stop_gaine	ENSG0000(	transcript	ENST0000C
NA	NA	NA	NA	NA	frameshift_	ENSG0000(	transcript	ENST0000C
NA	NA	NA	NA	NA	stop_lost	ENSG0000(	transcript	ENST0000C
NA	NA	NA	NA	NA	splice_acce	ENSG0000(	transcript	ENST0000C
NA	NA	NA	NA	NA	splice_acce	ENSG0000(	transcript	ENST0000C
NA	NA	NA	NA	NA	stop_lost	ENSG0000(	transcript	ENST0000C
NA	NA	NA	NA	NA	frameshift_	ENSG0000(	transcript	ENST0000C
NA	NA	NA	NA	NA	stop_gaine	ENSG0000(	transcript	ENST0000C
NA	NA	NA	NA	NA	stop_gaine	ENSG0000(	transcript	ENST0000C
NA	NA	NA	NA	NA	stop_gaine	ENSG0000(	transcript	ENST0000C
NA	NA	NA	NA	NA	frameshift_	ENSG0000(	transcript	ENST0000C
NA	NA	NA	NA	NA	stop_gaine	ENSG0000(	transcript	ENST0000C
NA	NA	NA	NA	NA	splice_don	ENSG0000(	transcript	ENST0000C
NA	NA	NA	NA	NA	stop_gaine	ENSG0000(	transcript	ENST0000C
NA	NA	NA	NA	NA	splice_don	ENSG0000(	transcript	ENST0000C
NA	NA	NA	NA	NA	splice_don	ENSG0000(	transcript	ENST0000C
NA	NA	NA	NA	NA	stop_gaine	ENSG0000(	transcript	ENST0000C
NA	NA	NA	NA	NA	splice_don	ENSG0000(	transcript	ENST0000C
NA	Benign	Primary_cil	0.32840	0.26198	splice_don	ENSG0000(	transcript	ENST0000C
NA	Benign	not_provid	NA	NA	splice_acce	ENSG0000(	transcript	ENST0000C
NA	NA	NA	NA	NA	splice_don	ENSG0000(	transcript	ENST0000C
NA	NA	NA	NA	NA	splice_don	ENSG0000(	transcript	ENST0000C
NA	NA	NA	NA	NA	splice_acce	ENSG0000(	transcript	ENST0000C
NA	NA	NA	NA	NA	splice_acce	ENSG0000(	transcript	ENST0000C
NA	NA	NA	NA	NA	splice_acce	ENSG0000(	transcript	ENST0000C
NA	NA	NA	NA	NA	splice_acce	ENSG0000(	transcript	ENST0000C
NA	NA	NA	NA	NA	splice_don	ENSG0000(	transcript	ENST0000C
NA	NA	NA	NA	NA	frameshift_	ENSG0000(	transcript	ENST0000C
NA	NA	NA	NA	NA	frameshift_	ENSG0000(	transcript	ENST0000C
NA	NA	NA	NA	NA	splice_don	ENSG0000(	transcript	ENST0000C
NA	NA	NA	NA	NA	splice_don	ENSG0000(	transcript	ENST0000C
NA	NA	NA	NA	NA	splice_don	ENSG0000(	transcript	ENST0000C
NA	NA	NA	NA	NA	splice_acce	ENSG0000(	transcript	ENST0000C
NA	NA	NA	NA	NA	splice_don	ENSG0000(	transcript	ENST0000C
NA	NA	NA	NA	NA	splice_don	ENSG0000(	transcript	ENST0000C
NA	NA	NA	NA	NA	frameshift_	ENSG0000(	transcript	ENST0000C
NA	NA	NA	NA	NA	frameshift_	ENSG0000(	transcript	ENST0000C
NA	Benign	not_provid	NA	NA	structural_	ENSG0000(	interaction	3D4J:A_20:
NA	NA	NA	NA	NA	splice_acce	ENSG0000(	transcript	ENST0000C
NA	NA	NA	NA	NA	frameshift_	ENSG0000(	transcript	ENST0000C
NA	Benign/Like	Morquio_s	0.06169	0.04493	structural_	ENSG0000(	interaction	4FDI:A_32-
NA	Benign	Morquio_s	0.27636	0.31669	structural_	ENSG0000(	interaction	4FDJ:A_40-
NA	Benign/Like	Morquio_s	NA	0.04253	structural_	ENSG0000(	interaction	4FDI:A_138
NA	Conflicting_	Morquio_s	0.02177	0.01158	structural_	ENSG0000(	interaction	4FDI:A_106

NA	NA	NA	NA	NA	frameshift_	ENSG0000(	transcript	ENST0000C
NA	Likely_beni	not_specifi	NA	0.07508	stop_gaine	ENSG0000(	transcript	ENST0000C
NA	NA	NA	NA	NA	splice_acce	ENSG0000(	transcript	ENST0000C
NA	NA	NA	NA	NA	frameshift_	ENSG0000(	transcript	ENST0000C
NA	NA	NA	NA	NA	stop_gaine	ENSG0000(	transcript	ENST0000C
NA	NA	NA	NA	NA	splice_acce	ENSG0000(	transcript	ENST0000C
NA	NA	NA	NA	NA	frameshift_	ENSG0000(	transcript	ENST0000C
NA	NA	NA	NA	NA	frameshift_	ENSG0000(	transcript	ENST0000C
NA	NA	NA	NA	NA	frameshift_	ENSG0000(	transcript	ENST0000C
NA	NA	NA	NA	NA	frameshift_	ENSG0000(	transcript	ENST0000C
NA	NA	NA	NA	NA	splice_acce	ENSG0000(	transcript	ENST0000C
NA	NA	NA	NA	NA	splice_don	ENSG0000(	transcript	ENST0000C
NA	NA	NA	NA	NA	stop_gaine	ENSG0000(	transcript	ENST0000C
NA	NA	NA	NA	NA	stop_gaine	ENSG0000(	transcript	ENST0000C
NA	NA	NA	NA	NA	frameshift_	ENSG0000(	transcript	ENST0000C
NA	NA	NA	NA	NA	splice_don	ENSG0000(	transcript	ENST0000C
NA	NA	NA	NA	NA	frameshift_	ENSG0000(	transcript	ENST0000C
NA	NA	NA	NA	NA	splice_don	ENSG0000(	transcript	ENST0000C
NA	NA	NA	NA	NA	structural_	ENSG0000(	interaction	1UW5:A_5
NA	NA	NA	NA	NA	structural_	ENSG0000(	interaction	1UW5:A_6
NA	NA	NA	NA	NA	structural_	ENSG0000(	interaction	4IJH:A_4-A
Mean_corp	NA	NA	NA	NA	structural_	ENSG0000(	interaction	1JMC:A_35
NA	NA	NA	NA	NA	structural_	ENSG0000(	interaction	1FGU:B_30
NA	NA	NA	NA	NA	splice_don	ENSG0000(	transcript	ENST0000C
NA	NA	NA	NA	NA	start_lost	ENSG0000(	transcript	ENST0000C
NA	NA	NA	NA	NA	splice_don	ENSG0000(	transcript	ENST0000C
NA	Benign/Like	Spongy_de	NA	0.20367	structural_	ENSG0000(	interaction	2Q51:A_23
NA	Benign	Palmoplant	0.30454	0.32768	stop_gaine	ENSG0000(	transcript	ENST0000C
NA	NA	NA	NA	NA	start_lost	ENSG0000(	transcript	ENST0000C
NA	Benign	not_specifi	NA	NA	frameshift_	ENSG0000(	transcript	ENST0000C
NA	NA	NA	NA	NA	stop_gaine	ENSG0000(	transcript	ENST0000C
NA	NA	NA	NA	NA	splice_don	ENSG0000(	transcript	ENST0000C
NA	NA	NA	NA	NA	frameshift_	ENSG0000(	transcript	ENST0000C
NA	NA	NA	NA	NA	frameshift_	ENSG0000(	transcript	ENST0000C
NA	NA	NA	NA	NA	splice_don	ENSG0000(	transcript	ENST0000C
NA	NA	NA	NA	NA	splice_acce	ENSG0000(	transcript	ENST0000C
NA	NA	NA	NA	NA	splice_acce	ENSG0000(	transcript	ENST0000C
NA	NA	NA	NA	NA	splice_acce	ENSG0000(	transcript	ENST0000C
NA	NA	NA	NA	NA	stop_gaine	ENSG0000(	transcript	ENST0000C
Mean_plat	Benign/Like	CIC-DUX_S	NA	NA	splice_acce	ENSG0000(	transcript	ENST0000C
NA	Likely_beni	not_specifi	NA	NA	frameshift_	ENSG0000(	transcript	ENST0000C
NA	Likely_beni	not_specifi	NA	NA	frameshift_	ENSG0000(	transcript	ENST0000C
NA	Likely_beni	not_specifi	0.24011	NA	frameshift_	ENSG0000(	transcript	ENST0000C
NA	NA	NA	NA	NA	frameshift_	ENSG0000(	transcript	ENST0000C
NA	NA	NA	NA	NA	splice_don	ENSG0000(	transcript	ENST0000C
NA	NA	NA	NA	NA	splice_acce	ENSG0000(	transcript	ENST0000C
NA	NA	NA	NA	NA	intron_vari	ENSG0000(	transcript	ENST0000C

NA	NA	NA	NA	NA	splice_acce	ENSG0000(	transcript	ENST0000C
NA	NA	NA	NA	NA	intron_vari	ENSG0000(	transcript	ENST0000C
NA	NA	NA	NA	NA	splice_acce	ENSG0000(	transcript	ENST0000C
NA	NA	NA	NA	NA	intron_vari	ENSG0000(	transcript	ENST0000C
NA	NA	NA	NA	NA	structural_	ENSG0000(	interaction	1P32:A_13
NA	NA	NA	NA	NA	structural_	ENSG0000(	interaction	4IM6:A_94
NA	NA	NA	NA	NA	structural_	ENSG0000(	interaction	4IM6:A_94
NA	NA	NA	NA	NA	structural_	ENSG0000(	interaction	4IM6:A_87
NA	NA	NA	NA	NA	splice_acce	ENSG0000(	transcript	ENST0000C
NA	NA	NA	NA	NA	splice_don	ENSG0000(	transcript	ENST0000C
NA	NA	NA	NA	NA	splice_acce	ENSG0000(	transcript	ENST0000C
NA	NA	NA	NA	NA	splice_acce	ENSG0000(	transcript	ENST0000C
NA	NA	NA	NA	NA	splice_acce	ENSG0000(	transcript	ENST0000C
NA	NA	NA	NA	NA	splice_don	ENSG0000(	transcript	ENST0000C
NA	Benign	not_provid	NA	NA	splice_don	ENSG0000(	transcript	ENST0000C
NA	NA	NA	NA	NA	splice_don	ENSG0000(	transcript	ENST0000C
NA	NA	NA	NA	NA	gene_fusio	ENSG0000(	gene_varia	ENSG0000C
NA	NA	NA	NA	NA	structural_	ENSG0000(	interaction	3I4W:A_36
NA	NA	NA	NA	NA	frameshift_	ENSG0000(	transcript	ENST0000C
NA	NA	NA	NA	NA	stop_lost	ENSG0000(	transcript	ENST0000C
NA	NA	NA	NA	NA	stop_gaine	ENSG0000(	transcript	ENST0000C
NA	NA	NA	NA	NA	frameshift_	ENSG0000(	transcript	ENST0000C
NA	NA	NA	NA	NA	frameshift_	ENSG0000(	transcript	ENST0000C
NA	NA	NA	NA	NA	frameshift_	ENSG0000(	transcript	ENST0000C
NA	NA	NA	NA	NA	structural_	ENSG0000(	interaction	4NSW:A_1
NA	NA	NA	NA	NA	splice_don	ENSG0000(	transcript	ENST0000C
NA	NA	NA	NA	NA	splice_acce	ENSG0000(	transcript	ENST0000C
NA	NA	NA	NA	NA	frameshift_	ENSG0000(	transcript	ENST0000C
NA	NA	NA	NA	NA	stop_gaine	ENSG0000(	transcript	ENST0000C
NA	NA	NA	NA	NA	frameshift_	ENSG0000(	transcript	ENST0000C
NA	NA	NA	NA	NA	splice_acce	ENSG0000(	transcript	ENST0000C
NA	NA	NA	NA	NA	splice_acce	ENSG0000(	transcript	ENST0000C
NA	NA	NA	NA	NA	splice_acce	ENSG0000(	transcript	ENST0000C
NA	NA	NA	NA	NA	splice_acce	ENSG0000(	transcript	ENST0000C
NA	NA	NA	NA	NA	splice_don	ENSG0000(	transcript	ENST0000C
NA	NA	NA	NA	NA	splice_don	ENSG0000(	transcript	ENST0000C
NA	NA	NA	NA	NA	splice_don	ENSG0000(	transcript	ENST0000C
NA	NA	NA	NA	NA	splice_don	ENSG0000(	transcript	ENST0000C
NA	NA	NA	NA	NA	splice_don	ENSG0000(	transcript	ENST0000C
NA	NA	NA	NA	NA	splice_acce	ENSG0000(	transcript	ENST0000C
NA	NA	NA	NA	NA	splice_acce	ENSG0000(	transcript	ENST0000C
NA	NA	NA	NA	NA	splice_acce	ENSG0000(	transcript	ENST0000C
NA	NA	NA	NA	NA	splice_acce	ENSG0000(	transcript	ENST0000C
NA	NA	NA	NA	NA	splice_acce	ENSG0000(	transcript	ENST0000C
NA	NA	NA	NA	NA	splice_acce	ENSG0000(	transcript	ENST0000C
NA	NA	NA	NA	NA	splice_acce	ENSG0000(	transcript	ENST0000C
NA	NA	NA	NA	NA	splice_acce	ENSG0000(	transcript	ENST0000C

NA	NA	NA	NA	NA	splice_acce	ENSG0000(	transcript	ENST0000C
NA	NA	NA	NA	NA	splice_acce	ENSG0000(	transcript	ENST0000C
NA	NA	NA	NA	NA	splice_acce	ENSG0000(	transcript	ENST0000C
NA	NA	NA	NA	NA	splice_acce	ENSG0000(	transcript	ENST0000C
NA	NA	NA	NA	NA	splice_acce	ENSG0000(	transcript	ENST0000C
NA	NA	NA	NA	NA	splice_acce	ENSG0000(	transcript	ENST0000C
NA	NA	NA	NA	NA	splice_acce	ENSG0000(	transcript	ENST0000C
NA	NA	NA	NA	NA	splice_acce	ENSG0000(	transcript	ENST0000C
NA	NA	NA	NA	NA	splice_acce	ENSG0000(	transcript	ENST0000C
NA	NA	NA	NA	NA	splice_acce	ENSG0000(	transcript	ENST0000C
NA	NA	NA	NA	NA	splice_acce	ENSG0000(	transcript	ENST0000C
NA	NA	NA	NA	NA	splice_acce	ENSG0000(	transcript	ENST0000C
NA	NA	NA	NA	NA	splice_acce	ENSG0000(	transcript	ENST0000C
NA	NA	NA	NA	NA	splice_acce	ENSG0000(	transcript	ENST0000C
NA	NA	NA	NA	NA	splice_acce	ENSG0000(	transcript	ENST0000C
NA	NA	NA	NA	NA	splice_acce	ENSG0000(	transcript	ENST0000C
NA	NA	NA	NA	NA	splice_acce	ENSG0000(	transcript	ENST0000C
NA	NA	NA	NA	NA	splice_acce	ENSG0000(	transcript	ENST0000C
NA	NA	NA	NA	NA	splice_acce	ENSG0000(	transcript	ENST0000C
NA	NA	NA	NA	NA	splice_acce	ENSG0000(	transcript	ENST0000C
NA	NA	NA	NA	NA	splice_acce	ENSG0000(	transcript	ENST0000C
NA	NA	NA	NA	NA	splice_acce	ENSG0000(	transcript	ENST0000C
NA	NA	NA	NA	NA	splice_acce	ENSG0000(	transcript	ENST0000C
NA	NA	NA	NA	NA	splice_acce	ENSG0000(	transcript	ENST0000C
NA	NA	NA	NA	NA	splice_acce	ENSG0000(	transcript	ENST0000C
NA	NA	NA	NA	NA	structural_	ENSG0000(	interaction	2G9N:A_13
NA	NA	NA	NA	NA	frameshift_	ENSG0000(	transcript	ENST0000C
NA	NA	NA	NA	NA	structural_	ENSG0000(	interaction	2BEI:A_89-
NA	NA	NA	NA	NA	frameshift_	ENSG0000(	transcript	ENST0000C
NA	NA	NA	NA	NA	frameshift_	ENSG0000(	transcript	ENST0000C
NA	NA	NA	NA	NA	frameshift_	ENSG0000(	transcript	ENST0000C
NA	NA	NA	NA	NA	stop_gaine	ENSG0000(	transcript	ENST0000C
NA	NA	NA	NA	NA	stop_gaine	ENSG0000(	transcript	ENST0000C
NA	Benign/Like	Li-Fraumen	0.01204	0.00539	structural_	ENSG0000(	interaction	1GZH:A_16
NA	NA	NA	NA	NA	frameshift_	ENSG0000(	transcript	ENST0000C
NA	Likely_beni	not_provid	NA	NA	stop_gaine	ENSG0000(	transcript	ENST0000C
NA	NA	NA	NA	NA	structural_	ENSG0000(	interaction	4NRE:A_86
NA	NA	NA	NA	NA	structural_	ENSG0000(	interaction	4NRE:A_68
NA	NA	NA	NA	NA	structural_	ENSG0000(	interaction	4NRE:A_23
NA	NA	NA	NA	NA	structural_	ENSG0000(	interaction	4NRE:A_48
NA	NA	NA	NA	NA	structural_	ENSG0000(	interaction	4NRE:A_55
NA	NA	NA	NA	NA	structural_	ENSG0000(	interaction	4NRE:A_24
NA	NA	NA	NA	NA	structural_	ENSG0000(	interaction	4NRE:A_17
NA	NA	NA	NA	NA	splice_don	ENSG0000(	transcript	ENST0000C
NA	NA	NA	NA	NA	stop_gaine	ENSG0000(	transcript	ENST0000C
NA	NA	NA	NA	NA	splice_don	ENSG0000(	transcript	ENST0000C
NA	Benign	not_provid	NA	NA	frameshift_	ENSG0000(	transcript	ENST0000C

NA	NA	NA	NA	NA	frameshift_	ENSG0000(	transcript	ENST0000C
NA	NA	NA	NA	NA	frameshift_	ENSG0000(	transcript	ENST0000C
NA	NA	NA	NA	NA	splice_don	ENSG0000(	transcript	ENST0000C
NA	Benign	Leigh_synd	0.52231	0.49181	stop_gaine	ENSG0000(	transcript	ENST0000C
NA	NA	NA	NA	NA	splice_don	ENSG0000(	transcript	ENST0000C
NA	NA	NA	NA	NA	splice_acce	ENSG0000(	transcript	ENST0000C
NA	NA	NA	NA	NA	splice_don	ENSG0000(	transcript	ENST0000C
Reaction_t	Benign	History_of_	0.48727	NA	frameshift_	ENSG0000(	transcript	ENST0000C
Reaction_t	Benign	History_of_	0.48727	NA	frameshift_	ENSG0000(	transcript	ENST0000C
NA	Benign	Smith-Mag	0.41218	NA	frameshift_	ENSG0000(	transcript	ENST0000C
NA	Benign	Smith-Mag	0.41218	NA	frameshift_	ENSG0000(	transcript	ENST0000C
NA	NA	NA	NA	NA	frameshift_	ENSG0000(	transcript	ENST0000C
NA	NA	NA	NA	NA	structural_	ENSG0000(	interaction	4CGY:A_26
NA	NA	NA	NA	NA	splice_don	ENSG0000(	transcript	ENST0000C
NA	NA	NA	NA	NA	frameshift_	ENSG0000(	transcript	ENST0000C
NA	NA	NA	NA	NA	splice_don	ENSG0000(	transcript	ENST0000C
NA	NA	NA	NA	NA	splice_don	ENSG0000(	transcript	ENST0000C
NA	Benign	SjÃƒÂ¶gren	0.61541	0.67851	stop_gaine	ENSG0000(	transcript	ENST0000C
NA	NA	NA	NA	NA	splice_don	ENSG0000(	transcript	ENST0000C
NA	NA	NA	NA	NA	frameshift_	ENSG0000(	transcript	ENST0000C
NA	NA	NA	NA	NA	splice_acce	ENSG0000(	transcript	ENST0000C
NA	NA	NA	NA	NA	splice_don	ENSG0000(	transcript	ENST0000C
NA	NA	NA	NA	NA	splice_don	ENSG0000(	transcript	ENST0000C
NA	NA	NA	NA	NA	splice_don	ENSG0000(	transcript	ENST0000C
NA	NA	NA	NA	NA	splice_don	ENSG0000(	transcript	ENST0000C
NA	NA	NA	NA	NA	splice_don	ENSG0000(	transcript	ENST0000C
NA	Benign/Like	Cone-Rod_	0.00086	0.00120	structural_	ENSG0000(	interaction	3GQQ:A_89
NA	NA	NA	NA	NA	splice_acce	ENSG0000(	transcript	ENST0000C
NA	NA	NA	NA	NA	frameshift_	ENSG0000(	transcript	ENST0000C
NA	NA	NA	NA	NA	splice_acce	ENSG0000(	transcript	ENST0000C
NA	NA	NA	NA	NA	splice_acce	ENSG0000(	transcript	ENST0000C
NA	NA	NA	NA	NA	splice_don	ENSG0000(	transcript	ENST0000C
NA	NA	NA	NA	NA	frameshift_	ENSG0000(	transcript	ENST0000C
NA	NA	NA	NA	NA	splice_don	ENSG0000(	transcript	ENST0000C
NA	NA	NA	NA	NA	start_lost	ENSG0000(	transcript	ENST0000C
NA	not_provid	not_provid	0.00702	0.00140	splice_don	ENSG0000(	transcript	ENST0000C
Breast_can	NA	NA	NA	NA	splice_acce	ENSG0000(	transcript	ENST0000C
NA	NA	NA	NA	NA	splice_acce	ENSG0000(	transcript	ENST0000C
NA	NA	NA	NA	NA	splice_acce	ENSG0000(	transcript	ENST0000C
NA	NA	NA	NA	NA	splice_acce	ENSG0000(	transcript	ENST0000C
NA	NA	NA	NA	NA	splice_acce	ENSG0000(	transcript	ENST0000C
NA	NA	NA	NA	NA	splice_acce	ENSG0000(	transcript	ENST0000C
NA	NA	NA	NA	NA	stop_lost	ENSG0000(	transcript	ENST0000C
NA	NA	NA	NA	NA	splice_don	ENSG0000(	transcript	ENST0000C
NA	NA	NA	NA	NA	frameshift_	ENSG0000(	transcript	ENST0000C
NA	NA	NA	NA	NA	splice_acce	ENSG0000(	transcript	ENST0000C
NA	NA	NA	NA	NA	splice_acce	ENSG0000(	transcript	ENST0000C

NA	NA	NA	NA	NA	bidirection	ENSG0000(	gene_varia	ENSG00000
NA	NA	NA	NA	NA	bidirection	ENSG0000(	gene_varia	ENSG00000
NA	NA	NA	NA	NA	bidirection	ENSG0000(	gene_varia	ENSG00000
NA	NA	NA	NA	NA	bidirection	ENSG0000(	gene_varia	ENSG00000
NA	NA	NA	NA	NA	bidirection	ENSG0000(	gene_varia	ENSG00000
NA	NA	NA	NA	NA	bidirection	ENSG0000(	gene_varia	ENSG00000
NA	NA	NA	NA	NA	splice_acce	ENSG0000(	transcript	ENST00000
NA	NA	NA	NA	NA	splice_acce	ENSG0000(	transcript	ENST00000
NA	NA	NA	NA	NA	stop_gaine	ENSG0000(	transcript	ENST00000
NA	NA	NA	NA	NA	frameshift_	ENSG0000(	transcript	ENST00000
NA	NA	NA	NA	NA	start_lost	ENSG0000(	transcript	ENST00000
NA	NA	NA	NA	NA	splice_don	ENSG0000(	transcript	ENST00000
NA	NA	NA	NA	NA	structural_	ENSG0000(	interaction	1E42:A_70
NA	NA	NA	NA	NA	structural_	ENSG0000(	interaction	3I35:A_207
NA	NA	NA	NA	NA	splice_don	ENSG0000(	transcript	ENST00000
NA	NA	NA	NA	NA	splice_don	ENSG0000(	transcript	ENST00000
NA	NA	NA	NA	NA	frameshift_	ENSG0000(	transcript	ENST00000
NA	NA	NA	NA	NA	stop_lost	ENSG0000(	transcript	ENST00000
NA	NA	NA	NA	NA	structural_	ENSG0000(	interaction	1EM2:A_28
NA	NA	NA	NA	NA	structural_	ENSG0000(	interaction	1HNN:A_97
NA	Benign	not_provid	NA	NA	structural_	ENSG0000(	interaction	1N7I:A_175
NA	NA	NA	NA	NA	splice_acce	ENSG0000(	transcript	ENST00000
NA	NA	NA	NA	NA	frameshift_	ENSG0000(	transcript	ENST00000
NA	NA	NA	NA	NA	frameshift_	ENSG0000(	transcript	ENST00000
NA	NA	NA	NA	NA	frameshift_	ENSG0000(	transcript	ENST00000
NA	NA	NA	NA	NA	frameshift_	ENSG0000(	transcript	ENST00000
NA	NA	NA	NA	NA	frameshift_	ENSG0000(	transcript	ENST00000
NA	NA	NA	NA	NA	frameshift_	ENSG0000(	transcript	ENST00000
NA	NA	NA	NA	NA	frameshift_	ENSG0000(	transcript	ENST00000
NA	NA	NA	NA	NA	frameshift_	ENSG0000(	transcript	ENST00000
NA	NA	NA	NA	NA	frameshift_	ENSG0000(	transcript	ENST00000
NA	NA	NA	NA	NA	splice_acce	ENSG0000(	transcript	ENST00000
Lymphocyt	NA	NA	NA	NA	start_lost	ENSG0000(	transcript	ENST00000
NA	NA	NA	NA	NA	frameshift_	ENSG0000(	transcript	ENST00000
NA	NA	NA	NA	NA	frameshift_	ENSG0000(	transcript	ENST00000
NA	NA	NA	NA	NA	frameshift_	ENSG0000(	transcript	ENST00000
NA	NA	NA	NA	NA	frameshift_	ENSG0000(	transcript	ENST00000
NA	NA	NA	NA	NA	splice_acce	ENSG0000(	transcript	ENST00000
NA	NA	NA	NA	NA	start_lost	ENSG0000(	transcript	ENST00000
NA	NA	NA	NA	NA	splice_don	ENSG0000(	transcript	ENST00000
NA	NA	NA	NA	NA	splice_acce	ENSG0000(	transcript	ENST00000
NA	Likely_beni	not_provid	NA	NA	structural_	ENSG0000(	interaction	3MWD:B_6
NA	NA	NA	NA	NA	structural_	ENSG0000(	interaction	3MWD:B_5
NA	NA	NA	NA	NA	splice_don	ENSG0000(	transcript	ENST00000
NA	NA	NA	NA	NA	structural_	ENSG0000(	interaction	2W4R:C_54
NA	Benign	not_provid	NA	NA	splice_acce	ENSG0000(	transcript	ENST00000
NA	NA	NA	NA	NA	frameshift_	ENSG0000(	transcript	ENST00000

NA	NA	NA	NA	NA	splice_don	ENSG0000(	transcript	ENST0000C
NA	NA	NA	NA	NA	frameshift_	ENSG0000(	transcript	ENST0000C
NA	NA	NA	NA	NA	frameshift_	ENSG0000(	transcript	ENST0000C
NA	NA	NA	NA	NA	frameshift_	ENSG0000(	transcript	ENST0000C
NA	NA	NA	NA	NA	frameshift_	ENSG0000(	transcript	ENST0000C
NA	NA	NA	NA	NA	frameshift_	ENSG0000(	transcript	ENST0000C
NA	NA	NA	NA	NA	frameshift_	ENSG0000(	transcript	ENST0000C
NA	NA	NA	NA	NA	frameshift_	ENSG0000(	transcript	ENST0000C
NA	NA	NA	NA	NA	structural_	ENSG0000(	interaction	2C11:B_39
NA	NA	NA	NA	NA	splice_don	ENSG0000(	transcript	ENST0000C
NA	Benign	Hereditary_	0.01762	0.01118	structural_	ENSG0000(	interaction	1JNX:X_16
NA	NA	NA	NA	NA	splice_don	ENSG0000(	transcript	ENST0000C
NA	NA	NA	NA	NA	structural_	ENSG0000(	interaction	3I4U:A_10
NA	NA	NA	NA	NA	splice_acce	ENSG0000(	transcript	ENST0000C
NA	NA	NA	NA	NA	splice_acce	ENSG0000(	transcript	ENST0000C
NA	NA	NA	NA	NA	structural_	ENSG0000(	interaction	1J4X:A_61-
NA	NA	NA	NA	NA	splice_don	ENSG0000(	transcript	ENST0000C
NA	NA	NA	NA	NA	splice_acce	ENSG0000(	transcript	ENST0000C
NA	NA	NA	NA	NA	splice_don	ENSG0000(	transcript	ENST0000C
NA	NA	NA	NA	NA	splice_don	ENSG0000(	transcript	ENST0000C
NA	NA	NA	NA	NA	stop_gaine	ENSG0000(	transcript	ENST0000C
NA	NA	NA	NA	NA	stop_gaine	ENSG0000(	transcript	ENST0000C
NA	NA	NA	NA	NA	splice_acce	ENSG0000(	transcript	ENST0000C
NA	NA	NA	NA	NA	stop_gaine	ENSG0000(	transcript	ENST0000C
NA	NA	NA	NA	NA	stop_gaine	ENSG0000(	transcript	ENST0000C
NA	NA	NA	NA	NA	structural_	ENSG0000(	interaction	3W5K:A_1
NA	NA	NA	NA	NA	structural_	ENSG0000(	interaction	1QGK:A_54
NA	Benign	not_provid	NA	NA	frameshift_	ENSG0000(	transcript	ENST0000C
NA	NA	NA	NA	NA	frameshift_	ENSG0000(	transcript	ENST0000C
NA	NA	NA	NA	NA	splice_don	ENSG0000(	transcript	ENST0000C
NA	NA	NA	NA	NA	structural_	ENSG0000(	interaction	4MP2:A_8
NA	Uncertain_	Autosomal_	0.00418	0.00459	frameshift_	ENSG0000(	transcript	ENST0000C
NA	NA	NA	NA	NA	splice_acce	ENSG0000(	transcript	ENST0000C
NA	NA	NA	NA	NA	splice_acce	ENSG0000(	transcript	ENST0000C
NA	NA	NA	NA	NA	splice_acce	ENSG0000(	transcript	ENST0000C
NA	NA	NA	NA	NA	splice_acce	ENSG0000(	transcript	ENST0000C
NA	NA	NA	NA	NA	splice_acce	ENSG0000(	transcript	ENST0000C
NA	NA	NA	NA	NA	structural_	ENSG0000(	interaction	2ZIU:B_311
NA	NA	NA	NA	NA	splice_don	ENSG0000(	transcript	ENST0000C
White_mat	NA	NA	NA	NA	splice_don	ENSG0000(	transcript	ENST0000C
NA	NA	NA	NA	NA	splice_acce	ENSG0000(	transcript	ENST0000C
NA	NA	NA	NA	NA	structural_	ENSG0000(	interaction	1LN2:A_10
NA	NA	NA	NA	NA	structural_	ENSG0000(	interaction	1LN1:A_13
NA	NA	NA	NA	NA	frameshift_	ENSG0000(	transcript	ENST0000C
NA	NA	NA	NA	NA	disruptive_	ENSG0000(	transcript	ENST0000C
NA	NA	NA	NA	NA	gene_fusio	ENSG0000(	gene_varia	ENSG0000C
NA	NA	NA	NA	NA	gene_fusio	ENSG0000(	gene_varia	ENSG0000C

NA	NA	NA	NA	NA	gene_fusio	ENSG0000(	gene_varia	ENSG00000(
NA	NA	NA	NA	NA	gene_fusio	ENSG0000(	gene_varia	ENSG00000(
NA	NA	NA	NA	NA	gene_fusio	ENSG0000(	gene_varia	ENSG00000(
NA	Benign	not_provid	NA	NA	stop_gaine	ENSG0000(	transcript	ENST0000(
Blood_prot	Pathogenic	Myelopero	0.00426	0.00080	splice_acce	ENSG0000(	transcript	ENST0000(
NA	NA	NA	NA	NA	splice_acce	ENSG0000(	transcript	ENST0000(
NA	NA	NA	NA	NA	structural_	ENSG0000(	interaction	3H7H:A_5-.
NA	Benign/Like	not_specifi	NA	0.00200	stop_gaine	ENSG0000(	transcript	ENST0000(
NA	NA	NA	NA	NA	frameshift_	ENSG0000(	transcript	ENST0000(
NA	NA	NA	NA	NA	splice_don	ENSG0000(	transcript	ENST0000(
NA	NA	NA	NA	NA	splice_don	ENSG0000(	transcript	ENST0000(
NA	NA	NA	NA	NA	splice_acce	ENSG0000(	transcript	ENST0000(
Osteoarthr	NA	NA	NA	NA	stop_gaine	ENSG0000(	transcript	ENST0000(
NA	NA	NA	NA	NA	splice_acce	ENSG0000(	transcript	ENST0000(
NA	NA	NA	NA	NA	splice_acce	ENSG0000(	transcript	ENST0000(
NA	NA	NA	NA	NA	splice_acce	ENSG0000(	transcript	ENST0000(
NA	NA	NA	NA	NA	splice_acce	ENSG0000(	transcript	ENST0000(
NA	NA	NA	NA	NA	splice_don	ENSG0000(	transcript	ENST0000(
NA	NA	NA	NA	NA	stop_gaine	ENSG0000(	transcript	ENST0000(
NA	NA	NA	NA	NA	frameshift_	ENSG0000(	transcript	ENST0000(
NA	NA	NA	NA	NA	splice_don	ENSG0000(	transcript	ENST0000(
NA	NA	NA	NA	NA	frameshift_	ENSG0000(	transcript	ENST0000(
NA	NA	NA	NA	NA	structural_	ENSG0000(	interaction	3P23:A_80
PR_interva	NA	NA	NA	NA	splice_acce	ENSG0000(	transcript	ENST0000(
PR_interva	NA	NA	NA	NA	splice_acce	ENSG0000(	transcript	ENST0000(
NA	NA	NA	NA	NA	splice_acce	ENSG0000(	transcript	ENST0000(
LDL_choles	NA	NA	NA	NA	structural_	ENSG0000(	interaction	4JHS:A_30(
NA	NA	NA	NA	NA	stop_lost	ENSG0000(	transcript	ENST0000(
NA	NA	NA	NA	NA	structural_	ENSG0000(	interaction	3WPT:A_2
NA	NA	NA	NA	NA	splice_acce	ENSG0000(	transcript	ENST0000(
NA	NA	NA	NA	NA	splice_acce	ENSG0000(	transcript	ENST0000(
NA	Likely_beni	not_provid	NA	NA	frameshift_	ENSG0000(	transcript	ENST0000(
NA	Benign/Like	Hereditary_	NA	NA	frameshift_	ENSG0000(	transcript	ENST0000(
NA	NA	NA	NA	NA	splice_acce	ENSG0000(	transcript	ENST0000(
NA	Benign	not_specifi	NA	NA	frameshift_	ENSG0000(	transcript	ENST0000(
NA	NA	NA	NA	NA	stop_gaine	ENSG0000(	transcript	ENST0000(
NA	NA	NA	NA	NA	splice_acce	ENSG0000(	transcript	ENST0000(
NA	NA	NA	NA	NA	frameshift_	ENSG0000(	transcript	ENST0000(
NA	NA	NA	NA	NA	splice_don	ENSG0000(	transcript	ENST0000(
NA	NA	NA	NA	NA	stop_gaine	ENSG0000(	transcript	ENST0000(
NA	NA	NA	NA	NA	splice_don	ENSG0000(	transcript	ENST0000(
NA	NA	NA	NA	NA	start_lost	ENSG0000(	transcript	ENST0000(
NA	NA	NA	NA	NA	splice_don	ENSG0000(	transcript	ENST0000(
NA	NA	NA	NA	NA	frameshift_	ENSG0000(	transcript	ENST0000(
NA	NA	NA	NA	NA	splice_don	ENSG0000(	transcript	ENST0000(
NA	NA	NA	NA	NA	splice_don	ENSG0000(	transcript	ENST0000(
NA	NA	NA	NA	NA	splice_don	ENSG0000(	transcript	ENST0000(

NA	NA	NA	NA	NA	splice_don	ENSG0000(	transcript	ENST0000C
NA	NA	NA	NA	NA	splice_acce	ENSG0000(	transcript	ENST0000C
NA	NA	NA	NA	NA	splice_acce	ENSG0000(	transcript	ENST0000C
NA	NA	NA	NA	NA	frameshift_	ENSG0000(	transcript	ENST0000C
NA	NA	NA	NA	NA	splice_don	ENSG0000(	transcript	ENST0000C
NA	NA	NA	NA	NA	splice_don	ENSG0000(	transcript	ENST0000C
NA	NA	NA	NA	NA	splice_acce	ENSG0000(	transcript	ENST0000C
NA	NA	NA	NA	NA	frameshift_	ENSG0000(	transcript	ENST0000C
NA	NA	NA	NA	NA	frameshift_	ENSG0000(	transcript	ENST0000C
NA	NA	NA	NA	NA	frameshift_	ENSG0000(	transcript	ENST0000C
NA	NA	NA	NA	NA	frameshift_	ENSG0000(	transcript	ENST0000C
NA	NA	NA	NA	NA	frameshift_	ENSG0000(	transcript	ENST0000C
NA	NA	NA	NA	NA	frameshift_	ENSG0000(	transcript	ENST0000C
NA	NA	NA	NA	NA	start_lost	ENSG0000(	transcript	ENST0000C
NA	NA	NA	NA	NA	structural_	ENSG0000(	interaction	2I7D:A_15-
NA	NA	NA	NA	NA	structural_	ENSG0000(	interaction	1P4U:A_58
NA	NA	NA	NA	NA	stop_gaine	ENSG0000(	transcript	ENST0000C
NA	NA	NA	NA	NA	frameshift_	ENSG0000(	transcript	ENST0000C
NA	NA	NA	NA	NA	stop_gaine	ENSG0000(	transcript	ENST0000C
NA	NA	NA	NA	NA	splice_don	ENSG0000(	transcript	ENST0000C
NA	NA	NA	NA	NA	stop_gaine	ENSG0000(	transcript	ENST0000C
NA	NA	NA	NA	NA	frameshift_	ENSG0000(	transcript	ENST0000C
NA	NA	NA	NA	NA	frameshift_	ENSG0000(	transcript	ENST0000C
NA	NA	NA	NA	NA	splice_don	ENSG0000(	transcript	ENST0000C
NA	Likely_beni	Epidermoly	0.02926	0.01518	structural_	ENSG0000(	interaction	3F7Q:A_12
NA	NA	NA	NA	NA	stop_gaine	ENSG0000(	transcript	ENST0000C
NA	NA	NA	NA	NA	splice_acce	ENSG0000(	transcript	ENST0000C
NA	NA	NA	NA	NA	splice_acce	ENSG0000(	transcript	ENST0000C
NA	NA	NA	NA	NA	structural_	ENSG0000(	interaction	4V24:A_37
NA	NA	NA	NA	NA	structural_	ENSG0000(	interaction	3VZB:A_20
NA	NA	NA	NA	NA	structural_	ENSG0000(	interaction	4V24:A_23
NA	NA	NA	NA	NA	splice_acce	ENSG0000(	transcript	ENST0000C
NA	NA	NA	NA	NA	splice_don	ENSG0000(	transcript	ENST0000C
NA	NA	NA	NA	NA	splice_don	ENSG0000(	transcript	ENST0000C
NA	NA	NA	NA	NA	splice_don	ENSG0000(	transcript	ENST0000C
NA	NA	NA	NA	NA	structural_	ENSG0000(	interaction	1W4R:A_5:
NA	NA	NA	NA	NA	stop_gaine	ENSG0000(	transcript	ENST0000C
NA	NA	NA	NA	NA	splice_acce	ENSG0000(	transcript	ENST0000C
NA	NA	NA	NA	NA	frameshift_	ENSG0000(	transcript	ENST0000C
NA	NA	NA	NA	NA	frameshift_	ENSG0000(	transcript	ENST0000C
NA	NA	NA	NA	NA	frameshift_	ENSG0000(	transcript	ENST0000C
NA	NA	NA	NA	NA	stop_lost	ENSG0000(	transcript	ENST0000C
NA	NA	NA	NA	NA	structural_	ENSG0000(	interaction	2HYI:C_111
NA	Benign	Pityriasis_r	0.03658	0.02196	stop_gaine	ENSG0000(	transcript	ENST0000C
NA	NA	NA	NA	NA	stop_gaine	ENSG0000(	transcript	ENST0000C
NA	Benign	not_specifi	0.01328	0.01018	structural_	ENSG0000(	interaction	4MHX:A_3:
NA	NA	NA	NA	NA	structural_	ENSG0000(	interaction	4NSP:A_29

NA	NA	NA	NA	NA	stop_lost	ENSG0000(	transcript	ENST0000C
NA	NA	NA	NA	NA	bidirection	ENSG0000(	gene_varia	ENSG0000C
NA	NA	NA	NA	NA	splice_don	ENSG0000(	transcript	ENST0000C
NA	NA	NA	NA	NA	splice_don	ENSG0000(	transcript	ENST0000C
NA	NA	NA	NA	NA	frameshift_	ENSG0000(	transcript	ENST0000C
NA	NA	NA	NA	NA	frameshift_	ENSG0000(	transcript	ENST0000C
NA	NA	NA	NA	NA	splice_acce	ENSG0000(	transcript	ENST0000C
Lung_funct	NA	NA	NA	NA	splice_acce	ENSG0000(	transcript	ENST0000C
NA	Uncertain_	not_provid	0.00024	NA	stop_gaine	ENSG0000(	transcript	ENST0000C
NA	NA	NA	NA	NA	splice_acce	ENSG0000(	transcript	ENST0000C
NA	NA	NA	NA	NA	frameshift_	ENSG0000(	transcript	ENST0000C
NA	NA	NA	NA	NA	frameshift_	ENSG0000(	transcript	ENST0000C
NA	NA	NA	NA	NA	stop_lost	ENSG0000(	transcript	ENST0000C
NA	NA	NA	NA	NA	structural_	ENSG0000(	interaction	3UEM:A_2
NA	NA	NA	NA	NA	splice_acce	ENSG0000(	transcript	ENST0000C
NA	NA	NA	NA	NA	structural_	ENSG0000(	interaction	4UYU:B_33
NA	NA	NA	NA	NA	protein_pr	ENSG0000(	interaction	4DRB:A_40
NA	NA	NA	NA	NA	protein_pr	ENSG0000(	interaction	4DRA:C_33
NA	NA	NA	NA	NA	structural_	ENSG0000(	interaction	2C2H:A_22
NA	NA	NA	NA	NA	structural_	ENSG0000(	interaction	1WNT:A_8
NA	NA	NA	NA	NA	frameshift_	ENSG0000(	transcript	ENST0000C
NA	Likely_beni	Epileptic_e	0.00113	0.00020	structural_	ENSG0000(	interaction	4W82:A_1
NA	NA	NA	NA	NA	structural_	ENSG0000(	interaction	4W82:A_1
NA	NA	NA	NA	NA	structural_	ENSG0000(	interaction	4PIV:A_12
NA	NA	NA	NA	NA	structural_	ENSG0000(	interaction	3HHD:A_1
NA	NA	NA	NA	NA	stop_gaine	ENSG0000(	transcript	ENST0000C
NA	NA	NA	NA	NA	splice_acce	ENSG0000(	transcript	ENST0000C
NA	NA	NA	NA	NA	structural_	ENSG0000(	interaction	3UZP:A_10
NA	NA	NA	NA	NA	splice_acce	ENSG0000(	transcript	ENST0000C
NA	NA	NA	NA	NA	splice_acce	ENSG0000(	transcript	ENST0000C
NA	NA	NA	NA	NA	splice_acce	ENSG0000(	transcript	ENST0000C
NA	NA	NA	NA	NA	splice_acce	ENSG0000(	transcript	ENST0000C
NA	NA	NA	NA	NA	splice_acce	ENSG0000(	transcript	ENST0000C
NA	NA	NA	NA	NA	splice_acce	ENSG0000(	transcript	ENST0000C
NA	NA	NA	NA	NA	splice_don	ENSG0000(	transcript	ENST0000C
NA	NA	NA	NA	NA	frameshift_	ENSG0000(	transcript	ENST0000C
NA	NA	NA	NA	NA	splice_don	ENSG0000(	transcript	ENST0000C
NA	Benign/Like	Hypertropl	0.00159	0.00060	structural_	ENSG0000(	interaction	2R15:A_15
NA	Benign	not_specifi	0.21420	0.18351	structural_	ENSG0000(	interaction	2R15:A_14
NA	Benign	not_specifi	0.07287	0.05212	structural_	ENSG0000(	interaction	3RBS:A_13
NA	NA	NA	NA	NA	splice_don	ENSG0000(	transcript	ENST0000C
NA	Benign	not_provid	NA	NA	splice_don	ENSG0000(	transcript	ENST0000C
NA	Benign	not_specifi	0.32956	0.25559	frameshift_	ENSG0000(	transcript	ENST0000C
NA	NA	NA	NA	NA	frameshift_	ENSG0000(	transcript	ENST0000C
NA	NA	NA	NA	NA	splice_don	ENSG0000(	transcript	ENST0000C
NA	NA	NA	NA	NA	splice_don	ENSG0000(	transcript	ENST0000C
NA	NA	NA	NA	NA	splice_acce	ENSG0000(	transcript	ENST0000C

NA	NA	NA	NA	NA	frameshift_	ENSG0000(	transcript	ENST0000C
NA	NA	NA	NA	NA	splice_don	ENSG0000(	transcript	ENST0000C
NA	NA	NA	NA	NA	bidirection	ENSG0000(	gene_varia	ENSG0000C
NA	NA	NA	NA	NA	bidirection	ENSG0000(	gene_varia	ENSG0000C
NA	NA	NA	NA	NA	bidirection	ENSG0000(	gene_varia	ENSG0000C
NA	NA	NA	NA	NA	exon_loss_	ENSG0000(	transcript	ENST0000C
NA	NA	NA	NA	NA	splice_acce	ENSG0000(	transcript	ENST0000C
NA	NA	NA	NA	NA	splice_acce	ENSG0000(	transcript	ENST0000C
NA	NA	NA	NA	NA	structural_	ENSG0000(	interaction	2CZH:B_14
NA	NA	NA	NA	NA	splice_acce	ENSG0000(	transcript	ENST0000C
NA	NA	NA	NA	NA	splice_acce	ENSG0000(	transcript	ENST0000C
NA	NA	NA	NA	NA	splice_acce	ENSG0000(	transcript	ENST0000C
NA	NA	NA	NA	NA	splice_acce	ENSG0000(	transcript	ENST0000C
NA	NA	NA	NA	NA	splice_acce	ENSG0000(	transcript	ENST0000C
NA	NA	NA	NA	NA	splice_acce	ENSG0000(	transcript	ENST0000C
NA	NA	NA	NA	NA	splice_don	ENSG0000(	transcript	ENST0000C
NA	NA	NA	NA	NA	frameshift_	ENSG0000(	transcript	ENST0000C
NA	NA	NA	NA	NA	splice_don	ENSG0000(	transcript	ENST0000C
NA	NA	NA	NA	NA	splice_acce	ENSG0000(	transcript	ENST0000C
NA	NA	NA	NA	NA	splice_acce	ENSG0000(	transcript	ENST0000C
NA	NA	NA	NA	NA	splice_acce	ENSG0000(	transcript	ENST0000C
NA	NA	NA	NA	NA	start_lost	ENSG0000(	transcript	ENST0000C
NA	NA	NA	NA	NA	splice_don	ENSG0000(	transcript	ENST0000C
NA	Benign	not_provid	NA	NA	bidirection	ENSG0000(	gene_varia	ENSG0000C
NA	NA	NA	NA	NA	splice_acce	ENSG0000(	transcript	ENST0000C
NA	NA	NA	NA	NA	structural_	ENSG0000(	interaction	2ETK:A_26
NA	NA	NA	NA	NA	splice_don	ENSG0000(	transcript	ENST0000C
NA	NA	NA	NA	NA	splice_don	ENSG0000(	transcript	ENST0000C
NA	NA	NA	NA	NA	structural_	ENSG0000(	interaction	3GD8:A_41
NA	NA	NA	NA	NA	stop_gaine	ENSG0000(	transcript	ENST0000C
NA	Benign/Like	Cardiomyo	NA	NA	frameshift_	ENSG0000(	transcript	ENST0000C
NA	NA	NA	NA	NA	splice_don	ENSG0000(	transcript	ENST0000C
NA	NA	NA	NA	NA	structural_	ENSG0000(	interaction	4GWM:A_5
NA	NA	NA	NA	NA	structural_	ENSG0000(	interaction	4GWM:A_7
NA	NA	NA	NA	NA	structural_	ENSG0000(	interaction	4GWM:A_2
NA	NA	NA	NA	NA	splice_acce	ENSG0000(	transcript	ENST0000C
NA	NA	NA	NA	NA	splice_acce	ENSG0000(	transcript	ENST0000C
NA	NA	NA	NA	NA	structural_	ENSG0000(	interaction	3IHY:D_328
NA	Benign	Schinzel-Gi	0.51458	0.49381	frameshift_	ENSG0000(	transcript	ENST0000C
NA	NA	NA	NA	NA	splice_don	ENSG0000(	transcript	ENST0000C
NA	NA	NA	NA	NA	start_lost	ENSG0000(	transcript	ENST0000C
NA	Benign	Deafness,_	0.04572	0.02236	start_lost	ENSG0000(	transcript	ENST0000C
NA	NA	NA	NA	NA	start_lost	ENSG0000(	transcript	ENST0000C
NA	NA	NA	NA	NA	frameshift_	ENSG0000(	transcript	ENST0000C
NA	NA	NA	NA	NA	frameshift_	ENSG0000(	transcript	ENST0000C
NA	NA	NA	NA	NA	frameshift_	ENSG0000(	transcript	ENST0000C
NA	NA	NA	NA	NA	frameshift_	ENSG0000(	transcript	ENST0000C

NA	NA	NA	NA	NA	splice_don	ENSG0000(	transcript	ENST0000C
NA	NA	NA	NA	NA	splice_acce	ENSG0000(	transcript	ENST0000C
NA	Benign	not_provid	NA	NA	frameshift_	ENSG0000(	transcript	ENST0000C
NA	NA	NA	NA	NA	bidirection	ENSG0000(	gene_varia	ENSG00000
NA	NA	NA	NA	NA	bidirection	ENSG0000(	gene_varia	ENSG00000
NA	NA	NA	NA	NA	bidirection	ENSG0000(	gene_varia	ENSG00000
NA	NA	NA	NA	NA	bidirection	ENSG0000(	gene_varia	ENSG00000
NA	NA	NA	NA	NA	bidirection	ENSG0000(	gene_varia	ENSG00000
NA	NA	NA	NA	NA	bidirection	ENSG0000(	gene_varia	ENSG00000
NA	NA	NA	NA	NA	splice_don	ENSG0000(	transcript	ENST0000C
NA	NA	NA	NA	NA	stop_gaine	ENSG0000(	transcript	ENST0000C
NA	NA	NA	NA	NA	splice_don	ENSG0000(	transcript	ENST0000C
NA	NA	NA	NA	NA	splice_acce	ENSG0000(	transcript	ENST0000C
NA	NA	NA	NA	NA	splice_acce	ENSG0000(	transcript	ENST0000C
NA	NA	NA	NA	NA	splice_acce	ENSG0000(	transcript	ENST0000C
NA	NA	NA	NA	NA	splice_acce	ENSG0000(	transcript	ENST0000C
NA	NA	NA	NA	NA	splice_acce	ENSG0000(	transcript	ENST0000C
NA	NA	NA	NA	NA	splice_acce	ENSG0000(	transcript	ENST0000C
NA	NA	NA	NA	NA	splice_acce	ENSG0000(	transcript	ENST0000C
NA	NA	NA	NA	NA	splice_acce	ENSG0000(	transcript	ENST0000C
NA	NA	NA	NA	NA	splice_don	ENSG0000(	transcript	ENST0000C
NA	NA	NA	NA	NA	frameshift_	ENSG0000(	transcript	ENST0000C
NA	Benign/Lik	Combined_	0.22157	0.17272	structural_	ENSG0000(	interaction	4GKX:A_11
NA	NA	NA	NA	NA	splice_acce	ENSG0000(	transcript	ENST0000C
NA	NA	NA	NA	NA	splice_don	ENSG0000(	transcript	ENST0000C
NA	NA	NA	NA	NA	frameshift_	ENSG0000(	transcript	ENST0000C
NA	NA	NA	NA	NA	frameshift_	ENSG0000(	transcript	ENST0000C
NA	NA	NA	NA	NA	structural_	ENSG0000(	interaction	1WZ9:A_16
NA	NA	NA	NA	NA	stop_gaine	ENSG0000(	transcript	ENST0000C
NA	NA	NA	NA	NA	stop_gaine	ENSG0000(	transcript	ENST0000C
NA	NA	NA	NA	NA	structural_	ENSG0000(	interaction	1JRR:A_29
NA	NA	NA	NA	NA	structural_	ENSG0000(	interaction	1BY7:A_25
NA	NA	NA	NA	NA	splice_don	ENSG0000(	transcript	ENST0000C
NA	NA	NA	NA	NA	splice_don	ENSG0000(	transcript	ENST0000C
NA	NA	NA	NA	NA	splice_don	ENSG0000(	transcript	ENST0000C
NA	NA	NA	NA	NA	splice_don	ENSG0000(	transcript	ENST0000C
NA	NA	NA	NA	NA	frameshift_	ENSG0000(	transcript	ENST0000C
NA	NA	NA	NA	NA	stop_gaine	ENSG0000(	transcript	ENST0000C
NA	NA	NA	NA	NA	gene_fusio	ENSG0000(	gene_varia	ENSG00000
NA	NA	NA	NA	NA	splice_don	ENSG0000(	transcript	ENST0000C
NA	NA	NA	NA	NA	stop_lost	ENSG0000(	transcript	ENST0000C
NA	NA	NA	NA	NA	splice_acce	ENSG0000(	transcript	ENST0000C
NA	NA	NA	NA	NA	structural_	ENSG0000(	interaction	2WEK:A_19
NA	NA	NA	NA	NA	structural_	ENSG0000(	interaction	2WEK:A_75
NA	NA	NA	NA	NA	splice_don	ENSG0000(	transcript	ENST0000C
NA	NA	NA	NA	NA	splice_don	ENSG0000(	transcript	ENST0000C
NA	NA	NA	NA	NA	stop_lost	ENSG0000(	transcript	ENST0000C

NA	NA	NA	NA	NA	splice_acce	ENSG0000(	transcript	ENST0000C
NA	NA	NA	NA	NA	stop_gaine	ENSG0000(	transcript	ENST0000C
NA	NA	NA	NA	NA	frameshift_	ENSG0000(	transcript	ENST0000C
NA	NA	NA	NA	NA	splice_don	ENSG0000(	transcript	ENST0000C
NA	NA	NA	NA	NA	frameshift_	ENSG0000(	transcript	ENST0000C
NA	NA	NA	NA	NA	splice_don	ENSG0000(	transcript	ENST0000C
NA	NA	NA	NA	NA	frameshift_	ENSG0000(	transcript	ENST0000C
NA	NA	NA	NA	NA	frameshift_	ENSG0000(	transcript	ENST0000C
NA	NA	NA	NA	NA	frameshift_	ENSG0000(	transcript	ENST0000C
NA	NA	NA	NA	NA	frameshift_	ENSG0000(	transcript	ENST0000C
NA	NA	NA	NA	NA	stop_gaine	ENSG0000(	transcript	ENST0000C
NA	NA	NA	NA	NA	structural_	ENSG0000(	interaction	3U10:A_56
NA	NA	NA	NA	NA	structural_	ENSG0000(	interaction	4BOC:A_87
NA	NA	NA	NA	NA	splice_don	ENSG0000(	transcript	ENST0000C
NA	NA	NA	NA	NA	structural_	ENSG0000(	interaction	3ZZY:A_18
NA	NA	NA	NA	NA	frameshift_	ENSG0000(	transcript	ENST0000C
NA	NA	NA	NA	NA	frameshift_	ENSG0000(	transcript	ENST0000C
NA	NA	NA	NA	NA	frameshift_	ENSG0000(	transcript	ENST0000C
NA	NA	NA	NA	NA	frameshift_	ENSG0000(	transcript	ENST0000C
NA	NA	NA	NA	NA	splice_acce	ENSG0000(	transcript	ENST0000C
NA	NA	NA	NA	NA	frameshift_	ENSG0000(	transcript	ENST0000C
NA	NA	NA	NA	NA	frameshift_	ENSG0000(	transcript	ENST0000C
NA	NA	NA	NA	NA	start_lost	ENSG0000(	transcript	ENST0000C
NA	NA	NA	NA	NA	splice_don	ENSG0000(	transcript	ENST0000C
NA	Benign/Lik	Leigh_synd	0.07554	0.03654	structural_	ENSG0000(	interaction	3ORH:B_2C
NA	NA	NA	NA	NA	frameshift_	ENSG0000(	transcript	ENST0000C
NA	NA	NA	NA	NA	splice_don	ENSG0000(	transcript	ENST0000C
NA	NA	NA	NA	NA	splice_don	ENSG0000(	transcript	ENST0000C
NA	NA	NA	NA	NA	stop_lost	ENSG0000(	transcript	ENST0000C
NA	NA	NA	NA	NA	frameshift_	ENSG0000(	transcript	ENST0000C
NA	NA	NA	NA	NA	structural_	ENSG0000(	interaction	2C47:A_24
NA	NA	NA	NA	NA	splice_don	ENSG0000(	transcript	ENST0000C
NA	NA	NA	NA	NA	frameshift_	ENSG0000(	transcript	ENST0000C
NA	NA	NA	NA	NA	start_lost	ENSG0000(	transcript	ENST0000C
NA	NA	NA	NA	NA	splice_acce	ENSG0000(	transcript	ENST0000C
NA	NA	NA	NA	NA	splice_acce	ENSG0000(	transcript	ENST0000C
NA	NA	NA	NA	NA	splice_acce	ENSG0000(	transcript	ENST0000C
NA	NA	NA	NA	NA	splice_acce	ENSG0000(	transcript	ENST0000C
NA	NA	NA	NA	NA	splice_acce	ENSG0000(	transcript	ENST0000C
NA	NA	NA	NA	NA	splice_don	ENSG0000(	transcript	ENST0000C
NA	NA	NA	NA	NA	stop_gaine	ENSG0000(	transcript	ENST0000C
NA	NA	NA	NA	NA	splice_don	ENSG0000(	transcript	ENST0000C
NA	NA	NA	NA	NA	stop_lost	ENSG0000(	transcript	ENST0000C
NA	NA	NA	NA	NA	stop_lost	ENSG0000(	transcript	ENST0000C
NA	NA	NA	NA	NA	stop_gaine	ENSG0000(	transcript	ENST0000C
NA	NA	NA	NA	NA	frameshift_	ENSG0000(	transcript	ENST0000C
NA	NA	NA	NA	NA	frameshift_	ENSG0000(	transcript	ENST0000C

NA	NA	NA	NA	NA	splice_acce	ENSG0000(	transcript	ENST0000C
NA	NA	NA	NA	NA	frameshift_	ENSG0000(	transcript	ENST0000C
NA	NA	NA	NA	NA	frameshift_	ENSG0000(	transcript	ENST0000C
NA	NA	NA	NA	NA	stop_gaine	ENSG0000(	transcript	ENST0000C
NA	NA	NA	NA	NA	splice_don	ENSG0000(	transcript	ENST0000C
NA	NA	NA	NA	NA	structural_	ENSG0000(	interaction	3DB3:A_13
NA	NA	NA	NA	NA	structural_	ENSG0000(	interaction	2PB7:A_46
NA	NA	NA	NA	NA	structural_	ENSG0000(	interaction	2PB7:A_42
NA	NA	NA	NA	NA	structural_	ENSG0000(	interaction	2FH7:A_14
NA	NA	NA	NA	NA	structural_	ENSG0000(	interaction	2YD9:A_25
NA	NA	NA	NA	NA	structural_	ENSG0000(	interaction	2YD2:A_56
NA	NA	NA	NA	NA	structural_	ENSG0000(	interaction	2X36:A_87
NA	NA	NA	NA	NA	structural_	ENSG0000(	interaction	2X36:A_87
NA	NA	NA	NA	NA	stop_lost	ENSG0000(	transcript	ENST0000C
NA	NA	NA	NA	NA	splice_acce	ENSG0000(	transcript	ENST0000C
NA	NA	NA	NA	NA	splice_acce	ENSG0000(	transcript	ENST0000C
NA	NA	NA	NA	NA	splice_regi	ENSG0000(	transcript	ENST0000C
NA	NA	NA	NA	NA	splice_acce	ENSG0000(	transcript	ENST0000C
NA	Benign	not_specifi	0.00482	0.00180	structural_	ENSG0000(	interaction	3BJI:B_239
NA	NA	NA	NA	NA	splice_don	ENSG0000(	transcript	ENST0000C
NA	NA	NA	NA	NA	splice_acce	ENSG0000(	transcript	ENST0000C
NA	NA	NA	NA	NA	splice_acce	ENSG0000(	transcript	ENST0000C
NA	NA	NA	NA	NA	frameshift_	ENSG0000(	transcript	ENST0000C
NA	NA	NA	NA	NA	frameshift_	ENSG0000(	transcript	ENST0000C
NA	NA	NA	NA	NA	frameshift_	ENSG0000(	transcript	ENST0000C
NA	NA	NA	NA	NA	frameshift_	ENSG0000(	transcript	ENST0000C
NA	NA	NA	NA	NA	frameshift_	ENSG0000(	transcript	ENST0000C
NA	NA	NA	NA	NA	frameshift_	ENSG0000(	transcript	ENST0000C
NA	NA	NA	NA	NA	frameshift_	ENSG0000(	transcript	ENST0000C
NA	NA	NA	NA	NA	frameshift_	ENSG0000(	transcript	ENST0000C
NA	NA	NA	NA	NA	frameshift_	ENSG0000(	transcript	ENST0000C
NA	NA	NA	NA	NA	frameshift_	ENSG0000(	transcript	ENST0000C
NA	NA	NA	NA	NA	frameshift_	ENSG0000(	transcript	ENST0000C
NA	Benign/Like	Hemophag	0.01088	0.00459	structural_	ENSG0000(	interaction	4CCA:A_26
NA	Conflicting_	Familial_he	NA	NA	structural_	ENSG0000(	interaction	4CCA:A_22
NA	NA	NA	NA	NA	structural_	ENSG0000(	interaction	2H2R:A_17
NA	NA	NA	NA	NA	structural_	ENSG0000(	interaction	4GJ0:B_20
NA	NA	NA	NA	NA	structural_	ENSG0000(	interaction	2DYL:A_16
NA	NA	NA	NA	NA	stop_lost&	ENSG0000(	transcript	ENST0000C
NA	NA	NA	NA	NA	start_lost&	ENSG0000(	transcript	ENST0000C
NA	NA	NA	NA	NA	start_lost	ENSG0000(	transcript	ENST0000C
NA	NA	NA	NA	NA	frameshift_	ENSG0000(	transcript	ENST0000C
NA	NA	NA	NA	NA	splice_acce	ENSG0000(	transcript	ENST0000C
NA	NA	NA	NA	NA	splice_acce	ENSG0000(	transcript	ENST0000C
NA	NA	NA	NA	NA	frameshift_	ENSG0000(	transcript	ENST0000C
NA	NA	NA	NA	NA	frameshift_	ENSG0000(	transcript	ENST0000C
NA	NA	NA	NA	NA	frameshift_	ENSG0000(	transcript	ENST0000C

NA	NA	NA	NA	NA	frameshift_	ENSG0000(	transcript	ENST0000C
NA	NA	NA	NA	NA	frameshift_	ENSG0000(	transcript	ENST0000C
NA	NA	NA	NA	NA	stop_gaine	ENSG0000(	transcript	ENST0000C
NA	NA	NA	NA	NA	splice_don	ENSG0000(	transcript	ENST0000C
NA	NA	NA	NA	NA	structural_	ENSG0000(	interaction	3H2U:B_13
NA	NA	NA	NA	NA	stop_lost	ENSG0000(	transcript	ENST0000C
Chronic_in	Benign/Like	Tyrosine_k	0.00511	0.00100	structural_	ENSG0000(	interaction	3LXN:A_92
NA	Benign	Tyrosine_k	0.00800	0.00379	structural_	ENSG0000(	interaction	4PO6:A_53
NA	NA	NA	NA	NA	structural_	ENSG0000(	interaction	2QYK:A_37
NA	NA	NA	NA	NA	frameshift_	ENSG0000(	transcript	ENST0000C
NA	NA	NA	NA	NA	frameshift_	ENSG0000(	transcript	ENST0000C
NA	NA	NA	NA	NA	structural_	ENSG0000(	interaction	2GF9:A_25
NA	NA	NA	NA	NA	frameshift_	ENSG0000(	transcript	ENST0000C
NA	NA	NA	NA	NA	stop_gaine	ENSG0000(	transcript	ENST0000C
NA	NA	NA	NA	NA	splice_don	ENSG0000(	transcript	ENST0000C
NA	NA	NA	NA	NA	splice_don	ENSG0000(	transcript	ENST0000C
NA	NA	NA	NA	NA	frameshift_	ENSG0000(	transcript	ENST0000C
NA	NA	NA	NA	NA	splice_acce	ENSG0000(	transcript	ENST0000C
NA	not_provid	not_provid	NA	NA	frameshift_	ENSG0000(	transcript	ENST0000C
NA	NA	NA	NA	NA	splice_don	ENSG0000(	transcript	ENST0000C
NA	NA	NA	NA	NA	stop_lost	ENSG0000(	transcript	ENST0000C
NA	NA	NA	NA	NA	frameshift_	ENSG0000(	transcript	ENST0000C
NA	NA	NA	NA	NA	frameshift_	ENSG0000(	transcript	ENST0000C
NA	NA	NA	NA	NA	frameshift_	ENSG0000(	transcript	ENST0000C
NA	NA	NA	NA	NA	splice_acce	ENSG0000(	transcript	ENST0000C
NA	NA	NA	NA	NA	splice_don	ENSG0000(	transcript	ENST0000C
NA	NA	NA	NA	NA	splice_acce	ENSG0000(	transcript	ENST0000C
NA	NA	NA	NA	NA	frameshift_	ENSG0000(	transcript	ENST0000C
NA	Likely_beni	not_provid	NA	NA	structural_	ENSG0000(	interaction	4LDQ:A_70
NA	NA	NA	NA	NA	splice_don	ENSG0000(	transcript	ENST0000C
NA	NA	NA	NA	NA	frameshift_	ENSG0000(	transcript	ENST0000C
NA	NA	NA	NA	NA	frameshift_	ENSG0000(	transcript	ENST0000C
NA	NA	NA	NA	NA	frameshift_	ENSG0000(	transcript	ENST0000C
NA	NA	NA	NA	NA	frameshift_	ENSG0000(	transcript	ENST0000C
NA	NA	NA	NA	NA	frameshift_	ENSG0000(	transcript	ENST0000C
NA	NA	NA	NA	NA	conservativ	ENSG0000(	transcript	ENST0000C
NA	NA	NA	NA	NA	splice_don	ENSG0000(	transcript	ENST0000C
NA	NA	NA	NA	NA	frameshift_	ENSG0000(	transcript	ENST0000C
NA	NA	NA	NA	NA	frameshift_	ENSG0000(	transcript	ENST0000C
NA	NA	NA	NA	NA	frameshift_	ENSG0000(	transcript	ENST0000C
NA	NA	NA	NA	NA	frameshift_	ENSG0000(	transcript	ENST0000C
NA	NA	NA	NA	NA	frameshift_	ENSG0000(	transcript	ENST0000C
NA	NA	NA	NA	NA	structural_	ENSG0000(	interaction	4OTD:A_61
NA	NA	NA	NA	NA	structural_	ENSG0000(	interaction	4OTD:A_73
NA	NA	NA	NA	NA	splice_acce	ENSG0000(	transcript	ENST0000C
NA	NA	NA	NA	NA	splice_acce	ENSG0000(	transcript	ENST0000C
NA	NA	NA	NA	NA	splice_acce	ENSG0000(	transcript	ENST0000C

NA	NA	NA	NA	NA	splice_don	ENSG0000(	transcript	ENST0000C
NA	NA	NA	NA	NA	stop_gaine	ENSG0000(	transcript	ENST0000C
NA	NA	NA	NA	NA	splice_don	ENSG0000(	transcript	ENST0000C
NA	NA	NA	NA	NA	start_lost	ENSG0000(	transcript	ENST0000C
NA	NA	NA	NA	NA	splice_don	ENSG0000(	transcript	ENST0000C
NA	NA	NA	NA	NA	structural_	ENSG0000(	interaction	3QBT:A_9-/
NA	NA	NA	NA	NA	structural_	ENSG0000(	interaction	3QBT:A_86
NA	NA	NA	NA	NA	splice_acce	ENSG0000(	transcript	ENST0000C
NA	NA	NA	NA	NA	frameshift_	ENSG0000(	transcript	ENST0000C
NA	NA	NA	NA	NA	splice_acce	ENSG0000(	transcript	ENST0000C
NA	NA	NA	NA	NA	frameshift_	ENSG0000(	transcript	ENST0000C
NA	NA	NA	NA	NA	frameshift_	ENSG0000(	transcript	ENST0000C
NA	NA	NA	NA	NA	frameshift_	ENSG0000(	transcript	ENST0000C
NA	NA	NA	NA	NA	frameshift_	ENSG0000(	transcript	ENST0000C
NA	NA	NA	NA	NA	frameshift_	ENSG0000(	transcript	ENST0000C
NA	NA	NA	NA	NA	frameshift_	ENSG0000(	transcript	ENST0000C
NA	NA	NA	NA	NA	frameshift_	ENSG0000(	transcript	ENST0000C
NA	NA	NA	NA	NA	frameshift_	ENSG0000(	transcript	ENST0000C
NA	NA	NA	NA	NA	frameshift_	ENSG0000(	transcript	ENST0000C
NA	NA	NA	NA	NA	frameshift_	ENSG0000(	transcript	ENST0000C
NA	NA	NA	NA	NA	frameshift_	ENSG0000(	transcript	ENST0000C
NA	NA	NA	NA	NA	frameshift_	ENSG0000(	transcript	ENST0000C
NA	Benign	not_specifi	0.03325	NA	frameshift_	ENSG0000(	transcript	ENST0000C
NA	Benign	not_specifi	0.03325	NA	frameshift_	ENSG0000(	transcript	ENST0000C
NA	Benign	not_specifi	0.03325	NA	frameshift_	ENSG0000(	transcript	ENST0000C
NA	Benign	not_specifi	NA	NA	frameshift_	ENSG0000(	transcript	ENST0000C
NA	Benign	not_specifi	NA	NA	frameshift_	ENSG0000(	transcript	ENST0000C
NA	Benign	not_specifi	NA	NA	frameshift_	ENSG0000(	transcript	ENST0000C
NA	NA	NA	NA	NA	frameshift_	ENSG0000(	transcript	ENST0000C
NA	NA	NA	NA	NA	frameshift_	ENSG0000(	transcript	ENST0000C
NA	NA	NA	NA	NA	frameshift_	ENSG0000(	transcript	ENST0000C
NA	NA	NA	NA	NA	frameshift_	ENSG0000(	transcript	ENST0000C
NA	NA	NA	NA	NA	frameshift_	ENSG0000(	transcript	ENST0000C
NA	NA	NA	NA	NA	frameshift_	ENSG0000(	transcript	ENST0000C
NA	NA	NA	NA	NA	splice_don	ENSG0000(	transcript	ENST0000C
NA	NA	NA	NA	NA	frameshift_	ENSG0000(	transcript	ENST0000C
NA	Benign	not_specifi	0.22607	0.18191	splice_acce	ENSG0000(	transcript	ENST0000C
NA	NA	NA	NA	NA	structural_	ENSG0000(	interaction	3KHF:A_95
NA	NA	NA	NA	NA	structural_	ENSG0000(	interaction	2QYM:A_2
NA	NA	NA	NA	NA	structural_	ENSG0000(	interaction	2QYM:A_2
NA	NA	NA	NA	NA	frameshift_	ENSG0000(	transcript	ENST0000C
NA	NA	NA	NA	NA	start_lost	ENSG0000(	transcript	ENST0000C
NA	NA	NA	NA	NA	frameshift_	ENSG0000(	transcript	ENST0000C
NA	NA	NA	NA	NA	frameshift_	ENSG0000(	transcript	ENST0000C
NA	NA	NA	NA	NA	splice_don	ENSG0000(	transcript	ENST0000C
NA	NA	NA	NA	NA	stop_gaine	ENSG0000(	transcript	ENST0000C
NA	NA	NA	NA	NA	frameshift_	ENSG0000(	transcript	ENST0000C

NA	NA	NA	NA	NA	splice_acce	ENSG0000(	transcript	ENST0000C
NA	NA	NA	NA	NA	splice_acce	ENSG0000(	transcript	ENST0000C
NA	NA	NA	NA	NA	splice_acce	ENSG0000(	transcript	ENST0000C
NA	NA	NA	NA	NA	stop_lost	ENSG0000(	transcript	ENST0000C
NA	NA	NA	NA	NA	frameshift_	ENSG0000(	transcript	ENST0000C
NA	NA	NA	NA	NA	frameshift_	ENSG0000(	transcript	ENST0000C
NA	NA	NA	NA	NA	frameshift_	ENSG0000(	transcript	ENST0000C
NA	NA	NA	NA	NA	frameshift_	ENSG0000(	transcript	ENST0000C
NA	NA	NA	NA	NA	frameshift_	ENSG0000(	transcript	ENST0000C
NA	NA	NA	NA	NA	frameshift_	ENSG0000(	transcript	ENST0000C
NA	NA	NA	NA	NA	frameshift_	ENSG0000(	transcript	ENST0000C
NA	NA	NA	NA	NA	frameshift_	ENSG0000(	transcript	ENST0000C
NA	NA	NA	NA	NA	frameshift_	ENSG0000(	transcript	ENST0000C
NA	NA	NA	NA	NA	stop_gaine	ENSG0000(	transcript	ENST0000C
NA	NA	NA	NA	NA	frameshift_	ENSG0000(	transcript	ENST0000C
NA	NA	NA	NA	NA	frameshift_	ENSG0000(	transcript	ENST0000C
NA	NA	NA	NA	NA	frameshift_	ENSG0000(	transcript	ENST0000C
NA	NA	NA	NA	NA	stop_gaine	ENSG0000(	transcript	ENST0000C
NA	NA	NA	NA	NA	stop_gaine	ENSG0000(	transcript	ENST0000C
NA	NA	NA	NA	NA	stop_gaine	ENSG0000(	transcript	ENST0000C
NA	NA	NA	NA	NA	splice_acce	ENSG0000(	transcript	ENST0000C
NA	NA	NA	NA	NA	splice_don	ENSG0000(	transcript	ENST0000C
NA	NA	NA	NA	NA	splice_acce	ENSG0000(	transcript	ENST0000C
NA	NA	NA	NA	NA	stop_lost&	ENSG0000(	transcript	ENST0000C
NA	NA	NA	NA	NA	frameshift_	ENSG0000(	transcript	ENST0000C
NA	NA	NA	NA	NA	frameshift_	ENSG0000(	transcript	ENST0000C
NA	NA	NA	NA	NA	frameshift_	ENSG0000(	transcript	ENST0000C
NA	Benign	not_provid	NA	NA	frameshift_	ENSG0000(	transcript	ENST0000C
NA	NA	NA	NA	NA	frameshift_	ENSG0000(	transcript	ENST0000C
NA	NA	NA	NA	NA	splice_don	ENSG0000(	transcript	ENST0000C
NA	NA	NA	NA	NA	splice_don	ENSG0000(	transcript	ENST0000C
NA	NA	NA	NA	NA	splice_acce	ENSG0000(	transcript	ENST0000C
NA	NA	NA	NA	NA	splice_acce	ENSG0000(	transcript	ENST0000C
NA	NA	NA	NA	NA	splice_don	ENSG0000(	transcript	ENST0000C
NA	NA	NA	NA	NA	structural_	ENSG0000(	interaction	4B93:B_83
NA	NA	NA	NA	NA	structural_	ENSG0000(	interaction	4B93:B_77
NA	NA	NA	NA	NA	splice_acce	ENSG0000(	transcript	ENST0000C
NA	NA	NA	NA	NA	stop_lost	ENSG0000(	transcript	ENST0000C
NA	NA	NA	NA	NA	protein_pr	ENSG0000(	interaction	4BXO:A_2C
NA	NA	NA	NA	NA	splice_acce	ENSG0000(	transcript	ENST0000C
NA	NA	NA	NA	NA	splice_don	ENSG0000(	transcript	ENST0000C
NA	NA	NA	NA	NA	splice_don	ENSG0000(	transcript	ENST0000C
NA	NA	NA	NA	NA	splice_don	ENSG0000(	transcript	ENST0000C
NA	NA	NA	NA	NA	splice_don	ENSG0000(	transcript	ENST0000C
NA	NA	NA	NA	NA	splice_don	ENSG0000(	transcript	ENST0000C
NA	NA	NA	NA	NA	frameshift_	ENSG0000(	transcript	ENST0000C
NA	NA	NA	NA	NA	frameshift_	ENSG0000(	transcript	ENST0000C

NA	NA	NA	NA	NA	frameshift_	ENSG0000(	transcript	ENST0000C
NA	NA	NA	NA	NA	frameshift_	ENSG0000(	transcript	ENST0000C
NA	NA	NA	NA	NA	frameshift_	ENSG0000(	transcript	ENST0000C
NA	NA	NA	NA	NA	stop_gaine	ENSG0000(	transcript	ENST0000C
NA	NA	NA	NA	NA	frameshift_	ENSG0000(	transcript	ENST0000C
NA	NA	NA	NA	NA	structural_	ENSG0000(	interaction	3T2N:A_92
NA	NA	NA	NA	NA	bidirection	ENSG0000(	gene_varia	ENSG0000C
NA	NA	NA	NA	NA	bidirection	ENSG0000(	gene_varia	ENSG0000C
NA	NA	NA	NA	NA	stop_gaine	ENSG0000(	transcript	ENST0000C
NA	NA	NA	NA	NA	stop_gaine	ENSG0000(	transcript	ENST0000C
NA	NA	NA	NA	NA	frameshift_	ENSG0000(	transcript	ENST0000C
NA	NA	NA	NA	NA	stop_gaine	ENSG0000(	transcript	ENST0000C
NA	NA	NA	NA	NA	splice_acce	ENSG0000(	transcript	ENST0000C
NA	NA	NA	NA	NA	frameshift_	ENSG0000(	transcript	ENST0000C
NA	NA	NA	NA	NA	frameshift_	ENSG0000(	transcript	ENST0000C
NA	NA	NA	NA	NA	frameshift_	ENSG0000(	transcript	ENST0000C
NA	Conflicting_	not_provid	NA	NA	splice_acce	ENSG0000(	transcript	ENST0000C
NA	NA	NA	NA	NA	splice_don	ENSG0000(	transcript	ENST0000C
NA	NA	NA	NA	NA	splice_don	ENSG0000(	transcript	ENST0000C
NA	NA	NA	NA	NA	splice_don	ENSG0000(	transcript	ENST0000C
NA	NA	NA	NA	NA	splice_acce	ENSG0000(	transcript	ENST0000C
NA	NA	NA	NA	NA	structural_	ENSG0000(	interaction	4PHJ:B_16
NA	NA	NA	NA	NA	splice_don	ENSG0000(	transcript	ENST0000C
NA	NA	NA	NA	NA	splice_acce	ENSG0000(	transcript	ENST0000C
NA	NA	NA	NA	NA	splice_acce	ENSG0000(	transcript	ENST0000C
NA	NA	NA	NA	NA	splice_acce	ENSG0000(	transcript	ENST0000C
NA	NA	NA	NA	NA	splice_acce	ENSG0000(	transcript	ENST0000C
NA	NA	NA	NA	NA	stop_lost	ENSG0000(	transcript	ENST0000C
NA	NA	NA	NA	NA	splice_acce	ENSG0000(	transcript	ENST0000C
NA	NA	NA	NA	NA	splice_acce	ENSG0000(	transcript	ENST0000C
NA	NA	NA	NA	NA	splice_acce	ENSG0000(	transcript	ENST0000C
NA	NA	NA	NA	NA	splice_acce	ENSG0000(	transcript	ENST0000C
NA	Benign	not_provid	NA	NA	frameshift_	ENSG0000(	transcript	ENST0000C
NA	Benign	not_provid	NA	NA	frameshift_	ENSG0000(	transcript	ENST0000C
NA	NA	NA	NA	NA	splice_acce	ENSG0000(	transcript	ENST0000C
NA	NA	NA	NA	NA	splice_acce	ENSG0000(	transcript	ENST0000C
NA	NA	NA	NA	NA	splice_acce	ENSG0000(	transcript	ENST0000C
NA	NA	NA	NA	NA	splice_acce	ENSG0000(	transcript	ENST0000C
NA	NA	NA	NA	NA	splice_acce	ENSG0000(	transcript	ENST0000C
NA	NA	NA	NA	NA	splice_acce	ENSG0000(	transcript	ENST0000C
NA	NA	NA	NA	NA	stop_gaine	ENSG0000(	transcript	ENST0000C
NA	Benign	not_provid	NA	NA	stop_gaine	ENSG0000(	transcript	ENST0000C
NA	Benign	not_provid	NA	NA	stop_gaine	ENSG0000(	transcript	ENST0000C
NA	Benign	Focal_segn	0.41049	0.33926	structural_	ENSG0000(	interaction	2R0O:B_18
NA	NA	NA	NA	NA	stop_lost	ENSG0000(	transcript	ENST0000C
NA	Benign	not_provid	NA	NA	structural_	ENSG0000(	interaction	1J8F:A_15
NA	NA	NA	NA	NA	splice_don	ENSG0000(	transcript	ENST0000C

NA	NA	NA	NA	NA	frameshift_	ENSG0000	transcript	ENST00000
NA	NA	NA	NA	NA	structural_	ENSG0000	interaction	3H7H:B_19
NA	NA	NA	NA	NA	structural_	ENSG0000	interaction	1G86:A_80
NA	NA	NA	NA	NA	frameshift_	ENSG0000	transcript	ENST00000
NA	NA	NA	NA	NA	frameshift_	ENSG0000	transcript	ENST00000
NA	NA	NA	NA	NA	frameshift_	ENSG0000	transcript	ENST00000
NA	NA	NA	NA	NA	frameshift_	ENSG0000	transcript	ENST00000
NA	NA	NA	NA	NA	frameshift_	ENSG0000	transcript	ENST00000
NA	NA	NA	NA	NA	frameshift_	ENSG0000	transcript	ENST00000
NA	NA	NA	NA	NA	frameshift_	ENSG0000	transcript	ENST00000
NA	NA	NA	NA	NA	frameshift_	ENSG0000	transcript	ENST00000
NA	NA	NA	NA	NA	frameshift_	ENSG0000	transcript	ENST00000
NA	NA	NA	NA	NA	splice_acce	ENSG0000	transcript	ENST00000
NA	NA	NA	NA	NA	structural_	ENSG0000	interaction	1HDO:A_1
NA	NA	NA	NA	NA	structural_	ENSG0000	interaction	1HDO:A_3
NA	NA	NA	NA	NA	structural_	ENSG0000	interaction	4CRH:A_21
Blood_prot	NA	NA	NA	NA	structural_	ENSG0000	interaction	1M5K:F_16
NA	NA	NA	NA	NA	stop_gaine	ENSG0000	transcript	ENST00000
NA	drug_respo	Efavirenz_r	NA	0.31570	structural_	ENSG0000	interaction	3IBD:A_17
NA	NA	NA	NA	NA	structural_	ENSG0000	interaction	3IBD:A_16
NA	NA	NA	NA	NA	structural_	ENSG0000	interaction	2P85:B_11
NA	NA	NA	NA	NA	structural_	ENSG0000	interaction	2P85:A_94
NA	NA	NA	NA	NA	structural_	ENSG0000	interaction	2P85:A_89
NA	Benign	not_specifi	0.21478	0.27197	frameshift_	ENSG0000	transcript	ENST00000
NA	NA	NA	NA	NA	frameshift_	ENSG0000	transcript	ENST00000
NA	NA	NA	NA	NA	frameshift_	ENSG0000	transcript	ENST00000
NA	Benign/Lik	not_specifi	NA	NA	frameshift_	ENSG0000	transcript	ENST00000
NA	NA	NA	NA	NA	frameshift_	ENSG0000	transcript	ENST00000
NA	NA	NA	NA	NA	frameshift_	ENSG0000	transcript	ENST00000
NA	NA	NA	NA	NA	frameshift_	ENSG0000	transcript	ENST00000
NA	NA	NA	NA	NA	splice_acce	ENSG0000	transcript	ENST00000
NA	NA	NA	NA	NA	splice_don	ENSG0000	transcript	ENST00000
NA	NA	NA	NA	NA	frameshift_	ENSG0000	transcript	ENST00000
NA	NA	NA	NA	NA	stop_gaine	ENSG0000	transcript	ENST00000
NA	NA	NA	NA	NA	stop_gaine	ENSG0000	transcript	ENST00000
NA	NA	NA	NA	NA	splice_acce	ENSG0000	transcript	ENST00000
NA	NA	NA	NA	NA	frameshift_	ENSG0000	transcript	ENST00000
NA	NA	NA	NA	NA	stop_gaine	ENSG0000	transcript	ENST00000
NA	NA	NA	NA	NA	splice_acce	ENSG0000	transcript	ENST00000
NA	NA	NA	NA	NA	frameshift_	ENSG0000	transcript	ENST00000
NA	NA	NA	NA	NA	frameshift_	ENSG0000	transcript	ENST00000
NA	NA	NA	NA	NA	start_lost&	ENSG0000	transcript	ENST00000
NA	Benign	not_specifi	0.19826	0.31829	frameshift_	ENSG0000	transcript	ENST00000
NA	NA	NA	NA	NA	structural_	ENSG0000	interaction	3OP0:A_15
NA	NA	NA	NA	NA	splice_acce	ENSG0000	transcript	ENST00000
NA	drug_respo	Xeroderma	0.50847	0.66893	structural_	ENSG0000	interaction	2A1I:A_11
NA	NA	NA	NA	NA	stop_gaine	ENSG0000	transcript	ENST00000

NA	NA	NA	NA	NA	splice_don	ENSG0000(	transcript	ENST0000C
NA	Benign	not_specifi	0.00293	0.00080	splice_acce	ENSG0000(	transcript	ENST0000C
NA	NA	NA	NA	NA	splice_acce	ENSG0000(	transcript	ENST0000C
NA	NA	NA	NA	NA	splice_acce	ENSG0000(	transcript	ENST0000C
NA	NA	NA	NA	NA	splice_acce	ENSG0000(	transcript	ENST0000C
NA	NA	NA	NA	NA	splice_acce	ENSG0000(	transcript	ENST0000C
NA	NA	NA	NA	NA	splice_acce	ENSG0000(	transcript	ENST0000C
NA	NA	NA	NA	NA	splice_acce	ENSG0000(	transcript	ENST0000C
NA	NA	NA	NA	NA	splice_acce	ENSG0000(	transcript	ENST0000C
NA	NA	NA	NA	NA	splice_acce	ENSG0000(	transcript	ENST0000C
NA	NA	NA	NA	NA	splice_don	ENSG0000(	transcript	ENST0000C
NA	NA	NA	NA	NA	splice_don	ENSG0000(	transcript	ENST0000C
NA	NA	NA	NA	NA	splice_acce	ENSG0000(	transcript	ENST0000C
NA	NA	NA	NA	NA	stop_gaine	ENSG0000(	transcript	ENST0000C
NA	NA	NA	NA	NA	splice_acce	ENSG0000(	transcript	ENST0000C
NA	NA	NA	NA	NA	structural_	ENSG0000(	interaction	1WAO:3_4
NA	Benign	not_specifi	NA	0.08227	protein_pr	ENSG0000(	interaction	4LG6:A_28
NA	NA	NA	NA	NA	start_lost	ENSG0000(	transcript	ENST0000C
NA	NA	NA	NA	NA	splice_don	ENSG0000(	transcript	ENST0000C
NA	NA	NA	NA	NA	splice_don	ENSG0000(	transcript	ENST0000C
NA	NA	NA	NA	NA	splice_don	ENSG0000(	transcript	ENST0000C
NA	NA	NA	NA	NA	stop_gaine	ENSG0000(	transcript	ENST0000C
NA	NA	NA	NA	NA	frameshift_	ENSG0000(	transcript	ENST0000C
NA	NA	NA	NA	NA	frameshift_	ENSG0000(	transcript	ENST0000C
NA	NA	NA	NA	NA	structural_	ENSG0000(	interaction	1X9N:A_75
NA	NA	NA	NA	NA	structural_	ENSG0000(	interaction	1X9N:A_33
NA	NA	NA	NA	NA	frameshift_	ENSG0000(	transcript	ENST0000C
NA	NA	NA	NA	NA	start_lost	ENSG0000(	transcript	ENST0000C
NA	NA	NA	NA	NA	frameshift_	ENSG0000(	transcript	ENST0000C
NA	NA	NA	NA	NA	stop_lost	ENSG0000(	transcript	ENST0000C
Blood_met	Benign,_as	SECRETOR/	0.38942	0.32169	stop_gaine	ENSG0000(	transcript	ENST0000C
NA	NA	NA	NA	NA	structural_	ENSG0000(	interaction	1YDE:A_13
NA	NA	NA	NA	NA	splice_acce	ENSG0000(	transcript	ENST0000C
NA	NA	NA	NA	NA	structural_	ENSG0000(	interaction	1YDE:A_4-/
NA	NA	NA	NA	NA	frameshift_	ENSG0000(	transcript	ENST0000C
NA	NA	NA	NA	NA	frameshift_	ENSG0000(	transcript	ENST0000C
NA	NA	NA	NA	NA	frameshift_	ENSG0000(	transcript	ENST0000C
NA	NA	NA	NA	NA	stop_gaine	ENSG0000(	transcript	ENST0000C
NA	NA	NA	NA	NA	frameshift_	ENSG0000(	transcript	ENST0000C
NA	NA	NA	NA	NA	stop_gaine	ENSG0000(	transcript	ENST0000C
NA	NA	NA	NA	NA	frameshift_	ENSG0000(	transcript	ENST0000C
NA	NA	NA	NA	NA	splice_don	ENSG0000(	transcript	ENST0000C
NA	NA	NA	NA	NA	splice_don	ENSG0000(	transcript	ENST0000C
NA	NA	NA	NA	NA	frameshift_	ENSG0000(	transcript	ENST0000C
NA	NA	NA	NA	NA	frameshift_	ENSG0000(	transcript	ENST0000C
NA	NA	NA	NA	NA	frameshift_	ENSG0000(	transcript	ENST0000C
NA	NA	NA	NA	NA	frameshift_	ENSG0000(	transcript	ENST0000C

NA	NA	NA	NA	NA	frameshift_	ENSG0000(	transcript	ENST0000C
NA	NA	NA	NA	NA	frameshift_	ENSG0000(	transcript	ENST0000C
NA	NA	NA	NA	NA	frameshift_	ENSG0000(	transcript	ENST0000C
NA	NA	NA	NA	NA	frameshift_	ENSG0000(	transcript	ENST0000C
NA	NA	NA	NA	NA	splice_don	ENSG0000(	transcript	ENST0000C
NA	NA	NA	NA	NA	splice_don	ENSG0000(	transcript	ENST0000C
NA	NA	NA	NA	NA	frameshift_	ENSG0000(	transcript	ENST0000C
NA	NA	NA	NA	NA	frameshift_	ENSG0000(	transcript	ENST0000C
NA	NA	NA	NA	NA	splice_acce	ENSG0000(	transcript	ENST0000C
NA	NA	NA	NA	NA	splice_don	ENSG0000(	transcript	ENST0000C
NA	NA	NA	NA	NA	frameshift_	ENSG0000(	transcript	ENST0000C
NA	Conflicting_	Early_infan	0.00065	NA	splice_don	ENSG0000(	transcript	ENST0000C
NA	NA	NA	NA	NA	splice_acce	ENSG0000(	transcript	ENST0000C
NA	NA	NA	NA	NA	splice_don	ENSG0000(	transcript	ENST0000C
NA	NA	NA	NA	NA	splice_acce	ENSG0000(	transcript	ENST0000C
NA	NA	NA	NA	NA	splice_acce	ENSG0000(	transcript	ENST0000C
NA	NA	NA	NA	NA	frameshift_	ENSG0000(	transcript	ENST0000C
NA	NA	NA	NA	NA	stop_gaine	ENSG0000(	transcript	ENST0000C
NA	NA	NA	NA	NA	splice_don	ENSG0000(	transcript	ENST0000C
NA	NA	NA	NA	NA	splice_acce	ENSG0000(	transcript	ENST0000C
Blood_prot	NA	NA	NA	NA	splice_don	ENSG0000(	transcript	ENST0000C
NA	Likely_beni	not_provid	NA	NA	splice_don	ENSG0000(	transcript	ENST0000C
Blood_prot	NA	NA	NA	NA	frameshift_	ENSG0000(	transcript	ENST0000C
NA	NA	NA	NA	NA	frameshift_	ENSG0000(	transcript	ENST0000C
NA	Benign	Glutaric_ac	NA	NA	frameshift_	ENSG0000(	transcript	ENST0000C
NA	NA	NA	NA	NA	splice_acce	ENSG0000(	transcript	ENST0000C
NA	Benign	not_specifi	NA	NA	frameshift_	ENSG0000(	transcript	ENST0000C
NA	NA	NA	NA	NA	stop_gaine	ENSG0000(	transcript	ENST0000C
NA	NA	NA	NA	NA	frameshift_	ENSG0000(	transcript	ENST0000C
NA	NA	NA	NA	NA	start_lost	ENSG0000(	transcript	ENST0000C
NA	NA	NA	NA	NA	splice_acce	ENSG0000(	transcript	ENST0000C
NA	NA	NA	NA	NA	structural_	ENSG0000(	interaction	3DW8:A_1:
NA	NA	NA	NA	NA	protein_pr	ENSG0000(	interaction	2IE3:A_574
NA	NA	NA	NA	NA	splice_don	ENSG0000(	transcript	ENST0000C
NA	Benign	not_specifi	NA	NA	frameshift_	ENSG0000(	transcript	ENST0000C
NA	NA	NA	NA	NA	frameshift_	ENSG0000(	transcript	ENST0000C
NA	NA	NA	NA	NA	stop_gaine	ENSG0000(	transcript	ENST0000C
NA	NA	NA	NA	NA	frameshift_	ENSG0000(	transcript	ENST0000C
NA	NA	NA	NA	NA	frameshift_	ENSG0000(	transcript	ENST0000C
NA	NA	NA	NA	NA	frameshift_	ENSG0000(	transcript	ENST0000C
NA	NA	NA	NA	NA	splice_acce	ENSG0000(	transcript	ENST0000C
NA	NA	NA	NA	NA	splice_acce	ENSG0000(	transcript	ENST0000C
NA	NA	NA	NA	NA	splice_don	ENSG0000(	transcript	ENST0000C
NA	NA	NA	NA	NA	frameshift_	ENSG0000(	transcript	ENST0000C
NA	NA	NA	NA	NA	splice_don	ENSG0000(	transcript	ENST0000C
NA	NA	NA	NA	NA	stop_gaine	ENSG0000(	transcript	ENST0000C
NA	NA	NA	NA	NA	splice_acce	ENSG0000(	transcript	ENST0000C

NA	NA	NA	NA	NA	frameshift_	ENSG0000(	transcript	ENST0000C
NA	NA	NA	NA	NA	frameshift_	ENSG0000(	transcript	ENST0000C
NA	NA	NA	NA	NA	frameshift_	ENSG0000(	transcript	ENST0000C
NA	Benign/Like	Cardiomyo	0.04615	0.02276	protein_pr	ENSG0000(	interaction	1J1D:B_252
NA	Benign/Like	Hypertroph	0.08639	0.11721	start_lost	ENSG0000(	transcript	ENST0000C
NA	NA	NA	NA	NA	frameshift_	ENSG0000(	transcript	ENST0000C
NA	NA	NA	NA	NA	protein_pr	ENSG0000(	interaction	4FXW:A_4C
NA	NA	NA	NA	NA	splice_don	ENSG0000(	transcript	ENST0000C
NA	NA	NA	NA	NA	frameshift_	ENSG0000(	transcript	ENST0000C
NA	NA	NA	NA	NA	stop_lost&	ENSG0000(	transcript	ENST0000C
NA	NA	NA	NA	NA	splice_acce	ENSG0000(	transcript	ENST0000C
NA	NA	NA	NA	NA	splice_don	ENSG0000(	transcript	ENST0000C
NA	NA	NA	NA	NA	stop_gaine	ENSG0000(	transcript	ENST0000C
NA	NA	NA	NA	NA	stop_gaine	ENSG0000(	transcript	ENST0000C
NA	Benign	Spermatog	0.76247	0.76777	splice_don	ENSG0000(	transcript	ENST0000C
NA	NA	NA	NA	NA	splice_acce	ENSG0000(	transcript	ENST0000C
NA	NA	NA	NA	NA	splice_acce	ENSG0000(	transcript	ENST0000C
NA	NA	NA	NA	NA	frameshift_	ENSG0000(	transcript	ENST0000C
NA	NA	NA	NA	NA	stop_gaine	ENSG0000(	transcript	ENST0000C
NA	NA	NA	NA	NA	stop_gaine	ENSG0000(	transcript	ENST0000C
NA	NA	NA	NA	NA	splice_don	ENSG0000(	transcript	ENST0000C
NA	NA	NA	NA	NA	stop_gaine	ENSG0000(	transcript	ENST0000C
NA	NA	NA	NA	NA	start_lost	ENSG0000(	transcript	ENST0000C
NA	NA	NA	NA	NA	frameshift_	ENSG0000(	transcript	ENST0000C
NA	NA	NA	NA	NA	stop_gaine	ENSG0000(	transcript	ENST0000C
NA	NA	NA	NA	NA	splice_don	ENSG0000(	transcript	ENST0000C
NA	NA	NA	NA	NA	frameshift_	ENSG0000(	transcript	ENST0000C
NA	NA	NA	NA	NA	frameshift_	ENSG0000(	transcript	ENST0000C
NA	NA	NA	NA	NA	frameshift_	ENSG0000(	transcript	ENST0000C
NA	NA	NA	NA	NA	gene_fusio	ENSG0000(	gene_varia	ENSG0000C
NA	NA	NA	NA	NA	gene_fusio	ENSG0000(	gene_varia	ENSG0000C
NA	NA	NA	NA	NA	gene_fusio	ENSG0000(	gene_varia	ENSG0000C
NA	NA	NA	NA	NA	stop_gaine	ENSG0000(	transcript	ENST0000C
NA	NA	NA	NA	NA	splice_acce	ENSG0000(	transcript	ENST0000C
NA	NA	NA	NA	NA	splice_acce	ENSG0000(	transcript	ENST0000C
NA	Benign	not_specifi	NA	NA	frameshift_	ENSG0000(	transcript	ENST0000C
NA	Benign	not_specifi	NA	NA	frameshift_	ENSG0000(	transcript	ENST0000C
NA	NA	NA	NA	NA	frameshift_	ENSG0000(	transcript	ENST0000C
NA	NA	NA	NA	NA	splice_don	ENSG0000(	transcript	ENST0000C
NA	NA	NA	NA	NA	splice_don	ENSG0000(	transcript	ENST0000C
NA	NA	NA	NA	NA	splice_don	ENSG0000(	transcript	ENST0000C
NA	NA	NA	NA	NA	splice_don	ENSG0000(	transcript	ENST0000C
NA	NA	NA	NA	NA	splice_don	ENSG0000(	transcript	ENST0000C
NA	NA	NA	NA	NA	splice_don	ENSG0000(	transcript	ENST0000C
NA	NA	NA	NA	NA	splice_don	ENSG0000(	transcript	ENST0000C
NA	NA	NA	NA	NA	splice_don	ENSG0000(	transcript	ENST0000C
NA	NA	NA	NA	NA	protein_pr	ENSG0000(	interaction	4BX9:A_39

NA	NA	NA	NA	NA	splice_don	ENSG0000(	transcript	ENST0000C
Ribavirin-ir	drug_respc	Inosine_tri	0.07710	0.08946	structural_	ENSG0000(	interaction	2CAR:A_7-/
NA	NA	NA	NA	NA	splice_don	ENSG0000(	transcript	ENST0000C
NA	Benign	not_specifi	NA	NA	frameshift_	ENSG0000(	transcript	ENST0000C
NA	NA	NA	NA	NA	stop_gaine	ENSG0000(	transcript	ENST0000C
NA	NA	NA	NA	NA	structural_	ENSG0000(	interaction	1CWR:A_4!
Prion_dise	Benign	Alzheimer_	0.30778	0.26657	structural_	ENSG0000(	interaction	1I4M:A_12
NA	NA	NA	NA	NA	frameshift_	ENSG0000(	transcript	ENST0000C
NA	NA	NA	NA	NA	frameshift_	ENSG0000(	transcript	ENST0000C
NA	NA	NA	NA	NA	frameshift_	ENSG0000(	transcript	ENST0000C
NA	NA	NA	NA	NA	stop_gaine	ENSG0000(	transcript	ENST0000C
NA	Benign/Like	Arteriohep	0.03370	0.03355	structural_	ENSG0000(	interaction	2VJ2:A_30
NA	Benign	Arteriohep	0.10877	0.18191	structural_	ENSG0000(	interaction	4CBZ:A_32
NA	NA	NA	NA	NA	splice_don	ENSG0000(	transcript	ENST0000C
NA	NA	NA	NA	NA	splice_don	ENSG0000(	transcript	ENST0000C
NA	Benign	not_specifi	0.03518	0.01997	start_lost	ENSG0000(	transcript	ENST0000C
NA	Benign	Congenital_	0.00284	0.00100	frameshift_	ENSG0000(	transcript	ENST0000C
NA	NA	NA	NA	NA	splice_don	ENSG0000(	transcript	ENST0000C
NA	NA	NA	NA	NA	splice_don	ENSG0000(	transcript	ENST0000C
NA	NA	NA	NA	NA	splice_acce	ENSG0000(	transcript	ENST0000C
NA	NA	NA	NA	NA	splice_acce	ENSG0000(	transcript	ENST0000C
NA	NA	NA	NA	NA	splice_acce	ENSG0000(	transcript	ENST0000C
NA	NA	NA	NA	NA	stop_gaine	ENSG0000(	transcript	ENST0000C
NA	NA	NA	NA	NA	splice_acce	ENSG0000(	transcript	ENST0000C
NA	NA	NA	NA	NA	frameshift_	ENSG0000(	transcript	ENST0000C
NA	NA	NA	NA	NA	splice_don	ENSG0000(	transcript	ENST0000C
NA	Benign	not_specifi	NA	0.21166	splice_acce	ENSG0000(	transcript	ENST0000C
NA	NA	NA	NA	NA	structural_	ENSG0000(	interaction	2CH9:A_90
NA	Benign	Polyneurop	NA	0.53594	splice_don	ENSG0000(	transcript	ENST0000C
NA	NA	NA	NA	NA	frameshift_	ENSG0000(	transcript	ENST0000C
NA	NA	NA	NA	NA	frameshift_	ENSG0000(	transcript	ENST0000C
NA	NA	NA	NA	NA	splice_acce	ENSG0000(	transcript	ENST0000C
NA	NA	NA	NA	NA	structural_	ENSG0000(	interaction	2NZJ:A_15
Type_1_dia	NA	NA	NA	NA	stop_gaine	ENSG0000(	transcript	ENST0000C
NA	NA	NA	NA	NA	splice_don	ENSG0000(	transcript	ENST0000C
NA	NA	NA	NA	NA	splice_acce	ENSG0000(	transcript	ENST0000C
NA	NA	NA	NA	NA	splice_acce	ENSG0000(	transcript	ENST0000C
NA	NA	NA	NA	NA	splice_acce	ENSG0000(	transcript	ENST0000C
NA	NA	NA	NA	NA	splice_acce	ENSG0000(	transcript	ENST0000C
NA	NA	NA	NA	NA	splice_acce	ENSG0000(	transcript	ENST0000C
NA	NA	NA	NA	NA	splice_acce	ENSG0000(	transcript	ENST0000C
NA	NA	NA	NA	NA	splice_acce	ENSG0000(	transcript	ENST0000C
NA	NA	NA	NA	NA	splice_acce	ENSG0000(	transcript	ENST0000C
NA	NA	NA	NA	NA	frameshift_	ENSG0000(	transcript	ENST0000C
NA	NA	NA	NA	NA	frameshift_	ENSG0000(	transcript	ENST0000C
NA	NA	NA	NA	NA	stop_gaine	ENSG0000(	transcript	ENST0000C
Blood_prot	NA	NA	NA	NA	splice_acce	ENSG0000(	transcript	ENST0000C

NA	NA	NA	NA	NA	frameshift_	ENSG0000(	transcript	ENST0000C
NA	NA	NA	NA	NA	splice_acce	ENSG0000(	transcript	ENST0000C
NA	NA	NA	NA	NA	splice_regi	ENSG0000(	transcript	ENST0000C
NA	NA	NA	NA	NA	splice_regi	ENSG0000(	transcript	ENST0000C
NA	NA	NA	NA	NA	frameshift_	ENSG0000(	transcript	ENST0000C
NA	NA	NA	NA	NA	frameshift_	ENSG0000(	transcript	ENST0000C
NA	NA	NA	NA	NA	frameshift_	ENSG0000(	transcript	ENST0000C
NA	NA	NA	NA	NA	structural_	ENSG0000(	interaction	3S3J:A_629
NA	NA	NA	NA	NA	structural_	ENSG0000(	interaction	4PYG:E_13
NA	NA	NA	NA	NA	stop_gaine	ENSG0000(	transcript	ENST0000C
NA	NA	NA	NA	NA	splice_acce	ENSG0000(	transcript	ENST0000C
NA	NA	NA	NA	NA	splice_acce	ENSG0000(	transcript	ENST0000C
NA	NA	NA	NA	NA	splice_acce	ENSG0000(	transcript	ENST0000C
NA	NA	NA	NA	NA	splice_regi	ENSG0000(	transcript	ENST0000C
NA	NA	NA	NA	NA	frameshift_	ENSG0000(	transcript	ENST0000C
NA	Benign	Severe_cor	0.20570	0.26578	structural_	ENSG0000(	interaction	3IAR:A_15(
NA	Benign/Like	Severe_cor	0.05885	0.05272	structural_	ENSG0000(	interaction	3IAR:A_80-
NA	Benign	not_specifi	0.99554	0.98223	structural_	ENSG0000(	interaction	3IAR:A_12-
NA	NA	NA	NA	NA	frameshift_	ENSG0000(	transcript	ENST0000C
NA	NA	NA	NA	NA	frameshift_	ENSG0000(	transcript	ENST0000C
NA	NA	NA	NA	NA	frameshift_	ENSG0000(	transcript	ENST0000C
NA	NA	NA	NA	NA	frameshift_	ENSG0000(	transcript	ENST0000C
NA	NA	NA	NA	NA	frameshift_	ENSG0000(	transcript	ENST0000C
NA	NA	NA	NA	NA	splice_acce	ENSG0000(	transcript	ENST0000C
NA	NA	NA	NA	NA	splice_acce	ENSG0000(	transcript	ENST0000C
NA	NA	NA	NA	NA	splice_acce	ENSG0000(	transcript	ENST0000C
NA	NA	NA	NA	NA	splice_acce	ENSG0000(	transcript	ENST0000C
NA	NA	NA	NA	NA	structural_	ENSG0000(	interaction	3GEB:A_42
NA	NA	NA	NA	NA	splice_acce	ENSG0000(	transcript	ENST0000C
NA	NA	NA	NA	NA	splice_acce	ENSG0000(	transcript	ENST0000C
NA	NA	NA	NA	NA	splice_acce	ENSG0000(	transcript	ENST0000C
NA	NA	NA	NA	NA	splice_acce	ENSG0000(	transcript	ENST0000C
NA	NA	NA	NA	NA	splice_acce	ENSG0000(	transcript	ENST0000C
NA	NA	NA	NA	NA	splice_acce	ENSG0000(	transcript	ENST0000C
NA	NA	NA	NA	NA	splice_don	ENSG0000(	transcript	ENST0000C
NA	NA	NA	NA	NA	splice_acce	ENSG0000(	transcript	ENST0000C
NA	NA	NA	NA	NA	structural_	ENSG0000(	interaction	2IAG:A_17
NA	NA	NA	NA	NA	start_lost	ENSG0000(	transcript	ENST0000C
NA	NA	NA	NA	NA	frameshift_	ENSG0000(	transcript	ENST0000C
NA	NA	NA	NA	NA	structural_	ENSG0000(	interaction	1A5Y:A_19
NA	NA	NA	NA	NA	structural_	ENSG0000(	interaction	1A02:N_45
NA	NA	NA	NA	NA	splice_acce	ENSG0000(	transcript	ENST0000C
NA	NA	NA	NA	NA	splice_don	ENSG0000(	transcript	ENST0000C
NA	NA	NA	NA	NA	frameshift_	ENSG0000(	transcript	ENST0000C
NA	NA	NA	NA	NA	frameshift_	ENSG0000(	transcript	ENST0000C
NA	NA	NA	NA	NA	frameshift_	ENSG0000(	transcript	ENST0000C
NA	NA	NA	NA	NA	frameshift_	ENSG0000(	transcript	ENST0000C

NA	NA	NA	NA	NA	splice_acce	ENSG0000(	transcript	ENST0000C
NA	NA	NA	NA	NA	splice_don	ENSG0000(	transcript	ENST0000C
NA	Likely_beni	Phosphoen	0.17708	0.12220	structural_	ENSG0000(	interaction	1KHB:A_94
NA	Benign	Phosphoen	0.37883	0.29513	structural_	ENSG0000(	interaction	2GMV:A_1
NA	Likely_beni	Phosphoen	0.02960	0.01558	structural_	ENSG0000(	interaction	1NHX:A_23
Prudent_di	Likely_beni	Phosphoen	0.16768	0.08606	structural_	ENSG0000(	interaction	1KHB:A_27
NA	Likely_beni	Phosphoen	0.02982	0.01617	structural_	ENSG0000(	interaction	1KHE:A_72
NA	NA	NA	NA	NA	frameshift_	ENSG0000(	transcript	ENST0000C
NA	NA	NA	NA	NA	start_lost	ENSG0000(	transcript	ENST0000C
NA	NA	NA	NA	NA	splice_don	ENSG0000(	transcript	ENST0000C
NA	NA	NA	NA	NA	protein_pr	ENSG0000(	interaction	4R3O:C_6-I
NA	NA	NA	NA	NA	splice_don	ENSG0000(	transcript	ENST0000C
NA	NA	NA	NA	NA	stop_lost	ENSG0000(	transcript	ENST0000C
NA	NA	NA	NA	NA	splice_don	ENSG0000(	transcript	ENST0000C
NA	NA	NA	NA	NA	splice_don	ENSG0000(	transcript	ENST0000C
NA	NA	NA	NA	NA	splice_acce	ENSG0000(	transcript	ENST0000C
NA	NA	NA	NA	NA	structural_	ENSG0000(	interaction	4RCI:A_40(
Nicotine_d	NA	NA	NA	NA	splice_acce	ENSG0000(	transcript	ENST0000C
NA	Benign	Dyskeratos	0.93840	0.96546	structural_	ENSG0000(	interaction	3K51:B_58-
NA	NA	NA	NA	NA	structural_	ENSG0000(	interaction	4J6G:D_91-
Atopic_der	NA	NA	NA	NA	structural_	ENSG0000(	interaction	3MHD:D_1
NA	NA	NA	NA	NA	splice_don	ENSG0000(	transcript	ENST0000C
NA	NA	NA	NA	NA	splice_don	ENSG0000(	transcript	ENST0000C
NA	NA	NA	NA	NA	splice_acce	ENSG0000(	transcript	ENST0000C
NA	NA	NA	NA	NA	splice_don	ENSG0000(	transcript	ENST0000C
NA	NA	NA	NA	NA	splice_don	ENSG0000(	transcript	ENST0000C
NA	NA	NA	NA	NA	splice_don	ENSG0000(	transcript	ENST0000C
NA	NA	NA	NA	NA	splice_acce	ENSG0000(	transcript	ENST0000C
NA	NA	NA	NA	NA	bidirection	ENSG0000(	gene_varia	ENSG0000C
NA	NA	NA	NA	NA	splice_acce	ENSG0000(	transcript	ENST0000C
NA	NA	NA	NA	NA	structural_	ENSG0000(	interaction	2WIM:A_3
NA	NA	NA	NA	NA	splice_don	ENSG0000(	transcript	ENST0000C
NA	NA	NA	NA	NA	splice_don	ENSG0000(	transcript	ENST0000C
NA	NA	NA	NA	NA	splice_acce	ENSG0000(	transcript	ENST0000C
NA	NA	NA	NA	NA	splice_acce	ENSG0000(	transcript	ENST0000C
NA	NA	NA	NA	NA	splice_acce	ENSG0000(	transcript	ENST0000C
NA	NA	NA	NA	NA	frameshift_	ENSG0000(	transcript	ENST0000C
NA	NA	NA	NA	NA	splice_don	ENSG0000(	transcript	ENST0000C
NA	NA	NA	NA	NA	stop_gaine	ENSG0000(	transcript	ENST0000C
NA	NA	NA	NA	NA	splice_acce	ENSG0000(	transcript	ENST0000C
NA	NA	NA	NA	NA	splice_acce	ENSG0000(	transcript	ENST0000C
NA	NA	NA	NA	NA	splice_regi	ENSG0000(	transcript	ENST0000C
NA	NA	NA	NA	NA	splice_regi	ENSG0000(	transcript	ENST0000C
NA	NA	NA	NA	NA	splice_regi	ENSG0000(	transcript	ENST0000C
NA	NA	NA	NA	NA	splice_acce	ENSG0000(	transcript	ENST0000C
NA	NA	NA	NA	NA	splice_acce	ENSG0000(	transcript	ENST0000C
NA	NA	NA	NA	NA	splice_acce	ENSG0000(	transcript	ENST0000C

NA	NA	NA	NA	NA	splice_acce	ENSG0000(	transcript	ENST0000C
NA	NA	NA	NA	NA	splice_regi	ENSG0000(	transcript	ENST0000C
NA	NA	NA	NA	NA	splice_acce	ENSG0000(	transcript	ENST0000C
NA	NA	NA	NA	NA	splice_don	ENSG0000(	transcript	ENST0000C
NA	NA	NA	NA	NA	stop_gaine	ENSG0000(	transcript	ENST0000C
NA	NA	NA	NA	NA	frameshift_	ENSG0000(	transcript	ENST0000C
NA	NA	NA	NA	NA	stop_lost	ENSG0000(	transcript	ENST0000C
NA	NA	NA	NA	NA	frameshift_	ENSG0000(	transcript	ENST0000C
NA	NA	NA	NA	NA	gene_fusio	ENSG0000(	gene_varia	ENSG0000C
NA	NA	NA	NA	NA	splice_don	ENSG0000(	transcript	ENST0000C
NA	NA	NA	NA	NA	frameshift_	ENSG0000(	transcript	ENST0000C
NA	NA	NA	NA	NA	frameshift_	ENSG0000(	transcript	ENST0000C
NA	NA	NA	NA	NA	stop_gaine	ENSG0000(	transcript	ENST0000C
NA	NA	NA	NA	NA	splice_don	ENSG0000(	transcript	ENST0000C
NA	NA	NA	NA	NA	structural_	ENSG0000(	interaction	2HRB:A_84
NA	NA	NA	NA	NA	structural_	ENSG0000(	interaction	2HRB:A_7-
NA	NA	NA	NA	NA	structural_	ENSG0000(	interaction	2HRB:A_93
NA	NA	NA	NA	NA	structural_	ENSG0000(	interaction	2HRB:A_13
NA	NA	NA	NA	NA	splice_don	ENSG0000(	transcript	ENST0000C
NA	NA	NA	NA	NA	frameshift_	ENSG0000(	transcript	ENST0000C
NA	NA	NA	NA	NA	frameshift_	ENSG0000(	transcript	ENST0000C
NA	NA	NA	NA	NA	stop_lost	ENSG0000(	transcript	ENST0000C
NA	NA	NA	NA	NA	stop_gaine	ENSG0000(	transcript	ENST0000C
NA	NA	NA	NA	NA	splice_don	ENSG0000(	transcript	ENST0000C
NA	NA	NA	NA	NA	frameshift_	ENSG0000(	transcript	ENST0000C
NA	NA	NA	NA	NA	splice_acce	ENSG0000(	transcript	ENST0000C
NA	NA	NA	NA	NA	stop_gaine	ENSG0000(	transcript	ENST0000C
NA	Benign/Like	not_specifi	NA	NA	splice_acce	ENSG0000(	transcript	ENST0000C
NA	NA	NA	NA	NA	structural_	ENSG0000(	interaction	3K3E:B_24
NA	NA	NA	NA	NA	stop_gaine	ENSG0000(	transcript	ENST0000C
NA	NA	NA	NA	NA	structural_	ENSG0000(	interaction	3FHY:B_86
NA	NA	NA	NA	NA	structural_	ENSG0000(	interaction	2F7K:B_14
NA	NA	NA	NA	NA	splice_acce	ENSG0000(	transcript	ENST0000C
NA	NA	NA	NA	NA	splice_don	ENSG0000(	transcript	ENST0000C
NA	NA	NA	NA	NA	frameshift_	ENSG0000(	transcript	ENST0000C
NA	NA	NA	NA	NA	frameshift_	ENSG0000(	transcript	ENST0000C
Leukocyte_	NA	NA	NA	NA	stop_lost	ENSG0000(	transcript	ENST0000C
NA	NA	NA	NA	NA	stop_gaine	ENSG0000(	transcript	ENST0000C
NA	NA	NA	NA	NA	frameshift_	ENSG0000(	transcript	ENST0000C
NA	NA	NA	NA	NA	frameshift_	ENSG0000(	transcript	ENST0000C
NA	NA	NA	NA	NA	frameshift_	ENSG0000(	transcript	ENST0000C
NA	Benign	Leukocyte_	NA	NA	splice_acce	ENSG0000(	transcript	ENST0000C
NA	NA	NA	NA	NA	splice_don	ENSG0000(	transcript	ENST0000C
NA	NA	NA	NA	NA	frameshift_	ENSG0000(	transcript	ENST0000C
NA	NA	NA	NA	NA	splice_don	ENSG0000(	transcript	ENST0000C
NA	Benign	Knobloch_	NA	NA	stop_gaine	ENSG0000(	transcript	ENST0000C
NA	NA	NA	NA	NA	frameshift_	ENSG0000(	transcript	ENST0000C

NA	NA	NA	NA	NA	frameshift_	ENSG0000(	transcript	ENST0000C
NA	NA	NA	NA	NA	splice_acce	ENSG0000(	transcript	ENST0000C
NA	Benign	Myoscleros	NA	NA	frameshift_	ENSG0000(	transcript	ENST0000C
NA	Benign	not_provid	NA	NA	structural_	ENSG0000(	interaction	1W6K:A_1
NA	NA	NA	NA	NA	structural_	ENSG0000(	interaction	4XYN:A_33
NA	NA	NA	NA	NA	frameshift_	ENSG0000(	transcript	ENST0000C
NA	NA	NA	NA	NA	splice_don	ENSG0000(	transcript	ENST0000C
NA	NA	NA	NA	NA	splice_acce	ENSG0000(	transcript	ENST0000C
NA	NA	NA	NA	NA	stop_gaine	ENSG0000(	transcript	ENST0000C
NA	NA	NA	NA	NA	frameshift_	ENSG0000(	transcript	ENST0000C
NA	NA	NA	NA	NA	stop_gaine	ENSG0000(	transcript	ENST0000C
NA	NA	NA	NA	NA	frameshift_	ENSG0000(	transcript	ENST0000C
NA	NA	NA	NA	NA	frameshift_	ENSG0000(	transcript	ENST0000C
NA	NA	NA	NA	NA	splice_acce	ENSG0000(	transcript	ENST0000C
NA	NA	NA	NA	NA	stop_gaine	ENSG0000(	transcript	ENST0000C
NA	Benign	not_specifi	0.23525	0.22824	structural_	ENSG0000(	interaction	4A04:A_14
NA	NA	NA	NA	NA	frameshift_	ENSG0000(	transcript	ENST0000C
NA	Benign	Tramadol_	0.46797	0.37161	structural_	ENSG0000(	interaction	4PYI:A_62-
NA	Benign	Tramadol_	0.33917	0.29693	structural_	ENSG0000(	interaction	4PYI:A_112
Serum_me	drug_respo	CATECHOL-	0.47275	0.36921	structural_	ENSG0000(	interaction	4PYI:A_124
NA	NA	NA	NA	NA	structural_	ENSG0000(	interaction	4PYI:A_17C
NA	NA	NA	NA	NA	frameshift_	ENSG0000(	transcript	ENST0000C
NA	NA	NA	NA	NA	frameshift_	ENSG0000(	transcript	ENST0000C
NA	NA	NA	NA	NA	frameshift_	ENSG0000(	transcript	ENST0000C
NA	NA	NA	NA	NA	splice_acce	ENSG0000(	transcript	ENST0000C
NA	Benign	not_provid	NA	NA	splice_don	ENSG0000(	transcript	ENST0000C
NA	NA	NA	NA	NA	frameshift_	ENSG0000(	transcript	ENST0000C
NA	NA	NA	NA	NA	frameshift_	ENSG0000(	transcript	ENST0000C
NA	NA	NA	NA	NA	splice_acce	ENSG0000(	transcript	ENST0000C
NA	NA	NA	NA	NA	frameshift_	ENSG0000(	transcript	ENST0000C
NA	NA	NA	NA	NA	splice_don	ENSG0000(	transcript	ENST0000C
NA	NA	NA	NA	NA	splice_acce	ENSG0000(	transcript	ENST0000C
NA	NA	NA	NA	NA	splice_acce	ENSG0000(	transcript	ENST0000C
NA	NA	NA	NA	NA	splice_acce	ENSG0000(	transcript	ENST0000C
NA	NA	NA	NA	NA	frameshift_	ENSG0000(	transcript	ENST0000C
NA	NA	NA	NA	NA	stop_lost	ENSG0000(	transcript	ENST0000C
NA	NA	NA	NA	NA	splice_don	ENSG0000(	transcript	ENST0000C
NA	NA	NA	NA	NA	frameshift_	ENSG0000(	transcript	ENST0000C
NA	NA	NA	NA	NA	frameshift_	ENSG0000(	transcript	ENST0000C
NA	NA	NA	NA	NA	frameshift_	ENSG0000(	transcript	ENST0000C
NA	NA	NA	NA	NA	start_lost	ENSG0000(	transcript	ENST0000C
NA	NA	NA	NA	NA	frameshift_	ENSG0000(	transcript	ENST0000C
NA	NA	NA	NA	NA	frameshift_	ENSG0000(	transcript	ENST0000C
NA	NA	NA	NA	NA	frameshift_	ENSG0000(	transcript	ENST0000C
NA	NA	NA	NA	NA	bidirection	ENSG0000(	gene_varia	ENSG0000C
NA	NA	NA	NA	NA	bidirection	ENSG0000(	gene_varia	ENSG0000C
NA	NA	NA	NA	NA	splice_don	ENSG0000(	transcript	ENST0000C

NA	NA	NA	NA	NA	frameshift_	ENSG0000(	transcript	ENST0000C
NA	NA	NA	NA	NA	stop_lost	ENSG0000(	transcript	ENST0000C
NA	NA	NA	NA	NA	splice_don	ENSG0000(	transcript	ENST0000C
NA	NA	NA	NA	NA	splice_acce	ENSG0000(	transcript	ENST0000C
NA	NA	NA	NA	NA	splice_acce	ENSG0000(	transcript	ENST0000C
NA	NA	NA	NA	NA	splice_acce	ENSG0000(	transcript	ENST0000C
NA	NA	NA	NA	NA	frameshift_	ENSG0000(	transcript	ENST0000C
NA	NA	NA	NA	NA	frameshift_	ENSG0000(	transcript	ENST0000C
NA	NA	NA	NA	NA	frameshift_	ENSG0000(	transcript	ENST0000C
NA	NA	NA	NA	NA	frameshift_	ENSG0000(	transcript	ENST0000C
NA	NA	NA	NA	NA	splice_don	ENSG0000(	transcript	ENST0000C
NA	Pathogenic	Deficiency_	0.00192	0.00040	splice_acce	ENSG0000(	transcript	ENST0000C
NA	NA	NA	NA	NA	start_lost	ENSG0000(	transcript	ENST0000C
NA	NA	NA	NA	NA	structural_	ENSG0000(	interaction	4GDX:A_12
NA	NA	NA	NA	NA	stop_gaine	ENSG0000(	transcript	ENST0000C
NA	NA	NA	NA	NA	structural_	ENSG0000(	interaction	4GDX:A_32
NA	NA	NA	NA	NA	splice_don	ENSG0000(	transcript	ENST0000C
NA	Benign/Like	Congenital_	0.04253	0.02516	structural_	ENSG0000(	interaction	3QK3:A_10
NA	NA	NA	NA	NA	splice_don	ENSG0000(	transcript	ENST0000C
NA	NA	NA	NA	NA	stop_gaine	ENSG0000(	transcript	ENST0000C
NA	NA	NA	NA	NA	structural_	ENSG0000(	interaction	3AP1:A_17
NA	NA	NA	NA	NA	structural_	ENSG0000(	interaction	3AP2:A_17
NA	Benign	Cataract_2	0.01733	0.00899	structural_	ENSG0000(	interaction	3LWK:A_36
NA	Benign	not_specifi	0.89265	0.92372	structural_	ENSG0000(	interaction	3LWK:A_57
NA	NA	NA	NA	NA	splice_acce	ENSG0000(	transcript	ENST0000C
NA	NA	NA	NA	NA	splice_acce	ENSG0000(	transcript	ENST0000C
NA	NA	NA	NA	NA	stop_gaine	ENSG0000(	transcript	ENST0000C
NA	NA	NA	NA	NA	splice_acce	ENSG0000(	transcript	ENST0000C
NA	NA	NA	NA	NA	stop_gaine	ENSG0000(	transcript	ENST0000C
NA	NA	NA	NA	NA	frameshift_	ENSG0000(	transcript	ENST0000C
NA	NA	NA	NA	NA	splice_acce	ENSG0000(	transcript	ENST0000C
NA	NA	NA	NA	NA	splice_acce	ENSG0000(	transcript	ENST0000C
NA	NA	NA	NA	NA	structural_	ENSG0000(	interaction	4OMJ:B_11
NA	NA	NA	NA	NA	structural_	ENSG0000(	interaction	4UYB:A_29
NA	NA	NA	NA	NA	structural_	ENSG0000(	interaction	4UYB:A_31
NA	NA	NA	NA	NA	structural_	ENSG0000(	interaction	4UYB:A_10
NA	NA	NA	NA	NA	structural_	ENSG0000(	interaction	4TLG:A_10
NA	NA	NA	NA	NA	structural_	ENSG0000(	interaction	4TLG:A_95
NA	NA	NA	NA	NA	structural_	ENSG0000(	interaction	4TLG:A_84
NA	NA	NA	NA	NA	structural_	ENSG0000(	interaction	4TLG:A_66
NA	NA	NA	NA	NA	structural_	ENSG0000(	interaction	4TLG:A_3-A
NA	NA	NA	NA	NA	splice_don	ENSG0000(	transcript	ENST0000C
NA	NA	NA	NA	NA	start_lost	ENSG0000(	transcript	ENST0000C
NA	NA	NA	NA	NA	structural_	ENSG0000(	interaction	4TPT:A_36
NA	NA	NA	NA	NA	stop_gaine	ENSG0000(	transcript	ENST0000C
NA	NA	NA	NA	NA	frameshift_	ENSG0000(	transcript	ENST0000C
NA	NA	NA	NA	NA	stop_gaine	ENSG0000(	transcript	ENST0000C

NA	Benign	Parkinson_	0.44003	0.48742	start_lost	ENSG0000	transcript	ENST0000
NA	NA	NA	NA	NA	splice_acce	ENSG0000	transcript	ENST0000
NA	NA	NA	NA	NA	splice_don	ENSG0000	transcript	ENST0000
NA	NA	NA	NA	NA	frameshift_	ENSG0000	transcript	ENST0000
NA	NA	NA	NA	NA	splice_don	ENSG0000	transcript	ENST0000
NA	NA	NA	NA	NA	start_lost	ENSG0000	transcript	ENST0000
NA	NA	NA	NA	NA	splice_acce	ENSG0000	transcript	ENST0000
NA	Benign	not_specifi	0.19598	0.18790	structural_	ENSG0000	interaction	1EGJ:A_33
NA	NA	NA	NA	NA	splice_acce	ENSG0000	transcript	ENST0000
NA	NA	NA	NA	NA	splice_acce	ENSG0000	transcript	ENST0000
NA	NA	NA	NA	NA	splice_acce	ENSG0000	transcript	ENST0000
NA	Benign	Microcytic_	NA	NA	splice_acce	ENSG0000	transcript	ENST0000
NA	Benign	not_specifi	0.17026	0.24740	structural_	ENSG0000	interaction	1DS6:A_22
NA	NA	NA	NA	NA	structural_	ENSG0000	interaction	1HLC:B_41
NA	NA	NA	NA	NA	start_lost	ENSG0000	transcript	ENST0000
NA	NA	NA	NA	NA	structural_	ENSG0000	interaction	2CFR:A_98
NA	NA	NA	NA	NA	splice_don	ENSG0000	transcript	ENST0000
NA	NA	NA	NA	NA	frameshift_	ENSG0000	transcript	ENST0000
NA	NA	NA	NA	NA	structural_	ENSG0000	interaction	4DXT:A_60
NA	NA	NA	NA	NA	structural_	ENSG0000	interaction	3E1U:A_22
NA	Benign	Rubinstein-	0.65725	0.67392	structural_	ENSG0000	interaction	3I3J:A_106
NA	NA	NA	NA	NA	splice_acce	ENSG0000	transcript	ENST0000
Osteoarthr	NA	NA	NA	NA	frameshift_	ENSG0000	transcript	ENST0000
NA	NA	NA	NA	NA	frameshift_	ENSG0000	transcript	ENST0000
Birth_weig	NA	NA	NA	NA	stop_gaine	ENSG0000	transcript	ENST0000
NA	NA	NA	NA	NA	structural_	ENSG0000	interaction	4P0T:A_24
NA	Likely_beni	Debrisoqui	0.00793	0.00479	frameshift_	ENSG0000	transcript	ENST0000
NA	NA	NA	NA	NA	splice_don	ENSG0000	transcript	ENST0000
NA	NA	NA	NA	NA	frameshift_	ENSG0000	transcript	ENST0000
NA	NA	NA	NA	NA	splice_don	ENSG0000	transcript	ENST0000
NA	NA	NA	NA	NA	splice_don	ENSG0000	transcript	ENST0000
NA	NA	NA	NA	NA	structural_	ENSG0000	interaction	1KKQ:A_24
NA	NA	NA	NA	NA	splice_don	ENSG0000	transcript	ENST0000
NA	NA	NA	NA	NA	splice_acce	ENSG0000	transcript	ENST0000
NA	NA	NA	NA	NA	splice_acce	ENSG0000	transcript	ENST0000
NA	NA	NA	NA	NA	splice_regi	ENSG0000	transcript	ENST0000
NA	Benign	not_provid	NA	NA	splice_acce	ENSG0000	transcript	ENST0000
NA	NA	NA	NA	NA	structural_	ENSG0000	interaction	1CM8:A_1
NA	NA	NA	NA	NA	structural_	ENSG0000	interaction	1CM8:A_1
NA	NA	NA	NA	NA	structural_	ENSG0000	interaction	3GC9:A_19
NA	NA	NA	NA	NA	splice_acce	ENSG0000	transcript	ENST0000
NA	NA	NA	NA	NA	splice_acce	ENSG0000	transcript	ENST0000
NA	NA	NA	NA	NA	structural_	ENSG0000	interaction	2IBN:A_15
NA	NA	NA	NA	NA	structural_	ENSG0000	interaction	2IBN:A_18
NA	NA	NA	NA	NA	splice_don	ENSG0000	transcript	ENST0000
NA	Benign	Mitochond	NA	0.14277	structural_	ENSG0000	interaction	2WK5:A_3
NA	Benign	Mitochond	0.17702	0.16314	structural_	ENSG0000	interaction	1UOU:A_3

NA	NA	NA	NA	NA	splice_don	ENSG0000(	transcript	ENST0000C
NA	Uncertain_	Metachron	NA	0.00120	splice_don	ENSG0000(	transcript	ENST0000C
NA	NA	NA	NA	NA	splice_acce	ENSG0000(	transcript	ENST0000C
NA	NA	NA	NA	NA	frameshift_	ENSG0000(	transcript	ENST0000C
NA	NA	NA	NA	NA	frameshift_	ENSG0000(	transcript	ENST0000C
NA	NA	NA	NA	NA	structural_	ENSG0000(	interaction	2P5X:A_92
NA	NA	NA	NA	NA	splice_don	ENSG0000(	transcript	ENST0000C
NA	NA	NA	NA	NA	structural_	ENSG0000(	interaction	4A6D:A_25
NA	NA	NA	NA	NA	structural_	ENSG0000(	interaction	4A6D:A_28
NA	Benign	History_of_	0.11369	0.12080	structural_	ENSG0000(	interaction	2XB6:A_21
NA	NA	NA	NA	NA	structural_	ENSG0000(	interaction	3L5K:A_11
NA	NA	NA	NA	NA	splice_don	ENSG0000(	transcript	ENST0000C
NA	NA	NA	NA	NA	frameshift_	ENSG0000(	transcript	ENST0000C
NA	NA	NA	NA	NA	frameshift_	ENSG0000(	transcript	ENST0000C
NA	NA	NA	NA	NA	splice_don	ENSG0000(	transcript	ENST0000C
NA	NA	NA	NA	NA	splice_don	ENSG0000(	transcript	ENST0000C
NA	NA	NA	NA	NA	splice_acce	ENSG0000(	transcript	ENST0000C
NA	NA	NA	NA	NA	splice_acce	ENSG0000(	transcript	ENST0000C
NA	NA	NA	NA	NA	splice_acce	ENSG0000(	transcript	ENST0000C
NA	NA	NA	NA	NA	splice_acce	ENSG0000(	transcript	ENST0000C
NA	NA	NA	NA	NA	splice_acce	ENSG0000(	transcript	ENST0000C
NA	NA	NA	NA	NA	splice_acce	ENSG0000(	transcript	ENST0000C
NA	NA	NA	NA	NA	splice_acce	ENSG0000(	transcript	ENST0000C
NA	NA	NA	NA	NA	start_lost&	ENSG0000(	transcript	ENST0000C
NA	NA	NA	NA	NA	structural_	ENSG0000(	interaction	3W3M:A_1
NA	NA	NA	NA	NA	structural_	ENSG0000(	interaction	3W3G:A_6
NA	NA	NA	NA	NA	structural_	ENSG0000(	interaction	3W3J:A_75
NA	NA	NA	NA	NA	stop_gaine	ENSG0000(	transcript	ENST0000C
NA	NA	NA	NA	NA	stop_lost&	ENSG0000(	transcript	ENST0000C
NA	NA	NA	NA	NA	frameshift_	ENSG0000(	transcript	ENST0000C
NA	NA	NA	NA	NA	structural_	ENSG0000(	interaction	3ACL:A_20
NA	NA	NA	NA	NA	splice_don	ENSG0000(	transcript	ENST0000C
NA	Benign/Like	not_specifi	NA	NA	splice_don	ENSG0000(	transcript	ENST0000C
NA	NA	NA	NA	NA	structural_	ENSG0000(	interaction	1OI1:A_72-
NA	NA	NA	NA	NA	frameshift_	ENSG0000(	transcript	ENST0000C
NA	NA	NA	NA	NA	frameshift_	ENSG0000(	transcript	ENST0000C
NA	NA	NA	NA	NA	frameshift_	ENSG0000(	transcript	ENST0000C
NA	NA	NA	NA	NA	frameshift_	ENSG0000(	transcript	ENST0000C
NA	NA	NA	NA	NA	stop_gaine	ENSG0000(	transcript	ENST0000C
NA	NA	NA	NA	NA	splice_acce	ENSG0000(	transcript	ENST0000C
NA	NA	NA	NA	NA	splice_acce	ENSG0000(	transcript	ENST0000C
NA	NA	NA	NA	NA	splice_don	ENSG0000(	transcript	ENST0000C
NA	NA	NA	NA	NA	splice_don	ENSG0000(	transcript	ENST0000C
NA	Benign/Like	Non-syndro	NA	0.00053	structural_	ENSG0000(	interaction	1T3G:A_50
NA	NA	NA	NA	NA	splice_don	ENSG0000(	transcript	ENST0000C
NA	NA	NA	NA	NA	stop_gaine	ENSG0000(	transcript	ENST0000C
NA	NA	NA	NA	NA	frameshift_	ENSG0000(	transcript	ENST0000C

NA	NA	NA	NA	NA	splice_acce	ENSG0000(	transcript	ENST0000C
NA	NA	NA	NA	NA	splice_don	ENSG0000(	transcript	ENST0000C
NA	NA	NA	NA	NA	stop_lost	ENSG0000(	transcript	ENST0000C
NA	NA	NA	NA	NA	frameshift_	ENSG0000(	transcript	ENST0000C
NA	NA	NA	NA	NA	frameshift_	ENSG0000(	transcript	ENST0000C
NA	NA	NA	NA	NA	frameshift_	ENSG0000(	transcript	ENST0000C
NA	NA	NA	NA	NA	frameshift_	ENSG0000(	transcript	ENST0000C
NA	NA	NA	NA	NA	splice_acce	ENSG0000(	transcript	ENST0000C
NA	Conflicting_	Non-syndro	0.00585	0.00159	stop_gaine	ENSG0000(	transcript	ENST0000C
NA	NA	NA	NA	NA	splice_acce	ENSG0000(	transcript	ENST0000C
NA	NA	NA	NA	NA	splice_regi	ENSG0000(	transcript	ENST0000C
NA	NA	NA	NA	NA	splice_regi	ENSG0000(	transcript	ENST0000C
NA	NA	NA	NA	NA	splice_regi	ENSG0000(	transcript	ENST0000C
NA	NA	NA	NA	NA	splice_regi	ENSG0000(	transcript	ENST0000C
NA	Benign/Like	Retinitis_pi	0.01910	0.00662	structural_	ENSG0000(	interaction	3BH6:B_28
NA	NA	NA	NA	NA	splice_acce	ENSG0000(	transcript	ENST0000C
NA	NA	NA	NA	NA	splice_regi	ENSG0000(	transcript	ENST0000C
NA	NA	NA	NA	NA	frameshift_	ENSG0000(	transcript	ENST0000C
NA	NA	NA	NA	NA	splice_acce	ENSG0000(	transcript	ENST0000C
NA	NA	NA	NA	NA	splice_acce	ENSG0000(	transcript	ENST0000C
NA	NA	NA	NA	NA	frameshift_	ENSG0000(	transcript	ENST0000C
NA	NA	NA	NA	NA	frameshift_	ENSG0000(	transcript	ENST0000C
NA	NA	NA	NA	NA	frameshift_	ENSG0000(	transcript	ENST0000C
NA	NA	NA	NA	NA	frameshift_	ENSG0000(	transcript	ENST0000C
NA	NA	NA	NA	NA	splice_acce	ENSG0000(	transcript	ENST0000C
NA	NA	NA	NA	NA	splice_acce	ENSG0000(	transcript	ENST0000C
NA	NA	NA	NA	NA	splice_acce	ENSG0000(	transcript	ENST0000C
NA	Benign	not_provid	NA	NA	structural_	ENSG0000(	interaction	3G1N:B_43
NA	NA	NA	NA	NA	frameshift_	ENSG0000(	transcript	ENST0000C
NA	NA	NA	NA	NA	frameshift_	ENSG0000(	transcript	ENST0000C
NA	NA	NA	NA	NA	frameshift_	ENSG0000(	transcript	ENST0000C
NA	NA	NA	NA	NA	splice_don	ENSG0000(	transcript	ENST0000C
NA	NA	NA	NA	NA	start_lost	ENSG0000(	transcript	ENST0000C
NA	NA	NA	NA	NA	frameshift_	ENSG0000(	transcript	ENST0000C
NA	NA	NA	NA	NA	frameshift_	ENSG0000(	transcript	ENST0000C
NA	NA	NA	NA	NA	splice_acce	ENSG0000(	transcript	ENST0000C
NA	NA	NA	NA	NA	splice_acce	ENSG0000(	transcript	ENST0000C
NA	NA	NA	NA	NA	structural_	ENSG0000(	interaction	1E5W:A_13
NA	NA	NA	NA	NA	stop_gaine	ENSG0000(	transcript	ENST0000C
NA	NA	NA	NA	NA	splice_acce	ENSG0000(	transcript	ENST0000C
NA	NA	NA	NA	NA	splice_acce	ENSG0000(	transcript	ENST0000C
NA	NA	NA	NA	NA	splice_acce	ENSG0000(	transcript	ENST0000C
NA	NA	NA	NA	NA	structural_	ENSG0000(	interaction	1EQF:A_13
NA	NA	NA	NA	NA	splice_acce	ENSG0000(	transcript	ENST0000C
NA	NA	NA	NA	NA	splice_acce	ENSG0000(	transcript	ENST0000C
NA	Benign	History_of_	0.24877	0.41748	structural_	ENSG0000(	interaction	1VKG:A_53
NA	NA	NA	NA	NA	splice_acce	ENSG0000(	transcript	ENST0000C

NA	NA	NA	NA	NA	splice_don	ENSG0000(	transcript	ENST0000C
NA	NA	NA	NA	NA	stop_gaine	ENSG0000(	transcript	ENST0000C
NA	NA	NA	NA	NA	splice_don	ENSG0000(	transcript	ENST0000C
NA	NA	NA	NA	NA	splice_acce	ENSG0000(	transcript	ENST0000C
NA	NA	NA	NA	NA	splice_acce	ENSG0000(	transcript	ENST0000C
NA	NA	NA	NA	NA	splice_acce	ENSG0000(	transcript	ENST0000C
NA	NA	NA	NA	NA	splice_acce	ENSG0000(	transcript	ENST0000C
NA	NA	NA	NA	NA	splice_acce	ENSG0000(	transcript	ENST0000C
NA	NA	NA	NA	NA	splice_acce	ENSG0000(	transcript	ENST0000C
NA	NA	NA	NA	NA	3_prime_U	ENSG0000(	transcript	ENST0000C
NA	NA	NA	NA	NA	stop_lost	ENSG0000(	transcript	ENST0000C
NA	NA	NA	NA	NA	splice_acce	ENSG0000(	transcript	ENST0000C
NA	NA	NA	NA	NA	splice_acce	ENSG0000(	transcript	ENST0000C
NA	NA	NA	NA	NA	splice_acce	ENSG0000(	transcript	ENST0000C
NA	NA	NA	NA	NA	splice_acce	ENSG0000(	transcript	ENST0000C
NA	NA	NA	NA	NA	splice_acce	ENSG0000(	transcript	ENST0000C
NA	NA	NA	NA	NA	splice_acce	ENSG0000(	transcript	ENST0000C
NA	Benign	X-linked_a	0.32080	0.50808	structural_	ENSG0000(	interaction	4YHF:A_58
NA	NA	NA	NA	NA	frameshift_	ENSG0000(	transcript	ENST0000C
NA	NA	NA	NA	NA	frameshift_	ENSG0000(	transcript	ENST0000C
NA	NA	NA	NA	NA	stop_gaine	ENSG0000(	transcript	ENST0000C
NA	NA	NA	NA	NA	splice_acce	ENSG0000(	transcript	ENST0000C
NA	NA	NA	NA	NA	splice_acce	ENSG0000(	transcript	ENST0000C
NA	NA	NA	NA	NA	splice_acce	ENSG0000(	transcript	ENST0000C
NA	NA	NA	NA	NA	splice_acce	ENSG0000(	transcript	ENST0000C
NA	NA	NA	NA	NA	frameshift_	ENSG0000(	transcript	ENST0000C
NA	NA	NA	NA	NA	splice_acce	ENSG0000(	transcript	ENST0000C
NA	NA	NA	NA	NA	splice_acce	ENSG0000(	transcript	ENST0000C
NA	NA	NA	NA	NA	splice_acce	ENSG0000(	transcript	ENST0000C
NA	NA	NA	NA	NA	splice_acce	ENSG0000(	transcript	ENST0000C
NA	NA	NA	NA	NA	splice_acce	ENSG0000(	transcript	ENST0000C
NA	NA	NA	NA	NA	splice_acce	ENSG0000(	transcript	ENST0000C
NA	NA	NA	NA	NA	splice_acce	ENSG0000(	transcript	ENST0000C
NA	Benign	Deafness,_	0.43294	0.49113	stop_lost	ENSG0000(	transcript	ENST0000C
NA	NA	NA	NA	NA	splice_don	ENSG0000(	transcript	ENST0000C
NA	NA	NA	NA	NA	splice_acce	ENSG0000(	transcript	ENST0000C
NA	NA	NA	NA	NA	splice_regi	ENSG0000(	transcript	ENST0000C
NA	NA	NA	NA	NA	splice_don	ENSG0000(	transcript	ENST0000C
NA	NA	NA	NA	NA	frameshift_	ENSG0000(	transcript	ENST0000C
NA	NA	NA	NA	NA	splice_acce	ENSG0000(	transcript	ENST0000C
NA	NA	NA	NA	NA	splice_acce	ENSG0000(	transcript	ENST0000C
NA	NA	NA	NA	NA	splice_acce	ENSG0000(	transcript	ENST0000C
NA	NA	NA	NA	NA	splice_acce	ENSG0000(	transcript	ENST0000C
NA	NA	NA	NA	NA	splice_don	ENSG0000(	transcript	ENST0000C
NA	NA	NA	NA	NA	splice_don	ENSG0000(	transcript	ENST0000C
NA	NA	NA	NA	NA	frameshift_	ENSG0000(	transcript	ENST0000C
NA	NA	NA	NA	NA	frameshift_	ENSG0000(	transcript	ENST0000C

NA	not_provid	CIC-DUX_Sa	NA	NA	frameshift_	ENSG0000(	transcript	ENST0000C
NA	NA	NA	NA	NA	frameshift_	ENSG0000(	transcript	ENST0000C
NA	NA	NA	NA	NA	frameshift_	ENSG0000(	transcript	ENST0000C
NA	NA	NA	NA	NA	frameshift_	ENSG0000(	transcript	ENST0000C
NA	NA	NA	NA	NA	intron_vari	ENSG0000(	transcript	ENST0000C
NA	NA	NA	NA	NA	frameshift_	ENSG0000(	transcript	ENST0000C
NA	NA	NA	NA	NA	intron_vari	ENSG0000(	transcript	ENST0000C
NA	NA	NA	NA	NA	frameshift_	ENSG0000(	transcript	ENST0000C
NA	NA	NA	NA	NA	stop_lost	ENSG0000(	transcript	ENST0000C
NA	NA	NA	NA	NA	splice_don	ENSG0000(	transcript	ENST0000C
NA	NA	NA	NA	NA	splice_don	ENSG0000(	transcript	ENST0000C
NA	NA	NA	NA	NA	structural_	ENSG0000(	interaction	2WA0:A_2
NA	NA	NA	NA	NA	stop_gaine	ENSG0000(	transcript	ENST0000C
NA	NA	NA	NA	NA	splice_don	ENSG0000(	transcript	ENST0000C
NA	NA	NA	NA	NA	frameshift_	ENSG0000(	transcript	ENST0000C
NA	NA	NA	NA	NA	splice_don	ENSG0000(	transcript	ENST0000C
NA	NA	NA	NA	NA	frameshift_	ENSG0000(	transcript	ENST0000C
NA	NA	NA	NA	NA	splice_don	ENSG0000(	transcript	ENST0000C
NA	Benign	Anemia,_n	0.83279	0.78411	structural_	ENSG0000(	interaction	1QKI:A_17(
NA	NA	NA	NA	NA	splice_don	ENSG0000(	transcript	ENST0000C
NA	NA	NA	NA	NA	splice_acce	ENSG0000(	transcript	ENST0000C
NA	NA	NA	NA	NA	splice_acce	ENSG0000(	transcript	ENST0000C
NA	NA	NA	NA	NA	structural_	ENSG0000(	interaction	3ZLI:B_969

) annotation		LOF	Extra
SO: BioTyp	N of transc	Fraction af	Annotation of all potetional transcripts
protein_coding			G|splice_donor_variant&disruptive_inframe_deletion&splice_region
protein_coding			T|structural_interaction_variant|HIGH|ISG15|ENSG00000187608|in
protein_coding			A|structural_interaction_variant|HIGH|ISG15|ENSG00000187608|ir
processed_transcript			C|splice_donor_variant&intron_variant|HIGH|RP11-465B22.3|ENSG
protein_coding			A|stop_gained|HIGH|CPSF3L|ENSG00000127054|transcript|ENST0(
protein_co	20	0.25	C|splice_acceptor_variant&splice_region_variant&intron_variant|HI
protein_co	1	1	AATT|frameshift_variant|HIGH|AL645728.1|ENSG00000279244|tra
protein_co	1	1	AATT|frameshift_variant|HIGH|AL645728.1|ENSG00000279244|tra
protein_co	1	1	AATT|frameshift_variant|HIGH|AL645728.1|ENSG00000279244|tra
protein_co	32	0.06	C|start_lost|HIGH|MIB2|ENSG00000197530|transcript|ENST00000!
protein_co	9	0.67	G|frameshift_variant|HIGH|CDK11B|ENSG00000248333|transcript|
protein_co	9	0.89	C|splice_donor_variant&intron_variant|HIGH|CDK11B|ENSG000002
processed_transcript			C|splice_donor_variant&splice_region_variant&intron_variant|HIGH
protein_coding			T|structural_interaction_variant|HIGH|TP73|ENSG00000078900|int
processed_transcript			T|splice_donor_variant&intron_variant|HIGH|TP73-AS1|ENSG0000(
nonsense_mediated_decay			T|stop_gained|HIGH|LRRC47|ENSG00000130764|transcript|ENST0(
lincRNA			G|splice_acceptor_variant&intron_variant|HIGH|RP1-37J18.2|ENSG
protein_co	11	0.27	A|splice_acceptor_variant&intron_variant|HIGH|NPHP4|ENSG0000(
protein_coding			C|structural_interaction_variant|HIGH|KCNAB2|ENSG00000069424
protein_co	9	0.11	G|splice_acceptor_variant&intron_variant|HIGH|PER3|ENSG00000C
protein_coding			A|structural_interaction_variant|HIGH|PARK7|ENSG00000116288|i
protein_coding			C|structural_interaction_variant|HIGH|CA6|ENSG00000131686|inte
protein_co	3	0.33	C|frameshift_variant|HIGH|H6PD|ENSG00000049239|transcript|EN
protein_co	5	0.4	A|splice_donor_variant&intron_variant|HIGH|C1orf127|ENSG0000C
processed_pseudogene			G|splice_acceptor_variant&intron_variant|HIGH|CFL1P6|ENSG0000
protein_co	9	0.22	C|splice_donor_variant&intron_variant|HIGH|MAD2L2|ENSG00000
protein_co	9	0.11	T|splice_acceptor_variant&intron_variant|HIGH|MAD2L2|ENSG000
protein_co	7	0.57	T|stop_gained|HIGH|C1orf167|ENSG00000215910|transcript|ENST
protein_co	3	0.33	G|stop_lost|HIGH|NPPA|ENSG00000175206|transcript|ENST00000
protein_co	2	0.5	A|stop_gained|HIGH|AADACL3|ENSG00000188984|transcript|ENST
protein_co	1	1	T|stop_gained|HIGH|PRAMEF2|ENSG00000120952|transcript|ENST
protein_co	1	1	CG|frameshift_variant|HIGH|PRAMEF4|ENSG00000243073|transcri
processed_pseudogene			C|splice_acceptor_variant&splice_region_variant&intron_variant|HI
protein_coding			AC|frameshift_variant|HIGH|CASP9|ENSG00000132906|transcript|
processed_transcript			CTTT|splice_acceptor_variant&intron_variant|HIGH|DNAJC16|ENSG
processed_transcript			CTTT|splice_acceptor_variant&intron_variant|HIGH|DNAJC16|ENSG
processed_transcript			CTTT|splice_acceptor_variant&intron_variant|HIGH|DNAJC16|ENSG
processed_transcript			CTTT|splice_acceptor_variant&intron_variant|HIGH|DNAJC16|ENSG
protein_co	2	0.5	CGA|frameshift_variant|HIGH|TMEM82|ENSG00000162460|transcr
protein_co	2	0.5	TG|frameshift_variant|HIGH|TMEM82|ENSG00000162460|transcrip
protein_co	5	0.2	C|frameshift_variant|HIGH|SPEN|ENSG00000065526|transcript|EN
protein_coding			A|structural_interaction_variant|HIGH|SPEN|ENSG00000065526|in
protein_coding			T|structural_interaction_variant|HIGH|EPHA2|ENSG00000142627|i
protein_coding			A|structural_interaction_variant|HIGH|EPHA2|ENSG00000142627|i

protein_coding			T|structural_interaction_variant|HIGH|EPHA2|ENSG00000142627|i
protein_coding			T|structural_interaction_variant|HIGH|EPHA2|ENSG00000142627|i
lincRNA			ATTTTTTT|splice_acceptor_variant&intron_variant|HIGH|RP1-163M
lincRNA			ATTTTTTT|splice_acceptor_variant&intron_variant|HIGH|RP1-163M
lincRNA			ATTTTTTT|splice_acceptor_variant&intron_variant|HIGH|RP1-163M
lincRNA			ATTTTTTT|splice_acceptor_variant&intron_variant|HIGH|RP1-163M
lincRNA			ATTTTTTT|splice_acceptor_variant&intron_variant|HIGH|RP1-163M
lincRNA			ATTTTTTT|splice_acceptor_variant&intron_variant|HIGH|RP1-163M
lincRNA			CG|splice_donor_variant&intron_variant|HIGH|RP11-108M9.3|ENS
lincRNA			CG|splice_donor_variant&intron_variant|HIGH|RP11-108M9.3|ENS
protein_coding			T|structural_interaction_variant|HIGH|PADI2|ENSG00000117115|ir
protein_co	1	1	G|frameshift_variant&splice_donor_variant&splice_region_variant&
protein_coding			C|structural_interaction_variant|HIGH|PADI4|ENSG00000159339|ir
protein_coding			G|structural_interaction_variant|HIGH|PADI4|ENSG00000159339|ir
protein_coding			A|structural_interaction_variant|HIGH|PADI4|ENSG00000159339|ir
protein_coding			T|structural_interaction_variant|HIGH|ALDH4A1|ENSG0000015942
protein_coding			C|structural_interaction_variant|HIGH|ALDH4A1|ENSG0000015942
protein_coding			T|structural_interaction_variant|HIGH|ALDH4A1|ENSG0000015942
protein_coding			G|structural_interaction_variant|HIGH|ALDH4A1|ENSG0000015942
protein_coding			C|structural_interaction_variant|HIGH|ALDH4A1|ENSG0000015942
protein_coding			G|structural_interaction_variant|HIGH|ALDH4A1|ENSG0000015942
protein_coding			GC|frameshift_variant|HIGH|UBR4|ENSG00000127481|transcript|E
polymorph	4	0.75	T|frameshift_variant|HIGH|AKR7L|ENSG00000211454|transcript|E
polymorph	4	0.25	G|stop_lost|HIGH|AKR7L|ENSG00000211454|transcript|ENST0000
polymorph	4	0.5	C|frameshift_variant|HIGH|AKR7L|ENSG00000211454|transcript|E
protein_coding			C|splice_acceptor_variant&conservative_inframe_deletion&splice_r
nonsense_	4	0.25	C|frameshift_variant|HIGH|MINOS1-NBL1|ENSG00000270136|trans
processed_transcript			A|splice_acceptor_variant&intron_variant|HIGH|PLA2G5|ENSG0000
protein_co	3	0.67	A|stop_gained|HIGH|PLA2G2C|ENSG00000187980|transcript|ENST
unprocessed_pseudogene			C|splice_acceptor_variant&splice_region_variant&intron_variant|HI
unprocessed_pseudogene			C|splice_acceptor_variant&splice_region_variant&intron_variant|HI
unprocessed_pseudogene			C|splice_acceptor_variant&splice_region_variant&intron_variant|HI
nonsense_mediated_decay			G|stop_lost|HIGH|RAP1GAP|ENSG00000076864|transcript|ENST00
protein_coding			T|structural_interaction_variant|HIGH|RAP1GAP|ENSG0000007686
protein_co	15	0.07	G|frameshift_variant|HIGH|USP48|ENSG00000090686|transcript|E
protein_co	15	0.07	G|frameshift_variant|HIGH|USP48|ENSG00000090686|transcript|E
protein_co	4	0.5	A|splice_donor_variant&intron_variant|HIGH|CELA3B|ENSG000002
lincRNA			G|splice_donor_variant&intron_variant|HIGH|LINC00339|ENSG0000
pseudogene			C|splice_acceptor_variant&splice_donor_variant&splice_region_vari
pseudogene			C|splice_acceptor_variant&splice_donor_variant&splice_region_vari
pseudogene			G|splice_donor_variant&splice_region_variant&intron_variant&non
pseudogene			G|splice_donor_variant&splice_region_variant&intron_variant&non
protein_co	3	0.67	G|splice_acceptor_variant&intron_variant|HIGH|WNT4|ENSG00000
protein_coding			T|stop_gained|HIGH|LACTBL1|ENSG00000215906|transcript|ENST
protein_coding			A|structural_interaction_variant|HIGH|ASAP3|ENSG00000088280|i
protein_coding			A|structural_interaction_variant|HIGH|ASAP3|ENSG00000088280|i
unprocessed_pseudogene			T|splice_acceptor_variant&intron_variant|HIGH|RP11-223J15.2|ENS

nonsense_mediated_decay			T|stop_gained|HIGH|HMGCL|ENSG00000117305|transcript|ENST0(
protein_coding			T|structural_interaction_variant|HIGH|IL22RA1|ENSG00000142677
antisense			CTT|splice_acceptor_variant&intron_variant|HIGH|RP4-706G24.1|E
antisense			C|splice_region_variant&intron_variant|LOW|RP4-706G24.1|ENSG(
protein_co	14	0.36	A|frameshift_variant|HIGH|CNKSR1|ENSG00000142675|transcript|
protein_co	16	0.06	T|frameshift_variant|HIGH|UBXN11|ENSG00000158062|transcript|
protein_co	16	0.06	T|frameshift_variant|HIGH|UBXN11|ENSG00000158062|transcript|
protein_co	16	0.13	A|frameshift_variant|HIGH|UBXN11|ENSG00000158062|transcript|
protein_co	16	0.13	A|frameshift_variant|HIGH|UBXN11|ENSG00000158062|transcript|
protein_co	16	0.13	C|frameshift_variant|HIGH|UBXN11|ENSG00000158062|transcript|
protein_co	16	0.13	C|frameshift_variant|HIGH|UBXN11|ENSG00000158062|transcript|
protein_co	16	0.25	A|frameshift_variant|HIGH|UBXN11|ENSG00000158062|transcript|
protein_co	16	0.25	A|frameshift_variant|HIGH|UBXN11|ENSG00000158062|transcript|
protein_co	16	0.25	A|frameshift_variant|HIGH|UBXN11|ENSG00000158062|transcript|
protein_co	16	0.38	G|frameshift_variant|HIGH|UBXN11|ENSG00000158062|transcript|
protein_co	10	0.1	G|frameshift_variant|HIGH|AIM1L|ENSG00000176092|transcript|E
protein_co	10	0.1	A|frameshift_variant|HIGH|AIM1L|ENSG00000176092|transcript|E
protein_co	10	0.1	G|frameshift_variant|HIGH|AIM1L|ENSG00000176092|transcript|E
nonsense_	20	0.05	C|frameshift_variant|HIGH|RPS6KA1|ENSG00000117676|transcript
nonsense_mediated_decay			C|stop_lost|HIGH|RPS6KA1|ENSG00000117676|transcript|ENST000
protein_coding			C|structural_interaction_variant|HIGH|RPS6KA1|ENSG0000011767(
protein_co	11	0.36	GGCCGCCGCCCGGC|frameshift_variant|HIGH|SYTL1|ENSG0000014Z
protein_co	4	0.5	A|frameshift_variant|HIGH|FCN3|ENSG00000142748|transcript|EN
protein_co	6	0.17	TC|frameshift_variant|HIGH|SMPDL3B|ENSG00000130768|transcri|
protein_co	6	0.17	CG|frameshift_variant|HIGH|SMPDL3B|ENSG00000130768|transcri
protein_coding			A|structural_interaction_variant|HIGH|MECR|ENSG00000116353|ir
protein_co	15	0.07	T|splice_donor_variant&intron_variant|HIGH|ADGRB2|ENSG00000:
processed_transcript			C|splice_donor_variant&intron_variant|HIGH|PTP4A2|ENSG000001
protein_coding			TG|frameshift_variant|HIGH|DCDC2B|ENSG00000222046|transcrip
protein_coding			C|structural_interaction_variant|HIGH|RBBP4|ENSG00000162521|i
protein_co	5	0.6	GGC|frameshift_variant|HIGH|KIAA1522|ENSG00000162522|transc
protein_coding			T|structural_interaction_variant|HIGH|AK2|ENSG00000004455|inte
protein_coding			A|structural_interaction_variant|HIGH|AK2|ENSG00000004455|inte
protein_co	1	1	T|frameshift_variant|HIGH|A3GALT2|ENSG00000184389|transcript
protein_co	4	0.5	GCATGAAAAGGCCAAATATGAAGCCCTGGCCAAACTCGACAAAGCCCGA
protein_co	1	1	T|frameshift_variant|HIGH|GJB4|ENSG00000189433|transcript|EN!
lincRNA			C|splice_donor_variant&intron_variant|HIGH|RP11-244H3.1|ENSG(
nonsense_	11	0.09	T|splice_acceptor_variant&intron_variant|HIGH|EPHA10|ENSG0000(
protein_coding			T|structural_interaction_variant|HIGH|INPP5B|ENSG00000204084|
antisense			A|splice_donor_variant&intron_variant|HIGH|RP11-420K8.1|ENSG(
nonsense_	15	0.07	CA|frameshift_variant|HIGH|TRIT1|ENSG00000043514|transcript|E
protein_coding			G|structural_interaction_variant|HIGH|PPT1|ENSG00000131238|in
processed_transcript			CTTT|splice_acceptor_variant&intron_variant|HIGH|TMCO2|ENSG0
processed_transcript			CTTT|splice_acceptor_variant&intron_variant|HIGH|TMCO2|ENSG0
processed_transcript			CTTT|splice_acceptor_variant&intron_variant|HIGH|TMCO2|ENSG0
protein_co	4	0.25	T|frameshift_variant|HIGH|ZMPSTE24|ENSG00000084073|transcrip
protein_co	10	0.3	CG|frameshift_variant|HIGH|EXO5|ENSG00000164002|transcript|E

protein_coding			G|structural_interaction_variant|HIGH|CTPS1|ENSG00000171793|i
protein_co	1	1	CGGGACGCCCGCCTACTTCGGCGGCTG|frameshift_variant|HIGH|FOX
protein_co	1	1	CGGGACGCCCGCCTACTTCGGCGGCTGCAAGGGCGGCGCCTACGGCGGG
antisense			T|splice_donor_variant&intron_variant|HIGH|RP1-92O14.3|ENSG00
protein_co	5	0.2	C|splice_acceptor_variant&intron_variant|HIGH|KDM4A|ENSG0000
protein_coding			T|protein_protein_contact|HIGH|IPO13|ENSG00000117408|interac
nonsense_	11	0.18	C|splice_acceptor_variant&intron_variant|HIGH|CCDC24|ENSG0000
protein_coding			C|structural_interaction_variant|HIGH|DMAP1|ENSG00000178028|
pseudogene			A|splice_donor_variant&splice_region_variant&intron_variant|HIGH
protein_co	10	0.1	G|splice_acceptor_variant&splice_donor_variant&intron_variant|HI
protein_coding			C|structural_interaction_variant|HIGH|NSUN4|ENSG00000117481|
protein_co	4	0.25	A|stop_gained|HIGH|MOB3C|ENSG00000142961|transcript|ENST0
protein_co	14	0.29	G|frameshift_variant&splice_region_variant|HIGH|CYP4B1|ENSG00
nonsense_mediated_decay			A|stop_gained|HIGH|CYP4B1|ENSG00000142973|transcript|ENST0
lincRNA			G|splice_donor_variant&splice_region_variant&intron_variant&non_
protein_co	1	1	C|frameshift_variant|HIGH|AL356289.1|ENSG00000279096|transcr
protein_co	1	1	C|frameshift_variant|HIGH|AL356289.1|ENSG00000279096|transcr
protein_co	10	0.3	T|stop_gained|HIGH|SLC5A9|ENSG00000117834|transcript|ENST00
protein_co	18	0.06	G|frameshift_variant|HIGH|TTC39A|ENSG00000085831|transcript|
protein_co	18	0.06	G|frameshift_variant|HIGH|TTC39A|ENSG00000085831|transcript|
protein_co	18	0.06	G|frameshift_variant|HIGH|TTC39A|ENSG00000085831|transcript|
processed_pseudogene			A|splice_donor_variant&intron_variant|HIGH|DNAJC19P7|ENSG000
protein_co	3	0.67	G|start_lost|HIGH|ZYG11A|ENSG00000203995|transcript|ENST000
unprocessed_pseudogene			A|splice_donor_variant&intron_variant|HIGH|RP5-1024G6.9|ENSG0
unprocessed_pseudogene			A|splice_acceptor_variant&intron_variant|HIGH|RP5-1024G6.9|ENS
protein_co	8	0.5	G|splice_donor_variant&intron_variant|HIGH|YIPF1|ENSG0000005
protein_co	2	0.5	TG|frameshift_variant|HIGH|CDCP2|ENSG00000157211|transcript|
protein_co	2	0.5	TG|frameshift_variant|HIGH|CDCP2|ENSG00000157211|transcript|
protein_co	2	0.5	GC|frameshift_variant|HIGH|CDCP2|ENSG00000157211|transcript|
protein_coding			T|structural_interaction_variant|HIGH|ACOT11|ENSG00000162390
protein_coding			A|structural_interaction_variant|HIGH|ACOT11|ENSG00000162390
protein_coding			A|structural_interaction_variant|HIGH|ACOT11|ENSG00000162390
protein_co	2	0.5	C|frameshift_variant|HIGH|FAM151A|ENSG00000162391|transcrip
nonsense_	4	0.25	A|stop_gained|HIGH|MROH7-TTC4|ENSG00000271723|transcript|E
protein_coding			A|stop_gained|HIGH|TTC22|ENSG00000006555|transcript|ENST00
protein_co	2	1	GA|frameshift_variant|HIGH|LEXM|ENSG00000162398|transcript|E
protein_coding			G|structural_interaction_variant|HIGH|PCSK9|ENSG00000169174|i
lincRNA			C|splice_donor_variant&splice_region_variant&intron_variant&non_
lincRNA			TC|splice_acceptor_variant&intron_variant|HIGH|RP11-504A18.1|E
protein_coding			A|structural_interaction_variant|HIGH|PRKAA2|ENSG00000162409
protein_co	6	0.5	A|stop_gained|HIGH|C8B|ENSG00000021852|transcript|ENST0000
antisense			C|splice_donor_variant&intron_variant|HIGH|DAB1-AS1|ENSG0000
lincRNA			G|splice_donor_variant&intron_variant|HIGH|RP4-794H19.1|ENSG0
antisense			C|splice_donor_variant&intron_variant|HIGH|RP4-782L23.2|ENSG0
protein_co	14	0.64	C|splice_acceptor_variant&intron_variant|HIGH|NFIA|ENSG000001
protein_coding			CAA|frameshift_variant|HIGH|PATJ|ENSG00000132849|transcript|I
protein_coding			CAA|frameshift_variant|HIGH|PATJ|ENSG00000132849|transcript|I

protein_co	16	0.06	T|stop_gained|HIGH|PATJ|ENSG00000132849|transcript|ENST0000[illegible]
protein_co	1	1	G|frameshift_variant|HIGH|L1TD1|ENSG00000240563|transcript|E
lincRNA			T|splice_donor_variant&intron_variant|HIGH|RP11-5P4.2|ENSG000
lincRNA			A|splice_donor_variant&intron_variant|HIGH|LINC00466|ENSG000[illegible]
protein_co	5	0.2	A|splice_donor_variant&intron_variant|HIGH|PGM1|ENSG0000007
protein_coding			C|structural_interaction_variant|HIGH|JAK1|ENSG00000162434|int
protein_coding			A|structural_interaction_variant|HIGH|JAK1|ENSG00000162434|int
lincRNA			G|splice_acceptor_variant&intron_variant|HIGH|LINC01359|ENSG0
protein_coding			A|structural_interaction_variant|HIGH|PDE4B|ENSG00000184588|i
protein_co	6	0.33	A|splice_donor_variant&intron_variant|HIGH|TCTEX1D1|ENSG0000
protein_co	8	0.25	C|frameshift_variant|HIGH|C1orf141|ENSG00000203963|transcript
antisense			A|splice_donor_variant&intron_variant|HIGH|GNG12-AS1|ENSG000
protein_co	1	1	GT|frameshift_variant|HIGH|AL158839.1|ENSG00000279230|trans
protein_coding			T|structural_interaction_variant|HIGH|CTH|ENSG00000116761|inte
lincRNA			T|splice_acceptor_variant&intron_variant|HIGH|ZRANB2-AS2|ENSG
lincRNA			C|splice_donor_variant&intron_variant|HIGH|LINC01360|ENSG000[illegible]
protein_coding			A|structural_interaction_variant|HIGH|CRYZ|ENSG00000116791|in
protein_co	8	0.25	G|start_lost|HIGH|TYW3|ENSG00000162623|transcript|ENST00000
protein_co	18	0.06	T|splice_donor_variant&intron_variant|HIGH|USP33|ENSG0000007
protein_co	9	0.11	GT|frameshift_variant|HIGH|IFI44|ENSG00000137965|transcript|E
antisense			C|splice_acceptor_variant&splice_region_variant&intron_variant|H
lincRNA			G|splice_donor_variant&intron_variant|HIGH|LINC01362|ENSG000[illegible]
protein_co	2	0.5	T|stop_gained|HIGH|DNASE2B|ENSG00000137976|transcript|ENST
processed_transcript			CTTTTT|splice_acceptor_variant&intron_variant|HIGH|CLCA2|ENSG
processed_transcript			CTTTTT|splice_acceptor_variant&intron_variant|HIGH|CLCA2|ENSG
processed_transcript			CTTTTT|splice_acceptor_variant&intron_variant|HIGH|CLCA2|ENSG
processed_transcript			CTTTTT|splice_acceptor_variant&intron_variant|HIGH|CLCA2|ENSG
processed_transcript			C|splice_donor_variant&intron_variant|HIGH|CLCA3P|ENSG000001
antisense			C|splice_donor_variant&intron_variant|HIGH|RP4-651E10.4|ENSG0
processed_transcript			T|splice_donor_variant&intron_variant|HIGH|SEP15|ENSG0000018
protein_coding			A|structural_interaction_variant|HIGH|PKN2|ENSG00000065243|in
protein_coding			T|stop_gained|HIGH|RBMXL1|ENSG00000213516|transcript|ENST0
protein_co	3	1	A|frameshift_variant&start_lost|HIGH|RBMXL1|ENSG00000213516
protein_coding			T|structural_interaction_variant|HIGH|GBP1|ENSG00000117228|in
protein_coding			C|structural_interaction_variant|HIGH|GBP1|ENSG00000117228|in
protein_co	1	1	T|frameshift_variant|HIGH|GBP7|ENSG00000213512|transcript|EN
protein_co	1	1	AT|frameshift_variant|HIGH|GBP7|ENSG00000213512|transcript|E
protein_co	5	0.4	G|frameshift_variant|HIGH|GBP5|ENSG00000154451|transcript|EN
protein_co	5	0.4	GAGTC|frameshift_variant&stop_gained|HIGH|GBP5|ENSG0000015
lincRNA			C|splice_acceptor_variant&intron_variant|HIGH|RP11-147C23.1|EN
lincRNA			A|splice_donor_variant&intron_variant|HIGH|RP5-1070A16.1|ENSG
protein_co	5	0.6	CTT|splice_acceptor_variant&intron_variant|HIGH|SNX7|ENSG0000
protein_co	5	0.6	CTT|splice_acceptor_variant&intron_variant|HIGH|SNX7|ENSG0000
protein_co	8	0.13	G|splice_acceptor_variant&intron_variant|HIGH|AGL|ENSG0000016
lincRNA			T|splice_donor_variant&intron_variant|HIGH|LINC01349|ENSG000[illegible]
processed_transcript			A|splice_donor_variant&intron_variant|HIGH|S1PR1|ENSG0000017
protein_co	8	0.38	C|splice_donor_variant&intron_variant|HIGH|AMY2B|ENSG000002[illegible]

processed_transcript			TTCAAA|splice_acceptor_variant&intron_variant|HIGH|RP11-364B6
protein_coding			T|structural_interaction_variant|HIGH|PRMT6|ENSG00000198890|
protein_coding			T|structural_interaction_variant|HIGH|SARS|ENSG00000031698|in
protein_co	12	0.08	A|stop_gained|HIGH|PSRC1|ENSG00000134222|transcript|ENST00
protein_co	3	0.33	A|stop_gained|HIGH|MYBPHL|ENSG00000221986|transcript|ENST
protein_coding			A|structural_interaction_variant|HIGH|GNAI3|ENSG00000065135|i
antisense			CTTTT|splice_acceptor_variant&intron_variant|HIGH|LINC01397|EN
antisense			CTTTT|splice_acceptor_variant&intron_variant|HIGH|LINC01397|EN
antisense			CTTTT|splice_acceptor_variant&intron_variant|HIGH|LINC01397|EN
antisense			T|splice_donor_variant&intron_variant|HIGH|LAMTOR5-AS1|ENSG
processed_transcript			T|splice_donor_variant&intron_variant|HIGH|DRAM2|ENSG000001
protein_co	17	0.06	CTT|splice_acceptor_variant&intron_variant|HIGH|CHI3L2|ENSG000
protein_co	17	0.06	CTT|splice_acceptor_variant&intron_variant|HIGH|CHI3L2|ENSG000
protein_coding			T|structural_interaction_variant|HIGH|CHI3L2|ENSG00000064886|i
protein_coding			A|structural_interaction_variant|HIGH|CHI3L2|ENSG00000064886|
protein_coding			A|structural_interaction_variant|HIGH|CHI3L2|ENSG00000064886|
protein_coding			CCT|frameshift_variant|HIGH|CHIA|ENSG00000134216|transcript|E
protein_coding			G|structural_interaction_variant|HIGH|CHIA|ENSG00000134216|in
protein_co	5	0.2	GT|splice_acceptor_variant&intron_variant|HIGH|DDX20|ENSG0000
protein_co	4	0.25	A|stop_gained&splice_region_variant|HIGH|AMPD1|ENSG0000011
lincRNA			A|splice_acceptor_variant&intron_variant|HIGH|RP4-655J12.4|ENS
protein_co	5	0.6	T|stop_gained|HIGH|TRIM45|ENSG00000134253|transcript|ENST0
unprocessed_pseudogene			AG|splice_donor_variant&intron_variant|HIGH|HSD3BP3|ENSG0000
lincRNA			A|splice_donor_variant&intron_variant|HIGH|RP11-344P13.4|ENSG
protein_co	8	0.38	G|frameshift_variant|HIGH|NBPF15|ENSG00000266338|transcript|
protein_co	5	0.6	T|frameshift_variant&splice_region_variant|HIGH|NBPF20|ENSG000
protein_coding			A|structural_interaction_variant|HIGH|POLR3C|ENSG00000186141
protein_co	5	0.2	C|frameshift_variant|HIGH|NUDT17|ENSG00000186364|transcript|
protein_co	5	0.2	C|frameshift_variant|HIGH|NUDT17|ENSG00000186364|transcript|
protein_co	5	0.2	G|frameshift_variant|HIGH|NUDT17|ENSG00000186364|transcript
protein_co	2	1	A|stop_gained|HIGH|ANKRD35|ENSG00000198483|transcript|ENST
protein_co	2	1	GC|frameshift_variant|HIGH|ANKRD35|ENSG00000198483|transcri
protein_co	7	0.43	C|splice_acceptor_variant&intron_variant|HIGH|NBPF10|ENSG0000
protein_co	8	0.63	T|stop_gained|HIGH|NBPF12|ENSG00000268043|transcript|ENST0
protein_co	10	0.2	A|frameshift_variant|HIGH|CHD1L|ENSG00000131778|transcript|E
protein_coding			G|stop_lost|HIGH|ACP6|ENSG00000162836|transcript|ENST00000
lincRNA			T|splice_acceptor_variant&intron_variant|HIGH|RP11-495P10.1|EN
protein_co	50	0.2	TTTGCCGTTTCTTCC|splice_acceptor_variant&intron_variant|HIGH|P
processed_transcript			TA|splice_acceptor_variant&intron_variant|HIGH|PDE4DIP|ENSG00
protein_co	7	0.14	CTT|splice_acceptor_variant&intron_variant|HIGH|PRPF3|ENSG000
protein_co	7	0.14	CTT|splice_acceptor_variant&intron_variant|HIGH|PRPF3|ENSG000
protein_co	7	0.14	CTT|splice_acceptor_variant&intron_variant|HIGH|PRPF3|ENSG000
protein_co	7	0.14	GGTATACTAATATCTCTGCCTGACAGTTGACA|frameshift_variant&stop
antisense			A|splice_donor_variant&intron_variant|HIGH|ADAMTSL4-AS1|ENSG
nonsense_	12	0.08	A|splice_donor_variant&intron_variant|HIGH|ARNT|ENSG0000014
protein_co	2	0.5	G|start_lost|HIGH|ANXA9|ENSG00000143412|transcript|ENST0000
processed_transcript			A|splice_acceptor_variant&intron_variant|HIGH|PIP5K1A|ENSG000

nonsense_	5	0.8	CTTT|splice_acceptor_variant&intron_variant|HIGH|SNX27|ENSG00
nonsense_	5	0.8	CTTT|splice_acceptor_variant&intron_variant|HIGH|SNX27|ENSG00
nonsense_	5	0.8	CTTT|splice_acceptor_variant&intron_variant|HIGH|SNX27|ENSG00
nonsense_	5	0.8	CTTT|splice_acceptor_variant&intron_variant|HIGH|SNX27|ENSG00
nonsense_	5	0.8	CTTT|splice_acceptor_variant&intron_variant|HIGH|SNX27|ENSG00
antisense			ACCTC|splice_donor_variant&intron_variant|HIGH|RP11-98D18.3|E
)143450			G|bidirectional_gene_fusion|HIGH|OAZ3&TDRKH|ENSG0000014345
antisense			ATTT|splice_acceptor_variant&intron_variant|HIGH|RP11-98D18.9|
antisense			ATTT|splice_acceptor_variant&intron_variant|HIGH|RP11-98D18.9|
antisense			ATTT|splice_acceptor_variant&intron_variant|HIGH|RP11-98D18.9|
protein_coding			C|structural_interaction_variant|HIGH|THEM5|ENSG00000196407|
protein_coding			A|stop_gained|HIGH|HRNR|ENSG00000197915|transcript|ENST000
protein_co	1	1	A|frameshift_variant&start_lost|HIGH|HRNR|ENSG00000197915|tr
protein_co	1	1	C|frameshift_variant|HIGH|FLG|ENSG00000143631|transcript|ENS
protein_coding			A|stop_gained|HIGH|FLG|ENSG00000143631|transcript|ENST0000
antisense			T|splice_acceptor_variant&intron_variant|HIGH|FLG-AS1|ENSG0000
protein_coding			T|stop_gained|HIGH|FLG2|ENSG00000143520|transcript|ENST0000
protein_coding			G|stop_lost|HIGH|LCE1D|ENSG00000172155|transcript|ENST00000
protein_co	1	1	C|splice_donor_variant&intron_variant|HIGH|LCE6A|ENSG0000023
protein_co	2	1	A|frameshift_variant|HIGH|S100A3|ENSG00000188015|transcript|
protein_coding			C|structural_interaction_variant|HIGH|S100A3|ENSG00000188015|
protein_co	12	0.08	C|splice_acceptor_variant&splice_region_variant&intron_variant|HI
protein_co	12	0.08	C|splice_acceptor_variant&splice_region_variant&intron_variant|HI
protein_co	12	0.08	C|splice_acceptor_variant&splice_region_variant&intron_variant|HI
protein_co	13	0.15	GC|frameshift_variant|HIGH|DENND4B|ENSG00000198837|transcr
protein_co	13	0.15	TGC|frameshift_variant|HIGH|DENND4B|ENSG00000198837|transc
protein_coding			C|splice_acceptor_variant&disruptive_inframe_deletion&splice_regi
protein_co	3	0.33	C|splice_acceptor_variant&intron_variant|HIGH|EFNA3|ENSG00000
protein_co	1	1	C|splice_acceptor_variant&intron_variant|HIGH|EFNA3|ENSG00000
protein_co	3	0.33	C|splice_acceptor_variant&intron_variant|HIGH|EFNA3|ENSG00000
protein_co	1	1	C|splice_acceptor_variant&intron_variant|HIGH|EFNA3|ENSG00000
protein_co	29	0.03	T|splice_acceptor_variant&intron_variant|HIGH|MUC1|ENSG00000
antisense			C|splice_acceptor_variant&splice_region_variant&intron_variant|HI
antisense			C|splice_acceptor_variant&splice_region_variant&intron_variant|HI
antisense			C|splice_acceptor_variant&splice_region_variant&intron_variant|HI
protein_co	2	0.5	C|frameshift_variant|HIGH|MEX3A|ENSG00000254726|transcript|E
nonsense_	9	0.11	CTT|splice_acceptor_variant&intron_variant|HIGH|RHBG|ENSG0000
nonsense_	9	0.11	CTT|splice_acceptor_variant&intron_variant|HIGH|RHBG|ENSG0000
nonsense_	9	0.33	ATTT|splice_acceptor_variant&intron_variant|HIGH|RHBG|ENSG000
nonsense_	9	0.33	ATTT|splice_acceptor_variant&intron_variant|HIGH|RHBG|ENSG000
nonsense_	9	0.33	ATTT|splice_acceptor_variant&intron_variant|HIGH|RHBG|ENSG000
nonsense_	9	0.33	ATTT|splice_acceptor_variant&intron_variant|HIGH|RHBG|ENSG000
nonsense_	9	0.11	A|stop_gained|HIGH|RHBG|ENSG00000132677|transcript|ENST000
protein_co	9	0.11	TC|frameshift_variant|HIGH|RHBG|ENSG00000132677|transcript|E
protein_co	6	0.33	G|frameshift_variant|HIGH|TTC24|ENSG00000187862|transcript|E
protein_coding			AAC|frameshift_variant|HIGH|GPATCH4|ENSG00000160818|transc
nonsense_mediated_decay			T|stop_gained&splice_region_variant|HIGH|GPATCH4|ENSG000001

protein_coding			A|structural_interaction_variant|HIGH|NTRK1|ENSG00000198400|i
protein_coding			T|structural_interaction_variant|HIGH|ARHGEF11|ENSG00000132€
protein_coding			TG|frameshift_variant|HIGH|FCRL5|ENSG00000143297|transcript|I
unprocessed_pseudogene			C|splice_acceptor_variant&intron_variant|HIGH|RP4-801G22.2|ENS
protein_co	2	0.5	A|stop_gained|HIGH|CD5L|ENSG00000073754|transcript|ENST000
protein_co	1	1	A|stop_gained|HIGH|CD1A|ENSG00000158477|transcript|ENST000
polymorphic_pseudogene			T|stop_gained|HIGH|OR10X1|ENSG00000279111|transcript|ENST0
protein_co	7	0.57	T|stop_gained|HIGH|PYHIN1|ENSG00000163564|transcript|ENST0(
protein_coding			T|structural_interaction_variant|HIGH|IFI16|ENSG00000163565|int
protein_coding			T|structural_interaction_variant|HIGH|AIM2|ENSG00000163568|in
polymorph	1	1	GT|frameshift_variant|HIGH|OR10J4|ENSG00000249730|transcript
protein_coding			A|stop_gained|HIGH|OR10J1|ENSG00000196184|transcript|ENST0(
protein_coding			G|structural_interaction_variant|HIGH|DUSP23|ENSG00000158716
protein_coding			A|structural_interaction_variant|HIGH|DUSP23|ENSG00000158716
protein_coding			C|stop_lost|HIGH|FCRL6|ENSG00000181036|transcript|ENST00000
protein_coding			T|stop_gained|HIGH|FCRL6|ENSG00000181036|transcript|ENST000
protein_coding			A|frameshift_variant|HIGH|CD48|ENSG00000117091|transcript|EN
protein_coding			T|structural_interaction_variant|HIGH|F11R|ENSG00000158769|int
nonsense_	2	1	A|frameshift_variant&splice_region_variant|HIGH|RP11-544M22.13
protein_co	1	1	C|frameshift_variant&splice_donor_variant&splice_region_variant&
protein_co	1	1	C|frameshift_variant&splice_donor_variant&splice_region_variant&
protein_co	1	1	C|frameshift_variant&splice_donor_variant&splice_region_variant&
protein_co	1	1	C|frameshift_variant&splice_donor_variant&splice_region_variant&
protein_coding			T|structural_interaction_variant|HIGH|NECTIN4|ENSG00000143217
protein_coding			G|structural_interaction_variant|HIGH|NECTIN4|ENSG00000143217
protein_co	14	0.21	T|stop_gained|HIGH|FCGR2A|ENSG00000143226|transcript|ENST0
protein_coding			T|structural_interaction_variant|HIGH|HSPA6|ENSG00000173110|i
polymorph	12	0.17	G|splice_donor_variant&intron_variant|HIGH|FCGR2C|ENSG000002
protein_coding			T|structural_interaction_variant|HIGH|DUSP12|ENSG00000081721
nonsense_	2	0.5	A|splice_donor_variant&intron_variant|HIGH|C1orf111|ENSG00000
protein_coding			G|structural_interaction_variant|HIGH|UAP1|ENSG00000117143|ir
processed_pseudogene			T|splice_acceptor_variant&intron_variant|HIGH|RP11-267N12.2|EN
protein_co	12	0.58	T|splice_donor_variant&intron_variant|HIGH|NUF2|ENSG00000143
protein_co	14	0.14	A|splice_acceptor_variant&intron_variant|HIGH|MGST3|ENSG0000
protein_coding			A|structural_interaction_variant|HIGH|UCK2|ENSG00000143179|in
antisense			ATT|splice_acceptor_variant&intron_variant|HIGH|RP11-375H19.2|
antisense			ATT|splice_acceptor_variant&intron_variant|HIGH|RP11-375H19.2|
processed_transcript			C|splice_donor_variant&intron_variant|HIGH|FMO9P|ENSG000002
processed_transcript			A|splice_donor_variant&intron_variant|HIGH|RCSD1|ENSG0000019
processed_transcript			GCCGAGGCGCCTGGGTGGGA|splice_acceptor_variant&intron_varian
processed_transcript			A|splice_acceptor_variant&intron_variant|HIGH|MPZL1|ENSG00000
protein_coding			G|structural_interaction_variant|HIGH|ADCY10|ENSG00000143199
protein_coding			AG|frameshift_variant|HIGH|GPR161|ENSG00000143147|transcript
protein_co	9	0.33	T|stop_gained|HIGH|C1orf112|ENSG00000000460|transcript|ENST
protein_co	7	0.43	T|frameshift_variant|HIGH|FMO2|ENSG00000094963|transcript|EN
protein_coding			T|stop_gained|HIGH|FMO2|ENSG00000094963|transcript|ENST000
protein_coding			CT|frameshift_variant|HIGH|FMO2|ENSG00000094963|transcript|E

protein_coding			G|structural_interaction_variant|HIGH|MYOC|ENSG00000034971|i
protein_coding			A|structural_interaction_variant|HIGH|MYOC|ENSG00000034971|i
protein_co	5	0.6	A|splice_donor_variant&intron_variant|HIGH|C1orf105|ENSG00000
protein_co	4	0.25	G|splice_donor_variant&splice_region_variant&intron_variant|HIGH
protein_co	6	0.17	T|frameshift_variant|HIGH|KIAA0040|ENSG00000235750|transcript
protein_co	6	0.5	T|frameshift_variant|HIGH|KIAA0040|ENSG00000235750|transcript
protein_co	6	0.5	T|frameshift_variant|HIGH|KIAA0040|ENSG00000235750|transcript
lincRNA			T|splice_donor_variant&intron_variant|HIGH|C1orf220|ENSG00000
protein_co	12	0.17	A|splice_acceptor_variant&intron_variant|HIGH|TOR1AIP1|ENSG00
lincRNA			T|splice_acceptor_variant&intron_variant|HIGH|LINC01344|ENSG0
protein_coding			C|structural_interaction_variant|HIGH|NCF2|ENSG00000116701|in
protein_coding			G|stop_lost|HIGH|ARPC5|ENSG00000162704|transcript|ENST0000
lincRNA			T|splice_donor_variant&intron_variant|HIGH|GS1-279B7.1|ENSG00
protein_co	1	1	T|frameshift_variant|HIGH|AL596220.1|ENSG00000279374|transcr
protein_co	1	1	T|frameshift_variant|HIGH|AL596220.1|ENSG00000279374|transcr
protein_co	1	1	T|frameshift_variant|HIGH|AL596220.1|ENSG00000279374|transcr
protein_co	1	1	TAG|frameshift_variant|HIGH|AL596220.1|ENSG00000279374|tran
protein_co	1	1	TAG|frameshift_variant|HIGH|AL596220.1|ENSG00000279374|tran
protein_co	1	1	TAG|frameshift_variant|HIGH|AL596220.1|ENSG00000279374|tran
protein_co	3	0.33	G|start_lost|HIGH|GLRX2|ENSG00000023572|transcript|ENST0000
protein_co	6	0.33	A|splice_acceptor_variant&intron_variant|HIGH|CFHR3|ENSG00000
protein_co	6	0.17	C|splice_acceptor_variant&intron_variant|HIGH|CFHR3|ENSG00000
protein_coding			G|frameshift_variant|HIGH|CFHR3|ENSG00000116785|transcript|E
protein_co	1	1	CA|frameshift_variant|HIGH|CFHR5|ENSG00000134389|transcript|
protein_co	1	1	CAA|frameshift_variant|HIGH|CFHR5|ENSG00000134389|transcript
processed_transcript			C|splice_acceptor_variant&intron_variant|HIGH|F13B|ENSG000001
protein_coding			A|stop_gained|HIGH|NEK7|ENSG00000151414|transcript|ENST000
lincRNA			CT|splice_acceptor_variant&intron_variant|HIGH|RP11-92G12.3|EN
lincRNA			CTT|splice_acceptor_variant&intron_variant|HIGH|RP11-92G12.3|E
lincRNA			CTTTTTTTTTTTTTTT|splice_acceptor_variant&intron_variant|HIGH|R
lincRNA			CTTTTTTTTTTTTTTTT|splice_acceptor_variant&intron_variant|HIGH|
lincRNA			C|splice_region_variant&intron_variant|LOW|RP11-92G12.3|ENSG0
lincRNA			CT|splice_region_variant&intron_variant|LOW|RP11-92G12.3|ENSG
protein_co	8	0.38	G|frameshift_variant|HIGH|LAD1|ENSG00000159166|transcript|EN
protein_co	8	0.5	A|frameshift_variant|HIGH|LAD1|ENSG00000159166|transcript|EN
protein_co	8	0.5	A|frameshift_variant|HIGH|LAD1|ENSG00000159166|transcript|EN
unitary_pseudogene			G|splice_donor_variant&intron_variant|HIGH|PTPRVP|ENSG000002
protein_coding			T|frameshift_variant|HIGH|LGR6|ENSG00000133067|transcript|EN
protein_coding			C|protein_protein_contact|HIGH|UBE2T|ENSG00000077152|intera
processed_pseudogene			C|splice_acceptor_variant&intron_variant|HIGH|CYCSP4|ENSG0000
processed_pseudogene			C|splice_acceptor_variant&intron_variant|HIGH|CYCSP4|ENSG0000
protein_coding			A|structural_interaction_variant|HIGH|KDM5B|ENSG00000117139|
protein_co	13	0.08	GGC|frameshift_variant|HIGH|PPFIA4|ENSG00000143847|transcrip
)224671			T|bidirectional_gene_fusion|HIGH|ADORA1&RP11-335O13.8|ENSG
protein_coding			G|structural_interaction_variant|HIGH|CHI3L1|ENSG00000133048|
protein_coding			C|structural_interaction_variant|HIGH|CHI3L1|ENSG00000133048|i
protein_co	9	0.33	CAGACCATGGCCCCGCCCAGTCCCT|stop_gained&conservative_infran

lincRNA			G|splice_donor_variant&intron_variant|HIGH|LINC00303|ENSG0000
protein_co	2	0.5	T|splice_donor_variant&intron_variant|HIGH|REN|ENSG000001438
protein_co	2	0.5	C|frameshift_variant&stop_lost|HIGH|KISS1|ENSG00000170498|tra
processed_transcript			A|splice_acceptor_variant&intron_variant|HIGH|TMCC2|ENSG0000
protein_co	12	0.42	T|splice_donor_variant&intron_variant|HIGH|KLHDC8A|ENSG0000
processed_pseudogene			C|splice_donor_variant&intron_variant|HIGH|RP11-6B6.3|ENSG000
processed_pseudogene			C|splice_donor_variant&intron_variant|HIGH|RP11-6B6.3|ENSG000
protein_coding			G|frameshift_variant&splice_region_variant|HIGH|C1orf186|ENSG0
protein_coding			T|structural_interaction_variant|HIGH|MAPKAPK2|ENSG000001628
protein_co	11	0.09	C|splice_donor_variant&intron_variant|HIGH|CD55|ENSG00000196
protein_co	4	0.25	G|frameshift_variant|HIGH|CD34|ENSG00000174059|transcript|EN
protein_co	4	0.25	G|frameshift_variant|HIGH|CD34|ENSG00000174059|transcript|EN
protein_co	8	0.13	CTT|splice_acceptor_variant&intron_variant|HIGH|AL022397.1|ENS
protein_co	1	1	CTT|splice_acceptor_variant&intron_variant|HIGH|AL022397.1|ENS
processed_pseudogene			A|splice_acceptor_variant&intron_variant|HIGH|ST13P19|ENSG000
protein_coding			G|structural_interaction_variant|HIGH|NEK2|ENSG00000117650|in
antisense			T|splice_donor_variant&intron_variant|HIGH|RP11-122M14.1|ENS
antisense			A|splice_donor_variant&intron_variant|HIGH|RP11-122M14.1|ENS
protein_coding			G|structural_interaction_variant|HIGH|TATDN3|ENSG00000203705
protein_co	17	0.12	A|splice_acceptor_variant&intron_variant|HIGH|TATDN3|ENSG0000
protein_co	5	0.2	CTAG|splice_donor_variant&intron_variant|HIGH|KCTD3|ENSG0000
nonsense_	5	0.2	C|splice_acceptor_variant&intron_variant|HIGH|SPATA17|ENSG000
lincRNA			A|splice_donor_variant&intron_variant|HIGH|LINC00210|ENSG0000
protein_coding			G|frameshift_variant|HIGH|RRP15|ENSG00000067533|transcript|E
antisense			C|splice_donor_variant&intron_variant|HIGH|TGFB2-AS1|ENSG0000
protein_coding			T|structural_interaction_variant|HIGH|BPNT1|ENSG00000162813|i
protein_coding			A|structural_interaction_variant|HIGH|BPNT1|ENSG00000162813|i
lincRNA			CT|splice_acceptor_variant&intron_variant|HIGH|RP11-410C4.4|EN
lincRNA			T|splice_donor_variant&intron_variant|HIGH|RP11-378J18.9|ENSG
protein_coding			A|stop_gained|HIGH|TLR5|ENSG00000187554|transcript|ENST0000
protein_coding			G|structural_interaction_variant|HIGH|CAPN2|ENSG00000162909|
protein_coding			T|structural_interaction_variant|HIGH|CAPN2|ENSG00000162909|i
protein_coding			T|structural_interaction_variant|HIGH|NVL|ENSG00000143748|inte
protein_co	18	0.11	TAG|frameshift_variant|HIGH|DNAH14|ENSG00000185842|transcri
protein_co	18	0.11	TAG|frameshift_variant|HIGH|DNAH14|ENSG00000185842|transcri
protein_co	18	0.17	C|frameshift_variant|HIGH|DNAH14|ENSG00000185842|transcript|
protein_co	5	0.4	CTT|splice_acceptor_variant&intron_variant|HIGH|SRP9|ENSG0000
protein_co	5	0.4	CTT|splice_acceptor_variant&intron_variant|HIGH|SRP9|ENSG0000
processed_transcript			A|splice_donor_variant&splice_region_variant&intron_variant|HIGH
processed_transcript			A|splice_donor_variant&splice_region_variant&intron_variant|HIGH
processed_transcript			A|splice_donor_variant&splice_region_variant&intron_variant|HIGH
processed_transcript			A|splice_donor_variant&intron_variant|HIGH|TMEM63A|ENSG0000
processed_transcript			A|splice_donor_variant&intron_variant|HIGH|TMEM63A|ENSG0000
processed_transcript			A|splice_donor_variant&intron_variant|HIGH|TMEM63A|ENSG0000
processed_transcript			A|splice_donor_variant&intron_variant|HIGH|TMEM63A|ENSG0000
processed_transcript			A|splice_donor_variant&intron_variant|HIGH|TMEM63A|ENSG0000
processed_transcript			A|splice_donor_variant&intron_variant|HIGH|TMEM63A|ENSG0000

protein_coding			A|structural_interaction_variant|HIGH|PARP1|ENSG00000143799|i
protein_coding			T|structural_interaction_variant|HIGH|COQ8A|ENSG00000163050|
protein_coding			T|structural_interaction_variant|HIGH|COQ8A|ENSG00000163050|
protein_coding			T|structural_interaction_variant|HIGH|COQ8A|ENSG00000163050|
protein_coding			T|structural_interaction_variant|HIGH|COQ8A|ENSG00000163050|
protein_coding			C|structural_interaction_variant|HIGH|COQ8A|ENSG00000163050|
protein_coding			T|stop_gained|HIGH|C1orf145|ENSG00000162913|transcript|ENST
protein_coding			A|structural_interaction_variant|HIGH|OBSCN|ENSG00000154358|
protein_co	14	0.5	T|stop_gained|HIGH|OBSCN|ENSG00000154358|transcript|ENST00
protein_coding			T|structural_interaction_variant|HIGH|RHOU|ENSG00000116574|in
protein_co	1	1	T|frameshift_variant|HIGH|TMEM78|ENSG00000177800|transcript
protein_coding			T|structural_interaction_variant|HIGH|GALNT2|ENSG00000143641
protein_coding			T|structural_interaction_variant|HIGH|CAPN9|ENSG00000135773|i
protein_coding			C|structural_interaction_variant|HIGH|CAPN9|ENSG00000135773|i
protein_coding			A|structural_interaction_variant|HIGH|EGLN1|ENSG00000135766|i
nonsense_mediated_decay			A|stop_lost|HIGH|ARID4B|ENSG00000054267|transcript|ENST0000
protein_co	4	0.25	G|splice_acceptor_variant&intron_variant|HIGH|EDARADD|ENSG00
protein_coding			A|structural_interaction_variant|HIGH|LGALS8|ENSG00000116977|
protein_coding			T|structural_interaction_variant|HIGH|LGALS8|ENSG00000116977|
antisense			G|splice_acceptor_variant&splice_region_variant&intron_variant&n
protein_co	28	0.21	A|stop_gained|HIGH|LGALS8|ENSG00000116977|transcript|ENST0
protein_coding			C|structural_interaction_variant|HIGH|LGALS8|ENSG00000116977|
protein_coding			T|frameshift_variant|HIGH|ACTN2|ENSG00000077522|transcript|E
protein_co	7	0.14	CA|frameshift_variant|HIGH|FMN2|ENSG00000155816|transcript|
protein_co	7	0.14	C|frameshift_variant|HIGH|FMN2|ENSG00000155816|transcript|E
protein_coding			G|structural_interaction_variant|HIGH|EXO1|ENSG00000174371|in
protein_coding			T|structural_interaction_variant|HIGH|MAP1LC3C|ENSG000001977
unprocessed_pseudogene			G|splice_donor_variant&intron_variant|HIGH|FAM166AP1|ENSG00
protein_coding			C|structural_interaction_variant|HIGH|ADSS|ENSG00000035687|in
protein_co	16	0.06	G|frameshift_variant|HIGH|EFCAB2|ENSG00000203666|transcript|
protein_co	16	0.13	TCCTCC|frameshift_variant|HIGH|EFCAB2|ENSG00000203666|trans
protein_co	16	0.13	CTTATT|splice_acceptor_variant&intron_variant|HIGH|EFCAB2|ENS
protein_co	5	0.4	C|frameshift_variant|HIGH|ZNF669|ENSG00000188295|transcript|
protein_coding			G|frameshift_variant|HIGH|OR2B11|ENSG00000177535|transcript|
protein_co	11	0.64	A|splice_donor_variant&intron_variant|HIGH|GCSAML|ENSG00000
protein_co	1	1	GA|frameshift_variant|HIGH|OR2W3|ENSG00000238243|transcript
polymorphic_pseudogene			A|stop_gained|HIGH|OR2L8|ENSG00000279263|transcript|ENST00
protein_coding			C|frameshift_variant|HIGH|OR2T12|ENSG00000177201|transcript|
protein_coding			TCA|frameshift_variant|HIGH|OR2T35|ENSG00000177151|transcrip
protein_co	1	1	T|frameshift_variant|HIGH|OR2T35|ENSG00000177151|transcript|
protein_co	1	1	TC|frameshift_variant|HIGH|OR2T27|ENSG00000187701|transcript
protein_co	29	0.17	C|start_lost&splice_region_variant|HIGH|SH3YL1|ENSG0000003511
lincRNA			C|splice_donor_variant&splice_region_variant&intron_variant&non_
lincRNA			C|splice_donor_variant&splice_region_variant&intron_variant&non_
lincRNA			A|splice_donor_variant&intron_variant|HIGH|AC079779.5|ENSG000
lincRNA			C|splice_acceptor_variant&splice_region_variant&intron_variant&n
processed_transcript			CTAAA|splice_acceptor_variant&intron_variant|HIGH|PXDN|ENSG0

protein_coding			A|stop_gained|HIGH|TSSC1|ENSG00000032389|transcript|ENST000
processed_transcript			T|splice_donor_variant&intron_variant|HIGH|DCDC2C|ENSG000002
lincRNA			A|splice_donor_variant&intron_variant|HIGH|LINC01105|ENSG0000
lincRNA			T|splice_donor_variant&intron_variant|HIGH|MIR7515HG|ENSG000
lincRNA			A|splice_acceptor_variant&splice_region_variant&intron_variant&n
antisense			T|splice_donor_variant&intron_variant|HIGH|AC019048.1|ENSG000
lincRNA			G|splice_donor_variant&intron_variant|HIGH|AC011747.4|ENSG000
nonsense_	21	0.05	AT|frameshift_variant|HIGH|ITGB1BP1|ENSG00000119185|transcri
lincRNA			G|splice_donor_variant&intron_variant|HIGH|RP11-521D12.2|ENSG
protein_co	11	0.18	TA|frameshift_variant|HIGH|TAF1B|ENSG00000115750|transcript|
lincRNA			G|splice_acceptor_variant&intron_variant|HIGH|AC092687.3|ENSG
protein_coding			C|structural_interaction_variant|HIGH|PDIA6|ENSG00000143870|in
protein_co	8	0.13	C|splice_acceptor_variant&intron_variant|HIGH|PDIA6|ENSG000000
lincRNA			A|splice_acceptor_variant&intron_variant|HIGH|RP11-245G13.2|EN
lincRNA			CTAAA|splice_acceptor_variant&intron_variant|HIGH|AC062028.1|
protein_co	2	1	AC|frameshift_variant&start_lost|HIGH|C2orf50|ENSG0000015087
protein_co	10	0.1	A|splice_acceptor_variant&intron_variant|HIGH|GREB1|ENSG00000
protein_co	9	0.11	G|start_lost|HIGH|NBAS|ENSG00000151779|transcript|ENST00000
protein_co	8	0.13	C|splice_donor_variant&intron_variant|HIGH|DDX1|ENSG0000007
processed_transcript			CTTT|splice_acceptor_variant&intron_variant|HIGH|VSNL1|ENSG00
processed_transcript			CTTT|splice_acceptor_variant&intron_variant|HIGH|VSNL1|ENSG00
processed_transcript			CTTT|splice_acceptor_variant&intron_variant|HIGH|VSNL1|ENSG00
processed_transcript			CTTT|splice_acceptor_variant&intron_variant|HIGH|VSNL1|ENSG00
processed_transcript			CTTT|splice_acceptor_variant&intron_variant|HIGH|VSNL1|ENSG00
processed_transcript			CTTT|splice_acceptor_variant&intron_variant|HIGH|VSNL1|ENSG00
protein_co	8	0.25	C|frameshift_variant|HIGH|GEN1|ENSG00000178295|transcript|EN
protein_co	7	0.29,(NT5C	T|stop_gained|HIGH|NT5C1B|ENSG00000185013|transcript|ENST0
protein_co	8	0.13	C|frameshift_variant|HIGH|HS1BP3|ENSG00000118960|transcript|
protein_coding			T|stop_gained&splice_region_variant|HIGH|HS1BP3|ENSG0000011
protein_co	6	0.33	C|stop_gained|HIGH|TP53I3|ENSG00000115129|transcript|ENST00
protein_co	1	1.00,(FAM2	G|frameshift_variant|HIGH|FAM228B|ENSG00000219626|transcrip
nonsense_mediated_decay			A|stop_gained|HIGH|FAM228B|ENSG00000219626|transcript|ENST
nonsense_mediated_decay			CAA|frameshift_variant|HIGH|FAM228A|ENSG00000186453|transc
processed_pseudogene			C|splice_acceptor_variant&splice_region_variant&intron_variant&n
processed_pseudogene			C|splice_acceptor_variant&splice_region_variant&intron_variant&n
processed_pseudogene			C|splice_acceptor_variant&splice_region_variant&intron_variant&n
processed_pseudogene			C|splice_acceptor_variant&splice_region_variant&intron_variant&n
processed_pseudogene			C|splice_acceptor_variant&splice_region_variant&intron_variant&n
processed_pseudogene			G|splice_donor_variant&intron_variant|HIGH|TRMT112P6|ENSG000
protein_coding			G|structural_interaction_variant|HIGH|CAD|ENSG00000084774|int
protein_coding			C|structural_interaction_variant|HIGH|CAD|ENSG00000084774|int
protein_coding			T|structural_interaction_variant|HIGH|CAD|ENSG00000084774|inte
protein_coding			T|structural_interaction_variant|HIGH|CAD|ENSG00000084774|inte
protein_co	5	0.6	G|splice_acceptor_variant&intron_variant|HIGH|RBKS|ENSG000001
protein_co	11	0.18	C|splice_acceptor_variant&intron_variant|HIGH|BRE|ENSG0000015
antisense			C|splice_donor_variant&intron_variant|HIGH|FLJ31356|ENSG00000
nonsense_	12	0.08	T|splice_donor_variant&intron_variant|HIGH|CLIP4|ENSG0000011

protein_coding			T|stop_gained|HIGH|ALK|ENSG00000171094|transcript|ENST0000(
protein_coding			T|structural_interaction_variant|HIGH|XDH|ENSG00000158125|int
protein_coding			A|structural_interaction_variant|HIGH|XDH|ENSG00000158125|int
protein_coding			C|structural_interaction_variant|HIGH|XDH|ENSG00000158125|int
protein_coding			A|structural_interaction_variant|HIGH|XDH|ENSG00000158125|int
protein_coding			T|structural_interaction_variant|HIGH|XDH|ENSG00000158125|int
lincRNA			G|splice_acceptor_variant&intron_variant|HIGH|LINC01317|ENSG0
nonsense_mediated_decay			G|stop_lost&splice_region_variant|HIGH|FEZ2|ENSG00000171055|
protein_coding			C|structural_interaction_variant|HIGH|EIF2AK2|ENSG00000055332
protein_coding			AT|frameshift_variant|HIGH|PRKD3|ENSG00000115825|transcript|
protein_coding			C|structural_interaction_variant|HIGH|QPCT|ENSG00000115828|in
protein_coding			C|frameshift_variant|HIGH|RMDN2|ENSG00000115841|transcript|
protein_coding			T|structural_interaction_variant|HIGH|CYP1B1|ENSG00000138061|
antisense			T|splice_donor_variant&intron_variant|HIGH|CYP1B1-AS1|ENSG000
protein_coding			T|structural_interaction_variant|HIGH|GALM|ENSG00000143891|ir
protein_co	3	0.33	T|splice_acceptor_variant&intron_variant|HIGH|C2orf91|ENSG0000
protein_coding			C|structural_interaction_variant|HIGH|HAAO|ENSG00000162882|ir
processed_pseudogene			C|splice_acceptor_variant&splice_region_variant&intron_variant|HI
processed_pseudogene			CT|splice_acceptor_variant&splice_region_variant&intron_variant|H
processed_pseudogene			CTTTTT|splice_region_variant&intron_variant|LOW|FTOP1|ENSG00
processed_pseudogene			CTTTTTT|splice_region_variant&intron_variant|LOW|FTOP1|ENSG0
processed_pseudogene			CTTTTTTTTTTTTTT|splice_region_variant&intron_variant|LOW|FTOF
processed_pseudogene			CTTTTTTTTTTTTTTT|splice_region_variant&intron_variant|LOW|FTC
nonsense_	10	0.1	CTT|splice_acceptor_variant&intron_variant|HIGH|DYNC2LI1|ENSG
nonsense_	10	0.1	CTT|splice_acceptor_variant&intron_variant|HIGH|DYNC2LI1|ENSG
protein_coding			G|frameshift_variant|HIGH|SLC3A1|ENSG00000138079|transcript|
processed_transcript			ATTT|splice_acceptor_variant&intron_variant|HIGH|PRKCE|ENSG00
processed_transcript			ATTT|splice_acceptor_variant&intron_variant|HIGH|PRKCE|ENSG00
protein_co	5	0.2	CGG|frameshift_variant|HIGH|TMEM247|ENSG00000187600|trans
protein_co	6	0.33	C|splice_donor_variant&intron_variant|HIGH|EPCAM|ENSG000001
protein_co	6	0.17	CG|frameshift_variant|HIGH|EPCAM|ENSG00000119888|transcript
antisense			ATTT|splice_acceptor_variant&intron_variant|HIGH|AC079807.2|EN
processed_transcript			A|splice_acceptor_variant&intron_variant|HIGH|NRXN1|ENSG0000
lincRNA			A|splice_acceptor_variant&intron_variant|HIGH|AC139712.4|ENSG
protein_co	9	0.22	A|stop_gained|HIGH|ACYP2|ENSG00000170634|transcript|ENST00
protein_co	13	0.31	T|splice_donor_variant&intron_variant|HIGH|CLHC1|ENSG0000016
protein_coding			T|structural_interaction_variant|HIGH|PNPT1|ENSG00000138035|i
protein_coding			T|structural_interaction_variant|HIGH|PNPT1|ENSG00000138035|i
processed_transcript			CTT|splice_acceptor_variant&intron_variant|HIGH|KIAA1841|ENSG
processed_transcript			CTT|splice_acceptor_variant&intron_variant|HIGH|KIAA1841|ENSG
protein_co	11	0.55	ATTT|splice_acceptor_variant&intron_variant|HIGH|KIAA1841|ENS
protein_co	11	0.55	ATTT|splice_acceptor_variant&intron_variant|HIGH|KIAA1841|ENS
protein_co	11	0.55	ATTT|splice_acceptor_variant&intron_variant|HIGH|KIAA1841|ENS
protein_coding			T|frameshift_variant|HIGH|KIAA1841|ENSG00000162929|transcrip
protein_co	8	0.13	T|splice_acceptor_variant&intron_variant|HIGH|COMMD1|ENSG00
protein_co	8	0.13	T|splice_acceptor_variant&intron_variant|HIGH|COMMD1|ENSG00
lincRNA			T|splice_donor_variant&intron_variant|HIGH|LINC00309|ENSG0000

lincRNA			C|splice_acceptor_variant&intron_variant|HIGH|LINC00309|ENSG0(
lincRNA			T|splice_donor_variant&intron_variant|HIGH|AC074391.1|ENSG000
lincRNA			G|splice_donor_variant&intron_variant|HIGH|AC023115.2|ENSG000
antisense			A|splice_donor_variant&intron_variant|HIGH|AC015969.3|ENSG000
polymorphic_pseudogene			G|stop_lost|HIGH|GKN3P|ENSG00000283586|transcript|ENST0000
protein_co	10	0.3	T|start_lost|HIGH|NFU1|ENSG00000169599|transcript|ENST00000
protein_coding			C|structural_interaction_variant|HIGH|CD207|ENSG00000116031|i
protein_coding			T|structural_interaction_variant|HIGH|CD207|ENSG00000116031|i
protein_coding			G|structural_interaction_variant|HIGH|CD207|ENSG00000116031|i
protein_co	8	0.38	C|start_lost|HIGH|ATP6V1B1|ENSG00000116039|transcript|ENST0(
protein_co	8	0.13	G|frameshift_variant|HIGH|ATP6V1B1|ENSG00000116039|transcrip
protein_co	8	0.38	C|splice_acceptor_variant&intron_variant|HIGH|ATP6V1B1|ENSG00
protein_co	8	0.38	C|splice_acceptor_variant&intron_variant|HIGH|ATP6V1B1|ENSG00
nonsense_	2	0.5	T|start_lost|HIGH|AC007040.11|ENSG00000258881|transcript|ENS
protein_coding			C|structural_interaction_variant|HIGH|NAGK|ENSG00000124357|ir
protein_coding			A|structural_interaction_variant|HIGH|NAGK|ENSG00000124357|ir
protein_coding			AAG|frameshift_variant|HIGH|EXOC6B|ENSG00000144036|transcri
polymorphic_pseudogene			G|stop_lost|HIGH|NAT8B|ENSG00000204872|transcript|ENST0000
protein_coding			T|structural_interaction_variant|HIGH|STAMBP|ENSG00000124356
protein_coding			G|structural_interaction_variant|HIGH|HK2|ENSG00000159399|int
processed_transcript			T|splice_acceptor_variant&intron_variant|HIGH|CTNNA2|ENSG000
protein_co	6	0.17	G|stop_lost|HIGH|TGOLN2|ENSG00000152291|transcript|ENST000
protein_co	15	0.53	T|start_lost|HIGH|CAPG|ENSG00000042493|transcript|ENST00000
processed_transcript			A|splice_donor_variant&intron_variant|HIGH|USP39|ENSG0000016
protein_coding			C|structural_interaction_variant|HIGH|EIF2AK3|ENSG00000172071
IG_V_pseudogene			T|splice_acceptor_variant&intron_variant|HIGH|IGKV2-26|ENSG000
IG_V_gene	1	1	AC|frameshift_variant|HIGH|IGKV1-27|ENSG00000244575|transcrip
IG_V_gene			T|stop_gained|HIGH|IGKV3D-11|ENSG00000211632|transcript|ENS
protein_co	8	0.13	A|frameshift_variant|HIGH|ANKRD36C|ENSG00000174501|transcri
protein_coding			G|structural_interaction_variant|HIGH|SNRNP200|ENSG000001440
protein_co	7	0.14	GGGGAA|frameshift_variant|HIGH|FER1L5|ENSG00000249715|trar
protein_coding			G|frameshift_variant|HIGH|ANKRD36|ENSG00000135976|transcrip
nonsense_	58	0.03	G|start_lost|HIGH|LINC01125|ENSG00000228486|transcript|ENST0
protein_coding			A|structural_interaction_variant|HIGH|ZAP70|ENSG00000115085|i
protein_co	21	0.1	T|stop_gained&splice_region_variant|HIGH|VWA3B|ENSG0000016
protein_coding			C|stop_lost|HIGH|COA5|ENSG00000183513|transcript|ENST00000
protein_co	6	0.33	A|start_lost|HIGH|UNC50|ENSG00000115446|transcript|ENST0000
protein_coding			C|structural_interaction_variant|HIGH|REV1|ENSG00000135945|in
protein_co	15	0.2	C|frameshift_variant|HIGH|AFF3|ENSG00000144218|transcript|EN
protein_co	15	0.2	C|frameshift_variant|HIGH|AFF3|ENSG00000144218|transcript|EN
protein_co	1	1	G|start_lost|HIGH|NMS|ENSG00000204640|transcript|ENST000003
lincRNA			A|splice_donor_variant&intron_variant|HIGH|AC092168.4|ENSG000
protein_co	13	0.08	G|splice_donor_variant&intron_variant|HIGH|IL1R1|ENSG0000011
protein_co	3	0.33	T|splice_donor_variant&splice_region_variant&intron_variant|HIGH
protein_co	2	1	A|stop_gained|HIGH|RGPD3|ENSG00000153165|transcript|ENST00
protein_co	1	1	A|stop_gained|HIGH|SULT1C3|ENSG00000196228|transcript|ENST(
protein_coding			A|structural_interaction_variant|HIGH|SULT1C3|ENSG0000019622

protein_coding			C|structural_interaction_variant|HIGH|SULT1C3|ENSG00000196228
protein_co	9	0.11	ATT|splice_acceptor_variant&intron_variant|HIGH|SULT1C2|ENSG0
protein_co	9	0.11	ATT|splice_acceptor_variant&intron_variant|HIGH|SULT1C2|ENSG0
protein_coding			T|structural_interaction_variant|HIGH|LIMS1|ENSG00000169756|ir
protein_co	15	0.67	T|splice_donor_variant&intron_variant|HIGH|LIMS1|ENSG0000016
antisense			TA|splice_donor_variant&intron_variant|HIGH|LIMS1-AS1|ENSG000
protein_co	8	0.38	CT|splice_acceptor_variant&intron_variant|HIGH|CCDC138|ENSG00
protein_co	1	1	G|frameshift_variant|HIGH|AC109344.1|ENSG00000279515|transcr
protein_coding			T|stop_gained|HIGH|ACOXL|ENSG00000153093|transcript|ENST00
nonsense_mediated_decay			C|protein_protein_contact|HIGH|BCL2L11|ENSG00000153094|inter
protein_co	10	0.1	A|stop_gained|HIGH|ANAPC1|ENSG00000153107|transcript|ENST0
processed_pseudogene			C|splice_acceptor_variant&intron_variant|HIGH|AC093166.4|ENSG
processed_pseudogene			C|splice_acceptor_variant&intron_variant|HIGH|AC093166.4|ENSG
processed_pseudogene			C|splice_acceptor_variant&intron_variant|HIGH|AC093166.4|ENSG
processed_pseudogene			C|splice_acceptor_variant&intron_variant|HIGH|AC093166.4|ENSG
protein_co	7	0.14	G|splice_acceptor_variant&intron_variant|HIGH|MERTK|ENSG0000
protein_coding			C|structural_interaction_variant|HIGH|MERTK|ENSG00000153208|
protein_co	7	0.14	C|start_lost|HIGH|RGPD8|ENSG00000169629|transcript|ENST0000
protein_coding			T|frameshift_variant|HIGH|POLR1B|ENSG00000125630|transcript|
protein_co	18	0.33	C|frameshift_variant|HIGH|RABL2A|ENSG00000144134|transcript|
processed_pseudogene			C|splice_acceptor_variant&splice_region_variant&intron_variant|HI
processed_pseudogene			CT|splice_region_variant&intron_variant|LOW|AC092170.1|ENSG00
processed_pseudogene			CTT|splice_region_variant&intron_variant|LOW|AC092170.1|ENSG0
processed_transcript			C|splice_donor_variant&splice_region_variant&intron_variant|HIGH
protein_coding			A|structural_interaction_variant|HIGH|STEAP3|ENSG00000115107|
protein_co	12	0.17	G|stop_gained|HIGH|DBI|ENSG00000155368|transcript|ENST0000
protein_coding			C|stop_lost|HIGH|CFAP221|ENSG00000163075|transcript|ENST000
protein_co	12	0.08	AT|frameshift_variant|HIGH|PTPN4|ENSG00000088179|transcript|
protein_co	12	0.17	AT|splice_acceptor_variant&intron_variant|HIGH|PTPN4|ENSG0000
protein_co	12	0.17	CTT|splice_acceptor_variant&intron_variant|HIGH|PTPN4|ENSG000
protein_co	12	0.17	CTT|splice_acceptor_variant&intron_variant|HIGH|PTPN4|ENSG000
protein_coding			G|structural_interaction_variant|HIGH|CLASP1|ENSG00000074054|
antisense			G|splice_acceptor_variant&intron_variant|HIGH|AC079154.1|ENSG
retained_intron			G|splice_donor_variant&intron_variant|HIGH|AC114783.1|ENSG000
protein_co	10	0.5	T|stop_gained|HIGH|AC114783.1|ENSG00000237524|transcript|EN
lincRNA			C|splice_donor_variant&intron_variant|HIGH|AC068282.3|ENSG000
protein_coding			C|structural_interaction_variant|HIGH|POLR2D|ENSG00000144231
protein_co	3	0.67	C|frameshift_variant|HIGH|POTEI|ENSG00000196834|transcript|EN
protein_co	7	0.14	A|frameshift_variant|HIGH|MZT2A|ENSG00000173272|transcript|E
protein_co	10	0.1	C|splice_donor_variant&intron_variant|HIGH|CCNT2|ENSG0000008
processed_transcript			GTT|splice_acceptor_variant&intron_variant|HIGH|R3HDM1|ENSG0
processed_transcript			GTT|splice_acceptor_variant&intron_variant|HIGH|R3HDM1|ENSG0
protein_coding			G|structural_interaction_variant|HIGH|KYNU|ENSG00000115919|ir
lincRNA			ATT|splice_acceptor_variant&intron_variant|HIGH|TEX41|ENSG000
lincRNA			ATT|splice_acceptor_variant&intron_variant|HIGH|TEX41|ENSG000
processed_transcript			GTT|splice_acceptor_variant&intron_variant|HIGH|MBD5|ENSG000
processed_transcript			GTT|splice_acceptor_variant&intron_variant|HIGH|MBD5|ENSG000

protein_coding			G|structural_interaction_variant|HIGH|TNFAIP6|ENSG0000012361C
nonsense_	7	0.14	C|splice_acceptor_variant&intron_variant|HIGH|ARL5A|ENSG00000
protein_co	4	0.25	CA|frameshift_variant|HIGH|FMNL2|ENSG00000157827|transcript|
processed_pseudogene			A|transcript_ablation|HIGH|AC079150.2|ENSG00000226338|transc
processed_pseudogene			A|transcript_ablation|HIGH|AC079150.2|ENSG00000226338|transc
protein_co	8	0.25	A|splice_donor_variant&intron_variant|HIGH|CYTIP|ENSG0000011
protein_coding			T|structural_interaction_variant|HIGH|ACVR1|ENSG00000115170|i
protein_co	4	0.25	GA|frameshift_variant&splice_region_variant|HIGH|UPP2|ENSG000
protein_coding			G|structural_interaction_variant|HIGH|UPP2|ENSG00000007001|in
antisense			C|splice_acceptor_variant&intron_variant|HIGH|CCDC148-AS1|ENS
protein_co	9	0.11	A|splice_donor_variant&intron_variant|HIGH|MARCH7|ENSG00000
protein_coding			A|structural_interaction_variant|HIGH|DPP4|ENSG00000197635|in
protein_coding			T|structural_interaction_variant|HIGH|DPP4|ENSG00000197635|in
protein_co	11	0.27	C|splice_acceptor_variant&intron_variant|HIGH|DPP4|ENSG000001
protein_co	3	0.33	A|stop_gained|HIGH|IFIH1|ENSG00000115267|transcript|ENST000
protein_co	3	0.33	G|splice_donor_variant&intron_variant|HIGH|IFIH1|ENSG00000115
lincRNA			T|splice_donor_variant&intron_variant|HIGH|AC092684.1|ENSG000
protein_co	25	0.08	C|splice_acceptor_variant&intron_variant|HIGH|COBLL1|ENSG0000
antisense			C|splice_donor_variant&splice_region_variant&intron_variant&non_
antisense			A|splice_acceptor_variant&intron_variant|HIGH|AC013463.2|ENSG
protein_co	17	0.06	A|splice_acceptor_variant&intron_variant|HIGH|SCN2A|ENSG00000
nonsense_mediated_decay			A|frameshift_variant|HIGH|SCN2A|ENSG00000136531|transcript|E
nonsense_mediated_decay			A|frameshift_variant|HIGH|SCN2A|ENSG00000136531|transcript|E
nonsense_	9	0.11	T|splice_donor_variant&intron_variant|HIGH|GALNT3|ENSG000001
protein_co	7	0.57	C|splice_acceptor_variant&intron_variant|HIGH|SCN7A|ENSG00000
protein_co	7	0.29	T|frameshift_variant|HIGH|XIRP2|ENSG00000163092|transcript|EN
protein_co	7	0.14	C|splice_donor_variant&intron_variant|HIGH|XIRP2|ENSG0000016
protein_co	8	0.88	C|splice_acceptor_variant&intron_variant|HIGH|DHRS9|ENSG00000
protein_co	10	0.2	GT|frameshift_variant|HIGH|FASTKD1|ENSG00000138399|transcrip
protein_co	11	0.27	A|splice_donor_variant&intron_variant|HIGH|PPIG|ENSG0000013
processed_pseudogene			T|splice_donor_variant&intron_variant|HIGH|PTCHD3P2|ENSG0000
protein_co	7	0.14	CA|frameshift_variant|HIGH|KLHL23|ENSG00000213160|transcript
processed_transcript			C|splice_donor_variant&intron_variant|HIGH|SP5|ENSG000002043
protein_co	12	0.25	CT|splice_acceptor_variant&intron_variant|HIGH|DCAF17|ENSG000
protein_co	12	0.25	CT|splice_acceptor_variant&intron_variant|HIGH|DCAF17|ENSG000
processed_transcript			A|splice_donor_variant&intron_variant|HIGH|SCRN3|ENSG0000014
protein_coding			T|frameshift_variant|HIGH|SCRN3|ENSG00000144306|transcript|E
sense_overlapping			T|splice_donor_variant&intron_variant|HIGH|AC074286.1|ENSG000
antisense			CTT|splice_acceptor_variant&intron_variant|HIGH|TTN-AS1|ENSG0
antisense			CTT|splice_acceptor_variant&intron_variant|HIGH|TTN-AS1|ENSG0
lincRNA			T|splice_donor_variant&intron_variant|HIGH|AC068196.1|ENSG000
processed_transcript			CTT|splice_acceptor_variant&intron_variant|HIGH|PPP1R1C|ENSG0
processed_transcript			CTT|splice_acceptor_variant&intron_variant|HIGH|PPP1R1C|ENSG0
antisense			G|splice_acceptor_variant&intron_variant|HIGH|AC064871.3|ENSG
retained_intron			A|splice_donor_variant&intron_variant|HIGH|NUP35|ENSG0000016
nonsense_mediated_decay			T|stop_gained|HIGH|NUP35|ENSG00000163002|transcript|ENST00
nonsense_	10	0.1	A|splice_donor_variant&intron_variant|HIGH|NUP35|ENSG0000016

protein_coding			AG|frameshift_variant|HIGH|FSIP2|ENSG00000188738|transcript|E
protein_co	1	1	CT|frameshift_variant|HIGH|AC018867.1|ENSG00000281192|trans
protein_co	21	0.05	TA|frameshift_variant|HIGH|PMS1|ENSG00000064933|transcript|E
protein_coding			C|structural_interaction_variant|HIGH|HIBCH|ENSG00000198130|i
protein_co	14	0.14	G|start_lost|HIGH|HIBCH|ENSG00000198130|transcript|ENST0000
lincRNA			C|splice_donor_variant&intron_variant|HIGH|AC064834.2|ENSG000
protein_coding			C|structural_interaction_variant|HIGH|STK17B|ENSG00000081320|
protein_co	19	0.05	C|frameshift_variant|HIGH|CCDC150|ENSG00000144395|transcript
protein_coding			A|frameshift_variant|HIGH|ANKRD44|ENSG00000065413|transcrip
protein_coding			T|frameshift_variant&stop_lost|HIGH|FTCDNL1|ENSG00000226124
protein_coding			T|frameshift_variant&stop_lost|HIGH|FTCDNL1|ENSG00000226124
unitary_pseudogene			A|splice_donor_variant&intron_variant|HIGH|AOX3P|ENSG0000024
processed_transcript			A|splice_donor_variant&intron_variant|HIGH|AOX2P|ENSG0000024
protein_co	15	0.07	A|start_lost|HIGH|CLK1|ENSG00000013441|transcript|ENST000004
protein_co	25	0.12	A|splice_acceptor_variant&intron_variant|HIGH|CFLAR|ENSG00000
protein_co	20	0.1	C|start_lost|HIGH|CASP8|ENSG00000064012|transcript|ENST0000
protein_coding			C|structural_interaction_variant|HIGH|CASP8|ENSG00000064012|i
protein_coding			A|structural_interaction_variant|HIGH|CASP8|ENSG00000064012|i
protein_coding			C|stop_gained|HIGH|ALS2CR12|ENSG00000155749|transcript|ENS
processed_transcript			C|splice_donor_variant&intron_variant|HIGH|ALS2|ENSG00000003
protein_co	4	0.25	T|stop_gained|HIGH|KIAA2012|ENSG00000283310|transcript|ENST
nonsense_mediated_decay			AT|frameshift_variant|HIGH|ICA1L|ENSG00000163596|transcript|E
nonsense_mediated_decay			AT|frameshift_variant|HIGH|ICA1L|ENSG00000163596|transcript|E
nonsense_mediated_decay			AT|frameshift_variant|HIGH|ICA1L|ENSG00000163596|transcript|E
protein_coding			CA|frameshift_variant|HIGH|CARF|ENSG00000138380|transcript|E
protein_co	10	0.1	T|stop_gained|HIGH|NBEAL1|ENSG00000144426|transcript|ENST0
protein_coding			C|structural_interaction_variant|HIGH|RAPH1|ENSG00000173166|i
lincRNA			A|splice_donor_variant&intron_variant|HIGH|AC009965.2|ENSG000
protein_coding			T|frameshift_variant|HIGH|NRP2|ENSG00000118257|transcript|EN
protein_coding			TA|frameshift_variant&stop_gained|HIGH|NRP2|ENSG0000011825
lincRNA			T|splice_donor_variant&intron_variant|HIGH|AC007879.2|ENSG000
protein_coding			C|structural_interaction_variant|HIGH|IDH1|ENSG00000138413|int
protein_coding			T|structural_interaction_variant|HIGH|IDH1|ENSG00000138413|int
protein_coding			A|structural_interaction_variant|HIGH|IDH1|ENSG00000138413|int
protein_co	4	0.25	A|stop_gained|HIGH|PTH2R|ENSG00000144407|transcript|ENST00
protein_co	14	0.07	AC|frameshift_variant|HIGH|IKZF2|ENSG00000030419|transcript|E
protein_coding			T|structural_interaction_variant|HIGH|BARD1|ENSG00000138376|i
protein_coding			G|structural_interaction_variant|HIGH|BARD1|ENSG00000138376|
protein_co	3	0.33	GA|frameshift_variant|HIGH|ABCA12|ENSG00000144452|transcrip
processed_transcript			A|splice_donor_variant&intron_variant|HIGH|ATIC|ENSG0000013
lincRNA			C|splice_acceptor_variant&intron_variant|HIGH|AC012668.2|ENSG
processed_transcript			T|splice_acceptor_variant&intron_variant|HIGH|PECR|ENSG000001
protein_co	4	0.25	A|frameshift_variant|HIGH|DIRC3|ENSG00000231672|transcript|E
protein_co	15	0.07	C|splice_donor_variant&intron_variant|HIGH|CTDSP1|ENSG000001
nonsense_mediated_decay			C|stop_lost|HIGH|CNOT9|ENSG00000144580|transcript|ENST0000
protein_co	18	0.06	C|splice_donor_variant&intron_variant|HIGH|DNAJB2|ENSG000001
protein_co	25	0.04	C|frameshift_variant|HIGH|SPEG|ENSG00000072195|transcript|EN

protein_co	25	0.04	C|frameshift_variant|HIGH|SPEG|ENSG00000072195|transcript|EN
protein_co	25	0.08	TA|frameshift_variant|HIGH|SPEG|ENSG00000072195|transcript|E
protein_co	1	1	A|frameshift_variant|HIGH|MOGAT1|ENSG00000124003|transcript
processed_transcript			C|splice_donor_variant&intron_variant|HIGH|ACSL3|ENSG0000012
protein_co	8	0.13	CAA|frameshift_variant|HIGH|AP1S3|ENSG00000152056|transcript
protein_co	1	1	ACATTG|frameshift_variant|HIGH|RP11-811O14.1|ENSG000002834
protein_coding			A|structural_interaction_variant|HIGH|IRS1|ENSG00000169047|int
antisense			C|splice_acceptor_variant&intron_variant|HIGH|AC097662.2|ENSG
antisense			A|splice_acceptor_variant&intron_variant|HIGH|AC097662.2|ENSG
protein_co	27	0.41	TCTGGAAAAGCA|splice_donor_variant&intron_variant|HIGH|MFF|E
protein_co	27	0.22	GGTGAGCAGGGCAGCATT|splice_donor_variant&intron_variant|HIG
protein_co	27	0.22	GGTGAGCAGGGCAGCATT|splice_donor_variant&intron_variant|HIG
protein_co	27	0.33	GAAA|splice_donor_variant&intron_variant|HIGH|MFF|ENSG00000
protein_coding			T|stop_gained|HIGH|C2orf83|ENSG00000042304|transcript|ENST0
unprocessed_pseudogene			CTAAAA|splice_acceptor_variant&intron_variant|HIGH|AC064853.3
unprocessed_pseudogene			CTAAAATAAAA|splice_acceptor_variant&intron_variant|HIGH|AC06
protein_co	4	0.25	CTT|splice_acceptor_variant&intron_variant|HIGH|CCL20|ENSG000
protein_co	4	0.25	CTT|splice_acceptor_variant&intron_variant|HIGH|CCL20|ENSG000
antisense			A|splice_acceptor_variant&intron_variant|HIGH|AC009950.2|ENSG
protein_co	10	0.1	C|start_lost|HIGH|SP140L|ENSG00000185404|transcript|ENST0000
protein_co	10	0.1	T|frameshift_variant|HIGH|SPATA3|ENSG00000173699|transcript|
nonsense_mediated_decay			A|stop_gained|HIGH|SPATA3|ENSG00000173699|transcript|ENST0
lincRNA			T|splice_donor_variant&intron_variant|HIGH|LINC00471|ENSG0000
protein_co	5	0.2	A|splice_donor_variant&intron_variant|HIGH|PDE6D|ENSG0000015
processed_transcript			A|splice_donor_variant&intron_variant|HIGH|DIS3L2|ENSG0000014
protein_co	16	0.06	G|splice_acceptor_variant&intron_variant|HIGH|DIS3L2|ENSG0000
processed_transcript			G|splice_acceptor_variant&intron_variant|HIGH|EIF4E2|ENSG0000
protein_co	46	0.13	CCG|frameshift_variant|HIGH|GIGYF2|ENSG00000204120|transcrip
protein_co	46	0.13	CCG|frameshift_variant|HIGH|GIGYF2|ENSG00000204120|transcrip
protein_co	46	0.13	AGCAG|frameshift_variant|HIGH|GIGYF2|ENSG00000204120|trans
protein_co	46	0.13	AGCAG|frameshift_variant|HIGH|GIGYF2|ENSG00000204120|trans
protein_coding			A|structural_interaction_variant|HIGH|NEU2|ENSG00000115488|in
protein_coding			T|structural_interaction_variant|HIGH|NEU2|ENSG00000115488|in
nonsense_mediated_decay			TTG|frameshift_variant|HIGH|INPP5D|ENSG00000168918|transcrip
nonsense_	14	0.07	C|splice_acceptor_variant&intron_variant|HIGH|INPP5D|ENSG0000
protein_co	14	0.07	C|frameshift_variant&splice_donor_variant&splice_region_variant&
protein_co	8	0.13	A|splice_donor_variant&intron_variant|HIGH|MROH2A|ENSG00000
protein_co	14	0.14	CCAGG|frameshift_variant|HIGH|AGAP1|ENSG00000157985|transc
nonsense_	7	0.14	A|splice_acceptor_variant&intron_variant|HIGH|COPS8|ENSG00000
nonsense_	2	0.5	CTTT|splice_acceptor_variant&intron_variant|HIGH|UBE2F-SCLY|EN
nonsense_	2	0.5	CTTT|splice_acceptor_variant&intron_variant|HIGH|UBE2F-SCLY|EN
nonsense_	2	0.5	CTTT|splice_acceptor_variant&intron_variant|HIGH|UBE2F-SCLY|EN
antisense			G|splice_donor_variant&intron_variant|HIGH|RP11-5O24.1|ENSG0
lincRNA			C|splice_acceptor_variant&intron_variant|HIGH|AC144525.1|ENSG
protein_co	1	1	G|frameshift_variant|HIGH|PRR21|ENSG00000221961|transcript|E
protein_coding			GGCCGTGGATGAAGA|frameshift_variant|HIGH|PRR21|ENSG000002
protein_coding			A|structural_interaction_variant|HIGH|GPC1|ENSG00000063660|in

protein_co	24	0.33	A|splice_acceptor_variant&intron_variant|HIGH|ANKMY1|ENSG000
protein_co	6	0.33	G|frameshift_variant|HIGH|AQP12B|ENSG00000185176|transcript|
protein_co	5	0.2	C|frameshift_variant&splice_region_variant|HIGH|AQP12A|ENSG00
protein_coding			A|structural_interaction_variant|HIGH|AGXT|ENSG00000172482|in
protein_co	3	0.33	C|splice_donor_variant&intron_variant|HIGH|CROCC2|ENSG000002
protein_co	22	0.05	G|start_lost|HIGH|MTERF4|ENSG00000122085|transcript|ENST000
protein_coding			C|structural_interaction_variant|HIGH|PASK|ENSG00000115687|int
protein_coding			T|structural_interaction_variant|HIGH|PASK|ENSG00000115687|int
protein_co	16	0.31	T|frameshift_variant|HIGH|PASK|ENSG00000115687|transcript|EN
protein_co	16	0.06	G|start_lost|HIGH|PASK|ENSG00000115687|transcript|ENST00000
processed_transcript			GTTTTTTTTTT|splice_acceptor_variant&intron_variant|HIGH|FARP2
processed_transcript			GTTTTTTTTTT|splice_acceptor_variant&intron_variant|HIGH|FARP2
protein_co	16	0.06	G|frameshift_variant|HIGH|D2HGDH|ENSG00000180902|transcript
nonsense_	16	0.06	CG|frameshift_variant|HIGH|CHL1|ENSG00000134121|transcript|E
nonsense_	16	0.06	CG|frameshift_variant|HIGH|CHL1|ENSG00000134121|transcript|E
nonsense_	16	0.06	C|frameshift_variant|HIGH|CHL1|ENSG00000134121|transcript|EN
nonsense_	16	0.06	C|frameshift_variant|HIGH|CHL1|ENSG00000134121|transcript|EN
nonsense_	16	0.06	C|frameshift_variant|HIGH|CHL1|ENSG00000134121|transcript|EN
nonsense_	16	0.06	C|frameshift_variant|HIGH|CHL1|ENSG00000134121|transcript|EN
nonsense_mediated_decay			A|stop_gained|HIGH|CNTN6|ENSG00000134115|transcript|ENST00
antisense			T|splice_acceptor_variant&intron_variant|HIGH|CNTN4-AS1|ENSG0
protein_co	1	1	C|splice_acceptor_variant&splice_region_variant&intron_variant|HI
protein_co	1	1	C|splice_acceptor_variant&splice_region_variant&intron_variant|HI
protein_co	1	1	C|splice_acceptor_variant&splice_region_variant&intron_variant|HI
protein_co	1	1	C|splice_acceptor_variant&splice_region_variant&intron_variant|HI
lincRNA			C|splice_donor_variant&intron_variant|HIGH|AC027119.1|ENSG000
nonsense_mediated_decay			C|structural_interaction_variant|HIGH|GRM7|ENSG00000196277|in
nonsense_mediated_decay			A|frameshift_variant|HIGH|GRM7|ENSG00000196277|transcript|EN
antisense			T|splice_acceptor_variant&intron_variant|HIGH|LMCD1-AS1|ENSG0
protein_co	13	0.08	G|splice_donor_variant&intron_variant|HIGH|SRGAP3|ENSG000001
processed_pseudogene			AAT|splice_acceptor_variant&splice_region_variant&intron_variant
processed_pseudogene			AAT|splice_acceptor_variant&splice_region_variant&intron_variant
processed_pseudogene			AAT|splice_acceptor_variant&splice_region_variant&intron_variant
processed_pseudogene			AAT|splice_acceptor_variant&splice_region_variant&intron_variant
processed_pseudogene			AAT|splice_acceptor_variant&splice_region_variant&intron_variant
protein_coding			C|frameshift_variant|HIGH|CPNE9|ENSG00000144550|transcript|E
retained_intron			T|splice_donor_variant&intron_variant|HIGH|TTLL3|ENSG0000214
protein_co	33	0.06	T|stop_gained|HIGH|TTLL3|ENSG00000214021|transcript|ENST000
protein_coding			C|frameshift_variant|HIGH|TAMM41|ENSG00000144559|transcript
nonsense_	12	0.08	C|splice_donor_variant&intron_variant|HIGH|TAMM41|ENSG00000
protein_coding			T|structural_interaction_variant|HIGH|PPARG|ENSG00000132170|i
protein_co	7	0.14	C|frameshift_variant|HIGH|GRIP2|ENSG00000144596|transcript|EN
antisense			TC|splice_acceptor_variant&intron_variant|HIGH|SH3BP5-AS1|ENS
protein_coding			A|stop_gained|HIGH|GALNT15|ENSG00000131386|transcript|ENST
protein_co	18	0.11	C|splice_acceptor_variant&intron_variant|HIGH|SATB1|ENSG00000
lincRNA			T|splice_donor_variant&intron_variant|HIGH|SGO1-AS1|ENSG0000
processed_transcript			T|splice_donor_variant&intron_variant|HIGH|ZNF385D|ENSG00000

processed_pseudogene			G|splice_acceptor_variant&intron_variant|HIGH|RANP7|ENSG0000(
protein_co	13	0.15	G|splice_acceptor_variant&intron_variant|HIGH|NEK10|ENSG00000
antisense			T|splice_acceptor_variant&intron_variant|HIGH|RBMS3-AS2|ENSG(
protein_coding			T|stop_gained|HIGH|ZNF860|ENSG00000197385|transcript|ENST0(
protein_co	19	0.16	GT|splice_acceptor_variant&intron_variant|HIGH|CNOT10|ENSG00(
protein_co	19	0.16	GT|splice_acceptor_variant&intron_variant|HIGH|CNOT10|ENSG00(
protein_coding			A|structural_interaction_variant|HIGH|PDCD6IP|ENSG0000017024ε
protein_coding			C|frameshift_variant|HIGH|CTDSPL|ENSG00000144677|transcript|I
protein_co	4	0.75	A|frameshift_variant|HIGH|SLC22A14|ENSG00000144671|transcrip
protein_coding			C|structural_interaction_variant|HIGH|XYLB|ENSG00000093217|int
protein_coding			C|structural_interaction_variant|HIGH|XYLB|ENSG00000093217|int
protein_coding			T|structural_interaction_variant|HIGH|XYLB|ENSG00000093217|int
protein_coding			A|structural_interaction_variant|HIGH|XYLB|ENSG00000093217|int
processed_pseudogene			CAA|splice_acceptor_variant&intron_variant|HIGH|GEMIN2P2|ENS(
processed_pseudogene			C|splice_region_variant&intron_variant|LOW|GEMIN2P2|ENSG0000(
antisense			G|splice_acceptor_variant&intron_variant|HIGH|VIPR1-AS1|ENSG0(
protein_coding			A|structural_interaction_variant|HIGH|NKTR|ENSG00000114857|in
protein_co	3	0.33	T|frameshift_variant|HIGH|ZNF852|ENSG00000178917|transcript|I
protein_co	6	0.17	A|stop_gained|HIGH|TGM4|ENSG00000163810|transcript|ENST00(
protein_co	4	0.5	C|splice_acceptor_variant&intron_variant|HIGH|LIMD1|ENSG00000
protein_co	2	1	T|frameshift_variant|HIGH|CCR5|ENSG00000160791|transcript|EN
protein_coding			T|stop_gained|HIGH|AC104304.4|ENSG00000283473|transcript|EN
protein_co	3	0.33	C|splice_donor_variant&intron_variant|HIGH|PRSS45|ENSG000001
protein_co	2	0.5	G|start_lost|HIGH|PRSS42|ENSG00000178055|transcript|ENST000(
protein_coding			C|splice_acceptor_variant&disruptive_inframe_deletion&splice_regi
protein_co	6	0.17	C|frameshift_variant|HIGH|PTPN23|ENSG00000076201|transcript|
protein_co	20	0.05	A|frameshift_variant&splice_acceptor_variant&splice_region_varian
protein_coding			A|structural_interaction_variant|HIGH|USP4|ENSG00000114316|in
protein_coding			C|structural_interaction_variant|HIGH|USP4|ENSG00000114316|in
protein_co	14	0.07	C|frameshift_variant|HIGH|USP4|ENSG00000114316|transcript|EN
protein_co	21	0.1	A|stop_gained|HIGH|MST1|ENSG00000173531|transcript|ENST000
protein_co	21	0.05	G|splice_acceptor_variant&intron_variant|HIGH|MST1|ENSG00000
nonsense_mediated_decay			C|frameshift_variant|HIGH|RBM6|ENSG00000004534|transcript|EN
nonsense_mediated_decay			C|frameshift_variant|HIGH|RBM6|ENSG00000004534|transcript|EN
nonsense_mediated_decay			C|frameshift_variant|HIGH|RBM6|ENSG00000004534|transcript|EN
nonsense_mediated_decay			C|frameshift_variant|HIGH|RBM6|ENSG00000004534|transcript|EN
nonsense_mediated_decay			C|frameshift_variant|HIGH|RBM6|ENSG00000004534|transcript|EN
nonsense_mediated_decay			C|frameshift_variant|HIGH|RBM6|ENSG00000004534|transcript|EN
protein_co	20	0.05	C|splice_acceptor_variant&intron_variant|HIGH|RBM6|ENSG00000
protein_co	20	0.05	C|splice_acceptor_variant&intron_variant|HIGH|RBM6|ENSG00000
protein_co	9	0.22	C|splice_acceptor_variant&intron_variant|HIGH|SLC38A3|ENSG000
protein_co	9	0.22	C|splice_acceptor_variant&intron_variant|HIGH|SLC38A3|ENSG000
nonsense_	12	0.08	T|stop_gained|HIGH|GNAI2|ENSG00000114353|transcript|ENST00(
protein_co	4	0.25	C|start_lost&conservative_inframe_deletion|HIGH|MANF|ENSG000
protein_co	4	0.25	G|start_lost&disruptive_inframe_deletion|HIGH|MANF|ENSG00000
protein_co	4	0.25	G|start_lost&disruptive_inframe_deletion|HIGH|MANF|ENSG00000
protein_co	4	0.25	G|start_lost&disruptive_inframe_deletion|HIGH|MANF|ENSG00000

protein_co	4	0.25	G|start_lost|HIGH|MANF|ENSG00000145050|transcript|ENST0000(
protein_co	4	0.25	G|start_lost|HIGH|MANF|ENSG00000145050|transcript|ENST0000(
protein_coding			A|structural_interaction_variant|HIGH|TWF2|ENSG00000247596|ir
nonsense_	9	0.11	G|splice_acceptor_variant&intron_variant|HIGH|GLYCTK|ENSG0000(
protein_coding			C|frameshift_variant|HIGH|DNAH1|ENSG00000114841|transcript|E
protein_coding			C|frameshift_variant|HIGH|DNAH1|ENSG00000114841|transcript|E
protein_co	11	0.55	CCA|splice_acceptor_variant&intron_variant|HIGH|PHF7|ENSG0000(
protein_co	9	0.11	GC|splice_acceptor_variant&intron_variant|HIGH|STAB1|ENSG0000(
protein_coding			C|frameshift_variant|HIGH|ITIH1|ENSG00000055957|transcript|EN
protein_coding			C|structural_interaction_variant|HIGH|PRKCD|ENSG00000163932|i
protein_coding			A|structural_interaction_variant|HIGH|TKT|ENSG00000163931|inte
protein_coding			C|structural_interaction_variant|HIGH|TKT|ENSG00000163931|inte
protein_co	14	0.07	A|splice_donor_variant&intron_variant|HIGH|TKT|ENSG000001639
protein_coding			T|stop_gained|HIGH|IL17RB|ENSG00000056736|transcript|ENST00
protein_coding			A|structural_interaction_variant|HIGH|ACTR8|ENSG00000113812|i
protein_coding			A|structural_interaction_variant|HIGH|ACTR8|ENSG00000113812|i
protein_coding			C|structural_interaction_variant|HIGH|APPL1|ENSG00000157500|ir
antisense			ATTT|splice_acceptor_variant&intron_variant|HIGH|FAM3D-AS1|EN
antisense			ATTT|splice_acceptor_variant&intron_variant|HIGH|FAM3D-AS1|EN
antisense			ATTT|splice_acceptor_variant&intron_variant|HIGH|FAM3D-AS1|EN
protein_coding			T|structural_interaction_variant|HIGH|PTPRG|ENSG00000144724|i
protein_coding			C|structural_interaction_variant|HIGH|PTPRG|ENSG00000144724|i
antisense			G|splice_donor_variant&intron_variant|HIGH|SYNPR-AS1|ENSG000
protein_co	5	0.4	CTT|splice_acceptor_variant&intron_variant|HIGH|KBTBD8|ENSG00
protein_co	5	0.4	CTT|splice_acceptor_variant&intron_variant|HIGH|KBTBD8|ENSG00
lincRNA			TTTTATTTATTTA|splice_acceptor_variant&intron_variant|HIGH|SUC
lincRNA			TTTTATTTATTTATTTA|splice_acceptor_variant&intron_variant|HIGH
lincRNA			TTTTATTTATTTATTTATTTA|splice_acceptor_variant&intron_variant|
lincRNA			TTTTATTTATTTATTTATTTATTTA|splice_acceptor_variant&intron_var
processed_transcript			GT|splice_acceptor_variant&intron_variant|HIGH|FAM19A1|ENSG0
)114861			A|bidirectional_gene_fusion|HIGH|FOXP1&RP11-154H23.4|ENSG00
protein_co	1	1	CA|frameshift_variant|HIGH|EBLN2|ENSG00000255423|transcript|
unprocessed_pseudogene			T|splice_donor_variant&intron_variant|HIGH|MYLKP1|ENSG000002
transcribed_unprocessed_pseudo			A|splice_donor_variant&intron_variant|HIGH|FAM86DP|ENSG0000
unprocessed_pseudogene			G|splice_donor_variant&intron_variant|HIGH|ENPP7P2|ENSG0000(
protein_coding			A|stop_gained|HIGH|ZNF717|ENSG00000227124|transcript|ENST0
protein_co	5	0.2	T|splice_donor_variant&intron_variant|HIGH|ZNF717|ENSG000002
protein_coding			G|structural_interaction_variant|HIGH|ROBO1|ENSG00000169855|
protein_coding			C|structural_interaction_variant|HIGH|GBE1|ENSG00000114480|in
protein_coding			C|structural_interaction_variant|HIGH|GBE1|ENSG00000114480|in
lincRNA			C|splice_donor_variant&intron_variant|HIGH|RP11-260O18.1|ENSG
lincRNA			G|splice_donor_variant&intron_variant|HIGH|LINC00971|ENSG0000(
protein_co	6	0.17	T|frameshift_variant|HIGH|RP11-249L12.1|ENSG00000283434|tran
protein_coding			A|stop_gained|HIGH|RP11-249L12.1|ENSG00000283434|transcript
protein_coding			A|structural_interaction_variant|HIGH|EPHA3|ENSG00000044524|i
protein_coding			C|structural_interaction_variant|HIGH|MINA|ENSG00000170854|ir
protein_coding			G|structural_interaction_variant|HIGH|MINA|ENSG00000170854|ir

protein_co	3	0.67	T|stop_gained&splice_region_variant|HIGH|GABRR3|ENSG0000018
polymorph	1	1	A|frameshift_variant|HIGH|OR5AC1|ENSG00000213439|transcript|
protein_coding			C|stop_lost&splice_region_variant|HIGH|OR5AC2|ENSG00000196
protein_co	2	1	A|frameshift_variant|HIGH|OR5H15|ENSG00000233412|transcript|
polymorphic_pseudogene			A|stop_gained|HIGH|OR5H8|ENSG00000232535|transcript|ENST00
protein_co	1	1	T|frameshift_variant|HIGH|OR5K4|ENSG00000196098|transcript|E
protein_co	1	1	GA|frameshift_variant|HIGH|OR5K3|ENSG00000206536|transcript|
protein_coding			A|stop_gained|HIGH|OR5K2|ENSG00000231861|transcript|ENST00
protein_coding			C|structural_interaction_variant|HIGH|CPOX|ENSG00000080819|in
antisense			T|splice_donor_variant&intron_variant|HIGH|ST3GAL6-AS1|ENSG00
nonsense_	7	0.14	CG|frameshift_variant|HIGH|NIT2|ENSG00000114021|transcript|E
protein_co	29	0.17	C|start_lost|HIGH|ABI3BP|ENSG00000154175|transcript|ENST0000
protein_coding			A|structural_interaction_variant|HIGH|CBLB|ENSG00000114423|in
lincRNA			CTTT|splice_acceptor_variant&intron_variant|HIGH|DUBR|ENSG000
lincRNA			CTTT|splice_acceptor_variant&intron_variant|HIGH|DUBR|ENSG000
lincRNA			CTTT|splice_acceptor_variant&intron_variant|HIGH|DUBR|ENSG000
lincRNA			C|splice_acceptor_variant&intron_variant|HIGH|RP11-446H18.5|EN
protein_co	6	0.17	C|splice_donor_variant&intron_variant|HIGH|DZIP3|ENSG0000019
protein_co	2	0.5	GGGGGATTA|frameshift_variant&stop_gained|HIGH|RETNLB|ENSG
protein_co	3	0.33	A|splice_donor_variant&intron_variant|HIGH|GUCA1C|ENSG00000
protein_co	3	1	A|stop_gained|HIGH|GUCA1C|ENSG00000138472|transcript|ENST0
protein_co	8	0.25	G|start_lost|HIGH|DPPA4|ENSG00000121570|transcript|ENST0000
protein_co	7	0.14	GT|splice_acceptor_variant&intron_variant|HIGH|C3orf52|ENSG000
protein_coding			CA|frameshift_variant|HIGH|ATG3|ENSG00000144848|transcript|E
protein_co	5	0.8	G|frameshift_variant|HIGH|CD200R1L|ENSG00000206531|transcrip
protein_coding			T|splice_donor_variant&5_prime_UTR_variant&intron_variant|HIGH
nonsense_	13	0.08	A|splice_donor_variant&intron_variant|HIGH|CFAP44|ENSG000002
nonsense_mediated_decay			C|stop_gained|HIGH|ZNF80|ENSG00000174255|transcript|ENST00
)259976			G|gene_fusion|HIGH|MIR568&ZBTB20|ENSG00000259976&ENSG00
)259976			G|gene_fusion|HIGH|MIR568&ZBTB20|ENSG00000259976&ENSG00
)259976			G|gene_fusion|HIGH|MIR568&ZBTB20|ENSG00000259976&ENSG00
)259976			G|gene_fusion|HIGH|MIR568&ZBTB20|ENSG00000259976&ENSG00
)259976			G|gene_fusion|HIGH|MIR568&ZBTB20|ENSG00000259976&ENSG00
antisense			A|splice_donor_variant&intron_variant|HIGH|ZBTB20-AS1|ENSG000
protein_co	7	0.14	A|frameshift_variant|HIGH|LSAMP|ENSG00000185565|transcript|E
processed_transcript			G|splice_acceptor_variant&intron_variant|HIGH|B4GALT4|ENSG000
nonsense_mediated_decay			C|stop_lost|HIGH|TIMMDC1|ENSG00000113845|transcript|ENST00
protein_coding			G|structural_interaction_variant|HIGH|HGD|ENSG00000113924|int
processed_transcript			A|splice_donor_variant&intron_variant|HIGH|RABL3|ENSG0000014
protein_co	4	0.5	C|frameshift_variant|HIGH|POLQ|ENSG00000051341|transcript|EN
protein_co	1	1	T|stop_gained|HIGH|ARGFX|ENSG00000186103|transcript|ENST00
protein_coding			T|protein_protein_contact|HIGH|KPNA1|ENSG00000114030|intera
protein_coding			T|structural_interaction_variant|HIGH|PARP14|ENSG00000173193|
nonsense_	6	0.17	A|splice_donor_variant&intron_variant|HIGH|PARP14|ENSG000001
protein_co	22	0.09	G|start_lost|HIGH|KALRN|ENSG00000160145|transcript|ENST0000
protein_coding			T|structural_interaction_variant|HIGH|UMPS|ENSG00000114491|ir
protein_coding			G|structural_interaction_variant|HIGH|UMPS|ENSG00000114491|ir

lincRNA			G|splice_acceptor_variant&intron_variant|HIGH|RP11-379B18.5|EN
protein_coding			C|splice_acceptor_variant&5_prime_UTR_variant&intron_variant|H
protein_coding			T|frameshift_variant|HIGH|SLC41A3|ENSG00000114544|transcript|
processed_transcript			G|splice_donor_variant&intron_variant|HIGH|SLC41A3|ENSG00000
protein_co	8	0.25	A|stop_gained|HIGH|CFAP100|ENSG00000163885|transcript|ENST
nonsense_mediated_decay			A|splice_donor_variant&3_prime_UTR_variant&intron_variant|HIGH
protein_coding			C|structural_interaction_variant|HIGH|MGLL|ENSG00000074416|ir
protein_coding			T|protein_protein_contact|HIGH|RAB7A|ENSG00000075785|intera
unprocessed_pseudogene			AG|splice_donor_variant&intron_variant|HIGH|RP11-529F4.1|ENSG
nonsense_mediated_decay			A|stop_gained&splice_region_variant|HIGH|MBD4|ENSG0000012900
antisense			G|splice_donor_variant&intron_variant|HIGH|RP11-93K22.6|ENSG0
protein_co	3	0.33	G|frameshift_variant|HIGH|ALG1L2|ENSG00000251287|transcript|
transcribed_unprocessed_pseudo			G|splice_acceptor_variant&intron_variant|HIGH|FAM86HP|ENSG00
nonsense_	5	1	T|stop_gained|HIGH|COL6A5|ENSG00000172752|transcript|ENST0
nonsense_mediated_decay			A|frameshift_variant|HIGH|COL6A5|ENSG00000172752|transcript|
protein_co	3	0.33,(ACAD	A|splice_donor_variant&intron_variant|HIGH|ACAD11|ENSG000002
antisense			T|splice_acceptor_variant&intron_variant|HIGH|NPHP3-AS1|ENSG0
antisense			A|splice_donor_variant&intron_variant|HIGH|NPHP3-AS1|ENSG000
protein_coding			G|structural_interaction_variant|HIGH|TOPBP1|ENSG00000163781
protein_coding			T|structural_interaction_variant|HIGH|RAB6B|ENSG00000154917|i
protein_co	5	0.6	CCAAT|splice_acceptor_variant&intron_variant|HIGH|PPP2R3A|ENS
protein_co	11	0.64	C|splice_acceptor_variant&intron_variant|HIGH|MRAS|ENSG00000
protein_co	3	0.33	C|frameshift_variant|HIGH|FOXL2NB|ENSG00000206262|transcript
nonsense_	14	0.14	A|splice_donor_variant&intron_variant|HIGH|MRPS22|ENSG000001
protein_coding			A|structural_interaction_variant|HIGH|NMNAT3|ENSG0000016386
antisense			G|splice_donor_variant&intron_variant|HIGH|CLSTN2-AS1|ENSG000
protein_coding			A|structural_interaction_variant|HIGH|SPSB4|ENSG00000175093|ir
protein_co	17	0.06	G|splice_donor_variant&intron_variant|HIGH|PXYLP1|ENSG000001
antisense			CTTT|splice_acceptor_variant&intron_variant|HIGH|PAQR9-AS1|EN
antisense			CTTT|splice_acceptor_variant&intron_variant|HIGH|PAQR9-AS1|EN
antisense			CTTT|splice_acceptor_variant&intron_variant|HIGH|PAQR9-AS1|EN
nonsense_mediated_decay			T|stop_gained|HIGH|U2SURP|ENSG00000163714|transcript|ENST0
protein_coding			C|structural_interaction_variant|HIGH|GYG1|ENSG00000163754|in
protein_co	9	0.44	T|stop_gained|HIGH|HLTF|ENSG00000071794|transcript|ENST0000
protein_coding			CTTAA|frameshift_variant&stop_gained|HIGH|WWTR1|ENSG00000
protein_co	12	0.08	A|splice_donor_variant&intron_variant|HIGH|WWTR1|ENSG000000
lincRNA			T|splice_donor_variant&intron_variant|HIGH|RP11-167H9.5|ENSG0
lincRNA			T|splice_acceptor_variant&intron_variant|HIGH|RP11-373I8.1|ENS
protein_coding			T|stop_gained|HIGH|IGSF10|ENSG00000152580|transcript|ENST00
lincRNA			A|splice_donor_variant&intron_variant|HIGH|RP11-217E22.5|ENSG
protein_co	18	0.06	A|splice_donor_variant&intron_variant|HIGH|MME|ENSG0000019
protein_coding			C|structural_interaction_variant|HIGH|GMPS|ENSG00000163655|ir
protein_co	15	0.07	A|stop_gained|HIGH|KCNAB1|ENSG00000169282|transcript|ENST
protein_co	11	0.09	AT|frameshift_variant|HIGH|LEKR1|ENSG00000197980|transcript|
protein_co	19	0.11	G|splice_donor_variant&intron_variant|HIGH|VEPH1|ENSG0000019
protein_coding			T|structural_interaction_variant|HIGH|LXN|ENSG00000079257|inte
protein_co	8	0.13	C|frameshift_variant|HIGH|TRIM59|ENSG00000213186|transcript|

lincRNA			A|splice_donor_variant&intron_variant|HIGH|RP11-3P17.4|ENSG00
protein_coding			A|structural_interaction_variant|HIGH|SERPINI1|ENSG0000016353(
nonsense_	3	0.33	C|splice_donor_variant&intron_variant|HIGH|SAMD7|ENSG000001
processed_transcript			C|splice_acceptor_variant&intron_variant|HIGH|FNDC3B|ENSG0000
protein_coding			G|structural_interaction_variant|HIGH|TNFSF10|ENSG0000012185
protein_coding			G|structural_interaction_variant|HIGH|TNFSF10|ENSG0000012185
protein_co	16	0.31	GTT|splice_acceptor_variant&intron_variant|HIGH|ECT2|ENSG0000
protein_co	16	0.31	GTT|splice_acceptor_variant&intron_variant|HIGH|ECT2|ENSG0000
nonsense_mediated_decay			T|frameshift_variant|HIGH|TBL1XR1|ENSG00000177565|transcript
nonsense_mediated_decay			T|frameshift_variant|HIGH|TBL1XR1|ENSG00000177565|transcript
nonsense_mediated_decay			T|frameshift_variant|HIGH|TBL1XR1|ENSG00000177565|transcript
nonsense_mediated_decay			T|frameshift_variant|HIGH|TBL1XR1|ENSG00000177565|transcript
nonsense_mediated_decay			T|frameshift_variant|HIGH|TBL1XR1|ENSG00000177565|transcript
lincRNA			C|splice_acceptor_variant&splice_region_variant&intron_variant|HI
lincRNA			C|splice_acceptor_variant&splice_region_variant&intron_variant|HI
lincRNA			C|splice_acceptor_variant&splice_region_variant&intron_variant|HI
lincRNA			C|splice_acceptor_variant&splice_region_variant&intron_variant|HI
lincRNA			C|splice_acceptor_variant&splice_region_variant&intron_variant|HI
lincRNA			C|splice_acceptor_variant&splice_region_variant&intron_variant|HI
nonsense_	10	0.1	ATTTT|splice_acceptor_variant&intron_variant|HIGH|KCNMB2|ENS
nonsense_	10	0.1	ATTTT|splice_acceptor_variant&intron_variant|HIGH|KCNMB2|ENS
nonsense_	10	0.1	ATTTT|splice_acceptor_variant&intron_variant|HIGH|KCNMB2|ENS
protein_coding			G|structural_interaction_variant|HIGH|PIK3CA|ENSG00000121879|
protein_co	7	0.71	C|frameshift_variant|HIGH|KCNMB3|ENSG00000171121|transcript
protein_co	16	0.06	GCTGT|frameshift_variant|HIGH|NDUFB5|ENSG00000136521|trans
protein_co	7	0.43	TA|frameshift_variant|HIGH|CCDC39|ENSG00000145075|transcript
lincRNA			A|splice_acceptor_variant&intron_variant|HIGH|LINC01206|ENSG0
processed_pseudogene			G|splice_acceptor_variant&intron_variant|HIGH|RP11-531F16.3|EN
processed_pseudogene			G|splice_acceptor_variant&intron_variant|HIGH|RP11-531F16.3|EN
processed_pseudogene			C|splice_acceptor_variant&intron_variant|HIGH|RP11-531F16.3|EN
processed_pseudogene			C|splice_acceptor_variant&intron_variant|HIGH|RP11-531F16.3|EN
protein_coding			A|structural_interaction_variant|HIGH|LAMP3|ENSG00000078081|
retained_intron			T|splice_donor_variant&intron_variant|HIGH|MCF2L2|ENSG000000
protein_co	15	0.2	A|splice_acceptor_variant&intron_variant|HIGH|KLHL24|ENSG0000
transcribed_unprocessed_pseudo			G|splice_acceptor_variant&intron_variant|HIGH|CYP2AB1P|ENSG00
protein_co	4	0.25	C|splice_acceptor_variant&intron_variant|HIGH|HTR3D|ENSG00000
protein_co	4	0.25	C|splice_donor_variant&intron_variant|HIGH|HTR3D|ENSG0000018
unprocessed_pseudogene			A|splice_acceptor_variant&intron_variant|HIGH|RP11-778D9.9|ENS
protein_coding			A|frameshift_variant|HIGH|HTR3E|ENSG00000186038|transcript|E
protein_coding			T|structural_interaction_variant|HIGH|ECE2|ENSG00000145194|int
protein_coding			C|structural_interaction_variant|HIGH|ECE2|ENSG00000145194|int
protein_coding			G|structural_interaction_variant|HIGH|EPHB3|ENSG00000182580|i
protein_coding			A|structural_interaction_variant|HIGH|EPHB3|ENSG00000182580|i
protein_coding			T|structural_interaction_variant|HIGH|EPHB3|ENSG00000182580|i
protein_coding			T|stop_gained|HIGH|KNG1|ENSG00000113889|transcript|ENST000
processed_transcript			A|splice_donor_variant&intron_variant|HIGH|PSMD10P2|ENSG000
protein_co	22	0.05	A|splice_donor_variant&intron_variant|HIGH|ST6GAL1|ENSG00000

protein_coding			T|structural_interaction_variant|HIGH|MASP1|ENSG00000127241|
protein_coding			A|structural_interaction_variant|HIGH|MASP1|ENSG00000127241|
protein_co	3	0.67	A|frameshift_variant|HIGH|CLDN16|ENSG00000113946|transcript|
protein_co	2	0.5	A|splice_donor_variant&intron_variant|HIGH|HRASLS|ENSG000001
retained_intron			T|splice_donor_variant&intron_variant|HIGH|ATP13A5|ENSG00000
protein_co	5	0.2	A|stop_gained|HIGH|ATP13A5|ENSG00000187527|transcript|ENST
lincRNA			G|splice_donor_variant&intron_variant|HIGH|RP11-528A4.2|ENSG(
protein_coding			A|stop_gained|HIGH|CPN2|ENSG00000178772|transcript|ENST000
protein_coding			A|stop_gained|HIGH|LRRC15|ENSG00000172061|transcript|ENST0
nonsense_	7	0.14	C|splice_acceptor_variant&intron_variant|HIGH|ZDHHC19|ENSG000
processed_transcript			T|splice_donor_variant&intron_variant|HIGH|TCTEX1D2|ENSG0000
protein_coding			A|stop_gained|HIGH|TM4SF19|ENSG00000145107|transcript|ENST
protein_coding			T|structural_interaction_variant|HIGH|DLG1|ENSG00000075711|in
protein_co	13	0.23	T|stop_gained|HIGH|IQCG|ENSG00000114473|transcript|ENST000
antisense			A|splice_acceptor_variant&splice_region_variant&intron_variant&n
protein_co	12	0.08	C|splice_donor_variant&intron_variant|HIGH|PDE6B|ENSG0000013
protein_co	13	0.08	T|frameshift_variant|HIGH|PCGF3|ENSG00000185619|transcript|E
protein_co	13	0.08	C|frameshift_variant|HIGH|PCGF3|ENSG00000185619|transcript|E
protein_co	22	0.14	C|splice_acceptor_variant&intron_variant|HIGH|TMEM175|ENSG00
protein_coding			G|structural_interaction_variant|HIGH|IDUA|ENSG00000127415|in
protein_coding			C|structural_interaction_variant|HIGH|IDUA|ENSG00000127415|in
protein_coding			T|structural_interaction_variant|HIGH|IDUA|ENSG00000127415|in
protein_coding			C|structural_interaction_variant|HIGH|IDUA|ENSG00000127415|in
protein_coding			A|structural_interaction_variant|HIGH|IDUA|ENSG00000127415|in
protein_coding			A|structural_interaction_variant|HIGH|IDUA|ENSG00000127415|in
protein_coding			A|structural_interaction_variant|HIGH|IDUA|ENSG00000127415|in
retained_intron			G|splice_donor_variant&splice_region_variant&intron_variant&non
retained_intron			G|splice_donor_variant&splice_region_variant&intron_variant&non
protein_coding			A|stop_gained|HIGH|RNF212|ENSG00000178222|transcript|ENST0
nonsense_mediated_decay			A|stop_gained&splice_region_variant|HIGH|RNF212|ENSG0000017
protein_coding			C|splice_donor_variant&splice_region_variant&5_prime_UTR_variar
antisense			C|splice_acceptor_variant&intron_variant|HIGH|CTBP1-AS2|ENSG0
protein_co	1	1	GTGCCCATGTGGAGTGCCCGCCTGCTCACACA|frameshift_variant|HIG
protein_co	1	1	A|frameshift_variant|HIGH|CRIPAK|ENSG00000179979|transcript|E
protein_co	1	1	A|frameshift_variant|HIGH|CRIPAK|ENSG00000179979|transcript|E
protein_co	1	1	A|frameshift_variant|HIGH|CRIPAK|ENSG00000179979|transcript|E
protein_co	1	1	TCA|frameshift_variant|HIGH|CRIPAK|ENSG00000179979|transcrip
protein_co	1	1	TCA|frameshift_variant|HIGH|CRIPAK|ENSG00000179979|transcrip
protein_co	1	1	CGGAGTGCCCGCCTGCTCACACGTGCCGACGT|frameshift_variant|HIG
protein_co	1	1	CGTGGAGTGCCCGCCTGCTCACACGTGCCCAT|frameshift_variant|HIG
protein_co	1	1	T|frameshift_variant|HIGH|CRIPAK|ENSG00000179979|transcript|E
protein_co	1	1	A|frameshift_variant|HIGH|CRIPAK|ENSG00000179979|transcript|E
protein_co	1	1	A|frameshift_variant|HIGH|CRIPAK|ENSG00000179979|transcript|E
protein_co	1	1	T|frameshift_variant|HIGH|CRIPAK|ENSG00000179979|transcript|E
protein_co	1	1	T|frameshift_variant|HIGH|CRIPAK|ENSG00000179979|transcript|E
protein_coding			G|structural_interaction_variant|HIGH|POLN|ENSG00000130997|ir
protein_coding			T|stop_gained|HIGH|GRK4|ENSG00000125388|transcript|ENST000

protein_coding			A|frameshift_variant|HIGH|HTT|ENSG00000197386|transcript|ENS
protein_coding			A|frameshift_variant|HIGH|HTT|ENSG00000197386|transcript|ENS
protein_coding			A|frameshift_variant|HIGH|HTT|ENSG00000197386|transcript|ENS
protein_coding			ACAGCAGCAACAG|frameshift_variant|HIGH|HTT|ENSG0000019738
protein_coding			A|frameshift_variant|HIGH|HTT|ENSG00000197386|transcript|ENS
protein_coding			A|frameshift_variant|HIGH|HTT|ENSG00000197386|transcript|ENS
protein_coding			ACAG|frameshift_variant|HIGH|HTT|ENSG00000197386|transcript|
protein_coding			ACAG|frameshift_variant|HIGH|HTT|ENSG00000197386|transcript|
protein_coding			ACAG|frameshift_variant|HIGH|HTT|ENSG00000197386|transcript|
protein_coding			CAG|frameshift_variant|HIGH|HTT|ENSG00000197386|transcript|E
protein_coding			A|frameshift_variant|HIGH|HTT|ENSG00000197386|transcript|ENS
protein_coding			A|frameshift_variant|HIGH|HTT|ENSG00000197386|transcript|ENS
protein_coding			A|frameshift_variant|HIGH|HTT|ENSG00000197386|transcript|ENS
protein_coding			A|frameshift_variant|HIGH|HTT|ENSG00000197386|transcript|ENS
protein_coding			A|structural_interaction_variant|HIGH|HGFAC|ENSG00000109758|
lincRNA			G|splice_acceptor_variant&intron_variant|HIGH|RP11-529E10.7|EN
processed_transcript			CTTT|splice_acceptor_variant&intron_variant|HIGH|STK32B|ENSG0
processed_transcript			CTTT|splice_acceptor_variant&intron_variant|HIGH|STK32B|ENSG0
processed_transcript			CTTT|splice_acceptor_variant&intron_variant|HIGH|STK32B|ENSG0
processed_transcript			A|splice_donor_variant&intron_variant|HIGH|STK32B|ENSG000001
protein_co	2	1	T|splice_donor_variant&intron_variant|HIGH|C4orf50|ENSG000001
protein_co	2	0.5	C|frameshift_variant|HIGH|C4orf50|ENSG00000181215|transcript|
lincRNA			A|splice_acceptor_variant&intron_variant|HIGH|AC093323.3|ENSG
processed_transcript			G|splice_donor_variant&intron_variant|HIGH|ABLIM2|ENSG000001
processed_transcript			T|splice_donor_variant&intron_variant|HIGH|ACOX3|ENSG0000008
protein_co	9	0.22	A|stop_gained|HIGH|TRMT44|ENSG00000155275|transcript|ENST(
processed_pseudogene			C|splice_acceptor_variant&intron_variant|HIGH|AC006445.7|ENSG
processed_pseudogene			C|splice_acceptor_variant&intron_variant|HIGH|AC006445.7|ENSG
lincRNA			T|splice_donor_variant&intron_variant|HIGH|CPEB2-AS1|ENSG0000
protein_co	9	0.22	C|frameshift_variant|HIGH|CPEB2|ENSG00000137449|transcript|E
protein_co	17	0.18	T|stop_gained|HIGH|CC2D2A|ENSG00000048342|transcript|ENST0
protein_coding			T|stop_lost|HIGH|BST1|ENSG00000109743|transcript|ENST000005
nonsense_mediated_decay			C|stop_lost|HIGH|CD38|ENSG00000004468|transcript|ENST000005
protein_coding			A|structural_interaction_variant|HIGH|CD38|ENSG00000004468|in
antisense			CTTT|splice_acceptor_variant&intron_variant|HIGH|TAPT1-AS1|ENS
antisense			CTTT|splice_acceptor_variant&intron_variant|HIGH|TAPT1-AS1|ENS
antisense			CTTT|splice_acceptor_variant&intron_variant|HIGH|TAPT1-AS1|ENS
nonsense_mediated_decay			T|stop_lost|HIGH|QDPR|ENSG00000151552|transcript|ENST00000
nonsense_mediated_decay			G|stop_lost|HIGH|MED28|ENSG00000118579|transcript|ENST0000
lincRNA			T|splice_donor_variant&intron_variant|HIGH|RP11-608O21.1|ENSG
protein_coding			T|structural_interaction_variant|HIGH|SLIT2|ENSG00000145147|in
unprocessed_pseudogene			C|splice_acceptor_variant&intron_variant|HIGH|RP11-10G12.2|ENS
polymorph	7	0.29	T|stop_lost|HIGH|GBA3|ENSG00000249948|transcript|ENST00000
lincRNA			G|splice_acceptor_variant&intron_variant|HIGH|RP11-552M14.1|E
protein_co	8	0.13	T|splice_donor_variant&intron_variant|HIGH|CCDC149|ENSG0000C
protein_co	9	0.22	CT|splice_acceptor_variant&intron_variant|HIGH|TBC1D19|ENSG00
lincRNA			C|splice_acceptor_variant&splice_region_variant&intron_variant|HI

antisense			CTT|splice_acceptor_variant&intron_variant|HIGH|RP11-431M7.3|
antisense			CTT|splice_acceptor_variant&intron_variant|HIGH|RP11-431M7.3|
protein_coding			C|frameshift_variant|HIGH|PTTG2|ENSG00000250254|transcript|
retained_intron			CT|splice_acceptor_variant&intron_variant|HIGH|TBC1D1|ENSG000
protein_coding			C|structural_interaction_variant|HIGH|TLR10|ENSG00000174123|i
protein_coding			G|structural_interaction_variant|HIGH|TLR10|ENSG00000174123|i
protein_coding			C|structural_interaction_variant|HIGH|TLR6|ENSG00000174130|int
protein_coding			G|structural_interaction_variant|HIGH|UGDH|ENSG00000109814|i
protein_coding			C|structural_interaction_variant|HIGH|UGDH|ENSG00000109814|i
nonsense_	8	0.13	C|splice_acceptor_variant&intron_variant|HIGH|TMEM33|ENSG000
nonsense_mediated_decay			A|splice_donor_variant&splice_region_variant&3_prime_UTR_varia
protein_co	4	0.25	C|frameshift_variant|HIGH|GUF1|ENSG00000151806|transcript|EN
processed_pseudogene			C|splice_acceptor_variant&intron_variant|HIGH|PRDX4P1|ENSG000
protein_co	5	0.4	C|frameshift_variant|HIGH|CWH43|ENSG00000109182|transcript|
processed_pseudogene			A|splice_donor_variant&splice_region_variant&intron_variant&non
processed_pseudogene			T|splice_acceptor_variant&splice_region_variant&intron_variant|HI
processed_pseudogene			T|splice_acceptor_variant&splice_region_variant&intron_variant|HI
lincRNA			C|splice_donor_variant&intron_variant|HIGH|RP11-177B4.2|ENSG0
protein_co	13	0.31	C|frameshift_variant|HIGH|FIP1L1|ENSG00000145216|transcript|E
protein_coding			G|structural_interaction_variant|HIGH|KIT|ENSG00000157404|inte
protein_coding			T|structural_interaction_variant|HIGH|KIT|ENSG00000157404|inte
protein_coding			T|structural_interaction_variant|HIGH|KDR|ENSG00000128052|inte
protein_coding			C|structural_interaction_variant|HIGH|PAICS|ENSG00000128050|i
processed_pseudogene			G|splice_acceptor_variant&splice_region_variant&intron_variant&n
protein_coding			A|structural_interaction_variant|HIGH|EPHA5|ENSG00000145242|i
protein_co	3	0.67	T|start_lost|HIGH|TMPRSS11A|ENSG00000187054|transcript|ENST
protein_co	5	0.2	T|splice_donor_variant&intron_variant|HIGH|UGT2B7|ENSG000001
protein_co	7	0.57	T|stop_gained|HIGH|UGT2A1|ENSG00000173610|transcript|ENST0
protein_co	7	0.57	A|splice_acceptor_variant&intron_variant|HIGH|MUC7|ENSG00000
protein_co	7	0.57	AC|frameshift_variant|HIGH|MUC7|ENSG00000171195|transcript|
protein_coding			T|structural_interaction_variant|HIGH|DCK|ENSG00000156136|inte
protein_co	8	0.13	A|start_lost|HIGH|GC|ENSG00000145321|transcript|ENST0000050
protein_coding			C|stop_lost|HIGH|THAP6|ENSG00000174796|transcript|ENST00000
protein_co	4	0.5	T|splice_acceptor_variant&intron_variant|HIGH|CDKL2|ENSG00000
nonsense_	7	0.29	C|splice_acceptor_variant&intron_variant|HIGH|PPEF2|ENSG00000
protein_co	7	0.43	G|splice_donor_variant&intron_variant|HIGH|PPEF2|ENSG0000015
nonsense_	18	0.06	A|splice_donor_variant&intron_variant|HIGH|NUP54|ENSG0000013
protein_coding			GAC|frameshift_variant|HIGH|CNOT6L|ENSG00000138767|transcri
protein_coding			GAC|frameshift_variant|HIGH|CNOT6L|ENSG00000138767|transcri
protein_coding			GAC|frameshift_variant|HIGH|CNOT6L|ENSG00000138767|transcri
protein_coding			GAC|frameshift_variant|HIGH|CNOT6L|ENSG00000138767|transcri
protein_coding			GAC|frameshift_variant|HIGH|CNOT6L|ENSG00000138767|transcri
protein_coding			GAC|frameshift_variant|HIGH|CNOT6L|ENSG00000138767|transcri
nonsense_mediated_decay			C|stop_lost|HIGH|FRAS1|ENSG00000138759|transcript|ENST0000
lincRNA			A|splice_donor_variant&intron_variant|HIGH|RP11-452C8.1|ENSG0
processed_transcript			C|splice_acceptor_variant&intron_variant|HIGH|RASGEF1B|ENSG00
protein_co	5	0.2	A|stop_gained|HIGH|COQ2|ENSG00000173085|transcript|ENST000

protein_co	9	0.11	AG|frameshift_variant|HIGH|FAM175A|ENSG00000163322|transcri
protein_co	12	0.08	T|splice_donor_variant&intron_variant|HIGH|C4orf36|ENSG000001
protein_co	2	1	TA|splice_donor_variant&intron_variant|HIGH|HSD17B13|ENSG000
protein_co	2	1	C|stop_gained|HIGH|HSD17B13|ENSG00000170509|transcript|ENS
protein_co	2	1	C|frameshift_variant|HIGH|DSPP|ENSG00000152591|transcript|EN
protein_co	2	1	G|frameshift_variant|HIGH|DSPP|ENSG00000152591|transcript|EN
processed_transcript			T|splice_acceptor_variant&intron_variant|HIGH|HERC3|ENSG00000
protein_co	22	0.14	C|splice_acceptor_variant&intron_variant|HIGH|FAM13A|ENSG000
protein_co	13	0.08	CT|splice_acceptor_variant&intron_variant|HIGH|SNCA|ENSG00000
protein_co	4	0.25	G|splice_acceptor_variant&intron_variant|HIGH|MMRN1|ENSG000
processed_	13	0.08	G|splice_acceptor_variant&intron_variant|HIGH|CCSER1|ENSG0000
protein_coding			C|structural_interaction_variant|HIGH|HPGDS|ENSG00000163106|
protein_coding			G|structural_interaction_variant|HIGH|PDLIM5|ENSG00000163110
lincRNA			G|splice_donor_variant&intron_variant|HIGH|RP11-431J17.1|ENSG
processed_transcript			C|splice_donor_variant&intron_variant|HIGH|EIF4E|ENSG00000151
nonsense_	12	0.08	A|splice_donor_variant&intron_variant|HIGH|METAP1|ENSG00000
protein_coding			A|structural_interaction_variant|HIGH|ADH4|ENSG00000198099|ir
protein_coding			G|structural_interaction_variant|HIGH|ADH4|ENSG00000198099|ir
protein_co	12	0.25	T|stop_gained|HIGH|ADH4|ENSG00000198099|transcript|ENST000
antisense			C|splice_acceptor_variant&intron_variant|HIGH|RP11-696N14.1|EN
antisense			A|splice_acceptor_variant&intron_variant|HIGH|RP11-696N14.1|EN
retained_intron			G|splice_donor_variant&intron_variant|HIGH|ADH1A|ENSG000001
protein_co	4	0.75	A|stop_gained|HIGH|ADH1C|ENSG00000248144|transcript|ENST00
nonsense_mediated_decay			ACAGT|frameshift_variant&splice_region_variant|HIGH|C4orf17|EN
protein_co	11	0.45	A|start_lost|HIGH|MTTP|ENSG00000138823|transcript|ENST00000
protein_co	3	0.33	G|frameshift_variant|HIGH|DDIT4L|ENSG00000145358|transcript|
antisense			T|splice_acceptor_variant&intron_variant|HIGH|RP11-588P8.1|ENS
protein_co	8	0.63	G|frameshift_variant|HIGH|BANK1|ENSG00000153064|transcript|
protein_co	12	0.08	T|splice_donor_variant&intron_variant|HIGH|NFKB1|ENSG0000010
protein_co	12	0.17	G|start_lost|HIGH|NFKB1|ENSG00000109320|transcript|ENST0000
protein_coding			T|structural_interaction_variant|HIGH|NFKB1|ENSG00000109320|i
protein_co	33	0.03	CT|frameshift_variant&splice_region_variant|HIGH|UBE2D3|ENSG0
protein_co	33	0.03	CT|frameshift_variant&splice_region_variant|HIGH|UBE2D3|ENSG0
protein_co	33	0.03	CT|frameshift_variant&splice_region_variant|HIGH|UBE2D3|ENSG0
protein_coding			C|structural_interaction_variant|HIGH|CENPE|ENSG00000138778|i
protein_coding			G|structural_interaction_variant|HIGH|CENPE|ENSG00000138778|i
lincRNA			A|splice_donor_variant&intron_variant|HIGH|RP11-297P16.4|ENSG
lincRNA			G|splice_donor_variant&intron_variant|HIGH|RP11-556I14.2|ENSG
protein_co	20	0.05	T|start_lost|HIGH|TBCK|ENSG00000145348|transcript|ENST00000
protein_coding			C|structural_interaction_variant|HIGH|PAPSS1|ENSG00000138801|
nonsense_	10	0.1	C|splice_acceptor_variant&intron_variant|HIGH|HADH|ENSG00000
antisense			T|splice_acceptor_variant&intron_variant|HIGH|SEC24B-AS1|ENSG
protein_co	12	0.25	GC|frameshift_variant|HIGH|EGF|ENSG00000138798|transcript|EN
protein_co	5	0.2	A|stop_gained|HIGH|ENPEP|ENSG00000138792|transcript|ENST00
protein_coding			A|stop_gained|HIGH|PITX2|ENSG00000164093|transcript|ENST000
nonsense_	15	0.07	C|frameshift_variant|HIGH|LARP7|ENSG00000174720|transcript|E
transcribed_unprocessed_pseudo			G|splice_acceptor_variant&intron_variant|HIGH|CEP170P1|ENSG00

processed_transcript			A|splice_donor_variant&intron_variant|HIGH|RP11-33B1.1|ENSG0C
protein_coding			C|structural_interaction_variant|HIGH|PDE5A|ENSG00000138735|i
protein_coding			C|structural_interaction_variant|HIGH|MAD2L1|ENSG00000164109
protein_coding			C|structural_interaction_variant|HIGH|MAD2L1|ENSG00000164109
processed_pseudogene			T|splice_acceptor_variant&splice_region_variant&intron_variant&n
processed_pseudogene			T|splice_acceptor_variant&splice_region_variant&intron_variant&n
processed_pseudogene			T|splice_acceptor_variant&splice_region_variant&intron_variant&n
processed_pseudogene			T|splice_acceptor_variant&splice_region_variant&intron_variant&n
protein_coding			T|protein_protein_contact|HIGH|CCNA2|ENSG00000145386|intera
protein_co	16	0.19	ATT|splice_acceptor_variant&intron_variant|HIGH|KIAA1109|ENSG
protein_co	16	0.19	ATT|splice_acceptor_variant&intron_variant|HIGH|KIAA1109|ENSG
protein_co	11	0.45	GT|splice_acceptor_variant&intron_variant|HIGH|PLK4|ENSG00000
protein_co	11	0.45	AT|splice_acceptor_variant&intron_variant|HIGH|PLK4|ENSG00000
protein_coding			C|frameshift_variant|HIGH|PGRMC2|ENSG00000164040|transcript
lincRNA			AT|splice_acceptor_variant&intron_variant|HIGH|RP11-422J15.1|EI
lincRNA			T|splice_acceptor_variant&intron_variant|HIGH|RP11-789C2.1|ENS
lincRNA			A|splice_donor_variant&intron_variant|HIGH|RP11-553P9.2|ENSG
lincRNA			T|splice_donor_variant&intron_variant|HIGH|LINC00616|ENSG000
protein_co	3	0.33	T|frameshift_variant&splice_acceptor_variant&splice_region_varian
protein_co	3	0.33	T|frameshift_variant&splice_acceptor_variant&splice_region_varian
protein_co	3	0.33	G|frameshift_variant|HIGH|MAML3|ENSG00000196782|transcript|
protein_co	3	0.33	T|frameshift_variant|HIGH|MAML3|ENSG00000196782|transcript|
protein_co	3	0.33	G|frameshift_variant|HIGH|MAML3|ENSG00000196782|transcript|
protein_co	3	0.33	G|frameshift_variant|HIGH|MAML3|ENSG00000196782|transcript|
protein_co	3	0.67	TTGC|frameshift_variant|HIGH|MAML3|ENSG00000196782|transcr
protein_co	3	0.67	TTGC|frameshift_variant|HIGH|MAML3|ENSG00000196782|transcr
protein_co	3	0.33	CTGCTGT|splice_donor_variant&intron_variant|HIGH|MAML3|ENS
protein_co	8	0.25	A|splice_donor_variant&intron_variant|HIGH|ELMOD2|ENSG00000
processed_pseudogene			C|splice_acceptor_variant&intron_variant|HIGH|RP11-780M14.1|EI
lincRNA			T|splice_donor_variant&splice_region_variant&intron_variant&non_
protein_co	15	0.07	G|splice_acceptor_variant&intron_variant|HIGH|GYPB|ENSG00000
nonsense_	15	0.07	AT|frameshift_variant|HIGH|GYPB|ENSG00000250361|transcript|E
protein_coding			C|structural_interaction_variant|HIGH|ANAPC10|ENSG0000016416
protein_co	10	0.4	ATT|splice_acceptor_variant&intron_variant|HIGH|ABCE1|ENSG000
protein_co	10	0.4	ATT|splice_acceptor_variant&intron_variant|HIGH|ABCE1|ENSG000
lincRNA			C|splice_donor_variant&intron_variant|HIGH|RP11-24I21.1|ENSG0
nonsense_	6	0.5	T|frameshift_variant|HIGH|RP11-526A4.1|ENSG00000234828|trans
processed_transcript			T|splice_donor_variant&intron_variant|HIGH|RP11-526A4.1|ENSG0
protein_co	2	0.5	GCAGGT|frameshift_variant|HIGH|PRSS48|ENSG00000189099|tran
protein_coding			G|stop_lost&splice_region_variant|HIGH|PRSS48|ENSG00000189099
nonsense_	6	0.17	T|frameshift_variant|HIGH|MND1|ENSG00000121211|transcript|EI
protein_coding			T|frameshift_variant|HIGH|DCHS2|ENSG00000197410|transcript|E
protein_coding			C|structural_interaction_variant|HIGH|PLRG1|ENSG00000171566|i
antisense			T|splice_donor_variant&intron_variant|HIGH|AC097467.2|ENSG000
antisense			A|splice_donor_variant&intron_variant|HIGH|AC097467.2|ENSG000
protein_co	7	0.14	A|splice_donor_variant&intron_variant|HIGH|GUCY1B3|ENSG00000
lincRNA			C|splice_acceptor_variant&intron_variant|HIGH|RP11-171N4.1|ENS

nonsense_	14	0.07	GT|splice_acceptor_variant&intron_variant|HIGH|GRIA2|ENSG0000
antisense			G|splice_donor_variant&intron_variant|HIGH|RP11-597D13.9|ENSG
protein_co	17	0.06	A|splice_acceptor_variant&intron_variant|HIGH|TMEM144|ENSG00
protein_co	1	1	T|splice_donor_variant&intron_variant|HIGH|TRIM61|ENSG000001
protein_coding			A|stop_gained|HIGH|RP11-668G10.2|ENSG00000229894|transcript
protein_co	16	0.06	T|splice_acceptor_variant&intron_variant|HIGH|DDX60L|ENSG0000
protein_co	7	0.14	CT|frameshift_variant|HIGH|CBR4|ENSG00000145439|transcript|E
protein_coding			T|structural_interaction_variant|HIGH|NEK1|ENSG00000137601|in
protein_co	11	0.09	AT|splice_acceptor_variant&intron_variant|HIGH|CLCN3|ENSG0000
protein_co	3	0.67	T|frameshift_variant|HIGH|SPATA4|ENSG00000150628|transcript|
protein_co	2	0.5	GTT|splice_acceptor_variant&intron_variant|HIGH|NEIL3|ENSG000
protein_co	2	0.5	GTT|splice_acceptor_variant&intron_variant|HIGH|NEIL3|ENSG000
protein_coding			G|structural_interaction_variant|HIGH|DCTD|ENSG00000129187|ir
transcribed_processed_pseudoge			G|splice_acceptor_variant&intron_variant|HIGH|FAM92A1P2|ENSG
lincRNA			ATT|splice_acceptor_variant&intron_variant|HIGH|RP11-386B13.3|
lincRNA			ATT|splice_acceptor_variant&intron_variant|HIGH|RP11-386B13.3|
protein_co	4	0.25	T|frameshift_variant|HIGH|C4orf47|ENSG00000205129|transcript|
protein_coding			T|structural_interaction_variant|HIGH|TLR3|ENSG00000164342|int
protein_coding			T|structural_interaction_variant|HIGH|TLR3|ENSG00000164342|int
protein_co	7	0.57	T|stop_gained|HIGH|KLKB1|ENSG00000164344|transcript|ENST000
protein_coding			A|structural_interaction_variant|HIGH|F11|ENSG00000088926|inte
processed_transcript			T|splice_donor_variant&intron_variant|HIGH|TRIML2|ENSG000001
antisense			A|splice_donor_variant&intron_variant|HIGH|RP11-366H4.3|ENSG0
lincRNA			A|splice_acceptor_variant&intron_variant|HIGH|RP11-366H4.1|ENS
nonsense_	20	0.05	TG|frameshift_variant|HIGH|SDHA|ENSG00000073578|transcript|E
protein_coding			C|frameshift_variant|HIGH|SDHA|ENSG00000073578|transcript|EN
protein_coding			C|structural_interaction_variant|HIGH|PDCD6|ENSG00000249915|i
nonsense_	10	0.2	C|splice_donor_variant&intron_variant|HIGH|EXOC3|ENSG0000018
protein_co	4	0.25	A|frameshift_variant|HIGH|CEP72|ENSG00000112877|transcript|E
protein_co	4	0.5	TAA|frameshift_variant|HIGH|ZDHHC11B|ENSG00000206077|trans
protein_co	4	0.5	C|frameshift_variant|HIGH|ZDHHC11B|ENSG00000206077|transcri
protein_co	4	0.25	C|start_lost|HIGH|ZDHHC11B|ENSG00000206077|transcript|ENST0
protein_coding			T|stop_gained|HIGH|ZDHHC11|ENSG00000188818|transcript|ENST
protein_co	11	0.09	C|frameshift_variant|HIGH|ZDHHC11|ENSG00000188818|transcript
protein_co	11	0.09	C|frameshift_variant&splice_region_variant|HIGH|ZDHHC11|ENSG0
protein_co	11	0.09	GT|frameshift_variant|HIGH|ZDHHC11|ENSG00000188818|transcri
nonsense_mediated_decay			A|stop_gained|HIGH|SLC6A19|ENSG00000174358|transcript|ENST
protein_co	2	0.5	G|stop_gained|HIGH|SLC6A18|ENSG00000164363|transcript|ENST
protein_co	5	0.2	TACCCGGCCGCACGCTCTC|splice_donor_variant&intron_variant|HIG
protein_coding			C|stop_lost|HIGH|NDUFS6|ENSG00000145494|transcript|ENST000
protein_coding			T|stop_gained|HIGH|C5orf38|ENSG00000186493|transcript|ENST0
unprocessed_pseudogene			C|splice_acceptor_variant&intron_variant|HIGH|RP11-404K5.2|ENS
processed_transcript			ATT|splice_acceptor_variant&intron_variant|HIGH|MTRR|ENSG000
processed_transcript			ATT|splice_acceptor_variant&intron_variant|HIGH|MTRR|ENSG000
protein_coding			T|structural_interaction_variant|HIGH|MTRR|ENSG00000124275|ir
sense_overlapping			T|splice_donor_variant&intron_variant|HIGH|CTD-2001E22.1|ENSG
protein_co	5	0.4	A|stop_gained|HIGH|ROPN1L|ENSG00000145491|transcript|ENST0

lincRNA			T|splice_donor_variant&splice_region_variant&intron_variant&non_
protein_coding			A|structural_interaction_variant|HIGH|MYO10|ENSG00000145555|
lincRNA			G|splice_acceptor_variant&intron_variant|HIGH|C5orf17|ENSG0000
protein_coding			A|structural_interaction_variant|HIGH|TARS|ENSG00000113407|in
protein_coding			T|stop_gained|HIGH|ADAMTS12|ENSG00000151388|transcript|EN
nonsense_mediated_decay			T|stop_lost|HIGH|AMACR|ENSG00000242110|transcript|ENST0000
processed_transcript			C|splice_donor_variant&intron_variant|HIGH|AMACR|ENSG000002
)242110			C|gene_fusion|HIGH|AMACR&C1QTNF3-AMACR|ENSG0000024211(
protein_co	28	0.14	AT|splice_acceptor_variant&intron_variant|HIGH|RAI14|ENSG0000
protein_co	28	0.14	AT|splice_acceptor_variant&intron_variant|HIGH|RAI14|ENSG0000
protein_co	5	0.2	CTTT|splice_acceptor_variant&intron_variant|HIGH|BRIX1|ENSG000
protein_co	5	0.2	CTTT|splice_acceptor_variant&intron_variant|HIGH|BRIX1|ENSG000
protein_co	5	0.2	CTTT|splice_acceptor_variant&intron_variant|HIGH|BRIX1|ENSG000
protein_co	5	0.2	TA|splice_acceptor_variant&intron_variant|HIGH|BRIX1|ENSG0000
protein_co	5	0.2	TA|splice_acceptor_variant&intron_variant|HIGH|BRIX1|ENSG0000
protein_co	6	0.17	A|splice_donor_variant&intron_variant|HIGH|AGXT2|ENSG0000011
protein_co	6	0.17	T|splice_donor_variant&intron_variant|HIGH|AGXT2|ENSG0000011
)274441			C|bidirectional_gene_fusion|HIGH|CTD-2282P23.2&SLC1A3|ENSG00
protein_co	7	0.14	G|splice_acceptor_variant&intron_variant|HIGH|WDR70|ENSG0000
lincRNA			ATT|splice_acceptor_variant&intron_variant|HIGH|LINC00604|ENS(
lincRNA			A|splice_region_variant&intron_variant|LOW|LINC00604|ENSG0000
protein_co	7	0.14	T|frameshift_variant|HIGH|TTC33|ENSG00000113638|transcript|EN
protein_coding			T|stop_gained|HIGH|TTC33|ENSG00000113638|transcript|ENST000
protein_co	2	0.5	C|frameshift_variant|HIGH|CARD6|ENSG00000132357|transcript|E
protein_co	6	0.17	T|stop_gained|HIGH|MROH2B|ENSG00000171495|transcript|ENST
protein_co	8	0.25	G|splice_donor_variant&intron_variant|HIGH|C6|ENSG0000003953
protein_co	4	0.5	CTT|splice_acceptor_variant&intron_variant|HIGH|C5orf51|ENSG00
protein_co	4	0.5	CTT|splice_acceptor_variant&intron_variant|HIGH|C5orf51|ENSG00
processed_pseudogene			T|splice_acceptor_variant&intron_variant|HIGH|SERBP1P6|ENSG00
nonsense_	20	0.05	ATTT|splice_acceptor_variant&intron_variant|HIGH|ZNF131|ENSG0
nonsense_	20	0.05	ATTT|splice_acceptor_variant&intron_variant|HIGH|ZNF131|ENSG0
protein_coding			T|structural_interaction_variant|HIGH|HMGCS1|ENSG00000112972
protein_coding			G|structural_interaction_variant|HIGH|HMGCS1|ENSG0000011297
protein_co	10	0.1	T|splice_donor_variant&intron_variant|HIGH|NNT|ENSG00000112
protein_co	22	0.05	CTT|splice_acceptor_variant&intron_variant|HIGH|PARP8|ENSG000
protein_co	22	0.05	CTT|splice_acceptor_variant&intron_variant|HIGH|PARP8|ENSG000
protein_coding			C|structural_interaction_variant|HIGH|MOCS2|ENSG00000164172|
processed_transcript			CT|splice_acceptor_variant&intron_variant|HIGH|SKIV2L2|ENSG000
nonsense_mediated_decay			A|stop_gained|HIGH|DDX4|ENSG00000152670|transcript|ENST000
protein_co	1	1	G|frameshift_variant|HIGH|AC008914.1|ENSG00000279768|transc
protein_co	1	1	GT|frameshift_variant|HIGH|AC008914.1|ENSG00000279768|trans
protein_co	1	1	GTTT|frameshift_variant|HIGH|AC008914.1|ENSG00000279768|tra
protein_coding			G|frameshift_variant|HIGH|ANKRD55|ENSG00000164512|transcrip
protein_co	11	0.09	A|splice_donor_variant&intron_variant|HIGH|MIER3|ENSG0000015
protein_coding			G|structural_interaction_variant|HIGH|PLK2|ENSG00000145632|in
processed_transcript			T|splice_acceptor_variant&intron_variant|HIGH|GAPT|ENSG000001
lincRNA			G|splice_acceptor_variant&intron_variant|HIGH|C5orf64|ENSG0000

processed_pseudogene			C|splice_acceptor_variant&splice_region_variant&intron_variant|HI
processed_pseudogene			C|splice_acceptor_variant&splice_region_variant&intron_variant|HI
processed_pseudogene			C|splice_acceptor_variant&splice_region_variant&intron_variant|HI
processed_pseudogene			C|splice_acceptor_variant&splice_region_variant&intron_variant|HI
)113595			C|bidirectional_gene_fusion|HIGH|TRAPPC13&TRIM23|ENSG00000
)113595			C|bidirectional_gene_fusion|HIGH|TRAPPC13&TRIM23|ENSG00000
)113595			C|bidirectional_gene_fusion|HIGH|TRAPPC13&TRIM23|ENSG00000
)113595			C|bidirectional_gene_fusion|HIGH|TRAPPC13&TRIM23|ENSG00000
)113595			C|bidirectional_gene_fusion|HIGH|TRAPPC13&TRIM23|ENSG00000
)113595			C|bidirectional_gene_fusion|HIGH|TRAPPC13&TRIM23|ENSG00000
protein_coding			C|structural_interaction_variant|HIGH|CD180|ENSG00000134061|i
protein_coding			T|structural_interaction_variant|HIGH|PIK3R1|ENSG00000145675|i
protein_co	9	0.11	TC|splice_acceptor_variant&intron_variant|HIGH|SERF1A|ENSG000
protein_coding			T|structural_interaction_variant|HIGH|TNPO1|ENSG00000083312|i
protein_co	4	0.25	A|stop_gained|HIGH|ANKDD1B|ENSG00000189045|transcript|ENS
protein_co	4	0.25	A|stop_gained|HIGH|ANKDD1B|ENSG00000189045|transcript|ENS
protein_co	2	1	TC|frameshift_variant|HIGH|AC113404.1|ENSG00000254893|trans
protein_co	2	1	CA|frameshift_variant|HIGH|AC113404.1|ENSG00000254893|trans
protein_co	2	1	GTA|frameshift_variant|HIGH|AC113404.1|ENSG00000254893|trar
protein_coding			A|structural_interaction_variant|HIGH|SV2C|ENSG00000122012|in
protein_co	28	0.04	T|stop_gained|HIGH|WDR41|ENSG00000164253|transcript|ENST0
protein_coding			GC|frameshift_variant|HIGH|WDR41|ENSG00000164253|transcript
protein_coding			T|structural_interaction_variant|HIGH|ARSB|ENSG00000113273|int
protein_coding			T|structural_interaction_variant|HIGH|BHMT|ENSG00000145692|ir
antisense			G|splice_donor_variant&intron_variant|HIGH|CTC-431G16.2|ENSG
processed_transcript			T|splice_donor_variant&intron_variant|HIGH|THBS4|ENSG0000011
processed_pseudogene			GTCCCTC|splice_acceptor_variant&intron_variant|HIGH|CTC-512J14
processed_pseudogene			GTCCCTCTCCCTC|splice_acceptor_variant&intron_variant|HIGH|CTC
nonsense_	5	0.2	A|splice_acceptor_variant&intron_variant|HIGH|RASGRF2|ENSG000
protein_coding			T|structural_interaction_variant|HIGH|ACOT12|ENSG00000172497
protein_co	8	0.25	A|splice_acceptor_variant&intron_variant|HIGH|RPS23|ENSG00000
processed_pseudogene			T|splice_acceptor_variant&intron_variant|HIGH|ST13P12|ENSG000
protein_co	6	0.33	A|splice_acceptor_variant&intron_variant|HIGH|XRCC4|ENSG00000
processed_pseudogene			C|splice_acceptor_variant&splice_region_variant&intron_variant&n
lincRNA			A|splice_acceptor_variant&intron_variant|HIGH|CTC-493L21.1|ENS
antisense			GT|splice_acceptor_variant&intron_variant|HIGH|TMEM161B-AS1|
protein_co	11	0.18	TC|splice_acceptor_variant&intron_variant|HIGH|ADGRV1|ENSG00
lincRNA			G|splice_donor_variant&intron_variant|HIGH|CTC-458G6.2|ENSG0
protein_coding			CG|frameshift_variant|HIGH|POU5F2|ENSG00000248483|transcrip
protein_co	9	0.33	T|stop_gained|HIGH|FAM81B|ENSG00000153347|transcript|ENST0
protein_co	16	0.06	T|splice_acceptor_variant&intron_variant|HIGH|RHOBTB3|ENSG000
protein_coding			T|structural_interaction_variant|HIGH|ERAP1|ENSG00000164307|ir
protein_coding			C|structural_interaction_variant|HIGH|ERAP1|ENSG00000164307|i
protein_coding			T|structural_interaction_variant|HIGH|ERAP2|ENSG00000164308|ir
protein_coding			T|structural_interaction_variant|HIGH|ERAP2|ENSG00000164308|ir
protein_coding			T|structural_interaction_variant|HIGH|ERAP2|ENSG00000164308|ir
lincRNA			A|splice_acceptor_variant&intron_variant|HIGH|CTD-2215E18.1|EN

lincRNA			A|splice_acceptor_variant&splice_region_variant&intron_variant|HI
unprocessed_pseudogene			A|splice_donor_variant&intron_variant|HIGH|RP11-346J10.2|ENSG
0247402			C|gene_fusion|HIGH|CTD-2340E1.2&LINC00492|ENSG0000024740
0247402			C|gene_fusion|HIGH|CTD-2340E1.2&LINC00492|ENSG0000024740
0247402			C|gene_fusion|HIGH|CTD-2340E1.2&LINC00492|ENSG0000024740
0247402			C|gene_fusion|HIGH|CTD-2340E1.2&LINC00492|ENSG0000024740
processed_transcript			A|splice_donor_variant&intron_variant|HIGH|PJA2|ENSG0000019
processed_pseudogene			AT|splice_acceptor_variant&intron_variant|HIGH|CTD-2128F4.1|EN
processed_pseudogene			ATT|splice_acceptor_variant&intron_variant|HIGH|CTD-2128F4.1|E
processed_pseudogene			ATTT|splice_acceptor_variant&intron_variant|HIGH|CTD-2128F4.1|
processed_transcript			T|splice_acceptor_variant&intron_variant|HIGH|EPB41L4A|ENSG00
protein_co	11	0.18	CTAAAA|splice_acceptor_variant&intron_variant|HIGH|EPB41L4A|E
protein_coding			C|structural_interaction_variant|HIGH|APC|ENSG00000134982|inte
protein_coding			A|structural_interaction_variant|HIGH|APC|ENSG00000134982|inte
protein_coding			A|protein_protein_contact|HIGH|APC|ENSG00000134982|interacti
nonsense_	4	0.25	C|splice_acceptor_variant&intron_variant|HIGH|CTC-487M23.8|EN
protein_coding			G|frameshift_variant&stop_lost|HIGH|MCC|ENSG00000171444|tra
nonsense_	13	0.15	T|splice_donor_variant&intron_variant|HIGH|ATG12|ENSG0000014
protein_coding			A|stop_gained|HIGH|AC026449.1|ENSG00000280329|transcript|EN
protein_co	10	0.2	TATATATATATATATGGAAC|frameshift_variant|HIGH|LVRN|ENSG000
protein_co	7	1	CTT|splice_acceptor_variant&intron_variant|HIGH|TNFAIP8|ENSG0
protein_co	7	1	CTT|splice_acceptor_variant&intron_variant|HIGH|TNFAIP8|ENSG0
protein_coding			A|structural_interaction_variant|HIGH|HSD17B4|ENSG0000013383
protein_co	3	0.33	A|frameshift_variant|HIGH|ZNF474|ENSG00000164185|transcript|
protein_coding			C|structural_interaction_variant|HIGH|PPIC|ENSG00000168938|int
protein_coding			C|structural_interaction_variant|HIGH|PPIC|ENSG00000168938|int
protein_co	2	0.5	T|frameshift_variant|HIGH|PPIC|ENSG00000168938|transcript|ENS
0223652			T|bidirectional_gene_fusion|HIGH|AC106786.1&PRDM6|ENSG0000
0223652			T|bidirectional_gene_fusion|HIGH|AC106786.1&PRDM6|ENSG0000
lincRNA			C|splice_donor_variant&intron_variant|HIGH|CTD-2308B18.4|ENSG
antisense			T|splice_acceptor_variant&intron_variant|HIGH|RP11-517I3.1|ENS
protein_coding			GA|frameshift_variant|HIGH|ALDH7A1|ENSG00000164904|transcri
sense_intronic			CTT|splice_acceptor_variant&intron_variant|HIGH|CTC-573M9.1|EN
sense_intronic			CTT|splice_acceptor_variant&intron_variant|HIGH|CTC-573M9.1|EN
sense_intronic			CTT|splice_acceptor_variant&intron_variant|HIGH|CTC-573M9.1|EN
sense_intronic			CTT|splice_acceptor_variant&intron_variant|HIGH|CTC-573M9.1|EN
nonsense_	10	0.3	C|splice_donor_variant&intron_variant|HIGH|HINT1|ENSG0000016
protein_co	21	0.1	CTG|frameshift_variant&splice_region_variant|HIGH|ACSL6|ENSG0
processed_pseudogene			C|splice_donor_variant&intron_variant|HIGH|AC063976.7|ENSG000
protein_co	7	0.14	GAACCTTGCTTCTGAAAGGGAGACTGCTGT|frameshift_variant|HIGH|
protein_co	6	0.17	C|frameshift_variant|HIGH|PITX1|ENSG00000069011|transcript|EN
0277619			C|gene_fusion|HIGH|C5orf66&CTC-276P9.4|ENSG00000224186&EN
0277619			C|gene_fusion|HIGH|C5orf66&CTC-276P9.4|ENSG00000224186&EN
0277619			C|gene_fusion|HIGH|C5orf66&CTC-276P9.4|ENSG00000224186&EN
lincRNA			G|splice_donor_variant&intron_variant|HIGH|C5orf66-AS2|ENSG00
lincRNA			T|splice_donor_variant&intron_variant|HIGH|C5orf66-AS2|ENSG00
protein_coding			T|structural_interaction_variant|HIGH|H2AFY|ENSG00000113648|i

protein_coding			A|stop_gained|HIGH|DCANP1|ENSG00000251380|transcript|ENST(
protein_co	7	0.43	G|splice_acceptor_variant&intron_variant|HIGH|SLC25A48|ENSG00
protein_coding			C|structural_interaction_variant|HIGH|TGFBI|ENSG00000120708|ir
protein_co	12	0.33	AT|splice_acceptor_variant&intron_variant|HIGH|SMAD5|ENSG000
protein_co	12	0.33	AT|splice_acceptor_variant&intron_variant|HIGH|SMAD5|ENSG000
retained_intron			G|splice_donor_variant&intron_variant|HIGH|KLHL3|ENSG0000014
protein_coding			T|structural_interaction_variant|HIGH|KLHL3|ENSG00000146021|ir
processed_pseudogene			C|splice_acceptor_variant&splice_region_variant&intron_variant|HI
processed_pseudogene			C|splice_acceptor_variant&splice_region_variant&intron_variant|HI
processed_pseudogene			C|splice_acceptor_variant&splice_region_variant&intron_variant|HI
processed_pseudogene			C|splice_acceptor_variant&splice_region_variant&intron_variant|HI
processed_pseudogene			C|splice_acceptor_variant&splice_region_variant&intron_variant|HI
nonsense_	13	0.08	A|frameshift_variant|HIGH|HSPA9|ENSG00000113013|transcript|E
nonsense_	13	0.08	A|frameshift_variant|HIGH|HSPA9|ENSG00000113013|transcript|E
nonsense_	13	0.08	A|frameshift_variant|HIGH|HSPA9|ENSG00000113013|transcript|E
protein_coding			C|structural_interaction_variant|HIGH|HSPA9|ENSG00000113013|i
nonsense_mediated_decay			A|stop_gained|HIGH|HSPA9|ENSG00000113013|transcript|ENST00
processed_pseudogene			C|splice_acceptor_variant&splice_region_variant&intron_variant&n
processed_pseudogene			C|splice_acceptor_variant&splice_region_variant&intron_variant&n
processed_pseudogene			C|splice_acceptor_variant&splice_region_variant&intron_variant&n
protein_co	7	0.14	T|stop_gained|HIGH|SLC23A1|ENSG00000170482|transcript|ENST(
protein_co	6	0.17	AGT|frameshift_variant|HIGH|SRA1|ENSG00000213523|transcript|
protein_co	6	0.17	CG|frameshift_variant|HIGH|SRA1|ENSG00000213523|transcript|E
processed_pseudogene			T|splice_donor_variant&intron_variant|HIGH|RP11-515C16.7|ENSG
protein_co	4	1	AAAGACACC|frameshift_variant|HIGH|PCDHA4|ENSG00000204967
protein_co	4	1	G|frameshift_variant|HIGH|PCDHA4|ENSG00000204967|transcript|
protein_co	4	1	A|frameshift_variant|HIGH|PCDHA4|ENSG00000204967|transcript|
lincRNA			A|splice_acceptor_variant&intron_variant|HIGH|CH17-140K24.1|EN
protein_co	2	0.5	C|frameshift_variant|HIGH|PCDHB3|ENSG00000113205|transcript|
protein_co	1	1	TCC|frameshift_variant|HIGH|PCDHB8|ENSG00000120322|transcri
protein_co	1	1	G|frameshift_variant|HIGH|PCDHB8|ENSG00000120322|transcript|
antisense			C|splice_acceptor_variant&intron_variant|HIGH|CH17-140K24.6|EN
protein_co	1	1	GT|frameshift_variant&stop_gained|HIGH|SLC25A2|ENSG0000012C
protein_co	2	1	G|frameshift_variant|HIGH|PCDHGA8|ENSG00000253767|transcrip
protein_coding			T|frameshift_variant|HIGH|PCDHGA10|ENSG00000253846|transcri
protein_coding			T|stop_gained|HIGH|PCDHGA10|ENSG00000253846|transcript|ENS
protein_coding			C|frameshift_variant&stop_lost&splice_region_variant|HIGH|PCDH(
unprocessed_pseudogene			A|splice_acceptor_variant&intron_variant|HIGH|PCDHGB9P|ENSG0
protein_coding			A|structural_interaction_variant|HIGH|GNPDA1|ENSG00000113552
lincRNA			A|splice_donor_variant&intron_variant|HIGH|CTD-2050E21.2|ENSG
lincRNA			T|splice_donor_variant&intron_variant|HIGH|CTB-1H10.1|ENSG000
protein_coding			G|structural_interaction_variant|HIGH|DPYSL3|ENSG00000113657|
protein_coding			C|structural_interaction_variant|HIGH|DPYSL3|ENSG00000113657|
protein_co	13	0.77	T|frameshift_variant|HIGH|HTR4|ENSG00000164270|transcript|EN
nonsense_mediated_decay			T|stop_lost|HIGH|SH3TC2|ENSG00000169247|transcript|ENST0000
protein_co	12	0.08	G|splice_acceptor_variant&intron_variant|HIGH|ABLIM3|ENSG0000(
protein_co	12	0.08	T|frameshift_variant|HIGH|CSNK1A1|ENSG00000113712|transcript

protein_co	12	0.08	T|frameshift_variant|HIGH|CSNK1A1|ENSG00000113712|transcript
protein_co	3	0.67	C|frameshift_variant|HIGH|TIGD6|ENSG00000164296|transcript|EI
protein_coding			A|structural_interaction_variant|HIGH|CSF1R|ENSG00000182578|ii
protein_coding			C|structural_interaction_variant|HIGH|CSF1R|ENSG00000182578|ii
protein_coding			C|structural_interaction_variant|HIGH|CSF1R|ENSG00000182578|ii
protein_coding			A|structural_interaction_variant|HIGH|CSF1R|ENSG00000182578|ii
unprocessed_pseudogene			T|splice_donor_variant&intron_variant|HIGH|CTB-23I7.1|ENSG0000
protein_co	2	0.5	C|splice_donor_variant&intron_variant|HIGH|FAT2|ENSG00000086
protein_co	7	0.14	G|splice_donor_variant&intron_variant|HIGH|ATOX1|ENSG0000017
protein_coding			TC|frameshift_variant|HIGH|GRIA1|ENSG00000155511|transcript|E
protein_coding			A|structural_interaction_variant|HIGH|GALNT10|ENSG0000016457
protein_co	15	0.07	C|splice_acceptor_variant&intron_variant|HIGH|LARP1|ENSG00000
protein_co	4	0.25	G|splice_acceptor_variant&intron_variant|HIGH|SGCD|ENSG000001
protein_co	24	0.04	A|splice_donor_variant&intron_variant|HIGH|CYFIP2|ENSG0000005
antisense			A|splice_donor_variant&intron_variant|HIGH|CTB-47B11.3|ENSG00
)248544			C|gene_fusion|HIGH|CTB-109A12.1&CTB-47B11.3|ENSG000002514
)248544			C|gene_fusion|HIGH|CTB-109A12.1&CTB-47B11.3|ENSG000002514
)248544			C|gene_fusion|HIGH|CTB-109A12.1&CTB-47B11.3|ENSG000002514
)248544			C|gene_fusion|HIGH|CTB-109A12.1&CTB-47B11.3|ENSG000002514
)248544			C|gene_fusion|HIGH|CTB-109A12.1&CTB-47B11.3|ENSG000002514
protein_co	10	0.1	T|splice_acceptor_variant&intron_variant|HIGH|CLINT1|ENSG00000
protein_coding			G|structural_interaction_variant|HIGH|EBF1|ENSG00000164330|in
protein_co	12	0.08	CGG|splice_acceptor_variant&intron_variant|HIGH|RNF145|ENSG0
lincRNA			T|splice_donor_variant&intron_variant|HIGH|AC008703.1|ENSG000
antisense			CTTT|splice_acceptor_variant&intron_variant|HIGH|AC008697.1|EN
antisense			CTTT|splice_acceptor_variant&intron_variant|HIGH|AC008697.1|EN
antisense			CTTT|splice_acceptor_variant&intron_variant|HIGH|AC008697.1|EN
antisense			CT|splice_acceptor_variant&intron_variant|HIGH|CTC-340A15.2|EN
protein_co	17	0.06	A|splice_donor_variant&intron_variant|HIGH|DOCK2|ENSG0000013
protein_coding			T|structural_interaction_variant|HIGH|DOCK2|ENSG00000134516|i
nonsense_	10	0.1	G|splice_acceptor_variant&intron_variant|HIGH|KCNIP1|ENSG0000
protein_coding			T|structural_interaction_variant|HIGH|STK10|ENSG00000072786|ir
protein_co	14	0.07	T|frameshift_variant|HIGH|ERGIC1|ENSG00000113719|transcript|E
protein_co	14	0.07	GT|frameshift_variant&stop_gained|HIGH|ERGIC1|ENSG000001137
antisense			CA|splice_donor_variant&intron_variant|HIGH|CTC-308K20.1|ENSG
protein_co	5	0.4	ATT|splice_acceptor_variant&intron_variant|HIGH|BNIP1|ENSG000
protein_co	5	0.4	ATT|splice_acceptor_variant&intron_variant|HIGH|BNIP1|ENSG000
protein_co	7	0.43	CTGAA|splice_acceptor_variant&intron_variant|HIGH|BOD1|ENSG0
protein_co	7	0.43	CTGAA|splice_acceptor_variant&intron_variant|HIGH|BOD1|ENSG0
protein_co	7	0.43	CCACACTCTGCCTCAAACCAT|splice_acceptor_variant&intron_variant
lincRNA			C|splice_acceptor_variant&splice_region_variant&intron_variant|HI
lincRNA			C|splice_acceptor_variant&splice_region_variant&intron_variant|HI
lincRNA			C|splice_acceptor_variant&splice_region_variant&intron_variant|HI
protein_co	12	0.08	CTT|splice_acceptor_variant&intron_variant|HIGH|SFXN1|ENSG000
protein_co	12	0.08	CTT|splice_acceptor_variant&intron_variant|HIGH|SFXN1|ENSG000
protein_coding			A|stop_gained|HIGH|RNF44|ENSG00000146083|transcript|ENST00
protein_co	1	1	T|frameshift_variant|HIGH|GPRIN1|ENSG00000169258|transcript|I

protein_co	1	1	T|frameshift_variant|HIGH|GPRIN1|ENSG00000169258|transcript|I
protein_coding			C|splice_donor_variant&splice_region_variant&5_prime_UTR_varia
protein_coding			T|structural_interaction_variant|HIGH|B4GALT7|ENSG0000027847
processed_transcript			A|splice_donor_variant&intron_variant|HIGH|FAM153A|ENSG0000
protein_co	5	0.2	C|splice_acceptor_variant&intron_variant|HIGH|ZFP2|ENSG000001
protein_co	19	0.05	CTT|splice_acceptor_variant&intron_variant|HIGH|CANX|ENSG0000
protein_co	19	0.05	CTT|splice_acceptor_variant&intron_variant|HIGH|CANX|ENSG0000
protein_co	19	0.05	CTT|splice_acceptor_variant&intron_variant|HIGH|CANX|ENSG0000
protein_co	19	0.05	C|splice_donor_variant&intron_variant|HIGH|CANX|ENSG0000012
protein_coding			T|structural_interaction_variant|HIGH|SQSTM1|ENSG00000161011
protein_coding			A|structural_interaction_variant|HIGH|FLT4|ENSG00000037280|int
protein_co	7	0.57	G|start_lost|HIGH|ZFP62|ENSG00000196670|transcript|ENST0000
protein_co	1	1	T|frameshift_variant|HIGH|OR2V2|ENSG00000182613|transcript|E
antisense			A|splice_donor_variant&intron_variant|HIGH|CTC-338M12.4|ENSG
protein_co	6	0.17	A|frameshift_variant|HIGH|TRIM52|ENSG00000183718|transcript|
processed_transcript			T|splice_donor_variant&intron_variant|HIGH|GMDS|ENSG0000011
lincRNA			A|splice_acceptor_variant&intron_variant|HIGH|GMDS-AS1|ENSG0
lincRNA			ATT|splice_acceptor_variant&intron_variant|HIGH|RP1-40E16.2|EN
lincRNA			A|splice_region_variant&intron_variant|LOW|RP1-40E16.2|ENSG00
protein_coding			C|structural_interaction_variant|HIGH|RIPK1|ENSG00000137275|ir
protein_co	10	0.1	A|stop_gained|HIGH|PSMG4|ENSG00000180822|transcript|ENST0
processed_pseudogene			A|splice_acceptor_variant&splice_region_variant&intron_variant|HI
protein_co	3	0.67	CA|frameshift_variant&start_lost|HIGH|DSP|ENSG00000096696|tra
protein_coding			T|structural_interaction_variant|HIGH|DSP|ENSG00000096696|inte
protein_coding			A|structural_interaction_variant|HIGH|DSP|ENSG00000096696|inte
protein_coding			T|structural_interaction_variant|HIGH|TXNDC5|ENSG00000239264
protein_coding			A|stop_gained|HIGH|EEF1E1|ENSG00000124802|transcript|ENST0
nonsense_	18	0.06	C|splice_acceptor_variant&intron_variant|HIGH|OFCC1|ENSG00000
lincRNA			G|splice_acceptor_variant&intron_variant|HIGH|RP11-716O23.2|EN
lincRNA			G|splice_acceptor_variant&splice_region_variant&intron_variant&n
protein_co	10	0.1	ACCCCTTCCCTTCCTCT|frameshift_variant|HIGH|PHACTR1|ENSG000
processed_transcript			G|splice_donor_variant&intron_variant|HIGH|TBC1D7|ENSG000001
protein_coding			T|structural_interaction_variant|HIGH|SIRT5|ENSG00000124523|in
protein_coding			C|structural_interaction_variant|HIGH|TPMT|ENSG00000137364|ir
unprocessed_pseudogene			TTTTTG|splice_acceptor_variant&intron_variant|HIGH|RP11-528A1
antisense			G|splice_donor_variant&intron_variant|HIGH|RP4-625H18.2|ENSG
antisense			C|splice_acceptor_variant&intron_variant|HIGH|RP4-625H18.2|ENS
antisense			C|splice_donor_variant&intron_variant|HIGH|RP11-239H6.2|ENSG
lincRNA			AT|splice_acceptor_variant&intron_variant|HIGH|CASC15|ENSG000
processed_transcript			A|splice_donor_variant&splice_region_variant&intron_variant&non_
protein_co	6	0.17	TACACACACACACACACACACACACACAC|splice_acceptor_variant&int
protein_co	6	0.17	TACACACACACACACACACACACACACAC|splice_acceptor_variant&int
protein_co	6	0.17	TACACACACACACACACACACACACACAC|splice_acceptor_variant&int
protein_co	6	0.17	TACACACACACACACACACACACACACAC|splice_acceptor_variant&int
protein_co	6	0.17	TACACACACACACACACACACACACACAC|splice_acceptor_variant&int
protein_co	6	0.17	TACACACACACACACACACACACACACAC|splice_acceptor_variant&int
protein_coding			G|stop_lost|HIGH|C6orf229|ENSG00000260286|transcript|ENST00

protein_co	1	1	T|splice_donor_variant&intron_variant|HIGH|RP3-369A17.6|ENSG0
protein_co	3	0.67	T|stop_gained|HIGH|SLC17A4|ENSG00000146039|transcript|ENST(
protein_co	1	1	T|stop_gained|HIGH|TRIM38|ENSG00000112343|transcript|ENST0
protein_co	19	0.47	A|splice_donor_variant&intron_variant|HIGH|BTN3A2|ENSG000001
protein_co	19	0.37	G|splice_donor_variant&intron_variant|HIGH|BTN3A2|ENSG000001
protein_co	15	0.2	A|stop_gained|HIGH|BTN3A3|ENSG00000111801|transcript|ENST0
protein_co	7	0.57	A|start_lost|HIGH|BTN2A1|ENSG00000112763|transcript|ENST000
protein_co	1	1	GA|frameshift_variant|HIGH|HIST1H4K|ENSG00000273542|transcri
protein_co	8	0.13	T|stop_gained|HIGH|ZSCAN9|ENSG00000137185|transcript|ENST0
protein_co	3	0.33	A|splice_donor_variant&intron_variant|HIGH|ZKSCAN3|ENSG00000
protein_coding			A|frameshift_variant|HIGH|ZSCAN12|ENSG00000158691|transcript
protein_co	9	0.11	TC|frameshift_variant|HIGH|PSORS1C1|ENSG00000204540|transcr
protein_co	1	1	AGT|frameshift_variant|HIGH|HLA-DRB5|ENSG00000198502|transc
protein_coding			GT|frameshift_variant|HIGH|HLA-DRB5|ENSG00000198502|transcr
protein_coding			G|conservative_inframe_deletion|MODERATE|HLA-DRB5|ENSG0000
protein_coding			TGC|frameshift_variant|HIGH|HLA-DRB5|ENSG00000198502|transc
protein_coding			TGC|frameshift_variant|HIGH|HLA-DRB5|ENSG00000198502|transc
protein_coding			TGC|frameshift_variant|HIGH|HLA-DRB5|ENSG00000198502|transc
nonsense_mediated_decay			C|frameshift_variant|HIGH|SYNGAP1|ENSG00000197283|transcript
protein_coding			T|structural_interaction_variant|HIGH|BAK1|ENSG00000030110|in
protein_co	3	0.33	C|splice_acceptor_variant&intron_variant|HIGH|IP6K3|ENSG000001
nonsense_	28	0.36	T|frameshift_variant|HIGH|TCP11|ENSG00000124678|transcript|EI
protein_co	13	0.08	C|stop_gained|HIGH|SRPK1|ENSG00000096063|transcript|ENST00
processed_pseudogene			T|splice_acceptor_variant&splice_region_variant&intron_variant&n
nonsense_	12	0.08	A|stop_gained|HIGH|BRPF3|ENSG00000096070|transcript|ENST00
protein_co	5	0.2	A|stop_gained|HIGH|PNPLA1|ENSG00000180316|transcript|ENST0
protein_co	10	0.1	CTTTTTTTT|splice_acceptor_variant&intron_variant|HIGH|KCTD20|I
protein_co	10	0.1	CTTTTTTTT|splice_acceptor_variant&intron_variant|HIGH|KCTD20|I
protein_co	10	0.1	CTTTTTTTT|splice_acceptor_variant&intron_variant|HIGH|KCTD20|I
protein_co	10	0.1	CTTTTTTTT|splice_acceptor_variant&intron_variant|HIGH|KCTD20|I
protein_co	8	0.38	CTTT|splice_acceptor_variant&intron_variant|HIGH|SRSF3|ENSG000
protein_co	8	0.38	CTTT|splice_acceptor_variant&intron_variant|HIGH|SRSF3|ENSG000
protein_co	8	0.38	CTTT|splice_acceptor_variant&intron_variant|HIGH|SRSF3|ENSG000
protein_co	8	0.38	CTTT|splice_acceptor_variant&intron_variant|HIGH|SRSF3|ENSG000
protein_co	8	0.38	CTTT|splice_acceptor_variant&intron_variant|HIGH|SRSF3|ENSG000
protein_coding			T|structural_interaction_variant|HIGH|PPIL1|ENSG00000137168|in
protein_coding			G|structural_interaction_variant|HIGH|PPIL1|ENSG00000137168|in
protein_coding			A|frameshift_variant|HIGH|MTCH1|ENSG00000137409|transcript|I
protein_coding			A|structural_interaction_variant|HIGH|RNF8|ENSG00000112130|in
protein_coding			A|stop_gained|HIGH|KCNK16|ENSG00000095981|transcript|ENST0
protein_co	3	0.67	A|splice_donor_variant&intron_variant|HIGH|TSPO2|ENSG0000011
protein_coding			C|structural_interaction_variant|HIGH|APOBEC2|ENSG0000012470
protein_coding			T|stop_gained|HIGH|OARD1|ENSG00000124596|transcript|ENST00
processed_transcript			G|splice_acceptor_variant&intron_variant|HIGH|ADCY10P1|ENSG00
protein_coding			A|structural_interaction_variant|HIGH|TREM1|ENSG00000124731|
protein_co	4	0.25	T|splice_donor_variant&intron_variant|HIGH|PGC|ENSG000000960
protein_co	4	0.25	GT|frameshift_variant|HIGH|PGC|ENSG00000096088|transcript|EN

protein_coding			CCCCCA|frameshift_variant|HIGH|USP49|ENSG00000164663|transc
protein_coding			T|stop_gained|HIGH|C6orf132|ENSG00000188112|transcript|ENST
protein_coding			A|structural_interaction_variant|HIGH|UBR2|ENSG00000024048|in
protein_co	3	0.67	T|splice_acceptor_variant&intron_variant|HIGH|UBR2|ENSG00000C
protein_co	3	0.67	T|splice_acceptor_variant&intron_variant|HIGH|UBR2|ENSG00000C
protein_co	1	1	T|start_lost|HIGH|C6orf226|ENSG00000221821|transcript|ENST00
protein_coding			A|structural_interaction_variant|HIGH|DNPH1|ENSG00000112667|
protein_co	5	0.2	AT|splice_acceptor_variant&intron_variant|HIGH|CAPN11|ENSG000
protein_co	5	0.2	TGGCTGCC|frameshift_variant|HIGH|CAPN11|ENSG00000137225|t
protein_coding			G|structural_interaction_variant|HIGH|ENPP4|ENSG00000001561|i
protein_coding			G|structural_interaction_variant|HIGH|ENPP4|ENSG00000001561|i
protein_coding			T|structural_interaction_variant|HIGH|PLA2G7|ENSG00000146070|
antisense			G|splice_acceptor_variant&intron_variant|HIGH|RP3-365O12.2|ENS
protein_co	39	0.08	ATTT|splice_acceptor_variant&intron_variant|HIGH|EFHC1|ENSG00
protein_co	39	0.08	ATTT|splice_acceptor_variant&intron_variant|HIGH|EFHC1|ENSG00
protein_co	39	0.08	ATTT|splice_acceptor_variant&intron_variant|HIGH|EFHC1|ENSG00
protein_co	39	0.08	ATTT|splice_acceptor_variant&intron_variant|HIGH|EFHC1|ENSG00
protein_coding			C|structural_interaction_variant|HIGH|GSTA1|ENSG00000243955|i
protein_coding			C|structural_interaction_variant|HIGH|GSTA3|ENSG00000174156|i
protein_coding			A|stop_gained|HIGH|ELOVL5|ENSG00000012660|transcript|ENST0
protein_co	7	0.43	CT|splice_acceptor_variant&intron_variant|HIGH|PRIM2|ENSG0000
protein_coding			T|protein_protein_contact|HIGH|PRIM2|ENSG00000146143|intera
unprocessed_pseudogene			T|splice_acceptor_variant&intron_variant|HIGH|RP11-448N11.2|EN
protein_co	5	0.2	A|stop_gained|HIGH|ADGRB3|ENSG00000135298|transcript|ENST0
protein_coding			A|frameshift_variant|HIGH|SMAP1|ENSG00000112305|transcript|E
protein_coding			T|stop_lost|HIGH|SMAP1|ENSG00000112305|transcript|ENST0000
antisense			T|splice_donor_variant&intron_variant|HIGH|RP11-154D6.1|ENSG0
processed_transcript			A|splice_donor_variant&intron_variant|HIGH|RIMS1|ENSG0000007
protein_coding			G|splice_acceptor_variant&splice_region_variant&5_prime_UTR_va
protein_co	7	0.14	A|stop_gained|HIGH|KHDC1|ENSG00000135314|transcript|ENST00
protein_coding			T|structural_interaction_variant|HIGH|MB21D1|ENSG0000016443
processed_transcript			CAAA|splice_donor_variant&intron_variant|HIGH|SENP6|ENSG0000
processed_transcript			CAAA|splice_donor_variant&intron_variant|HIGH|SENP6|ENSG0000
protein_co	4	0.25	A|frameshift_variant|HIGH|IMPG1|ENSG00000112706|transcript|E
nonsense_	4	0.25	C|frameshift_variant|HIGH|IRAK1BP1|ENSG00000146243|transcrip
sense_intronic			G|splice_donor_variant&intron_variant|HIGH|RP5-991C6.4|ENSG00
protein_coding			T|structural_interaction_variant|HIGH|TPBG|ENSG00000146242|in
protein_coding			T|structural_interaction_variant|HIGH|ME1|ENSG00000065833|int
unprocessed_pseudogene			T|splice_donor_variant&intron_variant|HIGH|RP3-455E7.1|ENSG00
protein_coding			C|structural_interaction_variant|HIGH|NT5E|ENSG00000135318|in
protein_co	6	0.17	A|splice_donor_variant&intron_variant|HIGH|CGA|ENSG00000135
retained_intron			A|splice_acceptor_variant&intron_variant|HIGH|ORC3|ENSG00000
protein_co	7	0.29	T|splice_acceptor_variant&intron_variant|HIGH|GABRR1|ENSG0000
protein_co	6	0.17	T|frameshift_variant|HIGH|CASP8AP2|ENSG00000118412|transcrip
protein_co	6	0.17	T|frameshift_variant|HIGH|CASP8AP2|ENSG00000118412|transcrip
protein_coding			C|structural_interaction_variant|HIGH|BACH2|ENSG00000112182|i
lincRNA			T|splice_donor_variant&intron_variant|HIGH|CASC6|ENSG0000022

protein_coding			GTATA|frameshift_variant|HIGH|MANEA|ENSG00000172469|transc
protein_coding			ATATATATG|frameshift_variant|HIGH|MANEA|ENSG00000172469|t
protein_coding			ATATATATG|frameshift_variant|HIGH|MANEA|ENSG00000172469|t
protein_coding			ATATATATG|frameshift_variant|HIGH|MANEA|ENSG00000172469|t
protein_coding			ATATATATG|frameshift_variant|HIGH|MANEA|ENSG00000172469|t
protein_coding			C|frameshift_variant|HIGH|MANEA|ENSG00000172469|transcript|
protein_co	2	0.5	CTT|splice_acceptor_variant&intron_variant|HIGH|UFL1|ENSG0000
protein_co	2	0.5	C|splice_region_variant&intron_variant|LOW|UFL1|ENSG00000014
processed_transcript			T|splice_donor_variant&intron_variant|HIGH|NDUFAF4|ENSG0000C
lincRNA			A|splice_donor_variant&intron_variant|HIGH|RP11-436D23.1|ENSG
protein_coding			T|frameshift_variant|HIGH|CCNC|ENSG00000112237|transcript|EN
protein_coding			G|structural_interaction_variant|HIGH|RTN4IP1|ENSG00000130347
protein_coding			C|splice_acceptor_variant&conservative_inframe_deletion&splice_r
protein_co	5	0.2	C|start_lost|HIGH|ARMC2|ENSG00000118690|transcript|ENST0000
protein_co	5	0.2	C|splice_acceptor_variant&intron_variant|HIGH|ARMC2|ENSG0000
protein_co	24	0.17	ATT|splice_acceptor_variant&intron_variant|HIGH|CEP57L1|ENSG0
protein_co	24	0.17	ATT|splice_acceptor_variant&intron_variant|HIGH|CEP57L1|ENSG0
protein_co	24	0.08	T|splice_acceptor_variant&intron_variant|HIGH|CEP57L1|ENSG000
unitary_pseudogene			A|splice_acceptor_variant&intron_variant|HIGH|CCDC162P|ENSG0C
protein_co	3	0.33	T|frameshift_variant|HIGH|SMPD2|ENSG00000135587|transcript|E
protein_co	12	0.08	A|frameshift_variant|HIGH|AK9|ENSG00000155085|transcript|ENS
protein_co	12	0.08	A|frameshift_variant|HIGH|AK9|ENSG00000155085|transcript|ENS
protein_co	12	0.08	A|frameshift_variant|HIGH|AK9|ENSG00000155085|transcript|ENS
protein_co	5	0.6	GT|splice_acceptor_variant&intron_variant|HIGH|FIG4|ENSG00000
protein_co	5	0.6	GT|splice_acceptor_variant&intron_variant|HIGH|FIG4|ENSG00000
protein_coding			A|protein_protein_contact|HIGH|WASF1|ENSG00000112290|intera
protein_co	8	0.63	TC|splice_acceptor_variant&intron_variant|HIGH|AMD1|ENSG0000
protein_co	8	0.63	TC|splice_acceptor_variant&intron_variant|HIGH|AMD1|ENSG0000
protein_co	4	1	CTTTT|splice_acceptor_variant&intron_variant|HIGH|RPF2|ENSG00
protein_co	4	1	CTTTT|splice_acceptor_variant&intron_variant|HIGH|RPF2|ENSG00
protein_coding			A|stop_gained|HIGH|MFSD4B|ENSG00000173214|transcript|ENST(
antisense			A|splice_acceptor_variant&intron_variant|HIGH|RP3-399L15.3|ENS
lincRNA			T|splice_donor_variant&intron_variant|HIGH|RP3-399L15.2|ENSG0
protein_coding			G|structural_interaction_variant|HIGH|COL10A1|ENSG0000012350
protein_co	3	0.33	G|splice_donor_variant&intron_variant|HIGH|FAM26F|ENSG00000
protein_co	6	0.33	CTT|splice_acceptor_variant&intron_variant|HIGH|RWDD1|ENSG0C
protein_co	6	0.33	CTT|splice_acceptor_variant&intron_variant|HIGH|RWDD1|ENSG0C
protein_co	3	1	AGG|frameshift_variant|HIGH|GPRC6A|ENSG00000173612|transcri
protein_co	3	1	TA|frameshift_variant|HIGH|GPRC6A|ENSG00000173612|transcript
protein_co	3	1	A|stop_gained|HIGH|GPRC6A|ENSG00000173612|transcript|ENST0
protein_coding			A|structural_interaction_variant|HIGH|ROS1|ENSG00000047936|in
protein_coding			G|structural_interaction_variant|HIGH|ROS1|ENSG00000047936|in
protein_co	9	0.11	C|splice_donor_variant&splice_region_variant&intron_variant|HIGH
processed_transcript			G|splice_donor_variant&intron_variant|HIGH|PKIB|ENSG0000013[illegible]
antisense			A|splice_donor_variant&intron_variant|HIGH|RP11-532N4.2|ENSG(
lincRNA			C|splice_acceptor_variant&intron_variant|HIGH|RP11-138M12.1|EI
lincRNA			T|splice_acceptor_variant&intron_variant|HIGH|RP1-293L8.2|ENSG

protein_coding			G|stop_lost|HIGH|SAMD3|ENSG00000164483|transcript|ENST0000
protein_coding			T|structural_interaction_variant|HIGH|ARG1|ENSG00000118520|in
processed_transcript			A|splice_donor_variant&intron_variant|HIGH|STX7|ENSG0000079
protein_coding			T|stop_gained|HIGH|TAAR9|ENSG00000237110|transcript|ENST00
protein_coding			T|stop_gained|HIGH|TAAR2|ENSG00000146378|transcript|ENST00
nonsense_	16	0.13	A|splice_donor_variant&intron_variant|HIGH|VNN2|ENSG0000011
lincRNA			AG|splice_donor_variant&intron_variant|HIGH|LINC00326|ENSG00
protein_co	30	0.03	C|start_lost|HIGH|SGK1|ENSG00000118515|transcript|ENST00000
antisense			G|splice_acceptor_variant&intron_variant|HIGH|CTA-212D2.2|ENS
antisense			G|splice_acceptor_variant&intron_variant|HIGH|RP11-356I2.4|ENS
protein_coding			G|splice_donor_variant&splice_region_variant&5_prime_UTR_varia
protein_coding			G|structural_interaction_variant|HIGH|TNFAIP3|ENSG0000011850
protein_co	1	1	AG|frameshift_variant|HIGH|PBOV1|ENSG00000254440|transcript
protein_coding			A|structural_interaction_variant|HIGH|HEBP2|ENSG00000051620|i
antisense			A|splice_donor_variant&intron_variant|HIGH|RP1-225E12.2|ENSG0
protein_coding			A|stop_gained|HIGH|TXLNB|ENSG00000164440|transcript|ENST00
processed_transcript			A|splice_donor_variant&intron_variant|HIGH|VDAC1P8|ENSG0000
protein_co	2	0.5	C|frameshift_variant|HIGH|ZC2HC1B|ENSG00000118491|transcript
protein_co	2	0.5	C|frameshift_variant|HIGH|ZC2HC1B|ENSG00000118491|transcript
processed_transcript			C|splice_donor_variant&intron_variant|HIGH|UTRN|ENSG0000015
protein_co	2	1	C|stop_gained|HIGH|ULBP3|ENSG00000131019|transcript|ENST00
protein_co	3	0.67	G|frameshift_variant|HIGH|MYCT1|ENSG00000120279|transcript|E
protein_coding			T|stop_gained|HIGH|OPRM1|ENSG00000112038|transcript|ENST0
protein_coding			T|stop_gained|HIGH|OPRM1|ENSG00000112038|transcript|ENST0
protein_coding			A|structural_interaction_variant|HIGH|SNX9|ENSG00000130340|in
sense_intronic			A|splice_donor_variant&intron_variant|HIGH|SYNJ2-IT1|ENSG0000
nonsense_mediated_decay			T|stop_gained|HIGH|SERAC1|ENSG00000122335|transcript|ENST0
protein_co	3	0.67	C|frameshift_variant|HIGH|FNDC1|ENSG00000164694|transcript|E
protein_coding			T|structural_interaction_variant|HIGH|ACAT2|ENSG00000120437|i
protein_coding			G|structural_interaction_variant|HIGH|ACAT2|ENSG00000120437|i
antisense			T|splice_donor_variant&intron_variant|HIGH|AIRN|ENSG0000026
processed_transcript			T|splice_donor_variant&intron_variant|HIGH|LPAL2|ENSG000002
protein_co	3	0.33	T|splice_donor_variant&intron_variant|HIGH|LPA|ENSG000001986
protein_coding			T|structural_interaction_variant|HIGH|PARK2|ENSG00000185345|i
protein_co	11	0.09	A|splice_donor_variant&intron_variant|HIGH|PARK2|ENSG0000018
protein_co	11	0.09	C|splice_donor_variant&intron_variant|HIGH|PARK2|ENSG0000018
protein_coding			G|structural_interaction_variant|HIGH|PDE10A|ENSG00000112541
protein_coding			A|structural_interaction_variant|HIGH|PDE10A|ENSG00000112541
protein_coding			T|structural_interaction_variant|HIGH|PDE10A|ENSG00000112541|
protein_coding			A|structural_interaction_variant|HIGH|RNASET2|ENSG000002629
protein_co	5	0.4	A|stop_gained|HIGH|UNC93A|ENSG00000112494|transcript|ENST0
protein_co	5	0.2	C|splice_donor_variant&intron_variant|HIGH|UNC93A|ENSG00000
protein_co	5	0.4	T|stop_gained|HIGH|UNC93A|ENSG00000112494|transcript|ENST0
protein_co	10	0.1	C|frameshift_variant|HIGH|TCP10|ENSG00000203690|transcript|E
protein_coding			T|stop_gained|HIGH|TCP10|ENSG00000203690|transcript|ENST00
protein_co	10	0.1	C|splice_donor_variant&intron_variant|HIGH|TCP10|ENSG0000020
lincRNA			G|splice_donor_variant&splice_region_variant&intron_variant&non

protein_co	4	0.25	C|splice_acceptor_variant&intron_variant|HIGH|DACT2|ENSG0000(
protein_co	12	0.17	A|frameshift_variant&splice_region_variant|HIGH|WDR27|ENSG000
processed_transcript			T|splice_donor_variant&intron_variant|HIGH|WDR27|ENSG000001
protein_co	8	0.75	A|frameshift_variant|HIGH|TBP|ENSG00000112592|transcript|ENS
protein_co	8	0.75	A|frameshift_variant|HIGH|TBP|ENSG00000112592|transcript|ENS
protein_co	8	0.75	ACAGCAGCAGCAGCAGCAG|frameshift_variant|HIGH|TBP|ENSG0000
protein_co	8	0.75	ACAGCAGCAGCAGCAGCAG|frameshift_variant|HIGH|TBP|ENSG0000
protein_co	8	0.75	ACAGCAGCAGCAGCAGCAG|frameshift_variant|HIGH|TBP|ENSG0000
protein_coding			C|structural_interaction_variant|HIGH|TBP|ENSG00000112592|inte
protein_co	7	0.14	GCGTC|frameshift_variant|HIGH|GET4|ENSG00000239857|transcri
protein_coding			A|structural_interaction_variant|HIGH|ADAP1|ENSG00000105963|
protein_co	7	0.14	T|frameshift_variant|HIGH|GPER1|ENSG00000164850|transcript|E
processed_pseudogene			T|splice_acceptor_variant&intron_variant|HIGH|IMMP1LP3|ENSG0
processed_pseudogene			T|splice_acceptor_variant&intron_variant|HIGH|IMMP1LP3|ENSG0
protein_coding			G|splice_donor_variant&splice_region_variant&5_prime_UTR_varia
protein_co	7	0.14	A|frameshift_variant|HIGH|LFNG|ENSG00000106003|transcript|EN
protein_co	7	0.14	A|frameshift_variant|HIGH|LFNG|ENSG00000106003|transcript|EN
protein_coding			T|stop_gained|HIGH|AMZ1|ENSG00000174945|transcript|ENST000
processed_transcript			T|splice_donor_variant&intron_variant|HIGH|GNA12|ENSG000001
protein_co	8	0.13	C|start_lost|HIGH|GNA12|ENSG00000146535|transcript|ENST0000
processed_	2	0.5	A|splice_acceptor_variant&intron_variant|HIGH|RNF216P1|ENSG0
protein_coding			C|frameshift_variant&stop_lost|HIGH|TNRC18|ENSG00000182095|
protein_coding			C|frameshift_variant&stop_lost|HIGH|TNRC18|ENSG00000182095|
protein_coding			C|frameshift_variant&stop_lost|HIGH|TNRC18|ENSG00000182095|
protein_coding			C|frameshift_variant&stop_lost|HIGH|TNRC18|ENSG00000182095|
protein_coding			C|structural_interaction_variant|HIGH|FSCN1|ENSG00000075618|i
protein_coding			G|structural_interaction_variant|HIGH|PMS2|ENSG00000122512|ir
protein_coding			C|structural_interaction_variant|HIGH|CYTH3|ENSG00000008256|i
protein_co	11	0.18	T|splice_donor_variant&splice_region_variant&intron_variant|HIGH
retained_intron			C|splice_acceptor_variant&intron_variant|HIGH|AC005532.5|ENSG
protein_co	6	0.5	TAGCAGCAGCAGCAGCAGC|start_lost&conservative_inframe_inserti
protein_co	6	0.5	TAGCAGCAGCAGCAGCAGC|start_lost&conservative_inframe_inserti
protein_co	6	0.5	TAGCAGCAGCAGCAGCAGC|start_lost&conservative_inframe_inserti
protein_co	6	0.5	TAGCAGCAGCAGCAGCAGC|start_lost&conservative_inframe_inserti
protein_co	6	0.5	TAGCAGCAGCAGCAGCAGC|start_lost&conservative_inframe_inserti
protein_co	6	0.5	TAGCAGCAGCAGCAGCAGC|start_lost&conservative_inframe_inserti
protein_co	18	0.06	T|frameshift_variant|HIGH|ICA1|ENSG00000003147|transcript|ENS
protein_co	18	0.06	A|frameshift_variant|HIGH|ICA1|ENSG00000003147|transcript|ENS
protein_co	18	0.06	A|frameshift_variant|HIGH|ICA1|ENSG00000003147|transcript|ENS
protein_co	6	0.33	TA|frameshift_variant|HIGH|VWDE|ENSG00000146530|transcript|
nonsense_	6	0.33	A|stop_gained|HIGH|VWDE|ENSG00000146530|transcript|ENST000
protein_co	5	0.2	C|start_lost|HIGH|AC005281.1|ENSG00000226690|transcript|ENST
protein_co	5	0.4	GA|frameshift_variant|HIGH|AC005281.1|ENSG00000226690|trans
protein_co	10	0.1	C|splice_donor_variant&intron_variant|HIGH|SCIN|ENSG0000006
lincRNA			G|splice_acceptor_variant&intron_variant|HIGH|AC011288.2|ENSG
protein_coding			C|frameshift_variant|HIGH|SOSTDC1|ENSG00000171243|transcript
protein_co	14	0.79	GT|splice_acceptor_variant&intron_variant|HIGH|BZW2|ENSG0000

antisense			C|splice_acceptor_variant&intron_variant|HIGH|AC003075.4|ENSG(
protein_co	1	1	C|frameshift_variant|HIGH|PRPS1L1|ENSG00000229937|transcript
protein_co	6	0.17	C|splice_donor_variant&intron_variant|HIGH|ABCB5|ENSG0000000
protein_co	6	0.5	C|start_lost|HIGH|ABCB5|ENSG00000004846|transcript|ENST0000(
protein_co	12	0.25	T|stop_gained|HIGH|DNAH11|ENSG00000105877|transcript|ENST(
processed_transcript			A|splice_donor_variant&intron_variant|HIGH|DNAH11|ENSG00000
protein_co	7	0.14	C|frameshift_variant&splice_region_variant|HIGH|TOMM7|ENSG00
protein_co	7	0.14	C|frameshift_variant&splice_region_variant|HIGH|TOMM7|ENSG00
protein_co	12	0.08	G|frameshift_variant|HIGH|NUPL2|ENSG00000136243|transcript|E
nonsense_	8	0.13	C|frameshift_variant|HIGH|FAM221A|ENSG00000188732|transcrip
nonsense_mediated_decay			A|stop_gained|HIGH|FAM221A|ENSG00000188732|transcript|ENS
nonsense_	8	0.13	C|splice_donor_variant&intron_variant|HIGH|FAM221A|ENSG0000(
protein_co	13	0.08	C|splice_acceptor_variant&intron_variant|HIGH|STK31|ENSG00000
antisense			A|splice_acceptor_variant&splice_donor_variant&splice_region_var
transcribed_unprocessed_pseudo			T|splice_acceptor_variant&splice_region_variant&intron_variant|HI
)283618			G|bidirectional_gene_fusion|HIGH|Clostridiales-1&PLEKHA8|ENSG0
)283618			G|bidirectional_gene_fusion|HIGH|Clostridiales-1&PLEKHA8|ENSG0
lincRNA			C|splice_acceptor_variant&intron_variant|HIGH|RP4-777O23.2|ENS
antisense			G|splice_donor_variant&intron_variant|HIGH|AC005154.6|ENSG00(
protein_coding			G|structural_interaction_variant|HIGH|INMT|ENSG00000241644|ir
protein_coding			A|structural_interaction_variant|HIGH|INMT|ENSG00000241644|in
protein_coding			C|stop_lost|HIGH|INMT|ENSG00000241644|transcript|ENST00000(
protein_coding			CT|frameshift_variant|HIGH|CCDC129|ENSG00000180347|transcrip
unprocessed_pseudogene			CT|splice_acceptor_variant&intron_variant|HIGH|DPY19L1P1|ENSG
protein_coding			C|frameshift_variant&stop_lost|HIGH|RP9|ENSG00000164610|tran
protein_coding			C|stop_lost|HIGH|NPSR1|ENSG00000187258|transcript|ENST00000(
protein_coding			T|stop_gained|HIGH|NPSR1|ENSG00000187258|transcript|ENST00(
protein_coding			T|stop_gained|HIGH|NPSR1|ENSG00000187258|transcript|ENST00(
protein_co	13	0.38	CTT|splice_acceptor_variant&intron_variant|HIGH|ANLN|ENSG0000(
protein_co	13	0.38	CTT|splice_acceptor_variant&intron_variant|HIGH|ANLN|ENSG0000(
protein_co	7	0.14	G|splice_acceptor_variant&intron_variant|HIGH|STARD3NL|ENSG0(
TR_V_gene			T|stop_gained|HIGH|TRGV11|ENSG00000211693|transcript|ENST0
protein_co	10	0.5	CA|frameshift_variant|HIGH|POU6F2|ENSG00000106536|transcript
protein_co	10	0.5	CAG|frameshift_variant|HIGH|POU6F2|ENSG00000106536|transcri
unprocessed_pseudogene			AAAATAAAT|splice_acceptor_variant&intron_variant|HIGH|TCP1P1
unprocessed_pseudogene			A|splice_region_variant&intron_variant|LOW|TCP1P1|ENSG0000002
lincRNA			T|splice_donor_variant&intron_variant|HIGH|AC005537.2|ENSG00(
processed_transcript			T|splice_donor_variant&intron_variant|HIGH|URGCP|ENSG0000010
nonsense_	19	0.11	G|frameshift_variant|HIGH|POLM|ENSG00000122678|transcript|EI
protein_co	10	0.1	AG|frameshift_variant|HIGH|MYL7|ENSG00000106631|transcript|E
protein_coding			G|structural_interaction_variant|HIGH|CAMK2B|ENSG0000005840
protein_coding			G|structural_interaction_variant|HIGH|CAMK2B|ENSG0000005840
protein_coding			A|structural_interaction_variant|HIGH|NPC1L1|ENSG00000015520|
protein_coding			T|structural_interaction_variant|HIGH|CCM2|ENSG00000136280|ir
protein_coding			T|structural_interaction_variant|HIGH|IGFBP1|ENSG00000146678|
protein_co	11	0.09	A|stop_gained|HIGH|IGFBP3|ENSG00000146674|transcript|ENST00
lincRNA			T|splice_acceptor_variant&intron_variant|HIGH|AC011294.3|ENSG(

lincRNA			G|splice_donor_variant&intron_variant|HIGH|AC011294.3|ENSG00
protein_co	8	0.38	T|splice_donor_variant&intron_variant|HIGH|ABCA13|ENSG000001
protein_coding			T|structural_interaction_variant|HIGH|DDC|ENSG00000132437|int
protein_coding			A|structural_interaction_variant|HIGH|DDC|ENSG00000132437|int
protein_coding			A|structural_interaction_variant|HIGH|DDC|ENSG00000132437|int
protein_coding			A|structural_interaction_variant|HIGH|GRB10|ENSG00000106070|i
sense_overlapping			C|splice_donor_variant&intron_variant|HIGH|VSTM2A-OT1|ENSG00
protein_coding			A|structural_interaction_variant|HIGH|EGFR|ENSG00000146648|in
protein_coding			T|structural_interaction_variant|HIGH|EGFR|ENSG00000146648|in
unprocessed_pseudogene			T|splice_donor_variant&intron_variant|HIGH|PSPHP1|ENSG0000002
processed_pseudogene			C|splice_acceptor_variant&splice_region_variant&intron_variant&n
processed_pseudogene			CAA|splice_region_variant&non_coding_transcript_exon_variant|LC
processed_pseudogene			CAAAA|splice_region_variant&non_coding_transcript_exon_variant
processed_pseudogene			CAAAAA|splice_region_variant&non_coding_transcript_exon_varian
processed_pseudogene			CAAAAAA|splice_region_variant&non_coding_transcript_exon_varia
processed_pseudogene			CAAAAAAA|splice_region_variant&non_coding_transcript_exon_var
processed_pseudogene			C|splice_acceptor_variant&intron_variant|HIGH|TRIM60P16|ENSG0
unprocessed_pseudogene			G|splice_donor_variant&intron_variant|HIGH|PHKG1P4|ENSG0000
lincRNA			A|splice_acceptor_variant&intron_variant|HIGH|RP11-114G11.5|EN
unprocessed_pseudogene			T|splice_donor_variant&intron_variant|HIGH|SEPT14P1|ENSG0000
unprocessed_pseudogene			T|splice_acceptor_variant&intron_variant|HIGH|SEPT14P1|ENSG00
unprocessed_pseudogene			TT|splice_acceptor_variant&intron_variant|HIGH|SEPT14P1|ENSG0
unprocessed_pseudogene			TT|splice_acceptor_variant&intron_variant|HIGH|SEPT14P1|ENSG0
unprocessed_pseudogene			A|splice_donor_variant&intron_variant|HIGH|TNRC18P2|ENSG0000
protein_coding			A|stop_gained|HIGH|ZNF117|ENSG00000152926|transcript|ENST0
protein_coding			A|stop_gained|HIGH|ERV3-1|ENSG00000213462|transcript|ENST00
protein_co	1	1	G|splice_acceptor_variant&splice_donor_variant&intron_variant|HI
protein_co	1	1	G|splice_acceptor_variant&splice_donor_variant&intron_variant|HI
unprocessed_pseudogene			C|splice_acceptor_variant&splice_region_variant&intron_variant&n
unprocessed_pseudogene			C|splice_acceptor_variant&splice_region_variant&intron_variant&n
unprocessed_pseudogene			C|splice_acceptor_variant&splice_region_variant&intron_variant&n
unprocessed_pseudogene			C|splice_acceptor_variant&splice_region_variant&intron_variant&n
protein_co	1	1	C|splice_acceptor_variant&splice_donor_variant&intron_variant|HI
lincRNA			G|splice_acceptor_variant&splice_donor_variant&intron_variant|HI
lincRNA			G|splice_acceptor_variant&splice_donor_variant&intron_variant|HI
lincRNA			C|splice_acceptor_variant&splice_donor_variant&splice_region_vari
lincRNA			C|splice_acceptor_variant&splice_donor_variant&splice_region_vari
protein_coding			A|structural_interaction_variant|HIGH|GUSB|ENSG00000169919|in
lincRNA			A|splice_donor_variant&intron_variant|HIGH|LINC01372|ENSG0000
unprocessed_pseudogene			T|splice_donor_variant&intron_variant|HIGH|MTCO1P57|ENSG000
unprocessed_pseudogene			T|splice_donor_variant&intron_variant|HIGH|MTCO1P57|ENSG000
protein_co	3	0.67	T|stop_gained|HIGH|TYW1B|ENSG00000277149|transcript|ENST00
protein_co	3	1	CA|frameshift_variant|HIGH|TYW1B|ENSG00000277149|transcript
protein_co	5	1	C|frameshift_variant|HIGH|POM121|ENSG00000196313|transcript
protein_co	2	1	T|stop_gained|HIGH|CCL26|ENSG00000006606|transcript|ENST000
protein_co	2	0.5	A|splice_donor_variant&intron_variant|HIGH|CCL24|ENSG0000010
protein_coding			A|structural_interaction_variant|HIGH|YWHAG|ENSG00000170027

processed_transcript			C|splice_donor_variant&splice_region_variant&intron_variant&non_
processed_transcript			C|splice_acceptor_variant&intron_variant|HIGH|MAGI2|ENSG0000(
protein_coding			A|stop_gained|HIGH|MAGI2|ENSG00000187391|transcript|ENST00
protein_coding			G|structural_interaction_variant|HIGH|GNAI1|ENSG00000127955|i
protein_coding			C|structural_interaction_variant|HIGH|HGF|ENSG00000019991|inte
protein_co	5	0.2	C|splice_donor_variant&intron_variant|HIGH|GRM3|ENSG0000019
nonsense_	38	0.03	CTT|splice_acceptor_variant&intron_variant|HIGH|DMTF1|ENSG000
nonsense_	38	0.03	CTT|splice_acceptor_variant&intron_variant|HIGH|DMTF1|ENSG000
processed_transcript			A|splice_acceptor_variant&intron_variant|HIGH|CLDN12|ENSG0000(
lincRNA			A|splice_donor_variant&intron_variant|HIGH|RP11-142A5.1|ENSG0
processed_transcript			A|splice_donor_variant&intron_variant|HIGH|MTERF1|ENSG000001
protein_coding			C|structural_interaction_variant|HIGH|KRIT1|ENSG00000001631|in
protein_coding			T|stop_gained|HIGH|RBM48|ENSG00000127993|transcript|ENST00
protein_coding			A|structural_interaction_variant|HIGH|CDK6|ENSG00000105810|in
protein_coding			A|stop_gained|HIGH|SAMD9L|ENSG00000177409|transcript|ENST(
protein_co	20	0.05	G|start_lost|HIGH|VPS50|ENSG00000004766|transcript|ENST0000(
protein_co	20	0.05	CTT|splice_acceptor_variant&intron_variant|HIGH|VPS50|ENSG000
protein_co	20	0.05	CTT|splice_acceptor_variant&intron_variant|HIGH|VPS50|ENSG000
nonsense_	7	0.29	T|splice_donor_variant&intron_variant|HIGH|CALCR|ENSG0000000
protein_coding			C|structural_interaction_variant|HIGH|PDK4|ENSG00000004799|in
protein_coding			A|structural_interaction_variant|HIGH|PDK4|ENSG00000004799|in
protein_co	9	0.11	C|start_lost|HIGH|BUD31|ENSG00000106245|transcript|ENST0000
protein_co	25	0.04	C|splice_donor_variant&intron_variant|HIGH|ZNF655|ENSG000001
protein_coding			C|stop_lost|HIGH|CYP3A5|ENSG00000106258|transcript|ENST0000
protein_co	15	0.07	C|splice_acceptor_variant&intron_variant|HIGH|CYP3A5|ENSG0000
protein_co	17	0.41	T|frameshift_variant&splice_region_variant|HIGH|CYP3A43|ENSG0(
nonsense_	17	0.18	ATTT|splice_acceptor_variant&intron_variant|HIGH|CYP3A43|ENSG
nonsense_	17	0.18	ATTT|splice_acceptor_variant&intron_variant|HIGH|CYP3A43|ENSG
nonsense_	17	0.18	ATTT|splice_acceptor_variant&intron_variant|HIGH|CYP3A43|ENSG
protein_co	5	0.2	C|splice_acceptor_variant&intron_variant|HIGH|TRIM4|ENSG00000
protein_co	15	0.07	G|frameshift_variant|HIGH|ZNF3|ENSG00000166526|transcript|EN
protein_coding			A|structural_interaction_variant|HIGH|AP4M1|ENSG00000221838|
protein_co	19	0.05	CAGTG|splice_donor_variant&intron_variant|HIGH|STAG3|ENSG000
protein_co	2	0.5	T|stop_gained|HIGH|PVRIG|ENSG00000213413|transcript|ENST000
protein_co	4	0.5	C|frameshift_variant|HIGH|NYAP1|ENSG00000166924|transcript|E
protein_co	4	0.5	C|frameshift_variant|HIGH|NYAP1|ENSG00000166924|transcript|E
protein_co	4	0.5	C|frameshift_variant|HIGH|NYAP1|ENSG00000166924|transcript|E
protein_co	8	0.88	A|splice_donor_variant&intron_variant|HIGH|ZAN|ENSG00000146
protein_co	8	0.88	A|stop_gained|HIGH|ZAN|ENSG00000146839|transcript|ENST0000
protein_co	8	0.88	T|frameshift_variant|HIGH|ZAN|ENSG00000146839|transcript|ENS
protein_co	8	0.88	GTT|frameshift_variant|HIGH|ZAN|ENSG00000146839|transcript|E
protein_co	8	0.88	GTT|frameshift_variant|HIGH|ZAN|ENSG00000146839|transcript|E
protein_co	8	0.88	A|splice_donor_variant&intron_variant|HIGH|ZAN|ENSG00000146
protein_co	8	0.88	G|frameshift_variant|HIGH|ZAN|ENSG00000146839|transcript|ENS
protein_coding			G|structural_interaction_variant|HIGH|EPHB4|ENSG00000196411|i
protein_co	4	0.5	A|frameshift_variant|HIGH|MUC3A|ENSG00000169894|transcript|
protein_co	4	0.5	AGT|frameshift_variant|HIGH|MUC3A|ENSG00000169894|transcrip

protein_co	4	0.75	T|frameshift_variant|HIGH|MUC3A|ENSG00000169894|transcript|
protein_co	4	0.75	T|frameshift_variant|HIGH|MUC3A|ENSG00000169894|transcript|
protein_co	4	0.75	CACAT|frameshift_variant|HIGH|MUC3A|ENSG00000169894|transc
protein_co	4	0.75	C|frameshift_variant|HIGH|MUC3A|ENSG00000169894|transcript|
protein_co	4	0.75	ACCCCC|frameshift_variant|HIGH|MUC3A|ENSG00000169894|trans
protein_co	4	0.75	A|frameshift_variant|HIGH|MUC3A|ENSG00000169894|transcript|
protein_co	4	0.75	CGT|frameshift_variant|HIGH|MUC3A|ENSG00000169894|transcrip
antisense			G|splice_acceptor_variant&intron_variant|HIGH|RP11-395B7.2|ENS
protein_co	4	0.5	TCAGAA|frameshift_variant|HIGH|MUC17|ENSG00000169876|tran
protein_co	4	0.5	CAG|frameshift_variant|HIGH|MUC17|ENSG00000169876|transcrip
protein_co	9	0.11	AACAGGGAAAGG|frameshift_variant|HIGH|FIS1|ENSG0000021425
protein_co	7	0.43	T|frameshift_variant|HIGH|RASA4B|ENSG00000170667|transcript|
antisense			A|splice_donor_variant&intron_variant|HIGH|NFE4|ENSG00000230
processed_transcript			CTTT|splice_acceptor_variant&intron_variant|HIGH|CDHR3|ENSG00
processed_transcript			CTTT|splice_acceptor_variant&intron_variant|HIGH|CDHR3|ENSG00
protein_coding			A|structural_interaction_variant|HIGH|PIK3CG|ENSG00000105851|
protein_coding			G|structural_interaction_variant|HIGH|PIK3CG|ENSG00000105851|
protein_coding			T|structural_interaction_variant|HIGH|PIK3CG|ENSG00000105851|
protein_coding			A|frameshift_variant|HIGH|LAMB4|ENSG00000091128|transcript|E
protein_co	17	0.06	C|splice_acceptor_variant&intron_variant|HIGH|NRCAM|ENSG0000
processed_pseudogene			A|splice_acceptor_variant&splice_region_variant&intron_variant&n
processed_pseudogene			AAAAACAAAAC|non_coding_transcript_exon_variant|MODIFIER|NP
processed_pseudogene			AAAAACAAAACAAAAC|non_coding_transcript_exon_variant|MODIF
protein_co	3	0.33	C|frameshift_variant|HIGH|PPP1R3A|ENSG00000154415|transcript
processed_pseudogene			TAA|splice_acceptor_variant&intron_variant|HIGH|RP11-222O23.1|
processed_pseudogene			T|splice_region_variant&intron_variant|LOW|RP11-222O23.1|ENSG
protein_coding			C|protein_protein_contact|HIGH|TES|ENSG00000135269|interactic
nonsense_	9	0.11	GCC|frameshift_variant|HIGH|CAV1|ENSG00000105974|transcript|
protein_coding			A|structural_interaction_variant|HIGH|MET|ENSG00000105976|int
protein_coding			T|structural_interaction_variant|HIGH|MET|ENSG00000105976|int
processed_transcript			C|splice_acceptor_variant&intron_variant|HIGH|MET|ENSG0000010
processed_pseudogene			GT|splice_acceptor_variant&intron_variant|HIGH|AC002543.2|ENS
protein_co	2	0.5	GCACC|frameshift_variant&start_lost|HIGH|WNT16|ENSG0000000
protein_co	2	0.5	GCACC|frameshift_variant&start_lost|HIGH|WNT16|ENSG0000000
antisense			A|splice_donor_variant&intron_variant|HIGH|RP5-1101C3.1|ENSG0
antisense			A|splice_donor_variant&intron_variant|HIGH|RP5-1101C3.1|ENSG0
protein_co	1	1	T|start_lost|HIGH|RNF133|ENSG00000188050|transcript|ENST0000
protein_co	7	0.71	C|frameshift_variant&splice_region_variant|HIGH|ASB15|ENSG0000
protein_co	5	1	G|start_lost|HIGH|HYAL4|ENSG00000106302|transcript|ENST0000
protein_coding			C|stop_gained|HIGH|POT1|ENSG00000128513|transcript|ENST000
lincRNA			A|splice_acceptor_variant&intron_variant|HIGH|AC005276.1|ENSG
protein_co	15	0.07	T|splice_donor_variant&intron_variant|HIGH|GRM8|ENSG0000017
protein_co	7	0.14	C|stop_lost|HIGH|PAX4|ENSG00000106331|transcript|ENST000003
protein_coding			T|structural_interaction_variant|HIGH|LRRC4|ENSG00000128594|ir
protein_coding			C|structural_interaction_variant|HIGH|SND1|ENSG00000197157|in
processed_transcript			C|splice_donor_variant&intron_variant|HIGH|RP11-274B21.14|ENS
protein_coding			A|structural_interaction_variant|HIGH|FLNC|ENSG00000128591|in

protein_co	15	0.07	G|splice_donor_variant&intron_variant|HIGH|IRF5|ENSG0000012[illegible]
protein_coding			C|splice_acceptor_variant&splice_region_variant&3_prime_UTR_va[illegible]
protein_coding			C|structural_interaction_variant|HIGH|TNPO3|ENSG00000064419|[illegible]
protein_co	1	1	C|frameshift_variant|HIGH|RP11-286H14.4|ENSG00000230626|tra[illegible]
processed_pseudogene			G|splice_acceptor_variant&splice_region_variant&intron_variant|H[illegible]
processed_pseudogene			G|splice_acceptor_variant&splice_region_variant&intron_variant|H[illegible]
processed_pseudogene			G|splice_acceptor_variant&splice_region_variant&intron_variant|H[illegible]
processed_pseudogene			G|splice_acceptor_variant&intron_variant|HIGH|AC105443.2|ENSG
protein_co	2	0.5	T|frameshift_variant&start_lost|HIGH|TMEM140|ENSG0000014685
lincRNA			C|splice_donor_variant&intron_variant|HIGH|AC024084.1|ENSG000
protein_coding			A|structural_interaction_variant|HIGH|PARP12|ENSG00000059378
protein_co	6	0.17	CTT|splice_acceptor_variant&intron_variant|HIGH|ADCK2|ENSG000
protein_co	6	0.17	CTT|splice_acceptor_variant&intron_variant|HIGH|ADCK2|ENSG000
protein_coding			T|structural_interaction_variant|HIGH|CLEC5A|ENSG00000258227|
protein_coding			T|stop_gained|HIGH|CLCN1|ENSG00000188037|transcript|ENST000
polymorph	1	1	C|frameshift_variant|HIGH|OR10AC1|ENSG00000176510|transcript
polymorph	1	1	G|frameshift_variant|HIGH|OR10AC1|ENSG00000176510|transcrip
protein_co	6	1	G|frameshift_variant|HIGH|TCAF2|ENSG00000170379|transcript|E
protein_co	4	0.5	ATGGAGGCTGAGGAGGCCCAGCG|frameshift_variant&stop_gained|H
nonsense_	13	0.15	CT|frameshift_variant|HIGH|TPK1|ENSG00000196511|transcript|E
protein_co	13	0.08	T|stop_gained|HIGH|TPK1|ENSG00000196511|transcript|ENST000
protein_co	13	0.08	T|splice_donor_variant&intron_variant|HIGH|TPK1|ENSG00000196
nonsense_	13	0.15	T|splice_acceptor_variant&intron_variant|HIGH|TPK1|ENSG000001
protein_coding			T|structural_interaction_variant|HIGH|TPK1|ENSG00000196511|in
protein_co	1	1	T|start_lost|HIGH|C7orf33|ENSG00000170279|transcript|ENST000
unprocessed_pseudogene			T|splice_acceptor_variant&splice_region_variant&intron_variant&n
unprocessed_pseudogene			T|splice_acceptor_variant&splice_region_variant&intron_variant&n
unprocessed_pseudogene			T|splice_acceptor_variant&splice_region_variant&intron_variant&n
unprocessed_pseudogene			T|splice_acceptor_variant&splice_region_variant&intron_variant&n
unprocessed_pseudogene			T|splice_acceptor_variant&splice_region_variant&intron_variant&n
unprocessed_pseudogene			T|splice_acceptor_variant&splice_region_variant&intron_variant&n
protein_co	9	0.44	CT|splice_acceptor_variant&intron_variant|HIGH|KRBA1|ENSG000
protein_co	11	0.09	T|frameshift_variant|HIGH|SSPO|ENSG00000197558|transcript|EN
protein_co	11	0.09	G|splice_donor_variant&intron_variant|HIGH|SSPO|ENSG00000197
protein_co	11	0.09	G|frameshift_variant&splice_region_variant|HIGH|SSPO|ENSG0000
protein_co	11	0.09	GC|splice_acceptor_variant&splice_donor_variant&intron_variant|H
protein_co	11	0.09	TA|frameshift_variant&stop_gained|HIGH|SSPO|ENSG0000019755
protein_co	11	0.09	T|frameshift_variant|HIGH|SSPO|ENSG00000197558|transcript|EN
protein_co	11	0.09	G|splice_acceptor_variant&intron_variant|HIGH|SSPO|ENSG000001
protein_co	11	0.09	T|stop_gained|HIGH|SSPO|ENSG00000197558|transcript|ENST000
retained_intron			G|splice_acceptor_variant&intron_variant|HIGH|SSPO|ENSG000001
protein_co	6	0.5	A|stop_gained|HIGH|ACTR3C|ENSG00000106526|transcript|ENST0
protein_coding			T|structural_interaction_variant|HIGH|GIMAP2|ENSG00000106560
protein_coding			C|structural_interaction_variant|HIGH|GIMAP2|ENSG00000106560
protein_coding			T|structural_interaction_variant|HIGH|AOC1|ENSG00000002726|in
protein_co	26	0.08	CG|frameshift_variant|HIGH|AGAP3|ENSG00000133612|transcript|
protein_co	26	0.08	CG|frameshift_variant|HIGH|AGAP3|ENSG00000133612|transcript|

protein_co	26	0.04	G|frameshift_variant|HIGH|AGAP3|ENSG00000133612|transcript|E
protein_co	3	0.33	T|frameshift_variant|HIGH|IQCA1L|ENSG00000278685|transcript|E
nonsense_mediated_decay			T|splice_donor_variant&splice_region_variant&3_prime_UTR_variar
nonsense_	11	0.55	G|splice_donor_variant&intron_variant|HIGH|GALNTL5|ENSG0000C
protein_co	13	0.08	G|splice_donor_variant&intron_variant|HIGH|GALNT11|ENSG0000C
protein_coding			G|structural_interaction_variant|HIGH|DPP6|ENSG00000130226|in
protein_coding			T|structural_interaction_variant|HIGH|DPP6|ENSG00000130226|in
protein_coding			C|structural_interaction_variant|HIGH|DPP6|ENSG00000130226|in
protein_coding			T|structural_interaction_variant|HIGH|DPP6|ENSG00000130226|in
protein_co	14	0.43	C|splice_donor_variant&intron_variant|HIGH|RNF32|ENSG0000010
)224629			C|bidirectional_gene_fusion|HIGH|RP5-1142J19.2&UBE3C|ENSG000
antisense			C|splice_donor_variant&intron_variant|HIGH|AC011899.10|ENSG00
protein_co	5	0.2	A|splice_donor_variant&intron_variant|HIGH|WDR60|ENSG000001
nonsense_mediated_decay			G|stop_lost|HIGH|ERICH1|ENSG00000104714|transcript|ENST0000
lincRNA			C|splice_acceptor_variant&splice_region_variant&intron_variant|HI
lincRNA			G|splice_acceptor_variant&intron_variant|HIGH|GS1-57L11.1|ENSG
protein_coding			G|structural_interaction_variant|HIGH|ANGPT2|ENSG00000091879
protein_coding			G|structural_interaction_variant|HIGH|ANGPT2|ENSG00000091879
unprocessed_pseudogene			C|splice_donor_variant&intron_variant|HIGH|FAM90A20P|ENSG000
protein_coding			G|stop_lost|HIGH|SPAG11A|ENSG00000178287|transcript|ENST00
unprocessed_pseudogene			T|splice_donor_variant&intron_variant|HIGH|ENPP7P1|ENSG00000
processed_transcript			A|splice_acceptor_variant&intron_variant|HIGH|FAM86B3P|ENSG0
processed_transcript			A|splice_donor_variant&intron_variant|HIGH|ERI1|ENSG00000104
lincRNA			G|splice_acceptor_variant&intron_variant|HIGH|RP11-115J16.3|EN
lincRNA			A|splice_donor_variant&intron_variant|HIGH|RP11-115J16.2|ENSG
lincRNA			T|splice_donor_variant&intron_variant|HIGH|RP11-115J16.2|ENSG
processed_pseudogene			G|splice_acceptor_variant&intron_variant|HIGH|RP11-375N15.1|EN
processed_pseudogene			G|splice_acceptor_variant&intron_variant|HIGH|RP11-375N15.1|EN
retained_intron			A|splice_donor_variant&intron_variant|HIGH|TNKS|ENSG00000173
protein_coding			T|frameshift_variant|HIGH|PRSS55|ENSG00000184647|transcript|E
protein_co	2	0.5	CCTCTCTTCTT|frameshift_variant|HIGH|RP1L1|ENSG00000183638|
protein_co	2	0.5	GCAGCCCTTCTATTACTTTAGTCCCCTCTAACTGCACCC|frameshift_varia
protein_co	2	0.5	GCAGCCCTTCTTCTGTTTTAGTTTCCTCTAACTGCACCC|frameshift_varia
processed_transcript			A|splice_acceptor_variant&intron_variant|HIGH|PINX1|ENSG00000
processed_transcript			G|splice_acceptor_variant&intron_variant|HIGH|TDH|ENSG000001
protein_coding			T|structural_interaction_variant|HIGH|FDFT1|ENSG00000079459|in
protein_coding			C|structural_interaction_variant|HIGH|FDFT1|ENSG00000079459|in
protein_coding			C|structural_interaction_variant|HIGH|FDFT1|ENSG00000079459|in
unprocessed_pseudogene			T|splice_acceptor_variant&intron_variant|HIGH|FAM90A2P|ENSG0
protein_co	19	0.11	G|frameshift_variant|HIGH|FAM86B1|ENSG00000186523|transcrip
protein_co	4	0.5	G|frameshift_variant|HIGH|FAM86B2|ENSG00000145002|transcrip
unprocessed_pseudogene			A|splice_acceptor_variant&splice_region_variant&intron_variant|HI
protein_coding			T|structural_interaction_variant|HIGH|DLC1|ENSG00000164741|int
protein_co	14	0.07	T|splice_donor_variant&intron_variant|HIGH|MSR1|ENSG0000003
protein_co	6	0.17	CA|frameshift_variant|HIGH|MICU3|ENSG00000155970|transcript|
protein_co	13	0.08	C|splice_donor_variant&splice_region_variant&intron_variant|HIGH
protein_co	13	0.08	C|splice_donor_variant&splice_region_variant&intron_variant|HIGH

nonsense_	11	0.09	T|frameshift_variant|HIGH|VPS37A|ENSG00000155975|transcript|
protein_co	11	0.18	G|splice_acceptor_variant&intron_variant|HIGH|FGL1|ENSG000001
lincRNA			A|splice_donor_variant&intron_variant|HIGH|RP11-156K13.3|ENSG
protein_co	69	0.04	TGCACCTGTGCTGTATATCTAAGACATACA|frameshift_variant&stop_g
protein_co	69	0.06	C|frameshift_variant|HIGH|ASAH1|ENSG00000104763|transcript|E
nonsense_	69	0.04	C|stop_lost&splice_region_variant|HIGH|ASAH1|ENSG0000010476
protein_co	10	0.1	C|splice_acceptor_variant&intron_variant|HIGH|NAT1|ENSG000001
protein_coding			A|structural_interaction_variant|HIGH|NAT1|ENSG00000171428|in
0278886			T|gene_fusion|HIGH|PSD3&RP11-108A14.1|ENSG00000156011&EN
0278886			T|gene_fusion|HIGH|PSD3&RP11-108A14.1|ENSG00000156011&EN
0278886			T|gene_fusion|HIGH|PSD3&RP11-108A14.1|ENSG00000156011&EN
0278886			T|gene_fusion|HIGH|PSD3&RP11-108A14.1|ENSG00000156011&EN
0278886			A|gene_fusion|HIGH|PSD3&RP11-108A14.1|ENSG00000156011&EN
0278886			A|gene_fusion|HIGH|PSD3&RP11-108A14.1|ENSG00000156011&EN
0278886			A|gene_fusion|HIGH|PSD3&RP11-108A14.1|ENSG00000156011&EN
0278886			A|gene_fusion|HIGH|PSD3&RP11-108A14.1|ENSG00000156011&EN
0278886			A|gene_fusion|HIGH|PSD3&RP11-108A14.1|ENSG00000156011&EN
0278886			A|gene_fusion|HIGH|PSD3&RP11-108A14.1|ENSG00000156011&EN
processed_transcript			G|splice_donor_variant&intron_variant|HIGH|CSGALNACT1|ENSG0
protein_coding			G|stop_gained|HIGH|LPL|ENSG00000175445|transcript|ENST0000
nonsense_	6	0.17	C|splice_acceptor_variant&intron_variant|HIGH|ATP6V1B2|ENSG0
lincRNA			G|splice_donor_variant&intron_variant|HIGH|RP11-369E15.4|ENSG
nonsense_mediated_decay			A|stop_gained|HIGH|HR|ENSG00000168453|transcript|ENST00000
nonsense_mediated_decay			G|stop_gained|HIGH|BMP1|ENSG00000168487|transcript|ENST000
protein_co	21	0.05	G|splice_donor_variant&intron_variant|HIGH|PDLIM2|ENSG000001
protein_coding			G|frameshift_variant|HIGH|PEBP4|ENSG00000134020|transcript|E
protein_co	10	0.1	A|splice_acceptor_variant&intron_variant|HIGH|R3HCC1|ENSG0000
protein_co	15	0.13	AGTATT|splice_donor_variant&intron_variant|HIGH|LOXL2|ENSG00
protein_co	2	1	T|frameshift_variant|HIGH|STC1|ENSG00000159167|transcript|EN
protein_co	2	1	T|frameshift_variant|HIGH|STC1|ENSG00000159167|transcript|EN
protein_co	2	1	T|frameshift_variant|HIGH|STC1|ENSG00000159167|transcript|EN
processed_transcript			A|splice_acceptor_variant&intron_variant|HIGH|SDAD1P1|ENSG000
protein_coding			C|structural_interaction_variant|HIGH|DPYSL2|ENSG00000092964|
protein_coding			T|structural_interaction_variant|HIGH|DPYSL2|ENSG00000092964|
protein_coding			G|structural_interaction_variant|HIGH|EPHX2|ENSG00000120915|i
protein_coding			A|structural_interaction_variant|HIGH|EPHX2|ENSG00000120915|i
protein_coding			A|structural_interaction_variant|HIGH|EPHX2|ENSG00000120915|i
protein_coding			AGTC|structural_interaction_variant|HIGH|EPHX2|ENSG000001209
protein_coding			T|structural_interaction_variant|HIGH|EPHX2|ENSG00000120915|i
protein_co	4	0.25	C|splice_donor_variant&intron_variant|HIGH|PBK|ENSG000001680
sense_intronic			C|splice_acceptor_variant&intron_variant|HIGH|RP11-181B11.1|EN
processed_transcript			A|splice_donor_variant&intron_variant|HIGH|EXTL3|ENSG0000001
lincRNA			G|splice_acceptor_variant&intron_variant|HIGH|RP11-489E7.1|ENS
protein_co	14	0.64	A|splice_acceptor_variant&intron_variant|HIGH|SARAF|ENSG0000
protein_coding			T|frameshift_variant|HIGH|UBXN8|ENSG00000104691|transcript|E
protein_co	3	0.33	T|frameshift_variant|HIGH|TEX15|ENSG00000133863|transcript|EN
protein_coding			A|structural_interaction_variant|HIGH|DUSP26|ENSG00000133878

lincRNA			CTT|splice_acceptor_variant&intron_variant|HIGH|RP11-317N12.1|
lincRNA			CTT|splice_acceptor_variant&intron_variant|HIGH|RP11-317N12.1|
protein_co	1	1	C|frameshift_variant|HIGH|ZNF703|ENSG00000183779|transcript|
protein_co	3	0.33	G|stop_gained|HIGH|ADGRA2|ENSG00000020181|transcript|ENST
protein_coding			G|stop_lost|HIGH|C8orf86|ENSG00000196166|transcript|ENST000
protein_co	9	0.44	CG|splice_donor_variant&intron_variant|HIGH|PLEKHA2|ENSG0000
processed_transcript			T|splice_donor_variant&intron_variant|HIGH|ADAM3A|ENSG00000
protein_coding			A|stop_gained|HIGH|IDO2|ENSG00000188676|transcript|ENST000
protein_coding			A|structural_interaction_variant|HIGH|ANK1|ENSG00000029534|in
protein_coding			T|structural_interaction_variant|HIGH|ANK1|ENSG00000029534|in
protein_co	3	1	T|stop_gained|HIGH|POMK|ENSG00000185900|transcript|ENST000
processed_transcript			CTTT|splice_acceptor_variant&intron_variant|HIGH|SNTG1|ENSG00
processed_transcript			CTTT|splice_acceptor_variant&intron_variant|HIGH|SNTG1|ENSG00
processed_transcript			CTTT|splice_acceptor_variant&intron_variant|HIGH|SNTG1|ENSG00
protein_co	4	0.75	T|stop_gained|HIGH|PXDNL|ENSG00000147485|transcript|ENST00
antisense			C|splice_donor_variant&intron_variant|HIGH|RP11-26M5.3|ENSG0
protein_coding			G|splice_donor_variant&splice_region_variant&5_prime_UTR_varia
lincRNA			A|splice_acceptor_variant&intron_variant|HIGH|RP11-716D16.1|EN
nonsense_	17	0.06	T|splice_donor_variant&intron_variant|HIGH|NSMAF|ENSG000000
lincRNA			C|splice_donor_variant&intron_variant|HIGH|RP11-379I19.3|ENSG
protein_coding			C|structural_interaction_variant|HIGH|CA8|ENSG00000178538|inte
processed_transcript			C|splice_donor_variant&intron_variant|HIGH|CLVS1|ENSG0000017
lincRNA			T|splice_acceptor_variant&intron_variant|HIGH|LINC00251|ENSG0
protein_co	15	0.27	C|frameshift_variant|HIGH|MTFR1|ENSG00000066855|transcript|E
protein_co	5	0.6	C|frameshift_variant|HIGH|C8orf44|ENSG00000213865|transcript|
protein_coding			T|frameshift_variant|HIGH|C8orf34|ENSG00000165084|transcript|
processed_pseudogene			A|splice_acceptor_variant&intron_variant|HIGH|SDCBPP2|ENSG000
protein_co	6	0.17	T|splice_acceptor_variant&intron_variant|HIGH|TRPA1|ENSG00000
lincRNA			T|splice_acceptor_variant&splice_donor_variant&splice_region_vari
protein_co	29	0.03	AG|frameshift_variant|HIGH|STAU2|ENSG00000040341|transcript|
protein_co	7	0.86	C|splice_acceptor_variant&intron_variant|HIGH|UBE2W|ENSG0000
lincRNA			C|splice_donor_variant&intron_variant|HIGH|MIR2052HG|ENSG000
protein_co	4	0.25	GTTTATTTATTTATTTA|splice_acceptor_variant&intron_variant|HIGH
protein_co	4	0.25	GTTTATTTATTTATTTA|splice_acceptor_variant&intron_variant|HIGH
protein_co	8	0.13	A|frameshift_variant&start_lost|HIGH|HEY1|ENSG00000164683|tra
protein_co	5	0.2	C|frameshift_variant&start_lost|HIGH|ZBTB10|ENSG00000205189|
antisense			A|splice_donor_variant&intron_variant|HIGH|RP11-257P3.3|ENSG0
lincRNA			C|splice_donor_variant&splice_region_variant&intron_variant&non_
lincRNA			C|splice_acceptor_variant&intron_variant|HIGH|RP11-731N10.1|EN
unprocessed_pseudogene			C|splice_acceptor_variant&intron_variant|HIGH|RP11-120I21.3|ENS
unprocessed_pseudogene			C|splice_acceptor_variant&intron_variant|HIGH|RP11-120I21.3|ENS
unprocessed_pseudogene			C|splice_acceptor_variant&intron_variant|HIGH|RP11-120I21.3|ENS
unprocessed_pseudogene			C|splice_acceptor_variant&intron_variant|HIGH|RP11-120I21.3|ENS
protein_co	11	0.55	TCC|splice_acceptor_variant&intron_variant|HIGH|RALYL|ENSG000
protein_co	11	0.55	TCC|splice_acceptor_variant&intron_variant|HIGH|RALYL|ENSG000
protein_co	6	1	CTT|splice_acceptor_variant&intron_variant|HIGH|LRRCC1|ENSG00
protein_co	6	1	CTT|splice_acceptor_variant&intron_variant|HIGH|LRRCC1|ENSG00

protein_co	13	0.23	T|splice_donor_variant&intron_variant|HIGH|C8orf59|ENSG000001
protein_coding			G|structural_interaction_variant|HIGH|CA13|ENSG00000185015|in
protein_co	25	0.08	T|splice_donor_variant&intron_variant|HIGH|CA1|ENSG000001337
protein_coding			C|structural_interaction_variant|HIGH|CA2|ENSG00000104267|inte
protein_coding			T|frameshift_variant&stop_lost|HIGH|SLC7A13|ENSG00000164893
protein_co	3	0.33	A|splice_donor_variant&intron_variant|HIGH|SLC7A13|ENSG00000
protein_coding			T|splice_acceptor_variant&5_prime_UTR_variant&intron_variant|H
antisense			G|splice_acceptor_variant&intron_variant|HIGH|RP11-386D6.1|EN
protein_co	19	0.05	A|splice_donor_variant&intron_variant|HIGH|DECR1|ENSG0000010
lincRNA			T|splice_acceptor_variant&intron_variant|HIGH|LINC01030|ENSG0
nonsense_	7	0.29	GTT|splice_acceptor_variant&intron_variant|HIGH|LRRC69|ENSG00
nonsense_	7	0.29	GTT|splice_acceptor_variant&intron_variant|HIGH|LRRC69|ENSG00
nonsense_	7	0.43	A|splice_donor_variant&intron_variant|HIGH|LRRC69|ENSG000002
protein_co	22	0.41	CTT|splice_acceptor_variant&intron_variant|HIGH|TMEM67|ENSG0
protein_co	22	0.41	CTT|splice_acceptor_variant&intron_variant|HIGH|TMEM67|ENSG0
protein_co	22	0.41	CTT|splice_acceptor_variant&intron_variant|HIGH|TMEM67|ENSG0
nonsense_	22	0.05	C|splice_acceptor_variant&intron_variant|HIGH|TMEM67|ENSG000
nonsense_	20	0.1	CT|splice_acceptor_variant&intron_variant|HIGH|INTS8|ENSG0000
protein_co	4	0.5	CGGCGGGCTCCAGGCGA|frameshift_variant|HIGH|LAPTM4B|ENSG0
protein_co	4	0.25	A|splice_acceptor_variant&intron_variant|HIGH|LAPTM4B|ENSG00
protein_co	18	0.06	A|splice_donor_variant&intron_variant|HIGH|MATN2|ENSG000001
protein_coding			C|stop_lost|HIGH|MATN2|ENSG00000132561|transcript|ENST0000
protein_co	8	0.13	T|splice_donor_variant&intron_variant|HIGH|NIPAL2|ENSG000001
protein_coding			G|stop_gained|HIGH|VPS13B|ENSG00000132549|transcript|ENST0
protein_co	12	0.17	ATT|splice_acceptor_variant&intron_variant|HIGH|VPS13B|ENSG00
protein_co	12	0.17	ATT|splice_acceptor_variant&intron_variant|HIGH|VPS13B|ENSG00
protein_co	12	0.17	ATT|splice_acceptor_variant&intron_variant|HIGH|VPS13B|ENSG00
protein_co	12	0.17	ATT|splice_acceptor_variant&intron_variant|HIGH|VPS13B|ENSG00
protein_co	12	0.17	ATT|splice_acceptor_variant&intron_variant|HIGH|VPS13B|ENSG00
protein_co	12	0.17	ATT|splice_acceptor_variant&intron_variant|HIGH|VPS13B|ENSG00
protein_co	11	0.18	C|splice_acceptor_variant&intron_variant|HIGH|COX6C|ENSG00000
protein_co	25	0.24	G|splice_acceptor_variant&splice_donor_variant&splice_region_var
protein_co	25	0.16	GGATGAGGT|splice_acceptor_variant&intron_variant|HIGH|PABPC1
protein_co	25	0.16	GGATGAGGT|splice_acceptor_variant&intron_variant|HIGH|PABPC1
protein_co	25	0.16	GGATGAGGT|splice_acceptor_variant&intron_variant|HIGH|PABPC1
protein_co	25	0.16	GGATGAGGT|splice_acceptor_variant&intron_variant|HIGH|PABPC1
protein_co	25	0.24	C|frameshift_variant|HIGH|PABPC1|ENSG00000070756|transcript|
protein_co	25	0.24	TC|frameshift_variant|HIGH|PABPC1|ENSG00000070756|transcript
protein_coding			C|structural_interaction_variant|HIGH|PABPC1|ENSG00000070756
protein_co	25	0.24	CTTTA|splice_acceptor_variant&intron_variant|HIGH|PABPC1|ENSG
protein_coding			T|structural_interaction_variant|HIGH|YWHAZ|ENSG00000164924|
lincRNA			C|splice_acceptor_variant&intron_variant|HIGH|FLJ42969|ENSG000
protein_co	11	0.09	CG|frameshift_variant|HIGH|RRM2B|ENSG00000048392|transcript
protein_co	2	1	A|frameshift_variant|HIGH|ODF1|ENSG00000155087|transcript|EN
protein_co	2	1	A|frameshift_variant|HIGH|ODF1|ENSG00000155087|transcript|EN
antisense			A|splice_acceptor_variant&intron_variant|HIGH|AZIN1-AS1|ENSG00
processed_pseudogene			A|splice_donor_variant&splice_region_variant&intron_variant&non_

processed_pseudogene			A|splice_donor_variant&splice_region_variant&intron_variant&non_
protein_co	7	0.29	G|start_lost|HIGH|FZD6|ENSG00000164930|transcript|ENST00000!
protein_co	4	0.25	G|start_lost|HIGH|CTHRC1|ENSG00000164932|transcript|ENST000
protein_co	15	0.4	T|splice_acceptor_variant&intron_variant|HIGH|OXR1|ENSG000001
protein_co	15	0.2	A|stop_gained|HIGH|OXR1|ENSG00000164830|transcript|ENST000
protein_co	4	0.25	C|splice_donor_variant&intron_variant|HIGH|PKHD1L1|ENSG00000
protein_co	4	0.5	CTTT|splice_acceptor_variant&intron_variant|HIGH|PKHD1L1|ENSG
protein_co	4	0.5	CTTT|splice_acceptor_variant&intron_variant|HIGH|PKHD1L1|ENSG
protein_co	4	0.5	CTTT|splice_acceptor_variant&intron_variant|HIGH|PKHD1L1|ENSG
protein_co	15	0.27	T|splice_donor_variant&intron_variant|HIGH|TBC1D31|ENSG00000
protein_co	1	1	C|splice_donor_variant&splice_region_variant&intron_variant|HIGH
protein_coding			A|structural_interaction_variant|HIGH|TATDN1|ENSG00000147687
protein_coding			T|stop_gained|HIGH|NDUFB9|ENSG00000147684|transcript|ENST0
lincRNA			CTT|splice_acceptor_variant&intron_variant|HIGH|RP11-136O12.2|
lincRNA			CTT|splice_acceptor_variant&intron_variant|HIGH|RP11-136O12.2|
lincRNA			CTT|splice_acceptor_variant&intron_variant|HIGH|RP11-136O12.2|
lincRNA			C|splice_donor_variant&intron_variant|HIGH|CCDC26|ENSG000002
processed_pseudogene			C|splice_acceptor_variant&intron_variant|HIGH|MTRF1LP2|ENSG00
lincRNA			T|splice_donor_variant&intron_variant|HIGH|CTD-2501M5.1|ENSG
lincRNA			G|splice_acceptor_variant&intron_variant|HIGH|CTD-2501M5.1|EN
protein_coding			C|stop_lost|HIGH|WISP1|ENSG00000104415|transcript|ENST00000
protein_co	17	0.24	C|splice_donor_variant&intron_variant|HIGH|ZFAT|ENSG00000066
protein_co	17	0.06	C|frameshift_variant&splice_region_variant|HIGH|ZFAT|ENSG00000
lincRNA			G|splice_acceptor_variant&intron_variant|HIGH|RP11-30J20.1|ENS
protein_coding			A|structural_interaction_variant|HIGH|AGO2|ENSG00000123908|ir
protein_co	22	0.05	C|start_lost|HIGH|DENND3|ENSG00000105339|transcript|ENST000
antisense			G|splice_acceptor_variant&splice_region_variant&intron_variant&n
polymorph	5	1	T|splice_acceptor_variant&intron_variant|HIGH|MROH5|ENSG0000
processed_transcript			G|splice_acceptor_variant&splice_region_variant&intron_variant&n
processed_transcript			CGAGAGGCAAGGG|splice_acceptor_variant&intron_variant|HIGH|JI
processed_transcript			G|splice_acceptor_variant&intron_variant|HIGH|JRK|ENSG0000023
processed_transcript			G|splice_acceptor_variant&intron_variant|HIGH|JRK|ENSG0000023
protein_coding			A|stop_gained|HIGH|PSCA|ENSG00000167653|transcript|ENST000
protein_coding			G|structural_interaction_variant|HIGH|CYP11B2|ENSG0000017914
protein_coding			T|structural_interaction_variant|HIGH|CYP11B2|ENSG00000179142
protein_coding			C|structural_interaction_variant|HIGH|CYP11B2|ENSG00000179142
protein_co	1	1.00,(ZFP4:	C|start_lost|HIGH|RP13-582O9.6|ENSG00000264668|transcript|EN
nonsense_	19	0.05	AG|frameshift_variant|HIGH|TOP1MT|ENSG00000184428|transcrip
protein_coding			AG|frameshift_variant|HIGH|ZC3H3|ENSG00000014164|transcript|
protein_co	2	0.5	T|stop_gained|HIGH|ZC3H3|ENSG00000014164|transcript|ENST00
protein_coding			G|structural_interaction_variant|HIGH|PLEC|ENSG00000178209|int
protein_coding			T|structural_interaction_variant|HIGH|SHARPIN|ENSG0000017952€
protein_coding			A|structural_interaction_variant|HIGH|SHARPIN|ENSG0000017952€
nonsense_	14	0.07	CTT|splice_acceptor_variant&intron_variant|HIGH|HSF1|ENSG0000
nonsense_	14	0.07	CTT|splice_acceptor_variant&intron_variant|HIGH|HSF1|ENSG0000
protein_co	6	0.17	C|frameshift_variant|HIGH|KIFC2|ENSG00000167702|transcript|EN
protein_co	18	0.06	G|frameshift_variant|HIGH|ZNF7|ENSG00000147789|transcript|EN

protein_coding			G|stop_lost|HIGH|FOXD4|ENSG00000170122|transcript|ENST0000
protein_co	1	1	CCG|frameshift_variant|HIGH|FOXD4|ENSG00000170122|transcrip
protein_co	1	1	G|frameshift_variant|HIGH|FOXD4|ENSG00000170122|transcript|E
antisense			C|splice_acceptor_variant&intron_variant|HIGH|RP11-165F24.3|EN
antisense			G|splice_donor_variant&splice_region_variant&intron_variant&non_
antisense			G|splice_donor_variant&splice_region_variant&intron_variant&non_
antisense			G|splice_donor_variant&splice_region_variant&intron_variant&non_
antisense			G|splice_donor_variant&splice_region_variant&intron_variant&non_
antisense			G|splice_donor_variant&splice_region_variant&intron_variant&non_
antisense			G|splice_donor_variant&splice_region_variant&intron_variant&non_
protein_co	1	1	C|splice_acceptor_variant&splice_region_variant&intron_variant|HI
protein_co	1	1	C|splice_acceptor_variant&splice_region_variant&intron_variant|HI
protein_co	1	1	C|splice_acceptor_variant&splice_region_variant&intron_variant|HI
lincRNA			C|splice_acceptor_variant&intron_variant|HIGH|RFX3-AS1|ENSG00
processed_pseudogene			C|splice_acceptor_variant&splice_region_variant&intron_variant|HI
processed_pseudogene			CT|splice_region_variant&intron_variant|LOW|HNRNPA1P41|ENSG
processed_pseudogene			CTTTTTTTTT|intron_variant|MODIFIER|HNRNPA1P41|ENSG0000022
processed_pseudogene			CTTTTTTTTTT|intron_variant|MODIFIER|HNRNPA1P41|ENSG000002
protein_coding			T|stop_gained|HIGH|INSL6|ENSG00000120210|transcript|ENST000
protein_co	6	0.5	T|stop_gained|HIGH|ERMP1|ENSG00000099219|transcript|ENST00
protein_coding			T|frameshift_variant|HIGH|TPD52L3|ENSG00000170777|transcript
processed_transcript			G|splice_donor_variant&intron_variant|HIGH|KDM4C|ENSG000001
protein_coding			C|structural_interaction_variant|HIGH|MPDZ|ENSG00000107186|in
protein_co	9	0.22	T|stop_gained|HIGH|TTC39B|ENSG00000155158|transcript|ENST00
protein_co	4	0.25	T|stop_gained|HIGH|CNTLN|ENSG00000044459|transcript|ENST00
protein_co	4	0.25	T|splice_acceptor_variant&intron_variant|HIGH|CNTLN|ENSG00000
protein_coding			G|frameshift_variant|HIGH|ADAMTSL1|ENSG00000178031|transcri
protein_coding			T|stop_gained|HIGH|IFNA10|ENSG00000186803|transcript|ENST00
protein_co	1	1	AT|frameshift_variant|HIGH|IFNA17|ENSG00000234829|transcript|
protein_coding			A|stop_gained|HIGH|IFNA5|ENSG00000147873|transcript|ENST000
protein_coding			A|stop_gained|HIGH|IFNE|ENSG00000184995|transcript|ENST0000
protein_coding			A|structural_interaction_variant|HIGH|MTAP|ENSG00000099810|in
protein_coding			T|structural_interaction_variant|HIGH|MTAP|ENSG00000099810|in
sense_intronic			G|splice_donor_variant&splice_region_variant&intron_variant&non_
lincRNA			T|splice_acceptor_variant&intron_variant|HIGH|RP11-56F10.3|ENS
protein_co	1	1	GTGTT|frameshift_variant|HIGH|IFNK|ENSG00000147896|transcrip
protein_coding			T|structural_interaction_variant|HIGH|DDX58|ENSG00000107201|i
protein_coding			A|structural_interaction_variant|HIGH|B4GALT1|ENSG0000008606
protein_coding			C|stop_gained|HIGH|KIAA1161|ENSG00000164976|transcript|ENST
protein_co	2	0.5	C|splice_donor_variant&intron_variant|HIGH|KIAA1161|ENSG00000
retained_intron			A|splice_acceptor_variant&intron_variant|HIGH|GALT|ENSG000002
protein_co	3	1	G|frameshift_variant|HIGH|FAM205C|ENSG00000187791|transcrip
protein_co	10	0.1	T|splice_acceptor_variant&splice_region_variant&intron_variant|HI
protein_co	6	0.33	ATAGT|frameshift_variant&stop_gained|HIGH|FAM166B|ENSG0000
protein_co	8	0.13	AG|frameshift_variant|HIGH|TPM2|ENSG00000198467|transcript|E
antisense			C|splice_acceptor_variant&intron_variant|HIGH|RP11-112J3.15|ENS
lincRNA			G|splice_acceptor_variant&intron_variant|HIGH|RP11-112J3.16|EN

protein_co	8	0.13	T|splice_donor_variant&intron_variant|HIGH|TMEM8B|ENSG0000(
polymorphic_pseudogene			C|stop_lost|HIGH|OR13C7|ENSG00000243641|transcript|ENST000(
polymorphic_pseudogene			T|stop_lost|HIGH|OR13C7|ENSG00000243641|transcript|ENST000(
polymorph	1	1	C|frameshift_variant|HIGH|OR13C7|ENSG00000243641|transcript|
protein_co	2	0.5	A|splice_donor_variant&intron_variant|HIGH|ALDH1B1|ENSG0000(
unitary_pseudogene			T|splice_acceptor_variant&splice_region_variant&intron_variant|HI
protein_co	15	0.4	C|frameshift_variant|HIGH|CNTNAP3B|ENSG00000154529|transcri
protein_co	15	0.2	G|frameshift_variant|HIGH|CNTNAP3B|ENSG00000154529|transcri
protein_co	3	0.33	A|frameshift_variant|HIGH|ANKRD20A4|ENSG00000172014|transc
protein_coding			A|frameshift_variant|HIGH|FOXD4L5|ENSG00000204779|transcript
protein_co	1	1	G|frameshift_variant|HIGH|FOXD4L5|ENSG00000204779|transcript
protein_co	33	0.18	G|frameshift_variant|HIGH|CBWD5|ENSG00000147996|transcript|
protein_co	7	0.14	T|frameshift_variant|HIGH|ANKRD20A3|ENSG00000276203|transc
protein_co	7	0.14	A|frameshift_variant|HIGH|ANKRD20A1|ENSG00000260691|transc
protein_co	19	0.16	GA|frameshift_variant|HIGH|CBWD3|ENSG00000196873|transcript
lincRNA			C|splice_donor_variant&splice_region_variant&intron_variant&non_
lincRNA			C|splice_donor_variant&splice_region_variant&intron_variant&non_
lincRNA			G|splice_donor_variant&splice_region_variant&intron_variant&non_
protein_coding			T|structural_interaction_variant|HIGH|GDA|ENSG00000119125|int
protein_coding			C|structural_interaction_variant|HIGH|NMRK1|ENSG00000106733|
protein_coding			A|frameshift_variant|HIGH|PCSK5|ENSG00000099139|transcript|E
protein_coding			AAATGGAATGGAATGG|frameshift_variant|HIGH|PCSK5|ENSG0000(
protein_coding			AAATGGAATGGAATGG|frameshift_variant|HIGH|PCSK5|ENSG0000(
protein_coding			G|frameshift_variant|HIGH|PCSK5|ENSG00000099139|transcript|E
protein_coding			G|frameshift_variant|HIGH|PCSK5|ENSG00000099139|transcript|E
protein_coding			TG|frameshift_variant|HIGH|PCSK5|ENSG00000099139|transcript|
protein_coding			TG|frameshift_variant|HIGH|PCSK5|ENSG00000099139|transcript|
protein_coding			AATGGAATGGAATGG|frameshift_variant|HIGH|PCSK5|ENSG000000
protein_coding			AATGGAATGGAATGG|frameshift_variant|HIGH|PCSK5|ENSG000000
protein_coding			AATGGAATGGAATGG|frameshift_variant|HIGH|PCSK5|ENSG000000
protein_coding			AATGGAATGGAATGG|frameshift_variant|HIGH|PCSK5|ENSG000000
protein_coding			TC|frameshift_variant|HIGH|PCSK5|ENSG00000099139|transcript|I
protein_coding			GAATC|frameshift_variant&stop_gained|HIGH|PCSK5|ENSG000000
protein_coding			GAATC|frameshift_variant&stop_gained|HIGH|PCSK5|ENSG000000
protein_coding			GGAATC|frameshift_variant|HIGH|PCSK5|ENSG00000099139|trans
protein_coding			A|stop_gained|HIGH|PCSK5|ENSG00000099139|transcript|ENST000
protein_coding			C|stop_lost|HIGH|PCSK5|ENSG00000099139|transcript|ENST0000C
protein_coding			C|stop_lost|HIGH|PCSK5|ENSG00000099139|transcript|ENST0000C
processed_transcript			C|splice_acceptor_variant&intron_variant|HIGH|PRUNE2|ENSG000(
protein_coding			T|stop_lost|HIGH|VPS13A|ENSG00000197969|transcript|ENST0000
processed_transcript			A|splice_donor_variant&intron_variant|HIGH|CEP78|ENSG0000014
protein_coding			G|structural_interaction_variant|HIGH|PSAT1|ENSG00000135069|i
antisense			T|splice_donor_variant&intron_variant|HIGH|RP11-575L7.2|ENSG0
antisense			C|splice_donor_variant&intron_variant|HIGH|RP11-575L7.4|ENSG0
processed_pseudogene			C|splice_acceptor_variant&splice_region_variant&intron_variant&n
processed_pseudogene			CA|splice_region_variant&non_coding_transcript_exon_variant|LOV
processed_pseudogene			CAAA|splice_region_variant&non_coding_transcript_exon_variant|L

protein_co	3	0.67	CTTT|splice_acceptor_variant&intron_variant|HIGH|NAA35|ENSG00
protein_co	3	0.67	CTTT|splice_acceptor_variant&intron_variant|HIGH|NAA35|ENSG00
protein_coding			A|structural_interaction_variant|HIGH|DAPK1|ENSG00000196730|i
protein_coding			T|structural_interaction_variant|HIGH|DAPK1|ENSG00000196730|i
protein_coding			C|stop_lost|HIGH|CTSL|ENSG00000135047|transcript|ENST000003
protein_coding			C|structural_interaction_variant|HIGH|CKS2|ENSG00000123975|int
protein_coding			T|structural_interaction_variant|HIGH|SEMA4D|ENSG00000187764
lincRNA			T|splice_donor_variant&intron_variant|HIGH|RP5-1050E16.2|ENSG
protein_coding			A|structural_interaction_variant|HIGH|SYK|ENSG00000165025|inte
protein_coding			C|structural_interaction_variant|HIGH|SYK|ENSG00000165025|inte
protein_coding			A|structural_interaction_variant|HIGH|SYK|ENSG00000165025|inte
protein_coding			T|structural_interaction_variant|HIGH|SYK|ENSG00000165025|inte
protein_coding			C|structural_interaction_variant|HIGH|SYK|ENSG00000165025|inte
protein_coding			A|structural_interaction_variant|HIGH|ROR2|ENSG00000169071|in
protein_co	4	0.5	T|splice_donor_variant&intron_variant|HIGH|ZNF484|ENSG0000001
unprocessed_pseudogene			C|splice_acceptor_variant&intron_variant|HIGH|ALOX15P2|ENSG00
nonsense_mediated_decay			T|structural_interaction_variant|HIGH|CARD19|ENSG00000165233
protein_co	8	0.13	G|start_lost|HIGH|ERCC6L2|ENSG00000182150|transcript|ENST00
protein_coding			C|stop_lost&splice_region_variant|HIGH|MFSD14C|ENSG00000196
protein_coding			A|protein_protein_contact|HIGH|NCBP1|ENSG00000136937|intera
protein_co	2	1	A|stop_gained|HIGH|HEMGN|ENSG00000136929|transcript|ENST0
protein_coding			G|stop_lost|HIGH|NANS|ENSG00000095380|transcript|ENST00000
antisense			CTAGGGAGG|splice_acceptor_variant&intron_variant|HIGH|RP11-9
processed_transcript			G|splice_acceptor_variant&splice_region_variant&intron_variant&n
protein_co	4	0.25	A|splice_donor_variant&intron_variant|HIGH|CYLC2|ENSG0000015
protein_co	4	0.25	A|splice_donor_variant&intron_variant|HIGH|CYLC2|ENSG0000015
protein_coding			G|frameshift_variant|HIGH|OR13C5|ENSG00000277556|transcript|
protein_co	1	1	A|frameshift_variant|HIGH|OR13C5|ENSG00000277556|transcript|
protein_co	1	1	T|frameshift_variant|HIGH|OR13C2|ENSG00000276119|transcript|
protein_co	1	1	A|frameshift_variant|HIGH|OR13C2|ENSG00000276119|transcript|
protein_co	3	0.67	A|splice_donor_variant&intron_variant|HIGH|NIPSNAP3B|ENSG000
protein_co	12	0.08	CCCA|frameshift_variant|HIGH|ZNF462|ENSG00000148143|transcr
protein_co	5	0.2	C|frameshift_variant|HIGH|RAD23B|ENSG00000119318|transcript|
protein_co	5	0.2	C|frameshift_variant|HIGH|RAD23B|ENSG00000119318|transcript|
protein_co	1	1	G|start_lost|HIGH|ACTL7B|ENSG00000148156|transcript|ENST000
protein_coding			A|structural_interaction_variant|HIGH|PTPN3|ENSG00000070159|i
processed_pseudogene			G|splice_acceptor_variant&splice_region_variant&intron_variant|HI
processed_pseudogene			GTTTTTTTTTT|intron_variant|MODIFIER|YBX1P6|ENSG0000021353
processed_pseudogene			GTTTTTTTTTTT|intron_variant|MODIFIER|YBX1P6|ENSG000002135
processed_pseudogene			GTTTTTTTTTTTT|intron_variant|MODIFIER|YBX1P6|ENSG00000213
protein_co	2	0.5	A|frameshift_variant|HIGH|C9orf152|ENSG00000188959|transcript
protein_co	5	0.4	TA|splice_donor_variant&intron_variant|HIGH|C9orf84|ENSG00000
antisense			T|splice_acceptor_variant&splice_region_variant&intron_variant&n
protein_coding			A|stop_gained|HIGH|ZNF883|ENSG00000228623|transcript|ENST0
protein_coding			A|structural_interaction_variant|HIGH|HDHD3|ENSG00000119431|
protein_coding			T|structural_interaction_variant|HIGH|ALAD|ENSG00000148218|in
protein_coding			G|structural_interaction_variant|HIGH|ALAD|ENSG00000148218|in

protein_co	3	0.67	A|splice_donor_variant&intron_variant|HIGH|C9orf43|ENSG000001
protein_coding			C|structural_interaction_variant|HIGH|TNFSF15|ENSG00000181634
antisense			A|splice_donor_variant&intron_variant|HIGH|RP11-67K19.3|ENSG(
nonsense_	19	0.11	C|splice_acceptor_variant&intron_variant|HIGH|CDK5RAP2|ENSG00
nonsense_	19	0.11	C|splice_acceptor_variant&intron_variant|HIGH|CDK5RAP2|ENSG00
processed_transcript			A|splice_donor_variant&intron_variant|HIGH|GSN|ENSG000001481
protein_coding			T|stop_gained|HIGH|OR1J1|ENSG00000136834|transcript|ENST00(
protein_co	1	1	AT|frameshift_variant|HIGH|OR1J2|ENSG00000197233|transcript|
polymorphic_pseudogene			A|stop_gained|HIGH|OR1B1|ENSG00000280094|transcript|ENST00
polymorphic_pseudogene			CA|frameshift_variant|HIGH|OR1B1|ENSG00000280094|transcript|
protein_co	10	0.1	C|splice_acceptor_variant&intron_variant|HIGH|RC3H2|ENSG00000(
nonsense_mediated_decay			C|frameshift_variant|HIGH|RABGAP1|ENSG00000011454|transcript
nonsense_mediated_decay			C|frameshift_variant|HIGH|RABGAP1|ENSG00000011454|transcript
protein_coding			AG|frameshift_variant|HIGH|CRB2|ENSG00000148204|transcript|E
protein_co	3	0.67	C|frameshift_variant|HIGH|OLFML2A|ENSG00000185585|transcript
protein_co	3	0.33	T|splice_donor_variant&intron_variant|HIGH|ARPC5L|ENSG000001
protein_coding			A|structural_interaction_variant|HIGH|MAPKAP1|ENSG0000011948
protein_coding			G|structural_interaction_variant|HIGH|MVB12B|ENSG0000019681
protein_coding			C|stop_gained|HIGH|PTRH1|ENSG00000187024|transcript|ENST00
protein_coding			T|structural_interaction_variant|HIGH|SH2D3C|ENSG00000095370
protein_coding			A|protein_protein_contact|HIGH|CDK9|ENSG00000136807|interac
protein_coding			A|structural_interaction_variant|HIGH|DNM1|ENSG00000106976|i
nonsense_mediated_decay			A|stop_gained|HIGH|DNM1|ENSG00000106976|transcript|ENST00
processed_transcript			G|splice_acceptor_variant&intron_variant|HIGH|SPTAN1|ENSG0000(
protein_co	5	0.2	AGGGGCCTGGTGCT|frameshift_variant|HIGH|ZDHHC12|ENSG00000(
protein_coding			C|structural_interaction_variant|HIGH|KYAT1|ENSG00000171097|i
protein_co	10	0.1	A|frameshift_variant|HIGH|KYAT1|ENSG00000171097|transcript|E
protein_co	10	0.1	A|frameshift_variant|HIGH|KYAT1|ENSG00000171097|transcript|E
protein_coding			G|structural_interaction_variant|HIGH|PHYHD1|ENSG00000175287
protein_co	1	1	AT|frameshift_variant&start_lost|HIGH|DOLK|ENSG00000175283|t
protein_coding			G|stop_lost|HIGH|PTPA|ENSG00000119383|transcript|ENST00000
protein_co	6	0.17	A|stop_gained|HIGH|HMCN2|ENSG00000148357|transcript|ENST0
protein_co	6	0.17	GA|frameshift_variant|HIGH|HMCN2|ENSG00000148357|transcript
protein_coding			T|structural_interaction_variant|HIGH|ASS1|ENSG00000130707|int
protein_coding			A|structural_interaction_variant|HIGH|ASS1|ENSG00000130707|int
protein_coding			G|structural_interaction_variant|HIGH|ABL1|ENSG00000097007|in
protein_co	27	0.11	CTTT|splice_acceptor_variant&intron_variant|HIGH|NUP214|ENSG(
protein_co	27	0.11	CTTT|splice_acceptor_variant&intron_variant|HIGH|NUP214|ENSG(
protein_co	27	0.11	CTTT|splice_acceptor_variant&intron_variant|HIGH|NUP214|ENSG(
antisense			A|splice_donor_variant&intron_variant|HIGH|RP11-544A12.4|ENSG
protein_co	27	0.04	G|start_lost|HIGH|NUP214|ENSG00000126883|transcript|ENST00(
nonsense_	27	0.04	G|splice_acceptor_variant&intron_variant|HIGH|NUP214|ENSG000(
protein_co	16	0.13	T|stop_gained|HIGH|POMT1|ENSG00000130714|transcript|ENST00
protein_coding			T|structural_interaction_variant|HIGH|NTNG2|ENSG00000196358|
protein_coding			T|structural_interaction_variant|HIGH|NTNG2|ENSG00000196358|
protein_coding			T|structural_interaction_variant|HIGH|NTNG2|ENSG00000196358|
)188523			T|gene_fusion|HIGH|CFAP77&RNU5D-2P|ENSG00000188523&ENSG

protein_coding			C|structural_interaction_variant|HIGH|RALGDS|ENSG00000160271
protein_coding			T|stop_gained|HIGH|GBGT1|ENSG00000148288|transcript|ENST00
protein_co	5	0.8	C|frameshift_variant|HIGH|OBP2B|ENSG00000171102|transcript|E
protein_co	3	0.33	C|frameshift_variant&splice_region_variant|HIGH|ABO|ENSG00000
protein_co	3	0.33	T|stop_gained|HIGH|ABO|ENSG00000175164|transcript|ENST0000
protein_co	3	0.67	TC|splice_acceptor_variant&splice_donor_variant&intron_variant|H
protein_coding			A|structural_interaction_variant|HIGH|ADAMTS13|ENSG0000016030
protein_coding			G|structural_interaction_variant|HIGH|WDR5|ENSG00000196363|i
protein_coding			A|structural_interaction_variant|HIGH|RXRA|ENSG00000186350|in
protein_co	12	0.08	GAA|frameshift_variant|HIGH|OLFM1|ENSG00000130558|transcrip
protein_co	12	0.08	GCA|frameshift_variant|HIGH|OLFM1|ENSG00000130558|transcrip
processed_pseudogene			A|splice_donor_variant&intron_variant|HIGH|SOCS5P2|ENSG00000
processed_transcript			C|splice_acceptor_variant&intron_variant|HIGH|MRPS2|ENSG0000
protein_co	7	0.29	GC|frameshift_variant|HIGH|OBP2A|ENSG00000122136|transcript|
protein_co	2	1	C|frameshift_variant|HIGH|GLT6D1|ENSG00000204007|transcript|
protein_co	27	0.41	CGCCCTGCCCT|splice_acceptor_variant&intron_variant|HIGH|KCNT
protein_co	27	0.41	CGCCCTGCCCT|splice_acceptor_variant&intron_variant|HIGH|KCNT
protein_co	6	0.17	T|frameshift_variant|HIGH|GPSM1|ENSG00000160360|transcript|E
protein_coding			A|stop_gained|HIGH|GPSM1|ENSG00000160360|transcript|ENST0
protein_co	7	0.43	G|splice_donor_variant&intron_variant|HIGH|CARD9|ENSG0000018
protein_coding			A|structural_interaction_variant|HIGH|INPP5E|ENSG00000148384|
protein_coding			A|structural_interaction_variant|HIGH|INPP5E|ENSG00000148384|
protein_coding			G|structural_interaction_variant|HIGH|INPP5E|ENSG00000148384|
protein_co	8	0.38	A|stop_gained|HIGH|LCN10|ENSG00000187922|transcript|ENST00
protein_coding			C|structural_interaction_variant|HIGH|LCN15|ENSG00000177984|i
protein_coding			T|structural_interaction_variant|HIGH|PHPT1|ENSG00000054148|i
protein_co	4	0.5	GC|splice_acceptor_variant&intron_variant|HIGH|C8G|ENSG00000
processed_transcript			G|splice_acceptor_variant&intron_variant|HIGH|LCN12|ENSG00000
processed_transcript			G|splice_acceptor_variant&intron_variant|HIGH|LCN12|ENSG00000
protein_co	6	0.17	A|stop_gained|HIGH|NPDC1|ENSG00000107281|transcript|ENST0
protein_coding			C|structural_interaction_variant|HIGH|MAN1B1|ENSG0000017723
protein_co	3	0.67	A|splice_donor_variant&intron_variant|HIGH|FAM166A|ENSG0000
protein_coding			CT|frameshift_variant|HIGH|PNPLA7|ENSG00000130653|transcript
processed_transcript			T|splice_acceptor_variant&splice_region_variant&intron_variant&n
protein_coding			A|stop_lost|HIGH|IDI2|ENSG00000148377|transcript|ENST000002
protein_coding			T|stop_gained|HIGH|IDI2|ENSG00000148377|transcript|ENST0000
protein_coding			T|structural_interaction_variant|HIGH|IDI2|ENSG00000148377|inte
protein_co	5	0.4	A|splice_donor_variant&intron_variant|HIGH|IDI1|ENSG0000067
protein_co	6	0.17	C|stop_lost|HIGH|WDR37|ENSG00000047056|transcript|ENST0000
)282390			A|gene_fusion|HIGH|ADARB2&RP11-29A19.2|ENSG00000185736&
protein_coding			T|structural_interaction_variant|HIGH|PFKP|ENSG00000067057|int
protein_coding			T|structural_interaction_variant|HIGH|PITRM1|ENSG00000107959|
protein_coding			C|structural_interaction_variant|HIGH|PITRM1|ENSG00000107959|
protein_coding			G|stop_lost|HIGH|PITRM1|ENSG00000107959|transcript|ENST0000
protein_coding			G|structural_interaction_variant|HIGH|PITRM1|ENSG00000107959
protein_coding			G|structural_interaction_variant|HIGH|PITRM1|ENSG00000107959
protein_co	11	0.27	TGCACGCTAGGGAAGAGAGAGGAATG|frameshift_variant&stop_gain

protein_coding			T|stop_gained|HIGH|AKR1E2|ENSG00000165568|transcript|ENST0
protein_coding			A|structural_interaction_variant|HIGH|AKR1C1|ENSG00000187134
processed_transcript			G|splice_donor_variant&intron_variant|HIGH|AKR1C2|ENSG000001
protein_co	11	0.18	T|start_lost|HIGH|AKR1C3|ENSG00000196139|transcript|ENST000
protein_coding			C|structural_interaction_variant|HIGH|NET1|ENSG00000173848|in
processed_pseudogene			C|splice_acceptor_variant&splice_region_variant&intron_variant|HI
processed_pseudogene			C|splice_acceptor_variant&splice_region_variant&intron_variant|HI
processed_pseudogene			C|splice_acceptor_variant&splice_region_variant&intron_variant|HI
protein_co	4	0.75	T|frameshift_variant|HIGH|ANKRD16|ENSG00000134461|transcript
protein_coding			T|frameshift_variant|HIGH|IL15RA|ENSG00000134470|transcript|E
protein_coding			T|structural_interaction_variant|HIGH|PRKCQ|ENSG00000065675|i
protein_coding			A|structural_interaction_variant|HIGH|PRKCQ|ENSG00000065675|i
protein_coding			T|structural_interaction_variant|HIGH|PRKCQ|ENSG00000065675|i
processed_transcript			C|splice_acceptor_variant&splice_region_variant&intron_variant|HI
processed_transcript			C|splice_acceptor_variant&splice_region_variant&intron_variant|HI
processed_transcript			C|splice_acceptor_variant&splice_region_variant&intron_variant|HI
processed_transcript			C|splice_acceptor_variant&splice_region_variant&intron_variant|HI
protein_coding			C|structural_interaction_variant|HIGH|ECHDC3|ENSG00000134463
protein_coding			A|structural_interaction_variant|HIGH|ECHDC3|ENSG00000134463
protein_coding			T|structural_interaction_variant|HIGH|ECHDC3|ENSG00000134463
protein_coding			A|structural_interaction_variant|HIGH|ECHDC3|ENSG00000134463
protein_coding			T|structural_interaction_variant|HIGH|ECHDC3|ENSG00000134463
protein_coding			G|structural_interaction_variant|HIGH|SEPHS1|ENSG00000086475|
protein_coding			C|structural_interaction_variant|HIGH|SEPHS1|ENSG00000086475|
lincRNA			A|splice_donor_variant&intron_variant|HIGH|RP11-295P9.3|ENSG0
lincRNA			CTT|splice_acceptor_variant&intron_variant|HIGH|RP11-461K13.1|
lincRNA			CTTT|splice_acceptor_variant&intron_variant|HIGH|RP11-461K13.1
lincRNA			C|splice_region_variant&intron_variant|LOW|RP11-461K13.1|ENSG
protein_coding			A|structural_interaction_variant|HIGH|TRDMT1|ENSG00000107614
protein_coding			A|structural_interaction_variant|HIGH|TRDMT1|ENSG00000107614
protein_co	16	0.31	CTTTTTTTTTTTTT|splice_acceptor_variant&intron_variant|HIGH|CA
protein_co	16	0.31	CTTTTTTTTTTTTT|splice_acceptor_variant&intron_variant|HIGH|CA
protein_co	16	0.31	CTTTTTTTTTTTTT|splice_acceptor_variant&intron_variant|HIGH|CA
protein_co	16	0.31	CTTTTTTTTTTTTT|splice_acceptor_variant&intron_variant|HIGH|CA
protein_co	16	0.31	TTTG|splice_acceptor_variant&intron_variant|HIGH|CACNB2|ENSG
protein_co	16	0.31	TTTG|splice_acceptor_variant&intron_variant|HIGH|CACNB2|ENSG
protein_co	16	0.31	TTTG|splice_acceptor_variant&intron_variant|HIGH|CACNB2|ENSG
protein_co	16	0.31	TTTG|splice_acceptor_variant&intron_variant|HIGH|CACNB2|ENSG
protein_co	16	0.06	T|splice_acceptor_variant&intron_variant|HIGH|CACNB2|ENSG000
protein_co	2	1	A|frameshift_variant|HIGH|C10orf113|ENSG00000204683|transcri
protein_co	17	0.12	CTT|splice_acceptor_variant&intron_variant|HIGH|MLLT10|ENSG00
protein_co	17	0.12	CTT|splice_acceptor_variant&intron_variant|HIGH|MLLT10|ENSG00
processed_pseudogene			T|splice_acceptor_variant&splice_region_variant&intron_variant|HI
processed_pseudogene			T|splice_acceptor_variant&splice_region_variant&intron_variant|HI
processed_pseudogene			T|splice_acceptor_variant&splice_region_variant&intron_variant|HI
processed_pseudogene			T|splice_acceptor_variant&splice_region_variant&intron_variant|HI
processed_pseudogene			T|splice_acceptor_variant&splice_region_variant&intron_variant|HI

protein_coding			T|structural_interaction_variant|HIGH|GAD2|ENSG00000136750|in
unitary_pseudogene			A|splice_donor_variant&intron_variant|HIGH|RP13-16H11.9|ENSG(
protein_coding			C|structural_interaction_variant|HIGH|MAP3K8|ENSG00000107968
transcribed_unprocessed_pseudo			T|splice_acceptor_variant&intron_variant|HIGH|SVILP1|ENSG00000(
lincRNA			G|splice_acceptor_variant&splice_region_variant&intron_variant|HI
protein_co	9	0.22	C|start_lost|HIGH|ZNF438|ENSG00000183621|transcript|ENST0000(
protein_co	9	1	CT|frameshift_variant|HIGH|CCDC7|ENSG00000216937|transcript|
protein_co	9	0.22	T|splice_donor_variant&intron_variant|HIGH|CCDC7|ENSG0000021
protein_co	3	1	T|frameshift_variant|HIGH|AL356053.1|ENSG00000150076|transcr
protein_coding			C|structural_interaction_variant|HIGH|NRP1|ENSG00000099250|in
protein_coding			A|structural_interaction_variant|HIGH|NRP1|ENSG00000099250|in
protein_co	5	0.8	T|stop_gained|HIGH|ANKRD30A|ENSG00000148513|transcript|ENS
protein_co	5	0.8	A|frameshift_variant|HIGH|ANKRD30A|ENSG00000148513|transcri
transcribed_unprocessed_pseudo			T|splice_donor_variant&splice_region_variant&intron_variant&non_
transcribed_unprocessed_pseudo			C|splice_donor_variant&splice_region_variant&intron_variant&non_
transcribed_unprocessed_pseudo			C|splice_donor_variant&splice_region_variant&intron_variant&non_
protein_coding			T|structural_interaction_variant|HIGH|RET|ENSG00000165731|inte
protein_coding			C|frameshift_variant|HIGH|RASGEF1A|ENSG00000198915|transcrip
protein_co	2	1	A|frameshift_variant|HIGH|C10orf142|ENSG00000277288|transcrip
processed_transcript			G|splice_donor_variant&intron_variant|HIGH|CEP164P1|ENSG0000(
processed_transcript			C|splice_acceptor_variant&intron_variant|HIGH|CEP164P1|ENSG00
protein_coding			C|frameshift_variant|HIGH|AGAP4|ENSG00000188234|transcript|E
unprocessed_pseudogene			T|splice_donor_variant&intron_variant|HIGH|ANTXRLP1|ENSG0000(
unprocessed_pseudogene			A|splice_acceptor_variant&splice_region_variant&intron_variant&n
retained_intron			A|splice_donor_variant&intron_variant|HIGH|ANTXRL|ENSG000002
protein_co	6	0.83	TC|splice_acceptor_variant&intron_variant|HIGH|SYT15|ENSG0000(
protein_co	1	1	C|frameshift_variant|HIGH|AGAP9|ENSG00000204172|transcript|E
unprocessed_pseudogene			C|splice_acceptor_variant&intron_variant|HIGH|FAM35DP|ENSG00
protein_co	14	0.21	C|frameshift_variant|HIGH|FRMPD2|ENSG00000170324|transcript
processed_pseudogene			C|splice_acceptor_variant&intron_variant|HIGH|RP11-140C5.3|ENS
processed_pseudogene			CA|splice_acceptor_variant&intron_variant|HIGH|RP11-140C5.3|EN
protein_coding			C|structural_interaction_variant|HIGH|CHAT|ENSG00000070748|in
protein_coding			A|stop_gained|HIGH|C10orf53|ENSG00000178645|transcript|ENST
protein_co	4	0.25	C|splice_donor_variant&splice_region_variant&intron_variant|HIGH
protein_coding			G|splice_acceptor_variant&splice_region_variant&3_prime_UTR_va
protein_coding			G|splice_acceptor_variant&splice_region_variant&3_prime_UTR_va
protein_coding			G|splice_acceptor_variant&splice_region_variant&3_prime_UTR_va
protein_coding			G|splice_acceptor_variant&splice_region_variant&3_prime_UTR_va
protein_coding			G|splice_acceptor_variant&splice_region_variant&3_prime_UTR_va
protein_co	4	0.25	CTGTGTC|splice_donor_variant&intron_variant|HIGH|C10orf53|ENS
protein_co	4	0.25	CTGTGTC|splice_donor_variant&intron_variant|HIGH|C10orf53|ENS
protein_co	4	0.25	CTGTGTC|splice_donor_variant&intron_variant|HIGH|C10orf53|ENS
protein_coding			AC|frameshift_variant|HIGH|AGAP6|ENSG00000204149|transcript|
protein_co	3	0.67	A|frameshift_variant|HIGH|AGAP6|ENSG00000204149|transcript|E
protein_co	3	1	C|frameshift_variant|HIGH|AGAP6|ENSG00000204149|transcript|E
unprocessed_pseudogene			G|splice_acceptor_variant&intron_variant|HIGH|NUTM2HP|ENSG0(
protein_coding			T|structural_interaction_variant|HIGH|PRKG1|ENSG00000185532|i

protein_coding			G|structural_interaction_variant|HIGH|PRKG1|ENSG00000185532|i
lincRNA			A|splice_acceptor_variant&intron_variant|HIGH|LINC01468|ENSG0
antisense			G|splice_acceptor_variant&intron_variant|HIGH|RP11-443O13.3|EN
protein_co	9	0.11	G|splice_acceptor_variant&splice_donor_variant&intron_variant|HI
protein_co	9	0.11	T|splice_acceptor_variant&intron_variant|HIGH|CDK1|ENSG000001
protein_co	5	0.2	CATGGGTCTGAAAAAAGAATGAAAATTACTTCTTACAAATA|frameshift_
protein_co	3	0.33	A|stop_gained|HIGH|RTKN2|ENSG00000182010|transcript|ENST00
protein_coding			A|structural_interaction_variant|HIGH|JMJD1C|ENSG00000171988|
lincRNA			GCTTCTTCCTGCTGTTAAACCAAAGAAACACTCTCAGCA|splice_donor_v
lincRNA			A|splice_donor_variant&intron_variant|HIGH|LINC01515|ENSG0000
lincRNA			C|splice_donor_variant&intron_variant|HIGH|LINC01515|ENSG0000
protein_coding			C|structural_interaction_variant|HIGH|SIRT1|ENSG00000096717|in
protein_co	18	0.28	T|splice_acceptor_variant&splice_region_variant&intron_variant|HI
protein_co	3	0.33	G|start_lost|HIGH|C10orf35|ENSG00000171224|transcript|ENST00
protein_co	1	1	GCCCCCC|splice_acceptor_variant&intron_variant|HIGH|PALD1|EN
protein_co	1	1	G|splice_region_variant&intron_variant|LOW|PALD1|ENSG0000010
protein_co	1	1	GC|splice_region_variant&intron_variant|LOW|PALD1|ENSG000001
protein_co	1	1	GCC|splice_region_variant&intron_variant|LOW|PALD1|ENSG00000
protein_co	1	1	GCCC|splice_region_variant&intron_variant|LOW|PALD1|ENSG0000
protein_co	1	1	GCCCC|splice_region_variant&intron_variant|LOW|PALD1|ENSG000
protein_coding			T|structural_interaction_variant|HIGH|SGPL1|ENSG00000166224|ir
protein_co	19	0.11	C|splice_donor_variant&intron_variant|HIGH|ASCC1|ENSG0000013
protein_co	11	0.09	C|splice_acceptor_variant&intron_variant|HIGH|MICU1|ENSG00000
nonsense_	11	0.09	C|frameshift_variant|HIGH|MCU|ENSG00000156026|transcript|EN
protein_co	11	0.09	ATT|splice_acceptor_variant&intron_variant|HIGH|MCU|ENSG0000
protein_co	11	0.09	ATT|splice_acceptor_variant&intron_variant|HIGH|MCU|ENSG0000
protein_co	11	0.09	A|splice_donor_variant&intron_variant|HIGH|MCU|ENSG00000156
protein_coding			G|frameshift_variant&splice_region_variant|HIGH|NUDT13|ENSG00
protein_co	8	0.13	T|start_lost&conservative_inframe_deletion|HIGH|FAM149B1|ENS
protein_co	3	0.67	TG|frameshift_variant|HIGH|SYNPO2L|ENSG00000166317|transcrip
protein_coding			A|structural_interaction_variant|HIGH|DUSP13|ENSG00000079393
protein_co	15	0.07	C|frameshift_variant|HIGH|DUSP13|ENSG00000079393|transcript|
lincRNA			CTTT|splice_acceptor_variant&intron_variant|HIGH|ZNF503-AS1|EN
lincRNA			CTTT|splice_acceptor_variant&intron_variant|HIGH|ZNF503-AS1|EN
lincRNA			CTTT|splice_acceptor_variant&intron_variant|HIGH|ZNF503-AS1|EN
)225652			T|bidirectional_gene_fusion|HIGH|KCNMA1&KCNMA1-AS3|ENSG00
lincRNA			T|splice_donor_variant&intron_variant|HIGH|RP11-17G2.1|ENSG00
protein_coding			T|structural_interaction_variant|HIGH|PPIF|ENSG00000108179|inte
antisense			T|splice_acceptor_variant&intron_variant|HIGH|NUTM2B-AS1|ENS
antisense			A|splice_donor_variant&intron_variant|HIGH|TMEM254-AS1|ENSG
protein_co	13	0.08	CA|frameshift_variant|HIGH|TMEM254|ENSG00000133678|transcr
protein_co	13	0.08	CA|frameshift_variant|HIGH|TMEM254|ENSG00000133678|transcr
protein_co	13	0.08	A|frameshift_variant|HIGH|TMEM254|ENSG00000133678|transcrip
protein_co	4	0.5	GAGA|splice_donor_variant&intron_variant|HIGH|PLAC9|ENSG0000
protein_co	10	0.1	G|splice_donor_variant&intron_variant|HIGH|ANXA11|ENSG000001
protein_coding			A|structural_interaction_variant|HIGH|MAT1A|ENSG00000151224|
protein_coding			A|structural_interaction_variant|HIGH|MAT1A|ENSG00000151224|

protein_coding			G|structural_interaction_variant|HIGH|MAT1A|ENSG00000151224|
lincRNA			G|splice_acceptor_variant&intron_variant|HIGH|LINC01520|ENSG0
protein_coding			C|structural_interaction_variant|HIGH|WAPL|ENSG00000062650|ir
protein_coding			T|stop_lost|HIGH|SNCG|ENSG00000173267|transcript|ENST00000:
antisense			G|splice_acceptor_variant&intron_variant|HIGH|NUTM2A-AS1|ENS
lincRNA			G|splice_acceptor_variant&intron_variant|HIGH|RP11-57C13.6|ENS
protein_co	7	0.29	CTT|splice_acceptor_variant&intron_variant|HIGH|PTEN|ENSG0000
protein_co	7	0.29	CTT|splice_acceptor_variant&intron_variant|HIGH|PTEN|ENSG0000
protein_co	7	0.29	CTT|splice_acceptor_variant&intron_variant|HIGH|PTEN|ENSG0000
protein_co	3	0.33	C|splice_acceptor_variant&intron_variant|HIGH|LIPJ|ENSG0000020
protein_co	3	0.67	C|splice_acceptor_variant&splice_region_variant&intron_variant|HI
protein_co	2	1	A|splice_donor_variant&intron_variant|HIGH|LIPM|ENSG00000173
protein_coding			A|frameshift_variant|HIGH|LIPA|ENSG00000107798|transcript|ENS
protein_coding			A|structural_interaction_variant|HIGH|IDE|ENSG00000119912|inte
nonsense_mediated_decay			A|stop_gained|HIGH|MYOF|ENSG00000138119|transcript|ENST000
protein_coding			T|stop_lost|HIGH|LGI1|ENSG00000108231|transcript|ENST000006:
protein_coding			T|structural_interaction_variant|HIGH|CYP2C9|ENSG00000138109|
protein_coding			C|structural_interaction_variant|HIGH|CYP2C9|ENSG00000138109|
protein_coding			T|structural_interaction_variant|HIGH|CYP2C9|ENSG00000138109|
protein_coding			C|structural_interaction_variant|HIGH|CYP2C8|ENSG00000138115|
protein_co	13	0.08	TA|frameshift_variant|HIGH|CYP2C8|ENSG00000138115|transcript
protein_co	13	0.08	C|splice_donor_variant&intron_variant|HIGH|CYP2C8|ENSG000001
protein_co	6	0.17	C|start_lost|HIGH|PIK3AP1|ENSG00000155629|transcript|ENST000
protein_co	4	1	C|frameshift_variant&splice_acceptor_variant&splice_region_varian
protein_coding			A|structural_interaction_variant|HIGH|HOGA1|ENSG00000241935|
protein_coding			G|stop_lost|HIGH|MORN4|ENSG00000171160|transcript|ENST000
protein_co	1	1	G|splice_acceptor_variant&intron_variant|HIGH|AVPI1|ENSG00000
protein_coding			CCGCCG|frameshift_variant|HIGH|SFRP5|ENSG00000120057|transc
protein_coding			T|structural_interaction_variant|HIGH|DNMBP|ENSG00000107554|
protein_coding			A|structural_interaction_variant|HIGH|DNMBP|ENSG00000107554
nonsense_	13	0.08	C|splice_donor_variant&intron_variant|HIGH|SEC31B|ENSG000000
protein_coding			G|structural_interaction_variant|HIGH|HIF1AN|ENSG00000166135|
protein_coding			C|structural_interaction_variant|HIGH|SUFU|ENSG00000107882|in
)270316			T|gene_fusion|HIGH|AS3MT&BORCS7-ASMT|ENSG00000214435&E
protein_coding			T|stop_gained|HIGH|RPEL1|ENSG00000235376|transcript|ENST000
protein_co	4	0.75	C|splice_donor_variant&intron_variant|HIGH|SH3PXD2A|ENSG0000
protein_coding			G|structural_interaction_variant|HIGH|SLK|ENSG00000065613|inte
protein_co	7	0.43	A|frameshift_variant&splice_donor_variant&splice_region_variant&
protein_coding			T|structural_interaction_variant|HIGH|DUSP5|ENSG00000138166|i
protein_co	4	1	A|stop_gained|HIGH|NRAP|ENSG00000197893|transcript|ENST000
protein_co	12	0.08	C|frameshift_variant|HIGH|CASP7|ENSG00000165806|transcript|EI
protein_coding			C|structural_interaction_variant|HIGH|CASP7|ENSG00000165806|ir
protein_coding			G|structural_interaction_variant|HIGH|CASP7|ENSG00000165806|i
processed_pseudogene			C|splice_acceptor_variant&splice_region_variant&intron_variant|HI
processed_pseudogene			C|splice_acceptor_variant&splice_region_variant&intron_variant|HI
processed_pseudogene			C|splice_acceptor_variant&splice_region_variant&intron_variant|HI
processed_pseudogene			C|splice_acceptor_variant&splice_region_variant&intron_variant|HI

processed_pseudogene			C|splice_acceptor_variant&splice_region_variant&intron_variant|HI
protein_co	1	1	GA|frameshift_variant|HIGH|PNLIPRP3|ENSG00000203837|transcri
protein_coding			G|structural_interaction_variant|HIGH|PNLIPRP1|ENSG0000018702
protein_coding			C|structural_interaction_variant|HIGH|PNLIPRP1|ENSG0000018702
polymorph	5	0.4	G|stop_lost|HIGH|PNLIPRP2|ENSG00000266200|transcript|ENST00
processed_transcript			CTTT|splice_acceptor_variant&intron_variant|HIGH|FAM45A|ENSG
processed_transcript			CTTT|splice_acceptor_variant&intron_variant|HIGH|FAM45A|ENSG
protein_coding			A|protein_protein_contact|HIGH|RGS10|ENSG00000148908|intera
protein_coding			C|structural_interaction_variant|HIGH|FGFR2|ENSG00000066468|i
protein_co	1	1	T|stop_gained|HIGH|ARMS2|ENSG00000254636|transcript|ENST00
protein_co	5	0.2	C|frameshift_variant|HIGH|CHST15|ENSG00000182022|transcript|
protein_co	5	0.2	C|frameshift_variant|HIGH|CHST15|ENSG00000182022|transcript|
protein_co	5	0.2	C|frameshift_variant|HIGH|CHST15|ENSG00000182022|transcript|
protein_co	5	0.2	C|frameshift_variant|HIGH|CHST15|ENSG00000182022|transcript|
protein_co	5	0.2	C|frameshift_variant|HIGH|CHST15|ENSG00000182022|transcript|
protein_co	5	0.2	C|frameshift_variant|HIGH|CHST15|ENSG00000182022|transcript|
protein_coding			A|structural_interaction_variant|HIGH|OAT|ENSG00000065154|int
processed_pseudogene			T|splice_donor_variant&intron_variant|HIGH|NPM1P31|ENSG0000
protein_co	14	0.07	C|frameshift_variant|HIGH|CTBP2|ENSG00000175029|transcript|E
antisense			G|splice_acceptor_variant&intron_variant|HIGH|RP11-109A6.2|ENS
lincRNA			A|splice_donor_variant&intron_variant|HIGH|RP11-109A6.5|ENSG
lincRNA			A|splice_acceptor_variant&intron_variant|HIGH|RP11-109A6.5|ENS
protein_co	6	0.17	CCTAT|splice_acceptor_variant&intron_variant|HIGH|STK32C|ENSG
protein_coding			T|stop_gained|HIGH|PWWP2B|ENSG00000171813|transcript|ENST
protein_co	3	0.33	TG|frameshift_variant|HIGH|ADGRA1|ENSG00000197177|transcrip
protein_coding			T|structural_interaction_variant|HIGH|ADAM8|ENSG00000151651|
protein_co	1	1	C|frameshift_variant&start_lost&splice_region_variant|HIGH|RP11-
retained_intron			C|splice_donor_variant&intron_variant|HIGH|SCART1|ENSG000002
retained_intron			CTT|splice_acceptor_variant&intron_variant|HIGH|SCART1|ENSG00
retained_intron			C|splice_region_variant&intron_variant|LOW|SCART1|ENSG000002
protein_coding			A|structural_interaction_variant|HIGH|CYP2E1|ENSG00000130649|
retained_intron			G|splice_acceptor_variant&intron_variant|HIGH|CYP2E1|ENSG0000
protein_co	2	1	A|frameshift_variant|HIGH|FRG2B|ENSG00000225899|transcript|E
protein_coding			T|structural_interaction_variant|HIGH|SIRT3|ENSG00000142082|in
protein_coding			A|structural_interaction_variant|HIGH|SIRT3|ENSG00000142082|in
protein_co	8	0.13	T|frameshift_variant|HIGH|ANO9|ENSG00000185101|transcript|EN
protein_co	1	1	C|splice_acceptor_variant&splice_region_variant&intron_variant|HI
protein_co	2	0.5	CGGCCTCCCCCCGG|frameshift_variant|HIGH|DRD4|ENSG00000069
protein_co	2	0.5	ACCCCTGCGGCCCCGACTGTGCGCCCCCCGCGCCCG|frameshift_variant
protein_co	2	0.5	ACCCCTGCGGCCCCGACTGTGCGCCCCCCGCGCCCG|frameshift_variant
protein_co	7	0.14	G|frameshift_variant|HIGH|TMEM80|ENSG00000177042|transcript
nonsense_	9	0.11	ATT|splice_acceptor_variant&intron_variant|HIGH|TALDO1|ENSG00
nonsense_	9	0.11	ATT|splice_acceptor_variant&intron_variant|HIGH|TALDO1|ENSG00
nonsense_	9	0.11	ATT|splice_acceptor_variant&intron_variant|HIGH|TALDO1|ENSG00
protein_co	14	0.07	T|splice_donor_variant&intron_variant|HIGH|CRACR2B|ENSG0000C
protein_co	20	0.05	CTCCTTGGAGGTGCGAGGGCAGTGAGAGGCAAAGAAGGT|splice_dono
protein_co	20	0.05	CTCCTTGGAGGTGCGAGGGCAGTGAGAGGCAAAGAAGGT|splice_dono

protein_co	1	1	GGT|frameshift_variant|HIGH|MUC5AC|ENSG00000215182|transcr
protein_co	1	1	GGT|frameshift_variant|HIGH|MUC5AC|ENSG00000215182|transcr
protein_co	1	1	C|frameshift_variant&splice_acceptor_variant&splice_region_varian
protein_co	1	1	C|frameshift_variant|HIGH|MUC5AC|ENSG00000215182|transcript
protein_coding			A|structural_interaction_variant|HIGH|TH|ENSG00000180176|inter
protein_coding			T|structural_interaction_variant|HIGH|TH|ENSG00000180176|inter
protein_co	4	0.25	T|start_lost|HIGH|C11orf21|ENSG00000110665|transcript|ENST00
nonsense_	17	0.12	G|start_lost|HIGH|TSPAN32|ENSG00000064201|transcript|ENST00
processed_transcript			CAGACAGGGAAGGCGTTAGGAGGGGAGGCCT|splice_donor_variant&
protein_coding			A|structural_interaction_variant|HIGH|RRM1|ENSG00000167325|ir
protein_coding			G|structural_interaction_variant|HIGH|RRM1|ENSG00000167325|ir
polymorph	1	1	A|frameshift_variant|HIGH|OR52B4|ENSG00000221996|transcript|
protein_co	1	1	TAC|frameshift_variant|HIGH|C11orf40|ENSG00000171987|transcr
protein_co	1	1	CAG|frameshift_variant|HIGH|C11orf40|ENSG00000171987|transcı
protein_co	1	1	TG|frameshift_variant|HIGH|C11orf40|ENSG00000171987|transcri
protein_co	1	1	T|stop_gained|HIGH|C11orf40|ENSG00000171987|transcript|ENST
protein_co	2	1	C|frameshift_variant|HIGH|OR51F1|ENSG00000280021|transcript|
protein_co	2	1	TACCACCCC|frameshift_variant|HIGH|OR51T1|ENSG00000176900|
protein_coding			T|stop_gained|HIGH|OR52J3|ENSG00000205495|transcript|ENST00
polymorph	1	1	AC|frameshift_variant|HIGH|OR52E1|ENSG00000273085|transcript
protein_co	2	1	AC|frameshift_variant|HIGH|OR52A1|ENSG00000182070|transcript
polymorphic_pseudogene			G|frameshift_variant|HIGH|OR52Z1|ENSG00000176748|transcript|
antisense			A|splice_donor_variant&intron_variant|HIGH|AC104389.16|ENSG00
protein_coding			G|structural_interaction_variant|HIGH|HBB|ENSG00000244734|int
protein_co	1	1	C|frameshift_variant|HIGH|OR51B5|ENSG00000242180|transcript|
protein_co	1	1	TTATC|frameshift_variant|HIGH|OR51J1|ENSG00000184321|transc
protein_coding			T|stop_gained|HIGH|OR51Q1|ENSG00000167360|transcript|ENST0
protein_coding			A|stop_gained|HIGH|OR51I1|ENSG00000167359|transcript|ENST00
protein_co	1	1	TCA|frameshift_variant|HIGH|OR51I2|ENSG00000187918|transcrip
protein_co	1	1	GGGCT|frameshift_variant|HIGH|OR52D1|ENSG00000181609|trans
protein_coding			T|stop_gained|HIGH|OR52N4|ENSG00000181074|transcript|ENST0
protein_co	1	1	G|frameshift_variant|HIGH|OR52N1|ENSG00000181001|transcript|
protein_coding			A|stop_lost|HIGH|OR52E8|ENSG00000183269|transcript|ENST000
protein_co	2	0.5	A|stop_gained|HIGH|OR56A5|ENSG00000188691|transcript|ENST0
retained_intron			A|splice_acceptor_variant&intron_variant|HIGH|PRKCDBP|ENSG000
protein_co	24	0.63	C|frameshift_variant&splice_donor_variant&splice_region_variant&
protein_co	20	0.15	T|stop_gained|HIGH|DNHD1|ENSG00000179532|transcript|ENST00
protein_coding			T|protein_protein_contact|HIGH|ILK|ENSG00000166333|interactio
protein_co	1	1	T|frameshift_variant|HIGH|OR2D3|ENSG00000178358|transcript|E
283415			G|bidirectional_gene_fusion|HIGH|RP11-413N10.3&ZNF215|ENSG0
protein_coding			A|structural_interaction_variant|HIGH|NLRP14|ENSG00000158077|
protein_co	17	0.06	CA|frameshift_variant|HIGH|STK33|ENSG00000130413|transcript|
protein_co	17	0.06	GAACCA|frameshift_variant|HIGH|STK33|ENSG00000130413|trans
antisense			C|splice_acceptor_variant&intron_variant|HIGH|RP11-152H18.4|EN
protein_co	8	0.13	GTTTTTTTTTTTTTTTTT|splice_acceptor_variant&intron_variant|HIGH
protein_co	8	0.13	GTTTTTTTTTTTTTTTTT|splice_acceptor_variant&intron_variant|HIGH
protein_co	8	0.13	GTTTTTTTTTTTTTTTTT|splice_acceptor_variant&intron_variant|HIGH

protein_co	8	0.13	GTTTTTTTTTTTTTTTTT|splice_acceptor_variant&intron_variant|HIGH
protein_co	8	0.13	GTTTTTTTTTTTTTTTTT|splice_acceptor_variant&intron_variant|HIGH
antisense			G|splice_donor_variant&splice_region_variant&intron_variant&non_
protein_co	18	0.06	T|splice_donor_variant&intron_variant|HIGH|AMPD3|ENSG000001
processed_transcript			GT|splice_acceptor_variant&intron_variant|HIGH|AMPD3|ENSG000
antisense			T|splice_donor_variant&intron_variant|HIGH|MRVI1-AS1|ENSG000
protein_co	17	0.12	T|splice_acceptor_variant&intron_variant|HIGH|MRVI1|ENSG00000
lincRNA			CTT|splice_acceptor_variant&intron_variant|HIGH|RP13-631K18.3|
lincRNA			CTTT|splice_acceptor_variant&intron_variant|HIGH|RP13-631K18.3
lincRNA			CTTTT|splice_acceptor_variant&intron_variant|HIGH|RP13-631K18.
lincRNA			CTTTTT|splice_acceptor_variant&intron_variant|HIGH|RP13-631K18
lincRNA			CTTTTTT|splice_acceptor_variant&intron_variant|HIGH|RP13-631K1
protein_co	3	0.33	CA|frameshift_variant|HIGH|MICALCL|ENSG00000133808|transcrip
processed_transcript			CTTT|splice_acceptor_variant&intron_variant|HIGH|PARVA|ENSG00
processed_transcript			CTTT|splice_acceptor_variant&intron_variant|HIGH|PARVA|ENSG00
processed_transcript			CTTT|splice_acceptor_variant&intron_variant|HIGH|PARVA|ENSG00
protein_coding			T|structural_interaction_variant|HIGH|SPON1|ENSG00000262655|i
protein_coding			G|structural_interaction_variant|HIGH|SPON1|ENSG00000262655|
protein_co	12	0.08	C|splice_donor_variant&splice_region_variant&intron_variant|HIGH
protein_co	12	0.08	C|splice_donor_variant&intron_variant|HIGH|COPB1|ENSG0000012
protein_coding			A|structural_interaction_variant|HIGH|CYP2R1|ENSG00000186104|
processed_pseudogene			A|splice_acceptor_variant&splice_region_variant&intron_variant|HI
protein_coding			C|frameshift_variant|HIGH|NCR3LG1|ENSG00000188211|transcript
processed_transcript			T|splice_acceptor_variant&intron_variant|HIGH|ABCC8|ENSG00000
nonsense_mediated_decay			T|structural_interaction_variant|HIGH|USH1C|ENSG00000006611|i
protein_co	6	0.33	T|stop_gained|HIGH|OTOG|ENSG00000188162|transcript|ENST000
protein_co	23	0.13	T|stop_gained|HIGH|SERGEF|ENSG00000129158|transcript|ENST00
protein_coding			A|stop_gained|HIGH|MRGPRX3|ENSG00000179826|transcript|ENS
protein_co	2	0.5	CT|frameshift_variant&stop_gained|HIGH|MRGPRX4|ENSG0000017
protein_coding			A|structural_interaction_variant|HIGH|LDHA|ENSG00000134333|in
protein_coding			T|structural_interaction_variant|HIGH|LDHA|ENSG00000134333|in
protein_co	13	0.08	C|start_lost|HIGH|UEVLD|ENSG00000151116|transcript|ENST0000
protein_coding			G|structural_interaction_variant|HIGH|HTATIP2|ENSG00000109854
lincRNA			C|splice_donor_variant&intron_variant|HIGH|CTD-2019O4.1|ENSG0
lincRNA			G|splice_donor_variant&intron_variant|HIGH|LINC01495|ENSG000
protein_coding			A|structural_interaction_variant|HIGH|BBOX1|ENSG00000129151|i
protein_coding			A|structural_interaction_variant|HIGH|KIF18A|ENSG00000121621|
nonsense_	12	0.17	A|splice_acceptor_variant&intron_variant|HIGH|METTL15|ENSG000
antisense			A|splice_acceptor_variant&intron_variant|HIGH|RP5-1024C24.1|EN
protein_co	1	1	C|splice_donor_variant&intron_variant|HIGH|AC131263.1|ENSG000
nonsense_mediated_decay			T|stop_gained|HIGH|TCP11L1|ENSG00000176148|transcript|ENST0
processed_transcript			CT|splice_acceptor_variant&intron_variant|HIGH|HIPK3|ENSG0000
protein_coding			A|stop_gained|HIGH|C11orf91|ENSG00000205177|transcript|ENST
processed_pseudogene			C|splice_acceptor_variant&intron_variant|HIGH|CIR1P3|ENSG0000
protein_coding			T|structural_interaction_variant|HIGH|CAT|ENSG00000121691|inte
protein_coding			G|structural_interaction_variant|HIGH|APIP|ENSG00000149089|int
protein_coding			T|structural_interaction_variant|HIGH|APIP|ENSG00000149089|int

protein_co	8	0.13	C|splice_donor_variant&intron_variant|HIGH|PDHX|ENSG0000011(
processed_transcript			CTTT|splice_acceptor_variant&intron_variant|HIGH|PDHX|ENSG000
processed_transcript			C|splice_region_variant&intron_variant|LOW|PDHX|ENSG0000011(
processed_transcript			CT|splice_region_variant&intron_variant|LOW|PDHX|ENSG0000011
protein_co	38	0.03	A|splice_acceptor_variant&intron_variant|HIGH|CD44|ENSG00000(
protein_co	8	0.13	T|splice_donor_variant&intron_variant|HIGH|SLC1A2|ENSG0000011
nonsense_	3	0.33	A|splice_donor_variant&intron_variant|HIGH|RAG1|ENSG0000016(
lincRNA			C|splice_donor_variant&intron_variant|HIGH|LINC01499|ENSG0000(
protein_coding			T|structural_interaction_variant|HIGH|ALKBH3|ENSG00000166199|
sense_overlapping			C|splice_acceptor_variant&intron_variant|HIGH|RP11-613D13.4|EN
sense_overlapping			T|splice_donor_variant&intron_variant|HIGH|RP11-613D13.4|ENSG
retained_intron			G|splice_donor_variant&splice_region_variant&intron_variant|HIGH
lincRNA			A|splice_donor_variant&intron_variant|HIGH|RP11-45A12.2|ENSG(
nonsense_mediated_decay			G|frameshift_variant|HIGH|TP53I11|ENSG00000175274|transcript|
nonsense_	23	0.04	CT|frameshift_variant|HIGH|TP53I11|ENSG00000175274|transcript
protein_co	23	0.04	G|frameshift_variant|HIGH|TP53I11|ENSG00000175274|transcript|
protein_co	6	0.17	C|splice_acceptor_variant&intron_variant|HIGH|CREB3L1|ENSG000
nonsense_mediated_decay			GC|frameshift_variant|HIGH|MDK|ENSG00000110492|transcript|E
nonsense_mediated_decay			GC|frameshift_variant|HIGH|MDK|ENSG00000110492|transcript|E
nonsense_mediated_decay			GC|frameshift_variant|HIGH|MDK|ENSG00000110492|transcript|E
nonsense_mediated_decay			GC|frameshift_variant|HIGH|MDK|ENSG00000110492|transcript|E
nonsense_mediated_decay			GC|frameshift_variant|HIGH|MDK|ENSG00000110492|transcript|E
protein_co	30	0.47	CTT|splice_acceptor_variant&intron_variant|HIGH|ATG13|ENSG000
protein_co	30	0.47	CTT|splice_acceptor_variant&intron_variant|HIGH|ATG13|ENSG000
protein_coding			T|structural_interaction_variant|HIGH|F2|ENSG00000180210|inter
protein_co	11	0.91	C|splice_donor_variant&splice_region_variant&intron_variant|HIGH
protein_coding			A|structural_interaction_variant|HIGH|DDB2|ENSG00000134574|ir
processed_	16	0.06	C|splice_donor_variant&intron_variant|HIGH|DDB2|ENSG0000013
protein_co	23	0.22	T|stop_gained|HIGH|MADD|ENSG00000110514|transcript|ENST00
protein_coding			C|structural_interaction_variant|HIGH|MYBPC3|ENSG00000134571
protein_coding			A|structural_interaction_variant|HIGH|MYBPC3|ENSG00000134571
protein_co	4	0.25	GCA|frameshift_variant|HIGH|SPI1|ENSG00000066336|transcript|E
)255197			T|bidirectional_gene_fusion|HIGH|MIR4487&RP11-750H9.5|ENSG0
protein_co	13	0.08	ATT|splice_acceptor_variant&intron_variant|HIGH|NDUFS3|ENSG0(
protein_co	13	0.08	ATT|splice_acceptor_variant&intron_variant|HIGH|NDUFS3|ENSG0(
protein_co	6	0.33	CATAACAAAAGAAACCAGAAAGCTGACATACTTCCTACCCCAAAATA|spli
protein_co	6	0.33	CATAACAAAAGAAACCAGAAAGCTGACATACTTCCTACCCCAAAATA|spli
polymorphic_pseudogene			G|stop_gained|HIGH|OR4X2|ENSG00000172208|transcript|ENST0(
polymorphic_pseudogene			T|synonymous_variant|LOW|OR4X2|ENSG00000172208|transcript|
protein_coding			A|stop_gained|HIGH|OR4X1|ENSG00000176567|transcript|ENST00
unprocessed_pseudogene			G|splice_donor_variant&intron_variant|HIGH|TRIM51GP|ENSG0000(
protein_coding			C|structural_interaction_variant|HIGH|FOLH1|ENSG00000086205|i
protein_coding			A|structural_interaction_variant|HIGH|FOLH1|ENSG00000086205|i
nonsense_mediated_decay			G|stop_lost&splice_region_variant|HIGH|FOLH1|ENSG0000008620!
unprocessed_pseudogene			C|splice_donor_variant&splice_region_variant&intron_variant&non_
protein_coding			C|stop_lost|HIGH|TRIM48|ENSG00000150244|transcript|ENST0000
polymorphic_pseudogene			T|stop_gained|HIGH|OR4C16|ENSG00000279514|transcript|ENST0

protein_coding			A|stop_gained|HIGH|OR4C11|ENSG00000172188|transcript|ENST0
protein_coding			G|stop_gained|HIGH|OR4P4|ENSG00000181927|transcript|ENST0C
unprocessed_pseudogene			CGGGCGCCTTGCTGAGGCGGATCCG|splice_donor_variant&intron_va
protein_co	1	1	G|frameshift_variant|HIGH|OR8I2|ENSG00000172154|transcript|EI
protein_co	1	1	C|frameshift_variant|HIGH|OR8I2|ENSG00000172154|transcript|EI
protein_coding			G|stop_gained|HIGH|OR8I2|ENSG00000172154|transcript|ENST00
polymorph	1	1	ACT|frameshift_variant|HIGH|OR5AL1|ENSG00000272987|transcrip
protein_coding			T|stop_gained|HIGH|OR5M11|ENSG00000255223|transcript|ENST(
protein_co	1	1	C|frameshift_variant|HIGH|OR5M10|ENSG00000254834|transcript
protein_co	1	1	C|frameshift_variant|HIGH|OR5M1|ENSG00000255012|transcript|I
polymorphic_pseudogene			T|stop_gained|HIGH|OR5AR1|ENSG00000172459|transcript|ENST0
protein_co	1	1	T|frameshift_variant|HIGH|OR9G1|ENSG00000174914|transcript|E
antisense			A|splice_donor_variant&intron_variant|HIGH|RP11-872D17.4|ENSG
protein_co	38	0.03	A|splice_donor_variant&intron_variant|HIGH|CTNND1|ENSG00000
protein_co	1	1	A|frameshift_variant|HIGH|OR6Q1|ENSG00000279051|transcript|E
polymorphic_pseudogene			G|stop_lost&splice_region_variant|HIGH|OR1S1|ENSG0000028020
protein_co	1	1	A|frameshift_variant|HIGH|OR5B3|ENSG00000172769|transcript|E
protein_co	1	1	A|frameshift_variant|HIGH|OR5B12|ENSG00000172362|transcript|
protein_co	2	1.00,(ZFP9:	A|splice_acceptor_variant&intron_variant|HIGH|ZFP91|ENSG00000
protein_co	6	0.83	G|frameshift_variant|HIGH|FAM111B|ENSG00000189057|transcrip
protein_co	11	0.45	GGCAGATACTT|frameshift_variant|HIGH|FAM111A|ENSG00000166
unprocessed_pseudogene			A|splice_donor_variant&intron_variant|HIGH|WARSP1|ENSG00000
protein_co	1	1	G|frameshift_variant|HIGH|OR4D6|ENSG00000166884|transcript|E
protein_coding			T|stop_gained|HIGH|OR4D10|ENSG00000254466|transcript|ENST0
protein_coding			A|stop_gained|HIGH|OR10V1|ENSG00000172289|transcript|ENST0
unitary_pseudogene			A|splice_acceptor_variant&intron_variant|HIGH|OOSP1|ENSG0000(
processed_pseudogene			T|splice_acceptor_variant&splice_region_variant&intron_variant&n
processed_pseudogene			TCAA|splice_acceptor_variant&splice_region_variant&intron_varian
processed_pseudogene			TCAACAA|splice_region_variant&non_coding_transcript_exon_varia
processed_pseudogene			TCAACAACAA|non_coding_transcript_exon_variant|MODIFIER|RP11
nonsense_	21	0.05	T|splice_acceptor_variant&intron_variant|HIGH|MS4A6A|ENSG000
protein_co	11	0.91	A|frameshift_variant|HIGH|MS4A14|ENSG00000166928|transcript|
nonsense_	11	0.36	G|splice_acceptor_variant&intron_variant|HIGH|MS4A14|ENSG000
protein_co	5	0.8	T|stop_gained|HIGH|MS4A12|ENSG00000071203|transcript|ENST0
protein_coding			A|structural_interaction_variant|HIGH|PRPF19|ENSG00000110107|
protein_coding			T|structural_interaction_variant|HIGH|DDB1|ENSG00000167986|in
protein_coding			A|structural_interaction_variant|HIGH|DDB1|ENSG00000167986|ir
protein_co	5	0.2	CA|splice_acceptor_variant&intron_variant|HIGH|TMEM216|ENSG(
protein_co	5	0.4	C|splice_acceptor_variant&intron_variant|HIGH|TMEM216|ENSG00
lincRNA			G|splice_donor_variant&intron_variant|HIGH|DKFZP434K028|ENSG
protein_coding			A|stop_gained|HIGH|EML3|ENSG00000149499|transcript|ENST000
protein_coding			C|structural_interaction_variant|HIGH|NXF1|ENSG00000162231|in
processed_transcript			T|splice_donor_variant&intron_variant|HIGH|NXF1|ENSG00000162
protein_co	3	0.67	C|stop_gained|HIGH|SLC22A24|ENSG00000197658|transcript|ENS
protein_co	3	0.67	A|stop_gained|HIGH|SLC22A24|ENSG00000197658|transcript|ENS
protein_co	3	1	T|splice_donor_variant&intron_variant|HIGH|SLC22A24|ENSG0000(
nonsense_mediated_decay			G|stop_lost|HIGH|SLC22A25|ENSG00000196600|transcript|ENST0C

nonsense_	6	0.67	A|stop_gained|HIGH|SLC22A10|ENSG00000184999|transcript|ENST
protein_co	2	0.5	CA|frameshift_variant|HIGH|SLC22A9|ENSG00000149742|transcrip
protein_co	6	0.5	TG|frameshift_variant|HIGH|HRASLS5|ENSG00000168004|transcrip
protein_coding			A|splice_donor_variant&splice_region_variant&5_prime_UTR_varia
protein_co	12	0.08	T|splice_donor_variant&intron_variant|HIGH|OTUB1|ENSG0000016
protein_coding			T|frameshift_variant|HIGH|DNAJC4|ENSG00000110011|transcript|
protein_coding			T|structural_interaction_variant|HIGH|PLCB3|ENSG00000149782|ir
protein_coding			A|structural_interaction_variant|HIGH|PLCB3|ENSG00000149782|i
protein_coding			T|structural_interaction_variant|HIGH|PLCB3|ENSG00000149782|ir
miRNA			G|transcript_ablation|HIGH|MIR7155|ENSG00000278359|transcrip
protein_coding			A|structural_interaction_variant|HIGH|MEN1|ENSG00000133895|ir
protein_co	6	0.33	G|splice_donor_variant&splice_region_variant&intron_variant|HIGH
protein_coding			T|structural_interaction_variant|HIGH|ARL2|ENSG00000213465|int
processed_pseudogene			C|splice_acceptor_variant&splice_region_variant&intron_variant&n
processed_pseudogene			C|splice_acceptor_variant&splice_region_variant&intron_variant&n
processed_pseudogene			C|splice_acceptor_variant&splice_region_variant&intron_variant&n
processed_pseudogene			C|splice_acceptor_variant&splice_region_variant&intron_variant&n
protein_co	21	0.05	C|frameshift_variant|HIGH|LTBP3|ENSG00000168056|transcript|EN
protein_coding			T|structural_interaction_variant|HIGH|RELA|ENSG00000173039|int
protein_co	15	0.07	C|splice_donor_variant&intron_variant|HIGH|CFL1|ENSG0000017
protein_co	15	0.2	T|splice_donor_variant&intron_variant|HIGH|CFL1|ENSG0000017
protein_co	15	0.07	T|splice_acceptor_variant&intron_variant|HIGH|CFL1|ENSG000001
protein_coding			A|structural_interaction_variant|HIGH|MUS81|ENSG00000172732|
protein_coding			T|structural_interaction_variant|HIGH|MUS81|ENSG00000172732|
protein_co	17	0.24	GC|splice_acceptor_variant&intron_variant|HIGH|MUS81|ENSG000
protein_coding			T|frameshift_variant|HIGH|CTSW|ENSG00000172543|transcript|EN
protein_coding			A|structural_interaction_variant|HIGH|DPP3|ENSG00000254986|in
protein_co	3	0.67	T|stop_gained|HIGH|ACTN3|ENSG00000248746|transcript|ENST00
protein_coding			C|structural_interaction_variant|HIGH|KDM2A|ENSG00000173120|
protein_coding			A|structural_interaction_variant|HIGH|GRK2|ENSG00000173020|in
protein_coding			A|structural_interaction_variant|HIGH|RAD9A|ENSG00000172613|
protein_coding			A|protein_protein_contact|HIGH|PPP1CA|ENSG00000172531|inter
protein_co	1	1	CAGCTGTGG|frameshift_variant|HIGH|GPR152|ENSG00000175514|
nonsense_	17	0.24	A|stop_gained|HIGH|TMEM134|ENSG00000172663|transcript|ENS
protein_co	8	0.13	C|splice_donor_variant&intron_variant|HIGH|ALDH3B2|ENSG00000
protein_coding			T|structural_interaction_variant|HIGH|IGHMBP2|ENSG0000013274
protein_coding			T|structural_interaction_variant|HIGH|CCND1|ENSG00000110092|i
protein_coding			A|stop_gained|HIGH|PPFIA1|ENSG00000131626|transcript|ENST00
nonsense_mediated_decay			T|stop_gained|HIGH|CTTN|ENSG00000085733|transcript|ENST000
processed_transcript			T|splice_acceptor_variant&intron_variant|HIGH|SHANK2|ENSG0000
protein_co	21	0.05	C|frameshift_variant|HIGH|SHANK2|ENSG00000162105|transcript|
protein_co	21	0.05	AACTCGCC|frameshift_variant|HIGH|SHANK2|ENSG00000162105|t
protein_co	12	0.25	T|stop_gained|HIGH|DHCR7|ENSG00000172893|transcript|ENST00
protein_co	2	0.5	G|frameshift_variant&splice_acceptor_variant&splice_region_varian
protein_co	34	0.03	T|splice_donor_variant&intron_variant|HIGH|NUMA1|ENSG000001
protein_co	22	0.05	C|splice_acceptor_variant&intron_variant|HIGH|LRTOMT|ENSG000
protein_co	6	0.5	C|frameshift_variant|HIGH|FOLR3|ENSG00000110203|transcript|E

protein_coding			C|structural_interaction_variant|HIGH|PDE2A|ENSG00000186642|i
protein_coding			A|stop_gained|HIGH|ARAP1|ENSG00000186635|transcript|ENST00
protein_coding			C|splice_acceptor_variant&5_prime_UTR_variant&intron_variant|H
)175582			A|bidirectional_gene_fusion|HIGH|RAB6A&RP11-456I15.2|ENSG00(
)175582			A|bidirectional_gene_fusion|HIGH|RAB6A&RP11-456I15.2|ENSG00(
)175582			A|bidirectional_gene_fusion|HIGH|RAB6A&RP11-456I15.2|ENSG00(
)175582			A|bidirectional_gene_fusion|HIGH|RAB6A&RP11-456I15.2|ENSG00(
)175582			A|bidirectional_gene_fusion|HIGH|RAB6A&RP11-456I15.2|ENSG00(
)175582			A|bidirectional_gene_fusion|HIGH|RAB6A&RP11-456I15.2|ENSG00(
)175582			A|bidirectional_gene_fusion|HIGH|RAB6A&RP11-456I15.2|ENSG00(
)175582			A|bidirectional_gene_fusion|HIGH|RAB6A&RP11-456I15.2|ENSG00(
)175582			A|bidirectional_gene_fusion|HIGH|RAB6A&RP11-456I15.2|ENSG00(
)175582			A|bidirectional_gene_fusion|HIGH|RAB6A&RP11-456I15.2|ENSG00(
)175582			A|bidirectional_gene_fusion|HIGH|RAB6A&RP11-456I15.2|ENSG00(
)175582			A|bidirectional_gene_fusion|HIGH|RAB6A&RP11-456I15.2|ENSG00(
)175582			A|bidirectional_gene_fusion|HIGH|RAB6A&RP11-456I15.2|ENSG00(
)175582			A|bidirectional_gene_fusion|HIGH|RAB6A&RP11-456I15.2|ENSG00(
)175582			A|bidirectional_gene_fusion|HIGH|RAB6A&RP11-456I15.2|ENSG00(
)175582			A|bidirectional_gene_fusion|HIGH|RAB6A&RP11-456I15.2|ENSG00(
)175582			A|bidirectional_gene_fusion|HIGH|RAB6A&RP11-456I15.2|ENSG00(
)175582			A|bidirectional_gene_fusion|HIGH|RAB6A&RP11-456I15.2|ENSG00(
)175582			A|bidirectional_gene_fusion|HIGH|RAB6A&RP11-456I15.2|ENSG00(
)175582			A|bidirectional_gene_fusion|HIGH|RAB6A&RP11-456I15.2|ENSG00(
)175582			A|bidirectional_gene_fusion|HIGH|RAB6A&RP11-456I15.2|ENSG00(
)175582			A|bidirectional_gene_fusion|HIGH|RAB6A&RP11-456I15.2|ENSG00(
)175582			A|bidirectional_gene_fusion|HIGH|RAB6A&RP11-456I15.2|ENSG00(
)175582			A|bidirectional_gene_fusion|HIGH|RAB6A&RP11-456I15.2|ENSG00(
)175582			AT|bidirectional_gene_fusion|HIGH|RAB6A&RP11-456I15.2|ENSG0(
)175582			AT|bidirectional_gene_fusion|HIGH|RAB6A&RP11-456I15.2|ENSG0(
nonsense_mediated_decay			A|stop_gained|HIGH|MRPL48|ENSG00000175581|transcript|ENST(
retained_intron			C|splice_acceptor_variant&splice_region_variant&intron_variant|HI
protein_co	20	0.05	TG|frameshift_variant|HIGH|C2CD3|ENSG00000168014|transcript|
protein_co	20	0.05	TG|frameshift_variant|HIGH|C2CD3|ENSG00000168014|transcript|
protein_coding			A|stop_gained|HIGH|P4HA3|ENSG00000149380|transcript|ENST00
nonsense_mediated_decay			A|stop_gained|HIGH|XRRA1|ENSG00000166435|transcript|ENST00
protein_coding			TCGCACGGACCCGCGCACGGCGCC|frameshift_variant|HIGH|KLHL35|
protein_co	4	0.25	AG|splice_donor_variant&intron_variant|HIGH|WNT11|ENSG00000(
protein_coding			G|frameshift_variant&splice_acceptor_variant&splice_region_variar
protein_co	6	0.33	TA|frameshift_variant|HIGH|GDPD4|ENSG00000178795|transcript|
protein_coding			AT|frameshift_variant|HIGH|AAMDC|ENSG00000087884|transcript
protein_coding			C|structural_interaction_variant|HIGH|PRCP|ENSG00000137509|in
protein_coding			G|structural_interaction_variant|HIGH|PRCP|ENSG00000137509|in
protein_coding			G|structural_interaction_variant|HIGH|PRCP|ENSG00000137509|in
protein_co	19	0.05	TA|frameshift_variant|HIGH|CCDC90B|ENSG00000137500|transcrip
lincRNA			T|splice_donor_variant&intron_variant|HIGH|CTD-2005H7.2|ENSG(
processed_pseudogene			C|splice_acceptor_variant&splice_region_variant&intron_variant|HI
processed_pseudogene			CAAAA|splice_acceptor_variant&splice_region_variant&intron_varia

processed_pseudogene			CAAAAA|splice_acceptor_variant&intron_variant|HIGH|HNRNPCP8|
processed_pseudogene			CAAAAAAA|splice_acceptor_variant&intron_variant|HIGH|HNRNPCI
processed_pseudogene			CAAAAAAAA|splice_acceptor_variant&intron_variant|HIGH|HNRNP(
processed_transcript			A|splice_donor_variant&intron_variant|HIGH|FOLH1B|ENSG000001
protein_co	1	1	G|frameshift_variant|HIGH|TRIM64B|ENSG00000189253|transcript
protein_co	1	1	CT|frameshift_variant|HIGH|UBTFL1|ENSG00000255009|transcript
protein_co	1	1	T|frameshift_variant|HIGH|UBTFL1|ENSG00000255009|transcript|I
protein_coding			A|structural_interaction_variant|HIGH|NAALAD2|ENSG0000007761
unprocessed_pseudogene			G|splice_donor_variant&intron_variant|HIGH|RP11-676F20.2|ENSG
protein_co	10	0.1	T|splice_donor_variant&intron_variant|HIGH|MRE11A|ENSG00000(
nonsense_	16	0.06	G|splice_donor_variant&intron_variant|HIGH|CEP57|ENSG0000016
protein_coding			G|structural_interaction_variant|HIGH|MTMR2|ENSG00000087053
protein_coding			T|structural_interaction_variant|HIGH|MTMR2|ENSG00000087053
protein_co	15	0.33	CTTGT|splice_acceptor_variant&intron_variant|HIGH|MTMR2|ENS(
protein_co	15	0.33	CTGGGATACGGCCTCTTGATCTGAAGGATGCCA|splice_acceptor_variai
protein_co	15	0.33	CAGGT|splice_acceptor_variant&intron_variant|HIGH|MTMR2|ENS(
processed_transcript			C|splice_acceptor_variant&splice_region_variant&intron_variant|HI
processed_transcript			C|splice_acceptor_variant&splice_region_variant&intron_variant|HI
processed_transcript			C|splice_acceptor_variant&splice_region_variant&intron_variant|HI
processed_	15	0.13	CCTCAAATCAGGAGAAAAGTTGTCGGCAGAAGT|splice_acceptor_varia
lincRNA			C|splice_donor_variant&intron_variant|HIGH|RP11-49K4.2|ENSG00
protein_co	12	0.33	T|stop_gained|HIGH|CNTN5|ENSG00000149972|transcript|ENST00
protein_co	12	0.33	TGAGGAACCAGGCATTATTTTG|stop_gained&disruptive_inframe_ins(
protein_coding			C|structural_interaction_variant|HIGH|PGR|ENSG00000082175|int(
protein_coding			A|stop_gained|HIGH|MMP8|ENSG00000118113|transcript|ENST00
protein_co	2	0.5	C|splice_acceptor_variant&intron_variant|HIGH|MMP10|ENSG0000(
protein_coding			G|structural_interaction_variant|HIGH|MMP10|ENSG00000166670
protein_coding			A|structural_interaction_variant|HIGH|MMP10|ENSG00000166670
protein_coding			T|structural_interaction_variant|HIGH|MMP1|ENSG00000196611|i
protein_co	1	1	C|frameshift_variant|HIGH|MMP1|ENSG00000196611|transcript|E
protein_coding			A|structural_interaction_variant|HIGH|CASP1|ENSG00000137752|i
protein_coding			A|structural_interaction_variant|HIGH|ACAT1|ENSG00000075239|i
protein_co	25	0.12	CTTT|splice_acceptor_variant&intron_variant|HIGH|ATM|ENSG0000(
protein_co	25	0.12	CTTT|splice_acceptor_variant&intron_variant|HIGH|ATM|ENSG0000(
protein_co	25	0.12	CTTT|splice_acceptor_variant&intron_variant|HIGH|ATM|ENSG0000(
protein_co	5	0.2	T|splice_donor_variant&intron_variant|HIGH|COLCA2|ENSG000002
protein_co	2	0.5	CCCGCCA|frameshift_variant|HIGH|SIK2|ENSG00000170145|transc
protein_co	2	0.5	C|frameshift_variant|HIGH|SIK2|ENSG00000170145|transcript|ENS
protein_co	9	0.22	A|splice_acceptor_variant&intron_variant|HIGH|C11orf57|ENSG000
protein_coding			G|structural_interaction_variant|HIGH|NCAM1|ENSG00000149294
protein_co	22	0.05	A|splice_donor_variant&intron_variant|HIGH|TTC12|ENSG0000014
retained_intron			G|splice_acceptor_variant&intron_variant|HIGH|TTC12|ENSG00000(
protein_coding			C|structural_interaction_variant|HIGH|NNMT|ENSG00000166741|i
protein_coding			T|stop_gained|HIGH|RBM7|ENSG00000076053|transcript|ENST000
processed_pseudogene			TTATA|splice_acceptor_variant&intron_variant|HIGH|AP000936.5|I
processed_pseudogene			TTATATA|splice_acceptor_variant&intron_variant|HIGH|AP000936.!
protein_co	7	0.71	T|frameshift_variant|HIGH|TMPRSS13|ENSG00000137747|transcrip

protein_co	7	0.71	T|frameshift_variant|HIGH|TMPRSS13|ENSG00000137747|transcrip
protein_co	7	0.71	A|frameshift_variant|HIGH|TMPRSS13|ENSG00000137747|transcri
protein_co	7	0.71	A|frameshift_variant|HIGH|TMPRSS13|ENSG00000137747|transcri
protein_co	1	1	T|stop_gained|HIGH|AP002962.1|ENSG00000279906|transcript|EN
protein_co	20	0.2	G|splice_acceptor_variant&intron_variant|HIGH|TMPRSS4|ENSG00
protein_co	7	0.29	C|frameshift_variant|HIGH|MPZL2|ENSG00000149573|transcript|E
protein_coding			C|frameshift_variant|HIGH|TMEM25|ENSG00000149582|transcript
protein_co	17	0.24	A|frameshift_variant&splice_region_variant|HIGH|SLC37A4|ENSG00
protein_coding			C|splice_acceptor_variant&disruptive_inframe_deletion&splice_regi
protein_coding			A|structural_interaction_variant|HIGH|NLRX1|ENSG00000160703|i
protein_coding			C|structural_interaction_variant|HIGH|CBL|ENSG00000110395|inte
protein_coding			A|structural_interaction_variant|HIGH|USP2|ENSG00000036672|in
processed_transcript			G|splice_donor_variant&splice_region_variant&intron_variant&non
processed_transcript			T|splice_donor_variant&intron_variant|HIGH|TRIM29|ENSG000001
protein_coding			C|structural_interaction_variant|HIGH|SORL1|ENSG00000137642|i
protein_coding			A|structural_interaction_variant|HIGH|SORL1|ENSG00000137642|i
antisense			T|splice_donor_variant&intron_variant|HIGH|CTD-2216M2.1|ENSG
protein_co	16	0.06	G|start_lost|HIGH|GRAMD1B|ENSG00000023171|transcript|ENST0
protein_coding			G|stop_gained|HIGH|OR10D3|ENSG00000197309|transcript|ENST0
protein_coding			G|stop_gained|HIGH|OR8G1|ENSG00000197849|transcript|ENST0
protein_co	1	1	AG|frameshift_variant|HIGH|OR8B3|ENSG00000196661|transcript|
protein_coding			T|stop_gained|HIGH|ESAM|ENSG00000149564|transcript|ENST000
antisense			G|splice_acceptor_variant&intron_variant|HIGH|RP11-677M14.7|E
processed_transcript			T|splice_donor_variant&intron_variant|HIGH|PKNOX2|ENSG000001
protein_co	15	0.07	A|stop_gained|HIGH|FEZ1|ENSG00000149557|transcript|ENST000
protein_co	18	0.11	A|stop_gained|HIGH|CHEK1|ENSG00000149554|transcript|ENST00
protein_co	18	0.11	AT|splice_acceptor_variant&intron_variant|HIGH|CHEK1|ENSG000
protein_co	3	0.33	C|splice_acceptor_variant&intron_variant|HIGH|PATE4|ENSG00000
protein_coding			C|protein_protein_contact|HIGH|CDON|ENSG00000064309|interac
protein_coding			T|structural_interaction_variant|HIGH|TIRAP|ENSG00000150455|ir
nonsense_	7	0.14	CTTT|splice_acceptor_variant&intron_variant|HIGH|TIRAP|ENSG000
nonsense_	7	0.14	CTTT|splice_acceptor_variant&intron_variant|HIGH|TIRAP|ENSG000
nonsense_	7	0.14	CTTT|splice_acceptor_variant&intron_variant|HIGH|TIRAP|ENSG000
protein_coding			CTGGGGA|structural_interaction_variant|HIGH|DCPS|ENSG0000011
pseudogene			GC|splice_acceptor_variant&splice_donor_variant&intron_variant|H
protein_coding			C|structural_interaction_variant|HIGH|ETS1|ENSG00000134954|int
protein_co	7	0.57	T|stop_gained|HIGH|TP53AIP1|ENSG00000120471|transcript|ENST
processed_transcript			ATT|splice_acceptor_variant&intron_variant|HIGH|APLP2|ENSG000
processed_transcript			ATT|splice_acceptor_variant&intron_variant|HIGH|APLP2|ENSG000
processed_transcript			ATT|splice_acceptor_variant&intron_variant|HIGH|APLP2|ENSG000
processed_pseudogene			TTTTTTTTTT|splice_acceptor_variant&intron_variant|HIGH|RP11-56
processed_pseudogene			TTTTTTTTTT|splice_acceptor_variant&intron_variant|HIGH|RP11-56
lincRNA			G|splice_donor_variant&intron_variant|HIGH|RP11-890B15.2|ENSG
lincRNA			A|splice_donor_variant&intron_variant|HIGH|AP002856.6|ENSG000
lincRNA			C|splice_donor_variant&intron_variant|HIGH|AP005135.2|ENSG000
processed_transcript			A|splice_donor_variant&intron_variant|HIGH|SLC6A12|ENSG00000
protein_co	12	0.08	C|frameshift_variant|HIGH|SLC6A13|ENSG00000010379|transcript

protein_coding			A|stop_gained|HIGH|B4GALNT3|ENSG00000139044|transcript|ENS
protein_co	21	0.05	TC|frameshift_variant|HIGH|WNK1|ENSG00000060237|transcript|I
protein_co	21	0.05	TC|frameshift_variant|HIGH|WNK1|ENSG00000060237|transcript|I
protein_co	21	0.05	TC|frameshift_variant|HIGH|WNK1|ENSG00000060237|transcript|I
protein_coding			C|stop_gained|HIGH|RAD52|ENSG00000002016|transcript|ENST00
protein_co	19	0.11	T|stop_gained|HIGH|RAD52|ENSG00000002016|transcript|ENST00
nonsense_	19	0.16	CT|frameshift_variant|HIGH|RAD52|ENSG00000002016|transcript|
protein_co	31	0.03	C|start_lost|HIGH|CACNA1C|ENSG00000151067|transcript|ENST00
protein_coding			A|structural_interaction_variant|HIGH|FKBP4|ENSG00000004478|i
protein_co	10	0.1	A|splice_donor_variant&intron_variant|HIGH|RHNO1|ENSG000001
protein_co	8	0.5	G|splice_donor_variant&splice_region_variant&intron_variant|HIGH
protein_co	4	0.5	C|frameshift_variant|HIGH|PRMT8|ENSG00000111218|transcript|E
protein_co	6	0.33	TC|frameshift_variant|HIGH|AKAP3|ENSG00000111254|transcript|
protein_co	6	0.33	G|frameshift_variant|HIGH|AKAP3|ENSG00000111254|transcript|E
protein_co	5	0.2	GA|frameshift_variant|HIGH|GALNT8|ENSG00000130035|transcript
protein_coding			G|structural_interaction_variant|HIGH|VWF|ENSG00000110799|int
lincRNA			T|splice_donor_variant&intron_variant|HIGH|RP3-454B23.1|ENSG0
protein_co	9	0.44	C|frameshift_variant|HIGH|PLEKHG6|ENSG00000008323|transcript
protein_coding			C|structural_interaction_variant|HIGH|GAPDH|ENSG00000111640|
protein_coding			T|structural_interaction_variant|HIGH|GAPDH|ENSG00000111640|
protein_coding			C|structural_interaction_variant|HIGH|GAPDH|ENSG00000111640|
retained_intron			CAA|splice_donor_variant&intron_variant|HIGH|P3H3|ENSG000001
retained_intron			CAA|splice_donor_variant&intron_variant|HIGH|P3H3|ENSG000001
protein_coding			T|structural_interaction_variant|HIGH|USP5|ENSG00000111667|in
retained_intron			A|splice_acceptor_variant&intron_variant|HIGH|TPI1|ENSG000001
protein_co	5	0.6	T|splice_acceptor_variant&intron_variant|HIGH|SPSB2|ENSG00000
protein_co	5	0.2	A|splice_donor_variant&intron_variant|HIGH|SPSB2|ENSG0000011
protein_coding			C|structural_interaction_variant|HIGH|PTPN6|ENSG00000111679|i
protein_co	13	0.38	A|frameshift_variant|HIGH|LPCAT3|ENSG00000111684|transcript|I
antisense			C|splice_donor_variant&intron_variant|HIGH|C1RL-AS1|ENSG00000
protein_co	2	0.5	T|stop_gained|HIGH|ACSM4|ENSG00000215009|transcript|ENST00
protein_coding			T|frameshift_variant|HIGH|CD163L1|ENSG00000177675|transcript
protein_coding			T|frameshift_variant|HIGH|CD163L1|ENSG00000177675|transcript
protein_co	2	0.5	T|stop_gained|HIGH|APOBEC1|ENSG00000111701|transcript|ENST
protein_coding			C|frameshift_variant&stop_lost|HIGH|NANOG|ENSG00000111704|
protein_coding			T|frameshift_variant|HIGH|FAM90A1|ENSG00000171847|transcript
transcribed_unprocessed_pseudo			G|splice_donor_variant&intron_variant|HIGH|FAM86FP|ENSG00000
nonsense_	15	0.13	A|splice_donor_variant&intron_variant|HIGH|MFAP5|ENSG0000001
lincRNA			A|splice_donor_variant&splice_region_variant&intron_variant&non_
lincRNA			T|splice_donor_variant&intron_variant|HIGH|RP11-20D14.6|ENSG0
protein_co	9	0.33	GT|splice_acceptor_variant&intron_variant|HIGH|A2ML1|ENSG000
protein_co	9	0.33	G|frameshift_variant|HIGH|A2ML1|ENSG00000166535|transcript|E
protein_co	13	0.15	C|splice_acceptor_variant&splice_region_variant&intron_variant|HI
protein_co	7	0.14	A|stop_gained|HIGH|PZP|ENSG00000126838|transcript|ENST0000
processed_transcript			T|splice_donor_variant&intron_variant|HIGH|A2MP1|ENSG0000002
transcribed_unprocessed_pseudo			A|splice_donor_variant&intron_variant|HIGH|A2MP1|ENSG000002
processed_transcript			T|splice_donor_variant&intron_variant|HIGH|RP11-705C15.2|ENSG

processed_transcript			A|splice_donor_variant&intron_variant|HIGH|RP11-705C15.2|ENSG
processed_transcript			GT|splice_acceptor_variant&intron_variant|HIGH|RP11-705C15.2|E
processed_transcript			GT|splice_acceptor_variant&intron_variant|HIGH|RP11-705C15.2|E
protein_co	4	0.5	ATAAGT|frameshift_variant|HIGH|CLECL1|ENSG00000184293|trans
protein_coding			C|stop_gained|HIGH|CLEC7A|ENSG00000172243|transcript|ENST0(
processed_transcript			T|splice_acceptor_variant&intron_variant|HIGH|YBX3|ENSG000000
protein_coding			T|structural_interaction_variant|HIGH|LRP6|ENSG00000070018|int
protein_coding			C|structural_interaction_variant|HIGH|LRP6|ENSG00000070018|int
protein_co	4	0.75	CAA|frameshift_variant|HIGH|MANSC1|ENSG00000111261|transcr
protein_co	4	0.75	GA|frameshift_variant|HIGH|MANSC1|ENSG00000111261|transcrip
processed_pseudogene			CT|splice_acceptor_variant&intron_variant|HIGH|RPL21P136|ENSG
protein_co	5	0.6	A|splice_donor_variant&intron_variant|HIGH|APOLD1|ENSG00000
processed_transcript			T|splice_donor_variant&intron_variant|HIGH|DDX47|ENSG0000021
protein_co	9	0.44	A|stop_gained|HIGH|GSG1|ENSG00000111305|transcript|ENST000
lincRNA			A|splice_donor_variant&intron_variant|HIGH|LINC01559|ENSG0000
protein_co	4	0.25	C|frameshift_variant|HIGH|C12orf60|ENSG00000182993|transcript
protein_coding			C|structural_interaction_variant|HIGH|ERP27|ENSG00000139055|ir
protein_coding			A|structural_interaction_variant|HIGH|PTPRO|ENSG00000151490|i
nonsense_	7	0.57	A|splice_donor_variant&intron_variant|HIGH|PIK3C2G|ENSG00000
protein_coding			C|stop_lost|HIGH|PIK3C2G|ENSG00000139144|transcript|ENST000
protein_co	5	0.60,(RP11	TAATTG|frameshift_variant|HIGH|SLCO1B3|ENSG00000111700|trai
protein_co	17	0.29	C|frameshift_variant|HIGH|SLCO1A2|ENSG00000084453|transcript
processed_pseudogene			G|splice_acceptor_variant&intron_variant|HIGH|TCEB1P31|ENSG00
antisense			G|splice_acceptor_variant&splice_region_variant&intron_variant&n
protein_co	10	0.1	TACTC|splice_donor_variant&intron_variant|HIGH|ITPR2|ENSG0000
antisense			C|splice_donor_variant&intron_variant|HIGH|RP11-1060J15.4|ENS(
protein_co	22	0.05	G|start_lost|HIGH|CCDC91|ENSG00000123106|transcript|ENST000
antisense			T|splice_donor_variant&intron_variant|HIGH|RP11-310I24.1|ENSG(
protein_co	19	0.05	T|splice_donor_variant&intron_variant|HIGH|CAPRIN2|ENSG00000
lincRNA			ATT|splice_acceptor_variant&intron_variant|HIGH|LINC00941|ENS(
lincRNA			ATT|splice_acceptor_variant&intron_variant|HIGH|LINC00941|ENS(
processed_transcript			T|splice_donor_variant&intron_variant|HIGH|RP11-551L14.4|ENSG
protein_co	30	0.03	A|stop_gained|HIGH|DDX11|ENSG00000013573|transcript|ENST0C
unprocessed_pseudogene			A|splice_donor_variant&intron_variant|HIGH|CLUHP8|ENSG000002
unprocessed_pseudogene			A|splice_donor_variant&intron_variant|HIGH|CLUHP8|ENSG000002
processed_transcript			A|splice_acceptor_variant&intron_variant|HIGH|MUC19|ENSG0000C
processed_transcript			C|splice_donor_variant&intron_variant|HIGH|MUC19|ENSG000002
processed_transcript			A|splice_donor_variant&intron_variant|HIGH|MUC19|ENSG000002
processed_transcript			A|splice_acceptor_variant&intron_variant|HIGH|MUC19|ENSG0000C
protein_co	26	0.04	C|start_lost&disruptive_inframe_deletion|HIGH|PPHLN1|ENSG0000C
protein_co	9	0.11	T|splice_donor_variant&intron_variant|HIGH|PRICKLE1|ENSG0000C
snRNA			A|transcript_ablation|HIGH|U6|ENSG00000283545|transcript|ENST
processed_transcript			G|splice_acceptor_variant&intron_variant|HIGH|ARID2|ENSG00000C
protein_co	30	0.23	C|splice_donor_variant&intron_variant|HIGH|HDAC7|ENSG0000006
protein_co	8	0.88	G|start_lost|HIGH|VDR|ENSG00000111424|transcript|ENST000005
antisense			A|splice_acceptor_variant&intron_variant|HIGH|RP1-228P16.4|ENS
nonsense_mediated_decay			T|stop_gained|HIGH|PFKM|ENSG00000152556|transcript|ENST00C

<table>
<tr><td>protein_co</td><td>1</td><td>1</td><td>CA|frameshift_variant|HIGH|OR10AD1|ENSG00000172640|transcri</td></tr>
<tr><td>antisense</td><td></td><td></td><td>CTTT|splice_acceptor_variant&intron_variant|HIGH|RP11-370I10.6|</td></tr>
<tr><td>antisense</td><td></td><td></td><td>CTTT|splice_acceptor_variant&intron_variant|HIGH|RP11-370I10.6|</td></tr>
<tr><td>antisense</td><td></td><td></td><td>CTTT|splice_acceptor_variant&intron_variant|HIGH|RP11-370I10.6|</td></tr>
<tr><td>protein_co</td><td>2</td><td>1</td><td>A|splice_donor_variant&intron_variant|HIGH|LINC00935|ENSG0000</td></tr>
<tr><td>protein_coding</td><td></td><td></td><td>A|stop_gained|HIGH|FKBP11|ENSG00000134285|transcript|ENST0</td></tr>
<tr><td>protein_coding</td><td></td><td></td><td>A|protein_protein_contact|HIGH|DHH|ENSG00000139549|interact</td></tr>
<tr><td>lincRNA</td><td></td><td></td><td>A|splice_donor_variant&intron_variant|HIGH|RP11-70F11.8|ENSG0</td></tr>
<tr><td>protein_coding</td><td></td><td></td><td>C|structural_interaction_variant|HIGH|AQP2|ENSG00000167580|in</td></tr>
<tr><td>protein_co</td><td>4</td><td>0.5</td><td>TGA|frameshift_variant|HIGH|FAM186A|ENSG00000185958|transc</td></tr>
<tr><td>protein_co</td><td>4</td><td>0.5</td><td>CCTGCTGAGGGGTGAGAGGGATCCCCAGGGCCTGG|frameshift_varian</td></tr>
<tr><td>protein_co</td><td>4</td><td>0.5</td><td>CCTGCTGAGGGGTGAGAGGGATCCCCAGGGCCTGG|frameshift_varian</td></tr>
<tr><td>nonsense_mediated_decay</td><td></td><td></td><td>G|stop_lost|HIGH|SLC11A2|ENSG00000110911|transcript|ENST000</td></tr>
<tr><td>protein_co</td><td>1</td><td>1</td><td>AG|frameshift_variant|HIGH|CELA1|ENSG00000139610|transcript|</td></tr>
<tr><td>protein_coding</td><td></td><td></td><td>C|frameshift_variant&splice_region_variant|HIGH|CELA1|ENSG0000</td></tr>
<tr><td>protein_coding</td><td></td><td></td><td>AAG|frameshift_variant|HIGH|CELA1|ENSG00000139610|transcript</td></tr>
<tr><td>protein_coding</td><td></td><td></td><td>CG|frameshift_variant|HIGH|CELA1|ENSG00000139610|transcript|</td></tr>
<tr><td>processed_transcript</td><td></td><td></td><td>A|splice_donor_variant&intron_variant|HIGH|ANKRD33|ENSG0000</td></tr>
<tr><td>processed_transcript</td><td></td><td></td><td>A|splice_donor_variant&intron_variant|HIGH|KRT87P|ENSG000001</td></tr>
<tr><td>protein_co</td><td>1</td><td>1</td><td>T|stop_gained|HIGH|KRT83|ENSG00000170523|transcript|ENST000</td></tr>
<tr><td>protein_co</td><td>1</td><td>1</td><td>A|synonymous_variant|LOW|KRT83|ENSG00000170523|transcript|</td></tr>
<tr><td>protein_co</td><td>1</td><td>1</td><td>A|stop_gained|HIGH|KRT83|ENSG00000170523|transcript|ENST00</td></tr>
<tr><td>protein_co</td><td>1</td><td>1</td><td>A|stop_gained|HIGH|KRT76|ENSG00000185069|transcript|ENST00</td></tr>
<tr><td>protein_co</td><td>1</td><td>1</td><td>TA|splice_donor_variant&intron_variant|HIGH|KRT3|ENSG0000018</td></tr>
<tr><td>protein_coding</td><td></td><td></td><td>T|structural_interaction_variant|HIGH|TNS2|ENSG00000111077|in</td></tr>
<tr><td>protein_coding</td><td></td><td></td><td>T|structural_interaction_variant|HIGH|CSAD|ENSG00000139631|in</td></tr>
<tr><td>protein_coding</td><td></td><td></td><td>A|frameshift_variant|HIGH|PRR13|ENSG00000205352|transcript|E</td></tr>
<tr><td>protein_co</td><td>11</td><td>0.09</td><td>T|splice_donor_variant&intron_variant|HIGH|MAP3K12|ENSG0000</td></tr>
<tr><td>protein_co</td><td>18</td><td>0.06</td><td>G|splice_acceptor_variant&intron_variant|HIGH|COPZ1|ENSG0000</td></tr>
<tr><td>protein_co</td><td>18</td><td>0.11</td><td>G|start_lost|HIGH|COPZ1|ENSG00000111481|transcript|ENST0000</td></tr>
<tr><td>processed_transcript</td><td></td><td></td><td>G|splice_donor_variant&splice_region_variant&intron_variant|HIGH</td></tr>
<tr><td>protein_coding</td><td></td><td></td><td>C|structural_interaction_variant|HIGH|PDE1B|ENSG00000123360|i</td></tr>
<tr><td>nonsense_</td><td>3</td><td>0.33</td><td>T|frameshift_variant|HIGH|DCD|ENSG00000161634|transcript|ENS</td></tr>
<tr><td>lincRNA</td><td></td><td></td><td>G|splice_acceptor_variant&intron_variant|HIGH|RP11-850F7.7|ENS</td></tr>
<tr><td>protein_coding</td><td></td><td></td><td>A|stop_gained|HIGH|TESPA1|ENSG00000135426|transcript|ENST0</td></tr>
<tr><td>protein_coding</td><td></td><td></td><td>A|frameshift_variant|HIGH|OR9K2|ENSG00000170605|transcript|E</td></tr>
<tr><td>protein_coding</td><td></td><td></td><td>T|stop_gained|HIGH|OR6C74|ENSG00000197706|transcript|ENST0</td></tr>
<tr><td>protein_coding</td><td></td><td></td><td>CA|frameshift_variant|HIGH|OR6C1|ENSG00000205330|transcript|</td></tr>
<tr><td>protein_coding</td><td></td><td></td><td>C|frameshift_variant|HIGH|OR6C76|ENSG00000185821|transcript|</td></tr>
<tr><td>protein_coding</td><td></td><td></td><td>A|protein_protein_contact|HIGH|CDK2|ENSG00000123374|interac</td></tr>
<tr><td>protein_coding</td><td></td><td></td><td>T|protein_protein_contact|HIGH|RNF41|ENSG00000181852|intera</td></tr>
<tr><td>protein_co</td><td>10</td><td>0.1</td><td>T|start_lost|HIGH|COQ10A|ENSG00000135469|transcript|ENST000</td></tr>
<tr><td>protein_co</td><td>7</td><td>0.14</td><td>C|frameshift_variant&start_lost|HIGH|PTGES3|ENSG00000110958|</td></tr>
<tr><td>protein_coding</td><td></td><td></td><td>G|structural_interaction_variant|HIGH|PRIM1|ENSG00000198056|i</td></tr>
<tr><td>protein_co</td><td>3</td><td>0.33</td><td>A|frameshift_variant|HIGH|GPR182|ENSG00000166856|transcript|</td></tr>
<tr><td>protein_coding</td><td></td><td></td><td>A|stop_gained|HIGH|GPR182|ENSG00000166856|transcript|ENST0</td></tr>
<tr><td>protein_co</td><td>24</td><td>0.04</td><td>G|start_lost|HIGH|ARHGAP9|ENSG00000123329|transcript|ENST00</td></tr>
</table>

<table>
<tr><td>nonsense_</td><td>10</td><td>0.1</td><td>G|splice_donor_variant&intron_variant|HIGH|SLC26A10|ENSG0000</td></tr>
<tr><td>protein_coding</td><td></td><td></td><td>C|structural_interaction_variant|HIGH|METTL21B|ENSG00000123433</td></tr>
<tr><td>protein_co</td><td>10</td><td>0.10,(RP11</td><td>T|splice_donor_variant&intron_variant|HIGH|RP11-571M6.15|ENS</td></tr>
<tr><td>lincRNA</td><td></td><td></td><td>G|splice_donor_variant&intron_variant|HIGH|RP11-767I20.1|ENSG</td></tr>
<tr><td>nonsense_</td><td>16</td><td>0.06</td><td>GA|frameshift_variant|HIGH|SLC16A7|ENSG00000118596|transcrip</td></tr>
<tr><td>lincRNA</td><td></td><td></td><td>CT|splice_acceptor_variant&intron_variant|HIGH|RP11-316A16.1|E</td></tr>
<tr><td>lincRNA</td><td></td><td></td><td>CTT|splice_acceptor_variant&intron_variant|HIGH|RP11-316A16.1|</td></tr>
<tr><td>lincRNA</td><td></td><td></td><td>CTTT|splice_acceptor_variant&intron_variant|HIGH|RP11-316A16.1</td></tr>
<tr><td>protein_co</td><td>13</td><td>0.38</td><td>GT|splice_acceptor_variant&intron_variant|HIGH|MON2|ENSG0000</td></tr>
<tr><td>transcribed_unprocessed_pseudo</td><td></td><td></td><td>G|splice_donor_variant&intron_variant|HIGH|RP11-415I12.2|ENSG</td></tr>
<tr><td>protein_co</td><td>7</td><td>0.43</td><td>GTT|splice_acceptor_variant&intron_variant|HIGH|SRGAP1|ENSG00</td></tr>
<tr><td>protein_co</td><td>7</td><td>0.43</td><td>GTT|splice_acceptor_variant&intron_variant|HIGH|SRGAP1|ENSG00</td></tr>
<tr><td>antisense</td><td></td><td></td><td>GTT|splice_acceptor_variant&intron_variant|HIGH|RP11-168J19.2|</td></tr>
<tr><td>antisense</td><td></td><td></td><td>G|splice_region_variant&intron_variant|LOW|RP11-168J19.2|ENSG</td></tr>
<tr><td>protein_coding</td><td></td><td></td><td>G|structural_interaction_variant|HIGH|WIF1|ENSG00000156076|in</td></tr>
<tr><td>protein_co</td><td>6</td><td>0.67</td><td>A|frameshift_variant|HIGH|HELB|ENSG00000127311|transcript|EN</td></tr>
<tr><td>nonsense_</td><td>7</td><td>0.14</td><td>G|splice_acceptor_variant&intron_variant|HIGH|CPSF6|ENSG00000</td></tr>
<tr><td>protein_coding</td><td></td><td></td><td>A|protein_protein_contact|HIGH|CPSF6|ENSG00000111605|interac</td></tr>
<tr><td>protein_co</td><td>12</td><td>0.25</td><td>G|frameshift_variant|HIGH|BEST3|ENSG00000127325|transcript|E</td></tr>
<tr><td>protein_co</td><td>18</td><td>0.06</td><td>T|splice_donor_variant&intron_variant|HIGH|RAB3IP|ENSG0000012</td></tr>
<tr><td>processed_</td><td>43</td><td>0.05</td><td>ATT|splice_acceptor_variant&intron_variant|HIGH|CNOT2|ENSG000</td></tr>
<tr><td>processed_</td><td>43</td><td>0.05</td><td>ATT|splice_acceptor_variant&intron_variant|HIGH|CNOT2|ENSG000</td></tr>
<tr><td>protein_coding</td><td></td><td></td><td>A|structural_interaction_variant|HIGH|PTPRB|ENSG00000127329|i</td></tr>
<tr><td>protein_coding</td><td></td><td></td><td>G|structural_interaction_variant|HIGH|PTPRB|ENSG00000127329|i</td></tr>
<tr><td>protein_coding</td><td></td><td></td><td>A|structural_interaction_variant|HIGH|LGR5|ENSG00000139292|in</td></tr>
<tr><td>protein_coding</td><td></td><td></td><td>G|structural_interaction_variant|HIGH|LGR5|ENSG00000139292|in</td></tr>
<tr><td>protein_coding</td><td></td><td></td><td>A|structural_interaction_variant|HIGH|LGR5|ENSG00000139292|in</td></tr>
<tr><td>retained_intron</td><td></td><td></td><td>C|splice_donor_variant&intron_variant|HIGH|LGR5|ENSG00000139</td></tr>
<tr><td>protein_coding</td><td></td><td></td><td>G|structural_interaction_variant|HIGH|LGR5|ENSG00000139292|in</td></tr>
<tr><td>protein_coding</td><td></td><td></td><td>A|structural_interaction_variant|HIGH|LGR5|ENSG00000139292|in</td></tr>
<tr><td>protein_coding</td><td></td><td></td><td>T|structural_interaction_variant|HIGH|TPH2|ENSG00000139287|in</td></tr>
<tr><td>processed_transcript</td><td></td><td></td><td>G|splice_acceptor_variant&intron_variant|HIGH|TRHDE|ENSG00000</td></tr>
<tr><td>protein_co</td><td>4</td><td>0.75</td><td>A|frameshift_variant|HIGH|GLIPR1L2|ENSG00000180481|transcript</td></tr>
<tr><td>processed_transcript</td><td></td><td></td><td>ATT|splice_acceptor_variant&intron_variant|HIGH|NAV3|ENSG0000</td></tr>
<tr><td>processed_transcript</td><td></td><td></td><td>ATT|splice_acceptor_variant&intron_variant|HIGH|NAV3|ENSG0000</td></tr>
<tr><td>processed_transcript</td><td></td><td></td><td>ATT|splice_acceptor_variant&intron_variant|HIGH|NAV3|ENSG0000</td></tr>
<tr><td>processed_transcript</td><td></td><td></td><td>ATT|splice_acceptor_variant&intron_variant|HIGH|NAV3|ENSG0000</td></tr>
<tr><td>lincRNA</td><td></td><td></td><td>A|splice_donor_variant&intron_variant|HIGH|RP11-754N21.1|ENSG</td></tr>
<tr><td>processed_transcript</td><td></td><td></td><td>A|splice_donor_variant&intron_variant|HIGH|SYT1|ENSG00000067</td></tr>
<tr><td>protein_coding</td><td></td><td></td><td>T|structural_interaction_variant|HIGH|SYT1|ENSG00000067715|int</td></tr>
<tr><td>protein_coding</td><td></td><td></td><td>G|frameshift_variant|HIGH|LRRIQ1|ENSG00000133640|transcript|</td></tr>
<tr><td>lincRNA</td><td></td><td></td><td>T|splice_acceptor_variant&intron_variant|HIGH|RP11-13A1.1|ENSG</td></tr>
<tr><td>protein_co</td><td>5</td><td>0.2</td><td>G|start_lost|HIGH|DUSP6|ENSG00000139318|transcript|ENST0000</td></tr>
<tr><td>lincRNA</td><td></td><td></td><td>TTTTTGTTTTG|splice_acceptor_variant&intron_variant|HIGH|RP11-</td></tr>
<tr><td>lincRNA</td><td></td><td></td><td>TTTTTGTTTTG|splice_acceptor_variant&intron_variant|HIGH|RP11-</td></tr>
<tr><td>antisense</td><td></td><td></td><td>G|splice_donor_variant&intron_variant|HIGH|RP11-486A14.2|ENSG</td></tr>
<tr><td>processed_transcript</td><td></td><td></td><td>GTTTTACTATCTGCTAGGCCC|splice_donor_variant&intron_variant|H</td></tr>
</table>

processed_transcript			GTTTTACTATCTGCTAGGCCC|splice_donor_variant&intron_variant|H
sense_intronic			GT|splice_acceptor_variant&intron_variant|HIGH|RP11-887P2.6|EN
protein_coding			T|structural_interaction_variant|HIGH|PLXNC1|ENSG00000136040|
protein_co	11	0.36	TC|splice_acceptor_variant&intron_variant|HIGH|PLXNC1|ENSG000
protein_co	11	0.36	TC|splice_acceptor_variant&intron_variant|HIGH|PLXNC1|ENSG000
nonsense_	9	0.11	T|splice_donor_variant&intron_variant|HIGH|NDUFA12|ENSG00000
processed_transcript			T|splice_donor_variant&intron_variant|HIGH|RP11-167N24.3|ENSG
protein_coding			T|structural_interaction_variant|HIGH|METAP2|ENSG00000111142
protein_coding			A|structural_interaction_variant|HIGH|METAP2|ENSG00000111142
lincRNA			C|splice_donor_variant&intron_variant|HIGH|RMST|ENSG0000025
processed_transcript			C|splice_acceptor_variant&intron_variant|HIGH|GOLGA2P5|ENSG0
protein_co	23	0.04	G|splice_donor_variant&intron_variant|HIGH|MYBPC1|ENSG00000
nonsense_	10	0.1	C|splice_donor_variant&intron_variant|HIGH|CHPT1|ENSG0000011
protein_coding			T|frameshift_variant&stop_lost|HIGH|CHPT1|ENSG00000111666|tr
protein_coding			T|structural_interaction_variant|HIGH|PAH|ENSG00000171759|inte
)171759			C|bidirectional_gene_fusion|HIGH|ASCL1&PAH|ENSG00000139352
)171759			C|bidirectional_gene_fusion|HIGH|ASCL1&PAH|ENSG00000139352
)171759			C|bidirectional_gene_fusion|HIGH|ASCL1&PAH|ENSG00000139352
)171759			C|bidirectional_gene_fusion|HIGH|ASCL1&PAH|ENSG00000139352
processed_transcript			G|splice_donor_variant&intron_variant|HIGH|TTC41P|ENSG0000002
protein_co	10	0.4	GA|splice_donor_variant&intron_variant|HIGH|TDG|ENSG0000013
protein_co	10	0.4	C|splice_donor_variant&intron_variant|HIGH|TDG|ENSG000001393
protein_co	10	0.4	ATT|splice_donor_variant&intron_variant|HIGH|TDG|ENSG0000013
protein_co	10	0.4	ATT|splice_donor_variant&intron_variant|HIGH|TDG|ENSG0000013
protein_co	10	0.4	ATT|splice_donor_variant&intron_variant|HIGH|TDG|ENSG0000013
protein_co	10	0.4	T|splice_donor_variant&intron_variant|HIGH|TDG|ENSG000001393
protein_co	10	0.4	T|splice_donor_variant&intron_variant|HIGH|TDG|ENSG000001393
processed_transcript			T|splice_donor_variant&intron_variant|HIGH|HCFC2|ENSG0000011
protein_co	4	0.75	CTTT|splice_acceptor_variant&intron_variant|HIGH|POLR3B|ENSG0
protein_co	4	0.75	CTTT|splice_acceptor_variant&intron_variant|HIGH|POLR3B|ENSG0
protein_co	4	0.75	CTTT|splice_acceptor_variant&intron_variant|HIGH|POLR3B|ENSG0
protein_coding			A|frameshift_variant|HIGH|ASCL4|ENSG00000187855|transcript|EI
protein_coding			C|structural_interaction_variant|HIGH|FICD|ENSG00000198855|int
protein_co	3	0.33	G|frameshift_variant&splice_acceptor_variant&splice_region_variar
protein_coding			A|structural_interaction_variant|HIGH|DAO|ENSG00000110887|int
protein_coding			T|structural_interaction_variant|HIGH|ALKBH2|ENSG00000189046|
protein_coding			C|structural_interaction_variant|HIGH|ACACB|ENSG00000076555|i
protein_coding			A|structural_interaction_variant|HIGH|ACACB|ENSG00000076555|i
protein_coding			A|stop_gained|HIGH|FOXN4|ENSG00000139445|transcript|ENST0C
protein_coding			A|stop_gained|HIGH|UBE3B|ENSG00000151148|transcript|ENST00
protein_coding			A|structural_interaction_variant|HIGH|MVK|ENSG00000110921|int
protein_coding			A|structural_interaction_variant|HIGH|MVK|ENSG00000110921|int
protein_coding			A|structural_interaction_variant|HIGH|TRPV4|ENSG00000111199|i
protein_coding			G|structural_interaction_variant|HIGH|TRPV4|ENSG00000111199|i
processed_transcript			A|splice_donor_variant&intron_variant|HIGH|ANAPC7|ENSG00000
protein_coding			T|structural_interaction_variant|HIGH|PPP1CC|ENSG00000186298|
protein_co	4	0.75	G|frameshift_variant|HIGH|CCDC63|ENSG00000173093|transcript|

protein_co	27	0.11	G|frameshift_variant|HIGH|ATXN2|ENSG00000204842|transcript|E
protein_co	27	0.11	G|frameshift_variant|HIGH|ATXN2|ENSG00000204842|transcript|E
protein_co	27	0.11	T|frameshift_variant|HIGH|ATXN2|ENSG00000204842|transcript|E
retained_intron			A|splice_acceptor_variant&intron_variant|HIGH|MAPKAPK5|ENSG(
nonsense_mediated_decay			T|stop_gained|HIGH|TMEM116|ENSG00000198270|transcript|ENS
protein_co	11	0.09	A|splice_acceptor_variant&intron_variant|HIGH|OAS1|ENSG00000(
protein_coding			T|frameshift_variant|HIGH|OAS1|ENSG00000089127|transcript|EN
protein_coding			T|structural_interaction_variant|HIGH|OAS3|ENSG00000111331|in
protein_co	7	0.14	T|stop_gained|HIGH|OAS3|ENSG00000111331|transcript|ENST000
protein_co	7	0.14	G|stop_lost|HIGH|OAS2|ENSG00000111335|transcript|ENST00000
processed_transcript			T|splice_donor_variant&intron_variant|HIGH|DTX1|ENSG00000135
lincRNA			C|splice_donor_variant&intron_variant|HIGH|RP11-100F15.2|ENSG
protein_co	9	0.22	G|frameshift_variant|HIGH|RNFT2|ENSG00000135119|transcript|E
protein_co	9	0.22	TG|frameshift_variant|HIGH|RNFT2|ENSG00000135119|transcript|
protein_coding			G|structural_interaction_variant|HIGH|NOS1|ENSG00000089250|ir
antisense			A|splice_donor_variant&intron_variant|HIGH|RP11-465L8.1|ENSG0
protein_co	10	0.2	A|frameshift_variant&splice_acceptor_variant&splice_region_varian
protein_coding			A|stop_gained|HIGH|COQ5|ENSG00000110871|transcript|ENST000
protein_coding			A|frameshift_variant|HIGH|CABP1|ENSG00000157782|transcript|E
protein_coding			C|frameshift_variant|HIGH|CABP1|ENSG00000157782|transcript|E
protein_coding			T|frameshift_variant|HIGH|CABP1|ENSG00000157782|transcript|E
protein_coding			T|frameshift_variant|HIGH|CABP1|ENSG00000157782|transcript|E
protein_coding			T|frameshift_variant|HIGH|CABP1|ENSG00000157782|transcript|E
protein_coding			C|structural_interaction_variant|HIGH|ACADS|ENSG00000122971|i
protein_coding			T|structural_interaction_variant|HIGH|ACADS|ENSG00000122971|i
protein_coding			A|structural_interaction_variant|HIGH|OASL|ENSG00000135114|in
protein_co	16	0.06	C|frameshift_variant|HIGH|CAMKK2|ENSG00000110931|transcript
protein_co	16	0.06	C|frameshift_variant|HIGH|CAMKK2|ENSG00000110931|transcript
protein_co	16	0.06	C|frameshift_variant|HIGH|CAMKK2|ENSG00000110931|transcript
protein_co	16	0.06	C|frameshift_variant|HIGH|CAMKK2|ENSG00000110931|transcript
lincRNA			T|splice_donor_variant&intron_variant|HIGH|RP11-711D18.2|ENSG
protein_co	20	0.1	C|frameshift_variant|HIGH|KDM2B|ENSG00000089094|transcript|I
protein_co	4	1	C|frameshift_variant|HIGH|SETD1B|ENSG00000139718|transcript|I
protein_co	11	0.09	TC|frameshift_variant|HIGH|MLXIP|ENSG00000175727|transcript|I
TEC			T|splice_acceptor_variant&intron_variant|HIGH|RP11-512M8.11|EI
protein_coding			C|structural_interaction_variant|HIGH|CLIP1|ENSG00000130779|in
protein_co	9	0.11	A|splice_donor_variant&intron_variant|HIGH|CCDC92|ENSG000001
protein_co	5	0.20,(ZNF6	GACCT|splice_donor_variant&intron_variant|HIGH|ZNF664|ENSG0(
protein_co	5	0.20,(ZNF6	GACCT|splice_donor_variant&intron_variant|HIGH|ZNF664|ENSG0(
protein_co	5	0.20,(ZNF6	GACCT|splice_donor_variant&intron_variant|HIGH|ZNF664|ENSG0(
protein_co	5	0.20,(ZNF6	GCAAATCCAAGAGACACATCTTTGGAAGA|splice_donor_variant&intro
protein_coding			A|splice_acceptor_variant&splice_donor_variant&splice_region_var
protein_coding			A|stop_gained|HIGH|DHX37|ENSG00000150990|transcript|ENST00
lincRNA			C|splice_donor_variant&intron_variant|HIGH|LINC00943|ENSG000(
protein_coding			T|structural_interaction_variant|HIGH|PUS1|ENSG00000177192|in
protein_co	9	0.44	C|frameshift_variant|HIGH|EP400|ENSG00000183495|transcript|EI
protein_co	9	0.22	C|frameshift_variant|HIGH|EP400|ENSG00000183495|transcript|EI

protein_co	10	0.1	T|frameshift_variant|HIGH|GALNT9|ENSG00000182870|transcript|
protein_co	10	0.1	G|frameshift_variant|HIGH|GALNT9|ENSG00000182870|transcript|
protein_co	8	0.13	GC|splice_acceptor_variant&intron_variant|HIGH|FBRSL1|ENSG000
protein_co	5	0.4	G|frameshift_variant&splice_region_variant|HIGH|PXMP2|ENSG000
protein_coding			C|stop_lost|HIGH|PXMP2|ENSG00000176894|transcript|ENST0000
nonsense_	24	0.04	C|splice_donor_variant&intron_variant|HIGH|CHFR|ENSG00000072
unprocessed_pseudogene			AC|splice_donor_variant&intron_variant|HIGH|GGT4P|ENSG00000
transcribed_unprocessed_pseudo			A|splice_donor_variant&intron_variant|HIGH|PHF2P2|ENSG000002
unprocessed_pseudogene			A|splice_acceptor_variant&intron_variant|HIGH|GTF2IP3|ENSG000
antisense			A|splice_acceptor_variant&intron_variant|HIGH|RP11-301J16.7|EN
unprocessed_pseudogene			G|splice_donor_variant&intron_variant|HIGH|PSPC1P1|ENSG00000
unprocessed_pseudogene			C|splice_acceptor_variant&intron_variant|HIGH|ANKRD26P3|ENSG
unprocessed_pseudogene			G|splice_acceptor_variant&intron_variant|HIGH|PARP4P2|ENSG000
unprocessed_pseudogene			T|splice_acceptor_variant&splice_region_variant&intron_variant&n
nonsense_mediated_decay			A|stop_gained|HIGH|TPTE2|ENSG00000132958|transcript|ENST000
nonsense_	6	0.17	TGCGAA|frameshift_variant|HIGH|TPTE2|ENSG00000132958|trans
protein_coding			A|frameshift_variant|HIGH|GJB2|ENSG00000165474|transcript|EN
protein_coding			G|structural_interaction_variant|HIGH|CRYL1|ENSG00000165475|i
protein_co	3	0.33	GGGCCGCGGGCGCCCCGAGGGCGGGCAGGCGCCCCGCGC|frameshift_
protein_coding			TTTCAGTTTTCTTTGTTGCTGACATCTCGGATG|frameshift_variant&stop
protein_co	7	0.57	CAGTTTTCTT|splice_acceptor_variant&intron_variant|HIGH|SKA3|E
protein_co	7	0.57	CAGTTTTCTT|splice_acceptor_variant&intron_variant|HIGH|SKA3|E
protein_co	7	0.57	CAGTTTTCTT|splice_acceptor_variant&intron_variant|HIGH|SKA3|E
protein_co	7	0.43	CCAAAG|splice_acceptor_variant&intron_variant|HIGH|SKA3|ENSG
protein_co	7	0.43	CAAAGCTATGCTGTTCTTTGTAGATGGAATTTTCAAACCAGGAG|splice_a
protein_co	7	0.43	TCA|splice_acceptor_variant&intron_variant|HIGH|SKA3|ENSG000
protein_co	7	0.43	CACTTTTTTCCAACTGCTGGATGA|splice_acceptor_variant&intron_var
protein_co	7	0.29	T|frameshift_variant|HIGH|SKA3|ENSG00000165480|transcript|EN
protein_co	7	0.57	CCG|splice_acceptor_variant&intron_variant|HIGH|SKA3|ENSG000
protein_co	7	0.57	CGCTTTCCTCTCCGTCCAGCGCTCACTGCAGCCGGGCCG|splice_accepto
protein_co	7	0.29	A|stop_gained|HIGH|SKA3|ENSG00000165480|transcript|ENST000
sense_intronic			G|splice_acceptor_variant&splice_region_variant&intron_variant&n
protein_co	1	1	C|splice_donor_variant&intron_variant|HIGH|AL354828.2|ENSG000
protein_co	1	1	C|frameshift_variant|HIGH|AL354828.1|ENSG00000279282|transcr
protein_co	4	0.25	ACT|splice_acceptor_variant&intron_variant|HIGH|C1QTNF9B|ENS
protein_co	2	0.5	C|splice_acceptor_variant&splice_region_variant&intron_variant|HI
processed_pseudogene			T|splice_donor_variant&intron_variant|HIGH|PRUNEP1|ENSG00000
antisense			G|splice_donor_variant&intron_variant|HIGH|WASF3-AS1|ENSG000
lincRNA			CTAAA|splice_acceptor_variant&intron_variant|HIGH|RP11-428O18
processed_pseudogene			C|splice_acceptor_variant&intron_variant|HIGH|CYP51A1P2|ENSG
protein_co	4	0.5	A|splice_acceptor_variant&intron_variant|HIGH|TEX26|ENSG00000
unitary_pseudogene			C|splice_donor_variant&intron_variant|HIGH|WDR95P|ENSG00000
unitary_pseudogene			T|splice_donor_variant&splice_region_variant&intron_variant|HIGH
protein_co	2	1	TA|splice_acceptor_variant&intron_variant|HIGH|RXFP2|ENSG0000
protein_co	2	1	TA|splice_acceptor_variant&intron_variant|HIGH|RXFP2|ENSG0000
protein_co	2	1	TA|splice_acceptor_variant&intron_variant|HIGH|RXFP2|ENSG0000
nonsense_	7	0.14	CTT|splice_acceptor_variant&intron_variant|HIGH|BRCA2|ENSG000

nonsense_	7	0.14	CTT|splice_acceptor_variant&intron_variant|HIGH|BRCA2|ENSG000
protein_coding			T|stop_gained|HIGH|BRCA2|ENSG00000139618|transcript|ENST00
nonsense_	12	0.08	C|stop_gained|HIGH|N4BP2L1|ENSG00000139597|transcript|ENST
protein_co	9	0.22	CTT|splice_acceptor_variant&intron_variant|HIGH|PDS5B|ENSG000
protein_co	9	0.22	CTT|splice_acceptor_variant&intron_variant|HIGH|PDS5B|ENSG000
unprocessed_pseudogene			A|splice_acceptor_variant&intron_variant|HIGH|ANKRD26P2|ENSG
protein_co	12	0.33	AT|splice_acceptor_variant&intron_variant|HIGH|COG6|ENSG0000
protein_co	10	0.6	CTTTTTTT|splice_acceptor_variant&intron_variant|HIGH|DGKH|ENS
protein_co	10	0.6	CTTTTTTT|splice_acceptor_variant&intron_variant|HIGH|DGKH|ENS
protein_co	10	0.6	CTTTTTTT|splice_acceptor_variant&intron_variant|HIGH|DGKH|ENS
protein_co	10	0.6	CTTTTTTT|splice_acceptor_variant&intron_variant|HIGH|DGKH|ENS
protein_co	10	0.6	CTTTTTTT|splice_acceptor_variant&intron_variant|HIGH|DGKH|ENS
protein_co	10	0.6	CTTTTTTT|splice_acceptor_variant&intron_variant|HIGH|DGKH|ENS
processed_transcript			C|splice_donor_variant&intron_variant|HIGH|DGKH|ENSG0000010
protein_coding			C|structural_interaction_variant|HIGH|TNFSF11|ENSG0000012065
protein_coding			TTCAGG|frameshift_variant|HIGH|EPSTI1|ENSG00000133106|trans
protein_co	14	0.07	C|splice_acceptor_variant&intron_variant|HIGH|TPT1|ENSG000001
antisense			A|splice_acceptor_variant&intron_variant|HIGH|TPT1-AS1|ENSG000
protein_coding			A|stop_gained|HIGH|SPERT|ENSG00000174015|transcript|ENST000
protein_coding			A|stop_gained|HIGH|SPERT|ENSG00000174015|transcript|ENST000
protein_coding			G|structural_interaction_variant|HIGH|CPB2|ENSG00000080618|in
protein_co	4	0.25	C|frameshift_variant|HIGH|MED4|ENSG00000136146|transcript|EI
protein_co	1	1	A|start_lost|HIGH|MLNR|ENSG00000102539|transcript|ENST00000
protein_coding			A|stop_gained|HIGH|ARL11|ENSG00000152213|transcript|ENST000
)229323			A|bidirectional_gene_fusion|HIGH|DLEU1&DLEU1-AS1|ENSG000001
antisense			T|splice_donor_variant&intron_variant|HIGH|RNASEH2B-AS1|ENSG
protein_coding			TA|frameshift_variant|HIGH|RNASEH2B|ENSG00000136104|transcr
processed_transcript			T|splice_donor_variant&intron_variant|HIGH|GUCY1B2|ENSG00000
antisense			CTT|splice_acceptor_variant&intron_variant|HIGH|INTS6-AS1|ENSG
antisense			CTT|splice_acceptor_variant&intron_variant|HIGH|INTS6-AS1|ENSG
antisense			G|splice_acceptor_variant&intron_variant|HIGH|INTS6-AS1|ENSG00
protein_co	11	0.45	C|frameshift_variant&splice_region_variant|HIGH|NEK3|ENSG0000
unprocessed_pseudogene			A|splice_donor_variant&intron_variant|HIGH|TPTE2P3|ENSG00000
unprocessed_pseudogene			T|splice_donor_variant&intron_variant|HIGH|TPTE2P3|ENSG00000
protein_co	1	1	G|frameshift_variant|HIGH|HNRNPA1L2|ENSG00000139675|transc
lincRNA			C|splice_donor_variant&intron_variant|HIGH|LINC00458|ENSG0000
lincRNA			CTTTTT|splice_acceptor_variant&intron_variant|HIGH|RP11-538C21
lincRNA			CTTTTT|splice_acceptor_variant&intron_variant|HIGH|RP11-538C21
lincRNA			CTTTTT|splice_acceptor_variant&intron_variant|HIGH|RP11-538C21
lincRNA			CTTTTT|splice_acceptor_variant&intron_variant|HIGH|RP11-538C21
lincRNA			CTTTTT|splice_acceptor_variant&intron_variant|HIGH|RP11-538C21
lincRNA			C|splice_donor_variant&intron_variant|HIGH|LINC00348|ENSG0000
lincRNA			C|splice_acceptor_variant&intron_variant|HIGH|LINC00348|ENSG00
)118939			C|gene_fusion|HIGH|RP11-29G8.3&UCHL3|ENSG00000261553&EN
protein_co	19	0.05	C|frameshift_variant|HIGH|CLN5|ENSG00000102805|transcript|EN
protein_co	17	0.12	TGG|frameshift_variant|HIGH|SLAIN1|ENSG00000139737|transcrip
protein_co	17	0.12	GC|frameshift_variant|HIGH|SLAIN1|ENSG00000139737|transcript

lincRNA			C|splice_acceptor_variant&splice_region_variant&intron_variant|HI
lincRNA			C|splice_acceptor_variant&splice_region_variant&intron_variant|HI
lincRNA			C|splice_acceptor_variant&splice_region_variant&intron_variant|HI
lincRNA			C|splice_acceptor_variant&splice_region_variant&intron_variant|HI
antisense			G|splice_donor_variant&intron_variant|HIGH|RNF219-AS1|ENSG00
lincRNA			A|splice_donor_variant&intron_variant|HIGH|LINC00333|ENSG0000
lincRNA			A|splice_donor_variant&intron_variant|HIGH|LINC00430|ENSG0000
protein_co	1	1	CAG|frameshift_variant|HIGH|SOX21|ENSG00000125285|transcript
protein_co	1	1	CG|frameshift_variant|HIGH|SOX21|ENSG00000125285|transcript|
protein_co	1	1	CG|frameshift_variant|HIGH|SOX21|ENSG00000125285|transcript|
protein_co	1	1	CGGCCGACTGCGGGAAGAAGA|frameshift_variant|HIGH|SOX21|ENS
protein_co	1	1	GA|frameshift_variant|HIGH|SOX21|ENSG00000125285|transcript|
protein_coding			A|stop_lost&splice_region_variant|HIGH|OXGR1|ENSG0000016562
protein_co	7	0.29	C|frameshift_variant|HIGH|CLYBL|ENSG00000125246|transcript|EN
protein_co	7	0.29	C|frameshift_variant|HIGH|CLYBL|ENSG00000125246|transcript|EN
protein_co	7	0.57	T|stop_gained|HIGH|CLYBL|ENSG00000125246|transcript|ENST000
protein_co	5	0.2	C|frameshift_variant|HIGH|ZIC2|ENSG00000043355|transcript|ENS
)270632			G|bidirectional_gene_fusion|HIGH|PCCA&RP11-340C20.3|ENSG000
)270632			G|bidirectional_gene_fusion|HIGH|PCCA&RP11-340C20.3|ENSG000
)270632			G|bidirectional_gene_fusion|HIGH|PCCA&RP11-340C20.3|ENSG000
)270632			G|bidirectional_gene_fusion|HIGH|PCCA&RP11-340C20.3|ENSG000
)270632			G|bidirectional_gene_fusion|HIGH|PCCA&RP11-340C20.3|ENSG000
protein_coding			G|structural_interaction_variant|HIGH|METTL21C|ENSG000001397
lincRNA			A|splice_donor_variant&intron_variant|HIGH|LINC00343|ENSG0000
protein_coding			G|structural_interaction_variant|HIGH|LIG4|ENSG00000174405|int
protein_coding			A|structural_interaction_variant|HIGH|LIG4|ENSG00000174405|int
protein_co	3	0.33	A|splice_acceptor_variant&intron_variant|HIGH|RP11-153I24.5|ENS
antisense			G|splice_acceptor_variant&splice_region_variant&intron_variant&n
sense_intronic			G|splice_donor_variant&intron_variant|HIGH|ANKRD10-IT1|ENSG0
lincRNA			ATT|splice_acceptor_variant&intron_variant|HIGH|LINC00368|ENS
lincRNA			ATTT|splice_acceptor_variant&intron_variant|HIGH|LINC00368|ENS
lincRNA			ATTTT|splice_acceptor_variant&intron_variant|HIGH|LINC00368|EN
protein_co	32	0.03	T|frameshift_variant|HIGH|MCF2L|ENSG00000126217|transcript|E
protein_co	4	0.25	A|frameshift_variant|HIGH|ADPRHL1|ENSG00000153531|transcript
protein_co	4	0.25	C|frameshift_variant|HIGH|ADPRHL1|ENSG00000153531|transcript
lincRNA			C|splice_donor_variant&intron_variant|HIGH|LINC00452|ENSG0000
protein_co	2	0.5	G|splice_donor_variant&intron_variant|HIGH|ABC13-47488600E17.
protein_co	1	1	A|frameshift_variant|HIGH|OR11H12|ENSG00000257115|transcript
nonsense_	3	0.33	CTT|splice_acceptor_variant&intron_variant|HIGH|POTEM|ENSG00
nonsense_	3	0.33	CTT|splice_acceptor_variant&intron_variant|HIGH|POTEM|ENSG00
nonsense_	3	0.33	CTT|splice_acceptor_variant&intron_variant|HIGH|POTEM|ENSG00
protein_co	3	0.33	A|splice_acceptor_variant&intron_variant|HIGH|OR4N2|ENSG0000
polymorph	2	1	C|frameshift_variant|HIGH|OR4Q2|ENSG00000196383|transcript|E
protein_co	1	1	T|frameshift_variant|HIGH|OR4L1|ENSG00000176246|transcript|E
protein_co	1	1	CA|frameshift_variant|HIGH|OR11G2|ENSG00000196832|transcrip
polymorphic_pseudogene			C|stop_lost|HIGH|OR11H7|ENSG00000258806|transcript|ENST000
protein_coding			C|structural_interaction_variant|HIGH|APEX1|ENSG00000100823|i

protein_coding			T|structural_interaction_variant|HIGH|APEX1|ENSG00000100823|i
protein_coding			T|structural_interaction_variant|HIGH|PNP|ENSG00000198805|inte
protein_coding			T|structural_interaction_variant|HIGH|PNP|ENSG00000198805|inte
protein_co	11	0.18	T|splice_donor_variant&intron_variant|HIGH|RNASE9|ENSG000001
protein_co	2	0.5	T|splice_donor_variant&intron_variant|HIGH|EDDM3A|ENSG00000
protein_co	2	0.5	A|splice_donor_variant&intron_variant|HIGH|EDDM3A|ENSG00000
protein_co	25	0.04	C|frameshift_variant|HIGH|METTL17|ENSG00000165792|transcript
protein_co	25	0.04	TTG|frameshift_variant|HIGH|METTL17|ENSG00000165792|transcr
protein_coding			A|structural_interaction_variant|HIGH|NDRG2|ENSG00000165795|
nonsense_mediated_decay			G|stop_lost|HIGH|ARHGEF40|ENSG00000165801|transcript|ENST0
TR_V_gene	1	1	C|frameshift_variant|HIGH|TRAV19|ENSG00000211799|transcript|
TR_V_gene			A|stop_gained|HIGH|TRAV19|ENSG00000211799|transcript|ENST0
TR_V_gene	1	1	GC|frameshift_variant|HIGH|TRAV26-1|ENSG00000211807|transcri
TR_V_pseudogene			G|splice_acceptor_variant&intron_variant|HIGH|TRAV32|ENSG0000
TR_V_gene	1	1	A|splice_acceptor_variant&intron_variant|HIGH|TRAV26-2|ENSG00
protein_co	1	1	G|frameshift_variant|HIGH|PSMB11|ENSG00000222028|transcript
protein_coding			C|frameshift_variant|HIGH|RP11-244E17.1|ENSG00000283654|tran
protein_coding			C|downstream_gene_variant|MODIFIER|C14orf119|ENSG00000179
protein_coding			G|frameshift_variant|HIGH|RP11-244E17.1|ENSG00000283654|tran
protein_coding			A|stop_gained|HIGH|SLC7A8|ENSG00000092068|transcript|ENST0
protein_coding			A|structural_interaction_variant|HIGH|MYH7|ENSG00000092054|i
protein_coding			C|structural_interaction_variant|HIGH|DHRS4|ENSG00000157326|i
protein_coding			A|structural_interaction_variant|HIGH|DHRS4|ENSG00000157326|i
protein_coding			T|structural_interaction_variant|HIGH|DHRS4|ENSG00000157326|i
protein_co	11	0.09	C|splice_donor_variant&intron_variant|HIGH|DHRS4L2|ENSG00000
protein_co	11	0.45	T|stop_gained|HIGH|DHRS4L2|ENSG00000187630|transcript|ENST
protein_co	11	0.73	TA|frameshift_variant|HIGH|DHRS4L2|ENSG00000187630|transcrip
protein_coding			T|protein_protein_contact|HIGH|RNF31|ENSG00000092098|intera
protein_coding			A|structural_interaction_variant|HIGH|RNF31|ENSG00000092098|i
protein_co	15	0.07	G|splice_acceptor_variant&intron_variant|HIGH|IRF9|ENSG0000002
nonsense_	20	0.05	C|frameshift_variant|HIGH|IPO4|ENSG00000196497|transcript|ENS
protein_co	20	0.25,(RP11	C|splice_donor_variant&intron_variant|HIGH|IPO4|ENSG0000019
protein_co	11	0.09	A|frameshift_variant|HIGH|CHMP4A|ENSG00000254505|transcript
protein_co	11	0.09	A|frameshift_variant|HIGH|CHMP4A|ENSG00000254505|transcript
protein_co	10	0.1	C|frameshift_variant|HIGH|MDP1|ENSG00000213920|transcript|E
protein_coding			A|structural_interaction_variant|HIGH|GMPR2|ENSG00000100938|
antisense			A|splice_donor_variant&intron_variant|HIGH|RP11-104E19.1|ENSG
lincRNA			T|splice_donor_variant&intron_variant|HIGH|RP11-89K22.1|ENSG0
lincRNA			CTTTTTTT|splice_acceptor_variant&intron_variant|HIGH|LINC01551
lincRNA			CTTTTTTT|splice_acceptor_variant&intron_variant|HIGH|LINC01551
lincRNA			CTTTTTTT|splice_acceptor_variant&intron_variant|HIGH|LINC01551
lincRNA			A|splice_donor_variant&intron_variant|HIGH|RP11-966I7.3|ENSG0
protein_co	12	0.25	GT|splice_acceptor_variant&intron_variant|HIGH|AP4S1|ENSG0000
nonsense_	3	0.33	T|splice_acceptor_variant&intron_variant|HIGH|RP11-176H8.1|ENS
protein_coding			A|stop_gained|HIGH|GPR33|ENSG00000214943|transcript|ENST00
nonsense_	12	0.08	C|frameshift_variant|HIGH|BAZ1A|ENSG00000198604|transcript|E
antisense			A|splice_donor_variant&intron_variant|HIGH|RP11-81F13.2|ENSG0

protein_coding			C|structural_interaction_variant|HIGH|SEC23A|ENSG00000100934|
protein_co	12	0.58	CT|splice_acceptor_variant&intron_variant|HIGH|GEMIN2|ENSG000
protein_co	12	0.58	AT|splice_acceptor_variant&intron_variant|HIGH|GEMIN2|ENSG000
)179454			T|bidirectional_gene_fusion|HIGH|FAM179B&KLHL28|ENSG000001
protein_co	4	0.25	T|splice_donor_variant&intron_variant|HIGH|FKBP3|ENSG0000010
protein_co	10	0.4	T|splice_acceptor_variant&splice_region_variant&intron_variant|HI
protein_co	10	0.4	T|splice_acceptor_variant&splice_region_variant&intron_variant|HI
protein_coding			AAT|frameshift_variant|HIGH|SOS2|ENSG00000100485|transcript|
protein_co	6	0.17	T|frameshift_variant|HIGH|SOS2|ENSG00000100485|transcript|EN
protein_coding			A|structural_interaction_variant|HIGH|ATL1|ENSG00000198513|int
protein_co	7	0.43	C|splice_acceptor_variant&intron_variant|HIGH|PYGL|ENSG000001
lincRNA			G|splice_donor_variant&intron_variant|HIGH|RP11-255G12.2|ENSG
processed_pseudogene			C|splice_acceptor_variant&splice_region_variant&intron_variant&n
processed_pseudogene			CAAA|splice_acceptor_variant&splice_region_variant&intron_varian
processed_pseudogene			CAAAA|splice_region_variant&non_coding_transcript_exon_variant
processed_pseudogene			CAAAAA|splice_region_variant&non_coding_transcript_exon_varian
processed_pseudogene			CAAAAAAA|splice_region_variant&non_coding_transcript_exon_var
antisense			C|splice_donor_variant&intron_variant|HIGH|RP11-368P15.3|ENSG
lincRNA			CTTT|splice_acceptor_variant&intron_variant|HIGH|AL163953.3|EN
lincRNA			CTTT|splice_acceptor_variant&intron_variant|HIGH|AL163953.3|EN
lincRNA			CTTT|splice_acceptor_variant&intron_variant|HIGH|AL163953.3|EN
protein_coding			G|stop_lost|HIGH|BMP4|ENSG00000125378|transcript|ENST0000C
protein_co	13	0.38	C|frameshift_variant|HIGH|SAMD4A|ENSG00000020577|transcript
protein_coding			T|structural_interaction_variant|HIGH|LGALS3|ENSG00000131981|
protein_co	2	0.5	C|splice_acceptor_variant&splice_region_variant&intron_variant|HI
protein_co	2	0.5	C|splice_acceptor_variant&splice_region_variant&intron_variant|HI
protein_co	2	0.5	C|splice_acceptor_variant&splice_region_variant&intron_variant|HI
lincRNA			A|splice_donor_variant&intron_variant|HIGH|RP11-1012E15.2|ENS
protein_co	11	0.36	A|stop_gained|HIGH|C14orf105|ENSG00000100557|transcript|ENS
protein_co	11	0.09	C|splice_acceptor_variant&intron_variant|HIGH|C14orf105|ENSG00
protein_co	11	0.27	A|stop_gained|HIGH|C14orf105|ENSG00000100557|transcript|ENS
nonsense_mediated_decay			CAG|frameshift_variant&splice_region_variant|HIGH|ACTR10|ENSG
protein_co	15	0.07	C|splice_donor_variant&intron_variant|HIGH|KIAA0586|ENSG00000
protein_co	9	0.11	A|splice_donor_variant&intron_variant|HIGH|L3HYPDH|ENSG00000
nonsense_	6	0.33	A|stop_gained|HIGH|LRRC9|ENSG00000131951|transcript|ENST000
protein_co	5	0.2	G|start_lost|HIGH|SIX1|ENSG00000126778|transcript|ENST000005
protein_co	3	1	C|frameshift_variant|HIGH|TRMT5|ENSG00000126814|transcript|E
lincRNA			A|splice_donor_variant&intron_variant|HIGH|FLJ22447|ENSG00000
lincRNA			C|splice_donor_variant&intron_variant|HIGH|RP11-696D21.2|ENSG
protein_coding			A|stop_gained|HIGH|WDR89|ENSG00000140006|transcript|ENST00
protein_co	29	0.14	AT|splice_acceptor_variant&intron_variant|HIGH|SYNE2|ENSG0000
protein_co	29	0.21	A|stop_gained|HIGH|SYNE2|ENSG00000054654|transcript|ENST00
unitary_pseudogene			T|splice_donor_variant&splice_region_variant&intron_variant|HIGH
protein_coding			T|structural_interaction_variant|HIGH|HSPA2|ENSG00000126803|i
lincRNA			T|splice_donor_variant&intron_variant|HIGH|CTD-2509G16.2|ENSG
protein_co	16	0.31	C|frameshift_variant|HIGH|GPHN|ENSG00000171723|transcript|EN
nonsense_	11	0.27	CT|frameshift_variant|HIGH|FAM71D|ENSG00000172717|transcrip

processed_transcript			CTT|splice_acceptor_variant&intron_variant|HIGH|MPP5|ENSG000(
processed_transcript			CTT|splice_acceptor_variant&intron_variant|HIGH|MPP5|ENSG000(
protein_coding			T|stop_gained|HIGH|PLEKHH1|ENSG00000054690|transcript|ENST
processed_transcript			A|splice_acceptor_variant&intron_variant|HIGH|RAD51B|ENSG000(
unprocessed_pseudogene			C|splice_acceptor_variant&splice_region_variant&intron_variant|HI
unprocessed_pseudogene			CT|splice_region_variant&intron_variant|LOW|BLZF2P|ENSG00000
unprocessed_pseudogene			CTT|splice_region_variant&intron_variant|LOW|BLZF2P|ENSG0000(
unprocessed_pseudogene			CTTT|splice_region_variant&intron_variant|LOW|BLZF2P|ENSG0000(
nonsense_	10	0.1	A|splice_donor_variant&intron_variant|HIGH|GALNT16|ENSG00000(
protein_co	11	0.18	A|splice_acceptor_variant&intron_variant|HIGH|SLC39A9|ENSG000
nonsense_	5	0.2	G|splice_acceptor_variant&intron_variant|HIGH|SYNJ2BP-COX16|EI
processed_transcript			A|splice_donor_variant&intron_variant|HIGH|DPF3|ENSG00000205
protein_co	12	0.08	CTT|splice_acceptor_variant&intron_variant|HIGH|RBM25|ENSG00(
protein_co	12	0.08	CTT|splice_acceptor_variant&intron_variant|HIGH|RBM25|ENSG00(
protein_co	12	0.17	A|frameshift_variant|HIGH|RBM25|ENSG00000119707|transcript|I
antisense			A|splice_acceptor_variant&intron_variant|HIGH|RP4-647C14.2|ENS
nonsense_mediated_decay			G|frameshift_variant&splice_acceptor_variant&splice_region_variar
nonsense_mediated_decay			G|frameshift_variant&splice_acceptor_variant&splice_region_variar
nonsense_	11	0.64	C|splice_acceptor_variant&intron_variant|HIGH|PAPLN|ENSG00000(
antisense			ATTTTTTTTTTTTTTTTT|splice_acceptor_variant&intron_variant|HIGH
antisense			ATTTTTTTTTTTTTTTTT|splice_acceptor_variant&intron_variant|HIGH
antisense			ATTTTTTTTTTTTTTTTT|splice_acceptor_variant&intron_variant|HIGH
antisense			ATTTTTTTTTTTTTTTTT|splice_acceptor_variant&intron_variant|HIGH
antisense			ATTTTTTTTTTTTTTTTT|splice_acceptor_variant&intron_variant|HIGH
protein_coding			G|structural_interaction_variant|HIGH|ACOT2|ENSG00000119673|
protein_coding			T|structural_interaction_variant|HIGH|ACOT2|ENSG00000119673|i
protein_coding			A|structural_interaction_variant|HIGH|ACOT4|ENSG00000177465|i
protein_co	1	1	TTCAA|frameshift_variant|HIGH|ACOT4|ENSG00000177465|transcr
protein_co	1	1	G|frameshift_variant|HIGH|ACOT4|ENSG00000177465|transcript|E
protein_coding			T|structural_interaction_variant|HIGH|PTGR2|ENSG00000140043|i
protein_co	10	0.4	T|splice_donor_variant&intron_variant|HIGH|NPC2|ENSG00000119
protein_co	14	0.07	GT|frameshift_variant|HIGH|GPATCH2L|ENSG00000089916|transcr
processed_transcript			C|splice_donor_variant&intron_variant|HIGH|LRRC74A|ENSG00000
antisense	1	1	G|upstream_gene_variant|MODIFIER|RP11-7F17.8|ENSG0000027З
protein_co	1	1	TTGCTGC|frameshift_variant|HIGH|IRF2BPL|ENSG00000119669|tra
protein_co	1	1	C|frameshift_variant|HIGH|IRF2BPL|ENSG00000119669|transcript|
protein_co	8	0.25	T|splice_donor_variant&splice_region_variant&intron_variant|HIGH
protein_co	1	1	T|frameshift_variant|HIGH|AF111168.1|ENSG00000280308|transcr
protein_coding			G|frameshift_variant|HIGH|CEP128|ENSG00000100629|transcript|
nonsense_	4	0.25	C|splice_acceptor_variant&intron_variant|HIGH|GTF2A1|ENSG0000
nonsense_mediated_decay			T|stop_gained|HIGH|GALC|ENSG00000054983|transcript|ENST000(
nonsense_	22	0.14	T|frameshift_variant|HIGH|SPATA7|ENSG00000042317|transcript|I
nonsense_	22	0.05	A|splice_donor_variant&intron_variant|HIGH|SPATA7|ENSG000000
protein_coding			C|frameshift_variant&splice_donor_variant&splice_region_variant&
protein_co	35	0.06	C|splice_donor_variant&intron_variant|HIGH|C14orf159|ENSG0000
protein_co	35	0.14	T|splice_donor_variant&intron_variant|HIGH|C14orf159|ENSG0000
nonsense_mediated_decay			G|frameshift_variant|HIGH|C14orf159|ENSG00000133943|transcrip

protein_coding			T|stop_gained|HIGH|ATXN3|ENSG00000066427|transcript|ENST00
protein_co	53	0.26,(AL04!	CG|frameshift_variant|HIGH|ATXN3|ENSG00000066427|transcript|
protein_co	53	0.26,(AL04!	CTGCTGCTGCTGCTGCTG|frameshift_variant|HIGH|ATXN3|ENSG000
protein_co	53	0.26,(AL04!	CTGCTGCTGCTGCTGCTG|frameshift_variant|HIGH|ATXN3|ENSG000
protein_co	53	0.26,(AL04!	CTGCTGCTGCTGCTGCTG|frameshift_variant|HIGH|ATXN3|ENSG000
protein_co	53	0.26,(AL04!	CTGCTGCTGCTGCTGCTG|frameshift_variant|HIGH|ATXN3|ENSG000
protein_co	53	0.26,(AL04!	CTGCTGCTGCTGCTGCTG|frameshift_variant|HIGH|ATXN3|ENSG000
protein_co	53	0.26,(AL04!	CTGCTGCTGCTGCTGCTG|frameshift_variant|HIGH|ATXN3|ENSG000
protein_co	53	0.28,(AL04!	GC|frameshift_variant|HIGH|ATXN3|ENSG00000066427|transcript|
protein_co	53	0.28	TGCTGCTGCTGCTGCTGCTGCTGC|frameshift_variant|HIGH|ATXN3|E
protein_co	1	1	TGCTGCTGCTGCTGCTGCTGCTGC|frameshift_variant|HIGH|ATXN3|E
protein_co	7	0.14	G|splice_donor_variant&intron_variant|HIGH|NDUFB1|ENSG00000
protein_coding			G|structural_interaction_variant|HIGH|LGMN|ENSG00000100600|i
protein_co	8	0.63	G|splice_donor_variant&intron_variant|HIGH|SERPINA9|ENSG0000
protein_co	9	0.11	C|splice_acceptor_variant&intron_variant|HIGH|CLMN|ENSG00000
protein_coding			A|structural_interaction_variant|HIGH|GLRX5|ENSG00000182512|i
protein_co	25	0.2	ATT|splice_acceptor_variant&intron_variant|HIGH|PAPOLA|ENSG0(
protein_co	25	0.2	ATT|splice_acceptor_variant&intron_variant|HIGH|PAPOLA|ENSG0(
protein_co	7	0.14	TTG|frameshift_variant|HIGH|VRK1|ENSG00000100749|transcript|
protein_co	3	0.67	T|start_lost&disruptive_inframe_deletion|HIGH|C14orf177|ENSG0(
protein_coding			A|structural_interaction_variant|HIGH|WARS|ENSG00000140105|ir
protein_co	44	0.05	A|splice_acceptor_variant&intron_variant|HIGH|WARS|ENSG00000
protein_co	12	0.08	G|splice_donor_variant&intron_variant|HIGH|BEGAIN|ENSG000001
lincRNA			C|splice_donor_variant&intron_variant|HIGH|MEG3|ENSG0000021
lincRNA			C|splice_acceptor_variant&intron_variant|HIGH|MEG9|ENSG00000
lincRNA			A|splice_donor_variant&intron_variant|HIGH|AL132709.1|ENSG000
protein_coding			G|structural_interaction_variant|HIGH|HSP90AA1|ENSG000000808
protein_coding			A|structural_interaction_variant|HIGH|HSP90AA1|ENSG000000808
protein_co	41	0.05	T|frameshift_variant|HIGH|MOK|ENSG00000080823|transcript|EN
protein_co	41	0.15	G|splice_donor_variant&intron_variant|HIGH|MOK|ENSG00000080
protein_co	41	0.12	A|stop_gained|HIGH|MOK|ENSG00000080823|transcript|ENST000(
protein_co	7	0.14	C|frameshift_variant|HIGH|EXOC3L4|ENSG00000205436|transcript
processed_pseudogene			C|splice_acceptor_variant&intron_variant|HIGH|RPL21P12|ENSG00
processed_pseudogene			C|splice_acceptor_variant&splice_region_variant&intron_variant|HI
processed_pseudogene			C|splice_acceptor_variant&splice_region_variant&intron_variant|HI
protein_co	26	0.35	GT|splice_acceptor_variant&intron_variant|HIGH|MARK3|ENSG000
protein_coding			T|structural_interaction_variant|HIGH|CKB|ENSG00000166165|inte
protein_coding			T|structural_interaction_variant|HIGH|CKB|ENSG00000166165|inte
lincRNA			G|splice_donor_variant&intron_variant|HIGH|RP11-260M19.2|ENS(
protein_coding			A|structural_interaction_variant|HIGH|AKT1|ENSG00000142208|in
protein_coding			A|structural_interaction_variant|HIGH|AKT1|ENSG00000142208|in
protein_co	4	0.25	T|frameshift_variant|HIGH|AHNAK2|ENSG00000185567|transcript|
protein_co	5	0.4	C|splice_donor_variant&intron_variant|HIGH|JAG2|ENSG00000184
protein_coding			T|structural_interaction_variant|HIGH|BTBD6|ENSG00000184887|i
protein_coding			A|stop_gained|HIGH|GOLGA6L6|ENSG00000277322|transcript|ENS
protein_co	2	0.5	T|stop_gained|HIGH|GOLGA8S|ENSG00000261739|transcript|ENST
protein_co	4	0.25	TG|frameshift_variant|HIGH|GOLGA6L2|ENSG00000174450|transc

protein_co	4	0.25	C|frameshift_variant|HIGH|GOLGA6L2|ENSG00000174450|transcri
protein_co	4	0.25	T|frameshift_variant|HIGH|GOLGA6L2|ENSG00000174450|transcri
protein_co	4	0.25	T|frameshift_variant|HIGH|GOLGA6L2|ENSG00000174450|transcri
lincRNA			A|splice_donor_variant&intron_variant|HIGH|PWRN1|ENSG000002
protein_co	8	0.13	G|frameshift_variant|HIGH|GABRG3|ENSG00000182256|transcript
protein_co	2	0.5	AT|frameshift_variant|HIGH|GOLGA6L7P|ENSG00000261649|trans
unprocessed_pseudogene			G|splice_donor_variant&intron_variant|HIGH|RP11-1H8.6|ENSG000
unprocessed_pseudogene			T|splice_donor_variant&intron_variant|HIGH|RP11-1H8.6|ENSG000
protein_coding			A|structural_interaction_variant|HIGH|AQR|ENSG00000021776|int
protein_coding			T|frameshift_variant&stop_lost&splice_region_variant|HIGH|NANO
lincRNA			G|splice_donor_variant&intron_variant|HIGH|RP11-624L4.1|ENSG0
protein_coding			T|structural_interaction_variant|HIGH|THBS1|ENSG00000137801|i
protein_coding			C|structural_interaction_variant|HIGH|PLCB2|ENSG00000137841|i
protein_co	13	0.08	G|frameshift_variant|HIGH|IVD|ENSG00000128928|transcript|ENS
protein_co	15	0.2	T|splice_donor_variant&intron_variant|HIGH|C15orf57|ENSG00000
protein_co	16	0.19	TGGGGC|frameshift_variant|HIGH|ZFYVE19|ENSG00000166140|tra
antisense			A|splice_donor_variant&intron_variant|HIGH|RP11-540O11.6|ENS
antisense			CA|splice_acceptor_variant&intron_variant|HIGH|OIP5-AS1|ENSG0
processed_transcript			CTT|splice_acceptor_variant&intron_variant|HIGH|NUSAP1|ENSG0
processed_transcript			CTT|splice_acceptor_variant&intron_variant|HIGH|NUSAP1|ENSG0
protein_co	6	0.17	T|splice_donor_variant&intron_variant|HIGH|LTK|ENSG00000062
protein_co	6	0.5	C|frameshift_variant|HIGH|LTK|ENSG00000062524|transcript|ENS
protein_co	9	0.22	GCCTGAA|splice_donor_variant&intron_variant|HIGH|TYRO3|ENSG
protein_co	9	0.22	G|frameshift_variant&splice_acceptor_variant&splice_donor_varian
protein_co	9	0.22	TTGGACAGCTTGGGCATCAGCGATGAACTAAAGTGAG|splice_donor_va
protein_co	9	0.22	TTGGACAGCTTGGGCATCAGCGATGAACTAAAGTGAG|splice_donor_va
protein_co	9	0.33	GGAGA|splice_donor_variant&intron_variant|HIGH|TYRO3|ENSG00
protein_co	9	0.44	TGG|splice_donor_variant&intron_variant|HIGH|TYRO3|ENSG0000
protein_co	9	0.44	TAACCTACCCCTCCAGACCCTGATCCG|splice_donor_variant&intron_v
protein_co	9	0.33	TGTGGGCGTTCGG|splice_donor_variant&intron_variant|HIGH|TYR
protein_co	9	0.33	GTGGGCGTTCGGGGTGACCAT|splice_donor_variant&intron_variant|
protein_co	2	0.5	T|stop_gained&splice_region_variant|HIGH|SPTBN5|ENSG0000013
protein_co	2	0.5	A|stop_gained|HIGH|SPTBN5|ENSG00000137877|transcript|ENST0
protein_co	8	0.13	TTGACTGGCTAGCTGCAGGGCGGCCTCCAGCTCAGACA|frameshift_var
protein_co	8	0.25	A|splice_donor_variant&intron_variant|HIGH|ADAL|ENSG0000016
nonsense_mediated_decay			T|stop_gained|HIGH|ZSCAN29|ENSG00000140265|transcript|ENST
protein_co	8	0.13	C|splice_acceptor_variant&splice_donor_variant&intron_variant|HI
nonsense_	12	0.17	A|splice_acceptor_variant&intron_variant|HIGH|CATSPER2|ENSG0
processed_transcript			A|splice_acceptor_variant&intron_variant|HIGH|CATSPER2P1|ENSG
protein_co	1	1	C|frameshift_variant|HIGH|AC018512.1|ENSG00000279808|transc
protein_co	1	1	C|frameshift_variant|HIGH|AC018512.1|ENSG00000279808|transc
protein_co	1	1	C|frameshift_variant|HIGH|AC018512.1|ENSG00000279808|transc
protein_co	9	0.11	C|start_lost|HIGH|PATL2|ENSG00000229474|transcript|ENST0000
unprocessed_pseudogene			A|splice_donor_variant&intron_variant|HIGH|CTD-2014N11.1|ENS
protein_co	5	0.4	T|frameshift_variant|HIGH|DUOX2|ENSG00000140279|transcript|
)259519			C|bidirectional_gene_fusion|HIGH|RP11-519G16.2&SHF|ENSG0000
)259519			CA|bidirectional_gene_fusion|HIGH|RP11-519G16.2&SHF|ENSG000

)259519			CAA|bidirectional_gene_fusion|HIGH|RP11-519G16.2&SHF|ENSG00
)259519			CAAA|bidirectional_gene_fusion|HIGH|RP11-519G16.2&SHF|ENSG
)259519			CAAAA|bidirectional_gene_fusion|HIGH|RP11-519G16.2&SHF|ENSG
antisense			T|splice_acceptor_variant&splice_region_variant&intron_variant&n
protein_coding			T|structural_interaction_variant|HIGH|GALK2|ENSG00000156958|i
protein_coding			A|structural_interaction_variant|HIGH|USP8|ENSG00000138592|in
lincRNA			A|splice_donor_variant&intron_variant|HIGH|CTD-2378E12.1|ENSG
antisense			A|splice_acceptor_variant&intron_variant|HIGH|RP11-23N2.4|ENS
protein_co	5	0.2	T|stop_gained|HIGH|UNC13C|ENSG00000137766|transcript|ENST
retained_ir	5	0.2	TTGTC|splice_acceptor_variant&intron_variant|HIGH|UNC13C|ENS
retained_intron			A|splice_acceptor_variant&intron_variant|HIGH|UNC13C|ENSG000
protein_coding			A|stop_gained|HIGH|DYX1C1|ENSG00000256061|transcript|ENST0
protein_coding			T|structural_interaction_variant|HIGH|NEDD4|ENSG00000069869|
)261174			G|gene_fusion|HIGH|RP11-844G16.1&TEX9|ENSG00000261174&E
)261174			A|gene_fusion|HIGH|RP11-844G16.1&TEX9|ENSG00000261174&E
protein_co	25	0.04	T|start_lost|HIGH|ALDH1A2|ENSG00000128918|transcript|ENST00
protein_co	26	0.08	G|frameshift_variant|HIGH|SLTM|ENSG00000137776|transcript|E
sense_overlapping			G|splice_acceptor_variant&intron_variant|HIGH|AC092755.4|ENSG
protein_co	38	0.03	T|stop_gained|HIGH|ANXA2|ENSG00000182718|transcript|ENST00
processed_	17	0.06	A|splice_acceptor_variant&intron_variant|HIGH|ICE2|ENSG000001
processed_transcript			A|splice_donor_variant&intron_variant|HIGH|RP11-299H22.3|ENSG
transcribed_unprocessed_pseudo			CT|splice_acceptor_variant&intron_variant|HIGH|RP11-299H22.3|E
antisense			C|splice_acceptor_variant&intron_variant|HIGH|RP11-1069G10.1|E
protein_coding			C|structural_interaction_variant|HIGH|FAM96A|ENSG00000166797
protein_co	18	0.28	T|splice_donor_variant&intron_variant|HIGH|CSNK1G1|ENSG00000
processed_transcript			GT|splice_acceptor_variant&intron_variant|HIGH|TRIP4|ENSG0000
processed_transcript			TTA|splice_acceptor_variant&intron_variant|HIGH|TRIP4|ENSG000
processed_transcript			TTA|splice_acceptor_variant&intron_variant|HIGH|TRIP4|ENSG000
protein_co	9	0.33	A|stop_gained|HIGH|PIF1|ENSG00000140451|transcript|ENST0000
protein_co	6	0.17	C|splice_donor_variant&intron_variant|HIGH|IGDCC4|ENSG000001
processed_pseudogene			C|splice_acceptor_variant&splice_region_variant&intron_variant|HI
processed_pseudogene			C|splice_acceptor_variant&splice_region_variant&intron_variant|HI
processed_pseudogene			C|splice_acceptor_variant&splice_region_variant&intron_variant|HI
lincRNA			T|splice_donor_variant&intron_variant|HIGH|IQCH-AS1|ENSG00000
processed_pseudogene			A|splice_donor_variant&intron_variant|HIGH|CARSP1|ENSG000002
processed_transcript			AT|splice_acceptor_variant&intron_variant|HIGH|GLCE|ENSG00000
protein_co	4	0.25	G|start_lost|HIGH|GLCE|ENSG00000138604|transcript|ENST00000
lincRNA			G|splice_acceptor_variant&intron_variant|HIGH|RP11-352D13.5|E
protein_co	26	0.04	C|splice_donor_variant&intron_variant|HIGH|TLE3|ENSG00000140
antisense			C|splice_donor_variant&intron_variant|HIGH|RP11-138H8.3|ENSG
protein_co	29	0.03	CTTTTT|splice_acceptor_variant&intron_variant|HIGH|LRRC49|ENS
protein_co	29	0.03	CTTTTT|splice_acceptor_variant&intron_variant|HIGH|LRRC49|ENS
protein_co	29	0.03	CTTTTT|splice_acceptor_variant&intron_variant|HIGH|LRRC49|ENS
nonsense_mediated_decay			C|stop_lost|HIGH|RP11-106M3.2|ENSG00000260729|transcript|E
protein_co	11	0.36	ATT|splice_acceptor_variant&intron_variant|HIGH|NEO1|ENSG000
protein_co	11	0.36	ATT|splice_acceptor_variant&intron_variant|HIGH|NEO1|ENSG000
protein_coding			T|structural_interaction_variant|HIGH|NEO1|ENSG00000067141|in

protein_co	19	0.05	G|splice_donor_variant&intron_variant|HIGH|CD276|ENSG0000010
antisense			C|splice_donor_variant&intron_variant|HIGH|LOXL1-AS1|ENSG0000
protein_coding			T|structural_interaction_variant|HIGH|CSK|ENSG00000103653|inte
protein_co	7	0.57	C|frameshift_variant|HIGH|C15orf39|ENSG00000167173|transcript
processed_pseudogene			G|splice_acceptor_variant&intron_variant|HIGH|CTD-2562G15.2|EN
processed_pseudogene			G|splice_acceptor_variant&intron_variant|HIGH|CTD-2562G15.2|EN
protein_co	1	1	A|frameshift_variant|HIGH|CSPG4|ENSG00000173546|transcript|E
transcribed_unprocessed_pseudo			G|splice_donor_variant&splice_region_variant&intron_variant|HIGH
nonsense_	16	0.13	T|splice_donor_variant&intron_variant|HIGH|NRG4|ENSG0000016
nonsense_	5	0.2	CTT|splice_acceptor_variant&intron_variant|HIGH|TMEM266|ENSG
nonsense_	5	0.2	CTT|splice_acceptor_variant&intron_variant|HIGH|TMEM266|ENSG
nonsense_	5	0.2	CTT|splice_acceptor_variant&intron_variant|HIGH|TMEM266|ENSG
nonsense_	5	0.2	CTT|splice_acceptor_variant&intron_variant|HIGH|TMEM266|ENSG
protein_co	23	0.3	C|start_lost|HIGH|SCAPER|ENSG00000140386|transcript|ENST0000
protein_co	23	0.09	A|splice_donor_variant&intron_variant|HIGH|SCAPER|ENSG000001
nonsense_	6	0.17	T|splice_donor_variant&intron_variant|HIGH|RCN2|ENSG00000117
transcribed_unprocessed_pseudo			T|splice_acceptor_variant&intron_variant|HIGH|RP11-114H24.2|EN
protein_co	2	0.5	C|start_lost|HIGH|SH2D7|ENSG00000183476|transcript|ENST0000
protein_co	12	0.08	C|frameshift_variant&start_lost|HIGH|DNAJA4|ENSG00000140403|
protein_coding			C|stop_lost|HIGH|PSMA4|ENSG00000041357|transcript|ENST0000
protein_co	5	0.2	TA|frameshift_variant|HIGH|ADAMTS7|ENSG00000136378|transcri
protein_coding			A|structural_interaction_variant|HIGH|MORF4L1|ENSG0000018578
protein_co	5	0.8	A|splice_donor_variant&intron_variant|HIGH|TMED3|ENSG000001
nonsense_	6	0.17	CT|frameshift_variant|HIGH|STARD5|ENSG00000172345|transcript
protein_coding			T|stop_gained|HIGH|TMC3|ENSG00000188869|transcript|ENST000
antisense			A|splice_acceptor_variant&intron_variant|HIGH|TMC3-AS1|ENSG00
protein_co	2	0.5	C|frameshift_variant|HIGH|MEX3B|ENSG00000183496|transcript|E
protein_co	2	0.5	C|frameshift_variant|HIGH|MEX3B|ENSG00000183496|transcript|E
protein_co	8	0.13	A|frameshift_variant|HIGH|SAXO2|ENSG00000188659|transcript|E
antisense			A|splice_acceptor_variant&intron_variant|HIGH|RP11-245C17.2|EN
antisense			G|splice_donor_variant&intron_variant|HIGH|LINC01584|ENSG0000
antisense			T|splice_acceptor_variant&intron_variant|HIGH|LINC01584|ENSG00
lincRNA			A|splice_donor_variant&intron_variant|HIGH|NTRK3-AS1|ENSG000
lincRNA			G|splice_donor_variant&intron_variant|HIGH|LINC01586|ENSG0000
protein_coding			C|structural_interaction_variant|HIGH|ISG20|ENSG00000172183|ir
protein_co	5	0.2	A|stop_gained|HIGH|HAPLN3|ENSG00000140511|transcript|ENST0
antisense			T|splice_donor_variant&intron_variant|HIGH|RP11-217B1.2|ENSG0
protein_co	2	0.5	CG|frameshift_variant|HIGH|MESP1|ENSG00000166823|transcript|
protein_co	2	0.5	CACGGGGCTCGG|frameshift_variant|HIGH|MESP1|ENSG0000016668
protein_co	2	0.5	CACGGGGCTCGG|frameshift_variant|HIGH|MESP1|ENSG0000016668
processed_pseudogene			C|splice_acceptor_variant&splice_region_variant&intron_variant|HI
processed_pseudogene			C|splice_acceptor_variant&splice_region_variant&intron_variant|HI
processed_pseudogene			C|splice_acceptor_variant&splice_region_variant&intron_variant|HI
processed_pseudogene			C|splice_acceptor_variant&splice_region_variant&intron_variant|HI
processed_pseudogene			C|splice_acceptor_variant&splice_region_variant&intron_variant|HI
protein_coding			A|structural_interaction_variant|HIGH|ANPEP|ENSG00000166825|i
protein_coding			T|structural_interaction_variant|HIGH|ANPEP|ENSG00000166825|i

protein_coding			A|structural_interaction_variant|HIGH|ANPEP|ENSG00000166825|i
protein_coding			T|structural_interaction_variant|HIGH|ANPEP|ENSG00000166825|i
protein_coding			C|structural_interaction_variant|HIGH|IDH2|ENSG00000182054|int
protein_coding			A|stop_gained|HIGH|SEMA4B|ENSG00000185033|transcript|ENST(
protein_coding			T|structural_interaction_variant|HIGH|FURIN|ENSG00000140564|ir
nonsense_	17	0.06	C|frameshift_variant|HIGH|FES|ENSG00000182511|transcript|ENST
protein_co	19	0.05	G|frameshift_variant|HIGH|PRC1|ENSG00000198901|transcript|EN
lincRNA			A|splice_donor_variant&intron_variant|HIGH|LINC01579|ENSG0000(
lincRNA			A|splice_acceptor_variant&intron_variant|HIGH|LINC01197|ENSG0(
antisense			A|splice_acceptor_variant&splice_region_variant&intron_variant&n
antisense			G|splice_donor_variant&intron_variant|HIGH|NR2F2-AS1|ENSG000
protein_co	4	0.25	T|stop_gained|HIGH|SPATA8|ENSG00000185594|transcript|ENST0(
lincRNA			A|splice_acceptor_variant&intron_variant|HIGH|LINC00923|ENSG0(
processed_transcript			T|splice_donor_variant&intron_variant|HIGH|IGF1R|ENSG0000014(
protein_co	3	1	AC|frameshift_variant|HIGH|PGPEP1L|ENSG00000183571|transcrip
)259475			C|bidirectional_gene_fusion|HIGH|RP11-654A16.3&RP11-6O2.2|EN
)259475			C|bidirectional_gene_fusion|HIGH|RP11-654A16.3&RP11-6O2.2|EN
)259475			C|bidirectional_gene_fusion|HIGH|RP11-654A16.3&RP11-6O2.2|EN
)259475			C|bidirectional_gene_fusion|HIGH|RP11-654A16.3&RP11-6O2.2|EN
)259475			C|bidirectional_gene_fusion|HIGH|RP11-654A16.3&RP11-6O2.2|EN
)259475			C|bidirectional_gene_fusion|HIGH|RP11-654A16.3&RP11-6O2.2|EN
protein_co	19	0.05	A|stop_gained|HIGH|TTC23|ENSG00000103852|transcript|ENST00(
retained_intron			T|splice_donor_variant&splice_region_variant&intron_variant|HIGH
retained_intron			T|splice_donor_variant&splice_region_variant&intron_variant|HIGH
protein_co	11	0.09	ACG|frameshift_variant|HIGH|LYSMD4|ENSG00000183060|transcri
processed_transcript			T|splice_donor_variant&intron_variant|HIGH|LINS1|ENSG0000014C
processed_transcript			C|splice_donor_variant&intron_variant|HIGH|RP11-66B24.7|ENSG0
lincRNA			A|splice_donor_variant&intron_variant|HIGH|RP11-66B24.2|ENSG0
antisense			G|splice_acceptor_variant&intron_variant|HIGH|RP11-505E24.3|EN
nonsense_	11	0.36	CACTT|splice_acceptor_variant&intron_variant|HIGH|LRRK1|ENSG0
protein_coding			T|stop_gained|HIGH|PCSK6|ENSG00000140479|transcript|ENST00(
protein_co	5	0.8	T|splice_acceptor_variant&intron_variant|HIGH|MPG|ENSG000001
protein_coding			A|splice_acceptor_variant&5_prime_UTR_variant&intron_variant|H
protein_co	16	0.13	A|splice_acceptor_variant&intron_variant|HIGH|FAM234A|ENSG00
protein_co	16	0.06	CACGG|frameshift_variant|HIGH|FAM234A|ENSG00000167930|tra
protein_co	8	0.38	A|stop_gained|HIGH|RGS11|ENSG00000076344|transcript|ENST00
protein_co	7	0.43	CA|frameshift_variant|HIGH|PDIA2|ENSG00000185615|transcript|I
protein_co	10	0.1	CCCGACCTGCTCTGCTCGCCTCCCCGACCTGCTCTGCTCGCCTCT|framesh
protein_coding			AG|frameshift_variant|HIGH|TMEM8A|ENSG00000129925|transcri
protein_co	12	0.08	CAG|frameshift_variant|HIGH|NME4|ENSG00000103202|transcript
protein_co	8	0.13	C|splice_donor_variant&intron_variant|HIGH|CAPN15|ENSG000001
nonsense_mediated_decay			GATGT|frameshift_variant|HIGH|PIGQ|ENSG00000007541|transcrip
nonsense_	12	0.08	A|frameshift_variant|HIGH|RAB40C|ENSG00000197562|transcript|
protein_co	11	0.09	G|frameshift_variant|HIGH|STUB1|ENSG00000103266|transcript|E
protein_co	6	0.17	TG|frameshift_variant|HIGH|METRN|ENSG00000103260|transcript
protein_co	6	0.17	TG|frameshift_variant|HIGH|METRN|ENSG00000103260|transcript
protein_coding			TG|frameshift_variant|HIGH|METRN|ENSG00000103260|transcript

protein_co	20	0.05	G|frameshift_variant|HIGH|CHTF18|ENSG00000127586|transcript|
protein_co	20	0.05	G|frameshift_variant|HIGH|CHTF18|ENSG00000127586|transcript|
protein_co	20	0.05	G|frameshift_variant|HIGH|CHTF18|ENSG00000127586|transcript|
protein_co	20	0.05	G|frameshift_variant|HIGH|CHTF18|ENSG00000127586|transcript|
protein_co	20	0.05	C|frameshift_variant|HIGH|CHTF18|ENSG00000127586|transcript|
protein_co	1	1	A|frameshift_variant|HIGH|PRR25|ENSG00000167945|transcript|E
nonsense_	17	0.12	T|splice_donor_variant&intron_variant|HIGH|LMF1|ENSG0000010
protein_co	17	0.06	T|splice_donor_variant&intron_variant|HIGH|LMF1|ENSG0000010
lincRNA			C|splice_acceptor_variant&splice_donor_variant&intron_variant|HI
protein_co	2	1	A|splice_donor_variant&intron_variant|HIGH|C1QTNF8|ENSG0000
protein_co	2	1	A|frameshift_variant|HIGH|TPSD1|ENSG00000095917|transcript|E
protein_coding			G|structural_interaction_variant|HIGH|UBE2I|ENSG00000103275|i
protein_co	17	0.12	T|frameshift_variant&splice_acceptor_variant&splice_region_varian
retained_ir	4	0.75	T|splice_acceptor_variant&intron_variant|HIGH|CCDC154|ENSG000
protein_coding			A|structural_interaction_variant|HIGH|NME3|ENSG00000103024|i
protein_co	3	0.67	CACCT|splice_donor_variant&intron_variant|HIGH|MRPS34|ENSG0
protein_co	6	0.17	C|frameshift_variant|HIGH|EME2|ENSG00000197774|transcript|EN
nonsense_	16	0.06	A|splice_donor_variant&intron_variant|HIGH|NUBP2|ENSG0000009
protein_co	5	0.2	C|frameshift_variant|HIGH|NDUFB10|ENSG00000140990|transcript
protein_co	7	0.57	A|stop_gained|HIGH|NOXO1|ENSG00000196408|transcript|ENST0
protein_coding			C|stop_lost|HIGH|SLC9A3R2|ENSG00000065054|transcript|ENST0
nonsense_	9	0.11	G|splice_acceptor_variant&intron_variant|HIGH|E4F1|ENSG000001
protein_co	31	0.03	CT|frameshift_variant|HIGH|SRRM2|ENSG00000167978|transcript|
nonsense_	8	0.13	G|frameshift_variant|HIGH|PRSS22|ENSG00000005001|transcript|
protein_co	1	1	C|splice_donor_variant&intron_variant|HIGH|LA16c-380H5.3|ENSG
protein_co	7	0.14	T|frameshift_variant|HIGH|TNFRSF12A|ENSG00000006327|transcri
protein_coding			CGG|frameshift_variant|HIGH|IL32|ENSG00000008517|transcript|E
protein_coding			C|structural_interaction_variant|HIGH|MEFV|ENSG00000103313|ir
protein_coding			T|structural_interaction_variant|HIGH|MEFV|ENSG00000103313|ir
protein_coding			C|structural_interaction_variant|HIGH|MEFV|ENSG00000103313|ir
protein_co	1	1	G|frameshift_variant|HIGH|OR2C1|ENSG00000168158|transcript|E
protein_coding			C|structural_interaction_variant|HIGH|TRAP1|ENSG00000126602|i
protein_coding			A|structural_interaction_variant|HIGH|TRAP1|ENSG00000126602|i
nonsense_mediated_decay			T|frameshift_variant|HIGH|SRL|ENSG00000185739|transcript|ENST
nonsense_mediated_decay			C|stop_lost|HIGH|ANKS3|ENSG00000168096|transcript|ENST00000
protein_co	8	0.13	C|splice_acceptor_variant&intron_variant|HIGH|ZNF500|ENSG0000
antisense			A|splice_donor_variant&intron_variant|HIGH|RP11-127I20.5|ENSG
nonsense_	8	0.13	GGTCT|frameshift_variant|HIGH|ALG1|ENSG00000033011|transcri
unprocessed_pseudogene			A|splice_donor_variant&intron_variant|HIGH|ENPP7P14|ENSG0000
sense_intronic			A|splice_donor_variant&intron_variant|HIGH|RP11-468I15.1|ENSG
processed_transcript			ATTT|splice_acceptor_variant&intron_variant|HIGH|RBFOX1|ENSG
processed_transcript			ATTT|splice_acceptor_variant&intron_variant|HIGH|RBFOX1|ENSG
processed_transcript			ATTT|splice_acceptor_variant&intron_variant|HIGH|RBFOX1|ENSG
protein_co	2	0.5	C|start_lost&splice_region_variant|HIGH|TMEM186|ENSG0000018
protein_coding			C|structural_interaction_variant|HIGH|PMM2|ENSG00000140650|i
antisense			C|splice_acceptor_variant&intron_variant|HIGH|RP11-77H9.2|ENSG
lincRNA			ATTTTTTTT|splice_acceptor_variant&intron_variant|HIGH|RP11-473

lincRNA			ATTTTTTTT|splice_acceptor_variant&intron_variant|HIGH|RP11-473
lincRNA			ATTTTTTTT|splice_acceptor_variant&intron_variant|HIGH|RP11-473
lincRNA			ATTTTTTTT|splice_acceptor_variant&intron_variant|HIGH|RP11-473
lincRNA			ATTTTTTTT|splice_acceptor_variant&intron_variant|HIGH|RP11-473
lincRNA			ATTTTTTTT|splice_acceptor_variant&intron_variant|HIGH|RP11-473
processed_transcript			G|splice_acceptor_variant&intron_variant|HIGH|TVP23A|ENSG0000
protein_co	1	1	CTT|frameshift_variant|HIGH|PRM3|ENSG00000178257|transcript|
protein_coding			A|structural_interaction_variant|HIGH|RMI2|ENSG00000175643|in
protein_coding			C|structural_interaction_variant|HIGH|RMI2|ENSG00000175643|in
protein_co	8	0.13	C|frameshift_variant|HIGH|CTD-3088G3.8|ENSG00000188897|tran
protein_co	8	0.13	C|frameshift_variant|HIGH|CTD-3088G3.8|ENSG00000188897|tran
antisense			TAGCC|splice_donor_variant&intron_variant|HIGH|RP11-166B2.5|E
antisense			A|splice_donor_variant&intron_variant|HIGH|RP11-166B2.5|ENSG0
antisense			C|splice_donor_variant&intron_variant|HIGH|RP11-166B2.5|ENSG0
protein_co	7	0.43	C|frameshift_variant|HIGH|RP11-166B2.1|ENSG00000234719|trans
protein_co	3	0.33	C|splice_acceptor_variant&splice_region_variant&intron_variant|HI
protein_co	3	0.33	C|splice_acceptor_variant&splice_region_variant&intron_variant|HI
protein_co	3	0.33	C|splice_acceptor_variant&splice_region_variant&intron_variant|HI
protein_co	3	0.33	C|splice_acceptor_variant&splice_region_variant&intron_variant|HI
protein_co	3	0.33	C|splice_acceptor_variant&splice_region_variant&intron_variant|HI
antisense			T|splice_acceptor_variant&intron_variant|HIGH|AC009134.1|ENSG
transcribed_unprocessed_pseudo			T|splice_donor_variant&splice_region_variant&intron_variant|HIGH
protein_coding			A|stop_gained|HIGH|TMC7|ENSG00000170537|transcript|ENST000
lincRNA			T|splice_donor_variant&intron_variant|HIGH|RP11-626G11.4|ENSG
protein_co	24	0.04	TC|frameshift_variant|HIGH|C16orf62|ENSG00000103544|transcrip
protein_co	24	0.04	TC|frameshift_variant|HIGH|C16orf62|ENSG00000103544|transcrip
protein_co	24	0.04	TC|frameshift_variant|HIGH|C16orf62|ENSG00000103544|transcrip
protein_co	14	0.21	C|splice_acceptor_variant&intron_variant|HIGH|IQCK|ENSG000001
antisense			T|splice_acceptor_variant&intron_variant|HIGH|CTD-2194A8.2|ENS
protein_co	16	0.38	T|stop_gained|HIGH|ACSM2A|ENSG00000183747|transcript|ENST0
protein_coding			G|structural_interaction_variant|HIGH|ACSM2A|ENSG00000183747
protein_coding			T|stop_gained|HIGH|ACSM2B|ENSG00000066813|transcript|ENST0
nonsense_	6	0.17	T|splice_donor_variant&intron_variant|HIGH|ACSM1|ENSG0000016
protein_co	15	0.2	G|stop_gained|HIGH|ACSM3|ENSG00000005187|transcript|ENST00
nonsense_	9	0.11	A|splice_donor_variant&intron_variant|HIGH|TMEM159|ENSG0000
protein_coding			C|structural_interaction_variant|HIGH|CRYM|ENSG00000103316|ir
lincRNA			C|splice_acceptor_variant&intron_variant|HIGH|CTB-31N19.5|ENSG
protein_co	11	0.55	CTTT|splice_acceptor_variant&intron_variant|HIGH|METTL9|ENSG0
protein_co	11	0.55	CTTT|splice_acceptor_variant&intron_variant|HIGH|METTL9|ENSG0
nonsense_	7	0.57	A|frameshift_variant|HIGH|C16orf52|ENSG00000185716|transcript
protein_co	17	0.12	AC|frameshift_variant|HIGH|GGA2|ENSG00000103365|transcript|E
protein_coding			T|stop_gained|HIGH|CHP2|ENSG00000166869|transcript|ENST000
processed_pseudogene			G|splice_acceptor_variant&splice_region_variant&intron_variant|HI
processed_pseudogene			G|splice_acceptor_variant&splice_region_variant&intron_variant|HI
processed_pseudogene			G|splice_acceptor_variant&splice_region_variant&intron_variant|HI
processed_pseudogene			G|splice_acceptor_variant&splice_region_variant&intron_variant&n
processed_pseudogene			G|splice_acceptor_variant&splice_region_variant&intron_variant&n

processed_pseudogene			G|splice_acceptor_variant&splice_region_variant&intron_variant&n
processed_pseudogene			G|splice_acceptor_variant&splice_region_variant&intron_variant&n
processed_pseudogene			G|splice_acceptor_variant&splice_region_variant&intron_variant&n
processed_pseudogene			G|splice_acceptor_variant&splice_region_variant&intron_variant&n
protein_coding			C|structural_interaction_variant|HIGH|KDM8|ENSG00000155666|ir
protein_co	2	1	GGACAAAGCAA|frameshift_variant|HIGH|NPIPB6|ENSG000001981
nonsense_	7	0.14	CA|frameshift_variant|HIGH|SGF29|ENSG00000176476|transcript|
protein_coding			G|structural_interaction_variant|HIGH|SGF29|ENSG00000176476|i
protein_coding			G|structural_interaction_variant|HIGH|SGF29|ENSG00000176476|i
protein_coding			T|structural_interaction_variant|HIGH|SULT1A2|ENSG0000019716
protein_coding			A|structural_interaction_variant|HIGH|SULT1A2|ENSG0000019716
protein_coding			T|structural_interaction_variant|HIGH|SULT1A1|ENSG00000196502
protein_co	26	0.04	A|splice_acceptor_variant&intron_variant|HIGH|ATXN2L|ENSG0000
protein_co	16	0.06	C|splice_donor_variant&intron_variant|HIGH|SH2B1|ENSG0000017
lincRNA			A|splice_donor_variant&intron_variant|HIGH|RP11-426C22.5|ENSG
)183336			A|bidirectional_gene_fusion|HIGH|BOLA2&RP11-345J4.6|ENSG000
protein_coding			T|structural_interaction_variant|HIGH|QPRT|ENSG00000103485|in
protein_coding			T|structural_interaction_variant|HIGH|DOC2A|ENSG00000149927|
retained_intron			CTT|splice_acceptor_variant&intron_variant|HIGH|PRR14|ENSG000
retained_intron			CTT|splice_acceptor_variant&intron_variant|HIGH|PRR14|ENSG000
protein_co	13	0.15	C|frameshift_variant|HIGH|PRR14|ENSG00000156858|transcript|E
protein_co	13	0.15	TCCCCCCCCCCCCCCC|frameshift_variant|HIGH|PRR14|ENSG00000
protein_co	13	0.15	TCCCCCCCCCCCCCCC|frameshift_variant|HIGH|PRR14|ENSG00000
protein_co	9	0.11	C|frameshift_variant|HIGH|FBRS|ENSG00000156860|transcript|EN
protein_co	9	0.11	C|frameshift_variant|HIGH|FBRS|ENSG00000156860|transcript|EN
protein_co	9	0.11	C|frameshift_variant|HIGH|FBRS|ENSG00000156860|transcript|EN
protein_co	9	0.11	C|frameshift_variant|HIGH|FBRS|ENSG00000156860|transcript|EN
protein_co	2	0.5	C|frameshift_variant|HIGH|SETD1A|ENSG00000099381|transcript|
protein_co	8	0.13	C|splice_donor_variant&intron_variant|HIGH|VKORC1|ENSG00000
protein_coding			T|structural_interaction_variant|HIGH|AHSP|ENSG00000169877|in
antisense			C|splice_acceptor_variant&intron_variant|HIGH|CTD-2358C21.4|EN
IG_V_gene	1	1	GTT|frameshift_variant|HIGH|IGHV3OR16-8|ENSG00000271130|tra
lincRNA			T|splice_donor_variant&intron_variant|HIGH|RP11-812E19.7|ENSG
protein_co	17	0.12	A|stop_gained|HIGH|PHKB|ENSG00000102893|transcript|ENST000
protein_co	12	0.08	T|stop_gained|HIGH|ABCC12|ENSG00000140798|transcript|ENST0
protein_coding			C|splice_acceptor_variant&conservative_inframe_deletion&splice_r
protein_co	7	0.29	C|frameshift_variant|HIGH|BRD7|ENSG00000166164|transcript|EN
protein_coding			GC|frameshift_variant|HIGH|NOD2|ENSG00000167207|transcript|
antisense			C|splice_donor_variant&intron_variant|HIGH|RP11-327F22.1|ENSG
lincRNA			A|splice_donor_variant&intron_variant|HIGH|RP11-152O14.1|ENSG
lincRNA			C|splice_donor_variant&intron_variant|HIGH|RP11-297L17.4|ENSG
protein_co	3	0.33	GT|frameshift_variant|HIGH|IRX3|ENSG00000177508|transcript|EN
protein_co	8	0.13	C|splice_donor_variant&intron_variant|HIGH|MMP2|ENSG0000008
protein_coding			C|structural_interaction_variant|HIGH|MMP2|ENSG00000087245|i
protein_co	10	0.1	G|start_lost|HIGH|SLC6A2|ENSG00000103546|transcript|ENST000
processed_transcript			G|splice_acceptor_variant&intron_variant|HIGH|BBS2|ENSG000001
processed_transcript			G|splice_donor_variant&intron_variant|HIGH|MT1DP|ENSG000002

protein_co	4	0.25	A|splice_acceptor_variant&intron_variant|HIGH|MT1G|ENSG00000
protein_co	21	0.05	T|splice_donor_variant&intron_variant|HIGH|NUP93|ENSG0000010
protein_coding			TA|frameshift_variant&stop_gained|HIGH|NUP93|ENSG00000102
protein_coding			A|frameshift_variant|HIGH|NLRC5|ENSG00000140853|transcript|E
protein_co	21	0.05	T|splice_acceptor_variant&intron_variant|HIGH|FAM192A|ENSG00
processed_pseudogene			C|splice_acceptor_variant&splice_region_variant&intron_variant|HI
processed_pseudogene			CAAA|splice_acceptor_variant&splice_region_variant&intron_varian
processed_pseudogene			CAAAAAAAAA|splice_acceptor_variant&intron_variant|HIGH|RPL23
processed_pseudogene			CAAAAAAAAAAA|splice_acceptor_variant&intron_variant|HIGH|RPL
processed_pseudogene			CAAAAAAAAAAAA|splice_acceptor_variant&intron_variant|HIGH|RI
processed_pseudogene			CAAAAAAAAAAAAA|splice_acceptor_variant&intron_variant|HIGH|
protein_co	15	0.2	A|stop_gained|HIGH|CIAPIN1|ENSG00000005194|transcript|ENST0
processed_transcript			A|splice_donor_variant&intron_variant|HIGH|DOK4|ENSG0000012
protein_co	9	0.22	T|splice_donor_variant&splice_region_variant&intron_variant|HIGH
protein_coding			G|structural_interaction_variant|HIGH|USB1|ENSG00000103005|in
protein_coding			A|structural_interaction_variant|HIGH|CSNK2A2|ENSG0000007077
lincRNA			A|splice_donor_variant&intron_variant|HIGH|RP11-481J2.2|ENSG0
protein_coding			C|structural_interaction_variant|HIGH|SETD6|ENSG00000103037|i
protein_coding			A|protein_protein_contact|HIGH|CNOT1|ENSG00000125107|intera
protein_coding			G|frameshift_variant|HIGH|CNOT1|ENSG00000125107|transcript|E
protein_coding			T|structural_interaction_variant|HIGH|CNOT1|ENSG00000125107|i
protein_co	1	1	T|frameshift_variant|HIGH|RP11-457D20.2|ENSG00000278499|tran
protein_co	19	0.05	T|start_lost|HIGH|CDH11|ENSG00000140937|transcript|ENST0000
lincRNA			A|splice_donor_variant&intron_variant|HIGH|RP11-256I9.3|ENSG0
lincRNA			G|splice_donor_variant&intron_variant|HIGH|LINC00922|ENSG000
lincRNA			G|splice_donor_variant&intron_variant|HIGH|LINC00922|ENSG000
protein_co	21	0.05	C|splice_acceptor_variant&intron_variant|HIGH|TK2|ENSG0000016
lincRNA			A|splice_donor_variant&intron_variant|HIGH|RP11-61A14.4|ENSG0
nonsense_	22	0.05	A|splice_donor_variant&intron_variant|HIGH|NAE1|ENSG0000015
protein_coding			G|structural_interaction_variant|HIGH|DUS2|ENSG00000167264|in
)265113			C|gene_fusion|HIGH|RP11-70O5.2&SNTB2|ENSG00000265113&EN
)265113			C|gene_fusion|HIGH|RP11-70O5.2&SNTB2|ENSG00000265113&EN
)265113			C|gene_fusion|HIGH|RP11-70O5.2&SNTB2|ENSG00000265113&EN
)265113			C|gene_fusion|HIGH|RP11-70O5.2&SNTB2|ENSG00000265113&EN
protein_coding			A|structural_interaction_variant|HIGH|NQO1|ENSG00000181019|i
protein_coding			A|structural_interaction_variant|HIGH|IL34|ENSG00000157368|int
protein_coding			T|structural_interaction_variant|HIGH|IL34|ENSG00000157368|inte
protein_coding			C|structural_interaction_variant|HIGH|IL34|ENSG00000157368|int
protein_coding			A|stop_gained|HIGH|IL34|ENSG00000157368|transcript|ENST0000
protein_co	23	0.09	G|splice_donor_variant&intron_variant|HIGH|HYDIN|ENSG0000015
nonsense_	27	0.04	A|frameshift_variant&start_lost|HIGH|IST1|ENSG00000182149|tra
protein_co	1	1	A|stop_gained|HIGH|PKD1L3|ENSG00000277481|transcript|ENST0
protein_co	1	1	C|frameshift_variant|HIGH|PKD1L3|ENSG00000277481|transcript|
protein_co	1	1	A|stop_gained|HIGH|PKD1L3|ENSG00000277481|transcript|ENST0
lincRNA			T|splice_acceptor_variant&intron_variant|HIGH|AC009120.5|ENSG
protein_co	11	0.18	C|splice_acceptor_variant&intron_variant|HIGH|CFDP1|ENSG0000
protein_co	7	0.14	C|start_lost|HIGH|TMEM170A|ENSG00000166822|transcript|ENST

sense_intronic			G|splice_acceptor_variant&intron_variant|HIGH|RP11-556H2.2|ENS
protein_co	18	0.06	GGTAAA|frameshift_variant|HIGH|WWOX|ENSG00000186153|tran
processed_transcript			C|splice_acceptor_variant&intron_variant|HIGH|CDYL2|ENSG00000
nonsense_mediated_decay			A|stop_gained|HIGH|CMC2|ENSG00000103121|transcript|ENST000
nonsense_	24	0.04	T|frameshift_variant|HIGH|CMC2|ENSG00000103121|transcript|EN
protein_coding			G|stop_lost|HIGH|CENPN|ENSG00000166451|transcript|ENST0000
protein_co	9	0.11	GTT|splice_acceptor_variant&intron_variant|HIGH|CENPN|ENSG000
protein_co	9	0.11	GTT|splice_acceptor_variant&intron_variant|HIGH|CENPN|ENSG000
polymorphic_pseudogene			G|stop_lost|HIGH|PKD1L2|ENSG00000166473|transcript|ENST0000
polymorph	12	0.17	GTACTGCCAAGCCTGGGGAAAGC|frameshift_variant|HIGH|PKD1L2|
polymorph	12	0.17	A|stop_gained|HIGH|PKD1L2|ENSG00000166473|transcript|ENST0
polymorph	12	0.17	A|stop_gained|HIGH|PKD1L2|ENSG00000166473|transcript|ENST0
polymorph	12	0.17	A|stop_gained|HIGH|PKD1L2|ENSG00000166473|transcript|ENST0
polymorph	12	0.17	G|frameshift_variant|HIGH|PKD1L2|ENSG00000166473|transcript|
polymorph	12	0.17	A|stop_gained|HIGH|PKD1L2|ENSG00000166473|transcript|ENST0
lincRNA			A|splice_donor_variant&splice_region_variant&intron_variant&non_
protein_coding			A|stop_gained|HIGH|SDR42E1|ENSG00000184860|transcript|ENST
lincRNA			T|splice_donor_variant&intron_variant|HIGH|CTD-2588J6.2|ENSG0
antisense			G|splice_donor_variant&intron_variant|HIGH|RP11-543N12.1|ENS
protein_coding			T|stop_gained|HIGH|RP11-505K9.4|ENSG00000260300|transcript|
protein_co	4	0.25	G|splice_donor_variant&intron_variant|HIGH|SLC38A8|ENSG00000
retained_intron			T|splice_donor_variant&intron_variant|HIGH|DNAAF1|ENSG000001
protein_co	15	0.13	C|splice_acceptor_variant&intron_variant|HIGH|ATP2C2|ENSG0000
antisense			C|splice_donor_variant&intron_variant|HIGH|RP11-517C16.4|ENSG
protein_co	11	0.09	C|splice_donor_variant&intron_variant|HIGH|CRISPLD2|ENSG00000
protein_co	7	0.57	CTTT|splice_acceptor_variant&intron_variant|HIGH|KIAA0513|ENS
protein_co	7	0.57	CTTT|splice_acceptor_variant&intron_variant|HIGH|KIAA0513|ENS
protein_co	7	0.57	CTTT|splice_acceptor_variant&intron_variant|HIGH|KIAA0513|ENS
processed_transcript			G|splice_acceptor_variant&intron_variant|HIGH|GSE1|ENSG000001
lincRNA			T|splice_donor_variant&intron_variant|HIGH|LINC00311|ENSG0000
protein_co	16	0.31	C|frameshift_variant|HIGH|GSE1|ENSG00000131149|transcript|EN
protein_co	16	0.31	C|frameshift_variant&splice_donor_variant&splice_region_variant&
antisense			C|splice_donor_variant&intron_variant|HIGH|RP11-568J23.4|ENSG
protein_co	11	0.09	C|splice_donor_variant&intron_variant|HIGH|IRF8|ENSG00000140
lincRNA			C|splice_donor_variant&intron_variant|HIGH|RP11-158I3.3|ENSG00
lincRNA			A|splice_acceptor_variant&intron_variant|HIGH|LINC00917|ENSG0
lincRNA			TA|splice_donor_variant&intron_variant|HIGH|LINC00917|ENSG000
antisense			T|splice_donor_variant&intron_variant|HIGH|RP11-21B21.4|ENSG0
protein_co	5	0.2	G|frameshift_variant|HIGH|ZFPM1|ENSG00000179588|transcript|E
protein_co	5	0.2	C|frameshift_variant|HIGH|ZFPM1|ENSG00000179588|transcript|E
protein_coding			C|structural_interaction_variant|HIGH|MVD|ENSG00000167508|int
nonsense_	17	0.06	A|splice_acceptor_variant&intron_variant|HIGH|MVD|ENSG000001
protein_co	16	0.06	T|frameshift_variant&splice_acceptor_variant&splice_region_varian
protein_coding			A|structural_interaction_variant|HIGH|GALNS|ENSG00000141012|i
protein_coding			A|structural_interaction_variant|HIGH|GALNS|ENSG00000141012|i
protein_coding			C|structural_interaction_variant|HIGH|GALNS|ENSG00000141012|i
protein_coding			A|structural_interaction_variant|HIGH|GALNS|ENSG00000141012|i

protein_co	15	0.07	CTACTTTCTGTGTCTGCATCCAGTCCAGGTGGGCCCTACTTTCTGTGTCTG(
protein_coding			A|stop_gained|HIGH|CDH15|ENSG00000129910|transcript|ENST0C
protein_co	9	0.22	TC|splice_acceptor_variant&intron_variant|HIGH|CPNE7|ENSG0000(
protein_coding			CTG|frameshift_variant|HIGH|SPATA33|ENSG00000167523|transcr
nonsense_mediated_decay			C|stop_gained|HIGH|FANCA|ENSG00000187741|transcript|ENST00
processed_transcript			C|splice_acceptor_variant&intron_variant|HIGH|FANCA|ENSG00000(
protein_co	25	0.04	CGCCCTTCTCTGTGGCT|frameshift_variant|HIGH|TCF25|ENSG00000
protein_co	25	0.04	CGCCCTTCTCTGTGGCT|frameshift_variant|HIGH|TCF25|ENSG00000
protein_co	25	0.04	CGCCCTTCTCTGTGGCT|frameshift_variant|HIGH|TCF25|ENSG00000
protein_co	2	0.5	CA|frameshift_variant|HIGH|CENPBD1|ENSG00000177946|transcri
processed_transcript			G|splice_acceptor_variant&intron_variant|HIGH|AFG3L1P|ENSG000
processed_transcript			A|splice_donor_variant&intron_variant|HIGH|AFG3L1P|ENSG00000
nonsense_mediated_decay			A|stop_gained|HIGH|GAS8|ENSG00000141013|transcript|ENST000
nonsense_mediated_decay			T|stop_gained|HIGH|GAS8|ENSG00000141013|transcript|ENST000
protein_co	5	0.2	CT|frameshift_variant|HIGH|PRDM7|ENSG00000126856|transcript
processed_transcript			C|splice_donor_variant&splice_region_variant&intron_variant&non_
nonsense_	7	0.29	AGGGCC|frameshift_variant|HIGH|FAM57A|ENSG00000167695|tra
retained_intron			T|splice_donor_variant&intron_variant|HIGH|NXN|ENSG00000167(
protein_coding			A|structural_interaction_variant|HIGH|PITPNA|ENSG00000174238|
protein_coding			T|structural_interaction_variant|HIGH|PITPNA|ENSG00000174238|
protein_coding			G|structural_interaction_variant|HIGH|RPA1|ENSG00000132383|in
protein_coding			G|structural_interaction_variant|HIGH|RPA1|ENSG00000132383|in
protein_coding			T|structural_interaction_variant|HIGH|RPA1|ENSG00000132383|in
retained_ir	14	0.29	C|splice_donor_variant&intron_variant|HIGH|CLUH|ENSG00000132
protein_co	14	0.07	G|start_lost|HIGH|SPATA22|ENSG00000141255|transcript|ENST00
protein_co	14	0.14	G|splice_donor_variant&splice_region_variant&intron_variant|HIGH
protein_coding			T|structural_interaction_variant|HIGH|ASPA|ENSG00000108381|in
nonsense_mediated_decay			A|stop_gained|HIGH|TRPV3|ENSG00000167723|transcript|ENST00
protein_co	11	0.09	T|start_lost|HIGH|TRPV3|ENSG00000167723|transcript|ENST00000
protein_co	11	0.36,(P2RX	T|frameshift_variant|HIGH|P2RX5|ENSG00000083454|transcript|EI
protein_coding			A|stop_gained|HIGH|GGT6|ENSG00000167741|transcript|ENST000
antisense			G|splice_donor_variant&intron_variant|HIGH|RP11-314A20.5|ENSG
protein_coding			C|frameshift_variant|HIGH|ZMYND15|ENSG00000141497|transcrip
protein_coding			C|frameshift_variant|HIGH|ZMYND15|ENSG00000141497|transcrip
protein_co	4	0.25	A|splice_donor_variant&intron_variant|HIGH|VMO1|ENSG0000018
nonsense_	13	0.08	TCCCCC|splice_acceptor_variant&intron_variant|HIGH|MINK1|ENS(
nonsense_	13	0.08	TCCCCC|splice_acceptor_variant&intron_variant|HIGH|MINK1|ENS(
nonsense_	13	0.08	TCCCCC|splice_acceptor_variant&intron_variant|HIGH|MINK1|ENS(
protein_coding			A|stop_gained|HIGH|C17orf107|ENSG00000205710|transcript|ENS
protein_co	2	0.5	G|splice_acceptor_variant&splice_region_variant&intron_variant|HI
protein_co	2	0.5	A|frameshift_variant|HIGH|GP1BA|ENSG00000185245|transcript|E
protein_co	2	0.5	A|frameshift_variant|HIGH|GP1BA|ENSG00000185245|transcript|E
protein_co	2	0.5	T|frameshift_variant|HIGH|GP1BA|ENSG00000185245|transcript|E
protein_co	2	1	C|frameshift_variant|HIGH|GP1BA|ENSG00000185245|transcript|E
lincRNA			C|splice_donor_variant&splice_region_variant&intron_variant&non_
lincRNA			T|splice_acceptor_variant&splice_region_variant&intron_variant&n(
antisense			T|intron_variant|MODIFIER|RP11-333E1.1|ENSG00000261879|tran

lincRNA			CA|splice_acceptor_variant&intron_variant|HIGH|RP11-333E1.2|EN
antisense			CA|intron_variant|MODIFIER|RP11-333E1.1|ENSG00000261879|tra
lincRNA			C|splice_acceptor_variant&intron_variant|HIGH|RP11-333E1.2|ENS
antisense			C|intron_variant|MODIFIER|RP11-333E1.1|ENSG00000261879|tran
protein_coding			A|structural_interaction_variant|HIGH|C1QBP|ENSG00000108561|i
protein_coding			C|structural_interaction_variant|HIGH|NLRP1|ENSG00000091592|i
protein_coding			C|structural_interaction_variant|HIGH|NLRP1|ENSG00000091592|i
protein_coding			A|structural_interaction_variant|HIGH|NLRP1|ENSG00000091592|i
protein_co	17	0.06	G|splice_acceptor_variant&intron_variant|HIGH|NLRP1|ENSG00000
processed_transcript			A|splice_donor_variant&intron_variant|HIGH|WSCD1|ENSG000001
processed_pseudogene			G|splice_acceptor_variant&splice_region_variant&intron_variant|HI
processed_pseudogene			G|splice_acceptor_variant&splice_region_variant&intron_variant|HI
processed_pseudogene			G|splice_acceptor_variant&splice_region_variant&intron_variant|HI
protein_co	5	0.4	C|splice_donor_variant&intron_variant|HIGH|PITPNM3|ENSG00000
processed_transcript			A|splice_donor_variant&intron_variant|HIGH|TXNDC17|ENSG00000
processed_transcript			C|splice_donor_variant&intron_variant|HIGH|XAF1|ENSG00000132
)219200			C|gene_fusion|HIGH|C17orf49&RNASEK|ENSG00000258315&ENSG
protein_coding			A|structural_interaction_variant|HIGH|DLG4|ENSG00000132535|in
protein_coding			C|frameshift_variant|HIGH|DVL2|ENSG00000004975|transcript|EN
protein_coding			G|stop_lost|HIGH|ELP5|ENSG00000170291|transcript|ENST000005
protein_coding			A|stop_gained|HIGH|ELP5|ENSG00000170291|transcript|ENST0000
protein_coding			C|frameshift_variant|HIGH|CLDN7|ENSG00000181885|transcript|E
protein_coding			C|frameshift_variant|HIGH|NEURL4|ENSG00000215041|transcript|
protein_coding			C|frameshift_variant|HIGH|NEURL4|ENSG00000215041|transcript|
protein_coding			T|structural_interaction_variant|HIGH|ACAP1|ENSG00000072818|i
protein_co	8	0.13	G|splice_donor_variant&intron_variant|HIGH|TNK1|ENSG0000017
protein_co	8	0.38	G|splice_acceptor_variant&intron_variant|HIGH|TNK1|ENSG00000
protein_co	2	1	A|frameshift_variant&splice_acceptor_variant&splice_region_varian
protein_coding			A|stop_gained|HIGH|SLC35G6|ENSG00000259224|transcript|ENST
protein_co	9	0.11	C|frameshift_variant|HIGH|POLR2A|ENSG00000181222|transcript|
protein_coding			TTA|splice_acceptor_variant&splice_donor_variant&splice_region_v
protein_coding			TTA|splice_acceptor_variant&splice_donor_variant&splice_region_v
protein_coding			TTA|splice_acceptor_variant&splice_donor_variant&splice_region_v
protein_coding			TTA|splice_acceptor_variant&splice_donor_variant&splice_region_v
protein_coding			TTATATATATA|splice_donor_variant&3_prime_UTR_variant&intron_
protein_coding			TTATATATATA|splice_donor_variant&3_prime_UTR_variant&intron_
protein_coding			TTATATATATA|splice_donor_variant&3_prime_UTR_variant&intron_
protein_coding			TTATATATATA|splice_donor_variant&3_prime_UTR_variant&intron_
protein_coding			TTATATATATA|splice_donor_variant&3_prime_UTR_variant&intron_
protein_coding			T|splice_acceptor_variant&splice_donor_variant&splice_region_vari
protein_coding			T|splice_acceptor_variant&splice_donor_variant&splice_region_vari
protein_co	10	0.1	T|splice_acceptor_variant&splice_region_variant&3_prime_UTR_var
protein_co	10	0.1	T|splice_acceptor_variant&splice_region_variant&3_prime_UTR_var
protein_co	10	0.1	T|splice_acceptor_variant&splice_region_variant&3_prime_UTR_var
protein_coding			T|splice_acceptor_variant&splice_region_variant&3_prime_UTR_var
protein_coding			T|splice_acceptor_variant&splice_region_variant&3_prime_UTR_var
protein_coding			T|splice_acceptor_variant&splice_region_variant&3_prime_UTR_var

protein_coding			T|splice_acceptor_variant&splice_region_variant&3_prime_UTR_var
protein_coding			T|splice_acceptor_variant&splice_region_variant&3_prime_UTR_var
protein_coding			A|splice_acceptor_variant&splice_region_variant&3_prime_UTR_va
protein_coding			T|splice_acceptor_variant&splice_region_variant&3_prime_UTR_var
protein_coding			T|splice_acceptor_variant&splice_region_variant&3_prime_UTR_var
protein_coding			T|splice_acceptor_variant&splice_region_variant&3_prime_UTR_var
protein_coding			T|splice_acceptor_variant&splice_region_variant&3_prime_UTR_var
protein_coding			T|splice_acceptor_variant&splice_region_variant&3_prime_UTR_var
protein_coding			A|splice_acceptor_variant&splice_region_variant&3_prime_UTR_va
protein_coding			A|splice_acceptor_variant&splice_region_variant&3_prime_UTR_va
protein_coding			A|splice_acceptor_variant&splice_region_variant&3_prime_UTR_va
protein_coding			T|splice_acceptor_variant&splice_region_variant&3_prime_UTR_var
protein_coding			T|splice_acceptor_variant&splice_region_variant&3_prime_UTR_var
protein_coding			A|splice_acceptor_variant&splice_region_variant&3_prime_UTR_va
protein_coding			A|splice_acceptor_variant&splice_region_variant&3_prime_UTR_va
protein_coding			T|splice_acceptor_variant&splice_region_variant&3_prime_UTR_var
protein_coding			T|splice_acceptor_variant&splice_region_variant&3_prime_UTR_var
protein_coding			T|splice_acceptor_variant&splice_region_variant&3_prime_UTR_var
protein_coding			T|splice_acceptor_variant&splice_region_variant&3_prime_UTR_var
protein_coding			T|splice_acceptor_variant&splice_region_variant&3_prime_UTR_var
protein_coding			T|splice_acceptor_variant&splice_region_variant&3_prime_UTR_var
protein_coding			T|splice_acceptor_variant&splice_region_variant&3_prime_UTR_var
protein_coding			T|splice_acceptor_variant&3_prime_UTR_variant&intron_variant|H
protein_coding			T|splice_acceptor_variant&3_prime_UTR_variant&intron_variant|H
protein_coding			T|splice_acceptor_variant&3_prime_UTR_variant&intron_variant|H
protein_coding			T|structural_interaction_variant|HIGH|EIF4A1|ENSG00000161960|i
protein_co	27	0.11	T|frameshift_variant|HIGH|MPDU1|ENSG00000129255|transcript|
protein_coding			A|structural_interaction_variant|HIGH|SAT2|ENSG00000141504|int
nonsense_	28	0.04	T|frameshift_variant|HIGH|TP53|ENSG00000141510|transcript|ENS
nonsense_	28	0.04	T|frameshift_variant|HIGH|TP53|ENSG00000141510|transcript|ENS
nonsense_	28	0.04	T|frameshift_variant|HIGH|TP53|ENSG00000141510|transcript|ENS
nonsense_mediated_decay			T|stop_gained|HIGH|TP53|ENSG00000141510|transcript|ENST0000
nonsense_mediated_decay			T|stop_gained|HIGH|TP53|ENSG00000141510|transcript|ENST0000
protein_coding			C|structural_interaction_variant|HIGH|TP53|ENSG00000141510|int
protein_co	6	0.33	C|frameshift_variant|HIGH|KDM6B|ENSG00000132510|transcript|
protein_co	7	0.14	T|stop_gained|HIGH|NAA38|ENSG00000183011|transcript|ENST00
protein_coding			T|structural_interaction_variant|HIGH|ALOX15B|ENSG0000017959
protein_coding			G|structural_interaction_variant|HIGH|ALOX15B|ENSG0000017959
protein_coding			T|structural_interaction_variant|HIGH|ALOX15B|ENSG0000017959
protein_coding			T|structural_interaction_variant|HIGH|ALOX15B|ENSG0000017959
protein_coding			A|structural_interaction_variant|HIGH|ALOX15B|ENSG0000017959
protein_coding			A|structural_interaction_variant|HIGH|ALOX15B|ENSG0000017959
protein_coding			G|structural_interaction_variant|HIGH|ALOX15B|ENSG0000017959
protein_co	4	0.25	G|splice_donor_variant&intron_variant|HIGH|VAMP2|ENSG000002
protein_coding			C|stop_gained|HIGH|TMEM107|ENSG00000179029|transcript|ENS
protein_co	6	0.17	T|splice_donor_variant&intron_variant|HIGH|TMEM220|ENSG0000
protein_co	15	0.07	ACGCGGATGGGGAACCCGGCGCCGAC|frameshift_variant|HIGH|DNA

protein_co	5	0.4	C|frameshift_variant|HIGH|MYOCD|ENSG00000141052|transcript|
protein_co	12	0.42	C|frameshift_variant|HIGH|ARHGAP44|ENSG00000006740|transcri
unprocessed_pseudogene			A|splice_donor_variant&intron_variant|HIGH|CDRT15P1|ENSG0000
nonsense_mediated_decay			A|stop_gained|HIGH|COX10|ENSG00000006695|transcript|ENST00
processed_pseudogene			A|splice_donor_variant&intron_variant|HIGH|SRP68P1|ENSG00000
transcribed_unprocessed_pseudo			C|splice_acceptor_variant&splice_region_variant&intron_variant|HI
processed_transcript			T|splice_donor_variant&intron_variant|HIGH|TBC1D27|ENSG00000
protein_co	6	0.33	A|frameshift_variant|HIGH|RAI1|ENSG00000108557|transcript|EN
protein_co	6	0.33	A|frameshift_variant|HIGH|RAI1|ENSG00000108557|transcript|EN
protein_co	6	0.33	ACAG|frameshift_variant|HIGH|RAI1|ENSG00000108557|transcript
protein_co	6	0.33	ACAG|frameshift_variant|HIGH|RAI1|ENSG00000108557|transcript
protein_co	9	0.11	T|frameshift_variant|HIGH|MIEF2|ENSG00000177427|transcript|E
protein_coding			A|structural_interaction_variant|HIGH|TOP3A|ENSG00000177302|
processed_transcript			GT|splice_donor_variant&intron_variant|HIGH|KRT17P5|ENSG0000
protein_co	15	0.53	G|frameshift_variant&start_lost|HIGH|LGALS9C|ENSG0000017191
antisense			G|splice_donor_variant&intron_variant|HIGH|AC090286.4|ENSG00
protein_co	27	0.3	A|splice_donor_variant&intron_variant|HIGH|EPN2|ENSG0000007
protein_co	26	0.12	T|stop_gained&splice_region_variant|HIGH|ALDH3A2|ENSG000000
transcribed_processed_pseudoge			G|splice_donor_variant&intron_variant|HIGH|CCDC144CP|ENSG000
protein_co	6	0.5	ATC|frameshift_variant&start_lost|HIGH|LGALS9B|ENSG000001702
retained_intron			C|splice_acceptor_variant&splice_region_variant&intron_variant|HI
protein_co	10	0.1	A|splice_donor_variant&intron_variant|HIGH|DHRS7B|ENSG00000
lincRNA			G|splice_donor_variant&intron_variant|HIGH|FAM27E5|ENSG0000
lincRNA			A|splice_donor_variant&intron_variant|HIGH|RP11-663N22.1|ENSG
processed_transcript			T|splice_donor_variant&intron_variant|HIGH|TBC1D3P5|ENSG0000
transcribed_unprocessed_pseudo			T|splice_donor_variant&intron_variant|HIGH|NOS2P1|ENSG000002
protein_coding			C|structural_interaction_variant|HIGH|UNC119|ENSG00000109103
nonsense_	16	0.06	G|splice_acceptor_variant&intron_variant|HIGH|SPAG5|ENSG0000
protein_coding			C|frameshift_variant|HIGH|RPL23A|ENSG00000198242|transcript|
unprocessed_pseudogene			C|splice_acceptor_variant&intron_variant|HIGH|RP11-20B24.3|ENS
unprocessed_pseudogene			C|splice_acceptor_variant&intron_variant|HIGH|RP11-20B24.3|ENS
processed_transcript			T|splice_donor_variant&intron_variant|HIGH|PIPOX|ENSG0000017
protein_co	10	0.1	TG|frameshift_variant|HIGH|SEZ6|ENSG00000063015|transcript|E
protein_co	12	0.17	C|splice_donor_variant&intron_variant|HIGH|TP53I13|ENSG000001
protein_co	12	0.17	A|start_lost|HIGH|EFCAB5|ENSG00000176927|transcript|ENST0000
protein_co	12	0.17	A|splice_donor_variant&intron_variant|HIGH|EFCAB5|ENSG000001
protein_co	4	0.75	A|splice_acceptor_variant&intron_variant|HIGH|TBC1D29|ENSG000
processed_transcript			CTTTTTT|splice_acceptor_variant&intron_variant|HIGH|LRRC37BP1
processed_transcript			CTTTTTT|splice_acceptor_variant&intron_variant|HIGH|LRRC37BP1
processed_transcript			CTTTTTT|splice_acceptor_variant&intron_variant|HIGH|LRRC37BP1
processed_transcript			CTTTTTT|splice_acceptor_variant&intron_variant|HIGH|LRRC37BP1
processed_transcript			CTTTTTT|splice_acceptor_variant&intron_variant|HIGH|LRRC37BP1
nonsense_mediated_decay			C|stop_lost|HIGH|NF1|ENSG00000196712|transcript|ENST0000047
nonsense_	23	0.09	A|splice_donor_variant&intron_variant|HIGH|NF1|ENSG000001967
nonsense_	5	0.2	GC|frameshift_variant|HIGH|COPRS|ENSG00000172301|transcript|
protein_co	4	0.5	CTT|splice_acceptor_variant&intron_variant|HIGH|SUZ12|ENSG000
protein_co	4	0.5	CTT|splice_acceptor_variant&intron_variant|HIGH|SUZ12|ENSG000

)214708			C|bidirectional_gene_fusion|HIGH|AC090616.2&RHOT1|ENSG0000(
)214708			C|bidirectional_gene_fusion|HIGH|AC090616.2&RHOT1|ENSG0000(
)214708			C|bidirectional_gene_fusion|HIGH|AC090616.2&RHOT1|ENSG0000(
)214708			C|bidirectional_gene_fusion|HIGH|AC090616.2&RHOT1|ENSG0000(
)214708			C|bidirectional_gene_fusion|HIGH|AC090616.2&RHOT1|ENSG0000(
)214708			C|bidirectional_gene_fusion|HIGH|AC090616.2&RHOT1|ENSG0000(
lincRNA			GAAAAAAAA|splice_acceptor_variant&splice_region_variant&intron
lincRNA			GAAAAAAAA|splice_acceptor_variant&splice_region_variant&intron
protein_co	11	0.45	T|stop_gained|HIGH|SLFN13|ENSG00000154760|transcript|ENST0(
protein_co	5	0.6	A|frameshift_variant|HIGH|SLFN12L|ENSG00000205045|transcript|
protein_co	5	0.2	C|start_lost|HIGH|SLFN12L|ENSG00000205045|transcript|ENST000
protein_co	1	1	T|splice_donor_variant&intron_variant|HIGH|SLFN14|ENSG000002:
protein_coding			C|structural_interaction_variant|HIGH|AP2B1|ENSG00000006125|i
protein_coding			T|structural_interaction_variant|HIGH|LASP1|ENSG00000002834|ir
antisense			G|splice_donor_variant&intron_variant|HIGH|RP5-906A24.2|ENSG(
processed_transcript			T|splice_donor_variant&intron_variant|HIGH|CACNB1|ENSG00000(
protein_co	10	0.3	C|frameshift_variant|HIGH|CDK12|ENSG00000167258|transcript|E
nonsense_mediated_decay			C|stop_lost|HIGH|STARD3|ENSG00000131748|transcript|ENST0000(
protein_coding			C|structural_interaction_variant|HIGH|STARD3|ENSG00000131748|
protein_coding			G|structural_interaction_variant|HIGH|PNMT|ENSG00000141744|ir
protein_coding			A|structural_interaction_variant|HIGH|PNMT|ENSG00000141744|ir
protein_co	15	0.2	C|splice_acceptor_variant&intron_variant|HIGH|GSDMB|ENSG0000C
protein_co	35	0.03	AACAC|frameshift_variant|HIGH|MED24|ENSG00000008838|transc
protein_co	35	0.03	AACAC|frameshift_variant|HIGH|MED24|ENSG00000008838|transc
protein_co	35	0.03	AACAC|frameshift_variant|HIGH|MED24|ENSG00000008838|transc
protein_co	35	0.03	CACACACACAT|frameshift_variant|HIGH|MED24|ENSG000000088:
protein_co	35	0.03	CACACACACAT|frameshift_variant|HIGH|MED24|ENSG000000088:
protein_co	35	0.03	CACACACACAT|frameshift_variant|HIGH|MED24|ENSG000000088:
protein_co	35	0.03	CACACACACAT|frameshift_variant|HIGH|MED24|ENSG000000088:
protein_co	35	0.17	GC|frameshift_variant|HIGH|MED24|ENSG00000008838|transcript
protein_co	11	0.09	C|frameshift_variant|HIGH|CASC3|ENSG00000108349|transcript|EI
protein_co	11	0.18	TTG|splice_acceptor_variant&intron_variant|HIGH|CASC3|ENSG000(
protein_co	3	0.33	C|start_lost|HIGH|CCR7|ENSG00000126353|transcript|ENST00000!
protein_co	1	1	C|frameshift_variant|HIGH|KRT24|ENSG00000167916|transcript|EI
protein_co	3	0.33	C|frameshift_variant&splice_donor_variant&splice_region_variant&
protein_co	1	1	G|frameshift_variant|HIGH|KRTAP4-3|ENSG00000196156|transcrip
protein_co	1	1	TGGAGATA|frameshift_variant|HIGH|KRTAP4-3|ENSG00000196156
protein_coding			A|splice_acceptor_variant&disruptive_inframe_deletion&splice_regi
protein_co	4	0.25	G|start_lost|HIGH|KRTAP9-1|ENSG00000240542|transcript|ENST0(
protein_co	4	0.25	TGCTGTGGGTCCAGCTGCTGCCAGCCTA|splice_donor_variant&intron_
unprocessed_pseudogene			T|splice_acceptor_variant&intron_variant|HIGH|KRT43P|ENSG0000
protein_coding			C|structural_interaction_variant|HIGH|ACLY|ENSG00000131473|int
protein_coding			G|structural_interaction_variant|HIGH|ACLY|ENSG00000131473|int
protein_co	14	0.07	A|splice_donor_variant&intron_variant|HIGH|NKIRAS2|ENSG00000
protein_coding			T|structural_interaction_variant|HIGH|DHX58|ENSG00000108771|i
protein_coding			A|splice_acceptor_variant&disruptive_inframe_deletion&splice_regi
protein_co	5	0.4	G|frameshift_variant&splice_donor_variant&splice_region_variant&

protein_co	5	0.4	C|splice_donor_variant&intron_variant|HIGH|KAT2A|ENSG0000010
protein_co	5	0.4	C|frameshift_variant&splice_region_variant|HIGH|KAT2A|ENSG000
protein_coding			C|frameshift_variant&stop_lost|HIGH|RAB5C|ENSG00000108774|tr
protein_coding			C|frameshift_variant&stop_lost|HIGH|RAB5C|ENSG00000108774|tr
protein_coding			C|frameshift_variant&stop_lost|HIGH|RAB5C|ENSG00000108774|tr
protein_coding			C|frameshift_variant&stop_lost|HIGH|RAB5C|ENSG00000108774|tr
protein_co	26	0.04	TA|frameshift_variant&splice_region_variant|HIGH|ATP6V0A1|ENSG
protein_co	26	0.04	G|frameshift_variant|HIGH|ATP6V0A1|ENSG00000033627|transcrip
protein_coding			A|structural_interaction_variant|HIGH|AOC3|ENSG00000131471|in
nonsense_	9	0.11	A|splice_donor_variant&intron_variant|HIGH|VAT1|ENSG00000108
protein_coding			T|structural_interaction_variant|HIGH|BRCA1|ENSG00000012048|i
processed_transcript			C|splice_donor_variant&intron_variant|HIGH|NBR2|ENSG0000019
protein_coding			A|structural_interaction_variant|HIGH|DHX8|ENSG00000067596|in
antisense			CTT|splice_acceptor_variant&intron_variant|HIGH|RP11-392O1.4|E
antisense			CTT|splice_acceptor_variant&intron_variant|HIGH|RP11-392O1.4|E
protein_coding			A|structural_interaction_variant|HIGH|DUSP3|ENSG00000108861|i
protein_co	21	0.05	T|splice_donor_variant&intron_variant|HIGH|MPP2|ENSG0000010
antisense			G|splice_acceptor_variant&intron_variant|HIGH|ASB16-AS1|ENSG0
protein_co	15	0.07	C|splice_donor_variant&intron_variant|HIGH|TMUB2|ENSG000001
protein_co	13	0.08	A|splice_donor_variant&intron_variant|HIGH|EFCAB13|ENSG00000
protein_co	13	0.08	T|stop_gained|HIGH|EFCAB13|ENSG00000178852|transcript|ENST
protein_co	13	0.08	T|stop_gained|HIGH|EFCAB13|ENSG00000178852|transcript|ENST
protein_co	13	0.23	A|splice_acceptor_variant&intron_variant|HIGH|EFCAB13|ENSG000
protein_co	13	0.15	T|stop_gained|HIGH|EFCAB13|ENSG00000178852|transcript|ENST
protein_co	13	0.23	G|stop_gained|HIGH|EFCAB13|ENSG00000178852|transcript|ENST
protein_coding			A|structural_interaction_variant|HIGH|KPNB1|ENSG00000108424|i
protein_coding			C|structural_interaction_variant|HIGH|KPNB1|ENSG00000108424|i
nonsense_	9	0.22	G|frameshift_variant|HIGH|TTLL6|ENSG00000170703|transcript|EN
nonsense_	17	0.06	C|frameshift_variant&splice_region_variant|HIGH|SNF8|ENSG00000
protein_coding			G|splice_donor_variant&5_prime_UTR_variant&intron_variant|HIGH
protein_coding			T|structural_interaction_variant|HIGH|PDK2|ENSG00000005882|int
protein_coding			G|frameshift_variant|HIGH|SGCA|ENSG00000108823|transcript|EN
lincRNA			CA|splice_acceptor_variant&intron_variant|HIGH|RP11-893F2.5|EN
lincRNA			CTTT|splice_acceptor_variant&intron_variant|HIGH|RP11-893F2.5|
lincRNA			CTTT|splice_acceptor_variant&intron_variant|HIGH|RP11-893F2.5|
lincRNA			CTTT|splice_acceptor_variant&intron_variant|HIGH|RP11-893F2.5|
protein_co	7	0.29	C|splice_acceptor_variant&splice_region_variant&intron_variant|HI
protein_coding			T|structural_interaction_variant|HIGH|EME1|ENSG00000154920|in
nonsense_	28	0.07	G|splice_donor_variant&splice_region_variant&intron_variant|HIGH
protein_co	9	0.22	A|splice_donor_variant&intron_variant|HIGH|NME2|ENSG0000024
protein_co	8	0.13	A|splice_acceptor_variant&intron_variant|HIGH|STXBP4|ENSG0000
protein_coding			C|structural_interaction_variant|HIGH|PCTP|ENSG00000141179|int
protein_coding			T|structural_interaction_variant|HIGH|PCTP|ENSG00000141179|int
protein_co	9	0.11	TCCCCC|frameshift_variant|HIGH|ANKFN1|ENSG00000153930|tran
protein_co	9	0.11	TCCCCCC|disruptive_inframe_insertion|MODERATE|ANKFN1|ENSG
)279281			GGAAAGAAAGAAA|gene_fusion|HIGH|MSI2&RP11-226M10.3|ENSG
)279281			GGAAAGAAAGAAA|gene_fusion|HIGH|MSI2&RP11-226M10.3|ENSG

)279281			GGAAAGAAAGAAA|gene_fusion|HIGH|MSI2&RP11-226M10.3|ENSG
)279281			GGAAAGAAAGAAA|gene_fusion|HIGH|MSI2&RP11-226M10.3|ENSG
)279281			G|gene_fusion|HIGH|MSI2&RP11-226M10.3|ENSG00000153944&E
protein_co	1	1	A|stop_gained|HIGH|EPX|ENSG00000121053|transcript|ENST0000(
protein_co	5	0.2	G|splice_acceptor_variant&intron_variant|HIGH|MPO|ENSG000000(
antisense			G|splice_acceptor_variant&intron_variant|HIGH|TSPOAP1-AS1|ENS
protein_coding			T|structural_interaction_variant|HIGH|SUPT4H1|ENSG0000021324(
nonsense_mediated_decay			A|stop_gained|HIGH|TRIM37|ENSG00000108395|transcript|ENST0
protein_coding			A|frameshift_variant|HIGH|TUBD1|ENSG00000108423|transcript|E
lincRNA			A|splice_donor_variant&intron_variant|HIGH|RP11-15E18.4|ENSG0
antisense			T|splice_donor_variant&intron_variant|HIGH|TBX2-AS1|ENSG00000(
processed_transcript			C|splice_acceptor_variant&intron_variant|HIGH|TBX4|ENSG000001
protein_coding			A|stop_gained|HIGH|NACA2|ENSG00000253506|transcript|ENST0C
processed_transcript			G|splice_acceptor_variant&intron_variant|HIGH|EFCAB3|ENSG0000(
processed_pseudogene			C|splice_acceptor_variant&intron_variant|HIGH|RPS23P7|ENSG000
processed_pseudogene			C|splice_acceptor_variant&intron_variant|HIGH|RPS23P7|ENSG000
processed_pseudogene			C|splice_acceptor_variant&intron_variant|HIGH|RPS23P7|ENSG000
protein_co	10	0.1	G|splice_donor_variant&intron_variant|HIGH|MARCH10|ENSG0000(
nonsense_	22	0.05	A|stop_gained|HIGH|ACE|ENSG00000159640|transcript|ENST0000
protein_coding			C|frameshift_variant|HIGH|ACE|ENSG00000159640|transcript|ENS
protein_co	16	0.13	A|splice_donor_variant&intron_variant|HIGH|FTSJ3|ENSG0000010(
protein_coding			C|frameshift_variant|HIGH|SMARCD2|ENSG00000108604|transcrip
protein_coding			C|structural_interaction_variant|HIGH|ERN1|ENSG00000178607|in
protein_co	19	0.32	TC|splice_acceptor_variant&intron_variant|HIGH|CEP95|ENSG0000
protein_co	19	0.32	TC|splice_acceptor_variant&intron_variant|HIGH|CEP95|ENSG0000
processed_transcript			C|splice_acceptor_variant&intron_variant|HIGH|PLEKHM1P1|ENSG
protein_coding			C|structural_interaction_variant|HIGH|APOH|ENSG00000091583|ir
protein_coding			G|stop_lost|HIGH|C17orf58|ENSG00000186665|transcript|ENST00(
protein_coding			G|structural_interaction_variant|HIGH|KPNA2|ENSG00000182481|
protein_co	7	0.29	ATT|splice_acceptor_variant&intron_variant|HIGH|KPNA2|ENSG000(
protein_co	7	0.29	ATT|splice_acceptor_variant&intron_variant|HIGH|KPNA2|ENSG000(
protein_co	19	0.37	T|frameshift_variant|HIGH|AMZ2|ENSG00000196704|transcript|EN
nonsense_	21	0.05	CT|frameshift_variant|HIGH|PRKAR1A|ENSG00000108946|transcrip
lincRNA			TC|splice_acceptor_variant&intron_variant|HIGH|LINC01482|ENSG(
protein_co	12	0.17	T|frameshift_variant|HIGH|ABCA10|ENSG00000154263|transcript|
protein_co	12	0.08	A|stop_gained&splice_region_variant|HIGH|ABCA10|ENSG0000015
nonsense_	12	0.08	C|splice_acceptor_variant&intron_variant|HIGH|ABCA10|ENSG0000(
protein_co	12	0.17	A|frameshift_variant|HIGH|ABCA10|ENSG00000154263|transcript|
nonsense_mediated_decay			C|splice_donor_variant&3_prime_UTR_variant&intron_variant|HIGH
protein_co	12	0.33	T|stop_gained|HIGH|ABCA10|ENSG00000154263|transcript|ENST0
processed_transcript			T|splice_donor_variant&intron_variant|HIGH|ABCA10|ENSG000001
protein_co	11	0.09	G|start_lost|HIGH|KCNJ16|ENSG00000153822|transcript|ENST0000(
protein_co	11	0.27	A|splice_donor_variant&intron_variant|HIGH|KCNJ16|ENSG000001
protein_co	1	1	C|frameshift_variant|HIGH|SOX9|ENSG00000125398|transcript|EN
lincRNA			A|splice_donor_variant&intron_variant|HIGH|LINC00511|ENSG0000(
protein_co	10	0.1	G|splice_donor_variant&splice_region_variant&intron_variant|HIGH
protein_co	10	0.1	G|splice_donor_variant&splice_region_variant&intron_variant|HIGH

protein_co	10	0.1	C|splice_donor_variant&splice_region_variant&intron_variant|HIGH
protein_coding			T|splice_acceptor_variant&splice_region_variant&5_prime_UTR_var
protein_coding			T|splice_acceptor_variant&splice_region_variant&5_prime_UTR_var
protein_coding			C|frameshift_variant&stop_lost|HIGH|C17orf80|ENSG0000014121
lincRNA			C|splice_donor_variant&intron_variant|HIGH|RP11-101O21.1|ENSG
lincRNA			T|splice_donor_variant&intron_variant|HIGH|RP11-101O21.1|ENSG
antisense			T|splice_acceptor_variant&intron_variant|HIGH|MGC16275|ENSG0
protein_coding			G|frameshift_variant&splice_region_variant|HIGH|TTYH2|ENSG000
protein_coding			T|frameshift_variant|HIGH|CD300C|ENSG00000167850|transcript|
protein_co	29	0.03	C|frameshift_variant|HIGH|NAT9|ENSG00000109065|transcript|EN
nonsense_	29	0.03	GA|frameshift_variant|HIGH|NAT9|ENSG00000109065|transcript|E
protein_coding			C|frameshift_variant&splice_acceptor_variant&splice_region_varian
protein_coding			G|frameshift_variant&splice_acceptor_variant&splice_region_variar
protein_co	2	0.5	C|start_lost|HIGH|OTOP3|ENSG00000182938|transcript|ENST0000
protein_coding			T|structural_interaction_variant|HIGH|NT5C|ENSG00000125458|in
protein_coding			G|structural_interaction_variant|HIGH|GGA3|ENSG00000125447|ir
nonsense_mediated_decay			C|stop_gained|HIGH|GGA3|ENSG00000125447|transcript|ENST000
nonsense_	36	0.11	CG|frameshift_variant|HIGH|MYO15B|ENSG00000266714|transcrip
nonsense_	36	0.06	A|stop_gained|HIGH|MYO15B|ENSG00000266714|transcript|ENST
protein_co	36	0.06	A|splice_donor_variant&intron_variant|HIGH|MYO15B|ENSG00000
nonsense_mediated_decay			G|stop_gained|HIGH|MYO15B|ENSG00000266714|transcript|ENST
protein_co	36	0.06	C|frameshift_variant|HIGH|MYO15B|ENSG00000266714|transcript
protein_co	36	0.06	C|frameshift_variant&splice_region_variant|HIGH|MYO15B|ENSG0
protein_co	36	0.08	C|splice_donor_variant&intron_variant|HIGH|MYO15B|ENSG00000
protein_coding			A|structural_interaction_variant|HIGH|ITGB4|ENSG00000132470|ir
protein_co	6	0.17	T|stop_gained|HIGH|ZACN|ENSG00000186919|transcript|ENST000
antisense			C|splice_acceptor_variant&intron_variant|HIGH|RP11-449J21.5|EN
antisense			C|splice_acceptor_variant&intron_variant|HIGH|RP11-449J21.5|EN
protein_coding			C|structural_interaction_variant|HIGH|SPHK1|ENSG00000176170|i
protein_coding			A|structural_interaction_variant|HIGH|SPHK1|ENSG00000176170|i
protein_coding			T|structural_interaction_variant|HIGH|SPHK1|ENSG00000176170|ir
processed_transcript			G|splice_acceptor_variant&intron_variant|HIGH|ST6GALNAC1|ENS
protein_co	47	0.02	A|splice_donor_variant&intron_variant|HIGH|SEPT9|ENSG0000018
lincRNA			C|splice_donor_variant&intron_variant|HIGH|RP11-13K12.2|ENSG0
processed_pseudogene			C|splice_donor_variant&intron_variant|HIGH|EIF5AP2|ENSG000002
protein_coding			T|structural_interaction_variant|HIGH|TK1|ENSG00000167900|inte
nonsense_mediated_decay			A|stop_gained|HIGH|LGALS3BP|ENSG00000108679|transcript|ENS
protein_co	16	0.13	T|splice_acceptor_variant&intron_variant|HIGH|CANT1|ENSG00000
protein_co	16	0.06	C|frameshift_variant|HIGH|CCDC40|ENSG00000141519|transcript|
protein_coding			G|frameshift_variant|HIGH|CCDC40|ENSG00000141519|transcript|
protein_coding			AG|frameshift_variant|HIGH|CCDC40|ENSG00000141519|transcript
protein_coding			C|stop_lost|HIGH|CCDC40|ENSG00000141519|transcript|ENST0000
protein_coding			A|structural_interaction_variant|HIGH|EIF4A3|ENSG00000141543|i
nonsense_	14	0.07	T|stop_gained|HIGH|CARD14|ENSG00000141527|transcript|ENST0
nonsense_	14	0.07	T|stop_gained|HIGH|CARD14|ENSG00000141527|transcript|ENST0
protein_coding			T|structural_interaction_variant|HIGH|SGSH|ENSG00000181523|in
protein_coding			A|structural_interaction_variant|HIGH|ENDOV|ENSG00000173818|

nonsense_	30	0.37	G|stop_lost|HIGH|ENDOV|ENSG00000173818|transcript|ENST0000
)141564			A|bidirectional_gene_fusion|HIGH|CTD-2561B21.3&RPTOR|ENSG00
antisense			T|splice_donor_variant&intron_variant|HIGH|CTD-2561B21.5|ENSG
antisense			T|splice_donor_variant&intron_variant|HIGH|AC027601.1|ENSG000
nonsense_	13	0.15	C|frameshift_variant|HIGH|TEPSIN|ENSG00000167302|transcript|E
protein_coding			T|frameshift_variant&stop_lost|HIGH|SLC38A10|ENSG0000015763
lincRNA			C|splice_acceptor_variant&intron_variant|HIGH|RP11-1055B8.4|EN
protein_co	16	0.13	T|splice_acceptor_variant&intron_variant|HIGH|ACTG1|ENSG00000
protein_co	3	0.67	T|stop_gained|HIGH|FSCN2|ENSG00000186765|transcript|ENST000
nonsense_	5	0.2	G|splice_acceptor_variant&intron_variant|HIGH|TSPAN10|ENSG000
protein_co	5	0.4	TTAAC|frameshift_variant&stop_gained|HIGH|TSPAN10|ENSG00000
protein_co	6	0.17	C|frameshift_variant|HIGH|PDE6G|ENSG00000185527|transcript|E
nonsense_mediated_decay			C|stop_lost|HIGH|CCDC137|ENSG00000185298|transcript|ENST000
protein_coding			A|structural_interaction_variant|HIGH|P4HB|ENSG00000185624|in
protein_co	3	0.33	G|splice_acceptor_variant&intron_variant|HIGH|MAFG|ENSG00000
protein_coding			A|structural_interaction_variant|HIGH|NOTUM|ENSG00000185269
protein_coding			A|protein_protein_contact|HIGH|STRA13|ENSG00000169689|intera
protein_coding			T|protein_protein_contact|HIGH|STRA13|ENSG00000169689|intera
protein_coding			T|structural_interaction_variant|HIGH|RAC3|ENSG00000169750|in
protein_coding			T|structural_interaction_variant|HIGH|DCXR|ENSG00000169738|in
protein_co	21	0.05	CTT|frameshift_variant&splice_region_variant|HIGH|DCXR|ENSG00
protein_coding			A|structural_interaction_variant|HIGH|FASN|ENSG00000169710|in
protein_coding			A|structural_interaction_variant|HIGH|FASN|ENSG00000169710|in
protein_coding			G|structural_interaction_variant|HIGH|FASN|ENSG00000169710|in
protein_coding			G|structural_interaction_variant|HIGH|FASN|ENSG00000169710|in
protein_coding			A|stop_gained|HIGH|CCDC57|ENSG00000176155|transcript|ENST0
antisense			A|splice_acceptor_variant&splice_region_variant&intron_variant&n
protein_coding			A|structural_interaction_variant|HIGH|CSNK1D|ENSG00000141551
protein_co	27	0.04	T|splice_acceptor_variant&intron_variant|HIGH|NARF|ENSG000001
protein_co	26	0.08	CTT|splice_acceptor_variant&intron_variant|HIGH|TBCD|ENSG0000
protein_co	26	0.08	CTT|splice_acceptor_variant&intron_variant|HIGH|TBCD|ENSG0000
protein_co	26	0.08	CTT|splice_acceptor_variant&intron_variant|HIGH|TBCD|ENSG0000
unprocessed_pseudogene			G|splice_acceptor_variant&splice_region_variant&intron_variant&n
processed_transcript			C|splice_acceptor_variant&intron_variant|HIGH|TUBB8P12|ENSG00
processed_transcript			G|splice_donor_variant&splice_region_variant&intron_variant&non
nonsense_	22	0.05	AAGCTGTTTCCCCTGGAGAGTCC|frameshift_variant|HIGH|ENOSF1|E
lincRNA			A|splice_donor_variant&intron_variant|HIGH|CTD-2015H3.2|ENSG(
protein_coding			C|structural_interaction_variant|HIGH|MYOM1|ENSG00000101605
protein_coding			A|structural_interaction_variant|HIGH|MYOM1|ENSG00000101605
protein_coding			T|structural_interaction_variant|HIGH|MYOM1|ENSG00000101605
protein_co	4	0.25	T|splice_donor_variant&intron_variant|HIGH|MYL12B|ENSG000001
protein_co	23	0.04	T|splice_donor_variant&intron_variant|HIGH|TGIF1|ENSG0000017
protein_co	23	0.04	C|frameshift_variant|HIGH|TGIF1|ENSG00000177426|transcript|EN
protein_co	23	0.04	G|frameshift_variant|HIGH|TGIF1|ENSG00000177426|transcript|EN
antisense			C|splice_donor_variant&intron_variant|HIGH|RP11-286N3.2|ENSG(
processed_transcript			A|splice_donor_variant&intron_variant|HIGH|PTPRM|ENSG0000017
protein_co	16	0.13	C|splice_acceptor_variant&intron_variant|HIGH|MTCL1|ENSG00000

protein_coding			GC|frameshift_variant|HIGH|NDUFV2|ENSG00000178127|transcrip
antisense			A|splice_donor_variant&intron_variant|HIGH|NDUFV2-AS1|ENSG00
)252680			A|bidirectional_gene_fusion|HIGH|RAB31&RNA5SP449|ENSG00000
)252680			A|bidirectional_gene_fusion|HIGH|RAB31&RNA5SP449|ENSG00000
)252680			A|bidirectional_gene_fusion|HIGH|RAB31&RNA5SP449|ENSG00000
protein_co	6	0.17	G|exon_loss_variant|HIGH|TXNDC2|ENSG00000168454|transcript|
nonsense_	30	0.03	A|splice_acceptor_variant&intron_variant|HIGH|MPPE1|ENSG0000
protein_co	30	0.13	T|splice_acceptor_variant&intron_variant|HIGH|MPPE1|ENSG0000
protein_coding			A|structural_interaction_variant|HIGH|IMPA2|ENSG00000141401|i
processed_transcript			CTTTTTTTT|splice_acceptor_variant&intron_variant|HIGH|RP11-64C
processed_transcript			CTTTTTTTT|splice_acceptor_variant&intron_variant|HIGH|RP11-64C
processed_transcript			CTTTTTTTT|splice_acceptor_variant&intron_variant|HIGH|RP11-64C
processed_transcript			CTTTTTTTT|splice_acceptor_variant&intron_variant|HIGH|RP11-64C
processed_transcript			CTTTTTTTT|splice_acceptor_variant&intron_variant|HIGH|RP11-64C
processed_transcript			CTTTTTTTT|splice_acceptor_variant&intron_variant|HIGH|RP11-64C
transcribed_processed_pseudoge			G|splice_donor_variant&intron_variant|HIGH|RP11-64C12.3|ENSG(
nonsense_	4	0.25	CCCGCGCACACACCCAT|frameshift_variant|HIGH|CIDEA|ENSG00000
processed_	4	0.25	A|splice_donor_variant&intron_variant|HIGH|CIDEA|ENSG0000017
lincRNA			ATTTT|splice_acceptor_variant&intron_variant|HIGH|RP11-861E21.
lincRNA			ATTTT|splice_acceptor_variant&intron_variant|HIGH|RP11-861E21.
lincRNA			ATTTT|splice_acceptor_variant&intron_variant|HIGH|RP11-861E21.
protein_co	10	0.1	A|start_lost|HIGH|PSMG2|ENSG00000128789|transcript|ENST0000
protein_co	19	0.05	T|splice_donor_variant&intron_variant|HIGH|LDLRAD4|ENSG00000
)267356			C|bidirectional_gene_fusion|HIGH|RP11-411B10.3&ZNF519|ENSG0
transcribed_unprocessed_pseudo			A|splice_acceptor_variant&intron_variant|HIGH|ANKRD20A5P|ENS
protein_coding			A|structural_interaction_variant|HIGH|ROCK1|ENSG00000067900|i
protein_co	10	0.1	A|splice_donor_variant&intron_variant|HIGH|CABLES1|ENSG00000
protein_coding			C|splice_donor_variant&splice_region_variant&5_prime_UTR_variar
protein_coding			T|structural_interaction_variant|HIGH|AQP4|ENSG00000171885|in
protein_co	5	0.2	T|stop_gained|HIGH|CDH2|ENSG00000170558|transcript|ENST000
protein_coding			TTC|frameshift_variant|HIGH|DSC2|ENSG00000134755|transcript|
antisense			C|splice_donor_variant&intron_variant|HIGH|DSG2-AS1|ENSG0000
protein_coding			A|structural_interaction_variant|HIGH|MEP1B|ENSG00000141434|
protein_coding			G|structural_interaction_variant|HIGH|MEP1B|ENSG00000141434|
protein_coding			A|structural_interaction_variant|HIGH|MEP1B|ENSG00000141434|
lincRNA			T|splice_acceptor_variant&intron_variant|HIGH|LINC01477|ENSG0
lincRNA			T|splice_acceptor_variant&intron_variant|HIGH|KC6|ENSG0000026
protein_coding			C|structural_interaction_variant|HIGH|PIK3C3|ENSG00000078142|i
protein_co	3	0.33	CTCTT|frameshift_variant|HIGH|SETBP1|ENSG00000152217|transc
protein_co	20	0.1	C|splice_donor_variant&intron_variant|HIGH|ATP5A1|ENSG000001
nonsense_	12	0.08	C|start_lost|HIGH|LOXHD1|ENSG00000167210|transcript|ENST000
protein_co	12	0.17	T|start_lost|HIGH|LOXHD1|ENSG00000167210|transcript|ENST000
protein_co	8	0.25	C|start_lost|HIGH|KATNAL2|ENSG00000167216|transcript|ENST00
protein_co	1	1	TGC|frameshift_variant|HIGH|TCEB3CL2|ENSG00000274744|transc
protein_co	1	1	TGC|frameshift_variant|HIGH|TCEB3CL|ENSG00000275553|transcri
protein_co	8	0.13	CA|frameshift_variant|HIGH|KATNAL2|ENSG00000167216|transcrip
protein_co	8	0.13	CA|frameshift_variant|HIGH|KATNAL2|ENSG00000167216|transcrip

lincRNA			C|splice_donor_variant&intron_variant|HIGH|CTD-2130O13.1|ENSG
protein_co	20	0.05	G|splice_acceptor_variant&intron_variant|HIGH|DYM|ENSG000001
protein_coding			C|frameshift_variant&splice_acceptor_variant&splice_region_varian
)265402			C|bidirectional_gene_fusion|HIGH|LINC01630&RSL24D1P9|ENSG00
)265402			C|bidirectional_gene_fusion|HIGH|LINC01630&RSL24D1P9|ENSG00
)265402			C|bidirectional_gene_fusion|HIGH|LINC01630&RSL24D1P9|ENSG00
)265402			C|bidirectional_gene_fusion|HIGH|LINC01630&RSL24D1P9|ENSG00
)265402			C|bidirectional_gene_fusion|HIGH|LINC01630&RSL24D1P9|ENSG00
)265402			C|bidirectional_gene_fusion|HIGH|LINC01630&RSL24D1P9|ENSG00
lincRNA			T|splice_donor_variant&intron_variant|HIGH|RP11-25O3.1|ENSG0
protein_co	4	0.5	A|stop_gained|HIGH|STARD6|ENSG00000174448|transcript|ENST0
processed_transcript			G|splice_donor_variant&intron_variant|HIGH|WDR7|ENSG0000009
antisense			CTTT|splice_acceptor_variant&intron_variant|HIGH|RP11-35G9.3|E
antisense			CTTT|splice_acceptor_variant&intron_variant|HIGH|RP11-35G9.3|E
antisense			CTTT|splice_acceptor_variant&intron_variant|HIGH|RP11-35G9.3|E
protein_co	5	0.6	ATTT|splice_acceptor_variant&intron_variant|HIGH|MALT1|ENSG0
protein_co	5	0.6	ATTT|splice_acceptor_variant&intron_variant|HIGH|MALT1|ENSG0
protein_co	5	0.6	ATTT|splice_acceptor_variant&intron_variant|HIGH|MALT1|ENSG0
protein_co	5	0.6	ATTT|splice_acceptor_variant&intron_variant|HIGH|MALT1|ENSG0
transcribed_unitary_pseudogene			G|splice_acceptor_variant&intron_variant|HIGH|OACYLP|ENSG0000
lincRNA			C|splice_donor_variant&intron_variant|HIGH|RP11-685A21.1|ENSG
protein_co	5	0.6	T|frameshift_variant|HIGH|GRP|ENSG00000134443|transcript|ENS
protein_coding			C|structural_interaction_variant|HIGH|LMAN1|ENSG00000074695|
transcribed_processed_pseudoge			T|splice_acceptor_variant&intron_variant|HIGH|RP11-824M15.3|EN
lincRNA			T|splice_donor_variant&intron_variant|HIGH|RP11-325K19.1|ENSG
non_stop_	12	0.08	A|frameshift_variant|HIGH|KIAA1468|ENSG00000134444|transcrip
non_stop_	12	0.08	A|frameshift_variant|HIGH|KIAA1468|ENSG00000134444|transcrip
protein_coding			G|structural_interaction_variant|HIGH|SERPINB5|ENSG0000020607
protein_co	9	0.56	T|stop_gained|HIGH|SERPINB11|ENSG00000206072|transcript|ENS
nonsense_mediated_decay			T|stop_gained|HIGH|SERPINB11|ENSG00000206072|transcript|ENS
protein_coding			G|structural_interaction_variant|HIGH|SERPINB2|ENSG0000019763
protein_coding			G|structural_interaction_variant|HIGH|SERPINB2|ENSG0000019763
sense_intronic			GAAGTACCTGGACTTTA|splice_donor_variant&intron_variant|HIGH|
sense_intronic			GAAGTACCTGGACTTTA|splice_donor_variant&intron_variant|HIGH|
sense_intronic			GAAGTACCTGGACTTTA|splice_donor_variant&intron_variant|HIGH|
sense_intronic			A|splice_donor_variant&intron_variant|HIGH|RP11-389J22.1|ENSG
protein_coding			T|frameshift_variant|HIGH|TMX3|ENSG00000166479|transcript|EN
protein_co	12	0.17	A|stop_gained|HIGH|CCDC102B|ENSG00000150636|transcript|ENS
)206052			T|gene_fusion|HIGH|DOK6&RP11-543H23.1|ENSG00000206052&EI
protein_co	29	0.03	T|splice_donor_variant&intron_variant|HIGH|CNDP2|ENSG0000013
protein_coding			T|stop_lost|HIGH|CNDP2|ENSG00000133313|transcript|ENST0000
protein_co	29	0.1	TC|splice_acceptor_variant&intron_variant|HIGH|CNDP2|ENSG0000
protein_coding			T|structural_interaction_variant|HIGH|ZADH2|ENSG00000180011|i
protein_coding			A|structural_interaction_variant|HIGH|ZADH2|ENSG00000180011|i
lincRNA			T|splice_donor_variant&intron_variant|HIGH|RP11-704C2.1|ENSG0
lincRNA			T|splice_donor_variant&intron_variant|HIGH|RP11-1O2.1|ENSG000
protein_coding			G|stop_lost|HIGH|MBP|ENSG00000197971|transcript|ENST000003

processed_transcript			A|splice_acceptor_variant&intron_variant|HIGH|MBP|ENSG000001
nonsense_mediated_decay			T|stop_gained|HIGH|ATP9B|ENSG00000166377|transcript|ENST00
protein_co	3	0.67	GT|frameshift_variant|HIGH|OR4F17|ENSG00000176695|transcript
processed_transcript			T|splice_donor_variant&intron_variant|HIGH|MIER2|ENSG0000010
protein_co	3	0.67	CG|frameshift_variant|HIGH|ODF3L2|ENSG00000181781|transcript
protein_co	10	0.1	A|splice_donor_variant&intron_variant|HIGH|MADCAM1|ENSG000
protein_co	10	0.7	TCCCAACACCACCTC|frameshift_variant|HIGH|MADCAM1|ENSG000
protein_co	10	0.7	GGAGCCTCCCA|frameshift_variant|HIGH|MADCAM1|ENSG0000009
protein_co	10	0.7	GGAGCCTCCCA|frameshift_variant|HIGH|MADCAM1|ENSG0000009
protein_co	10	0.7	GGAGCCTCCCA|frameshift_variant|HIGH|MADCAM1|ENSG0000009
protein_coding			T|stop_gained|HIGH|GZMM|ENSG00000197540|transcript|ENST00
protein_coding			C|structural_interaction_variant|HIGH|HCN2|ENSG00000099822|in
protein_coding			A|structural_interaction_variant|HIGH|POLRMT|ENSG00000099821
processed_transcript			A|splice_donor_variant&intron_variant|HIGH|PALM|ENSG0000009
protein_coding			T|structural_interaction_variant|HIGH|PTBP1|ENSG00000011304|i
protein_co	15	0.07	C|frameshift_variant|HIGH|MED16|ENSG00000175221|transcript|
protein_co	15	0.07	GGTAGCTGAGGA|frameshift_variant|HIGH|MED16|ENSG00000175
protein_co	2	0.5	C|frameshift_variant|HIGH|GRIN3B|ENSG00000116032|transcript|
protein_co	2	0.5	GCGTT|frameshift_variant|HIGH|GRIN3B|ENSG00000116032|trans
protein_co	19	0.05	G|splice_acceptor_variant&intron_variant|HIGH|ABCA7|ENSG0000
protein_co	19	0.11	C|frameshift_variant|HIGH|ABCA7|ENSG00000064687|transcript|E
protein_co	16	0.06	A|frameshift_variant&splice_region_variant|HIGH|ARHGAP45|ENS
protein_co	5	0.2	G|start_lost|HIGH|CBARP|ENSG00000099625|transcript|ENST0000
protein_co	46	0.04	A|splice_donor_variant&intron_variant|HIGH|CIRBP|ENSG0000009
protein_coding			A|structural_interaction_variant|HIGH|GAMT|ENSG00000130005|i
protein_co	13	0.08	T|frameshift_variant|HIGH|C19orf25|ENSG00000119559|transcript
processed_transcript			C|splice_donor_variant&intron_variant|HIGH|PCSK4|ENSG0000011
protein_co	4	0.5	C|splice_donor_variant&intron_variant|HIGH|PLK5|ENSG0000018
protein_coding			C|stop_lost|HIGH|ATP8B3|ENSG00000130270|transcript|ENST0000
protein_co	8	0.13	CA|frameshift_variant|HIGH|ATP8B3|ENSG00000130270|transcript
protein_coding			T|structural_interaction_variant|HIGH|CSNK1G2|ENSG0000013327
antisense			A|splice_donor_variant&intron_variant|HIGH|AC005306.3|ENSG000
protein_co	11	0.09	GT|frameshift_variant|HIGH|BTBD2|ENSG00000133243|transcript|
protein_co	3	0.33	G|start_lost|HIGH|JSRP1|ENSG00000167476|transcript|ENST0000
protein_co	14	0.07	ATTT|splice_acceptor_variant&intron_variant|HIGH|SPPL2B|ENSG0
protein_co	14	0.07	ATTT|splice_acceptor_variant&intron_variant|HIGH|SPPL2B|ENSG0
protein_co	14	0.07	ATTT|splice_acceptor_variant&intron_variant|HIGH|SPPL2B|ENSG0
protein_co	14	0.07	ATTT|splice_acceptor_variant&intron_variant|HIGH|SPPL2B|ENSG0
protein_co	14	0.07	ATTT|splice_acceptor_variant&intron_variant|HIGH|SPPL2B|ENSG0
protein_co	5	0.2	A|splice_donor_variant&intron_variant|HIGH|ZNF57|ENSG0000017
protein_coding			A|stop_gained|HIGH|ZNF77|ENSG00000175691|transcript|ENST00
protein_co	22	0.05	T|splice_donor_variant&intron_variant|HIGH|TLE2|ENSG0000065
nonsense_mediated_decay			C|stop_lost|HIGH|ZFR2|ENSG00000105278|transcript|ENST000005
nonsense_mediated_decay			C|stop_lost|HIGH|ANKRD24|ENSG00000089847|transcript|ENST00
protein_co	10	0.3	C|stop_gained|HIGH|STAP2|ENSG00000178078|transcript|ENST00
protein_coding			CGGGGCGCGGGGCTGCA|frameshift_variant|HIGH|MPND|ENSG000
protein_coding			CGGGGCGCGGGGCTGCA|frameshift_variant|HIGH|MPND|ENSG000

lincRNA			T|splice_acceptor_variant&intron_variant|HIGH|CTB-50L17.16|ENS
protein_co	2	1	C|frameshift_variant|HIGH|PLIN4|ENSG00000167676|transcript|EN
protein_co	2	1	GAC|frameshift_variant|HIGH|PLIN4|ENSG00000167676|transcript
protein_coding			T|stop_gained|HIGH|MYDGF|ENSG00000074842|transcript|ENST0
processed_	25	0.04	T|splice_donor_variant&intron_variant|HIGH|DPP9|ENSG00000142
protein_coding			G|structural_interaction_variant|HIGH|UHRF1|ENSG00000276043|
protein_coding			C|structural_interaction_variant|HIGH|UHRF1|ENSG00000276043|i
protein_coding			T|structural_interaction_variant|HIGH|UHRF1|ENSG00000276043|i
protein_coding			A|structural_interaction_variant|HIGH|PTPRS|ENSG00000105426|in
protein_coding			A|structural_interaction_variant|HIGH|PTPRS|ENSG00000105426|in
protein_coding			G|structural_interaction_variant|HIGH|PTPRS|ENSG00000105426|i
protein_coding			T|structural_interaction_variant|HIGH|LONP1|ENSG00000196365|i
protein_coding			T|structural_interaction_variant|HIGH|LONP1|ENSG00000196365|i
protein_coding			G|stop_lost|HIGH|PSPN|ENSG00000125650|transcript|ENST00000
retained_intron			T|splice_acceptor_variant&intron_variant|HIGH|DENND1C|ENSG00
processed_pseudogene			C|splice_acceptor_variant&intron_variant|HIGH|CTD-2396E7.7|ENS
processed_pseudogene			CTT|splice_region_variant&intron_variant|LOW|CTD-2396E7.7|ENS
processed_pseudogene			CT|splice_acceptor_variant&intron_variant|HIGH|CLIC4P2|ENSG000
protein_coding			A|structural_interaction_variant|HIGH|VAV1|ENSG00000141968|in
protein_co	8	0.13	G|splice_donor_variant&intron_variant|HIGH|ADGRE1|ENSG00000
protein_co	8	0.63	GTT|splice_acceptor_variant&intron_variant|HIGH|ADGRE1|ENSG0
protein_co	8	0.63	GTT|splice_acceptor_variant&intron_variant|HIGH|ADGRE1|ENSG0
protein_co	4	0.5	C|frameshift_variant|HIGH|CAMSAP3|ENSG00000076826|transcrip
protein_coding			T|frameshift_variant|HIGH|STXBP2|ENSG00000076944|transcript|E
protein_co	22	0.05	A|frameshift_variant|HIGH|STXBP2|ENSG00000076944|transcript|
protein_co	22	0.05	A|frameshift_variant|HIGH|STXBP2|ENSG00000076944|transcript|
protein_co	22	0.05	C|frameshift_variant|HIGH|STXBP2|ENSG00000076944|transcript|
protein_co	22	0.05	G|frameshift_variant|HIGH|STXBP2|ENSG00000076944|transcript|
protein_co	22	0.05	C|frameshift_variant|HIGH|STXBP2|ENSG00000076944|transcript|
protein_co	22	0.05	CTG|frameshift_variant&splice_region_variant|HIGH|STXBP2|ENSG
protein_co	22	0.05	A|frameshift_variant&splice_region_variant|HIGH|STXBP2|ENSG000
protein_co	22	0.05	C|frameshift_variant|HIGH|STXBP2|ENSG00000076944|transcript|
protein_co	22	0.05	T|frameshift_variant|HIGH|STXBP2|ENSG00000076944|transcript|E
protein_coding			T|structural_interaction_variant|HIGH|STXBP2|ENSG00000076944|
protein_coding			C|structural_interaction_variant|HIGH|STXBP2|ENSG00000076944|
protein_coding			T|structural_interaction_variant|HIGH|FCER2|ENSG00000104921|in
protein_coding			A|structural_interaction_variant|HIGH|FCER2|ENSG00000104921|in
protein_coding			T|structural_interaction_variant|HIGH|MAP2K7|ENSG00000076984
protein_coding			A|stop_lost&conservative_inframe_deletion|HIGH|SNAPC2|ENSG00
protein_co	5	0.2	C|start_lost&disruptive_inframe_deletion|HIGH|KANK3|ENSG00000
protein_co	4	0.5	C|start_lost|HIGH|PRAM1|ENSG00000133246|transcript|ENST0000
protein_co	5	0.2	C|frameshift_variant|HIGH|MUC16|ENSG00000181143|transcript|
protein_co	5	0.2	A|splice_acceptor_variant&intron_variant|HIGH|MUC16|ENSG0000
protein_co	5	0.2	G|splice_acceptor_variant&intron_variant|HIGH|MUC16|ENSG0000
protein_coding			GATGGT|frameshift_variant|HIGH|OR7G3|ENSG00000170920|trans
protein_co	1	1	A|frameshift_variant|HIGH|OR7G3|ENSG00000170920|transcript|E
protein_co	14	0.07	A|frameshift_variant|HIGH|ZNF559|ENSG00000188321|transcript|

protein_co	7	0.71	C|frameshift_variant|HIGH|ZNF266|ENSG00000174652|transcript|
protein_co	4	0.25	T|frameshift_variant|HIGH|ZNF812P|ENSG00000224689|transcript
protein_co	3	0.33	A|stop_gained|HIGH|RDH8|ENSG00000080511|transcript|ENST000
nonsense_mediated_decay			T|splice_donor_variant&splice_region_variant&3_prime_UTR_variar
protein_coding			G|structural_interaction_variant|HIGH|RAVER1|ENSG00000161847
nonsense_mediated_decay			G|stop_lost|HIGH|RAVER1|ENSG00000161847|transcript|ENST000
protein_coding			A|structural_interaction_variant|HIGH|TYK2|ENSG00000105397|int
protein_coding			T|structural_interaction_variant|HIGH|TYK2|ENSG00000105397|int
protein_coding			T|structural_interaction_variant|HIGH|PDE4A|ENSG00000065989|i
protein_co	10	0.1	C|frameshift_variant|HIGH|AP1M2|ENSG00000129354|transcript|E
protein_co	6	0.17	TG|frameshift_variant|HIGH|TSPAN16|ENSG00000130167|transcrip
protein_coding			G|structural_interaction_variant|HIGH|RAB3D|ENSG00000105514|
nonsense_	20	0.05	GA|frameshift_variant|HIGH|CCDC159|ENSG00000183401|transcri
protein_co	14	0.07	C|stop_gained|HIGH|ECSIT|ENSG00000130159|transcript|ENST000
nonsense_	14	0.07	T|splice_donor_variant&intron_variant|HIGH|ECSIT|ENSG0000013C
protein_co	12	0.08	T|splice_donor_variant&intron_variant|HIGH|ACP5|ENSG00000102
nonsense_	10	0.1	C|frameshift_variant|HIGH|ZNF433|ENSG00000197647|transcript|
antisense			G|splice_acceptor_variant&intron_variant|HIGH|CTD-2006C1.2|ENS
protein_co	3	0.33	A|frameshift_variant|HIGH|ZNF844|ENSG00000223547|transcript|
protein_co	7	0.14	C|splice_donor_variant&intron_variant|HIGH|ZNF788|ENSG000002
protein_coding			A|stop_lost|HIGH|ZNF443|ENSG00000180855|transcript|ENST000
protein_co	2	0.5	CTG|frameshift_variant|HIGH|ZNF443|ENSG00000180855|transcrip
protein_co	2	0.5	C|frameshift_variant|HIGH|ZNF443|ENSG00000180855|transcript|
protein_co	2	0.5	C|frameshift_variant|HIGH|ZNF443|ENSG00000180855|transcript|
unprocessed_pseudogene			C|splice_acceptor_variant&intron_variant|HIGH|MTATP6P27|ENSG
unprocessed_pseudogene			G|splice_donor_variant&intron_variant|HIGH|MTATP6P27|ENSG00
processed_transcript			G|splice_acceptor_variant&intron_variant|HIGH|HOOK2|ENSG0000
protein_coding			A|frameshift_variant|HIGH|STX10|ENSG00000104915|transcript|EI
protein_coding			A|structural_interaction_variant|HIGH|MRI1|ENSG00000037757|in
antisense			A|splice_donor_variant&intron_variant|HIGH|CTB-5E10.3|ENSG000
protein_co	1	1	C|frameshift_variant|HIGH|AC008686.1|ENSG00000280221|transcr
protein_co	1	1	CT|frameshift_variant|HIGH|AC008686.1|ENSG00000280221|trans
protein_co	1	1	CTTT|frameshift_variant|HIGH|AC008686.1|ENSG00000280221|tra
protein_co	1	1	CTTTT|frameshift_variant|HIGH|AC008686.1|ENSG00000280221|tr
protein_co	1	1	CTTTTTT|frameshift_variant|HIGH|AC008686.1|ENSG00000280221|
protein_co	1	1	CTT|conservative_inframe_deletion|MODERATE|AC008686.1|ENSG
protein_co	13	0.08	A|splice_donor_variant&intron_variant|HIGH|PODNL1|ENSG00000
protein_co	3	0.67	GGC|frameshift_variant|HIGH|PALM3|ENSG00000187867|transcrip
protein_co	3	0.33	GCTGT|frameshift_variant|HIGH|SAMD1|ENSG00000141858|transc
protein_co	3	0.33	CGG|frameshift_variant|HIGH|SAMD1|ENSG00000141858|transcrip
protein_co	3	0.33	GCAGC|frameshift_variant|HIGH|SAMD1|ENSG00000141858|trans
protein_co	3	0.33	CCGGGCGGCGACGGCGGGCGGGGGGCGCG|frameshift_variant|HIG
protein_coding			A|structural_interaction_variant|HIGH|PKN1|ENSG00000123143|in
protein_coding			G|structural_interaction_variant|HIGH|PKN1|ENSG00000123143|in
protein_co	15	0.07	CTTT|splice_acceptor_variant&intron_variant|HIGH|PKN1|ENSG000
protein_co	15	0.07	CTTT|splice_acceptor_variant&intron_variant|HIGH|PKN1|ENSG000
protein_co	15	0.07	CTTT|splice_acceptor_variant&intron_variant|HIGH|PKN1|ENSG000

protein_co	26	0.04	A|splice_donor_variant&intron_variant|HIGH|TECR|ENSG00000099
protein_co	13	0.62	T|stop_gained|HIGH|ADGRE2|ENSG00000127507|transcript|ENST0
protein_co	14	0.07	G|splice_donor_variant&intron_variant|HIGH|ILVBL|ENSG0000010
protein_co	4	0.25	G|start_lost|HIGH|PGLYRP2|ENSG00000161031|transcript|ENST00
retained_ir	17	0.18	A|splice_donor_variant&intron_variant|HIGH|CYP4F12|ENSG00000
protein_coding			T|structural_interaction_variant|HIGH|RAB8A|ENSG00000167461|i
protein_coding			T|structural_interaction_variant|HIGH|RAB8A|ENSG00000167461|i
protein_co	15	0.07	TCGCTGCCGCCGCCACCGCCCTCGGC|splice_acceptor_variant&intron_
protein_co	7	0.14	GC|frameshift_variant|HIGH|MED26|ENSG00000105085|transcript
protein_co	10	0.1	C|splice_acceptor_variant&intron_variant|HIGH|USHBP1|ENSG0000
protein_co	9	0.22	GC|frameshift_variant|HIGH|ANKLE1|ENSG00000160117|transcript
protein_coding			G|frameshift_variant|HIGH|ANKLE1|ENSG00000160117|transcript|
protein_coding			G|frameshift_variant|HIGH|ANKLE1|ENSG00000160117|transcript|
protein_coding			G|frameshift_variant|HIGH|ANKLE1|ENSG00000160117|transcript|
protein_coding			G|frameshift_variant|HIGH|ANKLE1|ENSG00000160117|transcript|
protein_coding			GTGTT|frameshift_variant|HIGH|ANKLE1|ENSG00000160117|transc
protein_coding			GTT|frameshift_variant|HIGH|ANKLE1|ENSG00000160117|transcrip
protein_coding			GTTTGTGTGTGTGTGTGTGTGTT|frameshift_variant|HIGH|ANKLE1|E
protein_coding			G|frameshift_variant|HIGH|ANKLE1|ENSG00000160117|transcript|
protein_coding			G|frameshift_variant|HIGH|ANKLE1|ENSG00000160117|transcript|
protein_coding			G|frameshift_variant|HIGH|ANKLE1|ENSG00000160117|transcript|
protein_coding			G|frameshift_variant|HIGH|ANKLE1|ENSG00000160117|transcript|
protein_coding			G|frameshift_variant|HIGH|ANKLE1|ENSG00000160117|transcript|
protein_coding			G|frameshift_variant|HIGH|ANKLE1|ENSG00000160117|transcript|
protein_coding			G|frameshift_variant|HIGH|ANKLE1|ENSG00000160117|transcript|
protein_coding			G|frameshift_variant|HIGH|ANKLE1|ENSG00000160117|transcript|
protein_coding			G|frameshift_variant|HIGH|ANKLE1|ENSG00000160117|transcript|
protein_coding			G|frameshift_variant|HIGH|ANKLE1|ENSG00000160117|transcript|
protein_coding			G|frameshift_variant|HIGH|ANKLE1|ENSG00000160117|transcript|
protein_coding			G|frameshift_variant|HIGH|ANKLE1|ENSG00000160117|transcript|
protein_coding			TTG|frameshift_variant&stop_lost|HIGH|ANKLE1|ENSG0000016011
protein_coding			TTG|frameshift_variant&stop_lost|HIGH|ANKLE1|ENSG0000016011
protein_coding			TTG|frameshift_variant&stop_lost|HIGH|ANKLE1|ENSG0000016011
protein_coding			A|frameshift_variant|HIGH|MAP1S|ENSG00000130479|transcript|E
processed_transcript			G|splice_donor_variant&intron_variant|HIGH|MAP1S|ENSG000001
protein_coding			C|frameshift_variant|HIGH|KCNN1|ENSG00000105642|transcript|E
protein_co	7	0.43	A|splice_acceptor_variant&intron_variant|HIGH|IL12RB1|ENSG0000
protein_coding			T|structural_interaction_variant|HIGH|MAST3|ENSG00000099308|i
protein_coding			C|structural_interaction_variant|HIGH|PDE4C|ENSG00000105650|i
protein_coding			T|structural_interaction_variant|HIGH|PDE4C|ENSG00000105650|i
nonsense_	17	0.06	A|frameshift_variant|HIGH|PDE4C|ENSG00000105650|transcript|E
protein_co	6	0.17	C|start_lost|HIGH|LSM4|ENSG00000130520|transcript|ENST00000
protein_co	15	0.07	GC|frameshift_variant|HIGH|SSBP4|ENSG00000130511|transcript|
protein_co	5	0.4	C|frameshift_variant|HIGH|TMEM59L|ENSG00000105696|transcrip
protein_co	12	0.75	C|splice_donor_variant&splice_region_variant&intron_variant|HIGH
protein_coding			T|stop_gained|HIGH|ARMC6|ENSG00000105676|transcript|ENST00
protein_co	8	0.5	C|frameshift_variant|HIGH|ZNF101|ENSG00000181896|transcript|

nonsense_	7	0.14	ATTT|splice_acceptor_variant&intron_variant|HIGH|ZNF93|ENSG00
nonsense_	7	0.14	ATTT|splice_acceptor_variant&intron_variant|HIGH|ZNF93|ENSG00
nonsense_	7	0.14	ATTT|splice_acceptor_variant&intron_variant|HIGH|ZNF93|ENSG00
nonsense_mediated_decay			G|stop_lost|HIGH|ZNF93|ENSG00000184635|transcript|ENST0000(
protein_co	4	0.5	G|frameshift_variant|HIGH|ZNF626|ENSG00000188171|transcript|
protein_co	4	0.25	GA|frameshift_variant|HIGH|ZNF626|ENSG00000188171|transcript
protein_co	4	0.25	GA|frameshift_variant|HIGH|ZNF626|ENSG00000188171|transcript
protein_co	3	0.33	CT|frameshift_variant|HIGH|ZNF66|ENSG00000160229|transcript|
protein_co	3	0.33	CT|frameshift_variant|HIGH|ZNF66|ENSG00000160229|transcript|
protein_co	3	0.33	T|frameshift_variant|HIGH|ZNF66|ENSG00000160229|transcript|E
protein_co	3	0.33	CTT|frameshift_variant|HIGH|ZNF66|ENSG00000160229|transcript
protein_co	3	0.33	CTT|frameshift_variant|HIGH|ZNF66|ENSG00000160229|transcript
protein_co	3	0.33	CTT|frameshift_variant|HIGH|ZNF66|ENSG00000160229|transcript
nonsense_mediated_decay			A|stop_gained&splice_region_variant|HIGH|ZNF714|ENSG0000016
protein_co	10	0.1	C|frameshift_variant|HIGH|ZNF714|ENSG00000160352|transcript|
protein_co	10	0.1	C|frameshift_variant|HIGH|ZNF714|ENSG00000160352|transcript|
protein_co	9	0.22	C|frameshift_variant|HIGH|ZNF429|ENSG00000197013|transcript|
protein_coding			A|stop_gained|HIGH|ZNF43|ENSG00000198521|transcript|ENST00
protein_coding			T|stop_gained|HIGH|ZNF208|ENSG00000160321|transcript|ENST0
protein_coding			A|stop_gained|HIGH|ZNF98|ENSG00000197360|transcript|ENST00
processed_transcript			T|splice_acceptor_variant&intron_variant|HIGH|GOLGA2P9|ENSG0
protein_co	1	1	G|splice_donor_variant&intron_variant|HIGH|ZNF492|ENSG000002
protein_co	1	1	T|splice_acceptor_variant&intron_variant|HIGH|ZNF492|ENSG0000
protein_coding			G|stop_lost&splice_region_variant|HIGH|ZNF99|ENSG0000021397
protein_coding			T|frameshift_variant|HIGH|ZNF99|ENSG00000213973|transcript|E
protein_co	2	0.5	G|frameshift_variant|HIGH|ZNF99|ENSG00000213973|transcript|E
protein_co	2	0.5	G|frameshift_variant|HIGH|ZNF99|ENSG00000213973|transcript|E
protein_coding			AG|frameshift_variant|HIGH|ZNF99|ENSG00000213973|transcript|
protein_co	3	0.67	C|frameshift_variant|HIGH|ZNF681|ENSG00000196172|transcript|
lincRNA			G|splice_donor_variant&intron_variant|HIGH|AC079466.1|ENSG00
lincRNA			C|splice_donor_variant&intron_variant|HIGH|AC007795.1|ENSG00
lincRNA			A|splice_acceptor_variant&intron_variant|HIGH|CTC-525D6.1|ENSG
antisense			GT|splice_acceptor_variant&intron_variant|HIGH|AC007796.1|ENS
lincRNA			C|splice_donor_variant&intron_variant|HIGH|AC011525.4|ENSG00
protein_coding			A|structural_interaction_variant|HIGH|ANKRD27|ENSG0000010518
protein_coding			T|structural_interaction_variant|HIGH|ANKRD27|ENSG0000010518
nonsense_	4	0.25	A|splice_acceptor_variant&splice_region_variant&intron_variant|HI
protein_coding			G|stop_lost|HIGH|CEP89|ENSG00000121289|transcript|ENST0000(
protein_coding			T|protein_protein_contact|HIGH|FAAP24|ENSG00000131944|inter
processed_pseudogene			C|splice_acceptor_variant&intron_variant|HIGH|RPS26P55|ENSG00
processed_transcript			TATATAC|splice_donor_variant&intron_variant|HIGH|SCGB2B2|ENS
processed_transcript			TATATAC|splice_donor_variant&intron_variant|HIGH|SCGB2B2|ENS
processed_transcript			TATATAC|splice_donor_variant&intron_variant|HIGH|SCGB2B2|ENS
processed_transcript			TATATAC|splice_donor_variant&intron_variant|HIGH|SCGB2B2|ENS
processed_transcript			TATATAC|splice_donor_variant&intron_variant|HIGH|SCGB2B2|ENS
protein_co	15	0.07	T|frameshift_variant&splice_acceptor_variant&splice_region_varian
protein_co	15	0.07	T|frameshift_variant&splice_acceptor_variant&splice_region_varian

protein_co	15	0.07	T|frameshift_variant&splice_acceptor_variant&splice_region_varian
protein_co	15	0.07	T|frameshift_variant&splice_acceptor_variant&splice_region_varian
protein_co	15	0.07	T|frameshift_variant&splice_acceptor_variant&splice_region_varian
protein_coding			A|stop_gained|HIGH|ZNF599|ENSG00000153896|transcript|ENST0
protein_co	4	0.25	T|frameshift_variant|HIGH|ZNF599|ENSG00000153896|transcript|
protein_coding			T|structural_interaction_variant|HIGH|HPN|ENSG00000105707|int
)227392			A|bidirectional_gene_fusion|HIGH|CTD-2527I21.15&HPN-AS1|ENSG
)227392			A|bidirectional_gene_fusion|HIGH|CTD-2527I21.15&HPN-AS1|ENSG
protein_coding			T|stop_gained|HIGH|FAM187B|ENSG00000177558|transcript|ENST
protein_co	1	1	T|stop_gained|HIGH|FAM187B|ENSG00000177558|transcript|ENST
protein_co	16	0.06	T|frameshift_variant|HIGH|USF2|ENSG00000105698|transcript|EN
protein_coding			T|stop_gained|HIGH|CD22|ENSG00000012124|transcript|ENST000
lincRNA			C|splice_acceptor_variant&intron_variant|HIGH|LINC01531|ENSG0
protein_co	68	0.07	CTG|frameshift_variant|HIGH|DMKN|ENSG00000161249|transcript
protein_co	68	0.07	CCACTGCTGCT|frameshift_variant|HIGH|DMKN|ENSG00000161249
protein_co	8	0.13	CT|frameshift_variant|HIGH|ETV2|ENSG00000105672|transcript|E
protein_co	5	0.2	TC|splice_acceptor_variant&intron_variant|HIGH|KMT2B|ENSG000
nonsense_	13	0.08	C|splice_donor_variant&intron_variant|HIGH|ARHGAP33|ENSG000
processed_transcript			T|splice_donor_variant&intron_variant|HIGH|LINC01529|ENSG000
processed_transcript			G|splice_donor_variant&intron_variant|HIGH|LINC01529|ENSG000
protein_co	6	0.17	G|splice_acceptor_variant&intron_variant|HIGH|CLIP3|ENSG00000
protein_coding			T|structural_interaction_variant|HIGH|CAPNS1|ENSG00000126247
protein_co	4	0.5	C|splice_donor_variant&intron_variant|HIGH|COX7A1|ENSG000001
antisense			CTTT|splice_acceptor_variant&intron_variant|HIGH|ZNF790-AS1|E
antisense			CTTT|splice_acceptor_variant&intron_variant|HIGH|ZNF790-AS1|E
antisense			CTTT|splice_acceptor_variant&intron_variant|HIGH|ZNF790-AS1|E
protein_co	16	0.06	C|splice_acceptor_variant&splice_donor_variant&intron_variant|HI
protein_coding			C|stop_lost|HIGH|ZNF568|ENSG00000198453|transcript|ENST0000
nonsense_	3	0.33,(ZNF5	A|splice_acceptor_variant&intron_variant|HIGH|ZNF585B|ENSG000
retained_ir	8	0.13	GT|splice_acceptor_variant&intron_variant|HIGH|ZNF383|ENSG000
protein_co	27	0.07	CTT|splice_acceptor_variant&intron_variant|HIGH|HKR1|ENSG0000
protein_co	27	0.07	CTT|splice_acceptor_variant&intron_variant|HIGH|HKR1|ENSG0000
protein_co	7	0.14	CTGTG|frameshift_variant|HIGH|ZNF527|ENSG00000189164|trans
protein_co	7	0.14	A|frameshift_variant|HIGH|ZNF527|ENSG00000189164|transcript|
antisense			ATT|splice_acceptor_variant&intron_variant|HIGH|ZNF571-AS1|EN
antisense			ATT|splice_acceptor_variant&intron_variant|HIGH|ZNF571-AS1|EN
protein_coding			A|splice_acceptor_variant&splice_region_variant&3_prime_UTR_va
protein_coding			A|splice_acceptor_variant&splice_region_variant&3_prime_UTR_va
protein_coding			A|splice_acceptor_variant&splice_region_variant&3_prime_UTR_va
protein_coding			A|splice_acceptor_variant&splice_region_variant&3_prime_UTR_va
protein_coding			A|stop_gained|HIGH|ZNF571|ENSG00000180479|transcript|ENST0
protein_coding			A|stop_gained|HIGH|GGN|ENSG00000179168|transcript|ENST0000
protein_coding			A|stop_gained|HIGH|GGN|ENSG00000179168|transcript|ENST0000
protein_coding			T|structural_interaction_variant|HIGH|ACTN4|ENSG00000130402|i
nonsense_mediated_decay			G|stop_lost|HIGH|CAPN12|ENSG00000182472|transcript|ENST000
protein_coding			T|structural_interaction_variant|HIGH|SIRT2|ENSG00000068903|in
protein_co	12	0.08	C|splice_donor_variant&intron_variant|HIGH|PAK4|ENSG00000130

polymorph	4	1	C|frameshift_variant|HIGH|IFNL4|ENSG00000272395|transcript|EN
protein_coding			A|structural_interaction_variant|HIGH|SUPT5H|ENSG00000196235
protein_coding			G|structural_interaction_variant|HIGH|CLC|ENSG00000105205|inte
protein_co	3	0.67	C|frameshift_variant|HIGH|FCGBP|ENSG00000275395|transcript|E
protein_co	3	0.67	C|frameshift_variant|HIGH|FCGBP|ENSG00000275395|transcript|E
protein_co	3	0.67	AGG|frameshift_variant|HIGH|FCGBP|ENSG00000275395|transcrip
protein_co	3	0.33	A|frameshift_variant|HIGH|FCGBP|ENSG00000275395|transcript|E
protein_co	3	0.33	TGC|frameshift_variant|HIGH|FCGBP|ENSG00000275395|transcript
protein_co	3	0.33	A|frameshift_variant|HIGH|FCGBP|ENSG00000275395|transcript|E
protein_co	3	0.33	CAGGGG|frameshift_variant|HIGH|FCGBP|ENSG00000275395|trans
protein_co	3	0.33	AGG|frameshift_variant|HIGH|FCGBP|ENSG00000275395|transcrip
protein_co	3	0.33	G|frameshift_variant|HIGH|FCGBP|ENSG00000275395|transcript|E
protein_co	7	0.86	C|splice_acceptor_variant&intron_variant|HIGH|ZNF780B|ENSG000
protein_coding			A|structural_interaction_variant|HIGH|BLVRB|ENSG00000090013|i
protein_coding			A|structural_interaction_variant|HIGH|BLVRB|ENSG00000090013|i
protein_coding			C|structural_interaction_variant|HIGH|SHKBP1|ENSG00000160410|
protein_coding			G|structural_interaction_variant|HIGH|SNRPA|ENSG00000077312|i
protein_coding			T|stop_gained|HIGH|CYP2A7|ENSG00000198077|transcript|ENST0
protein_coding			T|structural_interaction_variant|HIGH|CYP2B6|ENSG00000197408|
protein_coding			T|structural_interaction_variant|HIGH|CYP2B6|ENSG00000197408|
protein_coding			T|structural_interaction_variant|HIGH|CYP2A13|ENSG0000019783
protein_coding			C|structural_interaction_variant|HIGH|CYP2A13|ENSG0000019783
protein_coding			T|structural_interaction_variant|HIGH|CYP2A13|ENSG0000019783
protein_coding			GC|frameshift_variant|HIGH|CYP2F1|ENSG00000197446|transcript
protein_co	14	0.07	G|frameshift_variant|HIGH|TMEM91|ENSG00000142046|transcript
protein_coding			A|frameshift_variant|HIGH|TMEM91|ENSG00000142046|transcript
protein_co	8	0.13	GC|frameshift_variant|HIGH|BCKDHA|ENSG00000248098|transcrip
protein_coding			CT|frameshift_variant&stop_lost|HIGH|TMEM145|ENSG000001676
protein_coding			CT|frameshift_variant&stop_lost|HIGH|TMEM145|ENSG000001676
protein_coding			CT|frameshift_variant&stop_lost|HIGH|TMEM145|ENSG000001676
protein_co	7	0.43	C|splice_acceptor_variant&splice_region_variant&intron_variant|HI
unprocessed_pseudogene			G|splice_donor_variant&intron_variant|HIGH|CEACAMP1|ENSG000
protein_co	9	0.33	A|frameshift_variant|HIGH|PSG8|ENSG00000124467|transcript|EN
protein_co	11	0.55	A|stop_gained|HIGH|PSG1|ENSG00000231924|transcript|ENST000
protein_co	7	0.43	A|stop_gained|HIGH|PSG7|ENSG00000221878|transcript|ENST000
nonsense_	13	0.08	T|splice_acceptor_variant&intron_variant|HIGH|PSG11|ENSG00000
protein_co	17	0.06	T|frameshift_variant|HIGH|PSG4|ENSG00000243137|transcript|EN
protein_coding			C|stop_gained|HIGH|PSG9|ENSG00000183668|transcript|ENST000
protein_co	9	0.89	T|splice_acceptor_variant&intron_variant|HIGH|PSG9|ENSG000001
nonsense_	16	0.06	C|frameshift_variant|HIGH|PLAUR|ENSG00000011422|transcript|E
nonsense_	16	0.06	C|frameshift_variant|HIGH|PLAUR|ENSG00000011422|transcript|E
protein_co	9	0.11	G|start_lost&conservative_inframe_deletion|HIGH|ZNF283|ENSG00
protein_coding			C|frameshift_variant|HIGH|ZNF233|ENSG00000159915|transcript|
protein_coding			C|structural_interaction_variant|HIGH|CBLC|ENSG00000142273|int
protein_co	2	1	C|splice_acceptor_variant&intron_variant|HIGH|CBLC|ENSG000001
protein_coding			G|structural_interaction_variant|HIGH|ERCC1|ENSG00000012061|i
protein_coding			T|stop_gained|HIGH|EML2|ENSG00000125746|transcript|ENST000

antisense			T|splice_donor_variant&intron_variant|HIGH|EML2-AS1|ENSG0000
protein_co	4	0.25	T|splice_acceptor_variant&intron_variant|HIGH|SIX5|ENSG0000017
protein_co	20	0.05	C|splice_acceptor_variant&splice_donor_variant&splice_region_vari
protein_co	20	0.05	C|splice_acceptor_variant&splice_donor_variant&splice_region_vari
protein_co	20	0.05	C|splice_acceptor_variant&splice_donor_variant&splice_region_vari
protein_co	20	0.05	C|splice_acceptor_variant&splice_donor_variant&splice_region_vari
protein_co	20	0.05	C|splice_acceptor_variant&splice_donor_variant&splice_region_vari
protein_co	20	0.05	C|splice_acceptor_variant&splice_donor_variant&splice_region_vari
lincRNA			CT|splice_acceptor_variant&intron_variant|HIGH|CTC-344H19.4|EN
lincRNA			CT|splice_acceptor_variant&intron_variant|HIGH|CTC-344H19.4|EN
protein_co	4	0.25	TA|splice_donor_variant&intron_variant|HIGH|IGFL4|ENSG0000020
lincRNA			C|splice_donor_variant&intron_variant|HIGH|AC006262.4|ENSG000
antisense			T|splice_acceptor_variant&intron_variant|HIGH|AC006262.10|ENSG
protein_co	19	0.05	A|stop_gained|HIGH|HIF3A|ENSG00000124440|transcript|ENST000
nonsense_	19	0.05	G|splice_acceptor_variant&intron_variant|HIGH|HIF3A|ENSG00000
protein_coding			T|structural_interaction_variant|HIGH|PPP5C|ENSG00000011485|i
protein_coding			G|protein_protein_contact|HIGH|CCDC8|ENSG00000169515|intera
protein_co	5	0.2	C|start_lost|HIGH|PPP5D1|ENSG00000230510|transcript|ENST000
protein_co	24	0.04	A|splice_donor_variant&intron_variant|HIGH|STRN4|ENSG0000009
lincRNA			T|splice_donor_variant&intron_variant|HIGH|CTD-2571L23.8|ENSG
processed_transcript			G|splice_donor_variant&intron_variant|HIGH|GLTSCR2|ENSG00000
protein_co	12	0.08	T|stop_gained&splice_region_variant|HIGH|GLTSCR2|ENSG0000010
protein_coding			TA|frameshift_variant|HIGH|GLTSCR2|ENSG00000105373|transcrip
protein_co	24	0.21	T|frameshift_variant|HIGH|PLA2G4C|ENSG00000105499|transcript
protein_coding			A|structural_interaction_variant|HIGH|LIG1|ENSG00000105486|int
protein_coding			T|structural_interaction_variant|HIGH|LIG1|ENSG00000105486|int
protein_co	31	0.29	CTT|frameshift_variant|HIGH|CARD8|ENSG00000105483|transcript
protein_co	4	0.75	C|start_lost|HIGH|SYNGR4|ENSG00000105467|transcript|ENST000
protein_co	13	0.08	T|frameshift_variant&splice_region_variant|HIGH|CYTH2|ENSG0000
protein_coding			C|stop_lost|HIGH|CA11|ENSG00000063180|transcript|ENST000005
protein_coding			A|stop_gained|HIGH|FUT2|ENSG00000176920|transcript|ENST000
protein_coding			A|structural_interaction_variant|HIGH|HSD17B14|ENSG000000870
protein_co	4	0.25	G|splice_acceptor_variant&intron_variant|HIGH|HSD17B14|ENSG00
protein_coding			C|structural_interaction_variant|HIGH|HSD17B14|ENSG000000870
protein_co	2	0.5	A|frameshift_variant|HIGH|PPP1R15A|ENSG00000087074|transcrip
protein_co	2	0.5	A|frameshift_variant|HIGH|PPP1R15A|ENSG00000087074|transcrip
protein_co	2	0.5	A|frameshift_variant|HIGH|PPP1R15A|ENSG00000087074|transcrip
nonsense_mediated_decay			T|stop_gained|HIGH|DHDH|ENSG00000104808|transcript|ENST000
protein_co	4	0.75	TG|frameshift_variant|HIGH|DHDH|ENSG00000104808|transcript|
protein_co	4	0.5	T|stop_gained|HIGH|DHDH|ENSG00000104808|transcript|ENST000
protein_co	4	0.5	G|frameshift_variant|HIGH|DHDH|ENSG00000104808|transcript|E
protein_co	3	0.33	A|splice_donor_variant&intron_variant|HIGH|CGB7|ENSG0000019
processed_transcript			T|splice_donor_variant&intron_variant|HIGH|CTB-60B18.22|ENSG0
nonsense_	7	0.14	A|frameshift_variant|HIGH|LIN7B|ENSG00000104863|transcript|E
nonsense_	7	0.14	CA|frameshift_variant|HIGH|LIN7B|ENSG00000104863|transcript|
nonsense_	7	0.14	CA|frameshift_variant|HIGH|LIN7B|ENSG00000104863|transcript|
nonsense_	7	0.14	CA|frameshift_variant|HIGH|LIN7B|ENSG00000104863|transcript|

nonsense_	7	0.14	A|frameshift_variant|HIGH|LIN7B|ENSG00000104863|transcript|EN
nonsense_	7	0.14	A|frameshift_variant|HIGH|LIN7B|ENSG00000104863|transcript|EN
nonsense_	7	0.14	A|frameshift_variant|HIGH|LIN7B|ENSG00000104863|transcript|EN
nonsense_	7	0.14	A|frameshift_variant|HIGH|LIN7B|ENSG00000104863|transcript|EN
nonsense_	7	0.14	CAA|splice_donor_variant&intron_variant|HIGH|LIN7B|ENSG00000
nonsense_	7	0.14	CAA|splice_donor_variant&intron_variant|HIGH|LIN7B|ENSG00000
protein_co	2	1	AAC|frameshift_variant|HIGH|GFY|ENSG00000261949|transcript|E
protein_co	2	1	G|frameshift_variant|HIGH|GFY|ENSG00000261949|transcript|ENS
processed_transcript			T|splice_acceptor_variant&intron_variant|HIGH|RPL13A|ENSG0000
protein_co	31	0.06	C|splice_donor_variant&intron_variant|HIGH|IRF3|ENSG00000126
protein_co	15	0.47	C|frameshift_variant|HIGH|MED25|ENSG00000104973|transcript|E
protein_co	30	0.27	G|splice_donor_variant&intron_variant|HIGH|PNKP|ENSG0000003
nonsense_	30	0.07	C|splice_acceptor_variant&intron_variant|HIGH|PNKP|ENSG000000
protein_co	9	0.22	T|splice_donor_variant&intron_variant|HIGH|ZNF473|ENSG000001
protein_co	14	0.14	ATT|splice_acceptor_variant&intron_variant|HIGH|POLD1|ENSG000
protein_co	14	0.14	ATT|splice_acceptor_variant&intron_variant|HIGH|POLD1|ENSG000
protein_co	8	0.13	TG|frameshift_variant|HIGH|EMC10|ENSG00000161671|transcript
protein_coding			T|stop_gained|HIGH|CLEC11A|ENSG00000105472|transcript|ENST0
protein_co	16	0.06	C|splice_donor_variant&intron_variant|HIGH|KLK3|ENSG00000142
antisense			A|splice_acceptor_variant&intron_variant|HIGH|CTB-147C22.8|ENS
protein_co	7	0.43	G|splice_donor_variant&intron_variant|HIGH|KLK12|ENSG0000018
protein_co	2	1	T|splice_donor_variant&intron_variant|HIGH|KLK14|ENSG0000012
protein_co	7	0.57	C|frameshift_variant|HIGH|CD33|ENSG00000105383|transcript|EN
protein_coding			TG|frameshift_variant&splice_region_variant|HIGH|VSIG10L|ENSG0
protein_co	5	0.2	TG|frameshift_variant|HIGH|ETFB|ENSG00000105379|transcript|E
protein_co	11	0.09	T|splice_acceptor_variant&intron_variant|HIGH|SIGLEC10|ENSG000
protein_co	3	0.67	GC|frameshift_variant|HIGH|SIGLEC12|ENSG00000254521|transcri
protein_co	3	0.67	A|stop_gained|HIGH|SIGLEC12|ENSG00000254521|transcript|ENST
protein_co	2	1.00,(SIGLE	AG|frameshift_variant|HIGH|AC018755.18|ENSG00000268500|tran
protein_co	5	0.2	G|start_lost|HIGH|HAS1|ENSG00000105509|transcript|ENST00000
lincRNA			C|splice_acceptor_variant&intron_variant|HIGH|AC006272.2|ENSG
protein_coding			T|structural_interaction_variant|HIGH|PPP2R1A|ENSG0000010556
protein_coding			T|protein_protein_contact|HIGH|PPP2R1A|ENSG00000105568|inte
antisense			A|splice_donor_variant&splice_region_variant&intron_variant&non_
protein_coding			C|frameshift_variant|HIGH|ZNF480|ENSG00000198464|transcript|
protein_co	6	0.17	T|frameshift_variant|HIGH|ZNF880|ENSG00000221923|transcript|
protein_coding			A|stop_gained|HIGH|ZNF880|ENSG00000221923|transcript|ENST0
protein_co	6	0.17	ATG|frameshift_variant|HIGH|ZNF880|ENSG00000221923|transcri
protein_co	6	0.17	ATG|frameshift_variant|HIGH|ZNF880|ENSG00000221923|transcri
protein_co	6	0.17	ATG|frameshift_variant|HIGH|ZNF880|ENSG00000221923|transcri
protein_co	12	0.42	CTT|splice_acceptor_variant&intron_variant|HIGH|ZNF528|ENSG00
protein_co	12	0.42	CTT|splice_acceptor_variant&intron_variant|HIGH|ZNF528|ENSG00
processed_transcript			T|splice_donor_variant&intron_variant|HIGH|ZNF578|ENSG000002
nonsense_	7	0.14	CA|frameshift_variant|HIGH|ZNF468|ENSG00000204604|transcript
protein_co	16	0.25	T|splice_donor_variant&intron_variant|HIGH|ZNF160|ENSG000001
protein_co	26	0.08	T|stop_gained|HIGH|ZNF415|ENSG00000170954|transcript|ENST0
processed_transcript			A|splice_acceptor_variant&intron_variant|HIGH|CTD-2245F17.3|EN

protein_co	3	0.33	C|frameshift_variant|HIGH|ZNF813|ENSG00000198346|transcript|
protein_co	8	0.63	CGT|frameshift_variant|HIGH|LILRA2|ENSG00000239998|transcript
protein_co	8	0.63	T|frameshift_variant|HIGH|LILRA2|ENSG00000239998|transcript|E
protein_coding			A|protein_protein_contact|HIGH|TNNI3|ENSG00000129991|intera
protein_co	17	0.06	G|start_lost|HIGH|DNAAF3|ENSG00000167646|transcript|ENST000
protein_co	8	0.38	G|frameshift_variant|HIGH|BRSK1|ENSG00000160469|transcript|E
protein_coding			T|protein_protein_contact|HIGH|U2AF2|ENSG00000063244|intera
protein_co	6	0.17	T|splice_donor_variant&intron_variant|HIGH|NLRP11|ENSG000001
protein_co	2	1	T|frameshift_variant|HIGH|NLRP13|ENSG00000173572|transcript|
protein_coding			C|stop_lost&splice_region_variant|HIGH|NLRP8|ENSG0000017970
lincRNA			G|splice_acceptor_variant&intron_variant|HIGH|ZNF667-AS1|ENSG
antisense			A|splice_donor_variant&intron_variant|HIGH|ZIM2-AS1|ENSG0000
protein_coding			A|stop_gained|HIGH|USP29|ENSG00000131864|transcript|ENST00
protein_coding			A|stop_gained|HIGH|ZIM3|ENSG00000141946|transcript|ENST000
protein_co	8	0.13	T|splice_donor_variant&intron_variant|HIGH|AURKC|ENSG0000010
nonsense_	4	0.25	GT|splice_acceptor_variant&intron_variant|HIGH|ZNF547|ENSG000
protein_co	1	1	A|splice_acceptor_variant&intron_variant|HIGH|AC004076.7|ENSG
protein_coding			CA|frameshift_variant|HIGH|ZNF749|ENSG00000186230|transcript
protein_co	9	0.44,(AC00	T|stop_gained|HIGH|ZNF772|ENSG00000197128|transcript|ENST0
nonsense_mediated_decay			A|stop_gained|HIGH|ZNF772|ENSG00000197128|transcript|ENST0
protein_co	18	0.28	A|splice_donor_variant&intron_variant|HIGH|ZNF419|ENSG000001
protein_coding			A|stop_gained|HIGH|ZNF773|ENSG00000152439|transcript|ENST0
protein_co	12	0.5	G|start_lost|HIGH|ZNF211|ENSG00000121417|transcript|ENST000
protein_co	12	0.25	T|frameshift_variant|HIGH|ZNF211|ENSG00000121417|transcript|
protein_coding			A|stop_gained|HIGH|ZNF154|ENSG00000179909|transcript|ENST0
protein_co	6	0.33	T|splice_donor_variant&intron_variant|HIGH|ZNF586|ENSG000000
nonsense_	1	1.00,(ZNF4	TG|frameshift_variant|HIGH|CTD-2583A14.9|ENSG00000269476|tr
protein_co	12	0.42	A|frameshift_variant|HIGH|ZNF418|ENSG00000196724|transcript|
protein_co	12	0.42	AGG|frameshift_variant|HIGH|ZNF418|ENSG00000196724|transcri
)268201			T|gene_fusion|HIGH|CTD-3138B18.4&CTD-3138B18.6|ENSG000002
)268201			T|gene_fusion|HIGH|CTD-3138B18.4&CTD-3138B18.6|ENSG000002
)268201			T|gene_fusion|HIGH|CTD-3138B18.4&CTD-3138B18.6|ENSG000002
protein_coding			T|stop_gained|HIGH|ZNF837|ENSG00000152475|transcript|ENST0
lincRNA			G|splice_acceptor_variant&splice_region_variant&intron_variant&n
processed_transcript			TCTC|splice_acceptor_variant&intron_variant|HIGH|CENPBD1P1|E
protein_co	2	0.5	G|frameshift_variant|HIGH|DEFB126|ENSG00000125788|transcript
protein_co	2	0.5	A|frameshift_variant|HIGH|DEFB126|ENSG00000125788|transcript
protein_co	1	1	GT|frameshift_variant|HIGH|DEFB128|ENSG00000185982|transcri
protein_co	3	0.33	TTA|splice_donor_variant&intron_variant|HIGH|C20orf96|ENSG000
protein_co	3	0.33	TTA|splice_donor_variant&intron_variant|HIGH|C20orf96|ENSG000
protein_co	3	0.33	TTA|splice_donor_variant&intron_variant|HIGH|C20orf96|ENSG000
protein_co	3	0.33	TTA|splice_donor_variant&intron_variant|HIGH|C20orf96|ENSG000
protein_co	3	0.33	TTA|splice_donor_variant&intron_variant|HIGH|C20orf96|ENSG000
antisense			G|splice_donor_variant&intron_variant|HIGH|RP4-545L17.11|ENSG
lincRNA			G|splice_donor_variant&intron_variant|HIGH|RP5-968J1.1|ENSG00
lincRNA			T|splice_donor_variant&intron_variant|HIGH|RP11-128M1.1|ENSG
protein_coding			C|protein_protein_contact|HIGH|VPS16|ENSG00000215305|intera

antisense			C|splice_donor_variant&intron_variant|HIGH|UBOX5-AS1|ENSG000
protein_coding			A|structural_interaction_variant|HIGH|ITPA|ENSG00000125877|int
protein_co	7	0.57	C|splice_donor_variant&intron_variant|HIGH|SLC4A11|ENSG00000
protein_coding			A|frameshift_variant|HIGH|ADAM33|ENSG00000149451|transcript
protein_co	2	0.5	A|stop_gained|HIGH|SIGLEC1|ENSG00000088827|transcript|ENST0
protein_coding			T|structural_interaction_variant|HIGH|CDC25B|ENSG00000101224
protein_coding			G|structural_interaction_variant|HIGH|PRNP|ENSG00000171867|ir
protein_co	5	0.6	TGGGGGGGG|frameshift_variant|HIGH|SLC23A2|ENSG0000008905
protein_co	5	0.6	TGGGGGGGG|frameshift_variant|HIGH|SLC23A2|ENSG0000008905
protein_co	5	0.6	TGGGGGGGG|frameshift_variant|HIGH|SLC23A2|ENSG0000008905
protein_coding			A|stop_gained|HIGH|PLCB1|ENSG00000182621|transcript|ENST00
protein_coding			A|structural_interaction_variant|HIGH|JAG1|ENSG00000101384|in
protein_coding			A|structural_interaction_variant|HIGH|JAG1|ENSG00000101384|in
antisense			A|splice_donor_variant&intron_variant|HIGH|RP11-103J8.1|ENSG0
lincRNA			A|splice_donor_variant&intron_variant|HIGH|RP5-1069C8.2|ENSG0
protein_co	3	0.33	C|start_lost|HIGH|OTOR|ENSG00000125879|transcript|ENST00000
protein_coding			T|frameshift_variant&stop_lost|HIGH|BFSP1|ENSG00000125864|tr
processed_transcript			T|splice_donor_variant&intron_variant|HIGH|KAT14|ENSG0000014
protein_co	7	0.14	T|splice_donor_variant&intron_variant|HIGH|KAT14|ENSG0000014
protein_co	7	0.29	G|splice_acceptor_variant&intron_variant|HIGH|KAT14|ENSG00000
nonsense_	11	0.36	T|splice_acceptor_variant&intron_variant|HIGH|DZANK1|ENSG0000
nonsense_	11	0.27	A|splice_acceptor_variant&intron_variant|HIGH|DZANK1|ENSG0000
nonsense_	7	0.14	T|stop_gained|HIGH|CRNKL1|ENSG00000101343|transcript|ENST0
protein_co	7	0.29	G|splice_acceptor_variant&intron_variant|HIGH|CRNKL1|ENSG0000
protein_coding			CG|frameshift_variant|HIGH|KIZ|ENSG00000088970|transcript|ENS
lincRNA			G|splice_donor_variant&intron_variant|HIGH|RP11-359G22.2|ENSG
protein_co	3	0.33	G|splice_acceptor_variant&intron_variant|HIGH|CST3|ENSG000001
protein_coding			C|structural_interaction_variant|HIGH|CST7|ENSG00000077984|int
nonsense_	9	0.11	A|splice_donor_variant&intron_variant|HIGH|ABHD12|ENSG000001
protein_coding			AG|frameshift_variant|HIGH|ABHD12|ENSG00000100997|transcrip
protein_co	9	0.11	C|frameshift_variant|HIGH|FAM182B|ENSG00000175170|transcrip
antisense			G|splice_acceptor_variant&intron_variant|HIGH|LINC01598|ENSG0
protein_coding			A|structural_interaction_variant|HIGH|REM1|ENSG00000088320|ir
protein_co	4	0.25	T|stop_gained|HIGH|EFCAB8|ENSG00000215529|transcript|ENST0
unitary_pseudogene			A|splice_donor_variant&intron_variant|HIGH|BPIFB5P|ENSG000002
processed_pseudogene			C|splice_acceptor_variant&intron_variant|HIGH|TPM3P2|ENSG0000
processed_pseudogene			CTTTTCTTCATCTT|splice_acceptor_variant&intron_variant|HIGH|TP
processed_pseudogene			CTTTTCTTCATCTTT|splice_acceptor_variant&intron_variant|HIGH|T
processed_pseudogene			CTTTTCTTCATCTTTT|splice_acceptor_variant&intron_variant|HIGH|
processed_pseudogene			CTTTTCTTCATCTTTTT|splice_acceptor_variant&intron_variant|HIGH
processed_pseudogene			CTTTTCTTCATCTTTTTT|splice_acceptor_variant&intron_variant|HIGH
protein_co	6	0.17	CTT|splice_acceptor_variant&intron_variant|HIGH|ITCH|ENSG0000
protein_co	6	0.17	CTT|splice_acceptor_variant&intron_variant|HIGH|ITCH|ENSG0000
protein_co	4	1	C|frameshift_variant|HIGH|TP53INP2|ENSG00000078804|transcrip
protein_co	3	0.67	GTCTATCCACAGTTCCTCTGACCA|frameshift_variant&stop_gained|HI
protein_co	7	0.14	A|stop_gained|HIGH|SPAG4|ENSG00000061656|transcript|ENST00
protein_co	28	0.04	C|splice_acceptor_variant&intron_variant|HIGH|CPNE1|ENSG0000

<table>
<tr><td>protein_co</td><td>28</td><td>0.25</td><td>CA|frameshift_variant|HIGH|CPNE1|ENSG00000214078|transcript|</td></tr>
<tr><td>protein_co</td><td>3</td><td>0.33</td><td>CTTT|splice_acceptor_variant&intron_variant|HIGH|AAR2|ENSG000</td></tr>
<tr><td>protein_co</td><td>3</td><td>0.33</td><td>C|splice_region_variant&intron_variant|LOW|AAR2|ENSG00000131</td></tr>
<tr><td>protein_co</td><td>3</td><td>0.33</td><td>CT|splice_region_variant&intron_variant|LOW|AAR2|ENSG0000013</td></tr>
<tr><td>protein_co</td><td>3</td><td>0.33</td><td>C|frameshift_variant|HIGH|AAR2|ENSG00000131043|transcript|EN</td></tr>
<tr><td>protein_co</td><td>8</td><td>0.38</td><td>GCTTACAGACAGGGCCCCGCGGCCGGCACT|frameshift_variant&stop_</td></tr>
<tr><td>protein_co</td><td>8</td><td>0.38</td><td>GCTTACAGACAGGGCCCCGCGGCCGGCACT|frameshift_variant&stop_</td></tr>
<tr><td>protein_coding</td><td></td><td></td><td>T|structural_interaction_variant|HIGH|TGM2|ENSG00000198959|i</td></tr>
<tr><td>protein_coding</td><td></td><td></td><td>T|structural_interaction_variant|HIGH|TGM2|ENSG00000198959|i</td></tr>
<tr><td>protein_co</td><td>7</td><td>0.29</td><td>A|stop_gained|HIGH|KIAA1755|ENSG00000149633|transcript|ENS</td></tr>
<tr><td>processed_pseudogene</td><td></td><td></td><td>C|splice_acceptor_variant&splice_region_variant&intron_variant&n</td></tr>
<tr><td>processed_pseudogene</td><td></td><td></td><td>CAA|splice_acceptor_variant&splice_region_variant&intron_variant</td></tr>
<tr><td>processed_pseudogene</td><td></td><td></td><td>CAAAAA|splice_acceptor_variant&splice_region_variant&intron_var</td></tr>
<tr><td>processed_pseudogene</td><td></td><td></td><td>CAAAAAAAAAAAAA|splice_region_variant&non_coding_transcript_e</td></tr>
<tr><td>protein_co</td><td>5</td><td>0.4</td><td>G|frameshift_variant|HIGH|TTPAL|ENSG00000124120|transcript|E</td></tr>
<tr><td>protein_coding</td><td></td><td></td><td>C|structural_interaction_variant|HIGH|ADA|ENSG00000196839|int</td></tr>
<tr><td>protein_coding</td><td></td><td></td><td>C|structural_interaction_variant|HIGH|ADA|ENSG00000196839|int</td></tr>
<tr><td>protein_coding</td><td></td><td></td><td>T|structural_interaction_variant|HIGH|ADA|ENSG00000196839|int</td></tr>
<tr><td>nonsense_mediated_decay</td><td></td><td></td><td>G|frameshift_variant|HIGH|ACOT8|ENSG00000101473|transcript|E</td></tr>
<tr><td>protein_co</td><td>25</td><td>0.08</td><td>G|frameshift_variant|HIGH|SLC12A5|ENSG00000124140|transcript</td></tr>
<tr><td>protein_co</td><td>25</td><td>0.08</td><td>G|frameshift_variant|HIGH|SLC12A5|ENSG00000124140|transcript</td></tr>
<tr><td>protein_co</td><td>25</td><td>0.08</td><td>G|frameshift_variant|HIGH|SLC12A5|ENSG00000124140|transcript</td></tr>
<tr><td>protein_co</td><td>25</td><td>0.08</td><td>G|frameshift_variant|HIGH|SLC12A5|ENSG00000124140|transcript</td></tr>
<tr><td>protein_co</td><td>7</td><td>0.57</td><td>CTTT|splice_acceptor_variant&intron_variant|HIGH|CD40|ENSG000</td></tr>
<tr><td>protein_co</td><td>7</td><td>0.57</td><td>CTTT|splice_acceptor_variant&intron_variant|HIGH|CD40|ENSG000</td></tr>
<tr><td>protein_co</td><td>7</td><td>0.57</td><td>CTTT|splice_acceptor_variant&intron_variant|HIGH|CD40|ENSG000</td></tr>
<tr><td>protein_co</td><td>7</td><td>0.57</td><td>CTTT|splice_acceptor_variant&intron_variant|HIGH|CD40|ENSG000</td></tr>
<tr><td>protein_coding</td><td></td><td></td><td>T|structural_interaction_variant|HIGH|EYA2|ENSG00000064655|in</td></tr>
<tr><td>processed_pseudogene</td><td></td><td></td><td>C|splice_acceptor_variant&splice_region_variant&intron_variant|HI</td></tr>
<tr><td>processed_pseudogene</td><td></td><td></td><td>C|splice_acceptor_variant&splice_region_variant&intron_variant|HI</td></tr>
<tr><td>processed_pseudogene</td><td></td><td></td><td>C|splice_acceptor_variant&splice_region_variant&intron_variant|HI</td></tr>
<tr><td>processed_pseudogene</td><td></td><td></td><td>C|splice_acceptor_variant&splice_region_variant&intron_variant|HI</td></tr>
<tr><td>processed_pseudogene</td><td></td><td></td><td>C|splice_acceptor_variant&splice_region_variant&intron_variant|HI</td></tr>
<tr><td>antisense</td><td></td><td></td><td>AGGGGACCATGCTTCTCTCCCAC|splice_acceptor_variant&intron_vari</td></tr>
<tr><td>protein_co</td><td>10</td><td>0.1</td><td>AC|splice_donor_variant&intron_variant|HIGH|STAU1|ENSG000001</td></tr>
<tr><td>processed_transcript</td><td></td><td></td><td>GT|splice_acceptor_variant&intron_variant|HIGH|RP4-791K14.2|EN</td></tr>
<tr><td>protein_coding</td><td></td><td></td><td>A|structural_interaction_variant|HIGH|PTGIS|ENSG00000124212|i</td></tr>
<tr><td>protein_co</td><td>4</td><td>0.5</td><td>T|start_lost|HIGH|SLC9A8|ENSG00000197818|transcript|ENST000</td></tr>
<tr><td>protein_co</td><td>4</td><td>0.25</td><td>GT|frameshift_variant|HIGH|SLC9A8|ENSG00000197818|transcript</td></tr>
<tr><td>protein_coding</td><td></td><td></td><td>G|structural_interaction_variant|HIGH|PTPN1|ENSG00000196396|i</td></tr>
<tr><td>protein_coding</td><td></td><td></td><td>G|structural_interaction_variant|HIGH|NFATC2|ENSG00000101096</td></tr>
<tr><td>lincRNA</td><td></td><td></td><td>G|splice_acceptor_variant&intron_variant|HIGH|LINC01440|ENSG0</td></tr>
<tr><td>protein_co</td><td>2</td><td>0.5</td><td>G|splice_donor_variant&intron_variant|HIGH|GCNT7|ENSG000001</td></tr>
<tr><td>protein_co</td><td>2</td><td>0.5</td><td>C|frameshift_variant|HIGH|FAM209A|ENSG00000124103|transcrip</td></tr>
<tr><td>protein_co</td><td>2</td><td>0.5</td><td>CGTGT|frameshift_variant|HIGH|FAM209A|ENSG00000124103|tra</td></tr>
<tr><td>protein_co</td><td>1</td><td>1</td><td>CAATA|frameshift_variant|HIGH|FAM209B|ENSG00000213714|tra</td></tr>
<tr><td>protein_co</td><td>1</td><td>1</td><td>C|frameshift_variant|HIGH|FAM209B|ENSG00000213714|transcrip</td></tr>
</table>

<table>
<tr><td>lincRNA</td><td></td><td></td><td>A|splice_acceptor_variant&intron_variant|HIGH|RP11-560A15.4|EN</td></tr>
<tr><td>lincRNA</td><td></td><td></td><td>C|splice_donor_variant&intron_variant|HIGH|RP4-813D12.3|ENSG</td></tr>
<tr><td>protein_coding</td><td></td><td></td><td>T|structural_interaction_variant|HIGH|PCK1|ENSG00000124253|int</td></tr>
<tr><td>protein_coding</td><td></td><td></td><td>A|structural_interaction_variant|HIGH|PCK1|ENSG00000124253|int</td></tr>
<tr><td>protein_coding</td><td></td><td></td><td>T|structural_interaction_variant|HIGH|PCK1|ENSG00000124253|int</td></tr>
<tr><td>protein_coding</td><td></td><td></td><td>A|structural_interaction_variant|HIGH|PCK1|ENSG00000124253|int</td></tr>
<tr><td>protein_coding</td><td></td><td></td><td>A|structural_interaction_variant|HIGH|PCK1|ENSG00000124253|int</td></tr>
<tr><td>protein_co</td><td>9</td><td>0.11</td><td>G|frameshift_variant|HIGH|ZBP1|ENSG00000124256|transcript|EN</td></tr>
<tr><td>protein_co</td><td>9</td><td>0.33</td><td>T|start_lost|HIGH|ZBP1|ENSG00000124256|transcript|ENST000003</td></tr>
<tr><td>unprocessed_pseudogene</td><td></td><td></td><td>C|splice_donor_variant&intron_variant|HIGH|PIEZO1P2|ENSG0000</td></tr>
<tr><td>protein_coding</td><td></td><td></td><td>A|protein_protein_contact|HIGH|PSMA7|ENSG00000101182|intera</td></tr>
<tr><td>nonsense_</td><td>15</td><td>0.07</td><td>A|splice_donor_variant&intron_variant|HIGH|LAMA5|ENSG000001</td></tr>
<tr><td>protein_coding</td><td></td><td></td><td>C|stop_lost|HIGH|RPS21|ENSG00000171858|transcript|ENST0000C</td></tr>
<tr><td>antisense</td><td></td><td></td><td>C|splice_donor_variant&intron_variant|HIGH|RP5-908M14.5|ENSG</td></tr>
<tr><td>protein_co</td><td>7</td><td>0.43</td><td>A|splice_donor_variant&splice_region_variant&intron_variant|HIGH</td></tr>
<tr><td>lincRNA</td><td></td><td></td><td>T|splice_acceptor_variant&intron_variant|HIGH|LINC00029|ENSG0</td></tr>
<tr><td>protein_coding</td><td></td><td></td><td>A|structural_interaction_variant|HIGH|YTHDF1|ENSG00000149658</td></tr>
<tr><td>protein_co</td><td>20</td><td>0.1</td><td>C|splice_acceptor_variant&intron_variant|HIGH|CHRNA4|ENSG000</td></tr>
<tr><td>protein_coding</td><td></td><td></td><td>G|structural_interaction_variant|HIGH|TNFRSF6B|ENSG000002435C</td></tr>
<tr><td>protein_coding</td><td></td><td></td><td>C|structural_interaction_variant|HIGH|TNFRSF6B|ENSG000002435C</td></tr>
<tr><td>protein_coding</td><td></td><td></td><td>T|structural_interaction_variant|HIGH|TNFRSF6B|ENSG000002435C</td></tr>
<tr><td>processed_transcript</td><td></td><td></td><td>T|splice_donor_variant&intron_variant|HIGH|BAGE2|ENSG0000018</td></tr>
<tr><td>protein_co</td><td>5</td><td>0.6</td><td>A|splice_donor_variant&intron_variant|HIGH|TPTE|ENSG00000274</td></tr>
<tr><td>protein_co</td><td>5</td><td>0.8</td><td>T|splice_acceptor_variant&intron_variant|HIGH|TPTE|ENSG000002</td></tr>
<tr><td>transcribed_unprocessed_pseudo</td><td></td><td></td><td>T|splice_donor_variant&intron_variant|HIGH|ANKRD20A11P|ENSG</td></tr>
<tr><td>transcribed_unprocessed_pseudo</td><td></td><td></td><td>A|splice_donor_variant&intron_variant|HIGH|ABCC13|ENSG000002</td></tr>
<tr><td>lincRNA</td><td></td><td></td><td>T|splice_donor_variant&intron_variant|HIGH|AF127936.7|ENSG000</td></tr>
<tr><td>lincRNA</td><td></td><td></td><td>T|splice_acceptor_variant&splice_region_variant&intron_variant&n</td></tr>
<tr><td>0215386</td><td></td><td></td><td>G|bidirectional_gene_fusion|HIGH|MIR99AHG&SNORD74|ENSG000</td></tr>
<tr><td>lincRNA</td><td></td><td></td><td>A|splice_acceptor_variant&intron_variant|HIGH|AF212831.2|ENSG</td></tr>
<tr><td>protein_coding</td><td></td><td></td><td>C|structural_interaction_variant|HIGH|NCAM2|ENSG00000154654|</td></tr>
<tr><td>lincRNA</td><td></td><td></td><td>A|splice_donor_variant&intron_variant|HIGH|AP000233.2|ENSG000</td></tr>
<tr><td>lincRNA</td><td></td><td></td><td>T|splice_donor_variant&intron_variant|HIGH|AP000233.3|ENSG000</td></tr>
<tr><td>lincRNA</td><td></td><td></td><td>CTTT|splice_acceptor_variant&intron_variant|HIGH|AP000230.1|EN</td></tr>
<tr><td>lincRNA</td><td></td><td></td><td>CTTT|splice_acceptor_variant&intron_variant|HIGH|AP000230.1|EN</td></tr>
<tr><td>lincRNA</td><td></td><td></td><td>CTTT|splice_acceptor_variant&intron_variant|HIGH|AP000230.1|EN</td></tr>
<tr><td>protein_coding</td><td></td><td></td><td>G|frameshift_variant|HIGH|ADAMTS1|ENSG00000154734|transcrip</td></tr>
<tr><td>protein_co</td><td>6</td><td>0.17</td><td>G|splice_donor_variant&intron_variant|HIGH|ADAMTS1|ENSG0000</td></tr>
<tr><td>protein_co</td><td>6</td><td>0.17</td><td>T|stop_gained|HIGH|ADAMTS1|ENSG00000154734|transcript|ENST</td></tr>
<tr><td>lincRNA</td><td></td><td></td><td>G|splice_acceptor_variant&splice_region_variant&intron_variant&n</td></tr>
<tr><td>lincRNA</td><td></td><td></td><td>GTTTT|splice_acceptor_variant&intron_variant|HIGH|AJ006995.3|E</td></tr>
<tr><td>lincRNA</td><td></td><td></td><td>G|splice_region_variant&intron_variant|LOW|AJ006995.3|ENSG000</td></tr>
<tr><td>lincRNA</td><td></td><td></td><td>GT|splice_region_variant&intron_variant|LOW|AJ006995.3|ENSG00</td></tr>
<tr><td>lincRNA</td><td></td><td></td><td>GTT|splice_region_variant&intron_variant|LOW|AJ006995.3|ENSG0</td></tr>
<tr><td>processed_pseudogene</td><td></td><td></td><td>CT|splice_acceptor_variant&intron_variant|HIGH|HSPD1P7|ENSG00</td></tr>
<tr><td>processed_pseudogene</td><td></td><td></td><td>CT|splice_acceptor_variant&intron_variant|HIGH|HSPD1P7|ENSG00</td></tr>
<tr><td>processed_pseudogene</td><td></td><td></td><td>CT|splice_acceptor_variant&intron_variant|HIGH|HSPD1P7|ENSG00</td></tr>
</table>

processed_pseudogene			A|splice_acceptor_variant&intron_variant|HIGH|THUMPD1P1|ENSG
processed_pseudogene			ATT|splice_region_variant&intron_variant|LOW|THUMPD1P1|ENSG
lincRNA			G|splice_acceptor_variant&intron_variant|HIGH|BACH1-IT2|ENSG0
lincRNA			G|splice_donor_variant&intron_variant|HIGH|AF096876.1|ENSG000
protein_coding			T|stop_gained|HIGH|KRTAP13-2|ENSG00000182816|transcript|ENS
protein_coding			A|frameshift_variant|HIGH|KRTAP19-7|ENSG00000244362|transcri
nonsense_mediated_decay			T|stop_lost|HIGH|TCP10L|ENSG00000242220|transcript|ENST0000
protein_co	8	0.13	C|frameshift_variant|HIGH|C21orf59|ENSG00000159079|transcript
)265590			A|gene_fusion|HIGH|AP000275.65&C21orf59|ENSG00000265590&
lincRNA			G|splice_donor_variant&intron_variant|HIGH|AP000289.6|ENSG000
protein_coding			GA|frameshift_variant|HIGH|SON|ENSG00000159140|transcript|EN
protein_coding			G|frameshift_variant|HIGH|SON|ENSG00000159140|transcript|ENS
protein_co	1	1.00,(ATP5	A|stop_gained&splice_region_variant|HIGH|ATP5O|ENSG00000241
protein_co	1	1	G|splice_donor_variant&intron_variant|HIGH|AP000304.12|ENSG0
protein_coding			G|structural_interaction_variant|HIGH|CBR3|ENSG00000159231|in
protein_coding			T|structural_interaction_variant|HIGH|CBR3|ENSG00000159231|in
protein_coding			T|structural_interaction_variant|HIGH|CBR3|ENSG00000159231|in
protein_coding			A|structural_interaction_variant|HIGH|CBR3|ENSG00000159231|in
nonsense_	12	0.25	A|splice_donor_variant&intron_variant|HIGH|MORC3|ENSG000001
protein_co	5	0.2	G|frameshift_variant|HIGH|SIM2|ENSG00000159263|transcript|EN
protein_co	5	0.2	G|frameshift_variant|HIGH|SIM2|ENSG00000159263|transcript|EN
nonsense_mediated_decay			G|stop_lost|HIGH|PSMG1|ENSG00000183527|transcript|ENST0000
protein_co	3	0.33	A|stop_gained|HIGH|IGSF5|ENSG00000183067|transcript|ENST000
protein_co	6	0.17	T|splice_donor_variant&intron_variant|HIGH|FAM3B|ENSG0000018
protein_co	16	0.13	CG|frameshift_variant|HIGH|PRDM15|ENSG00000141956|transcrip
lincRNA			C|splice_acceptor_variant&intron_variant|HIGH|ZNF295-AS1|ENSG
protein_co	14	0.14	T|stop_gained|HIGH|UMODL1|ENSG00000177398|transcript|ENST
protein_co	8	0.5	CTA|splice_acceptor_variant&intron_variant|HIGH|TMPRSS3|ENSG
protein_coding			T|structural_interaction_variant|HIGH|PDE9A|ENSG00000160191|i
protein_co	4	0.25	T|stop_gained|HIGH|NDUFV3|ENSG00000160194|transcript|ENST0
protein_coding			A|structural_interaction_variant|HIGH|PDXK|ENSG00000160209|in
protein_coding			A|structural_interaction_variant|HIGH|PDXK|ENSG00000160209|in
processed_pseudogene			G|splice_acceptor_variant&intron_variant|HIGH|H2AFZP1|ENSG000
antisense			C|splice_donor_variant&intron_variant|HIGH|AP001062.9|ENSG000
protein_co	1	1	ATG|frameshift_variant|HIGH|KRTAP10-1|ENSG00000215455|trans
protein_co	1	1	A|frameshift_variant|HIGH|KRTAP10-1|ENSG00000215455|transcri
protein_coding			C|stop_lost|HIGH|KRTAP10-4|ENSG00000215454|transcript|ENST0
protein_co	2	0.5	T|stop_gained|HIGH|KRTAP10-6|ENSG00000188155|transcript|ENS
protein_co	2	1	GCT|frameshift_variant|HIGH|KRTAP10-6|ENSG00000188155|trans
protein_co	2	1	CCAGCTGCT|frameshift_variant|HIGH|KRTAP10-7|ENSG00000272 8
protein_co	1	1	GCC|frameshift_variant|HIGH|KRTAP10-10|ENSG00000221859|trar
protein_co	24	0.08	C|splice_acceptor_variant&intron_variant|HIGH|ITGB2|ENSG00000
protein_co	14	0.36	G|splice_donor_variant&splice_region_variant&intron_variant|HIGH
protein_co	7	0.14	TG|frameshift_variant|HIGH|COL18A1|ENSG00000182871|transcrip
protein_co	12	0.08	G|splice_donor_variant&intron_variant|HIGH|SLC19A1|ENSG00000
protein_coding			A|stop_gained|HIGH|SLC19A1|ENSG00000173638|transcript|ENST
protein_co	12	0.17	CGGTCCCCTCTCGCCGCCAG|frameshift_variant|HIGH|SLC19A1|ENSG

protein_co	7	0.57	G|frameshift_variant|HIGH|COL18A1|ENSG00000182871|transcript
sense_overlapping			T|splice_acceptor_variant&splice_region_variant&intron_variant&n
protein_co	8	0.63	AC|frameshift_variant|HIGH|COL6A2|ENSG00000142173|transcript
protein_coding			T|structural_interaction_variant|HIGH|LSS|ENSG00000160285|inte
protein_coding			G|structural_interaction_variant|HIGH|S100B|ENSG00000160307|i
protein_co	3	0.67	C|frameshift_variant|HIGH|POTEH|ENSG00000198062|transcript|E
unprocessed_pseudogene			A|splice_donor_variant&intron_variant|HIGH|KB-67B5.12|ENSG000
lincRNA			G|splice_acceptor_variant&intron_variant|HIGH|AC005301.9|ENSG
protein_co	4	0.25	A|stop_gained|HIGH|GAB4|ENSG00000215568|transcript|ENST000
protein_co	15	0.13	G|frameshift_variant|HIGH|BID|ENSG00000015475|transcript|ENS
protein_coding			T|stop_gained|HIGH|TUBA8|ENSG00000183785|transcript|ENST00
protein_co	1	1	CA|frameshift_variant|HIGH|RIMBP3|ENSG00000275793|transcript
protein_co	1	1	CGG|frameshift_variant|HIGH|RIMBP3|ENSG00000275793|transcri
antisense			C|splice_acceptor_variant&intron_variant|HIGH|AC000068.5|ENSG
protein_coding			A|stop_gained|HIGH|CLDN5|ENSG00000184113|transcript|ENST00
protein_coding			C|structural_interaction_variant|HIGH|TBX1|ENSG00000184058|in
non_stop_	21	0.05	T|frameshift_variant|HIGH|TXNRD2|ENSG00000184470|transcript|
protein_coding			T|structural_interaction_variant|HIGH|COMT|ENSG00000093010|i
protein_coding			G|structural_interaction_variant|HIGH|COMT|ENSG00000093010|i
protein_coding			A|structural_interaction_variant|HIGH|COMT|ENSG00000093010|i
protein_coding			T|structural_interaction_variant|HIGH|COMT|ENSG00000093010|i
protein_coding			A|frameshift_variant|HIGH|COMT|ENSG00000093010|transcript|E
protein_coding			A|frameshift_variant|HIGH|COMT|ENSG00000093010|transcript|E
protein_co	20	0.1	GC|frameshift_variant|HIGH|TRMT2A|ENSG00000099899|transcrip
protein_coding			G|splice_acceptor_variant&splice_region_variant&5_prime_UTR_va
retained_ir	31	0.03	CCCTA|splice_donor_variant&intron_variant|HIGH|MED15|ENSG00
protein_co	31	0.13	AGCAGCAG|frameshift_variant|HIGH|MED15|ENSG00000099917|t
protein_co	31	0.13	AGCAGCAG|frameshift_variant|HIGH|MED15|ENSG00000099917|t
processed_transcript			T|splice_acceptor_variant&intron_variant|HIGH|TMEM191A|ENSG
protein_co	18	0.06	T|frameshift_variant|HIGH|PI4KA|ENSG00000241973|transcript|E
antisense			A|splice_donor_variant&intron_variant|HIGH|THAP7-AS1|ENSG000
processed_transcript			C|splice_acceptor_variant&intron_variant|HIGH|TUBA3FP|ENSG000
processed_transcript			A|splice_acceptor_variant&splice_region_variant&intron_variant|HI
processed_transcript			A|splice_acceptor_variant&splice_region_variant&intron_variant|HI
IG_V_gene	1	1	T|frameshift_variant|HIGH|IGLV4-60|ENSG00000211639|transcript
IG_V_gene			C|stop_lost|HIGH|IGLV5-48|ENSG00000211647|transcript|ENST000
protein_co	5	0.2	G|splice_donor_variant&intron_variant|HIGH|GGTLC2|ENSG000001
IG_V_gene	1	1	G|frameshift_variant|HIGH|IGLV3-22|ENSG00000211661|transcript
IG_V_gene	1	1	TGATATAC|frameshift_variant|HIGH|IGLV3-22|ENSG00000211661|
IG_V_gene	1	1	A|frameshift_variant|HIGH|IGLV3-16|ENSG00000211665|transcript
IG_V_gene	1	1	C|start_lost|HIGH|IGLV3-12|ENSG00000211667|transcript|ENST000
IG_V_gene			G|frameshift_variant|HIGH|IGLV2-11|ENSG00000211668|transcript
protein_co	1	1.00,(IGLL5	C|frameshift_variant|HIGH|IGLL5|ENSG00000254709|transcript|EN
IG_J_gene	1	1	TA|frameshift_variant|HIGH|IGLJ2|ENSG00000211676|transcript|E
)100218			T|bidirectional_gene_fusion|HIGH|GNAZ&RSPH14|ENSG000001282
)100218			T|bidirectional_gene_fusion|HIGH|GNAZ&RSPH14|ENSG000001282
processed_transcript			C|splice_donor_variant&intron_variant|HIGH|GNAZ|ENSG0000012

protein_coding			CA|frameshift_variant|HIGH|RAB36|ENSG00000100228|transcript|
protein_coding			C|stop_lost|HIGH|BCR|ENSG00000186716|transcript|ENST0000039
antisense			A|splice_donor_variant&intron_variant|HIGH|AP000345.2|ENSG000
antisense			A|splice_acceptor_variant&intron_variant|HIGH|AP000345.2|ENSG
processed_transcript			G|splice_acceptor_variant&intron_variant|HIGH|KB-1572G7.2|ENSG
nonsense_	10	0.1	A|splice_acceptor_variant&intron_variant|HIGH|RGL4|ENSG000001
protein_co	14	0.36	C|frameshift_variant|HIGH|CABIN1|ENSG00000099991|transcript|I
protein_co	14	0.36	C|frameshift_variant|HIGH|CABIN1|ENSG00000099991|transcript|I
protein_co	14	0.36	C|frameshift_variant|HIGH|CABIN1|ENSG00000099991|transcript|I
protein_co	14	0.36	C|frameshift_variant|HIGH|CABIN1|ENSG00000099991|transcript|I
processed_transcript			C|splice_donor_variant&intron_variant|HIGH|POM121L9P|ENSG000
protein_co	5	0.4	A|splice_acceptor_variant&intron_variant|HIGH|UPB1|ENSG000001
protein_co	8	0.38	G|start_lost|HIGH|LRRC75B|ENSG00000178026|transcript|ENST000
protein_coding			T|structural_interaction_variant|HIGH|GGT1|ENSG00000100031|in
nonsense_mediated_decay			T|stop_gained|HIGH|GGT1|ENSG00000100031|transcript|ENST000
protein_coding			A|structural_interaction_variant|HIGH|GGT1|ENSG00000100031|in
processed_transcript			C|splice_donor_variant&intron_variant|HIGH|SGSM1|ENSG0000010
protein_coding			A|structural_interaction_variant|HIGH|CRYBB3|ENSG00000100053
antisense			C|splice_donor_variant&intron_variant|HIGH|CTA-125H2.2|ENSG00
protein_co	18	0.06	A|stop_gained|HIGH|HPS4|ENSG00000100099|transcript|ENST000
protein_coding			A|structural_interaction_variant|HIGH|TPST2|ENSG00000128294|ir
protein_coding			A|structural_interaction_variant|HIGH|TPST2|ENSG00000128294|ir
protein_coding			A|structural_interaction_variant|HIGH|CRYBA4|ENSG00000196431
protein_coding			C|structural_interaction_variant|HIGH|CRYBA4|ENSG00000196431
lincRNA			A|splice_acceptor_variant&intron_variant|HIGH|CTA-992D9.6|ENSG
antisense			T|splice_acceptor_variant&intron_variant|HIGH|RP11-375H17.1|EN
protein_co	8	0.13	A|stop_gained|HIGH|TTC28|ENSG00000100154|transcript|ENST000
protein_co	24	0.04	T|splice_acceptor_variant&intron_variant|HIGH|CHEK2|ENSG00000C
protein_coding			T|stop_gained|HIGH|RFPL1|ENSG00000128250|transcript|ENST000
protein_co	2	1	GAAGCTGTGGAAACACATCAAGCACAAGTATGAGAACAAGTA|frameshi
protein_co	10	0.1	CTAAA|splice_acceptor_variant&intron_variant|HIGH|RNF215|ENSG
protein_co	10	0.1	CTAAA|splice_acceptor_variant&intron_variant|HIGH|RNF215|ENSG
protein_coding			A|structural_interaction_variant|HIGH|SEC14L2|ENSG00000100003
protein_coding			A|structural_interaction_variant|HIGH|SEC14L3|ENSG00000100012
protein_coding			G|structural_interaction_variant|HIGH|SEC14L3|ENSG00000100012
protein_coding			G|structural_interaction_variant|HIGH|SEC14L3|ENSG00000100012
protein_coding			A|structural_interaction_variant|HIGH|SEC14L4|ENSG00000133488
protein_coding			C|structural_interaction_variant|HIGH|SEC14L4|ENSG00000133488
protein_coding			C|structural_interaction_variant|HIGH|SEC14L4|ENSG00000133488
protein_coding			A|structural_interaction_variant|HIGH|SEC14L4|ENSG00000133488
protein_coding			C|structural_interaction_variant|HIGH|SEC14L4|ENSG00000133488
antisense			T|splice_donor_variant&intron_variant|HIGH|MORC2-AS1|ENSG000
protein_co	24	0.04	G|start_lost|HIGH|SMTN|ENSG00000183963|transcript|ENST00000
protein_coding			G|structural_interaction_variant|HIGH|LIMK2|ENSG00000182541|i
protein_coding			T|stop_gained|HIGH|PRR14L|ENSG00000183530|transcript|ENST00
protein_co	1	1	A|frameshift_variant|HIGH|SLC5A4|ENSG00000100191|transcript|I
protein_co	1	1	A|stop_gained|HIGH|SLC5A4|ENSG00000100191|transcript|ENST00

protein_co	9	0.22	A|start_lost|HIGH|FBXO7|ENSG00000100225|transcript|ENST0000
protein_co	13	0.08	T|splice_acceptor_variant&intron_variant|HIGH|SYN3|ENSG000001
processed_transcript			C|splice_donor_variant&intron_variant|HIGH|MCM5|ENSG0000010
protein_co	12	0.25	A|frameshift_variant|HIGH|APOL4|ENSG00000100336|transcript|E
protein_co	12	0.67	C|splice_donor_variant&intron_variant|HIGH|APOL4|ENSG0000010
protein_co	12	0.08	T|start_lost|HIGH|APOL4|ENSG00000100336|transcript|ENST0000
lincRNA			A|splice_acceptor_variant&intron_variant|HIGH|RP4-633O19__A.1|
protein_coding			T|structural_interaction_variant|HIGH|CSF2RB|ENSG00000100368|
antisense			TTTTA|splice_acceptor_variant&intron_variant|HIGH|LL22NC01-81
antisense			TTTTATTTA|splice_acceptor_variant&intron_variant|HIGH|LL22NC0
antisense			TTTTATTTATTTA|splice_acceptor_variant&intron_variant|HIGH|LL2
protein_co	7	0.57	C|splice_acceptor_variant&splice_region_variant&intron_variant|HI
protein_coding			G|structural_interaction_variant|HIGH|RAC2|ENSG00000128340|in
protein_coding			A|structural_interaction_variant|HIGH|LGALS2|ENSG00000100079|
protein_co	23	0.04	C|start_lost|HIGH|GGA1|ENSG00000100083|transcript|ENST00000
protein_coding			T|structural_interaction_variant|HIGH|PDXP|ENSG00000241360|in
protein_co	3	0.67	G|splice_donor_variant&intron_variant|HIGH|SLC16A8|ENSG00000
protein_co	3	0.33	G|frameshift_variant&splice_acceptor_variant&splice_region_variar
protein_coding			C|structural_interaction_variant|HIGH|SUN2|ENSG00000100242|in
protein_coding			G|structural_interaction_variant|HIGH|APOBEC3G|ENSG000002397
protein_coding			A|structural_interaction_variant|HIGH|EP300|ENSG00000100393|i
processed_transcript			G|splice_acceptor_variant&intron_variant|HIGH|EP300|ENSG00000
protein_co	3	0.67	ACGCGCGCC|frameshift_variant|HIGH|CHADL|ENSG00000100399|t
nonsense_	9	0.11	C|frameshift_variant&splice_region_variant|HIGH|PMM1|ENSG000
protein_coding			A|stop_gained|HIGH|CENPM|ENSG00000100162|transcript|ENST0
protein_coding			G|structural_interaction_variant|HIGH|CENPM|ENSG00000100162|
protein_co	5	0.4	C|frameshift_variant|HIGH|CYP2D6|ENSG00000100197|transcript|
processed_transcript			TA|splice_donor_variant&intron_variant|HIGH|RRP7BP|ENSG00000
protein_co	10	0.2	C|frameshift_variant|HIGH|EFCAB6|ENSG00000186976|transcript|
protein_co	19	0.11	C|splice_donor_variant&intron_variant|HIGH|NUP50|ENSG0000009
protein_co	19	0.05	T|splice_donor_variant&intron_variant|HIGH|NUP50|ENSG0000009
protein_coding			T|structural_interaction_variant|HIGH|PPARA|ENSG00000186951|i
lincRNA			G|splice_donor_variant&intron_variant|HIGH|CITF22-49D8.1|ENSG
lincRNA			A|splice_acceptor_variant&intron_variant|HIGH|LINC01310|ENSG0
unprocessed_pseudogene			C|splice_acceptor_variant&intron_variant|HIGH|RPL5P35|ENSG000
unprocessed_pseudogene			CTT|splice_region_variant&intron_variant|LOW|RPL5P35|ENSG000
protein_coding			C|splice_acceptor_variant&disruptive_inframe_deletion&splice_regi
protein_coding			G|structural_interaction_variant|HIGH|MAPK12|ENSG0000018813
protein_coding			A|structural_interaction_variant|HIGH|MAPK12|ENSG0000018813
protein_coding			C|structural_interaction_variant|HIGH|MAPK11|ENSG00000185386
protein_co	9	0.78	GTCCC|splice_acceptor_variant&intron_variant|HIGH|PPP6R2|ENSG
protein_co	9	0.78	C|splice_acceptor_variant&intron_variant|HIGH|PPP6R2|ENSG0000
protein_coding			A|structural_interaction_variant|HIGH|MIOX|ENSG00000100253|ir
protein_coding			A|structural_interaction_variant|HIGH|MIOX|ENSG00000100253|ir
protein_co	10	0.3	T|splice_donor_variant&intron_variant|HIGH|NCAPH2|ENSG000000
protein_coding			A|structural_interaction_variant|HIGH|TYMP|ENSG00000025708|ir
protein_coding			T|structural_interaction_variant|HIGH|TYMP|ENSG00000025708|in

protein_co	8	0.63	C|splice_donor_variant&intron_variant|HIGH|TYMP|ENSG0000002[illegible]
nonsense_	8	0.13	T|splice_donor_variant&intron_variant|HIGH|ARSA|ENSG00000100
protein_co	3	0.67	G|splice_acceptor_variant&splice_region_variant&intron_variant|HI
protein_co	3	0.67	C|frameshift_variant|HIGH|SHANK3|ENSG00000251322|transcript|
protein_coding			C|frameshift_variant&stop_lost|HIGH|ASMTL|ENSG00000169093|t
protein_coding			C|structural_interaction_variant|HIGH|ASMTL|ENSG00000169093|i
protein_co	5	0.6	C|splice_donor_variant&intron_variant|HIGH|ASMT|ENSG0000019[illegible]
protein_coding			T|structural_interaction_variant|HIGH|ASMT|ENSG00000196433|ir
protein_coding			T|structural_interaction_variant|HIGH|ASMT|ENSG00000196433|ir
protein_coding			A|structural_interaction_variant|HIGH|NLGN4X|ENSG00000146938
protein_coding			T|structural_interaction_variant|HIGH|PUDP|ENSG00000130021|in
protein_co	3	0.33	A|splice_donor_variant&intron_variant|HIGH|VCX|ENSG000001825
protein_co	3	0.67	ACT|frameshift_variant|HIGH|VCX|ENSG00000182583|transcript|E
protein_co	3	0.67	ACT|frameshift_variant|HIGH|VCX|ENSG00000182583|transcript|E
protein_co	1	1	C|splice_donor_variant&intron_variant|HIGH|VCX2|ENSG00000177
protein_co	4	0.75	A|splice_donor_variant&intron_variant|HIGH|VCX3B|ENSG0000020
processed_transcript			CTT|splice_acceptor_variant&intron_variant|HIGH|TBL1X|ENSG000
processed_transcript			CTT|splice_acceptor_variant&intron_variant|HIGH|TBL1X|ENSG000
processed_pseudogene			G|splice_acceptor_variant&splice_region_variant&intron_variant|HI
processed_pseudogene			G|splice_acceptor_variant&splice_region_variant&intron_variant|HI
processed_pseudogene			G|splice_acceptor_variant&splice_region_variant&intron_variant|HI
processed_pseudogene			G|splice_acceptor_variant&splice_region_variant&intron_variant|HI
processed_pseudogene			G|splice_acceptor_variant&splice_region_variant&intron_variant|HI
protein_co	2	0.5	G|start_lost&splice_region_variant|HIGH|TLR8|ENSG00000101916|
protein_coding			T|structural_interaction_variant|HIGH|TLR8|ENSG00000101916|int
protein_coding			C|structural_interaction_variant|HIGH|TLR8|ENSG00000101916|int
protein_coding			A|structural_interaction_variant|HIGH|TLR8|ENSG00000101916|int
protein_co	2	0.5	A|stop_gained|HIGH|ATXN3L|ENSG00000123594|transcript|ENST0
nonsense_mediated_decay			T|stop_lost&splice_region_variant|HIGH|TCEANC|ENSG0000017689
nonsense_	19	0.05	T|frameshift_variant|HIGH|PIGA|ENSG00000165195|transcript|ENS
protein_coding			T|structural_interaction_variant|HIGH|PIR|ENSG00000087842|inte
antisense			C|splice_donor_variant&intron_variant|HIGH|NHS-AS1|ENSG00000
protein_co	5	0.2	CT|splice_donor_variant&splice_region_variant&intron_variant|HIG
protein_coding			A|structural_interaction_variant|HIGH|SCML2|ENSG00000102098|i
protein_co	13	0.08	T|frameshift_variant|HIGH|PDHA1|ENSG00000131828|transcript|E
protein_co	13	0.08	T|frameshift_variant|HIGH|PDHA1|ENSG00000131828|transcript|E
protein_co	13	0.08	T|frameshift_variant|HIGH|PDHA1|ENSG00000131828|transcript|E
protein_co	13	0.08	T|frameshift_variant|HIGH|PDHA1|ENSG00000131828|transcript|E
protein_co	4	0.25	A|stop_gained|HIGH|MAP3K15|ENSG00000180815|transcript|ENS
unprocessed_pseudogene			C|splice_acceptor_variant&intron_variant|HIGH|EIF5P2|ENSG00000
unprocessed_pseudogene			C|splice_acceptor_variant&intron_variant|HIGH|EIF5P2|ENSG00000
lincRNA			T|splice_donor_variant&intron_variant|HIGH|PTCHD1-AS|ENSG000
antisense			A|splice_donor_variant&intron_variant|HIGH|PCYT1B-AS1|ENSG000
protein_coding			T|structural_interaction_variant|HIGH|IL1RAPL1|ENSG0000016930[illegible]
protein_co	6	0.33	A|splice_donor_variant&intron_variant|HIGH|TAB3|ENSG00000157
protein_coding			T|stop_gained|HIGH|MAGEB16|ENSG00000189023|transcript|ENS
protein_co	7	0.14	CTG|frameshift_variant&stop_gained|HIGH|CFAP47|ENSG0000016[illegible]

antisense			G|splice_acceptor_variant&intron_variant|HIGH|RP11-87M18.2|EN
protein_co	2	0.5	A|splice_donor_variant&intron_variant|HIGH|SYTL5|ENSG0000014
protein_coding			A|stop_lost|HIGH|RPGR|ENSG00000156313|transcript|ENST00000
protein_coding			T|frameshift_variant|HIGH|BCOR|ENSG00000183337|transcript|EN
protein_co	33	0.03	C|frameshift_variant|HIGH|ATP6AP2|ENSG00000182220|transcript
protein_co	33	0.03	C|frameshift_variant|HIGH|ATP6AP2|ENSG00000182220|transcript
protein_co	33	0.03	C|frameshift_variant|HIGH|ATP6AP2|ENSG00000182220|transcript
nonsense_	22	0.27	T|splice_acceptor_variant&intron_variant|HIGH|DDX3X|ENSG00000
protein_coding			A|stop_gained|HIGH|ZNF674|ENSG00000251192|transcript|ENST0
processed_pseudogene			C|splice_acceptor_variant&splice_region_variant&intron_variant&n
processed_pseudogene			CTTTT|splice_region_variant&intron_variant|LOW|MKI67P1|ENSG0
processed_pseudogene			CTTTTT|splice_region_variant&intron_variant|LOW|MKI67P1|ENSG
processed_pseudogene			CTTTTTT|splice_region_variant&intron_variant|LOW|MKI67P1|ENS
processed_pseudogene			CTTTTTTTT|splice_region_variant&intron_variant|LOW|MKI67P1|EN
protein_coding			T|structural_interaction_variant|HIGH|RP2|ENSG00000102218|inte
processed_pseudogene			C|splice_acceptor_variant&intron_variant|HIGH|NPM1P49|ENSG00
processed_pseudogene			CTT|splice_region_variant&intron_variant|LOW|NPM1P49|ENSG00
protein_co	1	1	C|frameshift_variant|HIGH|AL022578.1|ENSG00000279988|transcr
unprocessed_pseudogene			G|splice_acceptor_variant&intron_variant|HIGH|SSXP3|ENSG00000
processed_pseudogene			AAAAAAAT|splice_acceptor_variant&intron_variant|HIGH|RP11-344
protein_co	8	0.63	C|frameshift_variant|HIGH|CCDC120|ENSG00000147144|transcript
nonsense_	8	0.13	A|frameshift_variant|HIGH|PRICKLE3|ENSG00000012211|transcript
nonsense_	8	0.13	A|frameshift_variant|HIGH|PRICKLE3|ENSG00000012211|transcript
protein_co	1	1	A|frameshift_variant&start_lost|HIGH|NUDT11|ENSG00000196368
protein_co	2	0.5	CTT|splice_acceptor_variant&intron_variant|HIGH|GPR173|ENSG00
protein_co	2	0.5	CTT|splice_acceptor_variant&intron_variant|HIGH|GPR173|ENSG00
protein_co	2	0.5	CTT|splice_acceptor_variant&intron_variant|HIGH|GPR173|ENSG00
protein_coding			C|structural_interaction_variant|HIGH|HUWE1|ENSG00000086758|
protein_co	4	0.75	AT|frameshift_variant|HIGH|FAM120C|ENSG00000184083|transcri
protein_co	4	0.75	GGCGGC|frameshift_variant|HIGH|FAM120C|ENSG00000184083|tr
protein_co	4	0.75	GGCGGC|frameshift_variant|HIGH|FAM120C|ENSG00000184083|tr
protein_co	2	0.5	TA|splice_donor_variant&intron_variant|HIGH|ITIH6|ENSG0000010
protein_co	3	0.67	C|start_lost|HIGH|PAGE5|ENSG00000158639|transcript|ENST0000
protein_co	1	1	G|frameshift_variant|HIGH|ZXDA|ENSG00000198205|transcript|EN
protein_co	1	1	GGC|frameshift_variant|HIGH|ZXDA|ENSG00000198205|transcript|
processed_pseudogene			CT|splice_acceptor_variant&intron_variant|HIGH|MORF4L1P5|ENS
protein_co	2	0.5	TA|splice_acceptor_variant&intron_variant|HIGH|ZC3H12B|ENSG00
protein_coding			C|structural_interaction_variant|HIGH|MSN|ENSG00000147065|int
protein_coding			T|stop_gained|HIGH|P2RY4|ENSG00000186912|transcript|ENST000
protein_co	5	0.2	ATTT|splice_acceptor_variant&intron_variant|HIGH|GDPD2|ENSG0
protein_co	5	0.2	ATTT|splice_acceptor_variant&intron_variant|HIGH|GDPD2|ENSG0
protein_co	5	0.2	ATTT|splice_acceptor_variant&intron_variant|HIGH|GDPD2|ENSG0
protein_coding			A|structural_interaction_variant|HIGH|TAF1|ENSG00000147133|in
nonsense_	7	0.14	ATT|splice_acceptor_variant&intron_variant|HIGH|PIN4|ENSG0000
nonsense_	7	0.14	ATT|splice_acceptor_variant&intron_variant|HIGH|PIN4|ENSG0000
protein_coding			T|structural_interaction_variant|HIGH|HDAC8|ENSG00000147099|
protein_co	3	0.33	C|splice_acceptor_variant&intron_variant|HIGH|KIAA2022|ENSG00

nonsense_	8	0.13	A|splice_donor_variant&intron_variant|HIGH|UPRT|ENSG0000009
protein_coding			A|stop_gained|HIGH|MAGEE2|ENSG00000186675|transcript|ENST
unprocessed_pseudogene			GT|splice_donor_variant&intron_variant|HIGH|RP13-52K8.1|ENSG
protein_co	3	1	CTT|splice_acceptor_variant&intron_variant|HIGH|ZNF711|ENSG00
protein_co	3	1	CTT|splice_acceptor_variant&intron_variant|HIGH|ZNF711|ENSG00
protein_co	3	1	CTT|splice_acceptor_variant&intron_variant|HIGH|ZNF711|ENSG00
processed_transcript			C|splice_acceptor_variant&intron_variant|HIGH|CHM|ENSG000001
processed_transcript			C|splice_acceptor_variant&intron_variant|HIGH|CHM|ENSG000001
processed_transcript			C|splice_acceptor_variant&intron_variant|HIGH|CHM|ENSG000001
protein_coding			C|3_prime_UTR_variant|MODIFIER|CHM|ENSG00000188419|trans
protein_coding			C|stop_lost|HIGH|PCDH11X|ENSG00000102290|transcript|ENST00
protein_co	5	1	GT|splice_acceptor_variant&intron_variant|HIGH|DIAPH2|ENSG000
protein_co	5	1	GT|splice_acceptor_variant&intron_variant|HIGH|DIAPH2|ENSG000
protein_co	5	0.2	CTTTT|splice_acceptor_variant&intron_variant|HIGH|CENPI|ENSG0
protein_co	5	0.2	CTTTT|splice_acceptor_variant&intron_variant|HIGH|CENPI|ENSG0
protein_co	5	0.2	CTTTT|splice_acceptor_variant&intron_variant|HIGH|CENPI|ENSG0
protein_co	5	0.2	CTTTT|splice_acceptor_variant&intron_variant|HIGH|CENPI|ENSG0
protein_coding			A|structural_interaction_variant|HIGH|BTK|ENSG00000010671|inte
protein_coding			TG|frameshift_variant|HIGH|TCEAL6|ENSG00000204071|transcript
protein_co	3	0.33	G|frameshift_variant|HIGH|TCP11X2|ENSG00000215029|transcript
protein_co	3	0.67	T|stop_gained|HIGH|H2BFM|ENSG00000101812|transcript|ENST0
protein_co	7	0.14	ATTTTTT|splice_acceptor_variant&intron_variant|HIGH|TBC1D8B|E
protein_co	7	0.14	ATTTTTT|splice_acceptor_variant&intron_variant|HIGH|TBC1D8B|E
protein_co	7	0.14	ATTTTTT|splice_acceptor_variant&intron_variant|HIGH|TBC1D8B|E
protein_co	3	1	AT|splice_acceptor_variant&intron_variant|HIGH|CLDN2|ENSG000
protein_coding			C|frameshift_variant|HIGH|LRCH2|ENSG00000130224|transcript|E
protein_co	18	0.33	CTT|splice_acceptor_variant&intron_variant|HIGH|STAG2|ENSG000
protein_co	18	0.33	CTT|splice_acceptor_variant&intron_variant|HIGH|STAG2|ENSG000
protein_co	18	0.33	CTTT|splice_acceptor_variant&intron_variant|HIGH|STAG2|ENSG00
protein_co	18	0.33	CTTT|splice_acceptor_variant&intron_variant|HIGH|STAG2|ENSG00
protein_co	18	0.33	CTTT|splice_acceptor_variant&intron_variant|HIGH|STAG2|ENSG00
protein_co	18	0.33	CTTT|splice_acceptor_variant&intron_variant|HIGH|STAG2|ENSG00
protein_co	18	0.33	CTTT|splice_acceptor_variant&intron_variant|HIGH|STAG2|ENSG00
nonsense_mediated_decay			G|stop_lost|HIGH|AIFM1|ENSG00000156709|transcript|ENST0000
transcribed_unprocessed_pseudo			A|splice_donor_variant&intron_variant|HIGH|RP1-274L7.4|ENSG00
transcribed_unprocessed_pseudo			CTT|splice_acceptor_variant&intron_variant|HIGH|RP1-274L7.4|EN
transcribed_unprocessed_pseudo			C|splice_region_variant&non_coding_transcript_exon_variant|LOW
transcribed_unprocessed_pseudo			G|splice_donor_variant&intron_variant|HIGH|RP1-274L7.4|ENSG00
protein_coding			G|frameshift_variant|HIGH|HS6ST2|ENSG00000171004|transcript|
processed_pseudogene			CT|splice_acceptor_variant&splice_region_variant&intron_variant&
processed_pseudogene			CTT|splice_acceptor_variant&splice_region_variant&intron_variant
processed_pseudogene			CTTT|splice_acceptor_variant&splice_region_variant&intron_variant
processed_pseudogene			CTTTT|splice_acceptor_variant&splice_region_variant&intron_varia
processed_pseudogene			C|splice_donor_variant&intron_variant|HIGH|RP11-432N13.2|ENSG
protein_co	33	0.03	C|splice_donor_variant&intron_variant|HIGH|FHL1|ENSG00000022
protein_co	12	0.25	GAA|frameshift_variant|HIGH|RBMX|ENSG00000147274|transcript
protein_co	12	0.5	AT|frameshift_variant&start_lost|HIGH|RBMX|ENSG00000147274|

protein_coding			T|frameshift_variant|HIGH|RBMX|ENSG00000147274|transcript|EN
protein_co	2	0.5	G|frameshift_variant|HIGH|MAGEC1|ENSG00000155495|transcript
protein_co	2	0.5	A|frameshift_variant|HIGH|MAGEC1|ENSG00000155495|transcript
protein_co	2	0.5	T|frameshift_variant|HIGH|MAGEC1|ENSG00000155495|transcript
protein_co	2	0.5	T|intron_variant|MODIFIER|MAGEC1|ENSG00000155495|transcript
protein_co	2	0.5	A|frameshift_variant|HIGH|MAGEC1|ENSG00000155495|transcript
protein_co	2	0.5	A|intron_variant|MODIFIER|MAGEC1|ENSG00000155495|transcript
protein_co	1	1	A|frameshift_variant|HIGH|SPANXN2|ENSG00000268988|transcript
polymorphic_pseudogene			T|stop_lost|HIGH|UBE2NL|ENSG00000276380|transcript|ENST0000
protein_co	5	0.2	C|splice_donor_variant&intron_variant|HIGH|PRRG3|ENSG0000013
antisense			C|splice_donor_variant&intron_variant|HIGH|RP11-366F6.2|ENSG0
protein_coding			T|structural_interaction_variant|HIGH|MAGEA4|ENSG00000147381
protein_co	4	0.5	C|stop_gained|HIGH|CSAG1|ENSG00000198930|transcript|ENST00
retained_ir	32	0.03	T|splice_donor_variant&intron_variant|HIGH|PNCK|ENSG0000013C
protein_coding			CA|frameshift_variant|HIGH|SLC6A8|ENSG00000130821|transcript
protein_co	8	0.13	A|splice_donor_variant&intron_variant|HIGH|BCAP31|ENSG000001
protein_coding			GTCCCC|frameshift_variant|HIGH|SRPK3|ENSG00000184343|transc
protein_co	24	0.25	C|splice_donor_variant&intron_variant|HIGH|ARHGAP4|ENSG00000
protein_coding			G|structural_interaction_variant|HIGH|G6PD|ENSG00000160211|ir
unprocessed_pseudogene			T|splice_donor_variant&intron_variant|HIGH|MAFIP|ENSG0000027
transcribed_unprocessed_pseudo			C|splice_acceptor_variant&splice_region_variant&intron_variant|HI
transcribed_unprocessed_pseudo			C|splice_acceptor_variant&splice_region_variant&intron_variant|HI
protein_coding			T|structural_interaction_variant|HIGH|UTY|ENSG00000183878|inte

_variant&intron_variant|HIGH|SAMD11|ENSG00000187634|transcript|ENST00000455979.1|protein_
iteraction|1Z2M:A_79-A_101:ENST00000379389|protein_coding|2/2|c.237C>T||||||,T|structural_int
iteraction|1Z2M:A_83-A_145:ENST00000379389|protein_coding|2/2|c.248G>A||||||,A|structural_in
i00000217801|transcript|ENST00000427998.5|processed_transcript|3/3|n.350+1G>C||||||,C|splice_
0000429572.5|protein_coding|4/4|c.850C>T|p.Gln284*|908/1104|850/870|284/289||,A|upstream_g
GH|CCNL2|ENSG00000221978|transcript|ENST00000400809.7|protein_coding|1/10|c.289-6_289-2de
nscript|ENST00000624594.1|protein_coding|3/3|c.250_251insAT|p.Ile84fs|251/276|251/276|84/91|
nscript|ENST00000624594.1|protein_coding|3/3|c.250_251insAT|p.Ile84fs|251/276|251/276|84/91|
nscript|ENST00000624594.1|protein_coding|3/3|c.250_251insAT|p.Ile84fs|251/276|251/276|84/91|
504599.5|protein_coding|2/19|c.2T>C|p.Met1?|329/3311|2/2910|1/969||,C|start_lost|HIGH|MIB2|
ENST00000407249.7|protein_coding|9/20|c.1042delT|p.Ser348fs|1154/2677|1042/2388|348/795||,
248333|transcript|ENST00000407249.7|protein_coding|1/19|c.-14+2T>G||||||,C|splice_donor_variar
|FAAP20|ENSG00000162585|transcript|ENST00000487186.5|processed_transcript|1/7|n.172+2_172
teraction|2XWC:A_173-A_240:ENST00000378295|protein_coding|5/14|c.519C>T||||||,T|structural_i
0227372|transcript|ENST00000624948.1|processed_transcript|3/3|n.463+1G>A||||||,T|upstream_ge
0000479239.1|nonsense_mediated_decay|2/5|c.96C>A|p.Tyr32*|98/434|96/150|32/49||WARNING_
i00000232596|transcript|ENST00000420522.1|lincRNA|1/4|n.83-2A>G||||||,G|splice_acceptor_varia
0131697|transcript|ENST00000378156.8|protein_coding|20/29|c.2818-2A>T||||||,A|splice_acceptor
|interaction|1ZSX:A_42-A_71:ENST00000352527|protein_coding|5/15|c.213T>C||||||,C|structural_ir
049246|transcript|ENST00000614998.4|protein_coding|19/22|c.3002-2A>G||||||,G|synonymous_var
nteraction|1PDW:A_66-A_98:ENST00000338639|protein_coding|5/7|c.293G>A||||||,A|structural_in
eraction|3FE4:A_76-A_197:ENST00000377436|protein_coding|2/8|c.228G>C||||||,C|structural_intera
IST00000602477.1|protein_coding|2/5|c.27_34delCCCAGGCA|p.Pro10fs|179/5590|27/2409|9/802||
0175262|transcript|ENST00000418570.6|protein_coding|6/7|c.792+2C>T||||||WARNING_TRANSCRIP
0215785|transcript|ENST00000400899.2|processed_pseudogene|1/1|n.381-1G>C||||||
116670|transcript|ENST00000376669.9|protein_coding|5/8|c.371+2T>G||||||,C|splice_donor_variar
00116670|transcript|ENST00000235310.7|protein_coding|1/10|c.-691-1G>A||||||,T|upstream_gene
T00000444493.5|protein_coding|4/10|c.793C>T|p.Gln265*|794/1881|793/1848|265/615||WARNING
376480.7|protein_coding|3/3|c.454T>C|p.Ter152Argext*?|553/855|454/456|152/151||,G|stop_lost
T00000359318.7|protein_coding|1/4|c.39C>A|p.Cys13*|73/3662|39/1224|13/407||,A|non_coding_tı
T00000240189.2|protein_coding|3/4|c.631G>T|p.Glu211*|718/1642|631/1425|211/474||
ipt|ENST00000235349.5|protein_coding|4/4|c.1255dupC|p.Arg419fs|1326/1828|1255/1437|419/478
GH|RP1-21O18.3|ENSG00000215720|transcript|ENST00000400795.3|processed_pseudogene|1/1|n.9
ENST00000546424.5|protein_coding|8/8|c.1237dupG|p.Val413fs|1482/4463|1237/1266|413/421||,
i00000116138|transcript|ENST00000495523.1|processed_transcript|1/1|n.78-3dupT||||||INFO_REAI
i00000116138|transcript|ENST00000495523.1|processed_transcript|1/1|n.78-3dupT||||||INFO_REAI
i00000116138|transcript|ENST00000495523.1|processed_transcript|1/1|n.78-3dupT||||||INFO_REAI
i00000116138|transcript|ENST00000495523.1|processed_transcript|1/1|n.78-3dupT||||||INFO_REAI
ript|ENST00000375782.1|protein_coding|5/6|c.920_921insGA|p.Leu308fs|1059/1520|921/1032|307/
ot|ENST00000375782.1|protein_coding|5/6|c.924dupG|p.Leu309fs|1063/1520|925/1032|309/343||
ST00000375759.7|protein_coding|11/15|c.9748_9749delGT|p.Val3250fs|9952/12232|9748/10995|3
teraction|1OW1:A_3576-A_3659:ENST00000375759|protein_coding|15/15|c.10977T>A||||||,A|syno
nteraction|2X10:A_438-A_462:ENST00000358432|protein_coding|6/17|c.1314G>A||||||,T|splice_reg
interaction|3FL7:A_329-A_426:ENST00000358432|protein_coding|5/17|c.987C>T||||||,A|synonymou

nteraction|2X10:A_67-A_191:ENST00000358432|protein_coding|3/17|c.573G>A||||||,T|structural_i
nteraction|2X10:A_101-A_190:ENST00000358432|protein_coding|3/17|c.570G>A||||||,T|structural_
9.7|ENSG00000279151|transcript|ENST00000624517.1|lincRNA|2/2|n.271-7_271-3dupTTTTT|||||IN
9.7|ENSG00000279151|transcript|ENST00000624517.1|lincRNA|2/2|n.271-7_271-3dupTTTTT|||||IN
9.7|ENSG00000279151|transcript|ENST00000624517.1|lincRNA|2/2|n.271-7_271-3dupTTTTT|||||IN
9.7|ENSG00000279151|transcript|ENST00000624517.1|lincRNA|2/2|n.271-7_271-3dupTTTTT|||||IN
9.7|ENSG00000279151|transcript|ENST00000624517.1|lincRNA|2/2|n.271-7_271-3dupTTTTT|||||IN
9.7|ENSG00000279151|transcript|ENST00000624517.1|lincRNA|2/2|n.271-7_271-3dupTTTTT|||||IN
G00000228549|transcript|ENST00000453554.1|lincRNA|1/1|n.1224+1dupG|||||INFO_REALIGN_3_P
G00000228549|transcript|ENST00000453554.1|lincRNA|1/1|n.1224+1dupG|||||INFO_REALIGN_3_P
nteraction|4N20:A_428-A_458:ENST00000375486|protein_coding|12/16|c.1374G>A||||||,T|structura
intron_variant|HIGH|AC004824.1|ENSG00000280226|transcript|ENST00000624583.1|protein_coding
nteraction|3APM:A_80-A_112:ENST00000375448|protein_coding|3/16|c.335G>C||||||,C|structural_i
nteraction|1WDA:A_309-A_648:ENST00000375448|protein_coding|8/16|c.926A>G||||||,G|structura
nteraction|1WDA:A_341-A_383:ENST00000375448|protein_coding|10/16|c.1149C>A||||||,A|structu
3|interaction|3V9G:A_313-A_528:ENST00000290597|protein_coding|15/16|c.1583C>A||||||,T|struc
3|interaction|3V9H:A_445-A_473:ENST00000290597|protein_coding|13/16|c.1417A>G||||||,C|struc
3|interaction|3V9G:A_353-A_470:ENST00000290597|protein_coding|13/16|c.1408G>A||||||,T|struc
3|interaction|3V9G:A_359-A_460:ENST00000290597|protein_coding|13/16|c.1380T>C||||||,G|struc
3|interaction|3V9G:A_410-A_441:ENST00000290597|protein_coding|12/16|c.1230A>G||||||,C|struc
3|interaction|3V9G:A_319-A_350:ENST00000290597|protein_coding|10/16|c.1050G>C||||||,G|struc
ENST00000375225.7|protein_coding|1/4|c.6dupG|p.Pro3fs|440/1533|6/777|2/258||,GC|upstream_g
NST00000429712.5|polymorphic_pseudogene|2/7|c.369delG|p.Thr124fs|489/1313|369/996|123/331
0429712.5|polymorphic_pseudogene|2/7|c.316T>C|p.Ter106Argext*?|436/1313|316/996|106/331||
NST00000429712.5|polymorphic_pseudogene|1/7|c.68_90delTGGACGCGCCCACCAGCGCCGCA|p.Met2
egion_variant&intron_variant|HIGH|PQLC2|ENSG00000040487|transcript|ENST00000432465.1|prote
script|ENST00000602293.5|nonsense_mediated_decay|2/9|c.32_35delACAG|p.His11fs|114/820|32/2
00127472|transcript|ENST00000469069.5|processed_transcript|2/6|n.246-1G>A||||||,A|intron_varia
00000247992.5|protein_coding|1/3|c.106C>T|p.Arg36*|106/453|106/453|36/150||,A|stop_gained|
GH|RP5-930J4.5|ENSG00000235432|transcript|ENST00000440621.1|unprocessed_pseudogene|3/3|n
GH|RP5-930J4.5|ENSG00000235432|transcript|ENST00000440621.1|unprocessed_pseudogene|3/3|n
GH|RP5-930J4.5|ENSG00000235432|transcript|ENST00000440621.1|unprocessed_pseudogene|3/3|n
0000471600.6|nonsense_mediated_decay|22/25|c.1807T>C|p.Ter603Glnext*?|1997/3238|1807/1809
4|interaction|1SRQ:C_312-C_381:ENST00000374765|protein_coding|16/25|c.1143G>A||||||,T|struc
NST00000529637.5|protein_coding|16/27|c.1975delT|p.Ser659fs|2009/3228|1975/3144|659/1047|
NST00000529637.5|protein_coding|16/27|c.1975delT|p.Ser659fs|2009/3228|1975/3144|659/1047|
19073|transcript|ENST00000337107.10|protein_coding|2/7|c.129+1G>A||||||,A|splice_donor_varia
00218510|transcript|ENST00000634451.1|lincRNA|2/2|n.214+1C>G||||||,G|upstream_gene_variant
iant&intron_variant&non_coding_transcript_exon_variant|HIGH|LINC00339|ENSG00000218510|trans
iant&intron_variant&non_coding_transcript_exon_variant|HIGH|LINC00339|ENSG00000218510|trans
_coding_transcript_exon_variant|HIGH|LINC00339|ENSG00000218510|transcript|ENST00000404210.
_coding_transcript_exon_variant|HIGH|LINC00339|ENSG00000218510|transcript|ENST00000404210.
0162552|transcript|ENST00000415567.1|protein_coding|1/1|c.236-1G>C|||||WARNING_TRANSCRIP
00000426928.6|protein_coding|6/6|c.686C>A|p.Ser229*|686/1641|686/1641|229/546||,T|stop_gai
nteraction|2B0O:E_455-E_611:ENST00000336689|protein_coding|18/25|c.1833C>T||||||,A|structura
nteraction|2B0O:E_454-E_477:ENST00000336689|protein_coding|15/25|c.1431C>T||||||,A|structura
SG00000229239|transcript|ENST00000451300.2|unprocessed_pseudogene|2/6|n.173-2A>T|||||

)000374487.6|nonsense_mediated_decay|1/10|c.159G>A|p.Trp53*|481/2151|159/234|53/77||
|interaction|3DGC:R_23-R_45:ENST00000270800|protein_coding|2/7|c.135G>A||||||,T|synonymous
NSG00000231953|transcript|ENST00000455902.2|antisense|1/1|n.85-3dupT||||||INFO_REALIGN_3_
)0000231953|transcript|ENST00000455902.2|antisense|1/1|n.85-3delT||||||INFO_REALIGN_3_PRIM
ENST00000374253.9|protein_coding|9/21|c.872delC|p.Pro291fs|911/2538|872/2163|291/720||INF
ENST00000374223.5|protein_coding|12/12|c.792_798delCGGCCCC|p.Gly265fs|1259/1312|792/834|
ENST00000374223.5|protein_coding|12/12|c.792_798delCGGCCCC|p.Gly265fs|1259/1312|792/834|
|ENST00000374223.5|protein_coding|12/12|c.772_788delCCCAGTCCCGGTCCCGG|p.Pro258fs|1249/13
|ENST00000374223.5|protein_coding|12/12|c.772_788delCCCAGTCCCGGTCCCGG|p.Pro258fs|1249/13
ENST00000374223.5|protein_coding|12/12|c.766_775delCCCGGCCCCA|p.Pro256fs|1236/1312|766/8
ENST00000374223.5|protein_coding|12/12|c.766_775delCCCGGCCCCA|p.Pro256fs|1236/1312|766/8
|ENST00000374223.5|protein_coding|12/12|c.757_764delGGTCCCGG|p.Gly253fs|1225/1312|757/834
|ENST00000374223.5|protein_coding|12/12|c.757_758delGG|p.Gly253fs|1219/1312|757/834|253/27
|ENST00000374223.5|protein_coding|12/12|c.757_758delGG|p.Gly253fs|1219/1312|757/834|253/27
|ENST00000374223.5|protein_coding|12/12|c.728_755delGTCCCGGTCCCGGTCCCGGCCCCAGTCC|p.Cys
NST00000308182.9|protein_coding|2/20|c.1614_1626delTGATGCCTCATTT|p.Asp539fs|1776/5245|16
NST00000308182.9|protein_coding|2/20|c.1607_1610delGTGC|p.Gly536fs|1760/5245|1607/4986|53
NST00000308182.9|protein_coding|2/20|c.1571_1604delTGTTCCCCACCTGGAAAGAGGTCGTGAAGGGC
|ENST00000374163.5|nonsense_mediated_decay|6/22|c.560_563delTATT|p.Leu187fs|619/3202|560
)00374163.5|nonsense_mediated_decay|7/22|c.613T>C|p.Ter205Argext*?|672/3202|613/615|205/2
5|interaction|3RNY:B_466-B_563:ENST00000374168|protein_coding|16/22|c.1398T>C||||||,C|struct
2765|transcript|ENST00000543823.1|protein_coding|8/14|c.888_900dupCCGGCGCCGCCGC|p.Ser301f
IST00000270879.8|protein_coding|5/8|c.349delC|p.Leu117fs|355/1030|349/900|117/299||,A|frame
pt|ENST00000411604.5|protein_coding|2/7|c.110dupC|p.Thr38fs|256/953|111/808|37/268||WARN
pt|ENST00000411604.5|protein_coding|2/7|c.109_110insG|p.Pro37fs|255/953|110/808|37/268||W
nteraction|2VCY:B_193-B_224:ENST00000263702|protein_coding|6/10|c.670C>T||||||,A|synonymou
121753|transcript|ENST00000533175.1|protein_coding|1/5|c.123+1G>A||||||WARNING_TRANSCRIP
84007|transcript|ENST00000532289.1|processed_transcript|1/1|n.45+2T>G||||||,C|upstream_gene
t|ENST00000409358.1|protein_coding|1/9|c.52dupG|p.Asp18fs|53/1349|53/1050|18/349||INFO_RE
nteraction|2XU7:B_151-B_171:ENST00000373493|protein_coding|4/12|c.453T>C||||||,C|structural_
ript|ENST00000373480.1|protein_coding|6/7|c.2130_2131insGC|p.Pro711fs|2234/5294|2131/3108|
eraction|2C9Y:A_129-A_210:ENST00000354858|protein_coding|4/6|c.386G>A||||||,T|structural_inte
eraction|2C9Y:A_18-A_96:ENST00000354858|protein_coding|1/6|c.54C>T||||||,A|structural_interact
|ENST00000442999.3|protein_coding|4/5|c.315_318delGACT|p.Thr106fs|318/1023|315/1023|105/3
TA|frameshift_variant|HIGH|HMGB4|ENSG00000176256|transcript|ENST00000519684.5|protein_cod
ST00000339480.1|protein_coding|2/2|c.155_158delTCTG|p.Val52fs|525/2840|155/801|52/266||INF
)0000241014|transcript|ENST00000311990.4|lincRNA|1/2|n.29+2T>G||||||,C|downstream_gene_va
)0183317|transcript|ENST00000432874.7|nonsense_mediated_decay|4/15|c.380-1G>A||||||WARNI
interaction|3MTC:A_291-A_331:ENST00000373024|protein_coding|10/24|c.873C>A||||||,T|structur
)0000226438|transcript|ENST00000289890.7|antisense|2/2|n.193+2C>T||||||,A|intron_variant|MO
NST00000486825.6|nonsense_mediated_decay|2/8|c.198dupT|p.Gly67fs|200/850|198/243|66/80||
teraction|3GRO:A_109-A_134:ENST00000433473|protein_coding|4/9|c.401T>C||||||,G|structural_in
0000188800|transcript|ENST00000468258.1|processed_transcript|1/2|n.37-3dupT||||||INFO_REAL
0000188800|transcript|ENST00000468258.1|processed_transcript|1/2|n.37-3dupT||||||INFO_REAL
0000188800|transcript|ENST00000468258.1|processed_transcript|1/2|n.37-3dupT||||||INFO_REAL
pt|ENST00000372759.3|protein_coding|4/10|c.443_444delTG|p.Val148fs|608/3108|443/1428|148/4
NST00000358527.6|protein_coding|4/4|c.1029_1030insG|p.Arg344fs|1430/1892|1030/1122|344/37

nteraction|2VO1:A_167-A_208:ENST00000372621|protein_coding|5/19|c.501A>G||||||,G|structural_
‹O6|ENSG00000204060|transcript|ENST00000372591.1|protein_coding|2/2|c.1008_1009insGGGACGC
GGCGGGGGCTT|frameshift_variant|HIGH|FOXO6|ENSG00000204060|transcript|ENST00000372591.1
0000234694|transcript|ENST00000424948.1|antisense|1/2|n.240+1G>A||||||,T|upstream_gene_vari
00066135|transcript|ENST00000372396.3|protein_coding|3/21|c.315-2delA||||||INFO_REALIGN_3_P
tion|2XWU:A_59-B_415:ENST00000325437-ENST00000372343|protein_coding|5/20|c.1245C>T|||||
00159214|transcript|ENST00000486064.5|nonsense_mediated_decay|2/7|c.127-2_127-1delAG||||||
interaction|3HM5:A_137-A_206:ENST00000315913|protein_coding|5/11|c.409G>C||||||,C|structura
l|AL355480.2|ENSG00000281112|transcript|ENST00000628958.1|pseudogene|2/2|n.57+1C>T||||||,
GH|MAST2|ENSG00000086015|transcript|ENST00000372009.3|protein_coding|19/22|c.2370+2A>G|
interaction|4FP9:A_97-A_131:ENST00000474844|protein_coding|2/6|c.291T>C||||||,C|structural_in
0000271139.12|protein_coding|1/4|c.70C>T|p.Arg24*|127/2800|70/807|24/268||,A|5_prime_UTR_
000142973|transcript|ENST00000371923.8|protein_coding|8/12|c.884_885delAT|p.Asp295fs|920/21
0000464439.6|nonsense_mediated_decay|8/12|c.923G>A|p.Trp308*|941/2019|923/978|308/325||,
_coding_transcript_exon_variant|HIGH|RP4-683M8.2|ENSG00000226133|transcript|ENST00000456800
ipt|ENST00000622918.1|protein_coding|1/1|c.191_192delGT|p.Cys64fs|1757/2640|191/573|64/190
ipt|ENST00000622918.1|protein_coding|1/1|c.191_192delGT|p.Cys64fs|1757/2640|191/573|64/190
0000236495.9|protein_coding|14/15|c.1852G>T|p.Glu618*|1902/3272|1852/2121|618/706||,T|stop
ENST00000262676.9|protein_coding|11/11|c.1103delG|p.Gly368fs|1264/6287|1103/1263|368/420|
ENST00000262676.9|protein_coding|11/11|c.1103delG|p.Gly368fs|1264/6287|1103/1263|368/420|
ENST00000262676.9|protein_coding|11/11|c.1103delG|p.Gly368fs|1264/6287|1103/1263|368/420|
000237663|transcript|ENST00000428811.2|processed_pseudogene|1/1|n.106+1C>T||||||,A|upstrea
00612017.4|protein_coding|3/14|c.1A>G|p.Met1?|648/3682|1/1254|1/417||,G|start_lost|HIGH|ZY
00000277397|transcript|ENST00000617613.1|unprocessed_pseudogene|1/1|n.102+2T>A|||||
G00000277397|transcript|ENST00000617613.1|unprocessed_pseudogene|1/1|n.103-1G>A|||||
3799|transcript|ENST00000072644.5|protein_coding|7/10|c.481+2T>C||||||,G|splice_donor_variant
ENST00000371330.1|protein_coding|4/4|c.1224dupC|p.Met409fs|2072/2723|1224/1350|408/449||
ENST00000371330.1|protein_coding|4/4|c.1224dupC|p.Met409fs|2072/2723|1224/1350|408/449||
ENST00000371330.1|protein_coding|4/4|c.1223_1224insG|p.Met409fs|2071/2723|1223/1350|408/4
|interaction|3FO5:A_408-A_570:ENST00000343744|protein_coding|12/16|c.1223C>T||||||,T|structu
|interaction|3FO5:A_391-A_493:ENST00000343744|protein_coding|14/16|c.1479G>A||||||,A|struct
|interaction|3FO5:A_425-A_549:ENST00000343744|protein_coding|16/16|c.1647G>A||||||,A|struct
t|ENST00000302250.6|protein_coding|7/8|c.1003_1031delCCTGGGGATGACGGTCTGAATGTGGAGTG|p
ENST00000414150.6|nonsense_mediated_decay|25/33|c.3846T>A|p.Cys1282*|4124/5867|3846/400
000371274.8|protein_coding|6/6|c.1024C>T|p.Arg342*|1059/2149|1024/1119|342/372||,A|downst
ENST00000371273.3|protein_coding|7/10|c.770dupA|p.Pro258fs|786/1651|771/1257|257/418||INF
nteraction|2P4E:A_460-A_524:ENST00000302118|protein_coding|9/12|c.1380A>G||||||,G|structura
_coding_transcript_exon_variant|HIGH|RP11-101C11.1|ENSG00000231090|transcript|ENST0000451
NSG00000260971|transcript|ENST00000569425.3|lincRNA|2/2|n.445-1dupG|||||
|interaction|2H6D:A_117-A_267:ENST00000371244|protein_coding|4/9|c.351G>A||||||,A|structural
0371237.8|protein_coding|9/12|c.1282C>T|p.Arg428*|1349/2037|1282/1776|428/591||,A|stop_gai
0226759|transcript|ENST00000591173.5|antisense|4/4|n.475+2T>C||||||,C|downstream_gene_vari
00000232453|transcript|ENST00000423408.5|lincRNA|1/3|n.119+1G>C||||||,G|intron_variant|MOD
0000226883|transcript|ENST00000443012.1|antisense|1/1|n.106+2T>G||||||,C|3_prime_UTR_varia
62599|transcript|ENST00000403491.7|protein_coding|8/10|c.1255-2A>C||||||,C|splice_acceptor_va
ENST00000316485.11|protein_coding|26/26|c.3532delA|p.Arg1178fs|3646/3657|3532/3543|1178/1
ENST00000316485.11|protein_coding|26/26|c.3532delA|p.Arg1178fs|3646/3657|3532/3543|1178/1

)0613764.4|protein_coding|26/29|c.3400G>T|p.Glu1134*|3497/4172|3400/3549|1134/1182||,T|ups
NST00000498273.1|protein_coding|4/4|c.1805_1808delTAAC|p.Leu602fs|2100/3849|1805/2598|602
)00227485|transcript|ENST00000418091.1|lincRNA|1/1|n.104+2C>T|||||
)0224209|transcript|ENST00000634725.1|lincRNA|7/10|n.1155+2A>T|||||,A|downstream_gene_va
9739|transcript|ENST00000371083.4|protein_coding|1/10|c.300+1G>A|||||,A|intron_variant|MOD
teraction|4L00:A_697-A_733:ENST00000342505|protein_coding|16/25|c.2199A>G|||||,C|structural_
teraction|4L00:A_585-A_659:ENST00000342505|protein_coding|14/25|c.1977C>T|||||,A|synonymo
0000226891|transcript|ENST00000415842.5|lincRNA|1/5|n.149-1G>C|||||,G|splice_acceptor_varia
nteraction|3G45:A_435-A_552:ENST00000329654|protein_coding|13/17|c.1305G>A|||||,A|structur
)0152760|transcript|ENST00000282670.6|protein_coding|4/4|c.336+1G>A|||||,A|splice_donor_vari
:|ENST00000371006.5|protein_coding|6/6|c.1017_1021delAATGA|p.Met340fs|1042/2053|1017/120
)00232284|transcript|ENST00000420587.5|antisense|9/9|n.1213+1G>A|||||,A|splice_donor_varian
cript|ENST00000624855.1|protein_coding|3/3|c.54_55insA|p.Leu19fs|54/57|54/57|18/18||WARNIN
eraction|2NMP:B_42-B_67:ENST00000370938|protein_coding|2/12|c.200C>T|||||,T|structural_inte
i00000229956|transcript|ENST00000413421.5|lincRNA|4/7|n.268-2A>T|||||,T|splice_acceptor_vari
)0233973|transcript|ENST00000440762.5|lincRNA|2/3|n.301+2T>C|||||,C|splice_donor_variant&in
teraction|1YB5:A_158-A_323:ENST00000340866|protein_coding|9/9|c.969A>T|||||,A|structural_int
)479111.5|protein_coding|5/7|c.1A>G|p.Met1?|424/1130|1/420|1/139||,G|start_lost|HIGH|TYW3|
7254|transcript|ENST00000524536.5|protein_coding|2/8|c.-138+1G>A|||||WARNING_TRANSCRIPT_
NST00000446486.1|protein_coding|1/2|c.149_150insT|p.Trp50fs|152/497|150/228|50/75||WARNIN
GH|RP5-837I24.2|ENSG00000234953|transcript|ENST00000443939.1|antisense|3/3|n.478-122_478-
00230817|transcript|ENST00000452901.5|lincRNA|9/10|n.1539+1A>G|||||
T00000370665.3|protein_coding|3/6|c.346A>T|p.Lys116*|379/1269|346/1086|116/361||,T|5_prime
00000137975|transcript|ENST00000490884.1|processed_transcript|3/3|n.379-3dupT|||||INFO_REA
00000137975|transcript|ENST00000490884.1|processed_transcript|3/3|n.379-3dupT|||||INFO_REA
00000137975|transcript|ENST00000490884.1|processed_transcript|3/3|n.379-3dupT|||||INFO_REA
00000137975|transcript|ENST00000490884.1|processed_transcript|3/3|n.379-3dupT|||||INFO_REA
53923|transcript|ENST00000466454.1|processed_transcript|4/14|n.575+2T>C|||||,C|splice_donor_
)0000236915|transcript|ENST00000456587.1|antisense|2/3|n.183+1A>G|||||,C|non_coding_transc
3291|transcript|ENST00000469566.5|processed_transcript|1/4|n.110+2C>A|||||,T|5_prime_UTR_v
nteraction|4CRS:A_670-A_956:ENST00000370521|protein_coding|14/22|c.2010G>A|||||,A|splice_re
)0000321792.5|protein_coding|2/2|c.642T>A|p.Tyr214*|1070/4799|642/1173|214/390||,T|stop_ga
|transcript|ENST00000321792.5|protein_coding|2/2|c.1delA|p.Met1fs|429/4799|1/1173|1/390||,A|
teraction|1F5N:A_239-A_265:ENST00000370473|protein_coding|6/11|c.793C>A|||||,T|structural_i
teraction|1DG3:A_13-A_78:ENST00000370473|protein_coding|3/11|c.232A>G|||||,C|structural_int
IST00000294671.2|protein_coding|10/11|c.1637delG|p.Arg546fs|1776/2454|1637/1917|546/638||,T
NST00000294671.2|protein_coding|10/11|c.1633_1634insA|p.Leu545fs|1772/2454|1633/1917|545/
IST00000370459.7|protein_coding|8/11|c.1214delG|p.Cys405fs|1342/2011|1214/1761|405/586||,G
i4451|transcript|ENST00000370459.7|protein_coding|5/11|c.502_505dupGACT|p.Ser169fs|633/2011
ISG00000237435|transcript|ENST00000595613.5|lincRNA|4/5|n.361-1G>C|||||,C|splice_acceptor_v
i00000226053|transcript|ENST00000418344.1|lincRNA|1/2|n.64+1G>A|||||
)0162627|transcript|ENST00000306121.7|protein_coding|5/8|c.839-3dupT|||||INFO_REALIGN_3_P
)0162627|transcript|ENST00000306121.7|protein_coding|5/8|c.839-3dupT|||||INFO_REALIGN_3_P
52688|transcript|ENST00000370165.7|protein_coding|1/33|c.-8-2A>G|||||,G|5_prime_UTR_prema
)0230402|transcript|ENST00000416343.2|lincRNA|1/2|n.125+1G>A|||||
0989|transcript|ENST00000475821.1|processed_transcript|1/1|n.65+1delG|||||,A|5_prime_UTR_v
40038|transcript|ENST00000361355.8|protein_coding|2/11|c.-47+2T>C|||||,C|splice_donor_varian

.2|ENSG00000215869|transcript|ENST00000418362.1|processed_transcript|1/1|n.883-2_883-1insAA
interaction|4Y30:A_194-A_289:ENST00000370078|protein_coding|1/1|c.581C>T||||||,T|structural_i
teraction|3VBB:A_223-A_297:ENST00000234677|protein_coding|7/11|c.891G>T||||||,T|structural_i
000369904.7|protein_coding|6/8|c.745A>T|p.Lys249*|831/1584|745/933|249/310||,A|synonymous
00000357155.1|protein_coding|6/9|c.763C>T|p.Arg255*|813/1372|763/1065|255/354||,A|intron_v
nteraction|2IHB:A_35-A_216:ENST00000369851|protein_coding|1/9|c.105G>A||||||,A|structural_in
NSG00000258673|transcript|ENST00000554808.1|antisense|3/3|n.465-3dupT||||||INFO_REALIGN_3_
NSG00000258673|transcript|ENST00000554808.1|antisense|3/3|n.465-3dupT||||||INFO_REALIGN_3_
NSG00000258673|transcript|ENST00000554808.1|antisense|3/3|n.465-3dupT||||||INFO_REALIGN_3_
00000224699|transcript|ENST00000625451.2|antisense|1/3|n.188+2C>T||||||,T|missense_variant|M
56171|transcript|ENST00000477769.1|processed_transcript|3/3|n.235+1G>A||||||,T|downstream_
000064886|transcript|ENST00000445067.6|protein_coding|1/12|c.-156-3dupT||||||INFO_REALIGN_
000064886|transcript|ENST00000445067.6|protein_coding|1/12|c.-156-3dupT||||||INFO_REALIGN_
interaction|4AY1:A_182-A_208:ENST00000369748|protein_coding|6/11|c.545C>T||||||,T|structural_
interaction|4AY1:A_318-A_338:ENST00000369748|protein_coding|9/11|c.952C>A||||||,T|structural_
interaction|4AY1:A_318-A_338:ENST00000369748|protein_coding|9/11|c.952C>A||||||,T|structural_
ENST00000422815.5|protein_coding|1/9|c.7_8dupTC|p.Ser4fs|9/1354|9/1263|3/420||WARNING_TR
teraction|2YBU:F_339-F_374:ENST00000343320|protein_coding|9/11|c.1015A>G||||||,G|structural_
00064703|transcript|ENST00000369702.4|protein_coding|2/10|c.397-3dupT||||||INFO_REALIGN_3_
6748|transcript|ENST00000520113.6|protein_coding|2/16|c.133C>T|p.Gln45*|149/2375|133/2343|
G00000233154|transcript|ENST00000423907.5|lincRNA|2/3|n.150-2A>T||||||,A|downstream_gene_
0000256649.8|protein_coding|1/6|c.263C>A|p.Ser88*|790/3515|263/1743|88/580||,T|stop_gained
00249798|transcript|ENST00000514706.1|unprocessed_pseudogene|1/2|n.145_145+1insG||||||,AG
00000228826|transcript|ENST00000437523.2|lincRNA|2/2|n.92+1G>T||||||
ENST00000581897.6|protein_coding|19/19|c.1880delA|p.His627fs|2512/4272|1880/2013|627/670|
000162825|transcript|ENST00000369373.9|protein_coding|5/138|c.564delC|p.Arg189fs|757/18760|
|interaction|2XUB:A_84-A_239:ENST00000334163|protein_coding|3/15|c.251G>A||||||,A|structural
|ENST00000334513.5|protein_coding|8/8|c.897delG|p.Cys300fs|909/1473|897/987|299/328||,TG|m
|ENST00000334513.5|protein_coding|8/8|c.897delG|p.Cys300fs|909/1473|897/987|299/328||,TG|m
|ENST00000334513.5|protein_coding|8/8|c.900_904delTAAAA|p.Cys300fs|912/1473|900/987|300/3
T00000355594.8|protein_coding|10/14|c.1981C>T|p.Gln661*|2068/3342|1981/3006|661/1001||,A|
pt|ENST00000355594.8|protein_coding|10/14|c.1525dupG|p.Ala509fs|1612/3342|1525/3006|509/1
00271425|transcript|ENST00000583866.8|protein_coding|13/89|c.1854-1C>G||||||,C|splice_accept
0000617844.4|protein_coding|12/34|c.1336C>T|p.Gln446*|1770/6326|1336/4374|446/1457||,T|st
NST00000369258.8|protein_coding|4/23|c.400_404delGCCTG|p.Ala134fs|420/2967|400/2694|134/8
609196.5|protein_coding|6/6|c.471G>C|p.Ter157Tyrext*?|473/1874|471/471|157/156||WARNING_
SG00000231551|transcript|ENST00000634638.1|lincRNA|3/6|n.494-2A>T||||||,T|splice_acceptor_v
DE4DIP|ENSG00000178104|transcript|ENST00000618462.4|protein_coding|8/45|c.835-16_835-3dup
000178104|transcript|ENST00000525630.5|processed_transcript|5/6|n.506-2_506-1insA||||||,TA|s
00117360|transcript|ENST00000324862.6|protein_coding|1/15|c.-48-4_-48-3dupTT||||||INFO_REAL
00117360|transcript|ENST00000324862.6|protein_coding|1/15|c.-48-4_-48-3dupTT||||||INFO_REAL
00117360|transcript|ENST00000324862.6|protein_coding|1/15|c.-48-4_-48-3dupTT||||||INFO_REAL
_gained|HIGH|PRPF3|ENSG00000117360|transcript|ENST00000324862.6|protein_coding|10/16|c.12
G00000203804|transcript|ENST00000617352.1|antisense|1/3|n.429+2C>T||||||,A|upstream_gene_v
437|transcript|ENST00000504358.1|nonsense_mediated_decay|3/6|c.*92+2C>T||||||,A|intron_var
0368947.8|protein_coding|3/14|c.1A>G|p.Met1?|477/1826|1/1038|1/345||,G|intron_variant|MOD
00143398|transcript|ENST00000486048.2|processed_transcript|2/3|n.268-2G>A||||||,A|upstream_

)000143376|transcript|ENST00000368841.6|nonsense_mediated_decay|8/11|c.*911-3dupT|||||INF
)000143376|transcript|ENST00000368841.6|nonsense_mediated_decay|8/11|c.*911-3dupT|||||INF
)000143376|transcript|ENST00000368841.6|nonsense_mediated_decay|8/11|c.*911-3dupT|||||INF
)000143376|transcript|ENST00000368841.6|nonsense_mediated_decay|8/11|c.*911-3dupT|||||INF
)000143376|transcript|ENST00000368841.6|nonsense_mediated_decay|8/11|c.*911-3dupT|||||INF
ENSG00000249602|transcript|ENST00000512280.1|antisense|1/2|n.156_158+1dupGAGG|||||,ACCT
50&ENSG00000182134|gene_variant|ENSG00000143450|||n.151770090_151770112delGGAGACCATG
ENSG00000203288|transcript|ENST00000389897.3|antisense|1/1|n.405-3dupT|||||INFO_REALIGN_
ENSG00000203288|transcript|ENST00000389897.3|antisense|1/1|n.405-3dupT|||||INFO_REALIGN_
ENSG00000203288|transcript|ENST00000389897.3|antisense|1/1|n.405-3dupT|||||INFO_REALIGN_
interaction|4AE7:A_146-A_197:ENST00000368817|protein_coding|5/6|c.590A>G|||||,C|missense_
000368801.2|protein_coding|3/3|c.3232C>T|p.Gln1078*|3308/9623|3232/8553|1078/2850||,A|intro
anscript|ENST00000368801.2|protein_coding|2/3|c.1delA|p.Met1fs|77/9623|1/8553|1/2850||,A|se
T00000368799.1|protein_coding|3/3|c.2282_2285delCAGT|p.Ser761fs|2321/12747|2282/12186|761
0368799.1|protein_coding|3/3|c.1501C>T|p.Arg501*|1537/12747|1501/12186|501/4061||,A|upstre
00237975|transcript|ENST00000392688.6|antisense|3/6|n.1081-2A>T|||||,T|splice_acceptor_variar
00388718.5|protein_coding|3/3|c.7130C>A|p.Ser2377*|7203/9124|7130/7176|2377/2391||,T|intror
0326233.6|protein_coding|2/2|c.343T>G|p.Ter115Glyext*?|386/430|343/345|115/114||
5942|transcript|ENST00000431011.2|protein_coding|1/1|c.-22+2T>C|||||
ENST00000368713.7|protein_coding|3/3|c.208delG|p.Val70fs|405/856|208/306|70/101||,A|framesh
interaction|3NSI:A_31-A_62:ENST00000368713|protein_coding|3/3|c.184C>G|||||,C|structural_int
GH|GATAD2B|ENSG00000143614|transcript|ENST00000634791.1|protein_coding|1/3|c.-99-3_-99-2d
GH|GATAD2B|ENSG00000143614|transcript|ENST00000634791.1|protein_coding|1/3|c.-99-3_-99-2d
GH|GATAD2B|ENSG00000143614|transcript|ENST00000634791.1|protein_coding|1/3|c.-99-3_-99-2d
ipt|ENST00000361217.8|protein_coding|18/28|c.2703_2704insG|p.Gln902fs|3122/5706|2703/4491|
ript|ENST00000361217.8|protein_coding|18/28|c.2702_2703insGC|p.Gln902fs|3121/5706|2702/449
ion_variant&intron_variant|HIGH|EFNA3|ENSG00000143590|transcript|ENST00000368408.3|protein_
)143590|transcript|ENST00000368408.3|protein_coding|3/4|c.509-2A>C|||||,C|splice_acceptor_var
)143590|transcript|ENST00000368408.3|protein_coding|3/4|c.509-2A>C|||||,C|splice_acceptor_var
)143590|transcript|ENST00000368408.3|protein_coding|3/4|c.509-1G>C|||||,C|splice_acceptor_var
)143590|transcript|ENST00000368408.3|protein_coding|3/4|c.509-1G>C|||||,C|splice_acceptor_var
185499|transcript|ENST00000611577.4|protein_coding|1/4|c.59-2G>A|||||,T|synonymous_variant
GH|RP11-243J18.2|ENSG00000227673|transcript|ENST00000432858.1|antisense|1/1|n.27-3_27-2del
GH|RP11-243J18.2|ENSG00000227673|transcript|ENST00000432858.1|antisense|1/1|n.27-3_27-2del
GH|RP11-243J18.2|ENSG00000227673|transcript|ENST00000432858.1|antisense|1/1|n.27-3_27-2del
ENST00000532414.2|protein_coding|1/2|c.157_170delCCCCCCGCCCCCAC|p.Pro53fs|170/6124|157/1
00132677|transcript|ENST00000612897.4|nonsense_mediated_decay|1/10|c.188-3dupT|||||INFO_
00132677|transcript|ENST00000612897.4|nonsense_mediated_decay|1/10|c.188-3dupT|||||INFO_
000132677|transcript|ENST00000613460.4|nonsense_mediated_decay|1/10|c.188-3dupT|||||INFO
000132677|transcript|ENST00000613460.4|nonsense_mediated_decay|1/10|c.188-3dupT|||||INFO
000132677|transcript|ENST00000613460.4|nonsense_mediated_decay|1/10|c.188-3dupT|||||INFO
000132677|transcript|ENST00000613460.4|nonsense_mediated_decay|1/10|c.188-3dupT|||||INFO
)00612897.4|nonsense_mediated_decay|3/11|c.300G>A|p.Trp100*|338/1862|300/462|100/153||,A
ENST00000537040.5|protein_coding|9/10|c.1271dupC|p.Asp425fs|1310/1789|1272/1377|424/458||
NST00000368236.7|protein_coding|5/11|c.1144delC|p.Gln382fs|1180/2005|1144/1749|382/582||IN
ript|ENST00000368232.8|protein_coding|8/8|c.1068_1069insGT|p.Phe357fs|1201/1972|1068/1113|
60818|transcript|ENST00000474904.5|nonsense_mediated_decay|6/8|c.336G>A|p.Trp112*|456/71

nteraction|4AOJ:A_558-A_580:ENST00000524377|protein_coding|14/17|c.1674G>A||||||,A|synonyr
94|interaction|1HTJ:F_447-F_484:ENST00000361409|protein_coding|16/40|c.1340G>A||||||,T|misse
ENST00000368189.3|protein_coding|8/8|c.1731dupC|p.Thr578fs|1824/4424|1731/1779|577/592||,T
G00000236731|transcript|ENST00000445687.1|unprocessed_pseudogene|1/1|n.248-2A>G|||||
00368174.4|protein_coding|3/6|c.328G>T|p.Glu110*|425/2191|328/1044|110/347||,A|downstrean
00289429.5|protein_coding|2/6|c.93G>A|p.Trp31*|626/2096|93/984|31/327||
0000623167.1|polymorphic_pseudogene|1/1|c.198G>A|p.Trp66*|198/981|198/981|66/326||
0000368140.5|protein_coding|6/9|c.1117C>T|p.Arg373*|1362/2083|1117/1479|373/492||,T|stop_g
teraction|3RLN:A_612-A_642:ENST00000295809|protein_coding|10/12|c.1924C>T||||||,T|structural_
teraction|4O7Q:A_32-A_64:ENST00000368130|protein_coding|2/6|c.94G>A||||||,T|structural_intera
|ENST00000504970.1|polymorphic_pseudogene|1/1|c.593dupT|p.Leu198fs|594/935|594/935|198/3
0000423932.4|protein_coding|1/1|c.792T>A|p.Cys264*|829/1090|792/963|264/320||,A|intron_vari
|interaction|4ERC:A_10-A_34:ENST00000368107|protein_coding|1/2|c.100C>G||||||,G|structural_ir
|interaction|4ERC:A_99-A_131:ENST00000368107|protein_coding|2/2|c.391G>A||||||,A|structural_i
339348.9|protein_coding|9/9|c.1192T>C|p.Ter398Argext*?|1200/1422|1192/1194|398/397||,C|sto
00368106.3|protein_coding|10/10|c.1267C>T|p.Gln423*|1268/1306|1267/1305|423/434||,T|3_prir
ST00000613788.1|protein_coding|3/3|c.725delG|p.Gly242fs|793/1500|725/759|242/252||,A|down
teraction|1NBQ:A_146-A_201:ENST00000368026|protein_coding|5/10|c.438G>A||||||,T|structural_i
|ENSG00000270149|transcript|ENST00000289779.7|nonsense_mediated_decay|3/13|c.91delG|p.Va
intron_variant|HIGH|AL591806.1|ENSG00000278883|transcript|ENST00000624422.1|protein_coding
intron_variant|HIGH|AL591806.1|ENSG00000278883|transcript|ENST00000624422.1|protein_coding
intron_variant|HIGH|AL591806.1|ENSG00000278883|transcript|ENST00000624422.1|protein_coding
intron_variant|HIGH|AL591806.1|ENSG00000278883|transcript|ENST00000624422.1|protein_coding
'|interaction|4JJH:A_42-A_146:ENST00000368012|protein_coding|2/9|c.436C>A||||||,T|structural_ir
7|interaction|4JJH:A_53-A_108:ENST00000368012|protein_coding|2/9|c.157T>C||||||,G|missense_v
0000271450.10|protein_coding|3/7|c.187C>T|p.Gln63*|225/2412|187/954|63/317||,T|stop_gained
nteraction|3FE1:A_198-A_335:ENST00000309758|protein_coding|1/1|c.592C>T||||||,T|structural_in
244682|transcript|ENST00000466542.6|polymorphic_pseudogene|6/6|c.798+1A>G||||||,G|splice_dc
|interaction|4JNB:A_132-A_170:ENST00000367943|protein_coding|3/6|c.510C>T||||||,T|structural_
171722|transcript|ENST00000493255.1|nonsense_mediated_decay|1/1|c.*133+1G>T||||||,A|upstr
nteraction|1JV1:A_248-A_330:ENST00000367926|protein_coding|6/10|c.989A>G||||||,G|structural_i
SG00000225755|transcript|ENST00000450164.2|processed_pseudogene|1/3|n.154-1G>T||||||,T|in
3228|transcript|ENST00000271452.7|protein_coding|5/13|c.337+1G>T||||||,T|splice_donor_variant&
0143198|transcript|ENST00000367885.5|protein_coding|1/6|c.-9-2G>A||||||,A|splice_acceptor_var
teraction|1UDW:A_25-A_136:ENST00000367879|protein_coding|4/7|c.408G>A||||||,A|structural_ir
ENSG00000203307|transcript|ENST00000366136.2|antisense|2/2|n.198-3dupT|||||INFO_REALIGN_
ENSG00000203307|transcript|ENST00000366136.2|antisense|2/2|n.198-3dupT|||||INFO_REALIGN_
15834|transcript|ENST00000477875.5|processed_transcript|5/6|n.904+2T>C||||||,C|splice_donor_v
8771|transcript|ENST00000475127.5|processed_transcript|2/2|n.323+1G>A||||||,A|splice_donor_v
t|HIGH|MPZL1|ENSG00000197965|transcript|ENST00000487858.1|processed_transcript|2/2|n.270-2
0197965|transcript|ENST00000487858.1|processed_transcript|2/2|n.270-1G>A||||||,A|TF_binding_s
|interaction|4CLF:A_296-A_385:ENST00000367851|protein_coding|11/33|c.1155T>C||||||,G|structu
t|ENST00000539777.5|protein_coding|1/5|c.53dupC|p.Gly19fs|364/7655|53/1356|18/451||,AG|5_p
00000286031.10|protein_coding|13/24|c.1201C>T|p.Gln401*|1901/4355|1201/2562|401/853||,T|st
NST00000209929.9|protein_coding|4/9|c.337delG|p.Val113fs|495/3851|337/1608|113/535||,T|fram
00209929.9|protein_coding|9/9|c.1414C>T|p.Gln472*|1572/3851|1414/1608|472/535||,T|3_prime
ENST00000209929.9|protein_coding|9/9|c.1588dupT|p.Cys530fs|1747/3851|1589/1608|530/535||IN

nteraction|4WXU:A_316-A_347:ENST00000037502|protein_coding|3/3|c.1041T>C||||||,G|structural
nteraction|4WXU:A_285-A_307:ENST00000037502|protein_coding|3/3|c.855G>T||||||,A|synonymou
0180999|transcript|ENST00000367727.8|protein_coding|5/6|c.341+1G>A||||||,A|splice_donor_varia
|ZBTB37|ENSG00000185278|transcript|ENST00000367704.5|protein_coding|1/3|c.-226+2_-226+6de
t|ENST00000619513.1|protein_coding|2/2|c.275_276delCA|p.Thr92fs|972/4340|275/462|92/153||,
t|ENST00000423313.5|protein_coding|4/4|c.204delG|p.Lys69fs|741/4564|204/300|68/99||,T|frame
t|ENST00000423313.5|protein_coding|4/4|c.195_202delCAAGAAGA|p.Asn65fs|739/4564|195/300|6
0213057|transcript|ENST00000367638.1|lincRNA|2/2|n.288+2C>T||||||,T|intron_variant|MODIFIER|
0000143337|transcript|ENST00000528443.6|protein_coding|2/9|c.554-1G>A||||||,A|splice_acceptor
0000228918|transcript|ENST00000608183.1|lincRNA|7/16|n.1637-2G>A||||||
teraction|1E96:B_115-B_181:ENST00000367535|protein_coding|5/15|c.542A>G||||||,C|structural_i
0367534.5|protein_coding|4/4|c.406T>C|p.Ter136Argext*?|429/622|406/408|136/135||,G|3_prime
000261024|transcript|ENST00000562817.2|lincRNA|2/3|n.103+1G>A||||||,T|non_coding_transcript
ipt|ENST00000623025.1|protein_coding|1/3|c.53_56delCTAT|p.Ser18fs|56/135|53/135|18/44||,TAT
ipt|ENST00000623025.1|protein_coding|1/3|c.53_56delCTAT|p.Ser18fs|56/135|53/135|18/44||,TAT
ipt|ENST00000623025.1|protein_coding|1/3|c.53_56delCTAT|p.Ser18fs|56/135|53/135|18/44||,TAT
script|ENST00000623025.1|protein_coding|1/3|c.18_19insCT|p.Ile7fs|18/135|18/135|6/44||,TAG|u
script|ENST00000623025.1|protein_coding|1/3|c.16_17insCT|p.Tyr6fs|16/135|16/135|6/44||,G|mis
script|ENST00000623025.1|protein_coding|1/3|c.16_17insCT|p.Tyr6fs|16/135|16/135|6/44||,G|mis
0367439.7|protein_coding|1/4|c.2T>C|p.Met1?|51/701|2/495|1/164||,G|sequence_feature|MODEF
0116785|transcript|ENST00000471440.6|protein_coding|4/4|c.614-1G>A||||||,A|splice_acceptor_va
0116785|transcript|ENST00000617219.1|protein_coding|4/4|c.563-1T>C||||||,C|downstream_gene_
NST00000617219.1|protein_coding|5/5|c.574delT|p.Cys192fs|645/1462|574/588|192/195||INFO_R
ENST00000256785.4|protein_coding|4/10|c.486dupA|p.Glu163fs|596/2810|487/1710|163/569||INF
|ENST00000256785.4|protein_coding|4/10|c.485_486dupAA|p.Glu163fs|596/2810|487/1710|163/5
43278|transcript|ENST00000490002.1|processed_transcript|2/2|n.364-1C>G||||||,C|intron_variant|
00367383.5|protein_coding|4/4|c.219G>A|p.Trp73*|403/586|219/360|73/119||,A|downstream_ge
NSG00000260088|transcript|ENST00000568695.1|lincRNA|1/2|n.117-3dupT||||||INFO_REALIGN_3_P
NSG00000260088|transcript|ENST00000568695.1|lincRNA|1/2|n.117-4_117-3dupTT||||||INFO_REA
RP11-92G12.3|ENSG00000260088|transcript|ENST00000568695.1|lincRNA|2/2|n.239-15_239-3dupTT
RP11-92G12.3|ENSG00000260088|transcript|ENST00000568695.1|lincRNA|2/2|n.239-16_239-3dupT
0000260088|transcript|ENST00000568695.1|lincRNA|2/2|n.239-4_239-3delTT||||||INFO_REALIGN_
00000260088|transcript|ENST00000568695.1|lincRNA|2/2|n.239-3delT||||||INFO_REALIGN_3_PRI
ST00000367313.4|protein_coding|3/10|c.530delG|p.Gly177fs|589/2336|530/1596|177/531||,G|fra
ST00000367313.4|protein_coding|3/10|c.525_526delGG|p.Leu175fs|585/2336|525/1596|175/531||
ST00000367313.4|protein_coding|3/10|c.525_526delGG|p.Leu175fs|585/2336|525/1596|175/531||
43323|transcript|ENST00000490225.5|unitary_pseudogene|12/28|n.2881+2T>G||||||,G|intron_var
ST00000439764.2|protein_coding|1/16|c.76delG|p.Ala26fs|76/2487|76/2487|26/828||,T|intron_var
ction|4CCG:A_5-X_310:ENST00000367274-ENST00000233741|protein_coding|2/7|c.15A>G||||||,C|p
0229120|transcript|ENST00000429682.2|processed_pseudogene|1/1|n.58-2delA||||||,CTT|splice_re
0229120|transcript|ENST00000429682.2|processed_pseudogene|1/1|n.58-2delA||||||,CTT|splice_re
interaction|5A1F:A_629-A_654:ENST00000367265|protein_coding|14/27|c.1887C>T||||||,A|synony
t|ENST00000447715.6|protein_coding|20/35|c.1752_1753insGC|p.Pro585fs|2194/6349|1753/3558|
00000163485&ENSG00000224671|gene_variant|ENSG00000224671|||n.203152596_203152574delGT
interaction|1HJV:A_300-A_364:ENST00000255409|protein_coding|10/10|c.1092T>C||||||,G|structu
interaction|1HJV:C_107-C_145:ENST00000255409|protein_coding|5/10|c.433A>G||||||,C|structural_
ne_insertion|HIGH|CHIT1|ENSG00000133063|transcript|ENST00000367229.5|protein_coding|10/11|

00176754|transcript|ENST00000427799.1|lincRNA|4/5|n.548+2T>C||||||,G|splice_donor_variant&in

39|transcript|ENST00000638118.1|protein_coding|1/11|c.-211+1G>A||||||,T|downstream_gene_va

nscript|ENST00000367194.4|protein_coding|3/3|c.417delA|p.Ter139fs|566/709|417/417|139/138||

0133069|transcript|ENST00000367159.2|processed_transcript|1/2|n.197-1G>A||||||,A|5_prime_UT

0162873|transcript|ENST00000367156.7|protein_coding|3/8|c.-515+1G>A||||||,T|splice_donor_vari

00236942|transcript|ENST00000411645.1|processed_pseudogene|1/1|n.182+1T>C||||||,C|5_prime

00236942|transcript|ENST00000411645.1|processed_pseudogene|1/1|n.182+1T>C||||||,C|5_prime

0000263961|transcript|ENST00000331555.9|protein_coding|2/6|c.8_9delCA|p.Thr3fs|647/1665|8/5

89|interaction|3KC3:B_178-B_316:ENST00000367103|protein_coding|8/10|c.948C>T||||||,T|structu

352|transcript|ENST00000367067.8|protein_coding|7/9|c.1104+1T>C||||||,C|downstream_gene_va

IST00000367036.7|protein_coding|1/5|c.82delT|p.Ser28fs|737/2538|82/684|28/227||,GAA|framesh

IST00000367036.7|protein_coding|1/5|c.82delT|p.Ser28fs|737/2538|82/684|28/227||,GAA|framesh

G00000283619|transcript|ENST00000629778.3|protein_coding|2/9|c.76-3dupT||||||INFO_REALIGN

G00000283619|transcript|ENST00000629778.3|protein_coding|2/9|c.76-3dupT||||||INFO_REALIGN

00228110|transcript|ENST00000442505.2|processed_pseudogene|1/1|n.578-2C>T||||||

teraction|2W5B:A_48-A_168:ENST00000366999|protein_coding|3/8|c.504T>C||||||,G|structural_int

G00000231057|transcript|ENST00000421933.1|antisense|1/5|n.101+2C>T||||||,T|upstream_gene_v

G00000231057|transcript|ENST00000443174.2|antisense|1/2|n.61+1G>A||||||,A|non_coding_trans

|interaction|2Y1H:A_7-A_258:ENST00000366974|protein_coding|1/10|c.21C>G||||||,G|structural_ir

00203705|transcript|ENST00000366973.8|protein_coding|8/9|c.601-1G>A||||||,A|splice_acceptor_v

00136636|transcript|ENST00000259154.8|protein_coding|17/17|c.1884_1886dupTAG||||||INFO_RE

00162814|transcript|ENST00000470448.5|nonsense_mediated_decay|3/7|c.241-1G>C||||||,C|intro

00231814|transcript|ENST00000431637.2|lincRNA|3/3|n.363+2T>A||||||

NST00000366932.3|protein_coding|5/5|c.816delC|p.Ser272fs|846/7771|816/849|272/282||,G|sequ

00232480|transcript|ENST00000414452.1|antisense|2/2|n.290+2T>G||||||,C|upstream_gene_variar

nteraction|2WEF:A_190-A_213:ENST00000322067|protein_coding|7/9|c.637G>A||||||,T|structural_i

nteraction|2WEF:A_64-A_84:ENST00000322067|protein_coding|3/9|c.190C>T||||||,A|structural_int

ISG00000231529|transcript|ENST00000446169.1|lincRNA|1/1|n.54-2dupA||||||

00000276997|transcript|ENST00000621440.1|lincRNA|4/4|n.492+1G>A||||||,T|non_coding_transcri

00366881.5|protein_coding|6/6|c.1174C>T|p.Arg392*|1815/3368|1174/2577|392/858||,A|stop_gai

interaction|1KFU:L_68-L_161:ENST00000295006|protein_coding|1/21|c.202A>G||||||,G|missense_v

nteraction|1KFU:L_100-L_166:ENST00000295006|protein_coding|4/21|c.498C>T||||||,T|synonymou

eraction|2X8A:A_683-A_723:ENST00000281701|protein_coding|17/23|c.2049G>A||||||,T|synonymou

ipt|ENST00000430092.5|protein_coding|27/84|c.4172_4173dupGA|p.Tyr1392fs|4389/13763|4174/1

ipt|ENST00000430092.5|protein_coding|27/84|c.4172_4173dupGA|p.Tyr1392fs|4389/13763|4174/1

|ENST00000430092.5|protein_coding|37/84|c.5774_5777delAAGA|p.Lys1925fs|5989/13763|5774/13

00143742|transcript|ENST00000366839.8|protein_coding|2/3|c.142-3dupT||||||INFO_REALIGN_3_PF

00143742|transcript|ENST00000366839.8|protein_coding|2/3|c.142-3dupT||||||INFO_REALIGN_3_PF

H|TMEM63A|ENSG00000196187|transcript|ENST00000474478.5|processed_transcript|1/3|n.2270+1_

H|TMEM63A|ENSG00000196187|transcript|ENST00000474478.5|processed_transcript|1/3|n.2270+1_

H|TMEM63A|ENSG00000196187|transcript|ENST00000474478.5|processed_transcript|1/3|n.2270+1_

00196187|transcript|ENST00000474478.5|processed_transcript|1/3|n.2270+1_2270+2delGT||||||,A|

00196187|transcript|ENST00000474478.5|processed_transcript|1/3|n.2270+1_2270+2delGT||||||,A|

00196187|transcript|ENST00000474478.5|processed_transcript|1/3|n.2270+1delG||||||,A|upstream

00196187|transcript|ENST00000474478.5|processed_transcript|1/3|n.2270+1delG||||||,A|upstream

00196187|transcript|ENST00000474478.5|processed_transcript|1/3|n.2270+1G>T||||||,A|upstream_

00196187|transcript|ENST00000474478.5|processed_transcript|1/3|n.2270+1G>T||||||,A|upstream_

nteraction|3OD8:A_13-A_81:ENST00000366794|protein_coding|2/23|c.243C>T||||||,A|structural_in
interaction|4PED:A_331-A_359:ENST00000366777|protein_coding|8/15|c.993C>T||||||,T|structural_
interaction|4PED:A_329-A_351:ENST00000366777|protein_coding|8/15|c.1053C>T||||||,T|structura
interaction|4PED:A_372-A_395:ENST00000366777|protein_coding|10/15|c.1185C>T||||||,T|structur
interaction|4PED:A_480-A_522:ENST00000366777|protein_coding|12/15|c.1440C>T||||||,T|structur
interaction|4PED:A_572-A_630:ENST00000366777|protein_coding|15/15|c.1716T>C||||||,C|structu
00000295012.5|protein_coding|2/2|c.288C>A|p.Tyr96*|288/889|288/342|96/113||,T|3_prime_UTR
interaction|4C4K:O_32-O_63:ENST00000284548|protein_coding|2/81|c.187G>A||||||,A|structural_i
000284548.15|protein_coding|31/81|c.8467A>T|p.Arg2823*|8541/20432|8467/19863|2823/6620||
nteraction|2Q3H:A_68-A_205:ENST00000366691|protein_coding|1/3|c.204C>T||||||,T|synonymous_
|ENST00000323223.2|protein_coding|1/1|c.234_235delCT|p.Phe79fs|293/2175|234/411|78/136||IN
|interaction|2FFV:A_452-A_560:ENST00000366672|protein_coding|16/16|c.1679C>T||||||,T|structu
nteraction|1ZIV:A_74-A_166:ENST00000271971|protein_coding|4/20|c.498C>T||||||,T|structural_in
interaction|1ZIV:A_115-A_200:ENST00000271971|protein_coding|5/20|c.600T>C||||||,C|synonymou
nteraction|2G19:A_290-A_371:ENST00000366641|protein_coding|3/5|c.1113C>T||||||,A|structural_
00474953.5|nonsense_mediated_decay|7/17|c.2087A>T|p.Ter696Leuext*?|2088/3463|2087/2088|69
000186197|transcript|ENST00000439430.5|protein_coding|2/7|c.-141-2A>G||||||WARNING_TRANS
interaction|3AP4:A_19-A_140:ENST00000341872|protein_coding|4/11|c.56T>A||||||,A|structural_ir
interaction|3AP4:A_36-A_104:ENST00000341872|protein_coding|4/11|c.106C>T||||||,T|structural_i
on_coding_transcript_exon_variant|HIGH|RP11-385F5.4|ENSG00000230325|transcript|ENST0000043
0000450372.6|protein_coding|9/12|c.635T>A|p.Leu212*|1016/6281|635/1080|212/359||,A|stop_ga
interaction|3VKM:B_9-B_184:ENST00000341872|protein_coding|9/11|c.552G>C||||||,C|structural_i
NST00000546208.5|protein_coding|10/23|c.95delC|p.Pro32fs|1136/5103|95/2061|32/686||INFO_R
ENST00000319653.13|protein_coding|5/18|c.2852_2853insA|p.Ala952fs|3083/6434|2853/5169|951/
NST00000319653.13|protein_coding|5/18|c.2857delA|p.Ile953fs|3087/6434|2857/5169|953/1722||I
nteraction|3QE9:Y_216-Y_279:ENST00000348581|protein_coding|7/14|c.836A>G||||||,G|structural_i
69|interaction|3VVW:B_66-B_99:ENST00000357246|protein_coding|4/4|c.295G>A||||||,T|missense
000277099|transcript|ENST00000621768.1|unprocessed_pseudogene|5/5|n.801+2T>C||||||,G|dow
teraction|2V40:A_111-A_226:ENST00000366535|protein_coding|8/13|c.677A>G||||||,C|missense_v
ENST00000366522.6|protein_coding|1/8|c.132_133delGC|p.Leu45fs|273/6167|132/810|44/269||IN
script|ENST00000366522.6|protein_coding|1/8|c.200_204dupCCTCC|p.Arg69fs|346/6167|205/810|69
G00000203666|transcript|ENST00000366522.6|protein_coding|7/7|c.782-6_782-2dupTTTTA||||||IN
ENST00000343381.10|protein_coding|1/4|c.154_155delCC|p.Pro52fs|328/1951|154/1395|52/464||,
ENST00000318749.7|protein_coding|1/1|c.23delT|p.Phe8fs|23/954|23/954|8/317||,G|downstream
169224|transcript|ENST00000366488.4|protein_coding|2/4|c.89+1G>A||||||,A|splice_donor_variant
:|ENST00000360358.3|protein_coding|1/1|c.893dupA|p.Ala300fs|894/945|894/945|298/314||INFO_
000623922.1|polymorphic_pseudogene|1/1|c.867T>A|p.Tyr289*|867/939|867/939|289/312||
ENST00000317996.1|protein_coding|1/1|c.5delA|p.Glu2fs|5/963|5/963|2/320||
pt|ENST00000317450.3|protein_coding|1/1|c.956_957dupTG|p.Ile320fs|957/972|957/972|319/323|
ENST00000317450.3|protein_coding|1/1|c.609_615delCGTGCTG|p.Cys203fs|615/972|609/972|203/3
:|ENST00000344889.3|protein_coding|1/1|c.452dupG|p.Gly152fs|452/954|452/954|151/317||
15|transcript|ENST00000403657.5|protein_coding|7/12|c.1A>G|p.Met1?|2197/3540|1/684|1/227||,
_coding_transcript_exon_variant|HIGH|AC079779.5|ENSG00000235779|transcript|ENST00000590255
_coding_transcript_exon_variant|HIGH|AC079779.5|ENSG00000235779|transcript|ENST00000590255
000235779|transcript|ENST00000592553.5|lincRNA|1/3|n.211+1G>T||||||,A|splice_region_variant&
on_coding_transcript_exon_variant|HIGH|AC079779.5|ENSG00000235779|transcript|ENST000006124
0000130508|transcript|ENST00000477810.1|processed_transcript|2/3|n.293-5_293-2dupTTTA||||||

000443925.6|protein_coding|6/6|c.541G>T|p.Gly181*|642/1771|541/945|181/314||,A|intron_varian
214866|transcript|ENST00000537457.1|processed_transcript|4/7|n.379+2C>T||||||,T|intron_variant
00232044|transcript|ENST00000456327.1|lincRNA|4/4|n.1166+1G>A||||||,A|upstream_gene_varian
000236172|transcript|ENST00000585872.5|lincRNA|5/5|n.511+1G>A||||||,T|upstream_gene_varian
on_coding_transcript_exon_variant|HIGH|MIR7515HG|ENSG00000236172|transcript|ENST0000058700
000223884|transcript|ENST00000428379.1|antisense|2/2|n.342+1G>A||||||,T|splice_donor_variant&
000236008|transcript|ENST00000454224.1|lincRNA|3/5|n.355+2T>C||||||,G|upstream_gene_varian
pt|ENST00000483795.5|nonsense_mediated_decay|4/6|c.302dupA|p.Asn101fs|421/1913|302/324|1
G00000244260|transcript|ENST00000483023.1|lincRNA|4/4|n.1539+2T>G||||||
ENST00000263663.9|protein_coding|3/15|c.197dupA|p.Asn66fs|386/2387|198/1767|66/588||INFO_
00000236162|transcript|ENST00000452909.1|lincRNA|3/4|n.506-2A>G||||||,G|upstream_gene_vari
nteraction|4GWR:A_180-A_214:ENST00000272227|protein_coding|7/13|c.641A>G||||||,C|structural
143870|transcript|ENST00000458536.1|protein_coding|1/4|c.-813-2A>G||||||WARNING_TRANSCRIP
NSG00000271952|transcript|ENST00000607419.1|lincRNA|1/1|n.177-1G>A||||||
ENSG00000145063|transcript|ENST00000447433.5|lincRNA|1/4|n.499-5_499-2dupTTTA||||||,CTAAA
3|transcript|ENST00000381585.7|protein_coding|1/3|c.-1dupC|p.Met1fs|283/2684|1/489|1/162||IN
0196208|transcript|ENST00000263834.9|protein_coding|9/9|c.1160-1G>A||||||,A|upstream_gene_v
417461.5|protein_coding|1/7|c.3G>C|p.Leu1?|3/1330|3/516|1/171||,G|splice_region_variant&syno
9785|transcript|ENST00000621973.1|protein_coding|1/21|c.16+1T>C||||||,C|downstream_gene_var
000163032|transcript|ENST00000483921.1|processed_transcript|2/2|n.234-3dupT||||||INFO_REALI
000163032|transcript|ENST00000483921.1|processed_transcript|2/2|n.234-3dupT||||||INFO_REALI
000163032|transcript|ENST00000483921.1|processed_transcript|2/2|n.234-3dupT||||||INFO_REALI
000163032|transcript|ENST00000483921.1|processed_transcript|2/2|n.234-3dupT||||||INFO_REALI
000163032|transcript|ENST00000483921.1|processed_transcript|2/2|n.234-3dupT||||||INFO_REALI
000163032|transcript|ENST00000483921.1|processed_transcript|2/2|n.234-3dupT||||||INFO_REALI
NST00000317402.11|protein_coding|14/14|c.2515_2519delAAGTT|p.Lys839fs|2640/9854|2515/2727|
00000359846.6|protein_coding|5/10|c.527C>A|p.Ser176*|605/2475|527/1833|176/610||,T|stop_gai
ENST00000415264.5|protein_coding|3/4|c.195delA|p.Gly66fs|197/414|195/222|65/73||WARNING_
8960|transcript|ENST00000415264.5|protein_coding|3/4|c.179G>A|p.Trp60*|181/414|179/222|60/7
0000238721.8|protein_coding|4/5|c.755C>G|p.Ser252*|1610/1998|755/999|252/332||,C|stop_gaine
t|ENST00000613899.4|protein_coding|9/11|c.496delC|p.Gln166fs|873/1238|496/564|166/187||INF
T00000611917.1|nonsense_mediated_decay|3/5|c.300C>A|p.Cys100*|300/757|300/327|100/108||V
ript|ENST00000456591.5|nonsense_mediated_decay|4/7|c.310_311insAA|p.Arg104fs|431/809|311/3
on_coding_transcript_exon_variant|HIGH|RPL36AP13|ENSG00000233747|transcript|ENST000004574
on_coding_transcript_exon_variant|HIGH|RPL36AP13|ENSG00000233747|transcript|ENST000004574
on_coding_transcript_exon_variant|HIGH|RPL36AP13|ENSG00000233747|transcript|ENST000004574
on_coding_transcript_exon_variant|HIGH|RPL36AP13|ENSG00000233747|transcript|ENST000004574
on_coding_transcript_exon_variant|HIGH|RPL36AP13|ENSG00000233747|transcript|ENST000004574
000268412|transcript|ENST00000599234.2|processed_pseudogene|1/1|n.120+1T>C||||||
eraction|4BY3:A_1559-A_1591:ENST00000264705|protein_coding|30/44|c.4773A>G||||||,G|structu
eraction|4BY3:A_1644-A_1674:ENST00000264705|protein_coding|31/44|c.4930T>C||||||,C|structura
eraction|4BY3:A_1471-A_1686:ENST00000264705|protein_coding|31/44|c.5058C>T||||||,T|structura
eraction|4BY3:A_1499-A_1743:ENST00000264705|protein_coding|32/44|c.5229C>T||||||,T|structura
171174|transcript|ENST00000302188.7|protein_coding|1/7|c.90-1G>C||||||,G|splice_acceptor_varia
58019|transcript|ENST00000361704.6|protein_coding|11/12|c.1089-2A>C||||||,C|splice_acceptor_va
0229951|transcript|ENST00000445878.1|antisense|2/2|n.276+1A>G||||||,C|upstream_gene_variant
5295|transcript|ENST00000456385.5|nonsense_mediated_decay|1/13|c.-16+1G>T||||||,T|upstream_

)453137.1|protein_coding|4/4|c.218G>A|p.Trp73*|218/577|218/459|73/152||WARNING_TRANSCRI
eraction|2E1Q:A_1175-A_1239:ENST00000379416|protein_coding|34/36|c.3717G>A||||||,T|structur
eraction|2E1Q:A_737-A_843:ENST00000379416|protein_coding|21/36|c.2211C>T||||||,A|structural_
eraction|2E1Q:A_646-A_779:ENST00000379416|protein_coding|18/36|c.1936A>G||||||,C|structural_
eraction|2E1Q:A_256-A_279:ENST00000379416|protein_coding|10/36|c.837C>T||||||,A|structural_i
eraction|2E1Q:A_125-A_209:ENST00000379416|protein_coding|8/36|c.627G>A||||||,T|structural_in
0000203386|transcript|ENST00000366209.6|lincRNA|5/5|n.494-1A>G|||||
transcript|ENST00000413938.5|nonsense_mediated_decay|4/9|c.191A>C|p.Ter64Serext*?|381/1940
|interaction|3UIU:A_471-A_527:ENST00000233057|protein_coding|15/17|c.1413C>G||||||,C|structu
ENST00000234179.6|protein_coding|18/18|c.2673dupA||3228/5900|2673/2673|891/890||,AT|fram
teraction|2AFM:A_44-A_360:ENST00000338415|protein_coding|7/7|c.1079A>C||||||,C|structural_in
ENST00000402091.3|protein_coding|2/2|c.1058_1059delGT|p.Cys353fs|1136/1973|1058/1077|353/
interaction|3PM0:A_368-A_489:ENST00000610745|protein_coding|3/3|c.1103G>A||||||,T|structura
)00232973|transcript|ENST00000427168.6|antisense|1/2|n.177+2G>T||||||,T|splice_region_variant
nteraction|1SNZ:A_190-A_229:ENST00000272252|protein_coding|4/7|c.568A>T||||||,T|structural_in
)0205086|transcript|ENST00000378711.2|protein_coding|3/3|c.386-1G>A||||||,T|upstream_gene_v
nteraction|2QNK:A_37-A_101:ENST00000294973|protein_coding|2/10|c.109A>G||||||,C|missense_v
GH|FTOP1|ENSG00000226491|transcript|ENST00000419363.1|processed_pseudogene|1/2|n.187-7_
HIGH|FTOP1|ENSG00000226491|transcript|ENST00000419363.1|processed_pseudogene|1/2|n.187-7_
000226491|transcript|ENST00000419363.1|processed_pseudogene|1/2|n.187-7_187-6delAA|||||
0000226491|transcript|ENST00000419363.1|processed_pseudogene|1/2|n.187-7delA|||||
'1|ENSG00000226491|transcript|ENST00000419363.1|processed_pseudogene|1/2|n.187-14_187-8du
)P1|ENSG00000226491|transcript|ENST00000419363.1|processed_pseudogene|1/2|n.187-15_187-8d
00000138036|transcript|ENST00000462426.1|nonsense_mediated_decay|1/7|c.9-3dupT||||||INFO_
00000138036|transcript|ENST00000462426.1|nonsense_mediated_decay|1/7|c.9-3dupT||||||INFO_
ENST00000410056.7|protein_coding|6/6|c.1139delT|p.Leu380fs|1139/1426|1139/1176|380/391||IN
)000171132|transcript|ENST00000467135.5|processed_transcript|7/7|n.637-3dupT||||||INFO_REALI
)000171132|transcript|ENST00000467135.5|processed_transcript|7/7|n.637-3dupT||||||INFO_REALI
cript|ENST00000434431.2|protein_coding|2/3|c.457_458insGG|p.Gln153fs|479/680|458/659|153/2
19888|transcript|ENST00000405271.5|protein_coding|1/9|c.-149+1G>C||||||,C|splice_donor_varian
|ENST00000419334.1|protein_coding|1/3|c.211dupG|p.Val71fs|223/609|212/598|71/198||WARNIN
NSG00000233230|transcript|ENST00000417692.1|antisense|1/1|n.164-4_164-3dupTT||||||INFO_RE
0179915|transcript|ENST00000416262.6|processed_transcript|2/2|n.266-1G>T||||||,A|splice_accep
00000236837|transcript|ENST00000443926.1|lincRNA|2/3|n.393-2A>T|||||
000607452.5|protein_coding|4/7|c.221G>A|p.Trp74*|476/831|221/519|74/172||,A|stop_gained|HI
2994|transcript|ENST00000401408.5|protein_coding|11/12|c.1384+2T>A||||||,T|splice_donor_varia
nteraction|3U1K:B_611-B_646:ENST00000447944|protein_coding|24/28|c.1938G>A||||||,T|structur
nteraction|3U1K:A_420-A_464:ENST00000447944|protein_coding|17/28|c.1390C>A||||||,T|structur
00000162929|transcript|ENST00000483700.1|processed_transcript|2/2|n.230-3dupT||||||INFO_REA
00000162929|transcript|ENST00000483700.1|processed_transcript|2/2|n.230-3dupT||||||INFO_REA
G00000162929|transcript|ENST00000402291.5|protein_coding|6/21|c.671-3dupT||||||INFO_REALIG
G00000162929|transcript|ENST00000402291.5|protein_coding|6/21|c.671-3dupT||||||INFO_REALIG
G00000162929|transcript|ENST00000402291.5|protein_coding|6/21|c.671-3dupT||||||INFO_REALIG
t|ENST00000295031.9|protein_coding|21/22|c.2084delG|p.Gly695fs|2461/3116|2084/2124|695/707
000173163|transcript|ENST00000458337.5|protein_coding|2/2|c.*39-2delA||||||WARNING_TRANS(
000173163|transcript|ENST00000458337.5|protein_coding|2/2|c.*39-2delA||||||WARNING_TRANS(
)0230923|transcript|ENST00000431020.1|lincRNA|5/5|n.2642+1G>A||||||,T|intron_variant|MODIFI

0000230923|transcript|ENST00000431020.1|lincRNA|2/5|n.2293-2A>G||||||,C|intron_variant|MODI
000204929|transcript|ENST00000606978.5|lincRNA|7/9|n.786+1G>T||||||,T|non_coding_transcript_
000235885|transcript|ENST00000594329.5|lincRNA|3/5|n.451+2T>G||||||,G|upstream_gene_varian
000203395|transcript|ENST00000366218.2|antisense|1/1|n.170+1G>T||||||,A|intron_variant|MODI
0637675.1|polymorphic_pseudogene|3/6|c.176A>G|p.Ter59Trpext*?|176/546|176/546|59/181||
303698.7|protein_coding|2/8|c.2T>A|p.Met1?|217/1034|2/693|1/230||,T|start_lost|HIGH|NFU1|EN
nteraction|3C22:A_288-A_308:ENST00000410009|protein_coding|6/6|c.862A>G||||||,C|structural_i
nteraction|3C22:A_231-A_254:ENST00000410009|protein_coding|5/6|c.762C>A||||||,T|structural_ir
interaction|3C22:B_203-B_237:ENST00000410009|protein_coding|4/6|c.711T>C||||||,G|structural_ir
00000234396.8|protein_coding|1/14|c.2T>C|p.Met1?|75/1891|2/1542|1/513||,C|start_lost|HIGH|AT
pt|ENST00000454446.5|protein_coding|1/6|c.86_87delAG|p.Glu29fs|201/863|86/748|29/248||WAR
0000116039|transcript|ENST00000234396.8|protein_coding|1/13|c.119-2A>C||||||,C|splice_accepto
0000116039|transcript|ENST00000234396.8|protein_coding|1/13|c.119-1G>C||||||,C|splice_accepto
T00000453130.1|nonsense_mediated_decay|1/4|c.3G>A|p.Met1?|4/423|3/147|1/48||,T|missense_
nteraction|2CH5:A_31-A_55:ENST00000244204|protein_coding|2/10|c.93T>C||||||,C|structural_inter
nteraction|2CH5:D_125-D_272:ENST00000244204|protein_coding|9/10|c.816G>A||||||,A|synonymo
pt|ENST00000410104.1|protein_coding|18/18|c.1995_1996dupCT|p.Leu666fs|2127/5795|1996/2046
0377712.3|polymorphic_pseudogene|1/1|c.502T>C|p.Ter168Glnext*?|537/765|502/679|168/225||V
|interaction|3RZU:A_280-A_307:ENST00000394070|protein_coding|7/10|c.921G>T||||||,T|structura
eraction|2NZT:B_470-B_766:ENST00000290573|protein_coding|16/18|c.2298A>G||||||,G|synonymo
00066032|transcript|ENST00000497804.1|processed_transcript|1/3|n.88-2A>T||||||,T|downstream_
00409015.5|protein_coding|4/4|c.1363T>C|p.Ter455Glnext*?|1425/2276|1363/1365|455/454||,G|s
263867.8|protein_coding|2/10|c.3G>A|p.Met1?|253/1442|3/1047|1/348||,T|start_lost|HIGH|CAPG
58883|transcript|ENST00000491659.5|processed_transcript|3/4|n.416+1G>A||||||,A|upstream_gene
|interaction|4X7L:A_597-A_893:ENST00000303236|protein_coding|11/17|c.1791A>G||||||,C|synony
000254098|transcript|ENST00000518305.1|IG_V_pseudogene|1/1|n.50-1G>A||||||,T|upstream_gen
pt|ENST00000498435.1|IG_V_gene|2/2|c.238dupG|p.Val80fs|269/382|238/351|80/116||WARNING_
T00000390277.3|IG_V_gene|2/2|c.61G>T|p.Glu21*|158/444|61/347|21/114||WARNING_TRANSCRI
pt|ENST00000456556.5|protein_coding|61/67|c.3835_3838delGAAA|p.Glu1279fs|3923/5428|3835/5
28|interaction|4F92:B_1184-B_1280:ENST00000323853|protein_coding|27/45|c.3550T>C||||||,G|st
script|ENST00000623246.3|protein_coding|23/55|c.1159_1163dupAAGGG|p.Gln389fs|2196/6513|1
t|ENST00000420699.6|protein_coding|1/76|c.12delC|p.Lys5fs|256/6269|12/5826|4/1941||,G|frame
00000596356.5|nonsense_mediated_decay|1/5|c.1A>G|p.Met1?|3/650|1/204|1/67||,G|start_lost|H
nteraction|2OZO:A_530-A_559:ENST00000264972|protein_coding|13/14|c.1677G>A||||||,A|structu
8658|transcript|ENST00000477737.5|protein_coding|8/28|c.1114G>T|p.Glu372*|1318/4454|1114/3
409997.1|protein_coding|3/3|c.221A>G|p.Ter74Trpext*?|331/557|221/222|74/73||,C|intron_varian
00409347.5|protein_coding|2/6|c.2T>A|p.Met1?|236/1276|2/831|1/276||,A|start_lost|HIGH|UNC50
teraction|3GQC:A_373-A_705:ENST00000258428|protein_coding|6/23|c.1118A>G||||||,C|missense_
ST00000409579.5|protein_coding|15/25|c.1863delC|p.Asp623fs|2096/4342|1863/3756|621/1251||,
ST00000409579.5|protein_coding|15/25|c.1856_1860delCCTCA|p.Thr619fs|2093/4342|1856/3756|6
376865.1|protein_coding|1/10|c.1A>G|p.Met1?|8/485|1/462|1/153||,G|sequence_feature|MODER
000228488|transcript|ENST00000443030.1|lincRNA|1/1|n.63+2G>T||||||
5594|transcript|ENST00000452403.5|protein_coding|2/4|c.-84+2T>G||||||WARNING_TRANSCRIPT_I
I|TGFBRAP1|ENSG00000135966|transcript|ENST00000258449.2|protein_coding|11/11|c.*186+2_*18
0000409886.3|protein_coding|1/23|c.25G>T|p.Glu9*|113/5594|25/5277|9/1758||,A|stop_gained|HI
00000329106.2|protein_coding|1/7|c.108G>A|p.Trp36*|108/915|108/915|36/304||
3|interaction|2REO:A_54-A_179:ENST00000329106|protein_coding|5/7|c.535G>A||||||,A|missense_

3|interaction|2REO:A_138-A_202:ENST00000329106|protein_coding|5/7|c.604T>C||||||,C|missense_
00000198203|transcript|ENST00000326853.9|protein_coding|3/8|c.278-3dupT||||||INFO_REALIGN_3
00000198203|transcript|ENST00000326853.9|protein_coding|3/8|c.278-3dupT||||||INFO_REALIGN_3
nteraction|4HI8:B_38-B_58:ENST00000332345|protein_coding|2/10|c.114C>T||||||,T|structural_inte
9756|transcript|ENST00000338045.7|protein_coding|2/9|c.342+1G>T||||||,T|splice_donor_variant&
000228763|transcript|ENST00000411710.1|antisense|1/2|n.163+2dupT||||||,TA|downstream_gene_
0000163006|transcript|ENST00000412964.6|protein_coding|1/13|c.94-3dupT||||||INFO_REALIGN_3_
ript|ENST00000623296.1|protein_coding|1/1|c.543_544delAG|p.Arg181fs|544/690|543/690|181/229
000439055.5|protein_coding|18/18|c.1729G>T|p.Gly577*|1953/2373|1729/1743|577/580||,T|upstr
raction|3IO8:C_97-D_65:ENST00000307677-ENST00000452231|nonsense_mediated_decay|2/4|c.195
00000341068.7|protein_coding|12/48|c.1393C>T|p.Gln465*|2166/8262|1393/5835|465/1944||,A|up
00000230958|transcript|ENST00000448785.1|processed_pseudogene|1/1|n.118-1delA||||||INFO_RI
00000230958|transcript|ENST00000448785.1|processed_pseudogene|1/1|n.118-1delA||||||INFO_RI
00000230958|transcript|ENST00000448785.1|processed_pseudogene|1/1|n.118-1delA||||||INFO_RI
00000230958|transcript|ENST00000448785.1|processed_pseudogene|1/1|n.118-1delA||||||INFO_RI
0153208|transcript|ENST00000616902.4|protein_coding|25/39|c.962-2A>G||||||,G|intron_variant|
interaction|4M3Q:A_644-A_714:ENST00000295408|protein_coding|16/19|c.2142G>C||||||,C|structu
0496537.1|protein_coding|1/4|c.2T>G|p.Met1?|87/541|2/456|1/151||WARNING_TRANSCRIPT_NO_
ENST00000541869.5|protein_coding|2/16|c.75_99delCGTGTACCGAGAGACTGGCGTCCGG|p.Tyr27fs|19
ENST00000393167.7|protein_coding|5/9|c.232delG|p.Ala78fs|457/2179|232/687|78/228||INFO_REA
GH|AC092170.1|ENSG00000227291|transcript|ENST00000440211.1|processed_pseudogene|1/1|n.73
0000227291|transcript|ENST00000440211.1|processed_pseudogene|1/1|n.73-4_73-3delAA||||||
00000227291|transcript|ENST00000440211.1|processed_pseudogene|1/1|n.73-4delA||||||
I|EN1|ENSG00000163064|transcript|ENST00000546667.1|processed_transcript|1/1|n.429+2_429+8d
interaction|2VQ3:B_159-B_184:ENST00000393106|protein_coding|4/6|c.550G>A||||||,A|structural_
0627305.2|protein_coding|1/4|c.141C>G|p.Tyr47*|225/620|141/447|47/148||,G|stop_gained|HIGH
000594141.1|protein_coding|2/2|c.140G>C|p.Ter47Serext*?|140/743|140/141|47/46||WARNING_TF
ENST00000431283.1|protein_coding|5/5|c.549dupT|p.Val184fs|550/630|550/594|184/197||WARNII
00088179|transcript|ENST00000263708.6|protein_coding|19/26|c.1814-3dupT||||||INFO_REALIGN_
000088179|transcript|ENST00000263708.6|protein_coding|20/26|c.1981-3dupT||||||INFO_REALIGN_
000088179|transcript|ENST00000263708.6|protein_coding|20/26|c.1981-3dupT||||||INFO_REALIGN_
|interaction|4K92:A_437-A_475:ENST00000263710|protein_coding|13/40|c.1311T>C||||||,G|structu
00000228400|transcript|ENST00000438816.1|antisense|1/1|n.500-1G>C||||||
000237524|transcript|ENST00000454927.7|retained_intron|1/2|n.196+1A>G||||||,G|splice_donor_v
NST00000436605.4|protein_coding|1/5|c.67C>T|p.Arg23*|67/479|67/399|23/132||WARNING_TRANS
000236682|transcript|ENST00000433673.1|lincRNA|1/1|n.56+1G>C||||||,C|upstream_gene_variant|
|interaction|2C35:A_35-A_72:ENST00000272645|protein_coding|2/4|c.105A>G||||||,C|structural_in
NST00000451531.6|protein_coding|13/15|c.1764delT|p.Asn588fs|2195/7020|1764/3228|588/1075||
ENST00000309451.6|protein_coding|1/3|c.33delG|p.Ser12fs|79/624|33/477|11/158||,A|upstream_g
32258|transcript|ENST00000446247.5|protein_coding|1/6|c.-175+2T>C||||||WARNING_TRANSCRIPT_
00000048991|transcript|ENST00000443537.2|processed_transcript|4/4|n.466-3dupT||||||INFO_REA
00000048991|transcript|ENST00000443537.2|processed_transcript|4/4|n.466-3dupT||||||INFO_REA
nteraction|3E9K:A_412-A_457:ENST00000264170|protein_coding|13/14|c.1234A>G||||||,G|structura
000226674|transcript|ENST00000628611.2|lincRNA|2/2|n.120-3dupT||||||INFO_REALIGN_3_PRIME,A
000226674|transcript|ENST00000628611.2|lincRNA|2/2|n.120-3dupT||||||INFO_REALIGN_3_PRIME,A
000204406|transcript|ENST00000637997.1|processed_transcript|3/3|n.412-3dupT||||||INFO_REALIG
000204406|transcript|ENST00000637997.1|processed_transcript|3/3|n.412-3dupT||||||INFO_REALIG

)|interaction|2WNO:A_144-A_244:ENST00000243347|protein_coding|4/6|c.431A>G||||||,G|missens
)162980|transcript|ENST00000446896.7|nonsense_mediated_decay|6/8|c.*270-2A>G||||||,C|upstre
|ENST00000288670.13|protein_coding|15/26|c.1673_1674insA|p.Pro560fs|2041/5575|1674/3279|55
ript|ENST00000443733.1|processed_pseudogene|1/1|n.-1_*24del|p.0?|||||,A|upstream_gene_varia
ript|ENST00000443733.1|processed_pseudogene|1/1|n.-1_*24del|p.0?|||||,A|upstream_gene_varia
5165|transcript|ENST00000435117.1|protein_coding|3/5|c.-184+1G>T||||||WARNING_TRANSCRIPT_
nteraction|4C02:A_180-A_230:ENST00000263640|protein_coding|7/11|c.690G>A||||||,T|structural_
)00007001|transcript|ENST00000605860.5|protein_coding|4/10|c.148-1_148insA|p.Val50fs|194/2435
nteraction|2XRF:A_62-A_141:ENST00000005756|protein_coding|3/7|c.186C>G||||||,G|structural_int
G00000227480|transcript|ENST00000412781.2|antisense|4/4|n.356-1G>C||||||,C|intron_variant|M
)136536|transcript|ENST00000409175.5|protein_coding|2/11|c.-101+1G>A||||||,A|upstream_gene_
teraction|1NU6:B_405-B_429:ENST00000360534|protein_coding|14/26|c.1215C>T||||||,A|structura
teraction|1PFQ:B_203-B_266:ENST00000360534|protein_coding|10/26|c.796G>A||||||,T|structural_
197635|transcript|ENST00000360534.7|protein_coding|2/25|c.95-2A>G||||||,C|splice_acceptor_vari
00263642.2|protein_coding|10/16|c.1879G>T|p.Glu627*|2275/3595|1879/3078|627/1025||
5267|transcript|ENST00000263642.2|protein_coding|8/15|c.1641+1G>C|||||
)00237844|transcript|ENST00000429636.1|lincRNA|2/4|n.196+1G>A|||||
)0082438|transcript|ENST00000629362.2|protein_coding|2/16|c.180-2A>G||||||,C|splice_acceptor_
_coding_transcript_exon_variant|HIGH|AC013463.2|ENSG00000236283|transcript|ENST00000627211
00000236283|transcript|ENST00000629817.2|antisense|4/6|n.533-2T>A||||||,A|synonymous_variar
)136531|transcript|ENST00000636985.1|protein_coding|1/26|c.-552-1G>A||||||,A|splice_acceptor_
NST00000636384.1|nonsense_mediated_decay|14/29|c.2161_2162delTT|p.Leu721fs|2481/9429|216
NST00000636384.1|nonsense_mediated_decay|14/29|c.2161_2162delTT|p.Leu721fs|2481/9429|216
115339|transcript|ENST00000437849.1|nonsense_mediated_decay|3/5|c.266+1G>A||||||WARNING_
)136546|transcript|ENST00000409855.5|protein_coding|23/24|c.3712-2A>G||||||,C|splice_acceptor
NST00000409043.5|protein_coding|3/10|c.462delG|p.Ser155fs|551/3292|462/2817|154/938||INFO_
3092|transcript|ENST00000409728.5|protein_coding|4/10|c.661+2T>C||||||,C|upstream_gene_varia
)073737|transcript|ENST00000602501.5|protein_coding|7/7|c.737-1G>C||||||,C|splice_acceptor_va
ot|ENST00000453153.6|protein_coding|14/15|c.2406dupA|p.Arg803fs|2753/2967|2406/2544|802/84
398|transcript|ENST00000260970.7|protein_coding|1/13|c.-70+1G>A||||||,A|splice_donor_variant&
)0231550|transcript|ENST00000424937.1|processed_pseudogene|1/1|n.1087+2G>A||||||,T|intron_v
|ENST00000448589.1|protein_coding|2/2|c.61dupA|p.Thr21fs|64/594|62/75|21/24||WARNING_TRA
35|transcript|ENST00000487037.1|processed_transcript|1/1|n.27+2T>C||||||,C|upstream_gene_var
)00115827|transcript|ENST00000375255.7|protein_coding|3/13|c.322-3dupT||||||INFO_REALIGN_3_
)00115827|transcript|ENST00000375255.7|protein_coding|3/13|c.322-3dupT||||||INFO_REALIGN_3_
44306|transcript|ENST00000549848.5|processed_transcript|1/3|n.422+1G>A||||||,A|splice_donor_v
NST00000272732.10|protein_coding|8/8|c.1251_1263delTTTATCAGTCAAA|p.Asn417fs|1333/3037|12
)00213963|transcript|ENST00000397057.6|sense_overlapping|4/8|n.464+1G>A||||||,T|splice_donor
00000237298|transcript|ENST00000625480.2|antisense|4/5|n.407-3dupT||||||INFO_REALIGN_3_PRIM
00000237298|transcript|ENST00000625480.2|antisense|4/5|n.407-3dupT||||||INFO_REALIGN_3_PRIM
)00238171|transcript|ENST00000456895.1|lincRNA|3/5|n.251+1G>A||||||,T|intron_variant|MODIFI
)0000150722|transcript|ENST00000495820.5|processed_transcript|1/4|n.395-3dupT||||||INFO_REAI
)0000150722|transcript|ENST00000495820.5|processed_transcript|1/4|n.395-3dupT||||||INFO_REAI
00000224643|transcript|ENST00000444562.5|antisense|1/2|n.81-2A>C||||||,G|upstream_gene_var
53002|transcript|ENST00000472844.1|retained_intron|2/2|n.457+2T>A||||||,A|intron_variant|MOD
)000452137.1|nonsense_mediated_decay|4/5|c.346C>T|p.Gln116*|346/755|346/411|116/136||,T|d
53002|transcript|ENST00000452137.1|nonsense_mediated_decay|4/4|c.*286+1G>A||||||,A|downst

NST00000343098.9|protein_coding|1/23|c.256dupG|p.Ala86fs|257/21054|257/20991|86/6996||IN
cript|ENST00000626288.1|protein_coding|2/2|c.62dupA|p.Lys22fs|62/72|62/72|21/23||WARNING_
NST00000409985.5|protein_coding|4/4|c.488dupA|p.Leu164fs|653/1882|489/546|163/181||INFO_
nteraction|3BPT:A_81-A_133:ENST00000359678|protein_coding|4/14|c.243T>G|||||,C|synonymous
0359678.9|protein_coding|1/14|c.2T>C|p.Met1?|297/1939|2/1161|1/386||,G|start_lost|HIGH|HIBC
000223466|transcript|ENST00000452381.1|lincRNA|1/1|n.209+2T>C|||||
interaction|3LM0:A_74-A_101:ENST00000263955|protein_coding|3/8|c.222A>G|||||,C|structural_i
|ENST00000389175.8|protein_coding|3/28|c.291_292delTG|p.Cys97fs|426/3685|291/3306|97/1101
t|ENST00000409153.5|protein_coding|16/16|c.1693_1694delCA|p.His565fs|1877/2724|1693/1740|
|transcript|ENST00000622774.1|protein_coding|4/4|c.442delT|p.Ter148fs|830/1997|442/444|148/1
|transcript|ENST00000622774.1|protein_coding|4/4|c.442delT|p.Ter148fs|830/1997|442/444|148/1
44301|transcript|ENST00000491025.5|unitary_pseudogene|10/18|n.1141+2G>A|||||,A|intron_vari
43478|transcript|ENST00000467645.1|processed_transcript|6/13|n.589+1G>A|||||,A|splice_donor_
409769.6|protein_coding|1/8|c.3G>T|p.Met1?|63/1192|3/924|1/307||,A|upstream_gene_variant|M
003402|transcript|ENST00000341222.10|protein_coding|5/5|c.607-1G>A|||||,A|splice_acceptor_v
0358485.8|protein_coding|1/9|c.2T>C|p.Met1?|198/2930|2/1617|1/538||,C|start_lost|HIGH|CASP8
nteraction|2Y1L:C_240-C_285:ENST00000432109|protein_coding|9/10|c.853G>C|||||,C|missense_v
nteraction|2Y1L:A_320-A_361:ENST00000432109|protein_coding|9/10|c.960G>A|||||,A|synonymou
T00000286190.9|protein_coding|14/14|c.1334C>G|p.Ser445*|1381/1769|1334/1338|445/445||,C|s
393|transcript|ENST00000496244.5|processed_transcript|4/4|n.352+1A>G|||||,C|sequence_featur
T00000541917.6|protein_coding|21/22|c.3247C>T|p.Arg1083*|3247/3543|3247/3543|1083/1180||,
NST00000438804.5|nonsense_mediated_decay|14/16|c.1264_1265delAA|p.Lys422fs|1734/2481|12
NST00000438804.5|nonsense_mediated_decay|14/16|c.1264_1265delAA|p.Lys422fs|1734/2481|12
NST00000438804.5|nonsense_mediated_decay|14/16|c.1264_1265delAA|p.Lys422fs|1734/2481|12
NST00000402905.7|protein_coding|16/16|c.2146dupA|p.Thr716fs|2468/5938|2147/2178|716/725||
0000449802.5|protein_coding|24/55|c.3463G>T|p.Glu1155*|3796/10938|3463/8085|1155/2694||
interaction|4GMV:A_371-A_489:ENST00000319170|protein_coding|8/14|c.1113A>G|||||,C|structur
000234714|transcript|ENST00000411436.1|lincRNA|1/4|n.136+1G>A|||||
ST00000357118.8|protein_coding|16/16|c.2697_2700delGCAC|p.His900fs|2728/3367|2697/2706|89
7|transcript|ENST00000357118.8|protein_coding|16/16|c.2701_2702insA|p.Cys901fs|2733/3367|27
000234902|transcript|ENST00000448786.1|lincRNA|2/3|n.467+1G>A|||||,T|splice_donor_variant&i
teraction|1T09:A_135-A_183:ENST00000345146|protein_coding|6/10|c.548A>G|||||,C|structural_ir
teraction|3MAP:B_144-B_178:ENST00000345146|protein_coding|6/10|c.532G>A|||||,T|structural_i
teraction|4KZO:C_105-C_134:ENST00000345146|protein_coding|4/10|c.315C>T|||||,A|structural_ir
000272847.6|protein_coding|3/13|c.245C>A|p.Ser82*|458/2713|245/1653|82/550||,A|5_prime_UT
NST00000442445.1|protein_coding|3/3|c.183_184insG|p.Leu62fs|241/551|183/279|61/92||,C|miss
nteraction|3C5R:A_507-A_538:ENST00000260947|protein_coding|6/11|c.1519G>A|||||,T|structura
interaction|3C5R:A_469-A_506:ENST00000260947|protein_coding|6/11|c.1518T>C|||||,G|structura
t|ENST00000412081.1|protein_coding|3/3|c.220dupT|p.Ser74fs|416/485|220/273|74/90||,GA|intro
363|transcript|ENST00000442048.3|processed_transcript|1/2|n.48+1G>A|||||,A|upstream_gene_va
00000237525|transcript|ENST00000417485.6|lincRNA|2/7|n.285-1G>C|||||,C|intron_variant|MOD
15425|transcript|ENST00000487318.6|processed_transcript|3/3|n.485-1G>A|||||,T|downstream_g
NST00000423123.1|protein_coding|4/5|c.212delG|p.Gly71fs|315/552|212/396|71/131||,A|intron_v
44579|transcript|ENST00000431127.1|protein_coding|1/3|c.274+1G>C|||||WARNING_TRANSCRIPT
0433439.1|nonsense_mediated_decay|2/3|c.124T>C|p.Ter42Argext*?|264/567|124/126|42/41||,C|
35924|transcript|ENST00000439026.1|protein_coding|1/5|c.-37+1G>C|||||WARNING_TRANSCRIPT
ST00000312358.11|protein_coding|36/41|c.8415delA|p.Leu2807fs|8547/10782|8415/9804|2805/32

ST00000312358.11|protein_coding|36/41|c.8437_8440delACAC|p.Thr2813fs|8569/10782|8437/9804
NST00000412982.5|protein_coding|5/7|c.500_501insA|p.Val168fs|502/1085|501/1029|167/342||,T/
:|ENST00000446656.3|protein_coding|5/6|c.834delG|p.Ala280fs|834/1048|834/1008|278/335||INFO
3983|transcript|ENST00000421680.2|processed_transcript|1/5|n.115+2T>C||||||,C|upstream_gene_
:|ENST00000444408.1|protein_coding|3/4|c.49_50insTT|p.Cys17fs|440/561|49/170|17/55||WARNIN
28|transcript|ENST00000638102.1|protein_coding|2/3|c.237_241dupCAATG|p.Val81fs|241/606|241
eraction|1QQG:A_90-A_198:ENST00000305123|protein_coding|1/2|c.270C>T||||||,A|synonymous_v
00000236432|transcript|ENST00000439598.6|antisense|12/13|n.1957-1T>G||||||,C|intron_variant|
00000236432|transcript|ENST00000439598.6|antisense|4/13|n.423-1G>T||||||,A|splice_acceptor_v
ENSG00000168958|transcript|ENST00000337110.11|protein_coding|2/7|c.-41_-41+1insCTGGAAAAGC
H|MFF|ENSG00000168958|transcript|ENST00000443428.6|protein_coding|3/3|c.38+1_38+2insGTGA
H|MFF|ENSG00000168958|transcript|ENST00000443428.6|protein_coding|3/3|c.38+1_38+2insGTGA
168958|transcript|ENST00000337110.11|protein_coding|3/7|c.181+1_181+2insAAA||||||,GAAATAAT
0000264387.8|protein_coding|3/3|c.423G>A|p.Trp141*|510/1844|423/453|141/150||,T|3_prime_U
|ENSG00000250599|transcript|ENST00000509872.1|unprocessed_pseudogene|2/3|n.805-5_805-1du
4853.3|ENSG00000250599|transcript|ENST00000509872.1|unprocessed_pseudogene|2/3|n.805-10_
00115009|transcript|ENST00000358813.4|protein_coding|1/3|c.77-3dupT||||||INFO_REALIGN_3_PR
00115009|transcript|ENST00000358813.4|protein_coding|1/3|c.77-3dupT||||||INFO_REALIGN_3_PR
00000225963|transcript|ENST00000594622.1|antisense|1/1|n.418-1G>A||||||,A|splice_acceptor_va
00458341.1|protein_coding|2/6|c.2T>C|p.Met1?|260/1727|2/390|1/129||,C|missense_variant|MOD
ENST00000440792.5|protein_coding|1/3|c.-7_20delCCCTGAATCCACACCACAGCAGCCTAG|p.Thr2fs||1/
0000454918.1|nonsense_mediated_decay|2/3|c.95C>A|p.Ser32*|159/332|95/114|32/37||,A|misser
00181798|transcript|ENST00000313064.2|lincRNA|1/2|n.258+1G>A|||||
56973|transcript|ENST00000428104.2|protein_coding|2/4|c.-22+1G>T||||||WARNING_TRANSCRIPT_
44535|transcript|ENST00000470087.1|processed_transcript|1/5|n.148+1G>A||||||,A|upstream_gene
00144535|transcript|ENST00000417808.1|protein_coding|1/1|c.91-2A>G||||||WARNING_TRANSCRIPT
00135930|transcript|ENST00000479834.1|processed_transcript|3/3|n.562-2A>G||||||,G|downstream
ot|ENST00000373563.8|protein_coding|27/29|c.3629_3630insGC|p.Gln1211fs|3825/5847|3630/3900
ot|ENST00000373563.8|protein_coding|27/29|c.3629_3630insGC|p.Gln1211fs|3825/5847|3630/3900
cript|ENST00000373563.8|protein_coding|27/29|c.3630_3631insG|p.Gln1211fs|3826/5847|3631/390
cript|ENST00000373563.8|protein_coding|27/29|c.3630_3631insG|p.Gln1211fs|3826/5847|3631/390
nteraction|1SNT:A_17-A_41:ENST00000233840|protein_coding|1/2|c.122G>A||||||,A|structural_inte
teraction|1VCU:A_230-A_251:ENST00000233840|protein_coding|2/2|c.753C>T||||||,T|structural_in
ot|ENST00000491070.1|nonsense_mediated_decay|1/2|c.119_120dupTG|p.His41fs|122/528|121/129
00168918|transcript|ENST00000491070.1|nonsense_mediated_decay|1/1|c.*344-1G>C||||||WARNIN
intron_variant|HIGH|DGKD|ENSG00000077044|transcript|ENST00000447484.5|protein_coding|1/5|c
00185038|transcript|ENST00000389758.3|protein_coding|35/41|c.4113+1G>A||||||,A|missense_varia
cript|ENST00000409457.5|protein_coding|10/10|c.1136_1139dupCAGG|p.Pro381fs|1738/2165|1140/
00198612|transcript|ENST00000419015.1|nonsense_mediated_decay|2/5|c.150-1G>A||||||,A|upstre
NSG00000258984|transcript|ENST00000449191.1|nonsense_mediated_decay|7/10|c.*67-3dupT|||||
NSG00000258984|transcript|ENST00000449191.1|nonsense_mediated_decay|7/10|c.*67-3dupT|||||
NSG00000258984|transcript|ENST00000449191.1|nonsense_mediated_decay|7/10|c.*67-3dupT|||||
0000283635|transcript|ENST00000637634.1|antisense|1/3|n.671+2T>G||||||,G|upstream_gene_var
00000226992|transcript|ENST00000441444.1|lincRNA|1/1|n.439-2A>G||||||
NST00000408934.1|protein_coding|1/1|c.143_170delTGGCCCTTCATCCACGGCTCTTCACCCA|p.Met48fs
221961|transcript|ENST00000408934.1|protein_coding|1/1|c.24_37dupTCTTCATCCACGGC|p.Pro13fs|
teraction|4AD7:D_264-D_421:ENST00000264039|protein_coding|4/9|c.790G>A||||||,A|missense_va

00144504|transcript|ENST00000401804.5|protein_coding|1/17|c.-17-1G>T||||||,A|splice_acceptor_
|ENST00000407834.3|protein_coding|1/3|c.455delG|p.Ser152fs|518/1094|455/924|152/307||,G|fra
0000184945|transcript|ENST00000429564.1|protein_coding|2/4|c.133delG|p.Val45fs|196/1110|133/
teraction|1H0C:A_78-A_218:ENST00000307503|protein_coding|6/11|c.654G>A||||||,A|structural_ir
226321|transcript|ENST00000443866.2|protein_coding|9/31|c.1251+2T>C||||||,C|splice_donor_vari
00406593.1|protein_coding|2/3|c.2T>C|p.Met1?|93/802|2/582|1/193||,G|structural_interaction_va
teraction|3DLS:E_1096-E_1266:ENST00000234040|protein_coding|17/18|c.3797T>G||||||,C|structur
teraction|3DLS:A_996-A_1019:ENST00000234040|protein_coding|12/18|c.3057G>A||||||,T|structura
ST00000358649.8|protein_coding|10/18|c.2579delC|p.Pro860fs|2643/4326|2579/3993|860/1330||,
433589.5|protein_coding|1/4|c.2T>C|p.Met1?|4/578|2/576|1/191||WARNING_TRANSCRIPT_NO_ST
|ENSG00000006607|transcript|ENST00000491425.1|processed_transcript|4/4|n.412-3dupT||||||INF
|ENSG00000006607|transcript|ENST00000491425.1|processed_transcript|4/4|n.412-3dupT||||||INF
|ENST00000445308.1|protein_coding|1/3|c.164_194delCGTGGCCCACCCACCATGTCCTTCCCTCCCC|p.P
NST00000453040.5|nonsense_mediated_decay|1/25|c.22dupG|p.Glu8fs|26/3960|23/282|8/93||INF
NST00000453040.5|nonsense_mediated_decay|1/25|c.22dupG|p.Glu8fs|26/3960|23/282|8/93||INF
ST00000453040.5|nonsense_mediated_decay|1/25|c.241delT|p.Trp81fs|244/3960|241/282|81/93||
ST00000453040.5|nonsense_mediated_decay|1/25|c.241delT|p.Trp81fs|244/3960|241/282|81/93||
ST00000453040.5|nonsense_mediated_decay|1/25|c.241delT|p.Trp81fs|244/3960|241/282|81/93||
ST00000453040.5|nonsense_mediated_decay|1/25|c.241delT|p.Trp81fs|244/3960|241/282|81/93||
0000397479.6|nonsense_mediated_decay|1/22|c.26C>A|p.Ser9*|104/3519|26/192|9/63||,A|stop_g
00000237990|transcript|ENST00000629672.1|antisense|2/3|n.281-1G>A||||||,T|downstream_gene_
GH|AC024060.1|ENSG00000281584|transcript|ENST00000629101.1|protein_coding|1/1|c.15-5_15-2
GH|AC024060.1|ENSG00000281584|transcript|ENST00000629101.1|protein_coding|1/1|c.15-5_15-2
GH|AC024060.1|ENSG00000281584|transcript|ENST00000629101.1|protein_coding|1/1|c.15-5_15-2
GH|AC024060.1|ENSG00000281584|transcript|ENST00000629101.1|protein_coding|1/1|c.15-5_15-2
000229642|transcript|ENST00000425894.1|lincRNA|1/2|n.240+1G>C||||||
nteraction|3MQ4:A_187-A_470:ENST00000467425|nonsense_mediated_decay|2/13|c.561T>C||||||,
NST00000440923.7|nonsense_mediated_decay|10/12|c.2710delT|p.Trp904fs|2859/3088|2710/2742
00000227110|transcript|ENST00000458666.1|antisense|1/3|n.44-1G>A||||||,T|intron_variant|MOD
196220|transcript|ENST00000618999.4|protein_coding|9/20|c.*164+1G>C||||||,G|splice_donor_var
|HIGH|DUSP5P2|ENSG00000235338|transcript|ENST00000426873.1|processed_pseudogene|2/2|n.6
|HIGH|DUSP5P2|ENSG00000235338|transcript|ENST00000426873.1|processed_pseudogene|2/2|n.6
|HIGH|DUSP5P2|ENSG00000235338|transcript|ENST00000426873.1|processed_pseudogene|2/2|n.6
|HIGH|DUSP5P2|ENSG00000235338|transcript|ENST00000426873.1|processed_pseudogene|2/2|n.6
|HIGH|DUSP5P2|ENSG00000235338|transcript|ENST00000426873.1|processed_pseudogene|2/2|n.6
NST00000383832.7|protein_coding|21/21|c.1633_1634delAG|p.Ser545fs|1823/2068|1633/1662|545
4021|transcript|ENST00000496526.5|retained_intron|6/11|n.3733+2C>T||||||,T|upstream_gene_var
00426895.8|protein_coding|11/13|c.2110C>T|p.Arg704*|2129/2767|2110/2748|704/915||,T|stop_
|ENST00000444133.6|protein_coding|7/7|c.1317_1320delAGTG|p.Gly441fs|1463/1619|1317/1359|
0144559|transcript|ENST00000457498.5|nonsense_mediated_decay|8/9|c.*1658+2T>G||||||,C|intro
nteraction|3T03:A_355-A_477:ENST00000287820|protein_coding|7/7|c.1431C>T||||||,T|structural_
NST00000619221.4|protein_coding|2/25|c.228delA|p.Arg78fs|228/7977|228/3423|76/1140||,C|5_pr
G00000224660|transcript|ENST00000420195.1|antisense|2/2|n.5373-3dupC||||||INFO_REALIGN_3_
T00000617544.3|protein_coding|5/5|c.488C>A|p.Ser163*|509/2775|488/591|163/196||,A|synonym
0182568|transcript|ENST00000457005.5|protein_coding|1/3|c.-124-2delA||||||WARNING_TRANSCRI
0231304|transcript|ENST00000634837.1|lincRNA|4/7|n.379+2C>T||||||,T|intron_variant|MODIFIER
0151789|transcript|ENST00000478967.5|processed_transcript|1/4|n.169+1G>A||||||,T|intron_variar

0226183|transcript|ENST00000418085.1|processed_pseudogene|1/1|n.149-1A>G|||||
0163491|transcript|ENST00000435584.5|protein_coding|5/5|c.459-1G>C|||||WARNING_TRANSCRIP
00000203506|transcript|ENST00000629416.2|antisense|1/4|n.135-1G>A|||||,T|splice_acceptor_var
0000360311.4|protein_coding|2/2|c.427C>T|p.Arg143*|976/3098|427/1899|143/632||,T|downstrea
000182973|transcript|ENST00000331889.10|protein_coding|2/17|c.118-3dupT|||||INFO_REALIGN_
000182973|transcript|ENST00000331889.10|protein_coding|2/17|c.118-3dupT|||||INFO_REALIGN_
3|interaction|2OEX:A_421-A_523:ENST00000307296|protein_coding|12/18|c.1569G>A|||||,A|struc
ENST00000447745.5|protein_coding|7/7|c.585delA|p.Asp196fs|585/793|585/591|195/196||WARNIN
t|ENST00000273173.4|protein_coding|3/10|c.713_716delTGTT|p.Leu238fs|804/2202|713/1785|238
teraction|4BC2:A_25-A_474:ENST00000207870|protein_coding|2/19|c.73G>C|||||,C|structural_inte
teraction|4BC2:A_17-A_50:ENST00000207870|protein_coding|3/19|c.150T>C|||||,C|structural_inte
teraction|4BC2:A_104-A_125:ENST00000207870|protein_coding|5/19|c.375G>T|||||,T|structural_in
teraction|4BC2:A_103-A_190:ENST00000207870|protein_coding|7/19|c.570G>A|||||,A|structural_i
G00000235339|transcript|ENST00000418195.1|processed_pseudogene|1/1|n.216-3dupA|||||INFO_
00235339|transcript|ENST00000418195.1|processed_pseudogene|1/1|n.216-3delA|||||INFO_REALI
0000232354|transcript|ENST00000593611.3|antisense|5/6|n.506-1G>C|||||,G|splice_region_varian
nteraction|2HE9:A_63-A_168:ENST00000232978|protein_coding|4/17|c.189G>A|||||,A|synonymous
ENST00000436261.5|protein_coding|4/4|c.1476_1477delGA|p.Asn494fs|1638/1968|1476/1632|492/
000296125.8|protein_coding|7/14|c.806G>A|p.Trp269*|874/3388|806/2055|269/684||,A|upstream_
0144791|transcript|ENST00000273317.4|protein_coding|1/7|c.1409-2delA|||||INFO_REALIGN_3_PR
ST00000292303.4|protein_coding|2/2|c.554_585delGTCAGTATCAATTCTGGAAGAATTTCCAGACA|p.Ser
NST00000444264.1|protein_coding|2/3|c.136C>T|p.Arg46*|367/1826|136/270|46/89||,T|downstrea
88086|transcript|ENST00000620911.4|protein_coding|2/3|c.388+1A>G|||||,C|intron_variant|MOD
00447340.1|protein_coding|1/3|c.2T>C|p.Met1?|56/1280|2/303|1/100||,G|intron_variant|MODIFIE
ion_variant&intron_variant|HIGH|PTPN23|ENSG00000076201|transcript|ENST00000265562.4|protein
ENST00000265562.4|protein_coding|20/25|c.2867_2868delAG|p.Gln956fs|2944/5244|2867/4911|95
nt&intron_variant|HIGH|SHISA5|ENSG00000164054|transcript|ENST00000612611.1|protein_coding|6
teraction|2Y6E:A_315-A_871:ENST00000265560|protein_coding|20/22|c.2613C>T|||||,A|structural
teraction|2Y6E:A_846-A_867:ENST00000265560|protein_coding|19/22|c.2536A>G|||||,C|structural
ST00000416417.5|protein_coding|7/7|c.834delT|p.Phe278fs|846/1536|834/942|278/313||,C|upstre
000449682.2|protein_coding|17/18|c.1951C>T|p.Arg651*|2313/2621|1951/2178|651/725||,A|stop_
173531|transcript|ENST00000449682.2|protein_coding|7/17|c.848-2A>C|||||,G|splice_acceptor_va
NST00000454079.5|nonsense_mediated_decay|14/20|c.2077_2080delTTTT|p.Phe693fs|2897/4324|2
NST00000454079.5|nonsense_mediated_decay|14/20|c.2077_2080delTTTT|p.Phe693fs|2897/4324|2
NST00000454079.5|nonsense_mediated_decay|14/20|c.2077_2080delTTTT|p.Phe693fs|2897/4324|2
NST00000454079.5|nonsense_mediated_decay|14/20|c.2077_2080delTTTT|p.Phe693fs|2897/4324|2
NST00000454079.5|nonsense_mediated_decay|14/20|c.2077_2080delTTTT|p.Phe693fs|2897/4324|2
NST00000454079.5|nonsense_mediated_decay|14/20|c.2077_2080delTTTT|p.Phe693fs|2897/4324|2
004534|transcript|ENST00000421682.1|protein_coding|3/4|c.77-2delA|||||INFO_REALIGN_3_PRIM
004534|transcript|ENST00000421682.1|protein_coding|3/4|c.77-2delA|||||INFO_REALIGN_3_PRIM
00188338|transcript|ENST00000614032.4|protein_coding|11/15|c.936-2_936-1delAG|||||,C|splice_
00188338|transcript|ENST00000614032.4|protein_coding|11/15|c.936-1G>C|||||,C|splice_accepto
000446079.5|nonsense_mediated_decay|4/10|c.262C>T|p.Arg88*|649/2086|262/441|88/146||,T|m
000145050|transcript|ENST00000528157.5|protein_coding|1/4|c.-5_1delGGAGGA|p.Met1del|283/11
0145050|transcript|ENST00000528157.5|protein_coding|1/4|c.2_7delTGAGGA|p.Met1_Arg2del|289/1
0145050|transcript|ENST00000528157.5|protein_coding|1/4|c.2_4delTGA|p.Met1del|289/1153|2/55
0145050|transcript|ENST00000528157.5|protein_coding|1/4|c.2_4delTGA|p.Met1del|289/1153|2/55

0528157.5|protein_coding|1/4|c.2T>G|p.Met1?|289/1153|2/558|1/185||,G|TF_binding_site_variant
0528157.5|protein_coding|1/4|c.2T>G|p.Met1?|289/1153|2/558|1/185||,G|TF_binding_site_variant
nteraction|2W0I:A_243-A_313:ENST00000305533|protein_coding|7/9|c.729C>T||||||,A|synonymous
00168237|transcript|ENST00000486393.5|nonsense_mediated_decay|3/4|c.530-2A>G||||||,G|splice
ENST00000420323.6|protein_coding|2/78|c.191_192delTG|p.Leu64fs|452/13104|191/12798|64/426
ENST00000420323.6|protein_coding|2/78|c.198_199delGG|p.Ala67fs|459/13104|198/12798|66/426
00010318|transcript|ENST00000327906.7|protein_coding|10/10|c.920-3_920-2dupCA||||||INFO_RE
00010327|transcript|ENST00000321725.10|protein_coding|48/68|c.5035-3dupC||||||INFO_REALIGN
ST00000405128.3|protein_coding|1/6|c.14delT|p.Leu5fs|153/1122|14/834|5/277||,C|upstream_ger
nteraction|1YRK:A_6-A_60:ENST00000330452|protein_coding|4/19|c.180T>C||||||,C|structural_inte
eraction|3MOS:A_88-A_283:ENST00000462138|protein_coding|7/14|c.848A>T||||||,A|structural_int
eraction|3MOS:A_151-A_181:ENST00000462138|protein_coding|5/14|c.541A>G||||||,C|structural_ir
31|transcript|ENST00000296289.10|protein_coding|5/14|c.-4+2C>T||||||,A|structural_interaction_v
0000288167.7|protein_coding|11/11|c.1450C>T|p.Gln484*|1459/2010|1450/1509|484/502||,T|upstr
nteraction|4FO0:A_105-A_400:ENST00000335754|protein_coding|10/13|c.1200C>T||||||,A|synonym
nteraction|4FO0:A_56-A_286:ENST00000335754|protein_coding|2/13|c.167C>T||||||,A|missense_va
nteraction|2ELB:A_12-A_175:ENST00000288266|protein_coding|1/22|c.36G>C||||||,C|structural_int
NSG00000244383|transcript|ENST00000464125.1|antisense|2/3|n.315-3dupT||||||INFO_REALIGN_3_
NSG00000244383|transcript|ENST00000464125.1|antisense|2/3|n.315-3dupT||||||INFO_REALIGN_3_
NSG00000244383|transcript|ENST00000464125.1|antisense|2/3|n.315-3dupT||||||INFO_REALIGN_3_
nteraction|3JXH:C_247-C_320:ENST00000474889|protein_coding|8/30|c.960C>T||||||,T|structural_i
nteraction|2H4V:A_878-A_908:ENST00000474889|protein_coding|18/30|c.2724T>C||||||,C|structur
00241359|transcript|ENST00000488201.1|antisense|3/4|n.294+2T>C||||||,G|intron_variant|MODIF
0000163376|transcript|ENST00000417314.2|protein_coding|3/3|c.1343-3dupT||||||INFO_REALIGN_3
0000163376|transcript|ENST00000417314.2|protein_coding|3/3|c.1343-3dupT||||||INFO_REALIGN_3
LG2-AS1|ENSG00000241316|transcript|ENST00000482677.5|lincRNA|6/6|n.1654-13_1654-2dupTTTA
|SUCLG2-AS1|ENSG00000241316|transcript|ENST00000482677.5|lincRNA|6/6|n.1654-17_1654-2dup
HIGH|SUCLG2-AS1|ENSG00000241316|transcript|ENST00000482677.5|lincRNA|6/6|n.1654-21_1654-
iant|HIGH|SUCLG2-AS1|ENSG00000241316|transcript|ENST00000482677.5|lincRNA|6/6|n.1654-25_1
00000183662|transcript|ENST00000491017.1|processed_transcript|3/4|n.387-3dupT||||||INFO_REAL
0000114861&ENSG00000277855|gene_variant|ENSG00000114861|||n.71583997_71583984delAGGGA
ENST00000533473.1|protein_coding|1/1|c.250dupA|p.Arg84fs|674/1679|251/819|84/272||INFO_RE
228868|transcript|ENST00000509077.1|unprocessed_pseudogene|2/4|n.336+1G>A||||||
0244026|transcript|ENST00000477247.6|transcribed_unprocessed_pseudogene|1/7|n.96+2G>T|||||
0239959|transcript|ENST00000462675.1|unprocessed_pseudogene|1/2|n.165+1G>C||||||
0000478296.5|protein_coding|4/4|c.1465G>T|p.Glu489*|1742/3875|1465/2595|489/864||,T|misser
27124|transcript|ENST00000471541.2|protein_coding|2/4|c.-94+1G>A||||||WARNING_TRANSCRIPT_
interaction|2V9Q:A_206-A_233:ENST00000464233|protein_coding|6/31|c.699T>C||||||,G|synonym
teraction|4BZY:A_507-A_576:ENST00000429644|protein_coding|12/16|c.1519A>G||||||,C|structura
teraction|4BZY:B_190-B_352:ENST00000429644|protein_coding|5/16|c.568A>G||||||,C|structural_ir
G00000239440|transcript|ENST00000470263.5|lincRNA|8/8|n.1191+2T>C||||||
00242641|transcript|ENST00000484892.5|lincRNA|17/20|n.2850+1G>C||||||
nscript|ENST00000637986.1|protein_coding|2/2|c.2009delA|p.Lys670fs|2510/2706|2009/2205|670/7
|ENST00000637986.1|protein_coding|2/2|c.2159G>A|p.Trp720*|2660/2706|2159/2205|720/734||,A
interaction|4P5Z:A_800-A_865:ENST00000336596|protein_coding|14/17|c.2400G>A||||||,A|synonyr
nteraction|2XDV:A_169-A_251:ENST00000333396|protein_coding|5/10|c.753T>G||||||,C|structural_i
nteraction|2XDV:A_68-A_227:ENST00000333396|protein_coding|4/10|c.679A>C||||||,G|structural_ir

3185|transcript|ENST00000472788.5|protein_coding|6/9|c.615T>A|p.Tyr205*|617/1416|615/1404|
ENST00000429239.1|polymorphic_pseudogene|1/1|c.258delT|p.Phe86fs|258/925|258/925|86/307|
78|transcript|ENST00000358642.2|protein_coding|1/1|c.928T>C|p.Ter310Glnext*?|928/930|928/93
|ENST00000356526.2|protein_coding|1/1|c.307delT|p.Ser103fs|307/942|307/942|103/313||INFO_R
0000394191.2|polymorphic_pseudogene|1/1|c.551T>A|p.Leu184*|551/931|551/931|184/309||WAR
NST00000354924.2|protein_coding|1/1|c.901delA|p.Ile301fs|901/966|901/966|301/321||INFO_REA
|ENST00000383695.1|protein_coding|1/1|c.904dupA|p.Ile302fs|905/966|905/966|302/321||INFO_R
0000427338.2|protein_coding|1/1|c.654T>A|p.Tyr218*|731/1060|654/951|218/316||,A|sequence_fe
teraction|2AEX:A_330-A_391:ENST00000264193|protein_coding|5/7|c.990A>G||||||,C|synonymous
0000239445|transcript|ENST00000461931.1|antisense|3/3|n.452+1G>A||||||,T|splice_donor_varian
NST00000460317.1|nonsense_mediated_decay|1/4|c.54dupG|p.Arg19fs|71/648|55/135|19/44||INF
00284322.9|protein_coding|1/35|c.1A>G|p.Met1?|111/4498|1/3228|1/1075||,C|start_lost|HIGH|AB
teraction|3ZNI:A_353-A_424:ENST00000264122|protein_coding|10/19|c.1272C>T||||||,A|structural
000243701|transcript|ENST00000473550.1|lincRNA|1/1|n.368-3dupT||||||INFO_REALIGN_3_PRIME,
000243701|transcript|ENST00000473550.1|lincRNA|1/1|n.368-3dupT||||||INFO_REALIGN_3_PRIME,
000243701|transcript|ENST00000473550.1|lincRNA|1/1|n.368-3dupT||||||INFO_REALIGN_3_PRIME,
ISG00000239828|transcript|ENST00000596305.5|lincRNA|2/2|n.268-2A>G||||||,C|intron_variant|M
3919|transcript|ENST00000497905.5|protein_coding|2/4|c.-73+1G>C||||||WARNING_TRANSCRIPT_N
00000163515|transcript|ENST00000295755.6|protein_coding|1/3|c.32_39dupTAATCCCC|p.Leu14fs|2
138472|transcript|ENST00000261047.7|protein_coding|3/3|c.442+1G>T||||||,A|missense_variant|M
00000261047.7|protein_coding|1/4|c.52G>T|p.Glu18*|185/982|52/630|18/209||,A|stop_gained|HIG
00335658.6|protein_coding|1/7|c.2T>C|p.Met1?|57/2817|2/915|1/304||,G|start_lost|HIGH|DPPA4|
000114529|transcript|ENST00000264848.9|protein_coding|3/5|c.397-3dupT||||||INFO_REALIGN_3_
NST00000402314.6|protein_coding|11/11|c.920dupT|p.Leu307fs|1308/3010|920/936|307/311||,CA
ot|ENST00000398214.5|protein_coding|4/6|c.608delA|p.His203fs|834/1296|608/816|203/271||,G|f
H|BOC|ENSG00000144857|transcript|ENST00000495514.5|protein_coding|2/20|c.-83_-82+1delAAG|
06530|transcript|ENST00000461734.1|nonsense_mediated_decay|9/9|c.*63+2C>T||||||WARNING_
000308095.4|nonsense_mediated_decay|1/2|c.735T>G|p.Tyr245*|1239/2555|735/822|245/273||,C
0000181722|gene_variant|ENSG00000259976|||n.114329722_114329710delTTTTTTTTTTTTT||||||,G
0000181722|gene_variant|ENSG00000259976|||n.114329722_114329710delTTTTTTTTTTTTT||||||,G
0000181722|gene_variant|ENSG00000259976|||n.114329722_114329710delTTTTTTTTTTTTT||||||,G
0000181722|gene_variant|ENSG00000259976|||n.114329722_114329710delTTTTTTTTTTTTT||||||,G
0000181722|gene_variant|ENSG00000259976|||n.114329722_114329710delTTTTTTTTTTTTT||||||,G
000241560|transcript|ENST00000460210.1|antisense|2/3|n.144+1G>A||||||,A|intron_variant|MODI
NST00000474851.1|protein_coding|2/5|c.86delG|p.Gly29fs|242/772|86/616|29/204||WARNING_TR
000121578|transcript|ENST00000487579.5|processed_transcript|4/4|n.478-1G>C||||||,G|upstream_
0000492164.5|nonsense_mediated_decay|2/6|c.272G>C|p.Ter91Serext*?|431/725|272/273|91/90||
teraction|1EY2:A_318-A_397:ENST00000283871|protein_coding|14/14|c.1191A>C||||||,G|splice_reg
4840|transcript|ENST00000468192.1|processed_transcript|1/4|n.204+1G>T||||||,A|intron_variant|
IST00000264233.5|protein_coding|16/30|c.4262_4268delTACTATT|p.Ile1421fs|4397/8775|4262/777
000334384.3|protein_coding|2/4|c.202C>T|p.Arg68*|212/992|202/948|68/315||
ction|3TJ3:B_395-D_20:ENST00000344337-ENST00000347635|protein_coding|12/14|c.1185G>A||||
|interaction|3Q6Z:A_883-A_922:ENST00000474629|protein_coding|6/17|c.2649C>T||||||,T|structura
73193|transcript|ENST00000460683.1|nonsense_mediated_decay|12/13|c.*1163+1G>A||||||WARN
0291478.9|protein_coding|1/27|c.1A>G|p.Met1?|164/10888|1/3870|1/1289||,G|start_lost|HIGH|K
nteraction|2V30:A_440-A_477:ENST00000232607|protein_coding|6/6|c.1320C>T||||||,T|synonymou
nteraction|2JGY:A_414-A_446:ENST00000232607|protein_coding|6/6|c.1336A>G||||||,G|structural_

ISG00000241288|transcript|ENST00000626334.1|lincRNA|4/5|n.339-1G>C||||||,G|downstream_gen
IGH|ROPN1B|ENSG00000114547|transcript|ENST00000508088.1|protein_coding|2/7|c.-260-1_-251d
|ENST00000315891.10|protein_coding|12/12|c.1502_1505delTGTT|p.Leu501fs|1744/1797|1502/152
114544|transcript|ENST00000504118.1|processed_transcript|1/1|n.170+1G>C||||||,G|3_prime_UTI
00000505024.1|protein_coding|4/16|c.399G>A|p.Trp133*|428/1994|399/1839|133/612||,A|stop_g
H|C3orf22|ENSG00000180697|transcript|ENST00000505070.5|nonsense_mediated_decay|5/6|c.*202
nteraction|3PE6:A_151-A_241:ENST00000434178|protein_coding|7/8|c.723A>G||||||,C|structural_in
ction|1YHN:A_73-B_249:ENST00000265062-ENST00000301336|protein_coding|4/6|c.219C>T||||||,T
G00000244932|transcript|ENST00000499853.2|unprocessed_pseudogene|3/4|n.270+1dupG|||||INF
071|transcript|ENST00000509828.1|nonsense_mediated_decay|2/4|c.106C>T|p.Gln36*|200/576|106
00000250643|transcript|ENST00000509201.1|antisense|1/3|n.97+2T>C|||||
ENST00000425059.1|protein_coding|6/8|c.469delC|p.Leu157fs|655/969|469/648|157/215||INFO_R
0000253540|transcript|ENST00000513466.5|transcribed_unprocessed_pseudogene|1/7|n.97-2A>C|||
0000312481.11|nonsense_mediated_decay|38/42|c.6814G>T|p.Glu2272*|7308/9226|6814/7848|22
ENST00000312481.11|nonsense_mediated_decay|40/42|c.7772delT|p.Leu2591fs|8266/9226|7772/7
240303|transcript|ENST00000264990.10|protein_coding|11/19|c.1414+1G>T||||||,A|splice_donor_v
00000248724|transcript|ENST00000489343.5|antisense|2/4|n.697-1G>T||||||,T|splice_acceptor_vari
00248724|transcript|ENST00000504440.1|antisense|4/10|n.330+1G>A|||||
|interaction|3UEN:A_598-A_637:ENST00000260810|protein_coding|12/28|c.1911T>C||||||,G|struct
nteraction|2E9S:A_27-A_68:ENST00000285208|protein_coding|2/8|c.81G>A||||||,T|structural_inter
G00000073711|transcript|ENST00000264977.7|protein_coding|12/13|c.3223-5_3223-2dupATCA||||
158186|transcript|ENST00000423968.6|protein_coding|5/5|c.528-2delA|||||INFO_REALIGN_3_PRIM
|ENST00000383165.3|protein_coding|2/3|c.200_209delATTCGAAGGC|p.His67fs|331/3538|200/528|
175110|transcript|ENST00000498505.5|nonsense_mediated_decay|4/8|c.*101+1G>A||||||,A|splice_
4|interaction|1NUP:A_50-A_87:ENST00000296202|protein_coding|4/6|c.150C>T||||||,A|structural_i
000250433|transcript|ENST00000513309.1|antisense|1/1|n.2138+2T>C||||||,G|intron_variant|MOD
nteraction|2V24:A_77-A_218:ENST00000310546|protein_coding|2/3|c.229C>A||||||,A|structural_int
55893|transcript|ENST00000505013.5|protein_coding|1/4|c.-54+2T>G|||||WARNING_TRANSCRIPT_
SG00000241570|transcript|ENST00000598139.5|antisense|1/1|n.141-3dupT|||||INFO_REALIGN_3_
SG00000241570|transcript|ENST00000598139.5|antisense|1/1|n.141-3dupT|||||INFO_REALIGN_3_
SG00000241570|transcript|ENST00000598139.5|antisense|1/1|n.141-3dupT|||||INFO_REALIGN_3_
00000488497.7|nonsense_mediated_decay|13/27|c.1261A>T|p.Arg421*|1722/3557|1261/1278|421/
teraction|3Q4S:A_102-A_135:ENST00000345003|protein_coding|3/8|c.304G>C||||||,C|structural_in
00310053.9|protein_coding|2/25|c.33G>A|p.Trp11*|227/5317|33/3030|11/1009||,T|stop_gained|H
018408|transcript|ENST00000360632.7|protein_coding|7/7|c.1199_1200insTTAA|p.Ter401fs|1539/2
018408|transcript|ENST00000460517.1|protein_coding|1/1|c.-4+2C>T|||||WARNING_TRANSCRIPT_
00000241048|transcript|ENST00000474444.1|lincRNA|4/5|n.349+1G>A||||||,T|downstream_gene_v
G00000244382|transcript|ENST00000474598.1|lincRNA|1/1|n.147-1G>A||||||,T|intron_variant|MOD
0000489791.1|protein_coding|2/2|c.249C>A|p.Tyr83*|249/524|249/261|83/86||WARNING_TRANSCF
G00000243486|transcript|ENST00000463297.1|lincRNA|4/5|n.209+1G>A||||||,A|intron_variant|MOD
5549|transcript|ENST00000462837.5|protein_coding|1/5|c.-11+1G>A|||||WARNING_TRANSCRIPT_II
nteraction|2VXO:A_456-A_528:ENST00000496455|protein_coding|13/16|c.1583A>C||||||,C|structura
00000475456.5|protein_coding|1/9|c.90T>A|p.Tyr30*|110/552|90/532|30/176||WARNING_TRANSCF
ENST00000356539.8|protein_coding|7/13|c.818_819insT|p.Glu273fs|933/2572|819/2079|273/692||
97415|transcript|ENST00000494677.5|protein_coding|1/4|c.-420+2T>C||||||,G|splice_donor_variant
eraction|2BO9:B_48-B_86:ENST00000264265|protein_coding|2/6|c.143G>A||||||,T|structural_intera
ENST00000309784.8|protein_coding|3/3|c.1107_1110delATCT|p.Ser370fs|1296/3867|1107/1212|36

)000240567|transcript|ENST00000473595.1|lincRNA|3/3|n.1446+1G>A|||||
5|interaction|3F02:A_192-A_219:ENST00000295777|protein_coding|4/9|c.576G>A||||||,A|structura
87033|transcript|ENST00000487910.1|nonsense_mediated_decay|5/8|c.*106+2T>C||||||,C|intron_v
)0075420|transcript|ENST00000476794.1|processed_transcript|1/2|n.37-1G>C||||||,C|intron_variar
3|interaction|1D0G:A_126-A_275:ENST00000241261|protein_coding|5/5|c.825T>C||||||,G|structura
3|interaction|1D4V:B_141-B_171:ENST00000241261|protein_coding|5/5|c.513T>C||||||,G|structural
)0114346|transcript|ENST00000417960.5|protein_coding|1/24|c.-345-3dupT||||||INFO_REALIGN_3_
)0114346|transcript|ENST00000417960.5|protein_coding|1/24|c.-345-3dupT||||||INFO_REALIGN_3_
|ENST00000637659.1|nonsense_mediated_decay|9/12|c.783_790delGATGGATG|p.Trp261fs|791/310
|ENST00000637659.1|nonsense_mediated_decay|9/12|c.783_790delGATGGATG|p.Trp261fs|791/310
|ENST00000637659.1|nonsense_mediated_decay|9/12|c.783_790delGATGGATG|p.Trp261fs|791/310
|ENST00000637659.1|nonsense_mediated_decay|9/12|c.783_790delGATGGATG|p.Trp261fs|791/310
|ENST00000637659.1|nonsense_mediated_decay|9/12|c.783_790delGATGGATG|p.Trp261fs|791/310
GH|RP11-33A14.1|ENSG00000223930|transcript|ENST00000411727.1|lincRNA|1/1|n.191-25_191-2d
GH|RP11-33A14.1|ENSG00000223930|transcript|ENST00000411727.1|lincRNA|1/1|n.191-25_191-2d
GH|RP11-33A14.1|ENSG00000223930|transcript|ENST00000411727.1|lincRNA|1/1|n.191-25_191-2d
GH|RP11-33A14.1|ENSG00000223930|transcript|ENST00000411727.1|lincRNA|1/1|n.191-25_191-2d
GH|RP11-33A14.1|ENSG00000223930|transcript|ENST00000411727.1|lincRNA|1/1|n.191-25_191-2d
GH|RP11-33A14.1|ENSG00000223930|transcript|ENST00000411727.1|lincRNA|1/1|n.191-25_191-2d
G00000197584|transcript|ENST00000422927.1|nonsense_mediated_decay|2/4|c.57-3dupT||||||INF
G00000197584|transcript|ENST00000422927.1|nonsense_mediated_decay|2/4|c.57-3dupT||||||INF
G00000197584|transcript|ENST00000422927.1|nonsense_mediated_decay|2/4|c.57-3dupT||||||INF
interaction|4JPS:A_334-A_391:ENST00000263967|protein_coding|7/21|c.1173A>G||||||,G|structura
|ENST00000314235.9|protein_coding|4/4|c.765delA|p.Val256fs|1277/1485|765/840|255/279||,C|fr
script|ENST00000472629.5|protein_coding|2/6|c.148_151dupGTCT|p.Phe51fs|167/744|152/534|51/
:|ENST00000442201.6|protein_coding|19/20|c.2660dupT|p.Ser888fs|2780/3279|2660/2826|887/941
0000242512|transcript|ENST00000627881.2|lincRNA|2/4|n.319-2T>A||||||,A|splice_acceptor_variar
ISG00000244346|transcript|ENST00000460975.1|processed_pseudogene|2/3|n.122-2delA||||||,GTT
ISG00000244346|transcript|ENST00000460975.1|processed_pseudogene|2/3|n.122-2delA||||||,GTT
SG00000244346|transcript|ENST00000460975.1|processed_pseudogene|1/3|n.78-2delA||||||,CTT|s
SG00000244346|transcript|ENST00000460975.1|processed_pseudogene|1/3|n.78-2delA||||||,CTT|s
interaction|4AKM:A_243-A_356:ENST00000265598|protein_coding|5/6|c.1068C>T||||||,A|structura
)53524|transcript|ENST00000459750.5|retained_intron|10/11|n.1200+1G>A||||||,T|splice_donor_va
)0114796|transcript|ENST00000454652.6|protein_coding|1/8|c.-215-1G>A||||||,A|splice_acceptor_v
)000233441|transcript|ENST00000454440.6|transcribed_unprocessed_pseudogene|5/8|n.770-2A>C|
)186090|transcript|ENST00000428798.6|protein_coding|1/7|c.67-2A>C||||||,C|synonymous_variant
36090|transcript|ENST00000382489.3|protein_coding|4/7|c.511+1G>C||||||,C|missense_variant|MC
SG00000233576|transcript|ENST00000445553.1|unprocessed_pseudogene|4/8|n.382-2G>A||||||,A|
NST00000440596.2|protein_coding|1/7|c.64delG|p.Glu22fs|64/1449|64/1449|22/482||INFO_REALIG
teraction|2PXX:A_127-A_170:ENST00000324557|protein_coding|3/3|c.508C>T||||||,T|missense_vari
teraction|2PXX:A_171-A_211:ENST00000324557|protein_coding|3/3|c.513T>C||||||,C|synonymous_
interaction|3P1I:A_55-A_93:ENST00000330394|protein_coding|2/16|c.165A>G||||||,G|structural_in
nteraction|3P1I:A_80-A_200:ENST00000330394|protein_coding|3/16|c.240G>A||||||,A|structural_ir
nteraction|3P1I:A_108-A_206:ENST00000330394|protein_coding|3/16|c.618C>T||||||,T|structural_i
)00287611.6|protein_coding|11/11|c.1234C>T|p.Arg412*|1501/1720|1234/1284|412/427||,T|stop_
)00226652|transcript|ENST00000627340.1|processed_transcript|1/1|n.303+1delG||||||INFO_REALIG
)073849|transcript|ENST00000430309.5|protein_coding|1/3|c.-325+2T>A||||||WARNING_TRANSCRI

interaction|4KKD:A_523-A_617:ENST00000296280|protein_coding|11/11|c.1851G>A||||||,T|structu
interaction|4KKD:B_576-B_681:ENST00000296280|protein_coding|11/11|c.1727G>T||||||,A|missens
ENST00000264734.2|protein_coding|1/5|c.166delG|p.Ala56fs|414/3273|166/918|56/305||INFO_RE
27252|transcript|ENST00000264735.2|protein_coding|2/3|c.454+1G>A|||||
187527|transcript|ENST00000488957.1|retained_intron|1/2|n.803+1G>A||||||,T|intron_variant|MC
00000342358.8|protein_coding|10/30|c.1063C>T|p.Gln355*|1181/4027|1063/3657|355/1218||
00000229155|transcript|ENST00000439074.1|lincRNA|2/2|n.506+2T>C||||||,G|non_coding_transcrip
00323830.3|protein_coding|2/2|c.1525C>T|p.Gln509*|1615/3028|1525/1638|509/545||,A|stop_gai
0000428839.1|protein_coding|3/3|c.280G>T|p.Glu94*|393/3248|280/1764|94/587||,A|stop_gained
000163958|transcript|ENST00000397544.6|nonsense_mediated_decay|6/7|c.774-2A>G||||||,C|upst
0213123|transcript|ENST00000491186.5|processed_transcript|1/1|n.203+1G>A||||||,T|upstream_g
00000446879.5|protein_coding|6/6|c.748C>T|p.Arg250*|874/1582|748/900|250/299||,A|upstream
teraction|3RL7:D_278-D_306:ENST00000346964|protein_coding|10/26|c.833G>A||||||,T|structural_
00265239.10|protein_coding|4/12|c.47G>A|p.Trp16*|472/2262|47/1332|16/443||,T|stop_gained|H
on_coding_transcript_exon_variant|HIGH|LMLN-AS1|ENSG00000232832|transcript|ENST0000042346
3256|transcript|ENST00000465426.5|protein_coding|1/3|c.-127+2T>C|||||WARNING_TRANSCRIPT_
NST00000400151.6|protein_coding|4/4|c.484_502delTGCGTCCTCACACCTGATG|p.Cys162fs|860/1175
NST00000620529.1|protein_coding|1/1|c.286_289delCTTG|p.Leu96fs|1073/1899|286/399|96/132||
0000127419|transcript|ENST00000507319.5|protein_coding|3/4|c.193-2A>C|||||WARNING_TRANS
teraction|3W81:A_33-A_404:ENST00000247933|protein_coding|1/14|c.99T>G||||||,G|structural_int
teraction|3W81:A_89-A_181:ENST00000247933|protein_coding|5/14|c.543T>C||||||,C|structural_in
teraction|3W81:A_276-A_297:ENST00000247933|protein_coding|7/14|c.891C>T||||||,T|structural_i
teraction|4OBR:B_314-B_360:ENST00000247933|protein_coding|7/14|c.942G>C||||||,C|synonymou
teraction|3W81:A_310-A_361:ENST00000247933|protein_coding|8/14|c.1081G>A||||||,A|structural
teraction|3W81:A_449-A_532:ENST00000247933|protein_coding|9/14|c.1345C>A||||||,A|structural
teraction|3W81:A_454-A_530:ENST00000247933|protein_coding|9/14|c.1360G>A||||||,A|structural
_coding_transcript_exon_variant|HIGH|RNF212|ENSG00000178222|transcript|ENST00000514024.5|r
_coding_transcript_exon_variant|HIGH|RNF212|ENSG00000178222|transcript|ENST00000514024.5|r
0000333673.5|protein_coding|4/4|c.562C>T|p.Gln188*|628/1019|562/822|188/273||,A|downstrea
8222|transcript|ENST00000506730.5|nonsense_mediated_decay|2/10|c.112C>T|p.Arg38*|188/753|
nt&intron_variant|HIGH|SPON2|ENSG00000159674|transcript|ENST00000515004.5|protein_coding|1
0000196810|transcript|ENST00000514984.1|antisense|1/1|n.242-2A>C||||||,C|upstream_gene_vari
H|CRIPAK|ENSG00000179979|transcript|ENST00000324803.5|protein_coding|1/1|c.78_79insCACATG
ENST00000324803.5|protein_coding|1/1|c.81_82delTG|p.Ala28fs|3041/4438|81/1341|27/446||INFO
ENST00000324803.5|protein_coding|1/1|c.205_206delCG|p.Arg69fs|3165/4438|205/1341|69/446||
ENST00000324803.5|protein_coding|1/1|c.327_355delGTGCCCATGTGGAGTGCCCGCCTGCTCAC|p.Cys1
t|ENST00000324803.5|protein_coding|1/1|c.325_326dupAC|p.Cys110fs|3287/4438|327/1341|109/4
t|ENST00000324803.5|protein_coding|1/1|c.325_326dupAC|p.Cys110fs|3287/4438|327/1341|109/4
H|CRIPAK|ENSG00000179979|transcript|ENST00000324803.5|protein_coding|1/1|c.487_517dupGAC
H|CRIPAK|ENSG00000179979|transcript|ENST00000324803.5|protein_coding|1/1|c.518_548dupCAT
ENST00000324803.5|protein_coding|1/1|c.645_648delGTGG|p.Met215fs|3605/4438|645/1341|215/
ENST00000324803.5|protein_coding|1/1|c.650_738delGTGTTCGCCTGCTCACACGTGCCGATGCGGAGTG
ENST00000324803.5|protein_coding|1/1|c.650_738delGTGTTCGCCTGCTCACACGTGCCGATGCGGAGTG
ENST00000324803.5|protein_coding|1/1|c.666_667delAC|p.Arg223fs|3626/4438|666/1341|222/446|
ENST00000324803.5|protein_coding|1/1|c.666_667delAC|p.Arg223fs|3626/4438|666/1341|222/446|
nteraction|4XVK:A_441-A_559:ENST00000382865|protein_coding|9/24|c.1323T>C||||||,G|structural_
00503518.2|protein_coding|2/2|c.70C>T|p.Gln24*|507/2136|70/255|24/84||,T|upstream_gene_var

T00000355072.9|protein_coding|1/67|c.84_85delGC|p.Gln29fs|229/13474|84/9429|28/3142||,A|up
T00000355072.9|protein_coding|1/67|c.87_102delGCAGCAGCAGCAGCAG|p.Gln29fs|232/13474|87/9
T00000355072.9|protein_coding|1/67|c.93_94delGC|p.Gln32fs|238/13474|93/9429|31/3142||,A|up
6|transcript|ENST00000355072.9|protein_coding|1/67|c.96_102delGCAGCAG|p.Gln32fs|241/13474|
T00000355072.9|protein_coding|1/67|c.99_100delGC|p.Gln34fs|244/13474|99/9429|33/3142||,A|u
T00000355072.9|protein_coding|1/67|c.99_100delGC|p.Gln34fs|244/13474|99/9429|33/3142||,A|u
ENST00000355072.9|protein_coding|1/67|c.102delG|p.Gln34fs|247/13474|102/9429|34/3142||,A|
ENST00000355072.9|protein_coding|1/67|c.102delG|p.Gln34fs|247/13474|102/9429|34/3142||,A|
ENST00000355072.9|protein_coding|1/67|c.102delG|p.Gln34fs|247/13474|102/9429|34/3142||,A|
NST00000355072.9|protein_coding|1/67|c.118_119insAG|p.Pro40fs|264/13474|119/9429|40/3142|
T00000355072.9|protein_coding|1/67|c.121_122delCC|p.Pro41fs|266/13474|121/9429|41/3142||,A
T00000355072.9|protein_coding|1/67|c.121_122delCC|p.Pro41fs|266/13474|121/9429|41/3142||,A
T00000355072.9|protein_coding|1/67|c.121_122delCC|p.Pro41fs|266/13474|121/9429|41/3142||,A
T00000355072.9|protein_coding|1/67|c.121_122delCC|p.Pro41fs|266/13474|121/9429|41/3142||,A
interaction|1YBW:A_412-A_556:ENST00000382774|protein_coding|13/14|c.1668G>A||||||,A|structu
SG00000270090|transcript|ENST00000602543.1|lincRNA|4/4|n.473-2A>G||||||,G|downstream_gen
0000152953|transcript|ENST00000513705.5|processed_transcript|2/2|n.333-3dupT||||||INFO_REAL
0000152953|transcript|ENST00000513705.5|processed_transcript|2/2|n.333-3dupT||||||INFO_REAL
0000152953|transcript|ENST00000513705.5|processed_transcript|2/2|n.333-3dupT||||||INFO_REAL
52953|transcript|ENST00000508728.1|processed_transcript|1/6|n.163+2T>A||||||,A|intron_variant
81215|transcript|ENST00000531445.2|protein_coding|9/11|c.4104+1G>A||||||,T|splice_donor_vari
ENST00000531445.2|protein_coding|6/12|c.2903_2904delAG|p.Glu968fs|2980/6860|2903/4527|968
00000170846|transcript|ENST00000307533.10|lincRNA|1/1|n.1257-1G>A||||||,A|splice_acceptor_v
63995|transcript|ENST00000515079.1|processed_transcript|1/4|n.87+2T>C||||||,G|intron_variant|
37008|transcript|ENST00000508302.1|processed_transcript|1/5|n.41+2T>A||||||,T|downstream_ger
00000504134.1|protein_coding|1/2|c.153C>A|p.Cys51*|155/660|153/658|51/218||WARNING_TRAN
00000249148|transcript|ENST00000510061.2|processed_pseudogene|1/1|n.110-2_110-1delAA|||||
00000249148|transcript|ENST00000510061.2|processed_pseudogene|1/1|n.110-2_110-1delAA|||||
00247624|transcript|ENST00000500394.6|lincRNA|3/7|n.277+1G>A||||||,T|upstream_gene_variant|
NST00000538197.5|protein_coding|1/12|c.557_561delACCTT|p.His186fs|557/6878|557/3105|186/10
0000503658.2|protein_coding|6/7|c.262C>T|p.Arg88*|359/605|262/369|88/122||,T|stop_gained|H
14989.1|protein_coding|5/5|c.291A>T|p.Ter97Cysext*?|293/801|291/291|97/96||WARNING_TRANS
502843.5|nonsense_mediated_decay|3/7|c.368A>C|p.Ter123Serext*?|508/1560|368/369|123/122||
teraction|1YH3:A_103-A_194:ENST00000226279|protein_coding|4/8|c.581G>A||||||,A|structural_in
SG00000263327|transcript|ENST00000570786.1|antisense|2/2|n.235-3dupT||||||INFO_REALIGN_3_
SG00000263327|transcript|ENST00000570786.1|antisense|2/2|n.235-3dupT||||||INFO_REALIGN_3_
SG00000263327|transcript|ENST00000570786.1|antisense|2/2|n.235-3dupT||||||INFO_REALIGN_3_
514300.1|nonsense_mediated_decay|2/7|c.109T>A|p.Ter37Argext*?|261/781|109/111|37/36||,T|ir
00503945.2|nonsense_mediated_decay|5/6|c.540A>G|p.Ter180Trpext*?|540/879|540/540|180/179|
00000248515|transcript|ENST00000511431.1|lincRNA|2/3|n.288+2C>T||||||
teraction|2V9S:A_403-A_426:ENST00000504154|protein_coding|13/37|c.1209A>T||||||,T|structural_
SG00000251516|transcript|ENST00000504151.1|unprocessed_pseudogene|2/3|n.176-2delA||||||
508166.5|polymorphic_pseudogene|5/5|c.1368A>T|p.Ter456Tyrext*?|1470/2164|1368/1410|456/46
NSG00000249645|transcript|ENST00000503394.1|lincRNA|3/4|n.379-2A>G||||||
0181982|transcript|ENST00000389609.8|protein_coding|1/12|c.-65+2T>A||||||,T|splice_donor_varia
0000109680|transcript|ENST00000264866.8|protein_coding|15/20|c.1085-3dupT||||||INFO_REALIGN
GH|RP11-617I14.1|ENSG00000251182|transcript|ENST00000515292.1|lincRNA|1/4|n.157-7_157-2de

ENSG00000247193|transcript|ENST00000499292.2|antisense|1/4|n.97-3dupT||||||INFO_REALIGN_3_
ENSG00000247193|transcript|ENST00000499292.2|antisense|1/4|n.97-3dupT||||||INFO_REALIGN_3_
NST00000504686.1|protein_coding|1/1|c.554_555delAT|p.His185fs|554/691|554/576|185/191||,C|i
000065882|transcript|ENST00000511238.5|retained_intron|3/3|n.516-3dupT||||||INFO_REALIGN_3_
nteraction|2J67:A_726-A_775:ENST00000361424|protein_coding|2/2|c.2323A>G||||||,C|structural_i
nteraction|2J67:A_691-A_724:ENST00000361424|protein_coding|2/2|c.2172T>C||||||,G|structural_i
teraction|4OM7:A_644-A_672:ENST00000436693|protein_coding|2/2|c.1932T>G||||||,C|structural_i
nteraction|3ITK:F_224-F_276:ENST00000316423|protein_coding|7/12|c.828T>C||||||,G|structural_ir
nteraction|3PTZ:F_133-F_221:ENST00000316423|protein_coding|4/12|c.399A>G||||||,C|structural_i
000109133|transcript|ENST00000506794.1|nonsense_mediated_decay|2/5|c.141-1G>C||||||,C|intro
nt&intron_variant|HIGH|SLC30A9|ENSG00000014824|transcript|ENST00000513699.5|nonsense_med
IST00000281543.5|protein_coding|12/17|c.1402_1403delGA|p.Glu468fs|1596/4449|1402/2010|468/
000248987|transcript|ENST00000507189.2|processed_pseudogene|1/1|n.515-1T>G||||||
ENST00000226432.8|protein_coding|12/16|c.1596delA|p.Leu533fs|1779/2472|1596/2100|532/699|
_coding_transcript_exon_variant|HIGH|RP11-1281K21.1|ENSG00000237961|transcript|ENST0000050
GH|MTND3P22|ENSG00000248946|transcript|ENST00000510932.2|processed_pseudogene|1/1|n.94
GH|MTND3P22|ENSG00000248946|transcript|ENST00000510932.2|processed_pseudogene|1/1|n.94
00000260120|transcript|ENST00000564513.1|lincRNA|2/2|n.125+1G>C||||||
NST00000337488.10|protein_coding|16/18|c.1459_1460delAG|p.Arg487fs|1653/2198|1459/1785|48
raction|4K9E:C_435-C_461:ENST00000288135|protein_coding|9/21|c.1383A>G||||||,G|structural_in
raction|1PKG:A_678-A_798:ENST00000288135|protein_coding|17/21|c.2394C>T||||||,T|structural_ir
eraction|2X1W:L_239-L_297:ENST00000263923|protein_coding|7/30|c.889G>A||||||,T|structural_int
nteraction|2H31:A_153-A_201:ENST00000264221|protein_coding|6/10|c.603A>C||||||,C|structural_i
on_coding_transcript_exon_variant|HIGH|EFTUD1P2|ENSG00000248998|transcript|ENST0000051481
interaction|4ET7:A_59-A_231:ENST00000273854|protein_coding|1/18|c.177C>T||||||,A|structural_ir
T00000508048.5|protein_coding|1/10|c.3G>A|p.Met1?|36/3247|3/1257|1/418||,T|start_lost|HIGH|
171234|transcript|ENST00000502942.5|protein_coding|1/5|c.-159+1G>T||||||WARNING_TRANSCRIP
00000503640.5|protein_coding|1/6|c.576T>A|p.Tyr192*|632/2595|576/1584|192/527||,T|stop_gain
0171195|transcript|ENST00000304887.5|protein_coding|1/2|c.-15-1G>A||||||,A|splice_acceptor_var
ENST00000304887.5|protein_coding|3/3|c.246dupC|p.Lys83fs|437/2443|247/1134|83/377||INFO_R
eraction|2NO6:B_22-B_122:ENST00000286648|protein_coding|3/7|c.364C>T||||||,T|structural_inter
4199.5|protein_coding|1/14|c.3G>T|p.Met1?|98/1777|3/1482|1/493||
0502620.1|protein_coding|4/5|c.295T>C|p.Ter99Glnext*?|380/582|295/297|99/98||,C|downstream_
0138769|transcript|ENST00000429927.6|protein_coding|9/11|c.1323-1G>A||||||,T|splice_acceptor_
0156194|transcript|ENST00000511880.7|nonsense_mediated_decay|15/17|c.*1995-1A>G||||||,C|spl
6194|transcript|ENST00000286719.11|protein_coding|8/16|c.746+2T>C||||||,G|splice_donor_varian
38750|transcript|ENST00000513352.1|nonsense_mediated_decay|3/4|c.*121+1G>T||||||,A|splice_c
pt|ENST00000515506.5|protein_coding|1/12|c.12_13dupGT|p.Ser5fs|14/4024|13/1407|5/468||WAI
pt|ENST00000515506.5|protein_coding|1/12|c.12_13dupGT|p.Ser5fs|14/4024|13/1407|5/468||WAI
pt|ENST00000515506.5|protein_coding|1/12|c.12_13dupGT|p.Ser5fs|14/4024|13/1407|5/468||WAI
pt|ENST00000515506.5|protein_coding|1/12|c.12_13dupGT|p.Ser5fs|14/4024|13/1407|5/468||WAI
pt|ENST00000515506.5|protein_coding|1/12|c.12_13dupGT|p.Ser5fs|14/4024|13/1407|5/468||WAI
pt|ENST00000515506.5|protein_coding|1/12|c.12_13dupGT|p.Ser5fs|14/4024|13/1407|5/468||WAI
0508909.5|nonsense_mediated_decay|3/4|c.118T>C|p.Ter40Argext*?|524/2297|118/120|40/39||,C|
00000248408|transcript|ENST00000506460.1|lincRNA|3/3|n.316+1G>A||||||
0000138670|transcript|ENST00000508294.1|processed_transcript|3/4|n.347-2A>G||||||,C|intron_va
000311469.8|protein_coding|1/7|c.64A>T|p.Arg22*|64/1641|64/1266|22/421||,A|upstream_gene_v

ipt|ENST00000503217.2|protein_coding|1/5|c.134dupC|p.Ala46fs|162/595|134/567|45/188||WARN
.63633|transcript|ENST00000506308.5|protein_coding|1/3|c.-74+2T>A|||||WARNING_TRANSCRIPT_
000170509|transcript|ENST00000328546.4|protein_coding|6/6|c.812+2dupT||||||,TA|splice_donor_
T00000328546.4|protein_coding|5/7|c.602C>G|p.Ser201*|667/2397|602/903|201/300||,C|stop_gai
ST00000282478.7|protein_coding|4/4|c.3262delA|p.Ser1088fs|3295/4281|3262/3906|1088/1301||,
IST00000282478.7|protein_coding|4/4|c.3264_3274delTGACAGCAGCA|p.Ser1088fs|3297/4281|3264/
0138641|transcript|ENST00000597259.1|processed_transcript|1/1|n.78-1G>T||||||,T|upstream_gen
00138640|transcript|ENST00000509094.5|protein_coding|3/4|c.428-2A>G||||||,C|splice_acceptor_
0145335|transcript|ENST00000506691.1|protein_coding|1/4|c.-72-2dupA|||||WARNING_TRANSCRI
00138722|transcript|ENST00000394980.5|protein_coding|1/8|c.-248-2A>G||||||,G|upstream_gene_
00184305|transcript|ENST00000504150.1|processed_transcript|2/3|n.196-2A>G||||||,G|splice_acce
interaction|1IYH:A_12-A_199:ENST00000295256|protein_coding|6/6|c.597C>G||||||,C|structural_int
|interaction|2UZC:A_22-A_60:ENST00000318007|protein_coding|2/10|c.65A>G||||||,G|structural_ir
00000253170|transcript|ENST00000517407.1|lincRNA|1/1|n.65+2T>G|||||
.247|transcript|ENST00000505194.5|processed_transcript|1/4|n.112+2C>G||||||,C|downstream_ge
164024|transcript|ENST00000503247.5|nonsense_mediated_decay|2/7|c.*69+1G>A|||||WARNING_
nteraction|3COS:A_351-A_375:ENST00000265512|protein_coding|8/9|c.1051C>T||||||,A|structural_i
nteraction|3COS:A_45-A_70:ENST00000265512|protein_coding|3/9|c.135T>C||||||,G|structural_inte
000508393.5|protein_coding|2/10|c.66T>A|p.Tyr22*|232/2151|66/1200|22/399||,T|stop_gained|HI
NSG00000246090|transcript|ENST00000506160.1|antisense|1/4|n.122-1G>C||||||,C|intron_variant|
NSG00000246090|transcript|ENST00000506454.1|antisense|4/5|n.609-1G>A||||||,A|intron_variant|
87758|transcript|ENST00000503461.5|retained_intron|4/5|n.479+1A>C||||||,G|sequence_feature|L
0000515683.5|protein_coding|3/9|c.232G>T|p.Gly78*|584/1740|232/1128|78/375||,A|stop_gained|
NSG00000138813|transcript|ENST00000477187.1|nonsense_mediated_decay|6/10|c.547-1_547insTC
0511045.5|protein_coding|1/18|c.3G>A|p.Met1?|16/3154|3/2766|1/921||,A|start_lost|HIGH|MTTP|
ENST00000273990.6|protein_coding|3/3|c.540delA|p.Lys180fs|755/2604|540/582|180/193||,G|upst
G00000249710|transcript|ENST00000515782.1|antisense|1/4|n.43-2A>T||||||,T|upstream_gene_va
ENST00000322953.8|protein_coding|13/17|c.2204delA|p.Asn735fs|2478/3372|2204/2358|735/785||
9320|transcript|ENST00000505458.5|protein_coding|1/23|c.-8+2G>T||||||,T|upstream_gene_varia
0511926.5|protein_coding|2/6|c.1A>G|p.Met1?|388/609|1/222|1/73||WARNING_TRANSCRIPT_NO_
nteraction|1NFI:B_317-B_350:ENST00000226574|protein_coding|11/24|c.1050C>T||||||,T|structura
00000109332|transcript|ENST00000618836.4|protein_coding|1/7|c.26dupA|p.Glu10fs|26/3472|26/4
00000109332|transcript|ENST00000618836.4|protein_coding|1/7|c.26dupA|p.Glu10fs|26/3472|26/4
00000109332|transcript|ENST00000618836.4|protein_coding|1/7|c.26dupA|p.Glu10fs|26/3472|26/4
nteraction|1T5C:A_226-A_338:ENST00000380026|protein_coding|12/47|c.1014A>G||||||,C|structur
nteraction|1T5C:A_81-A_295:ENST00000380026|protein_coding|4/47|c.243T>C||||||,G|structural_i
500000250920|transcript|ENST00000509399.1|lincRNA|3/3|n.439+2C>T||||||,A|intron_variant|MOD
00000248242|transcript|ENST00000515127.1|lincRNA|1/5|n.186+2T>C||||||,G|intron_variant|MOD
503516.1|protein_coding|5/7|c.3G>A|p.Met1?|435/601|3/169|1/55||WARNING_TRANSCRIPT_INCON
interaction|1X6V:A_420-A_454:ENST00000265174|protein_coding|10/12|c.1260A>G||||||,C|structu
138796|transcript|ENST00000511742.1|nonsense_mediated_decay|2/2|c.*228-1G>C||||||,C|intron_
00000247950|transcript|ENST00000499713.6|antisense|7/7|n.1277-1G>A||||||,T|upstream_gene_v
IST00000265171.9|protein_coding|24/24|c.3406dupC|p.Gln1136fs|3852/4880|3407/3624|1136/120
000265162.9|protein_coding|6/20|c.1238G>A|p.Trp413*|1580/6943|1238/2874|413/957||,A|seque
000557119.2|protein_coding|2/2|c.577C>T|p.Gln193*|1168/1889|577/795|193/264||,A|upstream_g
NST00000512589.5|nonsense_mediated_decay|3/5|c.286_290delATCAG|p.Ile96fs|421/556|286/306|
0000154608|transcript|ENST00000502249.6|transcribed_unprocessed_pseudogene|7/16|n.1372-1C>(

)000245958|transcript|ENST00000498873.5|processed_transcript|8/9|n.779+1G>A||||||,A|upstream
nteraction|3LFV:B_181-B_287:ENST00000354960|protein_coding|2/21|c.541T>G||||||,C|missense_v
)|interaction|2QYF:A_107-A_190:ENST00000296509|protein_coding|5/5|c.568A>G||||||,C|structural
)|interaction|2QYF:A_35-A_143:ENST00000296509|protein_coding|4/5|c.429A>G||||||,C|structural_
on_coding_transcript_exon_variant|HIGH|SETP12|ENSG00000251609|transcript|ENST00000514068.2
on_coding_transcript_exon_variant|HIGH|SETP12|ENSG00000251609|transcript|ENST00000514068.2
on_coding_transcript_exon_variant|HIGH|SETP12|ENSG00000251609|transcript|ENST00000514068.2
on_coding_transcript_exon_variant|HIGH|SETP12|ENSG00000251609|transcript|ENST00000514068.2
ction|1GY3:C_157-D_228:ENST00000266970-ENST00000274026|protein_coding|4/8|c.684G>A||||||
00000138688|transcript|ENST00000388738.7|protein_coding|6/83|c.628-3dupT||||||INFO_REALIGN
00000138688|transcript|ENST00000388738.7|protein_coding|6/83|c.628-3dupT||||||INFO_REALIGN
)142731|transcript|ENST00000270861.9|protein_coding|7/15|c.1831-3dupT||||||INFO_REALIGN_3_F
)142731|transcript|ENST00000270861.9|protein_coding|10/15|c.2189-3dupT||||||INFO_REALIGN_3_
|ENST00000520121.5|protein_coding|1/3|c.15_16delCC|p.Arg6fs|983/3785|15/744|5/247||,C|frame
NSG00000249618|transcript|ENST00000513875.5|lincRNA|15/16|n.1462-3dupT||||||INFO_REALIGN_
G00000251598|transcript|ENST00000420721.2|lincRNA|2/2|n.318-1G>A||||||
)0000248434|transcript|ENST00000514526.1|lincRNA|2/3|n.406+1G>A||||||,A|splice_donor_variant
)0248307|transcript|ENST00000503208.1|lincRNA|3/3|n.433+1G>A||||||,T|intron_variant|MODIFIEI
t&intron_variant|HIGH|MAML3|ENSG00000196782|transcript|ENST00000398940.1|protein_coding|2
t&intron_variant|HIGH|MAML3|ENSG00000196782|transcript|ENST00000398940.1|protein_coding|2
ENST00000509479.6|protein_coding|2/5|c.1515delG|p.Gln505fs|2372/6844|1515/3417|505/1138||
ENST00000509479.6|protein_coding|2/5|c.1494_1513delGCAGCAGCAGCAGCAGCAGC|p.Gln499fs|237
ENST00000509479.6|protein_coding|2/5|c.1506delG|p.Gln502fs|2363/6844|1506/3417|502/1138||
ENST00000509479.6|protein_coding|2/5|c.1506delG|p.Gln502fs|2363/6844|1506/3417|502/1138||
ipt|ENST00000509479.6|protein_coding|2/5|c.1494_1504delGCAGCAGCAGC|p.Gln499fs|2361/6844|
ipt|ENST00000509479.6|protein_coding|2/5|c.1494_1504delGCAGCAGCAGC|p.Gln499fs|2361/6844|
G00000196782|transcript|ENST00000398940.1|protein_coding|1/4|c.108+2_108+3insACAGCA||||||,
)179387|transcript|ENST00000507667.1|protein_coding|1/1|c.-10+1G>A||||||WARNING_TRANSCRIPT
NSG00000250345|transcript|ENST00000506341.2|processed_pseudogene|1/1|n.565-1C>G||||||,C|ir
_coding_transcript_exon_variant|HIGH|RP11-673E1.1|ENSG00000251600|transcript|ENST00000505000
250361|transcript|ENST00000429670.2|protein_coding|3/3|c.176-1G>C||||||,G|missense_variant|M
NST00000506679.5|nonsense_mediated_decay|2/6|c.41dupA|p.Asp14fs|93/606|41/129|14/42||,AT
2|interaction|1JHJ:A_64-A_139:ENST00000309439|protein_coding|5/5|c.417T>G||||||,C|structural_
)00164163|transcript|ENST00000296577.8|protein_coding|5/17|c.406-3dupT||||||INFO_REALIGN_3_
)00164163|transcript|ENST00000296577.8|protein_coding|5/17|c.406-3dupT||||||INFO_REALIGN_3_
)0000248210|transcript|ENST00000503100.1|lincRNA|10/12|n.2009+2T>C||||||
script|ENST00000511993.5|nonsense_mediated_decay|6/17|c.307_308delCC|p.Pro103fs|686/2138|3
)0000234828|transcript|ENST00000508106.5|processed_transcript|3/6|n.232+1G>A||||||,T|intron_v
script|ENST00000455694.6|protein_coding|2/5|c.127_131dupGTCAG|p.Ser44fs|134/989|132/987|44
)9|transcript|ENST00000455694.6|protein_coding|5/5|c.985T>G|p.Ter329Gluext*?|987/989|985/987
NST00000509752.5|nonsense_mediated_decay|5/10|c.295delC|p.His99fs|352/844|295/381|99/126|
NST00000339452.2|protein_coding|8/8|c.4095_4098delCAAA|p.Asn1365fs|4459/5064|4095/4110|13
nteraction|4YVD:A_297-A_344:ENST00000499023|protein_coding|11/15|c.1032A>G||||||,C|synonyr
)00250910|transcript|ENST00000512269.5|antisense|1/2|n.140+1G>T||||||,T|splice_donor_variant8
)00250910|transcript|ENST00000593486.5|antisense|2/4|n.187+1G>A||||||,A|upstream_gene_varia
)0061918|transcript|ENST00000502959.5|protein_coding|5/14|c.363+1G>A||||||,A|intron_variant|M
G00000251511|transcript|ENST00000510753.1|lincRNA|1/1|n.186-1G>C||||||

)0120251|transcript|ENST00000323661.10|nonsense_mediated_decay|13/16|c.2151-3dupT||||||INF
G00000248429|transcript|ENST00000503611.5|antisense|1/5|n.729+1A>G||||||,G|splice_donor_vari
)000164124|transcript|ENST00000508243.5|protein_coding|1/7|c.-176-1G>A||||||WARNING_TRANS
.83439|transcript|ENST00000329314.5|protein_coding|1/4|c.-476+1G>A||||||
:|ENST00000505354.2|protein_coding|1/1|c.1144G>T|p.Gly382*|1178/1868|1144/1662|382/553||,
)0181381|transcript|ENST00000505890.5|protein_coding|26/29|c.3492-1G>A||||||,T|splice_region_
NST00000306193.7|protein_coding|4/5|c.514dupA|p.Arg172fs|683/3443|514/714|172/237||,CT|fra
teraction|4APC:A_107-A_261:ENST00000507142|protein_coding|10/36|c.782G>A||||||,T|structural_
)0109572|transcript|ENST00000504131.6|protein_coding|2/13|c.19-3dupT||||||INFO_REALIGN_3_PI
ENST00000280191.6|protein_coding|6/6|c.836_839delGAGA|p.Arg279fs|948/1169|836/918|279/305
)0109674|transcript|ENST00000264596.3|protein_coding|5/9|c.703-3dupT||||||INFO_REALIGN_3_P
)0109674|transcript|ENST00000264596.3|protein_coding|5/9|c.703-3dupT||||||INFO_REALIGN_3_P
nteraction|2W4L:A_105-A_126:ENST00000438320|protein_coding|4/6|c.315T>C||||||,G|structural_ir
i00000230219|transcript|ENST00000504766.1|transcribed_processed_pseudogene|1/1|n.437-1C>G||
ENSG00000250754|transcript|ENST00000511746.1|lincRNA|2/4|n.448-3dupT||||||INFO_REALIGN_3
ENSG00000250754|transcript|ENST00000511746.1|lincRNA|2/4|n.448-3dupT||||||INFO_REALIGN_3
ENST00000378850.4|protein_coding|6/7|c.775delG|p.Val259fs|797/1134|775/930|259/309||,T|3_p
:eraction|1ZIW:A_412-A_437:ENST00000296795|protein_coding|4/5|c.1234C>T||||||,T|missense_va
teraction|1ZIW:A_434-A_459:ENST00000296795|protein_coding|4/5|c.1377C>T||||||,T|structural_in
)00264690.10|protein_coding|5/15|c.406G>T|p.Glu136*|593/2363|406/1917|136/638||,T|stop_gair
eraction|3SOR:A_507-A_613:ENST00000403665|protein_coding|15/15|c.1839G>A||||||,A|structural_
79046|transcript|ENST00000502707.1|processed_transcript|1/1|n.333+1G>A||||||,T|upstream_gen
)0000247130|transcript|ENST00000501322.2|antisense|1/1|n.452+1G>T||||||,A|upstream_gene_va
G00000248370|transcript|ENST00000502419.1|lincRNA|1/1|n.39-1G>A||||||
:NST00000509632.5|nonsense_mediated_decay|2/5|c.144dupG|p.Leu49fs|174/583|145/270|49/89|
IST00000264932.10|protein_coding|15/15|c.1945_1946delTT|p.Leu649fs|2060/2390|1945/1995|649
interaction|2ZN8:A_110-A_146:ENST00000264933|protein_coding|5/6|c.438T>C||||||,C|structural_i
30104|transcript|ENST00000515601.6|nonsense_mediated_decay|9/11|c.*3135+2T>C||||||,C|splice_
NST00000264935.5|protein_coding|6/12|c.849_853delGGAGC|p.Glu284fs|939/2434|849/1944|283/6
cript|ENST00000508859.6|protein_coding|8/11|c.1007_1008insTT|p.Cys337fs|1007/1631|1007/1149
pt|ENST00000508859.6|protein_coding|8/11|c.1002_1003delAT|p.Cys335fs|1003/1631|1002/1149|3
)0000382776.5|protein_coding|1/10|c.2T>G|p.Met1?|2/1116|2/1116|1/371||,C|missense_variant|M
T00000424784.3|protein_coding|11/11|c.569G>A|p.Trp190*|718/943|569/642|190/213||,T|3_prime
t|ENST00000424784.3|protein_coding|10/11|c.546_561delCGGAAATGGGAGCATAGTACT|p.Gly188fs|7
)0000188818|transcript|ENST00000424784.3|protein_coding|8/11|c.474_475delCA|p.His158fs|624/9
pt|ENST00000511539.1|protein_coding|2/2|c.394dupA|p.Thr132fs|499/1303|394/429|132/142||,GT
)00000515652.5|nonsense_mediated_decay|7/11|c.956G>A|p.Trp319*|1012/3337|956/1065|319/354
)00000324642.3|protein_coding|7/12|c.957C>G|p.Tyr319*|1080/2121|957/1887|319/628||
iH|MRPL36|ENSG00000171421|transcript|ENST00000505059.6|protein_coding|1/1|c.-28_-13+2dupG
)00469176.1|protein_coding|3/3|c.517T>C|p.Ter173Argext*?|520/550|517/519|173/172||,C|intron_
)0000397835.4|protein_coding|3/3|c.676C>T|p.Gln226*|676/681|676/681|226/226||,T|3_prime_UT
G00000205976|transcript|ENST00000512854.1|unprocessed_pseudogene|6/7|n.638-2A>G||||||
)00124275|transcript|ENST00000511639.1|processed_transcript|1/2|n.387-3dupT||||||INFO_REALIG
)00124275|transcript|ENST00000511639.1|processed_transcript|1/2|n.387-3dupT||||||INFO_REALIG
nteraction|2QTL:A_560-A_595:ENST00000440940|protein_coding|14/15|c.1783C>T||||||,T|structura
i00000248525|transcript|ENST00000504182.2|sense_overlapping|1/2|n.35+1G>A||||||,T|intron_var
)0000274134.4|protein_coding|2/5|c.135T>A|p.Tyr45*|427/1066|135/693|45/230||,A|stop_gained|

_coding_transcript_exon_variant|HIGH|RP11-1C1.5|ENSG00000249160|transcript|ENST00000504650.
interaction|3AU5:B_1740-B_1790:ENST00000513610|protein_coding|38/41|c.5370C>T||||||,A|syno
00248874|transcript|ENST00000512559.5|lincRNA|4/4|n.1558-2A>G|||||
teraction|4P3N:A_551-A_607:ENST00000265112|protein_coding|15/19|c.1653G>A||||||,A|structura
ST00000515401.1|protein_coding|3/3|c.689T>A|p.Leu230*|852/1003|689/699|230/232||,T|intron_
00506639.5|nonsense_mediated_decay|4/5|c.688T>A|p.Ter230Lysext*?|700/2182|688/690|230/229
42110|transcript|ENST00000514195.1|processed_transcript|4/4|n.633+1A>G||||||,C|missense_vari
0&ENSG00000273294|gene_variant|ENSG00000242110|||n.34008121_34008102delCGGCTGGGGCG
00039560|transcript|ENST00000515799.5|protein_coding|1/19|c.-270-3dupT||||||INFO_REALIGN_3_
00039560|transcript|ENST00000515799.5|protein_coding|1/19|c.-270-3dupT||||||INFO_REALIGN_3_
000113460|transcript|ENST00000336767.5|protein_coding|9/9|c.793-3dupT||||||INFO_REALIGN_3_
000113460|transcript|ENST00000336767.5|protein_coding|9/9|c.793-3dupT||||||INFO_REALIGN_3_
000113460|transcript|ENST00000336767.5|protein_coding|9/9|c.793-3dupT||||||INFO_REALIGN_3_
0113460|transcript|ENST00000336767.5|protein_coding|9/9|c.793-2dupA||||||INFO_REALIGN_3_PF
0113460|transcript|ENST00000336767.5|protein_coding|9/9|c.793-2dupA||||||INFO_REALIGN_3_PF
13492|transcript|ENST00000510428.1|protein_coding|12/12|c.*45+1G>T||||||,A|3_prime_UTR_vari
.3492|transcript|ENST00000231420.10|protein_coding|11/13|c.1188+1G>A||||||,T|splice_donor_va
0000274441&ENSG00000079215|gene_variant|ENSG00000274441|||n.36649361_36649327delTTTTT
00082068|transcript|ENST00000504564.1|protein_coding|10/11|c.1093-2A>G||||||,G|intron_variant
G00000250585|transcript|ENST00000637924.1|lincRNA|1/2|n.265-3dupT||||||INFO_REALIGN_3_PRI
00250585|transcript|ENST00000637924.1|lincRNA|1/2|n.265-3delT||||||INFO_REALIGN_3_PRIME
NST00000637375.1|protein_coding|3/3|c.357delT|p.Phe119fs|483/1824|357/399|119/132||,T|intror
000637375.1|protein_coding|3/3|c.327G>A|p.Trp109*|453/1824|327/399|109/132||,T|intron_variar
NST00000254691.9|protein_coding|1/3|c.88_89delTT|p.Leu30fs|287/4221|88/3114|30/1037||,C|fra
00000399564.4|protein_coding|6/42|c.572G>A|p.Trp191*|1023/5239|572/4758|191/1585||,T|upst
7|transcript|ENST00000263413.7|protein_coding|16/17|c.2381+2T>C||||||,G|splice_donor_variant8
0000205765|transcript|ENST00000381647.6|protein_coding|2/5|c.208-3dupT||||||INFO_REALIGN_3_
0000205765|transcript|ENST00000381647.6|protein_coding|2/5|c.208-3dupT||||||INFO_REALIGN_3_
0000248873|transcript|ENST00000507707.1|processed_pseudogene|1/1|n.320-2C>A||||||,T|downst
00000172262|transcript|ENST00000511736.5|nonsense_mediated_decay|5/6|c.*176-4_*176-3dupTT|
00000172262|transcript|ENST00000511736.5|nonsense_mediated_decay|5/6|c.*176-4_*176-3dupTT|
2|interaction|2P8U:A_195-A_383:ENST00000325110|protein_coding|8/11|c.1149G>A||||||,T|structu
2|interaction|2P8U:A_233-A_282:ENST00000325110|protein_coding|5/11|c.699T>C||||||,G|structur
992|transcript|ENST00000512422.5|protein_coding|1/4|c.-54+1G>T||||||WARNING_TRANSCRIPT_IN
000151883|transcript|ENST00000513738.5|protein_coding|3/4|c.254-3dupT||||||INFO_REALIGN_3_
000151883|transcript|ENST00000513738.5|protein_coding|3/4|c.254-3dupT||||||INFO_REALIGN_3_
interaction|4AP8:A_50-A_125:ENST00000396954|protein_coding|4/7|c.148A>G||||||,C|structural_ir
000039123|transcript|ENST00000503165.5|processed_transcript|5/5|n.520-3dupT||||||INFO_REALIG
000507645.5|nonsense_mediated_decay|2/7|c.147G>A|p.Trp49*|149/554|147/150|49/49||WARNIN
ript|ENST00000624860.1|protein_coding|2/2|c.54_55delAA|p.Lys18fs|55/72|54/72|18/23||WARNIN
cript|ENST00000624860.1|protein_coding|2/2|c.54delA|p.Thr19fs|54/72|54/72|18/23||WARNING_T
nscript|ENST00000624860.1|protein_coding|2/2|c.53dupA|p.Thr19fs|53/72|53/72|18/23||WARNIN
t|ENST00000434982.2|protein_coding|4/4|c.959delA|p.Asn320fs|1195/1518|959/981|320/326||,G|
55545|transcript|ENST00000440000.5|protein_coding|2/2|c.31+1G>T||||||WARNING_TRANSCRIPT_N
teraction|4I5P:A_113-A_155:ENST00000274289|protein_coding|2/14|c.339T>C||||||,G|structural_in
175857|transcript|ENST00000511178.1|processed_transcript|1/1|n.105-1G>T||||||,T|3_prime_UTR_
00178722|transcript|ENST00000507461.1|lincRNA|3/3|n.442-2A>G|||||

GH|CTD-2194F4.2|ENSG00000248288|transcript|ENST00000509786.1|processed_pseudogene|1/1|n.
GH|CTD-2194F4.2|ENSG00000248288|transcript|ENST00000509786.1|processed_pseudogene|1/1|n.
GH|CTD-2194F4.2|ENSG00000248288|transcript|ENST00000509786.1|processed_pseudogene|1/1|n.
GH|CTD-2194F4.2|ENSG00000248288|transcript|ENST00000509786.1|processed_pseudogene|1/1|n.
113597&ENSG00000113595|gene_variant|ENSG00000113595|||n.65624719_65624709delAAAAAAA
113597&ENSG00000113595|gene_variant|ENSG00000113595|||n.65624719_65624709delAAAAAAA
113597&ENSG00000113595|gene_variant|ENSG00000113595|||n.65624719_65624709delAAAAAAA
113597&ENSG00000113595|gene_variant|ENSG00000113595|||n.65624719_65624709delAAAAAAA
113597&ENSG00000113595|gene_variant|ENSG00000113595|||n.65624719_65624709delAAAAAAA
113597&ENSG00000113595|gene_variant|ENSG00000113595|||n.65624719_65624709delAAAAAAA
nteraction|3B2D:B_201-B_223:ENST00000256447|protein_coding|3/3|c.667A>G||||||,C|missense_v
interaction|1PHT:A_11-A_73:ENST00000521381|protein_coding|2/16|c.219C>T||||||,T|structural_in
00172058|transcript|ENST00000504458.1|protein_coding|2/2|c.117-3dupC||||||INFO_REALIGN_3_
interaction|1QBK:B_323-B_491:ENST00000506351|protein_coding|13/25|c.1473C>T||||||,T|structur
T00000601380.2|protein_coding|12/14|c.1388G>A|p.Trp463*|1564/2569|1388/1587|463/528||,A|c
T00000601380.2|protein_coding|13/14|c.1439G>A|p.Trp480*|1615/2569|1439/1587|480/528||,A|c
cript|ENST00000631802.1|protein_coding|1/2|c.201dupG|p.Arg68fs|349/1917|201/555|67/184||,TC
cript|ENST00000631802.1|protein_coding|1/2|c.195_196insT|p.Ala66fs|343/1917|195/555|65/184|
nscript|ENST00000631802.1|protein_coding|1/2|c.192_193dupTA|p.Thr65fs|341/1917|193/555|65/1
teraction|4JRA:C_522-C_543:ENST00000502798|protein_coding|10/13|c.1627G>A||||||,A|structural
0000509971.5|protein_coding|1/6|c.18T>A|p.Cys6*|109/543|18/452|6/149||WARNING_TRANSCRIPT
|ENST00000509971.5|protein_coding|1/6|c.10dupG|p.Ala4fs|101/543|10/452|4/149||WARNING_TF
teraction|1FSU:A_356-A_376:ENST00000264914|protein_coding|5/8|c.1126G>A||||||,T|missense_va
nteraction|1LT7:A_246-A_284:ENST00000274353|protein_coding|7/8|c.852C>T||||||,T|structural_int
00000250258|transcript|ENST00000421252.2|antisense|4/4|n.359+2T>C||||||,G|intron_variant|MO
3296|transcript|ENST00000515510.1|processed_transcript|2/4|n.215+1G>T||||||,T|upstream_gene_
4.7|ENSG00000184188|transcript|ENST00000507366.1|processed_pseudogene|1/1|n.234-6_234-1du
C-512J14.7|ENSG00000184188|transcript|ENST00000507366.1|processed_pseudogene|1/1|n.234-12_
000113319|transcript|ENST00000503795.1|nonsense_mediated_decay|24/27|c.3435-1G>A||||||,A|i
|interaction|3B7K:B_59-B_230:ENST00000307624|protein_coding|7/15|c.688G>A||||||,T|structural_
0186468|transcript|ENST00000510210.5|protein_coding|4/5|c.364-1G>T||||||,A|splice_acceptor_var
00248400|transcript|ENST00000508958.2|processed_pseudogene|1/1|n.224-1C>T||||||
0152422|transcript|ENST00000511817.1|protein_coding|7/7|c.894-1G>A||||||,A|splice_acceptor_va
on_coding_transcript_exon_variant|HIGH|RP11-1072N2.2|ENSG00000250253|transcript|ENST000005
G00000250544|transcript|ENST00000503843.1|lincRNA|1/2|n.138-1G>A||||||,A|non_coding_transc
ENSG00000247828|transcript|ENST00000501715.6|antisense|1/7|n.97-3dupT||||||INFO_REALIGN_3
000164199|transcript|ENST00000405460.6|protein_coding|55/89|c.11581-3dupC||||||INFO_REALIG
0000248528|transcript|ENST00000507491.1|lincRNA|3/3|n.418+1G>C||||||
t|ENST00000606183.3|protein_coding|1/1|c.6dupC|p.Gly3fs|80/8418|6/987|2/328||,CG|upstream_
00000283357.9|protein_coding|4/10|c.430C>T|p.Gln144*|476/1560|430/1359|144/452||,T|stop_ga
000164292|transcript|ENST00000514198.1|protein_coding|1/1|c.38-1G>T||||||WARNING_TRANSCR
nteraction|3QNF:A_342-A_725:ENST00000296754|protein_coding|15/20|c.2174G>A||||||,T|structur
nteraction|2YD0:A_414-A_528:ENST00000296754|protein_coding|11/20|c.1583A>G||||||,C|structur
nteraction|4JBS:A_97-A_164:ENST00000437043|protein_coding|2/19|c.291C>T||||||,T|structural_int
nteraction|4JBS:A_214-A_260:ENST00000437043|protein_coding|3/19|c.641C>T||||||,T|structural_in
nteraction|4JBS:A_200-A_392:ENST00000437043|protein_coding|7/19|c.1176G>T||||||,T|structural_
NSG00000251513|transcript|ENST00000504578.2|lincRNA|1/6|n.131-1G>A||||||,A|intron_variant|M

IGH|LINC01340|ENSG00000250331|transcript|ENST00000504012.1|lincRNA|1/3|n.98-35_98-2delTTA
00000275374|transcript|ENST00000621479.1|unprocessed_pseudogene|4/4|n.662+1G>T|||||
2&ENSG00000250958|gene_variant|ENSG00000247402|||n.102606192_102606202delAAAAAAAAAAA
2&ENSG00000250958|gene_variant|ENSG00000247402|||n.102606192_102606202delAAAAAAAAAAA
2&ENSG00000250958|gene_variant|ENSG00000247402|||n.102606192_102606202delAAAAAAAAAAA
2&ENSG00000250958|gene_variant|ENSG00000247402|||n.102606192_102606202delAAAAAAAAAAA
961|transcript|ENST00000511624.1|processed_transcript|1/3|n.173+1G>T|||||,A|intron_variant|M
ISG00000248867|transcript|ENST00000511296.1|processed_pseudogene|1/1|n.72-2dupA|||||
NSG00000248867|transcript|ENST00000511296.1|processed_pseudogene|1/1|n.72-3_72-2dupAA|||
ENSG00000248867|transcript|ENST00000511296.1|processed_pseudogene|1/1|n.72-4_72-2dupAAA|
000129595|transcript|ENST00000507810.5|processed_transcript|12/13|n.995-1G>A|||||
:NSG00000129595|transcript|ENST00000261486.5|protein_coding|22/22|c.1933-6_1933-2dupTTTTA|
eraction|3AU3:A_439-A_486:ENST00000508376|protein_coding|13/17|c.1458T>C|||||,C|structural_
eraction|3AU3:A_653-A_687:ENST00000508376|protein_coding|17/17|c.1959G>A|||||,A|structural
on|1T08:A_387-C_1493:ENST00000349496-ENST00000508376|protein_coding|17/17|c.4479G>A||||
SG00000272869|transcript|ENST00000506997.1|nonsense_mediated_decay|2/3|c.118-1G>C|||||,C
nscript|ENST00000514701.5|protein_coding|14/14|c.2139_*1delTGCCCAGTGAC|p.Thr713fs|2702/28
45782|transcript|ENST00000379594.7|nonsense_mediated_decay|2/4|c.*51+2T>A|||||,T|splice_do
NST00000623778.1|protein_coding|2/3|c.53G>A|p.Trp18*|53/63|53/63|18/20||WARNING_TRANSCR
000172901|transcript|ENST00000357872.8|protein_coding|17/20|c.2567_2568insATATATATATATATG
0000145779|transcript|ENST00000274456.6|protein_coding|1/1|c.2-3dupT|||||INFO_REALIGN_3_P
0000145779|transcript|ENST00000274456.6|protein_coding|1/1|c.2-3dupT|||||INFO_REALIGN_3_P
5|interaction|1ZBQ:A_106-A_160:ENST00000256216|protein_coding|6/24|c.317G>A|||||,A|structu
ENST00000296600.4|protein_coding|2/2|c.953delT|p.Leu318fs|1336/1964|953/1095|318/364||INFC
eraction|2ESL:B_47-B_190:ENST00000306442|protein_coding|5/5|c.569A>G|||||,C|structural_inter
eraction|2ESL:B_86-B_190:ENST00000306442|protein_coding|3/5|c.257A>G|||||,C|structural_inter
T00000306442.4|protein_coding|2/5|c.148delG|p.Asp50fs|264/1380|148/639|50/212||,T|downstre
0223652&ENSG00000061455|gene_variant|ENSG00000223652|||n.123089148_123089117delTGTGT
0223652&ENSG00000061455|gene_variant|ENSG00000223652|||n.123089148_123089117delTGTGT
00000248600|transcript|ENST00000502809.1|lincRNA|1/2|n.86+2T>C|||||,C|downstream_gene_v
G00000250602|transcript|ENST00000515808.5|antisense|2/2|n.266-1G>A|||||,T|intron_variant|M
pt|ENST00000635851.1|protein_coding|18/18|c.1613dupT|p.Thr539fs|1614/2135|1613/1635|538/5
NSG00000248634|transcript|ENST00000514867.1|sense_intronic|1/1|n.70-3dupT|||||INFO_REALIG
NSG00000248634|transcript|ENST00000514867.1|sense_intronic|1/1|n.70-3dupT|||||INFO_REALIG
NSG00000248634|transcript|ENST00000514867.1|sense_intronic|1/1|n.70-3dupT|||||INFO_REALIG
NSG00000248634|transcript|ENST00000514867.1|sense_intronic|1/1|n.70-3dupT|||||INFO_REALIG
9567|transcript|ENST00000513345.5|nonsense_mediated_decay|1/2|c.146+2T>G|||||,C|splice_do
0000164398|transcript|ENST00000543479.5|protein_coding|6/21|c.619_620dupCA|p.Gln207fs|620/
000224431|transcript|ENST00000425667.1|processed_pseudogene|1/1|n.3415+1T>C|||||,C|intron_
FSTL4|ENSG00000053108|transcript|ENST00000510685.1|protein_coding|1/4|c.107_108insACAGCAG
NST00000503586.1|protein_coding|1/3|c.102delT|p.Phe34fs|104/624|102/622|34/206||WARNING_T
NSG00000277619|gene_variant|ENSG00000277619|||n.135035890_135035907delCCCCATCCCCATCCC
NSG00000277619|gene_variant|ENSG00000277619|||n.135035890_135035907delCCCCATCCCCATCCC
NSG00000277619|gene_variant|ENSG00000277619|||n.135035890_135035907delCCCCATCCCCATCCC
000249647|transcript|ENST00000505663.1|lincRNA|2/3|n.254+2T>C|||||,G|splice_donor_variant&
000249647|transcript|ENST00000505663.1|lincRNA|2/3|n.254+1G>A|||||,T|splice_donor_variant&
nteraction|1ZR3:B_306-B_345:ENST00000312469|protein_coding|9/9|c.1035G>A|||||,T|structural_

00000503143.3|protein_coding|1/1|c.349A>T|p.Arg117*|589/3135|349/735|117/244||,A|3_prime_U
0000145832|transcript|ENST00000274513.9|protein_coding|4/4|c.422-2A>G|||||,G|splice_acceptor
nteraction|2VXP:A_540-A_572:ENST00000442011|protein_coding|12/17|c.1620T>C|||||,C|structura
00113658|transcript|ENST00000545279.5|protein_coding|1/7|c.-244-3dupT|||||INFO_REALIGN_3_
00113658|transcript|ENST00000545279.5|protein_coding|1/7|c.-244-3dupT|||||INFO_REALIGN_3_
6021|transcript|ENST00000509694.1|retained_intron|1/1|n.622+2T>C|||||,G|sequence_feature|LC
nteraction|4CH9:A_428-A_461:ENST00000309755|protein_coding|12/15|c.1383G>A|||||,T|structura
GH|RPS27AP18|ENSG00000249851|transcript|ENST00000515536.1|processed_pseudogene|1/1|n.26
GH|RPS27AP18|ENSG00000249851|transcript|ENST00000515536.1|processed_pseudogene|1/1|n.26
GH|RPS27AP18|ENSG00000249851|transcript|ENST00000515536.1|processed_pseudogene|1/1|n.26
GH|RPS27AP18|ENSG00000249851|transcript|ENST00000515536.1|processed_pseudogene|1/1|n.26
GH|RPS27AP18|ENSG00000249851|transcript|ENST00000515536.1|processed_pseudogene|1/1|n.26
NST00000523929.1|nonsense_mediated_decay|3/5|c.317delA|p.Asn106fs|319/780|317/489|106/16
NST00000523929.1|nonsense_mediated_decay|3/5|c.317delA|p.Asn106fs|319/780|317/489|106/16
NST00000523929.1|nonsense_mediated_decay|3/5|c.317delA|p.Asn106fs|319/780|317/489|106/16
nteraction|4KBO:A_279-A_316:ENST00000297185|protein_coding|9/17|c.948A>G|||||,C|synonymo
0000504902.5|nonsense_mediated_decay|4/8|c.250C>T|p.Arg84*|251/887|250/300|84/99||WARNIN
on_coding_transcript_exon_variant|HIGH|RPL12P21|ENSG00000242683|transcript|ENST0000047221
on_coding_transcript_exon_variant|HIGH|RPL12P21|ENSG00000242683|transcript|ENST0000047221
on_coding_transcript_exon_variant|HIGH|RPL12P21|ENSG00000242683|transcript|ENST0000047221
00000508270.1|protein_coding|1/4|c.135G>A|p.Trp45*|135/509|135/509|45/168||WARNING_TRAN
ENST00000336283.7|protein_coding|3/5|c.328_329insAC|p.Val110fs|1092/2107|328/711|110/236||
NST00000336283.7|protein_coding|3/5|c.327_328insC|p.Val110fs|1091/2107|327/711|109/236||,C
00000250515|transcript|ENST00000511215.1|processed_pseudogene|1/1|n.162+2G>A|||||
|transcript|ENST00000530339.1|protein_coding|1/4|c.206_207insAAGACACC|p.Gly70fs|207/5251|20
|ENST00000530339.1|protein_coding|1/4|c.213delC|p.Gly72fs|213/5251|213/2844|71/947||,G|fram
|ENST00000530339.1|protein_coding|1/4|c.217_218delGG|p.Gly73fs|217/5251|217/2844|73/947||,
NSG00000279558|transcript|ENST00000624102.1|lincRNA|2/2|n.237-1G>A|||||,A|intron_variant|M
ENST00000231130.3|protein_coding|1/1|c.1737_1749delGGCTGAGCCGGGC|p.Ala580fs|1914/3355|1
pt|ENST00000239444.3|protein_coding|1/1|c.698_699insCC|p.Val234fs|944/2740|699/2406|233/80
|ENST00000239444.3|protein_coding|1/1|c.701_702delTC|p.Val234fs|946/2740|701/2406|234/801|
NSG00000279375|transcript|ENST00000625144.1|antisense|1/1|n.46-2A>G|||||,C|upstream_gene_
0329|transcript|ENST00000239451.6|protein_coding|1/1|c.131dupA|p.Tyr44fs|311/1417|131/906|4
t|ENST00000398604.2|protein_coding|1/4|c.522delC|p.Asn175fs|522/4605|522/2799|174/932||INF
pt|ENST00000612503.1|protein_coding|1/1|c.2477delA|p.Lys826fs|2666/4462|2477/2553|826/850|
ST00000612503.1|protein_coding|1/1|c.2470A>T|p.Lys824*|2659/4462|2470/2553|824/850||,T|ups
GA12|ENSG00000253159|transcript|ENST00000613314.1|protein_coding|1/1|c.2461delT|p.Ter821fs|
0000276545|transcript|ENST00000618012.1|unprocessed_pseudogene|1/1|n.313-1G>A|||||,A|intr
2|interaction|1NE7:A_23-A_132:ENST00000311337|protein_coding|2/7|c.69C>T|||||,A|structural_ir
00000251205|transcript|ENST00000514942.2|lincRNA|1/3|n.218+1G>A|||||
00248842|transcript|ENST00000507391.1|lincRNA|1/1|n.147+2C>T|||||
|interaction|4BKN:A_178-A_238:ENST00000398514|protein_coding|8/14|c.714T>C|||||,G|structura
interaction|4BKN:A_141-A_165:ENST00000398514|protein_coding|4/14|c.421A>G|||||,C|structura
ST00000521530.5|protein_coding|3/6|c.311_312delCA|p.Thr104fs|318/1323|311/1164|104/387||,T
00511307.5|nonsense_mediated_decay|4/12|c.286T>A|p.Ter96Lysext*?|326/3276|286/288|96/95||,
00173210|transcript|ENST00000504238.5|protein_coding|18/18|c.1606-2A>G|||||,G|3_prime_UTR
:|ENST00000412431.2|protein_coding|2/3|c.433delT|p.Tyr145fs|1119/3088|433/501|145/166||,TAA

|ENST00000412431.2|protein_coding|2/3|c.433delT|p.Tyr145fs|1119/3088|433/501|145/166||,TAA
NST00000296736.3|protein_coding|2/2|c.1032delA|p.Glu345fs|1807/4006|1032/1566|344/521||,C|
nteraction|3DPK:A_766-A_903:ENST00000286301|protein_coding|21/22|c.2709C>T||||||,A|structura
nteraction|2I0V:A_759-A_845:ENST00000286301|protein_coding|19/22|c.2535C>G||||||,C|structura
nteraction|4LIQ:E_325-E_362:ENST00000286301|protein_coding|8/22|c.1085A>G||||||,C|missense_
nteraction|4DKD:C_98-C_159:ENST00000286301|protein_coding|3/22|c.294C>T||||||,A|structural_ir
00253897|transcript|ENST00000517788.1|unprocessed_pseudogene|8/8|n.950+1G>A||||||,T|downs
570|transcript|ENST00000261800.5|protein_coding|1/22|c.3259+2T>G||||||
77556|transcript|ENST00000524142.5|protein_coding|1/4|c.-229+2T>C||||||,G|5_prime_UTR_variar
ENST00000448073.8|protein_coding|1/16|c.31dupC|p.Leu11fs|59/2825|32/2751|11/916||INFO_REA
4|interaction|2D7I:A_464-A_588:ENST00000297107|protein_coding|12/12|c.1764G>A||||||,A|struct
155506|transcript|ENST00000518677.1|protein_coding|4/4|c.486-2A>C||||||WARNING_TRANSCRIP
170624|transcript|ENST00000517913.5|protein_coding|1/9|c.-281-2A>G||||||
55163|transcript|ENST00000611925.4|protein_coding|1/6|c.-24+2T>A||||||WARNING_TRANSCRIPT_
000248544|transcript|ENST00000520658.1|antisense|2/2|n.410+2C>T||||||,A|downstream_gene_v
05&ENSG00000248544|gene_variant|ENSG00000248544|||n.157384951_157384940delTTTTTTTTTTT
05&ENSG00000248544|gene_variant|ENSG00000248544|||n.157384951_157384940delTTTTTTTTTTT
05&ENSG00000248544|gene_variant|ENSG00000248544|||n.157384951_157384940delTTTTTTTTTTT
05&ENSG00000248544|gene_variant|ENSG00000248544|||n.157384951_157384940delTTTTTTTTTTT
05&ENSG00000248544|gene_variant|ENSG00000248544|||n.157384951_157384940delTTTTTTTTTTT
0113282|transcript|ENST00000522381.1|protein_coding|2/2|c.173-1G>A||||||,T|downstream_gene_
teraction|3MQI:A_315-A_344:ENST00000313708|protein_coding|10/16|c.1032T>C||||||,G|structura
0000145860|transcript|ENST00000611185.4|protein_coding|1/11|c.-319-2_-319-1insCC||||||,CGG|5
000253298|transcript|ENST00000518418.1|lincRNA|5/6|n.899+1G>A||||||,T|downstream_gene_vari
NSG00000249738|transcript|ENST00000636819.1|antisense|6/6|n.1587-3dupT||||||INFO_REALIGN_3
NSG00000249738|transcript|ENST00000636819.1|antisense|6/6|n.1587-3dupT||||||INFO_REALIGN_3
NSG00000249738|transcript|ENST00000636819.1|antisense|6/6|n.1587-3dupT||||||INFO_REALIGN_3
ISG00000241956|transcript|ENST00000519750.1|antisense|4/4|n.291-3dupT||||||INFO_REALIGN_3_
34516|transcript|ENST00000520908.5|protein_coding|1/37|c.-38+1G>A||||||,A|downstream_gene_
interaction|2YIN:A_1296-A_1438:ENST00000256935|protein_coding|43/52|c.4314C>T||||||,T|structu
0182132|transcript|ENST00000517344.1|nonsense_mediated_decay|1/3|c.89-2A>G||||||,G|splice_a
nteraction|2J7T:A_252-A_277:ENST00000176763|protein_coding|6/19|c.756G>A||||||,T|structural_ir
ENST00000326654.6|protein_coding|3/3|c.296delC|p.Pro99fs|511/3952|296/480|99/159||INFO_REA
719|transcript|ENST00000326654.6|protein_coding|3/3|c.432dupT|p.Lys145fs|648/3952|433/480|14
G00000204758|transcript|ENST00000518818.1|antisense|1/2|n.88+2dupT||||||,CA|splice_donor_var
00113734|transcript|ENST00000231668.13|protein_coding|2/6|c.178-3dupT||||||INFO_REALIGN_3_
00113734|transcript|ENST00000231668.13|protein_coding|2/6|c.178-3dupT||||||INFO_REALIGN_3_
00000145919|transcript|ENST00000311086.8|protein_coding|2/3|c.363-1_363insTTCA||||||,CTGAAC
00000145919|transcript|ENST00000311086.8|protein_coding|2/3|c.363-1_363insTTCA||||||,CTGAAC
|HIGH|BOD1|ENSG00000145919|transcript|ENST00000311086.8|protein_coding|2/3|c.363-2_363-1i
GH|LINC01484|ENSG00000253686|transcript|ENST00000518902.1|lincRNA|1/1|n.193-7_193-2delCA
GH|LINC01484|ENSG00000253686|transcript|ENST00000518902.1|lincRNA|1/1|n.193-7_193-2delCA
GH|LINC01484|ENSG00000253686|transcript|ENST00000518902.1|lincRNA|1/1|n.193-7_193-2delCA
00164466|transcript|ENST00000506963.1|protein_coding|1/6|c.-167-3dupT||||||WARNING_TRANS
00164466|transcript|ENST00000506963.1|protein_coding|1/6|c.-167-3dupT||||||WARNING_TRANS
000506378.1|protein_coding|5/5|c.610C>T|p.Gln204*|611/848|610/678|204/225||WARNING_TRAN
ENST00000303991.4|protein_coding|2/2|c.702_714delTCCTGGGTCTTTG|p.Asp234fs|892/4211|702/3

ENST00000303991.4|protein_coding|2/2|c.690_700delGAGGAAGGAGG|p.Arg231fs|878/4211|690/30
nt&intron_variant|HIGH|FGFR4|ENSG00000160867|transcript|ENST00000502906.5|protein_coding|1
7|interaction|4IRP:A_234-A_285:ENST00000029410|protein_coding|4/6|c.700C>T|||||,T|structural_
0170074|transcript|ENST00000514899.1|processed_transcript|1/2|n.99+1G>T|||||,A|splice_donor_
98939|transcript|ENST00000520301.5|protein_coding|2/3|c.-199-1G>C|||||,C|upstream_gene_vari
00127022|transcript|ENST00000509563.1|protein_coding|1/2|c.-125-3dupT|||||WARNING_TRANSC
00127022|transcript|ENST00000509563.1|protein_coding|1/2|c.-125-3dupT|||||WARNING_TRANSC
00127022|transcript|ENST00000509563.1|protein_coding|1/2|c.-125-3dupT|||||WARNING_TRANSC
7022|transcript|ENST00000509563.1|protein_coding|2/2|c.-4+2T>C|||||WARNING_TRANSCRIPT_NC
|interaction|4MJS:R_61-R_102:ENST00000389805|protein_coding|1/8|c.183C>T|||||,T|structural_i
teraction|4BSJ:A_391-A_448:ENST00000261937|protein_coding|10/30|c.1344C>T|||||,A|structural_
0512132.5|protein_coding|3/3|c.3G>C|p.Met1?|232/4010|3/2604|1/867||,G|start_lost|HIGH|ZFP62
NST00000328275.1|protein_coding|1/1|c.320_323delGTCT|p.Cys107fs|320/948|320/948|107/315||
00000233937|transcript|ENST00000417281.2|antisense|1/1|n.229+1G>A|||||,A|upstream_gene_va
ENST00000611618.1|protein_coding|1/2|c.59delG|p.Cys20fs|364/3466|59/894|20/297||,A|sequenc
2699|transcript|ENST00000476972.6|processed_transcript|1/2|n.96+1G>A|||||,T|intron_variant|M
0000250903|transcript|ENST00000505870.5|lincRNA|1/2|n.7234-1G>A|||||,A|splice_acceptor_vari
SG00000229282|transcript|ENST00000433761.1|lincRNA|1/1|n.136-3dupT|||||INFO_REALIGN_3_P
0000229282|transcript|ENST00000433761.1|lincRNA|1/1|n.136-3delT|||||INFO_REALIGN_3_PRIME
nteraction|4ITI:A_28-A_95:ENST00000380409|protein_coding|1/10|c.84T>C|||||,C|structural_intera
0000380306.8|protein_coding|2/3|c.117G>A|p.Trp39*|399/726|117/375|39/124||,A|upstream_gen
IGH|RP11-420L9.2|ENSG00000219992|transcript|ENST00000402155.2|processed_pseudogene|1/1|n.
anscript|ENST00000379802.7|protein_coding|1/24|c.1dupA|p.Met1fs|343/9796|2/8616|1/2871||IN
eraction|1LM7:A_2257-A_2374:ENST00000379802|protein_coding|24/24|c.7122C>T|||||,T|synonym
eraction|1LM5:A_2652-A_2725:ENST00000379802|protein_coding|24/24|c.8175C>A|||||,A|structu
|interaction|3UVT:A_403-A_428:ENST00000379757|protein_coding|10/10|c.1208G>A|||||,T|misse
0000515633.1|protein_coding|2/3|c.145C>T|p.Arg49*|145/460|145/171|49/56||WARNING_TRANSC
0181355|transcript|ENST00000492094.6|nonsense_mediated_decay|16/18|c.*1556-2A>G|||||WAR
NSG00000242753|transcript|ENST00000423149.1|lincRNA|2/4|n.266-2A>G|||||
on_coding_transcript_exon_variant|HIGH|RP11-125M16.1|ENSG00000234015|transcript|ENST00000
00112137|transcript|ENST00000406205.6|protein_coding|1/5|c.94_109dupTCCCTTCCTCTCCCCT|p.Se
145979|transcript|ENST00000607208.1|processed_transcript|2/3|n.170+2T>C|||||,G|downstream_
teraction|2B4Y:A_160-A_219:ENST00000606117|protein_coding|8/10|c.657C>T|||||,T|structural_i
nteraction|2BZG:A_182-A_240:ENST00000309983|protein_coding|9/9|c.719A>G|||||,C|structural_i
0.1|ENSG00000218976|transcript|ENST00000407347.2|unprocessed_pseudogene|2/2|n.996-6_996-2
00000228412|transcript|ENST00000638138.1|antisense|2/6|n.183+1G>C|||||,T|splice_donor_varia
SG00000228412|transcript|ENST00000607810.5|antisense|1/2|n.77-2A>G|||||,C|splice_acceptor_v
00000227803|transcript|ENST00000449143.2|antisense|1/2|n.91+2T>C|||||,C|missense_variant|M
00272168|transcript|ENST00000606336.5|lincRNA|4/6|n.702-3dupT|||||INFO_REALIGN_3_PRIME,
_coding_transcript_exon_variant|HIGH|GPLD1|ENSG00000112293|transcript|ENST00000492917.2|pr
ron_variant|HIGH|KIAA0319|ENSG00000137261|transcript|ENST00000543707.2|protein_coding|1/2
ron_variant|HIGH|KIAA0319|ENSG00000137261|transcript|ENST00000543707.2|protein_coding|1/2
ron_variant|HIGH|KIAA0319|ENSG00000137261|transcript|ENST00000543707.2|protein_coding|1/2
ron_variant|HIGH|KIAA0319|ENSG00000137261|transcript|ENST00000543707.2|protein_coding|1/2
ron_variant|HIGH|KIAA0319|ENSG00000137261|transcript|ENST00000543707.2|protein_coding|1/2
ron_variant|HIGH|KIAA0319|ENSG00000137261|transcript|ENST00000543707.2|protein_coding|1/2
000565469.3|protein_coding|2/2|c.691T>C|p.Ter231Glnext*?|723/814|691/693|231/230||,G|3_pri

00000282804|transcript|ENST00000562221.1|protein_coding|2/2|c.*6+1G>A||||||WARNING_TRANS
00000377905.8|protein_coding|11/12|c.1297C>T|p.Gln433*|1416/3616|1297/1494|433/497||,T|sto
0000357085.4|protein_coding|4/8|c.490C>T|p.Arg164*|966/9413|490/1398|164/465||
186470|transcript|ENST00000377708.6|protein_coding|2/10|c.-6+1G>A||||||,A|splice_donor_varian
186470|transcript|ENST00000377708.6|protein_coding|5/10|c.715+2T>G||||||,G|splice_donor_varia
00000244519.6|protein_coding|5/11|c.513G>A|p.Trp171*|756/3002|513/1755|171/584||,A|stop_ga
000312541.9|protein_coding|2/8|c.3G>A|p.Met1?|251/2920|3/1584|1/527||,A|start_lost|HIGH|BTN
ipt|ENST00000611927.1|protein_coding|1/1|c.143_144insT|p.Gly49fs|143/312|143/312|48/103||
0000425468.6|protein_coding|4/5|c.577C>T|p.Arg193*|742/1791|577/1338|193/445||,T|downstrea
0189298|transcript|ENST00000377255.3|protein_coding|2/6|c.-63+1G>A||||||,A|intron_variant|MO
:|ENST00000361028.5|protein_coding|5/5|c.1723delC|p.Leu575fs|1869/5495|1723/1815|575/604||
ipt|ENST00000259881.9|protein_coding|5/6|c.118dupC|p.His40fs|408/908|119/459|40/152||INFO_
cript|ENST00000374975.3|protein_coding|1/6|c.43_44insAC|p.Leu15fs|106/1260|43/801|15/266||
ipt|ENST00000374975.3|protein_coding|1/6|c.41_42delAG|p.Lys14fs|105/1260|41/801|14/266||
00198502|transcript|ENST00000374975.3|protein_coding|1/6|c.40_42delAAG|p.Lys14del|105/1260|
cript|ENST00000374975.3|protein_coding|1/6|c.39_40insGC|p.Lys14fs|102/1260|39/801|13/266||,C
cript|ENST00000374975.3|protein_coding|1/6|c.39_40insGC|p.Lys14fs|102/1260|39/801|13/266||,C
cript|ENST00000374975.3|protein_coding|1/6|c.39_40insGC|p.Lys14fs|102/1260|39/801|13/266||,C
t|ENST00000638142.1|nonsense_mediated_decay|12/21|c.1724_1725delCT|p.Pro575fs|1753/8162|1
teraction|4U2U:A_103-A_149:ENST00000374467|protein_coding|4/6|c.309G>A||||||,T|synonymous
161896|transcript|ENST00000634274.1|protein_coding|1/3|c.-225-2A>G||||||WARNING_TRANSCRIP
NST00000504758.5|nonsense_mediated_decay|1/11|c.17delG|p.Gly6fs|41/1978|17/165|6/54||,T|fra
000507909.1|protein_coding|2/3|c.65C>G|p.Ser22*|291/581|65/355|22/117||WARNING_TRANSCRII
on_coding_transcript_exon_variant|HIGH|DPRXP2|ENSG00000271304|transcript|ENST00000605269.1
000449261.6|nonsense_mediated_decay|9/11|c.2409G>A|p.Trp803*|2440/4816|2409/2466|803/82
00000457797.5|protein_coding|7/8|c.1467T>A|p.Tyr489*|1467/1602|1467/1602|489/533||,A|stop_
ENSG00000112078|transcript|ENST00000460983.1|protein_coding|2/4|c.-163-3dupT||||||WARNING
ENSG00000112078|transcript|ENST00000460983.1|protein_coding|2/4|c.-163-3dupT||||||WARNING
ENSG00000112078|transcript|ENST00000460983.1|protein_coding|2/4|c.-163-3dupT||||||WARNING
ENSG00000112078|transcript|ENST00000460983.1|protein_coding|2/4|c.-163-3dupT||||||WARNING
000112081|transcript|ENST00000373715.10|protein_coding|5/5|c.468-3dupT||||||INFO_REALIGN_3
000112081|transcript|ENST00000373715.10|protein_coding|5/5|c.468-3dupT||||||INFO_REALIGN_3
000112081|transcript|ENST00000373715.10|protein_coding|5/5|c.468-3dupT||||||INFO_REALIGN_3
000112081|transcript|ENST00000373715.10|protein_coding|5/5|c.468-3dupT||||||INFO_REALIGN_3
000112081|transcript|ENST00000373715.10|protein_coding|5/5|c.468-3dupT||||||INFO_REALIGN_3
teraction|2X7K:A_81-A_107:ENST00000373699|protein_coding|3/4|c.243G>A||||||,T|structural_inte
nteraction|2X7K:A_36-A_129:ENST00000373699|protein_coding|2/4|c.107G>C||||||,G|missense_var
ENST00000373616.9|protein_coding|1/12|c.30delC|p.Trp11fs|155/2006|30/1119|10/372||,A|frames
teraction|4AYC:A_415-A_448:ENST00000373479|protein_coding|7/8|c.1344G>A||||||,A|structural_i
00000373229.9|protein_coding|6/6|c.892C>T|p.Gln298*|906/1248|892/930|298/309||,A|stop_gaine
.2212|transcript|ENST00000373161.5|protein_coding|1/3|c.-21+1G>A||||||,A|splice_donor_variant&
1|interaction|2NYT:C_58-C_124:ENST00000244669|protein_coding|2/3|c.174T>C||||||,C|synonymou
0000469104.5|protein_coding|3/3|c.210G>A|p.Trp70*|423/704|210/267|70/88||,T|stop_gained|HIG
0000161912|transcript|ENST00000567255.2|processed_transcript|19/22|n.3795-1T>G||||||,G|upstr
interaction|1Q8M:D_25-D_127:ENST00000244709|protein_coding|2/4|c.73A>T||||||,A|structural_ir
088|transcript|ENST00000415707.1|protein_coding|1/2|c.71+2T>A||||||WARNING_TRANSCRIPT_NO_
NST00000415707.1|protein_coding|1/3|c.24dupA|p.Pro9fs|165/399|24/258|8/85||WARNING_TRANS

cript|ENST00000373006.5|protein_coding|7/7|c.1901_1905dupTGGGG|p.Asp636fs|2127/3177|1905/
'00000356542.5|protein_coding|3/3|c.281G>A|p.Trp94*|456/894|281/300|94/99||,T|intron_variant
ıteraction|3NY2:D_126-D_165:ENST00000372899|protein_coding|3/47|c.376G>A||||||,A|structural_
)24048|transcript|ENST00000372899.5|protein_coding|18/46|c.2098-2delA|||||INFO_REALIGN_3_F
)24048|transcript|ENST00000372899.5|protein_coding|18/46|c.2098-2delA|||||INFO_REALIGN_3_F
000408925.3|protein_coding|1/1|c.3G>A|p.Met1?|31/552|3/306|1/101||,T|downstream_gene_vari
interaction|4P5E:A_90-A_114:ENST00000230431|protein_coding|3/4|c.270C>T||||||,A|structural_in
)00137225|transcript|ENST00000532171.5|protein_coding|2/5|c.89-3dupT|||||WARNING_TRANSCI
ranscript|ENST00000398776.1|protein_coding|5/23|c.426_432dupGGCTGCC|p.Ile145fs|471/2728|43
interaction|4LQY:A_114-A_144:ENST00000321037|protein_coding|2/4|c.432C>G||||||,G|structural_i
interaction|4LQY:A_267-A_318:ENST00000321037|protein_coding|3/4|c.954C>G||||||,G|structural_i
|interaction|3D59:B_92-B_133:ENST00000274793|protein_coding|4/12|c.275G>A||||||,T|structural_
SG00000225730|transcript|ENST00000451135.1|antisense|1/3|n.109-2A>G||||||,G|intron_variant|N
)000096093|transcript|ENST00000636489.1|protein_coding|1/11|c.-122-3dupT|||||INFO_REALIGN_
)000096093|transcript|ENST00000636489.1|protein_coding|1/11|c.-122-3dupT|||||INFO_REALIGN_
)000096093|transcript|ENST00000636489.1|protein_coding|1/11|c.-122-3dupT|||||INFO_REALIGN_
)000096093|transcript|ENST00000636489.1|protein_coding|1/11|c.-122-3dupT|||||INFO_REALIGN_
nteraction|1PKW:B_125-B_171:ENST00000334575|protein_coding|5/7|c.375A>G||||||,C|structural_
nteraction|1TDI:A_73-A_155:ENST00000211122|protein_coding|4/7|c.217A>G||||||,C|structural_int
0000542638.5|protein_coding|7/7|c.736G>T|p.Gly246*|1107/2875|736/789|246/262||,A|synonymo
)0146143|transcript|ENST00000615550.4|protein_coding|2/13|c.155-3dupT|||||INFO_REALIGN_3_I
ction|4BPU:A_180-B_192:ENST00000338193-ENST00000615550|protein_coding|7/14|c.574C>T|||||
NSG00000217067|transcript|ENST00000405686.2|unprocessed_pseudogene|1/1|n.142-2delA|||||IN
)0000238918.12|protein_coding|1/16|c.83G>A|p.Trp28*|209/2927|83/2187|28/728||,A|upstream_
ENST00000619054.4|protein_coding|1/11|c.25delC|p.Leu9fs|124/3150|25/1374|9/457||INFO_REALI
0439432.1|protein_coding|5/5|c.375A>T|p.Ter125Cysext*?|376/419|375/375|125/124||WARNING_
)0000232295|transcript|ENST00000450998.5|antisense|4/4|n.321+1G>A||||||,T|splice_donor_varia
'9841|transcript|ENST00000431478.2|processed_transcript|2/5|n.331+1G>A||||||,A|intron_variant|
riant&intron_variant|HIGH|KHDC1|ENSG00000135314|transcript|ENST00000610435.4|protein_codin
)000370384.7|protein_coding|1/5|c.100C>T|p.Gln34*|601/1430|100/714|34/237||,A|non_coding_tr
)|interaction|4KM5:A_478-A_501:ENST00000370315|protein_coding|5/5|c.1503G>A||||||,T|structur
)0112701|transcript|ENST00000436928.7|processed_transcript|1/3|n.341dupA|||||INFO_REALIGN_
)0112701|transcript|ENST00000436928.7|processed_transcript|1/3|n.341dupA|||||INFO_REALIGN_
NST00000369952.3|protein_coding|1/4|c.80delC|p.Pro27fs|80/835|80/477|27/158||WARNING_TRA
t|ENST00000606868.5|nonsense_mediated_decay|4/5|c.520delA|p.Ile174fs|520/1634|520/534|174/
)000232031|transcript|ENST00000457116.1|sense_intronic|3/3|n.327+2T>C||||||,G|intron_variant|
teraction|4CNC:A_118-A_143:ENST00000369750|protein_coding|2/2|c.353C>T||||||,T|structural_int
eraction|2AW5:C_80-C_119:ENST00000369705|protein_coding|3/14|c.357G>A||||||,T|synonymous_
)000220291|transcript|ENST00000403057.1|unprocessed_pseudogene|2/5|n.445+2A>T|||||
teraction|4H2F:A_180-A_379:ENST00000257770|protein_coding|6/9|c.1136T>C||||||,C|missense_va
346|transcript|ENST00000610310.3|protein_coding|3/4|c.181+2C>T||||||,A|downstream_gene_vari
135336|transcript|ENST00000468486.2|retained_intron|3/3|n.206-1G>A||||||,A|intron_variant|MO
)0146276|transcript|ENST00000611484.4|protein_coding|2/10|c.-142-2G>A||||||,T|splice_acceptor
ɔt|ENST00000551025.3|protein_coding|8/9|c.4698delG|p.Thr1567fs|4933/6151|4698/5916|1566/19
ɔt|ENST00000551025.3|protein_coding|8/9|c.4703_4719delCTTTGCCCAGACATGGA|p.Ser1568fs|4938
interaction|3OHU:A_83-A_108:ENST00000257749|protein_coding|7/9|c.249A>G||||||,C|structural_i
4944|transcript|ENST00000437768.1|lincRNA|2/3|n.263+1G>A|||||

cript|ENST00000369293.5|protein_coding|2/2|c.564_567dupATAT|p.Val190fs|702/1076|568/597|19
transcript|ENST00000369293.5|protein_coding|2/2|c.567_568insATATATGT|p.Val190fs|702/1076|56
transcript|ENST00000369293.5|protein_coding|2/2|c.567_568insATATATGT|p.Val190fs|702/1076|56
transcript|ENST00000369293.5|protein_coding|2/2|c.567_568insATATATGT|p.Val190fs|702/1076|56
transcript|ENST00000369293.5|protein_coding|2/2|c.567_568insATATATGT|p.Val190fs|702/1076|56
ENST00000369293.5|protein_coding|2/2|c.582_583delGT|p.Ile196fs|716/1076|582/597|194/198||IN
00014123|transcript|ENST00000369278.4|protein_coding|17/18|c.1986-3dupT|||||INFO_REALIGN_3
123|transcript|ENST00000369278.4|protein_coding|17/18|c.1986-3delT|||||INFO_REALIGN_3_PRIM
0123545|transcript|ENST00000489477.1|processed_transcript|3/3|n.383+1G>A||||||,T|intron_varian
G00000271860|transcript|ENST00000457513.2|lincRNA|3/5|n.472+1G>A||||||,A|intron_variant|MOI
IST00000482541.2|protein_coding|5/5|c.423delT|p.Phe141fs|524/1704|423/432|141/143||,T|downs
7|interaction|2VN8:A_176-A_394:ENST00000369063|protein_coding|9/9|c.1182T>C||||||,G|structur
egion_variant&intron_variant|HIGH|PDSS2|ENSG00000164494|transcript|ENST00000449027.1|protei
00368972.7|protein_coding|4/17|c.2T>C|p.Met1?|598/3663|2/2109|1/702||,C|missense_variant|MO
00118690|transcript|ENST00000414610.1|protein_coding|2/2|c.225-2A>C|||||WARNING_TRANSCRIP
0000183137|transcript|ENST00000518329.5|protein_coding|1/2|c.-258-3dupT|||||WARNING_TRAN
0000183137|transcript|ENST00000518329.5|protein_coding|1/2|c.-258-3dupT|||||WARNING_TRAN
00183137|transcript|ENST00000521522.5|protein_coding|7/9|c.736-2A>T||||||,T|splice_acceptor_v
0000203799|transcript|ENST00000368966.9|unitary_pseudogene|24/46|n.3516-2G>A||||||,A|upstre
ENST00000458487.1|protein_coding|1/4|c.177delC|p.Ser60fs|179/613|177/611|59/202||WARNING_
T00000355283.1|protein_coding|5/5|c.883_884delGT|p.Val295fs|1131/2499|883/1011|295/336||,A
T00000355283.1|protein_coding|5/5|c.883delG|p.Val295fs|1130/2499|883/1011|295/336||,A|intro
T00000355283.1|protein_coding|5/5|c.883delG|p.Val295fs|1130/2499|883/1011|295/336||,A|intro
112367|transcript|ENST00000230124.7|protein_coding|4/22|c.447-3dupT|||||INFO_REALIGN_3_PR
112367|transcript|ENST00000230124.7|protein_coding|4/22|c.447-3dupT|||||INFO_REALIGN_3_PR
action|4N78:D_21-E_11:ENST00000359451-ENST00000530758|protein_coding|3/10|c.63C>T||||||,A|
00123505|transcript|ENST00000368885.7|protein_coding|6/8|c.616-3dupC|||||INFO_REALIGN_3_PI
00123505|transcript|ENST00000368885.7|protein_coding|6/8|c.616-3dupC|||||INFO_REALIGN_3_PI
000197498|transcript|ENST00000441448.6|protein_coding|6/9|c.394-4_394-3dupTT|||||INFO_REA
000197498|transcript|ENST00000441448.6|protein_coding|6/9|c.394-4_394-3dupTT|||||INFO_REA
00000368847.4|protein_coding|4/4|c.545T>A|p.Leu182*|898/5958|545/1557|182/518||
G00000228624|transcript|ENST00000520034.5|antisense|3/5|n.202-1G>A||||||,A|splice_acceptor_v
0000226079|transcript|ENST00000435802.1|lincRNA|1/1|n.160+1G>A|||||
0|interaction|1GR3:A_603-A_636:ENST00000243222|protein_coding|3/3|c.1809G>C||||||,G|structu
188820|transcript|ENST00000368605.2|protein_coding|2/2|c.525+2T>G||||||,G|upstream_gene_var
0000111832|transcript|ENST00000466444.6|protein_coding|5/6|c.548-3dupT|||||INFO_REALIGN_3_
0000111832|transcript|ENST00000466444.6|protein_coding|5/6|c.548-3dupT|||||INFO_REALIGN_3_
ipt|ENST00000310357.7|protein_coding|6/6|c.2324_2325insCC|p.Glu776fs|2346/2860|2324/2781|7
t|ENST00000310357.7|protein_coding|6/6|c.2323dupT|p.Tyr775fs|2345/2860|2323/2781|775/926||
00000310357.7|protein_coding|1/6|c.169C>T|p.Arg57*|191/2860|169/2781|57/926||,A|stop_gainec
teraction|3ZBF:A_2131-A_2205:ENST00000368508|protein_coding|42/43|c.6614G>T||||||,A|structu
nteraction|4UXL:A_2083-A_2127:ENST00000368508|protein_coding|40/43|c.6247A>C||||||,G|synony
H|TBC1D32|ENSG00000146350|transcript|ENST00000422369.1|protein_coding|1/5|c.-2+2_-2+5delTA
549|transcript|ENST00000618977.4|processed_transcript|2/4|n.268+1C>G||||||,G|splice_donor_var
00000235535|transcript|ENST00000587106.6|antisense|3/8|n.304+1G>A||||||,A|intron_variant|MO
NSG00000228704|transcript|ENST00000430672.1|lincRNA|1/2|n.163-2A>G|||||
00000224506|transcript|ENST00000423208.2|lincRNA|3/3|n.203-2A>T||||||,T|downstream_gene_v

)0324172.10|protein_coding|6/6|c.664T>C|p.Ter222Glnext*?|681/993|664/666|222/221||,G|3_prim
teraction|1WVA:A_50-A_90:ENST00000368087|protein_coding|3/8|c.270C>T||||||,T|structural_inte
950|transcript|ENST00000448348.3|processed_transcript|1/6|n.78+1delG||||||,A|5_prime_UTR_var
000434551.2|protein_coding|1/1|c.181A>T|p.Lys61*|181/1047|181/1047|61/348||
000367931.1|protein_coding|2/2|c.503G>A|p.Trp168*|503/1056|503/1056|168/351||,T|stop_gaine
2303|transcript|ENST00000418593.6|nonsense_mediated_decay|4/6|c.614+1G>T||||||,A|splice_dor
000231023|transcript|ENST00000457339.5|lincRNA|4/5|n.1853+1dupG|||||INFO_REALIGN_3_PRIM
461976.2|protein_coding|2/6|c.1A>G|p.Met1?|122/553|1/432|1/143||WARNING_TRANSCRIPT_NO_
G00000232876|transcript|ENST00000447508.1|antisense|1/1|n.455-2A>G||||||,G|intron_variant|M
G00000237499|transcript|ENST00000606998.1|antisense|1/3|n.593-2A>C||||||,G|upstream_gene_v
nt&intron_variant|HIGH|TNFAIP3|ENSG00000118503|transcript|ENST00000421450.1|protein_coding
|interaction|3ZJD:A_102-A_123:ENST00000237289|protein_coding|3/9|c.305A>G||||||,G|structural
|ENST00000527246.3|protein_coding|1/1|c.349dupC|p.Leu117fs|445/2506|349/408|117/135||,AG|i
nteraction|3R8J:A_143-A_189:ENST00000607197|protein_coding|4/4|c.427G>A||||||,A|missense_va
)0000231329|transcript|ENST00000586266.5|antisense|2/3|n.346+2C>T||||||,A|splice_region_variar
000367652.4|protein_coding|3/3|c.370C>T|p.Gln124*|371/398|370/397|124/131||WARNING_TRAN
)229036|transcript|ENST00000610068.5|processed_transcript|3/4|n.968+1G>A||||||,A|splice_donor
|ENST00000237275.8|protein_coding|4/8|c.336_337delAT|p.Ser113fs|440/1002|336/669|112/222|
|ENST00000237275.8|protein_coding|4/8|c.340_344delTTGAA|p.Leu114fs|444/1002|340/669|114/2
2818|transcript|ENST00000465299.1|processed_transcript|1/1|n.241+1G>C||||||,C|upstream_gene_
000438272.2|protein_coding|1/4|c.54C>G|p.Tyr18*|54/735|54/735|18/244||,C|stop_gained|HIGH|
ENST00000367245.5|protein_coding|1/2|c.66_69delTAGA|p.Asp22fs|74/3030|66/708|22/235||INFO_
0000419506.6|protein_coding|4/4|c.1231C>T|p.Gln411*|1281/1402|1231/1263|411/420||,T|intron_
0000337049.8|protein_coding|4/4|c.1201C>T|p.Arg401*|1251/1389|1201/1257|401/418||,T|synony
teraction|2RAJ:A_258-A_284:ENST00000392185|protein_coding|8/18|c.774G>A||||||,A|synonymous
0233496|transcript|ENST00000442328.1|sense_intronic|2/2|n.389+1G>A||||||,A|intron_variant|MC
0000607742.5|nonsense_mediated_decay|3/15|c.102T>A|p.Tyr34*|210/5155|102/129|34/42||,T|st
NST00000297267.13|protein_coding|14/23|c.4498_4501delACCA|p.Thr1500fs|4698/6552|4498/5685
nteraction|1WL4:A_116-A_261:ENST00000367048|protein_coding|7/9|c.783C>T||||||,T|structural_i
interaction|1WL4:A_284-A_391:ENST00000367048|protein_coding|7/9|c.851C>G||||||,G|structural_
257|transcript|ENST00000609176.1|antisense|1/2|n.1184+2T>A||||||,T|upstream_gene_variant|MC
3071|transcript|ENST00000435757.6|processed_transcript|9/9|n.1504+2G>A||||||,T|splice_donor_v
70|transcript|ENST00000316300.9|protein_coding|26/38|c.4289+1G>A||||||,T|downstream_gene_v
nteraction|4BM9:A_167-A_205:ENST00000366898|protein_coding|4/12|c.500G>A||||||,T|structural_
35345|transcript|ENST00000612540.1|protein_coding|10/12|c.340+1A>T|||||WARNING_TRANSCRIP
35345|transcript|ENST00000612540.1|protein_coding|2/12|c.101+2C>G|||||WARNING_TRANSCRIPT
|interaction|2OUN:A_672-A_738:ENST00000539869|protein_coding|21/22|c.2214T>C||||||,G|struct
|interaction|3UUO:A_511-A_603:ENST00000539869|protein_coding|17/22|c.1809C>T||||||,A|struct
|interaction|2ZMF:A_288-A_383:ENST00000366882|protein_coding|12/23|c.864G>A||||||,T|structur
7|interaction|3T0O:A_49-A_236:ENST00000508775|protein_coding|9/9|c.706C>T||||||,A|structural_
)0000230256.7|protein_coding|3/8|c.452G>A|p.Trp151*|627/2116|452/1374|151/457||,A|stop_gai
112494|transcript|ENST00000230256.7|protein_coding|4/7|c.625+1G>C||||||,C|downstream_gene_
)0000230256.7|protein_coding|5/8|c.676C>T|p.Arg226*|851/2116|676/1374|226/457||,T|stop_gain
NST00000476779.2|protein_coding|4/4|c.304delT|p.Cys102fs|455/646|304/324|102/107||,C|downs
000476779.2|protein_coding|4/4|c.300T>A|p.Cys100*|451/646|300/324|100/107||,T|downstream_
3690|transcript|ENST00000617120.4|protein_coding|2/8|c.27+1T>G||||||,C|5_prime_UTR_variant|
_coding_transcript_exon_variant|HIGH|RP11-503C24.3|ENSG00000228361|transcript|ENST0000043

164488|transcript|ENST00000607983.1|protein_coding|1/1|c.-171-2A>G||||||,C|missense_variant|
000184465|transcript|ENST00000448612.5|protein_coding|22/26|c.2225delG|p.Gly742fs|2335/3178
84465|transcript|ENST00000486490.1|processed_transcript|1/3|n.242+1G>A||||||,T|upstream_gen
T00000230354.10|protein_coding|3/8|c.228_229delGC|p.Gln77fs|464/1861|228/1020|76/339||,A|f
T00000230354.10|protein_coding|3/8|c.228_229delGC|p.Gln77fs|464/1861|228/1020|76/339||,A|f
00112592|transcript|ENST00000230354.10|protein_coding|3/8|c.231delG|p.Gln77fs|467/1861|231/
00112592|transcript|ENST00000230354.10|protein_coding|3/8|c.231delG|p.Gln77fs|467/1861|231/
00112592|transcript|ENST00000230354.10|protein_coding|3/8|c.231delG|p.Gln77fs|467/1861|231/
eraction|1C9B:B_169-B_257:ENST00000230354|protein_coding|6/8|c.771T>C||||||,C|structural_inte
pt|ENST00000426056.1|protein_coding|1/3|c.102_105dupGTCC|p.Phe36fs|132/502|106/476|36/157
interaction|3FEH:A_181-A_251:ENST00000265846|protein_coding|6/11|c.543C>T||||||,A|structural_
NST00000617001.1|protein_coding|2/2|c.1180_1181delCT|p.Leu394fs|1281/1900|1180/1227|394/4
0000271098|transcript|ENST00000604197.1|processed_pseudogene|1/1|n.167-1delA||||||INFO_RE
0000271098|transcript|ENST00000604197.1|processed_pseudogene|1/1|n.167-1delA||||||INFO_RE
nt&intron_variant|HIGH|CHST12|ENSG00000136213|transcript|ENST00000618655.1|protein_coding|
IST00000402506.5|protein_coding|2/9|c.159_166delGATGGATG|p.Trp53fs|285/2268|159/927|53/30
IST00000402506.5|protein_coding|2/9|c.159_166delGATGGATG|p.Trp53fs|285/2268|159/927|53/30
00407112.1|protein_coding|6/6|c.874C>T|p.Arg292*|1187/1862|874/894|292/297||,T|synonymou
46535|transcript|ENST00000496740.6|processed_transcript|2/3|n.472+1G>A||||||,T|upstream_gene
00407653.1|protein_coding|1/3|c.1A>G|p.Met1?|253/3785|1/918|1/305||,C|upstream_gene_varian
0000196204|transcript|ENST00000404006.5|processed_transcript|3/5|n.365-1G>A||||||,A|splice_ac
transcript|ENST00000399434.2|protein_coding|3/3|c.388_391delTTTT|p.Phe130fs|550/1459|388/39
transcript|ENST00000399434.2|protein_coding|3/3|c.388_391delTTTT|p.Phe130fs|550/1459|388/39
transcript|ENST00000399434.2|protein_coding|3/3|c.388_391delTTTT|p.Phe130fs|550/1459|388/39
transcript|ENST00000399434.2|protein_coding|3/3|c.388_391delTTTT|p.Phe130fs|550/1459|388/39
nteraction|3LLP:A_305-A_336:ENST00000382361|protein_coding|3/5|c.1008T>C||||||,C|structural_i
nteraction|1H7S:A_29-A_100:ENST00000265849|protein_coding|2/15|c.86G>C||||||,G|missense_var
nteraction|4KAX:B_305-B_344:ENST00000350796|protein_coding|12/13|c.1032T>G||||||,C|synonym
|CYTH3|ENSG00000008256|transcript|ENST00000350796.7|protein_coding|8/12|c.711+2_711+3delT
00000230825|transcript|ENST00000436578.1|retained_intron|1/1|n.536-1G>C||||||,C|splice_accep
on|HIGH|UMAD1|ENSG00000219545|transcript|ENST00000463725.5|protein_coding|2/5|c.-3_-1dup
on|HIGH|UMAD1|ENSG00000219545|transcript|ENST00000463725.5|protein_coding|2/5|c.-3_-1dup
on|HIGH|UMAD1|ENSG00000219545|transcript|ENST00000463725.5|protein_coding|2/5|c.-3_-1dup
on|HIGH|UMAD1|ENSG00000219545|transcript|ENST00000463725.5|protein_coding|2/5|c.-3_-1dup
on|HIGH|UMAD1|ENSG00000219545|transcript|ENST00000463725.5|protein_coding|2/5|c.-3_-1dup
on|HIGH|UMAD1|ENSG00000219545|transcript|ENST00000463725.5|protein_coding|2/5|c.-3_-1dup
ST00000407906.5|protein_coding|8/8|c.972_990delTTTTTTTTTTTTTTTTTTT|p.Phe324fs|1242/1615|97
ST00000407906.5|protein_coding|8/8|c.965_983delCTTTTTTTTTTTTTTTTTT|p.Ser322fs|1235/1615|96
ST00000407906.5|protein_coding|8/8|c.965_983delCTTTTTTTTTTTTTTTTTT|p.Ser322fs|1235/1615|96
ENST00000614403.4|protein_coding|16/26|c.2178dupT|p.Lys727fs|3478/4701|2178/2835|726/944|
000452576.6|nonsense_mediated_decay|8/30|c.1153C>T|p.Arg385*|1378/5936|1153/3909|385/13
00000635746.1|protein_coding|3/7|c.2T>C|p.Met1?|935/1895|2/615|1/204||,C|missense_variant|
cript|ENST00000636804.1|protein_coding|2/6|c.274dupA|p.Ile92fs|275/771|275/771|92/256||INFO
747|transcript|ENST00000417018.1|protein_coding|2/3|c.280+2T>C||||||WARNING_TRANSCRIPT_IN
00000229618|transcript|ENST00000443947.1|lincRNA|1/1|n.194-2A>G||||||,G|intron_variant|MOD
|ENST00000307068.4|protein_coding|2/2|c.592delA|p.Ser198fs|773/1869|592/621|198/206||,C|fra
0136261|transcript|ENST00000258761.7|protein_coding|4/11|c.340-3dupT||||||INFO_REALIGN_3_

00000237773|transcript|ENST00000452249.5|antisense|1/2|n.96-2A>G||||||,C|splice_acceptor_vari
|ENST00000506618.3|protein_coding|1/1|c.434_438delTGTAT|p.Leu145fs|519/1090|434/957|145/3
)4846|transcript|ENST00000404938.6|protein_coding|3/27|c.108+1G>C|||||
0443026.6|protein_coding|4/6|c.2T>C|p.Met1?|548/2247|2/396|1/131||,C|start_lost|HIGH|ABCB5|
)0000328843.10|protein_coding|1/83|c.100G>T|p.Glu34*|131/13917|100/13572|34/4523||,T|stop_
105877|transcript|ENST00000607413.5|processed_transcript|1/3|n.159+1G>A||||||,A|upstream_ge
)000196683|transcript|ENST00000372879.8|protein_coding|3/4|c.289delA|p.Thr97fs|301/514|289/3
)000196683|transcript|ENST00000372879.8|protein_coding|3/4|c.289delA|p.Thr97fs|301/514|289/3
:NST00000548367.2|protein_coding|1/3|c.21_24delTCCC|p.Pro8fs|21/249|21/249|7/82||WARNING_
t|ENST00000429719.5|nonsense_mediated_decay|3/8|c.245delT|p.Leu82fs|297/4090|245/345|82/1
T00000429719.5|nonsense_mediated_decay|3/8|c.245T>A|p.Leu82*|297/4090|245/345|82/114||,A
0188732|transcript|ENST00000446234.6|nonsense_mediated_decay|3/4|c.*38+2T>C||||||,C|3_prim
196335|transcript|ENST00000422637.5|protein_coding|3/6|c.82-1G>C||||||WARNING_TRANSCRIPT_
iant&intron_variant|HIGH|CTB-113D17.1|ENSG00000272568|transcript|ENST00000609389.5|antisens
GH|DPY19L2P3|ENSG00000227855|transcript|ENST00000414296.2|transcribed_unprocessed_pseudo
)0000283618&ENSG00000106086|gene_variant|ENSG00000283618|||n.30116002_30115971delTATG
)0000283618&ENSG00000106086|gene_variant|ENSG00000283618|||n.30116002_30115971delTATG
G00000264520|transcript|ENST00000583191.1|lincRNA|2/2|n.151-1G>C||||||,C|intron_variant|MC
000196295|transcript|ENST00000584621.5|antisense|1/4|n.89+2T>C||||||,G|splice_donor_variant&
nteraction|2A14:A_205-A_246:ENST00000013222|protein_coding|3/3|c.613A>G||||||,G|structural_ir
nteraction|2A14:A_231-A_258:ENST00000013222|protein_coding|3/3|c.773G>A||||||,A|missense_va
013222.5|protein_coding|3/3|c.791G>C|p.Ter264Serext*?|807/2559|791/792|264/263||,C|stop_lost
pt|ENST00000451887.6|protein_coding|15/15|c.3106dupT|p.Ser1036fs|3201/3611|3107/3189|1036/
i00000229358|transcript|ENST00000417811.2|unprocessed_pseudogene|2/21|n.326-2dupA||||||,CT
script|ENST00000297157.7|protein_coding|6/6|c.664delT|p.Ter222fs|682/1121|664/666|222/221||
)381539.3|protein_coding|10/10|c.1171T>C|p.Ter391Argext*?|1175/1233|1171/1173|391/390||,C|
000359791.5|protein_coding|9/9|c.1078C>T|p.Arg360*|1206/1410|1078/1134|360/377||,T|stop_ga
000359791.5|protein_coding|9/9|c.1078C>T|p.Arg360*|1206/1410|1078/1134|360/377||,T|stop_ga
)0011426|transcript|ENST00000265748.6|protein_coding|18/23|c.2749-3dupT||||||INFO_REALIGN_
)0011426|transcript|ENST00000265748.6|protein_coding|18/23|c.2749-3dupT||||||INFO_REALIGN_
0000010270|transcript|ENST00000453225.5|protein_coding|1/6|c.-311-2A>G||||||WARNING_TRANS
0000390340.2|TR_V_gene|1/1|c.110G>A|p.Trp37*|264/463|110/309|37/102||WARNING_TRANSCRI
t|ENST00000518318.6|protein_coding|5/10|c.550_551insA|p.Leu184fs|593/5925|551/1968|184/655
pt|ENST00000518318.6|protein_coding|5/10|c.552_553insAG|p.Gln185fs|595/5925|553/1968|185/6
|ENSG00000229161|transcript|ENST00000412860.1|unprocessed_pseudogene|2/2|n.1485-6_1485-3c
29161|transcript|ENST00000412860.1|unprocessed_pseudogene|2/2|n.1485-6_1485-3delAAAT|||||
)00232006|transcript|ENST00000629291.2|lincRNA|2/3|n.382+1G>A||||||,T|splice_donor_variant&i
)6608|transcript|ENST00000497914.5|processed_transcript|6/6|n.993+1G>A||||||,T|downstream_g
NST00000458246.5|nonsense_mediated_decay|5/10|c.729delG|p.Leu244fs|802/3218|729/813|243/
ENST00000447951.1|protein_coding|3/5|c.227dupC|p.Ser77fs|227/516|227/516|76/171||WARNING_
1|interaction|3BHH:A_194-A_260:ENST00000395749|protein_coding|8/24|c.582T>C||||||,G|structur
1|interaction|3BHH:A_135-A_191:ENST00000395749|protein_coding|8/24|c.573T>C||||||,G|structur
|interaction|3QNT:A_157-A_221:ENST00000289547|protein_coding|2/20|c.661C>T||||||,A|structura
nteraction|4WJ7:A_82-A_143:ENST00000258781|protein_coding|3/10|c.246C>T||||||,T|structural_in
interaction|1ZT3:A_230-A_251:ENST00000275525|protein_coding|4/4|c.690C>T||||||,T|structural_ir
)000448817.1|protein_coding|1/3|c.28C>T|p.Arg10*|203/571|28/396|10/131||WARNING_TRANSCR
00000233539|transcript|ENST00000487094.1|lincRNA|4/4|n.378-1G>A|||||

000233539|transcript|ENST00000487094.1|lincRNA|2/4|n.213+2T>C||||||
.79869|transcript|ENST00000435803.5|protein_coding|41/61|c.12459+1G>T||||||,T|splice_donor_va
eraction|3RBF:A_75-A_462:ENST00000357936|protein_coding|14/15|c.1385G>A||||||,T|structural_i
eraction|3RBF:A_239-A_267:ENST00000357936|protein_coding|7/15|c.717G>T||||||,A|structural_in
eraction|3RCH:A_78-A_451:ENST00000357936|protein_coding|3/15|c.234C>T||||||,A|structural_int
interaction|1NRV:A_437-A_461:ENST00000398810|protein_coding|14/16|c.1311C>T||||||,A|structur
0000224223|transcript|ENST00000456049.1|sense_overlapping|1/3|n.326+1G>C||||||,C|downstrea
teraction|1XKK:A_714-A_787:ENST00000275493|protein_coding|20/28|c.2361G>A||||||,A|structura
teraction|1XKK:A_836-A_865:ENST00000275493|protein_coding|21/28|c.2508C>T||||||,T|structural_
.26278|transcript|ENST00000450062.1|unprocessed_pseudogene|1/1|n.153+1A>T||||||
on_coding_transcript_exon_variant|HIGH|RP11-700P18.1|ENSG00000223559|transcript|ENST00000044
W|RP11-700P18.1|ENSG00000223559|transcript|ENST00000449426.1|processed_pseudogene|2/2|r
|LOW|RP11-700P18.1|ENSG00000223559|transcript|ENST00000449426.1|processed_pseudogene|2/
t|LOW|RP11-700P18.1|ENSG00000223559|transcript|ENST00000449426.1|processed_pseudogene|2
nt|LOW|RP11-700P18.1|ENSG00000223559|transcript|ENST00000449426.1|processed_pseudogene|
iant|LOW|RP11-700P18.1|ENSG00000223559|transcript|ENST00000449426.1|processed_pseudogene
00000229909|transcript|ENST00000444048.1|processed_pseudogene|1/1|n.298-1A>G||||||
0229508|transcript|ENST00000430144.1|unprocessed_pseudogene|1/1|n.126+1G>C||||||
NSG00000260653|transcript|ENST00000561575.5|lincRNA|4/4|n.1687-2G>A||||||,A|downstream_ge
0231523|transcript|ENST00000458703.1|unprocessed_pseudogene|3/5|n.294+1G>A||||||
000231523|transcript|ENST00000458703.1|unprocessed_pseudogene|1/5|n.76-2delC||||||
0000231523|transcript|ENST00000458703.1|unprocessed_pseudogene|1/5|n.76-2C>A||||||
0000231523|transcript|ENST00000458703.1|unprocessed_pseudogene|1/5|n.76-2C>A||||||
00223566|transcript|ENST00000417666.1|unprocessed_pseudogene|7/10|n.897+1C>T||||||
0000282869.10|protein_coding|4/4|c.1282C>T|p.Arg428*|2567/9080|1282/1452|428/483||,A|stop_
0000394323.2|protein_coding|2/2|c.667C>T|p.Arg223*|1168/2719|667/1815|223/604||,A|upstream
GH|AC104073.1|ENSG00000280410|transcript|ENST00000624631.1|protein_coding|2/2|c.463-1delG
GH|AC104073.1|ENSG00000280410|transcript|ENST00000624631.1|protein_coding|2/2|c.463-1delG
on_coding_transcript_exon_variant|HIGH|RP11-746P2.6|ENSG00000277544|transcript|ENST0000061
on_coding_transcript_exon_variant|HIGH|RP11-746P2.6|ENSG00000277544|transcript|ENST0000061
on_coding_transcript_exon_variant|HIGH|RP11-746P2.6|ENSG00000277544|transcript|ENST0000061
on_coding_transcript_exon_variant|HIGH|RP11-746P2.6|ENSG00000277544|transcript|ENST0000061
GH|AC104057.1|ENSG00000280427|transcript|ENST00000624636.1|protein_coding|2/3|c.463-1delG
GH|RP11-479O9.4|ENSG00000272693|transcript|ENST00000608973.2|lincRNA|2/2|n.463-1delG||||
GH|RP11-479O9.4|ENSG00000272693|transcript|ENST00000608973.2|lincRNA|2/2|n.463-1delG||||
iant&intron_variant&non_coding_transcript_exon_variant|HIGH|RP11-479O9.4|ENSG00000272693|tr
iant&intron_variant&non_coding_transcript_exon_variant|HIGH|RP11-479O9.4|ENSG00000272693|tr
teraction|1BHG:A_536-A_580:ENST00000304895|protein_coding|11/12|c.1740C>T||||||,A|structura
00235475|transcript|ENST00000430244.1|lincRNA|2/2|n.261+1G>A||||||
00232975|transcript|ENST00000432182.1|unprocessed_pseudogene|1/1|n.123+1A>T||||||,T|intron
00232975|transcript|ENST00000432182.1|unprocessed_pseudogene|1/1|n.123+1A>T||||||,T|intron
0000620995.4|protein_coding|9/14|c.1118G>A|p.Trp373*|1241/3113|1118/2007|373/668||,T|stop_
|ENST00000620995.4|protein_coding|8/14|c.1014dupT|p.Glu339fs|1137/3113|1014/2007|338/668|
|ENST00000395270.5|protein_coding|14/16|c.1293_1294delGT|p.Ser432fs|2334/7011|1293/3000|4
000005180.8|protein_coding|2/3|c.132G>A|p.Trp44*|213/526|132/285|44/94||,T|stop_gained|HIG
6178|transcript|ENST00000416943.1|protein_coding|1/3|c.-60+1G>T||||||
|interaction|2B05:A_128-A_150:ENST00000307630|protein_coding|2/2|c.450C>T||||||,A|structural_

_coding_transcript_exon_variant|HIGH|SSC4D|ENSG00000146700|transcript|ENST00000492979.2|pro
)187391|transcript|ENST00000637976.1|processed_transcript|1/1|n.28-2A>G|||||,C|upstream_ger
)000522342.3|protein_coding|4/4|c.538C>T|p.Gln180*|538/643|538/552|180/183||WARNING_TRAN
nteraction|1KJY:A_43-A_178:ENST00000351004|protein_coding|2/8|c.129A>G|||||,G|structural_int
eraction|1BHT:A_49-A_111:ENST00000222390|protein_coding|3/18|c.333A>G|||||,C|structural_inte
8822|transcript|ENST00000421579.1|protein_coding|1/1|c.-141+2T>C|||||WARNING_TRANSCRIPT_
000135164|transcript|ENST00000547146.6|nonsense_mediated_decay|10/17|c.1050-3dupT|||||INI
000135164|transcript|ENST00000547146.6|nonsense_mediated_decay|10/17|c.1050-3dupT|||||INI
)0157224|transcript|ENST00000451941.6|processed_transcript|2/6|n.80-1G>A|||||,A|splice_accep
)0000235450|transcript|ENST00000456952.1|lincRNA|1/1|n.112+2C>T|||||
127989|transcript|ENST00000454222.5|processed_transcript|4/4|n.221+2C>T|||||
nteraction|4HDQ:A_640-A_660:ENST00000340022|protein_coding|17/19|c.1980A>G|||||,C|structur
)000481551.5|protein_coding|4/4|c.1051C>T|p.Arg351*|1092/4093|1051/1131|351/376||,T|downs
teraction|1XO2:B_106-B_148:ENST00000265734|protein_coding|4/8|c.444A>T|||||,A|structural_in
)0000318238.8|protein_coding|5/5|c.1216C>T|p.Arg406*|2433/7134|1216/4755|406/1584||,A|stop
0544910.5|protein_coding|3/29|c.1A>G|p.Met1?|247/5736|1/2805|1/934||,G|3_prime_UTR_varian
)00004766|transcript|ENST00000458707.1|protein_coding|12/12|c.1214-3dupT|||||WARNING_TRA
)00004766|transcript|ENST00000458707.1|protein_coding|12/12|c.1214-3dupT|||||WARNING_TRA
4948|transcript|ENST00000415529.2|nonsense_mediated_decay|8/12|c.*27+1G>A|||||,T|splice_do
teraction|2ZDX:B_293-B_357:ENST00000005178|protein_coding|10/11|c.1071A>G|||||,C|synonym
teraction|2ZDX:A_138-A_332:ENST00000005178|protein_coding|10/11|c.996C>T|||||,A|structural_
)0431419.1|protein_coding|1/4|c.3G>C|p.Met1?|127/608|3/348|1/115||,C|upstream_gene_variant|
97343|transcript|ENST00000422647.5|protein_coding|1/3|c.-28+2T>C|||||WARNING_TRANSCRIPT_
)0439761.1|protein_coding|5/5|c.423A>G|p.Ter141Trpext*?|523/1075|423/423|141/140||,C|downs
)0106258|transcript|ENST00000339843.6|protein_coding|3/12|c.*467-1A>G|||||,C|splice_acceptor
)0000021461|transcript|ENST00000222382.5|protein_coding|2/13|c.74delA|p.Tyr25fs|74/1515|74/15
500000021461|transcript|ENST00000436834.5|nonsense_mediated_decay|5/9|c.*364-3dupT|||||IN
500000021461|transcript|ENST00000436834.5|nonsense_mediated_decay|5/9|c.*364-3dupT|||||IN
500000021461|transcript|ENST00000436834.5|nonsense_mediated_decay|5/9|c.*364-3dupT|||||IN
)146833|transcript|ENST00000354241.5|protein_coding|5/5|c.842-2A>G|||||,C|intron_variant|MO
IST00000413658.6|protein_coding|6/6|c.292_295delACTA|p.Thr98fs|585/1441|292/396|98/131||,G|
interaction|4MDR:A_225-A_271:ENST00000359593|protein_coding|10/15|c.812G>A|||||,A|structu
000066923|transcript|ENST00000422690.5|protein_coding|1/3|c.-65_-65+1insTGAG|||||WARNING_
)00317271.2|protein_coding|2/6|c.52G>T|p.Glu18*|415/1583|52/981|18/326||,T|3_prime_UTR_var
NST00000300179.6|protein_coding|4/7|c.1329_1330delTG|p.Ala444fs|1488/3581|1329/2526|443/8
NST00000300179.6|protein_coding|4/7|c.1335_1336delGG|p.Ala446fs|1494/3581|1335/2526|445/8
NST00000300179.6|protein_coding|4/7|c.1339_1352delGCCTTGCTCCCCGG|p.Ala447fs|1498/3581|13
339|transcript|ENST00000613979.4|protein_coding|11/47|c.1249+1G>A|||||,A|splice_donor_varian
)0613979.4|protein_coding|31/48|c.5649G>A|p.Trp1883*|5814/8669|5649/8439|1883/2812||,A|sto
T00000613979.4|protein_coding|31/48|c.5768delG|p.Cys1923fs|5933/8669|5768/8439|1923/2812|
NST00000613979.4|protein_coding|31/48|c.5769_5770insTT|p.Arg1924fs|5935/8669|5770/8439|19
NST00000613979.4|protein_coding|31/48|c.5769_5770insTT|p.Arg1924fs|5935/8669|5770/8439|19
339|transcript|ENST00000613979.4|protein_coding|35/47|c.6622+1G>A|||||,A|splice_donor_varian
ST00000613979.4|protein_coding|38/48|c.7032_7066delCTTCAGCTACCGCTTGCAAGGCCGCATGACCTAT
interaction|2BBA:A_108-A_182:ENST00000358173|protein_coding|4/17|c.546T>C|||||,G|structural_
ENST00000379458.8|protein_coding|2/12|c.1591_1592delGC|p.Ala531fs|1661/11226|1591/9972|53
ot|ENST00000379458.8|protein_coding|2/12|c.1593_1594insGT|p.Thr532fs|1664/11226|1594/9972|

ENST00000379458.8|protein_coding|2/12|c.6545_6549delCCTAC|p.Ser2182fs|6615/11226|6545/997

ENST00000379458.8|protein_coding|2/12|c.6545_6549delCCTAC|p.Ser2182fs|6615/11226|6545/997

ript|ENST00000379458.8|protein_coding|2/12|c.7152_7153insACAT|p.Pro2385fs|7223/11226|7153/

ENST00000379458.8|protein_coding|2/12|c.7156_7159delTTAC|p.Leu2386fs|7226/11226|7156/9972

script|ENST00000379458.8|protein_coding|2/12|c.8736_8737insCCCCC|p.Ser2913fs|8807/11226|873

ENST00000379458.8|protein_coding|2/12|c.8803_8804delTC|p.Ser2935fs|8873/11226|8803/9972|2

t|ENST00000379458.8|protein_coding|2/12|c.8807_8808insTG|p.Arg2937fs|8878/11226|8808/9972

G00000274993|transcript|ENST00000618276.1|antisense|5/5|n.1722-1G>C||||||,G|upstream_gene

script|ENST00000306151.8|protein_coding|3/13|c.4033_4034insGAACA|p.Asn1345fs|4098/14247|40

t|ENST00000306151.8|protein_coding|3/13|c.4033_4034insGA|p.Asn1345fs|4098/14247|4034/134

|transcript|ENST00000435848.1|protein_coding|4/4|c.379_389dupCCTTTCCCTGT|p.Pro132fs|614/6

ENST00000465829.5|protein_coding|10/21|c.859_865delCCAGGGC|p.Pro287fs|936/2931|859/2412|

257|transcript|ENST00000420058.1|antisense|2/3|n.390+1G>A||||||,A|intron_variant|MODIFIER|F

000128536|transcript|ENST00000474330.1|processed_transcript|3/3|n.1026-3dupT||||||INFO_REA

000128536|transcript|ENST00000474330.1|processed_transcript|3/3|n.1026-3dupT||||||INFO_REA

interaction|1E8Y:A_359-A_420:ENST00000359195|protein_coding|2/11|c.1076G>A||||||,A|structura

interaction|1HE8:A_355-A_522:ENST00000359195|protein_coding|2/11|c.1565A>G||||||,G|structur

interaction|3ENE:A_887-A_950:ENST00000359195|protein_coding|9/11|c.2850C>T||||||,T|structura

NST00000205386.8|protein_coding|34/34|c.5265delA|p.Lys1755fs|5345/5857|5265/5286|1755/176

0091129|transcript|ENST00000613830.4|protein_coding|21/21|c.2296-1A>G||||||,C|upstream_gen

on_coding_transcript_exon_variant|HIGH|NPM1P14|ENSG00000182965|transcript|ENST0000034374

M1P14|ENSG00000182965|transcript|ENST00000343743.4|processed_pseudogene|2/2|n.665-2_667

IER|NPM1P14|ENSG00000182965|transcript|ENST00000343743.4|processed_pseudogene|2/2|n.665

|ENST00000284601.3|protein_coding|4/4|c.1985_1986delAG|p.Gln662fs|2055/4384|1985/3369|662

|ENSG00000244565|transcript|ENST00000462471.1|processed_pseudogene|1/1|n.39-3dupA||||||IN

G00000244565|transcript|ENST00000462471.1|processed_pseudogene|1/1|n.39-3delA||||||INFO_RE

n|2XQN:A_34-T_374:ENST00000333999-ENST00000358204|protein_coding|7/7|c.1122G>C||||||,C|

ENST00000451122.5|nonsense_mediated_decay|1/3|c.124_125dupCC|p.Ala43fs|180/1653|126/261

eraction|2UZX:B_48-B_508:ENST00000397752|protein_coding|2/21|c.144G>A||||||,A|structural_int

eraction|2UZX:B_178-B_199:ENST00000397752|protein_coding|2/21|c.534C>T||||||,T|structural_int

05976|transcript|ENST00000495962.1|processed_transcript|1/5|n.105-2delA||||||,C|intron_variant|

G00000235945|transcript|ENST00000299783.6|processed_pseudogene|2/3|n.189-1dupA||||||,GT|d

2745|transcript|ENST00000361301.6|protein_coding|1/4|c.-3_1dupCCCA|p.Met1fs|51/1230|2/1068|

2745|transcript|ENST00000361301.6|protein_coding|1/4|c.-3_1dupCCCA|p.Met1fs|51/1230|2/1068|

0000240499|transcript|ENST00000602199.5|antisense|1/3|n.40+1G>A||||||,A|splice_donor_varian

0000240499|transcript|ENST00000626052.2|antisense|5/5|n.525+1G>A||||||,A|intron_variant|MO

0340112.2|protein_coding|1/1|c.3G>A|p.Met1?|241/1445|3/1131|1/376||,T|downstream_gene_va

00146809|transcript|ENST00000447789.5|protein_coding|7/7|c.721delG|p.Asp241fs|763/765|721/7

0223026.8|protein_coding|3/5|c.1A>G|p.Met1?|639/2407|1/1446|1/481||,G|start_lost|HIGH|HYAL

00487564.1|protein_coding|2/2|c.201T>G|p.Tyr67*|201/368|201/234|67/77||WARNING_TRANSCRI

00000197462|transcript|ENST00000411856.1|lincRNA|1/4|n.249-1G>A||||||

9603|transcript|ENST00000412160.1|protein_coding|1/1|c.-312+1G>A||||||WARNING_TRANSCRIPT_

378740.6|protein_coding|10/10|c.1022A>G|p.Ter341Trpext*?|1046/1088|1022/1023|341/340||,C|s

nteraction|3ZYI:A_279-A_303:ENST00000249363|protein_coding|2/2|c.837T>A||||||,T|synonymous_

teraction|3OMC:A_688-A_766:ENST00000354725|protein_coding|18/24|c.2064T>C||||||,C|structura

G00000242588|transcript|ENST00000605862.4|processed_transcript|5/9|n.445+2T>C||||||,C|splice_

teraction|3V8O:A_575-A_709:ENST00000325888|protein_coding|14/48|c.2125G>A||||||,A|structura

504|transcript|ENST00000249375.8|protein_coding|1/8|c.-12+2T>G||||||,G|upstream_gene_variant
riant&intron_variant|HIGH|IRF5|ENSG00000128604|transcript|ENST00000619830.1|protein_coding|7
interaction|4C0P:B_253-B_305:ENST00000265388|protein_coding|6/23|c.759C>G||||||,C|synonymo
nscript|ENST00000467614.2|protein_coding|1/2|c.230_233delTGGA|p.Leu77fs|230/1263|230/1218|
IGH|AC009518.2|ENSG00000233263|transcript|ENST00000418056.1|processed_pseudogene|1/1|n.2
IGH|AC009518.2|ENSG00000233263|transcript|ENST00000418056.1|processed_pseudogene|1/1|n.2
IGH|AC009518.2|ENSG00000233263|transcript|ENST00000418056.1|processed_pseudogene|1/1|n.2
00000229532|transcript|ENST00000433754.1|processed_pseudogene|1/2|n.115-2A>G|||||
59|transcript|ENST00000275767.3|protein_coding|2/2|c.-1_1delGA|p.Met1fs|223/1997|1/558|1/185
000230649|transcript|ENST00000440744.2|lincRNA|2/3|n.134+2T>C|||||
|interaction|2PQF:B_552-B_626:ENST00000263549|protein_coding|11/12|c.1656C>T||||||,A|structu
000133597|transcript|ENST00000476491.5|protein_coding|7/7|c.1741-3dupT||||||INFO_REALIGN_3
000133597|transcript|ENST00000476491.5|protein_coding|7/7|c.1741-3dupT||||||INFO_REALIGN_3
interaction|2YHF:F_126-F_163:ENST00000546910|protein_coding|7/7|c.489C>A||||||,T|structural_i
000343257.6|protein_coding|23/23|c.2680C>T|p.Arg894*|2767/3172|2680/2967|894/988||,T|dowr
t|ENST00000439431.2|polymorphic_pseudogene|1/1|c.920delA|p.Gln307fs|920/979|920/979|307/3
t|ENST00000439431.2|polymorphic_pseudogene|1/1|c.267delG|p.Pro90fs|267/979|267/979|89/325
NST00000444908.6|protein_coding|3/7|c.1257_1258delTC|p.Gln420fs|1342/2976|1257/2538|419/8
HIGH|ARHGEF5|ENSG00000050327|transcript|ENST00000056217.9|protein_coding|2/15|c.7_28dupG
NST00000482940.5|nonsense_mediated_decay|5/12|c.169dupA|p.Arg57fs|377/2815|169/240|57/79
00538212.6|protein_coding|3/7|c.71C>A|p.Ser24*|142/2243|71/570|24/189||,T|synonymous_varia
511|transcript|ENST00000538212.6|protein_coding|1/6|c.-22+1G>A||||||,T|intron_variant|MODIFIE
96511|transcript|ENST00000482940.5|nonsense_mediated_decay|1/11|c.-121-1G>A||||||,T|splice_
teraction|3S4Y:A_33-A_59:ENST00000360057|protein_coding|3/9|c.98G>A||||||,T|structural_intera
000307003.2|protein_coding|1/3|c.3G>T|p.Met1?|364/1354|3/534|1/177||
on_coding_transcript_exon_variant|HIGH|RP4-800G7.3|ENSG00000271664|transcript|ENST00000605
on_coding_transcript_exon_variant|HIGH|RP4-800G7.3|ENSG00000271664|transcript|ENST00000605
on_coding_transcript_exon_variant|HIGH|RP4-800G7.3|ENSG00000271664|transcript|ENST00000605
on_coding_transcript_exon_variant|HIGH|RP4-800G7.3|ENSG00000271664|transcript|ENST00000605
on_coding_transcript_exon_variant|HIGH|RP4-800G7.3|ENSG00000271664|transcript|ENST00000605
on_coding_transcript_exon_variant|HIGH|RP4-800G7.3|ENSG00000271664|transcript|ENST00000605
00133619|transcript|ENST00000496259.5|protein_coding|7/16|c.875-3dupT||||||INFO_REALIGN_3_
ST00000378016.4|protein_coding|7/107|c.889delG|p.Val297fs|889/15589|889/15453|297/5150||IN
7558|transcript|ENST00000378016.4|protein_coding|31/106|c.4924+1T>G||||||,G|non_coding_trans
0197558|transcript|ENST00000378016.4|protein_coding|59/107|c.8750_8753delTAGA|p.Ile2917fs|8
HIGH|SSPO|ENSG00000197558|transcript|ENST00000378016.4|protein_coding|62/106|c.9207dupC||
3|transcript|ENST00000378016.4|protein_coding|79/107|c.11582dupA|p.Tyr3861fs|11583/15589|11
ST00000378016.4|protein_coding|86/107|c.12610delC|p.Gln4204fs|12610/15589|12610/15453|420
197558|transcript|ENST00000378016.4|protein_coding|95/106|c.14003-1T>G||||||,G|splice_region_
00378016.4|protein_coding|105/107|c.15187C>T|p.Arg5063*|15187/15589|15187/15453|5063/515
197558|transcript|ENST00000465639.5|retained_intron|9/9|n.3307-2A>G||||||,G|splice_acceptor_v
00000478393.5|protein_coding|4/6|c.355C>T|p.Gln119*|355/5697|355/627|119/208||WARNING_TR
|interaction|2XTM:A_23-A_74:ENST00000223293|protein_coding|3/3|c.220G>T||||||,T|structural_ir
|interaction|2XTM:A_63-A_101:ENST00000223293|protein_coding|3/3|c.303G>C||||||,C|structural_
teraction|3HI7:A_321-A_342:ENST00000360937|protein_coding|2/5|c.1026C>T||||||,T|structural_in
|ENST00000397238.6|protein_coding|1/18|c.73dupG|p.Ala25fs|74/3225|74/2736|25/911||INFO_RE
|ENST00000397238.6|protein_coding|1/18|c.81dupG|p.Gln28fs|82/3225|82/2736|28/911||INFO_RE

ENST00000469901.5|protein_coding|1/6|c.113delC|p.Ala38fs|115/582|113/580|38/192||WARNING_
ENST00000615129.4|protein_coding|11/19|c.1375delG|p.Glu459fs|1417/2606|1375/2457|459/818||
nt&intron_variant|HIGH|NUB1|ENSG00000013374|transcript|ENST00000468404.5|nonsense_mediate
)106648|transcript|ENST00000448366.6|nonsense_mediated_decay|2/7|c.-40+1A>G|||||,G|splice_
)178234|transcript|ENST00000419245.5|protein_coding|1/3|c.-39+2T>G|||||WARNING_TRANSCRIP
nteraction|1XFD:C_208-C_241:ENST00000377770|protein_coding|7/26|c.723A>G|||||,G|synonymou
teraction|1XFD:A_315-A_357:ENST00000377770|protein_coding|9/26|c.945C>T|||||,T|structural_ir
teraction|1XFD:A_626-A_709:ENST00000377770|protein_coding|21/26|c.2127T>C|||||,C|structural
teraction|1XFD:A_791-A_822:ENST00000377770|protein_coding|24/26|c.2373C>T|||||,T|structural_
)5982|transcript|ENST00000317955.9|protein_coding|8/8|c.852+2T>C|||||,C|splice_donor_variant&
)00224629&ENSG00000009335|gene_variant|ENSG00000224629|||n.157263275_157263221delCGA(
)0000231980|transcript|ENST00000436489.1|antisense|1/1|n.104+1G>C|||||,C|intron_variant|MOD
.26870|transcript|ENST00000397143.3|protein_coding|1/2|c.-124+1G>A|||||WARNING_TRANSCRIP
)0523415.5|nonsense_mediated_decay|3/4|c.370T>C|p.Ter124Glnext*?|371/3630|370/372|124/123
GH|CTD-2281E23.3|ENSG00000254160|transcript|ENST00000517950.1|lincRNA|2/2|n.377-47_377-1(
G00000253853|transcript|ENST00000520024.1|lincRNA|4/4|n.522-2A>G|||||
)|interaction|1Z3S:A_283-A_369:ENST00000325203|protein_coding|7/9|c.1107T>C|||||,G|structura
)|interaction|1Z3S:A_365-A_392:ENST00000325203|protein_coding|7/9|c.1095T>C|||||,G|structura
000233295|transcript|ENST00000528221.1|unprocessed_pseudogene|3/3|n.432+2T>C|||||
000434307.6|protein_coding|4/4|c.310T>G|p.Ter104Gluext*?|477/777|310/312|104/103||,G|stop_
)249188|transcript|ENST00000509557.1|unprocessed_pseudogene|2/2|n.374+2C>T|||||,T|intron_v
)0000173295|transcript|ENST00000522601.5|processed_transcript|2/8|n.170-1G>A|||||,A|splice_ac
526|transcript|ENST00000522612.1|processed_transcript|3/3|n.280+1G>A|||||,A|intron_variant|M
SG00000253735|transcript|ENST00000521143.1|lincRNA|2/2|n.252-2A>C|||||,G|intron_variant|M(
00000254237|transcript|ENST00000521842.1|lincRNA|2/3|n.165+1G>T|||||,A|intron_variant|MOD
)0000254237|transcript|ENST00000522129.5|lincRNA|1/2|n.174+1G>A|||||
NSG00000249050|transcript|ENST00000507315.1|processed_pseudogene|1/3|n.261-2A>G|||||
NSG00000249050|transcript|ENST00000507315.1|processed_pseudogene|1/3|n.261-1A>G|||||
3273|transcript|ENST00000521039.5|retained_intron|1/5|n.78+1G>A|||||,A|intron_variant|MODIFI
ENST00000522210.1|protein_coding|5/5|c.804delG|p.Ser269fs|844/1005|804/831|268/276||INFO_R
transcript|ENST00000382483.3|protein_coding|4/4|c.3956_3957insAAGAAGAGAG|p.Val1320fs|4180/
ant|HIGH|RP1L1|ENSG00000183638|transcript|ENST00000382483.3|protein_coding|4/4|c.3955_3956
nt|HIGH|RP1L1|ENSG00000183638|transcript|ENST00000382483.3|protein_coding|4/4|c.3955_3956
254093|transcript|ENST00000517607.1|processed_transcript|3/3|n.266-1G>T|||||,A|downstream_
54316|transcript|ENST00000525246.5|processed_transcript|4/8|n.385-2A>G|||||,G|splice_acceptor
nteraction|1EZF:A_44-A_67:ENST00000220584|protein_coding|3/8|c.201C>T|||||,T|structural_inter
nteraction|1EZF:A_200-A_279:ENST00000220584|protein_coding|5/8|c.600T>C|||||,C|structural_in
nteraction|3WCH:A_211-A_293:ENST00000220584|protein_coding|5/8|c.631T>C|||||,C|structural_i
)0000205879|transcript|ENST00000511144.2|unprocessed_pseudogene|1/5|n.118-1G>A|||||,T|ups
t|ENST00000533852.6|protein_coding|6/8|c.650_653delACAG|p.Asp217fs|653/2043|650/993|217/3
t|ENST00000262365.8|protein_coding|6/8|c.650_653delACAG|p.Asp217fs|653/1268|650/993|217/3
IGH|RP11-369K16.1|ENSG00000251468|transcript|ENST00000506173.2|unprocessed_pseudogene|2/
teraction|3KUQ:A_1167-A_1265:ENST00000276297|protein_coding|12/18|c.3501G>A|||||,T|synony
3945|transcript|ENST00000445506.6|protein_coding|1/9|c.50+1G>A|||||,T|intron_variant|MODIFIE
ENST00000517398.1|protein_coding|4/4|c.261_262insA|p.Glu88fs|263/439|262/300|88/99||WARN
I|CNOT7|ENSG00000198791|transcript|ENST00000524358.5|protein_coding|1/4|c.-171+2_-171+6del
I|CNOT7|ENSG00000198791|transcript|ENST00000524358.5|protein_coding|1/4|c.-171+2_-171+6del

ENST00000520140.5|nonsense_mediated_decay|7/12|c.804_805delTA|p.Thr269fs|1115/2102|804/9!
.04760|transcript|ENST00000381841.4|protein_coding|1/8|c.-144-1G>C||||||,G|splice_acceptor_var
i00000254054|transcript|ENST00000519038.2|lincRNA|1/2|n.100+1G>A||||||,A|intron_variant|MOI
ained|HIGH|ASAH1|ENSG00000104763|transcript|ENST00000637561.1|protein_coding|5/5|c.473_5C
NST00000637561.1|protein_coding|5/5|c.414delA|p.Val139fs|504/929|414/528|138/175||,C|frame:
3|transcript|ENST00000520051.2|nonsense_mediated_decay|2/3|c.217T>G|p.Ter73Gluext*?|360/66
171428|transcript|ENST00000541942.1|protein_coding|1/3|c.-235-1G>C||||||,C|splice_acceptor_var
iteraction|2IJA:A_163-A_187:ENST00000307719|protein_coding|3/3|c.560G>A||||||,A|structural_int
ISG00000278886|gene_variant|ENSG00000278886|||n.18865250_18865225delTATATATATATATATAT
ISG00000278886|gene_variant|ENSG00000278886|||n.18865250_18865225delTATATATATATATATAT
ISG00000278886|gene_variant|ENSG00000278886|||n.18865250_18865225delTATATATATATATATAT
ISG00000278886|gene_variant|ENSG00000278886|||n.18865250_18865225delTATATATATATATATAT
ISG00000278886|gene_variant|ENSG00000278886|||n.18865289_18865241delAAATATATATATATATA
ISG00000278886|gene_variant|ENSG00000278886|||n.18865289_18865241delAAATATATATATATATA
ISG00000278886|gene_variant|ENSG00000278886|||n.18865293_18865247delAAAAAAATATATATAT
ISG00000278886|gene_variant|ENSG00000278886|||n.18865293_18865247delAAAAAAATATATATAT
ISG00000278886|gene_variant|ENSG00000278886|||n.18865293_18865247delAAAAAAATATATATAT
ISG00000278886|gene_variant|ENSG00000278886|||n.18865293_18865247delAAAAAAATATATATAT
0000147408|transcript|ENST00000523227.5|processed_transcript|1/2|n.313+2T>C||||||,G|upstrean
0311322.8|protein_coding|9/10|c.1421C>G|p.Ser474*|1891/3846|1421/1428|474/475||
0000147416|transcript|ENST00000520830.1|nonsense_mediated_decay|1/6|c.119-1G>C|||||WARN
i00000253147|transcript|ENST00000523852.5|lincRNA|3/3|n.759+1A>G||||||,G|splice_donor_variar
0517699.2|nonsense_mediated_decay|3/6|c.151C>T|p.Arg51*|152/772|151/162|51/53||WARNING_
000471755.5|nonsense_mediated_decay|14/16|c.1773T>G|p.Tyr591*|1779/2695|1773/1869|591/62
120913|transcript|ENST00000452226.5|protein_coding|1/7|c.-3+2T>G|||||WARNING_TRANSCRIPT_
NST00000256404.7|protein_coding|7/7|c.659delA|p.Asn220fs|751/895|659/684|220/227||,G|intron
00104679|transcript|ENST00000625275.2|protein_coding|4/8|c.309-1G>A||||||,A|missense_variant
0000134013|transcript|ENST00000520871.1|protein_coding|1/2|c.40+1_40+2insAATAC|||||WARNIN
ST00000290271.6|protein_coding|4/4|c.693_700delTGAGGAGG|p.Glu232fs|984/3883|693/744|231/
ST00000290271.6|protein_coding|4/4|c.693_697delTGAGG|p.Glu232fs|981/3883|693/744|231/247|
ST00000524323.1|protein_coding|4/4|c.486_490delTGAGG|p.Glu163fs|845/1748|486/537|162/178|
000228451|transcript|ENST00000519902.1|processed_transcript|1/1|n.567-1G>T||||||,A|upstream_
interaction|2GSE:A_110-A_142:ENST00000311151|protein_coding|4/14|c.426T>C||||||,C|structural_
interaction|2GSE:B_159-B_468:ENST00000311151|protein_coding|4/14|c.477C>T||||||,T|structural_
interaction|3I1Y:A_55-A_162:ENST00000521400|protein_coding|2/19|c.164A>G||||||,G|structural_ir
nteraction|5AIA:A_142-A_283:ENST00000521400|protein_coding|8/19|c.849G>A||||||,A|structural_
nteraction|1S8O:A_254-A_287:ENST00000521400|protein_coding|8/19|c.860G>A||||||,A|structural_
15|interaction|1S8O:A_403-A_432:ENST00000521400|protein_coding|13/19|c.1206_1208dupTCG|||
nteraction|3ANS:B_412-B_495:ENST00000521400|protein_coding|13/19|c.1236C>T||||||,T|structura
78|transcript|ENST00000521226.2|protein_coding|2/5|c.-45+2T>G|||||WARNING_TRANSCRIPT_INC
ISG00000254370|transcript|ENST00000522589.1|sense_intronic|1/1|n.166-2A>G||||||,C|upstream_
2232|transcript|ENST00000454906.6|processed_transcript|1/3|n.119+1G>A||||||,A|intron_variant|I
iG00000253604|transcript|ENST00000521908.1|lincRNA|1/1|n.128-2A>G|||||
0133872|transcript|ENST00000256255.10|protein_coding|2/5|c.283-2A>T||||||,A|splice_acceptor_va
ENST00000522968.1|protein_coding|1/2|c.32delG|p.Gly11fs|100/813|32/745|11/247||WARNING_TR
NST00000256246.4|protein_coding|1/4|c.5699_5700delGA|p.Arg1900fs|5775/10187|5699/8370|190
|interaction|4B04:A_119-A_158:ENST00000256261|protein_coding|3/4|c.357C>T||||||,A|structural_

ENSG00000253642|transcript|ENST00000523336.1|lincRNA|2/2|n.139-3dupT||||||INFO_REALIGN_3
ENSG00000253642|transcript|ENST00000523336.1|lincRNA|2/2|n.139-3dupT||||||INFO_REALIGN_3
ENST00000331569.5|protein_coding|2/2|c.1579_1583delGGCAG|p.Gly527fs|1808/3349|1579/1773|5
00000428068.5|protein_coding|1/6|c.72C>G|p.Tyr24*|72/571|72/571|24/189||WARNING_TRANSCR
00437935.2|protein_coding|2/2|c.422G>C|p.Ter141Serext*?|442/1527|422/423|141/140||,G|misse
00169499|transcript|ENST00000617275.4|protein_coding|1/11|c.-24+1dupG||||||INFO_REALIGN_3_
0197475|transcript|ENST00000490268.6|processed_transcript|5/21|n.485+1G>A||||||,T|splice_dono
00389060.8|protein_coding|10/10|c.1038T>A|p.Tyr346*|1038/1227|1038/1224|346/407||,A|stop_g
nteraction|3UD1:A_1109-A_1133:ENST00000289734|protein_coding|29/43|c.3399C>T||||||,A|structu
teraction|1N11:A_563-A_594:ENST00000289734|protein_coding|16/43|c.1782C>A||||||,T|structura
000331373.9|protein_coding|4/5|c.136C>T|p.Arg46*|391/1865|136/1053|46/350||,T|stop_gained|H
0000147481|transcript|ENST00000520697.1|processed_transcript|1/1|n.97-3dupT||||||INFO_REALIG
0000147481|transcript|ENST00000520697.1|processed_transcript|1/1|n.97-3dupT||||||INFO_REALIG
0000147481|transcript|ENST00000520697.1|processed_transcript|1/1|n.97-3dupT||||||INFO_REALIG
000356297.4|protein_coding|19/23|c.3774T>A|p.Cys1258*|3875/4805|3774/4392|1258/1463||,T|s
0000254314|transcript|ENST00000520496.1|antisense|1/1|n.151+2T>C||||||,C|intron_variant|MOD
nt&intron_variant|HIGH|TMEM68|ENSG00000167904|transcript|ENST00000617977.1|protein_coding
NSG00000253281|transcript|ENST00000519981.5|lincRNA|1/7|n.233-1G>T||||||,A|intron_variant|M
35681|transcript|ENST00000518229.5|nonsense_mediated_decay|3/7|c.*107+1G>A||||||,T|intron_
00000253879|transcript|ENST00000521274.1|lincRNA|2/2|n.120+1G>C||||||
eraction|2W2J:A_109-A_147:ENST00000317995|protein_coding|3/9|c.327A>G||||||,C|structural_int
7182|transcript|ENST00000518858.1|processed_transcript|1/1|n.1234+2T>C||||||,C|intron_variant|
0000280725|transcript|ENST00000627569.1|lincRNA|2/3|n.252-1G>A||||||
ENST00000262146.8|protein_coding|6/8|c.581_582delTG|p.Leu194fs|707/2640|581/1002|194/333||
ENST00000519561.5|protein_coding|2/3|c.82delG|p.Glu28fs|233/1835|82/480|28/159||INFO_REAL
ENST00000518698.5|protein_coding|1/14|c.59_72delGGCTCTCAGCGCCC|p.Arg20fs|350/2223|59/161
000247570|transcript|ENST00000424643.3|processed_pseudogene|2/2|n.814-2G>A||||||
0104321|transcript|ENST00000523582.5|protein_coding|23/23|c.2706-1G>A||||||WARNING_TRANS
iant&intron_variant|HIGH|RP11-1145L24.1|ENSG00000253784|transcript|ENST00000523881.1|lincRN
ENST00000521419.5|protein_coding|6/7|c.351_352insC|p.Ser118fs|658/972|351/378|117/125||,AG
00104343|transcript|ENST00000419880.7|protein_coding|6/6|c.563-2delA||||||,C|splice_acceptor_v
000254349|transcript|ENST00000518190.4|lincRNA|4/5|n.379+2T>C||||||,C|intron_variant|MODIFI
I|ZC2HC1A|ENSG00000104427|transcript|ENST00000263849.8|protein_coding|3/8|c.211-6_211-3dup
I|ZC2HC1A|ENSG00000104427|transcript|ENST00000263849.8|protein_coding|3/8|c.211-6_211-3dup
anscript|ENST00000523976.1|protein_coding|1/2|c.-2_5delACATGCC|p.Met1fs|217/1325|1/645|1/21
transcript|ENST00000379091.8|protein_coding|1/6|c.1delA|p.Met1fs|166/2181|1/1740|1/579||INF
00000253374|transcript|ENST00000523380.5|antisense|2/3|n.322+1G>A||||||,A|splice_donor_varia
_coding_transcript_exon_variant|HIGH|RP11-99H20.1|ENSG00000254123|transcript|ENST0000052098
NSG00000253836|transcript|ENST00000522123.1|lincRNA|5/5|n.483-2A>G||||||
SG00000253362|transcript|ENST00000518452.1|unprocessed_pseudogene|2/3|n.146-2delA||||||,CT
SG00000253362|transcript|ENST00000518452.1|unprocessed_pseudogene|2/3|n.146-2delA||||||,CT
SG00000253362|transcript|ENST00000518452.1|unprocessed_pseudogene|2/3|n.146-2delA||||||,CT
SG00000253362|transcript|ENST00000518452.1|unprocessed_pseudogene|2/3|n.146-2delA||||||,CT
00184672|transcript|ENST00000517638.5|protein_coding|7/8|c.725-3dupC||||||INFO_REALIGN_3_F
00184672|transcript|ENST00000517638.5|protein_coding|7/8|c.725-3dupC||||||INFO_REALIGN_3_F
000133739|transcript|ENST00000360375.7|protein_coding|2/18|c.311-3dupT||||||INFO_REALIGN_3
000133739|transcript|ENST00000360375.7|protein_coding|2/18|c.311-3dupT||||||INFO_REALIGN_3

.76731|transcript|ENST00000612977.4|protein_coding|2/4|c.-4+2T>A||||||,T|splice_donor_variant&
teraction|3DA2:A_57-A_178:ENST00000321764|protein_coding|6/7|c.532A>G||||||,G|missense_var
'42|transcript|ENST00000522662.5|protein_coding|2/4|c.-25+1G>A||||||WARNING_TRANSCRIPT_NC
eraction|4RFD:A_188-A_211:ENST00000285379|protein_coding|6/7|c.562T>C||||||,C|structural_inte
|transcript|ENST00000297524.7|protein_coding|4/4|c.1413_*7delGATGTCGG|p.Ter471fs|1524/1878
164893|transcript|ENST00000419776.2|protein_coding|1/4|c.658+2C>T||||||,A|synonymous_varian
IGH|CPNE3|ENSG00000085719|transcript|ENST00000620393.4|protein_coding|3/7|c.-131-2_-126del
SG00000254115|transcript|ENST00000519041.1|antisense|1/2|n.449-2A>G||||||
04325|transcript|ENST00000522161.5|protein_coding|3/11|c.42+2T>A||||||,A|upstream_gene_varia
0000253799|transcript|ENST00000517505.1|lincRNA|1/2|n.299-1G>A||||||,T|intron_variant|MODIF
0000214954|transcript|ENST00000520099.5|nonsense_mediated_decay|5/10|c.*573-3dupT||||||INF
0000214954|transcript|ENST00000520099.5|nonsense_mediated_decay|5/10|c.*573-3dupT||||||INF
.14954|transcript|ENST00000520099.5|nonsense_mediated_decay|9/10|c.*1122+2T>A||||||,A|splice
00000164953|transcript|ENST00000453321.7|protein_coding|1/27|c.224-3dupT||||||INFO_REALIGN_
00000164953|transcript|ENST00000453321.7|protein_coding|1/27|c.224-3dupT||||||INFO_REALIGN_
00000164953|transcript|ENST00000453321.7|protein_coding|1/27|c.224-3dupT||||||INFO_REALIGN_
000164953|transcript|ENST00000455946.5|nonsense_mediated_decay|3/6|c.407-2_407-1delAG|||||
0164941|transcript|ENST00000343161.8|nonsense_mediated_decay|21/28|c.*190-3dupT||||||INFO_
00000104341|transcript|ENST00000445593.6|protein_coding|1/7|c.207_222dupGCTCCAGGCGAGGCG
000104341|transcript|ENST00000517924.5|protein_coding|2/4|c.212-1G>A||||||WARNING_TRANSC
.32561|transcript|ENST00000519582.5|protein_coding|5/5|c.501+1G>A||||||WARNING_TRANSCRIPT
00522135.5|protein_coding|3/3|c.392A>C|p.Ter131Serext*?|394/416|392/393|131/130||WARNING_
04361|transcript|ENST00000341166.3|protein_coding|11/11|c.1067+1G>A||||||,T|upstream_gene_v
0000441350.2|protein_coding|8/8|c.1239T>G|p.Tyr413*|1342/1626|1239/1248|413/415||,G|down
0000132549|transcript|ENST00000358544.6|protein_coding|51/61|c.9406-3dupT||||||INFO_REALIGN
0000132549|transcript|ENST00000358544.6|protein_coding|51/61|c.9406-3dupT||||||INFO_REALIGN
0000132549|transcript|ENST00000358544.6|protein_coding|51/61|c.9406-3dupT||||||INFO_REALIGN
0000132549|transcript|ENST00000358544.6|protein_coding|51/61|c.9406-3dupT||||||INFO_REALIGN
0000132549|transcript|ENST00000358544.6|protein_coding|51/61|c.9406-3dupT||||||INFO_REALIGN
0000132549|transcript|ENST00000358544.6|protein_coding|51/61|c.9406-3dupT||||||INFO_REALIGN
0164919|transcript|ENST00000297564.6|protein_coding|1/3|c.-26-2A>G||||||,C|splice_acceptor_var
iant&intron_variant|HIGH|PABPC1|ENSG00000070756|transcript|ENST00000318607.9|protein_codin
1|ENSG00000070756|transcript|ENST00000318607.9|protein_coding|9/14|c.1337-1_1337insACCTCAT
1|ENSG00000070756|transcript|ENST00000318607.9|protein_coding|9/14|c.1337-1_1337insACCTCAT
1|ENSG00000070756|transcript|ENST00000318607.9|protein_coding|9/14|c.1337-1_1337insACCTCAT
1|ENSG00000070756|transcript|ENST00000318607.9|protein_coding|9/14|c.1337-1_1337insACCTCAT
ENST00000318607.9|protein_coding|8/15|c.999delA|p.Phe335fs|2128/3485|999/1911|333/636||,C
|ENST00000318607.9|protein_coding|3/15|c.467_468insG|p.Met158fs|1596/3485|467/1911|156/63
|interaction|1CVJ:G_100-G_144:ENST00000318607|protein_coding|3/15|c.431A>G||||||,C|structura
G00000070756|transcript|ENST00000318607.9|protein_coding|2/14|c.388-1_388insTAAA||||||,CTTT
interaction|1QJA:B_59-B_87:ENST00000395958|protein_coding|2/6|c.261G>A||||||,T|structural_int
000248599|transcript|ENST00000514926.1|lincRNA|1/2|n.784-1G>C||||||
|ENST00000522368.5|protein_coding|1/9|c.163dupC|p.Arg55fs|164/1613|163/1224|55/407||WARN
NST00000285402.3|protein_coding|2/2|c.665_672delGCCCCTGC|p.Ser222fs|821/1013|665/753|222/2
NST00000285402.3|protein_coding|2/2|c.674_683delACCCGTGCAG|p.Asn225fs|830/1013|674/753|22
0000253320|transcript|ENST00000518518.5|antisense|2/2|n.210-1G>A||||||,A|intron_variant|MOD
_coding_transcript_exon_variant|HIGH|MTCO1P4|ENSG00000253556|transcript|ENST00000520516.1

_coding_transcript_exon_variant|HIGH|MTCO1P4|ENSG00000253556|transcript|ENST00000520516.1
523739.5|protein_coding|3/8|c.1A>G|p.Met1?|265/2774|1/2025|1/674||,G|start_lost|HIGH|FZD6|E
00520337.1|protein_coding|1/4|c.1A>G|p.Met1?|120/1198|1/690|1/229||,G|upstream_gene_varia
164830|transcript|ENST00000517566.6|protein_coding|5/15|c.526-2delA|||||INFO_REALIGN_3_PRI
00449762.6|protein_coding|1/6|c.15G>A|p.Trp5*|247/2621|15/651|5/216||,A|stop_gained|HIGH|(
205038|transcript|ENST00000378402.9|protein_coding|47/77|c.7246+1G>C|||||
G00000205038|transcript|ENST00000378402.9|protein_coding|75/77|c.12331-3dupT|||||INFO_REA
G00000205038|transcript|ENST00000378402.9|protein_coding|75/77|c.12331-3dupT|||||INFO_REA
G00000205038|transcript|ENST00000378402.9|protein_coding|75/77|c.12331-3dupT|||||INFO_REA
0156787|transcript|ENST00000287380.5|protein_coding|19/21|c.2835+1G>T|||||,T|splice_donor_va
H|TMEM65|ENSG00000164983|transcript|ENST00000297632.7|protein_coding|1/6|c.304+2_304+4de
|interaction|2XIO:A_113-A_149:ENST00000276692|protein_coding|5/12|c.339C>T|||||,A|synonym
00000522532.5|protein_coding|4/4|c.511C>T|p.Arg171*|558/1319|511/666|171/221||,T|upstream_
ENSG00000253111|transcript|ENST00000521991.1|lincRNA|1/1|n.240-3dupT|||||INFO_REALIGN_3
ENSG00000253111|transcript|ENST00000521991.1|lincRNA|1/1|n.240-3dupT|||||INFO_REALIGN_3
ENSG00000253111|transcript|ENST00000521991.1|lincRNA|1/1|n.240-3dupT|||||INFO_REALIGN_3
229140|transcript|ENST00000446592.7|lincRNA|3/3|n.431+1A>G|||||,C|splice_donor_variant&intro
0000224110|transcript|ENST00000506027.1|processed_pseudogene|2/2|n.786-1A>C|||||
00000253507|transcript|ENST00000519695.5|lincRNA|3/4|n.480+1G>T|||||,T|splice_donor_variant
SG00000253507|transcript|ENST00000524275.5|lincRNA|3/4|n.462-2A>G|||||,G|splice_acceptor_v
0517423.5|protein_coding|3/3|c.466T>C|p.Ter156Glnext*?|527/716|466/468|156/155||,C|synonym
827|transcript|ENST00000377838.7|protein_coding|1/15|c.19+2T>G|||||,C|splice_donor_variant&i
0066827|transcript|ENST00000377838.7|protein_coding|1/16|c.17_18delCA|p.Ala6fs|193/4597|17/3
G00000254101|transcript|ENST00000521034.1|lincRNA|2/3|n.193-2A>G|||||
nteraction|3LUC:A_442-A_481:ENST00000519980|protein_coding|12/18|c.1443C>T|||||,A|structura
000519291.5|protein_coding|2/4|c.2T>C|p.Met1?|188/560|2/374|1/123||WARNING_TRANSCRIPT_IN
on_coding_transcript_exon_variant|HIGH|RP11-10J21.4|ENSG00000253307|transcript|ENST0000051
0226807|transcript|ENST00000430863.5|polymorphic_pseudogene|3/29|c.409-1G>A|||||,T|splice_
on_coding_transcript_exon_variant|HIGH|JRK|ENSG00000234616|transcript|ENST00000591357.5|pro
RK|ENSG00000234616|transcript|ENST00000591357.5|processed_transcript|1/2|n.265-2_265-1insCC
4616|transcript|ENST00000591357.5|processed_transcript|1/2|n.265-2A>C|||||,TGGAGGAGAGGAG
4616|transcript|ENST00000591357.5|processed_transcript|1/2|n.265-2A>C|||||,TGGAGGAGAGGAG
00301258.4|protein_coding|3/3|c.326G>A|p.Trp109*|409/1020|326/345|109/114||,A|upstream_ge
2|interaction|4DVQ:A_382-A_405:ENST00000323110|protein_coding|7/9|c.1144T>C|||||,G|structu
2|interaction|4DVQ:A_374-A_411:ENST00000323110|protein_coding|6/9|c.1120C>A|||||,T|structur
2|interaction|4DVQ:E_173-E_470:ENST00000323110|protein_coding|3/9|c.518A>G|||||,C|structura
IST00000522452.1|protein_coding|2/4|c.2T>C|p.Met1?|793/2897|2/600|1/199||,C|start_lost|HIGH|
pt|ENST00000522121.5|nonsense_mediated_decay|2/4|c.106dupC|p.Leu36fs|106/874|106/150|36/4
ENST00000262577.5|protein_coding|11/12|c.2800dupC|p.Leu934fs|2832/3280|2800/2847|934/948
000262577.5|protein_coding|11/12|c.2639C>A|p.Ser880*|2671/3280|2639/2847|880/948||
teraction|4Q59:A_71-A_126:ENST00000345136|protein_coding|5/32|c.378T>C|||||,G|structural_in
5|interaction|4EMO:D_52-D_75:ENST00000398712|protein_coding|1/9|c.154C>A|||||,T|structural_
5|interaction|4EMO:D_17-D_50:ENST00000398712|protein_coding|1/9|c.50C>T|||||,A|structural_ir
0185122|transcript|ENST00000528988.1|nonsense_mediated_decay|2/4|c.*60-3dupT|||||INFO_RE
0185122|transcript|ENST00000528988.1|nonsense_mediated_decay|2/4|c.*60-3dupT|||||INFO_RE
IST00000301332.2|protein_coding|17/17|c.2375_2376delGT|p.Arg792fs|2752/3646|2375/2517|792/
IST00000528130.1|protein_coding|4/4|c.302delC|p.Pro101fs|348/1134|302/405|101/134||INFO_RE

)0382500.3|protein_coding|1/1|c.1320G>C|p.Ter440Tyrext*?|1405/1974|1320/1320|440/439||,G|up
t|ENST00000382500.3|protein_coding|1/1|c.753_754insCG|p.Gly252fs|838/1974|753/1320|251/439
ENST00000382500.3|protein_coding|1/1|c.748_749delGG|p.Gly250fs|834/1974|748/1320|250/439||
SG00000227155|transcript|ENST00000585944.1|antisense|1/1|n.789-2A>G||||||,C|splice_acceptor_
_coding_transcript_exon_variant|HIGH|RP11-264I13.2|ENSG00000236199|transcript|ENST000004268
_coding_transcript_exon_variant|HIGH|RP11-264I13.2|ENSG00000236199|transcript|ENST000004268
_coding_transcript_exon_variant|HIGH|RP11-264I13.2|ENSG00000236199|transcript|ENST000004268
_coding_transcript_exon_variant|HIGH|RP11-264I13.2|ENSG00000236199|transcript|ENST000004268
_coding_transcript_exon_variant|HIGH|RP11-264I13.2|ENSG00000236199|transcript|ENST000004268
_coding_transcript_exon_variant|HIGH|RP11-264I13.2|ENSG00000236199|transcript|ENST000004268
GH|AL365202.1|ENSG00000279745|transcript|ENST00000624522.1|protein_coding|1/2|c.9-3_9-2del
GH|AL365202.1|ENSG00000279745|transcript|ENST00000624522.1|protein_coding|1/2|c.9-3_9-2del
GH|AL365202.1|ENSG00000279745|transcript|ENST00000624522.1|protein_coding|1/2|c.9-3_9-2del
000232104|transcript|ENST00000457566.1|lincRNA|1/1|n.324-1G>C||||||,C|splice_acceptor_variant
GH|HNRNPA1P41|ENSG00000220937|transcript|ENST00000397777.3|processed_pseudogene|1/1|n.
00000220937|transcript|ENST00000397777.3|processed_pseudogene|1/1|n.248-13_248-3delAAAAA
20937|transcript|ENST00000397777.3|processed_pseudogene|1/1|n.248-13_248-11delAAA||||||
220937|transcript|ENST00000397777.3|processed_pseudogene|1/1|n.248-13_248-12delAA||||||
)00381641.3|protein_coding|2/2|c.489G>A|p.Trp163*|555/758|489/642|163/213||,T|intron_variant
)000339450.9|protein_coding|1/15|c.240C>A|p.Tyr80*|330/5376|240/2715|80/904||,T|stop_gained
|ENST00000344545.6|protein_coding|1/1|c.417delG|p.Trp139fs|638/2296|417/423|139/140||INFO_
.07077|transcript|ENST00000496464.1|processed_transcript|2/3|n.340+1A>G||||||,G|intron_variant
nteraction|2FCF:A_1203-A_1241:ENST00000541718|protein_coding|25/46|c.3609A>G||||||,C|structu
)000512701.6|protein_coding|4/20|c.428T>A|p.Leu143*|465/10483|428/2049|143/682||,T|stop_ga
000380647.7|protein_coding|7/26|c.1087C>T|p.Gln363*|1171/5576|1087/4221|363/1406||,T|stop_
)044459|transcript|ENST00000380647.7|protein_coding|18/25|c.3115-2delA||||||INFO_REALIGN_3_
ipt|ENST00000380545.9|protein_coding|1/8|c.29delT|p.Phe10fs|138/1562|29/1392|10/463||INFO_
)000357374.2|protein_coding|1/1|c.60T>A|p.Cys20*|106/963|60/570|20/189||,T|upstream_gene_v
|ENST00000413767.2|protein_coding|1/1|c.170dupA|p.His57fs|219/980|170/570|57/189||,AT|dowr
)00610521.1|protein_coding|1/1|c.343C>T|p.Gln115*|399/987|343/570|115/189||
)0448696.4|protein_coding|1/1|c.211C>T|p.Gln71*|830/1475|211/627|71/208||,A|intron_variant|N
nteraction|1CB0:A_12-A_56:ENST00000580900|protein_coding|3/8|c.166G>A||||||,A|structural_inte
nteraction|1CB0:A_98-A_187:ENST00000580900|protein_coding|6/8|c.561C>T||||||,T|structural_inte
_coding_transcript_exon_variant|HIGH|RP11-149I2.4|ENSG00000266446|transcript|ENST0000057893
G00000254396|transcript|ENST00000522960.1|lincRNA|1/1|n.448-2A>T||||||,T|upstream_gene_var
t|ENST00000276943.2|protein_coding|1/2|c.34_37dupTTGT|p.Trp13fs|61/1140|38/624|13/207||INI
nteraction|2YKG:A_510-A_546:ENST00000379883|protein_coding|11/18|c.1637G>A||||||,T|structur
2|interaction|2AE7:A_199-A_386:ENST00000379731|protein_coding|2/6|c.597C>T||||||,A|structural
T00000297625.7|protein_coding|2/2|c.69C>G|p.Tyr23*|193/6398|69/2145|23/714||,C|upstream_ge
0164976|transcript|ENST00000297625.7|protein_coding|1/1|c.-64+2T>G||||||,C|upstream_gene_va
213930|transcript|ENST00000473529.5|retained_intron|4/6|n.644-1G>A||||||,A|3_prime_UTR_varia
t|ENST00000340783.10|protein_coding|4/5|c.355delG|p.Ala119fs|411/983|355/627|119/208||,G|fr
GH|DNAJB5|ENSG00000137094|transcript|ENST00000458263.6|protein_coding|1/2|c.-298-3_-298-2
)0215187|transcript|ENST00000399742.6|protein_coding|5/6|c.719_722dupACTA|p.Tyr241fs|793/95
ENST00000378300.9|protein_coding|9/9|c.909dupC|p.Trp304fs|995/1771|909/969|303/322||,AG|d
SG00000228843|transcript|ENST00000425499.2|antisense|1/1|n.214-2A>G||||||,C|3_prime_UTR_va
SG00000227388|transcript|ENST00000431981.2|lincRNA|2/2|n.196-2A>G||||||,G|upstream_gene_v

)137103|transcript|ENST00000439587.6|protein_coding|1/10|c.-928+2C>T||||||,T|upstream_gene_v
)0424348.1|polymorphic_pseudogene|1/1|c.936A>G|p.Ter312Trpext*?|936/959|936/959|312/318||
)0424348.1|polymorphic_pseudogene|1/1|c.559T>A|p.Ter187Argext*?|559/959|559/959|187/318||
ENST00000424348.1|polymorphic_pseudogene|1/1|c.435_436delAA|p.Ile145fs|436/959|435/959|14
)137124|transcript|ENST00000635162.1|protein_coding|2/2|c.-10+1G>A|||||WARNING_TRANSCRIP
GH|VN2R3P|ENSG00000234680|transcript|ENST00000422626.1|unitary_pseudogene|3/6|n.305-4_30
pt|ENST00000377561.6|protein_coding|12/24|c.1844delT|p.Leu615fs|2237/5379|1844/3867|615/12
pt|ENST00000377561.6|protein_coding|10/24|c.1599delG|p.Leu534fs|1992/5379|1599/3867|533/1
ript|ENST00000357336.3|protein_coding|4/15|c.565_568delAAAG|p.Lys189fs|846/4137|565/2472|1
:|ENST00000377420.1|protein_coding|1/1|c.1215delC|p.Trp406fs|2047/3109|1215/1251|405/416||,
:|ENST00000377420.1|protein_coding|1/1|c.887_888delTC|p.Phe296fs|1720/3109|887/1251|296/41
ENST00000382405.7|protein_coding|14/15|c.965_968delTCAA|p.Ile322fs|1143/1791|965/1188|322/
ript|ENST00000611388.1|protein_coding|6/15|c.775_778delCTTA|p.Leu259fs|890/3964|775/2472|2
ript|ENST00000562196.5|protein_coding|4/15|c.565_568delAAAG|p.Lys189fs|845/3379|565/2472|1
:|ENST00000360171.10|protein_coding|11/15|c.777dupA|p.Leu260fs|1329/2165|778/1188|260/395
_coding_transcript_exon_variant|HIGH|RP11-561O23.5|ENSG00000234394|transcript|ENST0000042
_coding_transcript_exon_variant|HIGH|RP11-561O23.5|ENSG00000234394|transcript|ENST0000042
_coding_transcript_exon_variant|HIGH|RP11-561O23.5|ENSG00000234394|transcript|ENST0000042
eraction|2UZ9:A_20-A_388:ENST00000238018|protein_coding|1/16|c.60C>T||||||,T|structural_inter
interaction|2QG6:A_119-A_168:ENST00000361092|protein_coding|6/9|c.357T>G||||||,C|structural_
NST00000376767.7|protein_coding|14/14|c.1988_1992delGAATG|p.Gly663fs|2500/2845|1988/2073|
)099139|transcript|ENST00000376767.7|protein_coding|14/14|c.2008_2027delAAATGGAATGGAATGG
)099139|transcript|ENST00000376767.7|protein_coding|14/14|c.2008_2027delAAATGGAATGGAATGG
NST00000376767.7|protein_coding|14/14|c.2008_2012delAAATG|p.Lys670fs|2520/2845|2008/2073|
NST00000376767.7|protein_coding|14/14|c.2008_2012delAAATG|p.Lys670fs|2520/2845|2008/2073|
ENST00000376767.7|protein_coding|14/14|c.2007dupG|p.Lys670fs|2520/2845|2008/2073|670/690|
ENST00000376767.7|protein_coding|14/14|c.2007dupG|p.Lys670fs|2520/2845|2008/2073|670/690|
)99139|transcript|ENST00000376767.7|protein_coding|14/14|c.2009_2010insTGGA|p.Lys670fs|2522/
)99139|transcript|ENST00000376767.7|protein_coding|14/14|c.2009_2010insTGGA|p.Lys670fs|2522/
)99139|transcript|ENST00000376767.7|protein_coding|14/14|c.2009_2010insTGGA|p.Lys670fs|2522/
)99139|transcript|ENST00000376767.7|protein_coding|14/14|c.2009_2010insTGGA|p.Lys670fs|2522/
ENST00000376767.7|protein_coding|14/14|c.2026_2027insC|p.Trp676fs|2539/2845|2027/2073|676/
99139|transcript|ENST00000376767.7|protein_coding|14/14|c.2027_2028insAATC|p.Trp676fs|2540/
99139|transcript|ENST00000376767.7|protein_coding|14/14|c.2027_2028insAATC|p.Trp676fs|2540/
cript|ENST00000376767.7|protein_coding|14/14|c.2062_2066dupCGAAT|p.Glu690fs|2579/2845|206
000376767.7|protein_coding|14/14|c.2057C>A|p.Ser686*|2569/2845|2057/2073|686/690||,A|intro
)376767.7|protein_coding|14/14|c.2071T>C|p.Ter691Glnext*?|2583/2845|2071/2073|691/690||,C|i
)376767.7|protein_coding|14/14|c.2072A>C|p.Ter691Serext*?|2584/2845|2072/2073|691/690||,C|i
)0106772|transcript|ENST00000466266.6|processed_transcript|6/6|n.697-2A>G||||||,C|splice_regic
)0419472.1|protein_coding|7/7|c.1149A>T|p.Ter383Cysext*?|1151/2781|1149/1149|383/382||WAR
8019|transcript|ENST00000487108.6|processed_transcript|1/3|n.636+1G>A||||||,A|upstream_gene
nteraction|3E77:A_99-A_122:ENST00000376588|protein_coding|4/9|c.297T>G||||||,G|structural_int
0000226877|transcript|ENST00000458016.5|antisense|2/4|n.132+2C>T||||||,T|upstream_gene_vari
0000231616|transcript|ENST00000615666.4|antisense|4/4|n.475+2T>C||||||,C|splice_donor_varian
on_coding_transcript_exon_variant|HIGH|RP11-213G2.5|ENSG00000231741|transcript|ENST0000044
V|RP11-213G2.5|ENSG00000231741|transcript|ENST00000442109.2|processed_pseudogene|2/3|n.5
OW|RP11-213G2.5|ENSG00000231741|transcript|ENST00000442109.2|processed_pseudogene|2/3|r

000135040|transcript|ENST00000361671.9|protein_coding|2/22|c.125-3dupT||||||INFO_REALIGN_
000135040|transcript|ENST00000361671.9|protein_coding|2/22|c.125-3dupT||||||INFO_REALIGN_
interaction|4PF4:A_38-A_95:ENST00000358077|protein_coding|3/26|c.114G>A||||||,A|structural_in
nteraction|1IG1:A_131-A_199:ENST00000358077|protein_coding|4/26|c.393C>T||||||,T|structural_i
42020.5|protein_coding|5/5|c.677A>C|p.Ter226Serext*?|801/933|677/678|226/225||,C|intron_vari
teraction|1CKS:B_20-B_50:ENST00000314355|protein_coding|2/3|c.60G>C||||||,C|splice_region_var
|interaction|3OL2:A_327-A_407:ENST00000356444|protein_coding|12/17|c.979G>A||||||,T|structu
00000226206|transcript|ENST00000421297.1|lincRNA|2/2|n.413+2G>T||||||,T|upstream_gene_vari
eraction|1A81:E_18-E_43:ENST00000375746|protein_coding|2/14|c.129G>A||||||,A|structural_inter
eraction|4FL2:A_347-A_434:ENST00000375746|protein_coding|10/14|c.1302G>C||||||,C|structural_i
eraction|1XBA:A_402-A_446:ENST00000375746|protein_coding|10/14|c.1338G>A||||||,A|structural_
eraction|1XBA:A_430-A_507:ENST00000375746|protein_coding|11/14|c.1521C>T||||||,T|structural_i
eraction|1XBC:A_493-A_515:ENST00000375746|protein_coding|11/14|c.1545T>C||||||,C|structural_i
nteraction|3ZZW:A_657-A_696:ENST00000375708|protein_coding|9/9|c.2088C>T||||||,A|structural_
27081|transcript|ENST00000375495.7|protein_coding|1/4|c.-31+1G>A||||||,T|splice_donor_variant
000188101|transcript|ENST00000340858.4|unprocessed_pseudogene|6/12|n.900-1T>C||||||
|interaction|4DWN:A_17-A_62:ENST00000466409|nonsense_mediated_decay|3/4|c.186C>T||||||,T|
000288985.11|protein_coding|1/14|c.1A>G|p.Met1?|306/4564|1/2139|1/712||,G|upstream_gene_v
312|transcript|ENST00000375223.4|protein_coding|5/5|c.405A>G|p.Ter135Trpext*?|619/619|405/4
ction|3FEY:A_3-C_321:ENST00000375147-ENST00000330459|protein_coding|1/23|c.9G>A||||||,A|p
00000259456.7|protein_coding|4/5|c.1135G>T|p.Glu379*|1279/2192|1135/1455|379/484||,A|stop_
427646.1|protein_coding|2/2|c.347A>G|p.Ter116Trpext*?|347/1378|347/348|116/115||WARNING_
2C4.3|ENSG00000267026|transcript|ENST00000628879.2|antisense|3/3|n.316-9_316-2dupCCTCCCTA
on_coding_transcript_exon_variant|HIGH|GRIN3A|ENSG00000198785|transcript|ENST00000479772.1
5833|transcript|ENST00000487798.5|protein_coding|6/6|c.*805+1G>A||||||,A|upstream_gene_vari
5833|transcript|ENST00000374798.7|protein_coding|7/7|c.*890+1G>A||||||,A|intron_variant|MOD
ENST00000374779.3|protein_coding|1/1|c.926delA|p.His309fs|926/957|926/957|309/318||
ENST00000374779.3|protein_coding|1/1|c.243_244delGC|p.Leu82fs|244/957|243/957|81/318||
ENST00000542196.2|protein_coding|1/1|c.513_516delTAAC|p.Asn171fs|516/957|513/957|171/318|
ENST00000542196.2|protein_coding|1/1|c.243_244delGC|p.Leu82fs|244/957|243/957|81/318||
000165028|transcript|ENST00000374762.3|protein_coding|5/5|c.667+1G>A||||||,A|splice_donor_va
ipt|ENST00000277225.9|protein_coding|3/13|c.3503_3504delGG|p.Arg1168fs|3792/10414|3503/75
ENST00000457811.1|protein_coding|4/4|c.321delA|p.Lys107fs|323/811|321/342|107/113||WARNIN
ENST00000457811.1|protein_coding|4/4|c.321delA|p.Lys107fs|323/811|321/342|107/113||WARNIN
00374667.4|protein_coding|1/1|c.2T>C|p.Met1?|1039/2379|2/1248|1/415||,G|sequence_feature|L
nteraction|2B49:A_715-A_838:ENST00000374541|protein_coding|22/26|c.2145C>T||||||,A|structur
IGH|YBX1P6|ENSG00000213539|transcript|ENST00000442962.2|processed_pseudogene|1/1|n.889-1
9|transcript|ENST00000442962.2|processed_pseudogene|1/1|n.889-15_889-12delAAAA||||||
39|transcript|ENST00000442962.2|processed_pseudogene|1/1|n.889-15_889-13delAAA||||||
539|transcript|ENST00000442962.2|processed_pseudogene|1/1|n.889-15_889-14delAA||||||
|ENST00000400613.4|protein_coding|1/2|c.115delA|p.Ile39fs|725/2717|115/720|39/239||,A|non_
0165181|transcript|ENST00000394777.8|protein_coding|1/22|c.133+2dupT||||||,TA|splice_donor_v
on_coding_transcript_exon_variant|HIGH|RP11-276E15.4|ENSG00000226609|transcript|ENST000004
00000619044.1|protein_coding|2/2|c.1021C>T|p.Arg341*|1932/2051|1021/1140|341/379||
interaction|3K1Z:A_135-A_160:ENST00000238379|protein_coding|2/2|c.480C>T||||||,A|structural_i
teraction|1E51:A_93-A_126:ENST00000409155|protein_coding|5/12|c.277G>A||||||,T|missense_var
teraction|1E51:A_36-A_56:ENST00000409155|protein_coding|4/12|c.168T>C||||||,G|structural_inte

157653|transcript|ENST00000288462.4|protein_coding|13/13|c.1171+1G>A||||||,A|splice_donor_va
4|interaction|2O0O:A_175-A_201:ENST00000374045|protein_coding|4/4|c.603A>G||||||,C|structura
00000230894|transcript|ENST00000423715.1|antisense|2/2|n.605+1G>A||||||,A|intron_variant|MO
0000136861|transcript|ENST00000473282.6|nonsense_mediated_decay|19/38|c.*851-2delA||||||,C
0000136861|transcript|ENST00000473282.6|nonsense_mediated_decay|19/38|c.*851-2delA||||||,C
180|transcript|ENST00000483960.5|processed_transcript|3/7|n.277+1G>A||||||,A|upstream_gene_v
000259357.3|protein_coding|1/1|c.705C>A|p.Cys235*|705/969|705/969|235/322||,T|downstream_
ENST00000335302.5|protein_coding|1/1|c.312dupT|p.Ile105fs|313/942|313/942|105/313||INFO_RE
0000623530.1|polymorphic_pseudogene|1/1|c.574C>T|p.Arg192*|612/995|574/957|192/318||,A|int
ENST00000623530.1|polymorphic_pseudogene|1/1|c.44dupT|p.Leu15fs|82/995|44/957|15/318||,C
0056586|transcript|ENST00000335387.9|protein_coding|9/9|c.1326-1A>G||||||,C|downstream_gen
t|ENST00000456584.5|nonsense_mediated_decay|15/28|c.1727delA|p.Lys576fs|2333/3897|1727/18
t|ENST00000456584.5|nonsense_mediated_decay|15/28|c.1727delA|p.Lys576fs|2333/3897|1727/18
NST00000359999.7|protein_coding|10/10|c.3411dupG|p.Trp1138fs|3503/3659|3412/3531|1138/11
t|ENST00000373580.7|protein_coding|6/8|c.1081_1084delACCA|p.Thr361fs|1081/6425|1081/1959|
36950|transcript|ENST00000353214.6|protein_coding|1/5|c.-984+2A>T||||||,T|upstream_gene_var
37|interaction|3VOQ:A_381-A_406:ENST00000265960|protein_coding|9/12|c.1143C>T||||||,A|struc
4|interaction|3TOW:A_51-A_99:ENST00000489637|protein_coding|3/6|c.297A>G||||||,G|structural_
000414832.2|protein_coding|3/4|c.344C>G|p.Ser115*|345/569|344/486|115/161||WARNING_TRAN
|interaction|3T6G:A_422-A_493:ENST00000373277|protein_coding|6/11|c.1266G>A||||||,T|structur
tion|4IMY:B_114-E_233:ENST00000261900-ENST00000373264|protein_coding|6/7|c.699G>A||||||,A
nteraction|2X2E:A_67-A_104:ENST00000372923|protein_coding|3/22|c.312G>A||||||,A|structural_i
000636280.1|nonsense_mediated_decay|7/10|c.924G>A|p.Trp308*|989/2462|924/963|308/320||,A
00197694|transcript|ENST00000636939.1|processed_transcript|3/3|n.78-2A>G||||||,G|upstream_ge
0160446|transcript|ENST00000452105.5|protein_coding|5/5|c.483-2_493dupAGCACCAGGCCCC|p.Leu
nteraction|1W7L:A_120-A_140:ENST00000302586|protein_coding|5/13|c.358A>G||||||,C|structural_
NST00000427720.1|protein_coding|4/5|c.375_376delAA|p.Lys125fs|376/477|375/477|125/158||WA
NST00000427720.1|protein_coding|4/5|c.375_376delAA|p.Lys125fs|376/477|375/477|125/158||WA
7|interaction|2OPW:A_160-A_200:ENST00000372592|protein_coding|11/13|c.600A>G||||||,G|struct
transcript|ENST00000372586.3|protein_coding|1/1|c.1dupA|p.Met1fs|317/2090|1/1617|1/538||,AT
435305.1|protein_coding|2/2|c.454T>G|p.Ter152Glyext*?|488/530|454/456|152/151||,G|stop_lost|
0000624552.3|protein_coding|14/98|c.2093C>A|p.Ser698*|2093/15610|2093/15180|698/5059||,A|
t|ENST00000611173.1|protein_coding|10/10|c.1670dupA|p.Arg558fs|1730/1920|1671/1788|557/59
teraction|2NZ2:A_26-A_167:ENST00000352480|protein_coding|7/15|c.501C>T||||||,T|structural_int
teraction|2NZ2:A_344-A_367:ENST00000352480|protein_coding|13/15|c.1101G>A||||||,A|structura
teraction|2G1T:B_467-B_499:ENST00000318560|protein_coding|9/11|c.1497A>G||||||,G|synonymo
00000126883|transcript|ENST00000359428.9|protein_coding|9/35|c.1006-3dupT||||||INFO_REALIGI
00000126883|transcript|ENST00000359428.9|protein_coding|9/35|c.1006-3dupT||||||INFO_REALIGI
00000126883|transcript|ENST00000359428.9|protein_coding|9/35|c.1006-3dupT||||||INFO_REALIGI
G00000236986|transcript|ENST00000589667.5|antisense|3/3|n.619+1delG||||||,A|splice_donor_var
000451030.5|protein_coding|2/26|c.1A>G|p.Met1?|248/5021|1/4560|1/1519||,G|missense_variant|
00126883|transcript|ENST00000453861.6|nonsense_mediated_decay|9/12|c.2631-2A>G||||||WARN
0000372228.7|protein_coding|8/20|c.751C>T|p.Gln251*|930/3123|751/2244|251/747||,T|stop_gair
interaction|3ZYG:A_153-A_246:ENST00000393229|protein_coding|3/8|c.738C>T||||||,T|structural_i
interaction|3ZYG:A_109-A_276:ENST00000393229|protein_coding|3/8|c.828C>T||||||,T|structural_i
interaction|3ZYG:A_292-A_329:ENST00000393229|protein_coding|4/8|c.876C>T||||||,T|structural_i
G00000252521|gene_variant|ENSG00000188523|||n.132421611_132421686delGAGAATCCTGGGTTCT

|interaction|3KH0:A_805-A_880:ENST00000372050|protein_coding|18/18|c.2640C>G||||||,C|structu
000372040.7|protein_coding|7/7|c.363C>A|p.Tyr121*|675/1980|363/1044|121/347||,T|stop_gaine
NST00000372034.7|protein_coding|4/7|c.302delT|p.Leu101fs|344/691|302/513|101/170||,C|frame
175164|transcript|ENST00000538324.2|protein_coding|9/9|c.1054delC|p.Arg352fs|1079/1147|1054
0538324.2|protein_coding|8/9|c.539G>A|p.Trp180*|564/1147|539/1122|180/373||,T|stop_gained|
IGH|ABO|ENSG00000175164|transcript|ENST00000538324.2|protein_coding|6/8|c.259-1_259insG||
323|interaction|3GHM:A_572-A_656:ENST00000371929|protein_coding|15/29|c.1716G>A||||||,A|st
nteraction|3EG6:A_165-A_202:ENST00000358625|protein_coding|9/14|c.606A>G||||||,G|synonymo
teraction|1XV9:A_254-A_339:ENST00000481739|protein_coding|5/10|c.762G>A||||||,A|structural_i
t|ENST00000371799.8|protein_coding|2/2|c.358_359insAA|p.Gly120fs|644/961|359/504|120/167||
t|ENST00000371799.8|protein_coding|2/2|c.452_453dupCA|p.Cys152fs|739/961|454/504|152/167|
224682|transcript|ENST00000449637.1|processed_pseudogene|3/4|n.1149+1T>A||||||
0122140|transcript|ENST00000488610.5|processed_transcript|3/4|n.312-1G>C||||||,C|upstream_ge
|ENST00000340780.7|protein_coding|7/7|c.587dupC|p.Leu198fs|630/741|588/687|196/228||INFO_
ENST00000371763.5|protein_coding|5/5|c.427delT|p.Trp143fs|681/1526|427/831|143/276||,C|fran
1|ENSG00000107147|transcript|ENST00000371757.6|protein_coding|28/30|c.3178-7_3178-3dupTGC
1|ENSG00000107147|transcript|ENST00000371757.6|protein_coding|28/30|c.3178-7_3178-3dupTGC
NST00000616132.4|protein_coding|9/9|c.1242delC|p.Thr415fs|1462/2270|1242/1374|414/457||IN
0000616132.4|protein_coding|9/9|c.1283C>A|p.Ser428*|1503/2270|1283/1374|428/457||,A|intron
37796|transcript|ENST00000371732.9|protein_coding|11/12|c.1434+1G>C||||||,G|splice_donor_var
interaction|2XSW:A_285-A_598:ENST00000371712|protein_coding|9/10|c.1794G>T||||||,A|structur
interaction|2XSW:A_483-A_507:ENST00000371712|protein_coding|7/10|c.1521C>T||||||,A|synonym
interaction|2XSW:A_416-A_596:ENST00000371712|protein_coding|5/10|c.1248T>C||||||,G|structur
000497771.5|protein_coding|5/6|c.481C>T|p.Gln161*|537/723|481/603|161/200||,A|stop_gained|
nteraction|2XST:A_113-A_152:ENST00000316144|protein_coding|5/7|c.454T>G||||||,C|missense_va
nteraction|2HW4:A_24-A_74:ENST00000247665|protein_coding|2/3|c.220C>T||||||,T|structural_int
176919|transcript|ENST00000224181.3|protein_coding|2/6|c.276-3dupC||||||INFO_REALIGN_3_PRI
0184925|transcript|ENST00000495410.1|processed_transcript|1/1|n.363-2A>G||||||,G|upstream_ge
0184925|transcript|ENST00000495410.1|processed_transcript|1/1|n.363-2A>G||||||,G|upstream_ge
0000371600.7|protein_coding|1/8|c.73C>T|p.Arg25*|746/2187|73/1212|25/403||,A|upstream_gene
9|interaction|1FMI:A_538-A_682:ENST00000371589|protein_coding|13/13|c.2046T>C||||||,C|struct
0188163|transcript|ENST00000344774.5|protein_coding|3/6|c.522+2C>T||||||,A|splice_donor_varia
|ENST00000406427.5|protein_coding|1/35|c.22dupA|p.Ser8fs|359/4806|22/4029|8/1342||,CT|fram
on_coding_transcript_exon_variant|HIGH|ARRDC1|ENSG00000197070|transcript|ENST00000483563.5
77517.1|protein_coding|5/5|c.684A>T|p.Ter228Cysext*?|749/1359|684/684|228/227||,A|3_prime_
0277517.1|protein_coding|5/5|c.431G>A|p.Trp144*|496/1359|431/684|144/227||,T|3_prime_UTR_
eraction|2PNY:A_73-A_166:ENST00000277517|protein_coding|3/5|c.219G>A||||||,T|synonymous_va
064|transcript|ENST00000427898.5|protein_coding|1/2|c.55+1delG||||||WARNING_TRANSCRIPT_IN
0381329.5|protein_coding|9/9|c.748T>C|p.Ter250Argext*?|949/1307|748/750|250/249||,C|splice_
ENSG00000282390|gene_variant|ENSG00000282390|||n.1293005_1292984delCTTCCCTCCCTCTCCCTC
teraction|4U1R:B_567-B_598:ENST00000381125|protein_coding|17/22|c.1794C>T||||||,T|structural_
|interaction|4L3T:A_869-A_1017:ENST00000224949|protein_coding|27/27|c.3049G>A||||||,T|struct
|interaction|4NGE:A_879-A_1007:ENST00000224949|protein_coding|27/27|c.3021A>G||||||,C|splice
00455371.5|protein_coding|4/4|c.553T>C|p.Ter185Glnext*?|553/673|553/555|185/184||WARNING_
|interaction|4L3T:A_115-A_169:ENST00000224949|protein_coding|5/27|c.506T>C||||||,G|structural
|interaction|4L3T:B_94-B_142:ENST00000224949|protein_coding|4/27|c.282T>C||||||,G|synonymou
ed|HIGH|PITRM1|ENSG00000107959|transcript|ENST00000380989.6|protein_coding|4/27|c.271_27

0000298375.11|protein_coding|9/10|c.901C>T|p.Arg301*|972/1566|901/963|301/320||,T|stop_gai
|interaction|1MRQ:A_170-A_323:ENST00000380872|protein_coding|5/9|c.509G>A||||||,A|structura
151632|transcript|ENST00000604428.5|processed_transcript|3/3|n.542+1G>C||||||,G|splice_donor_
00605149.5|protein_coding|1/9|c.3G>T|p.Met1?|328/1405|3/903|1/300||,T|start_lost|HIGH|AKR1C
teraction|3EO2:A_200-A_302:ENST00000355029|protein_coding|7/12|c.600T>C||||||,C|synonymous
GH|RPL12P28|ENSG00000240180|transcript|ENST00000430852.1|processed_pseudogene|1/1|n.398
GH|RPL12P28|ENSG00000240180|transcript|ENST00000430852.1|processed_pseudogene|1/1|n.398
GH|RPL12P28|ENSG00000240180|transcript|ENST00000430852.1|processed_pseudogene|1/1|n.398
t|ENST00000380092.8|protein_coding|2/7|c.356_357delTG|p.Val119fs|874/1646|356/1086|119/361
NST00000620865.4|protein_coding|7/7|c.875_897delTTTTCTGTACCATGTTTGTTTCC|p.Leu292fs|1016/
nteraction|1XJD:A_591-A_616:ENST00000263125|protein_coding|17/18|c.1846C>A||||||,T|structura
interaction|1XJD:A_440-A_520:ENST00000263125|protein_coding|15/18|c.1560C>T||||||,A|structura
nteraction|1XJD:A_442-A_520:ENST00000263125|protein_coding|12/18|c.1326G>A||||||,T|structura
GH|ECHDC3|ENSG00000134463|transcript|ENST00000495787.1|processed_transcript|2/2|n.120-5_1
GH|ECHDC3|ENSG00000134463|transcript|ENST00000495787.1|processed_transcript|2/2|n.120-5_1
GH|ECHDC3|ENSG00000134463|transcript|ENST00000495787.1|processed_transcript|2/2|n.120-5_1
GH|ECHDC3|ENSG00000134463|transcript|ENST00000495787.1|processed_transcript|2/2|n.120-5_1
|interaction|2VX2:A_140-A_252:ENST00000379215|protein_coding|4/5|c.420T>C||||||,C|structural_
|interaction|2VX2:A_142-A_162:ENST00000379215|protein_coding|4/5|c.484G>A||||||,A|structural_
|interaction|2VX2:A_147-A_167:ENST00000379215|protein_coding|4/5|c.501C>T||||||,T|structural_
|interaction|2VX2:B_242-B_298:ENST00000379215|protein_coding|5/5|c.726G>A||||||,A|structural_
|interaction|2VX2:A_162-A_256:ENST00000379215|protein_coding|5/5|c.768C>T||||||,T|structural_
|interaction|3FD5:A_295-A_373:ENST00000327347|protein_coding|8/9|c.885G>C||||||,G|structural_
interaction|3FD5:A_125-A_173:ENST00000327347|protein_coding|4/9|c.375C>G||||||,C|structural_
00000239665|transcript|ENST00000601075.5|lincRNA|3/3|n.765+1G>A||||||,A|splice_donor_variant
ENSG00000226140|transcript|ENST00000417542.1|lincRNA|3/3|n.289-3dupT||||||INFO_REALIGN_3_
|ENSG00000226140|transcript|ENST00000417542.1|lincRNA|3/3|n.289-4_289-3dupTT||||||INFO_RE
00000226140|transcript|ENST00000417542.1|lincRNA|3/3|n.289-3delT||||||INFO_REALIGN_3_PRIM
I|interaction|1G55:A_146-A_183:ENST00000377799|protein_coding|8/11|c.549G>T||||||,A|structura
I|interaction|1G55:A_60-A_101:ENST00000377799|protein_coding|4/11|c.301C>T||||||,A|missense_
CNB2|ENSG00000165995|transcript|ENST00000324631.11|protein_coding|1/13|c.121-8_121-3dupTT
CNB2|ENSG00000165995|transcript|ENST00000324631.11|protein_coding|1/13|c.121-8_121-3dupTT
CNB2|ENSG00000165995|transcript|ENST00000324631.11|protein_coding|1/13|c.121-8_121-3dupTT
CNB2|ENSG00000165995|transcript|ENST00000324631.11|protein_coding|1/13|c.121-8_121-3dupTT
00000165995|transcript|ENST00000324631.11|protein_coding|1/13|c.121-3_121-2insTGT||||||INFO
00000165995|transcript|ENST00000324631.11|protein_coding|1/13|c.121-3_121-2insTGT||||||INFO
00000165995|transcript|ENST00000324631.11|protein_coding|1/13|c.121-3_121-2insTGT||||||INFO
00000165995|transcript|ENST00000324631.11|protein_coding|1/13|c.121-3_121-2insTGT||||||INFO
00165995|transcript|ENST00000612743.1|protein_coding|2/2|c.35-2C>T||||||,T|synonymous_variar
ot|ENST00000534331.5|protein_coding|1/2|c.94_97delAGTG|p.Ser32fs|148/579|94/468|32/155||,A
0000078403|transcript|ENST00000377091.6|protein_coding|3/4|c.241-3dupT||||||INFO_REALIGN_3_
0000078403|transcript|ENST00000377091.6|protein_coding|3/4|c.241-3dupT||||||INFO_REALIGN_3_
GH|ADIPOR1P1|ENSG00000232497|transcript|ENST00000435632.1|processed_pseudogene|2/2|n.10
GH|ADIPOR1P1|ENSG00000232497|transcript|ENST00000435632.1|processed_pseudogene|2/2|n.10
GH|ADIPOR1P1|ENSG00000232497|transcript|ENST00000435632.1|processed_pseudogene|2/2|n.10
GH|ADIPOR1P1|ENSG00000232497|transcript|ENST00000435632.1|processed_pseudogene|2/2|n.10
GH|ADIPOR1P1|ENSG00000232497|transcript|ENST00000435632.1|processed_pseudogene|2/2|n.10

nteraction|2OKK:A_339-A_503:ENST00000259271|protein_coding|10/17|c.1017C>T||||||,T|structura
)0000227932|transcript|ENST00000636744.1|unitary_pseudogene|9/9|n.1115+1C>T||||||,A|intron_
3|interaction|4Y83:A_78-A_114:ENST00000263056|protein_coding|3/9|c.234T>C||||||,C|structural_i
)234814|transcript|ENST00000422642.6|transcribed_unprocessed_pseudogene|5/18|n.501-2A>T||||
IGH|RP11-14C22.6|ENSG00000230720|transcript|ENST00000416310.1|lincRNA|1/1|n.87-6_87-2delA(
)0331737.10|protein_coding|6/8|c.1A>G|p.Met1?|460/3243|1/2457|1/818||,C|start_lost|HIGH|ZNF
ENST00000362006.10|protein_coding|2/18|c.230dupT|p.Leu77fs|738/2093|231/1461|77/486||INFC
.6937|transcript|ENST00000362006.10|protein_coding|12/17|c.993+1G>T||||||,T|splice_donor_vari
ipt|ENST00000375028.7|protein_coding|15/19|c.1405_1406delAA|p.Lys469fs|1405/1975|1405/1815
teraction|2QQI:A_520-A_561:ENST00000374867|protein_coding|10/17|c.1683C>G||||||,C|structura
nteraction|2QQI:A_337-A_422:ENST00000374867|protein_coding|8/17|c.1266C>T||||||,A|structural_
ST00000611781.4|protein_coding|3/36|c.496G>T|p.Glu166*|496/4474|496/4194|166/1397||,T|stop
pt|ENST00000611781.4|protein_coding|27/36|c.2611delG|p.Ala871fs|2611/4474|2611/4194|871/13
_coding_transcript_exon_variant|HIGH|CCNYL2|ENSG00000182632|transcript|ENST00000472090.6|tr
_coding_transcript_exon_variant|HIGH|CCNYL2|ENSG00000182632|transcript|ENST00000472090.6|tr
_coding_transcript_exon_variant|HIGH|CCNYL2|ENSG00000182632|transcript|ENST00000472090.6|tr
eraction|2IVV:A_747-A_791:ENST00000340058|protein_coding|13/19|c.2372A>T||||||,T|structural_ir
ot|ENST00000374455.2|protein_coding|1/6|c.18_19delCT|p.Trp7fs|20/632|18/631|6/209||WARNIN(
ot|ENST00000619243.1|protein_coding|1/3|c.67delC|p.His23fs|78/1110|67/393|23/130||INFO_REAI
)0226937|transcript|ENST00000456938.6|processed_transcript|6/6|n.618+2T>C||||||,G|downstrean
)000226937|transcript|ENST00000594514.5|processed_transcript|1/4|n.321-2delA||||||,C|downstre
:NST00000492347.1|protein_coding|1/6|c.-13_8delTGGGGACAGGATGGCAGGAGC|p.Gly2fs|8/413|1/4
)0263482|transcript|ENST00000604933.2|unprocessed_pseudogene|7/13|n.581+1G>A||||||
on_coding_transcript_exon_variant|HIGH|ANTXRLP1|ENSG00000263482|transcript|ENST0000060493
274209|transcript|ENST00000610892.1|retained_intron|1/1|n.291+1G>A||||||,A|upstream_gene_va
)204176|transcript|ENST00000503753.5|protein_coding|4/8|c.652-3dupC||||||INFO_REALIGN_3_PF
:NST00000452145.6|protein_coding|8/8|c.1848delC|p.Asn616fs|1960/2387|1848/1977|616/658||,C
)000189014|transcript|ENST00000323387.5|unprocessed_pseudogene|6/7|n.2397-1G>C||||||
|ENST00000374201.7|protein_coding|26/29|c.3251delA|p.Lys1084fs|3554/4997|3251/3930|1084/13
5G00000271237|transcript|ENST00000605814.1|processed_pseudogene|1/2|n.225-2_225-1delAA||||
ISG00000271237|transcript|ENST00000605814.1|processed_pseudogene|1/2|n.225-1delA||||||INFC
teraction|2FY2:A_429-A_560:ENST00000339797|protein_coding|12/15|c.1287T>C||||||,C|structural
T00000374112.7|protein_coding|2/3|c.195C>A|p.Cys65*|207/2130|195/474|65/157||,A|stop_gainec
I|C10orf53|ENSG00000178645|transcript|ENST00000535836.1|protein_coding|3/3|c.*1349+1_*1349
riant&intron_variant|HIGH|C10orf53|ENSG00000178645|transcript|ENST00000535836.1|protein_cod
riant&intron_variant|HIGH|C10orf53|ENSG00000178645|transcript|ENST00000535836.1|protein_cod
riant&intron_variant|HIGH|C10orf53|ENSG00000178645|transcript|ENST00000535836.1|protein_cod
riant&intron_variant|HIGH|C10orf53|ENSG00000178645|transcript|ENST00000535836.1|protein_cod
riant&intron_variant|HIGH|C10orf53|ENSG00000178645|transcript|ENST00000535836.1|protein_cod
SG00000178645|transcript|ENST00000535836.1|protein_coding|3/3|c.*1349+1G>C||||||,G|splice_re
SG00000178645|transcript|ENST00000535836.1|protein_coding|3/3|c.*1349+1G>C||||||,G|splice_re
SG00000178645|transcript|ENST00000535836.1|protein_coding|3/3|c.*1349+1G>C||||||,G|splice_re
|ENST00000412531.3|protein_coding|1/8|c.55dupC|p.Gln19fs|128/2372|56/2061|19/686||INFO_RE/
ENST00000412531.3|protein_coding|1/8|c.208delC|p.Arg70fs|280/2372|208/2061|70/686||INFO_RE
:NST00000412531.3|protein_coding|8/8|c.791_792delAA|p.Lys264fs|863/2372|791/2061|264/686||
)0000232706|transcript|ENST00000442076.2|unprocessed_pseudogene|1/7|n.383-1A>G||||||
nteraction|3OCP:A_142-A_198:ENST00000373980|protein_coding|2/18|c.426C>T||||||,T|structural_

interaction|4KU7:A_282-A_321:ENST00000373980|protein_coding|7/18|c.845A>G||||||,G|structural
0000231131|transcript|ENST00000435813.6|lincRNA|2/2|n.332-1G>T||||||,A|splice_acceptor_variar
NSG00000236556|transcript|ENST00000433249.1|antisense|1/1|n.63-2A>G||||||,G|intron_variant|M
GH|CDK1|ENSG00000170312|transcript|ENST00000614696.4|protein_coding|7/8|c.660+2A>G|||||
170312|transcript|ENST00000614696.4|protein_coding|7/8|c.661-1A>T||||||,T|downstream_gene_v
variant|HIGH|TMEM26|ENSG00000196932|transcript|ENST00000277749.9|protein_coding|4/4|c.41
0000373789.7|protein_coding|4/12|c.322C>T|p.Arg108*|419/6641|322/1830|108/609||,A|stop_gain
|interaction|2YPD:A_2263-A_2316:ENST00000399262|protein_coding|20/26|c.6789C>T||||||,A|struc
ariant&intron_variant|HIGH|RP11-170M17.1|ENSG00000228566|transcript|ENST00000444770.1|linc
00228065|transcript|ENST00000433152.8|lincRNA|4/6|n.568+1G>A||||||,A|splice_donor_variant&ir
00228065|transcript|ENST00000626101.2|lincRNA|3/3|n.206+1G>C||||||,C|downstream_gene_varia
teraction|4IF6:A_332-A_496:ENST00000212015|protein_coding|5/9|c.994T>C||||||,C|structural_inte
GH|CCAR1|ENSG00000060339|transcript|ENST00000265872.10|protein_coding|22/24|c.3002-6_300
0000421716.1|protein_coding|1/4|c.1A>G|p.Met1?|94/403|1/310|1/102||WARNING_TRANSCRIPT_IN
SG00000107719|transcript|ENST00000263563.6|protein_coding|2/19|c.186-3dupC||||||INFO_REALI
07719|transcript|ENST00000263563.6|protein_coding|2/19|c.186-7_186-3delCCCCC||||||INFO_REAL
107719|transcript|ENST00000263563.6|protein_coding|2/19|c.186-6_186-3delCCCC||||||INFO_REAL
0107719|transcript|ENST00000263563.6|protein_coding|2/19|c.186-5_186-3delCCC||||||INFO_REAL
00107719|transcript|ENST00000263563.6|protein_coding|2/19|c.186-4_186-3delCC||||||INFO_REAL
000107719|transcript|ENST00000263563.6|protein_coding|2/19|c.186-3delC||||||INFO_REALIGN_3_
nteraction|4Q6R:A_314-A_346:ENST00000373202|protein_coding|11/15|c.942C>T||||||,T|structural_
8303|transcript|ENST00000394919.5|protein_coding|1/9|c.-34+1A>G||||||,C|splice_donor_variant&
0107745|transcript|ENST00000398761.8|protein_coding|5/11|c.542-2A>G||||||,C|splice_region_var
ST00000605597.5|nonsense_mediated_decay|1/8|c.134_135delTG|p.Val45fs|274/3224|134/267|45/
00156026|transcript|ENST00000357157.10|protein_coding|4/7|c.497-3dupT||||||INFO_REALIGN_3_F
00156026|transcript|ENST00000357157.10|protein_coding|4/7|c.497-3dupT||||||INFO_REALIGN_3_F
026|transcript|ENST00000357157.10|protein_coding|5/7|c.594+1G>A||||||,A|intron_variant|MODI
0000166321|transcript|ENST00000617744.4|protein_coding|8/8|c.705delC|p.Tyr236fs|907/928|705/
G00000138286|transcript|ENST00000242505.10|protein_coding|1/14|c.-2_1delGGA|p.Met1del|173/
ot|ENST00000394810.2|protein_coding|4/4|c.1517dupC|p.Pro507fs|1667/4921|1517/2934|506/977
|interaction|2GWO:A_77-A_102:ENST00000472493|protein_coding|3/4|c.306C>T||||||,A|structural_
ENST00000478873.6|protein_coding|1/4|c.234delA|p.Ala80fs|324/1335|234/1005|78/334||,C|upstr
NSG00000226051|transcript|ENST00000533822.1|lincRNA|1/3|n.156-3dupT||||||INFO_REALIGN_3_F
NSG00000226051|transcript|ENST00000533822.1|lincRNA|1/3|n.156-3dupT||||||INFO_REALIGN_3_F
NSG00000226051|transcript|ENST00000533822.1|lincRNA|1/3|n.156-3dupT||||||INFO_REALIGN_3_F
0000156113&ENSG00000225652|gene_variant|ENSG00000225652|||n.77376559_77376641delTTTTTT
0000282952|transcript|ENST00000635018.1|lincRNA|2/2|n.189+1G>A||||||,T|intron_variant|MODIF
eraction|3RCG:A_88-A_109:ENST00000225174|protein_coding|3/6|c.264C>T||||||,T|structural_inter
G00000225484|transcript|ENST00000596088.5|antisense|2/3|n.122-1G>A||||||,T|downstream_gen
00000230091|transcript|ENST00000412298.5|antisense|4/5|n.611+2C>T||||||,A|splice_donor_varia
ipt|ENST00000613758.4|protein_coding|2/5|c.123dupA|p.Glu42fs|178/2154|124/306|42/101||INFO
ipt|ENST00000613758.4|protein_coding|2/5|c.123dupA|p.Glu42fs|178/2154|124/306|42/101||INFO
ot|ENST00000613758.4|protein_coding|2/5|c.132_135delGAAA|p.Lys45fs|186/2154|132/306|44/101
00189129|transcript|ENST00000372263.3|protein_coding|3/3|c.283_283+1insAAG||||||INFO_REALI
122359|transcript|ENST00000265447.8|protein_coding|2/14|c.-45+2T>C||||||,G|upstream_gene_va
interaction|2OBV:A_27-A_377:ENST00000372213|protein_coding|9/9|c.1131C>T||||||,A|synonymo
interaction|2OBV:A_35-A_263:ENST00000372213|protein_coding|7/9|c.789C>T||||||,A|synonymou

|interaction|2OBV:A_142-A_314:ENST00000372213|protein_coding|5/9|c.426T>C||||||,G|synonymo
0000230962|transcript|ENST00000441457.1|lincRNA|3/5|n.1107-2A>G|||||
nteraction|4K6J:A_1036-A_1125:ENST00000298767|protein_coding|17/19|c.3375A>G||||||,C|structu
348795.8|protein_coding|4/5|c.381A>T|p.Ter127Cysext*?|449/684|381/381|127/126||,T|missense_
G00000223482|transcript|ENST00000446751.5|antisense|5/5|n.671-1G>C||||||,G|intron_variant|M
G00000225913|transcript|ENST00000438082.1|lincRNA|3/3|n.172-2A>G||||||,G|upstream_gene_va
00171862|transcript|ENST00000371953.7|protein_coding|7/8|c.802-3dupT||||||INFO_REALIGN_3_PI
00171862|transcript|ENST00000371953.7|protein_coding|7/8|c.802-3dupT||||||INFO_REALIGN_3_PI
00171862|transcript|ENST00000371953.7|protein_coding|7/8|c.802-3dupT||||||INFO_REALIGN_3_PI
4022|transcript|ENST00000371939.7|protein_coding|5/10|c.330-1G>C||||||,C|upstream_gene_varia
GH|LIPJ|ENSG00000204022|transcript|ENST00000371939.7|protein_coding|6/10|c.440-6_440-2delT
239|transcript|ENST00000404743.8|protein_coding|5/8|c.711+1delG||||||INFO_REALIGN_3_PRIME,
T00000371837.5|protein_coding|2/9|c.52delC|p.Leu18fs|274/2513|52/1032|18/343||,A|upstream_
raction|2G47:B_539-B_563:ENST00000265986|protein_coding|13/25|c.1617G>T||||||,A|structural_
000463743.5|nonsense_mediated_decay|28/34|c.3499C>T|p.Arg1167*|3501/4812|3499/3531|1167/
36155.1|protein_coding|8/8|c.864G>T|p.Ter288Tyrext*?|981/1618|864/864|288/287||,T|non_codir
interaction|1OG2:A_144-A_180:ENST00000260682|protein_coding|3/9|c.430C>T||||||,T|structural_
interaction|4NZ2:B_359-B_478:ENST00000260682|protein_coding|7/9|c.1075A>C||||||,C|missense_
interaction|1OG2:B_48-B_475:ENST00000260682|protein_coding|9/9|c.1425A>T||||||,T|structural_
interaction|1PQ2:A_182-A_264:ENST00000371270|protein_coding|5/9|c.792C>G||||||,C|structural_
|ENST00000628935.1|protein_coding|1/6|c.68dupT|p.Leu23fs|68/924|68/924|23/307||,TA|splice_r
38115|transcript|ENST00000539050.5|protein_coding|2/9|c.-43+1A>G||||||,C|upstream_gene_varia
000371109.3|protein_coding|1/10|c.1A>G|p.Met1?|183/1766|1/1215|1/404||,C|sequence_feature|I
t&intron_variant|HIGH|ANKRD2|ENSG00000165887|transcript|ENST00000307518.9|protein_coding|
interaction|3S5N:A_108-A_132:ENST00000370646|protein_coding|3/7|c.396G>A||||||,A|structural_
00478953.1|protein_coding|4/4|c.199T>C|p.Ter67Argext*?|383/2225|199/201|67/66||,G|synonymo
119986|transcript|ENST00000370626.3|protein_coding|1/2|c.-10-1G>C||||||,G|downstream_gene_
cript|ENST00000266066.3|protein_coding|1/3|c.19_20insCGGCG|p.Gly7fs|138/1836|19/954|7/317||
|interaction|4CC7:C_1519-C_1575:ENST00000324109|protein_coding|17/17|c.4725G>A||||||,T|struc
|interaction|4GLM:A_249-A_298:ENST00000324109|protein_coding|4/17|c.747C>T||||||,A|structura
75826|transcript|ENST00000479697.5|nonsense_mediated_decay|10/25|c.*1557+2C>G||||||,C|ups
|interaction|2ILM:A_12-A_41:ENST00000299163|protein_coding|1/8|c.121C>G||||||,G|structural_in
teraction|4KM8:A_407-A_433:ENST00000369902|protein_coding|11/12|c.1299T>C||||||,C|structura
NSG00000270316|gene_variant|ENSG00000270316|||n.102869498_102869533delCCTGAGTCGCAGG
000441178.2|protein_coding|1/1|c.178C>T|p.Gln60*|288/2130|178/687|60/228||
00107957|transcript|ENST00000355946.6|protein_coding|4/13|c.306+2T>G||||||,C|splice_donor_va
eraction|2J51:A_154-A_176:ENST00000369755|protein_coding|5/19|c.528A>G||||||,G|structural_int
intron_variant|HIGH|GSTO1|ENSG00000148834|transcript|ENST00000369713.9|protein_coding|4/6|
nteraction|2G6Z:A_203-A_258:ENST00000369583|protein_coding|3/4|c.607C>T||||||,T|structural_ir
000369358.8|protein_coding|38/42|c.4504C>T|p.Arg1502*|4692/5534|4504/5196|1502/1731||,A|st
NST00000369321.6|protein_coding|1/7|c.130_131delTT|p.Leu44fs|216/2620|130/1167|44/388||INF
nteraction|1F1J:A_181-A_220:ENST00000621345|protein_coding|5/7|c.541T>C||||||,C|structural_in
nteraction|1K88:B_255-B_300:ENST00000621345|protein_coding|7/7|c.765C>G||||||,G|structural_i
GH|CTB-1144G6.5|ENSG00000230165|transcript|ENST00000454983.1|processed_pseudogene|1/1|n
GH|CTB-1144G6.5|ENSG00000230165|transcript|ENST00000454983.1|processed_pseudogene|1/1|n
GH|CTB-1144G6.5|ENSG00000230165|transcript|ENST00000454983.1|processed_pseudogene|1/1|n
GH|CTB-1144G6.5|ENSG00000230165|transcript|ENST00000454983.1|processed_pseudogene|1/1|n

GH|CTB-1144G6.5|ENSG00000230165|transcript|ENST00000454983.1|processed_pseudogene|1/1|n
ipt|ENST00000369230.3|protein_coding|5/12|c.533_534insA|p.Ser179fs|680/2354|534/1404|178/46
1|interaction|2PPL:A_61-A_116:ENST00000358834|protein_coding|3/13|c.181A>G||||||,G|structura
1|interaction|2PPL:A_378-A_415:ENST00000358834|protein_coding|11/13|c.1134T>C||||||,C|struct
0000579578.6|polymorphic_pseudogene|11/13|c.1071A>G|p.Ter357Trpext*?|1097/1482|1071/1410|
00000119979|transcript|ENST00000497903.5|processed_transcript|2/3|n.366-3dupT|||||INFO_REA
00000119979|transcript|ENST00000497903.5|processed_transcript|2/3|n.366-3dupT|||||INFO_REA
ction|2IHB:A_180-B_99:ENST00000369851-ENST00000369101|protein_coding|3/4|c.297C>T||||||,A|
nteraction|1NUN:B_190-B_232:ENST00000457416|protein_coding|6/18|c.696A>G||||||,C|structural_
0000528446.1|protein_coding|1/2|c.112C>T|p.Arg38*|187/818|112/324|38/107||
ENST00000628426.1|protein_coding|6/6|c.1365_1366delCC|p.Arg456fs|1969/3553|1365/1521|455/
ENST00000628426.1|protein_coding|6/6|c.1365_1366delCC|p.Arg456fs|1969/3553|1365/1521|455/
ENST00000628426.1|protein_coding|6/6|c.1365_1366delCC|p.Arg456fs|1969/3553|1365/1521|455/
ENST00000628426.1|protein_coding|6/6|c.1365_1366delCC|p.Arg456fs|1969/3553|1365/1521|455/
ENST00000628426.1|protein_coding|6/6|c.1365_1366delCC|p.Arg456fs|1969/3553|1365/1521|455/
ENST00000628426.1|protein_coding|6/6|c.1365_1366delCC|p.Arg456fs|1969/3553|1365/1521|455/
eraction|1GBN:A_357-A_378:ENST00000368845|protein_coding|9/10|c.1134C>T||||||,A|structural_
0214807|transcript|ENST00000399021.2|processed_pseudogene|1/1|n.493+2G>T||||||
NST00000309035.10|protein_coding|1/9|c.524_525delAG|p.Gln175fs|656/3669|524/2958|175/985|
G00000237224|transcript|ENST00000593883.2|antisense|1/1|n.501-1G>C||||||,G|upstream_gene_
00000283141|transcript|ENST00000635764.1|lincRNA|2/6|n.450+2T>A||||||
G00000283141|transcript|ENST00000635764.1|lincRNA|3/6|n.638-1G>A||||||
00000165752|transcript|ENST00000368619.3|protein_coding|1/1|c.302-4_302-1dupATAG||||||,CCT
00000631148.2|protein_coding|3/3|c.1492C>T|p.Arg498*|1551/2013|1492/1500|498/499||,T|3_pr
t|ENST00000607359.5|protein_coding|9/16|c.1672dupG|p.Ala558fs|1673/6004|1673/3840|558/127
interaction|4DD8:C_334-C_365:ENST00000445355|protein_coding|11/23|c.1095G>A||||||,T|structu
108K14.8|ENSG00000254536|transcript|ENST00000468317.2|protein_coding|1/12|c.-7_10+16delGC
14279|transcript|ENST00000482993.6|retained_intron|4/9|n.2821+1G>C||||||,C|splice_donor_varia
0000214279|transcript|ENST00000488261.6|retained_intron|11/13|n.4041-3dupT|||||INFO_REALIG
14279|transcript|ENST00000488261.6|retained_intron|11/13|n.4041-3delT|||||INFO_REALIGN_3_
interaction|3GPH:A_179-A_446:ENST00000252945|protein_coding|4/9|c.535G>A||||||,A|structural_
0130649|transcript|ENST00000368520.1|retained_intron|5/5|n.1359-2A>G||||||,G|downstream_ge
NST00000425520.1|protein_coding|4/4|c.480delG|p.Arg160fs|533/890|480/837|160/278||,A|frame
teraction|3GLS:C_208-C_383:ENST00000382743|protein_coding|3/7|c.622G>A||||||,T|missense_var
nteraction|4BVH:A_159-A_345:ENST00000382743|protein_coding|3/7|c.477G>T||||||,A|synonymous
NST00000332826.6|protein_coding|4/23|c.307delC|p.His103fs|392/2542|307/2349|103/782||,T|ups
GH|LRRC56|ENSG00000161328|transcript|ENST00000270115.7|protein_coding|12/13|c.1182-3_118
696|transcript|ENST00000176183.5|protein_coding|3/4|c.849_850insGGCCTCCCCCGG|p.Ser284fs|8
t|HIGH|DRD4|ENSG00000069696|transcript|ENST00000176183.5|protein_coding|3/4|c.850_851insC
t|HIGH|DRD4|ENSG00000069696|transcript|ENST00000176183.5|protein_coding|3/4|c.850_851insC
t|ENST00000526170.5|protein_coding|6/6|c.622delA|p.Thr208fs|624/1047|622/663|208/220||WAR
0000177156|transcript|ENST00000528070.5|nonsense_mediated_decay|1/5|c.98-3dupT|||||INFO_
0000177156|transcript|ENST00000528070.5|nonsense_mediated_decay|1/5|c.98-3dupT|||||INFO_
0000177156|transcript|ENST00000528070.5|nonsense_mediated_decay|1/5|c.98-3dupT|||||INFO_
0177685|transcript|ENST00000528542.6|protein_coding|1/8|c.-349+2A>T||||||,T|upstream_gene_va
r_variant&intron_variant|HIGH|TSPAN4|ENSG00000214063|transcript|ENST00000530404.5|protein_
r_variant&intron_variant|HIGH|TSPAN4|ENSG00000214063|transcript|ENST00000530404.5|protein_

ipt|ENST00000621226.2|protein_coding|31/49|c.13498_13499insGT|p.Ala4500fs|13546/17448|1349
ipt|ENST00000621226.2|protein_coding|31/49|c.13498_13499insGT|p.Ala4500fs|13546/17448|1349
t&intron_variant|HIGH|MUC5AC|ENSG00000215182|transcript|ENST00000621226.2|protein_coding|
|ENST00000621226.2|protein_coding|37/49|c.15476_15485delACACTGTGAT|p.His5159fs|15523/174
raction|2XSN:A_199-A_497:ENST00000381178|protein_coding|14/14|c.1491C>T||||||,A|structural_i
raction|2XSN:A_233-A_259:ENST00000381178|protein_coding|7/14|c.777G>A||||||,T|structural_inte
000456145.2|protein_coding|1/5|c.3G>A|p.Met1?|55/1366|3/537|1/178||,T|5_prime_UTR_variant|
000339046.8|nonsense_mediated_decay|1/9|c.1A>G|p.Met1?|104/1202|1/432|1/143||,G|start_los
intron_variant|HIGH|SLC22A18|ENSG00000110628|transcript|ENST00000449603.5|processed_transc
nteraction|3HNC:B_284-B_327:ENST00000300738|protein_coding|9/19|c.850C>A||||||,A|synonymo
nteraction|3HNC:A_713-A_741:ENST00000300738|protein_coding|19/19|c.2223A>G||||||,G|sequen
ENST00000624801.3|polymorphic_pseudogene|1/1|c.121delC|p.Leu41fs|212/1124|121/945|41/314
ipt|ENST00000307616.1|protein_coding|4/4|c.599_600dupGT|p.Met201fs|600/654|600/654|200/21
ript|ENST00000307616.1|protein_coding|4/4|c.597_598dupCT|p.Cys200fs|598/654|598/654|200/21
ot|ENST00000307616.1|protein_coding|2/4|c.285dupC|p.Lys96fs|285/654|285/654|95/217||
00000307616.1|protein_coding|1/4|c.95G>A|p.Trp32*|95/654|95/654|32/217||
ENST00000380383.1|protein_coding|1/1|c.295delC|p.Arg99fs|295/960|295/960|99/319||,C|framesh
transcript|ENST00000380378.1|protein_coding|1/1|c.632_633insCACCACCC|p.Glu212fs|633/1065|63
0000380370.1|protein_coding|1/1|c.907C>T|p.Arg303*|907/936|907/936|303/311||
:|ENST00000445557.1|polymorphic_pseudogene|1/1|c.777dupC|p.Ile260fs|778/926|778/926|260/30
t|ENST00000328942.1|protein_coding|1/1|c.804dupG|p.Ser269fs|804/939|804/939|268/312||,AC|f
ENST00000510471.2|polymorphic_pseudogene|1/1|c.23delG|p.Gly8fs|23/897|23/897|8/298||,G|int
0000224091|transcript|ENST00000418080.1|antisense|1/1|n.47+1G>A||||||,A|upstream_gene_varia
eraction|1DXT:B_3-B_133:ENST00000335295|protein_coding|1/3|c.9T>C||||||,G|structural_interacti
ENST00000300773.3|protein_coding|1/1|c.197_213delCCACAGACCTGGGGCTG|p.Ala66fs|213/939|19
ript|ENST00000332043.1|protein_coding|1/1|c.567_570dupATCT|p.Cys191fs|571/951|571/951|191/
00000300778.4|protein_coding|1/1|c.706C>T|p.Arg236*|796/1096|706/954|236/317||,T|intron_vari
0000380211.1|protein_coding|1/1|c.43C>T|p.Gln15*|43/945|43/945|15/314||,A|intron_variant|MC
t|ENST00000341449.3|protein_coding|1/1|c.714_715dupCA|p.Asn239fs|716/939|716/939|239/312|
script|ENST00000322641.5|protein_coding|1/1|c.605_608dupGGCT|p.Thr204fs|631/1065|609/957|2
00000317254.3|protein_coding|1/1|c.514A>T|p.Arg172*|562/1037|514/966|172/321||,T|intron_var
|ENST00000317078.1|protein_coding|1/1|c.240delT|p.Asn82fs|240/963|240/963|80/320||,G|intron_
00537935.1|protein_coding|1/1|c.954A>T|p.Ter318Tyrext*?|986/1061|954/954|318/317||,A|intron_
00000532411.2|protein_coding|1/1|c.502C>T|p.Arg168*|502/942|502/942|168/313||,A|stop_gained
000170955|transcript|ENST00000524852.1|retained_intron|1/1|n.64-1G>T||||||,A|missense_variant
intron_variant|HIGH|APBB1|ENSG00000166313|transcript|ENST00000311051.7|protein_coding|7/14
0000254579.10|protein_coding|25/43|c.8782C>T|p.Arg2928*|9346/14862|8782/14262|2928/4753||
n|3F6Q:A_99-B_62:ENST00000299421-ENST00000332345|protein_coding|4/13|c.297C>T||||||,T|pro
NST00000317834.4|protein_coding|1/1|c.357delC|p.Leu121fs|385/1070|357/993|119/330||,T|intro
00000283415&ENSG00000149054|gene_variant|ENSG00000283415|||n.6926415_6926397delTCCCTT
|interaction|4N1J:A_22-A_55:ENST00000299481|protein_coding|2/12|c.164G>A||||||,A|structural_i
ENST00000315204.5|protein_coding|14/14|c.1512_1513insT|p.Gly505fs|2035/2707|1512/1545|504/
cript|ENST00000315204.5|protein_coding|14/14|c.1511_1512insTGGTT|p.Ala506fs|2034/2707|1511/
NSG00000254900|transcript|ENST00000534169.1|antisense|1/1|n.291-1G>C||||||,C|upstream_gene_
H|IPO7|ENSG00000205339|transcript|ENST00000379719.7|protein_coding|21/24|c.2490-3dupT|||||
H|IPO7|ENSG00000205339|transcript|ENST00000379719.7|protein_coding|21/24|c.2490-3dupT|||||
H|IPO7|ENSG00000205339|transcript|ENST00000379719.7|protein_coding|21/24|c.2490-3dupT|||||

H|IPO7|ENSG00000205339|transcript|ENST00000379719.7|protein_coding|21/24|c.2490-3dupT|||||
H|IPO7|ENSG00000205339|transcript|ENST00000379719.7|protein_coding|21/24|c.2490-3dupT|||||
_coding_transcript_exon_variant|HIGH|SBF2-AS1|ENSG00000246273|transcript|ENST00000499953.6|
33805|transcript|ENST00000444303.6|protein_coding|1/13|c.-278+2C>T||||||,T|splice_donor_varia
000133805|transcript|ENST00000530864.1|processed_transcript|1/5|n.37-3dupT||||||INFO_REALIGN
00177112|transcript|ENST00000529829.5|antisense|1/2|n.271+1G>T||||||,T|splice_donor_variant&
0072952|transcript|ENST00000534266.6|protein_coding|1/18|c.-670-2G>A||||||,T|splice_acceptor_v
ENSG00000254991|transcript|ENST00000476130.2|lincRNA|1/1|n.76-3dupT||||||INFO_REALIGN_3_
|ENSG00000254991|transcript|ENST00000476130.2|lincRNA|1/1|n.76-4_76-3dupTT||||||INFO_REAL
3|ENSG00000254991|transcript|ENST00000476130.2|lincRNA|1/1|n.76-5_76-3dupTTT||||||INFO_RE
3.3|ENSG00000254991|transcript|ENST00000476130.2|lincRNA|1/1|n.76-6_76-3dupTTTT||||||INFO_
18.3|ENSG00000254991|transcript|ENST00000476130.2|lincRNA|1/1|n.76-7_76-3dupTTTTT||||||INF
ot|ENST00000256186.2|protein_coding|3/9|c.1193_1194insA|p.Ser399fs|1485/3044|1194/2088|398/
0000197702|transcript|ENST00000526746.5|processed_transcript|2/3|n.163-3dupT||||||INFO_REAL
0000197702|transcript|ENST00000526746.5|processed_transcript|2/3|n.163-3dupT||||||INFO_REAL
0000197702|transcript|ENST00000526746.5|processed_transcript|2/3|n.163-3dupT||||||INFO_REAL
nteraction|2ZOT:A_44-A_128:ENST00000576479|protein_coding|1/16|c.132C>T||||||,T|structural_in
interaction|3Q13:A_231-A_288:ENST00000576479|protein_coding|7/16|c.864A>G||||||,G|synonymo
H|COPB1|ENSG00000129083|transcript|ENST00000529866.5|protein_coding|1/3|c.-58+2_-58+4delTA
29083|transcript|ENST00000249923.7|protein_coding|1/21|c.-58+2T>G||||||,C|5_prime_UTR_variar
|interaction|3C6G:B_59-B_482:ENST00000334636|protein_coding|1/5|c.177C>T||||||,A|structural_in
IGH|RP11-452G18.1|ENSG00000213779|transcript|ENST00000496042.1|processed_pseudogene|1/1|
:|ENST00000338965.8|protein_coding|5/5|c.1345_1346delTT|p.Leu449fs|1589/6438|1345/1365|449
0006071|transcript|ENST00000528202.5|processed_transcript|2/2|n.114-1G>A||||||,T|upstream_ge
nteraction|3K1R:A_135-A_163:ENST00000526313|nonsense_mediated_decay|5/20|c.403G>A||||||,T
000399391.6|protein_coding|19/55|c.2500C>T|p.Gln834*|2500/8778|2500/8778|834/2925||,T|stop
0000265965.9|protein_coding|3/11|c.285C>A|p.Cys95*|437/1566|285/1377|95/458||,T|stop_gaine
T00000396275.2|protein_coding|3/3|c.920G>A|p.Trp307*|1281/1639|920/969|307/322||,A|stop_g
79817|transcript|ENST00000618161.1|protein_coding|2/2|c.789dupT|p.Asn264fs|790/969|790/969|
nteraction|4OJN:F_129-F_157:ENST00000422447|protein_coding|4/8|c.387G>A||||||,A|structural_in
teraction|4OJN:A_135-A_161:ENST00000422447|protein_coding|5/8|c.483C>T||||||,T|structural_int
0535484.5|protein_coding|2/11|c.1A>G|p.Met1?|164/1963|1/1026|1/341||,C|missense_variant|MO
4|interaction|2BKA:A_197-A_227:ENST00000451739|protein_coding|5/5|c.591T>G||||||,G|structura
00000254820|transcript|ENST00000530837.1|lincRNA|1/1|n.34+2T>C|||||
00255323|transcript|ENST00000532380.1|lincRNA|2/3|n.242+2T>C|||||
interaction|3MS5:A_152-A_337:ENST00000528583|protein_coding|8/8|c.1011G>A||||||,A|structura
interaction|3LRE:A_33-A_334:ENST00000263181|protein_coding|7/17|c.1000C>T||||||,A|structural_
000169519|transcript|ENST00000437814.1|nonsense_mediated_decay|4/4|c.*71-1G>A||||||,A|splic
NSG00000254489|transcript|ENST00000525871.1|antisense|1/1|n.72-1G>A||||||,A|upstream_gene_
000280550|transcript|ENST00000628571.1|protein_coding|1/1|c.72+1delC||||||WARNING_TRANSCR
00000527661.5|nonsense_mediated_decay|12/13|c.1738C>T|p.Gln580*|1983/2468|1738/1788|580/
0110422|transcript|ENST00000534262.1|processed_transcript|1/3|n.35-3dupT||||||INFO_REALIGN_
T00000531145.1|protein_coding|2/3|c.196C>T|p.Gln66*|198/291|196/210|66/69||WARNING_TRANS
0216708|transcript|ENST00000405137.1|processed_pseudogene|1/1|n.73-1delA||||||INFO_REALIGN
eraction|1DGG:C_369-C_389:ENST00000241052|protein_coding|9/13|c.1167C>T||||||,T|synonymous
teraction|4M6R:B_125-B_193:ENST00000395787|protein_coding|5/7|c.375A>C||||||,G|structural_in
eraction|4M6R:A_56-A_76:ENST00000395787|protein_coding|4/7|c.227G>A||||||,T|structural_inter

)435|transcript|ENST00000533550.5|protein_coding|1/4|c.-21+1G>C||||||WARNING_TRANSCRIPT_II
)00110435|transcript|ENST00000477173.3|processed_transcript|2/2|n.162-3dupT||||||INFO_REALIG
)435|transcript|ENST00000477173.3|processed_transcript|2/2|n.162-4_162-3delTT||||||INFO_REALI
10435|transcript|ENST00000477173.3|processed_transcript|2/2|n.162-3delT||||||INFO_REALIGN_3_
)26508|transcript|ENST00000442151.6|protein_coding|8/8|c.879-1G>A||||||WARNING_TRANSCRIPT
10436|transcript|ENST00000395750.5|protein_coding|2/11|c.-11+1G>A||||||,T|intron_variant|MOD
5349|transcript|ENST00000534663.1|nonsense_mediated_decay|3/9|c.-412+1G>A||||||,A|downstre
)0255171|transcript|ENST00000529282.1|lincRNA|5/5|n.491+1G>C||||||,C|splice_donor_variant&in
|interaction|2IUW:A_208-A_242:ENST00000302708|protein_coding|9/10|c.726G>T||||||,T|structura
ISG00000254409|transcript|ENST00000528226.1|sense_overlapping|2/3|n.177-1G>C||||||,C|upstre
500000254409|transcript|ENST00000526408.1|sense_overlapping|5/6|n.502+1G>T||||||
H|ACCS|ENSG00000110455|transcript|ENST00000531190.5|retained_intron|4/4|n.599+1_599+9delGT
)0000255032|transcript|ENST00000530039.1|lincRNA|1/2|n.288+2C>T||||||,A|intron_variant|MODI
|ENST00000354556.8|nonsense_mediated_decay|7/9|c.705delG|p.Ter237fs|1149/1954|705/711|235
:|ENST00000354556.8|nonsense_mediated_decay|6/9|c.416dupA|p.Ala140fs|860/1954|416/711|139
|ENST00000531928.6|protein_coding|6/6|c.398delG|p.Gly133fs|670/1078|398/465|133/154||,G|TF_
)00157613|transcript|ENST00000530518.1|protein_coding|4/4|c.312-1G>C||||||WARNING_TRANSCR
NST00000489525.5|nonsense_mediated_decay|4/5|c.462_463delCC|p.Arg155fs|612/1339|462/471|
NST00000489525.5|nonsense_mediated_decay|4/5|c.462_463delCC|p.Arg155fs|612/1339|462/471|
NST00000489525.5|nonsense_mediated_decay|4/5|c.462_463delCC|p.Arg155fs|612/1339|462/471|
NST00000489525.5|nonsense_mediated_decay|4/5|c.462_463delCC|p.Arg155fs|612/1339|462/471|
NST00000489525.5|nonsense_mediated_decay|4/5|c.462_463delCC|p.Arg155fs|612/1339|462/471|
)00175224|transcript|ENST00000528494.5|protein_coding|1/17|c.-13-3dupT||||||INFO_REALIGN_3_
)00175224|transcript|ENST00000528494.5|protein_coding|1/17|c.-13-3dupT||||||INFO_REALIGN_3_
action|3P70:B_546-B_608:ENST00000311907|protein_coding|14/14|c.1824C>T||||||,T|structural_in
I|PACSIN3|ENSG00000165912|transcript|ENST00000298838.10|protein_coding|2/10|c.-38+2_-38+4d
nteraction|4E54:B_246-B_291:ENST00000256996|protein_coding|6/10|c.738G>A||||||,A|structural_i
4574|transcript|ENST00000620515.1|processed_transcript|3/3|n.283+1G>C||||||,C|splice_donor_va
000311027.9|protein_coding|13/36|c.2296C>T|p.Arg766*|2461/5990|2296/4944|766/1647||,T|stop
.|interaction|2V6H:A_194-A_236:ENST00000545968|protein_coding|6/35|c.706A>G||||||,C|structura
.|interaction|2V6H:A_164-A_253:ENST00000545968|protein_coding|4/35|c.492C>T||||||,A|structura
ENST00000533968.1|protein_coding|4/4|c.610_611dupTG|p.Met205fs|745/1290|611/738|204/245|
0000264583&ENSG00000255197|gene_variant|ENSG00000255197|||n.47401030_47400953delCCAG
)0000213619|transcript|ENST00000528192.5|protein_coding|4/4|c.382-3dupT||||||INFO_REALIGN_3
)0000213619|transcript|ENST00000528192.5|protein_coding|4/4|c.382-3dupT||||||INFO_REALIGN_3
ce_acceptor_variant&intron_variant|HIGH|MTCH2|ENSG00000109919|transcript|ENST00000302503.
ce_acceptor_variant&intron_variant|HIGH|MTCH2|ENSG00000109919|transcript|ENST00000302503.
)000624868.1|polymorphic_pseudogene|1/1|c.81C>G|p.Tyr27*|129/1025|81/912|27/303||
|ENST00000624868.1|polymorphic_pseudogene|1/1|c.81C>T|p.Tyr27Tyr|129/1025|81/912|27/303||
)000320048.1|protein_coding|1/1|c.819T>A|p.Tyr273*|819/918|819/918|273/305||
)0220948|transcript|ENST00000534741.1|unprocessed_pseudogene|3/5|n.738+1G>C||||||
nteraction|2C6C:A_115-A_353:ENST00000256999|protein_coding|9/19|c.1059A>G||||||,C|structura
nteraction|2C6C:A_111-A_357:ENST00000256999|protein_coding|3/19|c.333A>T||||||,A|structural_
5|transcript|ENST00000525826.5|nonsense_mediated_decay|2/17|c.223T>C|p.Ter75Glnext*?|416/2
_coding_transcript_exon_variant|HIGH|RP11-163O19.1|ENSG00000254714|transcript|ENST00000534
)0417545.4|protein_coding|5/6|c.673T>C|p.Ter225Argext*?|759/1445|673/675|225/224||,C|downs
)0000623907.1|polymorphic_pseudogene|1/1|c.49C>T|p.Gln17*|49/933|49/933|17/310||

)0000302231.4|protein_coding|1/1|c.469C>T|p.Gln157*|494/1045|469/933|157/310||,A|sequence_
)000314612.2|protein_coding|1/1|c.189C>G|p.Tyr63*|189/939|189/939|63/312||
riant|HIGH|RP11-738O11.13|ENSG00000254547|transcript|ENST00000533973.1|unprocessed_pseud
NST00000302124.6|protein_coding|1/1|c.497delT|p.Leu166fs|497/933|497/933|166/310||INFO_RE
NST00000302124.6|protein_coding|1/1|c.524delA|p.His175fs|524/933|524/933|175/310||,C|downs
000302124.6|protein_coding|1/1|c.867C>G|p.Tyr289*|867/933|867/933|289/310||,G|downstream_
pt|ENST00000440231.1|polymorphic_pseudogene|1/1|c.466_467dupCT|p.Tyr157fs|468/985|468/98
)0000528616.3|protein_coding|1/1|c.378T>A|p.Tyr126*|378/918|378/918|126/305||
|ENST00000526812.3|protein_coding|1/1|c.347_354delCTTCAATG|p.Ala116fs|354/948|347/948|116
ENST00000526538.1|protein_coding|1/1|c.429_432delTCTG|p.Cys143fs|432/948|429/948|143/315|
)0000624596.1|polymorphic_pseudogene|1/1|c.55C>T|p.Gln19*|79/1026|55/933|19/310||
NST00000312153.1|protein_coding|1/1|c.181_194delTTCACTGGAAATCT|p.Phe61fs|181/918|181/91
500000254662|transcript|ENST00000534162.1|antisense|1/1|n.95+1G>A||||||,A|upstream_gene_va
198561|transcript|ENST00000524630.5|protein_coding|1/18|c.-214+1G>A||||||,A|upstream_gene_
ENST00000302622.3|protein_coding|1/1|c.685delC|p.Leu229fs|708/1002|685/954|229/317||,A|ups
4|transcript|ENST00000624470.1|polymorphic_pseudogene|1/1|c.978A>G|p.Ter326Trpext*?|978/97
NST00000309403.2|protein_coding|1/1|c.90delC|p.Phe31fs|90/946|90/945|30/314||
ENST00000302572.2|protein_coding|1/1|c.589delA|p.Ile197fs|611/1054|589/945|197/314||
186660|transcript|ENST00000316059.6|protein_coding|4/10|c.618-1G>A||||||,A|splice_acceptor_v
t|ENST00000343597.3|protein_coding|4/4|c.394delT|p.Tyr132fs|585/3488|394/2205|132/734||INF
801|transcript|ENST00000531147.1|protein_coding|2/2|c.782_791dupGCAGATACTT|p.Phe264fs|142
225802|transcript|ENST00000533936.1|unprocessed_pseudogene|1/2|n.192+1G>A||||||
ENST00000300127.2|protein_coding|1/1|c.697delC|p.Arg233fs|720/1054|697/945|233/314||
)0000530162.1|protein_coding|1/1|c.691G>T|p.Glu231*|748/1024|691/936|231/311||
)0000307552.2|protein_coding|1/1|c.367C>T|p.Gln123*|386/1042|367/930|123/309||
0214788|transcript|ENST00000398992.2|unitary_pseudogene|8/9|n.996-2G>A||||||
on_coding_transcript_exon_variant|HIGH|RP11-736I10.2|ENSG00000255331|transcript|ENST0000052
t&non_coding_transcript_exon_variant|HIGH|RP11-736I10.2|ENSG00000255331|transcript|ENST0000
nt|LOW|RP11-736I10.2|ENSG00000255331|transcript|ENST00000528891.1|processed_pseudogene|2
L-736I10.2|ENSG00000255331|transcript|ENST00000528891.1|processed_pseudogene|2/2|n.386_38
00110077|transcript|ENST00000527254.5|nonsense_mediated_decay|3/6|c.283-1G>A||||||,T|upstr
|ENST00000300187.10|protein_coding|2/5|c.167_168delTT|p.Ile56fs|444/2997|167/2040|56/679||,
00166928|transcript|ENST00000530662.5|nonsense_mediated_decay|4/5|c.*77-2A>G||||||,G|splic
)0000016913.8|protein_coding|2/7|c.211C>T|p.Gln71*|268/1167|211/804|71/267||,T|stop_gained|
interaction|4LG8:A_273-A_318:ENST00000227524|protein_coding|11/16|c.954C>T||||||,A|synonym
teraction|2B5L:B_373-B_1012:ENST00000301764|protein_coding|9/27|c.1119G>A||||||,T|synonym
nteraction|3EI1:A_51-A_664:ENST00000301764|protein_coding|2/27|c.153C>T||||||,A|structural_int
)0000187049|transcript|ENST00000334888.9|protein_coding|4/4|c.432-2_432-1insA||||||,CA|down
)000187049|transcript|ENST00000515837.6|protein_coding|4/4|c.432-1G>C||||||,C|splice_acceptor
500000124915|transcript|ENST00000536405.5|lincRNA|5/5|n.1817+2T>C||||||,G|splice_donor_varia
)00529309.5|protein_coding|22/22|c.2647C>T|p.Gln883*|2720/2909|2647/2754|883/917||,A|stop_
teraction|1JKG:B_403-B_466:ENST00000294172|protein_coding|14/21|c.1209A>G||||||,C|structural
231|transcript|ENST00000533048.5|processed_transcript|1/8|n.117+1G>A||||||,T|upstream_gene_
T00000612278.4|protein_coding|9/10|c.1503T>G|p.Tyr501*|1945/2101|1503/1659|501/552||,C|sto
T00000612278.4|protein_coding|7/10|c.1225C>T|p.Gln409*|1667/2101|1225/1659|409/552||,A|sto
0197658|transcript|ENST00000326192.5|protein_coding|1/3|c.402+1G>A||||||,T|splice_donor_varia
)000525295.1|nonsense_mediated_decay|2/7|c.471G>C|p.Ter157Tyrext*?|471/1362|471/471|157/1

T00000533483.5|nonsense_mediated_decay|2/11|c.132G>A|p.Trp44*|298/2234|132/1119|44/372|
t|ENST00000279178.3|protein_coding|6/10|c.1005dupA|p.Pro336fs|1255/2343|1006/1662|336/55
t|ENST00000301790.4|protein_coding|1/6|c.69dupC|p.Lys24fs|229/1166|69/840|23/279||,TGG|fra
nt&intron_variant|HIGH|MARK2|ENSG00000072518|transcript|ENST00000543220.5|protein_coding|
57770|transcript|ENST00000543988.1|protein_coding|1/6|c.-33+1G>T||||||,T|intron_variant|MODIF
ENST00000535246.1|protein_coding|1/5|c.-10_2delACTTATTATGAA|p.Asn1fs||1/500|1/165||WARNI
nteraction|3OHM:B_225-B_734:ENST00000279230|protein_coding|8/31|c.675C>T||||||,T|structural_
nteraction|3OHM:B_404-B_696:ENST00000279230|protein_coding|11/31|c.1212G>A||||||,A|structu
nteraction|3OHM:B_726-B_845:ENST00000279230|protein_coding|21/31|c.2534C>T||||||,T|structur
t|ENST00000615925.1|miRNA|1/1|n.-19_*5delACCAGACATGGGGTTGTGGTCTGGGGTCTTGGGCCATCTG
nteraction|3U84:A_418-A_554:ENST00000312049|protein_coding|9/10|c.1254C>T||||||,A|structural
H|ATG2A|ENSG00000110046|transcript|ENST00000377264.7|protein_coding|25/40|c.3535+2_3535+
teraction|3DOE:A_115-A_150:ENST00000246747|protein_coding|5/5|c.450C>T||||||,T|structural_int
on_coding_transcript_exon_variant|HIGH|AP000436.4|ENSG00000270775|transcript|ENST000006052
on_coding_transcript_exon_variant|HIGH|AP000436.4|ENSG00000270775|transcript|ENST000006052
on_coding_transcript_exon_variant|HIGH|AP000436.4|ENSG00000270775|transcript|ENST000006052
on_coding_transcript_exon_variant|HIGH|AP000436.4|ENSG00000270775|transcript|ENST000006052
NST00000322147.8|protein_coding|1/27|c.150_156delCGCAGGC|p.Ala51fs|156/4046|150/3771|50/1
teraction|1NFI:A_207-A_291:ENST00000406246|protein_coding|8/11|c.871G>A||||||,T|structural_in
757|transcript|ENST00000534784.1|protein_coding|1/1|c.-49+2T>G|||||WARNING_TRANSCRIPT_IN
757|transcript|ENST00000525451.6|protein_coding|1/4|c.-532+1G>A||||||,T|splice_donor_variant&
72757|transcript|ENST00000532134.5|protein_coding|1/4|c.-215-1G>A|||||WARNING_TRANSCRIPT
interaction|4P0P:A_304-A_355:ENST00000308110|protein_coding|11/16|c.1065G>A||||||,A|structu
interaction|4P0P:A_310-A_416:ENST00000308110|protein_coding|12/16|c.1248G>T||||||,T|structur
00172732|transcript|ENST00000308110.8|protein_coding|14/15|c.1506-3dupC|||||INFO_REALIGN_
NST00000307886.7|protein_coding|1/10|c.29_36delTCCTGGCC|p.Leu10fs|75/1300|29/1131|10/376|
teraction|3FVY:A_678-A_702:ENST00000541961|protein_coding|17/18|c.2033G>A||||||,A|structura
000513398.1|protein_coding|15/21|c.1729C>T|p.Arg577*|1826/2939|1729/2706|577/901||,T|stop_
interaction|2YU1:A_351-A_481:ENST00000529006|protein_coding|11/21|c.1053T>C||||||,C|structu
nteraction|3KRW:A_32-A_212:ENST00000308595|protein_coding|1/21|c.96C>A||||||,A|synonymous_
interaction|3A1J:A_48-A_261:ENST00000307980|protein_coding|9/11|c.781G>A||||||,A|structural_i
action|3V4Y:A_255-B_207:ENST00000376745-ENST00000311772|protein_coding|6/7|c.765C>T|||||
|transcript|ENST00000312457.2|protein_coding|1/1|c.1120_1127dupCCACAGCT|p.Asn377fs|1132/14
T00000545682.5|nonsense_mediated_decay|3/7|c.250C>T|p.Arg84*|297/1063|250/579|84/192||,A
0132746|transcript|ENST00000349015.7|protein_coding|1/9|c.-245+2T>G||||||,C|upstream_gene_v
0|interaction|4B3G:A_237-A_368:ENST00000255078|protein_coding|8/15|c.1104C>T||||||,T|structu
interaction|2W9Z:A_170-A_223:ENST00000227507|protein_coding|4/5|c.669C>T||||||,T|synonymou
0000528853.1|protein_coding|2/2|c.179G>A|p.Trp60*|181/745|179/294|60/97||WARNING_TRANSC
00533931.1|nonsense_mediated_decay|4/5|c.382C>T|p.Arg128*|382/567|382/387|128/128||WARN
00162105|transcript|ENST00000445654.2|processed_transcript|1/3|n.84-2G>A||||||,T|intron_variar
ENST00000458632.2|protein_coding|5/5|c.583delC|p.Arg195fs|585/635|583/633|195/210||WARNI
ranscript|ENST00000458632.2|protein_coding|5/5|c.554_555insGGCGAGT|p.Pro186fs|556/635|554/
0000355527.7|protein_coding|6/9|c.452G>A|p.Trp151*|729/2666|452/1428|151/475||,T|stop_gaine
nt&intron_variant|HIGH|KRTAP5-10|ENSG00000204572|transcript|ENST00000376536.5|protein_codir
.37497|transcript|ENST00000541641.5|protein_coding|3/6|c.-33+1G>A|||||WARNING_TRANSCRIPT
00184154|transcript|ENST00000541614.5|protein_coding|4/4|c.438-2A>C||||||,C|3_prime_UTR_var
NST00000611028.2|protein_coding|3/5|c.320_321delAT|p.Tyr107fs|343/820|320/738|107/245||INF

nteraction|3ITU:C_591-C_617:ENST00000334456|protein_coding|21/31|c.1849A>G||||||,C|missense
0000536885.1|protein_coding|4/4|c.388C>T|p.Arg130*|389/639|388/534|130/177||WARNING_TRAN
IGH|RELT|ENSG00000054967|transcript|ENST00000545687.5|protein_coding|2/7|c.-182-2_-180delA
000175582&ENSG00000255928|gene_variant|ENSG00000175582|||n.73722353_73722326delAAAAA
000175582&ENSG00000255928|gene_variant|ENSG00000175582|||n.73722353_73722328delAAAAA
000175582&ENSG00000255928|gene_variant|ENSG00000175582|||n.73722353_73722328delAAAAA
000175582&ENSG00000255928|gene_variant|ENSG00000175582|||n.73722349_73722330delAAAAA
000175582&ENSG00000255928|gene_variant|ENSG00000175582|||n.73722349_73722330delAAAAA
000175582&ENSG00000255928|gene_variant|ENSG00000175582|||n.73722349_73722330delAAAAA
000175582&ENSG00000255928|gene_variant|ENSG00000175582|||n.73722349_73722330delAAAAA
000175582&ENSG00000255928|gene_variant|ENSG00000175582|||n.73722352_73722332delAAAAA
000175582&ENSG00000255928|gene_variant|ENSG00000175582|||n.73722352_73722332delAAAAA
000175582&ENSG00000255928|gene_variant|ENSG00000175582|||n.73722352_73722332delAAAAA
000175582&ENSG00000255928|gene_variant|ENSG00000175582|||n.73722352_73722332delAAAAA
000175582&ENSG00000255928|gene_variant|ENSG00000175582|||n.73722352_73722332delAAAAA
000175582&ENSG00000255928|gene_variant|ENSG00000175582|||n.73722353_73722334delAAAAA
000175582&ENSG00000255928|gene_variant|ENSG00000175582|||n.73722353_73722334delAAAAA
000175582&ENSG00000255928|gene_variant|ENSG00000175582|||n.73722353_73722334delAAAAA
000175582&ENSG00000255928|gene_variant|ENSG00000175582|||n.73722353_73722334delAAAAA
000175582&ENSG00000255928|gene_variant|ENSG00000175582|||n.73722350_73722336delAAAAA
000175582&ENSG00000255928|gene_variant|ENSG00000175582|||n.73722350_73722336delAAAAA
000175582&ENSG00000255928|gene_variant|ENSG00000175582|||n.73722350_73722336delAAAAA
000175582&ENSG00000255928|gene_variant|ENSG00000175582|||n.73722350_73722336delAAAAA
000175582&ENSG00000255928|gene_variant|ENSG00000175582|||n.73722350_73722338delAAAAA
000175582&ENSG00000255928|gene_variant|ENSG00000175582|||n.73722350_73722338delAAAAA
000175582&ENSG00000255928|gene_variant|ENSG00000175582|||n.73722350_73722338delAAAAA
000175582&ENSG00000255928|gene_variant|ENSG00000175582|||n.73722350_73722338delAAAAA
0000175582&ENSG00000255928|gene_variant|ENSG00000175582|||n.73722352_73722341delAAAA
0000175582&ENSG00000255928|gene_variant|ENSG00000175582|||n.73722352_73722341delAAAA
00000540162.5|nonsense_mediated_decay|4/9|c.132T>A|p.Cys44*|227/1004|132/159|44/52||,A|in
GH|C2CD3|ENSG00000168014|transcript|ENST00000366334.3|retained_intron|1/1|n.460-3_460-2de
ENST00000535954.1|protein_coding|1/3|c.165dupC|p.Ser56fs|165/574|165/574|55/190||WARNING
ENST00000535954.1|protein_coding|1/3|c.165dupC|p.Ser56fs|165/574|165/574|55/190||WARNING
0000427714.2|protein_coding|13/13|c.1678C>T|p.Gln560*|1707/2248|1678/1815|560/604||,A|3_pr
0000531449.5|nonsense_mediated_decay|2/8|c.172C>T|p.Arg58*|174/1588|172/189|58/62||WARN
|ENSG00000149243|transcript|ENST00000539798.1|protein_coding|1/6|c.44_45insGGCGCCGTGCGCG
0085741|transcript|ENST00000529461.1|protein_coding|2/2|c.-92+2_-92+3insC|||||WARNING_TRA
nt&intron_variant|HIGH|MYO7A|ENSG00000137474|transcript|ENST00000620575.4|protein_coding|2
|ENST00000376217.6|protein_coding|12/17|c.1191dupT|p.Ile398fs|1442/2502|1191/1872|397/623|
|ENST00000532481.5|protein_coding|4/4|c.255dupT|p.Leu86fs|346/690|256/267|86/88||INFO_REA
teraction|3N2Z:B_421-B_444:ENST00000313010|protein_coding|9/9|c.1331C>G||||||,C|missense_va
teraction|3N2Z:B_299-B_334:ENST00000313010|protein_coding|7/9|c.1002T>C||||||,G|synonymou
teraction|3N2Z:B_112-B_482:ENST00000313010|protein_coding|3/9|c.336A>C||||||,G|missense_va
ot|ENST00000529073.5|protein_coding|6/8|c.479dupT|p.Leu160fs|485/719|479/627|160/208||,TA|
00000255250|transcript|ENST00000532979.1|lincRNA|1/4|n.168+2C>T|||||
GH|HNRNPCP8|ENSG00000254748|transcript|ENST00000528764.1|processed_pseudogene|1/1|n.47
int|HIGH|HNRNPCP8|ENSG00000254748|transcript|ENST00000528764.1|processed_pseudogene|1/1

|ENSG00000254748|transcript|ENST00000528764.1|processed_pseudogene|1/1|n.476-2delA||||||IN
P8|ENSG00000254748|transcript|ENST00000528764.1|processed_pseudogene|1/1|n.476-2dupA||||
CP8|ENSG00000254748|transcript|ENST00000528764.1|processed_pseudogene|1/1|n.476-3_476-2du
134612|transcript|ENST00000525540.1|processed_transcript|9/13|n.1134+1G>A||||||,A|splice_donc
:|ENST00000329862.6|protein_coding|1/6|c.377delG|p.Ser126fs|377/2689|377/1350|126/449||
|ENST00000530464.2|protein_coding|1/1|c.772dupT|p.Trp258fs|773/1182|773/1182|258/393||INFO
ENST00000530464.2|protein_coding|1/1|c.997_1003delCTGCAAG|p.Leu333fs|997/1182|997/1182|33
.6|interaction|3FEC:A_101-A_347:ENST00000534061|protein_coding|3/19|c.301G>A||||||,A|structu
G00000255127|transcript|ENST00000528208.2|unprocessed_pseudogene|3/4|n.608+2C>G||||||,G|u
020922|transcript|ENST00000407439.7|protein_coding|2/19|c.29+1G>A||||||,T|upstream_gene_var
56037|transcript|ENST00000538095.1|nonsense_mediated_decay|2/3|c.*25+2T>G||||||,G|upstream
|interaction|1LW3:A_502-A_576:ENST00000346299|protein_coding|13/15|c.1504G>C||||||,G|struct
|interaction|1M7R:B_315-B_411:ENST00000346299|protein_coding|11/15|c.1233G>A||||||,T|struct
G00000087053|transcript|ENST00000346299.9|protein_coding|9/14|c.994-2_994-1insACAA||||||,CT
nt&intron_variant|HIGH|MTMR2|ENSG00000087053|transcript|ENST00000346299.9|protein_coding|
G00000087053|transcript|ENST00000346299.9|protein_coding|6/14|c.571-2_571-1insACCT||||||,CA
GH|MTMR2|ENSG00000087053|transcript|ENST00000470293.5|processed_transcript|3/5|n.283-11_
GH|MTMR2|ENSG00000087053|transcript|ENST00000470293.5|processed_transcript|3/5|n.283-11_
GH|MTMR2|ENSG00000087053|transcript|ENST00000470293.5|processed_transcript|3/5|n.283-11_
nt&intron_variant|HIGH|MTMR2|ENSG00000087053|transcript|ENST00000472423.5|processed_tran
0000256684|transcript|ENST00000543403.1|lincRNA|3/3|n.151+2T>C||||||,C|downstream_gene_var
0000524871.5|protein_coding|4/25|c.157C>T|p.Arg53*|447/6258|157/3303|53/1100||,T|stop_gaine
ertion|HIGH|CNTN5|ENSG00000149972|transcript|ENST00000524871.5|protein_coding|4/25|c.265_
eraction|3G8O:A_800-A_886:ENST00000325455|protein_coding|8/8|c.2658A>G||||||,C|structural_ir
0000236826.7|protein_coding|10/10|c.1348C>T|p.Gln450*|1447/3056|1348/1404|450/467||,A|3_pr
00166670|transcript|ENST00000279441.8|protein_coding|3/9|c.497-2A>G||||||,C|upstream_gene_v
|interaction|1Q3A:A_119-A_162:ENST00000279441|protein_coding|3/10|c.486T>C||||||,G|structura
|interaction|1Q3A:A_112-A_147:ENST00000279441|protein_coding|2/10|c.336C>T||||||,A|synonym
nteraction|1SU3:A_277-A_461:ENST00000315274|protein_coding|6/10|c.831G>A||||||,T|synonymo
NST00000315274.6|protein_coding|4/10|c.573delT|p.Ile191fs|641/1970|573/1410|191/469||,C|intr
nteraction|3E4C:A_280-A_328:ENST00000436863|protein_coding|7/10|c.984C>T||||||,A|structural_
nteraction|2F2S:A_157-A_288:ENST00000265838|protein_coding|6/12|c.471C>A||||||,A|structural_
00149311|transcript|ENST00000527805.5|protein_coding|9/26|c.1236-3dupT||||||WARNING_TRANS
00149311|transcript|ENST00000527805.5|protein_coding|9/26|c.1236-3dupT||||||WARNING_TRANS
00149311|transcript|ENST00000527805.5|protein_coding|9/26|c.1236-3dupT||||||WARNING_TRANS
214290|transcript|ENST00000526216.1|protein_coding|1/4|c.-285+2C>T||||||,T|upstream_gene_var
ript|ENST00000304987.3|protein_coding|15/15|c.2472delA|p.Pro825fs|2645/9678|2472/2781|824/9
ST00000304987.3|protein_coding|15/15|c.2517_2518delAG|p.Ala840fs|2690/9678|2517/2781|839/9
000150776|transcript|ENST00000526879.5|protein_coding|1/4|c.-38-1G>A||||||WARNING_TRANSCR
|interaction|3MTR:A_540-A_571:ENST00000621128|protein_coding|12/17|c.1620T>G||||||,G|struct
9292|transcript|ENST00000442859.5|protein_coding|2/9|c.-16+1G>A||||||WARNING_TRANSCRIPT_
0149292|transcript|ENST00000496311.1|retained_intron|1/2|n.278-2A>G||||||,G|upstream_gene_va
nteraction|2IIP:A_8-A_142:ENST00000299964|protein_coding|3/3|c.426T>C||||||,C|structural_intera
000544582.1|protein_coding|5/5|c.505C>T|p.Arg169*|511/692|505/663|169/220||,T|downstream_g
ENSG00000225236|transcript|ENST00000447151.1|processed_pseudogene|2/2|n.333-5_333-2dupTAT
5|ENSG00000225236|transcript|ENST00000447151.1|processed_pseudogene|2/2|n.333-7_333-2dup
pt|ENST00000445164.6|protein_coding|2/12|c.256_257delCC|p.Pro86fs|331/2361|256/1761|86/586

ot|ENST00000445164.6|protein_coding|2/12|c.256_257delCC|p.Pro86fs|331/2361|256/1761|86/586
pt|ENST00000445164.6|protein_coding|2/12|c.242_254delCAGCCCAGGCATC|p.Pro81fs|328/2361|24
pt|ENST00000445164.6|protein_coding|2/12|c.242_254delCAGCCCAGGCATC|p.Pro81fs|328/2361|24
NST00000623963.1|protein_coding|1/3|c.54G>A|p.Trp18*|54/942|54/942|18/313||,T|intron_variant
000137648|transcript|ENST00000437212.7|protein_coding|1/12|c.4-1A>G||||||,G|splice_acceptor_
NST00000278937.6|protein_coding|2/6|c.72delA|p.Ile24fs|201/2620|72/648|24/215||,C|frameshift_
:|ENST00000533102.5|protein_coding|9/9|c.1133_1140delTGTGGAAG|p.Val378fs|1193/1410|1133/1
0000137700|transcript|ENST00000357590.9|protein_coding|5/12|c.528delG|p.Val177fs|969/2356|52
ion_variant&intron_variant|HIGH|C2CD2L|ENSG00000172375|transcript|ENST00000336702.7|protein
nteraction|3UN9:A_793-A_822:ENST00000292199|protein_coding|9/10|c.2378C>A||||||,A|structura
eraction|1B47:B_101-B_171:ENST00000264033|protein_coding|3/16|c.513T>C||||||,C|structural_int
teraction|2IBI:A_346-A_367:ENST00000260187|protein_coding|5/13|c.1038C>T||||||,A|structural_in
_coding_transcript_exon_variant|HIGH|TRIM29|ENSG00000137699|transcript|ENST00000524956.4|p
37699|transcript|ENST00000524956.4|processed_transcript|1/3|n.47+2C>A||||||,T|upstream_gene_
nteraction|3WSX:A_247-A_269:ENST00000260197|protein_coding|6/48|c.807T>C||||||,C|synonymo
nteraction|3WSX:A_681-A_734:ENST00000260197|protein_coding|16/48|c.2200G>A||||||,A|missen
00000254710|transcript|ENST00000531681.1|antisense|2/4|n.221+1G>T||||||,T|intron_variant|MO
00000638086.1|protein_coding|1/20|c.1A>G|p.Met1?|198/2665|1/2304|1/767||,G|intron_variant|M
00000318666.6|protein_coding|1/1|c.756T>G|p.Tyr252*|810/1030|756/939|252/312||
0000341493.3|protein_coding|1/1|c.777C>G|p.Tyr259*|777/936|777/936|259/311||,G|sequence_fe
|ENST00000354597.3|protein_coding|1/1|c.550dupC|p.Leu184fs|567/987|550/942|184/313||
000444566.5|protein_coding|4/4|c.369G>A|p.Trp123*|505/560|369/424|123/140||WARNING_TRAN
NSG00000245498|transcript|ENST00000499143.6|antisense|3/4|n.1100-2A>G||||||,G|splice_accept
165495|transcript|ENST00000558729.5|processed_transcript|1/4|n.206+2C>T||||||,T|splice_donor_
00577924.1|protein_coding|2/5|c.181C>T|p.Gln61*|182/619|181/618|61/205||WARNING_TRANSCR
000427383.6|protein_coding|1/12|c.236G>A|p.Trp79*|271/1670|236/1479|79/492||,A|stop_gainec
00149554|transcript|ENST00000428830.6|protein_coding|13/13|c.*28-3dupT||||||INFO_REALIGN_3_
0237353|transcript|ENST00000457514.2|protein_coding|1/2|c.59-2A>C||||||,C|downstream_gene_v
ction|3N1F:B_90-D_875:ENST00000295731-ENST00000392693|protein_coding|14/20|c.2623A>G||||
nteraction|4LQD:A_147-A_180:ENST00000392678|protein_coding|5/5|c.539C>T||||||,T|structural_in
000150455|transcript|ENST00000479770.1|nonsense_mediated_decay|3/3|c.*953-3dupT||||||INFO_
000150455|transcript|ENST00000479770.1|nonsense_mediated_decay|3/3|c.*953-3dupT||||||INFO_
000150455|transcript|ENST00000479770.1|nonsense_mediated_decay|3/3|c.*953-3dupT||||||INFO_
10063|interaction|1XMM:C_45-C_77:ENST00000263579|protein_coding|2/6|c.225_230dupGGATGG|
HIGH|KIRREL3-AS3|ENSG00000218109|transcript|ENST00000404882.3|pseudogene|1/1|n.426+1dupC
teraction|3MFK:A_337-A_396:ENST00000319397|protein_coding|7/8|c.1011A>G||||||,C|structural_i
T00000531399.5|protein_coding|2/4|c.95C>A|p.Ser32*|305/806|95/375|32/124||,T|stop_gained|HI
000084234|transcript|ENST00000532456.5|processed_transcript|1/3|n.247-3dupT||||||INFO_REALIG
000084234|transcript|ENST00000532456.5|processed_transcript|1/3|n.247-3dupT||||||INFO_REALIG
000084234|transcript|ENST00000532456.5|processed_transcript|1/3|n.247-3dupT||||||INFO_REALIG
7O18.1|ENSG00000255358|transcript|ENST00000526902.1|processed_pseudogene|1/1|n.244-1G>A|
7O18.1|ENSG00000255358|transcript|ENST00000526902.1|processed_pseudogene|1/1|n.244-1G>A|
G00000254842|transcript|ENST00000533434.1|lincRNA|1/3|n.52+2T>C||||||,G|intron_variant|MODI
000227125|transcript|ENST00000447864.1|lincRNA|1/1|n.239+1G>A|||||
000255512|transcript|ENST00000532071.1|lincRNA|2/2|n.547+2T>G|||||
111181|transcript|ENST00000538272.5|processed_transcript|1/4|n.239+2C>T||||||,A|sequence_fea
|ENST00000436453.1|protein_coding|2/2|c.219delT|p.Val74fs|272/1101|219/294|73/97||,C|sequen

ST00000322843.3|protein_coding|13/13|c.1808G>A|p.Trp603*|1824/1926|1808/1910|603/635||WA
ENST00000340908.8|protein_coding|9/28|c.2175dupC|p.Ile726fs|2819/11208|2176/7905|726/2634|
ENST00000340908.8|protein_coding|9/28|c.2220dupC|p.Phe741fs|2864/11208|2221/7905|741/2634
ENST00000340908.8|protein_coding|9/28|c.2264dupC|p.Pro756fs|2908/11208|2265/7905|755/2634
000358495.7|protein_coding|12/12|c.1245T>G|p.Tyr415*|1384/2710|1245/1257|415/418||,C|stop_
000358495.7|protein_coding|11/12|c.1037C>A|p.Ser346*|1176/2710|1037/1257|346/418||,T|stop_
ENST00000461568.5|nonsense_mediated_decay|5/12|c.354_355insA|p.Gly119fs|432/1531|354/420
000496818.2|protein_coding|1/3|c.2T>C|p.Met1?|115/545|2/432|1/143||WARNING_TRANSCRIPT_N
nteraction|1QZ2:A_197-A_288:ENST00000001008|protein_coding|5/10|c.591C>A||||||,A|missense_
71792|transcript|ENST00000366285.5|protein_coding|2/3|c.-85+1G>A||||||WARNING_TRANSCRIPT_
H|TEAD4|ENSG00000197905|transcript|ENST00000359864.6|protein_coding|1/12|c.-123+2_-123+7de
ENST00000382622.3|protein_coding|2/10|c.95_96delAG|p.Gln32fs|485/2366|95/1185|32/394||,C|fr
ENST00000545990.6|protein_coding|5/6|c.2102dupG|p.Asp702fs|2627/3318|2102/2562|701/853||,
NST00000545990.6|protein_coding|5/6|c.2098delT|p.Ser700fs|2623/3318|2098/2562|700/853||,G|
t|ENST00000542998.5|protein_coding|4/4|c.315dupA|p.Cys106fs|318/577|316/483|106/160||WARI
teraction|3GXB:A_1547-A_1571:ENST00000261405|protein_coding|28/52|c.4641T>C||||||,G|structu
00000255775|transcript|ENST00000539206.1|lincRNA|1/2|n.223+1G>A||||||
|ENST00000011684.11|protein_coding|15/16|c.1909_1910delGA|p.Asp637fs|2057/2881|1909/2373
interaction|1U8F:O_105-O_127:ENST00000229239|protein_coding|6/9|c.381T>C||||||,C|structural_
interaction|1U8F:O_288-O_315:ENST00000229239|protein_coding|8/9|c.864C>T||||||,T|structural_
interaction|1U8F:O_275-O_295:ENST00000229239|protein_coding|8/9|c.885T>C||||||,C|structural_
110811|transcript|ENST00000536140.5|retained_intron|10/15|n.2088dupA||||||INFO_REALIGN_3_P
110811|transcript|ENST00000536140.5|retained_intron|10/15|n.2088dupA||||||INFO_REALIGN_3_P
teraction|3IHP:A_59-A_128:ENST00000389231|protein_coding|4/20|c.384C>T||||||,T|structural_inte
11669|transcript|ENST00000482209.1|retained_intron|1/1|n.227-1G>A||||||,A|upstream_gene_vari
111671|transcript|ENST00000524270.5|protein_coding|1/2|c.-96-1G>A||||||,T|splice_acceptor_vari
1671|transcript|ENST00000432205.5|protein_coding|3/3|c.-97+2C>T||||||WARNING_TRANSCRIPT_I
nteraction|2B3O:A_326-A_453:ENST00000456013|protein_coding|9/16|c.978T>C||||||,C|structural_
ENST00000261407.8|protein_coding|4/13|c.433_434delCA|p.His145fs|520/2268|433/1464|145/487|
0205885|transcript|ENST00000435921.6|antisense|1/2|n.61+1G>C||||||,C|upstream_gene_variant|
000399422.4|protein_coding|7/13|c.1069C>T|p.Gln357*|1117/2142|1069/1743|357/580||,T|down
|ENST00000539726.5|protein_coding|1/5|c.1_2delTC|p.Ser1fs|3/815|1/327|1/108||WARNING_TRAN
|ENST00000539726.5|protein_coding|1/5|c.1_2delTC|p.Ser1fs|3/815|1/327|1/108||WARNING_TRAN
00000229304.4|protein_coding|3/5|c.62G>A|p.Trp21*|83/879|62/711|21/236||,T|missense_varian
transcript|ENST00000229307.8|protein_coding|4/4|c.916_917delTG|p.Ter306fs|1135/5183|916/918
t|ENST00000538603.5|protein_coding|4/7|c.61delC|p.Gln21fs|620/2513|61/1395|21/464||,T|frame
0164845|transcript|ENST00000338711.9|transcribed_unprocessed_pseudogene|7/7|n.875+1G>C||||
97614|transcript|ENST00000544211.5|nonsense_mediated_decay|9/9|c.*61+1G>T||||||,A|splice_do
_coding_transcript_exon_variant|HIGH|RP11-20D14.6|ENSG00000249790|transcript|ENST0000051456
00000249790|transcript|ENST00000514568.2|lincRNA|1/1|n.487+2C>T||||||,T|intron_variant|MODI
00166535|transcript|ENST00000299698.11|protein_coding|25/35|c.3108-3dupT||||||INFO_REALIGN
ENST00000299698.11|protein_coding|29/36|c.3676_3677delGC|p.Ala1226fs|3856/5274|3676/4365|
GH|A2M|ENSG00000175899|transcript|ENST00000318602.11|protein_coding|17/35|c.2126-6_2126-
0261336.6|protein_coding|15/36|c.1792C>T|p.Gln598*|1821/4610|1792/4449|598/1482||,A|3_prin
56069|transcript|ENST00000545343.5|processed_transcript|3/3|n.483+1G>A||||||,T|splice_donor_v
56069|transcript|ENST00000566278.6|transcribed_unprocessed_pseudogene|6/31|n.784+1G>T|||||
00000256594|transcript|ENST00000574035.5|processed_transcript|1/4|n.162+2C>T||||||,T|upstrea

G00000256594|transcript|ENST00000611052.4|processed_transcript|2/4|n.229+1G>A||||||,A|splice_
NSG00000256594|transcript|ENST00000545363.6|processed_transcript|3/4|n.509-3dupT||||||INFO_
NSG00000256594|transcript|ENST00000545363.6|processed_transcript|3/4|n.509-3dupT||||||INFO_
script|ENST00000327839.3|protein_coding|1/2|c.149_153dupACTTA|p.Ser52fs|188/670|153/504|51/
0000304084.12|protein_coding|6/6|c.714T>G|p.Tyr238*|869/1130|714/744|238/247||,C|stop_gain
60138|transcript|ENST00000541351.3|processed_transcript|3/5|n.367-1G>A||||||,T|sequence_feat
teraction|4A0P:A_1018-A_1062:ENST00000261349|protein_coding|14/23|c.3184G>A||||||,T|missen
teraction|3S94:B_420-B_448:ENST00000261349|protein_coding|6/23|c.1344C>G||||||,C|synonymou
ipt|ENST00000535902.5|protein_coding|4/4|c.839_840insTT|p.Thr281fs|1403/5831|839/1296|280/4
t|ENST00000535902.5|protein_coding|4/4|c.838_839insT|p.Thr280fs|1402/5831|838/1296|280/43
00000271215|transcript|ENST00000605378.1|processed_pseudogene|1/3|n.127-1dupA||||||,CT|int
178878|transcript|ENST00000326765.10|protein_coding|1/1|c.96+1G>A||||||,A|splice_donor_variar
13782|transcript|ENST00000392155.2|processed_transcript|1/1|n.431+2C>T||||||,T|upstream_gene_
00432710.6|protein_coding|5/6|c.601C>T|p.Gln201*|734/1231|601/1020|201/339||,A|stop_gained
00180861|transcript|ENST00000318426.6|lincRNA|1/2|n.198+1G>T||||||,A|splice_donor_variant&in
|ENST00000330828.2|protein_coding|2/2|c.550_551delAT|p.Met184fs|754/1684|550/738|184/245
nteraction|4F9Z:A_167-A_221:ENST00000266397|protein_coding|5/7|c.501A>G||||||,C|structural_ir
interaction|2GJT:A_1012-A_1137:ENST00000281171|protein_coding|22/27|c.3036G>A||||||,A|struct
139144|transcript|ENST00000546003.5|nonsense_mediated_decay|26/31|c.*3077+1G>A||||||,A|sp
00266497.9|protein_coding|31/31|c.4337G>C|p.Ter1446Serext*?|4375/4764|4337/4338|1446/1445
nscript|ENST00000261196.6|protein_coding|2/14|c.205_209dupATTGA|p.Asp70fs|277/2840|210/210
|ENST00000307378.10|protein_coding|6/16|c.382_385delAACA|p.Asn128fs|1106/7682|382/2013|12
0000256021|transcript|ENST00000540463.1|processed_pseudogene|1/1|n.309-2A>C||||||,G|intron_
on_coding_transcript_exon_variant|HIGH|RP11-283G6.4|ENSG00000256234|transcript|ENST0000054
00123104|transcript|ENST00000381340.7|protein_coding|13/56|c.1409+2_1409+3insGAGT||||||
G00000256377|transcript|ENST00000542660.1|antisense|1/2|n.133+1A>G||||||,C|splice_donor_vari
00536154.5|protein_coding|3/6|c.1A>G|p.Met1?|170/549|1/380|1/125||WARNING_TRANSCRIPT_IN
00000257456|transcript|ENST00000549070.1|antisense|2/2|n.243+2G>T||||||,T|upstream_gene_va
110888|transcript|ENST00000540584.1|protein_coding|1/4|c.-40+2T>A||||||WARNING_TRANSCRIPT
G00000235884|transcript|ENST00000547804.1|lincRNA|1/4|n.287-3dupT||||||INFO_REALIGN_3_PRI
G00000235884|transcript|ENST00000547804.1|lincRNA|1/4|n.287-3dupT||||||INFO_REALIGN_3_PRI
00000226472|transcript|ENST00000613860.4|processed_transcript|4/5|n.401+1G>A||||||,T|3_prim
0000539702.1|protein_coding|1/6|c.60G>A|p.Trp20*|62/951|60/516|20/171||WARNING_TRANSCRIP
258278|transcript|ENST00000547527.2|unprocessed_pseudogene|5/9|n.709+1G>A||||||
258278|transcript|ENST00000547527.2|unprocessed_pseudogene|9/9|n.1190+1G>A||||||
00205592|transcript|ENST00000454784.8|processed_transcript|17/171|n.1985-1G>A||||||,A|intron_
05592|transcript|ENST00000454784.8|processed_transcript|34/171|n.4258+2T>C||||||,C|intron_var
05592|transcript|ENST00000454784.8|processed_transcript|90/171|n.20222+1delG||||||INFO_REAL
00205592|transcript|ENST00000454784.8|processed_transcript|131/171|n.22275-1G>A||||||,A|splic
00134283|transcript|ENST00000256678.12|protein_coding|3/10|c.2_4delTGA|p.Met1del|183/1820|2
0139174|transcript|ENST00000547113.1|protein_coding|1/3|c.-49+1G>A||||||WARNING_TRANSCRIP
T00000384129.2|snRNA|1/1|n.-2_*10delATGTGCTCGCTTCGGCAGCACATATACTAAAATTGGAACGATACA
0189079|transcript|ENST00000426776.5|processed_transcript|3/3|n.236-2A>G||||||,G|intron_variar
61273|transcript|ENST00000080059.11|protein_coding|13/25|c.1678+2T>G||||||,C|splice_donor_va
49336.5|protein_coding|3/10|c.2T>C|p.Met1?|153/4644|2/1284|1/427||,G|start_lost|HIGH|VDR|E
G00000257985|transcript|ENST00000546738.1|antisense|2/3|n.393-2G>A||||||,A|downstream_ger
00547581.5|nonsense_mediated_decay|6/26|c.475C>T|p.Arg159*|516/3579|475/531|159/176||,T|

pt|ENST00000310248.3|protein_coding|1/1|c.200dupT|p.Ser68fs|295/1305|200/954|67/317||,CA|d
|ENSG00000257735|transcript|ENST00000548257.1|antisense|2/3|n.98-3dupT|||||INFO_REALIGN_3
|ENSG00000257735|transcript|ENST00000548257.1|antisense|2/3|n.98-3dupT|||||INFO_REALIGN_3
|ENSG00000257735|transcript|ENST00000548257.1|antisense|2/3|n.98-3dupT|||||INFO_REALIGN_3
00257987|transcript|ENST00000548380.5|protein_coding|1/3|c.79+1G>A|||||,A|splice_donor_varia
0000552878.5|protein_coding|6/6|c.493C>T|p.Arg165*|576/1052|493/549|165/182||,A|downstrear
ion|2WG3:A_181-C_379:ENST00000266991-ENST00000296575|protein_coding|2/3|c.543C>T|||||,A
00000257771|transcript|ENST00000547443.1|lincRNA|4/4|n.412+1G>A|||||,A|splice_donor_variant
nteraction|4NEF:A_140-A_167:ENST00000199280|protein_coding|2/4|c.501T>C|||||,C|structural_int
ript|ENST00000327337.5|protein_coding|4/8|c.4753_4754insTC|p.Gln1585fs|4753/7127|4753/7056
t|HIGH|FAM186A|ENSG00000185958|transcript|ENST00000327337.5|protein_coding|4/8|c.4751_47
t|HIGH|FAM186A|ENSG00000185958|transcript|ENST00000327337.5|protein_coding|4/8|c.4751_47
000549625.1|nonsense_mediated_decay|4/6|c.241T>C|p.Ter81Argext*?|349/585|241/243|81/80||,C
ENST00000293636.1|protein_coding|7/8|c.628dupC|p.Leu210fs|669/952|628/777|210/258||
00139610|transcript|ENST00000293636.1|protein_coding|1/8|c.12_15delTTAT|p.Tyr5fs|56/952|12/7
:|ENST00000293636.1|protein_coding|1/8|c.7_8insCT|p.Val3fs|48/952|7/777|3/258||,AAG|downstre
ENST00000293636.1|protein_coding|1/8|c.6_7insC|p.Val3fs|47/952|6/777|2/258||,CG|downstream_
0167612|transcript|ENST00000547119.1|processed_transcript|5/5|n.649+1G>A|||||,A|upstream_ge
35477|transcript|ENST00000529785.1|processed_transcript|1/8|n.698+1G>T|||||,A|splice_donor_v
000293670.3|protein_coding|4/9|c.666C>A|p.Cys222*|729/1875|666/1482|222/493||
|ENST00000293670.3|protein_coding|4/9|c.666C>T|p.Cys222Cys|729/1875|666/1482|222/493||
000293670.3|protein_coding|3/9|c.601G>T|p.Glu201*|664/1875|601/1482|201/493||
000332411.2|protein_coding|3/9|c.826G>T|p.Glu276*|880/2529|826/1917|276/638||
6442|transcript|ENST00000417996.2|protein_coding|8/8|c.1570+2dupT|||||
teraction|3HQC:A_1281-A_1383:ENST00000314250|protein_coding|29/29|c.4149C>T|||||,T|structu
teraction|2JIS:A_283-A_382:ENST00000444623|protein_coding|14/17|c.1144G>A|||||,T|structural_
NST00000429243.6|protein_coding|4/4|c.439delT|p.Ser147fs|647/1229|439/447|147/148||INFO_RE
0139625|transcript|ENST00000547035.5|protein_coding|1/13|c.-38+2T>A|||||,T|upstream_gene_v
0111481|transcript|ENST00000552848.5|protein_coding|1/5|c.-81-2A>G|||||WARNING_TRANSCRIP
0549043.5|protein_coding|2/9|c.1A>G|p.Met1?|96/1232|1/558|1/185||,G|start_lost|HIGH|COPZ1|
H|GTSF1|ENSG00000170627|transcript|ENST00000549083.1|processed_transcript|1/2|n.501+1_501+
nteraction|1TAZ:A_374-A_492:ENST00000243052|protein_coding|14/16|c.1476T>C|||||,C|structura
T00000546807.5|nonsense_mediated_decay|3/6|c.214delC|p.His72fs|404/773|214/234|72/77||,T|i
G00000257634|transcript|ENST00000552524.1|lincRNA|2/2|n.346-2A>G|||||
0000528240.1|protein_coding|2/2|c.418C>T|p.Gln140*|418/580|418/450|140/149||WARNING_TRA
NST00000305377.5|protein_coding|1/1|c.38delT|p.Leu13fs|126/1122|38/1008|13/335||INFO_REAL
00000343870.4|protein_coding|1/1|c.184C>T|p.Arg62*|274/1105|184/939|62/312||
ENST00000379668.3|protein_coding|1/1|c.24dupA|p.Glu9fs|64/1064|25/939|9/312||INFO_REALIGN
ENST00000328314.3|protein_coding|1/1|c.933delA|p.Lys311fs|933/939|933/939|311/312||INFO_RI
tion|1H1P:A_28-D_247:ENST00000266970-ENST00000274026|protein_coding|1/7|c.84G>A|||||,A|p
ction|2GWF:A_244-B_219:ENST00000307179-ENST00000345093|protein_coding|7/7|c.657G>A|||||
000308197.9|protein_coding|1/5|c.3G>T|p.Met1?|264/1652|3/744|1/247||,T|sequence_feature|MC
transcript|ENST00000614328.4|protein_coding|2/9|c.1_2delAT|p.Met1fs|448/2077|1/495|1/164||,C
interaction|4BPU:A_330-A_350:ENST00000338193|protein_coding|10/13|c.990A>C|||||,G|structura
ENST00000622922.1|protein_coding|1/1|c.118_119delCA|p.Gln40fs|118/591|118/591|40/196||INFO
00000622922.1|protein_coding|1/1|c.438G>A|p.Trp146*|438/591|438/591|146/196||,A|3_prime_UT
0000551452.5|protein_coding|1/3|c.2T>C|p.Met1?|13/533|2/522|1/173||WARNING_TRANSCRIPT_N

)0135502|transcript|ENST00000474359.6|nonsense_mediated_decay|17/17|c.*458+2T>G||||||,G|m
27|interaction|4QPN:A_176-A_218:ENST00000300209|protein_coding|3/3|c.527T>C||||||,C|missens
G00000257921|transcript|ENST00000546504.1|protein_coding|3/3|c.288+2C>T||||||WARNING_TRA
00000257259|transcript|ENST00000550678.1|lincRNA|2/7|n.340+2T>C||||||,G|intron_variant|MOD
pt|ENST00000549033.5|nonsense_mediated_decay|4/7|c.334dupA|p.Arg112fs|483/1878|335/426|1
NSG00000257294|transcript|ENST00000552378.2|lincRNA|1/1|n.47-3dupT||||||INFO_REALIGN_3_P
ENSG00000257294|transcript|ENST00000552378.2|lincRNA|1/1|n.47-4_47-3dupTT||||||INFO_REALI
|ENSG00000257294|transcript|ENST00000552378.2|lincRNA|1/1|n.47-5_47-3dupTTT||||||INFO_RE
)0061987|transcript|ENST00000393630.7|protein_coding|30/34|c.4515-3dupT||||||INFO_REALIGN_
00000255583|transcript|ENST00000543539.1|transcribed_unprocessed_pseudogene|1/3|n.157+1T>C
)000196935|transcript|ENST00000355086.7|protein_coding|14/21|c.1679-3dupT||||||INFO_REALIGI
)000196935|transcript|ENST00000355086.7|protein_coding|14/21|c.1679-3dupT||||||INFO_REALIGI
ENSG00000198671|transcript|ENST00000360528.3|antisense|1/2|n.141-3dupT||||||INFO_REALIGN_
00000198671|transcript|ENST00000360528.3|antisense|1/2|n.141-3delT||||||INFO_REALIGN_3_PRI
teraction|2YGN:A_104-A_153:ENST00000286574|protein_coding|4/10|c.459T>C||||||,G|structural_i
ST00000247815.8|protein_coding|4/13|c.1566delG|p.Glu522fs|1625/12866|1566/3264|522/1087||,
)111605|transcript|ENST00000550075.5|nonsense_mediated_decay|2/3|c.271-2A>G||||||,G|intron_
ction|3P5T:A_158-M_118:ENST00000300291-ENST00000266679|protein_coding|3/11|c.354G>A|||||
NST00000331471.8|protein_coding|3/10|c.177delA|p.Lys59fs|349/1506|177/1197|59/398||,G|fram
27328|transcript|ENST00000549760.1|protein_coding|1/2|c.-26+2C>T||||||WARNING_TRANSCRIPT_
000111596|transcript|ENST00000553078.5|processed_transcript|2/3|n.201-3dupT||||||INFO_REALIC
000111596|transcript|ENST00000553078.5|processed_transcript|2/3|n.201-3dupT||||||INFO_REALIC
nteraction|2AHS:A_1829-A_1849:ENST00000261266|protein_coding|28/32|c.5547C>T||||||,A|struct
nteraction|2AHS:A_1783-A_1813:ENST00000261266|protein_coding|28/32|c.5439T>C||||||,G|struct
teraction|4KNG:B_59-B_83:ENST00000266674|protein_coding|1/18|c.177G>A||||||,A|synonymous_
teraction|4KNG:A_195-A_218:ENST00000266674|protein_coding|6/18|c.653A>G||||||,G|structural_
teraction|4KNG:A_270-A_294:ENST00000266674|protein_coding|9/18|c.882G>A||||||,A|structural_
)292|transcript|ENST00000550851.5|retained_intron|12/19|n.1451+1G>C||||||,C|splice_donor_varia
teraction|4KNG:A_400-A_424:ENST00000266674|protein_coding|14/18|c.1270C>G||||||,G|structura
teraction|4KNG:A_405-A_428:ENST00000266674|protein_coding|15/18|c.1282G>A||||||,A|structura
teraction|4V06:A_234-A_375:ENST00000333850|protein_coding|9/11|c.1125A>T||||||,T|structural_
)0072657|transcript|ENST00000552503.1|processed_transcript|4/4|n.512-2A>G||||||,G|intron_variar
t|ENST00000550916.5|protein_coding|2/6|c.283delT|p.Cys95fs|330/2609|283/1035|95/344||,A|frar
)0067798|transcript|ENST00000551277.1|processed_transcript|2/2|n.172-3dupT||||||INFO_REALIGI
)0067798|transcript|ENST00000551277.1|processed_transcript|2/2|n.172-3dupT||||||INFO_REALIGI
)0067798|transcript|ENST00000551277.1|processed_transcript|2/2|n.172-3dupT||||||INFO_REALIGI
)0067798|transcript|ENST00000551277.1|processed_transcript|2/2|n.172-3dupT||||||INFO_REALIGI
G00000258084|transcript|ENST00000548963.1|lincRNA|3/5|n.424+1G>A||||||,A|intron_variant|MOI
715|transcript|ENST00000549454.5|processed_transcript|3/5|n.369+1G>A||||||,A|downstream_ger
teraction|4V11:A_358-A_379:ENST00000261205|protein_coding|11/11|c.1074G>T||||||,T|structural_
ENST00000393217.6|protein_coding|27/27|c.5105delA|p.Asn1702fs|5166/5394|5105/5169|1702/17
G00000246363|transcript|ENST00000500381.2|lincRNA|2/2|n.305-1delG||||||
)0547291.1|protein_coding|1/2|c.2T>C|p.Met1?|123/1074|2/771|1/256||,G|downstream_gene_vari
202G11.2|ENSG00000257746|transcript|ENST00000551928.1|lincRNA|4/5|n.406-7_406-3dupTTTGT||
202G11.2|ENSG00000257746|transcript|ENST00000551928.1|lincRNA|4/5|n.406-7_406-3dupTTTGT||
G00000257252|transcript|ENST00000552835.5|antisense|3/3|n.407+2T>C||||||,G|downstream_gene
IGH|MRPL42|ENSG00000198015|transcript|ENST00000552938.1|processed_transcript|1/1|n.282+1_

IGH|MRPL42|ENSG00000198015|transcript|ENST00000552938.1|processed_transcript|1/1|n.282+1_
SG00000258303|transcript|ENST00000551257.1|sense_intronic|1/1|n.32-3dupT||||||INFO_REALIGN
|interaction|3NVN:B_233-B_253:ENST00000258526|protein_coding|1/31|c.759C>T||||||,T|structural
00136040|transcript|ENST00000258526.8|protein_coding|28/30|c.4387-3dupC||||||INFO_REALIGN_
00136040|transcript|ENST00000258526.8|protein_coding|28/30|c.4387-3dupC||||||INFO_REALIGN_
0184752|transcript|ENST00000552205.5|nonsense_mediated_decay|3/4|c.*233+1G>A||||||WARNIN
00000257943|transcript|ENST00000550470.2|processed_transcript|3/8|n.203+1G>A||||||,T|splice_
|interaction|1B59:A_135-A_469:ENST00000323666|protein_coding|4/11|c.405C>T||||||,T|structura
|interaction|1B59:A_284-A_365:ENST00000323666|protein_coding|10/11|c.1095C>A||||||,A|struct
5794|transcript|ENST00000538559.6|lincRNA|13/13|n.2320+2T>C||||||,C|upstream_gene_variant|
0000238105|transcript|ENST00000421840.2|processed_transcript|7/8|n.945-2A>G||||||,C|upstream
196091|transcript|ENST00000551300.5|protein_coding|2/31|c.-348+1A>G||||||,G|intron_variant|
1666|transcript|ENST00000552329.1|nonsense_mediated_decay|3/4|c.*77+1G>C||||||WARNING_T
ranscript|ENST00000549872.5|protein_coding|8/8|c.1196_*2delGACT|p.Ter399fs|1386/2001|1196/1
eraction|1DMW:A_245-A_265:ENST00000553106|protein_coding|7/13|c.735G>A||||||,T|structural_
&ENSG00000171759|gene_variant|ENSG00000171759|||n.102958414_102958394delTGCTGCTGCTGC
&ENSG00000171759|gene_variant|ENSG00000171759|||n.102958414_102958394delTGCTGCTGCTGC
&ENSG00000171759|gene_variant|ENSG00000171759|||n.102958414_102958394delTGCTGCTGCTGC
&ENSG00000171759|gene_variant|ENSG00000171759|||n.102958414_102958394delTGCTGCTGCTGC
14198|transcript|ENST00000551885.2|processed_transcript|4/5|n.671+2T>C||||||,G|upstream_gen
9372|transcript|ENST00000392872.7|protein_coding|8/9|c.964_964+1insA||||||,GAGGAT|splice_do
372|transcript|ENST00000392872.7|protein_coding|8/9|c.964+2T>C||||||,G|splice_donor_variant&i
39372|transcript|ENST00000392872.7|protein_coding|9/9|c.1090_1090+1insTT||||||,ATTGAGAGC|s
39372|transcript|ENST00000392872.7|protein_coding|9/9|c.1090_1090+1insTT||||||,ATTGAGAGC|s
39372|transcript|ENST00000392872.7|protein_coding|9/9|c.1090_1090+1insTT||||||,ATTGAGAGC|s
372|transcript|ENST00000392872.7|protein_coding|9/9|c.1090+1G>T||||||,GAGAGCGTGGAGT|splice
372|transcript|ENST00000392872.7|protein_coding|9/9|c.1090+1G>T||||||,GAGAGCGTGGAGT|splice
1727|transcript|ENST00000550335.5|processed_transcript|1/7|n.19+1G>T||||||,T|intron_variant|
00000013503|transcript|ENST00000228347.8|protein_coding|12/27|c.1102-3dupT||||||INFO_REALI
00000013503|transcript|ENST00000228347.8|protein_coding|12/27|c.1102-3dupT||||||INFO_REALI
00000013503|transcript|ENST00000228347.8|protein_coding|12/27|c.1102-3dupT||||||INFO_REALI
NST00000342331.4|protein_coding|1/1|c.515delG|p.Gly172fs|1346/2260|515/522|172/173||INFO_
eraction|4U07:A_145-A_175:ENST00000552695|protein_coding|3/3|c.524A>C||||||,C|structural_int
nt&intron_variant|HIGH|SELPLG|ENSG00000110876|transcript|ENST00000388962.4|protein_coding|2
teraction|2DU8:A_93-A_135:ENST00000228476|protein_coding|3/11|c.279G>A||||||,A|structural_in
|interaction|3BTY:A_170-A_203:ENST00000343075|protein_coding|4/4|c.608G>A||||||,T|structural_
interaction|2HJW:A_597-A_645:ENST00000338432|protein_coding|11/53|c.1791T>C||||||,C|structu
interaction|2HJW:A_651-A_703:ENST00000338432|protein_coding|12/53|c.1951G>A||||||,A|structu
0000468516.1|protein_coding|3/3|c.166C>T|p.Arg56*|167/1593|166/252|56/83||WARNING_TRANS
0000536398.5|protein_coding|9/9|c.711G>A|p.Trp237*|968/1120|711/735|237/244||,A|stop_gaine
teraction|2R3V:A_52-A_131:ENST00000228510|protein_coding|3/11|c.155G>A||||||,A|structural_in
teraction|2R3V:A_37-A_135:ENST00000228510|protein_coding|5/11|c.405G>A||||||,A|structural_in
nteraction|4DX1:A_265-A_313:ENST00000261740|protein_coding|5/16|c.795C>T||||||,A|structural_
nteraction|4DX1:A_228-A_263:ENST00000261740|protein_coding|5/16|c.789T>C||||||,G|structural_
196510|transcript|ENST00000481473.5|processed_transcript|1/3|n.604+1G>T||||||,A|upstream_ge
interaction|1IT6:B_64-B_269:ENST00000335007|protein_coding|6/7|c.807G>A||||||,T|structural_in
ENST00000552694.1|protein_coding|3/10|c.154delA|p.Ile52fs|291/1651|154/1455|52/484||INFO_

ENST00000377617.7|protein_coding|1/25|c.540delG|p.Gln180fs|702/4702|540/3942|180/1313||,G|
ENST00000377617.7|protein_coding|1/25|c.522delG|p.Gln174fs|684/4702|522/3942|174/1313||,G|
NST00000377617.7|protein_coding|1/25|c.501_520delGCAGCAGCAGCAGCAGCAGC|p.Gln168fs|682/4
00000089022|transcript|ENST00000547915.1|retained_intron|2/3|n.969-1G>A|||||,A|3_prime_UTR
T00000547878.6|nonsense_mediated_decay|5/13|c.216G>A|p.Trp72*|379/1716|216/243|72/80||,T
089127|transcript|ENST00000202917.9|protein_coding|5/5|c.1039-1G>A|||||,A|upstream_gene_va
ST00000551241.5|protein_coding|6/6|c.1072delA|p.Thr358fs|1105/2416|1072/1083|358/360||,T|3
teraction|4S3N:A_65-A_230:ENST00000228928|protein_coding|2/16|c.193C>T|||||,T|missense_var
00228928.11|protein_coding|12/16|c.2530C>T|p.Arg844*|2709/6719|2530/3264|844/1087||,T|ups
342315.8|protein_coding|11/11|c.2159A>G|p.Ter720Trpext*?|2373/3613|2159/2160|720/719||,G|s
144|transcript|ENST00000547974.5|processed_transcript|1/7|n.195+2C>T|||||,T|upstream_gene_
00000257997|transcript|ENST00000546791.1|lincRNA|2/3|n.152+1G>C|||||,C|downstream_gene_
NST00000622220.1|protein_coding|7/7|c.556_559delAAAG|p.Lys186fs|1039/2176|556/696|186/231
ENST00000622220.1|protein_coding|7/7|c.581dupG|p.Tyr195fs|1065/2176|582/696|194/231||INFO
nteraction|4D1N:B_356-B_394:ENST00000317775|protein_coding|6/29|c.1181A>C|||||,G|structural
00000256071|transcript|ENST00000544667.1|antisense|1/1|n.285+1G>A|||||,A|intron_variant|MO
nt&intron_variant|HIGH|PRKAB1|ENSG00000111725|transcript|ENST00000630317.1|protein_coding|
000547736.1|protein_coding|3/4|c.106C>T|p.Arg36*|406/574|106/274|36/90||WARNING_TRANSCR
NST00000453000.1|protein_coding|1/6|c.20_23delCGTG|p.Ala7fs|532/1857|20/921|7/306||INFO_R
NST00000453000.1|protein_coding|1/6|c.20_27delCGTGTGTG|p.Ala7fs|532/1857|20/921|7/306||IN
NST00000453000.1|protein_coding|1/6|c.20_21delCG|p.Ala7fs|532/1857|20/921|7/306||INFO_REA
NST00000453000.1|protein_coding|1/6|c.20_21delCG|p.Ala7fs|532/1857|20/921|7/306||INFO_REA
NST00000453000.1|protein_coding|1/6|c.40_43delTGCG|p.Cys14fs|552/1857|40/921|14/306||INFO_
interaction|2VIG:B_107-B_288:ENST00000242592|protein_coding|3/10|c.321T>C|||||,C|synonymou
nteraction|2VIG:A_79-A_120:ENST00000242592|protein_coding|3/10|c.360C>T|||||,T|structural_in
teraction|4XQ7:A_105-A_136:ENST00000257570|protein_coding|2/6|c.408C>T|||||,A|structural_in
ENST00000392474.6	protein_coding	16/16	c.1614delA	p.Gly539fs	1619/4453	1614/1671	538/556
ENST00000392474.6	protein_coding	16/16	c.1614delA	p.Gly539fs	1619/4453	1614/1671	538/556
ENST00000392474.6	protein_coding	16/16	c.1614delA	p.Gly539fs	1619/4453	1614/1671	538/556
ENST00000392474.6	protein_coding	16/16	c.1614delA	p.Gly539fs	1619/4453	1614/1671	538/556
G00000258435|transcript|ENST00000557474.1|lincRNA|1/1|n.268+1G>T|||||,T|intron_variant|MOD
ENST00000377071.8|protein_coding|1/23|c.83_84delCA|p.Thr28fs|157/4595|83/4011|28/1336||,C|
ENST00000619791.1|protein_coding|11/16|c.3995_3996delGG|p.Arg1332fs|3995/5901|3995/5901|1
ENST00000377037.3|protein_coding|2/2|c.537dupC|p.Asp180fs|675/805|538/654|180/217||INFO_R
NSG00000280389|transcript|ENST00000623601.1|TEC|1/1|n.342-1G>A|||||,T|upstream_gene_varia
teraction|2E3I:A_84-A_120:ENST00000540338|protein_coding|2/25|c.360A>G|||||,C|structural_int
119242|transcript|ENST00000539761.5|protein_coding|1/6|c.-229+1delG|||||WARNING_TRANSCRI
0000179195|transcript|ENST00000337815.8|protein_coding|2/4|c.-757_-757+1insACCT|||||,GACCT
0000179195|transcript|ENST00000337815.8|protein_coding|2/4|c.-757_-757+1insACCT|||||,GACCT
0000179195|transcript|ENST00000337815.8|protein_coding|2/4|c.-757_-757+1insACCT|||||,GACCT
on_variant|HIGH|ZNF664|ENSG00000179195|transcript|ENST00000337815.8|protein_coding|2/4|c.-7
iant&5_prime_UTR_variant&intron_variant|HIGH|ZNF664|ENSG00000179195|transcript|ENST000003
0000544745.1|protein_coding|23/23|c.2761C>T|p.Gln921*|2871/4112|2761/2889|921/962||,A|intro
00189238|transcript|ENST00000537374.1|lincRNA|1/3|n.273+2T>C|||||,C|upstream_gene_variant|
teraction|4IQM:A_324-A_399:ENST00000376649|protein_coding|5/6|c.1197C>T|||||,T|structural_i
NST00000332482.8|protein_coding|2/8|c.961_962delAG|p.Ser321fs|1070/2588|961/2479|321/825|
NST00000389562.6|protein_coding|42/52|c.7532_7539delAGCCGCAG|p.Gln2511fs|7567/12836|7532

ENST00000411988.6|protein_coding|3/8|c.252delG|p.Ser85fs|253/1727|252/1128|84/375||WARNIN
ENST00000411988.6|protein_coding|3/8|c.237delG|p.Gln80fs|238/1727|237/1128|79/375||WARNI
00112787|transcript|ENST00000434748.2|protein_coding|13/16|c.1964-3dupC|||||INFO_REALIGN_
000176894|transcript|ENST00000317479.7|protein_coding|1/5|c.116_120delCCACC|p.Ala39fs|181/9
0454379.2|protein_coding|2/2|c.250T>C|p.Ter84Glnext*?|250/537|250/252|84/83||WARNING_TRA
2609|transcript|ENST00000315585.11|nonsense_mediated_decay|1/15|c.-252+1A>G||||||,C|upstrea
280208|transcript|ENST00000623672.1|unprocessed_pseudogene|5/11|n.732+1dupG|||||
226057|transcript|ENST00000444553.5|transcribed_unprocessed_pseudogene|16/19|n.2215+1G>T||
00187721|transcript|ENST00000433887.1|unprocessed_pseudogene|2/3|n.279-2C>T||||||,A|upstre
SG00000226619|transcript|ENST00000450212.1|antisense|1/1|n.73-1G>A||||||,A|intron_variant|M
0227879|transcript|ENST00000442352.1|unprocessed_pseudogene|4/4|n.479+2T>C|||||
00000237636|transcript|ENST00000454044.1|unprocessed_pseudogene|14/14|n.2591-2A>G|||||
000224976|transcript|ENST00000446672.2|unprocessed_pseudogene|5/20|n.490-1T>G|||||
on_coding_transcript_exon_variant|HIGH|PARP4P2|ENSG00000224976|transcript|ENST00000446672.
000462409.5|nonsense_mediated_decay|14/16|c.970C>T|p.Arg324*|970/1220|970/1014|324/337||
cript|ENST00000462409.5|nonsense_mediated_decay|14/16|c.928_929insTTCGC|p.Gln310fs|928/122
ST00000382848.4|protein_coding|2/2|c.35delG|p.Gly12fs|173/2250|35/681|12/226||,A|frameshift_
nteraction|3F3S:A_7-A_87:ENST00000298248|protein_coding|3/8|c.261T>C||||||,G|synonymous_va
variant|HIGH|IL17D|ENSG00000172458|transcript|ENST00000304920.3|protein_coding|2/3|c.51_88
_gained&splice_region_variant|HIGH|SKA3|ENSG00000165480|transcript|ENST00000400018.7|prote
NSG00000165480|transcript|ENST00000314759.5|protein_coding|8/8|c.1239-2_1239-1insAAGAAAAC
NSG00000165480|transcript|ENST00000314759.5|protein_coding|8/8|c.1239-2_1239-1insAAGAAAAC
NSG00000165480|transcript|ENST00000314759.5|protein_coding|8/8|c.1239-2_1239-1insAAGAAAAC
00000165480|transcript|ENST00000314759.5|protein_coding|6/8|c.916-1_916insCTTTG||||||,CCAA
acceptor_variant&intron_variant|HIGH|SKA3|ENSG00000165480|transcript|ENST00000314759.5|prot
00165480|transcript|ENST00000314759.5|protein_coding|5/8|c.830-1_830insTG||||||,TCA|splice_ac
riant|HIGH|SKA3|ENSG00000165480|transcript|ENST00000314759.5|protein_coding|5/8|c.830-2_83
ST00000314759.5|protein_coding|3/9|c.208delC|p.Gln70fs|333/2919|208/1239|70/412||,T|framesh
00165480|transcript|ENST00000314759.5|protein_coding|2/8|c.166-1_166insCG||||||,CCG|splice_ac
r_variant&intron_variant|HIGH|SKA3|ENSG00000165480|transcript|ENST00000314759.5|protein_coc
00314759.5|protein_coding|1/9|c.79C>T|p.Arg27*|204/2919|79/1239|27/412||,A|stop_gained|HIG
on_coding_transcript_exon_variant|HIGH|ZDHHC20-IT1|ENSG00000236953|transcript|ENST0000041
000280175|transcript|ENST00000624023.1|protein_coding|3/14|c.206+1A>G|||||WARNING_TRANS
ipt|ENST00000623728.1|protein_coding|10/15|c.791delT|p.Leu264fs|791/1212|791/1212|264/403|
G00000205863|transcript|ENST00000382140.2|protein_coding|2/4|c.-15-2_-15-1dupAG||||||,ACT|5
GH|MTMR6|ENSG00000139505|transcript|ENST00000381801.5|protein_coding|6/13|c.727-7_727-2
0234706|transcript|ENST00000425846.1|processed_pseudogene|1/2|n.40+2G>A||||||,T|intron_vari
000237001|transcript|ENST00000627315.2|antisense|2/2|n.120+1G>C||||||,G|splice_donor_variant
3.6|ENSG00000275294|transcript|ENST00000620494.1|lincRNA|4/4|n.455-5_455-2dupTTTA||||||,CT
00000233588|transcript|ENST00000419457.1|processed_pseudogene|1/1|n.518-2delA||||||,C|upstr
0175664|transcript|ENST00000380473.7|protein_coding|3/6|c.313-1G>A||||||,A|splice_acceptor_va
231894|transcript|ENST00000425638.2|unitary_pseudogene|3/15|n.282+2T>C|||||
||WDR95P|ENSG00000231894|transcript|ENST00000425638.2|unitary_pseudogene|4/15|n.523+2_52
00133105|transcript|ENST00000298386.6|protein_coding|12/17|c.1002-2dupA|||||INFO_REALIGN_
00133105|transcript|ENST00000298386.6|protein_coding|12/17|c.1002-2dupA|||||INFO_REALIGN_
00133105|transcript|ENST00000298386.6|protein_coding|12/17|c.1002-2dupA|||||INFO_REALIGN_
000139618|transcript|ENST00000470094.1|nonsense_mediated_decay|3/5|c.457-3dupT|||||WARN

)00139618|transcript|ENST00000470094.1|nonsense_mediated_decay|3/5|c.457-3dupT||||||WARN
000544455.5|protein_coding|27/28|c.9976A>T|p.Lys3326*|10203/10984|9976/10257|3326/3418||,
00000459716.6|nonsense_mediated_decay|2/8|c.240T>G|p.Tyr80*|469/2519|240/411|80/136||,C|
)00083642|transcript|ENST00000315596.14|protein_coding|26/34|c.3057-3dupT||||||INFO_REALIGN
)00083642|transcript|ENST00000315596.14|protein_coding|26/34|c.3057-3dupT||||||INFO_REALIGN
00000235550|transcript|ENST00000445530.1|unprocessed_pseudogene|5/6|n.1271-2C>T||||||
0133103|transcript|ENST00000455146.7|protein_coding|6/18|c.624-3dupT||||||INFO_REALIGN_3_P
SG00000102780|transcript|ENST00000337343.8|protein_coding|5/29|c.623-9_623-3dupTTTTTTT|||||
SG00000102780|transcript|ENST00000337343.8|protein_coding|5/29|c.623-9_623-3dupTTTTTTT|||||
SG00000102780|transcript|ENST00000337343.8|protein_coding|5/29|c.623-9_623-3dupTTTTTTT|||||
SG00000102780|transcript|ENST00000337343.8|protein_coding|5/29|c.623-9_623-3dupTTTTTTT|||||
SG00000102780|transcript|ENST00000337343.8|protein_coding|5/29|c.623-9_623-3dupTTTTTTT|||||
SG00000102780|transcript|ENST00000337343.8|protein_coding|5/29|c.623-9_623-3dupTTTTTTT|||||
2780|transcript|ENST00000498255.6|processed_transcript|17/30|n.2595+2T>C||||||,C|intron_variar
)|interaction|3URF:A_168-A_308:ENST00000398795|protein_coding|5/5|c.924T>C||||||,C|synonymc
cript|ENST00000313640.11|protein_coding|13/13|c.1180_1184dupCCTGA|p.Glu395fs|1249/3160|11
33112|transcript|ENST00000527226.2|protein_coding|1/4|c.-10-2A>G||||||WARNING_TRANSCRIPT_
000170919|transcript|ENST00000412946.6|antisense|3/4|n.511-1G>A||||||,A|splice_acceptor_varia
000378966.3|protein_coding|2/2|c.105C>A|p.Cys35*|521/1842|105/1239|35/412||,A|stop_gained|
000378966.3|protein_coding|2/2|c.749C>A|p.Ser250*|1165/1842|749/1239|250/412||,A|stop_gain
teraction|4P10:A_152-A_226:ENST00000181383|protein_coding|7/11|c.678T>C||||||,G|structural_ir
NST00000417167.2|protein_coding|2/8|c.44_45delAT|p.His15fs|241/911|44/715|15/237||WARNING
)218721.1|protein_coding|1/2|c.3G>A|p.Met1?|3/1239|3/1239|1/412||,A|sequence_feature|LOW|
000282026.1|protein_coding|2/2|c.446G>A|p.Trp149*|781/3760|446/591|149/196||,A|intron_varia
176124&ENSG00000229323|gene_variant|ENSG00000229323|||n.50527456_50527438delCAAGGCCA
00000233672|transcript|ENST00000454605.5|antisense|1/2|n.282+2T>A||||||,T|splice_donor_varia
ript|ENST00000336617.7|protein_coding|11/11|c.925dupA|p.Ile309fs|1325/1629|926/939|309/312|
)123201|transcript|ENST00000532104.1|processed_transcript|1/11|n.156+2T>A||||||,T|downstrean
500000236778|transcript|ENST00000601034.2|antisense|1/1|n.163-3dupT||||||INFO_REALIGN_3_PF
500000236778|transcript|ENST00000601034.2|antisense|1/1|n.163-3dupT||||||INFO_REALIGN_3_PF
)000236778|transcript|ENST00000596180.5|antisense|3/3|n.363-2A>G||||||,G|downstream_gene_v
0136098|transcript|ENST00000610828.4|protein_coding|10/16|c.877delA|p.Thr293fs|1253/2396|87
198384|transcript|ENST00000441562.1|unprocessed_pseudogene|8/13|n.671+2G>A||||||
198384|transcript|ENST00000441562.1|unprocessed_pseudogene|12/13|n.1022+1C>T||||||
ript|ENST00000357495.3|protein_coding|1/1|c.830delA|p.Lys277fs|2537/3011|830/963|277/320||I
)0234787|transcript|ENST00000423442.6|lincRNA|4/4|n.633+1T>G||||||,C|downstream_gene_varia
L.2|ENSG00000277448|transcript|ENST00000615202.1|lincRNA|2/2|n.212-3dupT||||||INFO_REALIGI
L.2|ENSG00000277448|transcript|ENST00000615202.1|lincRNA|2/2|n.212-3dupT||||||INFO_REALIGI
L.2|ENSG00000277448|transcript|ENST00000615202.1|lincRNA|2/2|n.212-3dupT||||||INFO_REALIGI
L.2|ENSG00000277448|transcript|ENST00000615202.1|lincRNA|2/2|n.212-3dupT||||||INFO_REALIGI
L.2|ENSG00000277448|transcript|ENST00000615202.1|lincRNA|2/2|n.212-3dupT||||||INFO_REALIGI
)0226846|transcript|ENST00000428761.1|lincRNA|1/3|n.108+2T>C||||||
0000226846|transcript|ENST00000428761.1|lincRNA|3/3|n.217-2A>C||||||
SG00000118939|gene_variant|ENSG00000118939|||n.75549766_75549780delGGCGGCGGCGGCGAA|
ST00000636525.1|protein_coding|5/6|c.741delT|p.Leu248fs|741/2508|741/816|247/271||INFO_RE
t|ENST00000418532.5|protein_coding|1/7|c.220_221dupGG|p.Leu75fs|282/2855|222/1773|74/590
|ENST00000418532.5|protein_coding|1/7|c.230dupC|p.Leu78fs|291/2855|231/1773|77/590||INFO_

GH|RP11-318G21.4|ENSG00000233379|transcript|ENST00000435281.1|lincRNA|1/1|n.71-5_71-2delT
GH|RP11-318G21.4|ENSG00000233379|transcript|ENST00000435281.1|lincRNA|1/1|n.71-5_71-2delT
GH|RP11-318G21.4|ENSG00000233379|transcript|ENST00000435281.1|lincRNA|1/1|n.71-5_71-2delT
GH|RP11-318G21.4|ENSG00000233379|transcript|ENST00000435281.1|lincRNA|1/1|n.71-5_71-2delT
000234377|transcript|ENST00000606429.5|antisense|4/5|n.528+2T>G||||||,G|splice_donor_variant
00233349|transcript|ENST00000446314.1|lincRNA|2/2|n.168+1G>A||||||
00233528|transcript|ENST00000609962.2|lincRNA|3/3|n.241+1G>A||||||,A|intron_variant|MODIFIE
t|ENST00000376945.3|protein_coding|1/1|c.645_646insCT|p.Ala216fs|995/2778|645/831|215/276|
ENST00000376945.3|protein_coding|1/1|c.644dupC|p.Ala216fs|994/2778|644/831|215/276||,A|syr
ENST00000376945.3|protein_coding|1/1|c.644dupC|p.Ala216fs|994/2778|644/831|215/276||,A|syr
G00000125285|transcript|ENST00000376945.3|protein_coding|1/1|c.642_643insTCTTCTTCCCGCAGT
ENST00000376945.3|protein_coding|1/1|c.640_641insT|p.Ala214fs|990/2778|640/831|214/276||,G
1|transcript|ENST00000543457.5|protein_coding|4/4|c.1014A>T|p.Ter338Cysext*?|1305/1305|1014
NST00000443887.1|protein_coding|2/2|c.327_328delGT|p.Met111fs|327/610|327/444|109/147||W
NST00000443887.1|protein_coding|2/2|c.327_328delGT|p.Met111fs|327/610|327/444|109/147||W
000339105.8|protein_coding|6/9|c.775C>T|p.Arg259*|775/1169|775/1023|259/340||,T|stop_gainec
T00000376335.7|protein_coding|1/3|c.692_698delACCACCA|p.His231fs|985/2983|692/1599|231/53
00175198&ENSG00000270632|gene_variant|ENSG00000270632|||n.100198484_100198469delTGAA
00175198&ENSG00000270632|gene_variant|ENSG00000270632|||n.100198484_100198469delTGAA
00175198&ENSG00000270632|gene_variant|ENSG00000270632|||n.100198484_100198469delTGAA
00175198&ENSG00000270632|gene_variant|ENSG00000270632|||n.100198484_100198469delTGAA
00175198&ENSG00000270632|gene_variant|ENSG00000270632|||n.100198484_100198469delTGAA
80|interaction|4MTL:A_109-A_189:ENST00000267273|protein_coding|3/4|c.325T>C||||||,G|structu
00226620|transcript|ENST00000596398.5|lincRNA|1/1|n.289+1G>A||||||,A|upstream_gene_variant
teraction|3W5O:A_542-A_568:ENST00000356922|protein_coding|2/2|c.1704T>C||||||,G|structural_i
eraction|3W1B:A_9-A_146:ENST00000356922|protein_coding|2/2|c.26C>T||||||,A|structural_intera
SG00000283384|transcript|ENST00000637731.1|protein_coding|4/4|c.*286-2G>A||||||,A|intron_va
on_coding_transcript_exon_variant|HIGH|COL4A2-AS1|ENSG00000232814|transcript|ENST0000041
0000229152|transcript|ENST00000426991.2|sense_intronic|1/1|n.117+1G>C||||||,G|downstream_
G00000225870|transcript|ENST00000422994.1|lincRNA|1/2|n.58-3dupT||||||INFO_REALIGN_3_PRIM
G00000225870|transcript|ENST00000422994.1|lincRNA|1/2|n.58-4_58-3dupTT||||||INFO_REALIGN
NSG00000225870|transcript|ENST00000422994.1|lincRNA|1/2|n.58-5_58-3dupTTT||||||INFO_REALIG
NST00000486806.1|protein_coding|2/2|c.202delC|p.Arg68fs|202/361|202/282|68/93||WARNING_T
t|ENST00000612156.2|protein_coding|8/8|c.5171delC|p.Ala1724fs|5258/9759|5171/5904|1724/196
t|ENST00000612156.2|protein_coding|8/8|c.5165_5169delAACGG|p.Glu1722fs|5256/9759|5165/590
00229373|transcript|ENST00000609661.5|lincRNA|7/7|n.1159+1G>C||||||,C|non_coding_transcript_
.1|ENSG00000283199|transcript|ENST00000636427.1|protein_coding|4/5|c.456+2T>C||||||,G|intron
t|ENST00000550708.1|protein_coding|1/1|c.626delG|p.Arg209fs|698/1085|626/981|209/326||
000222036|transcript|ENST00000552966.5|nonsense_mediated_decay|3/11|c.811-3dupT||||||INFO
000222036|transcript|ENST00000552966.5|nonsense_mediated_decay|3/11|c.811-3dupT||||||INFO
000222036|transcript|ENST00000552966.5|nonsense_mediated_decay|3/11|c.811-3dupT||||||INFO
0176294|transcript|ENST00000557414.1|protein_coding|3/4|c.-207-1G>A||||||WARNING_TRANSCR
ENST00000541415.1|polymorphic_pseudogene|1/1|c.539delT|p.Leu180fs|539/944|539/944|180/313
NST00000315683.1|protein_coding|1/1|c.248_266delTAGATTTGCTCACTGACCA|p.Ile83fs|248/939|24
t|ENST00000357366.3|protein_coding|1/1|c.687dupA|p.Gly230fs|688/1111|688/1038|230/345||INF
00553765.1|polymorphic_pseudogene|1/1|c.679T>C|p.Ter227Glnext*?|679/940|679/940|227/312||
nteraction|1BIX:A_51-A_297:ENST00000216714|protein_coding|3/5|c.153G>C||||||,C|structural_int

nteraction|1BIX:A_50-A_315:ENST00000216714|protein_coding|5/5|c.945C>T||||||,T|structural_inte
eraction|1M73:E_20-E_46:ENST00000361505|protein_coding|2/6|c.60C>T||||||,T|structural_interact
eraction|1M73:E_57-E_85:ENST00000361505|protein_coding|2/6|c.171C>T||||||,T|structural_intera
.88655|transcript|ENST00000553706.5|protein_coding|4/4|c.1+1G>A||||||,T|splice_donor_variant&
)181562|transcript|ENST00000618864.1|protein_coding|1/2|c.-19+1G>T||||||,T|upstream_gene_var
)181562|transcript|ENST00000618864.1|protein_coding|2/2|c.89+1G>A||||||,C|splice_donor_varian
:|ENST00000554283.1|protein_coding|1/3|c.125_128delGCCC|p.Arg42fs|134/568|125/559|42/185||
ript|ENST00000554283.1|protein_coding|1/3|c.153_154insGT|p.Leu52fs|163/568|154/559|52/185||
interaction|2XMQ:B_248-B_277:ENST00000350792|protein_coding|14/16|c.831C>T||||||,A|structur
0000556399.5|nonsense_mediated_decay|5/23|c.1509A>G|p.Ter503Trpext*?|1615/4902|1509/1509
ENST00000390447.3|TR_V_gene|2/2|c.78delA|p.Ala27fs|195/467|78/350|26/115||WARNING_TRAN
0000390447.3|TR_V_gene|2/2|c.320C>A|p.Ser107*|437/467|320/350|107/115||WARNING_TRANSC
ipt|ENST00000390455.3|TR_V_gene|2/2|c.72dupC|p.Thr25fs|282/537|73/328|25/108||WARNING_T
00258905|transcript|ENST00000553993.1|TR_V_pseudogene|1/1|n.55-1C>G||||||,G|upstream_gene
0000211812|transcript|ENST00000390460.1|TR_V_gene|1/1|c.41-1G>A||||||WARNING_TRANSCRIPT_
|ENST00000408907.3|protein_coding|1/1|c.237delC|p.Ser80fs|296/2110|237/903|79/300||INFO_RE
nscript|ENST00000637218.1|protein_coding|1/16|c.17delT|p.Leu6fs|89/2656|17/2367|6/788||
)933|transcript|ENST00000319074.5|protein_coding||c.*6823delA|||||4442|
nscript|ENST00000637218.1|protein_coding|1/16|c.14_15delTG|p.Leu5fs|87/2656|14/2367|5/788||
0000621729.4|protein_coding|9/12|c.535C>T|p.Arg179*|918/1371|535/633|179/210||,A|synonymo
nteraction|4DB1:A_281-A_325:ENST00000355349|protein_coding|11/40|c.975C>T||||||,A|structural
nteraction|3O4R:A_33-A_58:ENST00000313250|protein_coding|2/8|c.174T>C||||||,C|structural_inte
interaction|3O4R:A_61-A_84:ENST00000313250|protein_coding|2/8|c.252G>A||||||,A|structural_int
nteraction|3O4R:B_161-B_204:ENST00000313250|protein_coding|5/8|c.483C>T||||||,T|missense_va
)187630|transcript|ENST00000559387.5|protein_coding|2/4|c.-73+2T>C||||||WARNING_TRANSCRIPT
00000335125.10|protein_coding|4/8|c.475C>T|p.Arg159*|601/1370|475/699|159/232||,T|stop_gai
ot|ENST00000335125.10|protein_coding|6/8|c.629_630insA|p.His211fs|756/1370|630/699|210/232|
ction|4OYK:A_82-C_55:ENST00000324103-ENST00000284274|protein_coding|2/21|c.246C>T||||||,T
nteraction|4OYJ:L_96-L_139:ENST00000324103|protein_coding|3/21|c.415G>A||||||,A|missense_va
13928|transcript|ENST00000560275.5|protein_coding|6/6|c.650-2A>G||||||WARNING_TRANSCRIPT_
ST00000560155.5|nonsense_mediated_decay|25/30|c.2476_2477delCT|p.Leu826fs|2559/3485|2476/
497|transcript|ENST00000354464.10|protein_coding|21/29|c.2121+2T>G||||||,C|splice_donor_varia
|ENST00000533011.5|protein_coding|1/5|c.56_57delAA|p.Lys19fs|150/733|56/640|19/212||WARNI
|ENST00000533011.5|protein_coding|1/5|c.56_57delAA|p.Lys19fs|150/733|56/640|19/212||WARNI
NST00000288087.11|protein_coding|6/6|c.494delT|p.Leu165fs|606/730|494/531|165/176||,C|frame
|interaction|2A7R:A_55-A_242:ENST00000355299|protein_coding|9/10|c.725G>A||||||,A|structural_
i00000258657|transcript|ENST00000555300.1|antisense|3/3|n.238+1G>A|||||
00000258098|transcript|ENST00000552425.1|lincRNA|1/1|n.81+1G>A|||||
.|ENSG00000186960|transcript|ENST00000548213.2|lincRNA|2/2|n.136-3dupT||||||INFO_REALIGN_
.|ENSG00000186960|transcript|ENST00000548213.2|lincRNA|2/2|n.136-3dupT||||||INFO_REALIGN_
.|ENSG00000186960|transcript|ENST00000548213.2|lincRNA|2/2|n.136-3dupT||||||INFO_REALIGN_
0000257748|transcript|ENST00000549013.1|lincRNA|1/3|n.79+1G>T||||||,A|upstream_gene_varian
)0100478|transcript|ENST00000216366.8|protein_coding|5/5|c.367-3dupT||||||INFO_REALIGN_3_PI
5G00000203546|transcript|ENST00000547760.1|nonsense_mediated_decay|3/3|c.*274-2C>A||||||W
0000399285.3|protein_coding|2/2|c.418C>T|p.Arg140*|473/1057|418/1002|140/333||
NST00000554865.1|nonsense_mediated_decay|2/4|c.195delA|p.Gly66fs|196/540|195/228|65/75||V
)0000258601|transcript|ENST00000557642.1|antisense|1/1|n.246+1G>A||||||,A|downstream_gene_

interaction|2NUP:A_33-A_556:ENST00000307712|protein_coding|15/20|c.1668A>G||||||,C|structur
000092208|transcript|ENST00000308317.10|protein_coding|6/9|c.565-3dupT||||||INFO_REALIGN_3
000092208|transcript|ENST00000308317.10|protein_coding|7/9|c.634-3dupT||||||INFO_REALIGN_3
98718&ENSG00000179454|gene_variant|ENSG00000179454|||n.45042325_45042297delTTTAAAATT
0442|transcript|ENST00000216330.7|protein_coding|1/7|c.-351+1G>A||||||,T|upstream_gene_varia
GH|FANCM|ENSG00000187790|transcript|ENST00000267430.9|protein_coding|8/22|c.1397-3_1397-
GH|FANCM|ENSG00000187790|transcript|ENST00000267430.9|protein_coding|17/22|c.4516-5_4516
ENST00000555794.2|protein_coding|6/6|c.1230_1231dupAT|p.Phe411fs|1233/2278|1231/1257|411
ST00000555794.2|protein_coding|6/6|c.1188_1189delTT|p.Phe396fs|1191/2278|1188/1257|396/41
teraction|4IDN:B_77-B_117:ENST00000358385|protein_coding|3/14|c.351G>A||||||,A|structural_int
00504|transcript|ENST00000216392.7|protein_coding|15/19|c.1828-2delA||||||,C|splice_acceptor_
G00000258942|transcript|ENST00000555649.1|lincRNA|3/3|n.651+2T>C||||||,G|intron_variant|MOI
on_coding_transcript_exon_variant|HIGH|RP11-452D12.1|ENSG00000261208|transcript|ENST000005
t&non_coding_transcript_exon_variant|HIGH|RP11-452D12.1|ENSG00000261208|transcript|ENST000
|LOW|RP11-452D12.1|ENSG00000261208|transcript|ENST00000565135.1|processed_pseudogene|3/
t|LOW|RP11-452D12.1|ENSG00000261208|transcript|ENST00000565135.1|processed_pseudogene|3
iant|LOW|RP11-452D12.1|ENSG00000261208|transcript|ENST00000565135.1|processed_pseudogene
G00000258985|transcript|ENST00000556988.1|antisense|1/1|n.319+2T>C||||||,C|3_prime_UTR_vari
NSG00000237356|transcript|ENST00000456100.5|lincRNA|2/3|n.453-3dupT||||||INFO_REALIGN_3_P
NSG00000237356|transcript|ENST00000456100.5|lincRNA|2/3|n.453-3dupT||||||INFO_REALIGN_3_P
NSG00000237356|transcript|ENST00000456100.5|lincRNA|2/3|n.453-3dupT||||||INFO_REALIGN_3_P
0558961.1|protein_coding|2/2|c.313T>C|p.Ter105Argext*?|313/495|313/315|105/104||,G|missense
|ENST00000251091.9|protein_coding|5/11|c.1114_1121delACGGCCAC|p.Thr372fs|1419/6565|1114/
interaction|3AYC:B_133-B_201:ENST00000254301|protein_coding|6/6|c.603A>T||||||,T|missense_v
GH|TBPL2|ENSG00000182521|transcript|ENST00000556755.1|protein_coding|2/3|c.-165-3_-165-2de
GH|TBPL2|ENSG00000182521|transcript|ENST00000556755.1|protein_coding|2/3|c.-165-3_-165-2de
GH|TBPL2|ENSG00000182521|transcript|ENST00000556755.1|protein_coding|2/3|c.-165-3_-165-2de
G00000259868|transcript|ENST00000612704.1|lincRNA|6/6|n.770+1G>A||||||
T00000422976.6|protein_coding|5/7|c.544C>T|p.Gln182*|586/1153|544/1011|182/336||,A|stop_g
0000100557|transcript|ENST00000216445.7|protein_coding|3/5|c.394-2A>G||||||,C|splice_acceptor
T00000422976.6|protein_coding|3/7|c.331C>T|p.Arg111*|373/1153|331/1011|111/336||,A|stop_g
G00000131966|transcript|ENST00000554402.6|nonsense_mediated_decay|12/14|c.868-2_868-1dupA
0100578|transcript|ENST00000554463.5|protein_coding|1/5|c.-57+2T>C||||||WARNING_TRANSCRIP
0126790|transcript|ENST00000487285.5|protein_coding|1/4|c.-6+1delG||||||,A|upstream_gene_var
000254271.8|nonsense_mediated_decay|23/33|c.3113G>A|p.Trp1038*|3317/4915|3113/3336|1038
54986.2|protein_coding|1/2|c.2T>C|p.Met1?|38/1959|2/336|1/111||
ENST00000261249.6|protein_coding|2/5|c.312_315delAATA|p.Ile105fs|700/5573|312/1530|104/509
0232774|transcript|ENST00000229465.10|lincRNA|1/4|n.221+1G>A||||||,A|splice_donor_variant&in
G00000258943|transcript|ENST00000557475.1|lincRNA|1/1|n.225+2delC||||||
0000267522.7|protein_coding|3/3|c.298C>T|p.Arg100*|544/1687|298/1164|100/387||,A|stop_gain
00054654|transcript|ENST00000344113.8|protein_coding|21/114|c.2647-3dupT||||||INFO_REALIGN_
000344113.8|protein_coding|61/115|c.12002G>A|p.Trp4001*|12214/21777|12002/20658|4001/688
I|TEX21P|ENSG00000234911|transcript|ENST00000447107.1|unitary_pseudogene|4/5|n.981+1_981+
nteraction|3I33:A_208-A_228:ENST00000394709|protein_coding|2/2|c.684C>T||||||,T|structural_in
G00000255002|transcript|ENST00000531609.1|lincRNA|1/3|n.129+1G>A||||||
NST00000315266.9|protein_coding|7/22|c.576_580delTCTTT|p.Leu193fs|1697/4193|576/2211|192/7
t|ENST00000534174.5|nonsense_mediated_decay|5/10|c.588_589insT|p.Thr197fs|811/1651|589/12

00072415|transcript|ENST00000554911.1|processed_transcript|1/2|n.193-3dupT|||||INFO_REALIGI
00072415|transcript|ENST00000554911.1|processed_transcript|1/2|n.193-3dupT|||||INFO_REALIGI
00000329153.9|protein_coding|29/29|c.4042C>T|p.Arg1348*|4174/6604|4042/4095|1348/1364||,T
00182185|transcript|ENST00000497460.5|processed_transcript|5/5|n.497-1G>A|||||,A|intron_vari
GH|BLZF2P|ENSG00000258565|transcript|ENST00000553776.1|unprocessed_pseudogene|2/3|n.343-
258565|transcript|ENST00000553776.1|unprocessed_pseudogene|2/3|n.343-5_343-3delAAA|||||
0258565|transcript|ENST00000553776.1|unprocessed_pseudogene|2/3|n.343-5_343-4delAA|||||
00258565|transcript|ENST00000553776.1|unprocessed_pseudogene|2/3|n.343-5delA|||||
0100626|transcript|ENST00000553471.6|nonsense_mediated_decay|14/14|c.*3649+1G>A|||||,A|u
000029364|transcript|ENST00000556605.5|protein_coding|6/6|c.694-1G>A|||||,A|splice_acceptor_
NSG00000258644|transcript|ENST00000618385.1|nonsense_mediated_decay|3/3|c.*355-2A>C|||||
683|transcript|ENST00000556891.5|processed_transcript|1/2|n.101+2C>T|||||,A|upstream_gene_
000119707|transcript|ENST00000531500.5|protein_coding|1/7|c.-137-3dupT|||||WARNING_TRANS
000119707|transcript|ENST00000531500.5|protein_coding|1/7|c.-137-3dupT|||||WARNING_TRANS
ENST00000261973.11|protein_coding|11/19|c.1210_1211delGA|p.Glu404fs|1495/6910|1210/2532|4
G00000258376|transcript|ENST00000554614.2|antisense|1/1|n.457-1G>T|||||,A|upstream_gene_
nt&intron_variant|HIGH|PAPLN|ENSG00000100767|transcript|ENST00000555123.5|nonsense_mediat
nt&intron_variant|HIGH|PAPLN|ENSG00000100767|transcript|ENST00000555123.5|nonsense_mediat
0100767|transcript|ENST00000555123.5|nonsense_mediated_decay|21/23|c.*151-2_*151-1delAG|||
H|RP1-240K6.3|ENSG00000251393|transcript|ENST00000515412.2|antisense|2/2|n.487-5_487-3dupT
H|RP1-240K6.3|ENSG00000251393|transcript|ENST00000515412.2|antisense|2/2|n.487-5_487-3dupT
H|RP1-240K6.3|ENSG00000251393|transcript|ENST00000515412.2|antisense|2/2|n.487-5_487-3dupT
H|RP1-240K6.3|ENSG00000251393|transcript|ENST00000515412.2|antisense|2/2|n.487-5_487-3dupT
H|RP1-240K6.3|ENSG00000251393|transcript|ENST00000515412.2|antisense|2/2|n.487-5_487-3dupT
interaction|3HLK:A_91-A_115:ENST00000238651|protein_coding|1/3|c.272A>G|||||,G|structural_ir
nteraction|3HLK:A_165-A_186:ENST00000238651|protein_coding|1/3|c.556C>T|||||,T|missense_va
interaction|3K2I:A_147-A_187:ENST00000326303|protein_coding|2/3|c.560C>A|||||,A|structural_ir
ipt|ENST00000326303.4|protein_coding|2/3|c.563_564insTCAA|p.Ala189fs|818/2362|564/1266|188
ENST00000326303.4|protein_coding|2/3|c.566_569delCTTA|p.Ala189fs|820/2362|566/1266|189/421
nteraction|2VNA:A_76-A_101:ENST00000267568|protein_coding|4/10|c.228A>T|||||,T|structural_i
655|transcript|ENST00000555619.5|protein_coding|4/4|c.441+1G>A|||||,T|splice_donor_variant&
ipt|ENST00000553588.1|protein_coding|2/2|c.215dupT|p.Leu72fs|229/776|216/303|72/100||INFO_
100565|transcript|ENST00000460005.1|processed_transcript|2/6|n.814+2T>C|||||,C|upstream_ge
729|transcript|ENST00000619017.1|antisense||n.-641delC||||641|
nscript|ENST00000238647.4|protein_coding|1/1|c.339_346delGCAACAGC|p.Gln114fs|1253/4157|33
ENST00000238647.4|protein_coding|1/1|c.224_305delCCGTCGGGGTCAAGACAGTGGCCCTGTCGGCTAA
I|TMEM63C|ENSG00000165548|transcript|ENST00000554766.5|protein_coding|1/7|c.-237+1_-237+3
ipt|ENST00000623612.1|protein_coding|1/1|c.263delC|p.Pro88fs|263/375|263/375|88/124||INFO_
ENST00000554827.5|protein_coding|8/8|c.888_889delAT|p.Leu296fs|891/1823|888/906|296/301||
00165417|transcript|ENST00000298173.7|nonsense_mediated_decay|2/9|c.133-2A>G|||||,C|intron
00556879.5|nonsense_mediated_decay|2/6|c.158G>A|p.Trp53*|158/595|158/189|53/62||WARNIN
ENST00000554168.5|nonsense_mediated_decay|1/4|c.121delG|p.Ala41fs|302/651|121/141|41/46||
042317|transcript|ENST00000557248.5|nonsense_mediated_decay|4/5|c.219+1G>A|||||,A|missens
intron_variant|HIGH|TDP1|ENSG00000042088|transcript|ENST00000393452.7|protein_coding|16/18
00133943|transcript|ENST00000523894.5|protein_coding|1/4|c.-82+2T>C|||||WARNING_TRANSCRI
00133943|transcript|ENST00000523816.5|protein_coding|7/15|c.441+2C>T|||||,T|splice_donor_var
pt|ENST00000517306.5|nonsense_mediated_decay|9/10|c.1445delC|p.Pro482fs|1447/2294|1445/15

000532032.5|protein_coding|10/10|c.1047C>A|p.Tyr349*|1057/1191|1047/1095|349/364||,T|dowr
ENST00000558190.5|protein_coding|10/11|c.916_917insC|p.Gly306fs|974/26812|916/1086|306/36
00066427|transcript|ENST00000558190.5|protein_coding|10/11|c.915_916insCAGCAGCAGCAGCAGC
00066427|transcript|ENST00000558190.5|protein_coding|10/11|c.915_916insCAGCAGCAGCAGCAGC
00066427|transcript|ENST00000558190.5|protein_coding|10/11|c.915_916insCAGCAGCAGCAGCAGC
00066427|transcript|ENST00000558190.5|protein_coding|10/11|c.915_916insCAGCAGCAGCAGCAGC
00066427|transcript|ENST00000558190.5|protein_coding|10/11|c.915_916insCAGCAGCAGCAGCAGC
00066427|transcript|ENST00000558190.5|protein_coding|10/11|c.915_916insCAGCAGCAGCAGCAGC
ENST00000558190.5|protein_coding|10/11|c.891_892insG|p.Gln298fs|949/26812|891/1086|297/36
NSG00000066427|transcript|ENST00000558190.5|protein_coding|10/11|c.890_891insGCAGCAGCAG
NSG00000066427|transcript|ENST00000558190.5|protein_coding|10/11|c.890_891insGCAGCAGCAG
183648|transcript|ENST00000555441.5|protein_coding|1/2|c.-6+2T>C||||||,G|upstream_gene_varia
nteraction|4AWB:B_106-B_158:ENST00000334869|protein_coding|6/14|c.474T>C||||||,G|structural
0170054|transcript|ENST00000337425.9|protein_coding|4/4|c.1104+2T>C||||||,G|splice_donor_var
165959|transcript|ENST00000555336.6|protein_coding|1/6|c.-210-2A>G|||||WARNING_TRANSCRIP
nteraction|2WUL:A_80-A_146:ENST00000331334|protein_coding|2/2|c.436G>A||||||,A|structural_ir
0000090060|transcript|ENST00000216277.12|protein_coding|17/21|c.1665-3dupT|||||INFO_REALIG
0000090060|transcript|ENST00000216277.12|protein_coding|17/21|c.1665-3dupT|||||INFO_REALIG
ENST00000557352.1|protein_coding|4/4|c.438_439dupTG|p.Gly147fs|441/599|440/528|147/175||W
0000176605|transcript|ENST00000325812.6|protein_coding|3/4|c.2_10delTGCATCGGA|p.Met1_Arg3
nteraction|1O5T:A_309-A_335:ENST00000355338|protein_coding|9/11|c.1003C>T||||||,A|structural
140105|transcript|ENST00000556295.5|protein_coding|1/3|c.-158-2A>T|||||WARNING_TRANSCRIP
183092|transcript|ENST00000355173.6|protein_coding|1/6|c.-16+1G>C||||||,G|upstream_gene_vari
4548|transcript|ENST00000521256.1|lincRNA|1/1|n.174+1G>C||||||,C|intron_variant|MODIFIER|MI
223403|transcript|ENST00000429368.1|lincRNA|1/1|n.146-1G>C||||||,C|upstream_gene_variant|M
000230805|transcript|ENST00000448840.6|lincRNA|3/3|n.354+1delG||||||,A|splice_donor_variant&
24|interaction|3Q6M:B_612-B_675:ENST00000216281|protein_coding|10/11|c.1834A>C||||||,G|syr
24|interaction|3Q6M:B_360-B_386:ENST00000216281|protein_coding|6/11|c.1080C>T||||||,A|syno
ST00000519058.5|protein_coding|4/4|c.338_339delGT|p.Cys113fs|654/917|338/378|113/125||,T|fr
0823|transcript|ENST00000361847.6|protein_coding|4/11|c.283+2T>C||||||,G|splice_donor_variant&
00361847.6|protein_coding|4/12|c.280C>T|p.Arg94*|512/1940|280/1260|94/419||,A|stop_gained|
|ENST00000560304.1|protein_coding|5/5|c.572delG|p.Gly191fs|573/657|572/636|191/211||WARN
0000259535|transcript|ENST00000559810.1|processed_pseudogene|1/1|n.375-1A>C||||||
GH|GCSHP2|ENSG00000259525|transcript|ENST00000560971.1|processed_pseudogene|1/1|n.465-3
GH|GCSHP2|ENSG00000259525|transcript|ENST00000560971.1|processed_pseudogene|1/1|n.465-3
000075413|transcript|ENST00000440884.7|protein_coding|3/15|c.298-3dupT|||||INFO_REALIGN_3_
eraction|3B6R:A_301-A_364:ENST00000348956|protein_coding|8/8|c.1092G>A||||||,T|structural_int
eraction|3B6R:A_152-A_213:ENST00000348956|protein_coding|4/8|c.456C>A||||||,T|structural_inte
G00000258913|transcript|ENST00000555282.1|lincRNA|1/1|n.203+2T>G||||||,G|splice_donor_varia
teraction|4EJN:A_206-A_417:ENST00000349310|protein_coding|13/15|c.1251C>T||||||,A|structural
teraction|3CQU:A_340-A_367:ENST00000349310|protein_coding|12/15|c.1020C>T||||||,A|structura
|ENST00000333244.5|protein_coding|7/7|c.6424_6427delGCCA|p.Ala2142fs|6547/18254|6424/1738
916|transcript|ENST00000331782.7|protein_coding|18/25|c.2365+2T>G||||||,C|splice_donor_variar
nteraction|2VKP:A_56-A_86:ENST00000463376|protein_coding|3/3|c.258C>T||||||,T|structural_inte
ST00000619213.1|protein_coding|8/9|c.1420G>T|p.Glu474*|1511/4013|1420/2175|474/724||
T00000562295.1|protein_coding|16/19|c.1408G>T|p.Glu470*|1408/1878|1408/1878|470/625||,T|dc
ript|ENST00000567107.5|protein_coding|8/8|c.2396dupC|p.Gly800fs|2449/3030|2396/2730|799/90

pt|ENST00000567107.5|protein_coding|8/8|c.2080delA|p.Arg694fs|2133/3030|2080/2730|694/909|
pt|ENST00000567107.5|protein_coding|8/8|c.2017delG|p.Glu673fs|2070/3030|2017/2730|673/909|
pt|ENST00000567107.5|protein_coding|8/8|c.1975_2015delGAAGATGCGGGAGCAGGAGGAGAAGATGT
59905|transcript|ENST00000568609.5|lincRNA|3/8|n.601+1G>A||||||,A|upstream_gene_variant|M
|ENST00000554696.5|protein_coding|1/6|c.66_69delGTCA|p.Gln22fs|66/774|66/774|22/257||WAR
cript|ENST00000567390.6|protein_coding|5/9|c.283dupA|p.Ile95fs|409/2397|283/1869|95/622||,A
000278582|transcript|ENST00000615639.1|unprocessed_pseudogene|1/1|n.180+1A>G||||||,G|dow
000278582|transcript|ENST00000615639.1|unprocessed_pseudogene|1/1|n.180+2G>T||||||,T|down
eraction|4PJ3:A_566-A_590:ENST00000156471|protein_coding|18/35|c.1698C>T||||||,A|structural_
GP8|ENSG00000255192|transcript|ENST00000629403.1|protein_coding|2/2|c.868_869delTG|p.Ter2
0000259345|transcript|ENST00000561058.5|lincRNA|2/5|n.150+2T>C||||||,G|splice_donor_variant
nteraction|3R6B:A_494-A_521:ENST00000260356|protein_coding|10/22|c.1563C>T||||||,T|synonym
nteraction|2FJU:B_249-B_773:ENST00000557821|protein_coding|22/32|c.2318A>G||||||,C|structura
T00000610693.4|protein_coding|2/5|c.326_327delTT|p.Phe109fs|350/668|326/644|109/213||WAR
0128891|transcript|ENST00000561011.5|protein_coding|1/3|c.-13+1G>A||||||,T|splice_donor_varia
nscript|ENST00000355341.8|protein_coding|1/11|c.118_122dupGGGGC|p.Arg48fs|624/2775|123/14
G00000259617|transcript|ENST00000561388.1|antisense|1/1|n.167+1G>A||||||,A|upstream_gene_
0000247556|transcript|ENST00000559368.5|antisense|3/3|n.333-2dupA||||||INFO_REALIGN_3_PRI
0000137804|transcript|ENST00000558582.5|processed_transcript|4/4|n.524-3dupT||||||INFO_REAL
0000137804|transcript|ENST00000558582.5|processed_transcript|4/4|n.524-3dupT||||||INFO_REAL
24|transcript|ENST00000561619.5|protein_coding|4/13|c.439+1G>A||||||,T|downstream_gene_vari
T00000263800.10|protein_coding|5/20|c.601_610delTCGCGGCGCT|p.Ser201fs|707/3046|601/2595|
00000092445|transcript|ENST00000263798.7|protein_coding|2/18|c.308_308+1insCCTGAA||||||,G
t&splice_region_variant&intron_variant|HIGH|TYRO3|ENSG00000092445|transcript|ENST0000026379
ariant&intron_variant|HIGH|TYRO3|ENSG00000092445|transcript|ENST00000263798.7|protein_codin
ariant&intron_variant|HIGH|TYRO3|ENSG00000092445|transcript|ENST00000263798.7|protein_codin
0000092445|transcript|ENST00000263798.7|protein_coding|12/18|c.1579+1_1579+2insAGAG||||||I
0092445|transcript|ENST00000263798.7|protein_coding|14/18|c.1753_1753+1dupGG||||||INFO_RE
ariant|HIGH|TYRO3|ENSG00000092445|transcript|ENST00000263798.7|protein_coding|15/18|c.187
O3|ENSG00000092445|transcript|ENST00000263798.7|protein_coding|17/18|c.2145_2145+1insTGGG
HIGH|TYRO3|ENSG00000092445|transcript|ENST00000263798.7|protein_coding|17/18|c.2145_2145
7877|transcript|ENST00000320955.7|protein_coding|46/68|c.7815G>A|p.Trp2605*|8043/11725|781
0000320955.7|protein_coding|31/68|c.5647C>T|p.Arg1883*|5875/11725|5647/11025|1883/3674||
iant&stop_gained|HIGH|TTBK2|ENSG00000128881|transcript|ENST00000622375.4|protein_coding|1
3803|transcript|ENST00000389651.8|protein_coding|1/9|c.-288+1G>A||||||,A|splice_donor_variant
00000566849.5|nonsense_mediated_decay|3/5|c.561G>A|p.Trp187*|710/2834|561/600|187/199||,
GH|ZSCAN29|ENSG00000140265|transcript|ENST00000396972.2|protein_coding|1/5|c.66+2A>G||||
0000166762|transcript|ENST00000419262.1|nonsense_mediated_decay|2/3|c.*40-2A>T||||||,A|spli
G00000205771|transcript|ENST00000381680.6|processed_transcript|2/3|n.405-2A>T||||||,A|splice_
ipt|ENST00000625006.1|protein_coding|1/2|c.24_25delAA|p.Ser9fs|25/48|24/48|8/15||WARNING_
ipt|ENST00000625006.1|protein_coding|1/2|c.24_25delAA|p.Ser9fs|25/48|24/48|8/15||WARNING_
ipt|ENST00000625006.1|protein_coding|1/2|c.24_25delAA|p.Ser9fs|25/48|24/48|8/15||WARNING_
0560110.1|protein_coding|2/5|c.1A>G|p.Met1?|390/765|1/376|1/124||WARNING_TRANSCRIPT_INC
G00000259742|transcript|ENST00000558917.1|unprocessed_pseudogene|3/4|n.282+2C>T||||||,A|d
ENST00000389039.10|protein_coding|22/34|c.2895_2898delGTTC|p.Phe966fs|3284/6533|2895/4647
00259519&ENSG00000138606|gene_variant|ENSG00000259519|||n.45199954_45199972delAAAAAAA
00259519&ENSG00000138606|gene_variant|ENSG00000259519|||n.45199955_45199972delAAAAAA

000259519&ENSG00000138606|gene_variant|ENSG00000259519|||n.45199956_45199972delAAAAA
00000259519&ENSG00000138606|gene_variant|ENSG00000259519|||n.45199957_45199972delAAAA
G00000259519&ENSG00000138606|gene_variant|ENSG00000259519|||n.45199958_45199972delAAA
on_coding_transcript_exon_variant|HIGH|RP11-154J22.1|ENSG00000259488|transcript|ENST0000061
nteraction|2A2C:A_142-A_407:ENST00000560031|protein_coding|10/10|c.1221C>T||||||,T|structura
teraction|2GFO:A_1011-A_1102:ENST00000307179|protein_coding|18/20|c.3033G>A||||||,A|structu
G00000273674|transcript|ENST00000613161.4|lincRNA|3/4|n.425+2G>T||||||,A|splice_donor_variar
G00000260618|transcript|ENST00000562062.1|antisense|2/3|n.345-1G>A||||||,A|downstream_gene
00000260323.15|protein_coding|1/32|c.283C>T|p.Arg95*|283/12946|283/6645|95/2214||
G00000137766|transcript|ENST00000561210.1|retained_intron|8/11|n.1160-6_1160-3dupTGTC|||||
00137766|transcript|ENST00000561210.1|retained_intron|11/11|n.1509-1G>A||||||,A|intron_variar
00000321149.7|protein_coding|10/10|c.1249G>T|p.Glu417*|1617/1993|1249/1263|417/420||,A|sto
interaction|2XBF:A_530-A_646:ENST00000435532|protein_coding|18/29|c.1589G>A||||||,T|missens
NSG00000151575|gene_variant|ENSG00000261174|||n.56323707_56323739delAGGAGGAGAAGGAGG
NSG00000151575|gene_variant|ENSG00000261174|||n.56323716_56323730delAGGAGGAAGAGGAGG
000558231.5|protein_coding|1/13|c.2T>A|p.Met1?|62/1664|2/1470|1/489||,T|intron_variant|MOD
NST00000380516.6|protein_coding|15/21|c.1994_1995delAG|p.Glu665fs|2083/4147|1994/3105|665/
00000227161|transcript|ENST00000441746.1|sense_overlapping|1/1|n.69-1G>C||||||,G|downstrea
0000559176.5|protein_coding|1/9|c.17G>A|p.Trp6*|19/744|17/742|6/246||WARNING_TRANSCRIPT_
28915|transcript|ENST00000561087.5|processed_transcript|1/5|n.106-1G>T||||||,A|splice_acceptor
G00000166104|transcript|ENST00000570077.1|processed_transcript|2/4|n.170+1G>T||||||,A|splice_
ENSG00000166104|transcript|ENST00000562306.5|transcribed_unprocessed_pseudogene|7/22|n.456
ENSG00000259370|transcript|ENST00000557994.1|antisense|3/3|n.416-2A>G||||||,C|3_prime_UTR_
7|interaction|3UX3:A_39-A_110:ENST00000300030|protein_coding|1/5|c.117A>G||||||,C|structural_
0169118|transcript|ENST00000607537.6|protein_coding|1/12|c.-225+1G>A||||||,T|splice_donor_var
0103671|transcript|ENST00000559833.5|processed_transcript|1/4|n.42-3dupT||||||INFO_REALIGN_
00103671|transcript|ENST00000559833.5|processed_transcript|1/4|n.42-3_42-2insTA||||||,TA|splic
00103671|transcript|ENST00000559833.5|processed_transcript|1/4|n.42-3_42-2insTA||||||,TA|splic
00333425.10|protein_coding|2/13|c.145G>T|p.Glu49*|240/2278|145/2124|49/707||,A|stop_gained|
03742|transcript|ENST00000352385.2|protein_coding|11/19|c.2122+2T>G||||||,C|splice_donor_var
GH|HNRNPA1P44|ENSG00000249271|transcript|ENST00000503999.2|processed_pseudogene|1/1|n.
GH|HNRNPA1P44|ENSG00000249271|transcript|ENST00000503999.2|processed_pseudogene|1/1|n.
GH|HNRNPA1P44|ENSG00000249271|transcript|ENST00000503999.2|processed_pseudogene|1/1|n.
0259673|transcript|ENST00000559285.1|lincRNA|1/4|n.171+1G>A||||||,T|upstream_gene_variant|M
271232|transcript|ENST00000603171.1|processed_pseudogene|1/1|n.732+2G>A||||||
0138604|transcript|ENST00000559798.2|processed_transcript|2/2|n.265-3dupT||||||INFO_REALIGN_
559420.2|protein_coding|1/3|c.1A>G|p.Met1?|206/4840|1/1662|1/553||,G|missense_variant|MOD
NSG00000259504|transcript|ENST00000558454.1|lincRNA|1/1|n.172-2A>G||||||,G|upstream_gene_
332|transcript|ENST00000627388.2|protein_coding|7/18|c.594+1_594+2delCA||||||,C|downstream_
00000259624|transcript|ENST00000557828.1|antisense|1/1|n.87+2T>G||||||
G00000137821|transcript|ENST00000544974.6|protein_coding|1/16|c.-298-3dupT||||||INFO_REALIG
G00000137821|transcript|ENST00000544974.6|protein_coding|1/16|c.-298-3dupT||||||INFO_REALIG
G00000137821|transcript|ENST00000544974.6|protein_coding|1/16|c.-298-3dupT||||||INFO_REALIG
NST00000379915.4|nonsense_mediated_decay|6/16|c.682T>G|p.Ter228Glyext*?|691/4374|682/684|
00067141|transcript|ENST00000261908.10|protein_coding|3/28|c.725-3dupT||||||INFO_REALIGN_3
00067141|transcript|ENST00000261908.10|protein_coding|3/28|c.725-3dupT||||||INFO_REALIGN_3
nteraction|3P4L:A_892-A_931:ENST00000261908|protein_coding|17/29|c.2676C>T||||||,T|structural_

```
)3855|transcript|ENST00000558689.5|protein_coding|2/3|c.-55+2T>G||||||WARNING_TRANSCRIPT_
)0261801|transcript|ENST00000564194.5|antisense|1/2|n.184+2T>G||||||,C|splice_donor_variant&
eraction|1BYG:A_222-A_264:ENST00000220003|protein_coding|9/13|c.792C>T||||||,T|structural_int
:|ENST00000394987.4|protein_coding|2/3|c.803_806delACTA|p.His268fs|1036/4430|803/3144|268/
NSG00000261714|transcript|ENST00000569073.2|processed_pseudogene|1/1|n.323-2delA||||||INF(
NSG00000261714|transcript|ENST00000569073.2|processed_pseudogene|1/1|n.323-2delA||||||INF(
NST00000308508.5|protein_coding|3/10|c.1552_1564delCTGGTGCTGGAGG|p.Leu518fs|1657/8290|1
H|RP11-24M17.5|ENSG00000187812|transcript|ENST00000566174.1|transcribed_unprocessed_pseud
9752|transcript|ENST00000461391.5|nonsense_mediated_decay|1/7|c.-57+1G>A||||||,T|splice_don
G00000169758|transcript|ENST00000561302.5|nonsense_mediated_decay|10/10|c.*923-3dupT|||||
G00000169758|transcript|ENST00000561302.5|nonsense_mediated_decay|10/10|c.*923-3dupT|||||
G00000169758|transcript|ENST00000561302.5|nonsense_mediated_decay|10/10|c.*923-3dupT|||||
G00000169758|transcript|ENST00000561302.5|nonsense_mediated_decay|10/10|c.*923-3dupT|||||
)0324767.11|protein_coding|1/31|c.1A>G|p.Met1?|60/4707|1/4203|1/1400||,C|start_lost|HIGH|SC
.40386|transcript|ENST00000563919.1|protein_coding|2/5|c.-60+2C>T||||||WARNING_TRANSCRIPT_
7906|transcript|ENST00000557805.1|nonsense_mediated_decay|4/5|c.*42+2C>T||||||WARNING_TR
ISG00000260776|transcript|ENST00000562938.5|transcribed_unprocessed_pseudogene|12/17|n.146
0409568.6|protein_coding|3/6|c.2T>C|p.Met1?|361/2162|2/948|1/315||,C|missense_variant|MODE
|transcript|ENST00000446172.2|protein_coding|1/7|c.1_2delAT|p.Met1fs|63/1399|1/1113|1/370||,(
)0558341.5|protein_coding|6/6|c.376T>C|p.Ter126Argext*?|421/719|376/378|126/125||,C|synonym
ipt|ENST00000388820.4|protein_coding|19/24|c.3232dupT|p.Tyr1078fs|3443/5490|3232/5061|1078
37|interaction|2AQL:B_186-B_257:ENST00000426013|protein_coding|9/12|c.558C>A||||||,A|structu
66557|transcript|ENST00000299705.9|protein_coding|1/2|c.168+1G>A||||||,A|splice_donor_variant
|ENST00000560156.1|nonsense_mediated_decay|1/3|c.263dupA|p.Ile89fs|305/579|263/333|88/11(
)00359440.5|protein_coding|22/22|c.3134C>A|p.Ser1045*|3270/4641|3134/3303|1045/1100||,T|st
)0000259343|transcript|ENST00000559277.1|antisense|1/1|n.62-1G>A||||||,A|downstream_gene_va
ENST00000558133.1|protein_coding|1/1|c.272_273delAT|p.Asp91fs|687/4333|272/477|91/158||,C|i
ENST00000558133.1|protein_coding|1/1|c.262_265delTCGA|p.Ser88fs|679/4333|262/477|88/158||,(
NST00000339465.5|protein_coding|3/3|c.528_529delAC|p.Arg177fs|597/3134|528/1197|176/398||
ISG00000259654|transcript|ENST00000558044.1|antisense|1/1|n.56-1G>A||||||,A|intron_variant|M
)0260477|transcript|ENST00000562495.2|antisense|3/3|n.432+2T>C||||||,G|upstream_gene_variar
)0000260477|transcript|ENST00000562495.2|antisense|2/3|n.235-2G>A||||||,T|upstream_gene_vari
)0260305|transcript|ENST00000569588.1|lincRNA|1/3|n.749+1G>A||||||,A|upstream_gene_variant
)0249487|transcript|ENST00000560368.1|lincRNA|1/2|n.247+2T>C||||||,G|upstream_gene_variant|
nteraction|1WLJ:A_35-A_156:ENST00000306072|protein_coding|2/4|c.105T>C||||||,C|structural_inte
)0000562889.5|protein_coding|2/6|c.49C>T|p.Gln17*|188/1547|49/1269|17/422||,A|intron_variant
)0000261441|transcript|ENST00000562356.1|antisense|1/1|n.99+1G>T||||||,T|upstream_gene_varia
|ENST00000300057.4|protein_coding|1/2|c.158dupC|p.Ser54fs|237/2369|158/807|53/268||,CG|ups
323|transcript|ENST00000300057.4|protein_coding|1/2|c.156_157insCCGAGCCCCGT|p.Ala53fs|235/2
323|transcript|ENST00000300057.4|protein_coding|1/2|c.156_157insCCGAGCCCCGT|p.Ala53fs|235/2
GH|MRPL15P1|ENSG00000259441|transcript|ENST00000559543.2|processed_pseudogene|1/1|n.45(
GH|MRPL15P1|ENSG00000259441|transcript|ENST00000559543.2|processed_pseudogene|1/1|n.45(
GH|MRPL15P1|ENSG00000259441|transcript|ENST00000559543.2|processed_pseudogene|1/1|n.45(
GH|MRPL15P1|ENSG00000259441|transcript|ENST00000559543.2|processed_pseudogene|1/1|n.45(
GH|MRPL15P1|ENSG00000259441|transcript|ENST00000559543.2|processed_pseudogene|1/1|n.45(
interaction|4FYQ:A_451-A_935:ENST00000300060|protein_coding|21/21|c.2803C>T||||||,A|structur
nteraction|4FYQ:A_690-A_752:ENST00000300060|protein_coding|17/21|c.2255G>A||||||,T|structur
```

interaction|4FYQ:A_118-A_166:ENST00000300060|protein_coding|2/21|c.354C>T||||||,A|structural_
nteraction|4FYQ:A_86-A_107:ENST00000300060|protein_coding|2/21|c.257G>A||||||,T|structural_i
teraction|4JA8:A_364-A_434:ENST00000330062|protein_coding|11/11|c.1302C>G||||||,C|structural_
)0000561085.1|protein_coding|5/5|c.257G>A|p.Trp86*|494/582|257/345|86/114||WARNING_TRAN
nteraction|4OMD:C_464-C_550:ENST00000268171|protein_coding|13/16|c.1392G>T||||||,T|structu
T00000470152.6|nonsense_mediated_decay|2/5|c.247delT|p.Cys83fs|449/1096|247/366|83/121||,C
IST00000555455.5|protein_coding|7/7|c.672_675delGTGT|p.Cys225fs|677/830|672/714|224/237||W
)0258754|transcript|ENST00000557481.6|lincRNA|1/6|n.249+1G>T||||||,A|upstream_gene_variant|
0000248441|transcript|ENST00000611265.4|lincRNA|4/4|n.574-1G>T||||||,A|intron_variant|MODIF
on_coding_transcript_exon_variant|HIGH|NR2F2-AS1|ENSG00000247809|transcript|ENST00000620029
)0247809|transcript|ENST00000620029.4|antisense|4/6|n.817+2T>C||||||,G|upstream_gene_variar
0000328504.3|protein_coding|2/3|c.100C>T|p.Arg34*|367/1083|100/318|34/105||,T|5_prime_UTR_
0000251209|transcript|ENST00000614972.4|lincRNA|1/5|n.599-1G>T||||||,A|intron_variant|MODIF
)443|transcript|ENST00000558751.1|processed_transcript|1/1|n.316+1G>T||||||,T|downstream_ge
ot|ENST00000378919.6|protein_coding|5/5|c.493dupG|p.Val165fs|699/995|493/591|165/196||,AC|
ISG00000259475&ENSG00000278090|gene_variant|ENSG00000259475|||n.99028541_99028517delA
ISG00000259475&ENSG00000278090|gene_variant|ENSG00000259475|||n.99028541_99028517delA
ISG00000259475&ENSG00000278090|gene_variant|ENSG00000259475|||n.99028541_99028517delA
ISG00000259475&ENSG00000278090|gene_variant|ENSG00000259475|||n.99028541_99028517delA
ISG00000259475&ENSG00000278090|gene_variant|ENSG00000259475|||n.99028541_99028517delA
ISG00000259475&ENSG00000278090|gene_variant|ENSG00000259475|||n.99028541_99028517delA
000434594.5|protein_coding|5/6|c.466C>T|p.Gln156*|467/844|466/789|156/262||WARNING_TRAN
I|LRRC28|ENSG00000168904|transcript|ENST00000559819.5|retained_intron|5/5|n.2000+2_2000+4c
I|LRRC28|ENSG00000168904|transcript|ENST00000559819.5|retained_intron|5/5|n.2000+2_2000+4c
pt|ENST00000378904.4|protein_coding|1/1|c.144_145dupCG|p.Val49fs|1172/2122|145/396|49/131
)471|transcript|ENST00000561233.1|processed_transcript|1/1|n.271+1G>A||||||,T|downstream_ge
)0000272808|transcript|ENST00000431060.4|processed_transcript|2/4|n.202+2T>C||||||,C|splice_d
)0000232386|transcript|ENST00000558254.5|lincRNA|2/3|n.386+1G>A||||||,A|upstream_gene_vari
ISG00000259376|transcript|ENST00000558979.1|antisense|1/1|n.69-1G>C||||||,G|intron_variant|M
)0000154237|transcript|ENST00000525284.5|nonsense_mediated_decay|28/32|c.*2613-6_*2613-3du
)00621185.4|protein_coding|3/3|c.369G>A|p.Trp123*|371/462|369/426|123/141||,T|stop_gained|
03152|transcript|ENST00000356432.7|protein_coding|1/3|c.25-1G>T||||||,T|splice_acceptor_variar
IGH|NPRL3|ENSG00000103148|transcript|ENST00000620134.4|protein_coding|2/15|c.-67-2_-38delA
)000167930|transcript|ENST00000301678.7|protein_coding|2/12|c.-26-1G>A||||||,A|splice_acceptor
nscript|ENST00000421000.1|protein_coding|4/4|c.595_598dupACGG|p.Gly200fs|601/739|599/702|2
000397770.7|protein_coding|13/17|c.970G>T|p.Glu324*|988/2390|970/1404|324/467||,A|stop_ga
ENST00000219406.10|protein_coding|10/11|c.1478dupA|p.Asn493fs|1497/1698|1479/1578|493/525
hift_variant|HIGH|MRPL28|ENSG00000086504|transcript|ENST00000429738.1|protein_coding|1/2|c.
pt|ENST00000448854.1|protein_coding|5/5|c.729dupC|p.Ter244fs|729/858|729/732|243/243||WAF
:|ENST00000433358.5|protein_coding|5/5|c.457_458insAG|p.Arg153fs|459/966|458/507|153/168||
L03326|transcript|ENST00000219611.6|protein_coding|3/13|c.-23+2T>C||||||,C|splice_donor_variar
pt|ENST00000443147.5|nonsense_mediated_decay|1/12|c.1_4dupATGT|p.Trp2fs|68/2054|5/1725|2
ENST00000565511.5|nonsense_mediated_decay|4/6|c.316delC|p.His106fs|366/783|316/393|106/13
NST00000566408.5|protein_coding|5/5|c.509delC|p.Pro170fs|511/684|509/555|170/184||WARNIN
|ENST00000567076.5|protein_coding|4/4|c.474dupG|p.Pro159fs|476/523|475/522|159/173||WARN
|ENST00000567076.5|protein_coding|4/4|c.474dupG|p.Pro159fs|476/523|475/522|159/173||WARN
|ENST00000567076.5|protein_coding|4/4|c.497dupG|p.Leu167fs|499/523|498/522|166/173||WARN

ENST00000631357.2|protein_coding|2/24|c.324_325delCC|p.Arg109fs|324/3555|324/3555|108/118
ENST00000631357.2|protein_coding|2/24|c.324_325delCC|p.Arg109fs|324/3555|324/3555|108/118
ENST00000631357.2|protein_coding|2/24|c.324_325delCC|p.Arg109fs|324/3555|324/3555|108/118
ENST00000631357.2|protein_coding|2/24|c.324_325delCC|p.Arg109fs|324/3555|324/3555|108/118
ENST00000631357.2|protein_coding|2/24|c.326_327delGG|p.Arg109fs|326/3555|326/3555|109/118
NST00000301698.1|protein_coding|3/3|c.706_722delACGCCGGACCGACACGG|p.Thr236fs|706/1209|
3227|transcript|ENST00000562226.5|nonsense_mediated_decay|4/7|c.*114+1G>A||||||,T|splice_do
3227|transcript|ENST00000562380.5|protein_coding|1/3|c.76+1G>A||||||WARNING_TRANSCRIPT_N
GH|RP11-161M6.3|ENSG00000260496|transcript|ENST00000599946.2|lincRNA|7/7|n.897+1delT||||
)184471|transcript|ENST00000621771.1|protein_coding|7/7|c.*4+1G>T||||||,A|splice_donor_varian
NST00000211076.4|protein_coding|2/5|c.127delG|p.Glu43fs|275/1499|127/729|43/242||INFO_REA
nteraction|1A3S:A_46-A_117:ENST00000397514|protein_coding|3/7|c.138A>G||||||,G|structural_in
t&intron_variant|HIGH|UNKL|ENSG00000059145|transcript|ENST00000508903.6|protein_coding|6/1
)00197599|transcript|ENST00000463299.2|retained_intron|2/2|n.323-1G>A||||||,T|splice_acceptor_
nteraction|1ZS6:A_53-A_94:ENST00000219302|protein_coding|4/5|c.282A>T||||||,A|structural_inte
0000074071|transcript|ENST00000177742.7|protein_coding|1/2|c.320_321+2dupAGGT||||||,CACCT
NST00000568449.5|protein_coding|1/8|c.164delG|p.Gly55fs|185/1234|164/1140|55/379||INFO_REA
95906|transcript|ENST00000564227.5|nonsense_mediated_decay|1/3|c.*309+1G>A||||||WARNING_
t|ENST00000543683.6|protein_coding|3/3|c.431_432delAA|p.Gln144fs|513/830|431/507|144/168||
0000397280.8|protein_coding|7/8|c.721C>T|p.Gln241*|725/1147|721/1131|241/376||,A|stop_gain
0000561844.5|protein_coding|6/6|c.496T>C|p.Ter166Glnext*?|498/738|496/498|166/165||WARNIN
.67967|transcript|ENST00000562589.5|nonsense_mediated_decay|1/5|c.155-2A>G||||||WARNING_
|ENST00000570539.1|protein_coding|1/2|c.112dupT|p.Ser38fs|113/431|113/126|38/41||WARNING_
ENST00000576381.1|nonsense_mediated_decay|1/5|c.253_254delAC|p.Thr85fs|256/850|253/348|8
00000270168|transcript|ENST00000573315.2|protein_coding|24/34|c.982+2T>C||||||,C|downstrea
ipt|ENST00000575124.1|protein_coding|1/3|c.318delG|p.Ser107fs|341/1133|318/681|106/226||INF
ENST00000525377.6|protein_coding|6/6|c.651_652dupGG|p.Ala218fs|751/997|653/666|218/221||I
nteraction|4CG4:A_663-A_695:ENST00000219596|protein_coding|10/10|c.2084A>G||||||,C|structura
nteraction|2WL1:A_588-A_732:ENST00000219596|protein_coding|9/10|c.1764G>A||||||,T|structural
nteraction|4CG4:A_476-A_566:ENST00000219596|protein_coding|5/10|c.1428A>G||||||,C|structural
ENST00000304936.3|protein_coding|1/1|c.818delT|p.Phe273fs|870/1406|818/939|273/312||INFO_R
nteraction|4Z1F:A_338-A_395:ENST00000246957|protein_coding|11/18|c.1185C>G||||||,C|missense
nteraction|4Z1F:A_311-A_344:ENST00000246957|protein_coding|9/18|c.1032C>T||||||,A|structural_
T00000572111.1|nonsense_mediated_decay|2/7|c.1411delG|p.Glu471fs|1419/5587|1411/1461|471/
)590730.5|nonsense_mediated_decay|3/7|c.241T>G|p.Ter81Glyext*?|342/2198|241/243|81/80||,C
)0103199|transcript|ENST00000585977.1|protein_coding|1/1|c.-121-2A>G||||||WARNING_TRANSCR
00000267077|transcript|ENST00000592465.1|antisense|1/1|n.196+2C>T||||||,A|3_prime_UTR_varia
pt|ENST00000591822.5|nonsense_mediated_decay|1/13|c.224_227dupTCTG|p.Trp76fs|229/1689|22
)0273963|transcript|ENST00000621765.1|unprocessed_pseudogene|2/2|n.402+1G>T||||||,A|intron_
00000260835|transcript|ENST00000564076.1|sense_intronic|1/1|n.124+1G>A||||||,A|intron_varian
)0000078328|transcript|ENST00000547427.5|processed_transcript|4/4|n.412-3dupT||||||INFO_REA
)0000078328|transcript|ENST00000547427.5|processed_transcript|4/4|n.412-3dupT||||||INFO_REA
)0000078328|transcript|ENST00000547427.5|processed_transcript|4/4|n.412-3dupT||||||INFO_REA
4857|transcript|ENST00000333050.6|protein_coding|1/2|c.1A>G|p.Met1?|35/1448|1/642|1/213||,
nteraction|2Q4R:A_197-A_225:ENST00000268261|protein_coding|7/8|c.590A>C||||||,C|missense_v
G00000260276|transcript|ENST00000565934.1|antisense|4/4|n.1999-1G>C||||||,C|5_prime_UTR_va
3I1.6|ENSG00000261392|transcript|ENST00000562893.1|lincRNA|1/2|n.101-3dupT||||||INFO_REAL

3I1.6|ENSG00000261392|transcript|ENST00000562893.1|lincRNA|1/2|n.101-3dupT|||||INFO_REALI(
3I1.6|ENSG00000261392|transcript|ENST00000562893.1|lincRNA|1/2|n.101-3dupT|||||INFO_REALI(
3I1.6|ENSG00000261392|transcript|ENST00000562893.1|lincRNA|1/2|n.101-3dupT|||||INFO_REALI(
3I1.6|ENSG00000261392|transcript|ENST00000562893.1|lincRNA|1/2|n.101-3dupT|||||INFO_REALI(
3I1.6|ENSG00000261392|transcript|ENST00000562893.1|lincRNA|1/2|n.101-3dupT|||||INFO_REALI(
)0166676|transcript|ENST00000572980.5|processed_transcript|1/8|n.124-2A>C|||||,G|splice_accep
|ENST00000327157.3|protein_coding|1/1|c.217_218dupAA|p.Leu74fs|264/424|218/312|73/103||,CT
teraction|3MXN:B_56-B_108:ENST00000312499|protein_coding|1/2|c.168G>A|||||,A|structural_in
teraction|3MXN:B_103-B_123:ENST00000312499|protein_coding|2/2|c.369A>C|||||,C|structural_ir
script|ENST00000598234.5|protein_coding|42/65|c.6334_6338delACCCA|p.Thr2112fs|6491/10568|6
script|ENST00000598234.5|protein_coding|39/65|c.5885_5886delCG|p.Ala1962fs|6039/10568|5885,
:NSG00000261216|transcript|ENST00000562802.1|antisense|1/1|n.272_272+1insCCAG|||||INFO_RI
)0000261216|transcript|ENST00000562802.1|antisense|1/1|n.272+1delG|||||INFO_REALIGN_3_PRI
)0000261216|transcript|ENST00000562802.1|antisense|1/1|n.272+1G>C|||||,C|upstream_gene_var
script|ENST00000620285.1|protein_coding|4/6|c.347_348delAT|p.His116fs|402/1216|347/753|116/2
GH|TNFRSF17|ENSG00000048462|transcript|ENST00000562385.1|protein_coding|2/3|c.251-13_251-
GH|TNFRSF17|ENSG00000048462|transcript|ENST00000562385.1|protein_coding|2/3|c.251-13_251-
GH|TNFRSF17|ENSG00000048462|transcript|ENST00000562385.1|protein_coding|2/3|c.251-13_251-
GH|TNFRSF17|ENSG00000048462|transcript|ENST00000562385.1|protein_coding|2/3|c.251-13_251-
GH|TNFRSF17|ENSG00000048462|transcript|ENST00000562385.1|protein_coding|2/3|c.251-13_251-
00000262116|transcript|ENST00000571939.1|antisense|1/2|n.104-1G>A|||||,T|synonymous_variar
I|RP11-1212A22.1|ENSG00000205746|transcript|ENST00000525846.6|transcribed_unprocessed_pseu
)00304381.9|protein_coding|16/16|c.2127C>A|p.Tyr709*|2257/3640|2127/2172|709/723||,A|stop_
i00000260430|transcript|ENST00000568032.1|lincRNA|1/2|n.145+2C>T|||||
ot|ENST00000563649.1|protein_coding|2/2|c.173dupC|p.Pro59fs|175/586|174/219|58/72||WARNIN
ot|ENST00000563649.1|protein_coding|2/2|c.173dupC|p.Pro59fs|175/586|174/219|58/72||WARNIN
ot|ENST00000563649.1|protein_coding|2/2|c.173dupC|p.Pro59fs|175/586|174/219|58/72||WARNIN
74628|transcript|ENST00000320394.10|protein_coding|1/9|c.-612-1G>C|||||,C|splice_acceptor_va
iG00000262995|transcript|ENST00000575772.1|antisense|2/3|n.254-1G>A|||||,T|upstream_gene_
)0000573854.5|protein_coding|3/14|c.343C>T|p.Arg115*|457/2837|343/1734|115/577||,T|stop_ga
7|interaction|2VZE:A_241-A_368:ENST00000573854|protein_coding|9/14|c.1104A>G|||||,G|structu
)0000569163.1|protein_coding|4/4|c.335C>A|p.Ser112*|335/592|335/450|112/149||WARNING_TRA
56743|transcript|ENST00000519745.5|nonsense_mediated_decay|9/10|c.*580+2T>A|||||,T|splice_
)000289416.9|protein_coding|10/14|c.1323C>G|p.Tyr441*|1798/2845|1323/1761|441/586||,G|stop
)0011638|transcript|ENST00000573688.1|nonsense_mediated_decay|3/3|c.*169+1G>A|||||,A|dow
nteraction|2I99:A_222-A_288:ENST00000219599|protein_coding|9/10|c.864C>G|||||,C|structural_i
i00000273956|transcript|ENST00000613759.1|lincRNA|1/1|n.41-2delA|||||
)0000197006|transcript|ENST00000358154.7|protein_coding|1/4|c.166-3dupT|||||INFO_REALIGN_
)0000197006|transcript|ENST00000358154.7|protein_coding|1/4|c.166-3dupT|||||INFO_REALIGN_
:|ENST00000331057.8|nonsense_mediated_decay|1/7|c.26delG|p.Gly9fs|119/1359|26/117|9/38||IN
ENST00000562944.5|protein_coding|4/4|c.299dupG|p.Pro101fs|336/388|299/351|100/116||WARNI
)00300113.2|protein_coding|7/7|c.562C>T|p.Gln188*|985/2382|562/591|188/196||,T|upstream_ger
IGH|RP11-266L9.1|ENSG00000260714|transcript|ENST00000569988.1|processed_pseudogene|2/2|n.
IGH|RP11-266L9.1|ENSG00000260714|transcript|ENST00000569988.1|processed_pseudogene|2/2|n.
IGH|RP11-266L9.1|ENSG00000260714|transcript|ENST00000569988.1|processed_pseudogene|2/2|n.
on_coding_transcript_exon_variant|HIGH|RP11-266L9.1|ENSG00000260714|transcript|ENST0000056
on_coding_transcript_exon_variant|HIGH|RP11-266L9.1|ENSG00000260714|transcript|ENST0000056

on_coding_transcript_exon_variant|HIGH|RP11-266L9.1|ENSG00000260714|transcript|ENST0000056
on_coding_transcript_exon_variant|HIGH|RP11-266L9.1|ENSG00000260714|transcript|ENST0000056
on_coding_transcript_exon_variant|HIGH|RP11-266L9.1|ENSG00000260714|transcript|ENST0000056
on_coding_transcript_exon_variant|HIGH|RP11-266L9.1|ENSG00000260714|transcript|ENST0000056
nteraction|3UYJ:A_340-A_382:ENST00000286096|protein_coding|7/8|c.1020G>C||||||,C|structural_i
56|transcript|ENST00000532254.1|protein_coding|7/7|c.918_919insTTGTC|p.Pro307fs|1604/2016|91
ENST00000567564.1|nonsense_mediated_decay|5/8|c.297dupA|p.Ala100fs|439/788|298/399|100/1
nteraction|3ME9:A_126-A_282:ENST00000317058|protein_coding|10/10|c.846A>G||||||,G|structura
nteraction|3ME9:B_165-B_289:ENST00000317058|protein_coding|10/10|c.867A>G||||||,G|structura
|interaction|1Z29:A_44-A_126:ENST00000335715|protein_coding|2/8|c.131G>A||||||,T|structural_
|interaction|1Z29:A_19-A_169:ENST00000335715|protein_coding|2/8|c.56C>T||||||,A|structural_ir
|interaction|1LS6:A_19-A_169:ENST00000314752|protein_coding|2/8|c.57G>A||||||,T|structural_in
00168488|transcript|ENST00000566946.5|protein_coding|5/6|c.661-1G>A||||||WARNING_TRANSCR
8188|transcript|ENST00000566209.1|protein_coding|2/3|c.-171+1G>C||||||WARNING_TRANSCRIPT
00000260517|transcript|ENST00000563477.1|lincRNA|2/2|n.276+1G>A||||||,A|downstream_gene_
00183336&ENSG00000260335|gene_variant|ENSG00000183336|||n.29449282_29449171del||||||,A
teraction|4KWV:A_134-A_158:ENST00000395384|protein_coding|2/4|c.473C>T||||||,T|structural_in
interaction|4MJJ:A_193-A_216:ENST00000350119|protein_coding|6/11|c.648G>A||||||,T|structural_
00156858|transcript|ENST00000563211.5|retained_intron|3/3|n.272-3dupT||||||INFO_REALIGN_3_
00156858|transcript|ENST00000563211.5|retained_intron|3/3|n.272-3dupT||||||INFO_REALIGN_3_
NST00000300835.8|protein_coding|8/12|c.828_829delGT|p.Ser277fs|1139/2198|828/1758|276/585
156858|transcript|ENST00000300835.8|protein_coding|8/12|c.836_837insCCCCCCCCCCCCCCC|p.Me
156858|transcript|ENST00000300835.8|protein_coding|8/12|c.836_837insCCCCCCCCCCCCCCC|p.Me
ST00000356166.10|protein_coding|7/18|c.1248_1251delACCA|p.Pro418fs|2336/5200|1248/2943|41
ST00000356166.10|protein_coding|7/18|c.1264_1273delTCCTTGTTCT|p.Ser422fs|2352/5200|1264/2
ST00000356166.10|protein_coding|7/18|c.1267_1273delTTGTTCT|p.Leu423fs|2355/5200|1267/2943
ST00000356166.10|protein_coding|7/18|c.1267_1273delTTGTTCT|p.Leu423fs|2355/5200|1267/2943
ENST00000262519.12|protein_coding|7/19|c.1413_1414delAG|p.Ala472fs|2099/6903|1413/5124|47
167397|transcript|ENST00000394971.7|protein_coding|1/2|c.267+2T>G||||||,C|upstream_gene_var
teraction|1Y01:A_45-A_75:ENST00000302312|protein_coding|3/3|c.224A>T||||||,T|missense_variar
ISG00000261731|transcript|ENST00000569175.2|antisense|1/1|n.241-2A>G||||||,C|upstream_gene
anscript|ENST00000565407.2|IG_V_gene|2/2|c.248_249insTT|p.Lys84fs|249/349|249/349|83/115||
00000260312|transcript|ENST00000563019.1|lincRNA|2/2|n.158+1G>A||||||,T|downstream_gene_
00323584.9|protein_coding|1/31|c.39G>A|p.Trp13*|63/4283|39/3282|13/1093||,A|stop_gained|HI
0000311303.7|protein_coding|22/29|c.3071G>A|p.Trp1024*|3417/5168|3071/4080|1024/1359||,T|
egion_variant&intron_variant|HIGH|ADCY7|ENSG00000121281|transcript|ENST00000254235.7|prote
IST00000394689.2|protein_coding|7/17|c.830_831delAG|p.Glu277fs|836/2145|830/1959|277/652||
ENST00000300589.6|protein_coding|11/12|c.3019dupC|p.Leu1007fs|3125/4486|3020/3123|1007/10
00000261644|transcript|ENST00000563315.2|antisense|3/5|n.1013+2T>G||||||,C|3_prime_UTR_va
00000261749|transcript|ENST00000566439.2|lincRNA|2/2|n.242+1G>A||||||
00000261261|transcript|ENST00000564331.1|lincRNA|3/3|n.410+2T>C||||||,C|downstream_gene_
NST00000558054.1|protein_coding|3/3|c.208dupA|p.Thr70fs|208/444|208/255|70/84||WARNING_T
37245|transcript|ENST00000570308.5|protein_coding|2/13|c.-76+1G>C||||||,C|upstream_gene_vari
nteraction|1CK7:A_416-A_465:ENST00000219070|protein_coding|9/13|c.1393A>C||||||,C|missense
00567238.1|protein_coding|1/12|c.2T>G|p.Met1?|178/3160|2/1539|1/512||,G|sequence_feature|L
125124|transcript|ENST00000566410.1|processed_transcript|1/1|n.147-1G>C||||||,G|missense_vari
05361|transcript|ENST00000463480.6|processed_transcript|1/2|n.48+2T>G||||||,G|splice_donor_va

)125144|transcript|ENST00000379811.3|protein_coding|1/2|c.29-2A>T||||||,A|splice_region_variant
)2900|transcript|ENST00000562496.5|protein_coding|1/4|c.-15+2C>T||||||WARNING_TRANSCRIPT_
00|transcript|ENST00000563486.1|protein_coding|3/3|c.401dupA|p.Tyr134fs|403/635|402/420|134
NST00000538805.5|protein_coding|28/28|c.3497_3500delAGAA|p.Lys1166fs|3498/3552|3497/3540|
000172775|transcript|ENST00000389447.9|protein_coding|6/6|c.*156-1G>A||||||,T|3_prime_UTR_
GH|RPL23AP91|ENSG00000260965|transcript|ENST00000569211.1|processed_pseudogene|1/1|n.27
t|HIGH|RPL23AP91|ENSG00000260965|transcript|ENST00000569211.1|processed_pseudogene|1/1|
AP91|ENSG00000260965|transcript|ENST00000569211.1|processed_pseudogene|1/1|n.277-2delA||
.23AP91|ENSG00000260965|transcript|ENST00000569211.1|processed_pseudogene|1/1|n.277-2dup
PL23AP91|ENSG00000260965|transcript|ENST00000569211.1|processed_pseudogene|1/1|n.277-3_2
RPL23AP91|ENSG00000260965|transcript|ENST00000569211.1|processed_pseudogene|1/1|n.277-4_
)0000565961.5|protein_coding|6/7|c.565C>T|p.Arg189*|685/1814|565/720|189/239||,A|stop_gaine
5170|transcript|ENST00000561918.1|processed_transcript|2/5|n.472+1G>T||||||,A|upstream_gene_
I|CNGB1|ENSG00000070729|transcript|ENST00000251102.12|protein_coding|18/32|c.1643+2_1643+
teraction|4H7W:A_90-A_250:ENST00000219281|protein_coding|7/7|c.748C>G||||||,G|missense_va
0|interaction|3OFM:A_156-A_194:ENST00000262506|protein_coding|7/12|c.582C>T||||||,A|synony
0000260186|transcript|ENST00000567448.1|lincRNA|2/3|n.164+1G>A||||||,A|intron_variant|MODI
nteraction|3QXY:A_37-A_89:ENST00000219315|protein_coding|2/8|c.265T>C||||||,C|structural_inte
action|4CRU:A_1572-B_60:ENST00000317147-ENST00000273064|protein_coding|34/49|c.4716C>T|||
ENST00000441024.6|protein_coding|31/31|c.4629delT|p.Leu1544fs|4914/4999|4629/4656|1543/155
interaction|4CRU:A_1376-A_1495:ENST00000317147|protein_coding|30/49|c.4128G>A||||||,T|syno
nscript|ENST00000614215.2|protein_coding|1/1|c.569delC|p.Pro190fs|569/1026|569/1026|190/341
0619158.1|protein_coding|1/1|c.3G>A|p.Met1?|4/3639|3/186|1/61||,T|missense_variant|MODERA
0000260364|transcript|ENST00000562656.1|lincRNA|1/4|n.125+1G>A||||||,A|intron_variant|MODI
00261742|transcript|ENST00000569736.5|lincRNA|4/8|n.486+2T>C||||||,G|splice_donor_variant&in
00261742|transcript|ENST00000569736.5|lincRNA|2/8|n.217+2T>C||||||,G|splice_donor_variant&in
6548|transcript|ENST00000564917.5|protein_coding|8/9|c.619-2A>G||||||,C|upstream_gene_varia
)0000246777|transcript|ENST00000501143.1|lincRNA|1/1|n.1149+1G>A||||||,A|upstream_gene_va
9593|transcript|ENST00000569348.5|nonsense_mediated_decay|2/6|c.*1+2C>T||||||,A|intron_varia
nteraction|4XP7:A_91-A_136:ENST00000565263|protein_coding|6/17|c.273A>G||||||,G|structural_in
SG00000168807|gene_variant|ENSG00000265113|||n.69241929_69241941delAAAAAAAAAAAAA||||
SG00000168807|gene_variant|ENSG00000265113|||n.69241929_69241941delAAAAAAAAAAAAA||||
SG00000168807|gene_variant|ENSG00000265113|||n.69241929_69241941delAAAAAAAAAAAAA||||
SG00000168807|gene_variant|ENSG00000265113|||n.69241929_69241941delAAAAAAAAAAAAA||||
nteraction|4CF6:A_139-A_183:ENST00000320623|protein_coding|4/6|c.415C>T||||||,A|missense_va
eraction|4DKC:A_50-A_166:ENST00000288098|protein_coding|2/6|c.148C>A||||||,A|structural_inte
eraction|4DKD:B_98-B_168:ENST00000288098|protein_coding|4/6|c.294C>T||||||,T|structural_inter
eraction|4DKF:A_102-A_123:ENST00000288098|protein_coding|4/6|c.367G>C||||||,C|structural_inte
)0288098.6|protein_coding|6/6|c.639C>A|p.Tyr213*|1022/1607|639/729|213/242||,A|stop_gained|
57423|transcript|ENST00000288168.14|protein_coding|1/14|c.28+2T>C||||||,G|splice_donor_varian
nscript|ENST00000536027.1|nonsense_mediated_decay|1/5|c.-1_5delCATGCC|p.Met1fs||1/258|1/8
0000620267.1|protein_coding|27/30|c.4756C>T|p.Arg1586*|4756/5199|4756/5199|1586/1732||,A|
ENST00000620267.1|protein_coding|22/30|c.3695_3698delCAAA|p.Thr1232fs|3698/5199|3695/519
0000620267.1|protein_coding|15/30|c.2365C>T|p.Arg789*|2365/5199|2365/5199|789/1732||
00000260884|transcript|ENST00000564455.1|lincRNA|1/1|n.25-1G>T||||||,T|upstream_gene_varia
)153774|transcript|ENST00000612761.1|protein_coding|1/1|c.30-2A>G||||||WARNING_TRANSCRIPT
00000569540.5|protein_coding|1/3|c.1A>G|p.Met1?|167/2331|1/321|1/106||,C|upstream_gene_va

SG00000260511|transcript|ENST00000562685.1|sense_intronic|1/2|n.37-2A>G||||||,G|downstream_
script|ENST00000402655.6|protein_coding|5/5|c.782_786dupAAGTA|p.Ser263fs|912/1594|787/936|
0166446|transcript|ENST00000567924.1|processed_transcript|1/1|n.163-2A>G||||||,C|upstream_ger
000561614.5|nonsense_mediated_decay|3/4|c.139G>T|p.Gly47*|141/683|139/162|47/53||,A|3_prin
NST00000561614.5|nonsense_mediated_decay|3/4|c.134delC|p.Pro45fs|136/683|134/162|45/53||,T
0299572.9|protein_coding|7/7|c.613T>G|p.Ter205Gluext*?|1349/1398|613/615|205/204||,G|3_prin
000166451|transcript|ENST00000393335.7|protein_coding|10/10|c.938-3dupT||||||INFO_REALIGN_
000166451|transcript|ENST00000393335.7|protein_coding|10/10|c.938-3dupT||||||INFO_REALIGN_
00533478.5|polymorphic_pseudogene|29/32|c.4889G>C|p.Ter1630Serext*?|5000/5669|4889/5324|1
ENSG00000166473|transcript|ENST00000533478.5|polymorphic_pseudogene|25/32|c.4186_4187insC
0000533478.5|polymorphic_pseudogene|20/32|c.3286C>T|p.Gln1096*|3397/5669|3286/5324|1096/
0000533478.5|polymorphic_pseudogene|17/32|c.2668A>T|p.Lys890*|2779/5669|2668/5324|890/17
0000533478.5|polymorphic_pseudogene|8/32|c.1063C>T|p.Arg355*|1174/5669|1063/5324|355/177
ENST00000525539.5|polymorphic_pseudogene|4/43|c.706_707delAA|p.Asn236fs|707/7379|706/737
0000525539.5|polymorphic_pseudogene|4/43|c.658C>T|p.Gln220*|658/7379|658/7379|220/2458||
_coding_transcript_exon_variant|HIGH|RP11-510J16.6|ENSG00000276519|transcript|ENST000006146
00000328945.6|protein_coding|3/3|c.88C>T|p.Gln30*|216/11566|88/1182|30/393||,A|stop_gained
0000261029|transcript|ENST00000563841.1|lincRNA|1/1|n.52+1G>T||||||,T|upstream_gene_variant
G00000261410|transcript|ENST00000564635.1|antisense|2/2|n.209+1G>C||||||,G|intron_variant|M
ENST00000561562.5|protein_coding|3/4|c.364C>T|p.Arg122*|365/723|364/525|122/174||WARNING
166558|transcript|ENST00000569816.1|protein_coding|1/2|c.-151+1G>C||||||WARNING_TRANSCRI
154099|transcript|ENST00000570298.5|retained_intron|9/10|n.4151+1G>T||||||,T|splice_region_va
00064270|transcript|ENST00000416219.6|protein_coding|25/27|c.2569-2A>C||||||,C|splice_accepto
G00000261243|transcript|ENST00000568771.1|antisense|1/1|n.177+1G>C||||||,C|missense_variant|
0103196|transcript|ENST00000563066.5|protein_coding|1/8|c.-232+2T>C||||||WARNING_TRANSCRI
G00000135709|transcript|ENST00000566428.5|protein_coding|2/12|c.330-3dupT||||||INFO_REALIG
G00000135709|transcript|ENST00000566428.5|protein_coding|2/12|c.330-3dupT||||||INFO_REALIG
G00000135709|transcript|ENST00000566428.5|protein_coding|2/12|c.330-3dupT||||||INFO_REALIG
131149|transcript|ENST00000574293.1|processed_transcript|1/1|n.533-2A>G||||||,G|intron_variant
00179219|transcript|ENST00000366314.4|lincRNA|1/1|n.600+2C>T||||||,T|upstream_gene_variant|
ST00000405402.6|protein_coding|4/15|c.477_478delGG|p.Ala160fs|686/4366|477/3342|159/1113|
intron_variant|HIGH|GSE1|ENSG00000131149|transcript|ENST00000405402.6|protein_coding|4/15|
00000269898|transcript|ENST00000602789.1|antisense|1/1|n.395+1G>C||||||,C|upstream_gene_va
968|transcript|ENST00000562492.5|protein_coding|1/4|c.-60+2T>C||||||,C|upstream_gene_variant|
0000269826|transcript|ENST00000595705.1|lincRNA|1/2|n.63+1G>C||||||,C|upstream_gene_variant
0000168367|transcript|ENST00000599664.5|lincRNA|1/3|n.134-2A>T||||||,A|upstream_gene_variar
000168367|transcript|ENST00000595169.5|lincRNA|1/2|n.89+2dupT||||||,TA|splice_donor_variant&
00000261744|transcript|ENST00000563243.1|antisense|2/3|n.289+2T>A||||||,T|intron_variant|MOD
ENST00000319555.7|protein_coding|10/10|c.1331_1332delAG|p.Glu444fs|1653/5380|1331/3021|44
ENST00000319555.7|protein_coding|10/10|c.1335delT|p.Leu446fs|1657/5380|1335/3021|445/1006|
teraction|3D4J:A_201-A_368:ENST00000301012|protein_coding|9/10|c.1104A>G||||||,C|structural_
167508|transcript|ENST00000565610.5|nonsense_mediated_decay|1/4|c.71-1G>T||||||,A|upstream_
t&intron_variant|HIGH|PIEZO1|ENSG00000103335|transcript|ENST00000301015.13|protein_coding|
interaction|4FDI:A_32-A_282:ENST00000268695|protein_coding|8/14|c.846C>T||||||,A|structural_ir
interaction|4FDJ:A_40-A_236:ENST00000268695|protein_coding|7/14|c.708C>T||||||,A|structural_ir
nteraction|4FDI:A_138-A_231:ENST00000268695|protein_coding|7/14|c.692C>G||||||,C|structural_
interaction|4FDI:A_106-A_311:ENST00000268695|protein_coding|3/14|c.318C>T||||||,A|structural_

CATCCAGTCCAGGTGGGCCT|frameshift_variant|HIGH|TRAPPC2L|ENSG00000167515|transcript|ENST0(
0000289746.2|protein_coding|14/14|c.2364C>A|p.Tyr788*|2429/2847|2364/2445|788/814||,A|dow
00178773|transcript|ENST00000268720.9|protein_coding|6/16|c.733-3dupC|||||INFO_REALIGN_3_
ipt|ENST00000301031.8|protein_coding|3/3|c.406_407insTG|p.His136fs|407/2190|407/420|136/13
0000563510.5|nonsense_mediated_decay|2/6|c.146C>G|p.Ser49*|147/633|146/153|49/50||WARNIN
0187741|transcript|ENST00000567284.6|processed_transcript|5/5|n.447-2A>G||||||,C|upstream_ge
141002|transcript|ENST00000565404.1|protein_coding|3/3|c.333_334insTGGCTGCCCTTCTCTG|p.Arg
141002|transcript|ENST00000565404.1|protein_coding|3/3|c.333_334insTGGCTGCCCTTCTCTG|p.Arg
141002|transcript|ENST00000565404.1|protein_coding|3/3|c.333_334insTGGCTGCCCTTCTCTG|p.Arg
pt|ENST00000314994.4|protein_coding|1/1|c.102dupT|p.Glu35fs|714/2744|102/564|34/187||,CA|u
000223959|transcript|ENST00000431757.5|processed_transcript|3/3|n.414-2A>G||||||,G|upstream_
0223959|transcript|ENST00000458301.5|processed_transcript|2/4|n.238+1G>A||||||,A|downstream
00565062.5|nonsense_mediated_decay|3/6|c.165C>A|p.Cys55*|234/1394|165/336|55/111||,A|5_p
00565062.5|nonsense_mediated_decay|3/6|c.241C>T|p.Arg81*|310/1394|241/336|81/111||,T|sync
|ENST00000449207.6|protein_coding|1/10|c.13dupA|p.Arg5fs|33/2008|13/1479|5/492||,CT|frames
_coding_transcript_exon_variant|HIGH|RPH3AL|ENSG00000181031|transcript|ENST00000572075.5|p
nscript|ENST00000570699.1|nonsense_mediated_decay|1/4|c.281_285dupCGGGC|p.Ser96fs|365/23
593|transcript|ENST00000575455.5|retained_intron|1/4|n.129+1G>A||||||,T|intron_variant|MODIFI
interaction|1UW5:A_59-A_84:ENST00000313486|protein_coding|3/12|c.177C>T||||||,A|structural_i
interaction|1UW5:A_6-A_137:ENST00000313486|protein_coding|1/12|c.18G>A||||||,T|structural_in
teraction|4IJH:A_4-A_103:ENST00000254719|protein_coding|1/17|c.12A>G||||||,G|structural_intera
teraction|1JMC:A_351-A_410:ENST00000254719|protein_coding|11/17|c.1051A>G||||||,G|missense
teraction|1FGU:B_306-B_352:ENST00000254719|protein_coding|11/17|c.1056C>T||||||,T|synonymo
2361|transcript|ENST00000574210.5|retained_intron|1/8|n.93+2T>G||||||,C|splice_donor_variant&i
000574051.5|protein_coding|2/6|c.2T>C|p.Met1?|46/672|2/628|1/208||WARNING_TRANSCRIPT_IN
H|SPATA22|ENSG00000141255|transcript|ENST00000572969.5|protein_coding|1/8|c.-74+2_-74+3del
teraction|2Q51:A_231-A_292:ENST00000263080|protein_coding|5/6|c.693C>T||||||,T|structural_int
000577016.5|nonsense_mediated_decay|4/7|c.394C>T|p.Arg132*|396/1621|394/438|132/145||WA
0616411.4|protein_coding|1/17|c.3G>A|p.Met1?|3/5742|3/2328|1/775||,T|3_prime_UTR_variant|M
NST00000547178.5|protein_coding|3/12|c.333delC|p.Asn112fs|424/1998|333/1266|111/421||,T|fra
000574584.1|protein_coding|4/4|c.454C>T|p.Arg152*|497/583|454/540|152/179||WARNING_TRANS
G00000261898|transcript|ENST00000570493.2|antisense|1/1|n.141+1delG||||||,G|3_prime_UTR_va
t|ENST00000592813.5|protein_coding|13/13|c.2046delT|p.Pro683fs|2085/2274|2046/2112|682/70
t|ENST00000592813.5|protein_coding|13/13|c.2049_2050delTG|p.Ala684fs|2088/2274|2049/2112|
32853|transcript|ENST00000441199.2|protein_coding|2/2|c.*2+1delG||||||,A|upstream_gene_varian
G00000141503|transcript|ENST00000571207.5|nonsense_mediated_decay|9/27|c.934-7_934-3dupCC
G00000141503|transcript|ENST00000571207.5|nonsense_mediated_decay|9/27|c.934-7_934-3dupCC
G00000141503|transcript|ENST00000571207.5|nonsense_mediated_decay|9/27|c.934-7_934-3dupCC
T00000381365.3|protein_coding|3/3|c.456G>A|p.Trp152*|683/3199|456/573|152/190||,A|3_prime
IGH|GP1BA|ENSG00000185245|transcript|ENST00000611961.1|protein_coding|2/2|c.1272+39_1273
NST00000329125.5|protein_coding|2/2|c.1305_1320delCACCCCAGAGCCCACC|p.Thr436fs|1380/2501
NST00000329125.5|protein_coding|2/2|c.1305_1320delCACCCCAGAGCCCACC|p.Thr436fs|1380/2501
NST00000329125.5|protein_coding|2/2|c.1322_1344delCAGAGCCCGCCCCCAGCCCGACC|p.Ser441fs|1
NST00000329125.5|protein_coding|2/2|c.1601_1602delAT|p.Tyr534fs|1676/2501|1601/1959|534/6
_coding_transcript_exon_variant|HIGH|RP11-333E1.2|ENSG00000263164|transcript|ENST00000575O5
on_coding_transcript_exon_variant|HIGH|RP11-333E1.2|ENSG00000263164|transcript|ENST0000057
script|ENST00000573772.1|antisense|1/1|n.115-6726_115-6644delCATGTTCCTGCCCTACCCTAACTAAT

ISG00000263164|transcript|ENST00000575056.1|lincRNA|1/2|n.339-2_339-1insT||||||
nscript|ENST00000573772.1|antisense|1/1|n.115-6733_115-6732insA||||||
G00000263164|transcript|ENST00000575056.1|lincRNA|1/2|n.339-2A>G||||||
script|ENST00000573772.1|antisense|1/1|n.115-6732T>C||||||
interaction|1P32:A_130-A_171:ENST00000225698|protein_coding|3/6|c.389C>T||||||,A|structural_i
nteraction|4IM6:A_946-A_978:ENST00000572272|protein_coding|8/17|c.2934A>G||||||,C|structura
nteraction|4IM6:A_947-A_970:ENST00000572272|protein_coding|8/17|c.2910A>G||||||,C|structura
nteraction|4IM6:A_878-A_904:ENST00000572272|protein_coding|6/17|c.2633C>T||||||,A|structural
)091592|transcript|ENST00000576905.5|protein_coding|1/2|c.-354-1G>C||||||WARNING_TRANSCRI
79314|transcript|ENST00000563763.5|processed_transcript|2/3|n.830+1G>A||||||,A|TF_binding_sit
IGH|RP11-636N17.1|ENSG00000262818|transcript|ENST00000570978.1|processed_pseudogene|1/1|
IGH|RP11-636N17.1|ENSG00000262818|transcript|ENST00000570978.1|processed_pseudogene|1/1|
IGH|RP11-636N17.1|ENSG00000262818|transcript|ENST00000570978.1|processed_pseudogene|1/1|
)091622|transcript|ENST00000262483.12|protein_coding|19/19|c.2619+2T>G||||||,C|splice_donor_
)129235|transcript|ENST00000576020.5|processed_transcript|1/3|n.116+1G>A||||||,A|structural_ir
530|transcript|ENST00000576459.1|processed_transcript|2/2|n.240+2T>C||||||,C|upstream_gene_v
00000219200|gene_variant|ENSG00000219200|||n.7014471_7014495delTGTTCCGTTTCTCCACCCTTCG
teraction|3I4W:A_360-A_389:ENST00000399506|protein_coding|10/20|c.1167C>T||||||,A|structura
ST00000005340.9|protein_coding|1/15|c.9_18delTAGCAGCACT|p.Ser4fs|301/3018|9/2211|3/736||,
573657.5|protein_coding|4/4|c.538T>G|p.Ter180Gluext*?|665/687|538/540|180/179||,G|stop_lost|
)0574993.5|protein_coding|6/6|c.818G>A|p.Trp273*|1035/2209|818/840|273/279||,A|stop_gained
NST00000575313.1|protein_coding|2/5|c.27delC|p.Ala10fs|51/605|27/581|9/192||WARNING_TRAN
ENST00000399464.6|protein_coding|1/29|c.66delT|p.Gly24fs|82/5200|66/4689|22/1562||,C|frame
ENST00000399464.6|protein_coding|1/29|c.55_56delCC|p.Pro19fs|72/5200|55/4689|19/1562||,C|f
nteraction|4NSW:A_13-A_166:ENST00000158762|protein_coding|6/22|c.498C>T||||||,T|structural_i
1292|transcript|ENST00000570896.5|protein_coding|1/13|c.-233+2T>G||||||,G|upstream_gene_vari
174292|transcript|ENST00000576812.5|protein_coding|9/12|c.1413-2A>G||||||,G|splice_acceptor_v
it&intron_variant|HIGH|TMEM102|ENSG00000181284|transcript|ENST00000323206.1|protein_coding
00000412468.3|protein_coding|2/2|c.977G>A|p.Trp326*|1162/1697|977/1017|326/338||,A|upstrea
ENST00000617998.4|protein_coding|29/29|c.5513_5514delCA|p.Thr1838fs|5912/6751|5513/5943|1
ariant&3_prime_UTR_variant&intron_variant|HIGH|SENP3|ENSG00000161956|transcript|ENST00000
ariant&3_prime_UTR_variant&intron_variant|HIGH|SENP3|ENSG00000161956|transcript|ENST00000
ariant&3_prime_UTR_variant&intron_variant|HIGH|SENP3|ENSG00000161956|transcript|ENST00000
ariant&3_prime_UTR_variant&intron_variant|HIGH|SENP3|ENSG00000161956|transcript|ENST00000
_variant|HIGH|SENP3|ENSG00000161956|transcript|ENST00000429205.6|protein_coding|11/12|c.*3
_variant|HIGH|SENP3|ENSG00000161956|transcript|ENST00000429205.6|protein_coding|11/12|c.*3
_variant|HIGH|SENP3|ENSG00000161956|transcript|ENST00000429205.6|protein_coding|11/12|c.*3
_variant|HIGH|SENP3|ENSG00000161956|transcript|ENST00000429205.6|protein_coding|11/12|c.*3
_variant|HIGH|SENP3|ENSG00000161956|transcript|ENST00000429205.6|protein_coding|11/12|c.*3
iant&3_prime_UTR_variant&intron_variant|HIGH|SENP3|ENSG00000161956|transcript|ENST0000042
iant&3_prime_UTR_variant&intron_variant|HIGH|SENP3|ENSG00000161956|transcript|ENST0000042
riant&intron_variant|HIGH|SENP3|ENSG00000161956|transcript|ENST00000429205.6|protein_coding
riant&intron_variant|HIGH|SENP3|ENSG00000161956|transcript|ENST00000429205.6|protein_coding
riant&intron_variant|HIGH|SENP3|ENSG00000161956|transcript|ENST00000429205.6|protein_coding
riant&intron_variant|HIGH|SENP3|ENSG00000161956|transcript|ENST00000429205.6|protein_coding
riant&intron_variant|HIGH|SENP3|ENSG00000161956|transcript|ENST00000429205.6|protein_coding
riant&intron_variant|HIGH|SENP3|ENSG00000161956|transcript|ENST00000429205.6|protein_coding

riant&intron_variant|HIGH|SENP3|ENSG00000161956|transcript|ENST00000429205.6|protein_coding
riant&intron_variant|HIGH|SENP3|ENSG00000161956|transcript|ENST00000429205.6|protein_coding
riant&intron_variant|HIGH|SENP3|ENSG00000161956|transcript|ENST00000429205.6|protein_coding
riant&intron_variant|HIGH|SENP3|ENSG00000161956|transcript|ENST00000429205.6|protein_coding
riant&intron_variant|HIGH|SENP3|ENSG00000161956|transcript|ENST00000429205.6|protein_coding
riant&intron_variant|HIGH|SENP3|ENSG00000161956|transcript|ENST00000429205.6|protein_coding
riant&intron_variant|HIGH|SENP3|ENSG00000161956|transcript|ENST00000429205.6|protein_coding
riant&intron_variant|HIGH|SENP3|ENSG00000161956|transcript|ENST00000429205.6|protein_coding
riant&intron_variant|HIGH|SENP3|ENSG00000161956|transcript|ENST00000429205.6|protein_coding
riant&intron_variant|HIGH|SENP3|ENSG00000161956|transcript|ENST00000429205.6|protein_coding
riant&intron_variant|HIGH|SENP3|ENSG00000161956|transcript|ENST00000429205.6|protein_coding
riant&intron_variant|HIGH|SENP3|ENSG00000161956|transcript|ENST00000429205.6|protein_coding
riant&intron_variant|HIGH|SENP3|ENSG00000161956|transcript|ENST00000429205.6|protein_coding
riant&intron_variant|HIGH|SENP3|ENSG00000161956|transcript|ENST00000429205.6|protein_coding
riant&intron_variant|HIGH|SENP3|ENSG00000161956|transcript|ENST00000429205.6|protein_coding
riant&intron_variant|HIGH|SENP3|ENSG00000161956|transcript|ENST00000429205.6|protein_coding
riant&intron_variant|HIGH|SENP3|ENSG00000161956|transcript|ENST00000429205.6|protein_coding
riant&intron_variant|HIGH|SENP3|ENSG00000161956|transcript|ENST00000429205.6|protein_coding
riant&intron_variant|HIGH|SENP3|ENSG00000161956|transcript|ENST00000429205.6|protein_coding
riant&intron_variant|HIGH|SENP3|ENSG00000161956|transcript|ENST00000429205.6|protein_coding
riant&intron_variant|HIGH|SENP3|ENSG00000161956|transcript|ENST00000429205.6|protein_coding
riant&intron_variant|HIGH|SENP3|ENSG00000161956|transcript|ENST00000429205.6|protein_coding
IGH|SENP3|ENSG00000161956|transcript|ENST00000429205.6|protein_coding|12/12|c.*388-1_*390
IGH|SENP3|ENSG00000161956|transcript|ENST00000429205.6|protein_coding|12/12|c.*388-1_*390
IGH|SENP3|ENSG00000161956|transcript|ENST00000429205.6|protein_coding|12/12|c.*388-1_*390
nteraction|2G9N:A_132-A_156:ENST00000293831|protein_coding|5/11|c.396C>T||||||,T|structural_
ENST00000250124.10|protein_coding|5/7|c.418delG|p.Val140fs|634/1601|418/744|140/247||INFO_
teraction|2BEI:A_89-A_126:ENST00000269298|protein_coding|6/6|c.376C>T||||||,A|structural_inter
ST00000635293.1|nonsense_mediated_decay|11/12|c.1147_1148delTT|p.Leu383fs|1408/1883|1147,
ST00000635293.1|nonsense_mediated_decay|11/12|c.1147_1148delTT|p.Leu383fs|1408/1883|1147,
ST00000635293.1|nonsense_mediated_decay|11/12|c.1147_1148delTT|p.Leu383fs|1408/1883|1147,
00635293.1|nonsense_mediated_decay|11/12|c.1148T>A|p.Leu383*|1408/1883|1148/1233|383/410
00635293.1|nonsense_mediated_decay|11/12|c.1148T>A|p.Leu383*|1408/1883|1148/1233|383/410
teraction|1GZH:A_169-A_213:ENST00000269305|protein_coding|6/11|c.639A>G||||||,C|structural_i
ENST00000254846.9|protein_coding|11/22|c.1641_1642delAA|p.Thr548fs|2030/6713|1641/5049|54
0000333775.9|protein_coding|1/2|c.168G>A|p.Trp56*|599/999|168/522|56/173||,T|5_prime_UTR_v
3|interaction|4NRE:A_86-A_109:ENST00000380183|protein_coding|2/14|c.258C>T||||||,T|structural
3|interaction|4NRE:A_68-A_115:ENST00000380183|protein_coding|2/14|c.345C>G||||||,G|structura
3|interaction|4NRE:A_235-A_328:ENST00000380183|protein_coding|6/14|c.705C>T||||||,T|structura
3|interaction|4NRE:A_480-A_547:ENST00000380183|protein_coding|10/14|c.1440C>T||||||,T|struct
3|interaction|4NRE:A_550-A_620:ENST00000380183|protein_coding|12/14|c.1650C>A||||||,A|struc
3|interaction|4NRE:A_249-A_552:ENST00000380183|protein_coding|12/14|c.1656G>A||||||,A|struc
3|interaction|4NRE:A_176-A_676:ENST00000380183|protein_coding|14/14|c.2026A>G||||||,G|struc
20205|transcript|ENST00000404970.3|protein_coding|1/3|c.-134+2T>C||||||,G|upstream_gene_var
T00000532998.5|protein_coding|3/3|c.302C>G|p.Ser101*|389/2358|302/324|101/107||,C|upstream
00187824|transcript|ENST00000581949.1|protein_coding|2/3|c.155+1G>A||||||WARNING_TRANSCR
H9|ENSG00000007174|transcript|ENST00000262442.8|protein_coding|1/69|c.44_68dupCGGATGGGC

ENST00000425538.5|protein_coding|7/14|c.694_697delATAG|p.Ile232fs|894/8466|694/2961|232/98
pt|ENST00000379672.9|protein_coding|20/21|c.2061_2062delGT|p.Ser688fs|2361/4228|2061/2457
)0141028|transcript|ENST00000577632.1|unprocessed_pseudogene|1/2|n.262+1G>A||||||,A|downs
)000580561.1|nonsense_mediated_decay|3/6|c.182G>A|p.Trp61*|184/1384|182/189|61/62||,A|syn
)266129|transcript|ENST00000584247.1|processed_pseudogene|1/1|n.163+2C>T||||||,A|3_prime_U
GH|KRT16P2|ENSG00000227300|transcript|ENST00000414673.5|transcribed_unprocessed_pseudoge
)128438|transcript|ENST00000261651.6|processed_transcript|12/13|n.3221+1G>A||||||,T|splice_do
ST00000353383.5|protein_coding|3/6|c.837_838delGC|p.Gln280fs|1306/7662|837/5721|279/1906||
ST00000353383.5|protein_coding|3/6|c.837_838delGC|p.Gln280fs|1306/7662|837/5721|279/1906||
|ENST00000353383.5|protein_coding|3/6|c.840delG|p.Gln280fs|1309/7662|840/5721|280/1906||,A
|ENST00000353383.5|protein_coding|3/6|c.840delG|p.Gln280fs|1309/7662|840/5721|280/1906||,A
NST00000395703.8|protein_coding|3/3|c.379delG|p.Asp127fs|520/718|379/492|127/163||,T|upstre
interaction|4CGY:A_268-A_509:ENST00000321105|protein_coding|13/19|c.1527C>T||||||,A|structur
)0205266|transcript|ENST00000444070.3|processed_transcript|1/6|n.216dupT||||||INFO_REALIGN_
|transcript|ENST00000328114.10|protein_coding|1/11|c.-1_1delGA|p.Met1fs|81/1730|1/1071|1/35
000196893|transcript|ENST00000354432.3|antisense|1/1|n.590+2T>C||||||,G|intron_variant|MODI
2134|transcript|ENST00000314728.9|protein_coding|9/10|c.1411+2T>A||||||,A|splice_donor_varian
72210|transcript|ENST00000630662.2|protein_coding|9/9|c.229A>T|p.Arg77*|1376/1558|229/294|
000154898|transcript|ENST00000433419.2|transcribed_processed_pseudogene|16/18|n.3572+2T>G|
98|transcript|ENST00000423676.7|protein_coding|1/11|c.-1_1dupGA|p.Met1fs|65/1713|1/1071|1/
GH|KRT16P3|ENSG00000214822|transcript|ENST00000580621.5|retained_intron|6/6|n.1689-3_168
109016|transcript|ENST00000582161.5|protein_coding|1/4|c.-308+1G>A||||||WARNING_TRANSCRIP
0178130|transcript|ENST00000613377.1|lincRNA|1/1|n.11+2T>G||||||,G|downstream_gene_variant
G00000265246|transcript|ENST00000581944.1|lincRNA|1/1|n.49+1G>T||||||
)0266433|transcript|ENST00000586223.5|processed_transcript|11/12|n.1595+2C>T||||||,T|intron_v
264216|transcript|ENST00000580780.1|transcribed_unprocessed_pseudogene|9/13|n.1132+2T>A|||
|interaction|3GQQ:A_89-A_148:ENST00000335765|protein_coding|2/5|c.267C>G||||||,C|structural_
)076382|transcript|ENST00000580567.6|nonsense_mediated_decay|2/3|c.178-2A>C||||||,G|splice_
ENST00000578181.1|protein_coding|3/3|c.490_491delAT|p.Met164fs|491/593|490/510|164/169||W
G00000264138|transcript|ENST00000582737.1|unprocessed_pseudogene|1/1|n.102-2delA||||||INF
G00000264138|transcript|ENST00000582737.1|unprocessed_pseudogene|1/1|n.102-2delA||||||INF
9761|transcript|ENST00000578748.6|processed_transcript|1/2|n.596+1G>T||||||,T|upstream_gene_
NST00000335960.10|protein_coding|5/18|c.1250dupC|p.Gly418fs|1429/4195|1250/2985|417/994||,
167543|transcript|ENST00000578749.5|protein_coding|1/5|c.-4+2T>C||||||WARNING_TRANSCRIPT_
)0394835.7|protein_coding|1/23|c.3G>A|p.Met1?|195/5132|3/4512|1/1503||,A|start_lost|HIGH|EI
76927|transcript|ENST00000394835.7|protein_coding|1/22|c.42+1G>A||||||,A|splice_donor_varian
)00266733|transcript|ENST00000579181.1|protein_coding|1/4|c.13-1G>A||||||,A|splice_acceptor_v
|ENSG00000250462|transcript|ENST00000417404.2|processed_transcript|1/1|n.1270-3dupT||||||IN
|ENSG00000250462|transcript|ENST00000417404.2|processed_transcript|1/1|n.1270-3dupT||||||IN
|ENSG00000250462|transcript|ENST00000417404.2|processed_transcript|1/1|n.1270-3dupT||||||IN
|ENSG00000250462|transcript|ENST00000417404.2|processed_transcript|1/1|n.1270-3dupT||||||IN
|ENSG00000250462|transcript|ENST00000417404.2|processed_transcript|1/1|n.1270-3dupT||||||IN
71572.6|nonsense_mediated_decay|16/17|c.1918T>C|p.Ter640Argext*?|1919/2028|1918/1920|640/
712|transcript|ENST00000471572.6|nonsense_mediated_decay|16/16|c.*92+1G>A||||||WARNING_T
ENST00000496655.1|nonsense_mediated_decay|3/5|c.233dupG|p.Cys78fs|325/1062|233/249|78/8
)00178691|transcript|ENST00000322652.9|protein_coding|4/15|c.456-3dupT||||||INFO_REALIGN_3_
)00178691|transcript|ENST00000322652.9|protein_coding|4/15|c.456-3dupT||||||INFO_REALIGN_3_

)214708&ENSG00000126858|gene_variant|ENSG00000214708|||n.32142475_32142452delCGGCGGC
)214708&ENSG00000126858|gene_variant|ENSG00000214708|||n.32142475_32142452delCGGCGGC
)214708&ENSG00000126858|gene_variant|ENSG00000214708|||n.32142475_32142452delCGGCGGC
)214708&ENSG00000126858|gene_variant|ENSG00000214708|||n.32142475_32142452delCGGCGGC
)214708&ENSG00000126858|gene_variant|ENSG00000214708|||n.32142475_32142452delCGGCGGC
)214708&ENSG00000126858|gene_variant|ENSG00000214708|||n.32142475_32142452delCGGCGGC
_variant&non_coding_transcript_exon_variant|HIGH|CTC-304I17.6|ENSG00000279668|transcript|ENS
_variant&non_coding_transcript_exon_variant|HIGH|CTC-304I17.6|ENSG00000279668|transcript|ENS
)000285013.10|protein_coding|3/6|c.42C>A|p.Tyr14*|318/8469|42/2694|14/897||,T|stop_gained|H
|ENST00000361112.6|protein_coding|3/5|c.474delC|p.Leu159fs|1353/2752|474/1854|158/617||,A|
)00260908.11|protein_coding|1/4|c.1A>G|p.Met1?|119/3119|1/1767|1/588||,C|upstream_gene_var
36320|transcript|ENST00000415846.3|protein_coding|3/3|c.1904+1G>A||||||,T|upstream_gene_var
nteraction|1E42:A_707-A_871:ENST00000621914|protein_coding|15/21|c.2121T>C||||||,C|structura
nteraction|3I35:A_207-A_229:ENST00000318008|protein_coding|7/7|c.687C>T||||||,T|structural_int
)0000266101|transcript|ENST00000579256.1|antisense|1/1|n.273+1_273+2delGT||||||,G|3_prime_
)67191|transcript|ENST00000577926.1|processed_transcript|1/3|n.119+1G>A||||||,T|upstream_ger
NST00000447079.4|protein_coding|2/14|c.1614_1627delTCTACCAACTACTA|p.Leu539fs|1647/8336|1
)0583884.5|nonsense_mediated_decay|3/7|c.184T>C|p.Ter62Glnext*?|185/602|184/186|62/61||W
|interaction|1EM2:A_289-A_399:ENST00000336308|protein_coding|14/15|c.1197T>C||||||,C|structu
nteraction|1HNN:A_97-A_152:ENST00000269582|protein_coding|3/3|c.456A>G||||||,G|structural_ir
nteraction|1N7I:A_175-A_204:ENST00000269582|protein_coding|3/3|c.523G>A||||||,A|structural_ir
)0073605|transcript|ENST00000360317.7|protein_coding|4/9|c.662-2A>G||||||,C|splice_acceptor_va
ript|ENST00000614384.4|protein_coding|5/5|c.387_390dupGTGT|p.Leu131fs|407/454|390/414|130
ript|ENST00000614384.4|protein_coding|5/5|c.387_390dupGTGT|p.Leu131fs|407/454|390/414|130
ript|ENST00000614384.4|protein_coding|5/5|c.387_390dupGTGT|p.Leu131fs|407/454|390/414|130
8|transcript|ENST00000614384.4|protein_coding|5/5|c.362_363insATGTGTGTGT|p.Met121fs|379/45
8|transcript|ENST00000614384.4|protein_coding|5/5|c.362_363insATGTGTGTGT|p.Met121fs|379/45
8|transcript|ENST00000614384.4|protein_coding|5/5|c.362_363insATGTGTGTGT|p.Met121fs|379/45
8|transcript|ENST00000614384.4|protein_coding|5/5|c.362_363insATGTGTGTGT|p.Met121fs|379/45
|ENST00000394126.5|protein_coding|20/25|c.2492dupG|p.Thr832fs|2911/3896|2492/3045|831/101
NST00000584997.1|protein_coding|1/3|c.18delT|p.Gln7fs|362/543|18/199|6/65||WARNING_TRANS
)00108349|transcript|ENST00000264645.11|protein_coding|6/13|c.786-4_786-3dupGT||||||INFO_RI
579344.1|protein_coding|2/3|c.1A>G|p.Met1?|344/1874|1/1119|1/372||,C|missense_variant|MODI
NST00000264651.2|protein_coding|2/8|c.666delT|p.Asn222fs|723/1881|666/1578|222/525||
intron_variant|HIGH|KRTAP4-7|ENSG00000240871|transcript|ENST00000612231.1|protein_coding|1
t|ENST00000391356.3|protein_coding|1/1|c.64_68delGAGAG|p.Glu22fs|90/964|64/588|22/195||
|transcript|ENST00000391356.3|protein_coding|1/1|c.66_67insTATCTCC|p.Ser23fs|88/964|66/588|2
ion_variant&intron_variant|HIGH|KRTAP4-1|ENSG00000198443|transcript|ENST00000620667.1|prote
)000398470.1|protein_coding|1/1|c.1A>G|p.Met1?|1/753|1/753|1/250||,G|upstream_gene_variant
_variant|HIGH|KRTAP9-1|ENSG00000240542|transcript|ENST00000634235.1|protein_coding|2/2|c.2
)0236473|transcript|ENST00000434019.1|unprocessed_pseudogene|4/6|n.627-1G>A||||||
teraction|3MWD:B_657-B_682:ENST00000352035|protein_coding|18/29|c.2045A>G||||||,C|structur
teraction|3MWD:B_563-B_590:ENST00000352035|protein_coding|16/29|c.1689T>C||||||,G|structur
168256|transcript|ENST00000585955.5|protein_coding|1/2|c.-15+1delG||||||WARNING_TRANSCRIP
nteraction|2W4R:C_544-C_654:ENST00000251642|protein_coding|14/14|c.1961G>A||||||,T|missens
ion_variant&intron_variant|HIGH|KAT2A|ENSG00000108773|transcript|ENST00000225916.9|protein_
intron_variant|HIGH|KAT2A|ENSG00000108773|transcript|ENST00000225916.9|protein_coding|1/18

)8773|transcript|ENST00000225916.9|protein_coding|1/17|c.339+2T>G||||||,C|splice_donor_varian
00108773|transcript|ENST00000225916.9|protein_coding|1/18|c.332_338delCTTGCAA|p.Ala111fs|39
ranscript|ENST00000551338.1|protein_coding|3/3|c.450_451delTT|p.Phe150fs|451/560|450/453|15
ranscript|ENST00000551338.1|protein_coding|3/3|c.450_451delTT|p.Phe150fs|451/560|450/453|15
ranscript|ENST00000551338.1|protein_coding|3/3|c.450_451delTT|p.Phe150fs|451/560|450/453|15
ranscript|ENST00000551338.1|protein_coding|3/3|c.450_451delTT|p.Phe150fs|451/560|450/453|15
G00000033627|transcript|ENST00000592324.1|protein_coding|5/6|c.413_414insA|p.Glu139fs|414/5
pt|ENST00000588138.1|protein_coding|2/7|c.86_95delCAGTCAGCCC|p.Pro29fs|86/573|86/573|29/1
nteraction|2C11:B_397-B_426:ENST00000308423|protein_coding|1/4|c.1277G>A||||||,A|structural_i
3828|transcript|ENST00000592388.1|nonsense_mediated_decay|2/2|c.*120+1G>T||||||WARNING_T
nteraction|1JNX:X_1652-X_1675:ENST00000357654|protein_coding|15/23|c.4956G>A||||||,T|structu
3496|transcript|ENST00000467245.5|processed_transcript|3/7|n.315+2T>C||||||,C|downstream_ger
nteraction|3I4U:A_1017-A_1068:ENST00000262415|protein_coding|20/23|c.3049G>A||||||,A|misser
ENSG00000267747|transcript|ENST00000588996.1|antisense|2/2|n.110-3dupT||||||INFO_REALIGN_3
ENSG00000267747|transcript|ENST00000588996.1|antisense|2/2|n.110-3dupT||||||INFO_REALIGN_3
interaction|1J4X:A_61-A_86:ENST00000226004|protein_coding|2/3|c.183C>T||||||,A|structural_inte
3852|transcript|ENST00000461854.5|protein_coding|4/13|c.222+1G>A||||||,T|intron_variant|MODI
00000267080|transcript|ENST00000591166.1|antisense|3/3|n.321-1G>C||||||,G|splice_acceptor_var
68591|transcript|ENST00000587630.1|protein_coding|2/2|c.*22+1G>C||||||WARNING_TRANSCRIPT
0178852|transcript|ENST00000518170.1|protein_coding|1/1|c.-86+1G>A||||||WARNING_TRANSCRIP
00000331493.6|protein_coding|9/25|c.631C>T|p.Arg211*|1042/3931|631/2922|211/973||,T|downs
00000331493.6|protein_coding|10/25|c.706C>T|p.Arg236*|1117/3931|706/2922|236/973||,T|intro
000178852|transcript|ENST00000331493.6|protein_coding|10/24|c.806-1G>A||||||,A|splice_accepto
00000331493.6|protein_coding|12/25|c.1297A>T|p.Lys433*|1708/3931|1297/2922|433/973||,T|sto
T00000331493.6|protein_coding|15/25|c.1638T>G|p.Tyr546*|2049/3931|1638/2922|546/973||,G|st
interaction|3W5K:A_195-A_240:ENST00000290158|protein_coding|5/22|c.585G>A||||||,A|synonym
nteraction|1QGK:A_544-A_596:ENST00000290158|protein_coding|13/22|c.1632T>C||||||,C|structur
NST00000376681.7|nonsense_mediated_decay|2/14|c.157_173delTGCCCGACTTTGGAAAG|p.Cys53fs|
0159210|transcript|ENST00000507302.6|nonsense_mediated_decay|4/9|c.246delA|p.Gly83fs|259/74
H|PDK2|ENSG00000005882|transcript|ENST00000007708.7|protein_coding|1/12|c.-233_-230+1delAT
teraction|4MP2:A_80-A_136:ENST00000503176|protein_coding|2/11|c.240C>T||||||,T|structural_in
NST00000511303.5|protein_coding|6/6|c.573delA|p.Gly193fs|573/668|573/591|191/196||,G|3_prim
SG00000249406|transcript|ENST00000509943.1|lincRNA|2/4|n.224-2dupA||||||INFO_REALIGN_3_P
ENSG00000249406|transcript|ENST00000509943.1|lincRNA|4/4|n.481-3dupT||||||INFO_REALIGN_3_
ENSG00000249406|transcript|ENST00000509943.1|lincRNA|4/4|n.481-3dupT||||||INFO_REALIGN_3_
ENSG00000249406|transcript|ENST00000509943.1|lincRNA|4/4|n.481-3dupT||||||INFO_REALIGN_3_
GH|MRPL27|ENSG00000108826|transcript|ENST00000508200.5|protein_coding|2/2|c.187-8_187-1d
nteraction|2ZIU:B_311-B_357:ENST00000338165|protein_coding|4/9|c.933A>T||||||,T|structural_int
H|SPATA20|ENSG00000006282|transcript|ENST00000505559.5|nonsense_mediated_decay|2/7|c.*23
3678|transcript|ENST00000512737.5|protein_coding|1/4|c.-5+1G>A||||||,A|splice_donor_variant&i
00166263|transcript|ENST00000398391.6|protein_coding|4/10|c.57-1G>A||||||,A|splice_region_vari
teraction|1LN2:A_10-A_121:ENST00000268896|protein_coding|1/6|c.29A>C||||||,C|structural_intera
teraction|1LN1:A_131-A_161:ENST00000268896|protein_coding|4/6|c.483C>T||||||,T|structural_int
script|ENST00000566473.6|protein_coding|19/20|c.2624_2625insCCCCC|p.Glu876fs|2625/3840|262
00000153930|transcript|ENST00000566473.6|protein_coding|19/20|c.2624_2625insCCCCCC|p.Pro875
G00000153944&ENSG00000279281|gene_variant|ENSG00000279281|||n.57450284_57450294delAAA
G00000153944&ENSG00000279281|gene_variant|ENSG00000279281|||n.57450284_57450294delAAA

G00000153944&ENSG00000279281|gene_variant|ENSG00000279281|||n.57450284_57450294delAAA
G00000153944&ENSG00000279281|gene_variant|ENSG00000279281|||n.57450284_57450294delAAA
NSG00000279281|gene_variant|ENSG00000279281|||n.57450288_57450298delAAAGAAAGAAA|||||
0225371.5|protein_coding|6/13|c.771C>A|p.Cys257*|881/2708|771/2148|257/715||,A|sequence_fe
005381|transcript|ENST00000225275.3|protein_coding|11/11|c.2031-2A>C||||||,G|downstream_ger
G00000265148|transcript|ENST00000580515.5|antisense|2/3|n.370-2A>G||||||,G|splice_acceptor_
5|interaction|3H7H:A_5-A_34:ENST00000225504|protein_coding|1/5|c.14C>A||||||,T|structural_inte
0000580122.5|nonsense_mediated_decay|5/6|c.307C>T|p.Gln103*|308/482|307/339|103/112||WA
ENST00000592145.1|protein_coding|3/3|c.7delG|p.Val3fs|390/544|7/161|3/52||WARNING_TRANSCR
00000267772|transcript|ENST00000592917.1|lincRNA|2/3|n.188+1G>A||||||,A|upstream_gene_vari
0267280|transcript|ENST00000592009.1|antisense|1/1|n.40+1G>A||||||,T|TF_binding_site_variant|
.21075|transcript|ENST00000589449.5|processed_transcript|5/7|n.683-2A>C||||||,C|downstream_g
0000521764.2|protein_coding|1/1|c.589C>T|p.Arg197*|611/777|589/648|197/215||
00172421|transcript|ENST00000635817.1|processed_transcript|4/6|n.461-1A>G||||||,G|upstream_g
000239527|transcript|ENST00000466210.1|processed_pseudogene|1/1|n.349-2_349-1delAA||||||INF
000239527|transcript|ENST00000466210.1|processed_pseudogene|1/1|n.349-2_349-1delAA||||||INF
000239527|transcript|ENST00000466210.1|processed_pseudogene|1/1|n.349-2_349-1delAA||||||INF
00173838|transcript|ENST00000583600.5|protein_coding|5/11|c.496+2T>C||||||,G|upstream_gene_
0582005.5|nonsense_mediated_decay|3/7|c.345C>A|p.Tyr115*|369/982|345/438|115/145||,A|ups
T00000428043.5|protein_coding|24/24|c.3712delT|p.Ser1238fs|3746/5120|3712/3729|1238/1242||
3592|transcript|ENST00000584574.5|protein_coding|1/3|c.-165+2C>T||||||WARNING_TRANSCRIPT_
pt|ENST00000323347.14|protein_coding|1/13|c.7_10delAGGC|p.Arg3fs|57/1935|7/1452|3/483||,C|
teraction|3P23:A_800-A_934:ENST00000433197|protein_coding|22/22|c.2802A>G||||||,C|synonym
00258890|transcript|ENST00000556440.6|protein_coding|2/19|c.149-3dupC||||||INFO_REALIGN_3_
00258890|transcript|ENST00000556440.6|protein_coding|2/19|c.149-3dupC||||||INFO_REALIGN_3_
00000214176|transcript|ENST00000580919.5|processed_transcript|5/6|n.857-1T>G||||||,C|splice_a
nteraction|4JHS:A_300-A_325:ENST00000205948|protein_coding|7/8|c.973T>G||||||,C|missense_va
000334461.7|protein_coding|2/3|c.226T>C|p.Ter76Argext*?|541/1535|226/228|76/75||,G|intron_v
interaction|3WPT:A_226-A_267:ENST00000330459|protein_coding|7/11|c.801A>G||||||,G|structura
000182481|transcript|ENST00000330459.7|protein_coding|9/10|c.1348-3dupT||||||INFO_REALIGN_
000182481|transcript|ENST00000330459.7|protein_coding|9/10|c.1348-3dupT||||||INFO_REALIGN_
NST00000612294.4|protein_coding|8/8|c.937_941delAGGTG|p.Arg313fs|1157/1498|937/1083|313/3
pt|ENST00000585608.5|nonsense_mediated_decay|4/7|c.355dupT|p.Ser119fs|485/848|356/396|11
00000267659|transcript|ENST00000585484.1|lincRNA|4/4|n.601-3dupC||||||INFO_REALIGN_3_PRIM
ENST00000269081.8|protein_coding|39/40|c.4515_4516delTC|p.Gln1506fs|5426/6362|4515/4632|1
4263|transcript|ENST00000269081.8|protein_coding|33/40|c.3964C>T|p.Arg1322*|4874/6362|3964
00154263|transcript|ENST00000522406.5|nonsense_mediated_decay|23/40|c.*1609-1A>G||||||,C|
ENST00000269081.8|protein_coding|13/40|c.1331_1334delCTGT|p.Ser444fs|2244/6362|1331/4632|
H|ABCA10|ENSG00000154263|transcript|ENST00000522406.5|nonsense_mediated_decay|12/41|c.*6
0000269081.8|protein_coding|9/40|c.783T>A|p.Tyr261*|1693/6362|783/4632|261/1543||,T|stop_
.54263|transcript|ENST00000521526.2|processed_transcript|4/4|n.548+1G>A||||||,T|upstream_gen
00586462.1|protein_coding|1/2|c.1A>G|p.Met1?|264/1897|1/1374|1/457||,G|intron_variant|MODI
53822|transcript|ENST00000283936.5|protein_coding|2/4|c.-272+1G>A||||||,A|splice_donor_variar
ST00000245479.2|protein_coding|3/3|c.715_718delACCA|p.Thr239fs|1087/3935|715/1530|239/509
00227036|transcript|ENST00000581183.1|lincRNA|1/4|n.66+1G>T||||||,A|upstream_gene_variant|
H|COG1|ENSG00000166685|transcript|ENST00000618996.4|protein_coding|1/15|c.-137+1_-137+24d
H|COG1|ENSG00000166685|transcript|ENST00000618996.4|protein_coding|1/15|c.-137+1_-137+24d

|COG1|ENSG00000166685|transcript|ENST00000618996.4|protein_coding|1/15|c.-137+1_-137+4del
riant&intron_variant|HIGH|COG1|ENSG00000166685|transcript|ENST00000618996.4|protein_coding|
riant&intron_variant|HIGH|COG1|ENSG00000166685|transcript|ENST00000618996.4|protein_coding|
)|transcript|ENST00000255557.8|protein_coding|5/5|c.1640_1643delAGTA|p.Lys547fs|2010/2163|16
G00000266357|transcript|ENST00000584003.1|lincRNA|3/4|n.360+2T>G||||||,C|splice_donor_variar
G00000266357|transcript|ENST00000581028.5|lincRNA|1/3|n.254+1G>A||||||,T|intron_variant|MOD
0000246731|transcript|ENST00000531617.1|antisense|1/1|n.97-1G>A||||||,T|upstream_gene_varia
00141540|transcript|ENST00000529107.5|protein_coding|1/14|c.65delT|p.Leu22fs|96/2015|65/154
ENST00000330793.1|protein_coding|4/4|c.663_667delTGAGA|p.Glu222fs|1028/1517|663/675|221/2
ST00000580632.5|protein_coding|4/5|c.374_375delCT|p.Pro125fs|384/799|374/624|125/207||,C|3
ENST00000584022.5|nonsense_mediated_decay|5/7|c.349dupT|p.Ser117fs|423/1103|349/471|117/1
t&intron_variant|HIGH|FADS6|ENSG00000172782|transcript|ENST00000614223.4|protein_coding|2/
t&intron_variant|HIGH|FADS6|ENSG00000172782|transcript|ENST00000614223.4|protein_coding|2/
0328801.4|protein_coding|1/7|c.2T>C|p.Met1?|2/2363|2/1791|1/596||,C|missense_variant|MODE
teraction|2I7D:A_15-A_77:ENST00000245552|protein_coding|1/5|c.45G>A||||||,T|structural_interac
nteraction|1P4U:A_586-A_657:ENST00000537686|protein_coding|14/17|c.1758G>C||||||,G|synonym
000621870.4|nonsense_mediated_decay|2/18|c.200C>G|p.Ser67*|213/4034|200/210|67/69||,C|sto
pt|ENST00000584516.5|nonsense_mediated_decay|1/33|c.684dupG|p.Pro229fs|685/5709|685/2283
00000584516.5|nonsense_mediated_decay|1/33|c.1218G>A|p.Trp406*|1218/5709|1218/2283|406/
266714|transcript|ENST00000610510.4|protein_coding|1/62|c.2186+1G>A||||||,A|splice_donor_va
00000584516.5|nonsense_mediated_decay|1/33|c.2189T>G|p.Leu730*|2189/5709|2189/2283|730/
|ENST00000584762.3|protein_coding|1/4|c.1_2delTG|p.Cys1fs|1/336|1/336|1/111||WARNING_TRA
0000266714|transcript|ENST00000584762.3|protein_coding|3/4|c.201_204delGGAG|p.Glu68fs|201/3
266714|transcript|ENST00000610510.4|protein_coding|21/62|c.4241+2T>C||||||,C|splice_donor_va
nteraction|3F7Q:A_1220-A_1243:ENST00000200181|protein_coding|30/40|c.3729G>A||||||,A|struct
00334586.9|protein_coding|7/9|c.841C>T|p.Gln281*|924/1335|841/1239|281/412||,T|3_prime_UT
SG00000267128|transcript|ENST00000586627.1|antisense|4/4|n.529-2delA||||||,C|upstream_gene_
SG00000267128|transcript|ENST00000586627.1|antisense|4/4|n.529-1G>C||||||,C|upstream_gene_
nteraction|4V24:A_372-A_407:ENST00000323374|protein_coding|6/6|c.1221T>C||||||,C|structural_
nteraction|3VZB:A_207-A_333:ENST00000592299|protein_coding|6/6|c.997G>A||||||,A|structural_i
nteraction|4V24:A_230-A_449:ENST00000323374|protein_coding|6/6|c.1347C>T||||||,T|structural_i
G00000070526|transcript|ENST00000590915.1|processed_transcript|1/1|n.251-1G>C||||||,G|upstre
4640|transcript|ENST00000590825.1|protein_coding|1/1|c.-417+1G>A||||||WARNING_TRANSCRIPT_
0000267665|transcript|ENST00000592572.1|lincRNA|2/3|n.3854+1G>C||||||,C|splice_donor_varian
267679|transcript|ENST00000585668.1|processed_pseudogene|1/2|n.93+1T>C||||||,C|upstream_ge
raction|1W4R:A_52-A_74:ENST00000301634|protein_coding|4/7|c.222G>A||||||,T|synonymous_va
T00000587310.5|nonsense_mediated_decay|4/6|c.118C>T|p.Arg40*|229/768|118/126|40/41||,A|st
0171302|transcript|ENST00000591773.5|protein_coding|1/5|c.-285-1G>A||||||,T|splice_acceptor_va
ENST00000374877.7|protein_coding|18/18|c.2852_2915delAGGACGCACACAGGCACGTGCACGAACAAC
ENST00000374877.7|protein_coding|18/18|c.3044_3045delAC|p.Asp1015fs|3075/3544|3044/3093|
t|ENST00000374877.7|protein_coding|18/18|c.3082dupG|p.Ala1028fs|3114/3544|3083/3093|1028/
00374877.7|protein_coding|18/18|c.3091T>C|p.Ter1031Argext*?|3122/3544|3091/3093|1031/1030
interaction|2HYI:C_111-C_160:ENST00000269349|protein_coding|5/12|c.480C>T||||||,A|structural_
0000575500.5|nonsense_mediated_decay|9/22|c.1102C>T|p.Arg368*|1293/3766|1102/1482|368/4
0000575500.5|nonsense_mediated_decay|10/22|c.1255C>T|p.Arg419*|1446/3766|1255/1482|419/
teraction|4MHX:A_387-A_437:ENST00000326317|protein_coding|8/8|c.1159G>A||||||,T|structural_
interaction|4NSP:A_29-A_214:ENST00000518137|protein_coding|2/10|c.85G>A||||||,A|missense_va

)0519117.5|nonsense_mediated_decay|3/6|c.293A>G|p.Ter98Trpext*?|310/617|293/294|98/97||,G
)000262662&ENSG00000141564|gene_variant|ENSG00000141564|||n.80960745_80960831delCTGAG
i00000261924|transcript|ENST00000573602.1|antisense|1/1|n.1365+1G>A||||||,T|downstream_ger
)00260005|transcript|ENST00000569559.1|antisense|3/3|n.466+1G>T||||||,T|downstream_gene_va
:NST00000570854.1|nonsense_mediated_decay|1/5|c.208delC|p.Arg70fs|232/849|208/552|70/183|
7|transcript|ENST00000288439.9|protein_coding|14/14|c.2284_*1delCACCTGCGGCATCGTCACATGGC
ISG00000262877|transcript|ENST00000571724.3|lincRNA|1/1|n.368-2A>G||||||,C|upstream_gene_v
)184009|transcript|ENST00000615544.4|protein_coding|6/6|c.*211-1G>A||||||,T|splice_acceptor_v
)00417245.6|protein_coding|1/5|c.130C>T|p.Gln44*|266/1665|130/1479|44/492||,T|stop_gained|
)00182612|transcript|ENST00000621293.4|nonsense_mediated_decay|2/3|c.675-2A>G||||||,G|dow
)182612|transcript|ENST00000574882.5|protein_coding|4/4|c.796_799dupAACT|p.Cys267fs|1001/1
:NST00000573076.5|protein_coding|2/4|c.138delC|p.Val47fs|614/1505|138/414|46/137||,C|5_prim
)00571916.1|nonsense_mediated_decay|2/5|c.184T>C|p.Ter62Argext*?|184/708|184/186|62/61||V
teraction|3UEM:A_238-A_288:ENST00000331483|protein_coding|5/11|c.714C>T||||||,A|structural_
)197063|transcript|ENST00000574686.1|protein_coding|1/3|c.-93-1G>C||||||WARNING_TRANSCRIP
|interaction|4UYU:B_338-B_384:ENST00000409678|protein_coding|10/11|c.1152C>T||||||,A|structu
action|4DRB:A_40-J_62:ENST00000309048-ENST00000392359|protein_coding|4/5|c.185C>T||||||,A|
action|4DRA:C_33-G_8:ENST00000309048-ENST00000392359|protein_coding|1/5|c.24C>A||||||,T|p
teraction|2C2H:A_22-A_159:ENST00000306897|protein_coding|6/6|c.477C>T||||||,T|structural_inte
teraction|1WNT:A_82-A_115:ENST00000306869|protein_coding|4/8|c.345G>A||||||,T|structural_in
)00169738|transcript|ENST00000579155.1|protein_coding|1/5|c.67_68dupAA|p.Cys24fs|75/430|68,
teraction|4W82:A_1652-A_1824:ENST00000306749|protein_coding|32/43|c.5470C>T||||||,A|structu
teraction|4W82:A_1674-A_1756:ENST00000306749|protein_coding|31/43|c.5268C>T||||||,A|structu
teraction|4PIV:A_1247-A_1273:ENST00000306749|protein_coding|23/43|c.3741T>C||||||,G|structu
teraction|3HHD:A_189-A_225:ENST00000306749|protein_coding|5/43|c.567T>C||||||,G|structural_
)0000392346.6|protein_coding|5/5|c.829C>T|p.Arg277*|1376/2964|829/1059|277/352||,A|stop_gai
on_coding_transcript_exon_variant|HIGH|RP11-1376P16.1|ENSG00000266654|transcript|ENST00000
|interaction|3UZP:A_107-A_286:ENST00000314028|protein_coding|6/9|c.858C>T||||||,A|structural_
141562|transcript|ENST00000580296.5|protein_coding|1/5|c.-324-1G>T||||||WARNING_TRANSCRIP
)0141556|transcript|ENST00000355528.8|protein_coding|7/38|c.772-3dupT||||||INFO_REALIGN_3_
)0141556|transcript|ENST00000355528.8|protein_coding|7/38|c.772-3dupT||||||INFO_REALIGN_3_
)0141556|transcript|ENST00000355528.8|protein_coding|7/38|c.772-3dupT||||||INFO_REALIGN_3_
on_coding_transcript_exon_variant|HIGH|FAM166AP6|ENSG00000262181|transcript|ENST0000575
)000173213|transcript|ENST00000594555.1|processed_transcript|1/1|n.131-2_131-1delAG||||||,C|
_coding_transcript_exon_variant|HIGH|ROCK1P1|ENSG00000263006|transcript|ENST00000608049.5|
NSG00000132199|transcript|ENST00000581475.5|nonsense_mediated_decay|6/14|c.445_466dupGG
)0000266450|transcript|ENST00000580524.1|lincRNA|2/2|n.408+1G>A|||||
|interaction|2R15:A_1592-A_1631:ENST00000356443|protein_coding|38/38|c.4776C>G||||||,C|stru
|interaction|2R15:A_1470-A_1554:ENST00000356443|protein_coding|35/38|c.4662C>T||||||,A|stru
|interaction|3RBS:A_1382-A_1408:ENST00000356443|protein_coding|30/38|c.4222G>A||||||,T|miss
118680|transcript|ENST00000581193.5|protein_coding|1/3|c.-16+2C>T||||||,T|upstream_gene_vari
7426|transcript|ENST00000549546.5|protein_coding|1/2|c.-45+2C>T||||||WARNING_TRANSCRIPT_IN
NST00000330513.9|protein_coding|1/3|c.246delT|p.Pro83fs|549/1980|246/1206|82/401||,C|upstrea
NST00000551402.1|protein_coding|2/2|c.346_347delCA|p.Gln116fs|659/1089|346/399|116/132||IN
)0000264000|transcript|ENST00000578391.1|antisense|2/2|n.306+2T>C||||||,C|intron_variant|MOI
73482|transcript|ENST00000578600.1|processed_transcript|1/1|n.304+1G>A||||||,A|intron_variant
)168502|transcript|ENST00000518815.1|protein_coding|4/9|c.550-1G>C||||||,C|splice_acceptor_va

t|ENST00000400033.1|protein_coding|1/9|c.10dupC|p.His4fs|128/942|11/759|4/252||INFO_REALIG
)000266053|transcript|ENST00000582375.1|antisense|3/3|n.578+1delG|||||,A|splice_donor_variar
)168461&ENSG00000252680|gene_variant|ENSG00000252680|||n.9844852_9844833delAAAACAAAA(
)168461&ENSG00000252680|gene_variant|ENSG00000252680|||n.9844852_9844833delAAAACAAAA(
)168461&ENSG00000252680|gene_variant|ENSG00000252680|||n.9844852_9844833delAAAACAAAA(
ENST00000611534.1|protein_coding|3/4|c.947+6_952delCAGAAGAAGCCATCCAGCCCAAGGAGGGTGA(
0154889|transcript|ENST00000592306.5|nonsense_mediated_decay|2/3|c.208-1G>T|||||,A|upstre
0154889|transcript|ENST00000590501.5|protein_coding|2/5|c.-321-1G>A|||||WARNING_TRANSCRI
interaction|2CZH:B_148-B_241:ENST00000269159|protein_coding|5/8|c.443G>A|||||,A|structural_i
:12.3|ENSG00000267733|transcript|ENST00000592801.5|processed_transcript|2/2|n.194-3dupT||||
:12.3|ENSG00000267733|transcript|ENST00000592801.5|processed_transcript|2/2|n.194-3dupT||||
:12.3|ENSG00000267733|transcript|ENST00000592801.5|processed_transcript|2/2|n.194-3dupT||||
:12.3|ENSG00000267733|transcript|ENST00000592801.5|processed_transcript|2/2|n.194-3dupT||||
:12.3|ENSG00000267733|transcript|ENST00000592801.5|processed_transcript|2/2|n.194-3dupT||||
:12.3|ENSG00000267733|transcript|ENST00000592801.5|processed_transcript|2/2|n.194-3dupT||||
)0000267733|transcript|ENST00000588436.1|transcribed_processed_pseudogene|1/3|n.191+1A>G||
)176194|transcript|ENST00000522713.5|nonsense_mediated_decay|1/6|c.185_200dupGCACACACCC/
6194|transcript|ENST00000521296.5|processed_transcript|1/4|n.255+1G>A|||||,A|splice_donor_va
1|ENSG00000267108|transcript|ENST00000589843.1|lincRNA|1/1|n.105-3dupT|||||INFO_REALIGN
1|ENSG00000267108|transcript|ENST00000589843.1|lincRNA|1/1|n.105-3dupT|||||INFO_REALIGN
1|ENSG00000267108|transcript|ENST00000589843.1|lincRNA|1/1|n.105-3dupT|||||INFO_REALIGN
)0586445.5|protein_coding|1/6|c.3G>A|p.Met1?|22/646|3/627|1/208||WARNING_TRANSCRIPT_NO_
)168675|transcript|ENST00000593236.1|protein_coding|3/3|c.-51+1G>T|||||WARNING_TRANSCRIPT
)0000267356&ENSG00000175322|gene_variant|ENSG00000267356|||n.14105167_14105250delTTCTC
G00000186481|transcript|ENST00000431648.8|transcribed_unprocessed_pseudogene|3/21|n.493-1G
interaction|2ETK:A_269-A_298:ENST00000399799|protein_coding|7/33|c.807C>T|||||,A|structural_
134508|transcript|ENST00000400473.6|protein_coding|1/9|c.-137+1G>A|||||,A|upstream_gene_va
nt&intron_variant|HIGH|CABYR|ENSG00000154040|transcript|ENST00000577705.1|protein_coding|4
teraction|3GD8:A_41-A_122:ENST00000383168|protein_coding|2/5|c.366G>A|||||,T|synonymous_
)00399380.7|protein_coding|1/15|c.62T>A|p.Leu21*|99/3737|62/2628|21/875||,T|intron_variant|M
ENST00000280904.10|protein_coding|16/16|c.2686_2687dupGA|p.Ala897fs|3131/12325|2687/2706
0264859|transcript|ENST00000579251.1|antisense|1/3|n.349+2T>G|||||,C|non_coding_transcript_
interaction|4GWM:A_50-A_162:ENST00000269202|protein_coding|4/15|c.150G>A|||||,A|structura
interaction|4GWM:A_76-A_116:ENST00000269202|protein_coding|6/15|c.348A>G|||||,G|structura
interaction|4GWM:A_264-A_421:ENST00000269202|protein_coding|9/15|c.792G>A|||||,A|structur
)0000261715|transcript|ENST00000566101.1|lincRNA|2/3|n.884-2A>T|||||
57313|transcript|ENST00000599934.1|lincRNA|1/3|n.126-1G>A|||||,T|intron_variant|MODIFIER|KC
interaction|3IHY:D_328-D_359:ENST00000262039|protein_coding|10/25|c.1077T>C|||||,C|structura
ript|ENST00000426838.8|protein_coding|4/4|c.681_682insTCTT|p.Thr228fs|1077/1785|682/729|228
.52234|transcript|ENST00000590324.5|protein_coding|3/5|c.-12+2T>G|||||WARNING_TRANSCRIPT_
)00536111.1|nonsense_mediated_decay|1/10|c.1A>G|p.Met1?|192/1358|1/921|1/306||,C|intron_va
)00536736.5|protein_coding|1/40|c.2T>A|p.Met1?|2/6848|2/6636|1/2211||,T|start_lost|HIGH|LOXH
000592005.5|protein_coding|3/7|c.2T>C|p.Met1?|675/1348|2/318|1/105||,C|start_lost|HIGH|KATN
ript|ENST00000620881.1|protein_coding|1/1|c.1272_1273dupGC|p.Gln425fs|1273/1641|1273/1641
ipt|ENST00000620688.2|protein_coding|1/1|c.849_850dupGC|p.Gln284fs|850/1218|850/1218|284/4
ɔt|ENST00000591522.1|protein_coding|4/4|c.280dupA|p.Ser94fs|281/994|281/465|94/154||WARNI
ɔt|ENST00000591522.1|protein_coding|4/4|c.280dupA|p.Ser94fs|281/994|281/465|94/154||WARNI

G00000267761|transcript|ENST00000600127.1|lincRNA|1/3|n.351+1G>C||||||,C|downstream_gene_
.41627|transcript|ENST00000578396.1|protein_coding|5/5|c.299-2A>C||||||,G|intron_variant|MOD
it&intron_variant|HIGH|MEX3C|ENSG00000176624|transcript|ENST00000616921.1|protein_coding|2
0000227115&ENSG00000265402|gene_variant|ENSG00000265402|||n.51613119_51613109delAAAAA
0000227115&ENSG00000265402|gene_variant|ENSG00000265402|||n.51613119_51613109delAAAAA
0000227115&ENSG00000265402|gene_variant|ENSG00000265402|||n.51613119_51613109delAAAAA
0000227115&ENSG00000265402|gene_variant|ENSG00000265402|||n.51613119_51613109delAAAAA
0000227115&ENSG00000265402|gene_variant|ENSG00000265402|||n.51613119_51613109delAAAAA
0000227115&ENSG00000265402|gene_variant|ENSG00000265402|||n.51613119_51613109delAAAAA
0000266335|transcript|ENST00000582700.1|lincRNA|1/1|n.578+1G>A||||||,T|upstream_gene_variar
00000307844.3|protein_coding|1/6|c.55C>T|p.Arg19*|55/663|55/663|19/220||,A|stop_gained|HIGH
01157|transcript|ENST00000587403.5|processed_transcript|1/2|n.220+2T>G||||||,G|intron_variant|
ENSG00000267040|transcript|ENST00000592201.1|antisense|1/2|n.170-3dupT||||||INFO_REALIGN_3
ENSG00000267040|transcript|ENST00000592201.1|antisense|1/2|n.170-3dupT||||||INFO_REALIGN_3
ENSG00000267040|transcript|ENST00000592201.1|antisense|1/2|n.170-3dupT||||||INFO_REALIGN_3
0000172175|transcript|ENST00000348428.7|protein_coding|2/16|c.377-3dupT||||||INFO_REALIGN_
0000172175|transcript|ENST00000348428.7|protein_coding|2/16|c.377-3dupT||||||INFO_REALIGN_
0000172175|transcript|ENST00000348428.7|protein_coding|2/16|c.377-3dupT||||||INFO_REALIGN_
0000172175|transcript|ENST00000348428.7|protein_coding|2/16|c.377-3dupT||||||INFO_REALIGN_
00224367|transcript|ENST00000417918.1|transcribed_unitary_pseudogene|10/17|n.1312-1A>G|||||
G00000267215|transcript|ENST00000589729.1|lincRNA|1/2|n.25+2T>C||||||,C|intron_variant|MODI
ST00000256857.6|protein_coding|3/3|c.422_425delGGAA|p.Arg141fs|520/848|422/447|141/148||IN
interaction|4GKX:A_117-A_195:ENST00000251047|protein_coding|2/13|c.351A>G||||||,C|structural
NSG00000267669|transcript|ENST00000592549.1|transcribed_processed_pseudogene|1/1|n.374-2C>
00000267098|transcript|ENST00000591869.1|lincRNA|3/7|n.128+1G>A|||||
t|ENST00000587725.5|non_stop_decay|22/22|c.3008delT|p.Leu1003fs|3223/3268|3008/3053|1003,
t|ENST00000587725.5|non_stop_decay|22/22|c.3008delT|p.Leu1003fs|3223/3268|3008/3053|1003,
75|interaction|1WZ9:A_161-A_319:ENST00000382771|protein_coding|7/7|c.955A>G||||||,G|structu
ST00000382749.9|protein_coding|5/9|c.268G>T|p.Glu90*|513/1918|268/1179|90/392||,T|stop_gair
ST00000536691.5|nonsense_mediated_decay|5/7|c.388C>T|p.Gln130*|450/1294|388/408|130/135|
32|interaction|1JRR:A_292-A_404:ENST00000299502|protein_coding|8/8|c.1212C>G||||||,G|structu
32|interaction|1BY7:A_25-A_413:ENST00000299502|protein_coding|8/8|c.1238C>G||||||,G|structura
RP11-389J22.1|ENSG00000263720|transcript|ENST00000581987.1|sense_intronic|1/1|n.757_757+1i
RP11-389J22.1|ENSG00000263720|transcript|ENST00000581987.1|sense_intronic|1/1|n.757_757+1i
RP11-389J22.1|ENSG00000263720|transcript|ENST00000581987.1|sense_intronic|1/1|n.757_757+1i
00000263720|transcript|ENST00000581987.1|sense_intronic|1/1|n.757+2C>A||||||,A|downstream_
NST00000443099.6|protein_coding|8/8|c.572_573delTT|p.Phe191fs|649/913|572/594|191/197||,T|u
ST00000360242.9|protein_coding|4/8|c.890G>A|p.Trp297*|1007/2711|890/1542|297/513||,A|stop_
NSG00000279221|gene_variant|ENSG00000206052|||n.69659823_69659852delTATATATATATATAACA
33313|transcript|ENST00000581272.5|protein_coding|1/4|c.-38+2C>T||||||WARNING_TRANSCRIPT_
0577355.2|protein_coding|4/4|c.375A>T|p.Ter125Cysext*?|375/536|375/375|125/124||WARNING_
00133313|transcript|ENST00000324262.8|protein_coding|10/11|c.1211-3dupC||||||INFO_REALIGN_
nteraction|2WEK:A_195-A_327:ENST00000322342|protein_coding|2/2|c.585G>A||||||,T|structural_
interaction|2WEK:A_75-A_99:ENST00000322342|protein_coding|2/2|c.225C>T||||||,A|structural_int
00000261194|transcript|ENST00000564156.1|lincRNA|1/1|n.370+1G>A|||||
000263547|transcript|ENST00000584677.1|lincRNA|3/5|n.2066+2C>T||||||,T|downstream_gene_var
397860.7|protein_coding|4/4|c.592T>C|p.Ter198Glnext*?|807/4844|592/594|198/197||,G|stop_lost

97971|transcript|ENST00000497479.1|processed_transcript|3/5|n.469-2A>T||||||,A|downstream_g
000586366.5|nonsense_mediated_decay|8/10|c.835C>T|p.Gln279*|854/2845|835/870|279/289||,T
:|ENST00000318050.4|protein_coding|1/1|c.75dupT|p.Val26fs|112/1054|76/918|26/305||INFO_REA
)5556|transcript|ENST00000592722.1|processed_transcript|4/4|n.437+1G>A||||||,T|downstream_ge
:|ENST00000315489.4|protein_coding|4/4|c.573dupC|p.Ala192fs|809/1604|573/870|191/289||,CGG
00099866|transcript|ENST00000587541.5|protein_coding|1/2|c.10+1G>A||||||,A|downstream_gene
00099866|transcript|ENST00000215637.7|protein_coding|4/5|c.699_700insAACACCACCTCCCC|p.Asp
)9866|transcript|ENST00000215637.7|protein_coding|4/5|c.700_701insGAGCCTCCCA|p.Asp234fs|747
)9866|transcript|ENST00000215637.7|protein_coding|4/5|c.700_701insGAGCCTCCCA|p.Asp234fs|747
)9866|transcript|ENST00000215637.7|protein_coding|4/5|c.700_701insGAGCCTCCCA|p.Asp234fs|747
)000264553.5|protein_coding|4/5|c.598C>T|p.Gln200*|635/940|598/774|200/257||,T|stop_gained|
nteraction|3U10:A_568-A_624:ENST00000251287|protein_coding|7/8|c.1872T>C||||||,C|structural_i
.|interaction|4BOC:A_876-A_906:ENST00000588649|protein_coding|10/21|c.2628C>T||||||,A|synon
9864|transcript|ENST00000587513.3|processed_transcript|1/3|n.56+1G>A||||||,A|downstream_ger
nteraction|3ZZY:A_182-A_228:ENST00000356948|protein_coding|6/15|c.546C>T||||||,T|structural_i
ENST00000617672.1|protein_coding|2/2|c.182_183delCT|p.Pro61fs|200/554|182/408|61/135||,C|se
221|transcript|ENST00000617672.1|protein_coding|2/2|c.158_168dupTCCTCAGCTAC|p.Pro57fs|185/
ENST00000234389.3|protein_coding|3/9|c.1090_1091delAT|p.Met364fs|1109/3281|1090/3132|364/
cript|ENST00000234389.3|protein_coding|3/9|c.1396_1397insCGTT|p.Gly466fs|1416/3281|1397/313
)0064687|transcript|ENST00000524850.5|protein_coding|1/5|c.-7-2A>G||||||WARNING_TRANSCRIPT
NST00000433129.5|protein_coding|2/46|c.146_152delTGGAGCA|p.Leu49fs|262/6704|146/6441|49/
G00000180448|transcript|ENST00000587186.5|protein_coding|1/3|c.92_105delACGGACAGGGCCCG|p
)0382477.6|protein_coding|2/10|c.2T>C|p.Met1?|277/2453|2/2178|1/725||,G|missense_variant|MC
9622|transcript|ENST00000588090.5|protein_coding|1/6|c.-7+1G>A||||||,A|splice_donor_variant&ir
nteraction|3ORH:B_209-B_229:ENST00000252288|protein_coding|6/6|c.626C>T||||||,A|structural_ir
:|ENST00000588871.5|protein_coding|4/4|c.364delC|p.Gln122fs|398/2128|364/483|122/160||,T|3_
5257|transcript|ENST00000591687.2|processed_transcript|1/4|n.24+2T>G||||||,C|TF_binding_site_
988|transcript|ENST00000334770.8|protein_coding|12/12|c.825+2T>C||||||,C|splice_donor_variant
)0310127.10|protein_coding|29/29|c.3903A>G|p.Ter1301Trpext*?|4142/5095|3903/3903|1301/130
:|ENST00000526092.6|protein_coding|14/14|c.1575dupT|p.Glu526fs|1755/2759|1575/1689|525/562
5|interaction|2C47:A_247-A_279:ENST00000255641|protein_coding|8/12|c.837C>T||||||,T|structura
000267283|transcript|ENST00000587498.1|antisense|1/1|n.467+1G>A||||||,A|upstream_gene_varia
ENST00000587825.1|protein_coding|3/7|c.378dupA|p.Pro127fs|379/961|378/960|126/319||WARN
)300961.10|protein_coding|2/7|c.3G>C|p.Met1?|68/1138|3/996|1/331||,G|missense_variant|MODE
0000005206|transcript|ENST00000614794.4|protein_coding|1/8|c.-141-5_-141-3dupTTT||||||INFO_
0000005206|transcript|ENST00000614794.4|protein_coding|1/8|c.-141-5_-141-3dupTTT||||||INFO_
0000005206|transcript|ENST00000614794.4|protein_coding|1/8|c.-141-5_-141-3dupTTT||||||INFO_
0000005206|transcript|ENST00000614794.4|protein_coding|1/8|c.-141-5_-141-3dupTTT||||||INFO_
0000005206|transcript|ENST00000614794.4|protein_coding|1/8|c.-141-5_-141-3dupTTT||||||INFO_
'1970|transcript|ENST00000614108.1|protein_coding|3/6|c.151+2T>A||||||,A|intron_variant|MODIF
000314531.4|protein_coding|3/4|c.298C>T|p.Gln100*|391/2002|298/1638|100/545||,A|downstrear
717|transcript|ENST00000591529.5|protein_coding|1/19|c.-12+1G>A||||||,T|upstream_gene_varian
586578.1|nonsense_mediated_decay|2/3|c.131A>G|p.Ter44Trpext*?|132/1019|131/132|44/43||WA
000595928.1|nonsense_mediated_decay|6/8|c.487T>C|p.Ter163Glnext*?|489/736|487/489|163/16
000594605.5|protein_coding|6/13|c.507C>G|p.Tyr169*|631/1426|507/1212|169/403||,C|stop_gain
00008382|transcript|ENST00000596722.5|protein_coding|1/11|c.43_58dupGGGCGCGGGGCTGCAG|p
00008382|transcript|ENST00000596722.5|protein_coding|1/11|c.43_58dupGGGCGCGGGGCTGCAG|p

```
G00000267011|transcript|ENST00000589066.1|lincRNA|1/1|n.247-1G>A||||||,T|upstream_gene_va
NST00000633942.1|protein_coding|5/8|c.2598_2599delTG|p.Gly867fs|2697/6484|2598/4119|866/13
|ENST00000633942.1|protein_coding|5/8|c.2595_2596insGT|p.Leu866fs|2693/6484|2595/4119|865
0000599630.1|protein_coding|5/5|c.461G>A|p.Trp154*|489/713|461/570|154/189||,T|upstream_ge
002|transcript|ENST00000600497.5|processed_transcript|3/4|n.272+1G>A||||||,T|splice_donor_va
interaction|3DB3:A_131-A_159:ENST00000612630|protein_coding|3/17|c.393A>G||||||,G|structural
interaction|2PB7:A_469-A_577:ENST00000612630|protein_coding|10/17|c.1407T>C||||||,C|structur
nteraction|2PB7:A_427-A_555:ENST00000612630|protein_coding|12/17|c.1665C>T||||||,T|structur
nteraction|2FH7:A_1480-A_1545:ENST00000587303|protein_coding|30/37|c.4635C>T||||||,A|struct
nteraction|2YD9:A_253-A_283:ENST00000588012|protein_coding|6/32|c.759C>T||||||,A|structural_
nteraction|2YD2:A_56-A_87:ENST00000588012|protein_coding|3/32|c.261A>C||||||,G|structural_in
nteraction|2X36:A_874-A_946:ENST00000360614|protein_coding|18/18|c.2836G>A||||||,T|missens
nteraction|2X36:A_878-A_911:ENST00000360614|protein_coding|18/18|c.2731G>A||||||,T|structur
597721.1|protein_coding|1/1|c.493T>C|p.Ter165Argext*?|3702/3911|493/495|165/164||,G|synony
000205744|transcript|ENST00000588988.5|retained_intron|4/4|n.437-1G>A||||||,T|downstream_g
G00000268742|transcript|ENST00000594355.1|processed_pseudogene|1/2|n.245-2delA||||||
G00000268742|transcript|ENST00000594355.1|processed_pseudogene|1/2|n.245-3dupA||||||
000269546|transcript|ENST00000601934.1|processed_pseudogene|2/2|n.603-2dupA||||||
nteraction|3BJI:B_239-B_279:ENST00000602142|protein_coding|9/27|c.835G>A||||||,A|missense_va
174837|transcript|ENST00000450315.7|protein_coding|5/17|c.418+2C>G||||||,G|missense_variant|
0000174837|transcript|ENST00000312053.8|protein_coding|13/20|c.1621-3dupT||||||INFO_REALIG
0000174837|transcript|ENST00000312053.8|protein_coding|13/20|c.1621-3dupT||||||INFO_REALIG
t|ENST00000446248.4|protein_coding|13/19|c.1662_1665delGAAA|p.Lys555fs|1763/4179|1662/383
ENST00000612033.1|protein_coding|2/7|c.37_46delCATGTGTGTG|p.His13fs|37/1188|37/1188|13/39
ENST00000612033.1|protein_coding|3/7|c.82_83delGT|p.Val28fs|82/1188|82/1188|28/395||WARNI
ENST00000612033.1|protein_coding|4/7|c.220_221delTG|p.Cys74fs|220/1188|220/1188|74/395||W
ENST00000612033.1|protein_coding|4/7|c.259_262delCTGT|p.Leu87fs|259/1188|259/1188|87/395|
ENST00000612033.1|protein_coding|4/7|c.289_292delATGT|p.Met97fs|289/1188|289/1188|97/395
ENST00000612033.1|protein_coding|4/7|c.311_312delGT|p.Arg104fs|311/1188|311/1188|104/395|
00000076944|transcript|ENST00000612033.1|protein_coding|5/7|c.432_432+1dupTG|p.His145fs|43
000076944|transcript|ENST00000612033.1|protein_coding|6/7|c.496_497delTG|p.Cys166fs|496/118
ENST00000612033.1|protein_coding|6/7|c.575_588delATCTGTGTGTGTGT|p.His192fs|575/1188|575/
ENST00000612033.1|protein_coding|6/7|c.598_605delCATGCGTG|p.His200fs|598/1188|598/1188|20
interaction|4CCA:A_266-A_345:ENST00000221283|protein_coding|13/19|c.1034C>T||||||,T|structu
interaction|4CCA:A_225-A_529:ENST00000221283|protein_coding|18/19|c.1586G>C||||||,C|structu
nteraction|2H2R:A_174-A_284:ENST00000346664|protein_coding|11/11|c.851G>A||||||,T|structural
nteraction|4GJ0:B_202-B_239:ENST00000346664|protein_coding|10/11|c.717C>T||||||,A|structural_
|interaction|2DYL:A_166-A_207:ENST00000397983|protein_coding|7/12|c.621C>T||||||,T|synonym
0000104976|transcript|ENST00000221573.10|protein_coding|5/5|c.1003_*12delTGAGGGGCATGGCG
0186994|transcript|ENST00000610351.1|protein_coding|1/4|c.2_13delTGGCGACAGCGA|p.Met1_Ala4
00423345.4|protein_coding|1/10|c.1A>G|p.Met1?|522/2667|1/2013|1/670||,C|start_lost|HIGH|PRA
ENST00000397910.8|protein_coding|44/84|c.39485_39486delCC|p.Thr13162fs|39690/43816|39485/
00181143|transcript|ENST00000397910.8|protein_coding|18/83|c.37139-1G>T||||||
00181143|transcript|ENST00000397910.8|protein_coding|18/83|c.37139-2A>C||||||
script|ENST00000305444.2|protein_coding|1/1|c.924_928dupACCAT|p.Ser310fs|928/939|928/939|3
ENST00000305444.2|protein_coding|1/1|c.710delC|p.Ala237fs|710/939|710/939|237/312||,A|upstre
ENST00000592504.5|protein_coding|6/6|c.412_413delCT|p.Leu138fs|667/1398|412/528|138/175||I
```

ENST00000588933.5|protein_coding|11/11|c.1329delA|p.Ala444fs|2295/3293|1329/1650|443/549||
|ENST00000453792.6|protein_coding|6/6|c.734_738delGAGAG|p.Gly245fs|1384/2011|734/1365|245
00587782.1|protein_coding|2/3|c.50G>A|p.Trp17*|51/702|50/237|17/78||WARNING_TRANSCRIPT_
nt&intron_variant|HIGH|EIF3G|ENSG00000130811|transcript|ENST00000593066.5|nonsense_mediate
|interaction|3H2U:B_134-B_178:ENST00000615032|protein_coding|3/10|c.534T>C||||||,G|structura
00591969.1|nonsense_mediated_decay|3/4|c.286T>C|p.Ter96Argext*?|288/742|286/288|96/95||W
teraction|3LXN:A_928-A_978:ENST00000264818|protein_coding|18/23|c.2783C>T||||||,A|structural
teraction|4PO6:A_53-A_89:ENST00000264818|protein_coding|1/23|c.157G>A||||||,T|structural_inte
nteraction|2QYK:A_375-A_547:ENST00000440014|protein_coding|14/15|c.1639C>T||||||,T|missens
ENST00000587069.5|protein_coding|4/4|c.339_340delCT|p.Cys114fs|340/544|339/387|113/128||W
pt|ENST00000316737.5|protein_coding|7/7|c.696dupG|p.Ser233fs|847/1040|697/738|233/245||INF
interaction|2GF9:A_25-A_74:ENST00000222120|protein_coding|2/5|c.75G>C||||||,G|structural_inte
pt|ENST00000590636.1|nonsense_mediated_decay|2/6|c.46dupA|p.Ser16fs|82/561|47/117|16/38||
00592312.5|protein_coding|4/6|c.488T>G|p.Leu163*|803/1286|488/804|163/267||,C|3_prime_UTR
0159|transcript|ENST00000592571.1|nonsense_mediated_decay|2/5|c.155+1G>A||||||WARNING_TR
575|transcript|ENST00000589792.5|protein_coding|1/2|c.-88+2T>A||||||WARNING_TRANSCRIPT_IN
ENST00000548669.1|nonsense_mediated_decay|2/6|c.47delA|p.Lys16fs|204/709|47/126|16/41||,C
G00000219665|transcript|ENST00000476474.5|antisense|3/3|n.414-2A>G||||||,G|splice_acceptor_
ENST00000439326.7|protein_coding|4/4|c.1044_1048delTCTTA|p.Tyr348fs|1219/6620|1044/2001|3
14189|transcript|ENST00000397759.3|protein_coding|2/2|c.581+2T>C||||||WARNING_TRANSCRIPT_
00301547.9|protein_coding|4/4|c.2015A>T|p.Ter672Leuext*?|2213/2663|2015/2016|672/671||,A|d
pt|ENST00000301547.9|protein_coding|4/4|c.1444_1445insCA|p.Gly482fs|1642/2663|1444/2016|48
ENST00000301547.9|protein_coding|4/4|c.1442_1443delTT|p.Leu481fs|1641/2663|1442/2016|481/
ENST00000301547.9|protein_coding|4/4|c.1442_1443delTT|p.Leu481fs|1641/2663|1442/2016|481/
00000234003|transcript|ENST00000416987.1|unprocessed_pseudogene|1/3|n.14-1A>C||||||,C|ups
000234003|transcript|ENST00000416987.1|unprocessed_pseudogene|2/3|n.95+2A>G||||||,G|upstr
00095066|transcript|ENST00000589765.1|processed_transcript|1/3|n.33-2A>C||||||,G|3_prime_UTR
NST00000587230.5|protein_coding|8/8|c.736delC|p.Leu246fs|801/1136|736/750|246/249||,A|fram
teraction|4LDQ:A_70-A_118:ENST00000040663|protein_coding|2/6|c.352G>A||||||,A|structural_int
00267633|transcript|ENST00000591826.1|antisense|1/1|n.302+2C>T||||||,A|3_prime_UTR_variant
ript|ENST00000624302.1|protein_coding|4/7|c.151_158delAAAAAAAA|p.Lys51fs|158/384|151/384|5
cript|ENST00000624302.1|protein_coding|4/7|c.151_157delAAAAAAA|p.Lys51fs|157/384|151/384|5
nscript|ENST00000624302.1|protein_coding|4/7|c.151_155delAAAAA|p.Lys52fs|155/384|151/384|5
anscript|ENST00000624302.1|protein_coding|4/7|c.151_154delAAAA|p.Lys52fs|154/384|151/384|5
|transcript|ENST00000624302.1|protein_coding|4/7|c.151_152delAA|p.Lys53fs|152/384|151/384|51
00000280221|transcript|ENST00000624302.1|protein_coding|4/7|c.151_156delAAAAAA|p.Lys51_Lys
132000|transcript|ENST00000588872.1|protein_coding|2/2|c.563+1G>T||||||WARNING_TRANSCRIPT
pt|ENST00000589048.1|protein_coding|5/5|c.481_482insGC|p.Ala161fs|757/855|481/579|161/192||
cript|ENST00000533683.6|protein_coding|1/5|c.330_331insACAG|p.Pro111fs|618/2164|330/1299|11
pt|ENST00000533683.6|protein_coding|1/5|c.327_328insCC|p.Ala110fs|615/2164|327/1299|109/43
cript|ENST00000533683.6|protein_coding|1/5|c.321_322insGCTG|p.Pro108fs|609/2164|321/1299|10
H|SAMD1|ENSG00000141858|transcript|ENST00000533683.6|protein_coding|1/5|c.312_313insCGCG
teraction|4OTD:A_619-A_641:ENST00000342216|protein_coding|15/22|c.1922G>A||||||,A|structura
teraction|4OTD:A_738-A_800:ENST00000342216|protein_coding|19/22|c.2400A>G||||||,G|structura
00123143|transcript|ENST00000592960.1|protein_coding|1/2|c.70-3dupT||||||WARNING_TRANSCR
00123143|transcript|ENST00000592960.1|protein_coding|1/2|c.70-3dupT||||||WARNING_TRANSCR
00123143|transcript|ENST00000592960.1|protein_coding|1/2|c.70-3dupT||||||WARNING_TRANSCR

797|transcript|ENST00000598298.5|protein_coding|3/10|c.-451+2T>A||||||WARNING_TRANSCRIPT_
0000601345.5|protein_coding|7/20|c.731G>A|p.Trp244*|817/3128|731/2496|244/831||,T|stop_ga
5135|transcript|ENST00000600984.5|protein_coding|1/5|c.-187+2T>C||||||WARNING_TRANSCRIPT_
000601792.1|protein_coding|2/4|c.2T>C|p.Met1?|37/578|2/543|1/180||WARNING_TRANSCRIPT_NC
186204|transcript|ENST00000518629.7|retained_intron|3/10|n.322+1G>A||||||,A|splice_donor_var
nteraction|3QBT:A_9-A_58:ENST00000300935|protein_coding|1/8|c.27C>T||||||,T|structural_intera
nteraction|3QBT:A_86-A_119:ENST00000300935|protein_coding|5/8|c.357C>T||||||,T|structural_int
_variant|HIGH|AP1M1|ENSG00000072958|transcript|ENST00000590263.5|protein_coding|1/5|c.-17+
|ENST00000598608.1|protein_coding|1/3|c.256dupG|p.Ala86fs|359/742|256/639|86/212||WARNIN
00130307|transcript|ENST00000598309.1|protein_coding|1/3|c.-200-2delA||||||WARNING_TRANSCI
:|ENST00000394458.7|protein_coding|1/9|c.69dupC|p.Glu24fs|184/3218|70/2010|24/669||INFO_RE
ENST00000598347.2|protein_coding|8/8|c.1751_1770delGTGTGTGTGTGTGTGTGTGT|p.Cys584fs|175
ENST00000598347.2|protein_coding|8/8|c.1751_1770delGTGTGTGTGTGTGTGTGTGT|p.Cys584fs|175
ENST00000598347.2|protein_coding|8/8|c.1751_1770delGTGTGTGTGTGTGTGTGTGT|p.Cys584fs|175
ENST00000598347.2|protein_coding|8/8|c.1751_1770delGTGTGTGTGTGTGTGTGTGT|p.Cys584fs|175
cript|ENST00000598347.2|protein_coding|8/8|c.1748_1749insTTGT|p.Leu591fs|1751/2003|1749/179
ot|ENST00000598347.2|protein_coding|8/8|c.1748_1749insTT|p.Leu591fs|1751/2003|1749/1794|58
NSG00000160117|transcript|ENST00000598347.2|protein_coding|8/8|c.1752_1753insTT|p.Val585fs|
ENST00000598347.2|protein_coding|8/8|c.1771_1786delTTGTGTGTGTGTGTGT|p.Leu591fs|1773/200
ENST00000598347.2|protein_coding|8/8|c.1771_1786delTTGTGTGTGTGTGTGT|p.Leu591fs|1773/200
ENST00000598347.2|protein_coding|8/8|c.1771_1784delTTGTGTGTGTGTGT|p.Leu591fs|1773/2003|
ENST00000598347.2|protein_coding|8/8|c.1771_1784delTTGTGTGTGTGTGT|p.Leu591fs|1773/2003|
ENST00000598347.2|protein_coding|8/8|c.1771_1778delTTGTGTGT|p.Leu591fs|1773/2003|1771/17
ENST00000598347.2|protein_coding|8/8|c.1771_1778delTTGTGTGT|p.Leu591fs|1773/2003|1771/17
ENST00000598347.2|protein_coding|8/8|c.1771_1778delTTGTGTGT|p.Leu591fs|1773/2003|1771/17
ENST00000598347.2|protein_coding|8/8|c.1771_1774delTTGT|p.Leu591fs|1773/2003|1771/1794|59
ENST00000598347.2|protein_coding|8/8|c.1771_1774delTTGT|p.Leu591fs|1773/2003|1771/1794|59
ENST00000598347.2|protein_coding|8/8|c.1771_1774delTTGT|p.Leu591fs|1773/2003|1771/1794|59
ENST00000598347.2|protein_coding|8/8|c.1771_1772delTT|p.Leu591fs|1773/2003|1771/1794|591/
ENST00000598347.2|protein_coding|8/8|c.1771_1772delTT|p.Leu591fs|1773/2003|1771/1794|591/
.7|transcript|ENST00000598347.2|protein_coding|8/8|c.1791_1792dupGT|p.Ter598fs|1795/2003|17
.7|transcript|ENST00000598347.2|protein_coding|8/8|c.1791_1792dupGT|p.Ter598fs|1795/2003|17
.7|transcript|ENST00000598347.2|protein_coding|8/8|c.1791_1792dupGT|p.Ter598fs|1795/2003|17
ENST00000594625.5|protein_coding|1/5|c.20_23delAATG|p.Glu7fs|36/585|20/569|7/188||WARNINC
30479|transcript|ENST00000597067.5|processed_transcript|1/4|n.186+2T>G||||||,G|upstream_gen
NST00000222249.13|protein_coding|11/11|c.1555_1556delGG|p.Gly519fs|1874/3625|1555/1632|51
00096996|transcript|ENST00000600835.6|protein_coding|1/17|c.-109-2A>T||||||,A|splice_acceptor_
interaction|3KHF:A_951-A_1032:ENST00000262811|protein_coding|23/27|c.2853C>T||||||,T|synony
nteraction|2QYM:A_240-A_272:ENST00000447275|protein_coding|10/15|c.816A>G||||||,C|structur
nteraction|2QYM:A_207-A_238:ENST00000447275|protein_coding|9/15|c.713G>A||||||,T|missense_
NST00000593594.1|nonsense_mediated_decay|2/3|c.251delG|p.Gly84fs|253/481|251/267|84/88||\
597776.1|protein_coding|1/3|c.1A>G|p.Met1?|120/446|1/327|1/108||WARNING_TRANSCRIPT_NO_
ENST00000593641.3|protein_coding|8/9|c.489dupC|p.Trp164fs|491/900|490/618|164/205||WARNII
ot|ENST00000262817.7|protein_coding|2/8|c.303_304delTG|p.Glu102fs|390/1613|303/1029|101/34
I|HOMER3|ENSG00000051128|transcript|ENST00000392351.7|protein_coding|3/9|c.171+2_171+4de
0000543877.1|protein_coding|3/4|c.82C>T|p.Gln28*|428/537|82/191|28/62||WARNING_TRANSCRIF
ENST00000592502.1|protein_coding|4/4|c.1080_1081delTA|p.His360fs|1190/4627|1080/1311|360/4

0000184635|transcript|ENST00000586021.5|nonsense_mediated_decay|3/4|c.227-3dupT|||||INFO_
0000184635|transcript|ENST00000586021.5|nonsense_mediated_decay|3/4|c.227-3dupT|||||INFO_
0000184635|transcript|ENST00000586021.5|nonsense_mediated_decay|3/4|c.227-3dupT|||||INFO_
0586021.5|nonsense_mediated_decay|4/5|c.254A>G|p.Ter85Trpext*?|397/583|254/255|85/84||,G|
ENST00000601440.5|protein_coding|4/4|c.1383_1549del|p.Cys464fs|1696/5963|1383/1587|461/52
:|ENST00000601440.5|protein_coding|4/4|c.1505dupT|p.Ile503fs|1652/5963|1505/1587|502/528||,
:|ENST00000601440.5|protein_coding|4/4|c.1505dupT|p.Ile503fs|1652/5963|1505/1587|502/528||,
ENST00000594534.5|protein_coding|4/4|c.290dupT|p.Phe98fs|429/757|291/618|97/205||INFO_REA
ENST00000594534.5|protein_coding|4/4|c.290dupT|p.Phe98fs|429/757|291/618|97/205||INFO_REA
NST00000594534.5|protein_coding|4/4|c.291delC|p.Leu102fs|429/757|291/618|97/205||,T|downstr
|ENST00000594534.5|protein_coding|4/4|c.305dupT|p.Leu102fs|444/757|306/618|102/205||INFO_
|ENST00000594534.5|protein_coding|4/4|c.305dupT|p.Leu102fs|444/757|306/618|102/205||INFO_
|ENST00000594534.5|protein_coding|4/4|c.305dupT|p.Leu102fs|444/757|306/618|102/205||INFO_
0352|transcript|ENST00000613286.4|nonsense_mediated_decay|5/7|c.159C>A|p.Cys53*|481/2696|
ENST00000596053.5|protein_coding|5/5|c.273delA|p.Lys91fs|624/800|273/333|91/110||INFO_REAL
ENST00000596053.5|protein_coding|5/5|c.273delA|p.Lys91fs|624/800|273/333|91/110||INFO_REAL
ENST00000358491.8|protein_coding|4/4|c.1580delA|p.His527fs|1788/2233|1580/2025|527/674||,C
000598288.5|protein_coding|5/5|c.250C>T|p.Gln84*|555/560|250/255|84/84||,A|intron_variant|M
0000599916.5|protein_coding|5/5|c.330T>A|p.Cys110*|485/740|330/384|110/127||
000593802.1|protein_coding|2/3|c.304C>T|p.Gln102*|403/493|304/394|102/130||WARNING_TRAN
0000269332|transcript|ENST00000600260.5|processed_transcript|9/9|n.1702-1G>T||||||,T|splice_a
229676|transcript|ENST00000456783.2|protein_coding|1/3|c.-94+2delT|||||
00229676|transcript|ENST00000456783.2|protein_coding|3/3|c.131-1G>T||||||,T|downstream_gene
3|transcript|ENST00000397104.5|protein_coding|8/8|c.3114A>C|p.Ter1038Tyrext*?|3114/3114|311
NST00000397104.5|protein_coding|8/8|c.3094delC|p.His1032fs|3094/3114|3094/3114|1032/1037||
NST00000397104.5|protein_coding|7/8|c.2708_2715delTTATGAAA|p.Leu903fs|2715/3114|2708/311
NST00000397104.5|protein_coding|7/8|c.2631_2706delACATAAGATAATTCATACTGGGGAGAAACCGTA
ENST00000596209.3|protein_coding|4/4|c.2518_2519insC|p.Met840fs|2609/7817|2518/2595|840/
ENST00000402377.3|protein_coding|4/4|c.609_610delTG|p.Cys203fs|752/6497|609/1938|203/645|
000266976|transcript|ENST00000587961.1|lincRNA|3/3|n.1662+2T>G||||||,G|intron_variant|MODI
000267617|transcript|ENST00000585883.1|lincRNA|2/2|n.202+2T>C|||||
G00000264515|transcript|ENST00000582581.5|lincRNA|2/9|n.410-2A>T||||||,A|splice_acceptor_var
G00000267662|transcript|ENST00000585336.1|antisense|1/3|n.38-3dupT||||||INFO_REALIGN_3_PR
000267465|transcript|ENST00000592644.1|lincRNA|1/1|n.62+2T>C||||||,C|intron_variant|MODIFIE
6|interaction|4B93:B_837-B_868:ENST00000306065|protein_coding|25/29|c.2511C>T||||||,A|syno
6|interaction|4B93:B_773-B_806:ENST00000306065|protein_coding|24/29|c.2418G>A||||||,T|struc
IGH|TDRD12|ENSG00000173809|transcript|ENST00000564769.2|nonsense_mediated_decay|5/6|c.*
0305768.9|protein_coding|19/19|c.2350T>C|p.Ter784Glnext*?|2439/5678|2350/2352|784/783||,G|
action|4BXO:A_2025-B_158:ENST00000267430-ENST00000588258|protein_coding|5/5|c.473C>T||||
0000239210|transcript|ENST00000471178.2|processed_pseudogene|1/1|n.116-2delA|||||
G00000205209|transcript|ENST00000595326.1|processed_transcript|3/3|n.372+2_372+3insGTATAT|
G00000205209|transcript|ENST00000595326.1|processed_transcript|3/3|n.372+2_372+3insGTATAT|
G00000205209|transcript|ENST00000595326.1|processed_transcript|3/3|n.372+2_372+3insGTATAT|
G00000205209|transcript|ENST00000595326.1|processed_transcript|3/3|n.372+2_372+3insGTATAT|
G00000205209|transcript|ENST00000595326.1|processed_transcript|3/3|n.372+2_372+3insGTATAT|
t&intron_variant|HIGH|ZNF302|ENSG00000089335|transcript|ENST00000627982.2|protein_coding|6
t&intron_variant|HIGH|ZNF302|ENSG00000089335|transcript|ENST00000627982.2|protein_coding|6

t&intron_variant|HIGH|ZNF302|ENSG00000089335|transcript|ENST00000627982.2|protein_coding|6
t&intron_variant|HIGH|ZNF302|ENSG00000089335|transcript|ENST00000627982.2|protein_coding|6
t&intron_variant|HIGH|ZNF302|ENSG00000089335|transcript|ENST00000627982.2|protein_coding|6
0000329285.12|protein_coding|4/4|c.1258G>T|p.Glu420*|1632/3099|1258/1767|420/588||,A|dow
ENST00000329285.12|protein_coding|4/4|c.1014_1015delCT|p.Tyr339fs|1389/3099|1014/1767|338/
eraction|3T2N:A_92-A_131:ENST00000262626|protein_coding|6/13|c.391C>T||||||,T|structural_inte
G00000179066&ENSG00000227392|gene_variant|ENSG00000227392|||n.35105993_35105965delAAC
G00000179066&ENSG00000227392|gene_variant|ENSG00000227392|||n.35105993_35105965delAAC
T00000324675.3|protein_coding|1/2|c.693G>A|p.Trp231*|742/1184|693/1110|231/369||,T|downstr
T00000324675.3|protein_coding|1/2|c.564G>A|p.Trp188*|613/1184|564/1110|188/369||,T|downstr
ST00000598058.1|protein_coding|4/4|c.447delG|p.Arg150fs|449/510|447/508|149/168||WARNING_
00544992.6|protein_coding|12/13|c.2251C>T|p.Arg751*|2292/2698|2251/2256|751/751||,T|synon
0000205786|transcript|ENST00000602653.5|lincRNA|2/6|n.191-2A>C||||||,C|upstream_gene_varian
:|ENST00000339686.7|protein_coding|5/16|c.850_851insCA|p.Gly284fs|1027/1927|850/1431|284/4
|transcript|ENST00000339686.7|protein_coding|5/16|c.849_850insAGCAGCAGTG|p.Gly284fs|1026/1
NST00000591135.1|protein_coding|2/2|c.231_232insT|p.Ala78fs|232/536|232/288|78/95||WARNIN
00272333|transcript|ENST00000420124.2|protein_coding|10/36|c.3529-3dupC||||||INFO_REALIGN_
00004777|transcript|ENST00000590893.5|nonsense_mediated_decay|5/6|c.*18+1G>C||||||,C|upstr
00225872|transcript|ENST00000637421.1|processed_transcript|8/8|n.1281+2T>A||||||,T|splice_don
00225872|transcript|ENST00000564335.1|processed_transcript|2/2|n.94+1G>C||||||,G|downstream
105270|transcript|ENST00000590559.2|protein_coding|1/4|c.-101-1G>C||||||WARNING_TRANSCRIP
|interaction|4PHJ:B_166-B_237:ENST00000246533|protein_coding|9/11|c.711C>T||||||,T|structural_
161281|transcript|ENST00000292907.7|protein_coding|2/3|c.102+2T>G||||||,C|splice_donor_varian
NSG00000267254|transcript|ENST00000587413.5|antisense|2/2|n.353-3dupT||||||INFO_REALIGN_3_
NSG00000267254|transcript|ENST00000587413.5|antisense|2/2|n.353-3dupT||||||INFO_REALIGN_3_
NSG00000267254|transcript|ENST00000587413.5|antisense|2/2|n.353-3dupT||||||INFO_REALIGN_3_
GH|ZNF568|ENSG00000198453|transcript|ENST00000616085.1|protein_coding|3/3|c.1117-1T>C||||
00455427.6|protein_coding|9/9|c.1714T>C|p.Ter572Argext*?|2043/2378|1714/1716|572/571||,C|st
000245680|transcript|ENST00000392156.7|nonsense_mediated_decay|2/4|c.73-1G>T||||||,A|splice_
000188283|transcript|ENST00000586068.5|retained_intron|1/4|n.350-3dupT||||||INFO_REALIGN_3_
00181666|transcript|ENST00000591259.5|protein_coding|4/4|c.257-3dupT||||||INFO_REALIGN_3_PI
00181666|transcript|ENST00000591259.5|protein_coding|4/4|c.257-3dupT||||||INFO_REALIGN_3_PI
cript|ENST00000436120.6|protein_coding|5/5|c.901_902insTGTG|p.Pro301fs|1009/2828|902/1830|3
ENST00000436120.6|protein_coding|5/5|c.904delT|p.Tyr302fs|1011/2828|904/1830|302/609||,A|se
SG00000267470|transcript|ENST00000588382.1|antisense|1/1|n.62-3dupT||||||INFO_REALIGN_3_P
SG00000267470|transcript|ENST00000588382.1|antisense|1/1|n.62-3dupT||||||INFO_REALIGN_3_P
riant&intron_variant|HIGH|ZNF571|ENSG00000180479|transcript|ENST00000328550.6|protein_codir
riant&intron_variant|HIGH|ZNF571|ENSG00000180479|transcript|ENST00000328550.6|protein_codir
riant&intron_variant|HIGH|ZNF571|ENSG00000180479|transcript|ENST00000328550.6|protein_codir
riant&intron_variant|HIGH|ZNF571|ENSG00000180479|transcript|ENST00000328550.6|protein_codir
0000328550.6|protein_coding|4/5|c.1600C>T|p.Gln534*|1719/4113|1600/1830|534/609||,A|stop_g
00334928.10|protein_coding|4/4|c.1939C>T|p.Gln647*|2072/2239|1939/1959|647/652||,C|missens
00334928.10|protein_coding|4/4|c.1939C>T|p.Gln647*|2072/2239|1939/1959|647/652||,C|missens
nteraction|2R0O:B_182-B_212:ENST00000252699|protein_coding|5/21|c.546C>T||||||,T|structural_
00594552.5|nonsense_mediated_decay|3/6|c.106T>C|p.Ter36Glnext*?|108/1046|106/108|36/35||,
teraction|1J8F:A_153-A_176:ENST00000249396|protein_coding|8/16|c.458G>A||||||,T|structural_ir
669|transcript|ENST00000602004.1|protein_coding|1/2|c.92+2T>C||||||WARNING_TRANSCRIPT_NC

NST00000606380.2|polymorphic_pseudogene|1/5|c.66delA|p.Gly23fs|343/1637|66/541|22/179||WA
|interaction|3H7H:B_199-B_240:ENST00000432763|protein_coding|11/30|c.720G>A||||||,A|structu
eraction|1G86:A_80-A_109:ENST00000221804|protein_coding|3/4|c.240T>C||||||,G|structural_inter
NST00000616721.4|protein_coding|16/28|c.7905_7915delCCCGCCGGGGA|p.Cys2635fs|7923/12787|
NST00000616721.4|protein_coding|7/28|c.3662_3663delAG|p.Glu1221fs|3671/12787|3662/12615|1
t|ENST00000616721.4|protein_coding|7/28|c.3659_3660insCC|p.Glu1221fs|3667/12787|3659/1261
:|ENST00000616721.4|protein_coding|7/28|c.3587_3588delGC|p.Arg1196fs|3596/12787|3587/12615|1
:|ENST00000616721.4|protein_coding|7/28|c.3583_3584insGC|p.Asp1195fs|3591/12787|3583/1261
NST00000616721.4|protein_coding|7/28|c.3563_3564delAG|p.Gln1188fs|3572/12787|3563/12615|1
script|ENST00000616721.4|protein_coding|7/28|c.3558_3559insCCCCT|p.Gly1187fs|3566/12787|355
t|ENST00000616721.4|protein_coding|7/28|c.3556_3557insCC|p.Val1186fs|3564/12787|3556/1261
NST00000616721.4|protein_coding|7/28|c.3552delA|p.Pro1185fs|3560/12787|3552/12615|1184/42
00128000|transcript|ENST00000221355.10|protein_coding|3/5|c.-404-2A>G||||||,C|splice_accepto
nteraction|1HDO:A_10-A_74:ENST00000263368|protein_coding|2/5|c.220C>T||||||,A|structural_int
nteraction|1HDO:A_31-A_64:ENST00000263368|protein_coding|2/5|c.192C>T||||||,A|structural_int
|interaction|4CRH:A_21-A_49:ENST00000291842|protein_coding|1/18|c.63T>C||||||,C|structural_int
interaction|1M5K:F_16-F_80:ENST00000243563|protein_coding|2/6|c.240A>G||||||,G|structural_int
0000301146.8|protein_coding|9/9|c.1400C>A|p.Ser467*|1942/2281|1400/1485|467/494||,T|stop_g
interaction|3IBD:A_172-A_203:ENST00000324071|protein_coding|4/9|c.516G>T||||||,T|structural_i
interaction|3IBD:A_162-A_487:ENST00000324071|protein_coding|9/9|c.1459C>T||||||,T|structural_
3|interaction|2P85:B_117-B_297:ENST00000330436|protein_coding|3/9|c.350C>T||||||,T|structural_
3|interaction|2P85:A_94-A_379:ENST00000330436|protein_coding|7/9|c.1135A>C||||||,C|structural
3|interaction|2P85:A_89-A_381:ENST00000330436|protein_coding|7/9|c.1141C>T||||||,T|structural_
|ENST00000331105.6|protein_coding|2/10|c.15dupC|p.Thr6fs|88/1720|16/1476|6/491||INFO_REAL
t|ENST00000604123.5|protein_coding|1/4|c.40_41delAA|p.Lys14fs|45/757|40/573|14/190||INFO_R
:|ENST00000539627.5|protein_coding|3/3|c.385_395delGGGGACTGGGC|p.Gly129fs|706/1843|385/3
t|ENST00000457836.6|protein_coding|8/9|c.972dupC|p.Thr325fs|978/1745|973/1347|325/448||INI
519|transcript|ENST00000598766.1|protein_coding|14/14|c.1464_1465delTT|p.Phe488fs|1464/2011|
519|transcript|ENST00000598766.1|protein_coding|14/14|c.1464_1465delTT|p.Phe488fs|1464/2011|
519|transcript|ENST00000598766.1|protein_coding|14/14|c.1464_1465delTT|p.Phe488fs|1464/2011|
GH|MEGF8|ENSG00000105429|transcript|ENST00000334370.8|protein_coding|29/40|c.5143-5_5143
00233681|transcript|ENST00000434624.1|unprocessed_pseudogene|3/4|n.703+2T>C||||||,G|intron
ST00000306511.4|protein_coding|2/5|c.357delC|p.Tyr120fs|455/1441|357/1281|119/426||,A|frame
00312439.10|protein_coding|4/6|c.862C>T|p.Arg288*|948/1882|862/1287|288/428||,A|stop_gaine
00617049.4|protein_coding|2/5|c.292C>T|p.Arg98*|390/1441|292/1281|98/426||,A|stop_gained|H
0243130|transcript|ENST00000593983.5|nonsense_mediated_decay|5/7|c.*840-1G>A||||||,T|synony
ST00000451895.1|protein_coding|2/4|c.48delG|p.Lys17fs|287/996|48/757|16/251||WARNING_TRAI
00244293.11|protein_coding|4/4|c.1002T>G|p.Tyr334*|1069/2073|1002/1209|334/402||,C|stop_ga
83668|transcript|ENST00000270077.7|protein_coding|1/5|c.65-1G>A||||||,T|splice_acceptor_variar
NST00000597107.1|nonsense_mediated_decay|2/4|c.91_97delAAAAAAA|p.Lys31fs|118/842|91/141|
NST00000597107.1|nonsense_mediated_decay|2/4|c.91_97delAAAAAAA|p.Lys31fs|118/842|91/141|
0000167637|transcript|ENST00000588797.5|protein_coding|6/6|c.-3_3delGAGATG|p.Met1del|546/2
ENST00000391958.6|protein_coding|5/5|c.1983delG|p.Leu662fs|2110/2784|1983/2013|661/670||,C
teraction|3OP0:A_153-A_219:ENST00000270279|protein_coding|2/11|c.458A>C||||||,C|structural_ir
42273|transcript|ENST00000270279.7|protein_coding|2/10|c.501-1G>C||||||,C|splice_acceptor_var
nteraction|2A1I:A_118-A_210:ENST00000013807|protein_coding|3/8|c.354T>C||||||,G|structural_ir
00589876.5|protein_coding|19/19|c.2262G>A|p.Trp754*|2289/2364|2262/2322|754/773||,T|upstre

0267757|transcript|ENST00000623430.1|antisense|1/2|n.76+1G>T||||||,T|splice_donor_variant&int
77045|transcript|ENST00000560168.1|protein_coding|1/2|c.131-1G>A||||||,T|synonymous_variant|
iant&intron_variant|HIGH|DMPK|ENSG00000104936|transcript|ENST00000618091.4|protein_coding|
iant&intron_variant|HIGH|DMPK|ENSG00000104936|transcript|ENST00000618091.4|protein_coding|
iant&intron_variant|HIGH|DMPK|ENSG00000104936|transcript|ENST00000618091.4|protein_coding|
iant&intron_variant|HIGH|DMPK|ENSG00000104936|transcript|ENST00000618091.4|protein_coding|
iant&intron_variant|HIGH|DMPK|ENSG00000104936|transcript|ENST00000618091.4|protein_coding|
iant&intron_variant|HIGH|DMPK|ENSG00000104936|transcript|ENST00000618091.4|protein_coding|
ISG00000268401|transcript|ENST00000595955.1|lincRNA|2/2|n.503-3dupT||||||INFO_REALIGN_3_P
ISG00000268401|transcript|ENST00000595955.1|lincRNA|2/2|n.503-3dupT||||||INFO_REALIGN_3_P
)4869|transcript|ENST00000601672.5|protein_coding|3/5|c.-264+2dupT||||||,TA|splice_donor_varia
000269729|transcript|ENST00000599127.1|lincRNA|2/4|n.95+2T>G||||||,C|intron_variant|MODIFIEI
G00000269745|transcript|ENST00000597337.1|antisense|1/1|n.96-1G>A||||||,T|upstream_gene_va
000300862.7|protein_coding|1/15|c.9G>A|p.Trp3*|78/2551|9/2004|3/667||,A|upstream_gene_varia
124440|transcript|ENST00000528563.5|nonsense_mediated_decay|8/10|c.*755-2A>G||||||,G|miss
nteraction|1WAO:3_44-3_74:ENST00000012443|protein_coding|2/13|c.132C>T||||||,T|synonymous_
ction|4LG6:A_280-B_507:ENST00000296785-ENST00000307522|protein_coding|1/1|c.1521G>C|||||
00414155.5|protein_coding|1/4|c.1A>G|p.Met1?|232/1882|1/516|1/171||,C|upstream_gene_variar
)0372|transcript|ENST00000597063.6|protein_coding|1/5|c.-153+1G>T||||||WARNING_TRANSCRIPT_
00000268746|transcript|ENST00000599924.1|lincRNA|1/2|n.86+1G>T||||||,T|upstream_gene_varia
)105373|transcript|ENST00000599582.1|processed_transcript|1/5|n.139+2T>G||||||,G|upstream_ge
)5373|transcript|ENST00000595143.5|protein_coding|8/10|c.670C>T|p.Arg224*|672/810|670/795|2
ot|ENST00000594182.1|protein_coding|5/5|c.290dupA|p.Asn97fs|291/303|291/300|97/99||WARNIN
|ENST00000599921.5|protein_coding|11/17|c.893delC|p.Pro298fs|1251/1984|893/1626|298/541||,
eraction|1X9N:A_754-A_845:ENST00000263274|protein_coding|26/28|c.2535G>T||||||,A|structural_
eraction|1X9N:A_338-A_409:ENST00000263274|protein_coding|13/28|c.1226G>A||||||,T|missense_
:|ENST00000391898.7|protein_coding|5/11|c.440_441dupAA|p.Val148fs|484/5263|441/1614|147/53
00600863.5|protein_coding|2/3|c.2T>C|p.Met1?|94/365|2/273|1/90||WARNING_TRANSCRIPT_NO_
00105443|transcript|ENST00000620214.1|protein_coding|4/4|c.376delA|p.Arg126fs|1630/2020|376,
596080.1|protein_coding|3/4|c.338A>G|p.Ter113Trpext*?|340/498|338/339|113/112||WARNING_TI
00425340.2|protein_coding|2/2|c.461G>A|p.Trp154*|578/3111|461/1032|154/343||,A|stop_gainec
76|interaction|1YDE:A_130-A_172:ENST00000263278|protein_coding|6/9|c.388C>T||||||,A|structur
0000087076|transcript|ENST00000599157.5|protein_coding|3/7|c.211-1G>C||||||,G|downstream_g
76|interaction|1YDE:A_4-A_31:ENST00000263278|protein_coding|2/9|c.91A>G||||||,C|structural_in
ot|ENST00000600406.1|protein_coding|1/1|c.620delT|p.Leu207fs|622/1725|620/774|207/257||WAI
ot|ENST00000600406.1|protein_coding|1/1|c.620delT|p.Leu207fs|622/1725|620/774|207/257||WAI
ot|ENST00000600406.1|protein_coding|1/1|c.620delT|p.Leu207fs|622/1725|620/774|207/257||WAI
000520557.1|nonsense_mediated_decay|3/5|c.196C>T|p.Arg66*|196/715|196/414|66/137||WARNII
ENST00000221403.6|protein_coding|4/7|c.517dupG|p.Ala173fs|558/1081|518/1005|173/334||INFO
000221403.6|protein_coding|5/7|c.697C>T|p.Gln233*|737/1081|697/1005|233/334||,T|stop_gainec
NST00000221403.6|protein_coding|6/7|c.881_882delAG|p.Glu294fs|921/1081|881/1005|294/334||I
5337|transcript|ENST00000596965.5|protein_coding|1/4|c.-1349+1delG||||||,A|5_prime_UTR_varia
)0000283251|transcript|ENST00000598442.1|processed_transcript|1/2|n.150+1G>A||||||,T|splice_d
NST00000595200.5|nonsense_mediated_decay|4/6|c.228delC|p.Tyr76fs|230/795|228/396|76/131||
ENST00000595200.5|nonsense_mediated_decay|4/6|c.243delA|p.Glu82fs|245/795|243/396|81/131|
ENST00000595200.5|nonsense_mediated_decay|4/6|c.243delA|p.Glu82fs|245/795|243/396|81/131|
ENST00000595200.5|nonsense_mediated_decay|4/6|c.243delA|p.Glu82fs|245/795|243/396|81/131|

NST00000595200.5|nonsense_mediated_decay|4/6|c.244_245delGA|p.Glu82fs|246/795|244/396|82/
NST00000595200.5|nonsense_mediated_decay|4/6|c.244_245delGA|p.Glu82fs|246/795|244/396|82/
NST00000595200.5|nonsense_mediated_decay|4/6|c.244delG|p.Glu82fs|246/795|244/396|82/131||
NST00000595200.5|nonsense_mediated_decay|4/6|c.244delG|p.Glu82fs|246/795|244/396|82/131||
104863|transcript|ENST00000595200.5|nonsense_mediated_decay|4/5|c.*26dupA||||||WARNING_
104863|transcript|ENST00000595200.5|nonsense_mediated_decay|4/5|c.*26dupA||||||WARNING_
NST00000610896.1|protein_coding|3/3|c.1364_1365insAC|p.His455fs|1365/1557|1365/1557|455/5
T00000610896.1|protein_coding|3/3|c.1370_1373delACGC|p.Asp457fs|1370/1557|1370/1557|457/5
0142541|transcript|ENST00000477613.6|processed_transcript|2/7|n.94-1G>T||||||,T|upstream_ger
456|transcript|ENST00000601809.5|protein_coding|1/4|c.-9+2T>G||||||WARNING_TRANSCRIPT_INC
ENST00000312865.10|protein_coding|17/18|c.2012_2013delAG|p.Gln671fs|2065/2332|2012/2244|6
9650|transcript|ENST00000322344.7|protein_coding|11/16|c.1029+2T>C||||||,G|splice_donor_varia
39650|transcript|ENST00000593946.5|nonsense_mediated_decay|1/15|c.152-2A>G||||||,C|splice_
42528|transcript|ENST00000598809.5|protein_coding|1/4|c.-300+2C>T||||||WARNING_TRANSCRIPT
000062822|transcript|ENST00000601098.5|protein_coding|1/5|c.-127-3dupT||||||WARNING_TRANS
000062822|transcript|ENST00000601098.5|protein_coding|1/5|c.-127-3dupT||||||WARNING_TRANS
|ENST00000376918.7|protein_coding|7/8|c.700dupG|p.Ala234fs|747/2014|701/765|234/254||INFO_
00000599973.1|protein_coding|4/4|c.778C>T|p.Arg260*|781/1026|778/954|260/317||,T|synonymo
515|transcript|ENST00000601503.5|protein_coding|2/4|c.60+2T>C||||||WARNING_TRANSCRIPT_INC
G00000269495|transcript|ENST00000601506.1|antisense|1/3|n.124-1G>A||||||,A|splice_acceptor_
6474|transcript|ENST00000525263.5|protein_coding|3/4|c.457+2T>C||||||,G|splice_donor_variant&
9437|transcript|ENST00000156499.6|protein_coding|2/7|c.26+1G>A||||||,T|splice_donor_variant&i
IST00000262262.4|protein_coding|3/7|c.466_469delGGCC|p.Gly156fs|487/1444|466/1095|156/364|
00000186806|transcript|ENST00000335624.4|protein_coding|10/10|c.2576dupC|p.Gln860fs|2576/33
NST00000354232.8|protein_coding|1/5|c.278dupC|p.Pro94fs|2746/3551|278/1041|93/346||,TG|do
000142512|transcript|ENST00000530476.1|protein_coding|1/2|c.46-1G>A||||||WARNING_TRANSCR
pt|ENST00000291707.7|protein_coding|1/8|c.196dupG|p.Ala66fs|252/2121|196/1788|66/595||,GC|
T00000291707.7|protein_coding|1/8|c.85C>T|p.Gln29*|141/2121|85/1788|29/595||,A|stop_gained|
nscript|ENST00000429354.3|protein_coding|5/9|c.827dupC|p.Ala277fs|957/1926|827/1656|276/551
601714.5|protein_coding|1/4|c.2T>C|p.Met1?|15/2086|2/1758|1/585||,G|upstream_gene_variant|
00000268316|transcript|ENST00000596964.1|lincRNA|1/1|n.444-2A>G||||||,C|downstream_gene_v
3|interaction|3DW8:A_125-A_159:ENST00000322088|protein_coding|4/15|c.477C>T||||||,T|structu
eraction|2IE3:A_574-C_110:ENST00000322088-ENST00000481195|protein_coding|14/15|c.1722C>T||
_coding_transcript_exon_variant|HIGH|CTD-2525I3.5|ENSG00000269102|transcript|ENST0000059486
ENST00000595962.5|protein_coding|2/5|c.9_10delTG|p.Cys3fs|75/4738|9/1608|3/535||INFO_REALI
ENST00000422689.2|protein_coding|4/4|c.317delA|p.Asn106fs|332/2230|317/1734|106/577||INFO_
0000422689.2|protein_coding|4/4|c.1239T>A|p.Cys413*|1254/2230|1239/1734|413/577||,A|downs
pt|ENST00000422689.2|protein_coding|4/4|c.1243_1244insTG|p.Lys415fs|1259/2230|1244/1734|41
pt|ENST00000422689.2|protein_coding|4/4|c.1243_1244insTG|p.Lys415fs|1259/2230|1244/1734|41
pt|ENST00000422689.2|protein_coding|4/4|c.1243_1244insTG|p.Lys415fs|1259/2230|1244/1734|41
000167555|transcript|ENST00000360465.7|protein_coding|2/6|c.-136-3dupT||||||INFO_REALIGN_3
000167555|transcript|ENST00000360465.7|protein_coding|2/6|c.-136-3dupT||||||INFO_REALIGN_3
58405|transcript|ENST00000596674.1|processed_transcript|2/3|n.165+2C>T||||||,T|downstream_g
|ENST00000602144.5|nonsense_mediated_decay|3/5|c.69_70insT|p.Gly24fs|190/571|69/234|23/77
70949|transcript|ENST00000599056.5|protein_coding|3/6|c.-46+1G>A||||||,T|splice_donor_variant
0000601215.5|protein_coding|2/4|c.186G>A|p.Trp62*|202/561|186/321|62/106||,T|stop_gained|H
ISG00000269001|transcript|ENST00000597550.5|processed_transcript|1/4|n.153-1G>A||||||,A|splic

ENST00000396403.8|protein_coding|4/4|c.1336_1337delAA|p.Lys446fs|1464/6151|1336/1854|446/(
t|ENST00000391738.7|protein_coding|4/8|c.511_512insGT|p.His171fs|601/1753|512/1452|171/483
NST00000391738.7|protein_coding|4/8|c.514_515delGC|p.Ala172fs|603/1753|514/1452|172/483||,
ction|1J1D:B_252-C_68:ENST00000367318-ENST00000344887|protein_coding|5/8|c.204G>T||||||,A|
000588076.1|protein_coding|1/4|c.2T>C|p.Leu1?|3/893|2/624|1/207||,G|missense_variant|MODER/
NST00000590333.5|protein_coding|16/21|c.1503_1519delTGGGGGCGCCGGGGAGC|p.Gly502fs|1630/
ction|4FXW:A_401-B_20:ENST00000308924-ENST00000377390|protein_coding|11/12|c.1203C>T||||
79873|transcript|ENST00000593208.1|protein_coding|1/3|c.-249+1G>A||||||WARNING_TRANSCRIP
ENST00000342929.3|protein_coding|5/11|c.1255_1258delTTTC|p.Phe419fs|1258/3132|1255/3132|4
)|transcript|ENST00000590542.1|protein_coding|10/10|c.3090G>C|p.Ter1030Tyrext*?|3090/3090|3(
00000166770|transcript|ENST00000586091.5|lincRNA|2/2|n.408-2A>G||||||,G|splice_acceptor_vari
0269793|transcript|ENST00000595954.1|antisense|2/3|n.222+2T>A||||||,A|splice_donor_variant&ir
000254181.8|protein_coding|4/4|c.2739C>A|p.Tyr913*|3193/3705|2739/2769|913/922||,A|stop_ga
00269834.1|protein_coding|5/5|c.1312A>T|p.Lys438*|1698/2627|1312/1419|438/472||,A|downstre
)5146|transcript|ENST00000448930.5|protein_coding|1/7|c.-45+1C>T||||||,T|splice_region_variant&
)00152433|transcript|ENST00000595335.5|nonsense_mediated_decay|2/4|c.25-3dupT||||||INFO_RE
00000268533|transcript|ENST00000597410.1|protein_coding|1/2|c.40-1G>A||||||WARNING_TRANS
|ENST00000334181.4|protein_coding|3/3|c.2230dupA|p.Thr744fs|2481/2587|2231/2337|744/778||
0000343280.8|protein_coding|3/5|c.123G>A|p.Trp41*|384/5419|123/1470|41/489||,T|stop_gained
0000450712.2|nonsense_mediated_decay|2/3|c.169C>T|p.Arg57*|380/587|169/219|57/72||,A|upst
05136|transcript|ENST00000424930.6|protein_coding|4/4|c.301+1G>A||||||,A|splice_donor_varian
0000599847.5|protein_coding|4/5|c.267G>A|p.Trp89*|409/5832|267/354|89/117||,A|stop_gained|
00347302.7|protein_coding|1/3|c.1A>G|p.Met1?|180/2472|1/1695|1/564||,G|start_lost|HIGH|ZNF
ENST00000254182.11|protein_coding|4/5|c.94delG|p.Val32fs|287/1959|94/1668|32/555||,T|frames
0000512439.6|protein_coding|3/4|c.574C>T|p.Arg192*|771/5687|574/1314|192/437||,A|stop_gain
83828|transcript|ENST00000598885.5|protein_coding|2/2|c.163+1delG||||||INFO_REALIGN_3_PRIM
anscript|ENST00000602124.1|nonsense_mediated_decay|4/6|c.46dupC|p.His16fs|453/1020|46/129
ENST00000595830.5|protein_coding|3/5|c.1903_1904delAC|p.Thr635fs|2045/3543|1903/2031|635/(
pt|ENST00000595830.5|protein_coding|3/5|c.1901_1902insCC|p.Thr635fs|2042/3543|1901/2031|63
83515&ENSG00000268201|gene_variant|ENSG00000268201|||n.58267677_58267689delAAAAAAAA/
83515&ENSG00000268201|gene_variant|ENSG00000268201|||n.58267677_58267689delAAAAAAAA/
83515&ENSG00000268201|gene_variant|ENSG00000268201|||n.58267677_58267689delAAAAAAAA/
0000427624.2|protein_coding|3/3|c.600C>A|p.Cys200*|923/2038|600/1596|200/531||,T|stop_gain
on_coding_transcript_exon_variant|HIGH|CTD-2619J13.14|ENSG00000232098|transcript|ENST00000
NSG00000213753|transcript|ENST00000487264.5|processed_transcript|2/3|n.347-5_347-3dupCTC|||
:|ENST00000382398.3|protein_coding|2/2|c.163_166delCAAA|p.Gln55fs|423/655|163/336|55/111||
|ENST00000382398.3|protein_coding|2/2|c.317_318delCC|p.Pro106fs|577/655|317/336|106/111||I
ɔt|ENST00000334391.4|protein_coding|2/2|c.183dupA|p.His62fs|241/373|183/282|61/93||
)00196476|transcript|ENST00000400269.4|protein_coding|1/10|c.17+2_17+3insTA||||||,TTTA|splice
)00196476|transcript|ENST00000400269.4|protein_coding|1/10|c.17+2_17+3insTA||||||,TTTA|splice
)00196476|transcript|ENST00000400269.4|protein_coding|1/10|c.17+2_17+3insTA||||||,TTTA|splice
)00196476|transcript|ENST00000400269.4|protein_coding|1/10|c.17+2_17+3insTA||||||,TTTA|splice
)00196476|transcript|ENST00000400269.4|protein_coding|1/10|c.17+2_17+3insTA||||||,TTTA|splice
i00000276026|transcript|ENST00000620712.1|antisense|1/1|n.67+2T>C||||||,G|upstream_gene_var
000230839|transcript|ENST00000447206.1|lincRNA|2/3|n.148+1G>C|||||
00000226644|transcript|ENST00000447956.5|lincRNA|1/1|n.172+1delG||||||INFO_REALIGN_3_PRIM
ction|4BX9:A_399-C_728:ENST00000267199-ENST00000380445|protein_coding|22/24|c.2182T>C||||

)00235958|transcript|ENST00000446537.5|antisense|4/6|n.1197+2T>C||||||,C|splice_donor_variant
teraction|2CAR:A_7-A_32:ENST00000380113|protein_coding|2/8|c.94C>A||||||,A|structural_interact
088836|transcript|ENST00000380056.7|protein_coding|15/18|c.2066+2T>G||||||,C|splice_donor_va
|ENST00000356518.6|protein_coding|22/22|c.2412_2419delAGTCCAGA|p.Gln804fs|2661/3677|2412
)0000344754.5|protein_coding|2/21|c.262G>T|p.Glu88*|262/6720|262/5130|88/1709||
|interaction|1CWR:A_458-A_502:ENST00000439880|protein_coding|14/16|c.1374C>T||||||,T|structu
nteraction|1I4M:A_129-A_163:ENST00000379440|protein_coding|2/2|c.385A>G||||||,G|structural_ir
7|transcript|ENST00000338244.5|protein_coding|12/17|c.1226_1233dupCCCCCCCC|p.Ile412fs|1608/
7|transcript|ENST00000338244.5|protein_coding|12/17|c.1226_1233dupCCCCCCCC|p.Ile412fs|1608/
7|transcript|ENST00000338244.5|protein_coding|12/17|c.1226_1233dupCCCCCCCC|p.Ile412fs|1608/
000637422.1|protein_coding|2/2|c.96G>A|p.Trp32*|96/122|96/105|32/34||WARNING_TRANSCRIPT_
teraction|2VJ2:A_308-A_333:ENST00000254958|protein_coding|7/26|c.924C>T||||||,A|structural_in
teraction|4CBZ:A_32-A_196:ENST00000254958|protein_coding|4/26|c.588C>T||||||,A|structural_int
)0000270792|transcript|ENST00000603180.5|antisense|2/3|n.151+1G>A||||||,A|intron_variant|MOI
)0000233048|transcript|ENST00000456265.1|lincRNA|6/11|n.1279+2C>T||||||
)246081.2|protein_coding|1/4|c.2T>C|p.Met1?|46/1477|2/387|1/128||,C|upstream_gene_variant|M
anscript|ENST00000377873.7|protein_coding|8/8|c.1995_1996delTT|p.Ter666fs|2036/2207|1995/19
9474|transcript|ENST00000484001.1|processed_transcript|1/1|n.194+1G>T||||||,T|5_prime_UTR_p
9474|transcript|ENST00000464792.1|protein_coding|1/1|c.-454+1G>T||||||WARNING_TRANSCRIPT_
)149474|transcript|ENST00000435364.7|protein_coding|4/9|c.683-2A>G||||||,G|splice_acceptor_va
)00089091|transcript|ENST00000377630.9|nonsense_mediated_decay|11/19|c.*431-2G>A||||||,T|sp
00089091|transcript|ENST00000377630.9|nonsense_mediated_decay|1/19|c.-19-1G>T||||||,A|splice
)0000496549.5|nonsense_mediated_decay|2/15|c.98T>A|p.Leu33*|130/4093|98/225|33/74||,T|splic
)00101343|transcript|ENST00000377327.8|protein_coding|1/14|c.68-1G>C||||||,G|splice_acceptor_v
ST00000619574.4|protein_coding|1/11|c.39dupG|p.Pro14fs|94/1971|40/1875|14/624||INFO_REALIG
G00000230400|transcript|ENST00000437696.2|lincRNA|1/2|n.229+1G>C||||||,G|splice_donor_varia
.01439|transcript|ENST00000398409.1|protein_coding|1/3|c.-70-2A>C||||||,G|5_prime_UTR_varian
teraction|2CH9:A_90-A_126:ENST00000480798|protein_coding|3/4|c.270G>C||||||,C|synonymous_v
100997|transcript|ENST00000465694.1|nonsense_mediated_decay|1/1|c.*146+2C>T||||||WARNING
t|ENST00000576316.5|protein_coding|5/5|c.580_581insC|p.Ile194fs|580/620|580/585|194/194||W
t|ENST00000376403.5|protein_coding|3/3|c.11delT|p.Met4fs|390/1681|11/459|4/152||,C|frameshi
)0000231934|transcript|ENST00000445151.1|antisense|3/3|n.585-2A>C||||||
nteraction|2NZJ:A_157-A_191:ENST00000201979|protein_coding|4/5|c.573C>A||||||,A|structural_in
)0000400522.8|protein_coding|23/27|c.2893C>T|p.Gln965*|2987/3961|2893/3867|965/1288||
233146|transcript|ENST00000457066.1|unitary_pseudogene|9/9|n.1023+1G>A||||||
00214200|transcript|ENST00000397772.2|processed_pseudogene|1/1|n.290-1delA||||||
M3P2|ENSG00000214200|transcript|ENST00000397772.2|processed_pseudogene|1/1|n.290-2_290-1
PM3P2|ENSG00000214200|transcript|ENST00000397772.2|processed_pseudogene|1/1|n.290-2_290-
TPM3P2|ENSG00000214200|transcript|ENST00000397772.2|processed_pseudogene|1/1|n.290-2_29(
|TPM3P2|ENSG00000214200|transcript|ENST00000397772.2|processed_pseudogene|1/1|n.290-2_29
H|TPM3P2|ENSG00000214200|transcript|ENST00000397772.2|processed_pseudogene|1/1|n.290-2_2
00078747|transcript|ENST00000262650.10|protein_coding|6/25|c.476-3dupT||||||INFO_REALIGN_3_
00078747|transcript|ENST00000262650.10|protein_coding|6/25|c.476-3dupT||||||INFO_REALIGN_3_
t|ENST00000374810.7|protein_coding|3/5|c.49_53delGAAGA|p.Glu17fs|438/4184|49/663|17/220||
IGH|PROCR|ENSG00000101000|transcript|ENST00000216968.4|protein_coding|3/4|c.323-9_336dupT
)000374273.7|protein_coding|1/12|c.273T>A|p.Cys91*|385/1453|273/1314|91/437||,A|upstream_g
)214078|transcript|ENST00000437340.5|protein_coding|14/15|c.1237-1A>G||||||WARNING_TRANS(

ENST00000317677.9|protein_coding|14/16|c.1218dupT|p.Ala407fs|1353/1961|1218/1629|406/542|
000131043|transcript|ENST00000397286.7|protein_coding|3/3|c.988-3dupT|||||INFO_REALIGN_3_
.043|transcript|ENST00000397286.7|protein_coding|3/3|c.988-4_988-3delTT|||||INFO_REALIGN_3_
31043|transcript|ENST00000397286.7|protein_coding|3/3|c.988-3delT|||||INFO_REALIGN_3_PRIME
IST00000397286.7|protein_coding|4/4|c.994_995delAG|p.Leu333fs|1087/1466|994/1197|332/398||
_gained|HIGH|MROH8|ENSG00000101353|transcript|ENST00000343811.8|protein_coding|1/24|c.170
_gained|HIGH|MROH8|ENSG00000101353|transcript|ENST00000343811.8|protein_coding|1/24|c.170
nteraction|3S3J:A_629-A_655:ENST00000361475|protein_coding|12/13|c.1887G>A||||||,T|structura
nteraction|4PYG:E_139-E_291:ENST00000361475|protein_coding|7/13|c.873G>A||||||,T|synonymou
T00000496900.2|protein_coding|3/8|c.1528C>T|p.Arg510*|1800/2557|1528/2199|510/732||,A|stop
on_coding_transcript_exon_variant|HIGH|RPL27AP|ENSG00000215467|transcript|ENST00000358609.
&non_coding_transcript_exon_variant|HIGH|RPL27AP|ENSG00000215467|transcript|ENST0000035860
iant&non_coding_transcript_exon_variant|HIGH|RPL27AP|ENSG00000215467|transcript|ENST000003
exon_variant|LOW|RPL27AP|ENSG00000215467|transcript|ENST00000358609.4|processed_pseudoge
NST00000262605.8|protein_coding|3/5|c.576_579delGAGT|p.Ser193fs|697/6212|576/1029|192/342
eraction|3IAR:A_150-A_178:ENST00000372874|protein_coding|6/12|c.534A>G||||||,C|synonymous_
eraction|3IAR:A_80-A_139:ENST00000372874|protein_coding|4/12|c.239A>G||||||,C|missense_vari
eraction|3IAR:A_12-A_326:ENST00000372874|protein_coding|2/12|c.36G>A||||||,T|splice_region_va
ENST00000461272.5|nonsense_mediated_decay|5/6|c.677delG|p.Gly226fs|751/1197|677/681|226/2
|ENST00000626701.1|protein_coding|2/3|c.256delA|p.Arg86fs|310/617|256/363|86/120||INFO_REA
|ENST00000626701.1|protein_coding|2/3|c.256delA|p.Arg86fs|310/617|256/363|86/120||INFO_REA
|ENST00000626701.1|protein_coding|2/3|c.258_271delAGGAGGAGGAGGAA|p.Gly87fs|312/617|258/
|ENST00000626701.1|protein_coding|2/3|c.258_271delAGGAGGAGGAGGAA|p.Gly87fs|312/617|258/
000101017|transcript|ENST00000372285.7|protein_coding|7/8|c.647-3dupT|||||INFO_REALIGN_3_
000101017|transcript|ENST00000372285.7|protein_coding|7/8|c.647-3dupT|||||INFO_REALIGN_3_
000101017|transcript|ENST00000372285.7|protein_coding|7/8|c.647-3dupT|||||INFO_REALIGN_3_
000101017|transcript|ENST00000372285.7|protein_coding|7/8|c.647-3dupT|||||INFO_REALIGN_3_
teraction|3GEB:A_421-A_523:ENST00000327619|protein_coding|16/16|c.1569C>T||||||,T|structural_
GH|RP5-1049G16.4|ENSG00000229853|transcript|ENST00000448675.1|processed_pseudogene|1/1|
GH|RP5-1049G16.4|ENSG00000229853|transcript|ENST00000448675.1|processed_pseudogene|1/1|
GH|RP5-1049G16.4|ENSG00000229853|transcript|ENST00000448675.1|processed_pseudogene|1/1|
GH|RP5-1049G16.4|ENSG00000229853|transcript|ENST00000448675.1|processed_pseudogene|1/1|
GH|RP5-1049G16.4|ENSG00000229853|transcript|ENST00000448675.1|processed_pseudogene|1/1|
iant|HIGH|CTD-2653D5.1|ENSG00000255438|transcript|ENST00000526566.2|antisense|1/3|n.373-24
124214|transcript|ENST00000456866.2|protein_coding|2/4|c.21+1dupG|||||WARNING_TRANSCRIPT
NSG00000283529|transcript|ENST00000637341.1|processed_transcript|6/7|n.732-3dupT|||||INFO_
nteraction|2IAG:A_177-A_199:ENST00000244043|protein_coding|5/10|c.531C>T||||||,A|structural_i
00361573.2|protein_coding|1/16|c.3G>T|p.Met1?|45/6094|3/1746|1/581||,T|start_lost|HIGH|SLC9A
|ENST00000417961.5|protein_coding|7/16|c.546dupT|p.Val183fs|757/6309|547/1794|183/597||INF
interaction|1A5Y:A_199-A_229:ENST00000371621|protein_coding|6/10|c.597A>G||||||,G|structural_
|interaction|1A02:N_456-N_501:ENST00000396009|protein_coding|4/10|c.1503A>C||||||,G|structu
0000235166|transcript|ENST00000416190.5|lincRNA|2/7|n.405-2A>G||||||,G|intron_variant|MODII
24091|transcript|ENST00000617620.1|protein_coding|1/3|c.192+1T>C||||||,G|5_prime_UTR_varian
t|ENST00000371328.3|protein_coding|1/2|c.110_113delAATA|p.Gln37fs|433/911|110/516|37/171|
nscript|ENST00000371328.3|protein_coding|1/2|c.115_116insTGTG|p.Gly39fs|439/911|116/516|39/
nscript|ENST00000371325.1|protein_coding|1/2|c.109_110insAATA|p.Pro37fs|206/694|110/516|37/
t|ENST00000371325.1|protein_coding|1/2|c.112_115delTGTG|p.Cys38fs|208/694|112/516|38/171||

NSG00000231078|transcript|ENST00000442780.1|lincRNA|1/2|n.78-2G>A|||||
00000226308|transcript|ENST00000426580.1|lincRNA|2/2|n.180+1G>C||||||,C|intron_variant|MODI
teraction|1KHB:A_94-A_115:ENST00000319441|protein_coding|3/10|c.282C>T||||||,T|structural_int
teraction|2GMV:A_151-A_423:ENST00000319441|protein_coding|4/10|c.453G>A||||||,A|structural_
teraction|1NHX:A_235-A_403:ENST00000319441|protein_coding|5/10|c.705C>T||||||,T|structural_ir
teraction|1KHB:A_276-A_545:ENST00000319441|protein_coding|6/10|c.826G>A||||||,A|structural_i
teraction|1KHE:A_72-A_368:ENST00000319441|protein_coding|7/10|c.1102G>A||||||,A|structural_i
ST00000453793.1|protein_coding|2/2|c.223delT|p.Ser75fs|225/357|223/355|75/117||WARNING_TF
371173.7|protein_coding|1/8|c.3G>A|p.Met1?|181/2195|3/1290|1/429||,T|start_lost|HIGH|ZBP1|E
0237121|transcript|ENST00000416241.1|unprocessed_pseudogene|13/13|n.1996+2T>G||||||
action|4R3O:C_6-D_3:ENST00000044462-ENST00000370873|protein_coding|1/7|c.9C>T||||||,A|prot
30702|transcript|ENST00000474128.1|nonsense_mediated_decay|2/7|c.*3+2C>T|||||WARNING_TF
0370592.2|protein_coding|3/3|c.271T>C|p.Ter91Glnext*?|285/401|271/273|91/90||,C|3_prime_UTF
00000233017|transcript|ENST00000615180.1|antisense|1/3|n.147+2T>C||||||,C|upstream_gene_va
H|DIDO1|ENSG00000101191|transcript|ENST00000395343.5|protein_coding|1/15|c.-200+2_-200+8de
0000125514|transcript|ENST00000370341.3|lincRNA|2/2|n.1139-2G>A||||||,T|splice_acceptor_varia
|interaction|4RCI:A_400-A_542:ENST00000370339|protein_coding|4/5|c.1200C>T||||||,A|structural_
00101204|transcript|ENST00000615287.4|protein_coding|4/6|c.71-2A>G||||||,C|splice_acceptor_va
09|interaction|3K51:B_58-B_85:ENST00000369996|protein_coding|1/3|c.255A>G||||||,G|structural_
09|interaction|4J6G:D_91-D_111:ENST00000369996|protein_coding|1/3|c.333T>C||||||,C|synonymo
09|interaction|3MHD:D_132-D_162:ENST00000369996|protein_coding|2/3|c.486C>T||||||,T|synonym
37172|transcript|ENST00000470054.5|processed_transcript|7/9|n.1596+1delG|||||INFO_REALIGN_3
391|transcript|ENST00000618007.4|protein_coding|6/23|c.119+1G>A||||||,A|splice_donor_variant&
74391|transcript|ENST00000618007.4|protein_coding|23/23|c.1521-1G>T||||||,T|splice_acceptor_v
00000215559|transcript|ENST00000451663.5|transcribed_unprocessed_pseudogene|21/24|n.3313+1
243064|transcript|ENST00000463099.1|transcribed_unprocessed_pseudogene|22/27|n.2974+1G>A||
000281903|transcript|ENST00000412426.5|lincRNA|3/3|n.349+1G>A||||||,T|intron_variant|MODIFI
on_coding_transcript_exon_variant|HIGH|AF127936.7|ENSG00000281903|transcript|ENST000006302
000215386&ENSG00000201025|gene_variant|ENSG00000215386|||n.16284687_16284699delTGTGTG
00000232886|transcript|ENST00000430064.1|lincRNA|1/3|n.50-1G>T|||||
|interaction|2WIM:A_37-A_82:ENST00000400546|protein_coding|3/18|c.246T>C||||||,C|structural_i
000232512|transcript|ENST00000453802.5|lincRNA|2/2|n.194+1G>A||||||,A|splice_donor_variant&
000223870|transcript|ENST00000426609.1|lincRNA|3/3|n.251+2C>T||||||
NSG00000273492|transcript|ENST00000608591.5|lincRNA|2/2|n.300-3dupT||||||INFO_REALIGN_3_F
NSG00000273492|transcript|ENST00000608591.5|lincRNA|2/2|n.300-3dupT||||||INFO_REALIGN_3_F
NSG00000273492|transcript|ENST00000608591.5|lincRNA|2/2|n.300-3dupT||||||INFO_REALIGN_3_F
ot|ENST00000451462.5|protein_coding|1/4|c.21delG|p.Pro8fs|23/601|21/599|7/198||WARNING_TR
00154734|transcript|ENST00000517452.1|protein_coding|1/2|c.16+2T>C|||||WARNING_TRANSCRIP
T00000517452.1|protein_coding|1/3|c.11G>A|p.Trp4*|31/405|11/385|4/127||WARNING_TRANSCRI
on_coding_transcript_exon_variant|HIGH|LINC00113|ENSG00000225298|transcript|ENST000004425
NSG00000234083|transcript|ENST00000433344.1|lincRNA|1/2|n.103-3dupT|||||INFO_REALIGN_3_
000234083|transcript|ENST00000433344.1|lincRNA|1/2|n.103-5_103-3delTTT|||||INFO_REALIGN_3
0000234083|transcript|ENST00000433344.1|lincRNA|1/2|n.103-4_103-3delTT|||||INFO_REALIGN_3
00000234083|transcript|ENST00000433344.1|lincRNA|1/2|n.103-3delT|||||INFO_REALIGN_3_PRIMI
0000215005|transcript|ENST00000447985.1|processed_pseudogene|1/1|n.633-1dupA||||||,CTT|spli
0000215005|transcript|ENST00000447985.1|processed_pseudogene|1/1|n.633-1dupA||||||,CTT|spli
0000215005|transcript|ENST00000447985.1|processed_pseudogene|1/1|n.633-1dupA||||||,CTT|spli

G00000215317|transcript|ENST00000400014.2|processed_pseudogene|2/2|n.767-2delA|||||
G00000215317|transcript|ENST00000400014.2|processed_pseudogene|2/2|n.767-3dupA|||||
0000228817|transcript|ENST00000436529.2|lincRNA|2/2|n.339-2A>G||||||,G|splice_acceptor_varia
000259981|transcript|ENST00000565564.1|lincRNA|1/1|n.275+1A>G|||||
ST00000399889.3|protein_coding|1/1|c.405T>A|p.Cys135*|449/867|405/528|135/175||,T|downstre
pt|ENST00000614763.1|protein_coding|2/2|c.213delC|p.Tyr72fs|213/216|213/216|71/71||WARNIN
0491828.5|nonsense_mediated_decay|3/4|c.325T>A|p.Ter109Argext*?|458/889|325/327|109/108|
:|ENST00000431599.1|protein_coding|2/2|c.81delT|p.Phe27fs|83/417|81/237|27/78||,C|intron_vari
ENSG00000159079|gene_variant|ENSG00000265590|||n.32612298_32612258delCGCGGGCCACGCAG
000226527|transcript|ENST00000421051.1|lincRNA|2/2|n.330+2T>C|||||
NST00000356577.8|protein_coding|12/12|c.7236dupA|p.Ala2413fs|7712/8813|7237/7281|2413/242
ST00000356577.8|protein_coding|12/12|c.7248delA|p.Arg2416fs|7723/8813|7248/7281|2416/2426|
.837|transcript|ENST00000290299.6|protein_coding|1/7|c.34C>T|p.Gln12*|251/932|34/642|12/213
0000249209|transcript|ENST00000429238.2|protein_coding|1/7|c.-215+2T>C||||||WARNING_TRAN
teraction|2HRB:A_84-A_128:ENST00000290354|protein_coding|1/3|c.250C>G||||||,G|structural_int
teraction|2HRB:A_7-A_85:ENST00000290354|protein_coding|1/3|c.255C>T||||||,T|structural_intera
teraction|2HRB:A_93-A_114:ENST00000290354|protein_coding|1/3|c.279C>T||||||,T|synonymous_v
teraction|2HRB:A_139-A_202:ENST00000290354|protein_coding|3/3|c.606G>A||||||,A|synonymous
.59256|transcript|ENST00000546482.5|nonsense_mediated_decay|3/7|c.*12+1G>A||||||WARNING_
NST00000483178.2|protein_coding|2/2|c.194delA|p.Asn65fs|194/824|194/222|65/73||WARNING_TR
NST00000483178.2|protein_coding|2/2|c.194delA|p.Asn65fs|194/824|194/222|65/73||WARNING_TR
0431628.1|nonsense_mediated_decay|2/6|c.190T>C|p.Ter64Glnext*?|190/760|190/192|64/63||,G|
000380588.4|protein_coding|2/9|c.53G>A|p.Trp18*|156/2066|53/1224|18/407||,A|upstream_gene_
83844|transcript|ENST00000398652.7|protein_coding|2/8|c.136+2A>T||||||,T|upstream_gene_varia
pt|ENST00000269844.4|protein_coding|3/31|c.262dupC|p.Arg88fs|373/4881|262/4524|88/1507||,C
00000237232|transcript|ENST00000412906.5|lincRNA|1/1|n.392-1G>C||||||,C|splice_acceptor_varia
00000408989.6|protein_coding|11/22|c.1993C>T|p.Gln665*|1993/5262|1993/4341|665/1446||,T|s
00000160183|transcript|ENST00000291532.7|protein_coding|7/12|c.617-3_617-2dupTA||||||,CTA|s
nteraction|3K3E:B_241-B_338:ENST00000335512|protein_coding|11/19|c.723C>T||||||,T|structural_
00000354250.6|protein_coding|3/4|c.598C>T|p.Arg200*|667/1575|598/1422|200/473||,T|intron_va
teraction|3FHY:B_86-B_129:ENST00000291565|protein_coding|6/11|c.387G>A||||||,A|structural_in
teraction|2F7K:B_142-B_178:ENST00000291565|protein_coding|6/11|c.426G>A||||||,A|synonymou
000213440|transcript|ENST00000416034.1|processed_pseudogene|1/1|n.236-2_236-1delAA||||||,G
000232969|transcript|ENST00000426029.1|antisense|1/1|n.95+2T>C||||||,C|upstream_gene_varian
script|ENST00000400375.1|protein_coding|1/1|c.477_478insCA|p.Ser160fs|522/1215|477/849|159/2
pt|ENST00000400375.1|protein_coding|1/1|c.475_476delGA|p.Asp159fs|521/1215|475/849|159/28
0000400374.4|protein_coding|1/1|c.1206A>C|p.Ter402Cysext*?|1236/1643|1206/1206|402/401||,
ST00000622352.2|protein_coding|1/3|c.768C>A|p.Cys256*|768/1191|768/1191|256/396||WARNING
script|ENST00000622352.2|protein_coding|1/3|c.119_120insAG|p.Cys41fs|119/1191|119/1191|40/3
04|transcript|ENST00000616689.1|protein_coding|1/3|c.131_132insCAGCTGCT|p.Ala45fs|162/794|1
nscript|ENST00000380095.2|protein_coding|1/1|c.86_87dupCC|p.Cys30fs|150/1100|88/756|30/251|
160255|transcript|ENST00000397846.7|protein_coding|3/3|c.148-2A>G||||||,C|splice_acceptor_var
H|POFUT2|ENSG00000186866|transcript|ENST00000349485.9|protein_coding|2/8|c.382+2_382+6del
pt|ENST00000359759.8|protein_coding|1/41|c.1124dupG|p.Leu376fs|1125/6586|1125/5265|375/17
173638|transcript|ENST00000417954.5|protein_coding|4/4|c.762+2T>C||||||,G|missense_variant|
00000417954.5|protein_coding|4/5|c.745C>T|p.Arg249*|747/3572|745/807|249/268||,A|stop_gain
G00000173638|transcript|ENST00000417954.5|protein_coding|4/5|c.719_737dupCTGGCGGCGAGAGG

t|ENST00000400337.6|protein_coding|34/42|c.2826_2833delCCCCCCAG|p.Gly945fs|2920/5436|2826
on_coding_transcript_exon_variant|HIGH|PCBP3-OT1|ENSG00000280604|transcript|ENST000006262З
:|ENST00000413758.1|protein_coding|10/11|c.485dupC|p.Asp163fs|486/750|486/750|162/249||WA
[truncated: 1,027,350 more chars]
