# Supplementary material for: Genome diversity in Ukraine
Source: Gigascience. 2021 Jan 13;10(1):giaa159. doi: 10.1093/gigascience/giaa159 (PMC7804371; doi:10.1093/gigascience/giaa159)
Supplement: giaa159_Response_to_Reviewer_Comments_Original_Submission [file giaa159_response_to_reviewer_comments_original_submission.pdf]

Author's Response To Reviewer Comments

Close

Dear Dr. Edmunds:

As you suggested, we have removed all the supplemental files that were making the manuscript too large and deposited them in GigaDB, and left only the small ones. We also updated the manuscript to reflect that change.

Thank you very much, and we are looking to towards the review

Taras

Close
