# Supplementary material for: Genome diversity in Ukraine
Source: Gigascience. 2021 Jan 13;10(1):giaa159. doi: 10.1093/gigascience/giaa159 (PMC7804371; doi:10.1093/gigascience/giaa159)
Supplement: giaa159_Response_to_Reviewer_Comments_Revision_1 [file giaa159_response_to_reviewer_comments_revision_1.pdf]

Author's Response To Reviewer Comments

Close

Dear Dr. Edmunds:

Thank you for considering our communication presenting a population genomics dataset from a largely unexplored region of Eastern Europe, entitled “Genome Diversity in Ukraine”. We have carefully considered your comments and comments from the reviewers, answered them to our best ability and made the appropriate changes in the revised manuscript.

After our last communication we have revised the additional data to exclude anything that has not been specifically published or publicly released. Specifically, we have replaced the recently retracted dataset from Russia with the newly released data from HGDP.

I would like to thank you again for taking time to review our manuscript, and to the reviewers for the helpful suggestions. I think that you will find our efforts satisfactory, and looking forward to publication of this important data

Sincerely yours

Taras Oleksyk

ANSWERS TO THE REVIEWERS:

Reviewer #1:

This manuscript describes 97 genome sequences from Ukraine.
All the sequencing, processing, and data depository are done professionally and the analyses are at standard quality.
This reviewer thinks this genomic resource should be publicized as soon as possible:
1) The data set are unique enough
2) The data set contains nearly 100 whole genome sequences.

ADMIXTURE and PCA show the expected characteristics of the population and its history.

Answer: Thank you very much for a kind review. We have undated the ADMIXTURE and PCA to reflect the currently publicly available data to which we hope to contribute with our research.

Reviewer #2:

This paper reported 97 whole sequencing data of representative Ukrainians and their variation annotations using DNBSEQ-G50 sequencing platform. Illumina NovaSeq6000 S4 sequencing for one individual was done as quality control of sequencing. Genomic variants including SVs, indels, CNVs, SNPs and microsatellites were annotated and compared to neighboring populations. The goal of this paper is to provide a genomic resource for Eastern Europe. A few issues are listed below.

Major issues

Comment 1. Accession numbers or IDs for all data are not provided yet.

Answer:
1) The list of the cross validated samples and the source technology of the data is presented in the

Supplementary File 3. All the supplementary materials mentioned in the paper are uploaded to GigaScience ftp: and should be accessible to the editors

2) Additionally, all the reads are uploaded to NCBI SRA and are processed, and ready to release:
SRA submission information: SUB7904361
BioProject status: Processed
PRJNA661978: Ukrainian Genomes \ UA genomes
BioSample: Processed
SRA: Processed

We can add reviewers to our project team and provide them early access to the reads before the paper is released.

Comment 2. In Table S2, the number of SNPs, filtered counts and percentage filtered has no value.

Answer: We updated Table S2 with the most current data as it is submitted in the corrected article.

Comment 3. Will the sequencing depth impact the assembled genomes? For example, the illumine sequencing depth was 60 while DNBSEQ-G50 sequencing data have about 30X coverage.

Answer: We only sampled only one sample with Illumina technology and only for the comparison. Higher coverage of the Illumina data (60x) could have contributed to the differences observed between the platforms.is why there was more SNPs identified and higher concordance. However, since there Is only one sample sequenced with both platforms so we cannot make further conclusions from this comparison.

Action: We have modified paragraph 2 page 6: "Evaluation tests show that current algorithms are platform dependent, in the sense that they exhibit their best performance for specific types of structural variation as well as for specific size ranges [21], and the algorithms designed for detection and archived datasets are predominantly for Illumina pair-end sequencing [22,23]. While it is possible that these results indicate Illumina's superiority at detecting structural variation, it also can also be the consequence of the bioinformatics tools for calling structural variants developed using mainly the Illumina data, as suggested by previous comparative evaluations of the two technologies [24,25]. Additionally, higher coverage of the Illumina data (60x) could have contributed to the differences observed between the platforms."

Comment 4. In the first paragraph of page 14, the authors mentioned that "genetics is not a reliable determinant of ethnicity", this is conclusion is not well supported with evidences. Another explanation can be that the self-identified ethnic group is not reliable.

Answer: What reviewer has in mind is probably human population, not ethnicity.

We believe that the term "ethnicity" recognizes differences between people mostly on the basis of language and shared culture. Oxford Dictionary defines "ethnicity" as "as the fact or state of belonging to a social group that has a common national or cultural tradition". Wikipedia defines it as "an ethnic group or ethnicity is a named social category of people who identify with each other on the basis of shared attributes that distinguish them from other groups such as a common set of traditions, ancestry, language, history, society, culture, nation, religion, or social treatment within their residing area". Encyclopedia Britannica defines ethnicity as a characteristic that "relates to culturally contingent features, characterizes all human groups. It refers to a sense of identity and membership in a group that shares common language, cultural traits (values, beliefs, religion, food habits, customs, etc.), and a sense of a common history."

Therefore, we still believe that individual ethnicity cannot be defined in genetic sense. A Chinese baby adopted in Ukraine and raised in a Ukrainian cultural environment will have a Ukrainian ethnicity despite of its genes, due to this person's upbringing.

On the other hand, genetics clearly can still assign people to ancestral populations with high certainty,

even in places like Eastern Europe, where differences between populations have been traditionally ignored by the medical research.

Action: We modified sentence in Paragraph 1, page 13. “Genetics is not a reliable determinant of ethnicity, but can be used to evaluate contributions of population ancestry”.

Minor issues

1. There are some language issues. For example, in the first paragraph of Page 4, "while the ethnic Ukrainians constitute approximately than three quarters of the total population of the modern Ukraine". "than" in this sentence should be removed.

Action: we corrected this typo: “while the ethnic Ukrainians constitute approximately three quarters of the total population of the modern Ukraine”. We additionally screened the manuscript for typos and grammatical errors and fixed what we could.

Close
