# Supplementary material for: Genome diversity in Ukraine
Source: Gigascience. 2021 Jan 13;10(1):giaa159. doi: 10.1093/gigascience/giaa159 (PMC7804371; doi:10.1093/gigascience/giaa159)
Supplement: giaa159_Reviewer_2_Report_Original_Submission — Chaochun Wei -- 9/14/2020 Reviewed [file giaa159_reviewer_2_report_original_submission.pdf]

Reviewer Report

Title: Genome Diversity in Ukraine

Version: Revision 1 **Date:** 9/14/2020

Reviewer name: Chaochun Wei

Reviewer Comments to Author:

This paper reported 97 whole sequencing data of representative Ukrainians and their variation annotations using DNBSEQ-G50 sequencing platform. Illumina NovaSeq6000 S4 sequencing for one individual was done as quality control of sequencing. Genomic variants including SVs, indels, CNVs, SNPs and microsatellites were annotated and compared to neighboring populations. The goal of this paper is to provide a genomic resource for Eastern Europe. A few issues are listed below.
Major issues
1. Accession numbers or IDs for all data are not provided yet.
2. In Table S2, the number of SNPs, filtered counts and percentage filtered has no value.
3. Will the sequencing depth impact the assembled genomes? For example, the illumine sequencing depth was 60 while DNBSEQ-G50 sequencing data have about 30X coverage.
4. In the first paragraph of page 14, the authors mentioned that "genetics is not a reliable determinant of ethnicity", this is conclusion is not well supported with evidences. Another explanation can be that the self-identified ethnic group is not reliable.
Minor issues
1. There are some language issues. For example, in the first paragraph of Page 4, "while the ethnic Ukrainians constitute approximately than three quarters of the total population of the modern Ukraine". "than" in this sentence should be removed.

Level of Interest

Please indicate how interesting you found the manuscript: Choose an item.

Quality of Written English

Please indicate the quality of language in the manuscript: Choose an item.

Declaration of Competing Interests

Please complete a declaration of competing interests, considering the following questions:

- Have you in the past five years received reimbursements, fees, funding, or salary from an organisation that may in any way gain or lose financially from the publication of this manuscript, either now or in the future?

- Do you hold any stocks or shares in an organisation that may in any way gain or lose financially from the publication of this manuscript, either now or in the future?
- Do you hold or are you currently applying for any patents relating to the content of the manuscript?
- Have you received reimbursements, fees, funding, or salary from an organization that holds or has applied for patents relating to the content of the manuscript?
- Do you have any other financial competing interests?
- Do you have any non-financial competing interests in relation to this paper?

If you can answer no to all of the above, write 'I declare that I have no competing interests' below. If your reply is yes to any, please give details below.

I declare that I have no competing interests.

I agree to the open peer review policy of the journal. I understand that my name will be included on my report to the authors and, if the manuscript is accepted for publication, my named report including any attachments I upload will be posted on the website along with the authors' responses. I agree for my report to be made available under an Open Access Creative Commons CC-BY license (http://creativecommons.org/licenses/by/4.0/). I understand that any comments which I do not wish to be included in my named report can be included as confidential comments to the editors, which will not be published.

Choose an item.

To further support our reviewers, we have joined with Publons, where you can gain additional credit to further highlight your hard work (see: https://publons.com/journal/530/gigascience). On publication of this paper, your review will be automatically added to Publons, you can then choose whether or not to claim your Publons credit. I understand this statement.

Yes Choose an item.
